# Supplementary material for: An Exploratory Analysis of Changes in Circulating Plasma Protein Profiles Following Image-Guided Ablation of Renal Tumours Provides Evidence for Effects on Multiple Biological Processes
Source: Cancers (Basel). 2021 Nov 30;13(23):6037. doi: 10.3390/cancers13236037 (PMC8656737; doi:10.3390/cancers13236037)

Figure S1. Longitudinal profiles of all plasma proteins on the Immunooncology panel, prior to and post-ablation. Results for each protein are shown as linear NPX values and are shown for patients receiving CRYO (n=25), MWA (n=2) and RFA (n=8) and as a single timepoint for healthy controls (n=11). Dotted line indicates lower limit of quantification

Uniprot ID: P10145, analyte:IL8

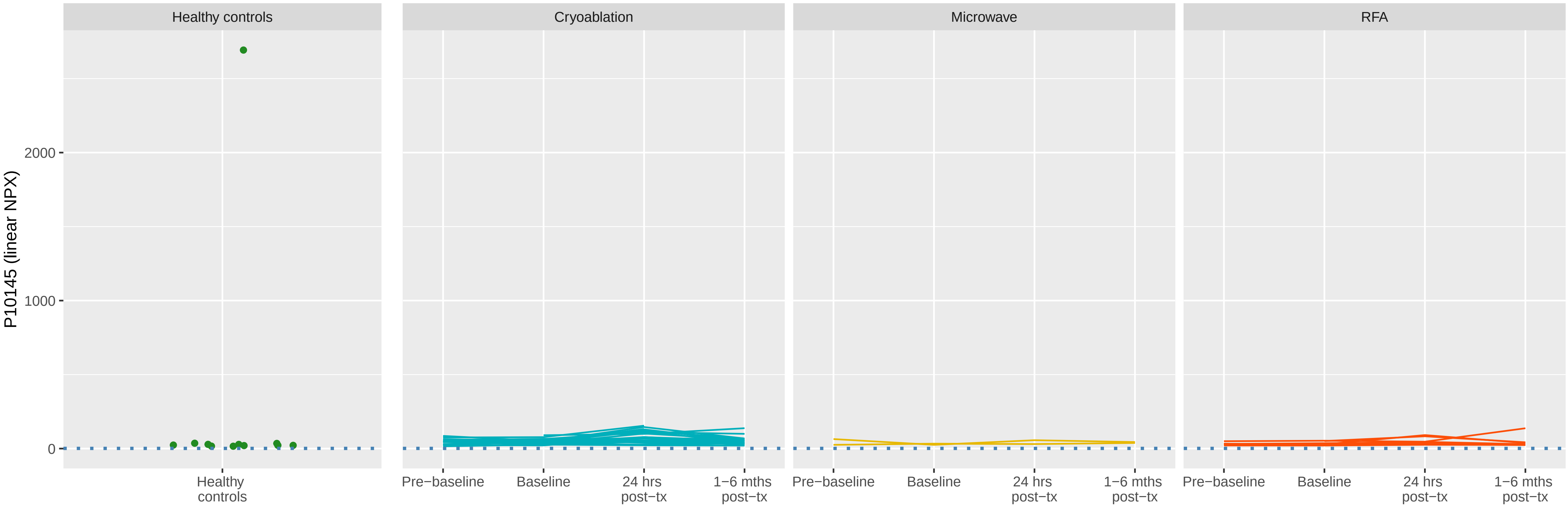

Uniprot ID: Q07011, analyte:TNFRSF9

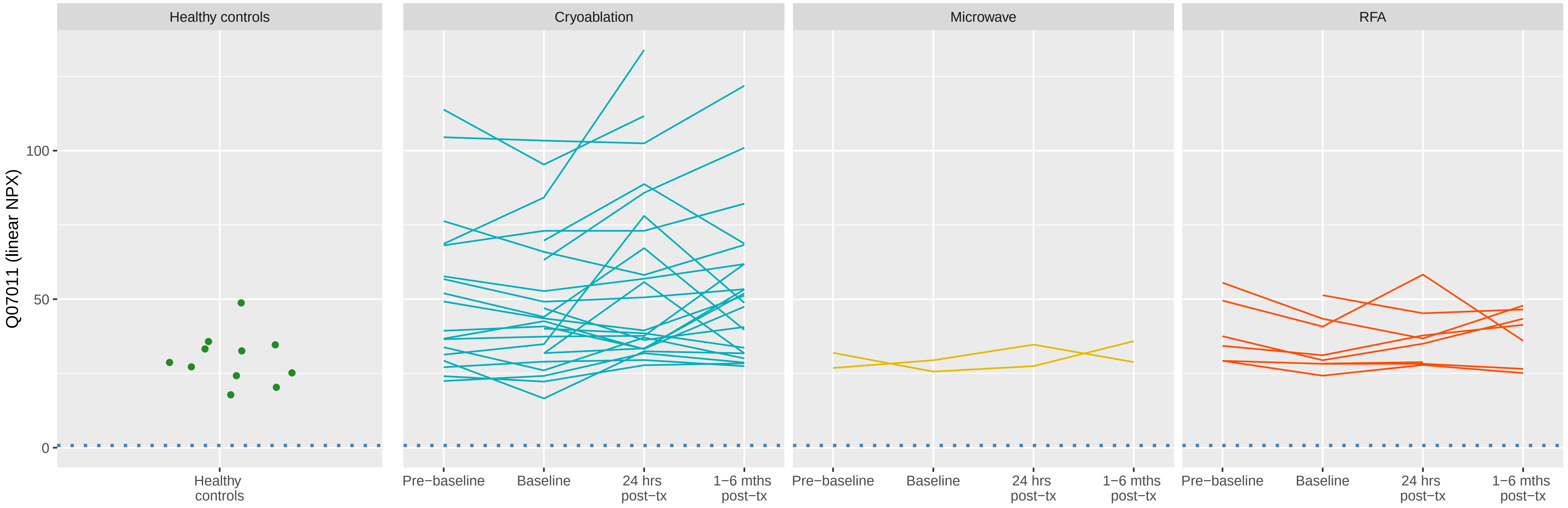

Uniprot ID: Q02763, analyte:TIE2

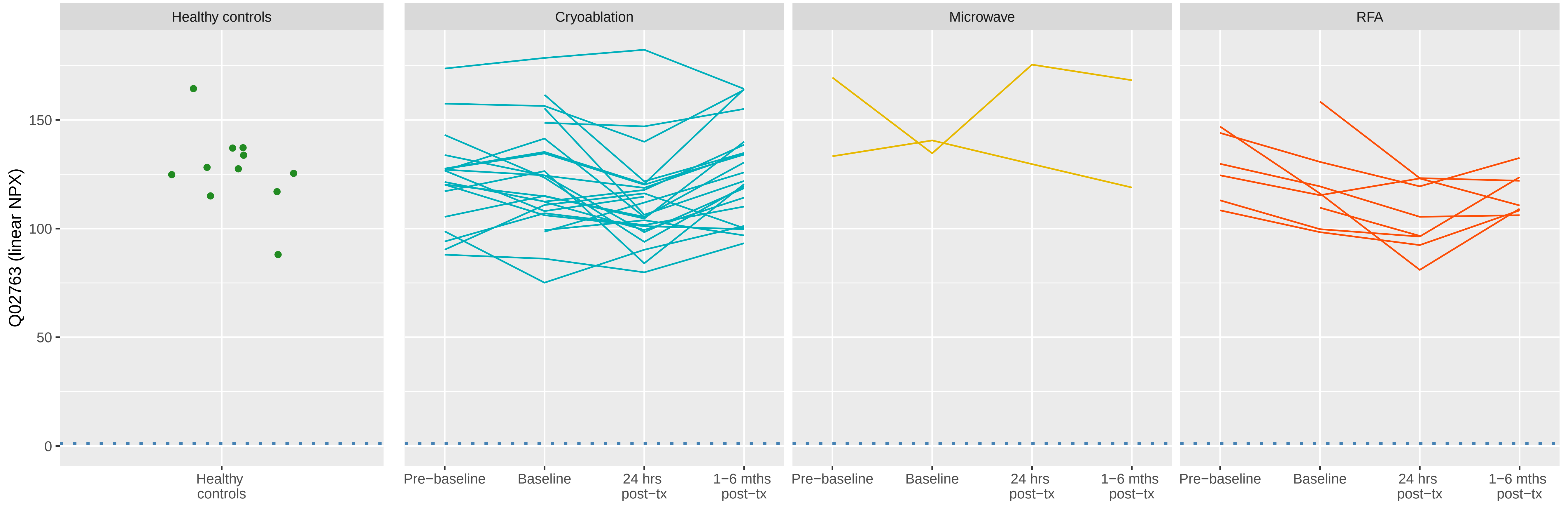

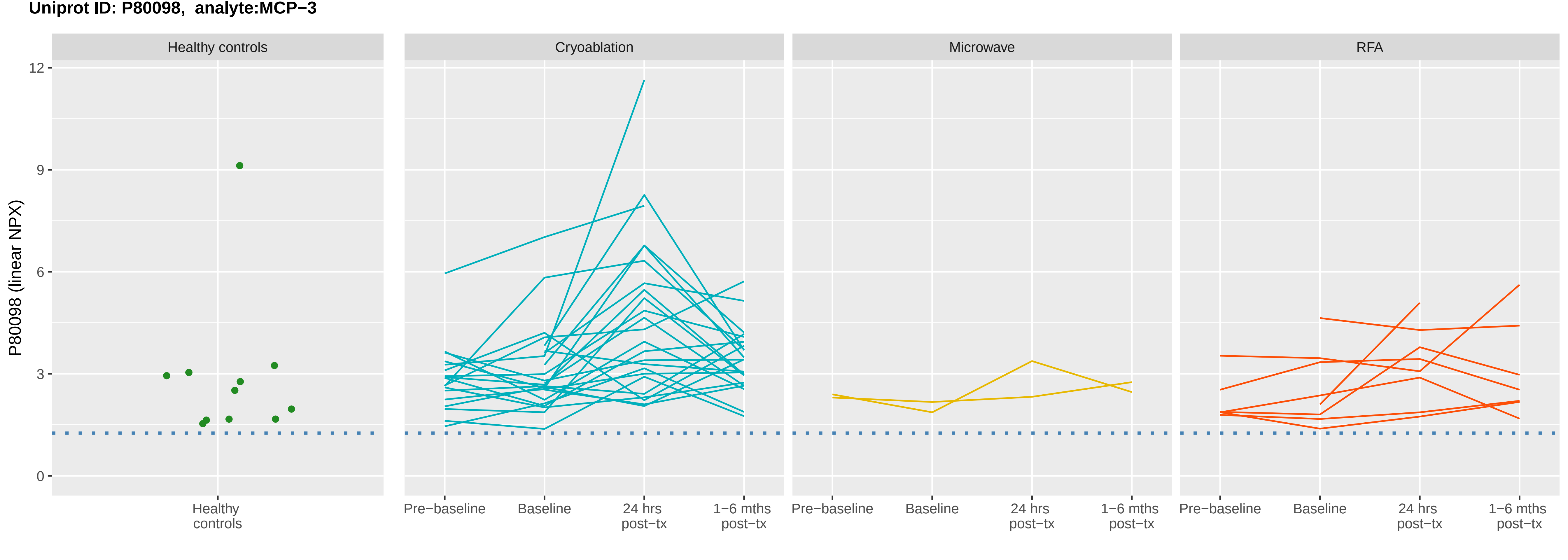

Uniprot ID: P80098, analyte:MCP−3
Healthy controls
Cryoablation
Microwave
RFA
P80098 (linear NPX)
12
9
6
3
0
Healthy controls
Pre−baseline
Baseline
24 hrs post−tx
1−6 mths post−tx

Uniprot ID: P29965, analyte:CD40−L

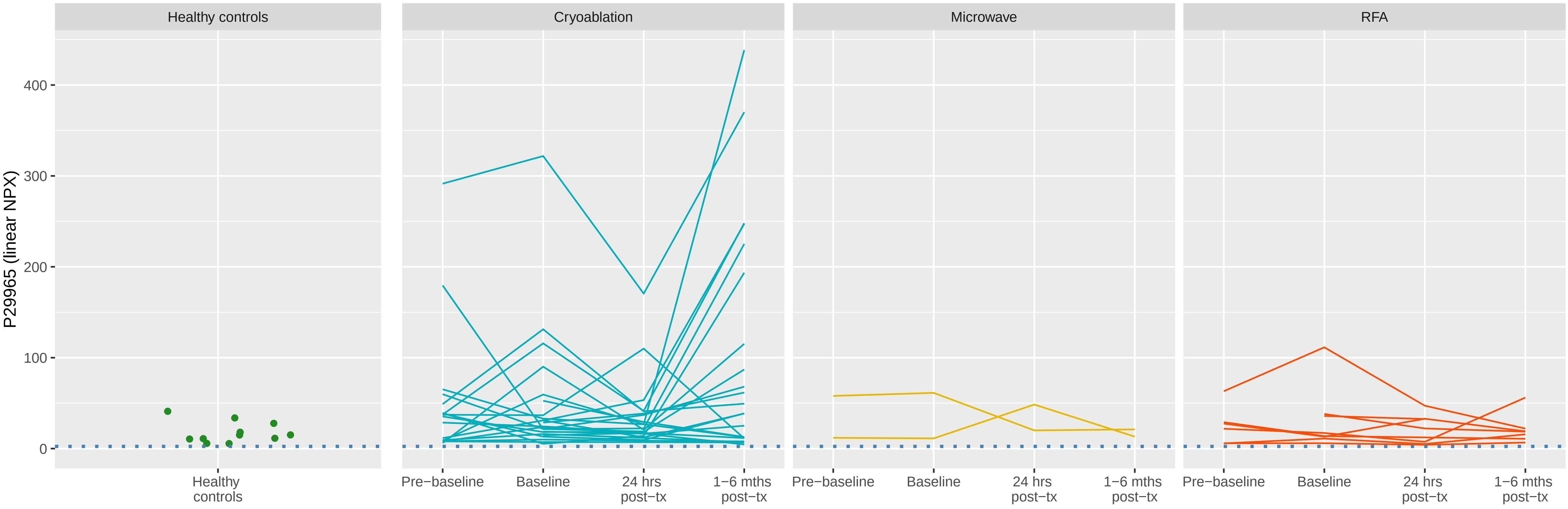

Uniprot ID: Q9BZW8, analyte:CD244

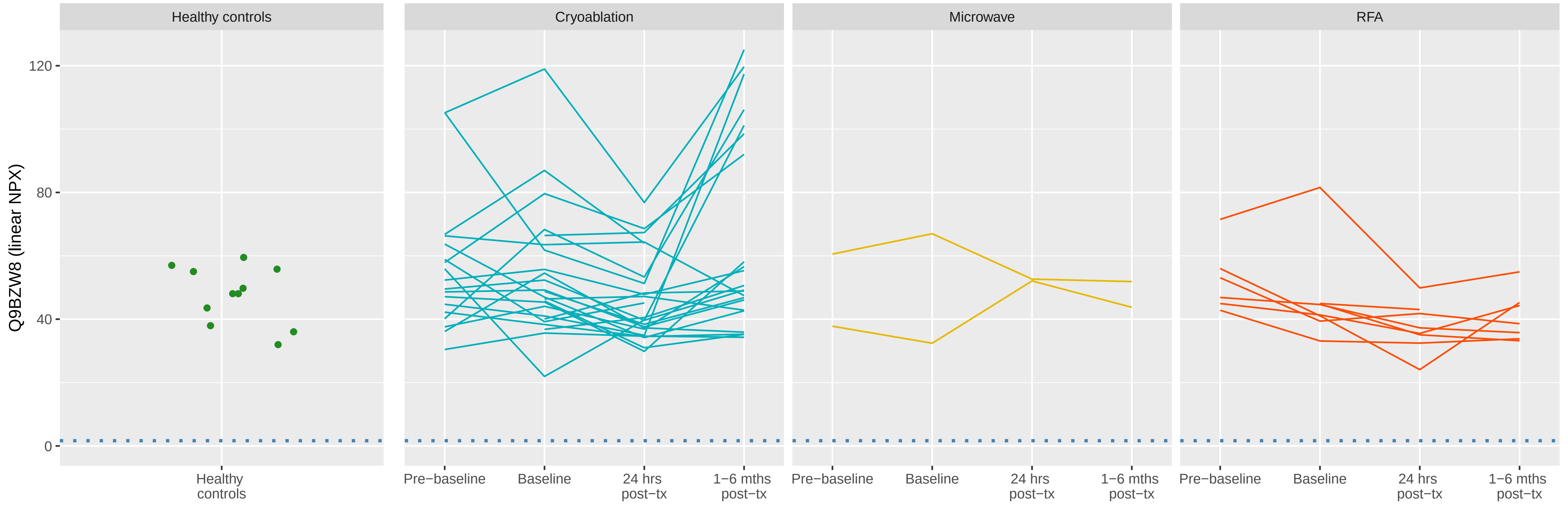

Uniprot ID: P01133, analyte:EGF

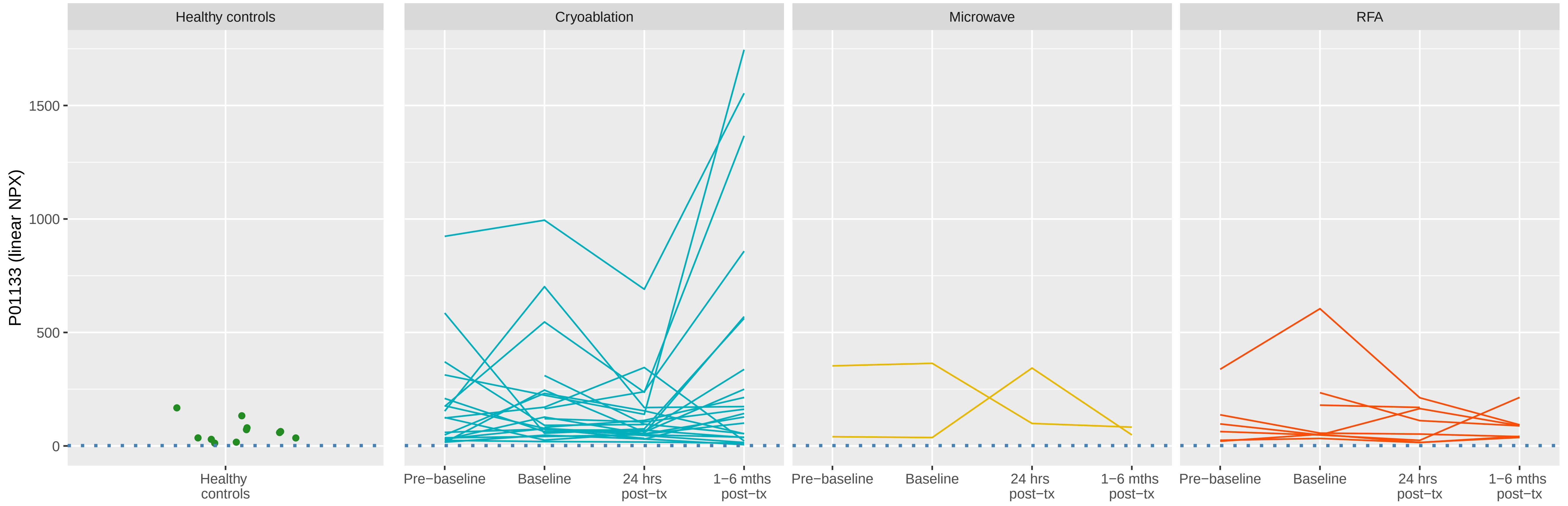

Uniprot ID: Q15389, analyte:ANGPT1

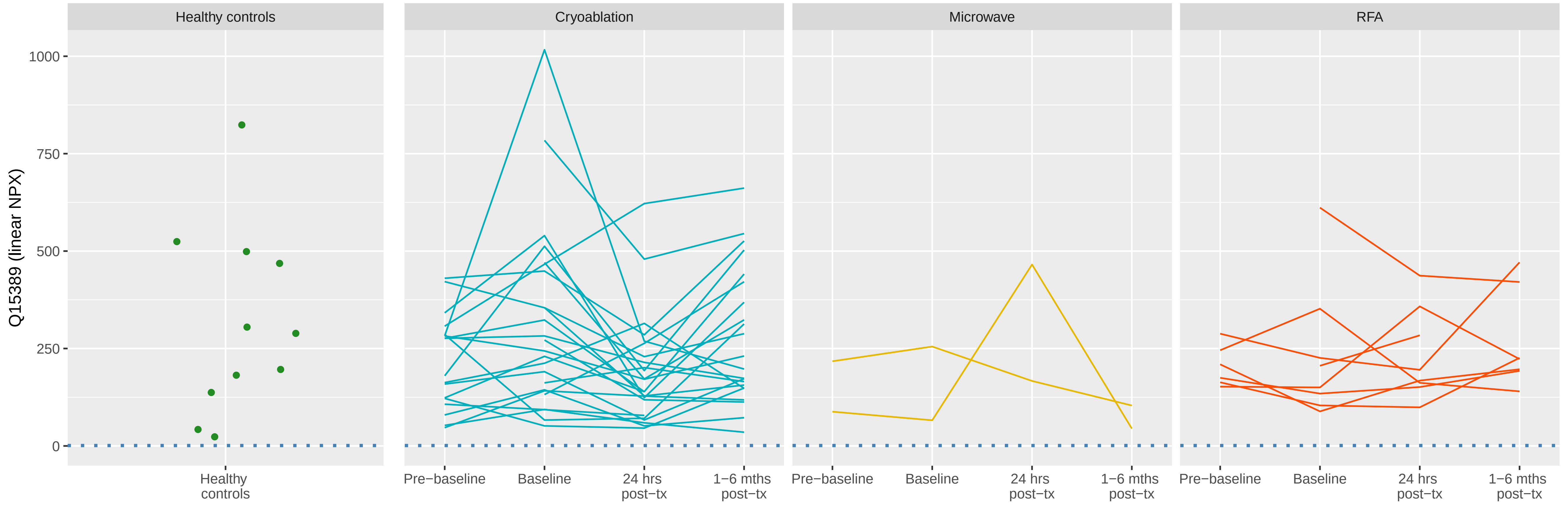

Uniprot ID: P13232, analyte:IL7

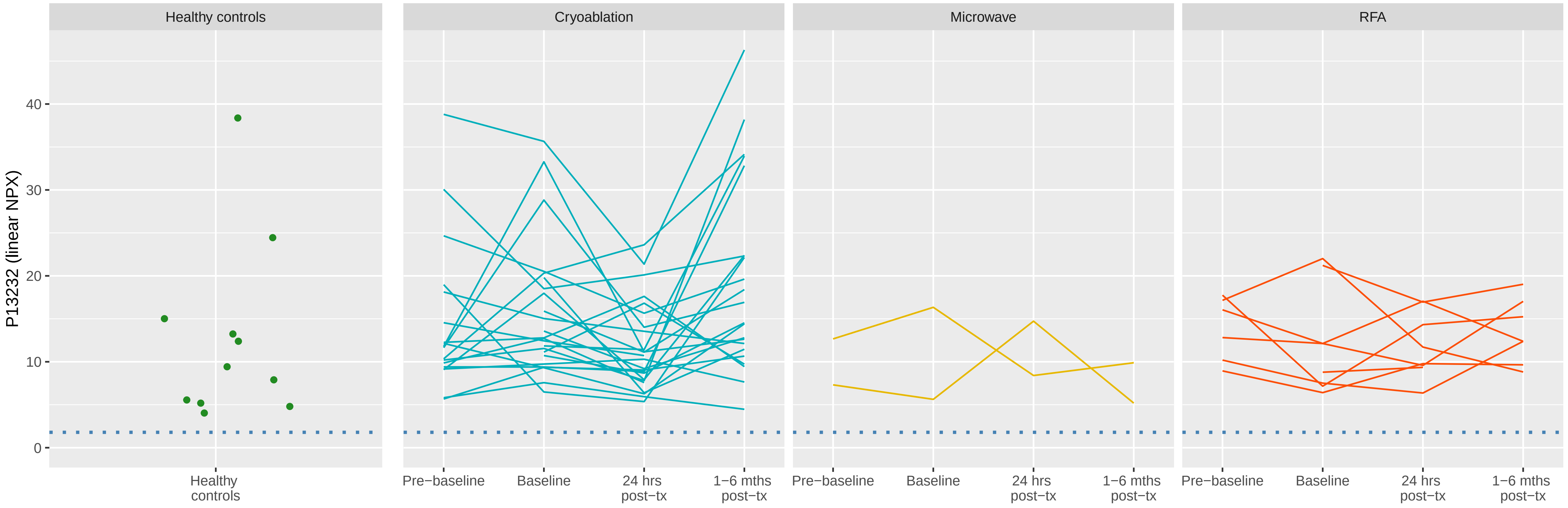

Uniprot ID: P49763, analyte:PGF

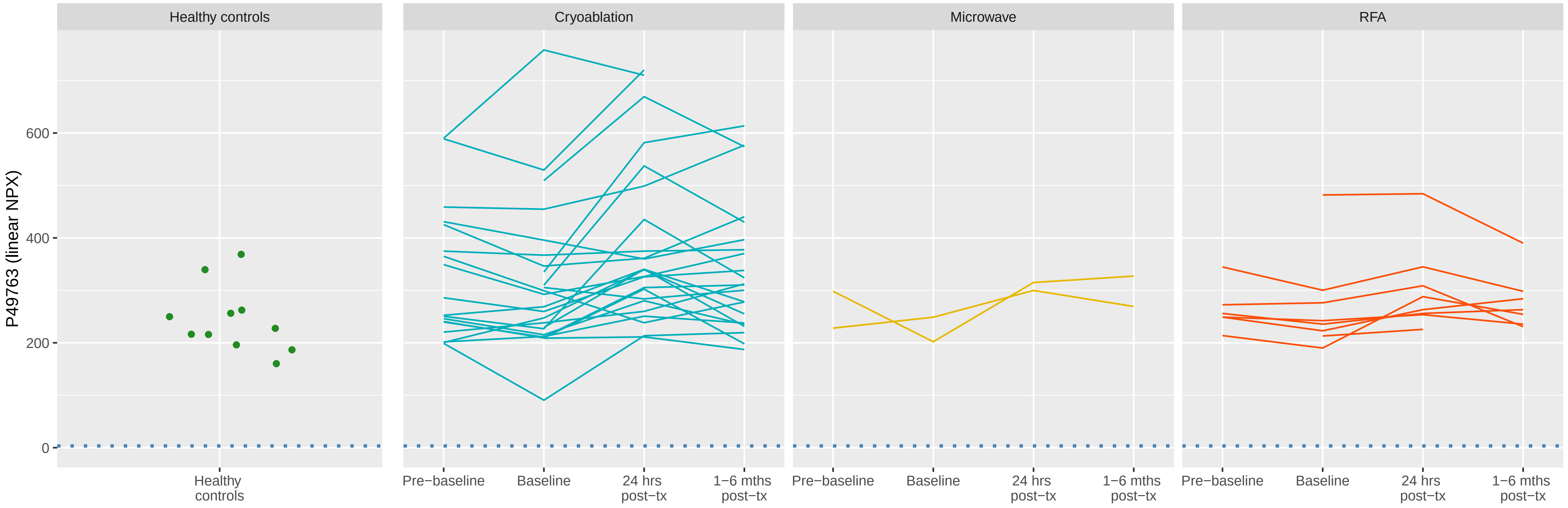

Uniprot ID: P05231, analyte:IL6

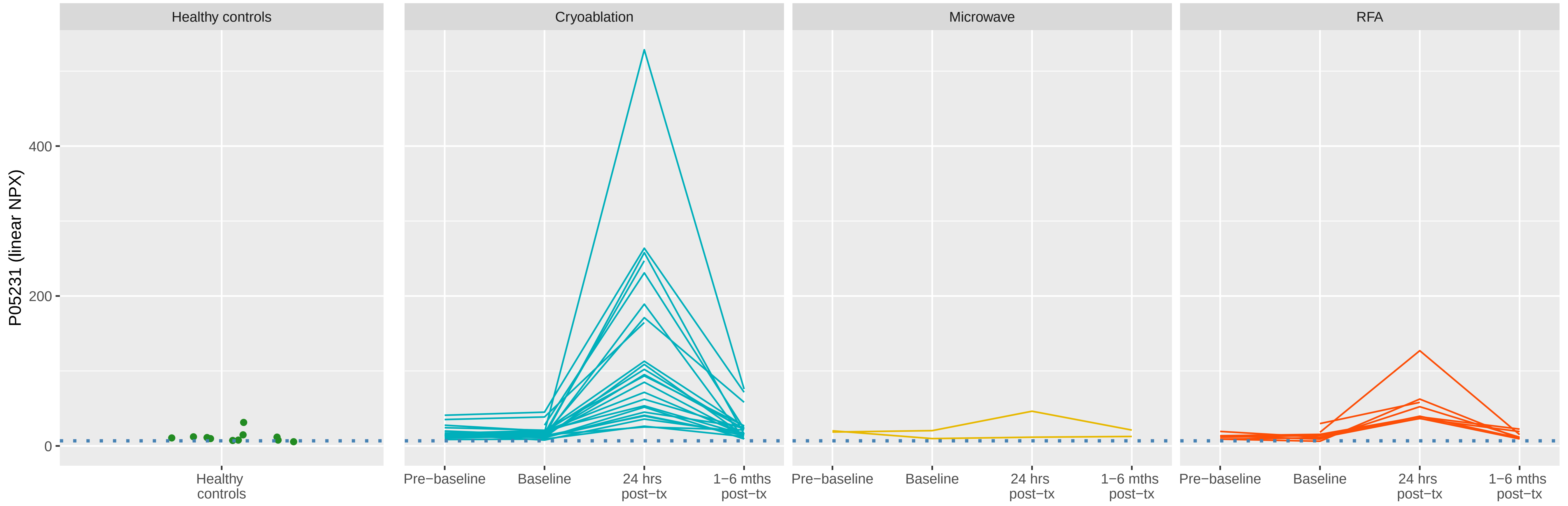

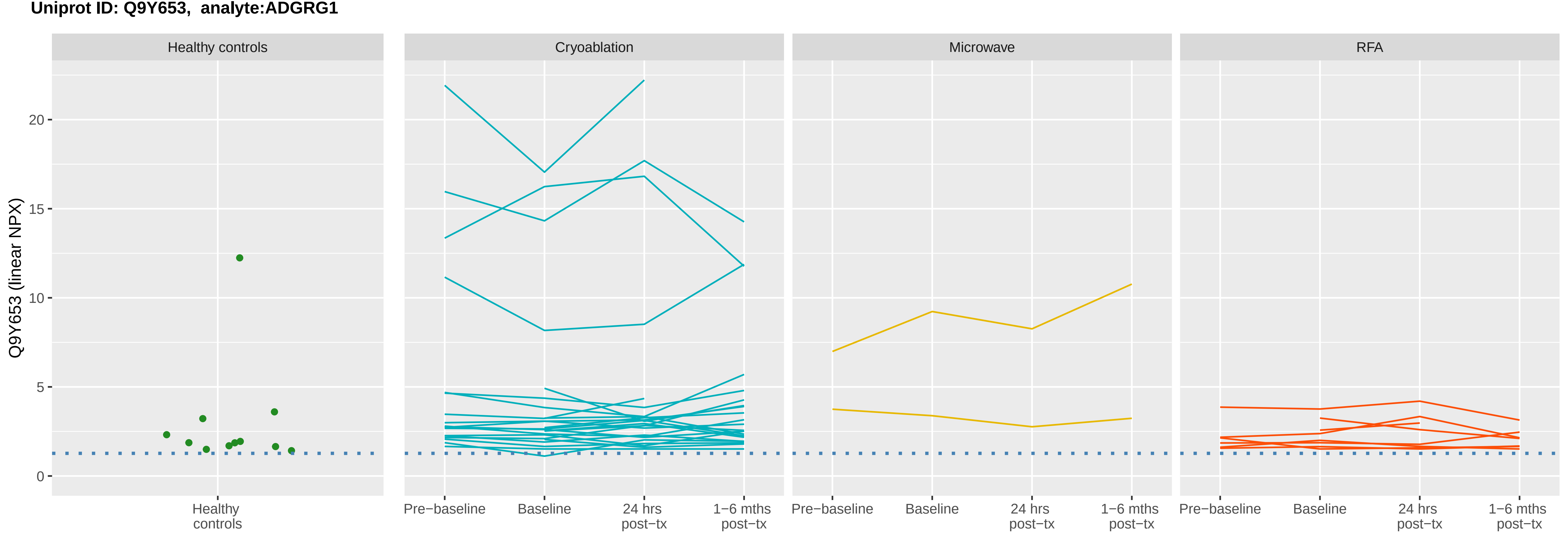

Uniprot ID: Q9Y653, analyte:ADGRG1
Healthy controls
Cryoablation
Microwave
RFA
Q9Y653 (linear NPX)
0
5
10
15
20
Healthy controls
Pre−baseline
Baseline
24 hrs post−tx
1−6 mths post−tx

Uniprot ID: P13500, analyte:MCP−1

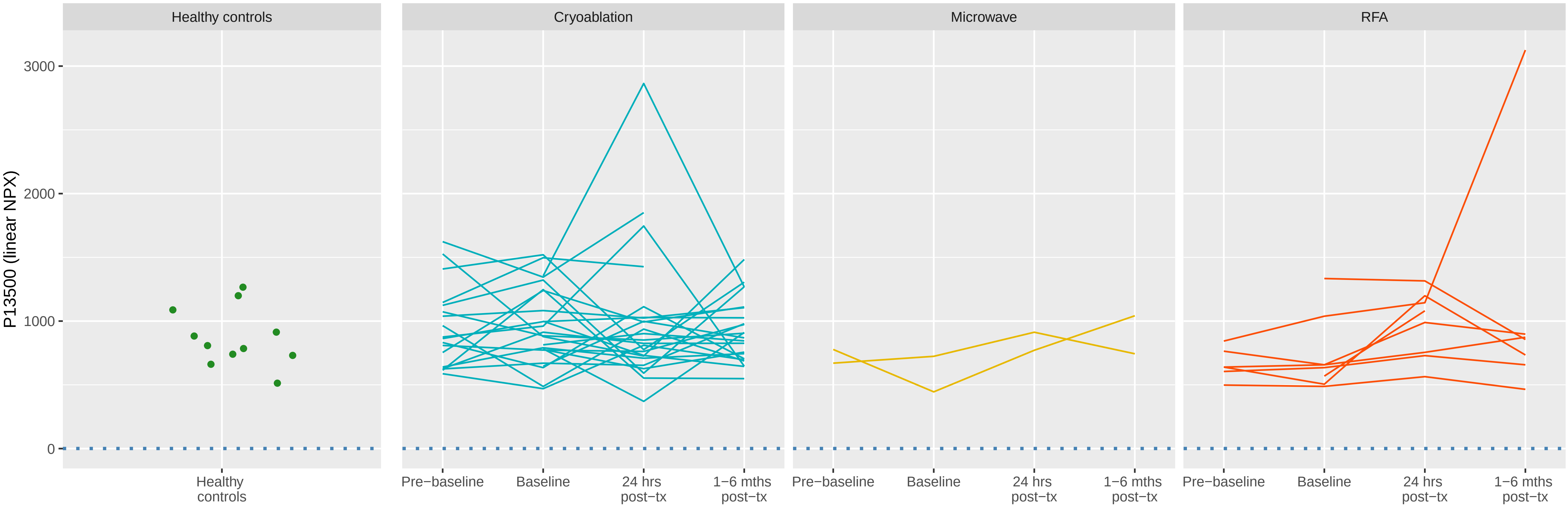

Uniprot ID: O95727, analyte:CRTAM

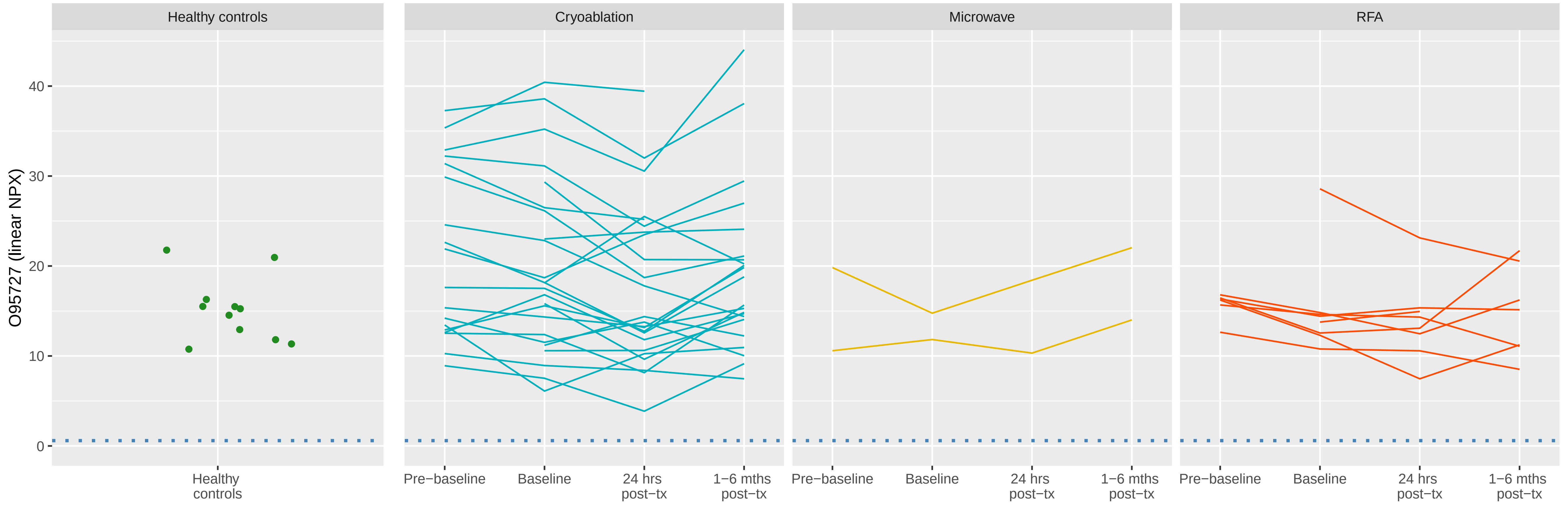

Uniprot ID: O14625, analyte:CXCL11

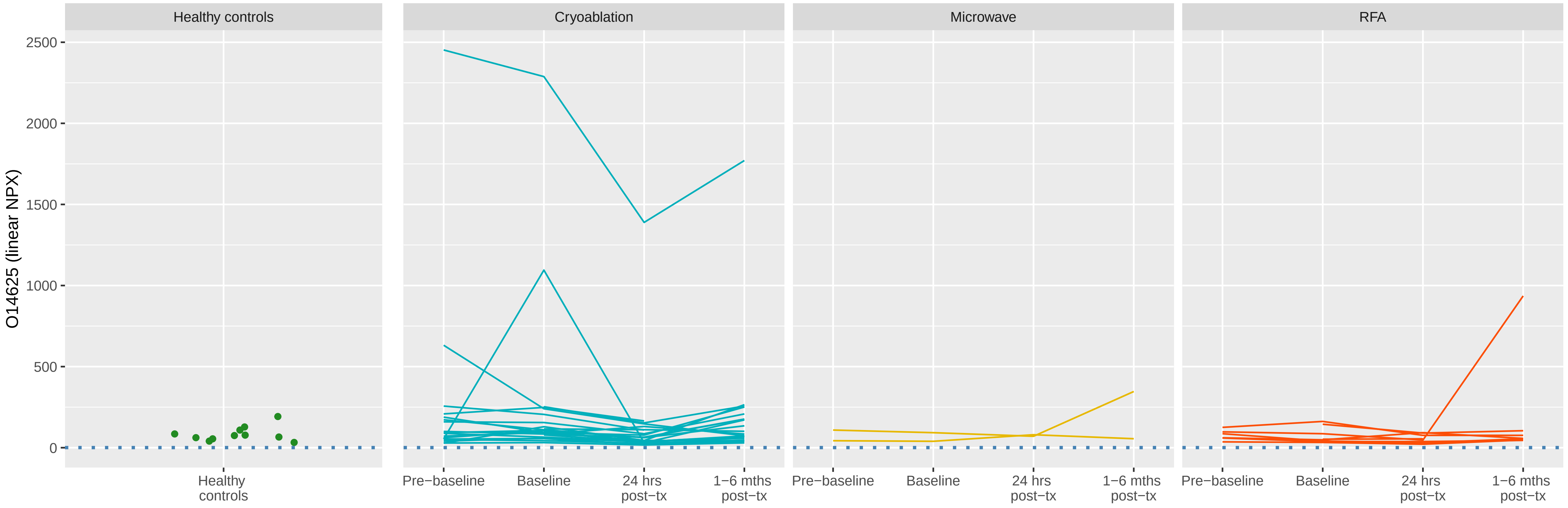

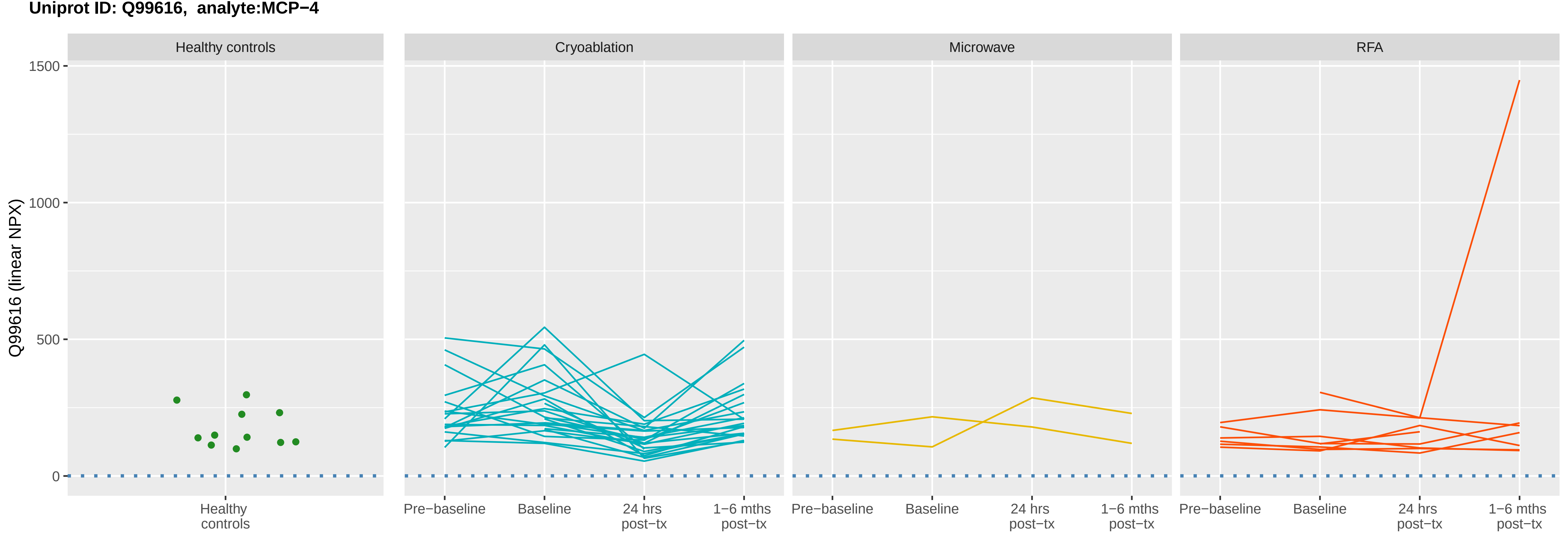

Uniprot ID: Q99616, analyte:MCP−4
Healthy controls
Cryoablation
Microwave
RFA
Q99616 (linear NPX)
1500
1000
500
0
Healthy controls
Pre−baseline
Baseline
24 hrs post−tx
1−6 mths post−tx

Uniprot ID: P50591, analyte:TRAIL

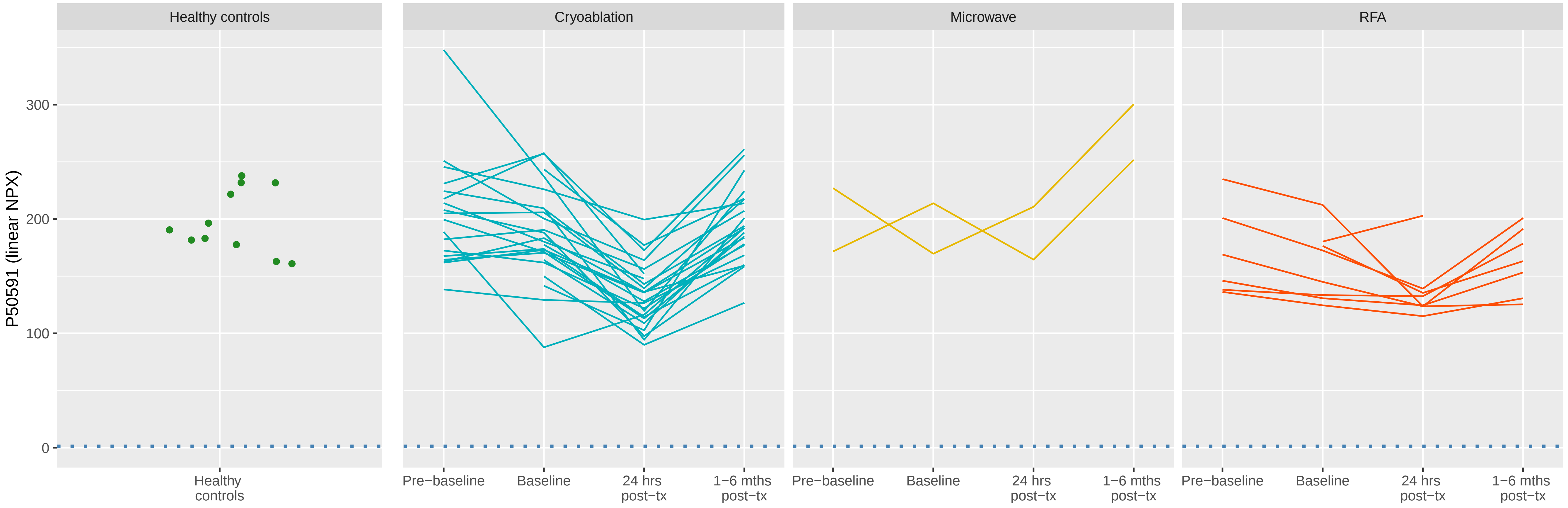

Uniprot ID: P09038, analyte:FGF2

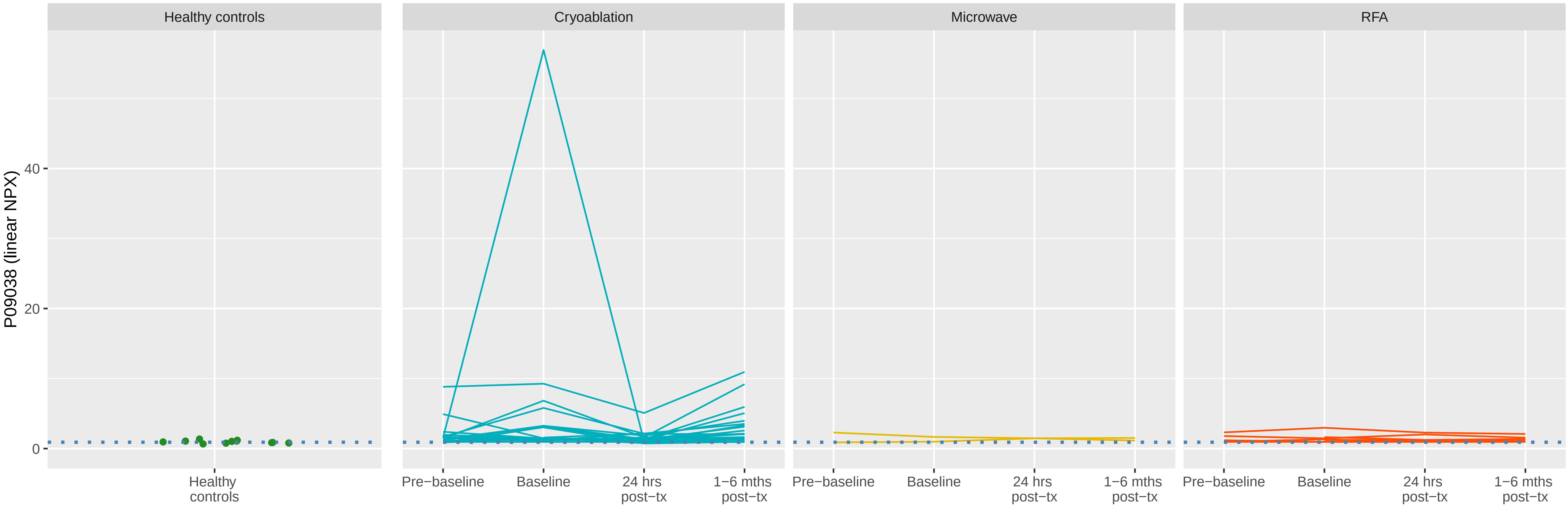

Uniprot ID: Q07325, analyte:CXCL9

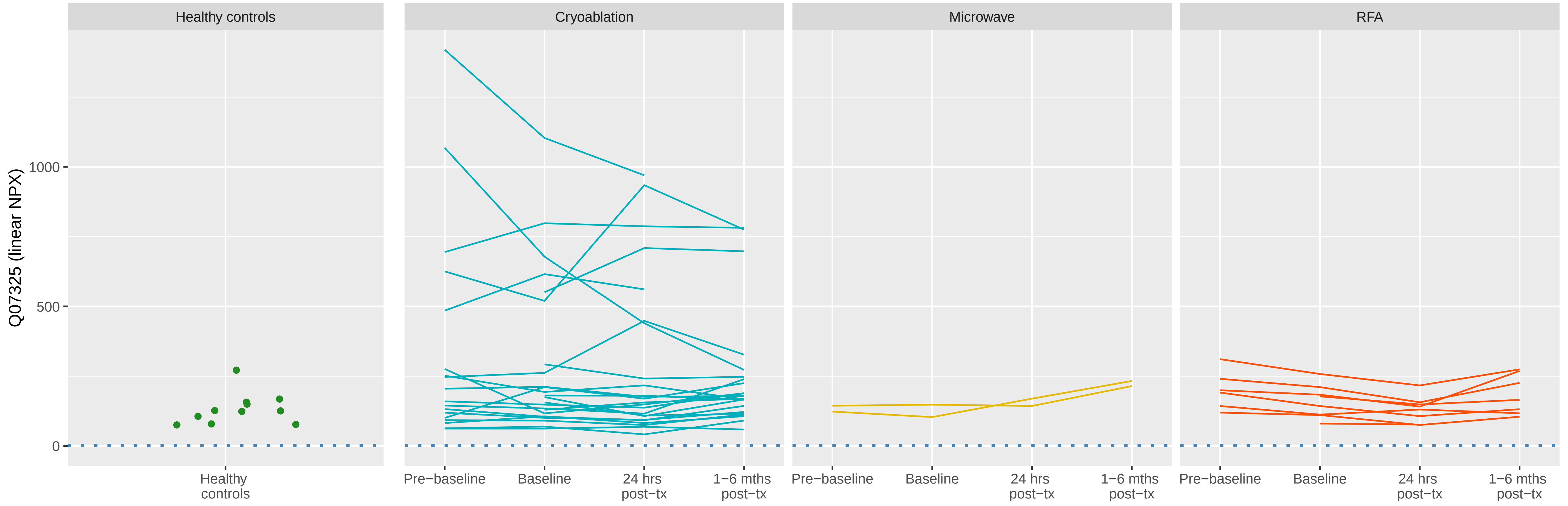

Uniprot ID: P01732, analyte:CD8A

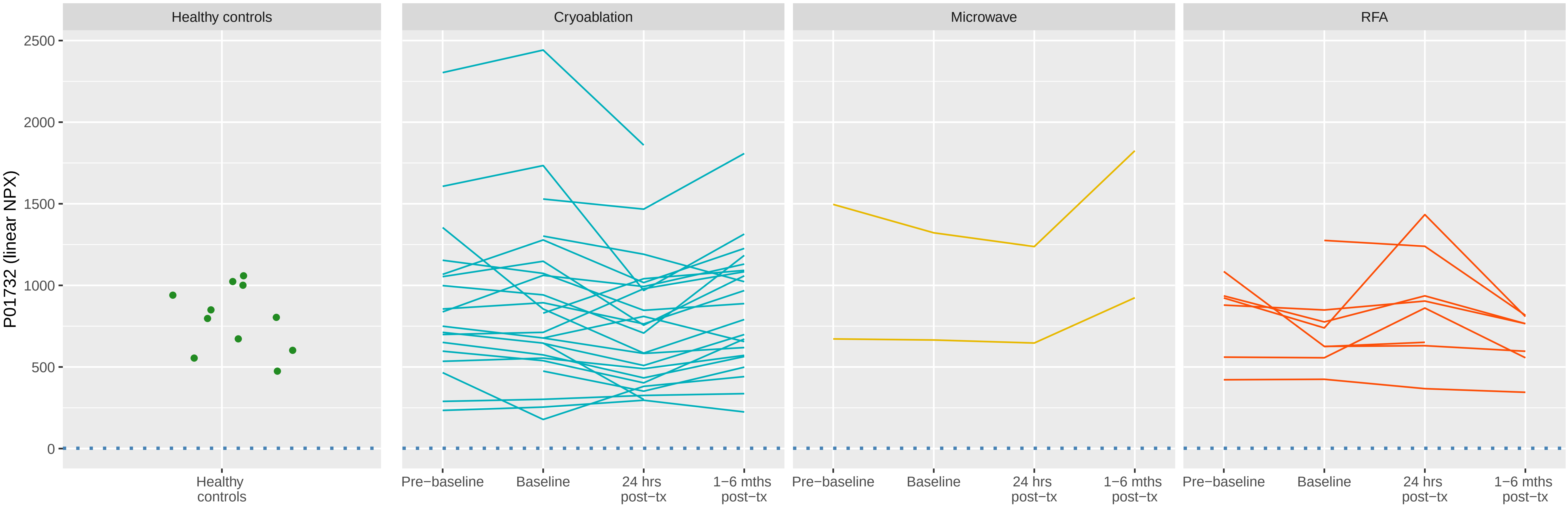

Uniprot ID: Q16790, analyte:CAIX

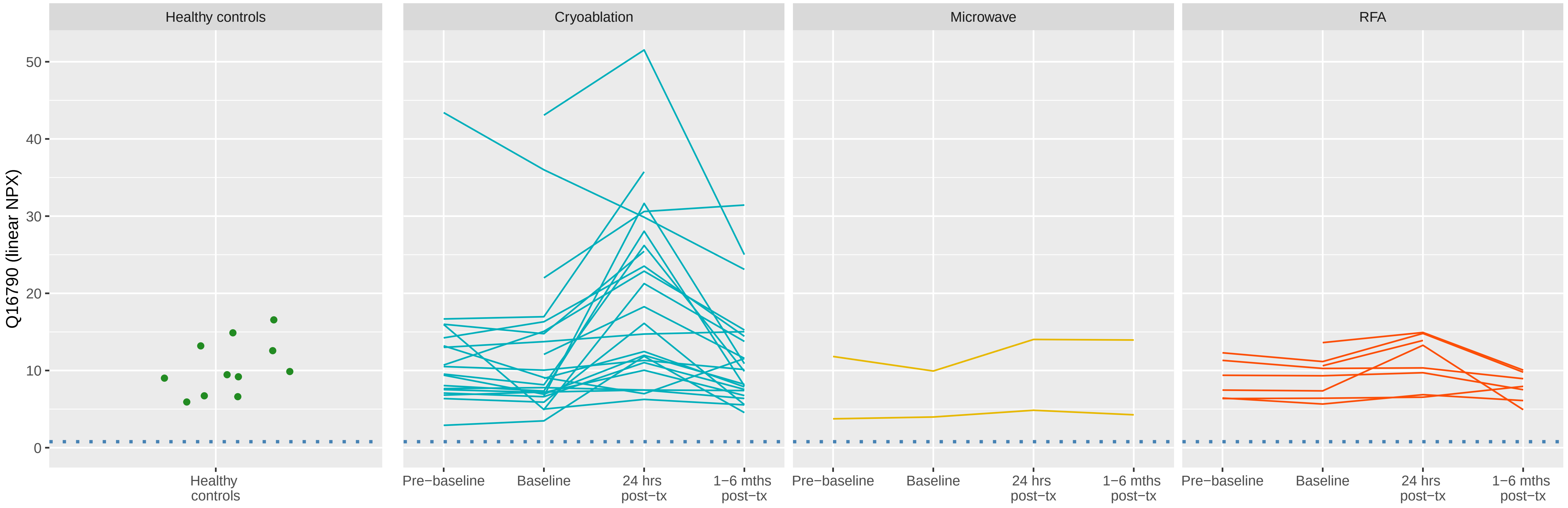

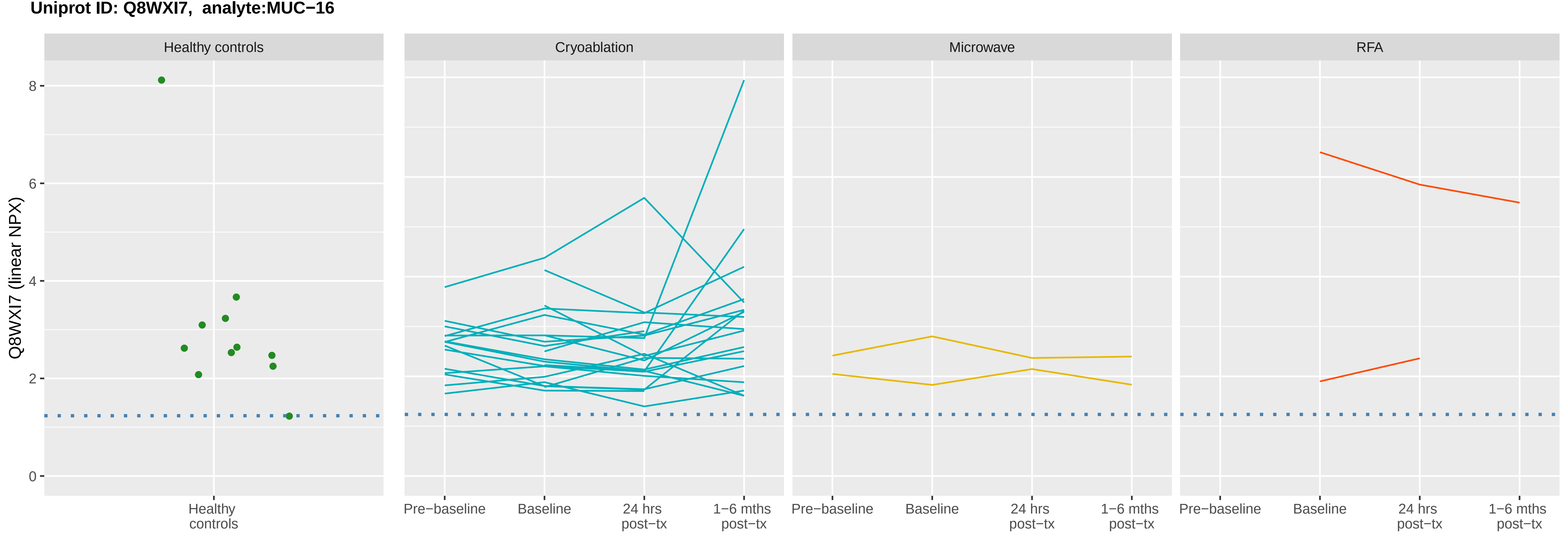

Uniprot ID: Q8WXI7, analyte:MUC−16
Healthy controls
Cryoablation
Microwave
RFA
Q8WXI7 (linear NPX)
8
6
4
2
0
Healthy controls
Pre−baseline
Baseline
24 hrs post−tx
1−6 mths post−tx

Uniprot ID: P00813, analyte:ADA

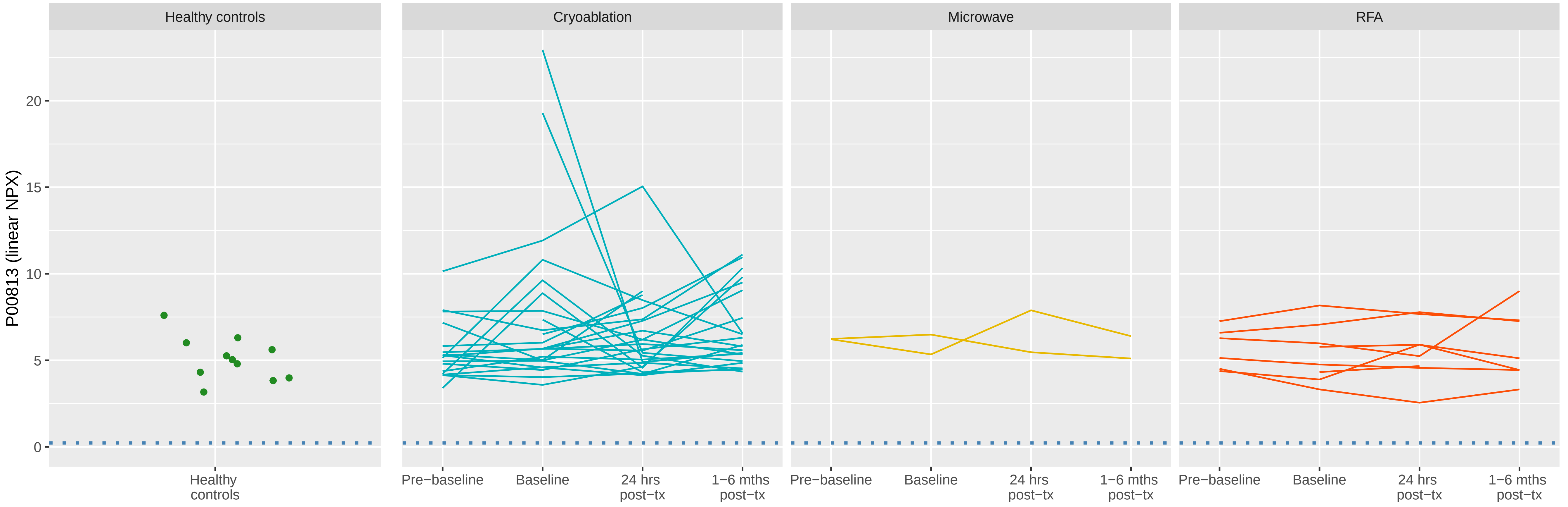

Uniprot ID: P01730, analyte:CD4

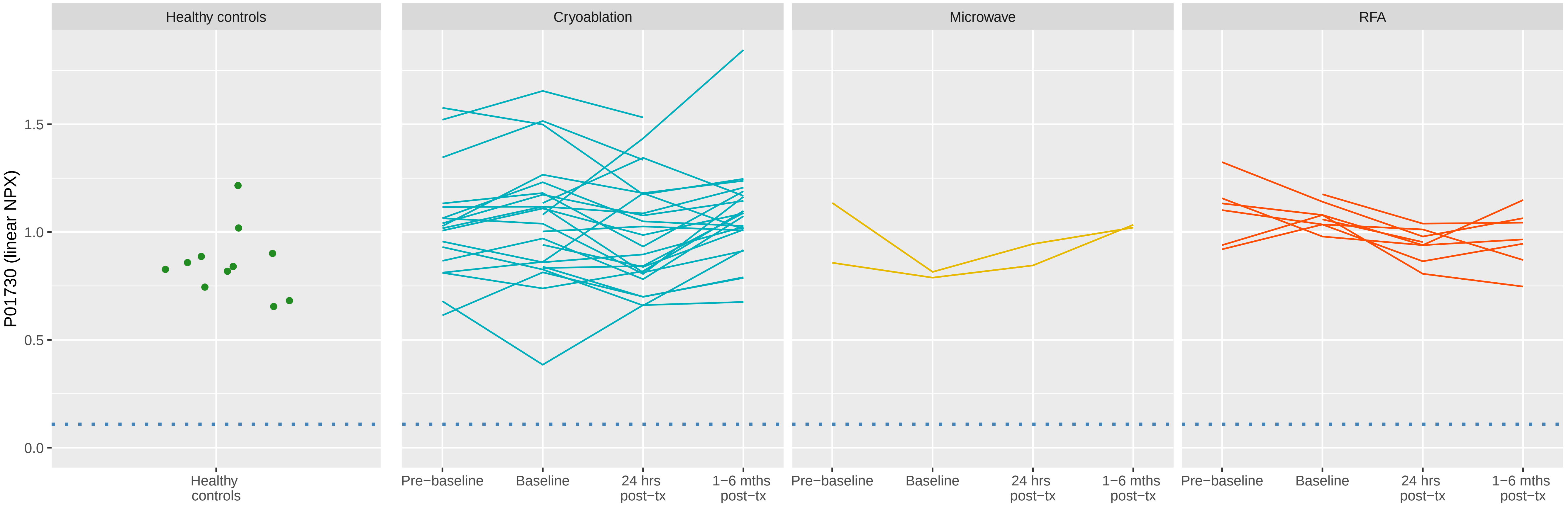

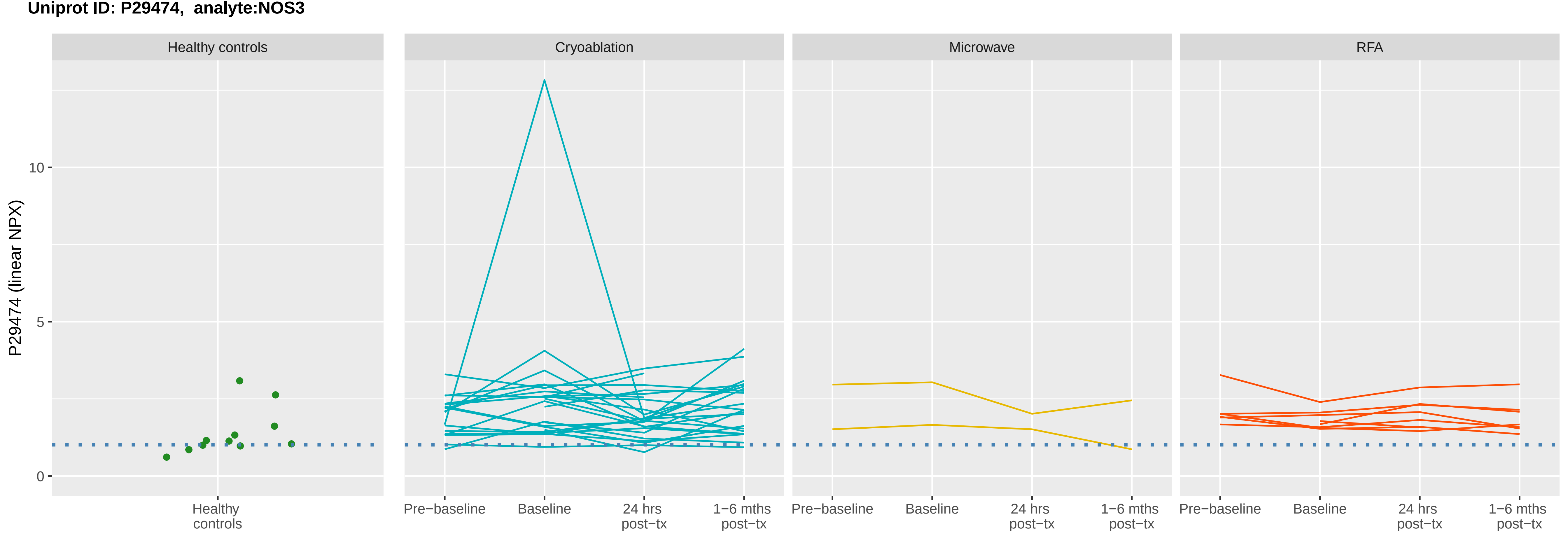

Uniprot ID: P29474, analyte:NOS3
Healthy controls
Cryoablation
Microwave
RFA
P29474 (linear NPX)
10
5
0
Healthy controls
Pre−baseline
Baseline
24 hrs post−tx
1−6 mths post−tx

Uniprot ID: O00182, analyte:Gal−9

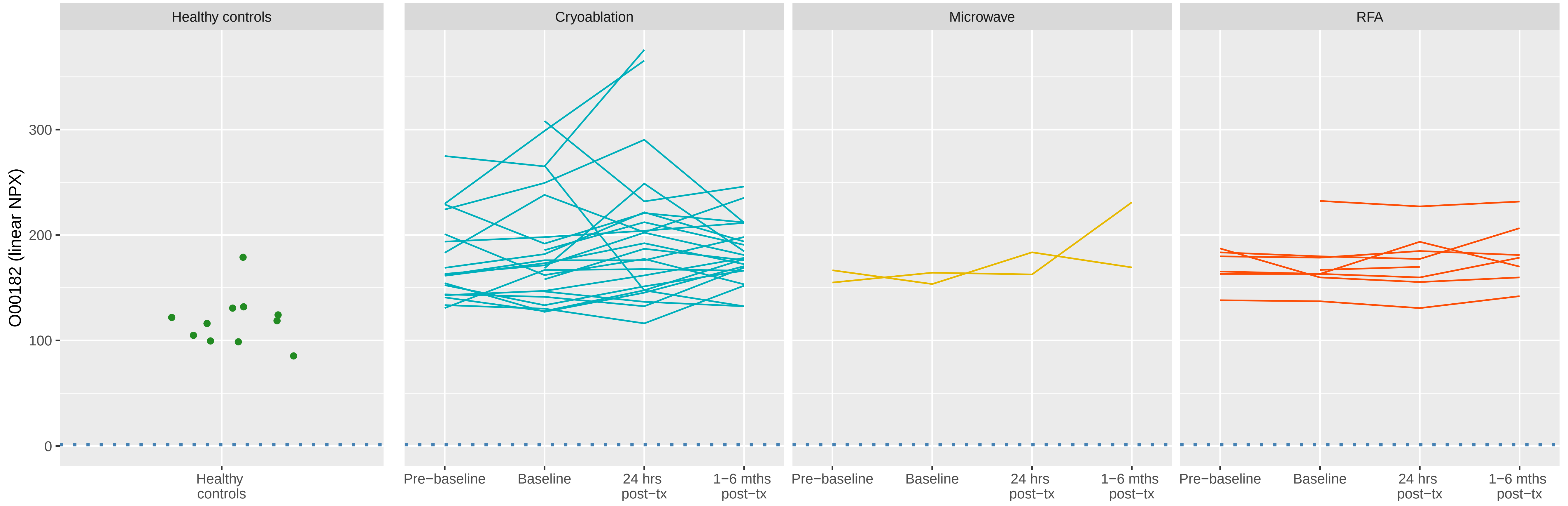

Uniprot ID: P35968, analyte:VEGFR−2

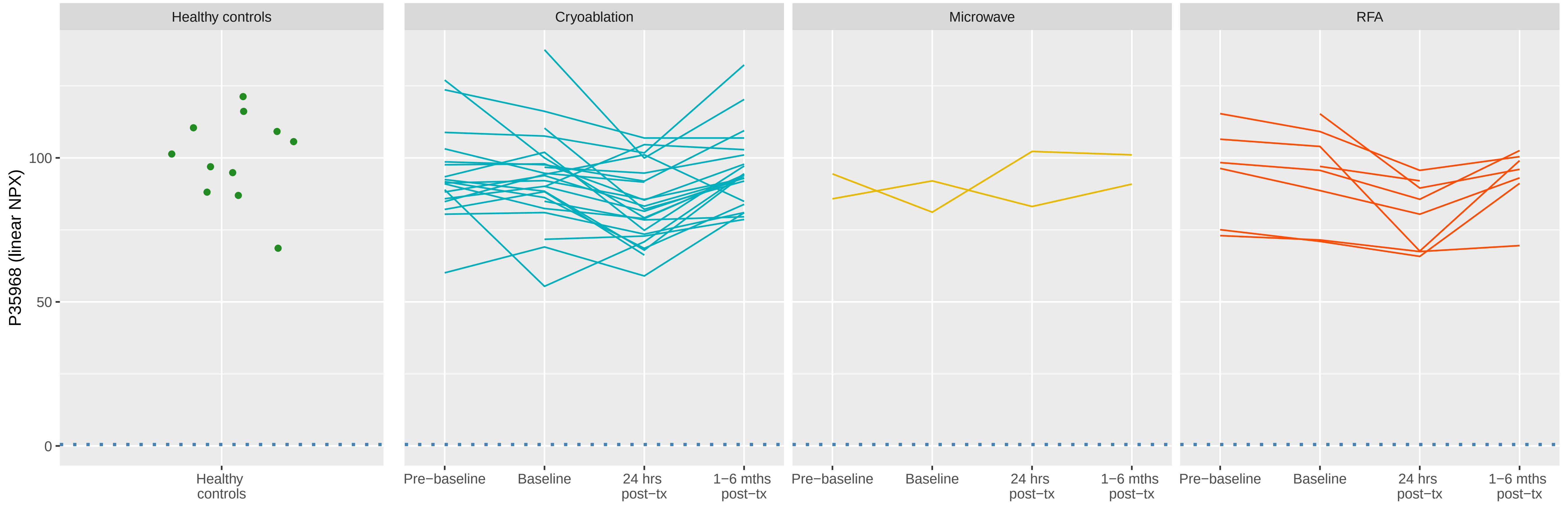

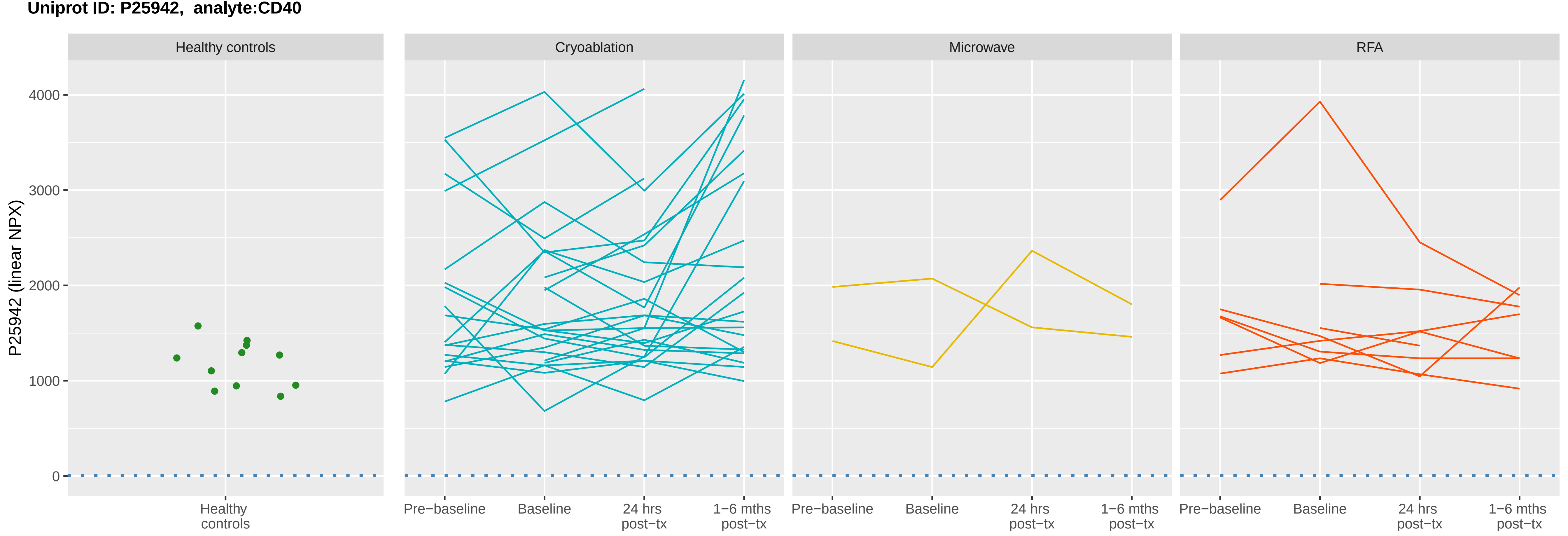
Uniprot ID: P25942, analyte:CD40
Healthy controls
Cryoablation
Microwave
RFA
P25942 (linear NPX)
4000
3000
2000
1000
0
Healthy controls
Pre−baseline
Baseline
24 hrs post−tx
1−6 mths post−tx

Uniprot ID: Q14116, analyte:IL18

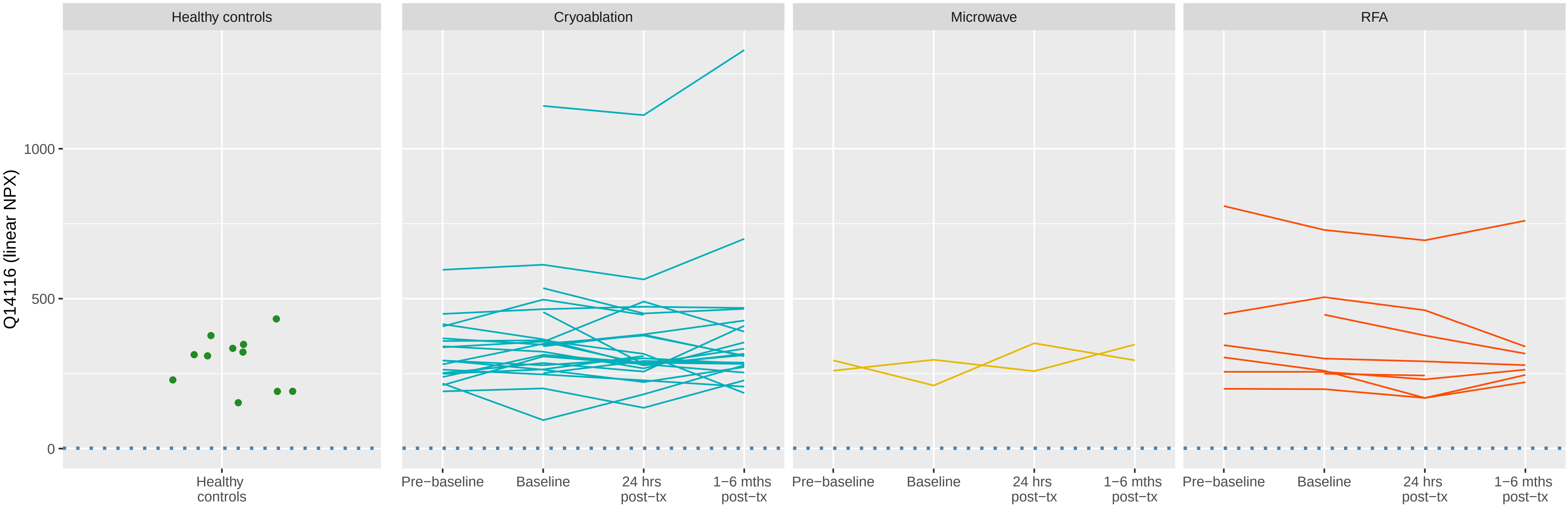

Uniprot ID: P20718, analyte:GZMH

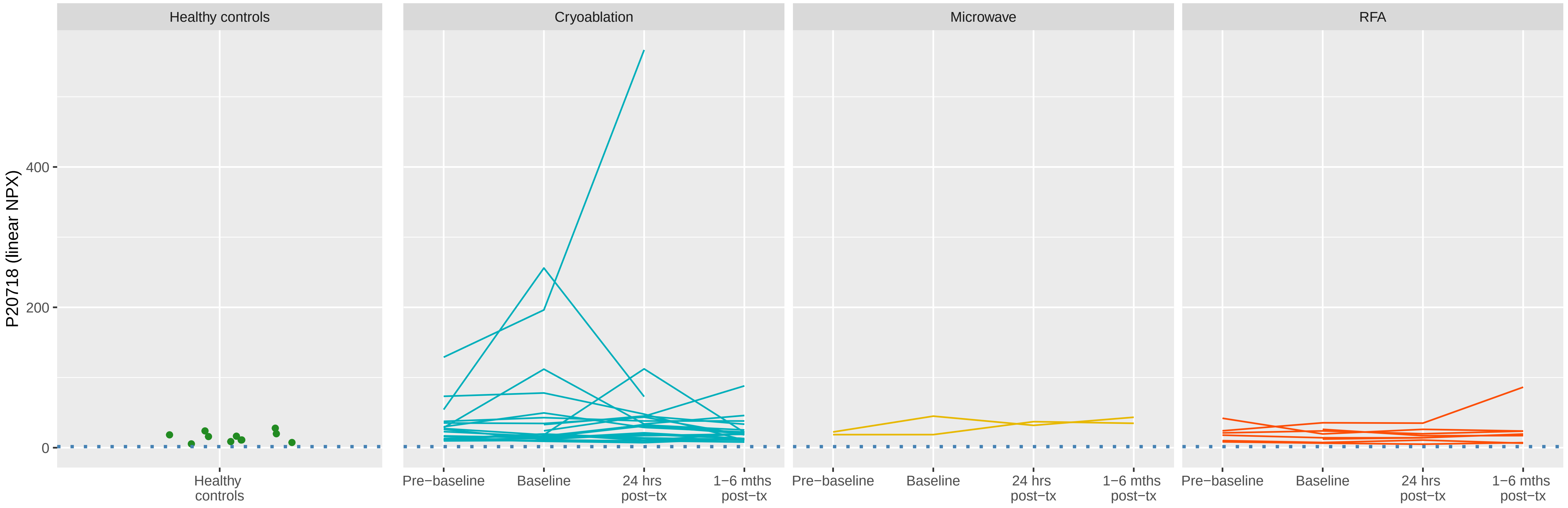

Uniprot ID: P43629, analyte:KIR3DL1

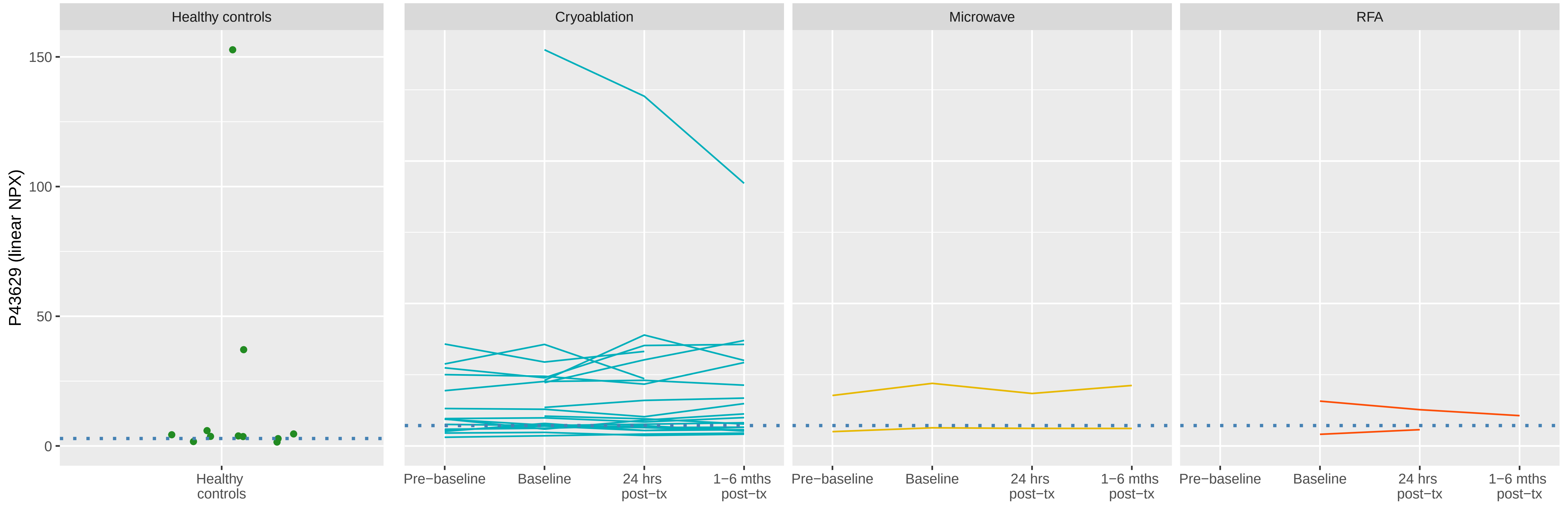

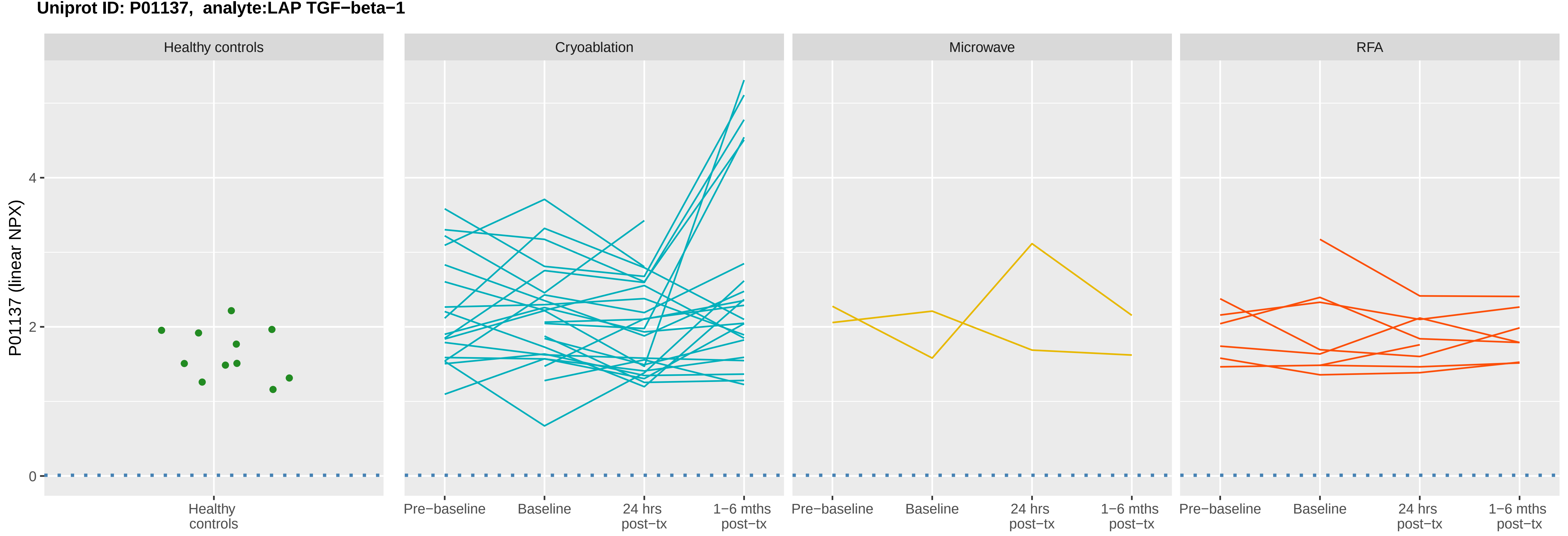

Uniprot ID: P01137, analyte:LAP TGF−beta−1
Healthy controls
Cryoablation
Microwave
RFA
P01137 (linear NPX)
4
2
0
Healthy controls
Pre−baseline
Baseline
24 hrs post−tx
1−6 mths post−tx

Uniprot ID: P09341, analyte:CXCL1

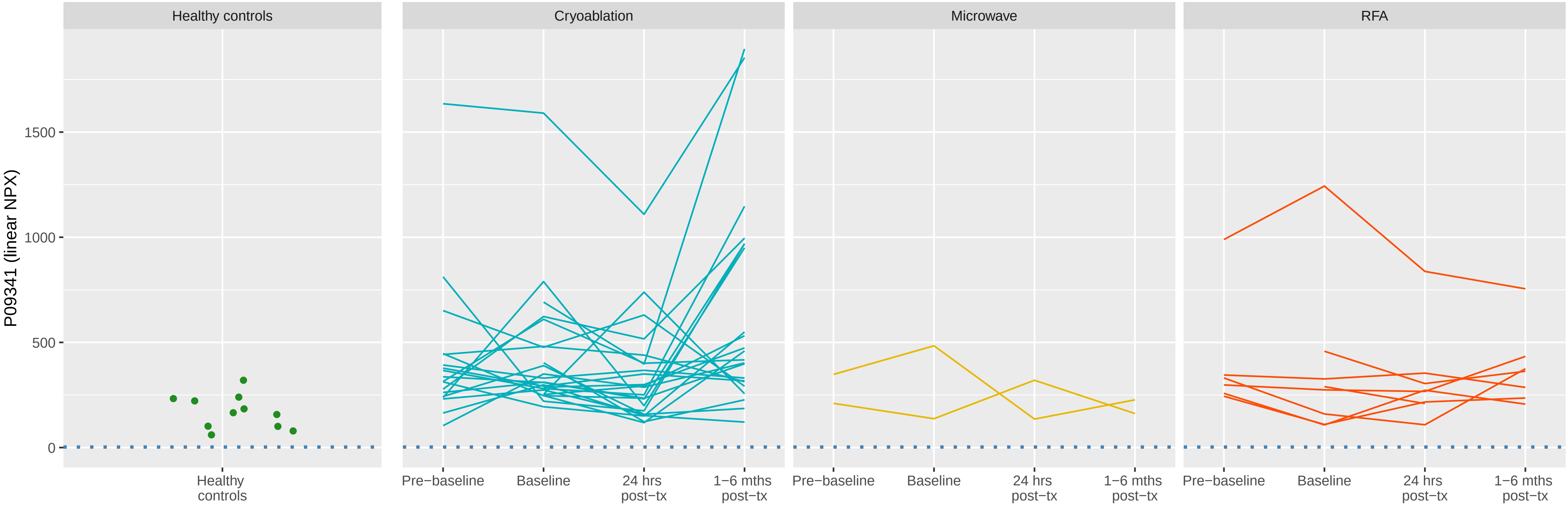

Uniprot ID: O43557, analyte:TNFSF14

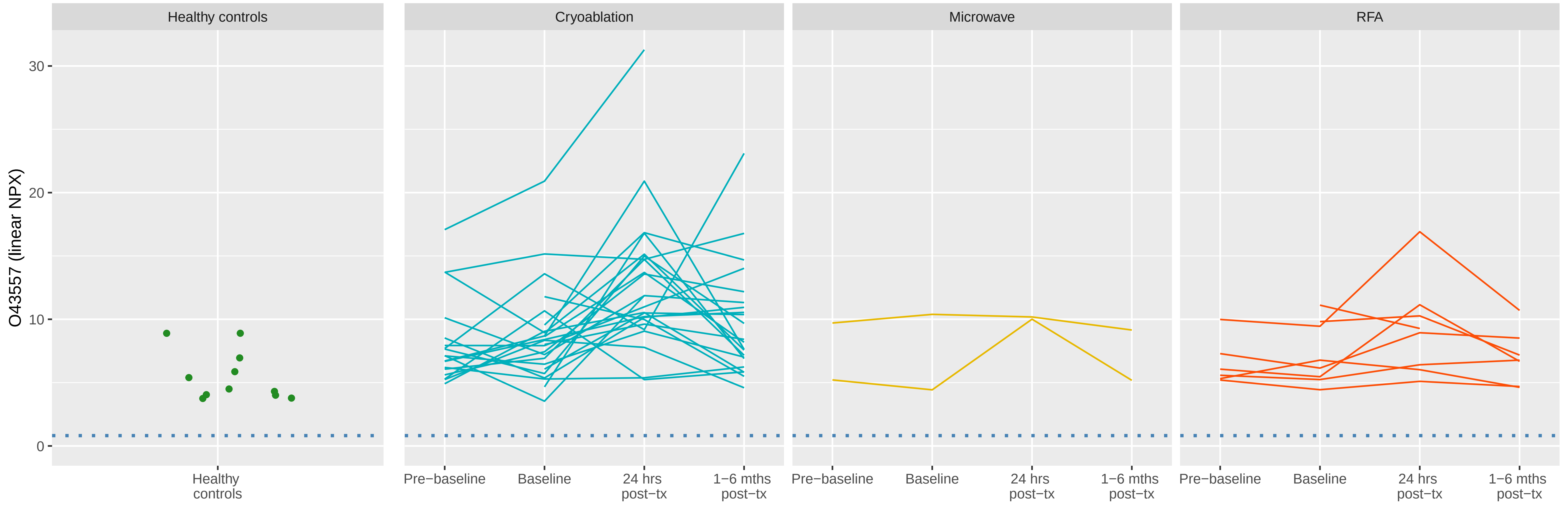

Uniprot ID: O43508, analyte:TWEAK

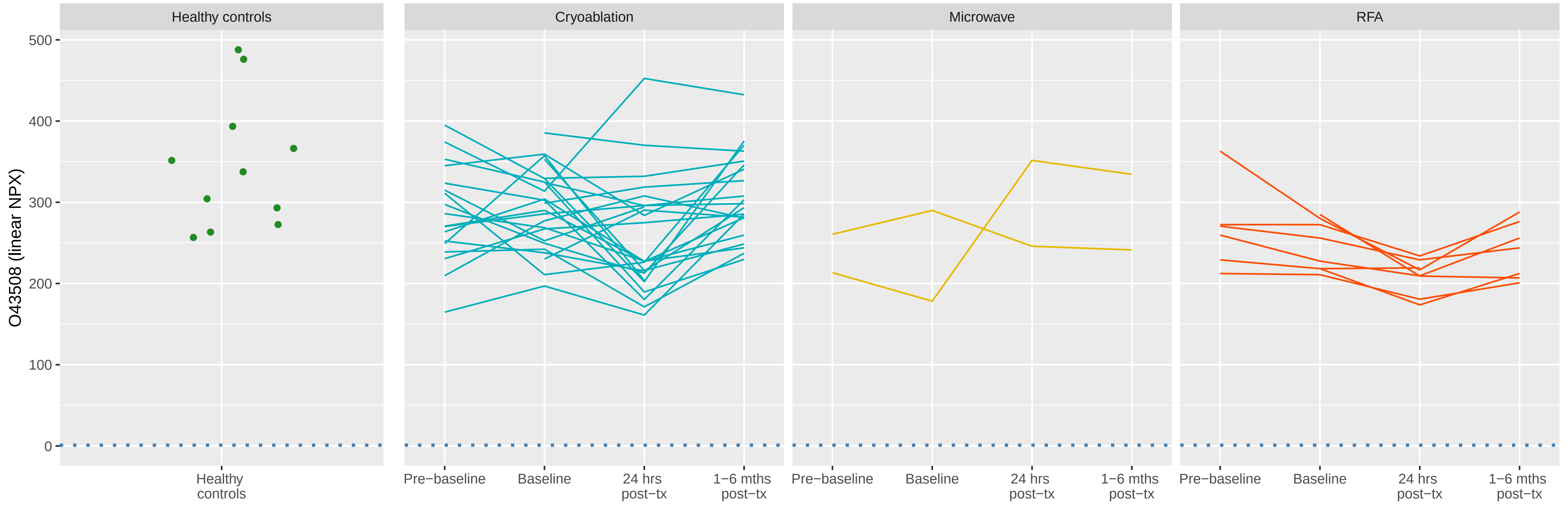

Uniprot ID: P01127, analyte:PDGF subunit B

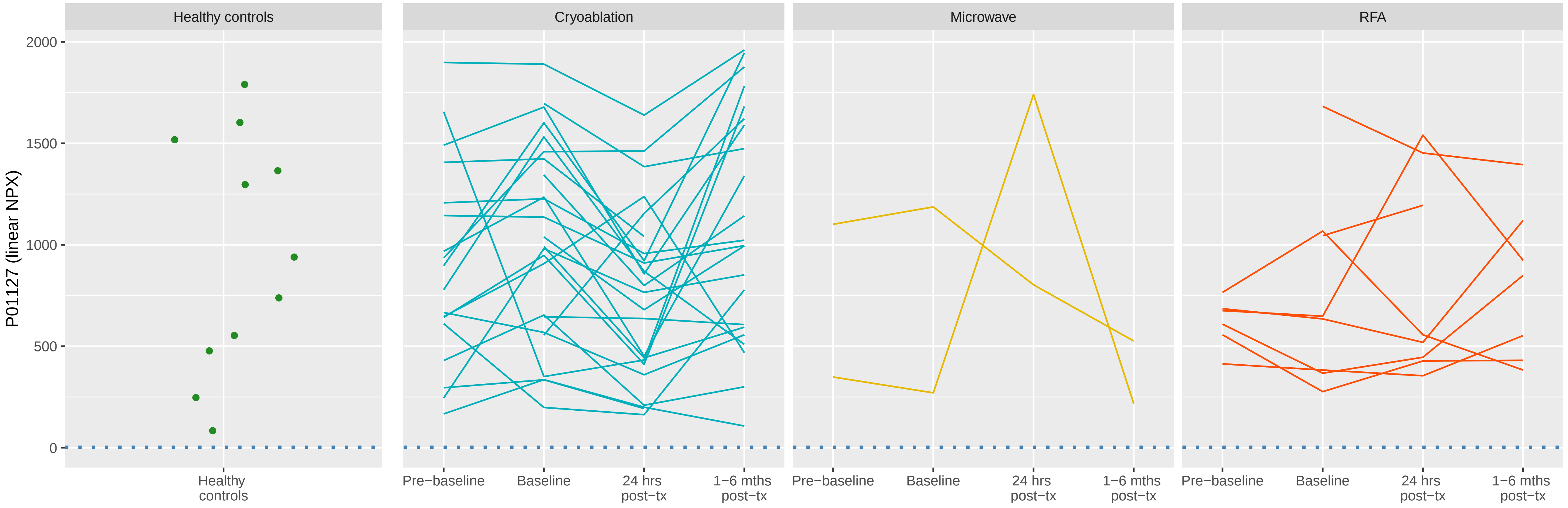

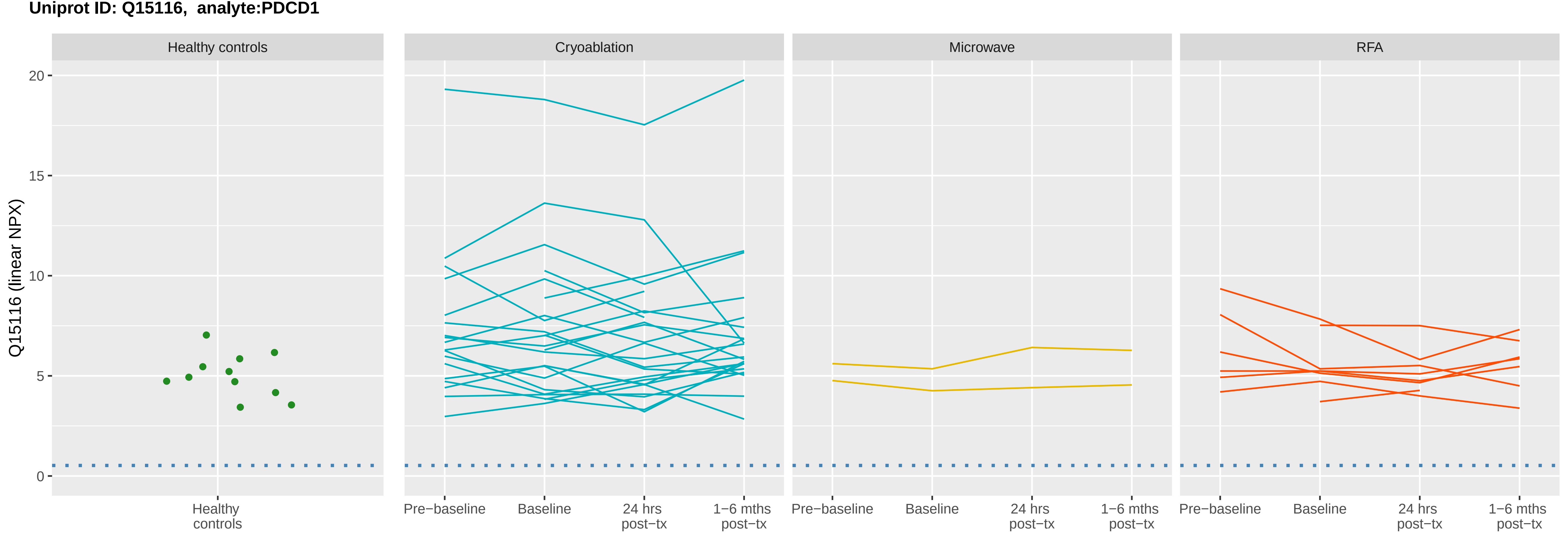
Uniprot ID: Q15116, analyte:PDCD1
Healthy controls
Cryoablation
Microwave
RFA
Q15116 (linear NPX)
20
15
10
5
0
Healthy controls
Pre−baseline
Baseline
24 hrs post−tx
1−6 mths post−tx

Uniprot ID: P48023, analyte:FASLG

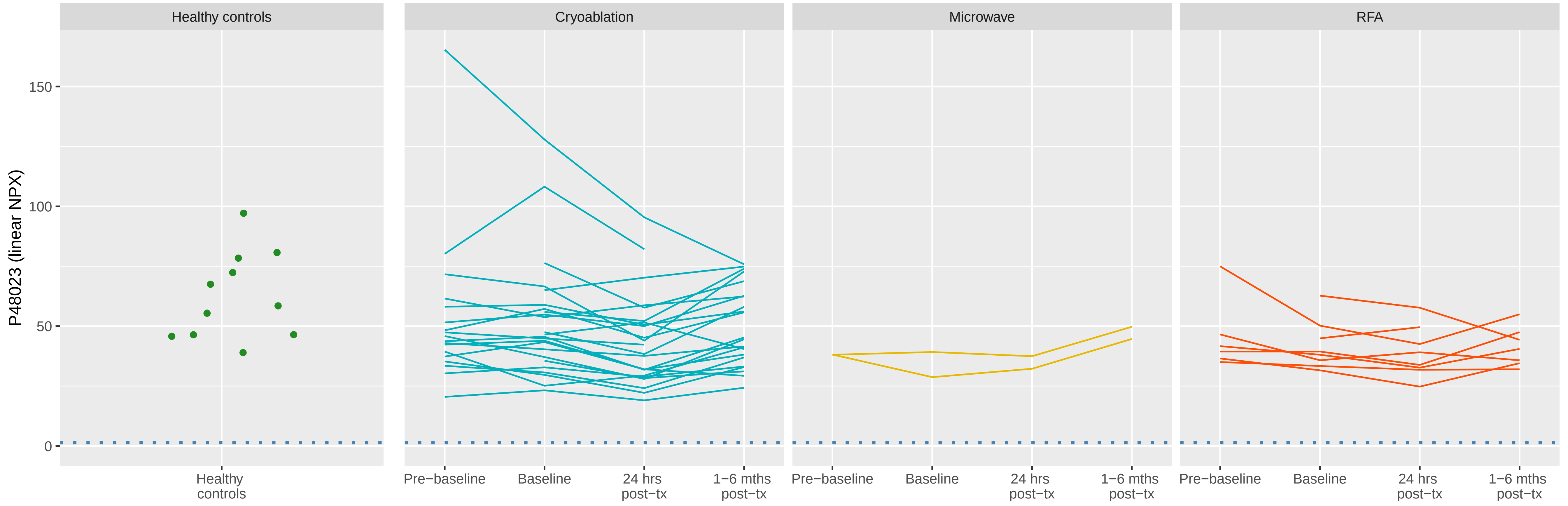

Uniprot ID: Q99731, analyte:CCL19

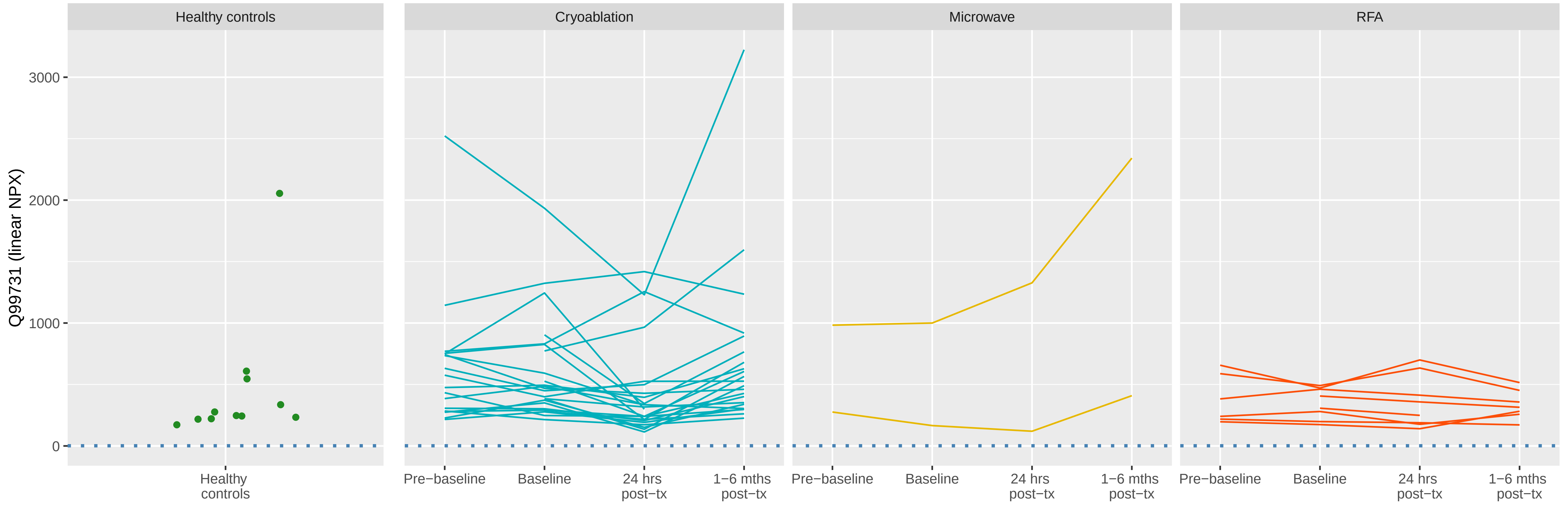

Uniprot ID: P80075, analyte:MCP−2

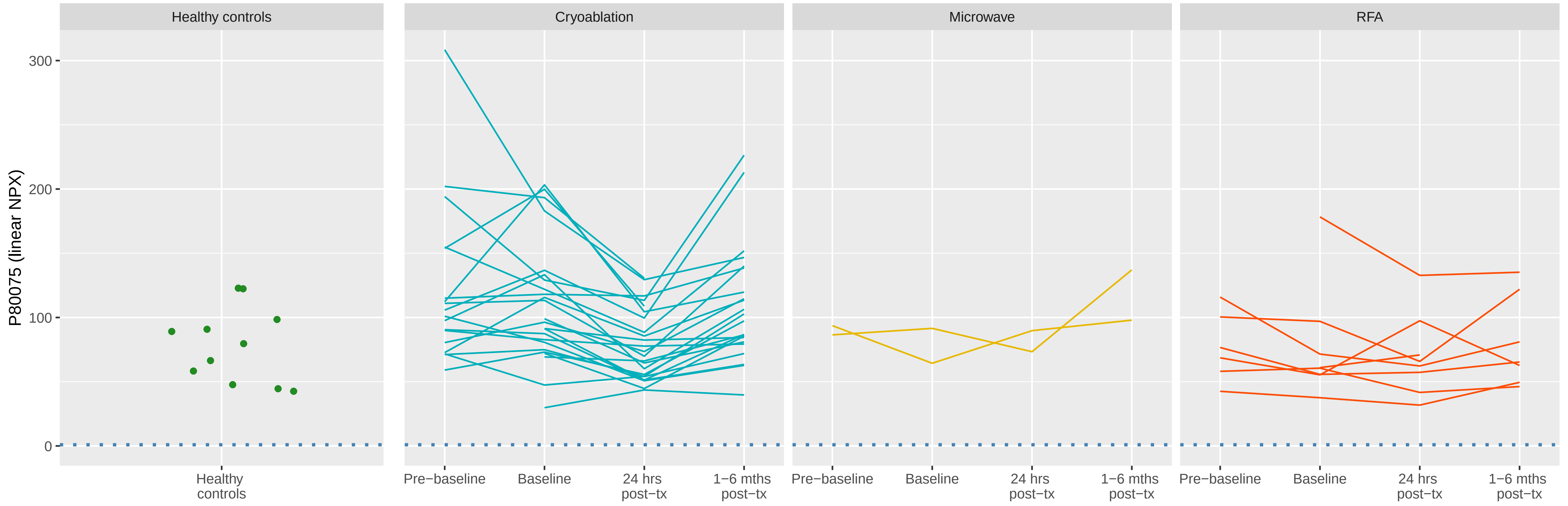

Uniprot ID: P13236, analyte:CCL4

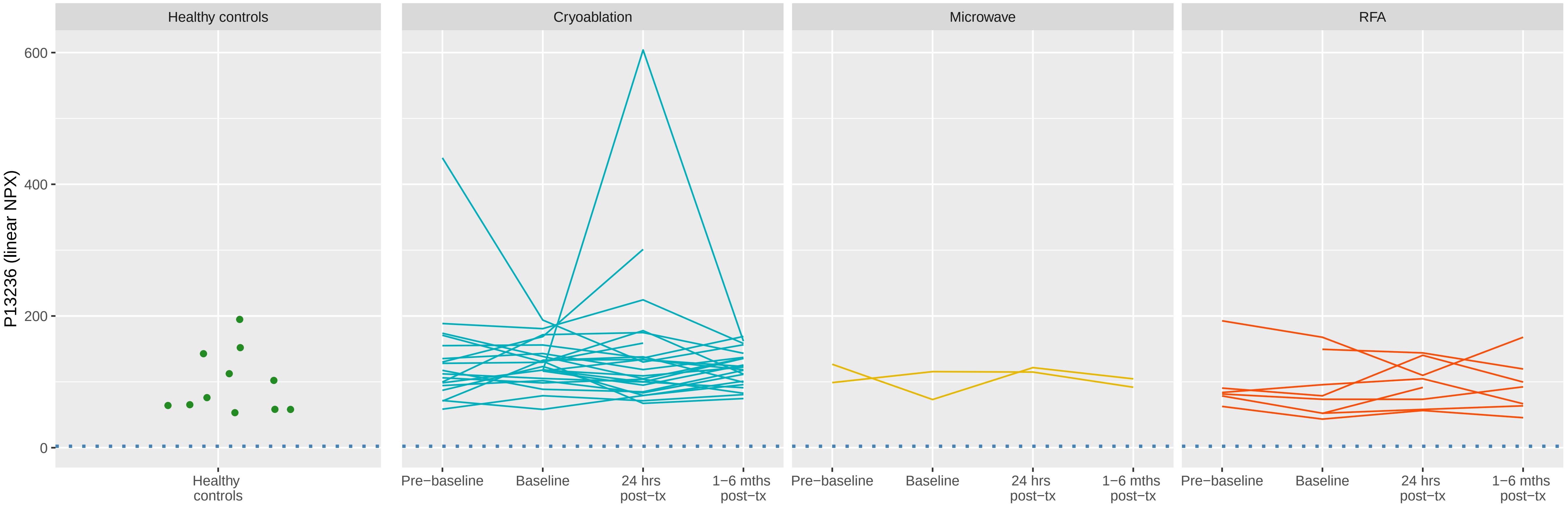

Uniprot ID: P40933, analyte:IL15

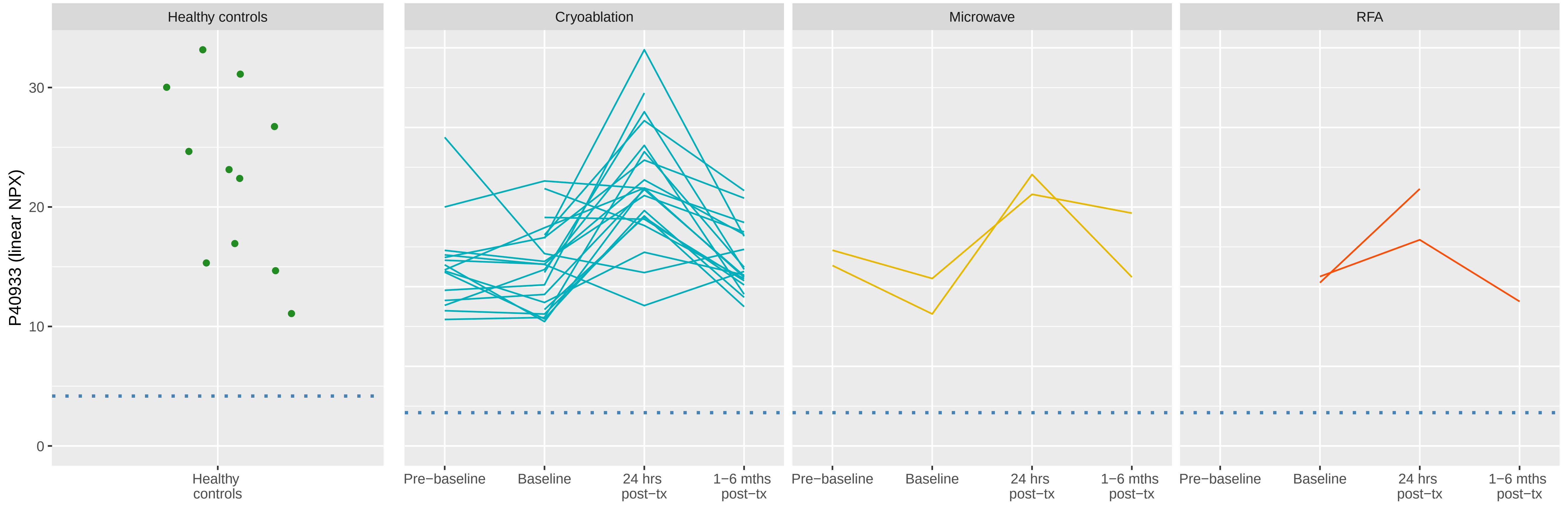

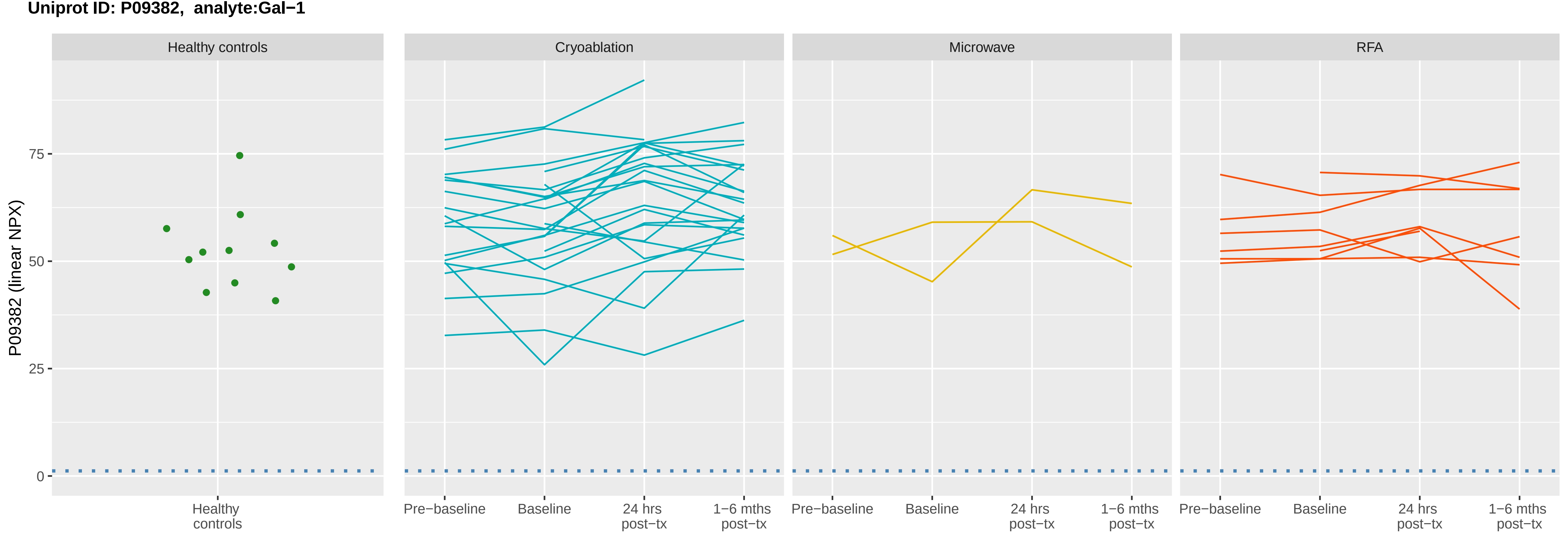
Uniprot ID: P09382, analyte:Gal−1
Healthy controls
Cryoablation
Microwave
RFA
P09382 (linear NPX)
75
50
25
0
Healthy controls
Pre−baseline
Baseline
24 hrs post−tx
1−6 mths post−tx

Uniprot ID: Q9NZQ7, analyte:PD−L1

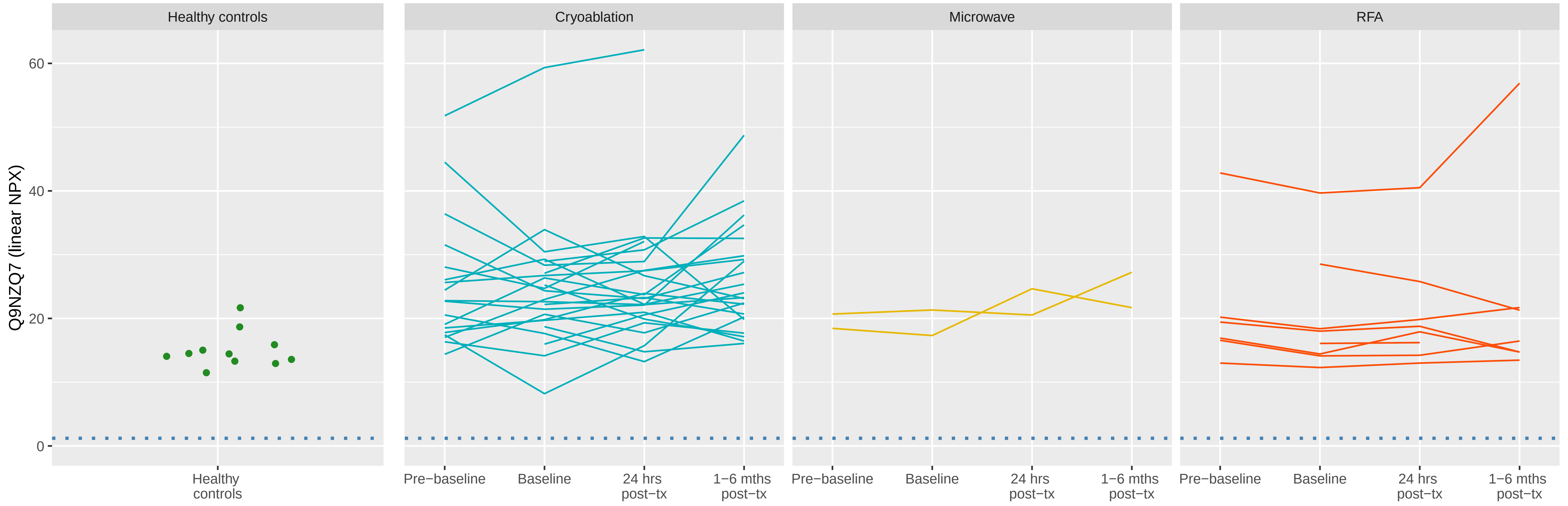

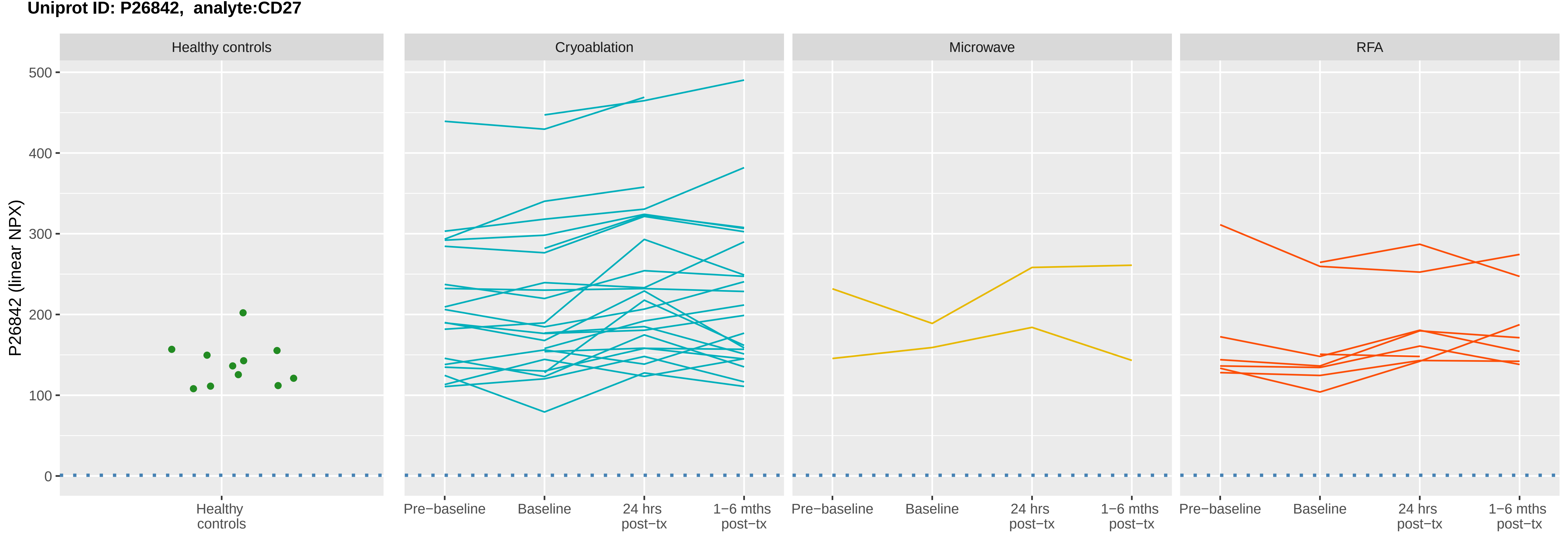
Uniprot ID: P26842, analyte:CD27
Healthy controls
Cryoablation
Microwave
RFA
P26842 (linear NPX)
500
400
300
200
100
0
Healthy controls
Pre−baseline
Baseline
24 hrs post−tx
1−6 mths post−tx

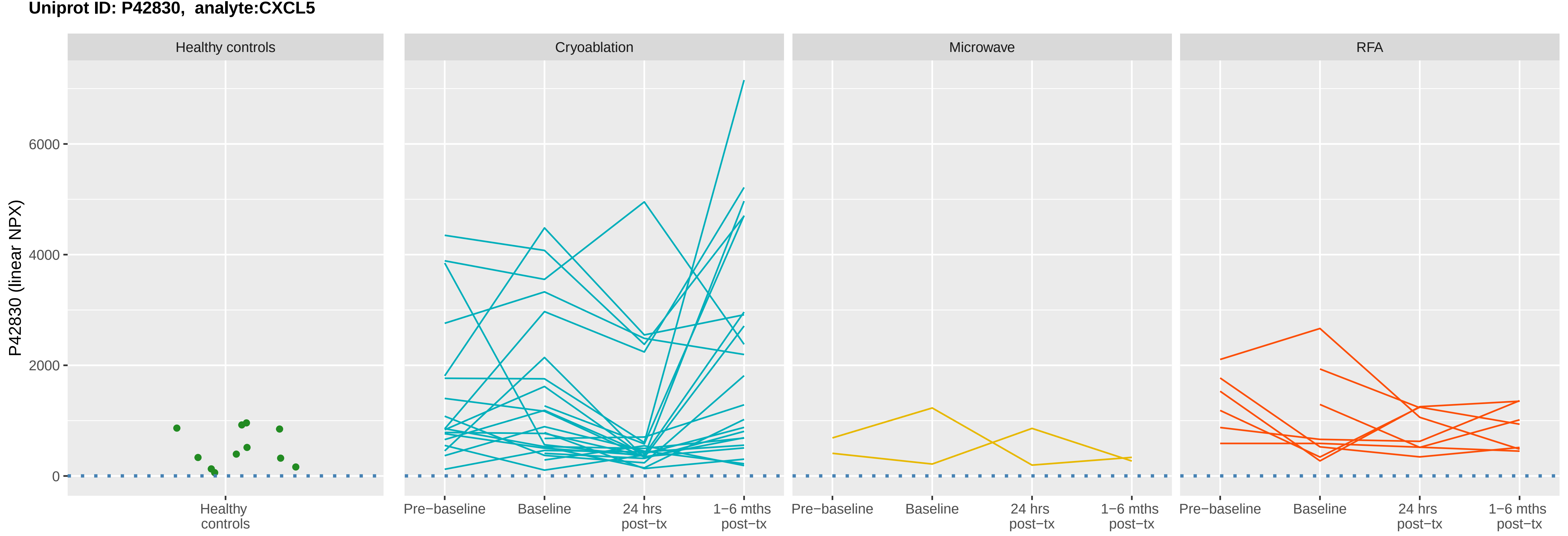
Uniprot ID: P42830, analyte:CXCL5
Healthy controls
Cryoablation
Microwave
RFA
P42830 (linear NPX)
0
2000
4000
6000
Healthy controls
Pre−baseline
Baseline
24 hrs post−tx
1−6 mths post−tx

Uniprot ID: P05113, analyte:IL5

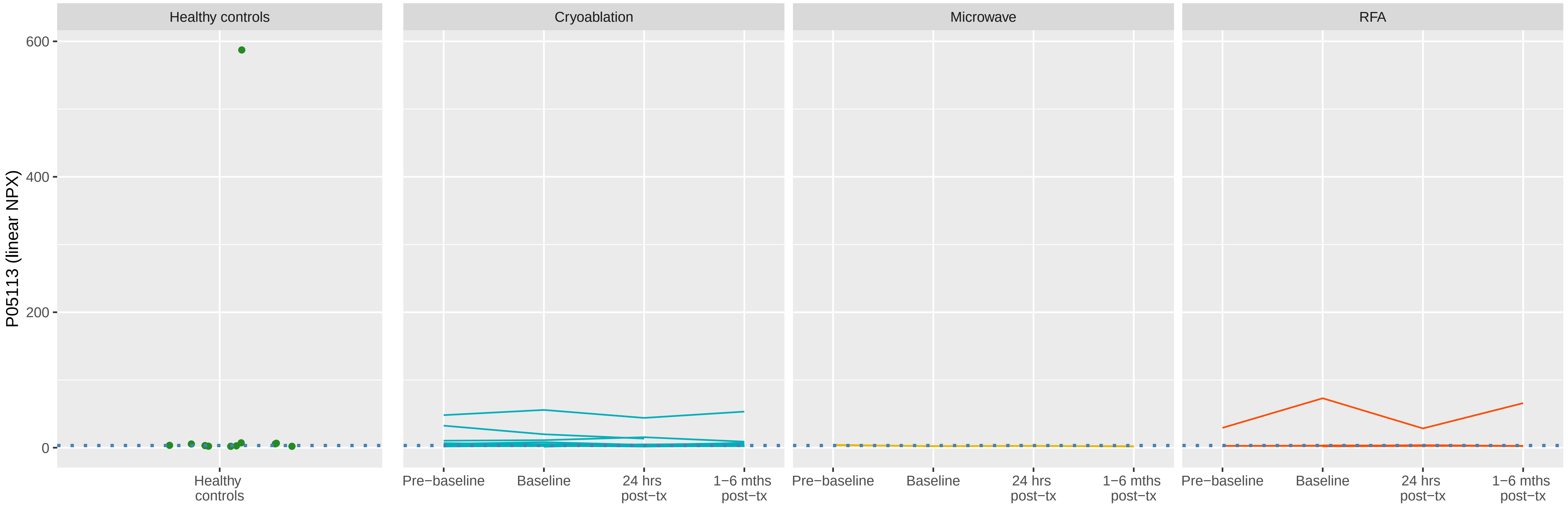

Uniprot ID: P14210, analyte:HGF

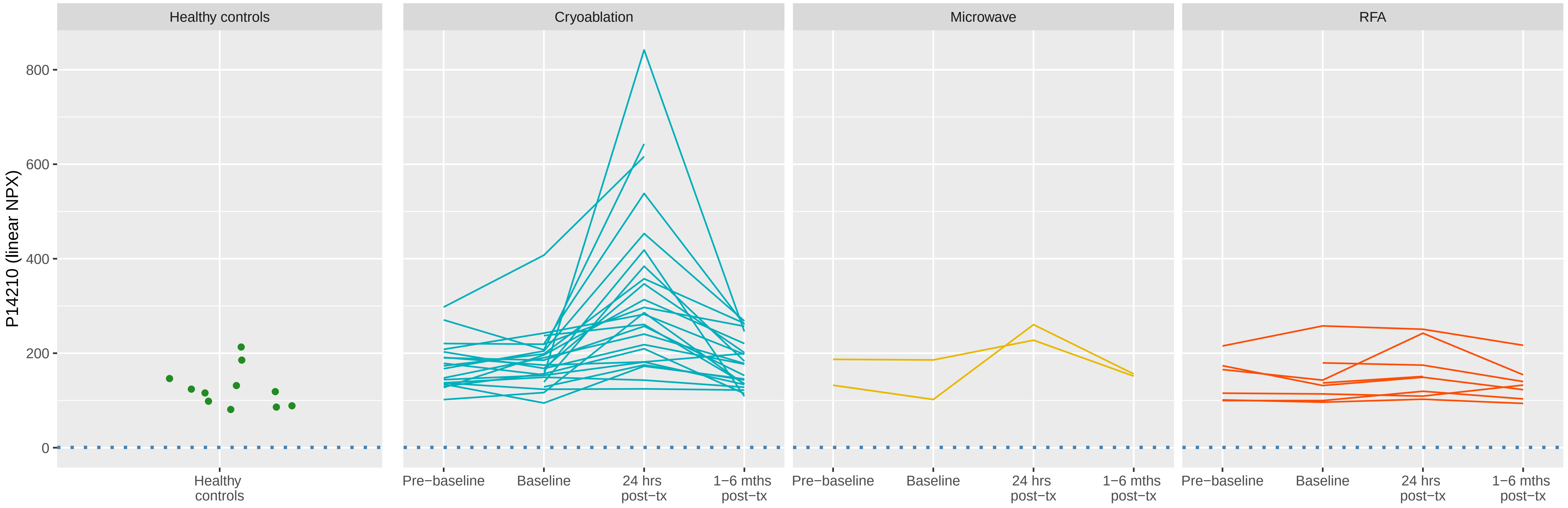

Uniprot ID: P12544, analyte:GZMA

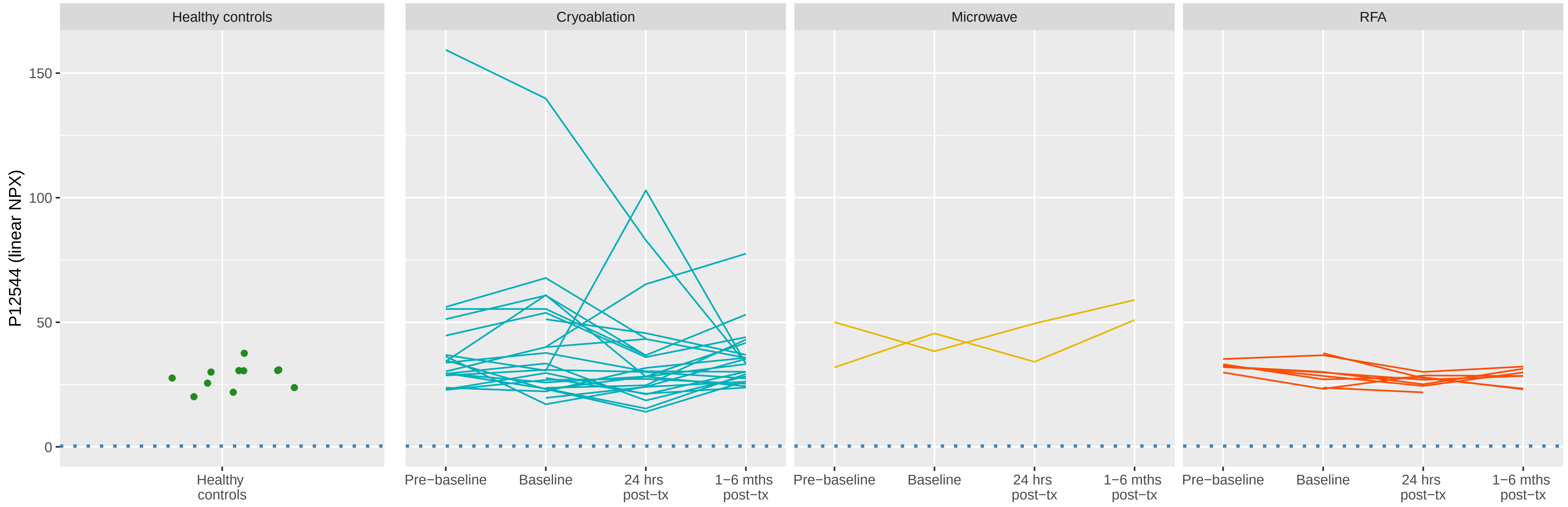

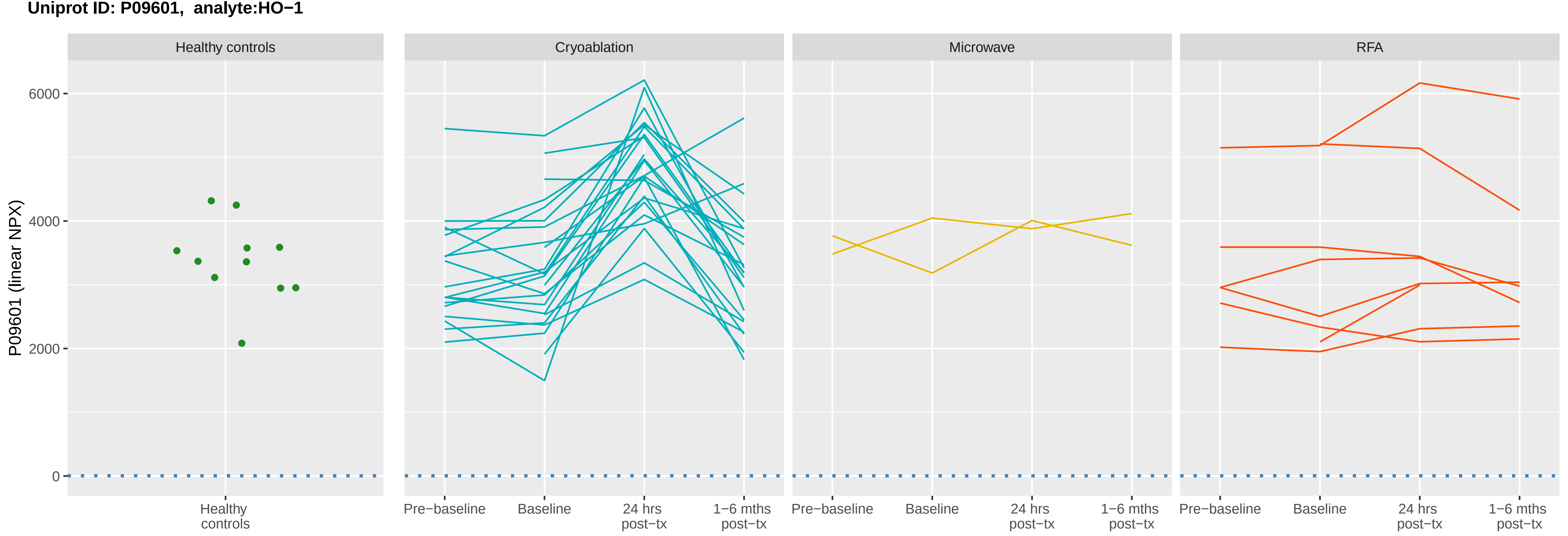

Uniprot ID: P09601, analyte:HO−1
Healthy controls
Cryoablation
Microwave
RFA
P09601 (linear NPX)
6000
4000
2000
0
Healthy controls
Pre−baseline
Baseline
24 hrs post−tx
1−6 mths post−tx

Uniprot ID: P78423, analyte:CX3CL1

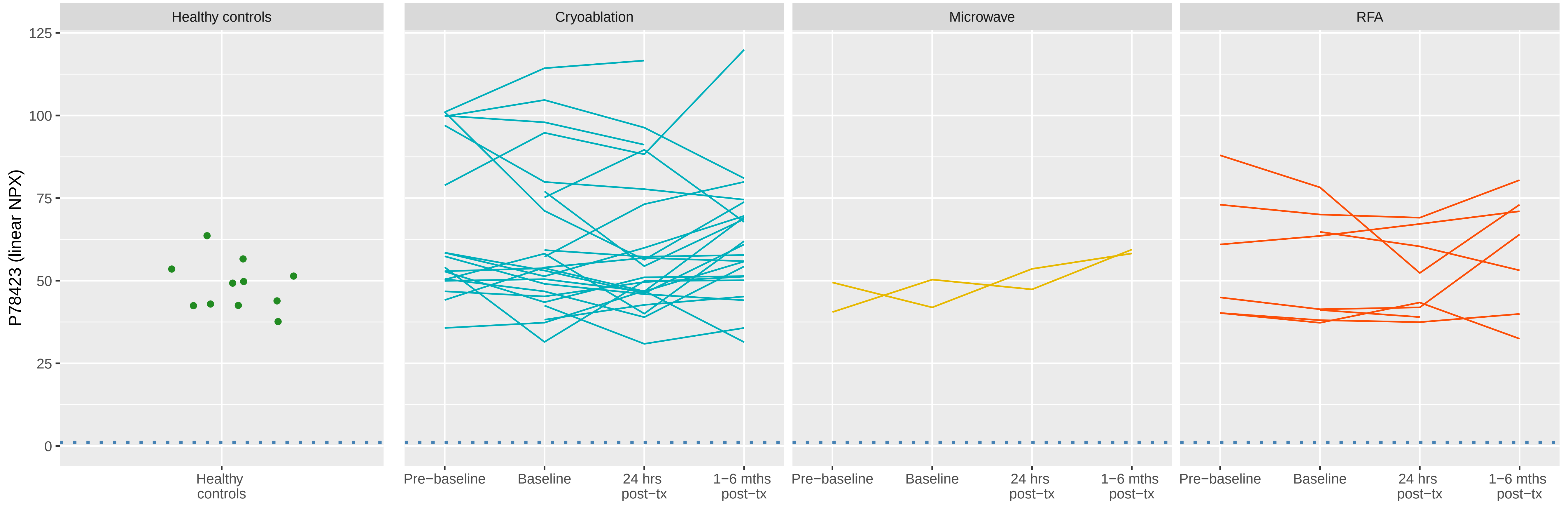

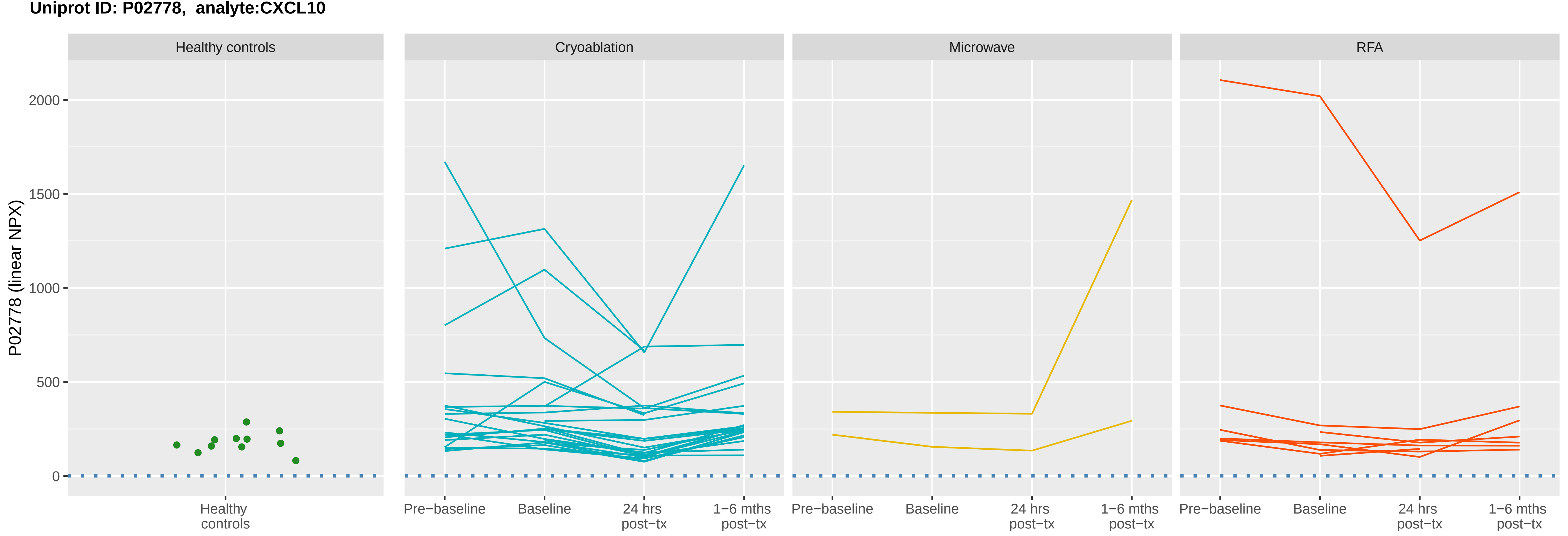
Uniprot ID: P02778, analyte:CXCL10
Healthy controls
Cryoablation
Microwave
RFA
P02778 (linear NPX)
2000
1500
1000
500
0
Healthy controls
Pre−baseline
Baseline
24 hrs post−tx
1−6 mths post−tx

Uniprot ID: P32970, analyte:CD70

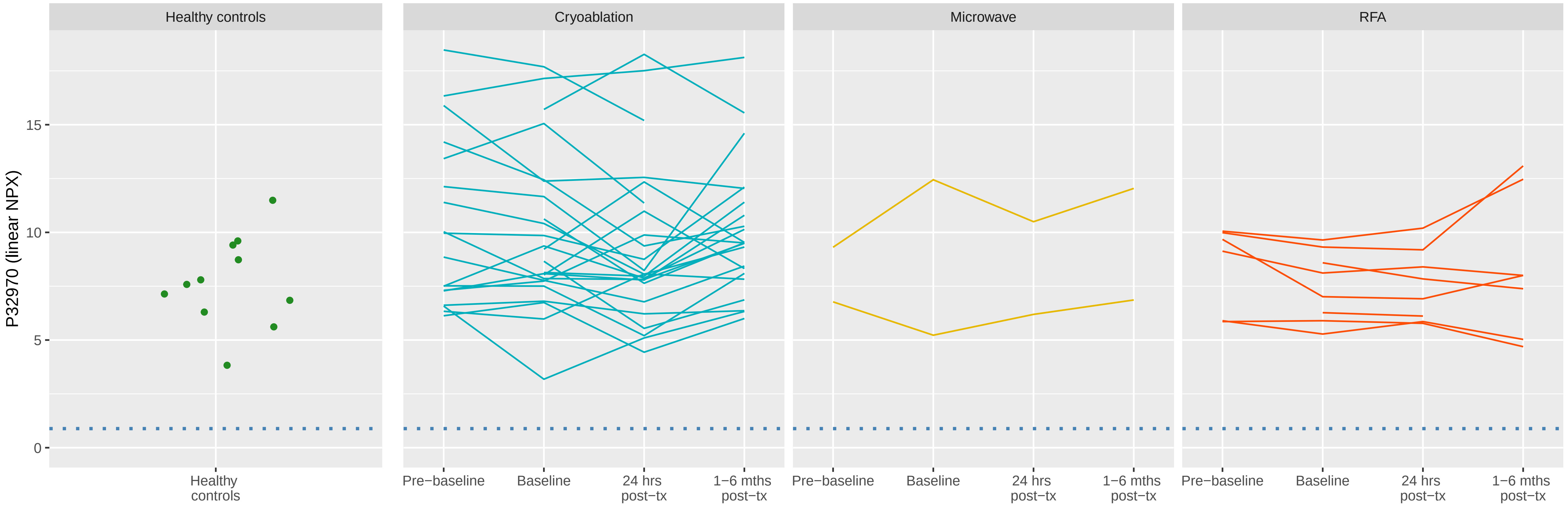

Uniprot ID: P22301, analyte:IL10

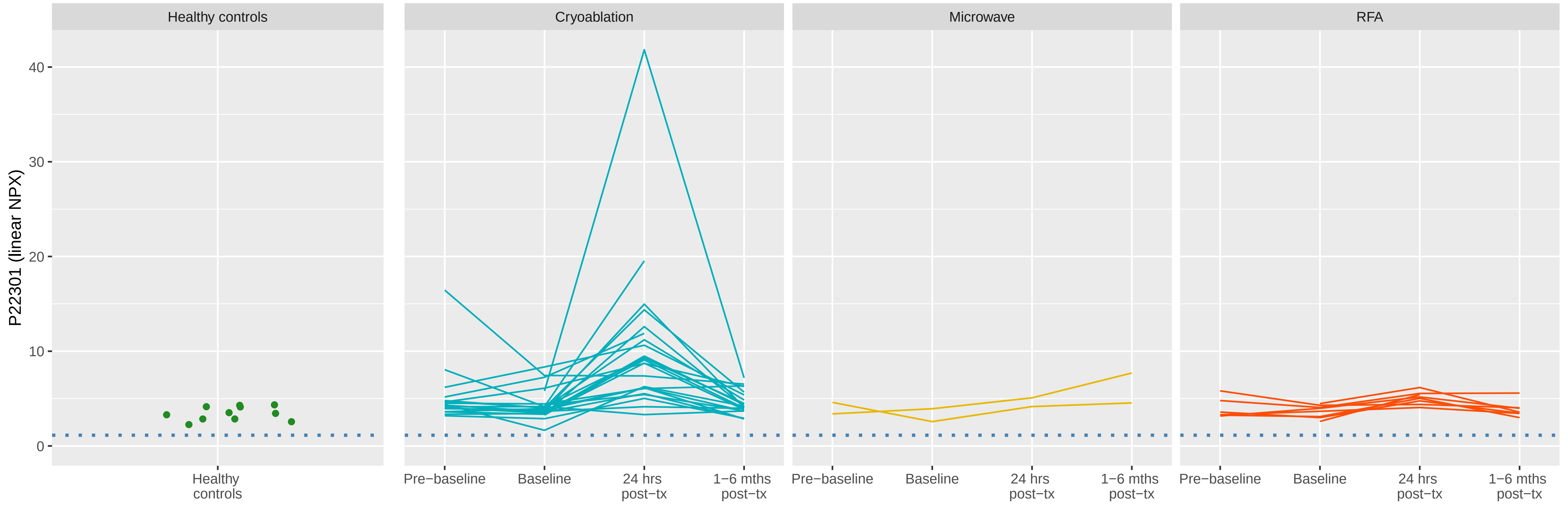

Uniprot ID: Q9NP84, analyte:TNFRSF12A

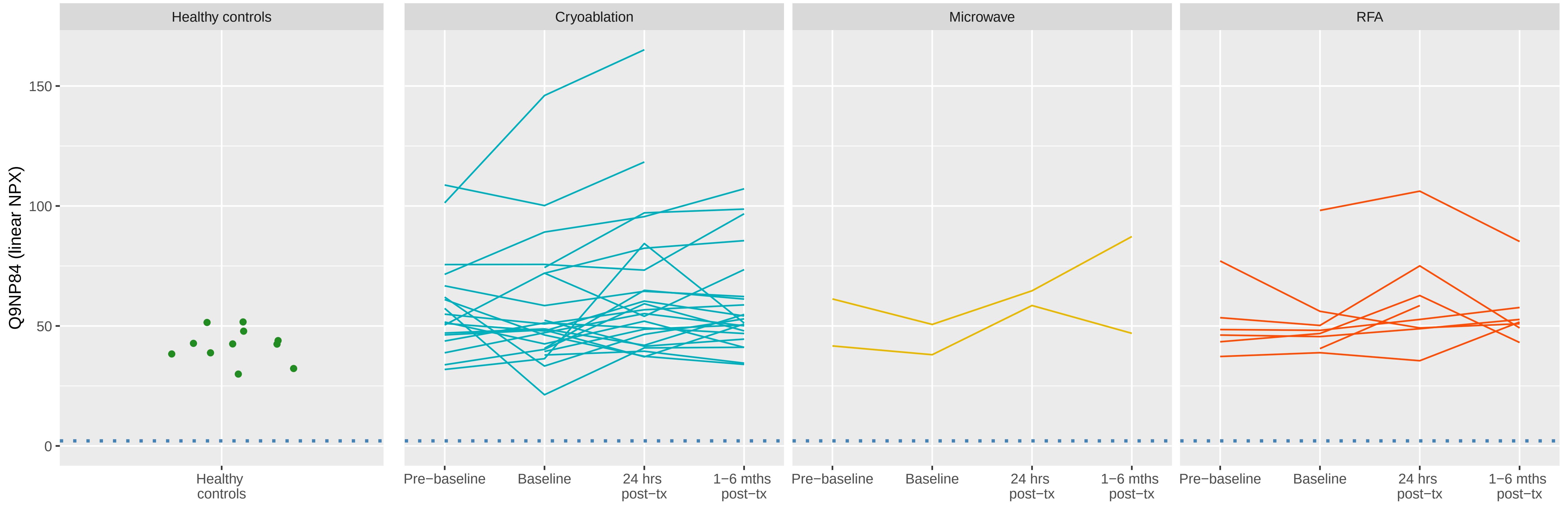

Uniprot ID: P55773, analyte:CCL23

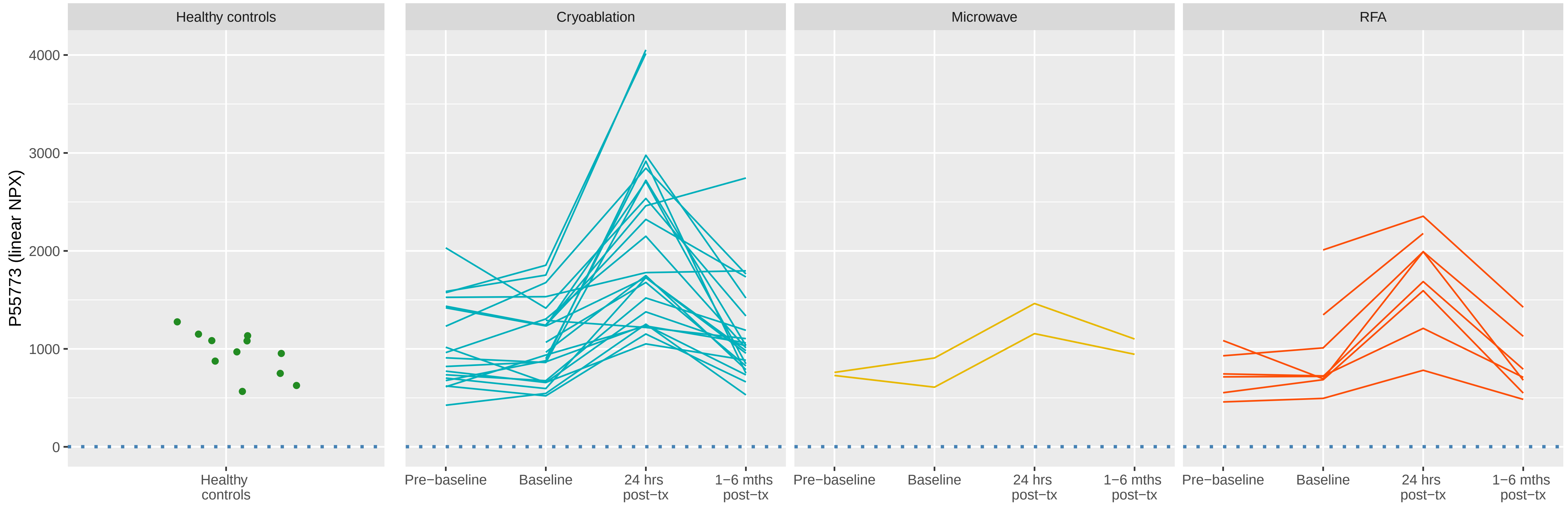

Uniprot ID: P06127, analyte:CD5

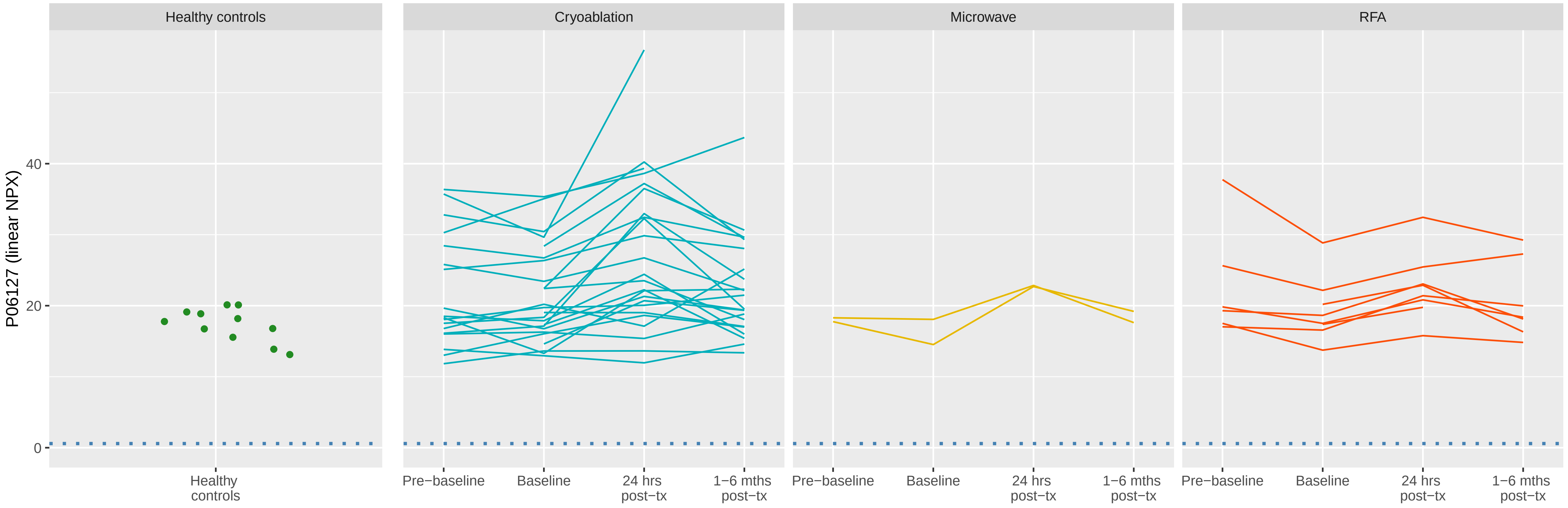

Uniprot ID: P10147, analyte:CCL3

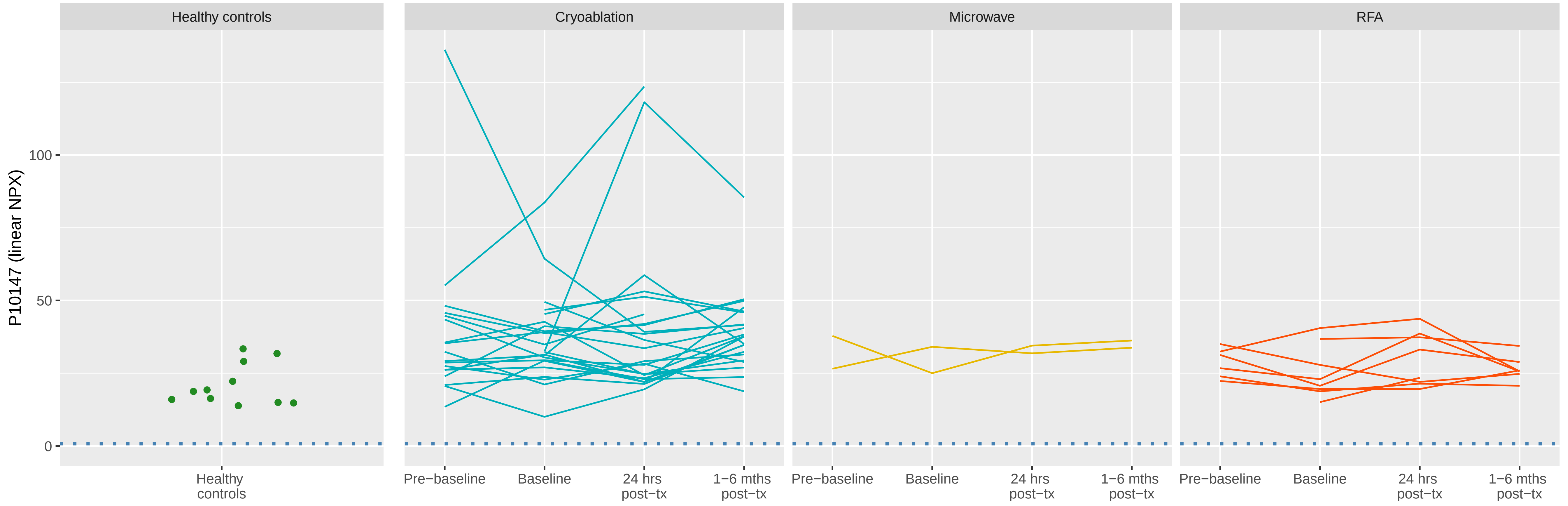

Uniprot ID: P09237, analyte:MMP7

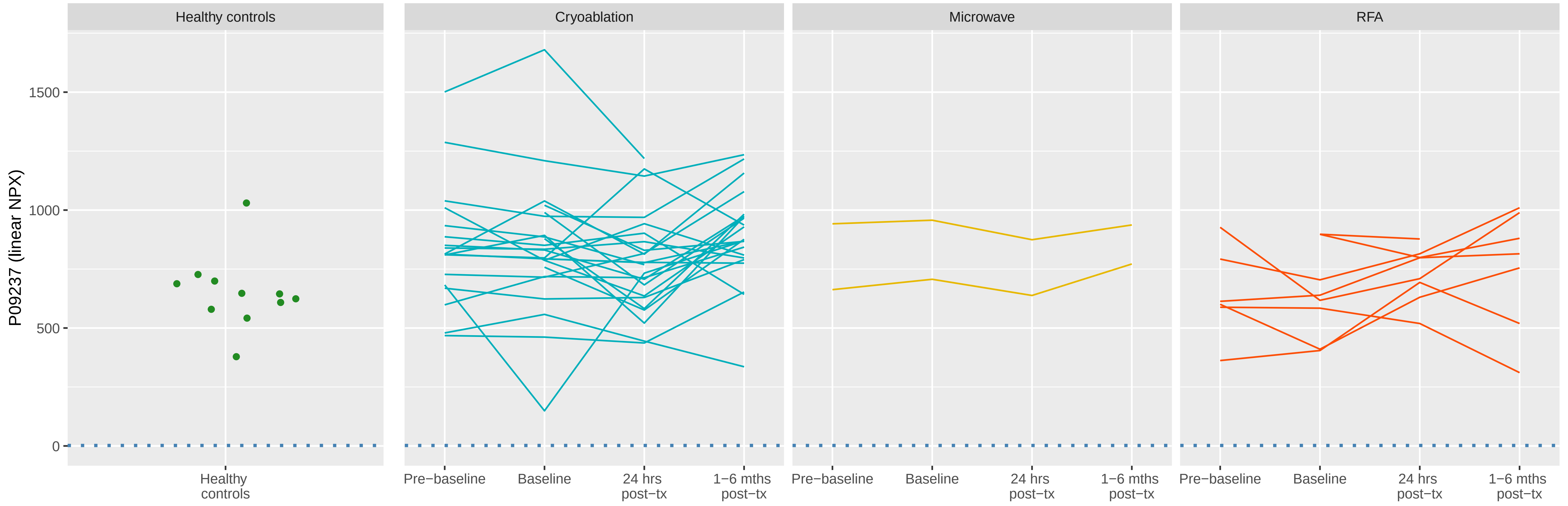

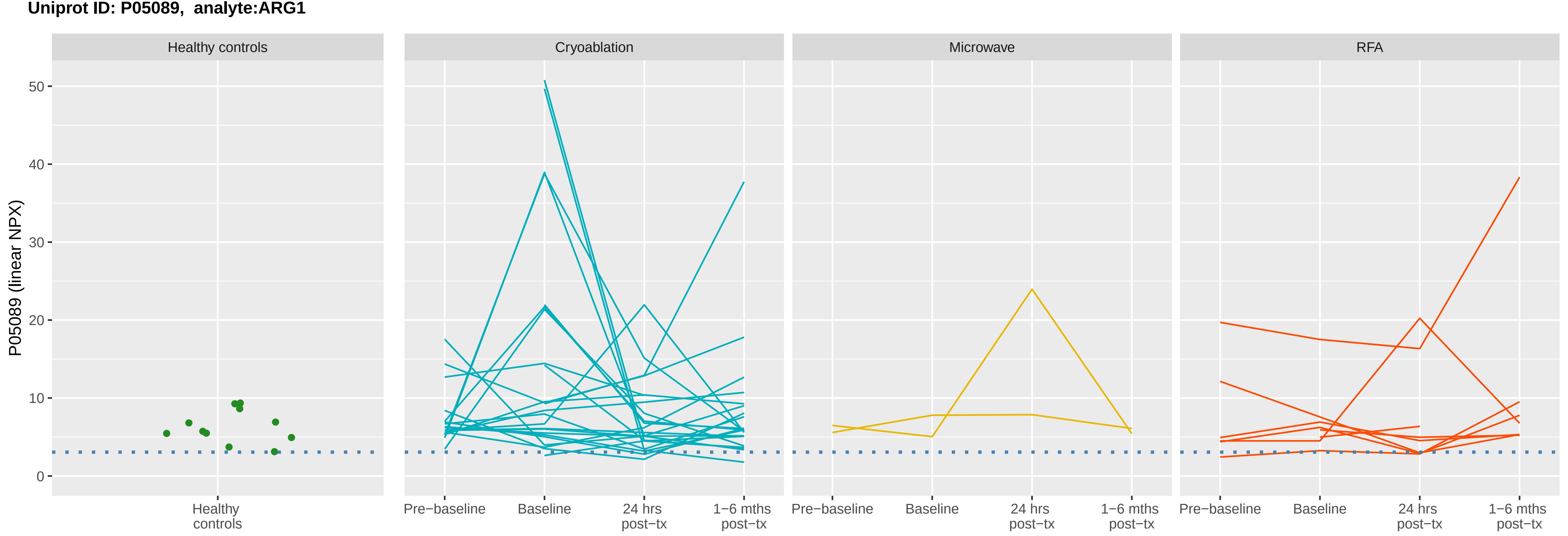
Uniprot ID: P05089, analyte:ARG1
P05089 (linear NPX)
Healthy controls
Cryoablation
Microwave
RFA
0
10
20
30
40
50
Healthy controls
Pre−baseline
Baseline
24 hrs post−tx
1−6 mths post−tx

Uniprot ID: O76036, analyte:NCR1

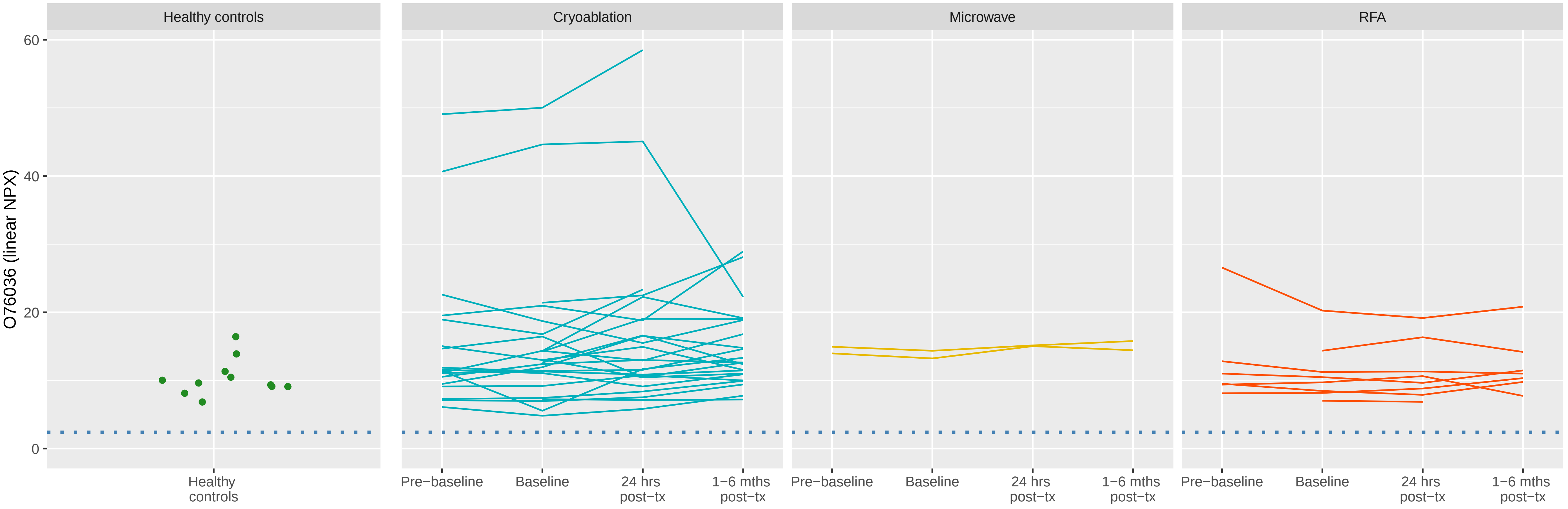

Uniprot ID: P07585, analyte:DCN

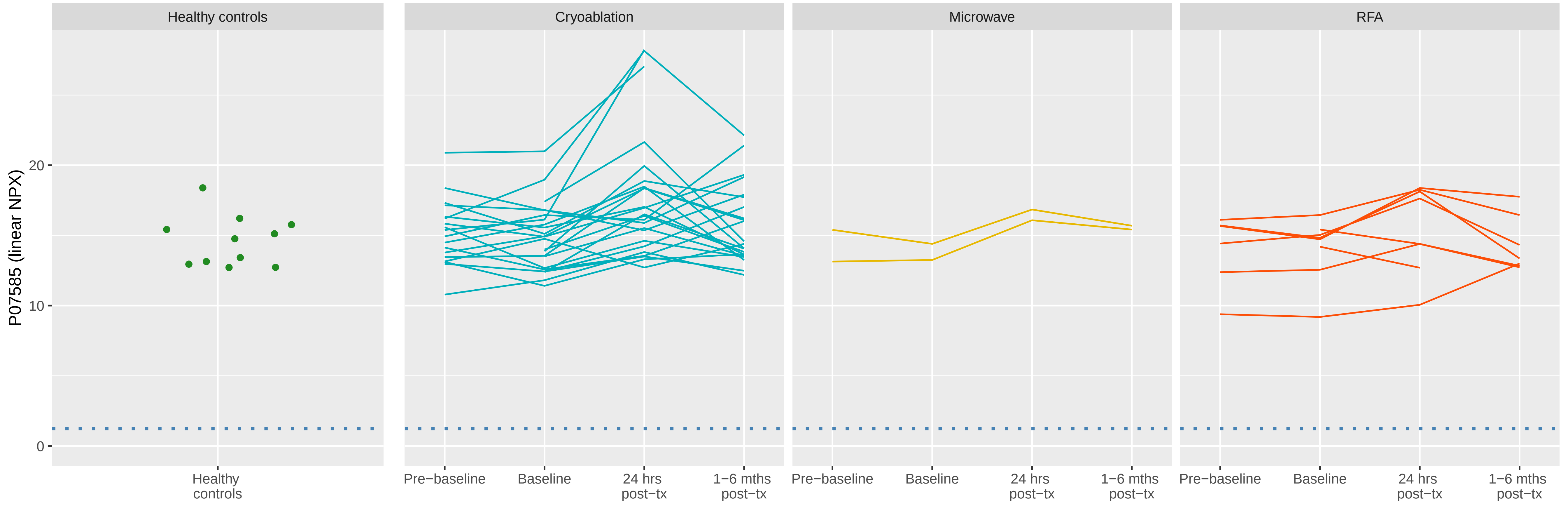

Uniprot ID: O75509, analyte:TNFRSF21

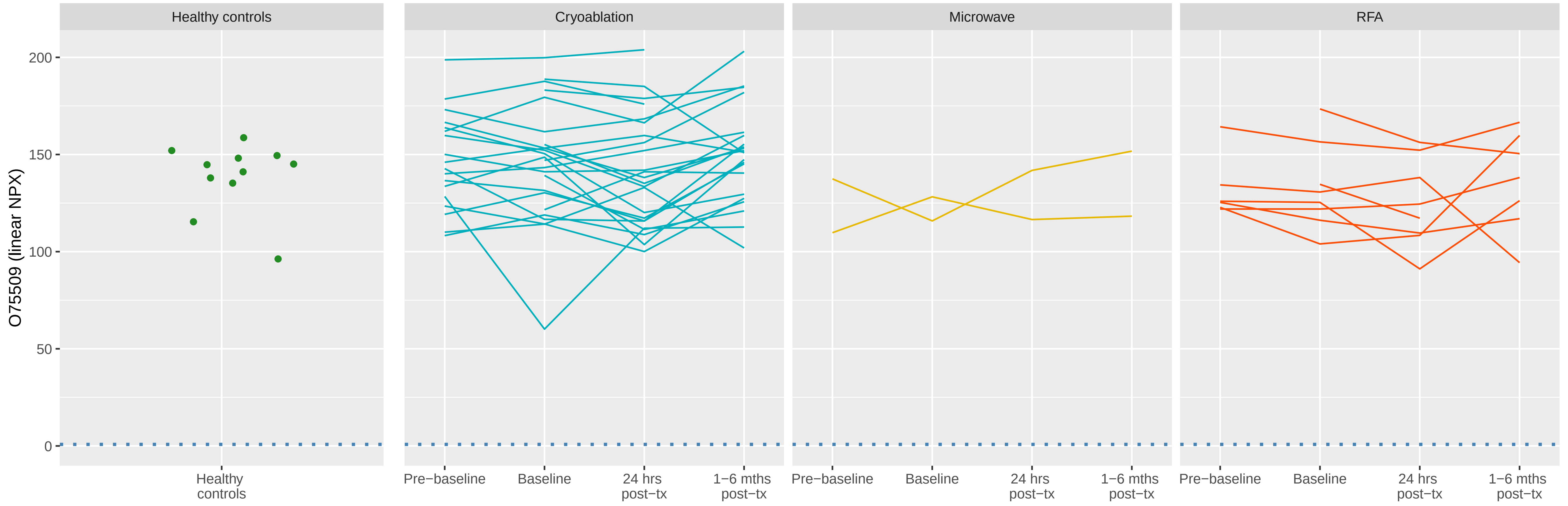

Uniprot ID: P43489, analyte:TNFRSF4

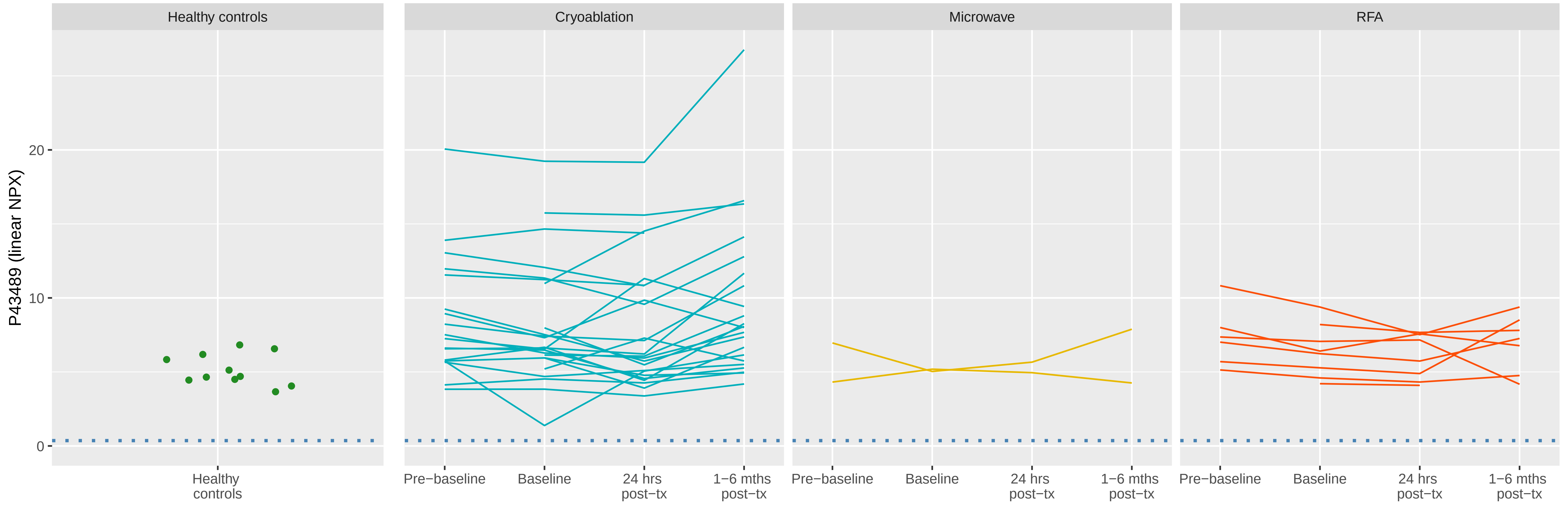

Uniprot ID: Q29983.Q29980, analyte:MIC−A/B

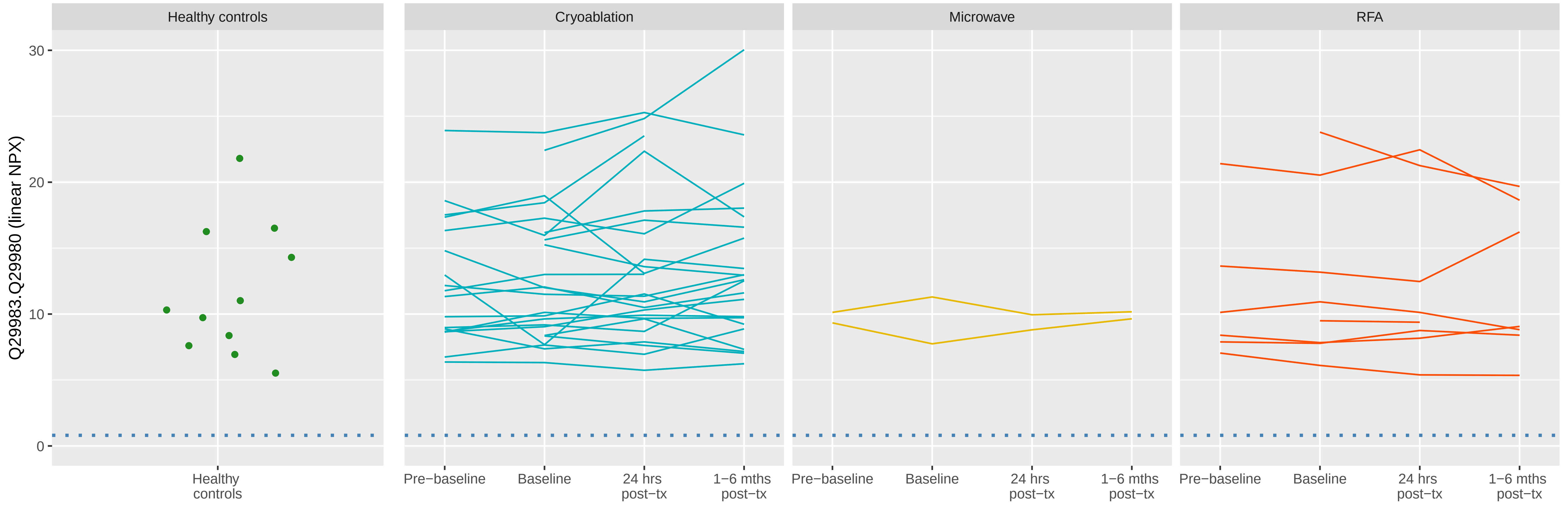

Uniprot ID: Q92583, analyte:CCL17

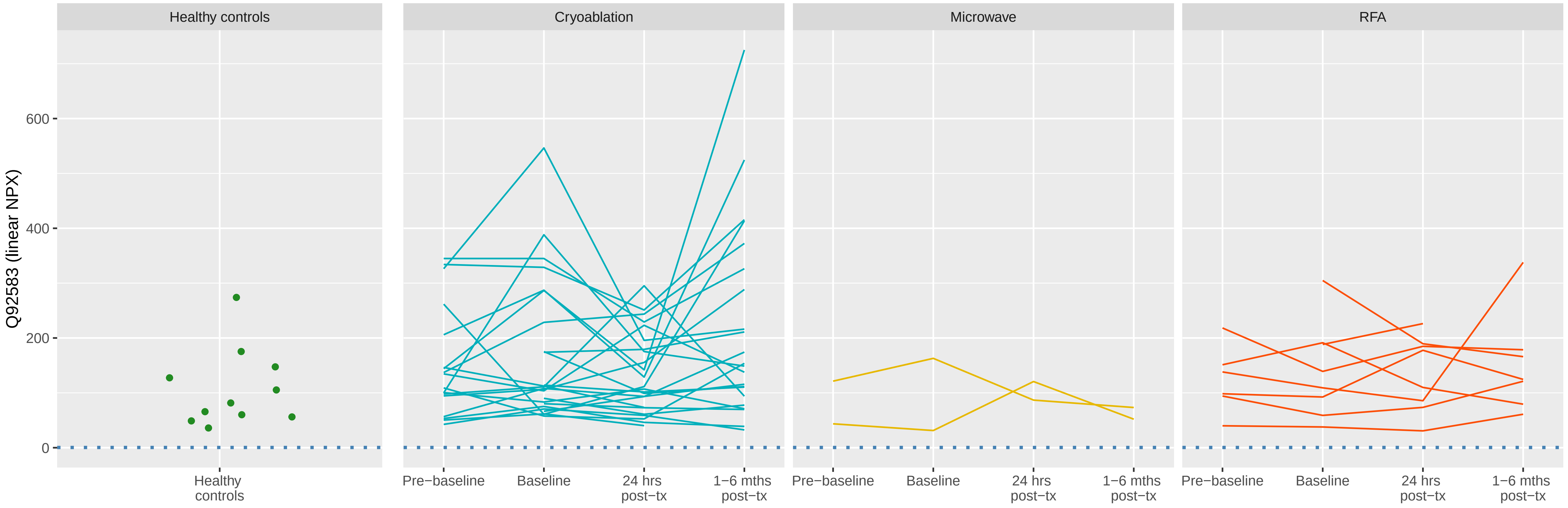

Uniprot ID: O15123, analyte:ANGPT2

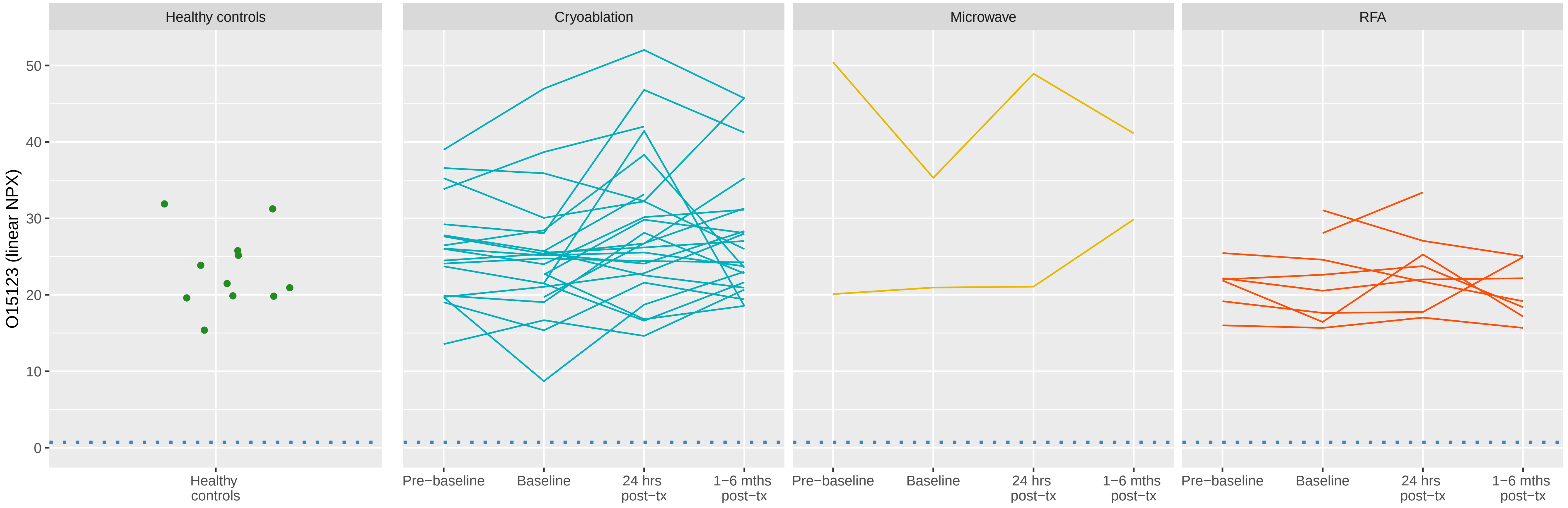

Uniprot ID: P21246, analyte:PTN

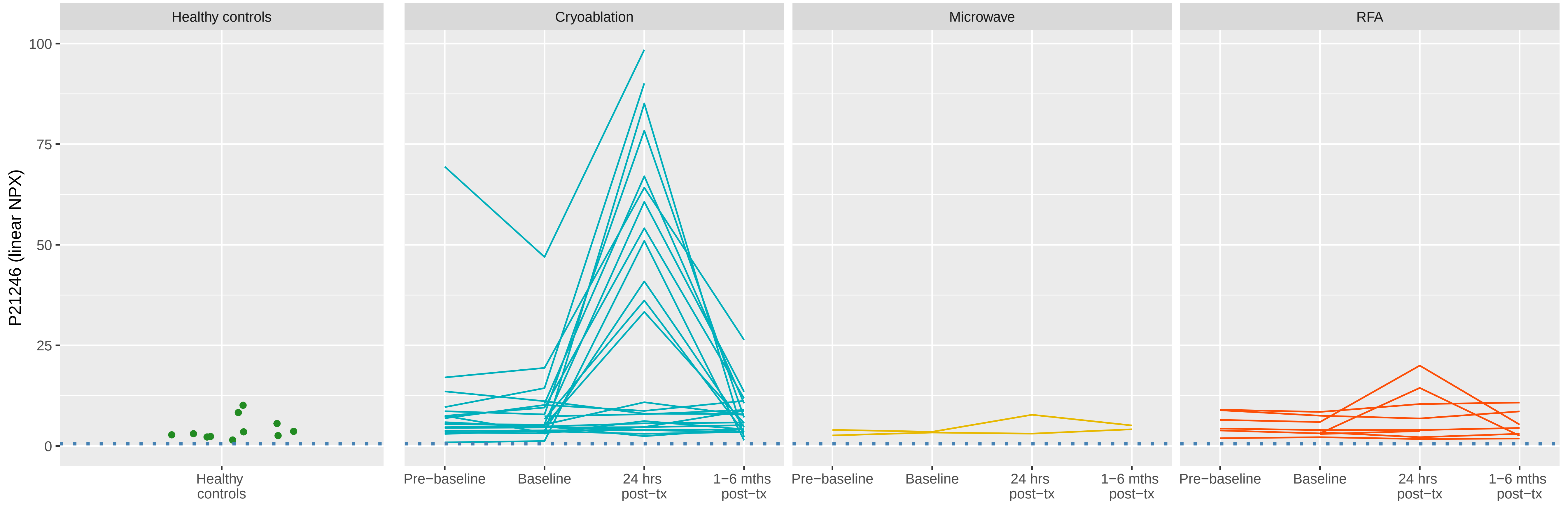

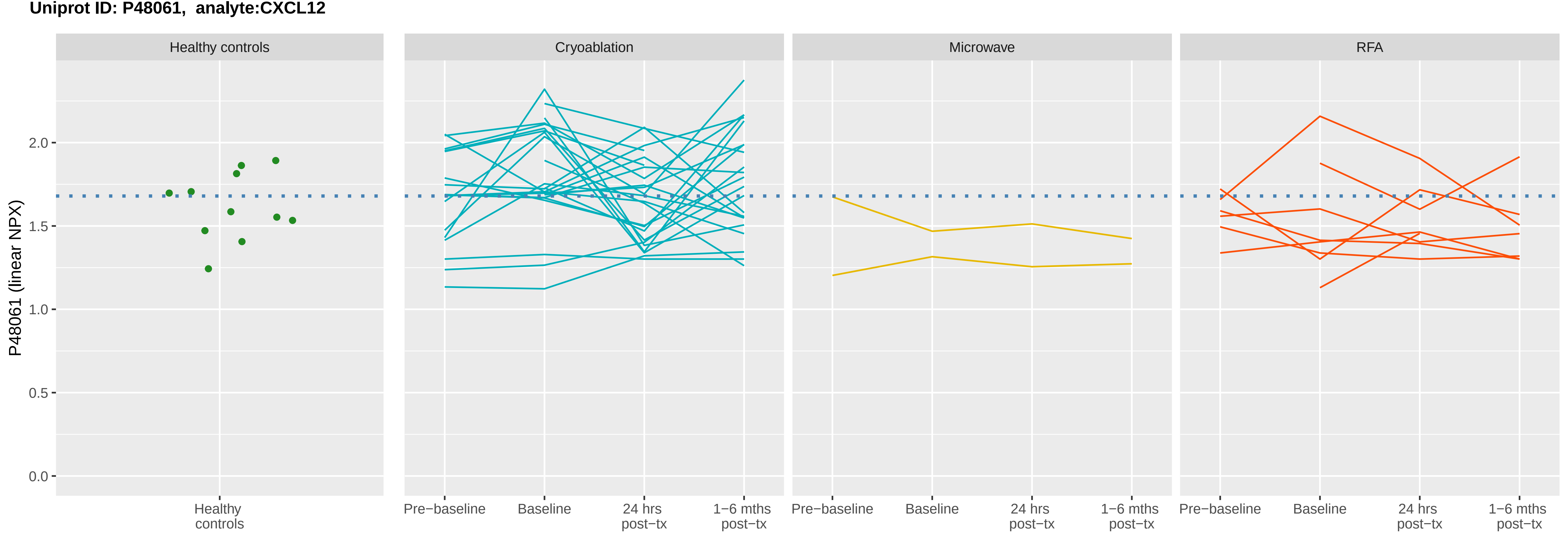

Uniprot ID: P48061, analyte:CXCL12
Healthy controls
Cryoablation
Microwave
RFA
P48061 (linear NPX)
2.0
1.5
1.0
0.5
0.0
Healthy controls
Pre−baseline
Baseline
24 hrs post−tx
1−6 mths post−tx

Uniprot ID: P01579, analyte:IFN−gamma

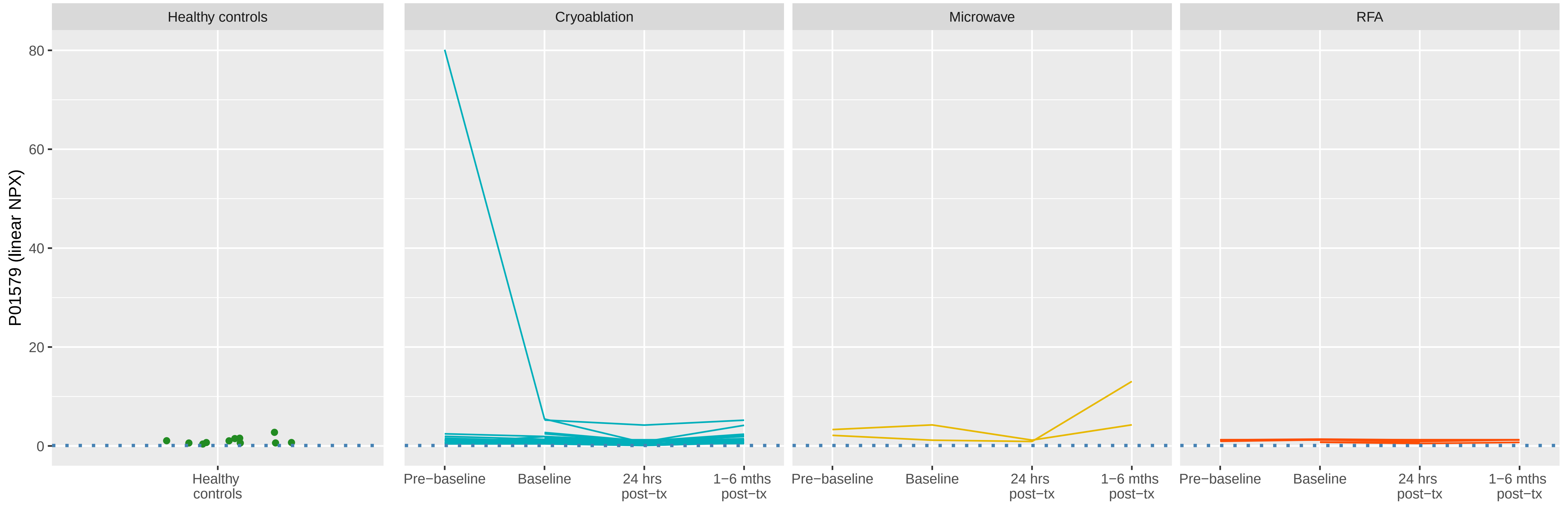

Uniprot ID: Q9UQV4, analyte:LAMP3

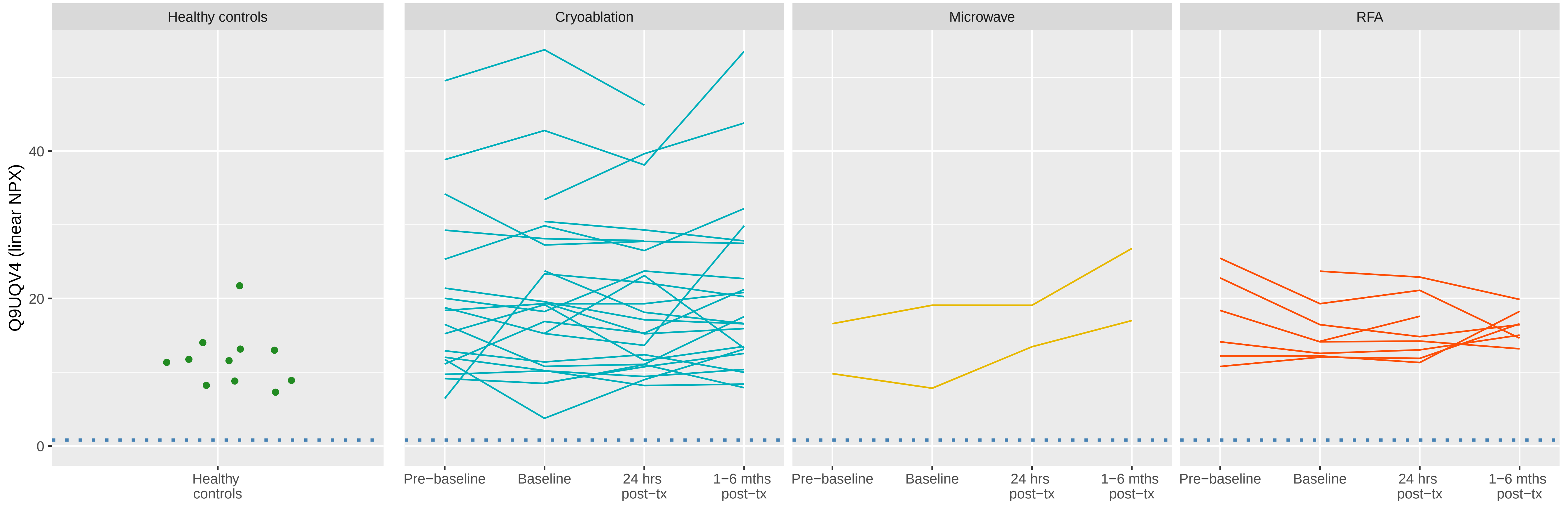

Uniprot ID: Q14790, analyte:CASP−8

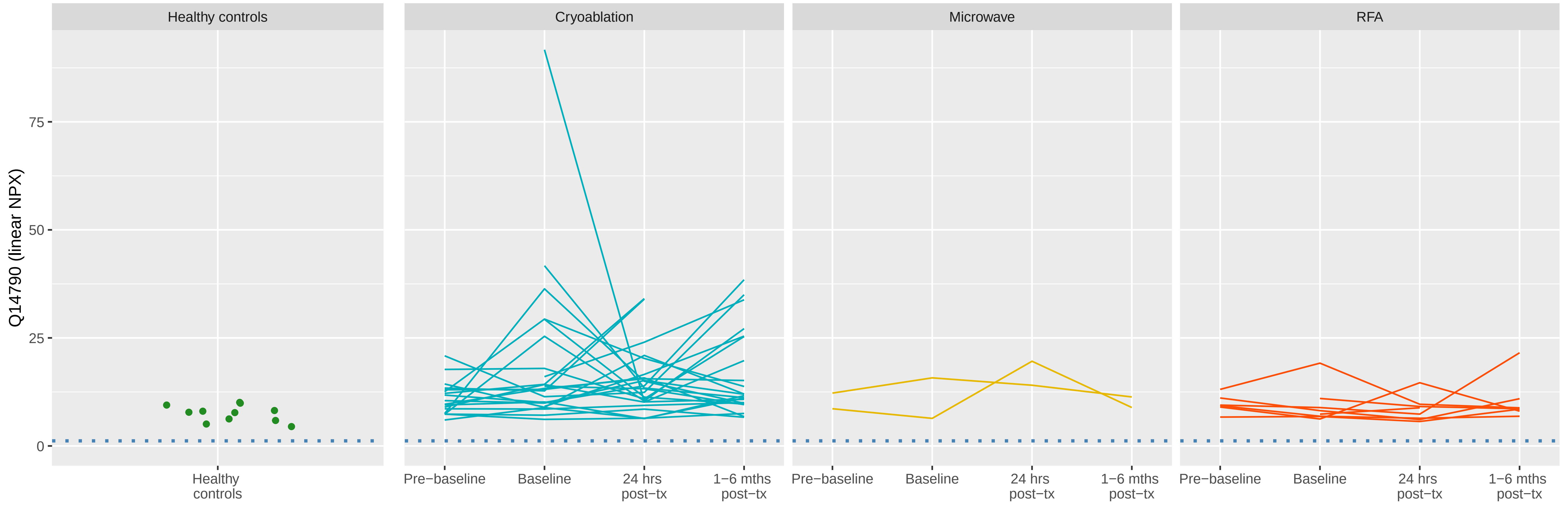

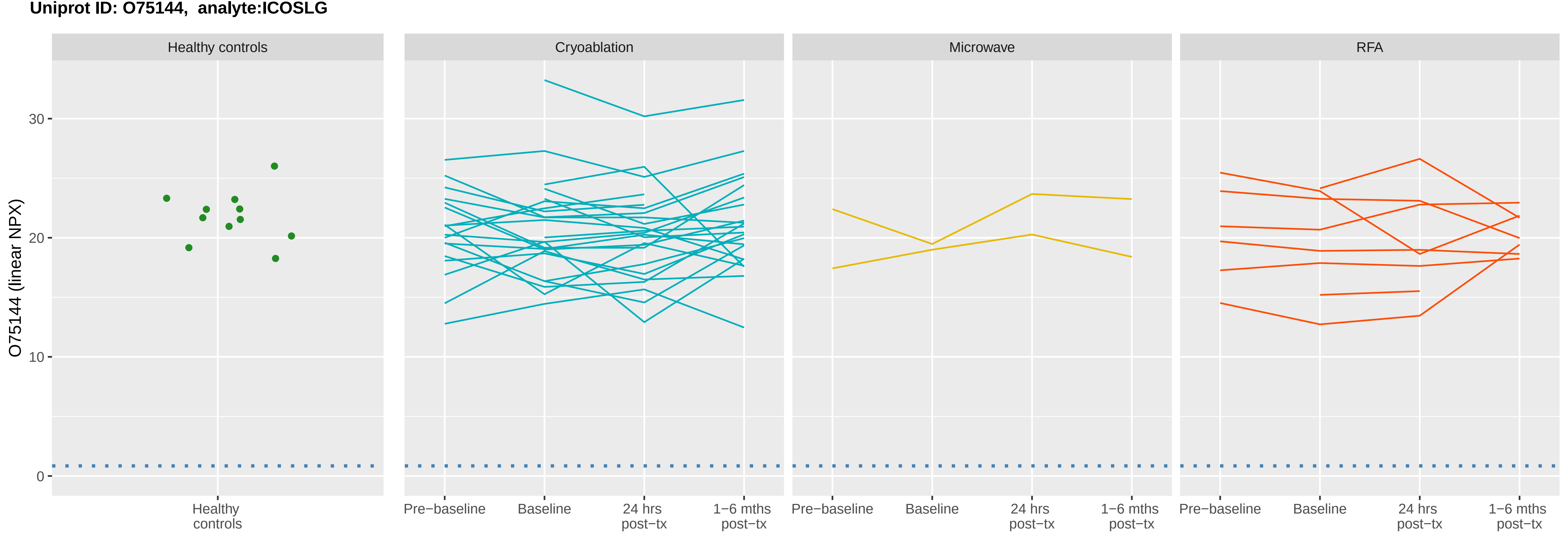

Uniprot ID: O75144, analyte:ICOSLG
Healthy controls
Cryoablation
Microwave
RFA
O75144 (linear NPX)
0
10
20
30
Healthy controls
Pre−baseline
Baseline
24 hrs post−tx
1−6 mths post−tx

Uniprot ID: P39900, analyte:MMP12

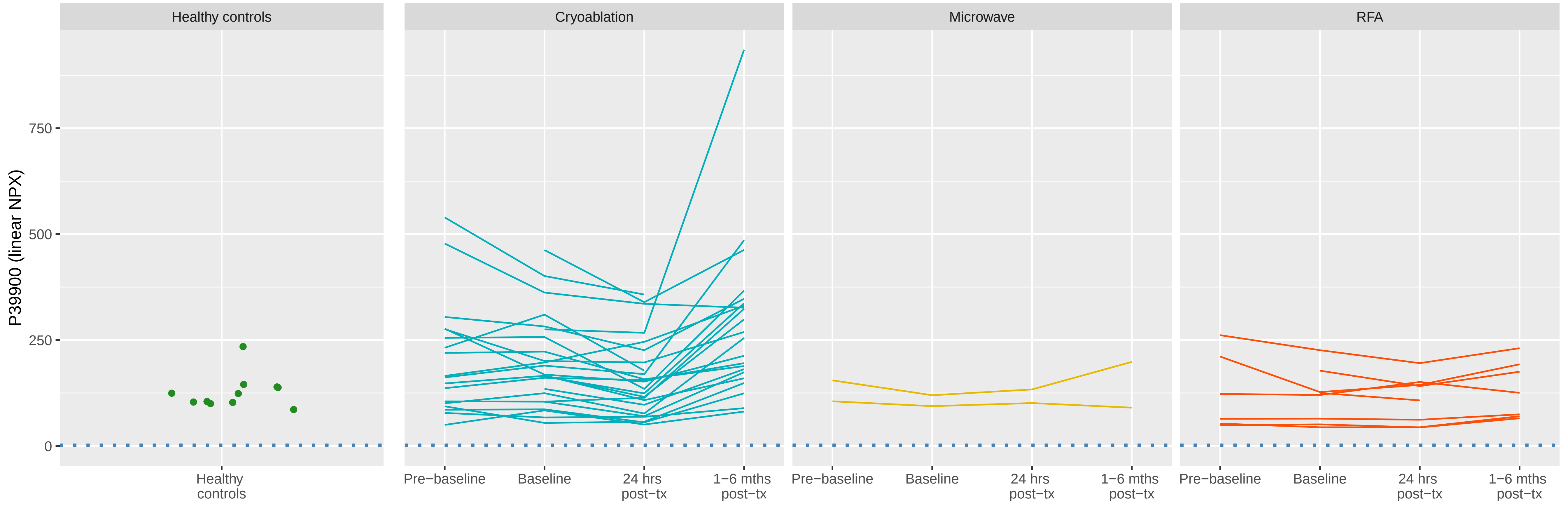

Uniprot ID: O43927, analyte:CXCL13

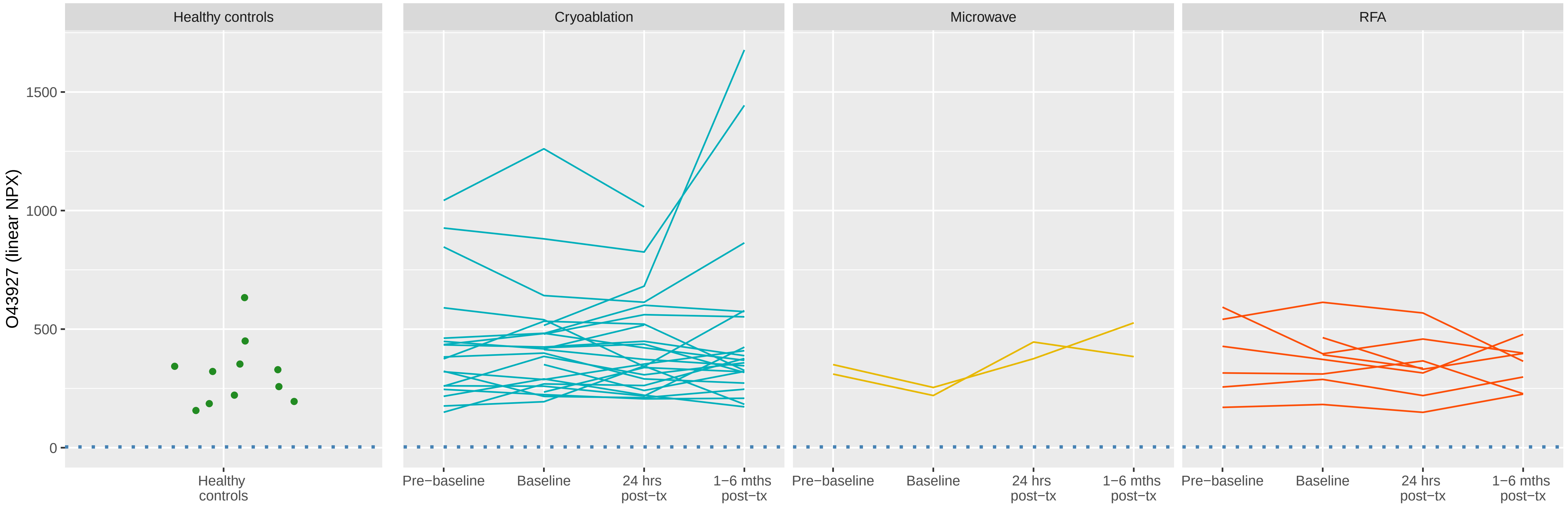

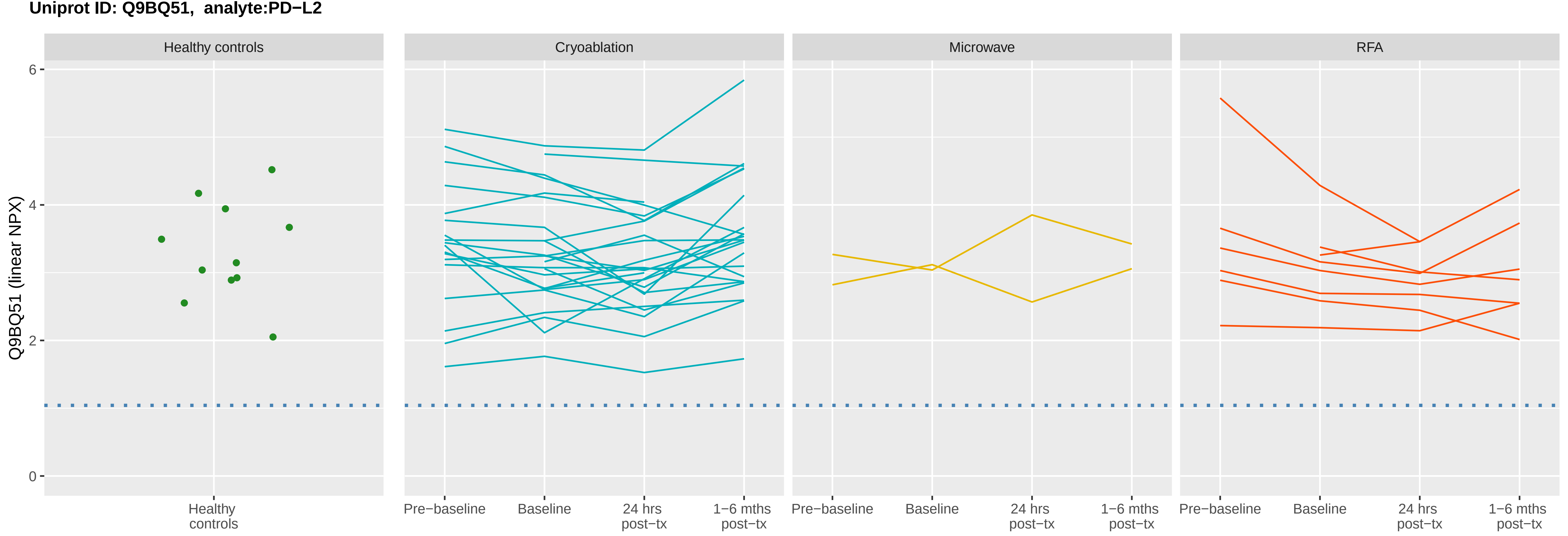
Uniprot ID: Q9BQ51, analyte:PD−L2
Healthy controls
Cryoablation
Microwave
RFA
Q9BQ51 (linear NPX)
0
2
4
6
Healthy controls
Pre−baseline
Baseline
24 hrs post−tx
1−6 mths post−tx

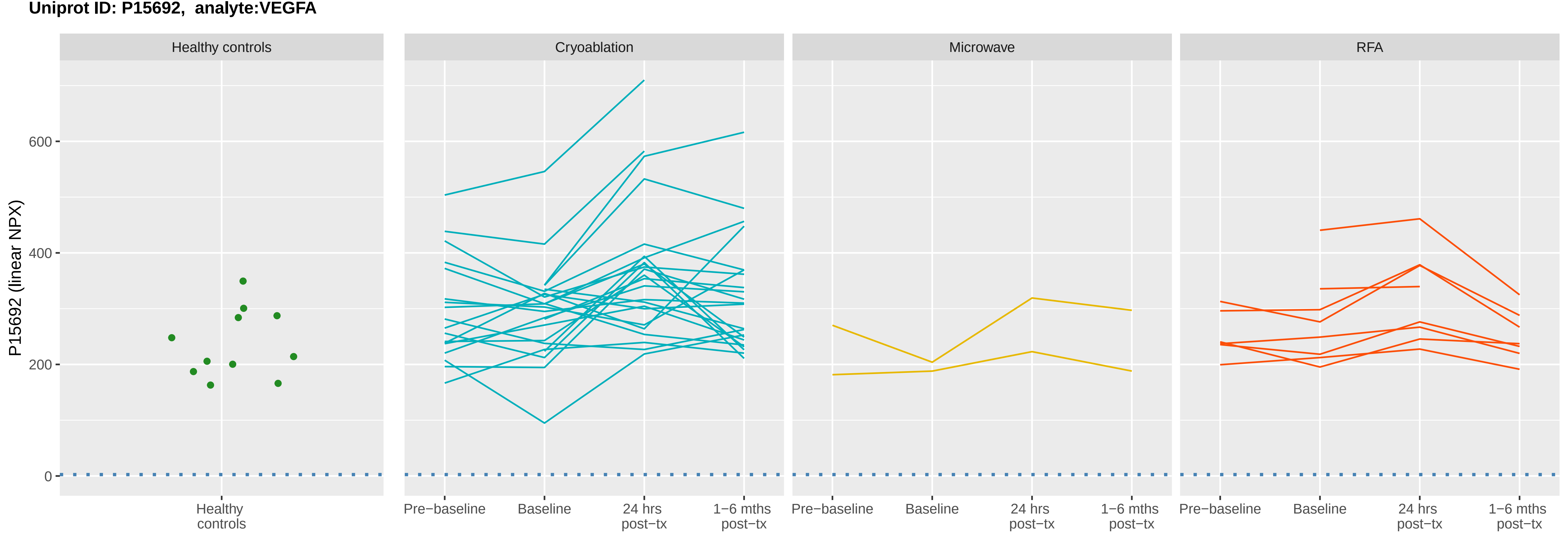

Uniprot ID: P15692, analyte:VEGFA
Healthy controls
Cryoablation
Microwave
RFA
P15692 (linear NPX)
600
400
200
0
Healthy controls
Pre−baseline
Baseline
24 hrs post−tx
1−6 mths post−tx

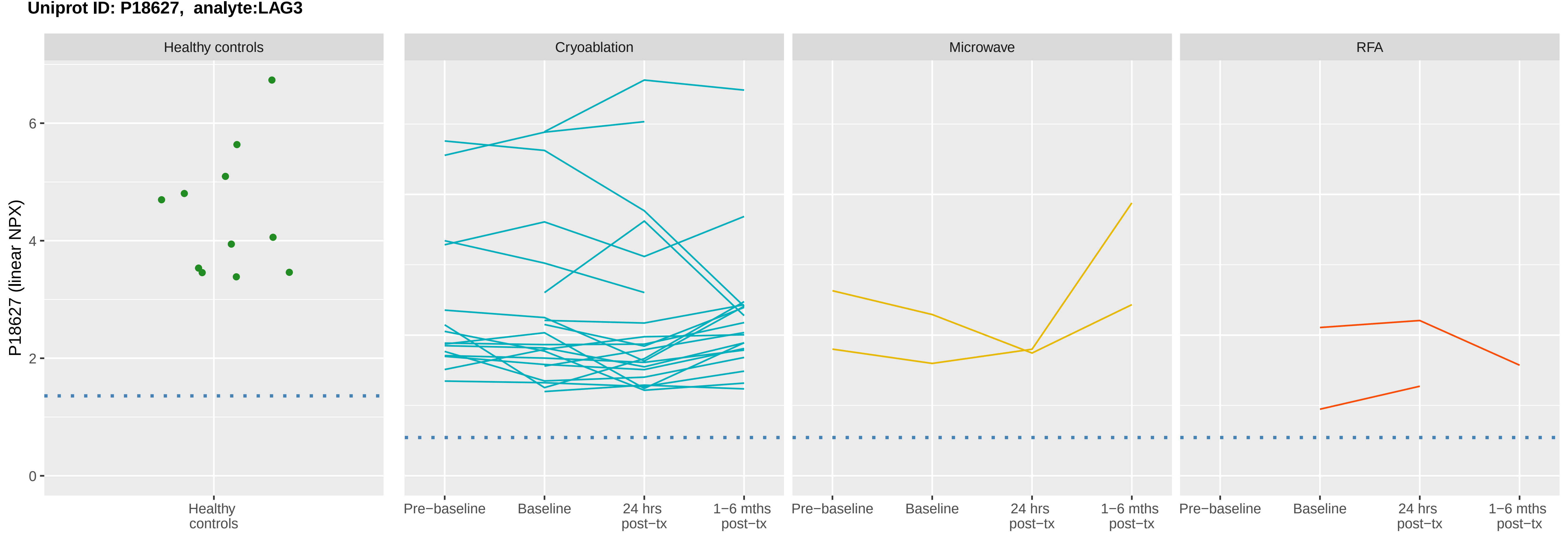

Uniprot ID: P18627, analyte:LAG3
Healthy controls
Cryoablation
Microwave
RFA
P18627 (linear NPX)
0
2
4
6
Healthy controls
Pre−baseline
Baseline
24 hrs post−tx
1−6 mths post−tx

Uniprot ID: P42701, analyte:IL12RB1

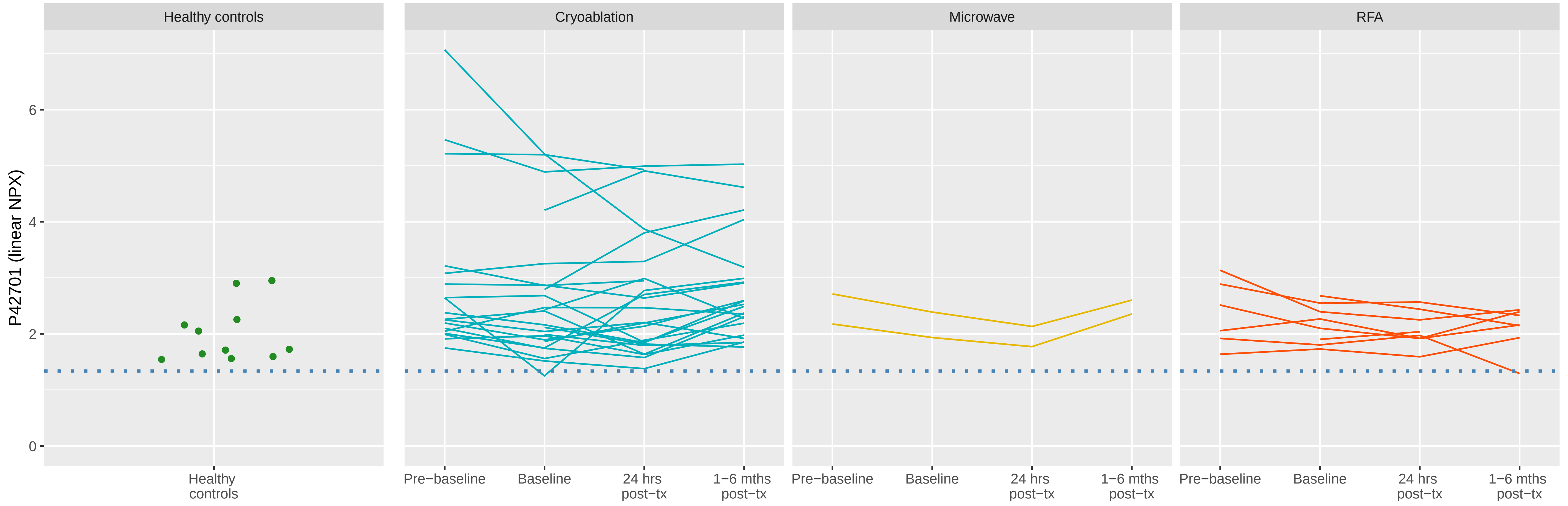

Uniprot ID: P78556, analyte:CCL20

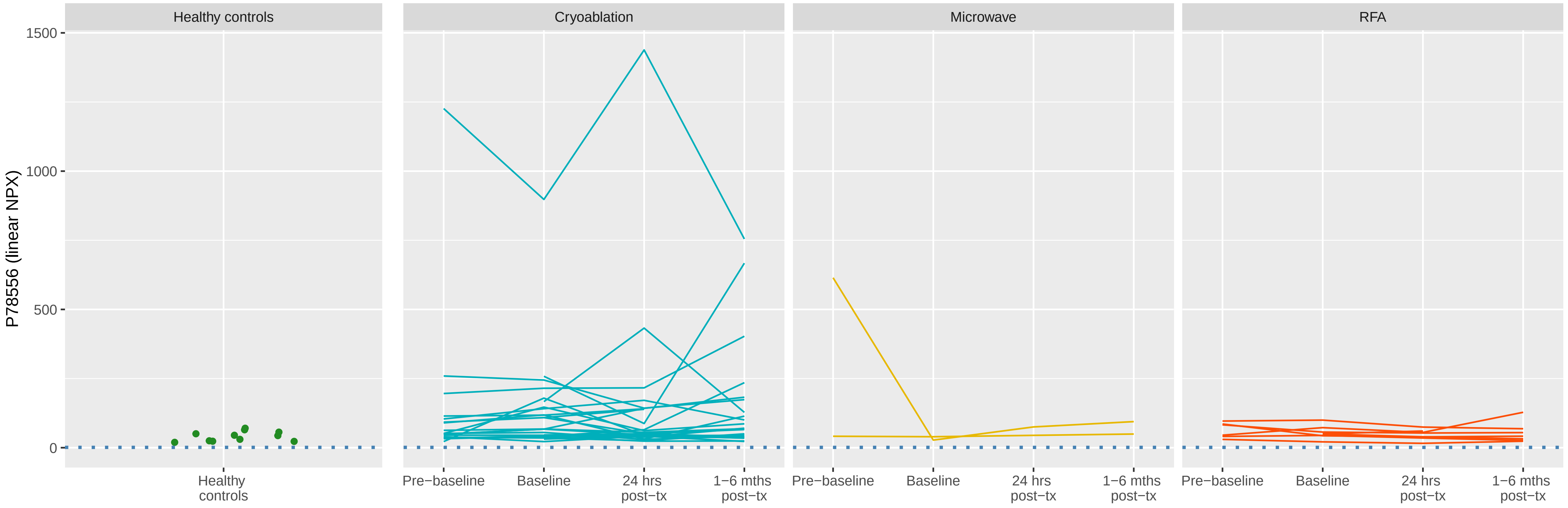

Uniprot ID: P01375, analyte:TNF

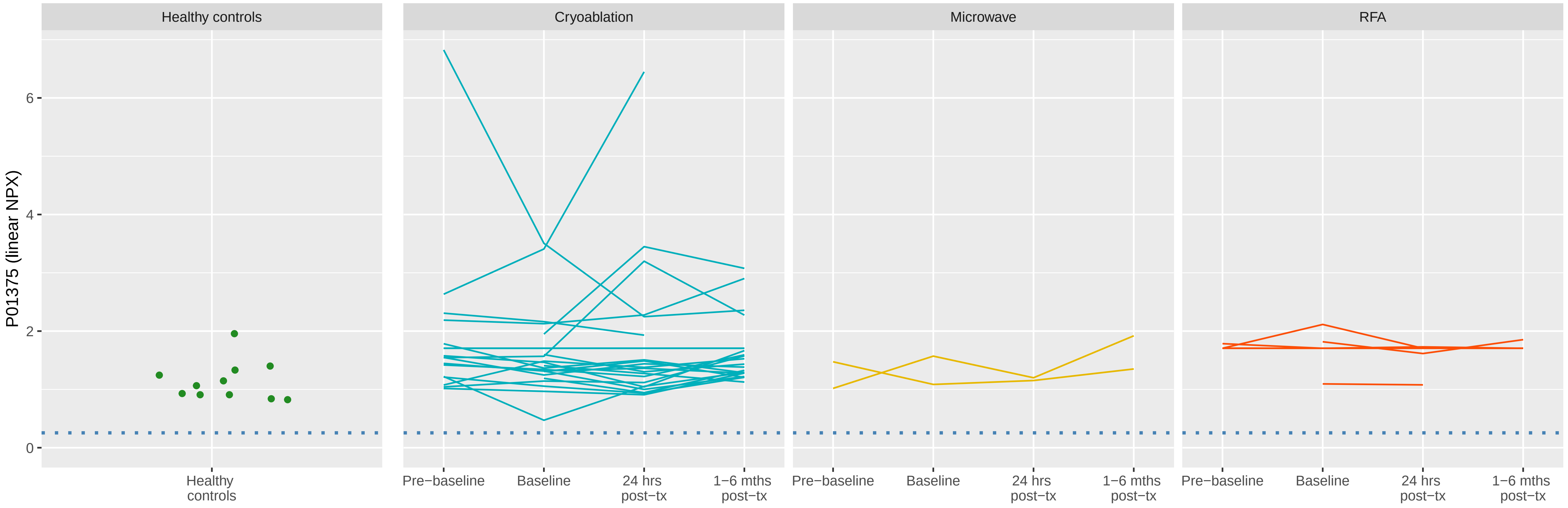

Uniprot ID: Q13241, analyte:KLRD1

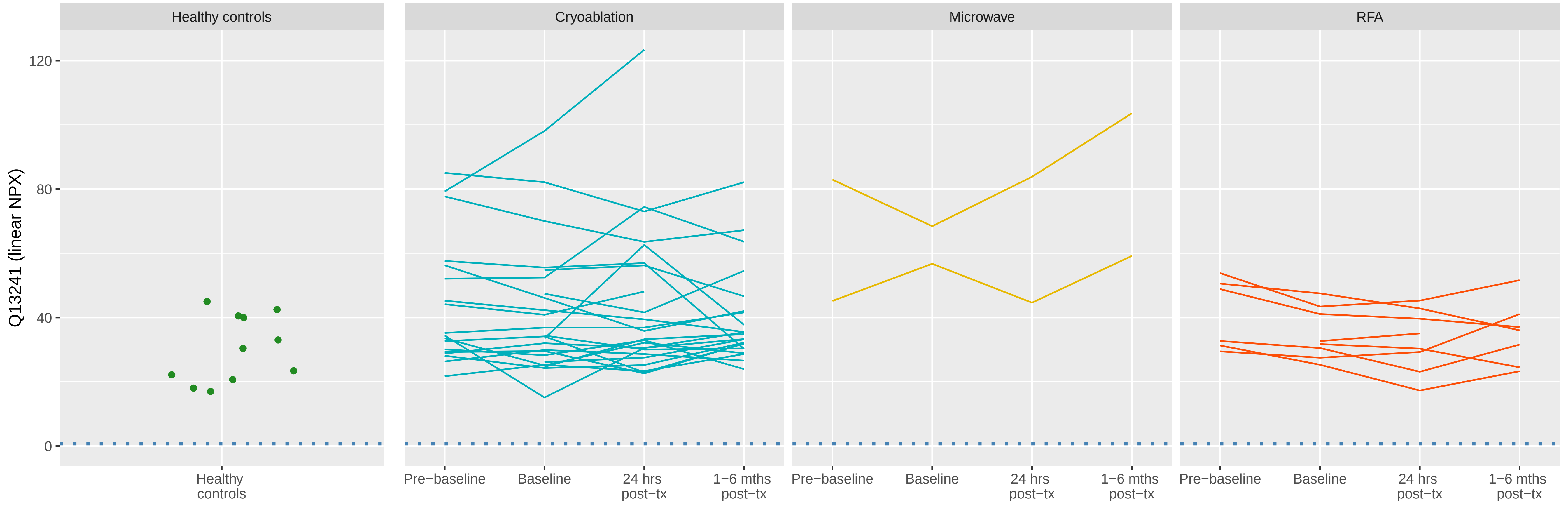

Uniprot ID: P10144, analyte:GZMB

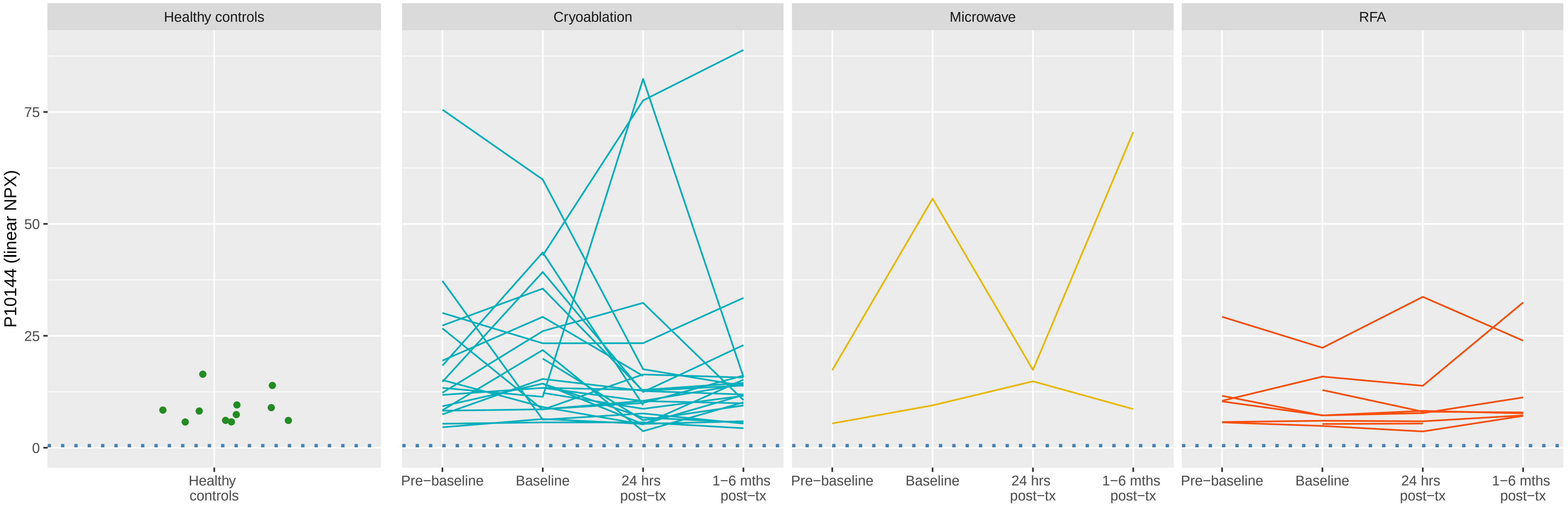

Uniprot ID: Q01151, analyte:CD83

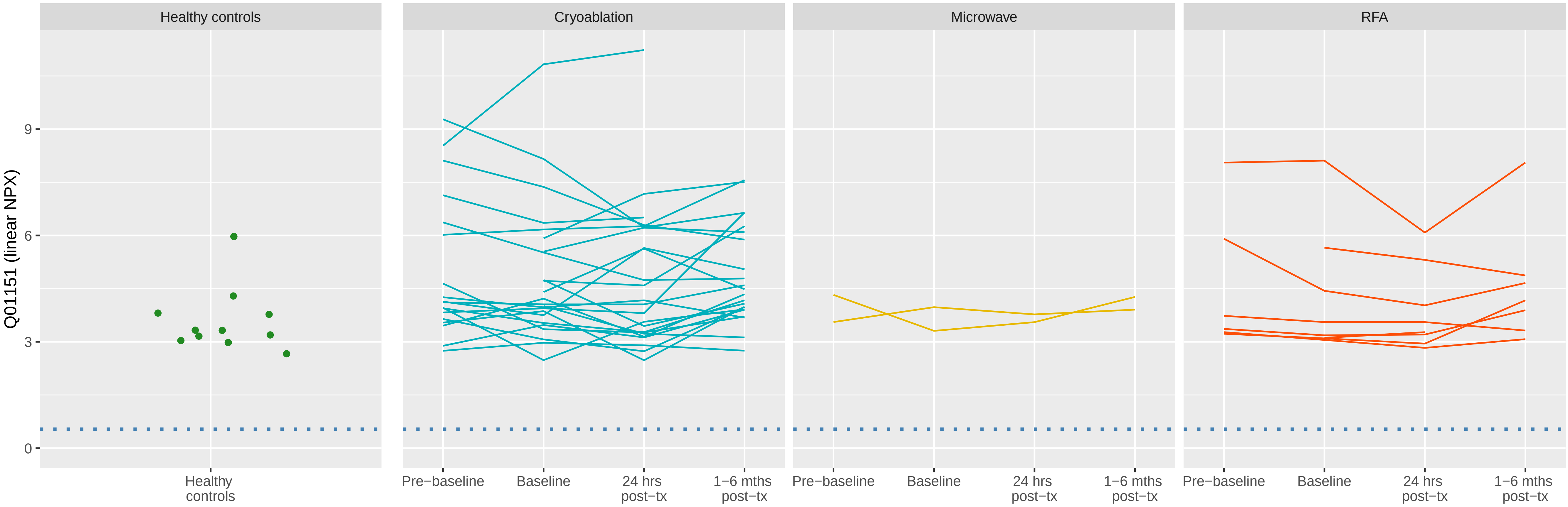

Uniprot ID: P29459.P29460, analyte:IL12

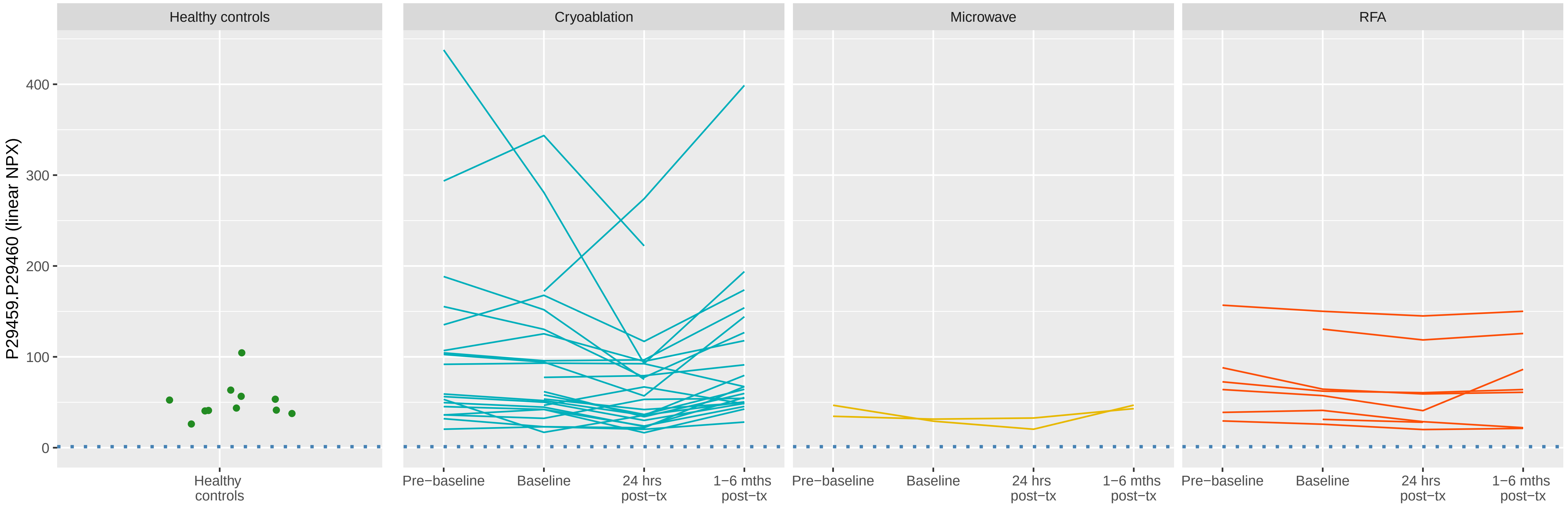

Uniprot ID: P09603, analyte:CSF−1

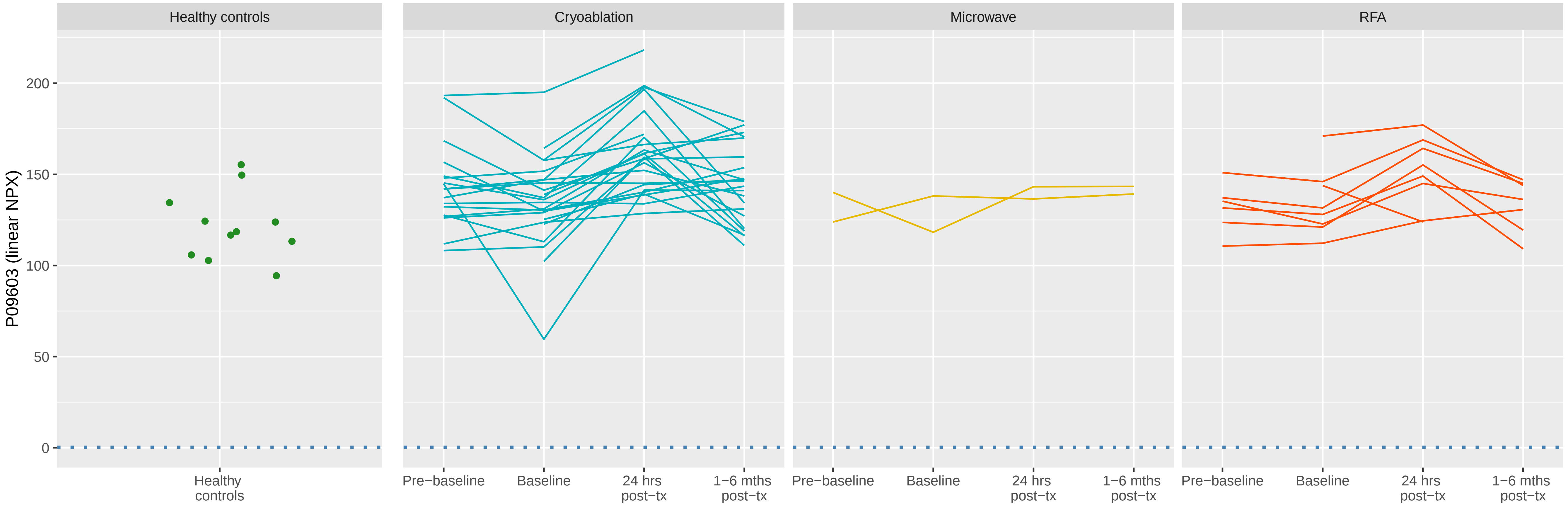

Supplement: Supplementary file 1 [file cancers-13-06037-s001.zip › Supplementary File/Figure S1.pdf]
