# Supplementary material for: An Exploratory Analysis of Changes in Circulating Plasma Protein Profiles Following Image-Guided Ablation of Renal Tumours Provides Evidence for Effects on Multiple Biological Processes
Source: Cancers (Basel). 2021 Nov 30;13(23):6037. doi: 10.3390/cancers13236037 (PMC8656737; doi:10.3390/cancers13236037)
Supplement: Supplementary file 1 [file cancers-13-06037-s001.zip › Supplementary File/Figure S10.pdf]

Figure S10. Longitudinal profiles of all plasma proteins on the Immunooncology panel, prior to and post-ablation. Results for each protein are expressed as a % of the reference baseline (TP2; 100%) and are shown for patients receiving CRYO (n=25) with the three patients who developed acute complications post-CRYO highlighted.

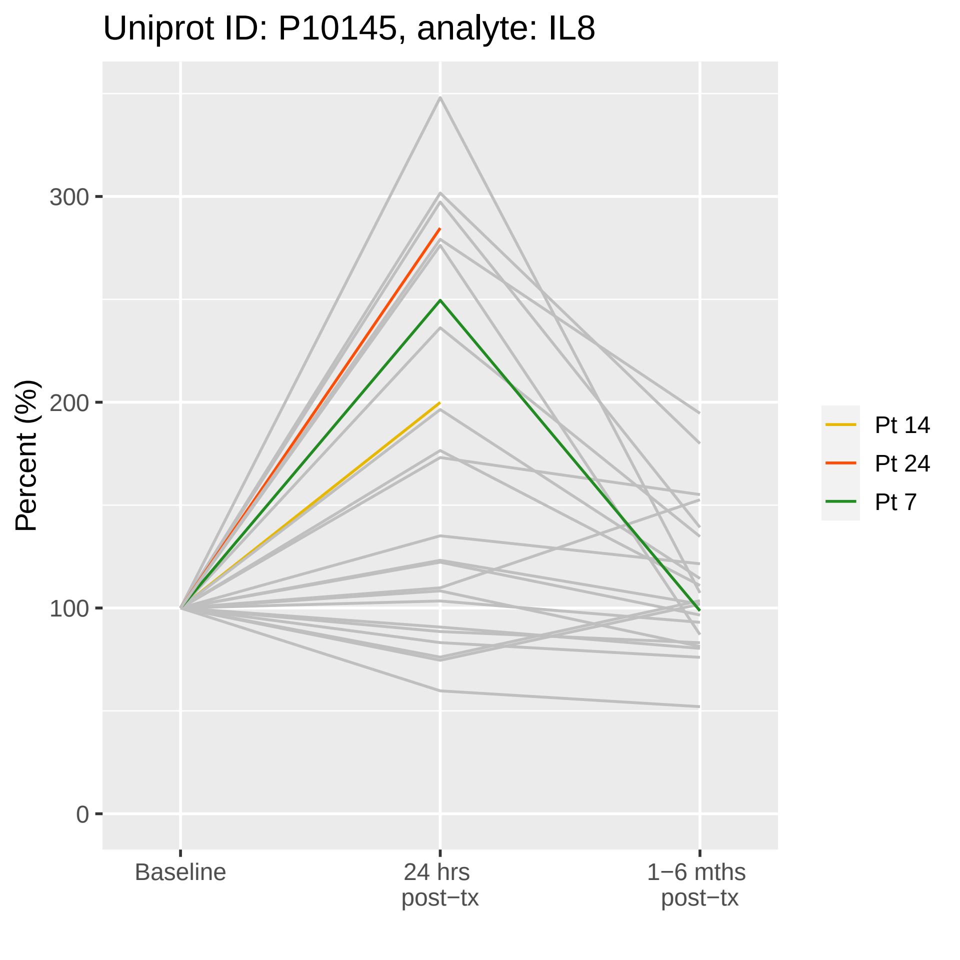

Uniprot ID: P10145, analyte: IL8
Percent (%)
300
200
100
0
Baseline
24 hrs post−tx
1−6 mths post−tx
Pt 14
Pt 24
Pt 7

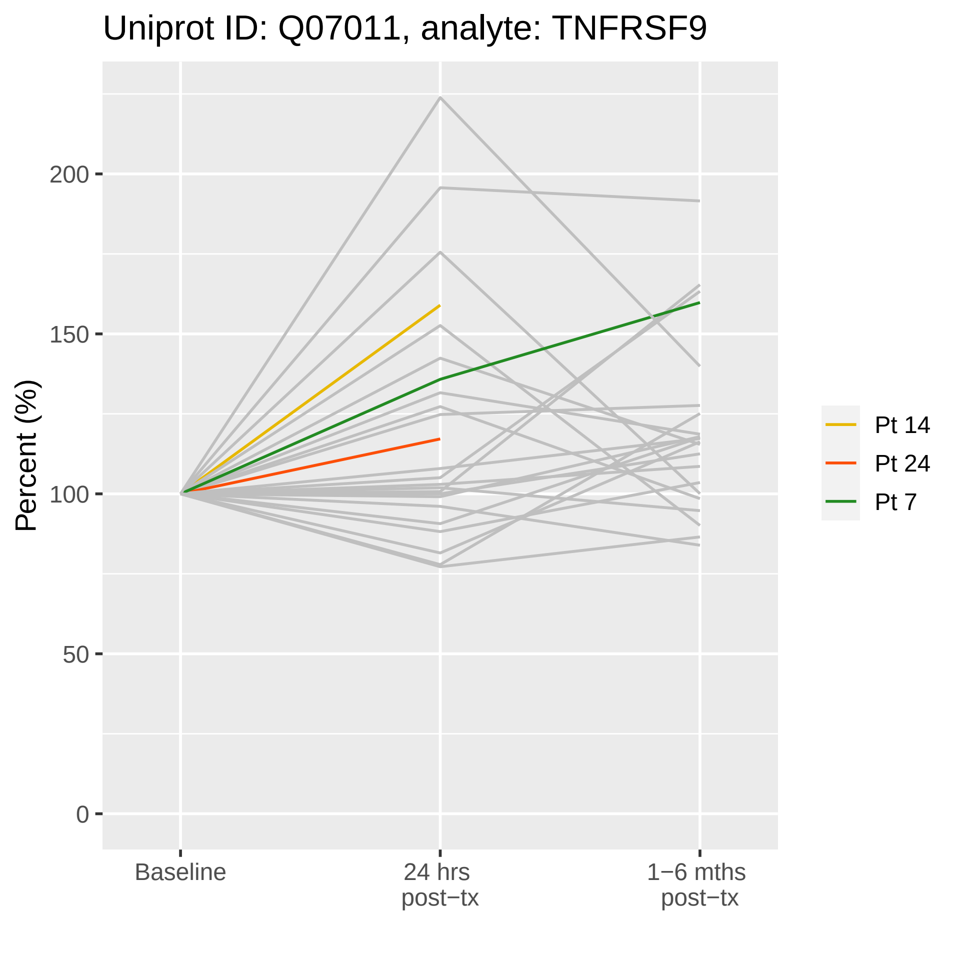

Uniprot ID: Q07011, analyte: TNFRSF9
Percent (%)
200
150
100
50
0
Baseline
24 hrs
post−tx
1−6 mths
post−tx
Pt 14
Pt 24
Pt 7

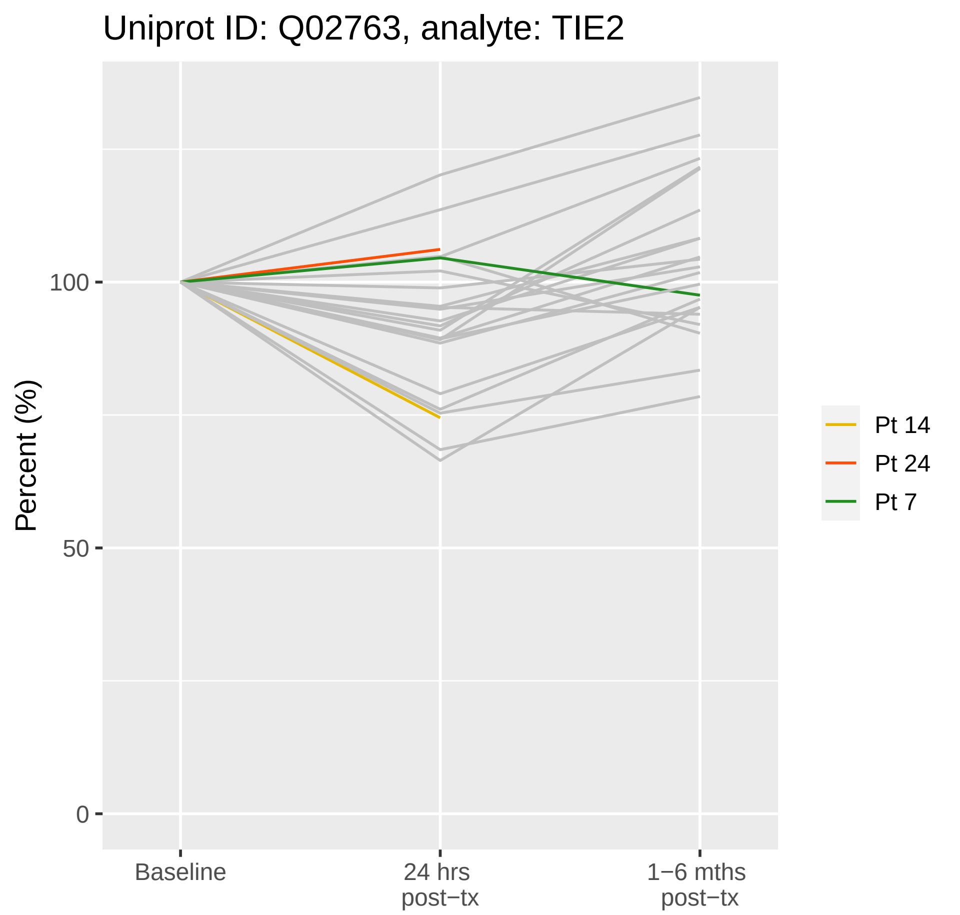
Uniprot ID: Q02763, analyte: TIE2
Percent (%)
100
50
0
Baseline
24 hrs post−tx
1−6 mths post−tx
Pt 14
Pt 24
Pt 7

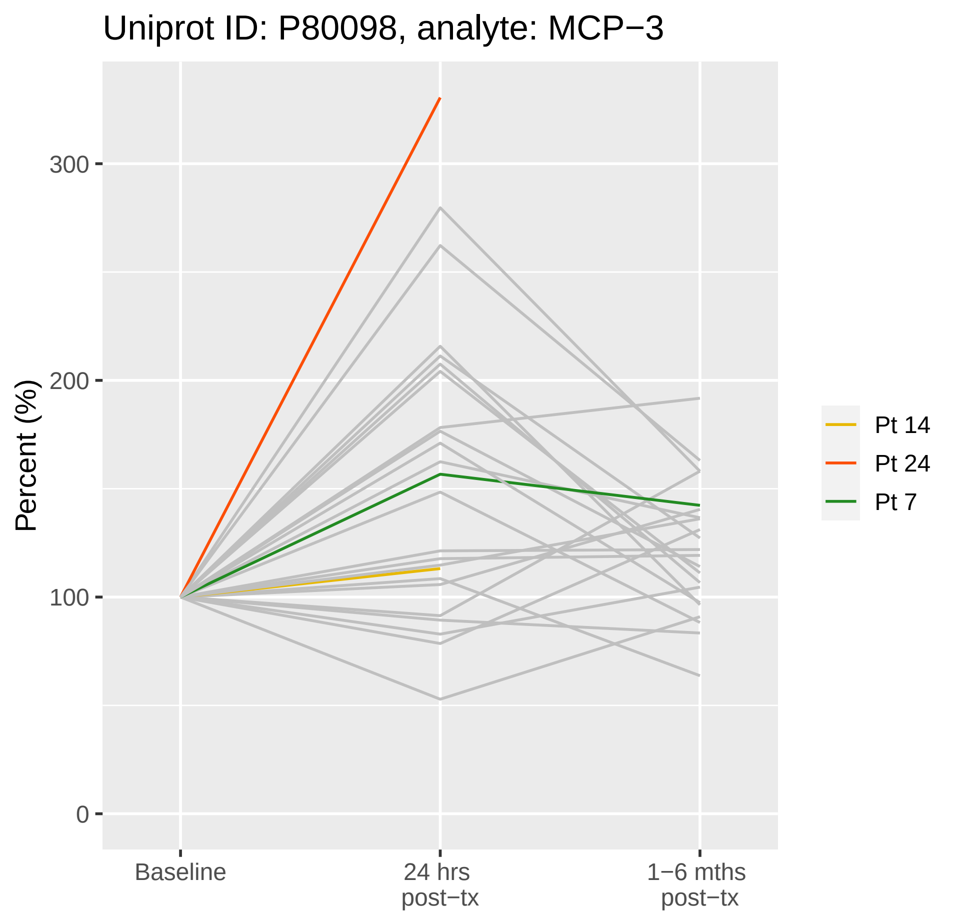

Uniprot ID: P80098, analyte: MCP−3
Percent (%)
300
200
100
0
Baseline
24 hrs
post−tx
1−6 mths
post−tx
Pt 14
Pt 24
Pt 7

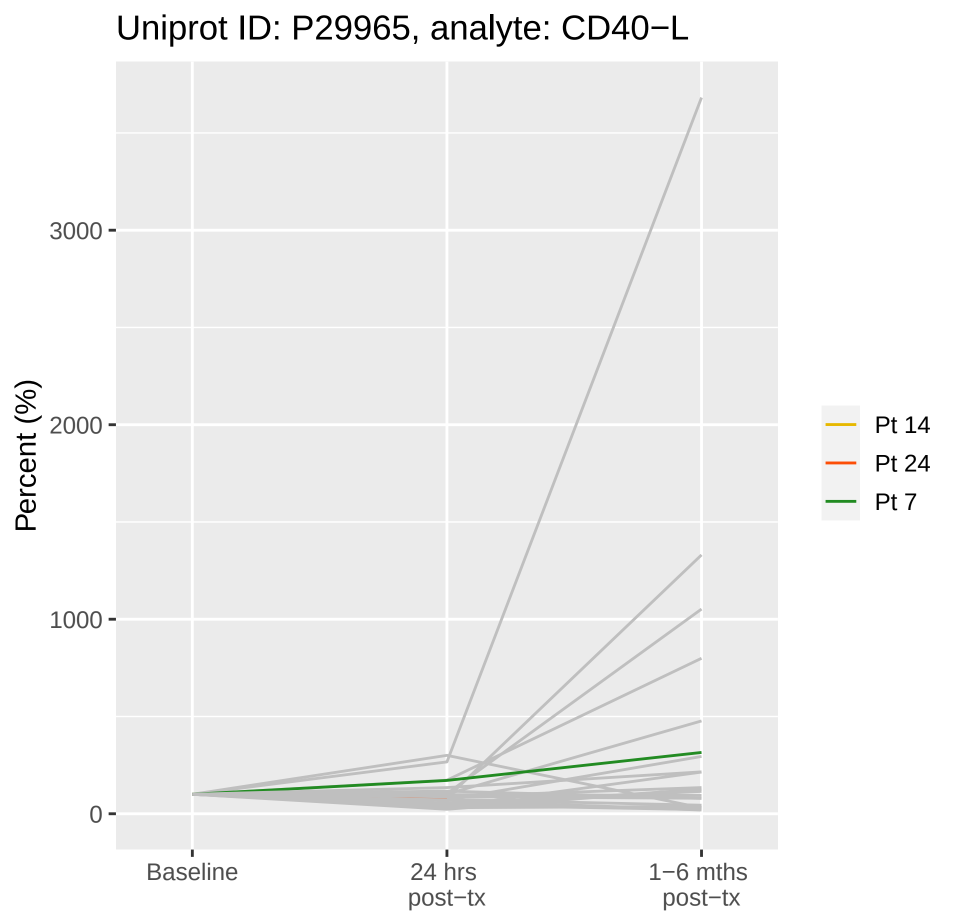
Uniprot ID: P29965, analyte: CD40−L
Percent (%)
3000
2000
1000
0
Baseline
24 hrs post−tx
1−6 mths post−tx
Pt 14
Pt 24
Pt 7

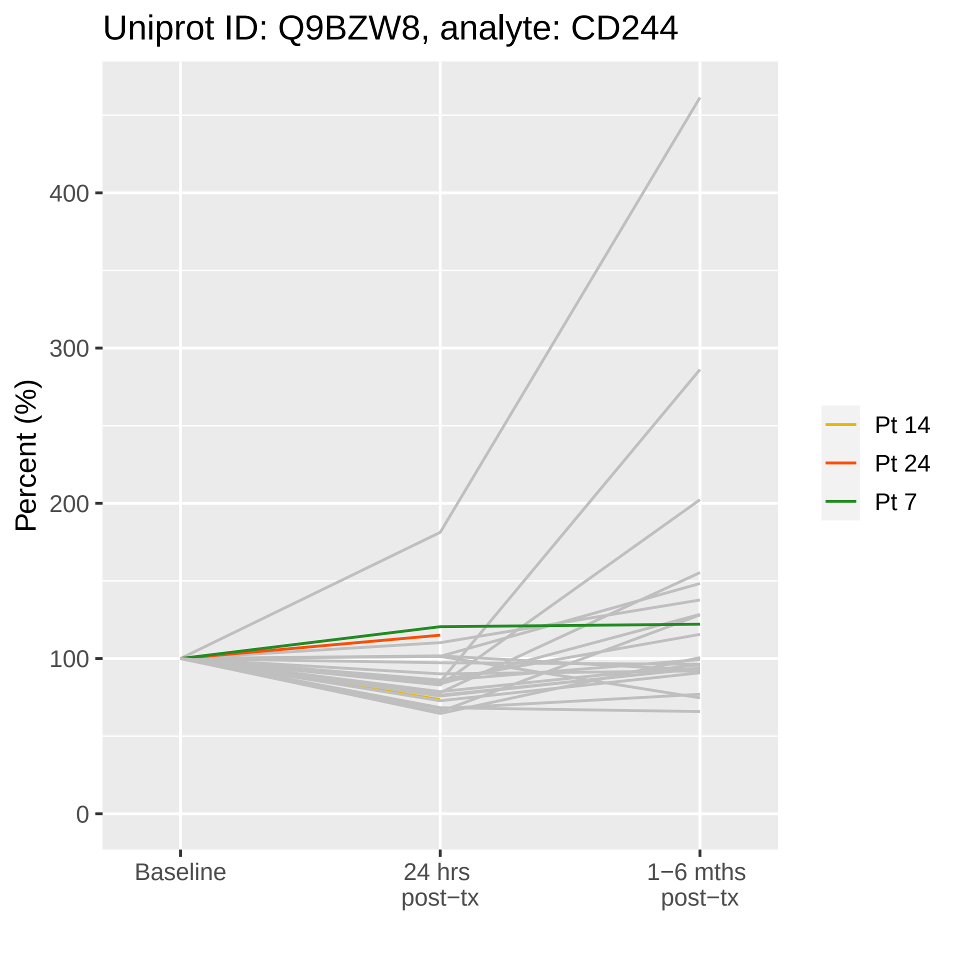

Uniprot ID: Q9BZW8, analyte: CD244
Percent (%)
0
100
200
300
400
Baseline
24 hrs post−tx
1−6 mths post−tx
Pt 14
Pt 24
Pt 7

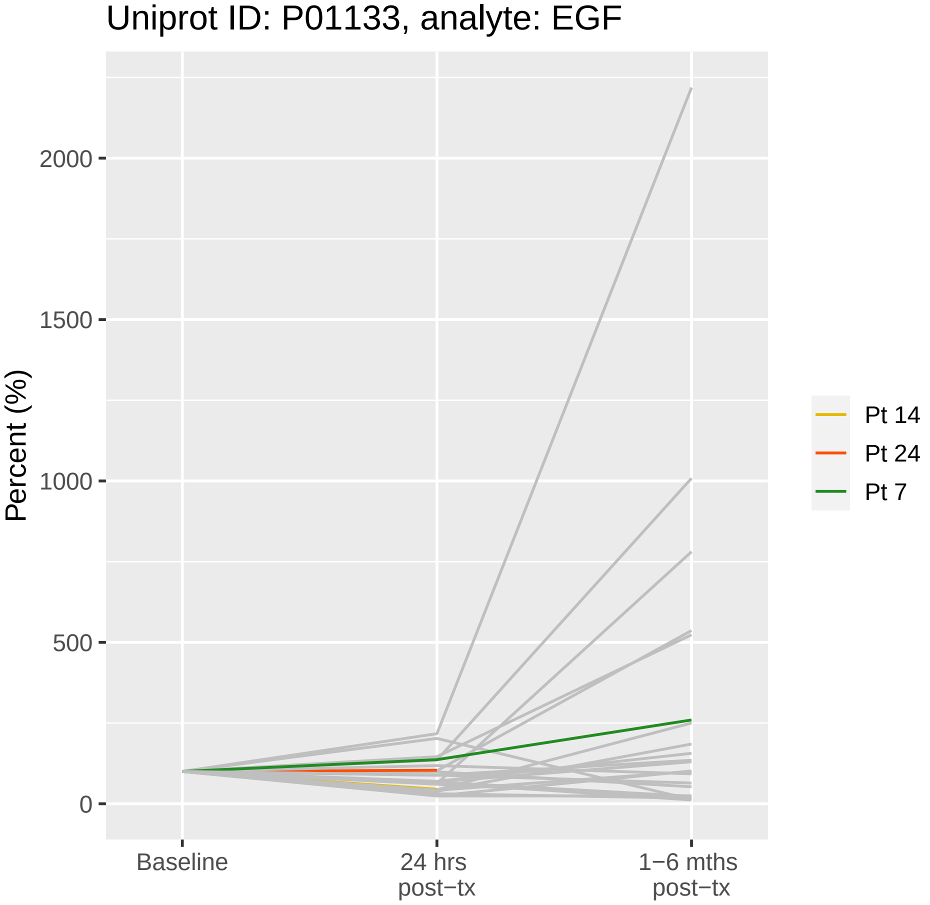
Uniprot ID: P01133, analyte: EGF
Percent (%)
2000
1500
1000
500
0
Baseline
24 hrs post−tx
1−6 mths post−tx
Pt 14
Pt 24
Pt 7

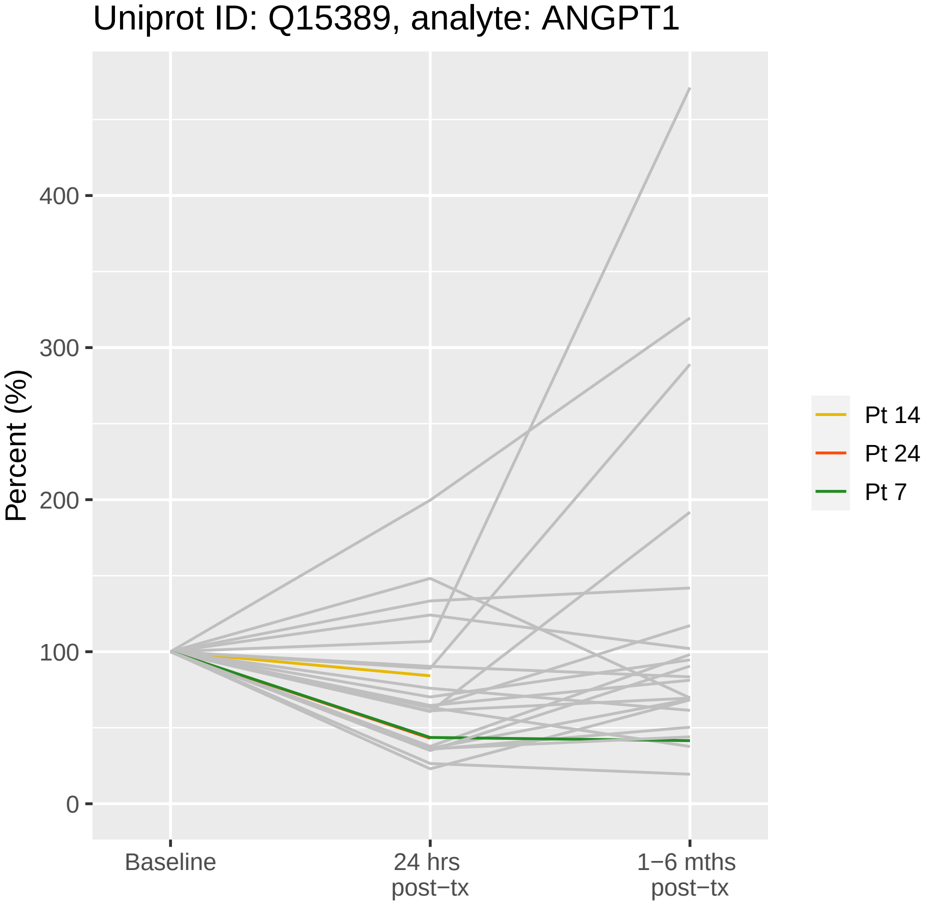

Uniprot ID: Q15389, analyte: ANGPT1
Percent (%)
400
300
200
100
0
Baseline
24 hrs
post−tx
1−6 mths
post−tx
Pt 14
Pt 24
Pt 7

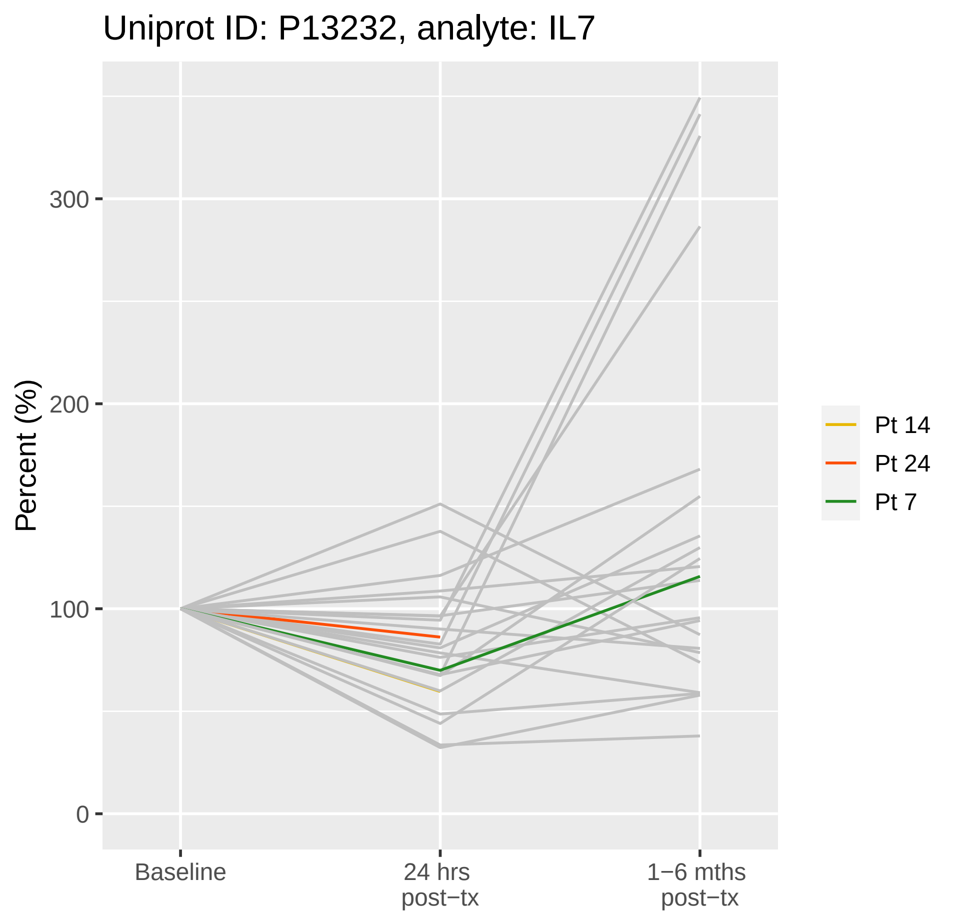
Uniprot ID: P13232, analyte: IL7
Percent (%)
300
200
100
0
Baseline
24 hrs post−tx
1−6 mths post−tx
Pt 14
Pt 24
Pt 7

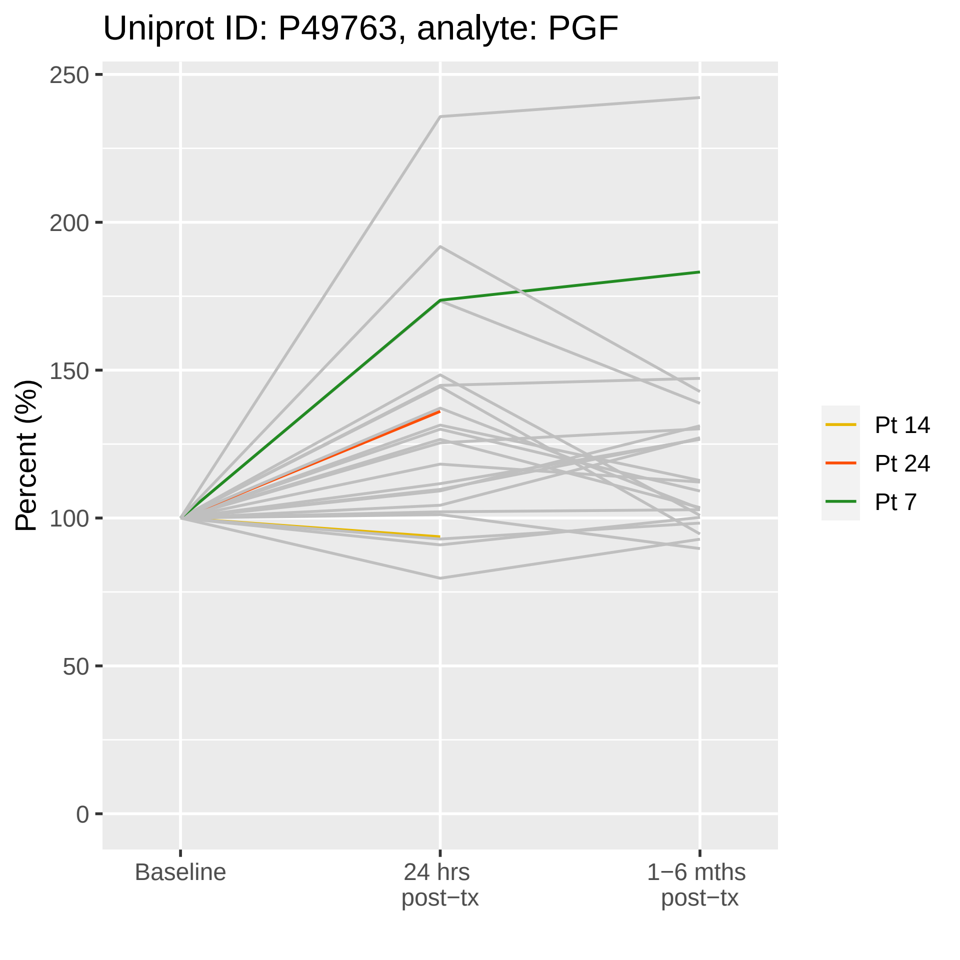

Uniprot ID: P49763, analyte: PGF
Percent (%)
250
200
150
100
50
0
Baseline
24 hrs post−tx
1−6 mths post−tx
Pt 14
Pt 24
Pt 7

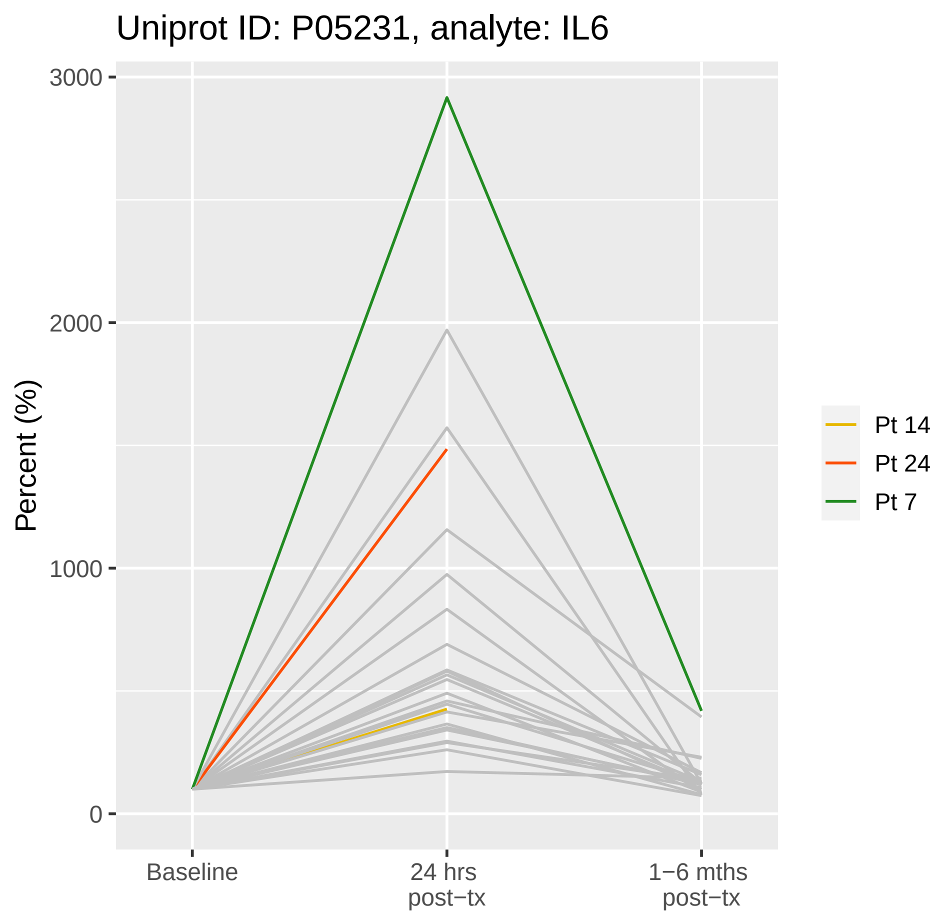
Uniprot ID: P05231, analyte: IL6
Percent (%)
3000
2000
1000
0
Baseline
24 hrs post−tx
1−6 mths post−tx
Pt 14
Pt 24
Pt 7

Uniprot ID: Q9Y653, analyte: ADGRG1

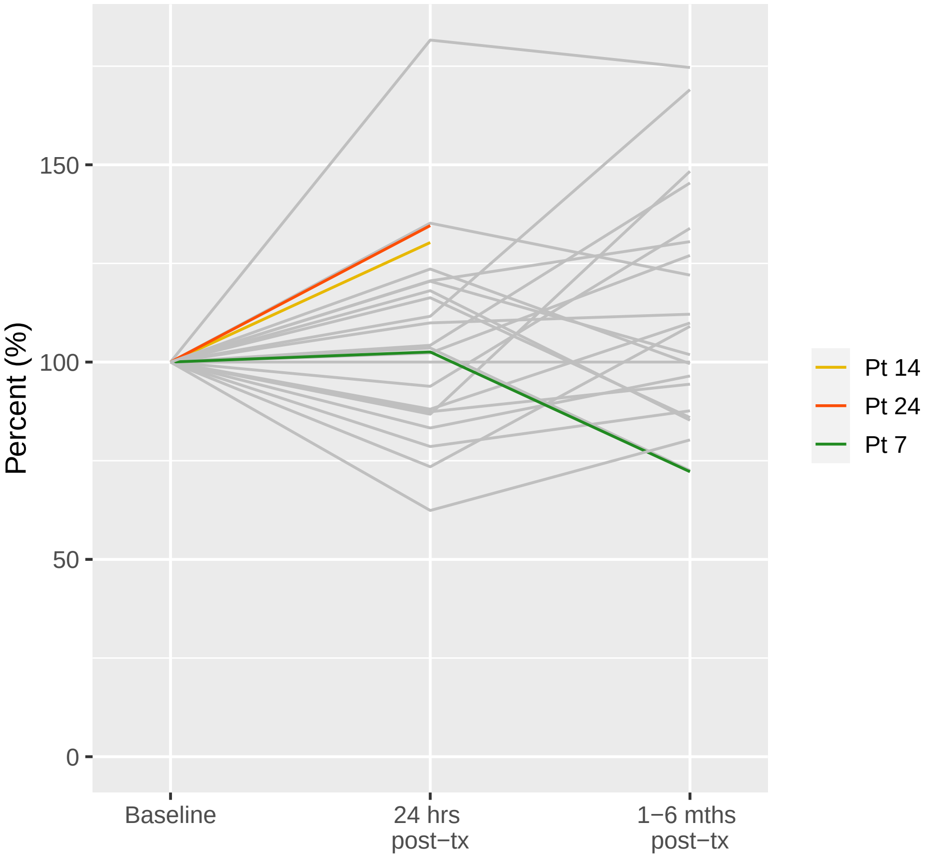

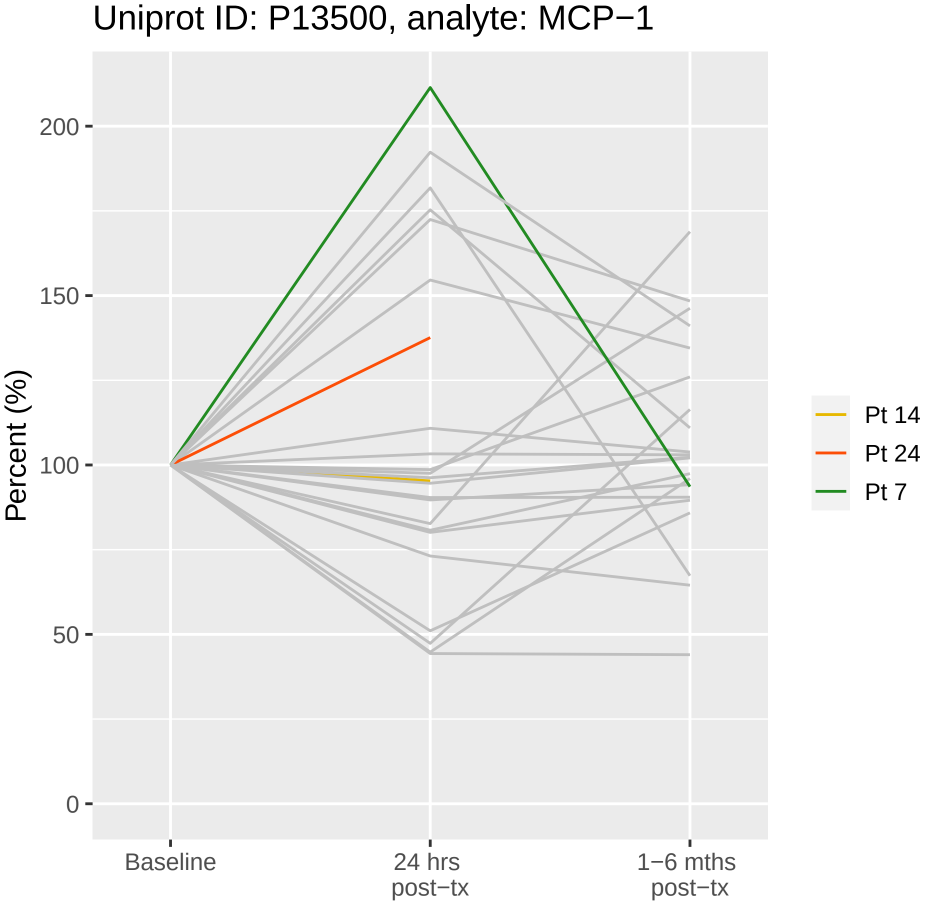

Uniprot ID: P13500, analyte: MCP−1
Percent (%)
200
150
100
50
0
Baseline
24 hrs post−tx
1−6 mths post−tx
Pt 14
Pt 24
Pt 7

Uniprot ID: O95727, analyte: CRTAM

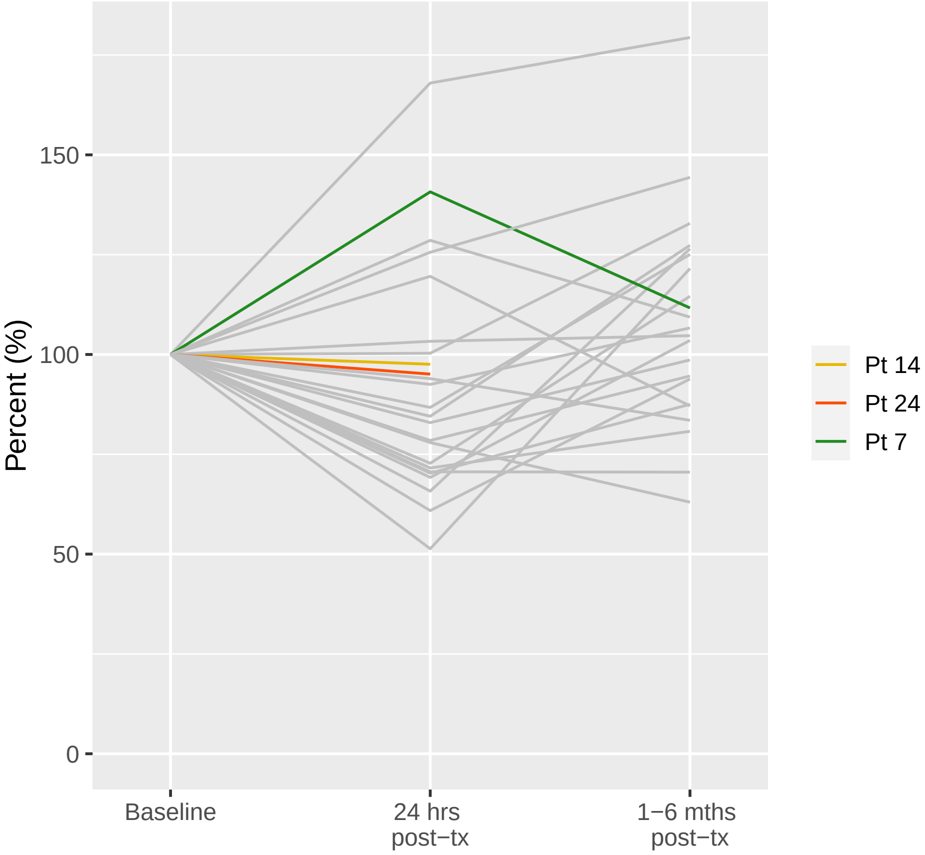

Uniprot ID: O14625, analyte: CXCL11

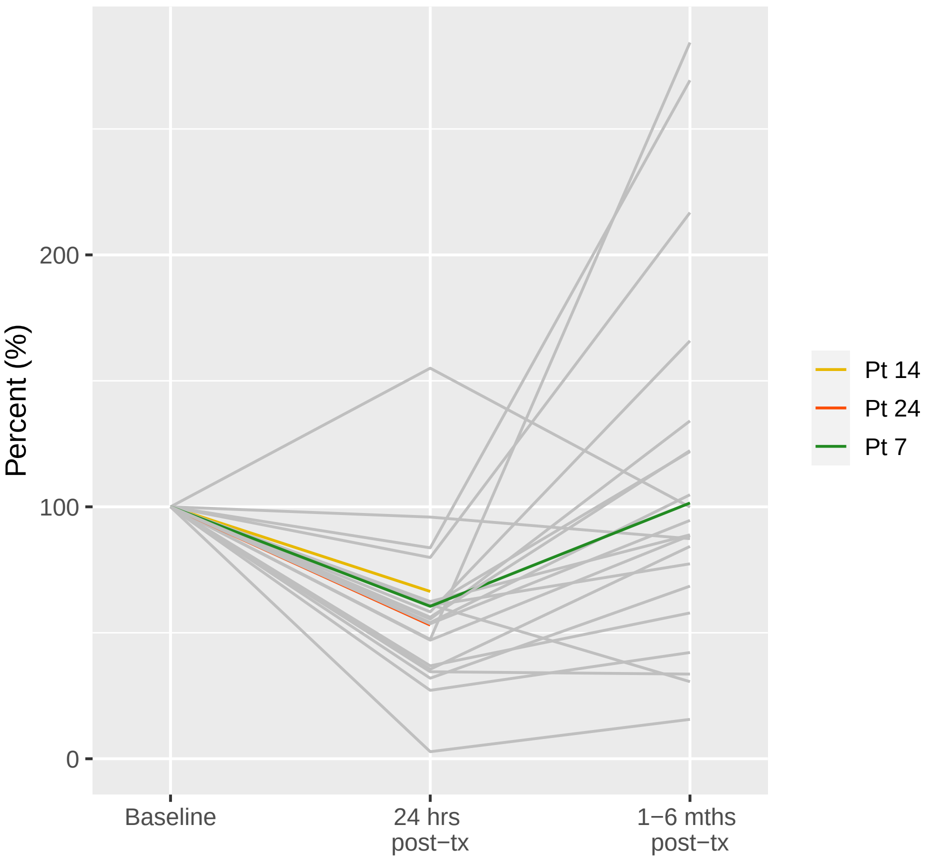

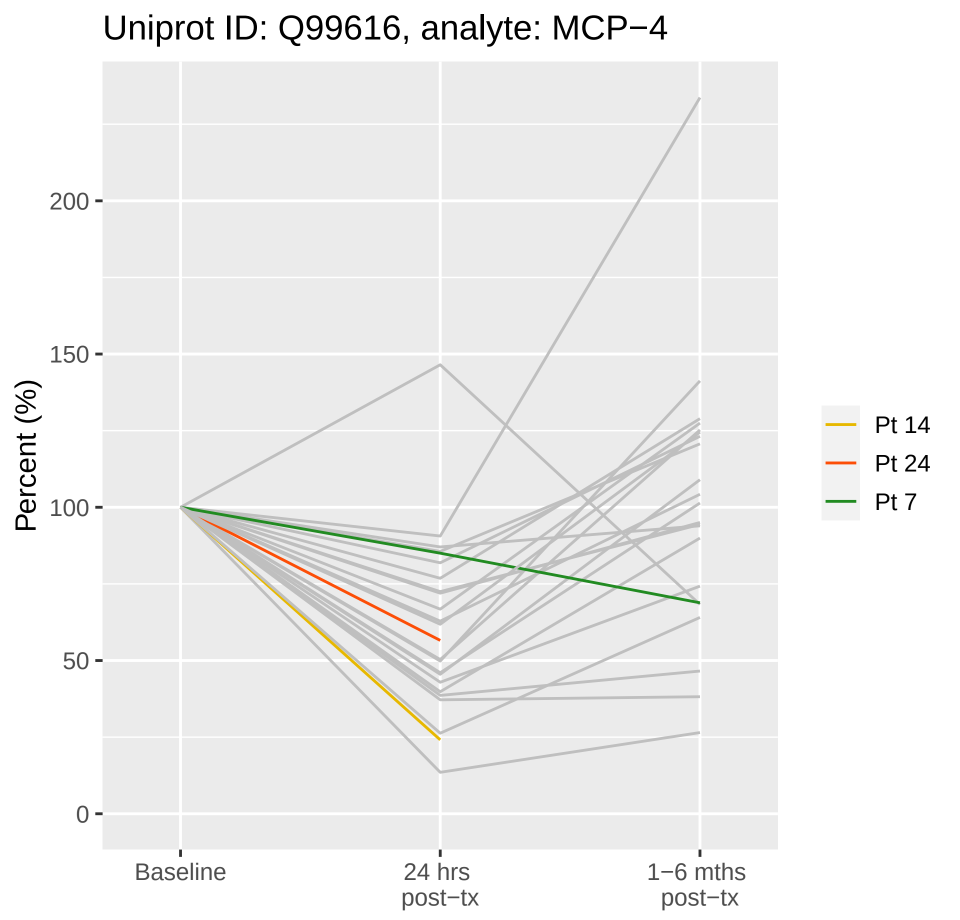
Uniprot ID: Q99616, analyte: MCP−4
Percent (%)
200
150
100
50
0
Baseline
24 hrs post−tx
1−6 mths post−tx
Pt 14
Pt 24
Pt 7

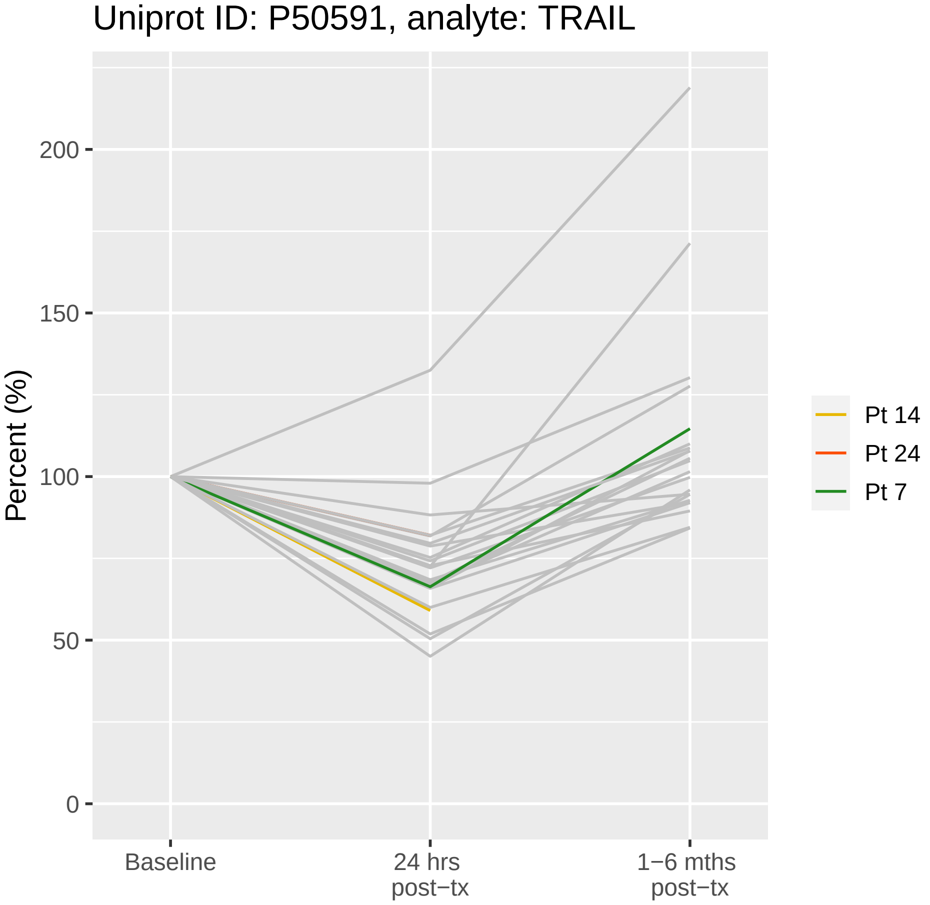
Uniprot ID: P50591, analyte: TRAIL
Percent (%)
200
150
100
50
0
Baseline
24 hrs post−tx
1−6 mths post−tx
Pt 14
Pt 24
Pt 7

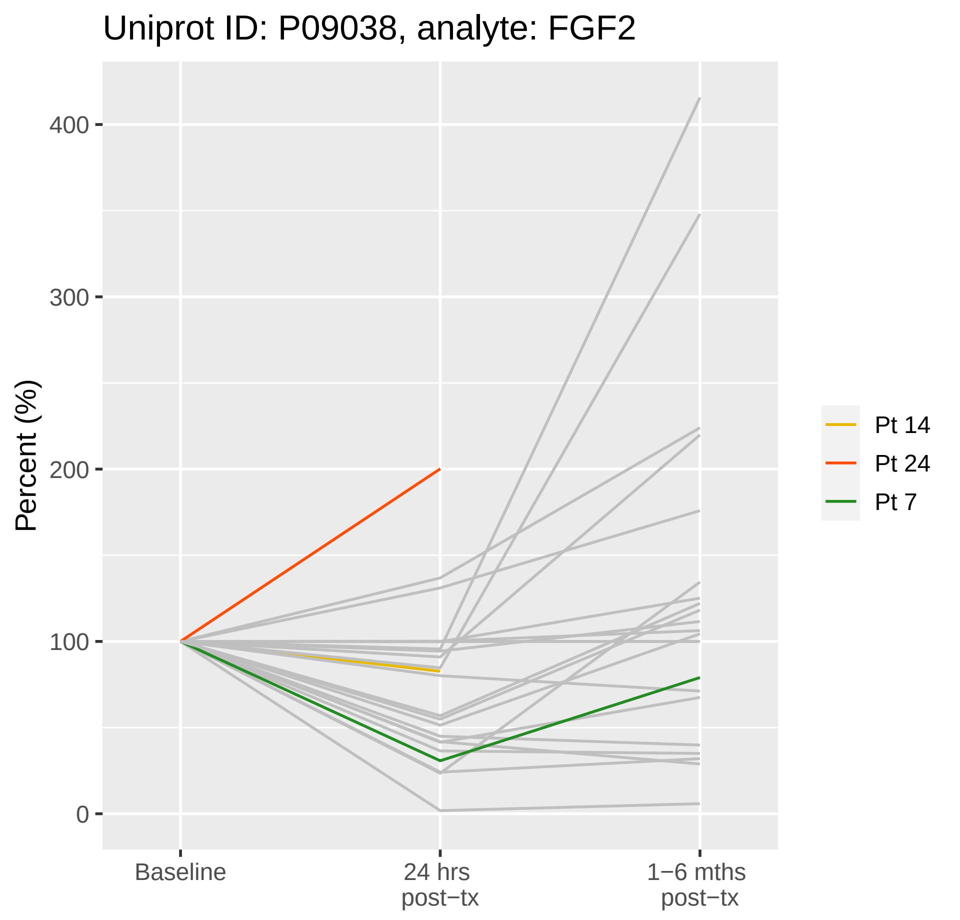

Uniprot ID: P09038, analyte: FGF2
Percent (%)
400
300
200
100
0
Baseline
24 hrs post−tx
1−6 mths post−tx
Pt 14
Pt 24
Pt 7

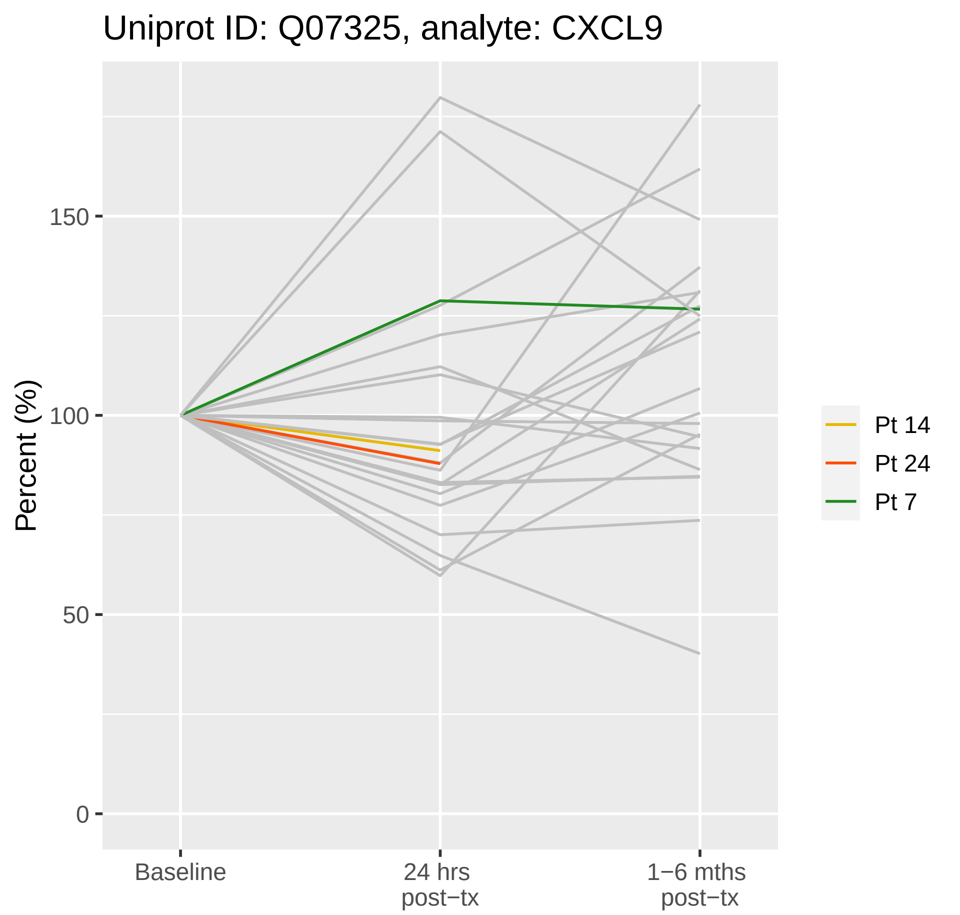

Uniprot ID: Q07325, analyte: CXCL9
Percent (%)
150
100
50
0
Baseline
24 hrs post−tx
1−6 mths post−tx
Pt 14
Pt 24
Pt 7

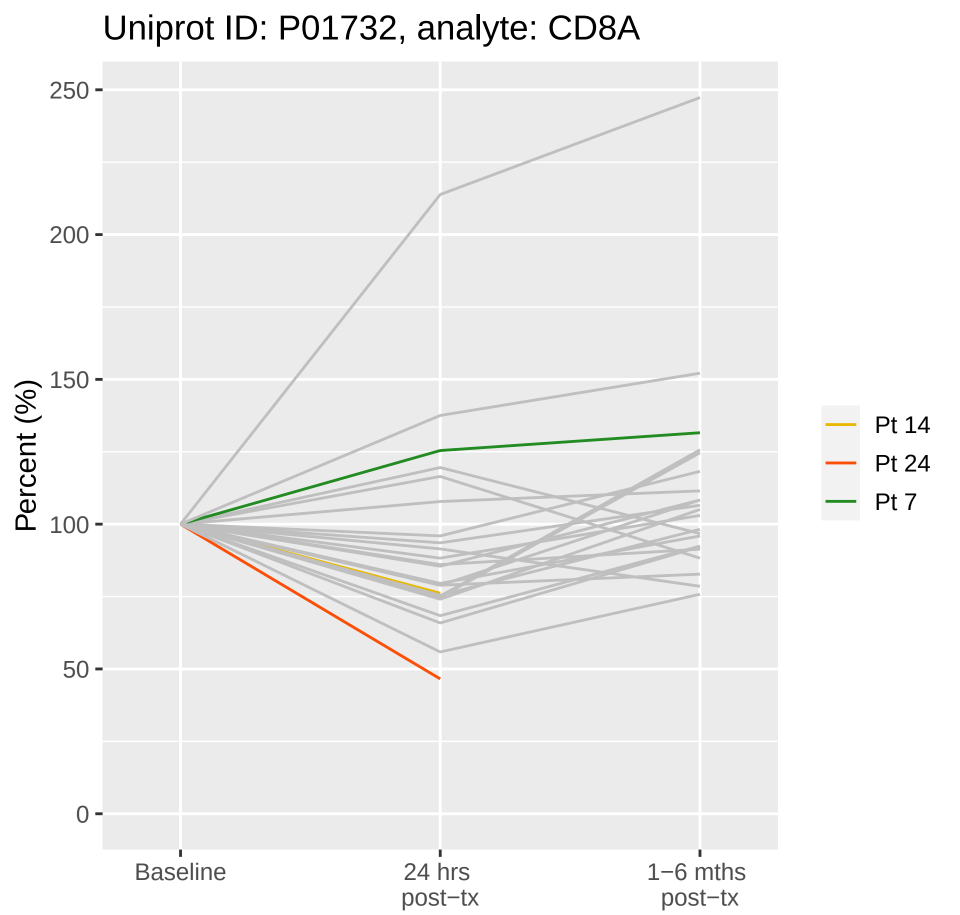
Uniprot ID: P01732, analyte: CD8A
Percent (%)
250
200
150
100
50
0
Baseline
24 hrs post−tx
1−6 mths post−tx
Pt 14
Pt 24
Pt 7

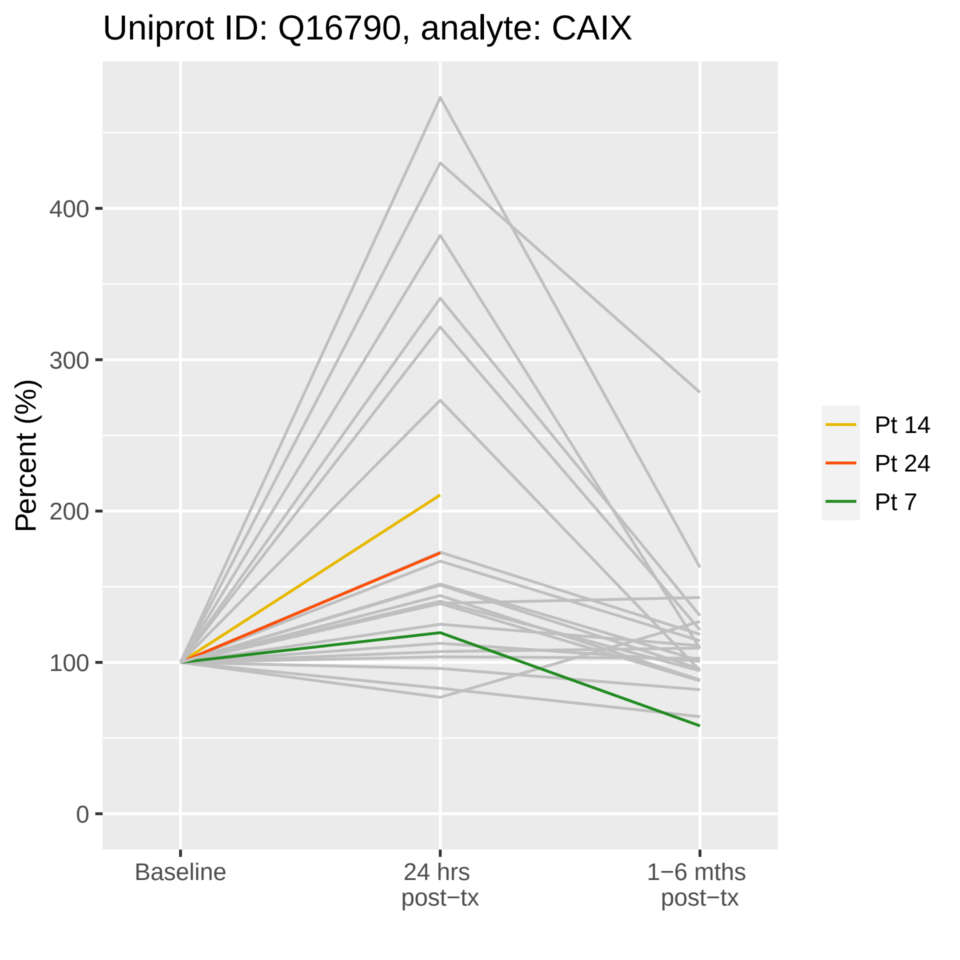

Uniprot ID: Q16790, analyte: CAIX
Percent (%)
400
300
200
100
0
Baseline
24 hrs post−tx
1−6 mths post−tx
Pt 14
Pt 24
Pt 7

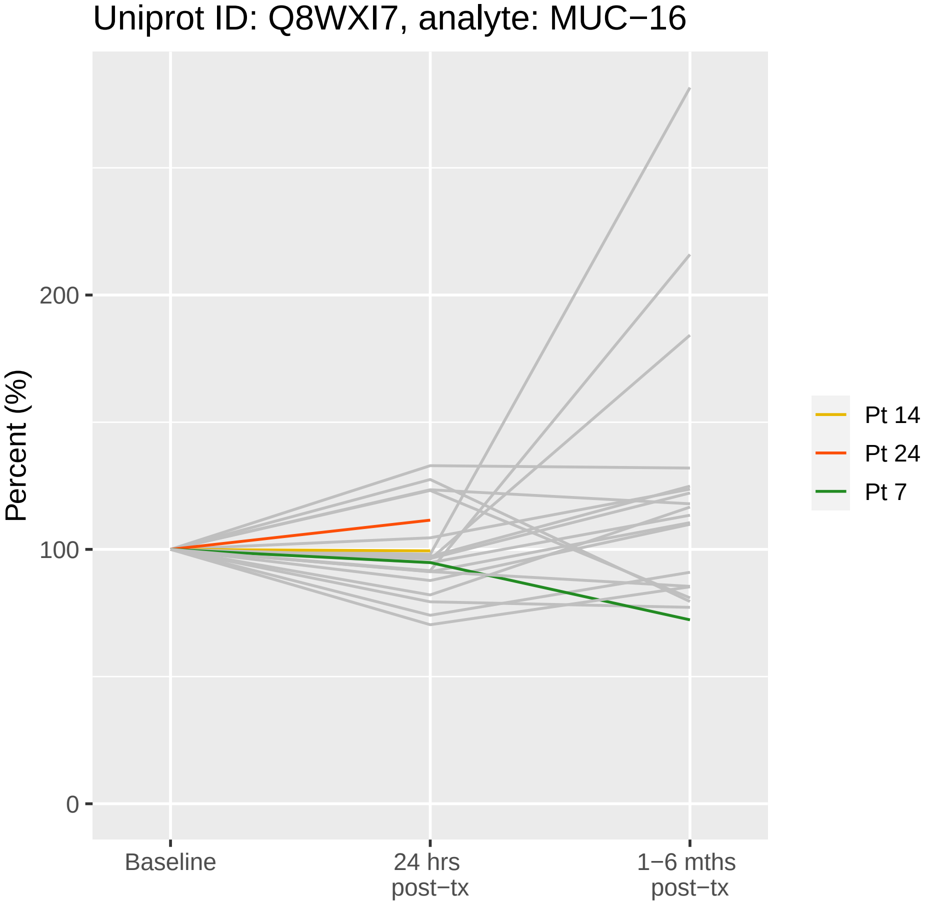

Uniprot ID: Q8WXI7, analyte: MUC−16
Percent (%)
200
100
0
Baseline
24 hrs
post−tx
1−6 mths
post−tx
Pt 14
Pt 24
Pt 7

Uniprot ID: P00813, analyte: ADA

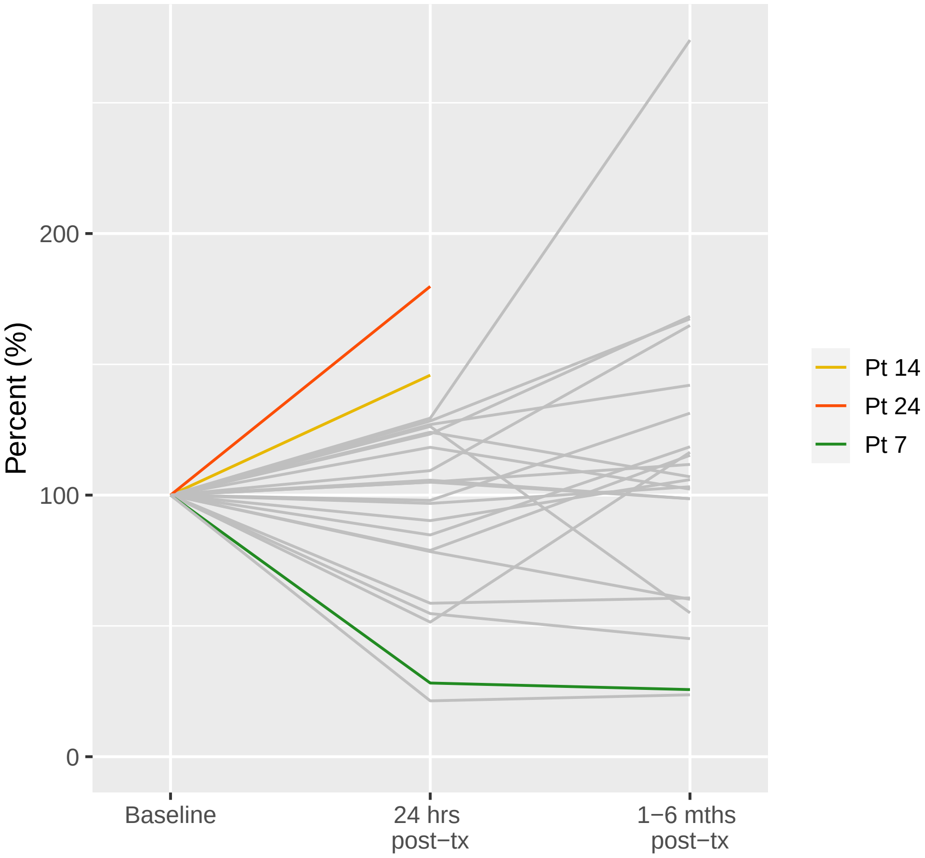

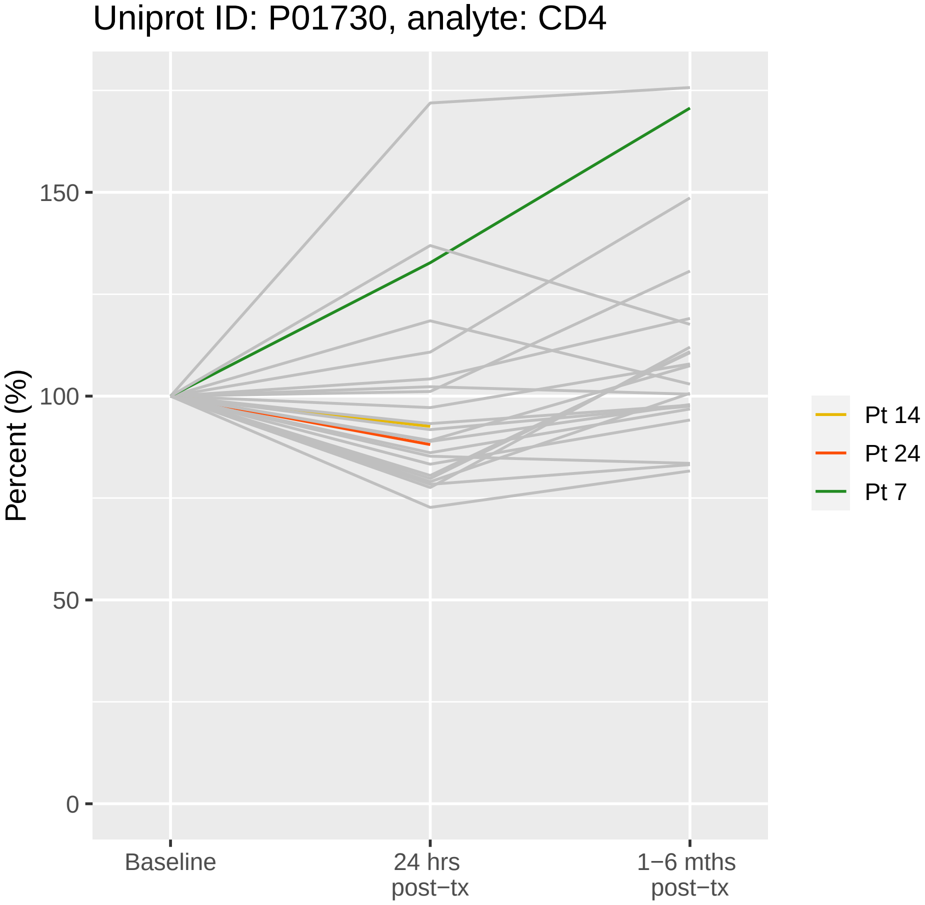

Uniprot ID: P01730, analyte: CD4
Percent (%)
150
100
50
0
Baseline
24 hrs
post−tx
1−6 mths
post−tx
Pt 14
Pt 24
Pt 7

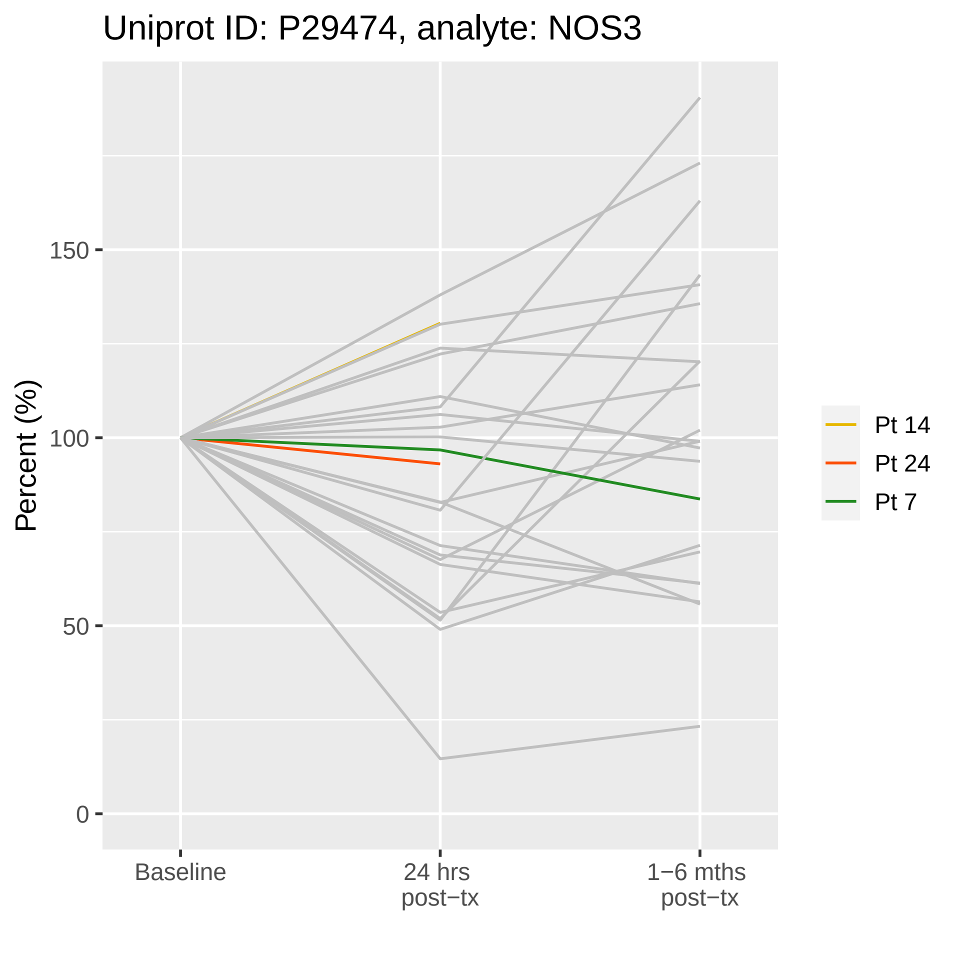

Uniprot ID: P29474, analyte: NOS3
Percent (%)
150
100
50
0
Baseline
24 hrs
post−tx
1−6 mths
post−tx
Pt 14
Pt 24
Pt 7

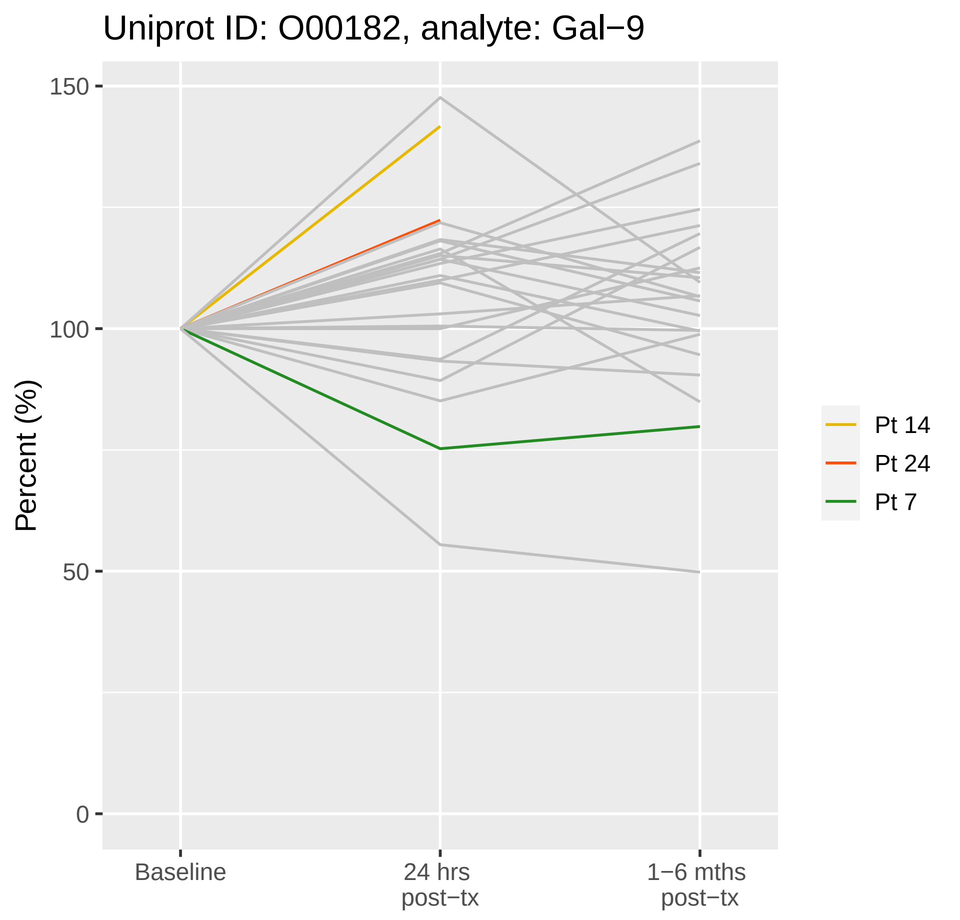

Uniprot ID: O00182, analyte: Gal−9
Percent (%)
150
100
50
0
Baseline
24 hrs post−tx
1−6 mths post−tx
Pt 14
Pt 24
Pt 7

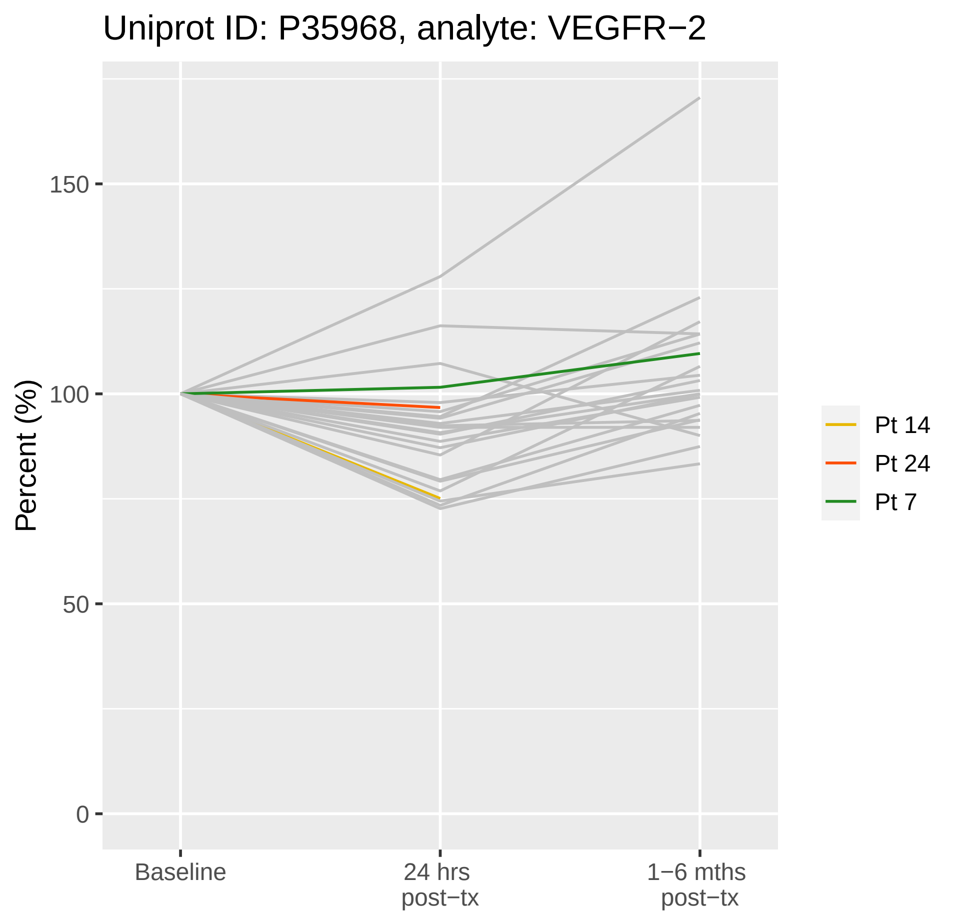
Uniprot ID: P35968, analyte: VEGFR−2
Percent (%)
150
100
50
0
Baseline
24 hrs
post−tx
1−6 mths
post−tx
Pt 14
Pt 24
Pt 7

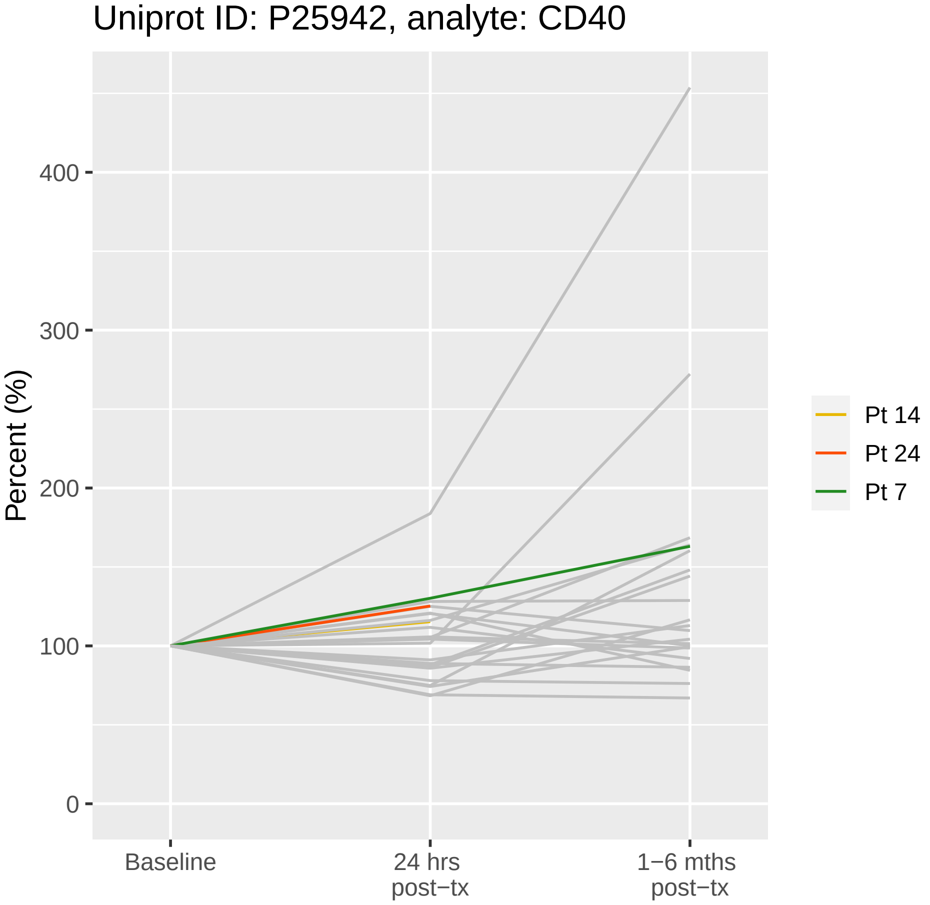

Uniprot ID: P25942, analyte: CD40
Percent (%)
400
300
200
100
0
Baseline
24 hrs
post−tx
1−6 mths
post−tx
Pt 14
Pt 24
Pt 7

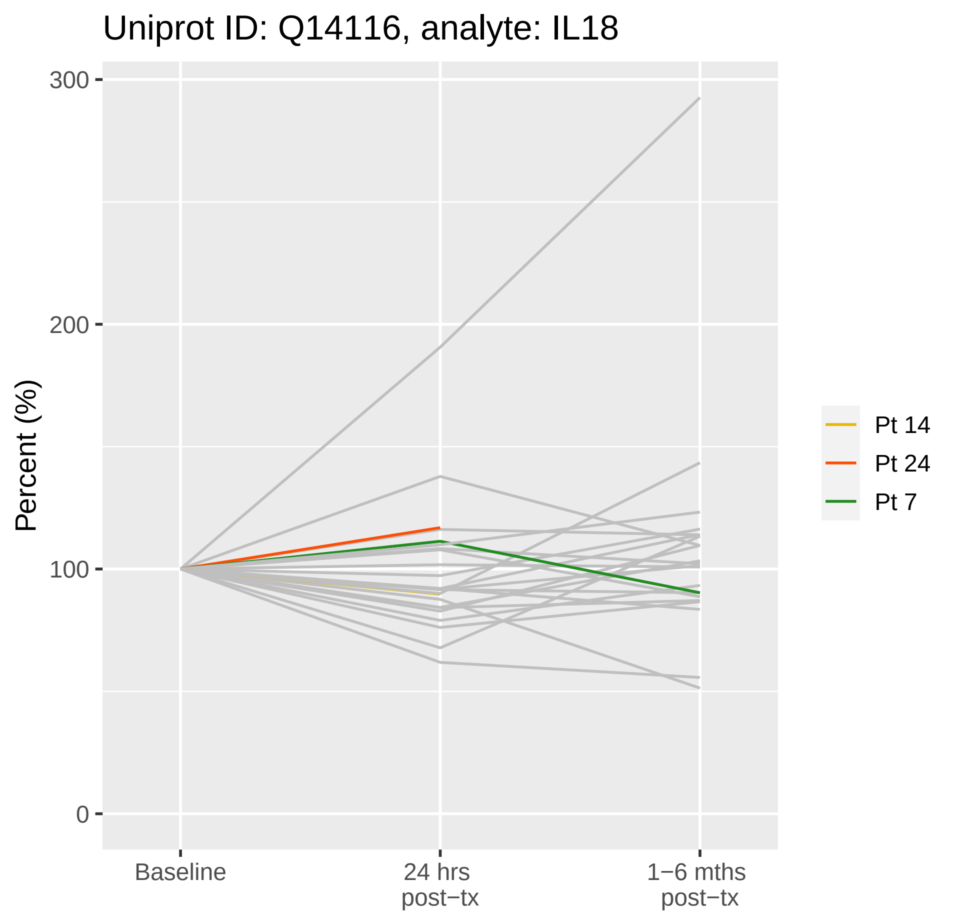

Uniprot ID: Q14116, analyte: IL18
Percent (%)
300
200
100
0
Baseline
24 hrs post−tx
1−6 mths post−tx
Pt 14
Pt 24
Pt 7

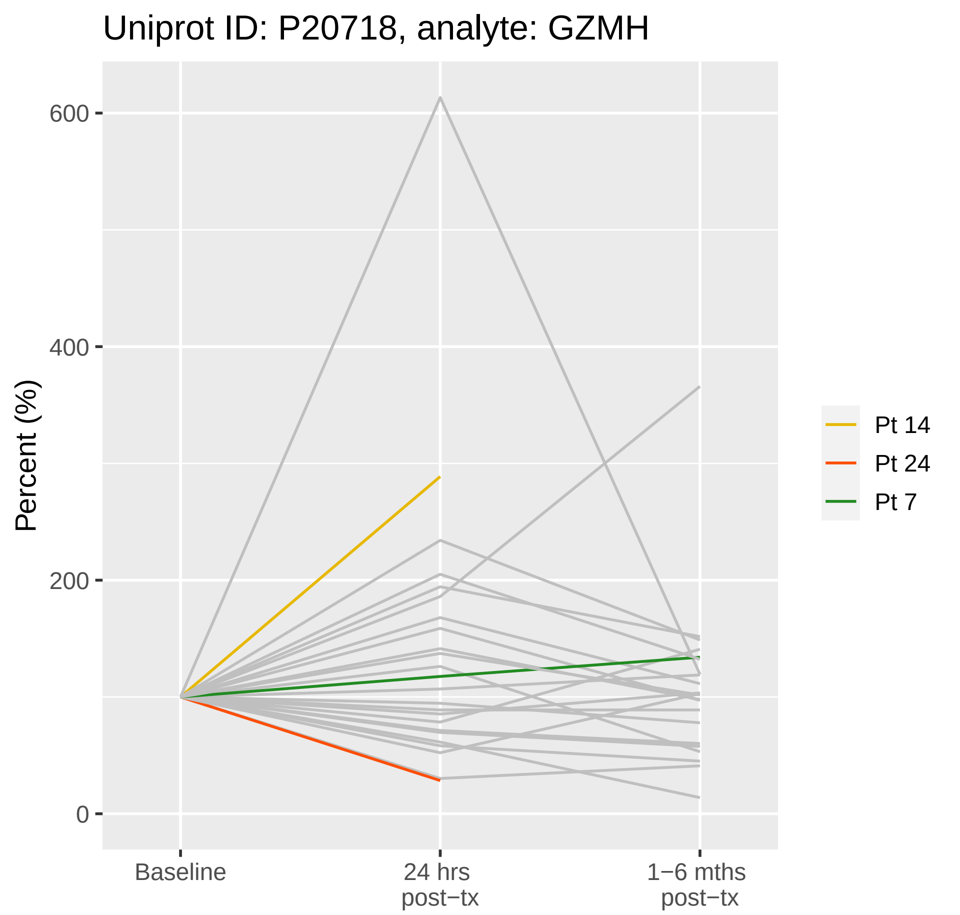
Uniprot ID: P20718, analyte: GZMH
Percent (%)
600
400
200
0
Baseline
24 hrs post−tx
1−6 mths post−tx
Pt 14
Pt 24
Pt 7

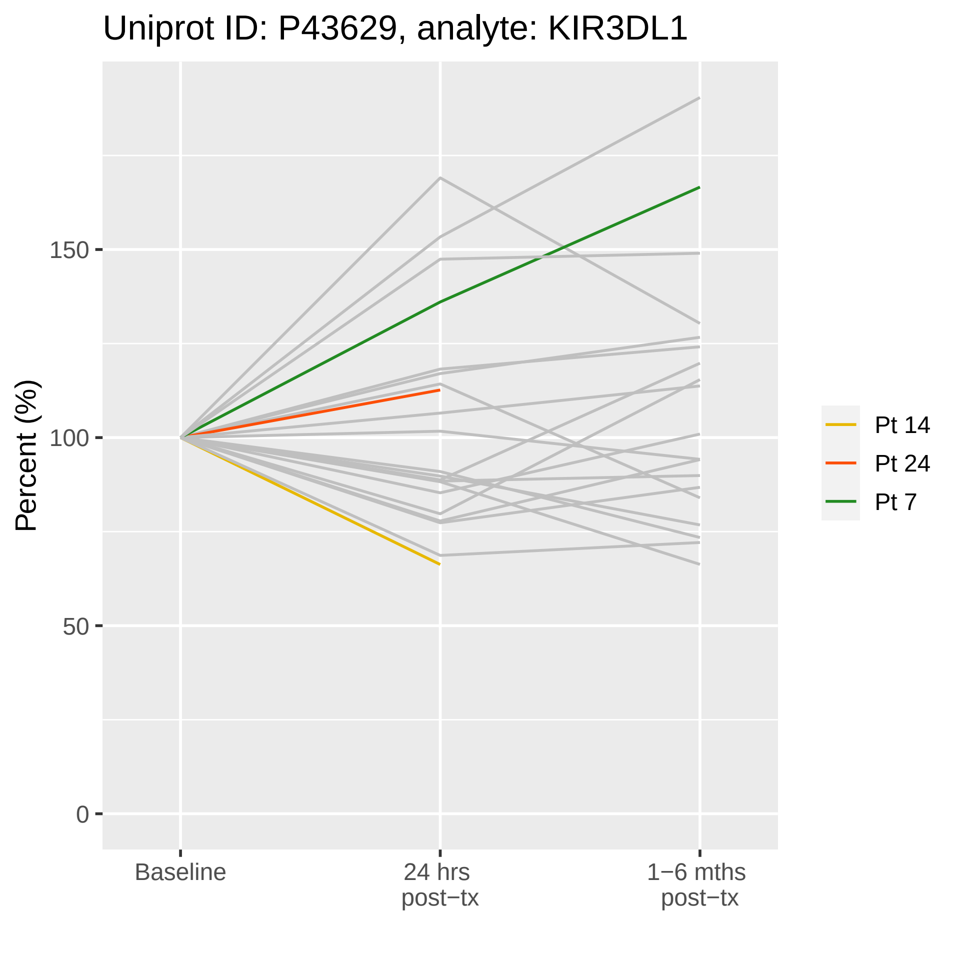

Uniprot ID: P43629, analyte: KIR3DL1
Percent (%)
150
100
50
0
Baseline
24 hrs post−tx
1−6 mths post−tx
Pt 14
Pt 24
Pt 7

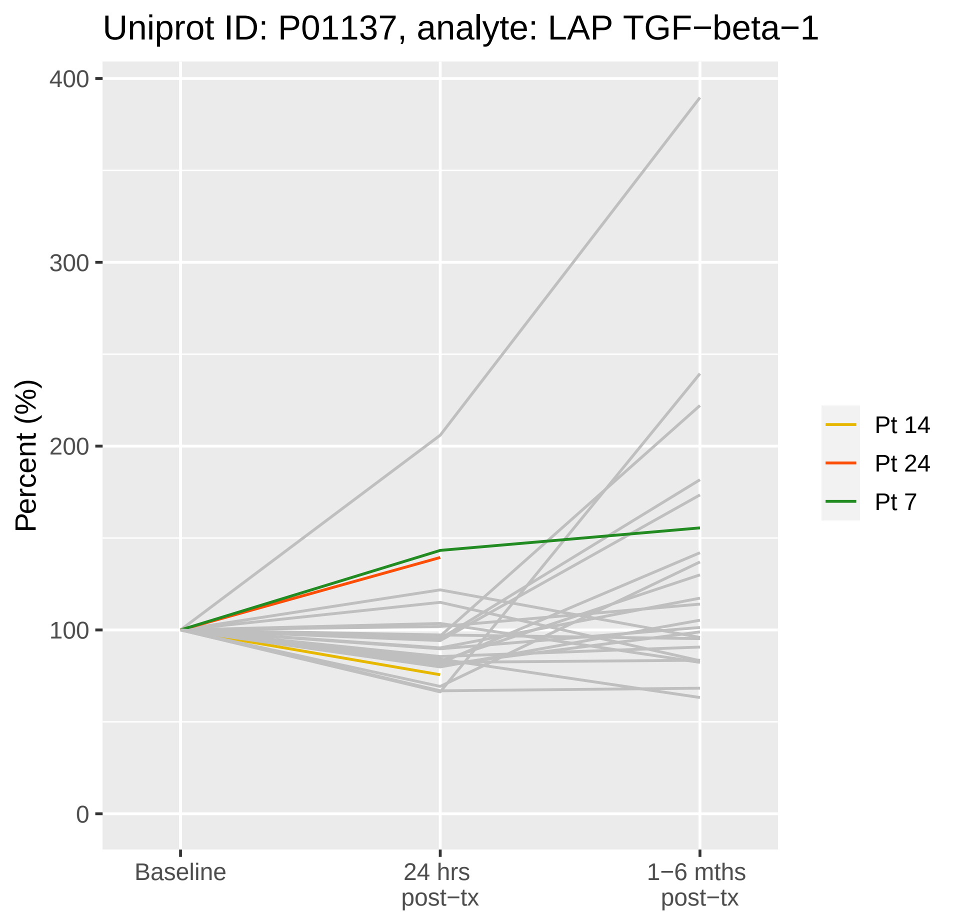

Uniprot ID: P01137, analyte: LAP TGF−beta−1
Percent (%)
400
300
200
100
0
Baseline
24 hrs
post−tx
1−6 mths
post−tx
Pt 14
Pt 24
Pt 7

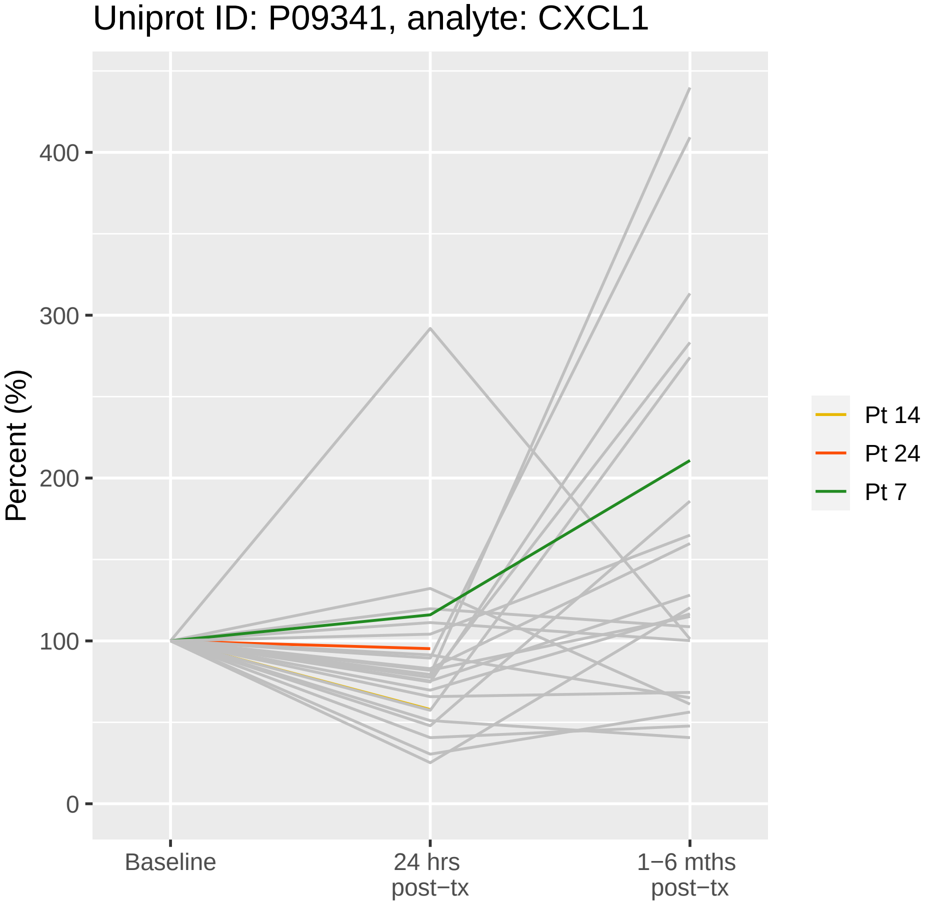

Uniprot ID: P09341, analyte: CXCL1
Percent (%)
400
300
200
100
0
Baseline
24 hrs post−tx
1−6 mths post−tx
Pt 14
Pt 24
Pt 7

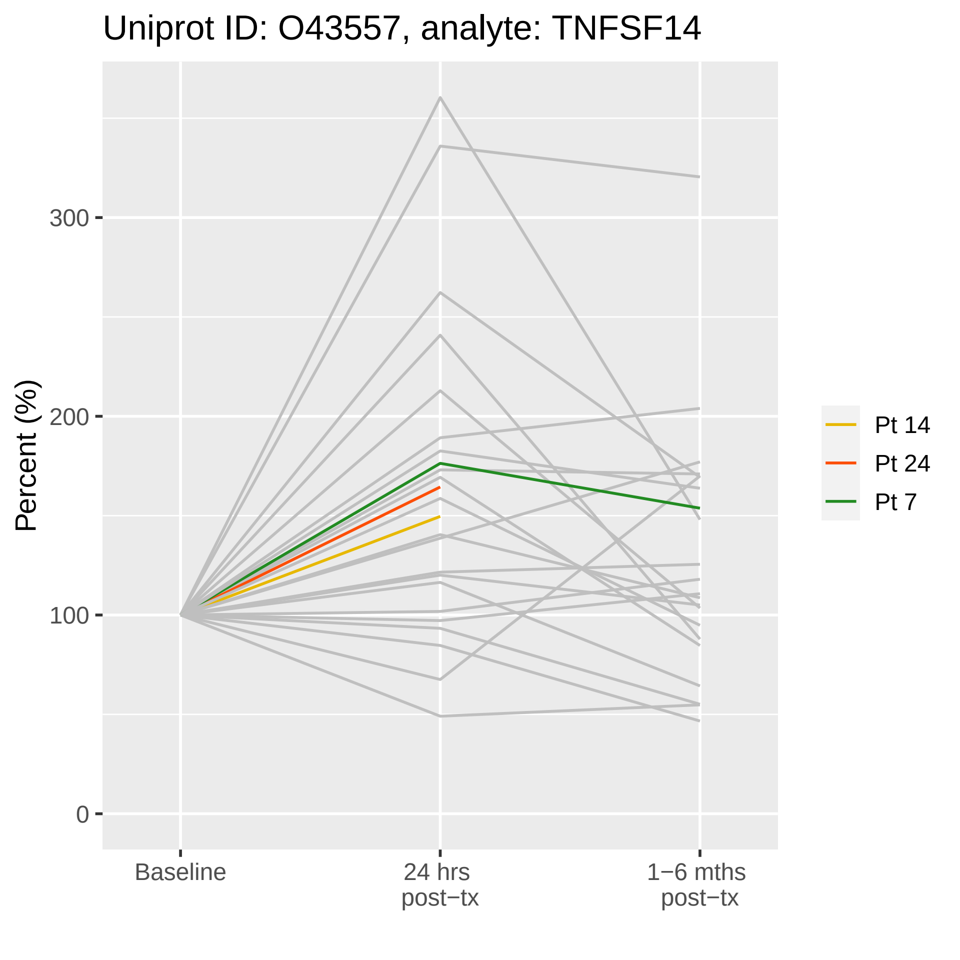

Uniprot ID: O43557, analyte: TNFSF14
Percent (%)
300
200
100
0
Baseline
24 hrs
post−tx
1−6 mths
post−tx
Pt 14
Pt 24
Pt 7

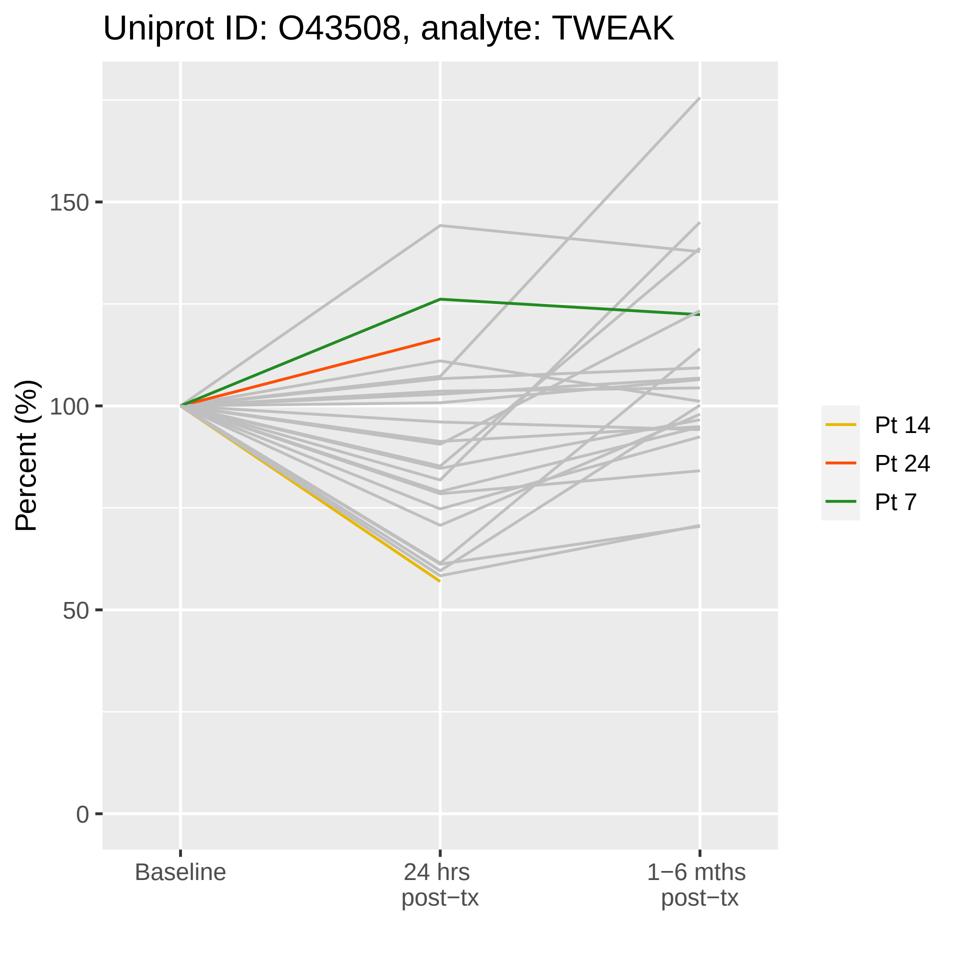

Uniprot ID: O43508, analyte: TWEAK
Percent (%)
150
100
50
0
Baseline
24 hrs post−tx
1−6 mths post−tx
Pt 14
Pt 24
Pt 7

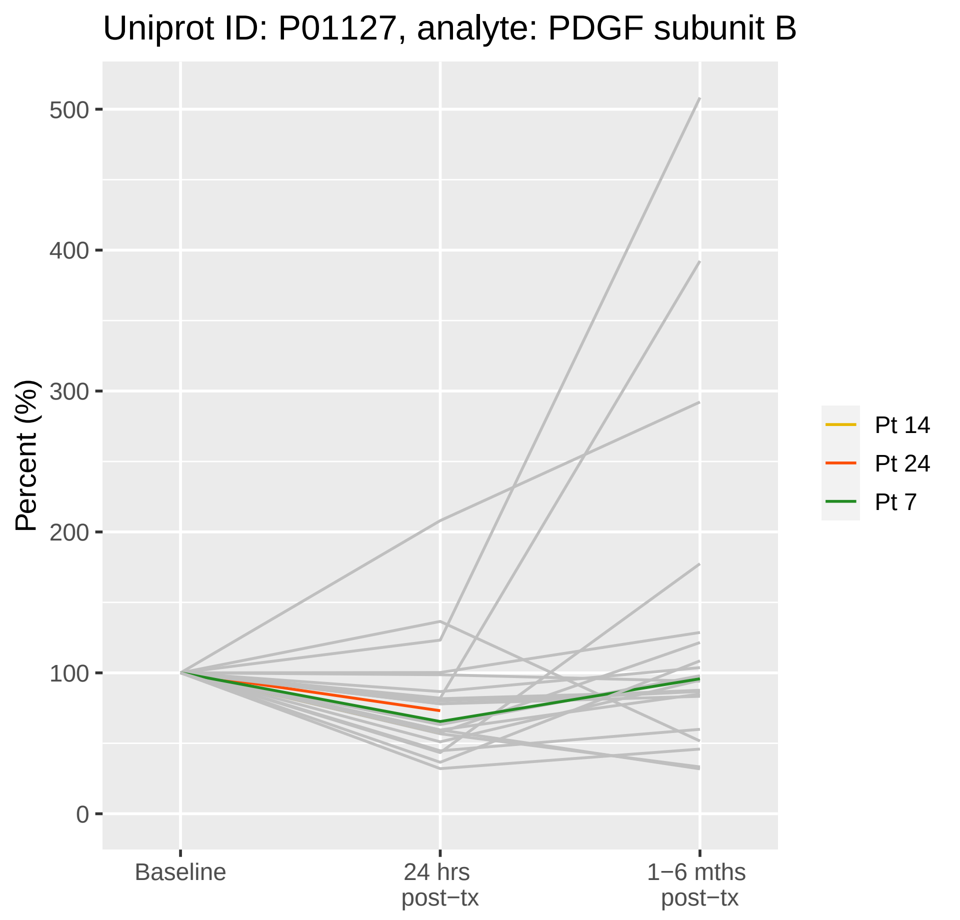

Uniprot ID: P01127, analyte: PDGF subunit B
Percent (%)
500
400
300
200
100
0
Baseline
24 hrs post−tx
1−6 mths post−tx
Pt 14
Pt 24
Pt 7

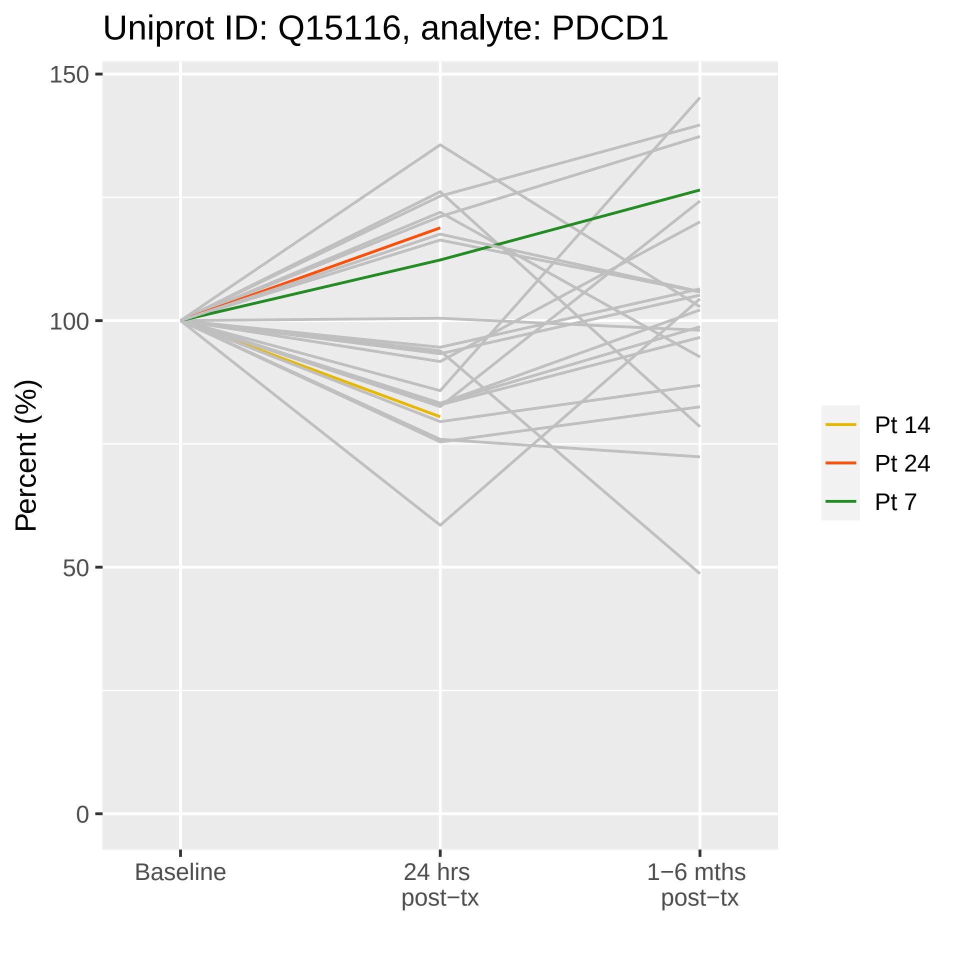

Uniprot ID: Q15116, analyte: PDCD1
Percent (%)
150
100
50
0
Baseline
24 hrs post−tx
1−6 mths post−tx
Pt 14
Pt 24
Pt 7

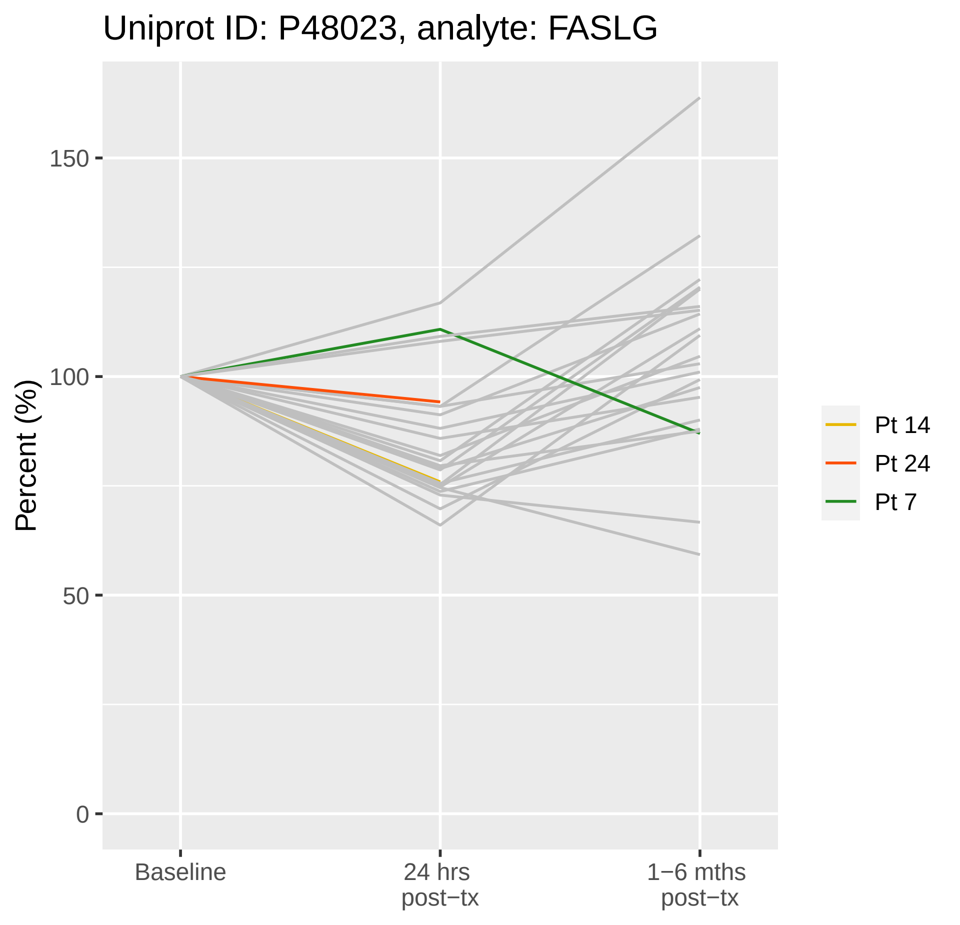

Uniprot ID: P48023, analyte: FASLG
Percent (%)
150
100
50
0
Baseline
24 hrs post−tx
1−6 mths post−tx
Pt 14
Pt 24
Pt 7

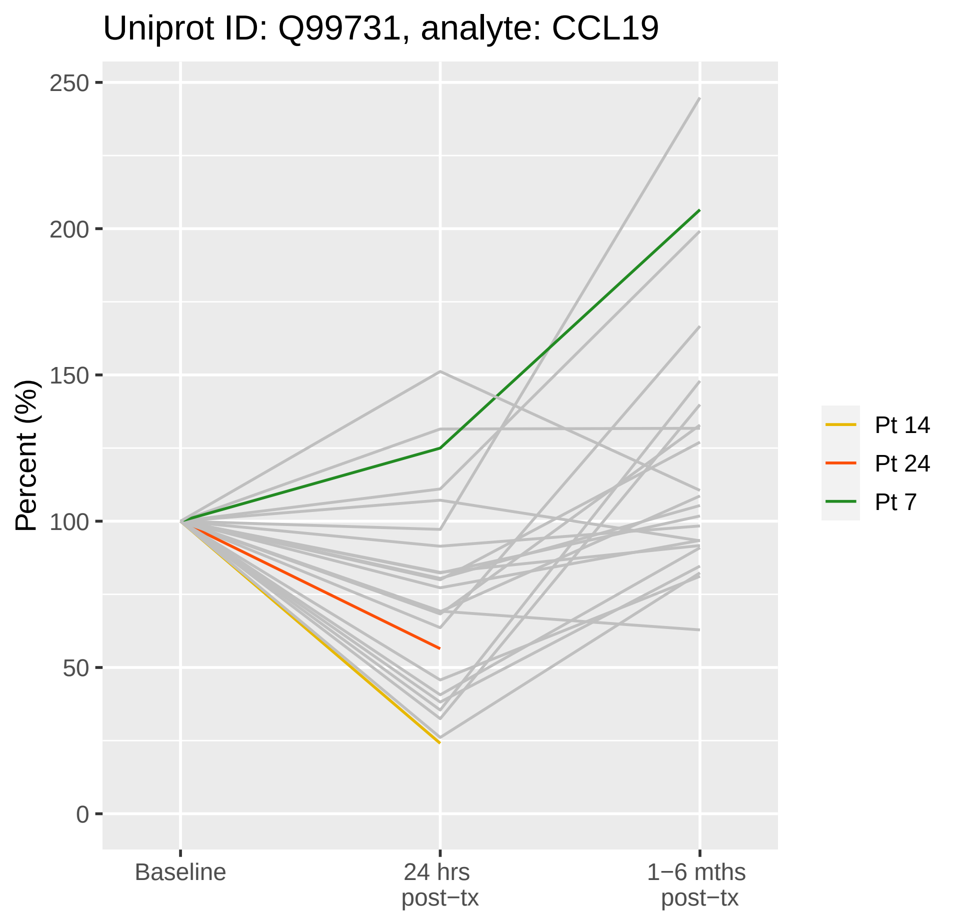

Uniprot ID: Q99731, analyte: CCL19
Percent (%)
250
200
150
100
50
0
Baseline
24 hrs post−tx
1−6 mths post−tx
Pt 14
Pt 24
Pt 7

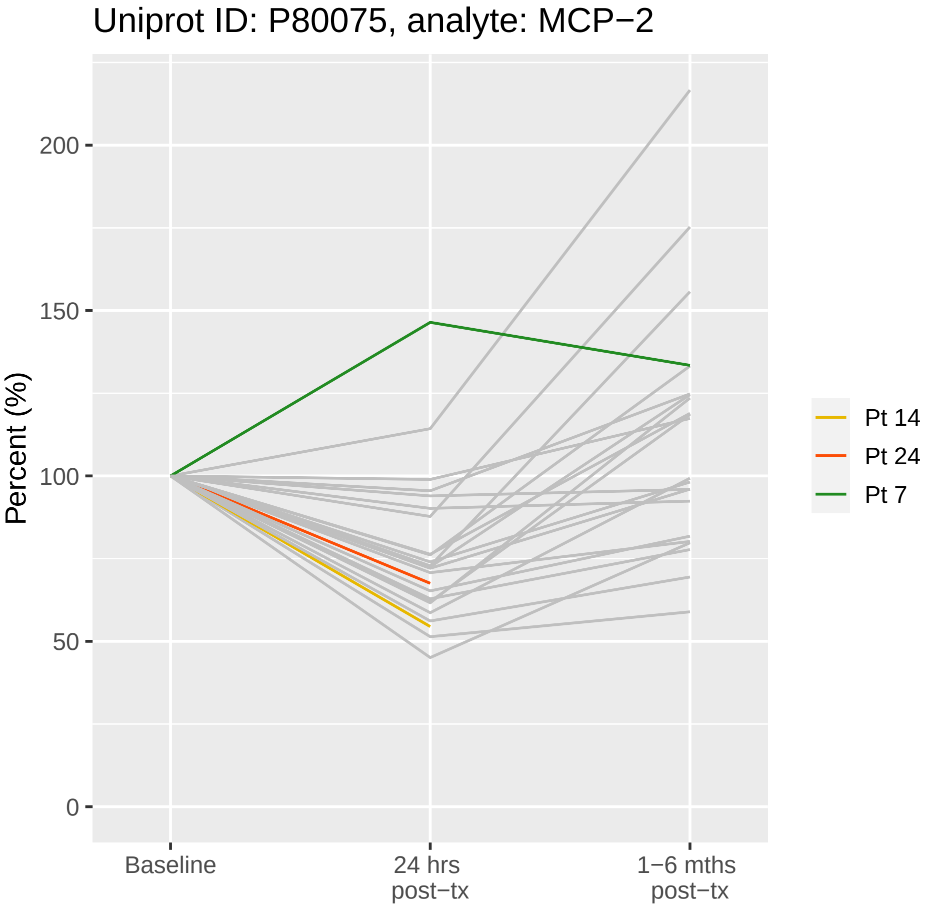

Uniprot ID: P80075, analyte: MCP−2
Percent (%)
200
150
100
50
0
Baseline
24 hrs post−tx
1−6 mths post−tx
Pt 14
Pt 24
Pt 7

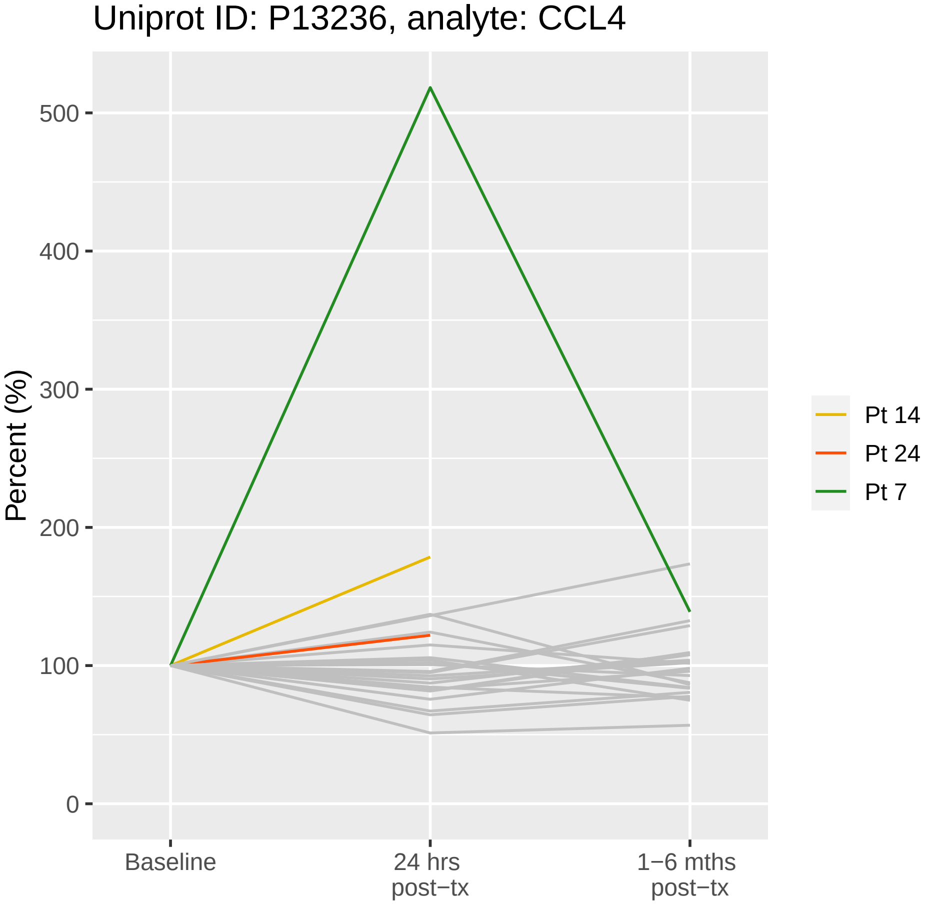

Uniprot ID: P13236, analyte: CCL4
Percent (%)
500
400
300
200
100
0
Baseline
24 hrs post−tx
1−6 mths post−tx
Pt 14
Pt 24
Pt 7

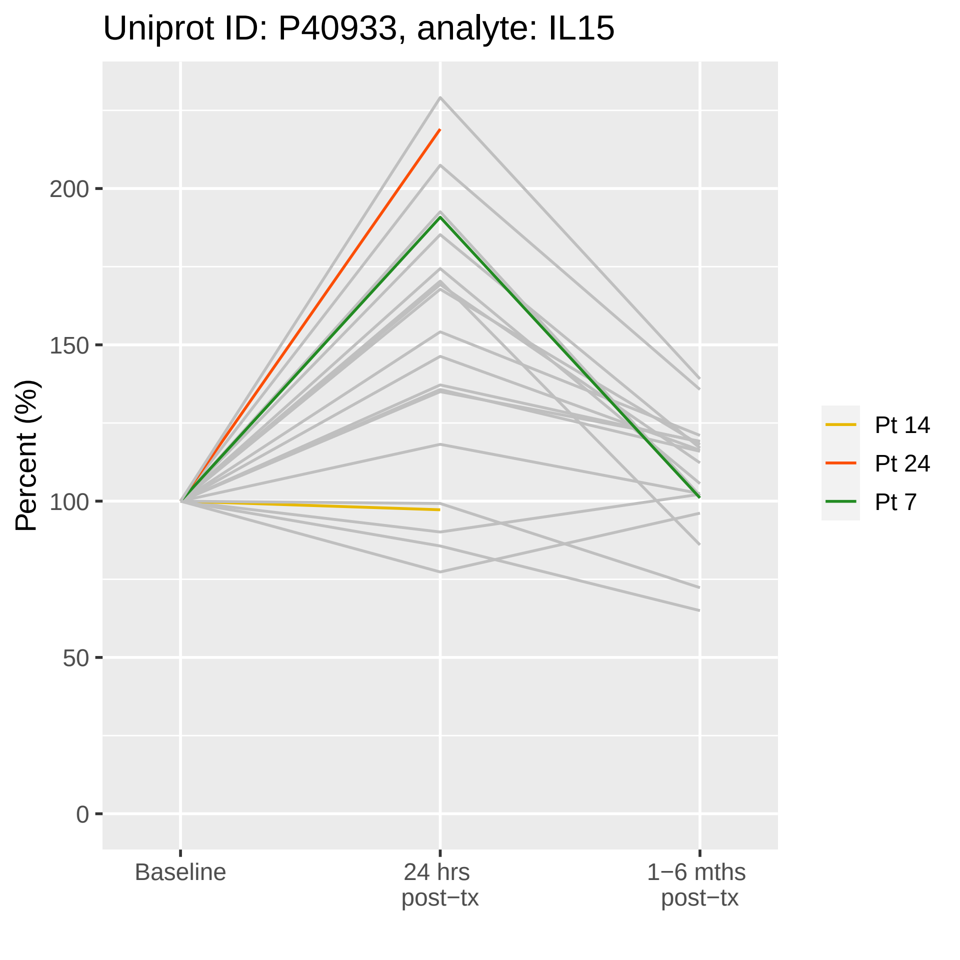
Uniprot ID: P40933, analyte: IL15
Percent (%)
200
150
100
50
0
Baseline
24 hrs
post−tx
1−6 mths
post−tx
Pt 14
Pt 24
Pt 7

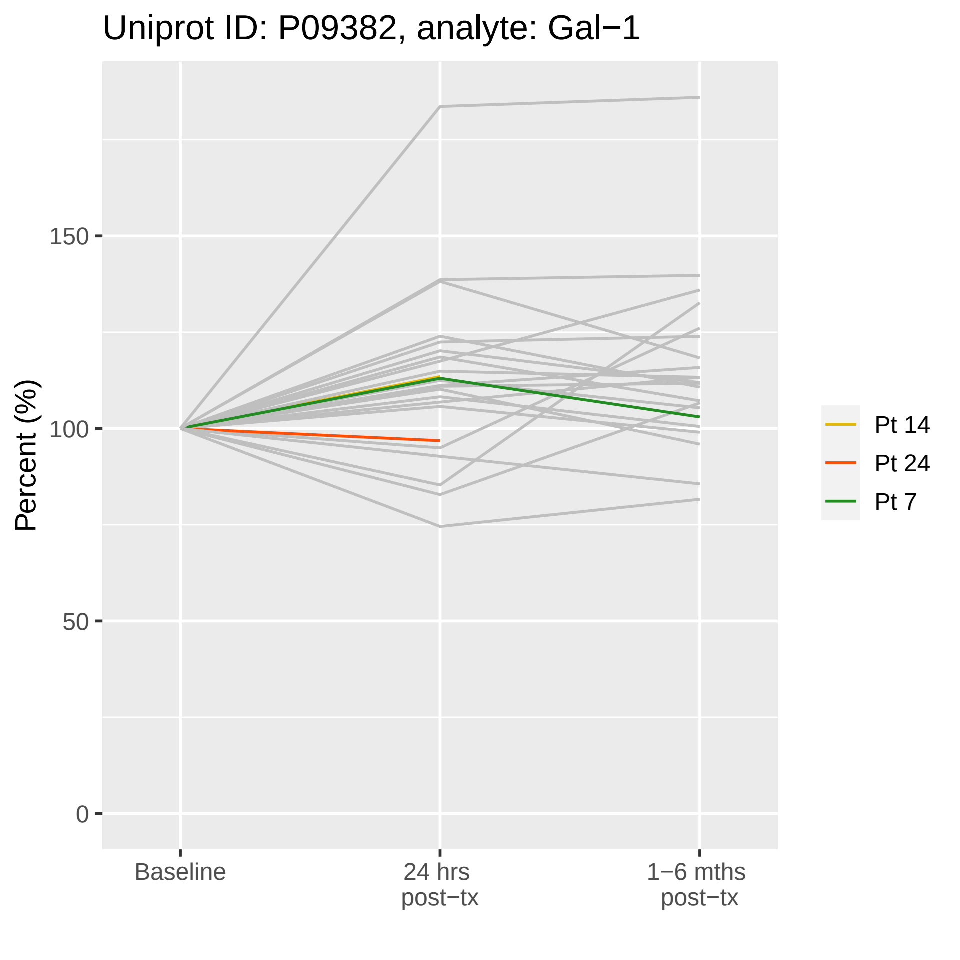
Uniprot ID: P09382, analyte: Gal−1
Percent (%)
150
100
50
0
Baseline
24 hrs
post−tx
1−6 mths
post−tx
Pt 14
Pt 24
Pt 7

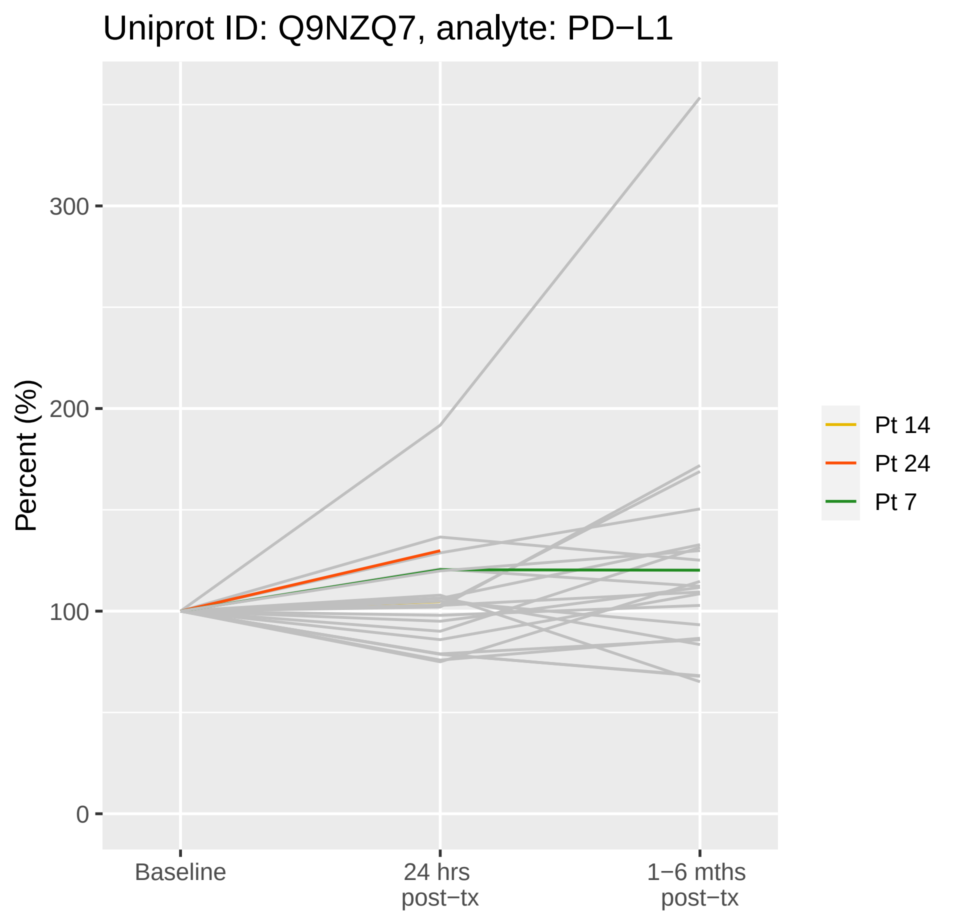
Uniprot ID: Q9NZQ7, analyte: PD−L1
Percent (%)
300
200
100
0
Baseline
24 hrs
post−tx
1−6 mths
post−tx
Pt 14
Pt 24
Pt 7

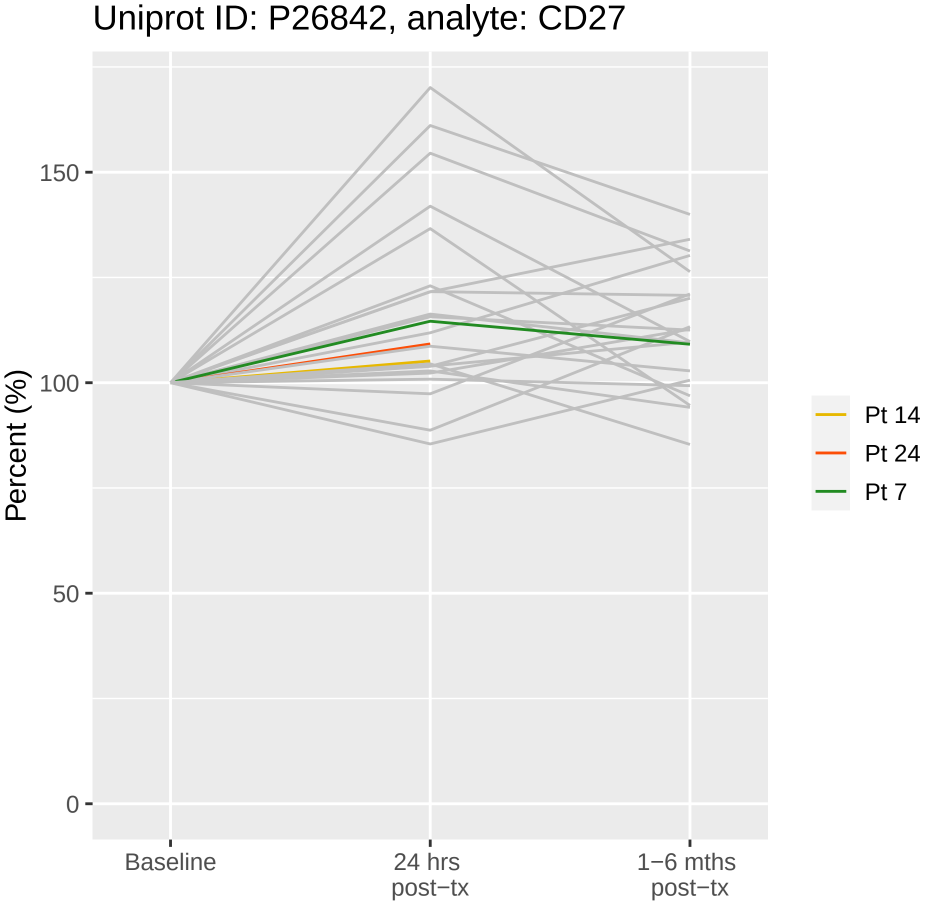

Uniprot ID: P26842, analyte: CD27
Percent (%)
150
100
50
0
Baseline
24 hrs
post−tx
1−6 mths
post−tx
Pt 14
Pt 24
Pt 7

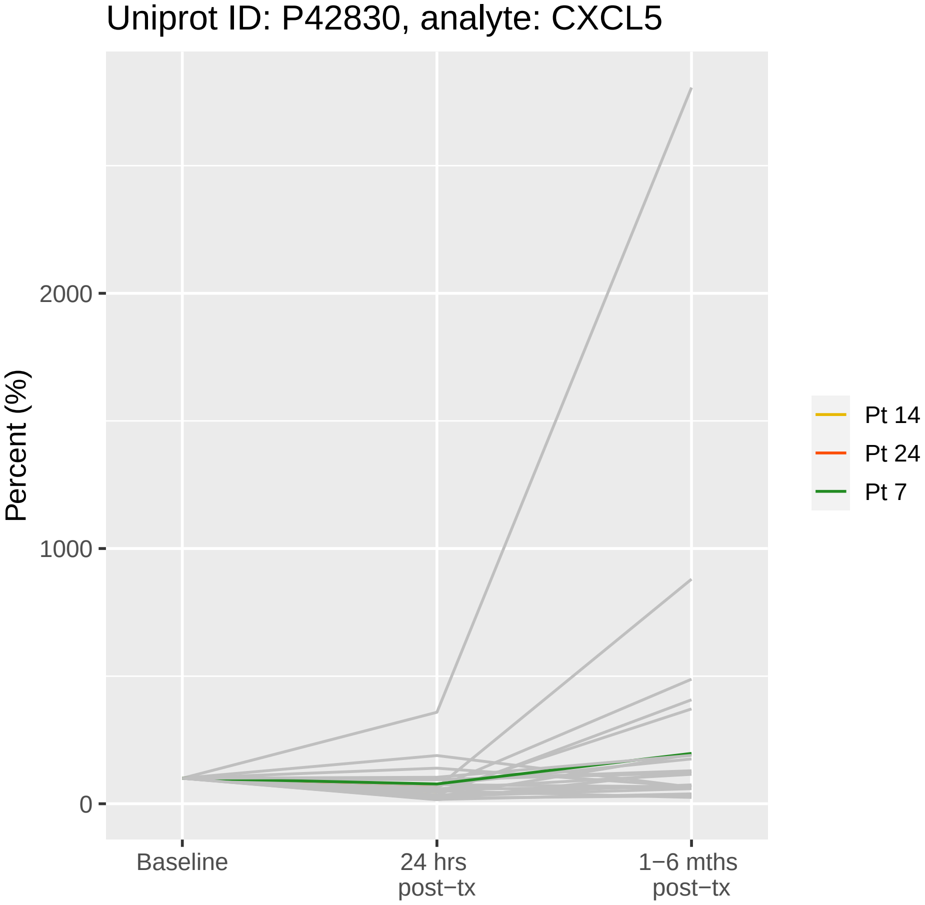
Uniprot ID: P42830, analyte: CXCL5
Percent (%)
2000
1000
0
Baseline
24 hrs post−tx
1−6 mths post−tx
Pt 14
Pt 24
Pt 7

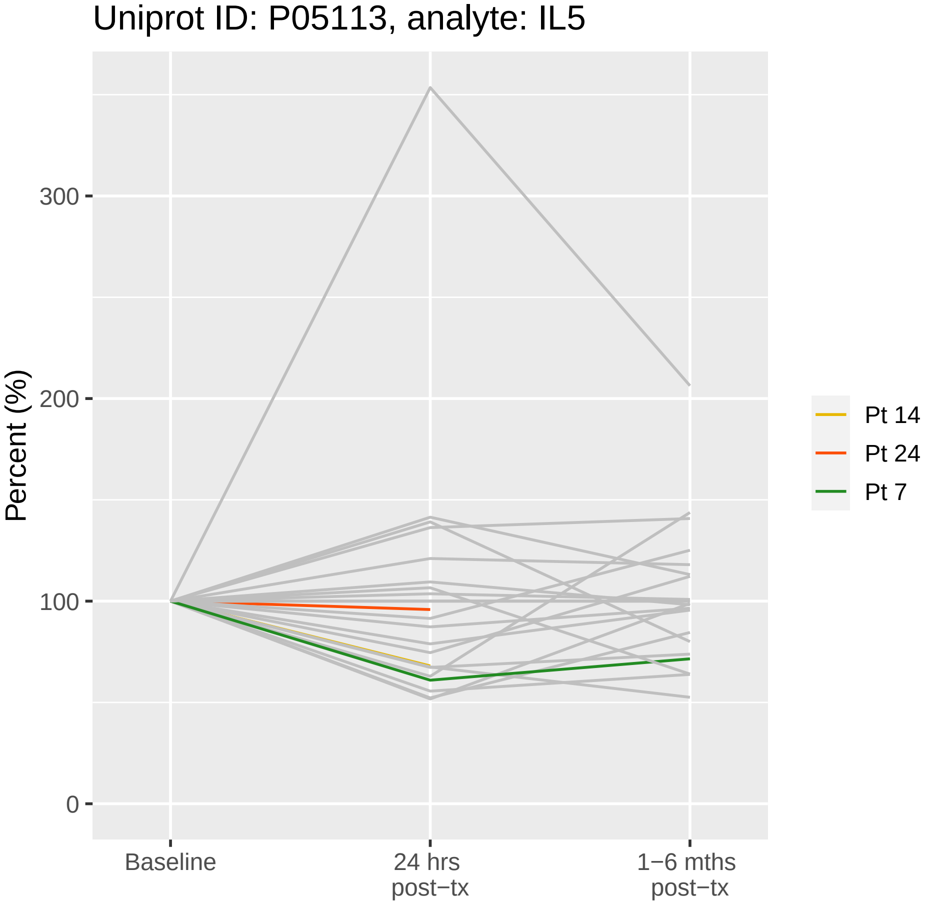
Uniprot ID: P05113, analyte: IL5
Percent (%)
300
200
100
0
Baseline
24 hrs post−tx
1−6 mths post−tx
Pt 14
Pt 24
Pt 7

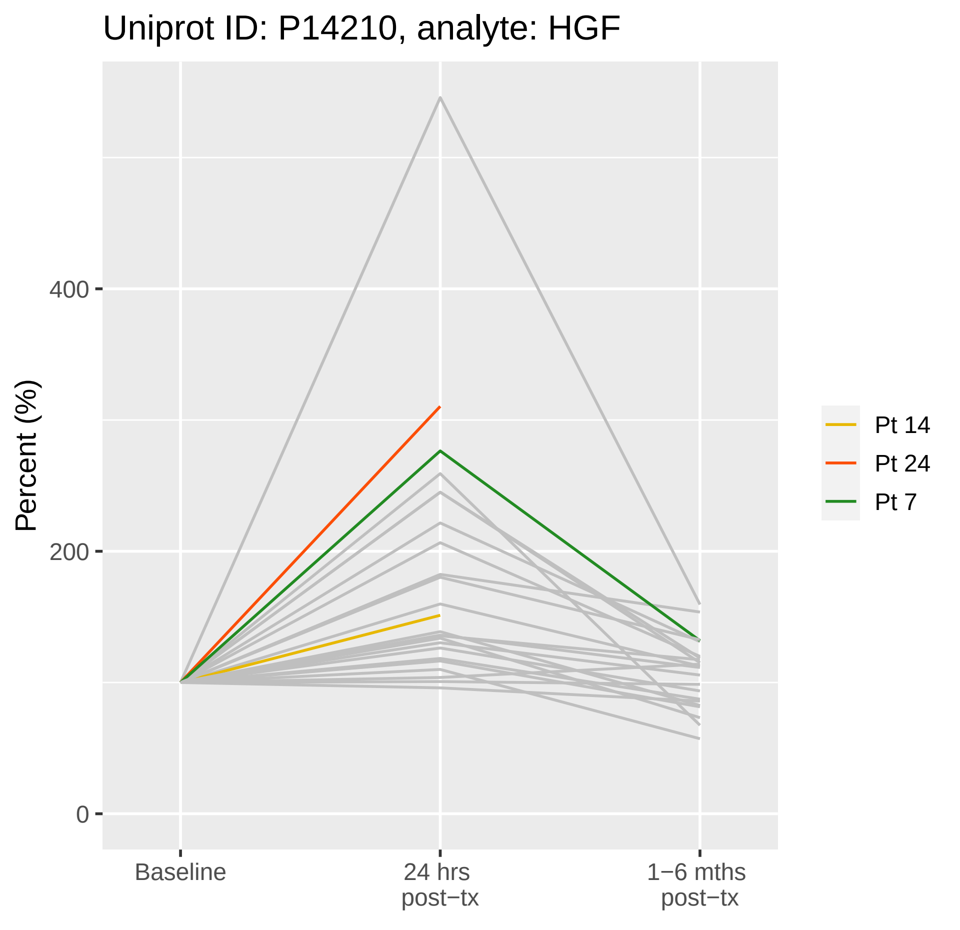

Uniprot ID: P14210, analyte: HGF
Percent (%)
400
200
0
Baseline
24 hrs post−tx
1−6 mths post−tx
Pt 14
Pt 24
Pt 7

Uniprot ID: P12544, analyte: GZMA

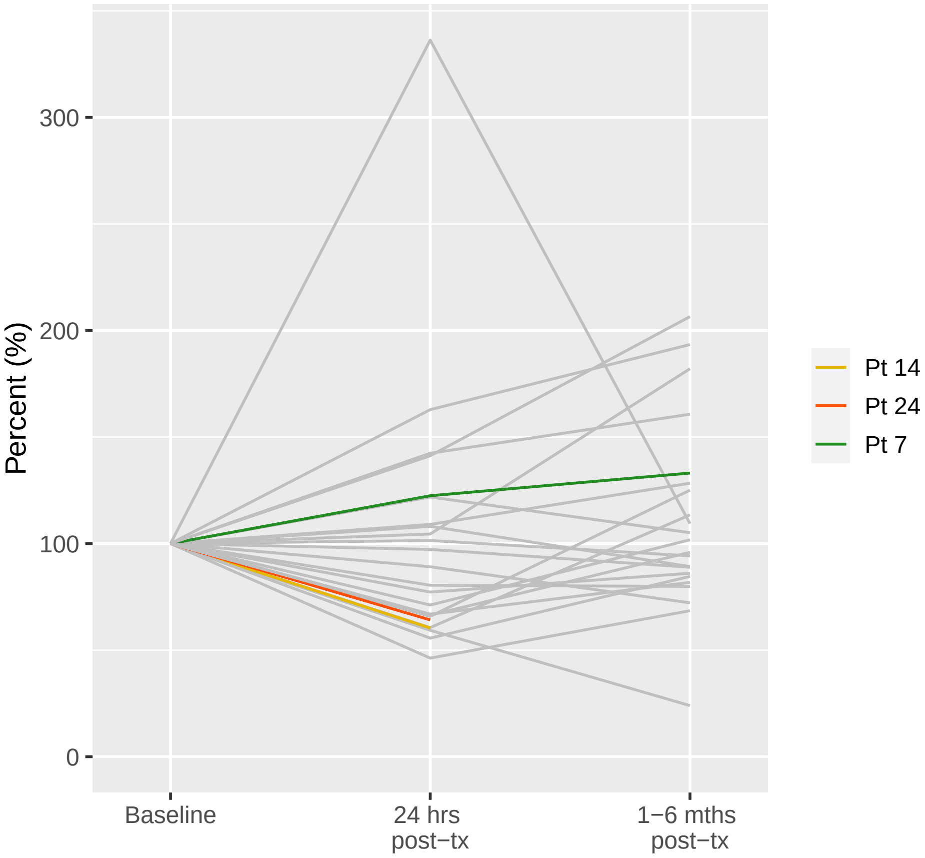

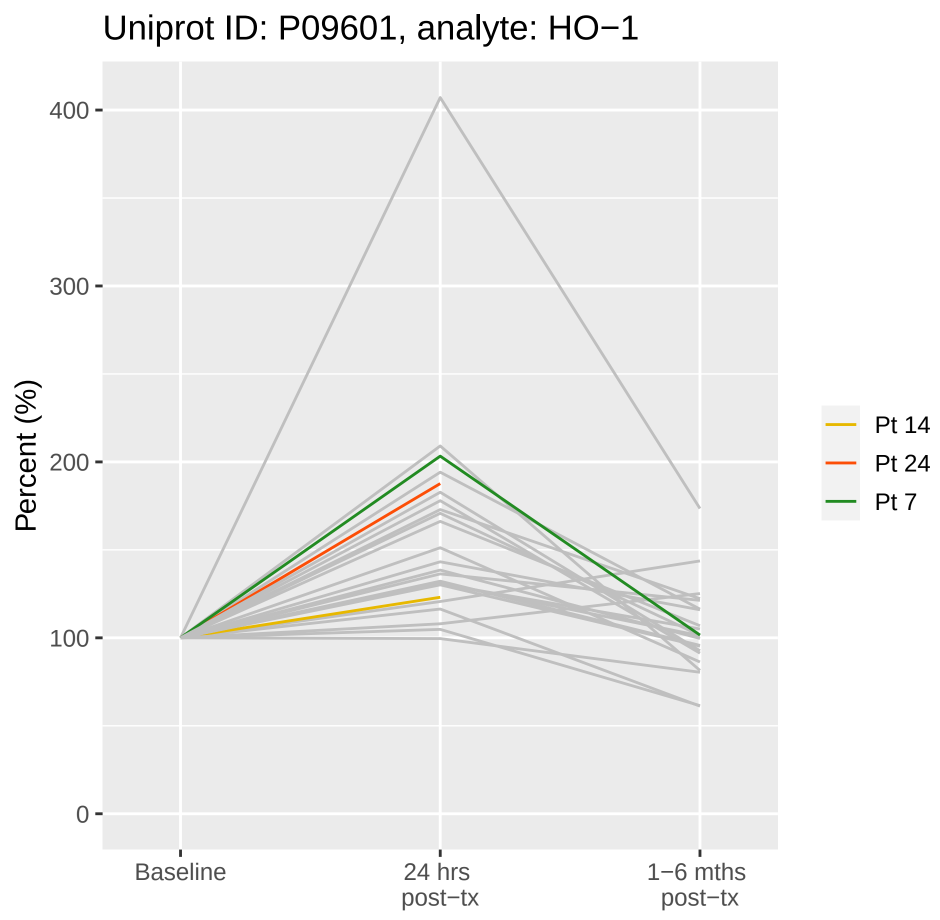

Uniprot ID: P09601, analyte: HO−1
Percent (%)
400
300
200
100
0
Baseline
24 hrs post−tx
1−6 mths post−tx
Pt 14
Pt 24
Pt 7

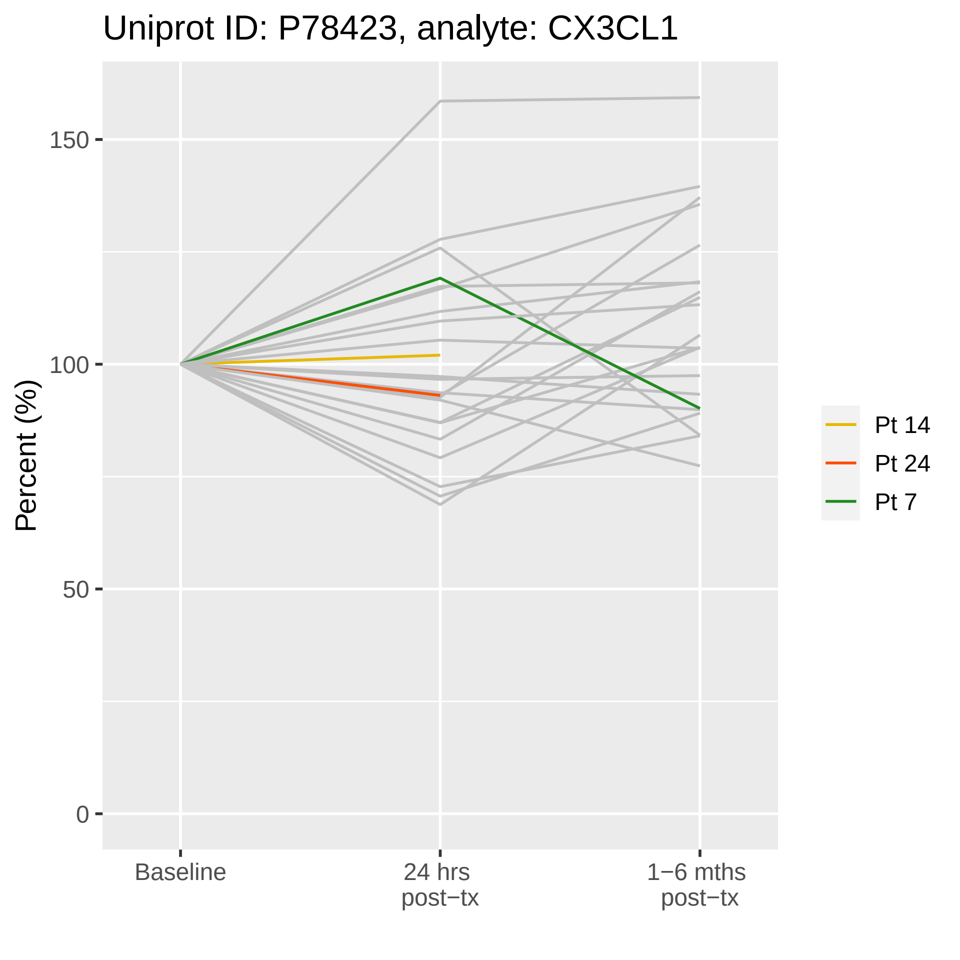

Uniprot ID: P78423, analyte: CX3CL1
Percent (%)
150
100
50
0
Baseline
24 hrs post−tx
1−6 mths post−tx
Pt 14
Pt 24
Pt 7

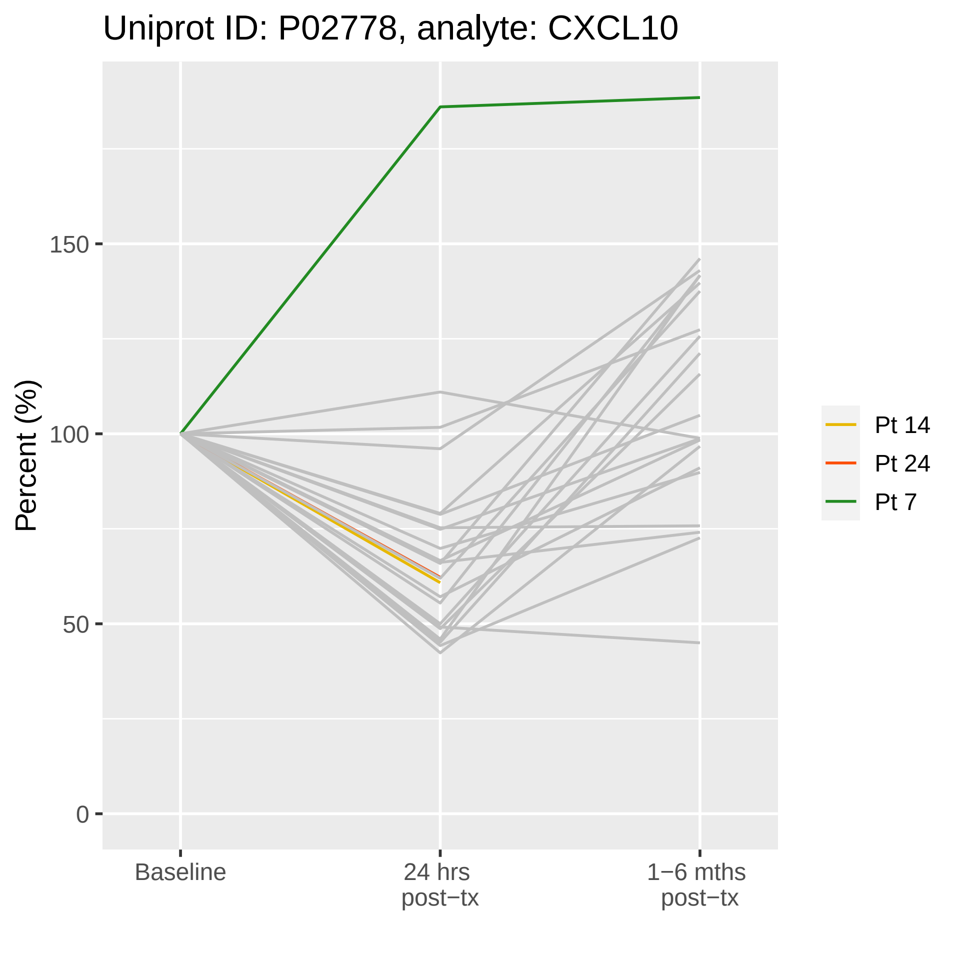
Uniprot ID: P02778, analyte: CXCL10
Percent (%)
150
100
50
0
Baseline
24 hrs
post−tx
1−6 mths
post−tx
Pt 14
Pt 24
Pt 7

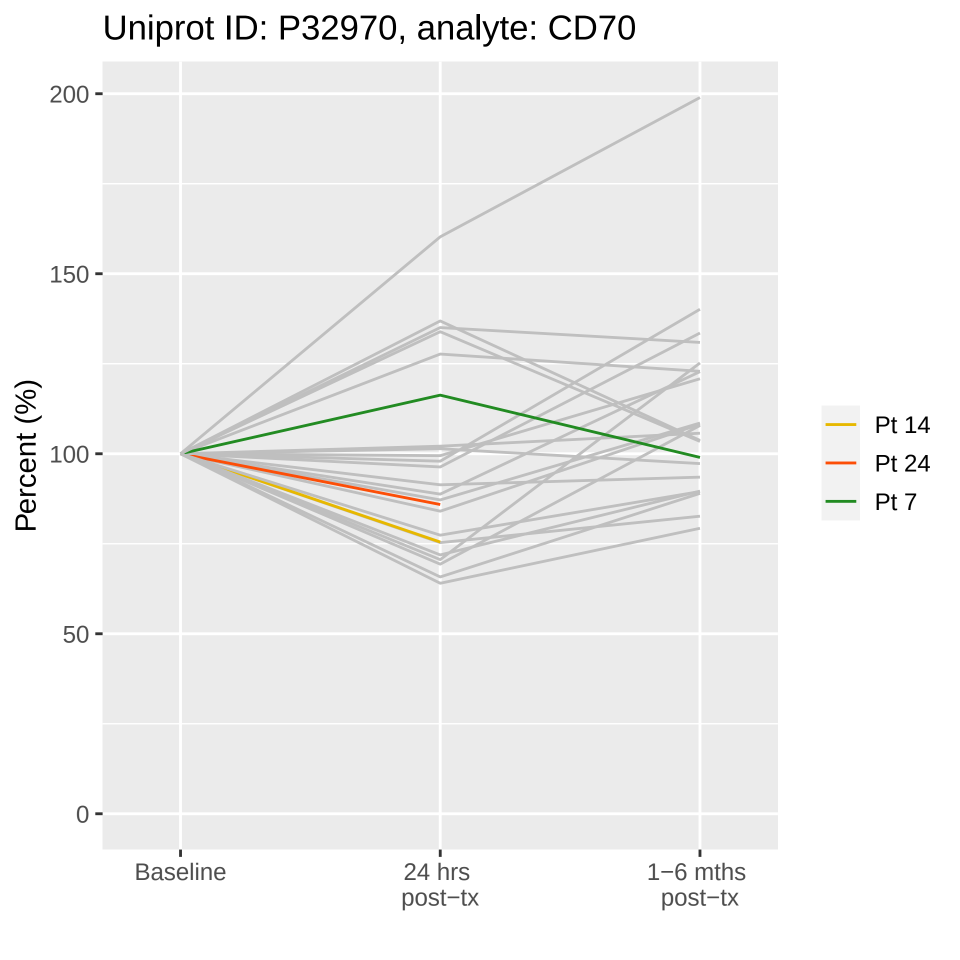

Uniprot ID: P32970, analyte: CD70
Percent (%)
200
150
100
50
0
Baseline
24 hrs post−tx
1−6 mths post−tx
Pt 14
Pt 24
Pt 7

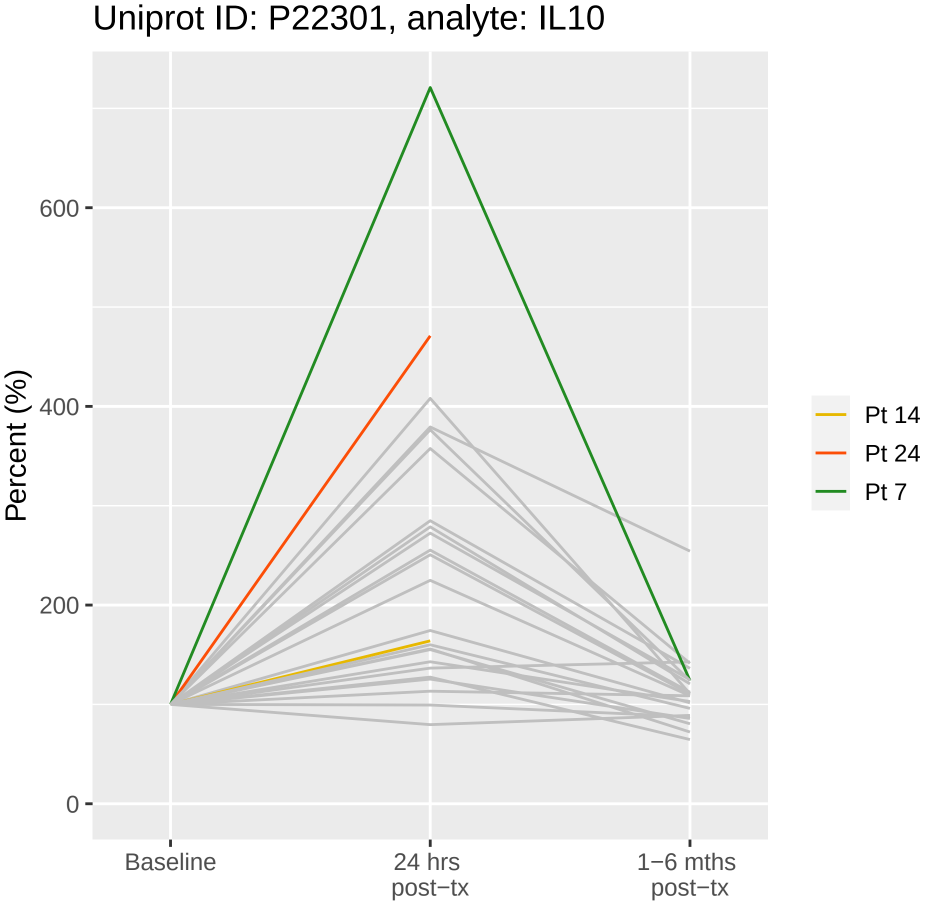
Uniprot ID: P22301, analyte: IL10
Percent (%)
600
400
200
0
Baseline
24 hrs post−tx
1−6 mths post−tx
Pt 14
Pt 24
Pt 7

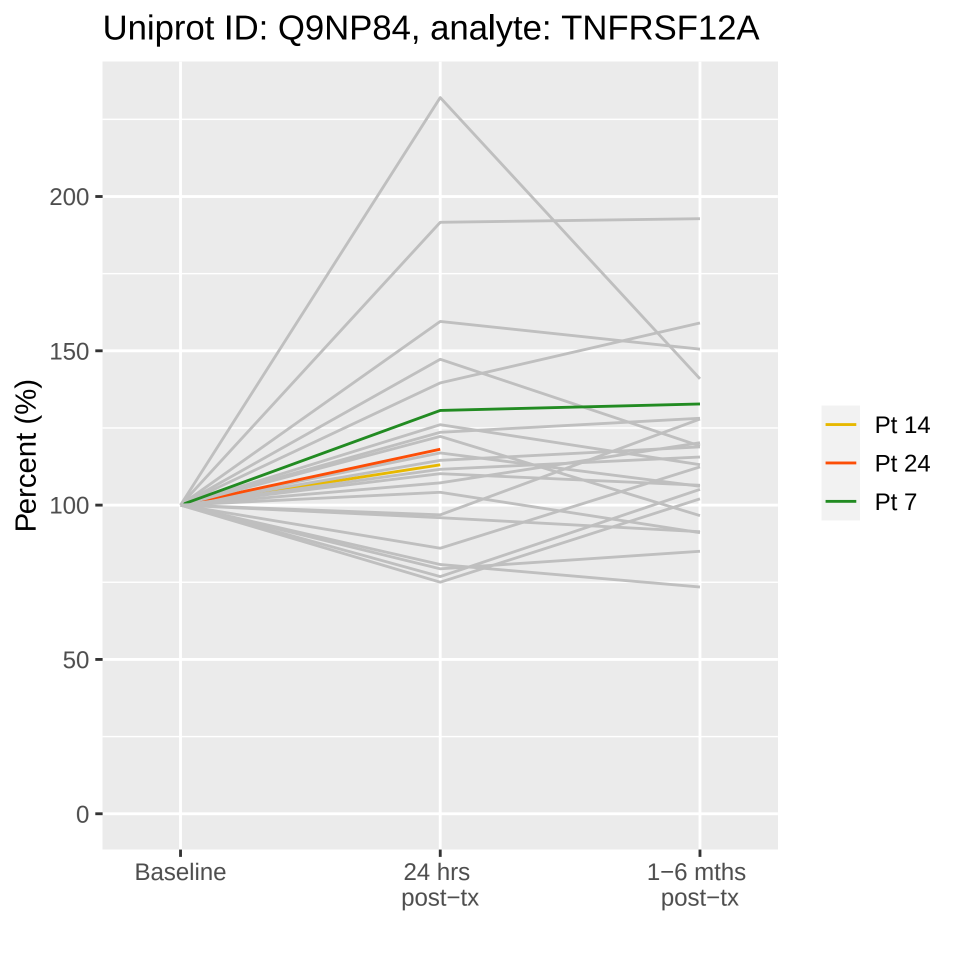
Uniprot ID: Q9NP84, analyte: TNFRSF12A
Percent (%)
200
150
100
50
0
Baseline
24 hrs post−tx
1−6 mths post−tx
Pt 14
Pt 24
Pt 7

Uniprot ID: P55773, analyte: CCL23

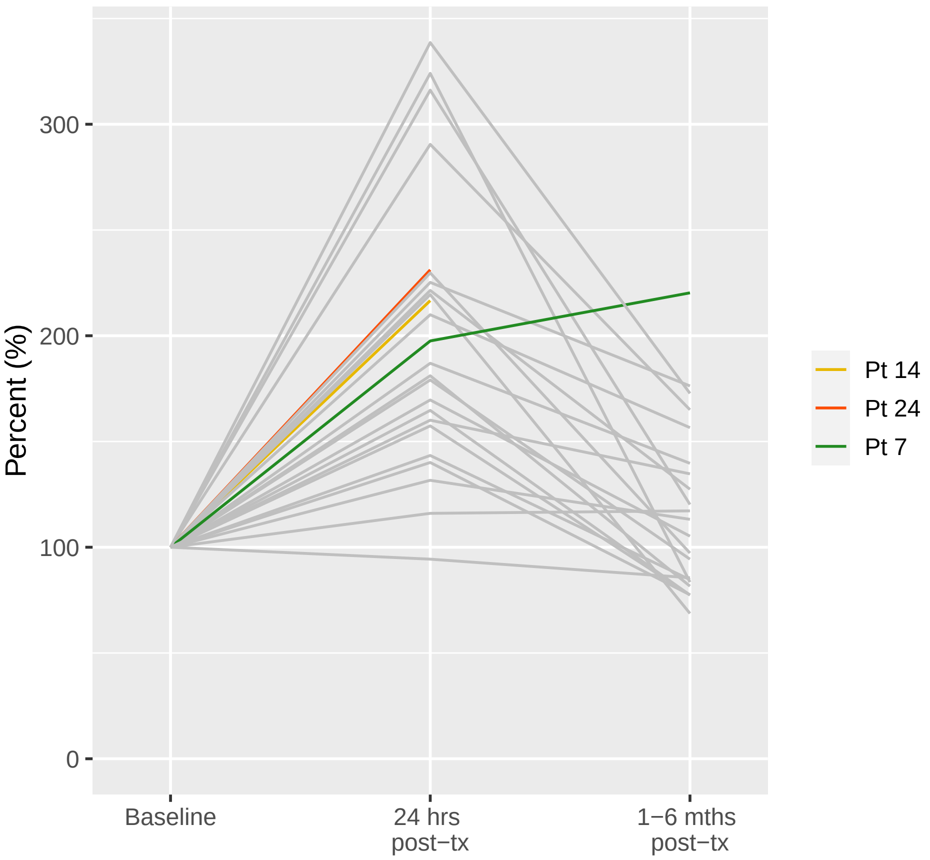

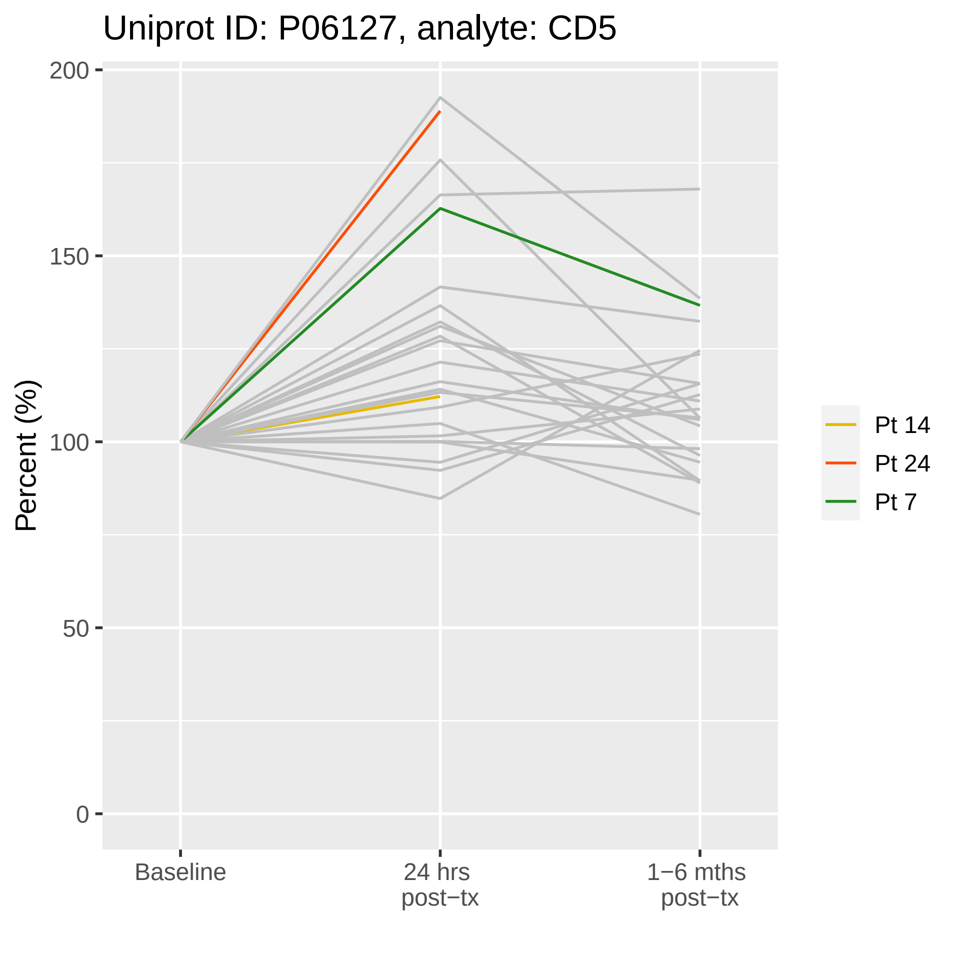

Uniprot ID: P06127, analyte: CD5
Percent (%)
200
150
100
50
0
Baseline
24 hrs post−tx
1−6 mths post−tx
Pt 14
Pt 24
Pt 7

Uniprot ID: P10147, analyte: CCL3

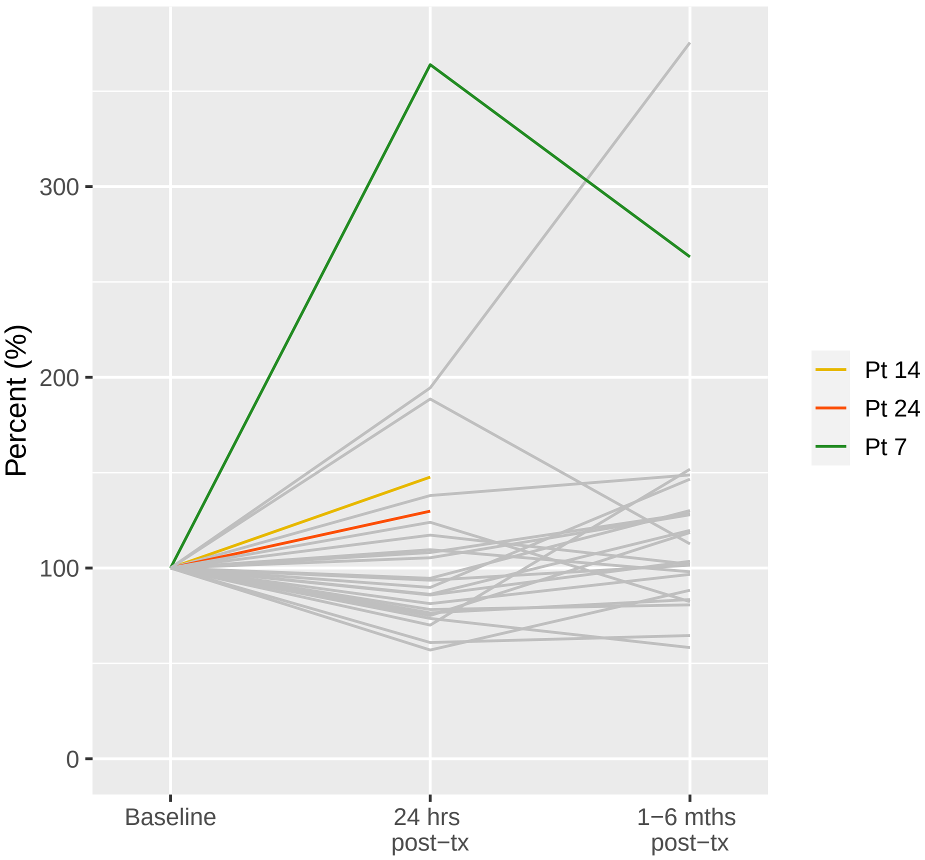

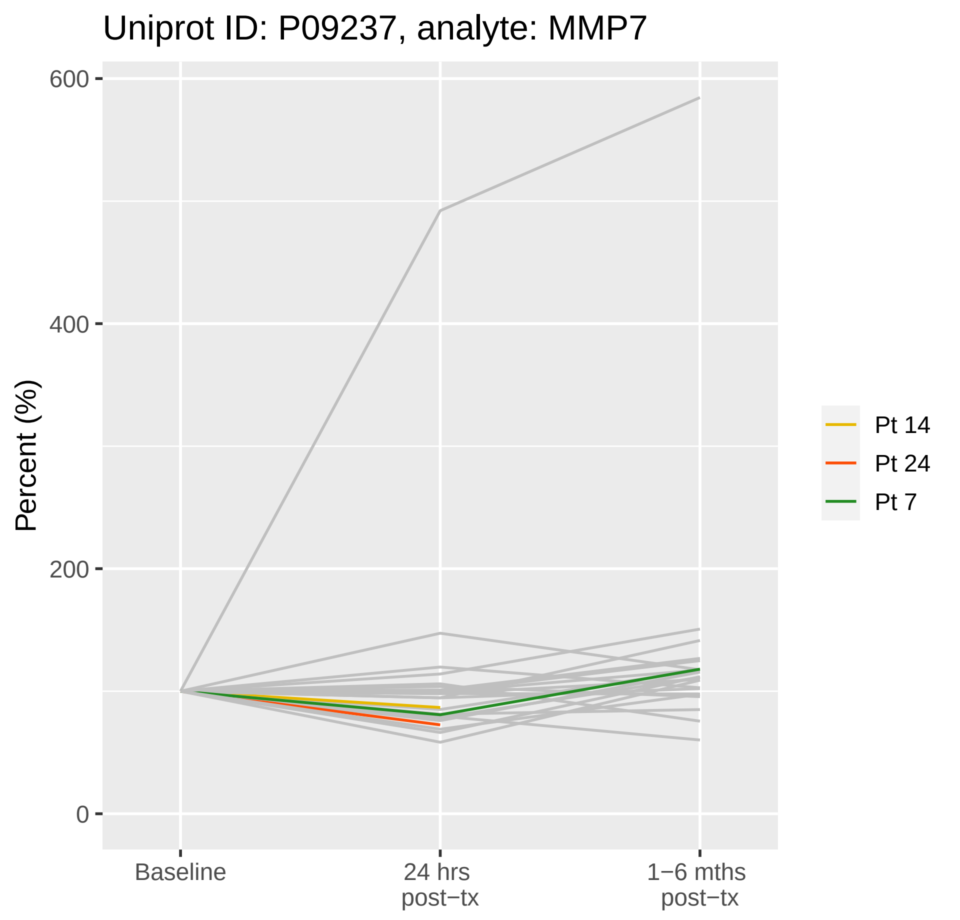

Uniprot ID: P09237, analyte: MMP7
600
400
200
0
Percent (%)
Baseline
24 hrs
post−tx
1−6 mths
post−tx
Pt 14
Pt 24
Pt 7

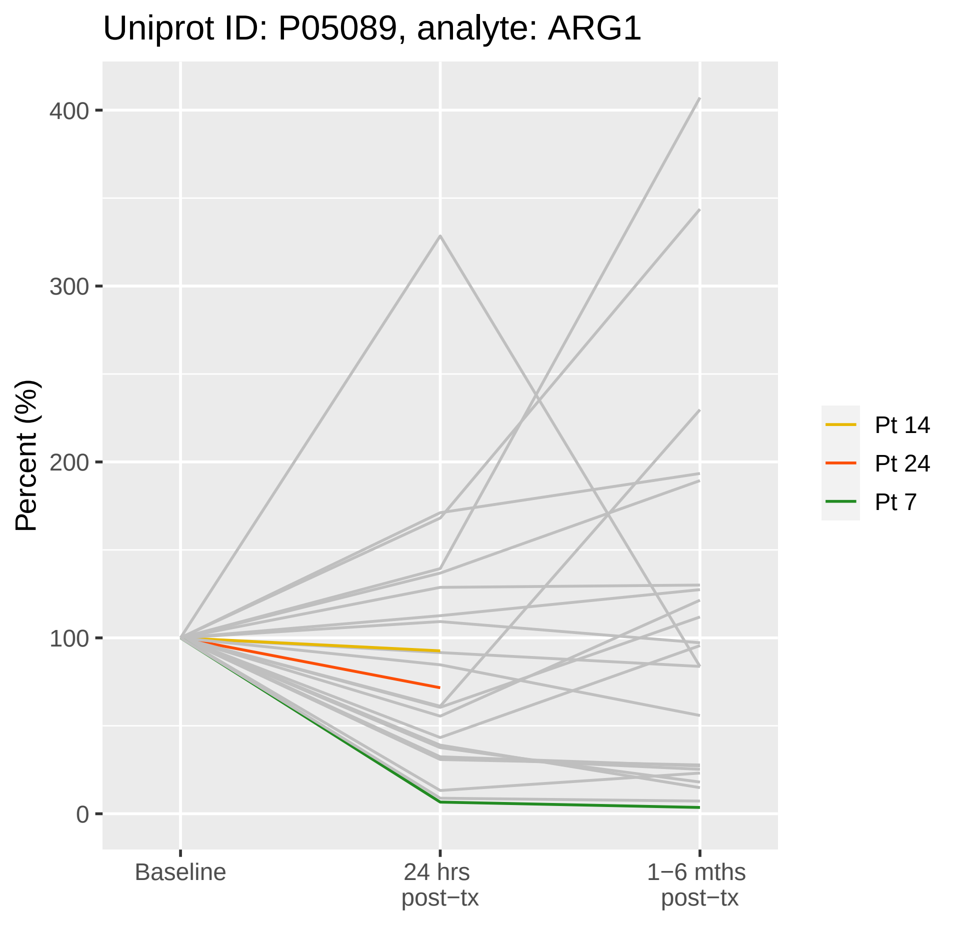
Uniprot ID: P05089, analyte: ARG1
Percent (%)
400
300
200
100
0
Baseline
24 hrs post−tx
1−6 mths post−tx
Pt 14
Pt 24
Pt 7

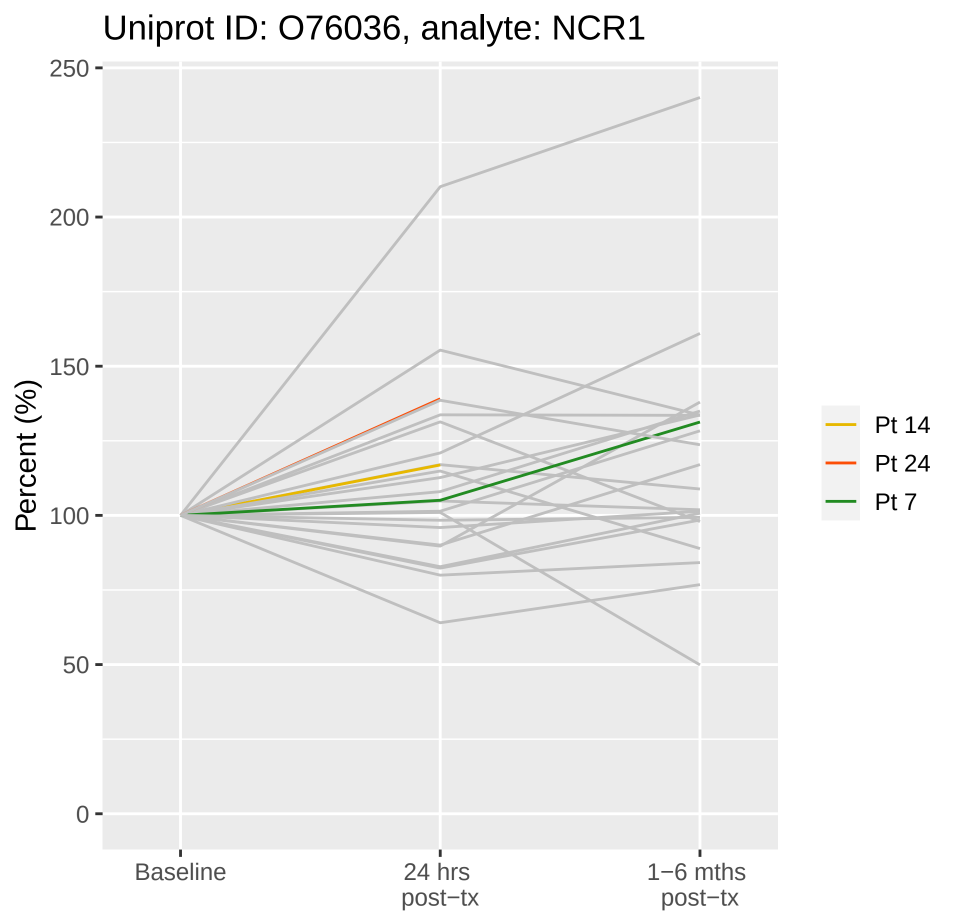
Uniprot ID: O76036, analyte: NCR1
Percent (%)
250
200
150
100
50
0
Baseline
24 hrs post−tx
1−6 mths post−tx
Pt 14
Pt 24
Pt 7

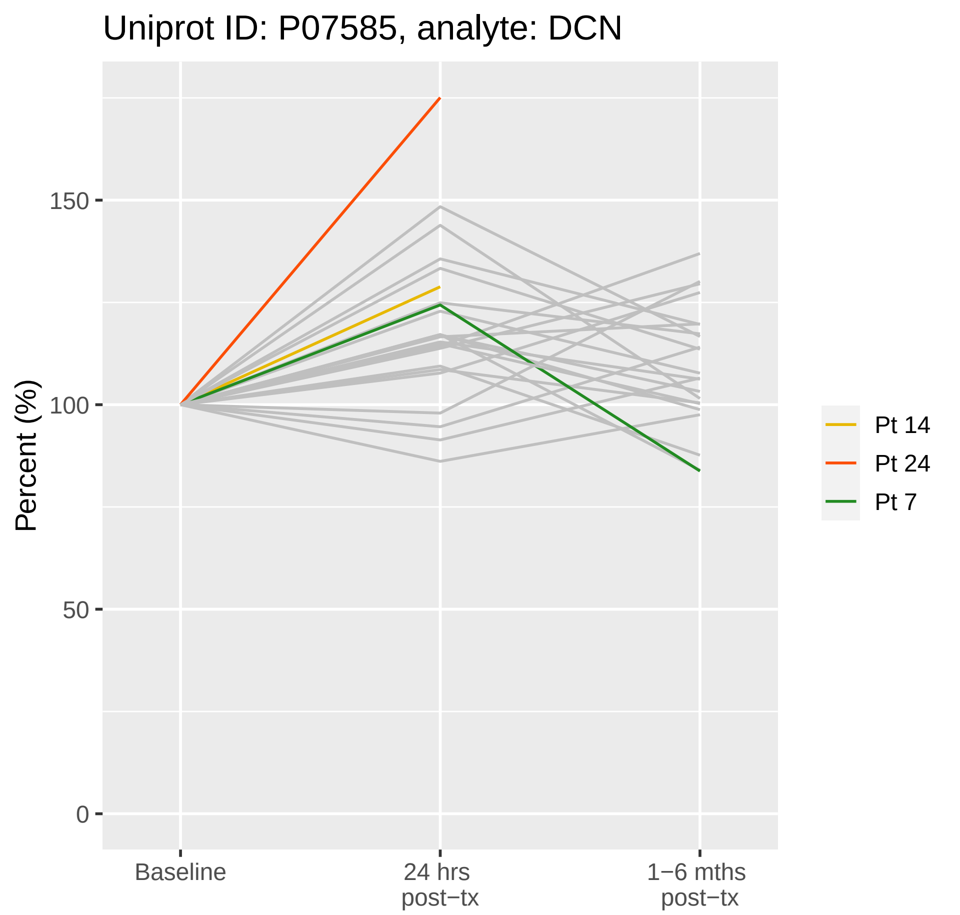

Uniprot ID: P07585, analyte: DCN
Percent (%)
150
100
50
0
Baseline
24 hrs post−tx
1−6 mths post−tx
Pt 14
Pt 24
Pt 7

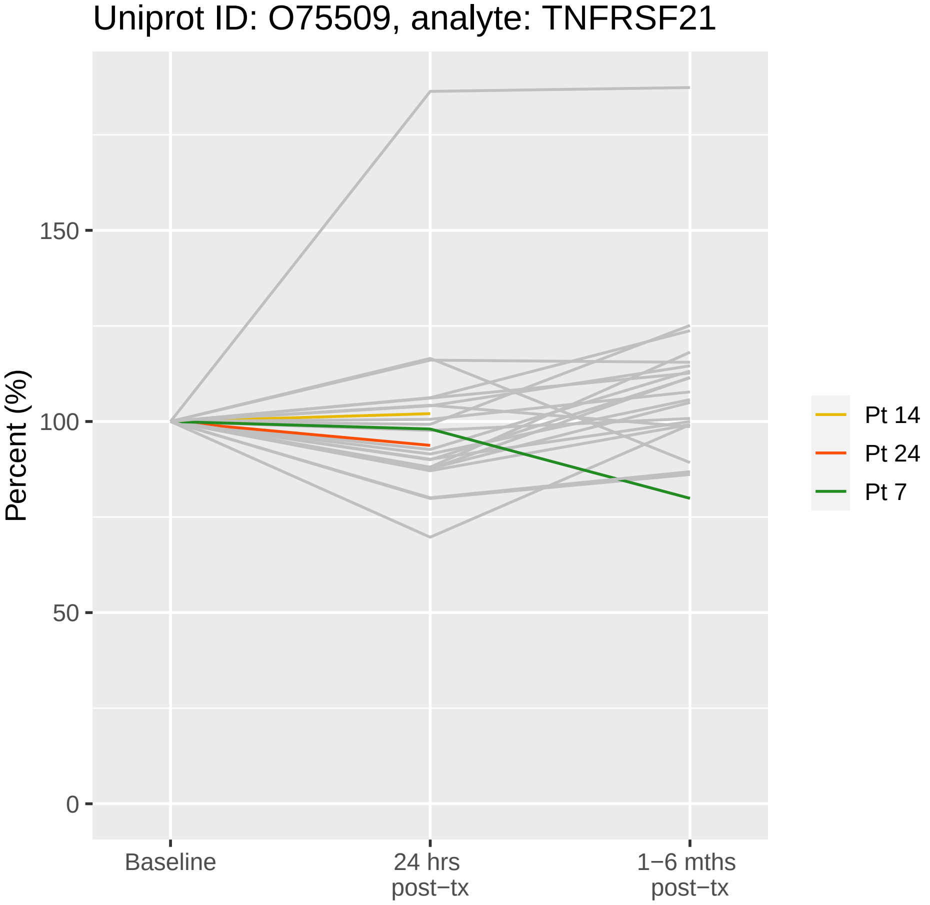

Uniprot ID: O75509, analyte: TNFRSF21
Percent (%)
150
100
50
0
Baseline
24 hrs post−tx
1−6 mths post−tx
Pt 14
Pt 24
Pt 7

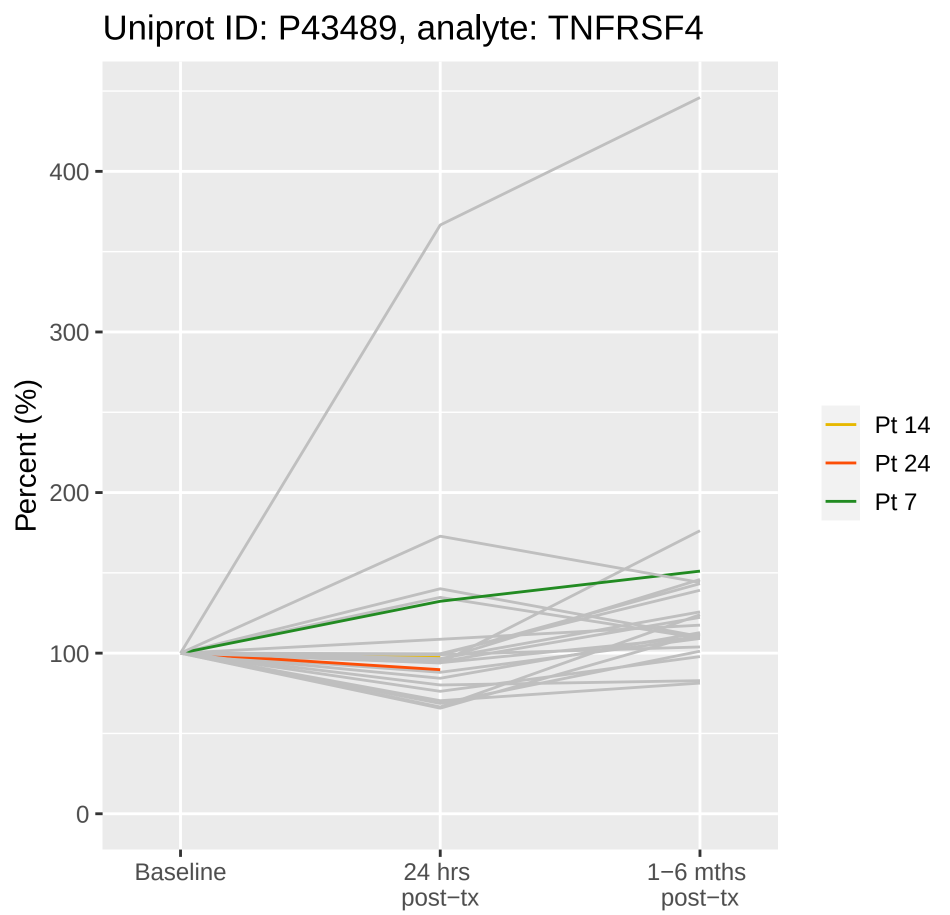
Uniprot ID: P43489, analyte: TNFRSF4
Percent (%)
400
300
200
100
0
Baseline
24 hrs
post−tx
1−6 mths
post−tx
Pt 14
Pt 24
Pt 7

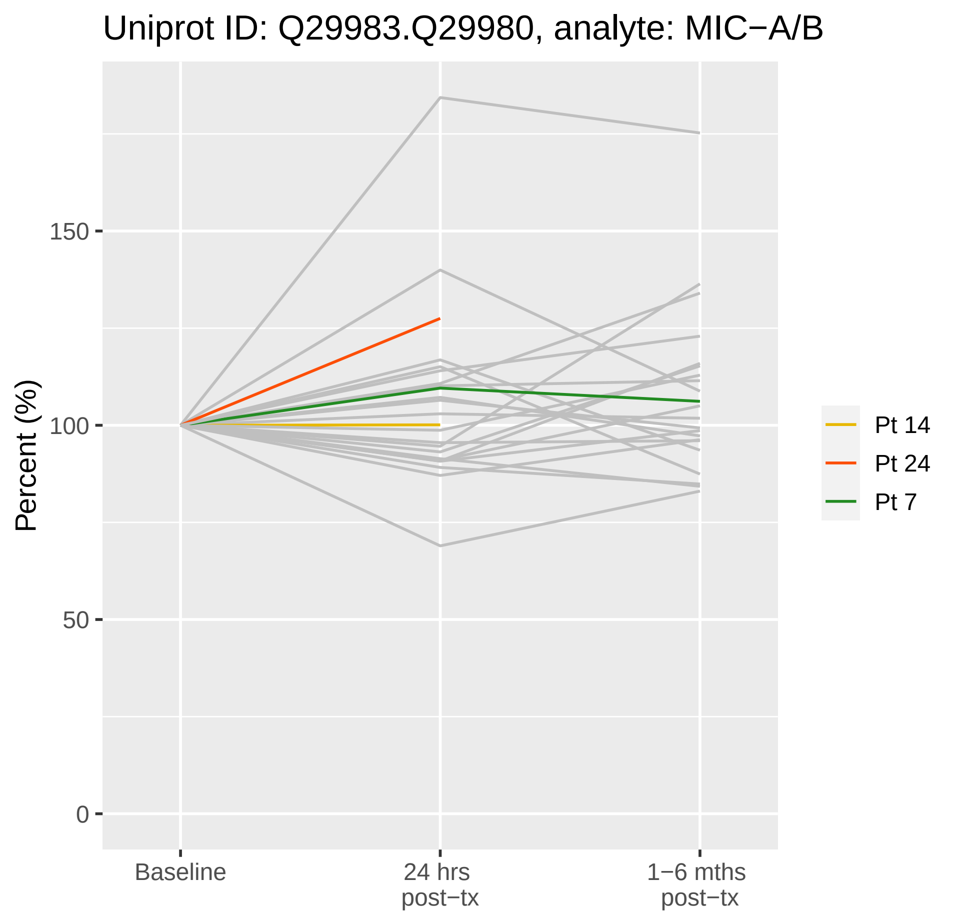

Uniprot ID: Q29983.Q29980, analyte: MIC−A/B
Percent (%)
150
100
50
0
Baseline
24 hrs
post−tx
1−6 mths
post−tx
Pt 14
Pt 24
Pt 7

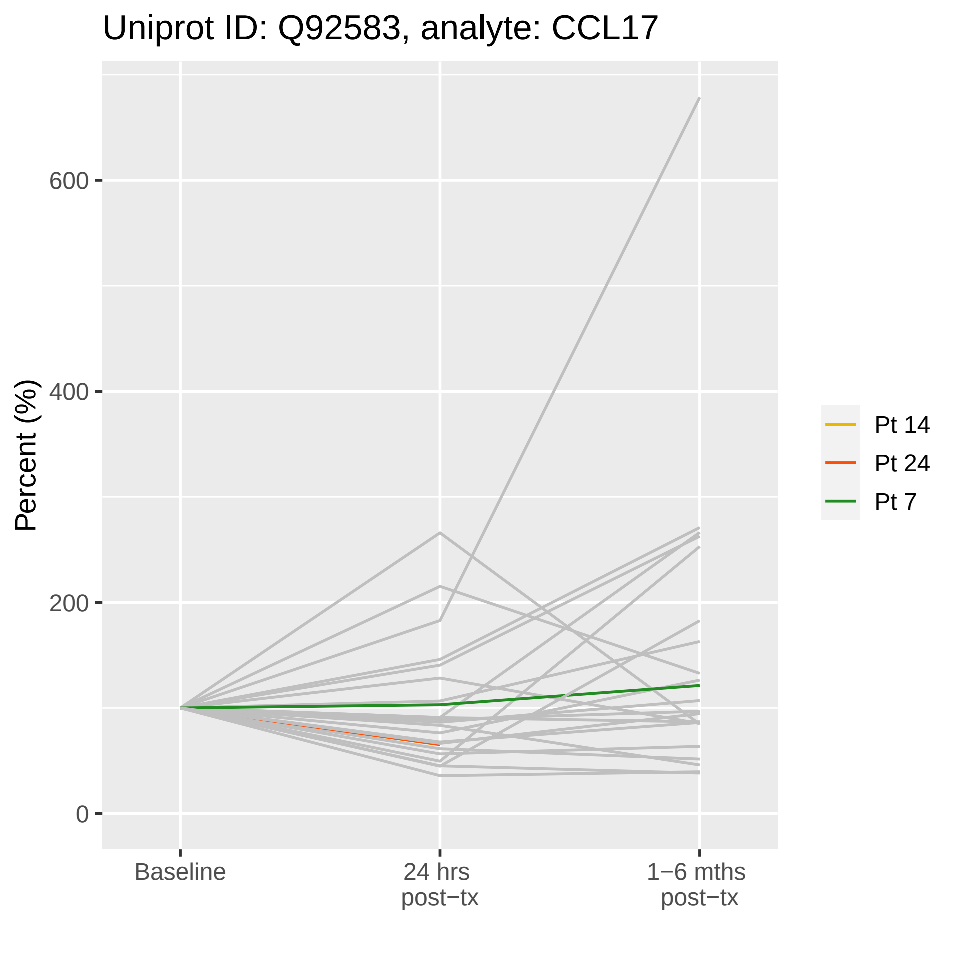
Uniprot ID: Q92583, analyte: CCL17
Percent (%)
0
200
400
600
Baseline
24 hrs post−tx
1−6 mths post−tx
Pt 14
Pt 24
Pt 7

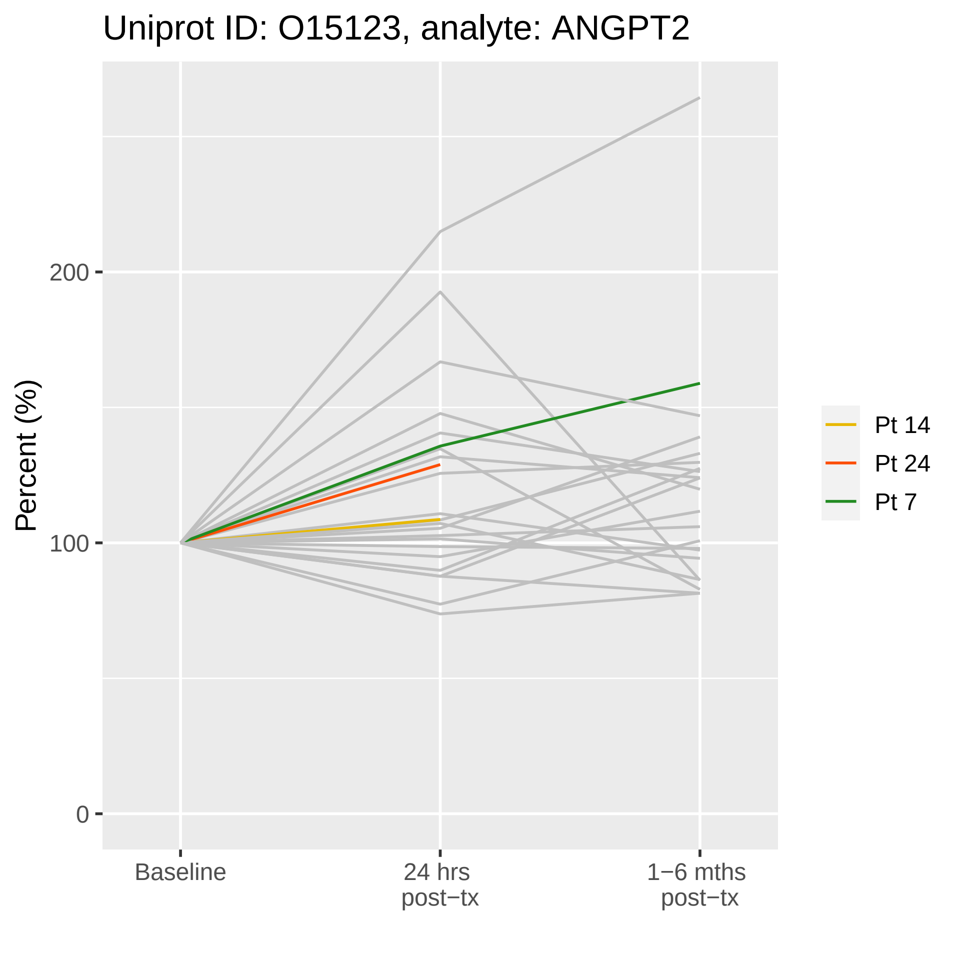
Uniprot ID: O15123, analyte: ANGPT2
Percent (%)
200
100
0
Baseline
24 hrs post−tx
1−6 mths post−tx
Pt 14
Pt 24
Pt 7

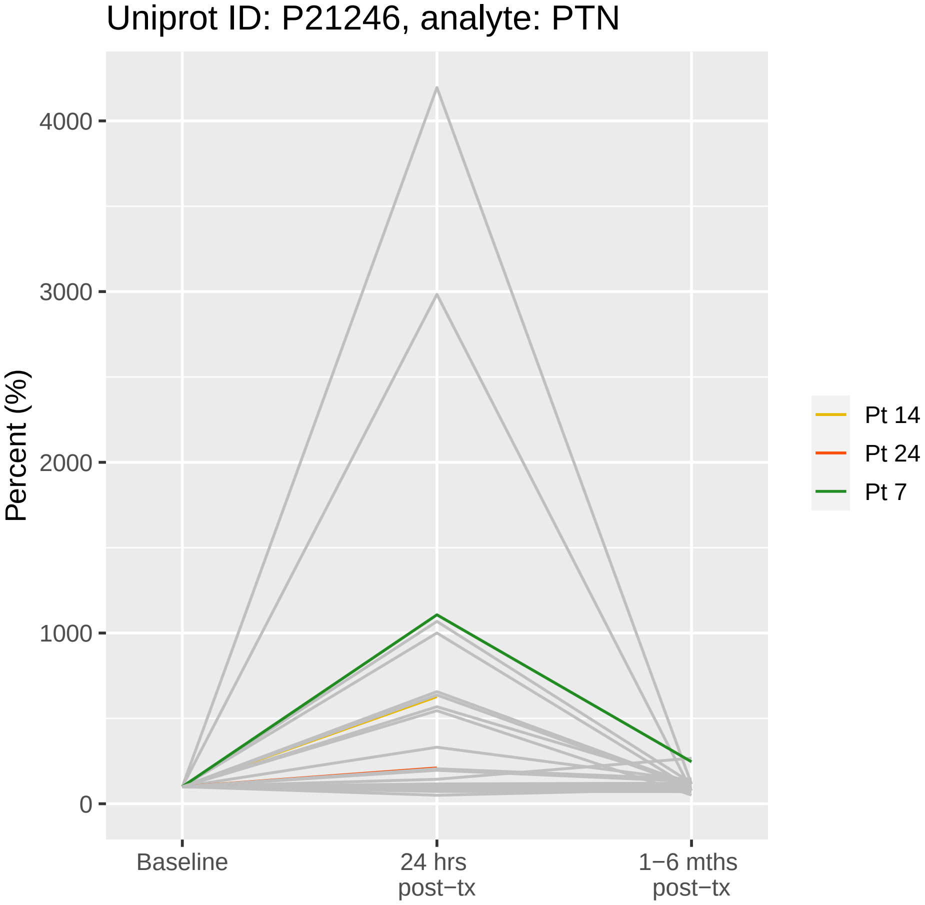

Uniprot ID: P21246, analyte: PTN
Percent (%)
4000
3000
2000
1000
0
Baseline
24 hrs post−tx
1−6 mths post−tx
Pt 14
Pt 24
Pt 7

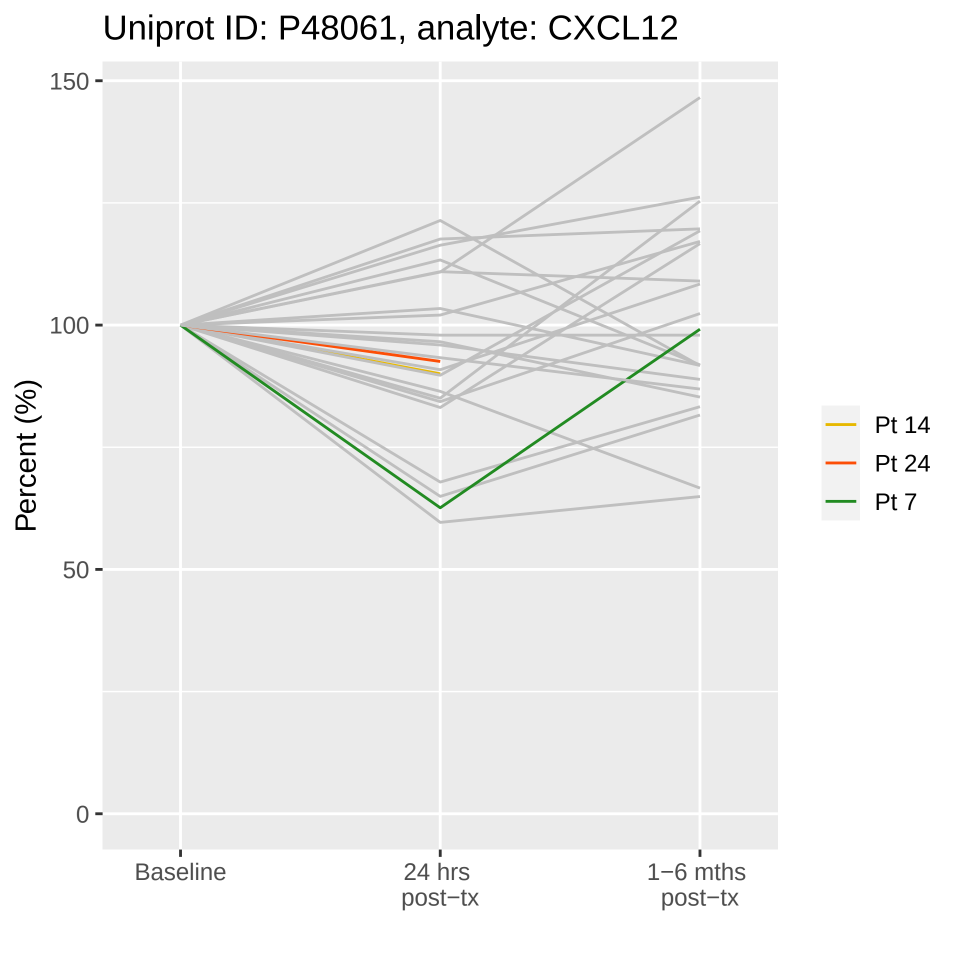

Uniprot ID: P48061, analyte: CXCL12
Percent (%)
150
100
50
0
Baseline
24 hrs post−tx
1−6 mths post−tx
Pt 14
Pt 24
Pt 7

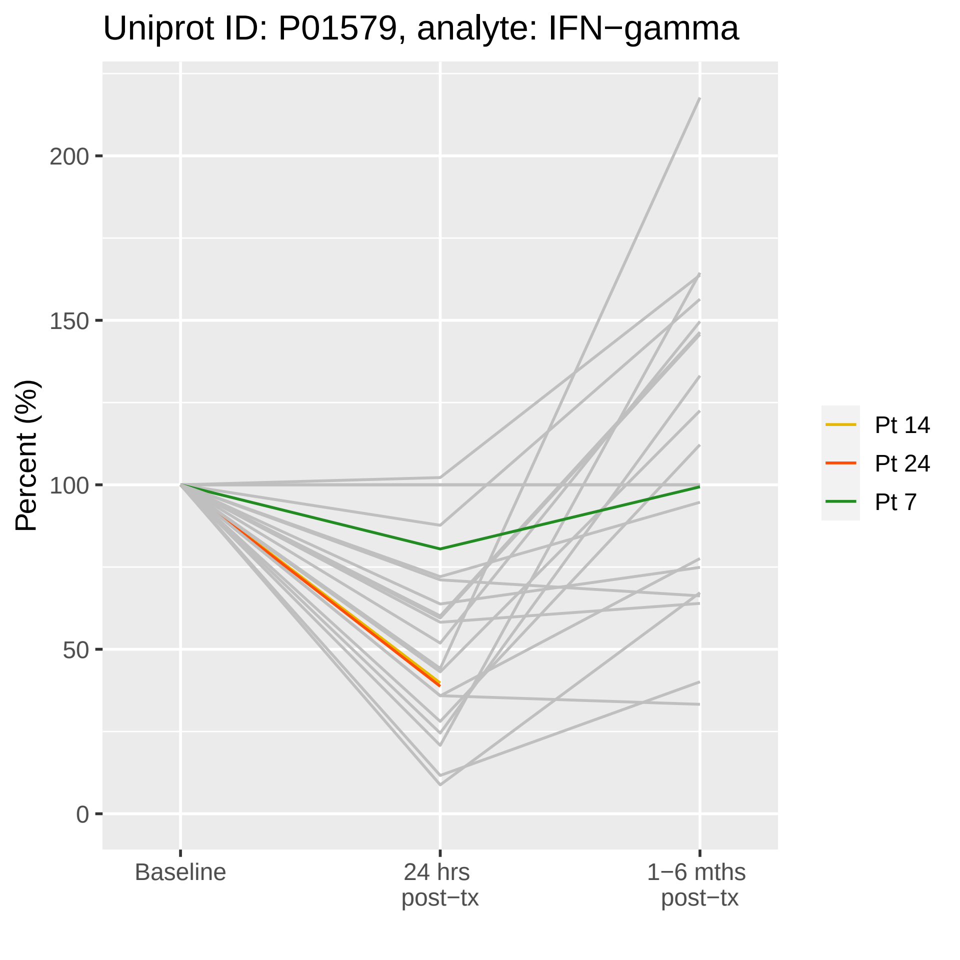
Uniprot ID: P01579, analyte: IFN−gamma
Percent (%)
200
150
100
50
0
Baseline
24 hrs post−tx
1−6 mths post−tx
Pt 14
Pt 24
Pt 7

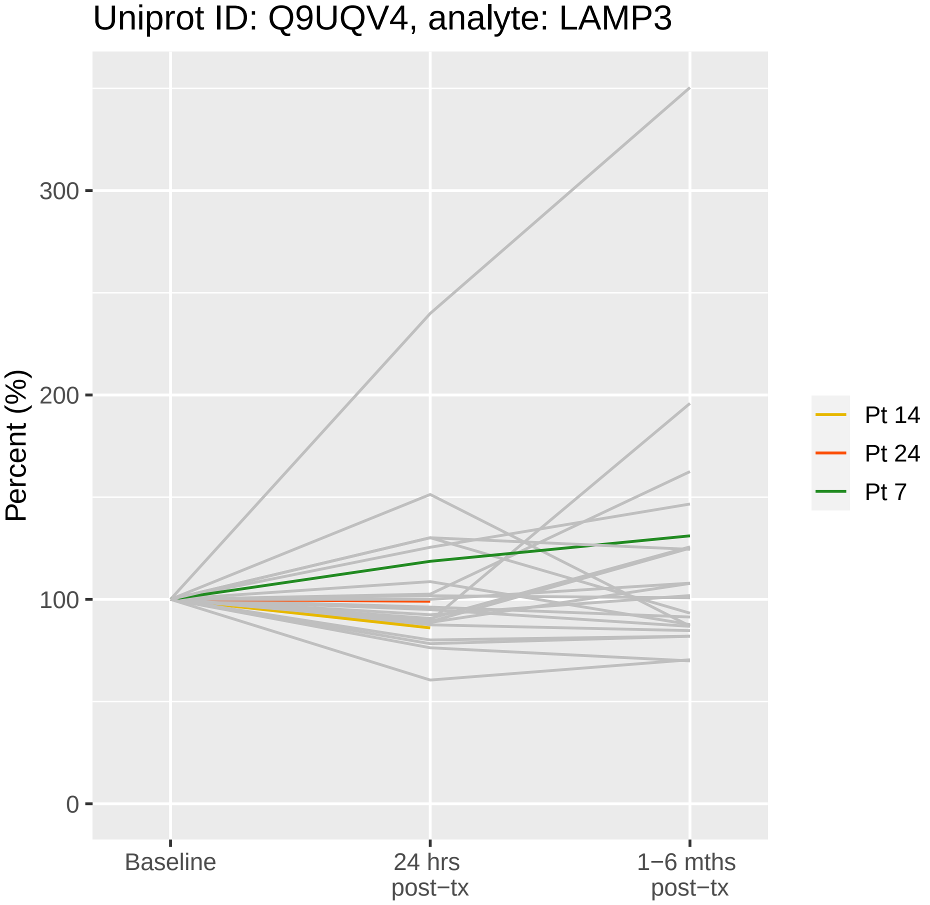

Uniprot ID: Q9UQV4, analyte: LAMP3
Percent (%)
0
100
200
300
Baseline
24 hrs post−tx
1−6 mths post−tx
Pt 14
Pt 24
Pt 7

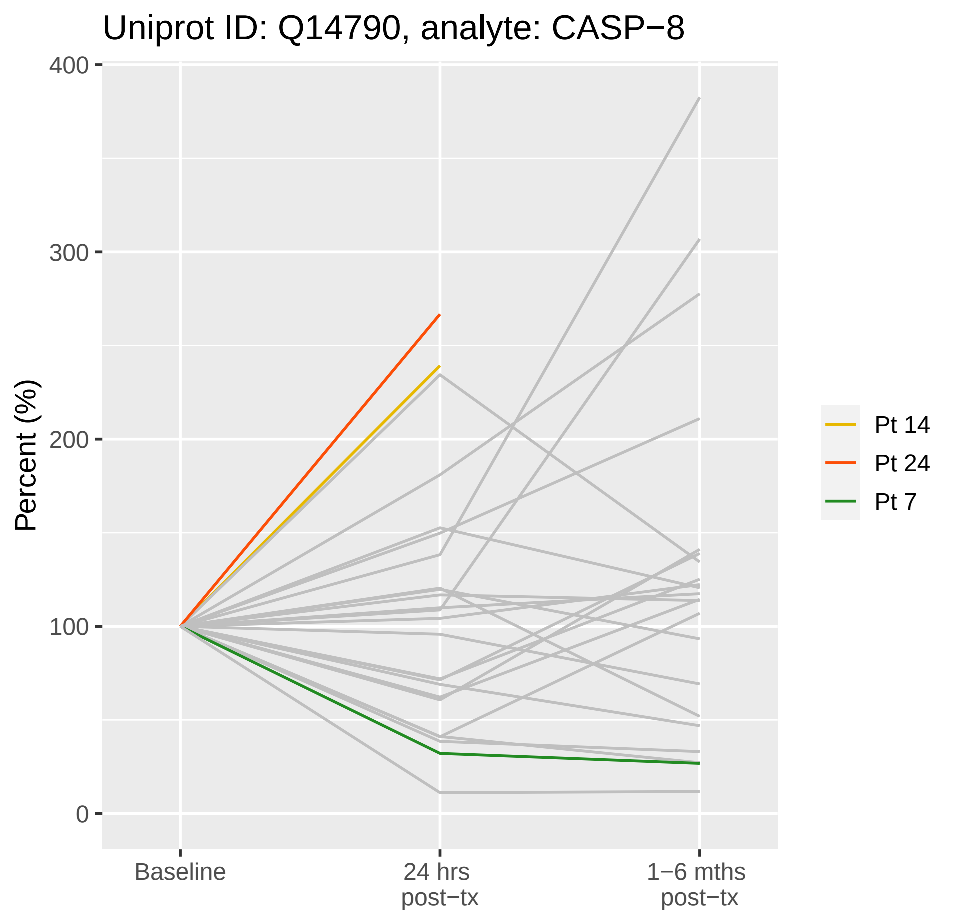
Uniprot ID: Q14790, analyte: CASP−8
Percent (%)
400
300
200
100
0
Baseline
24 hrs post−tx
1−6 mths post−tx
Pt 14
Pt 24
Pt 7

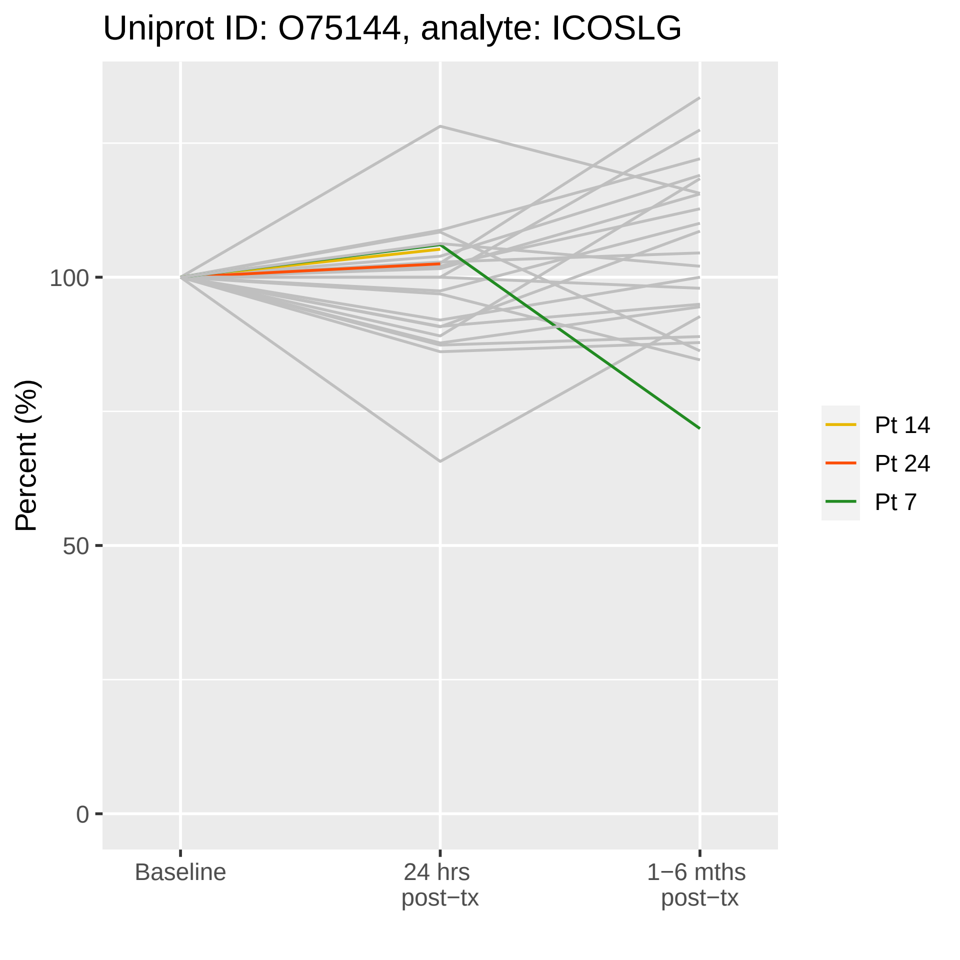

Uniprot ID: O75144, analyte: ICOSLG
Percent (%)
100
50
0
Baseline
24 hrs post−tx
1−6 mths post−tx
Pt 14
Pt 24
Pt 7

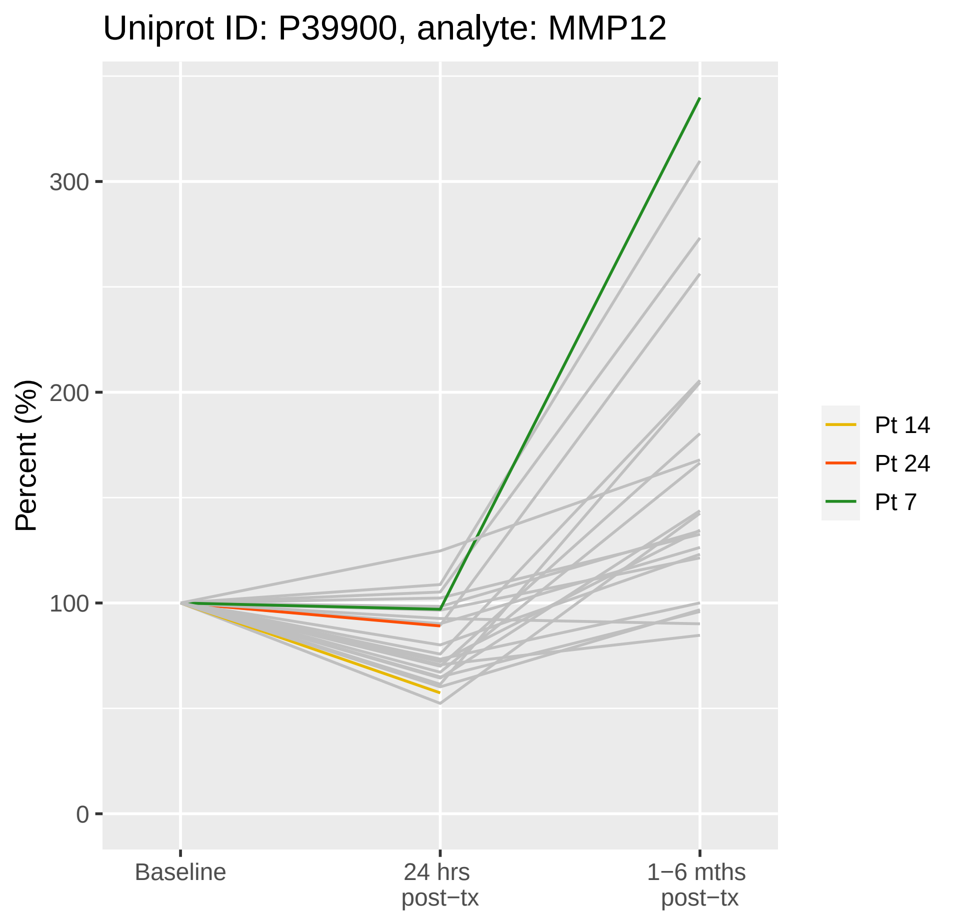

Uniprot ID: P39900, analyte: MMP12
Percent (%)
300
200
100
0
Baseline
24 hrs post−tx
1−6 mths post−tx
Pt 14
Pt 24
Pt 7

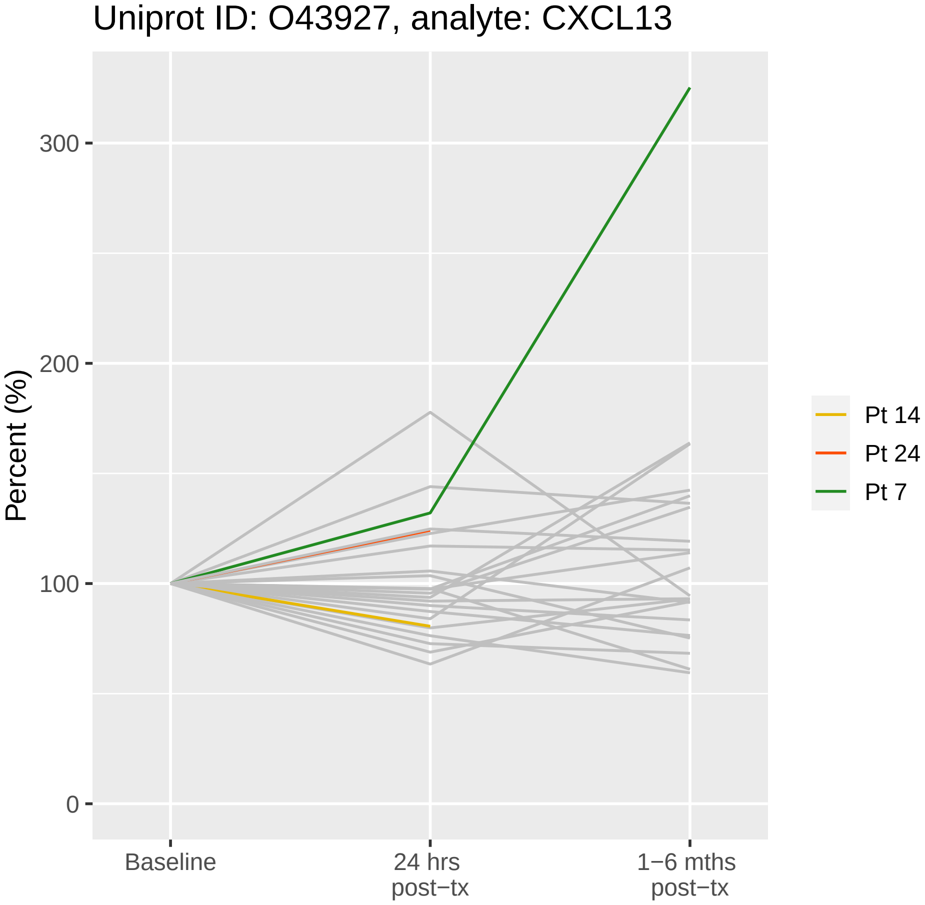
Uniprot ID: O43927, analyte: CXCL13
Percent (%)
300
200
100
0
Baseline
24 hrs post−tx
1−6 mths post−tx
Pt 14
Pt 24
Pt 7

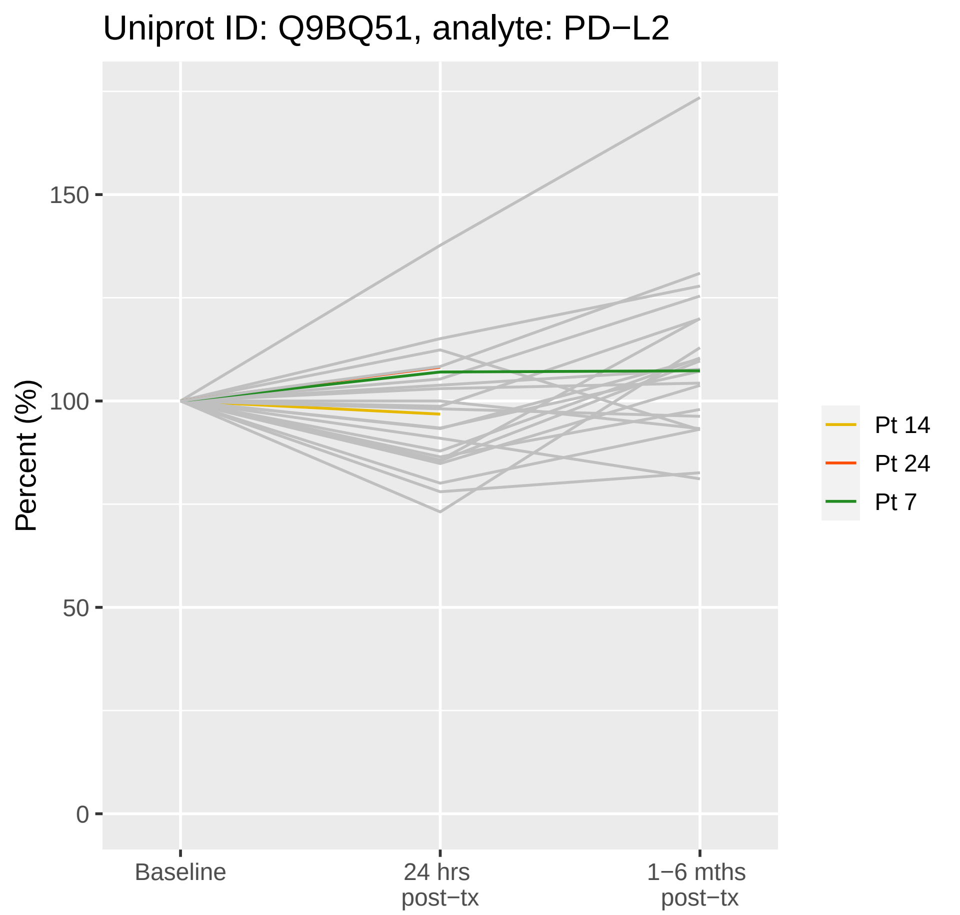
Uniprot ID: Q9BQ51, analyte: PD−L2
Percent (%)
150
100
50
0
Baseline
24 hrs post−tx
1−6 mths post−tx
Pt 14
Pt 24
Pt 7

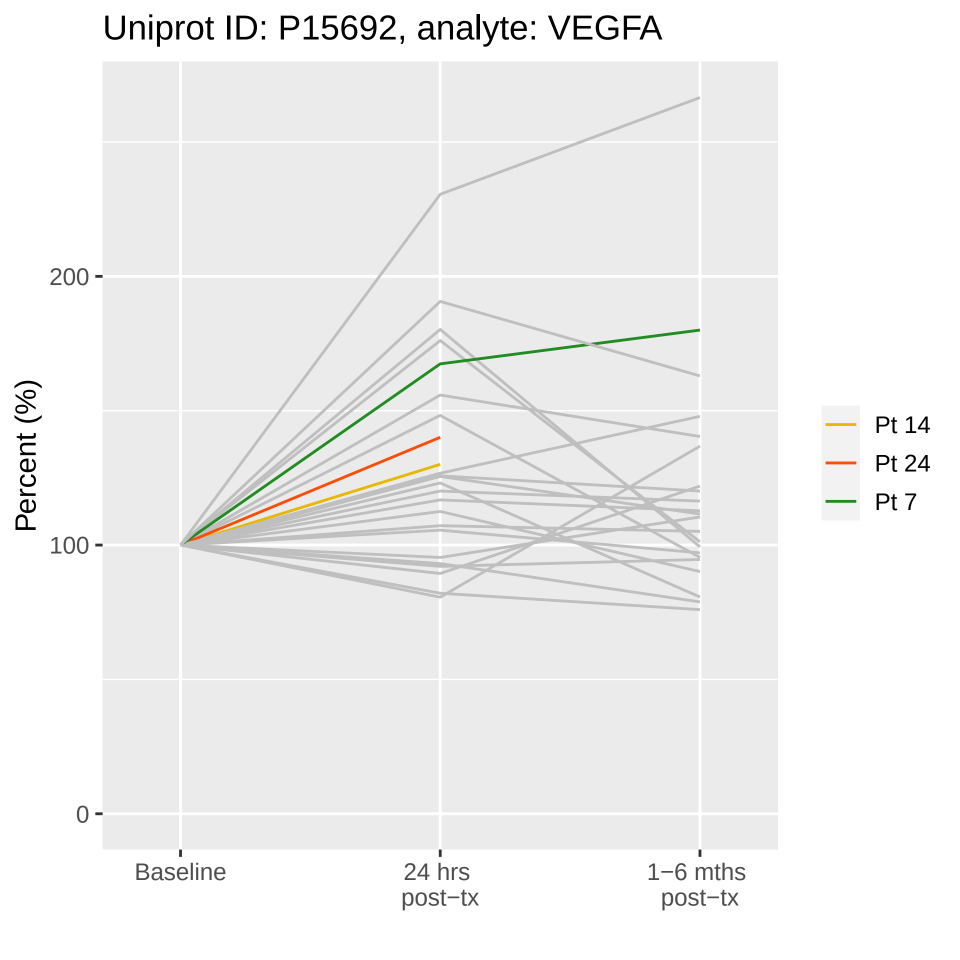

Uniprot ID: P15692, analyte: VEGFA
Percent (%)
200
100
0
Baseline
24 hrs post−tx
1−6 mths post−tx
Pt 14
Pt 24
Pt 7

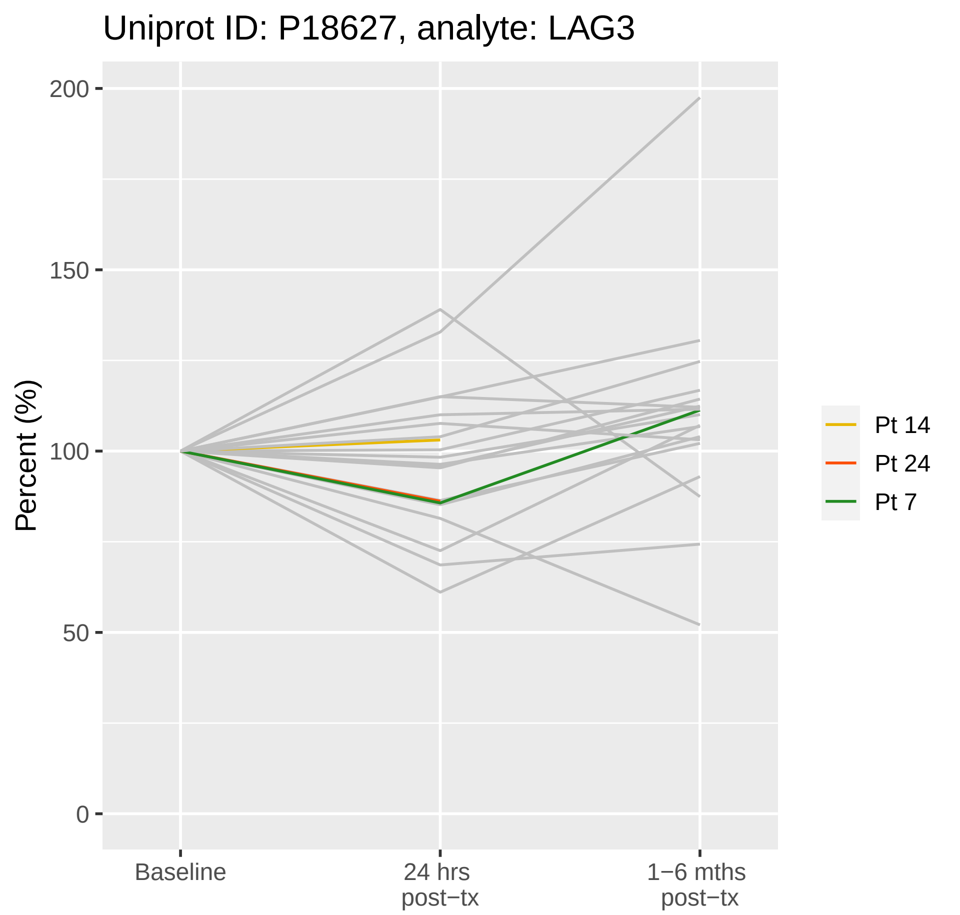

Uniprot ID: P18627, analyte: LAG3
Percent (%)
200
150
100
50
0
Baseline
24 hrs post−tx
1−6 mths post−tx
Pt 14
Pt 24
Pt 7

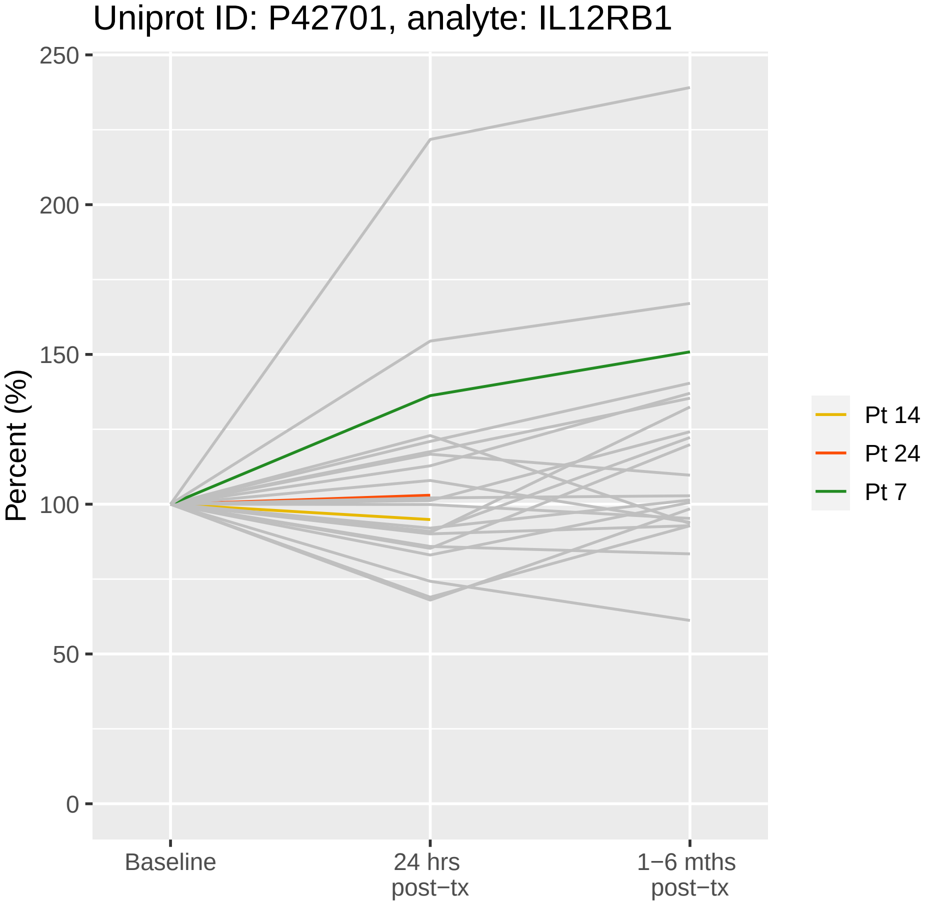
Uniprot ID: P42701, analyte: IL12RB1
Percent (%)
250
200
150
100
50
0
Baseline
24 hrs post−tx
1−6 mths post−tx
Pt 14
Pt 24
Pt 7

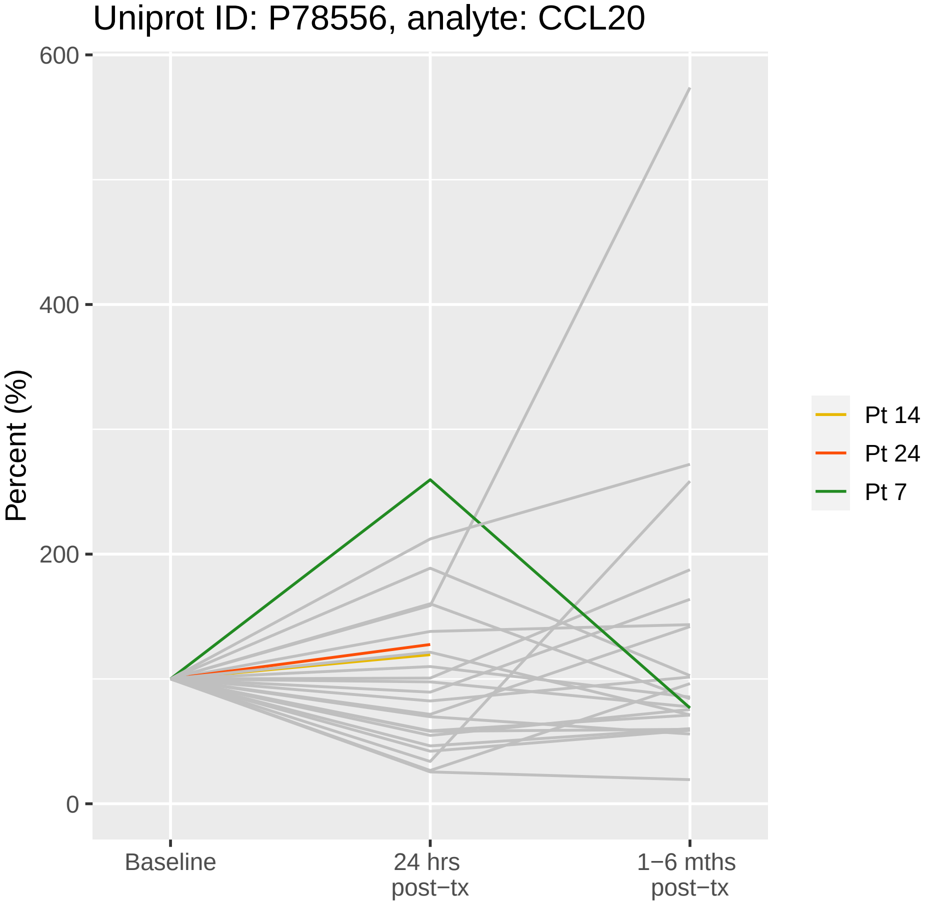
Uniprot ID: P78556, analyte: CCL20
Percent (%)
600
400
200
0
Baseline
24 hrs post−tx
1−6 mths post−tx
Pt 14
Pt 24
Pt 7

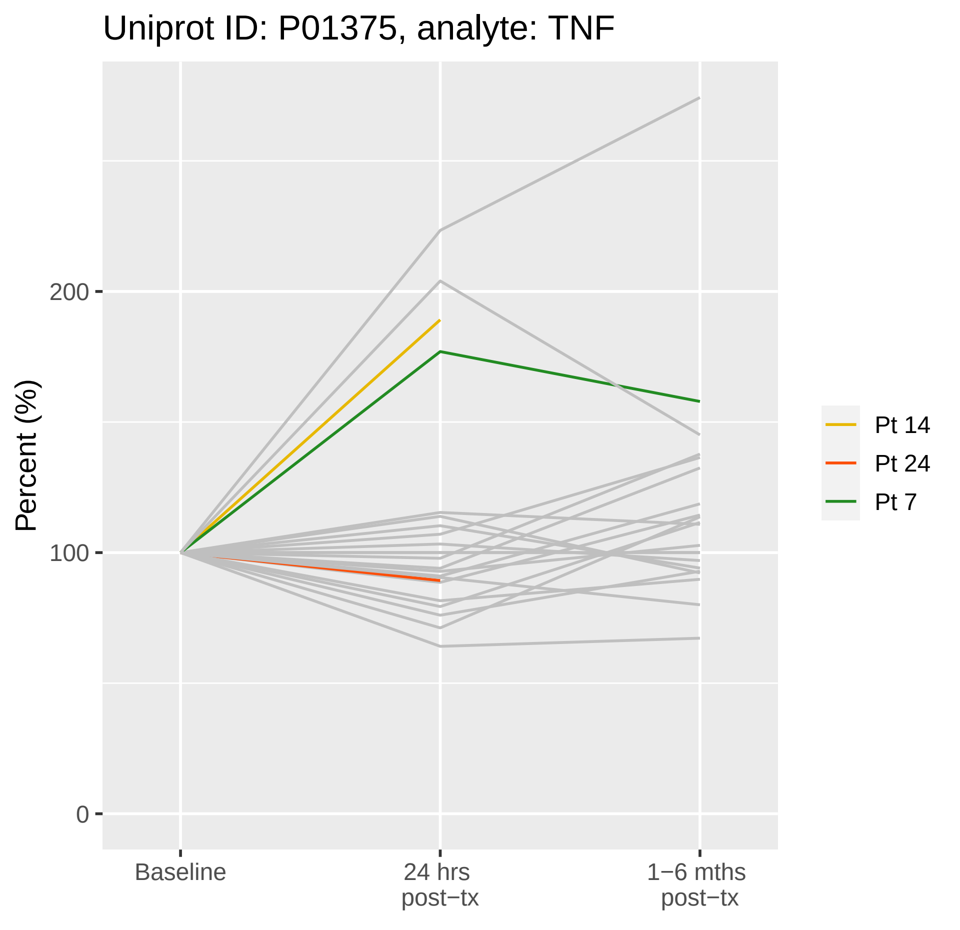

Uniprot ID: P01375, analyte: TNF
Percent (%)
200
100
0
Baseline
24 hrs post−tx
1−6 mths post−tx
Pt 14
Pt 24
Pt 7

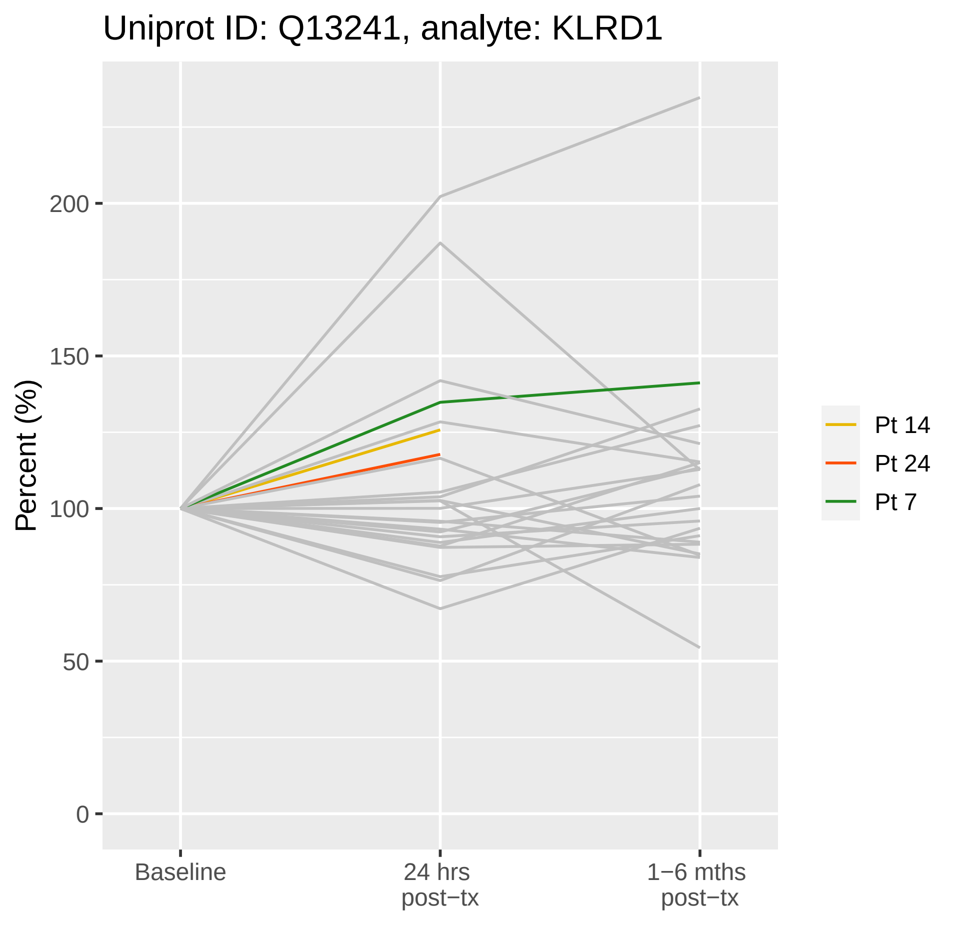

Uniprot ID: Q13241, analyte: KLRD1
Percent (%)
200
150
100
50
0
Baseline
24 hrs post−tx
1−6 mths post−tx
Pt 14
Pt 24
Pt 7

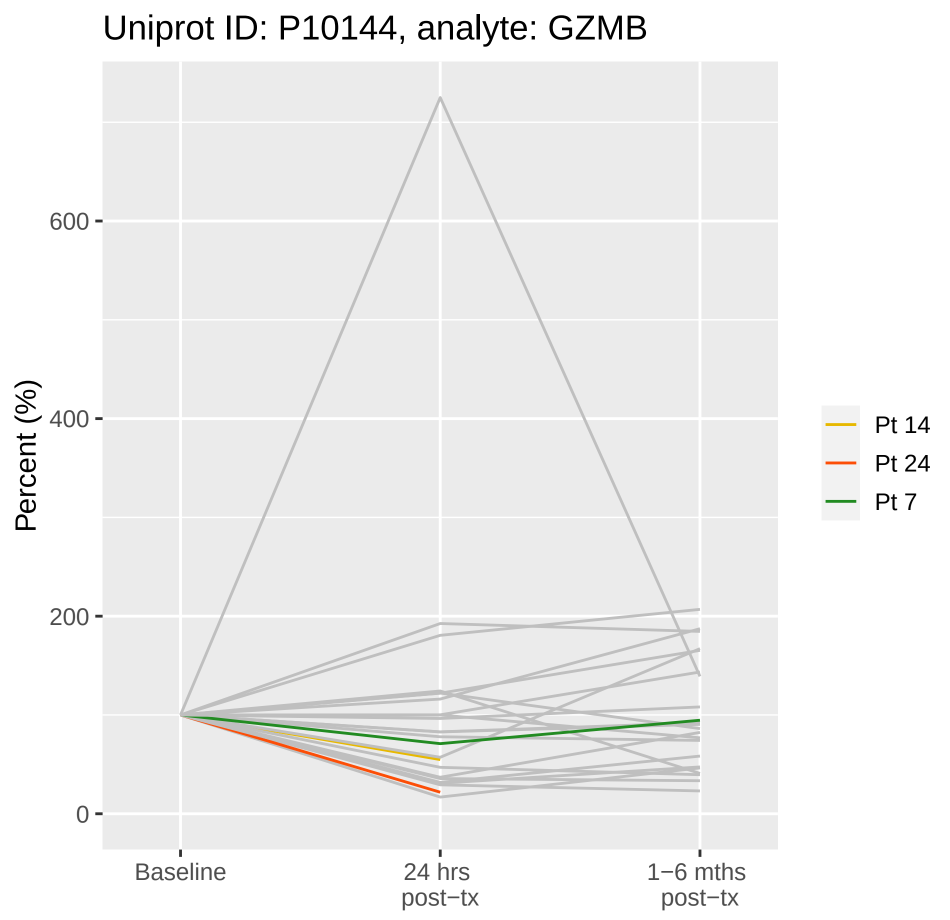

Uniprot ID: P10144, analyte: GZMB
Percent (%)
600
400
200
0
Baseline
24 hrs post−tx
1−6 mths post−tx
Pt 14
Pt 24
Pt 7

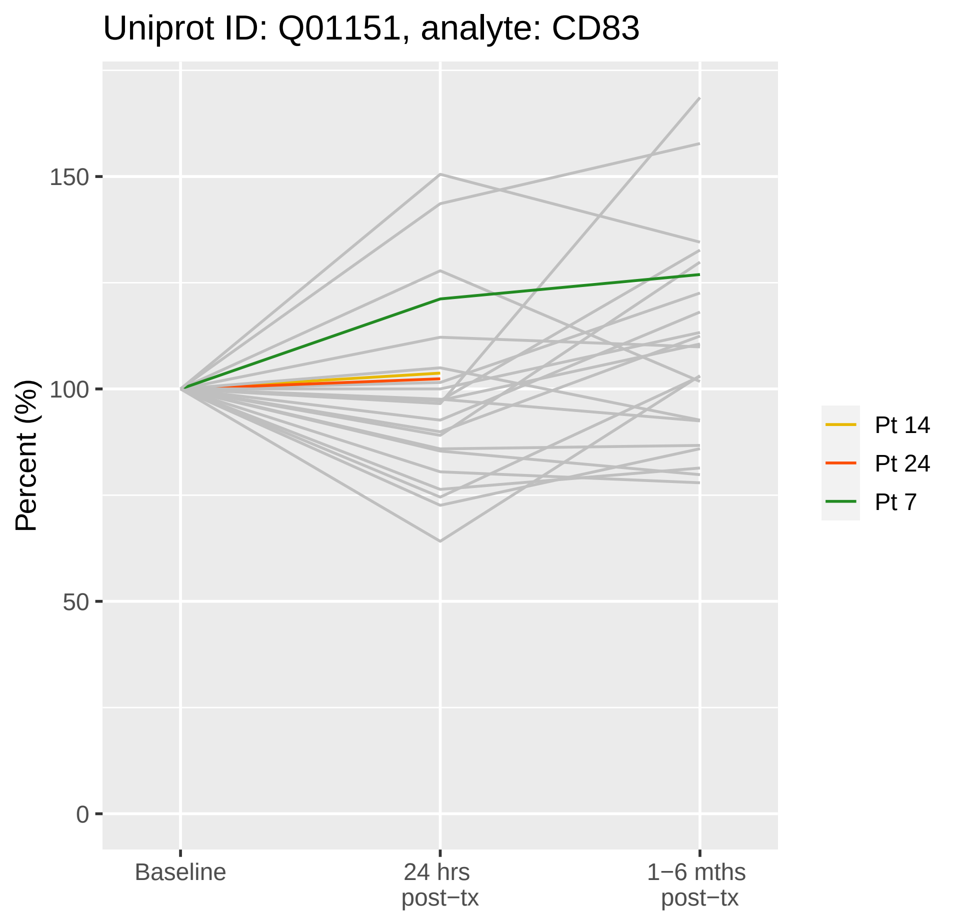

Uniprot ID: Q01151, analyte: CD83
Percent (%)
150
100
50
0
Baseline
24 hrs
post−tx
1−6 mths
post−tx
Pt 14
Pt 24
Pt 7

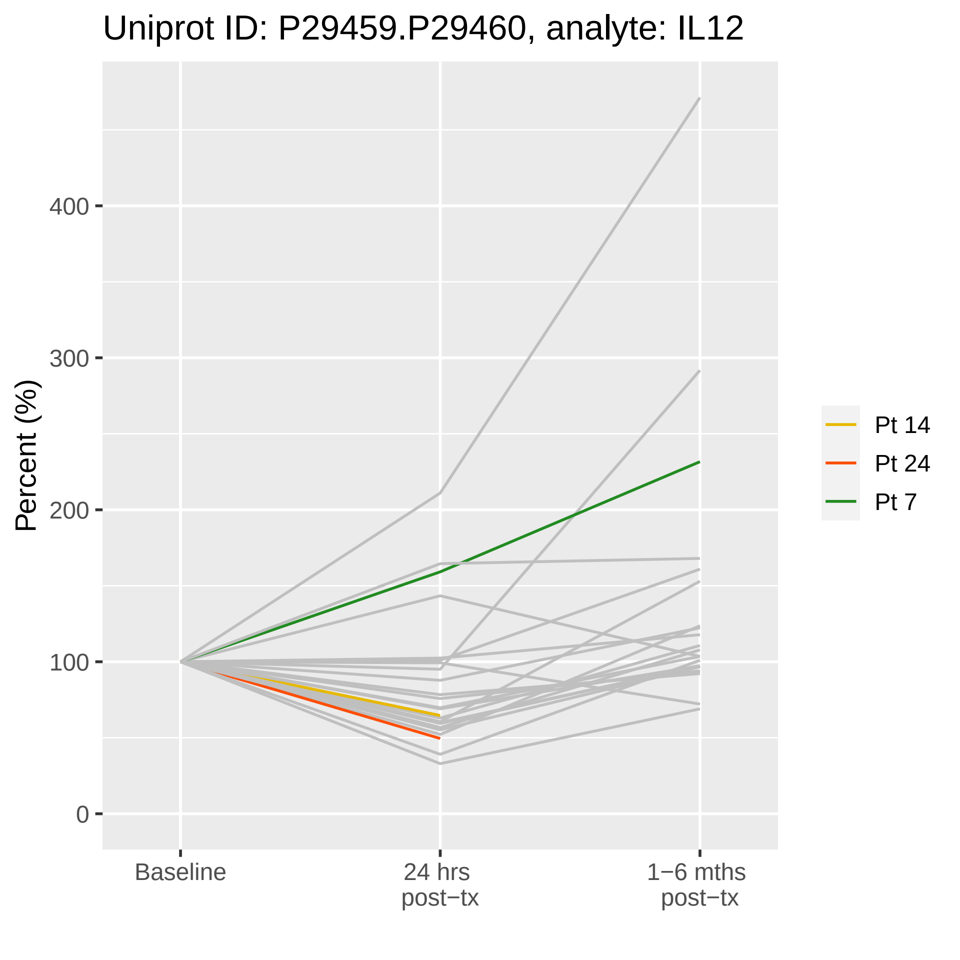
Uniprot ID: P29459.P29460, analyte: IL12
Percent (%)
400
300
200
100
0
Baseline
24 hrs post−tx
1−6 mths post−tx
Pt 14
Pt 24
Pt 7

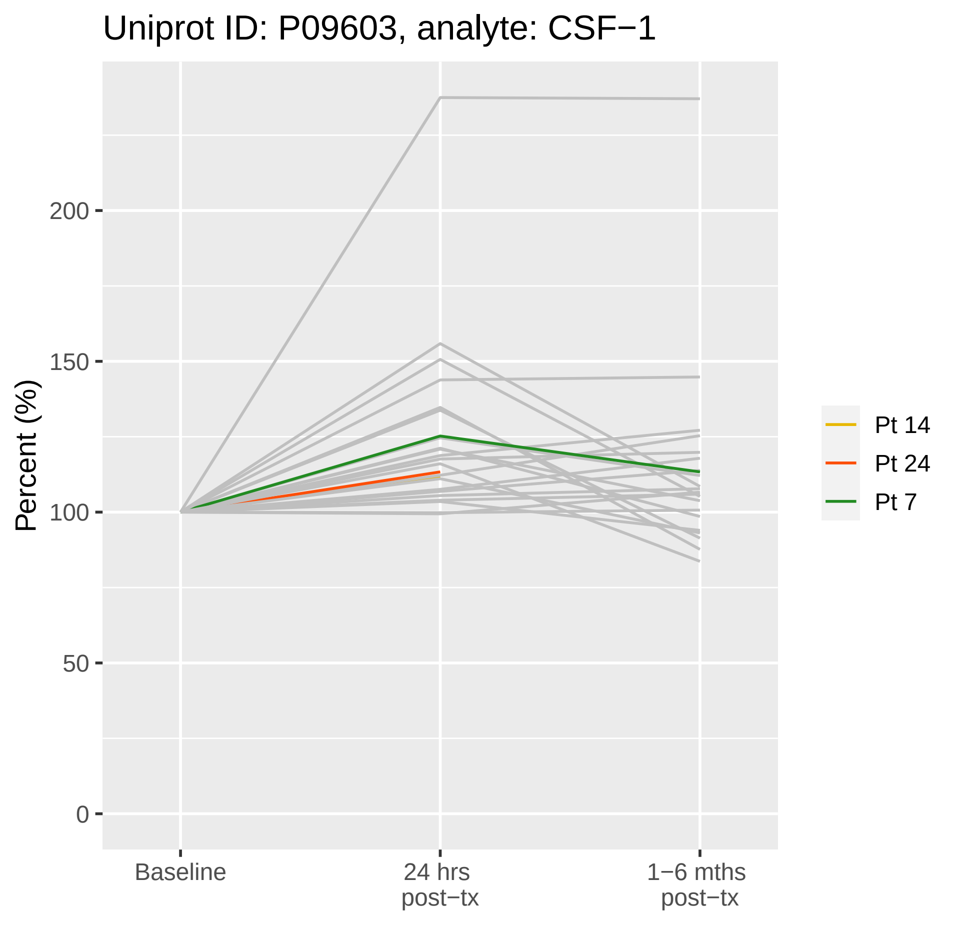

Uniprot ID: P09603, analyte: CSF−1
Percent (%)
200
150
100
50
0
Baseline
24 hrs post−tx
1−6 mths post−tx
Pt 14
Pt 24
Pt 7
