# Supplementary material for: An Exploratory Analysis of Changes in Circulating Plasma Protein Profiles Following Image-Guided Ablation of Renal Tumours Provides Evidence for Effects on Multiple Biological Processes
Source: Cancers (Basel). 2021 Nov 30;13(23):6037. doi: 10.3390/cancers13236037 (PMC8656737; doi:10.3390/cancers13236037)
Supplement: Supplementary file 1 [file cancers-13-06037-s001.zip › Supplementary File/Figure S11.pdf]

Figure S11. Longitudinal profiles of all plasma proteins on the Oncology II panel, prior to and post-ablation. Results for each protein are expressed as a % of the reference baseline (TP2; 100%) and are shown for patients receiving CRYO (n=25) with the three patients who developed acute complications post-CRYO highlighted.

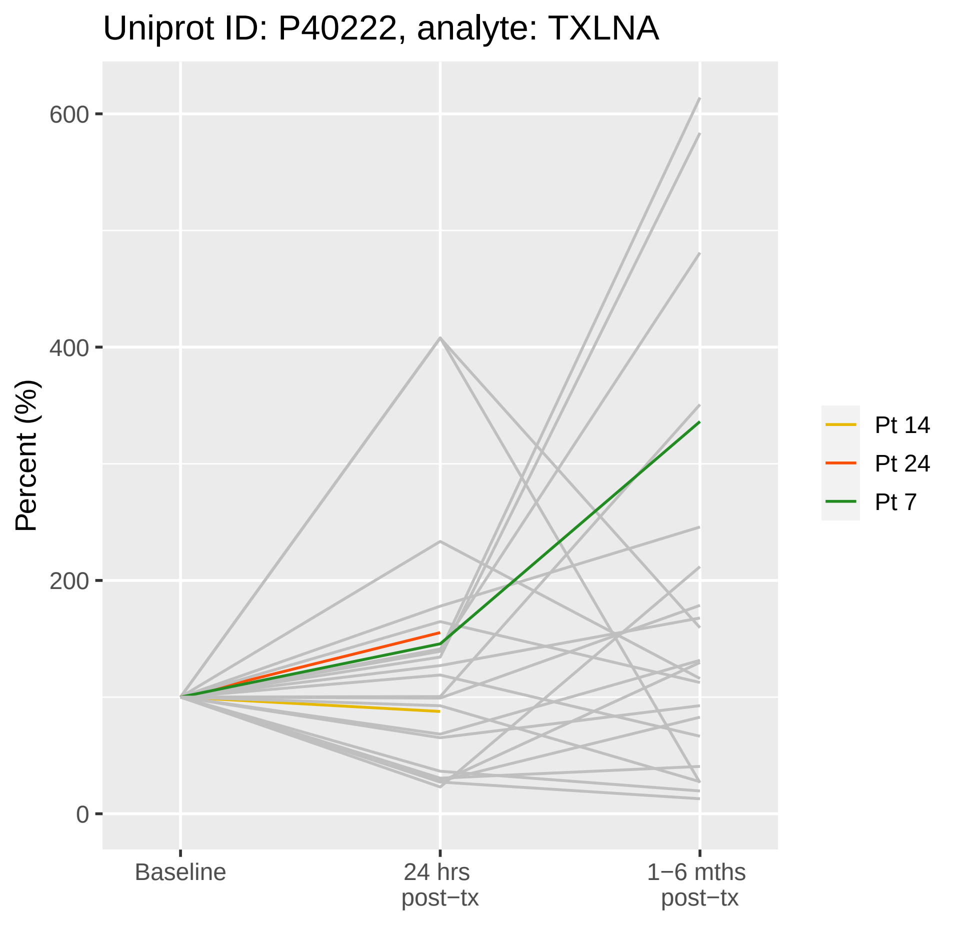
Uniprot ID: P40222, analyte: TXLNA
Percent (%)
600
400
200
0
Baseline
24 hrs post−tx
1−6 mths post−tx
Pt 14
Pt 24
Pt 7

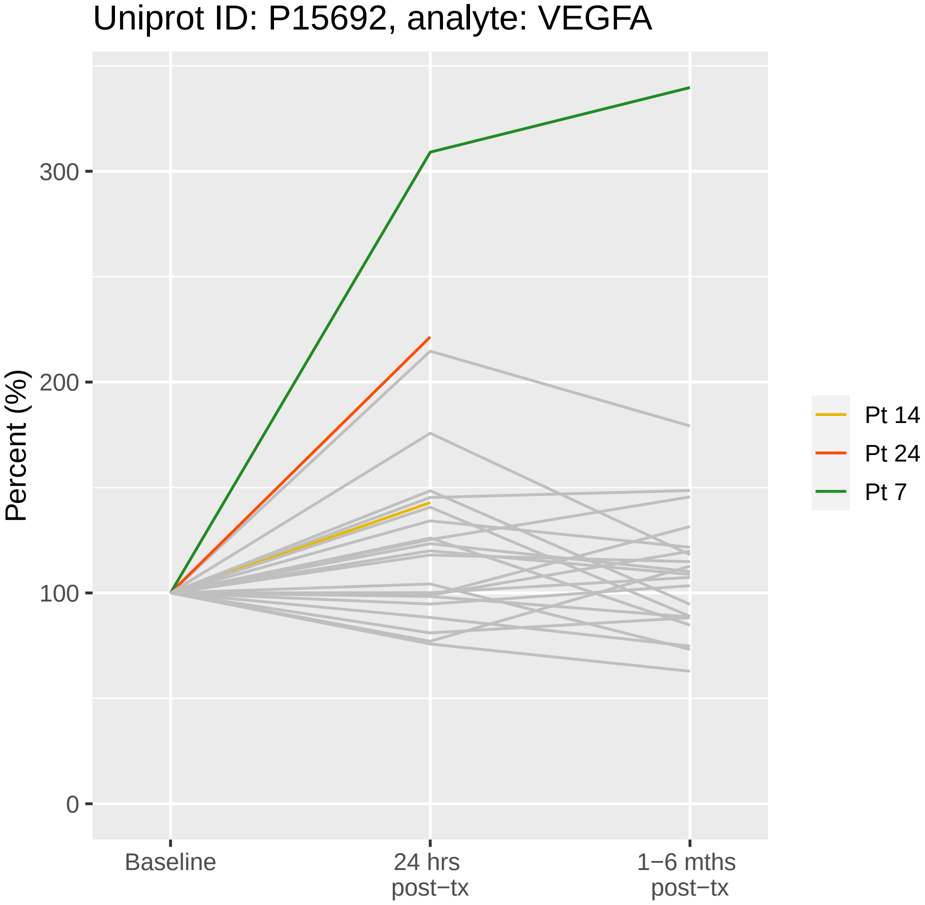

Uniprot ID: P15692, analyte: VEGFA
Percent (%)
300
200
100
0
Baseline
24 hrs
post−tx
1−6 mths
post−tx
Pt 14
Pt 24
Pt 7

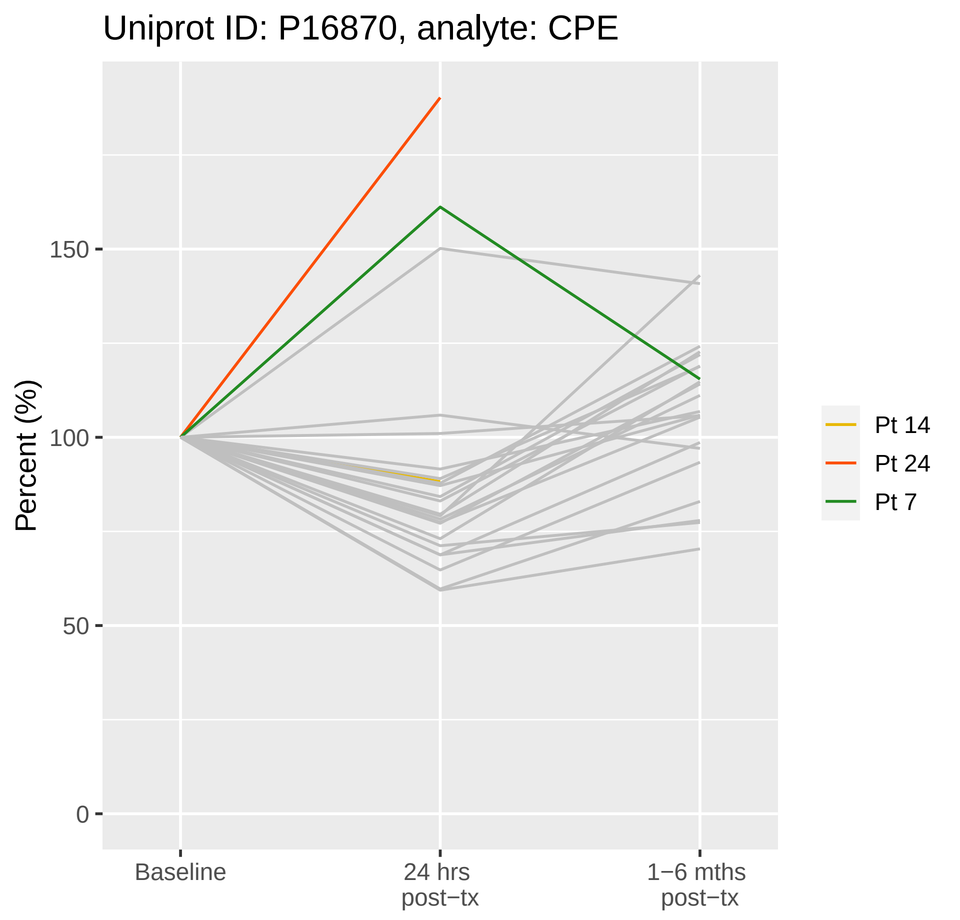
Uniprot ID: P16870, analyte: CPE
Percent (%)
150
100
50
0
Baseline
24 hrs post−tx
1−6 mths post−tx
Pt 14
Pt 24
Pt 7

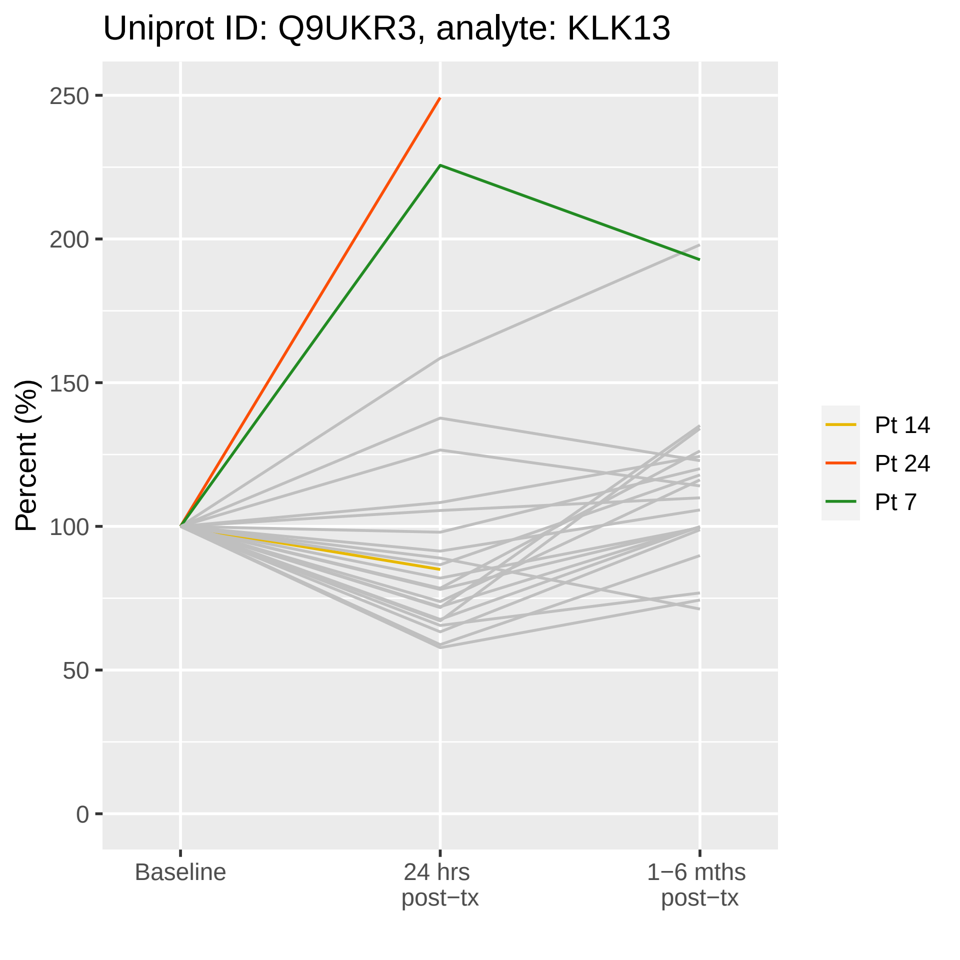
Uniprot ID: Q9UKR3, analyte: KLK13
Percent (%)
0
50
100
150
200
250
Baseline
24 hrs
post−tx
1−6 mths
post−tx
Pt 14
Pt 24
Pt 7

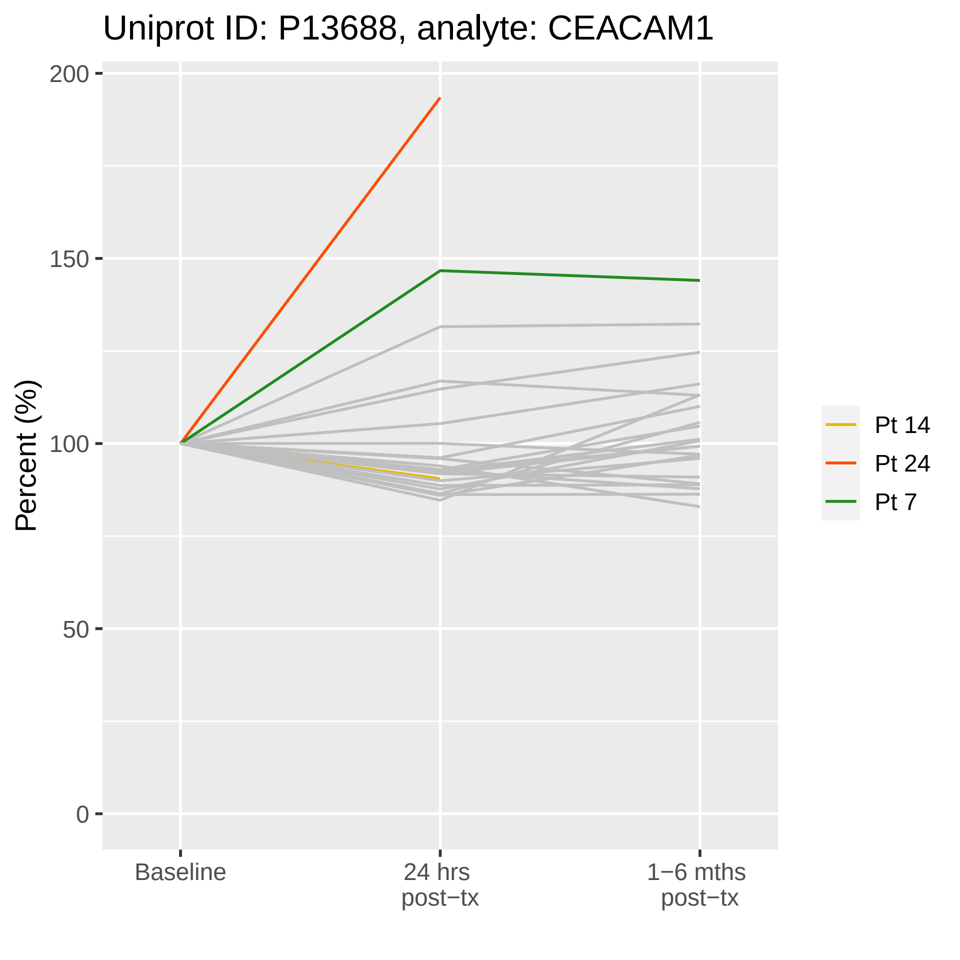
Uniprot ID: P13688, analyte: CEACAM1
Percent (%)
200
150
100
50
0
Baseline
24 hrs post−tx
1−6 mths post−tx
Pt 14
Pt 24
Pt 7

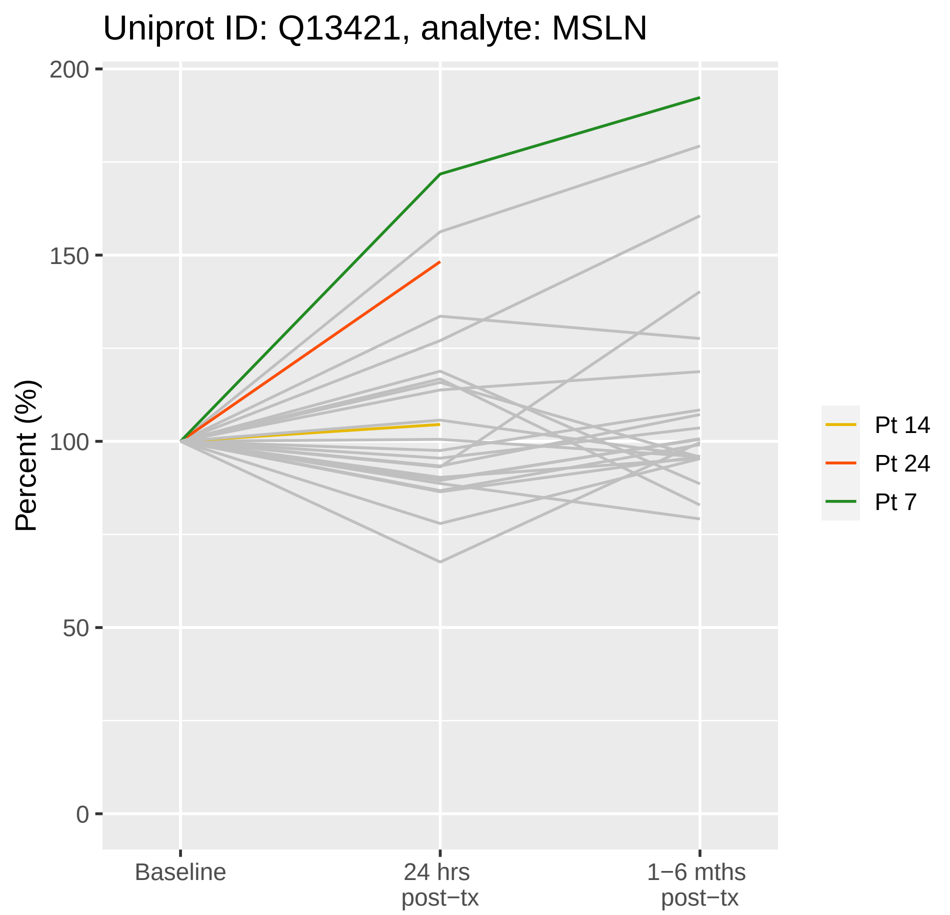

Uniprot ID: Q13421, analyte: MSLN
Percent (%)
200
150
100
50
0
Baseline
24 hrs post−tx
1−6 mths post−tx
Pt 14
Pt 24
Pt 7

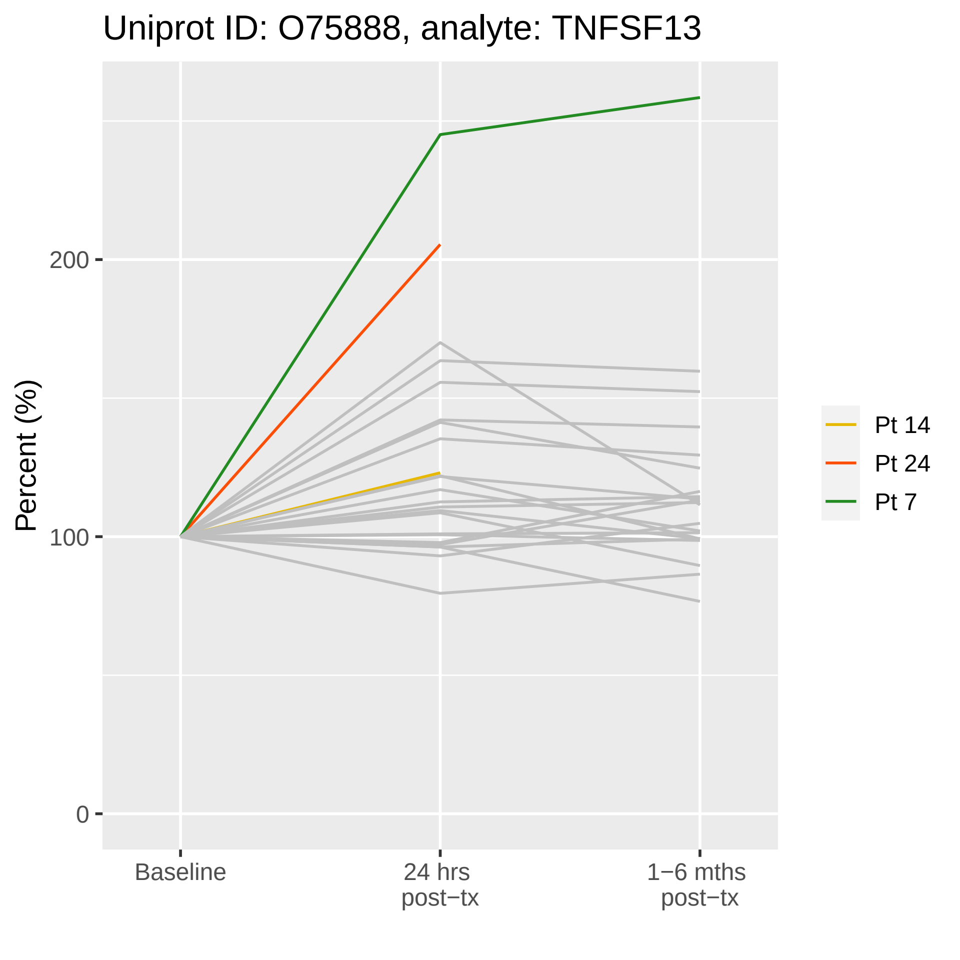

Uniprot ID: O75888, analyte: TNFSF13
Percent (%)
200
100
0
Baseline
24 hrs
post−tx
1−6 mths
post−tx
Pt 14
Pt 24
Pt 7

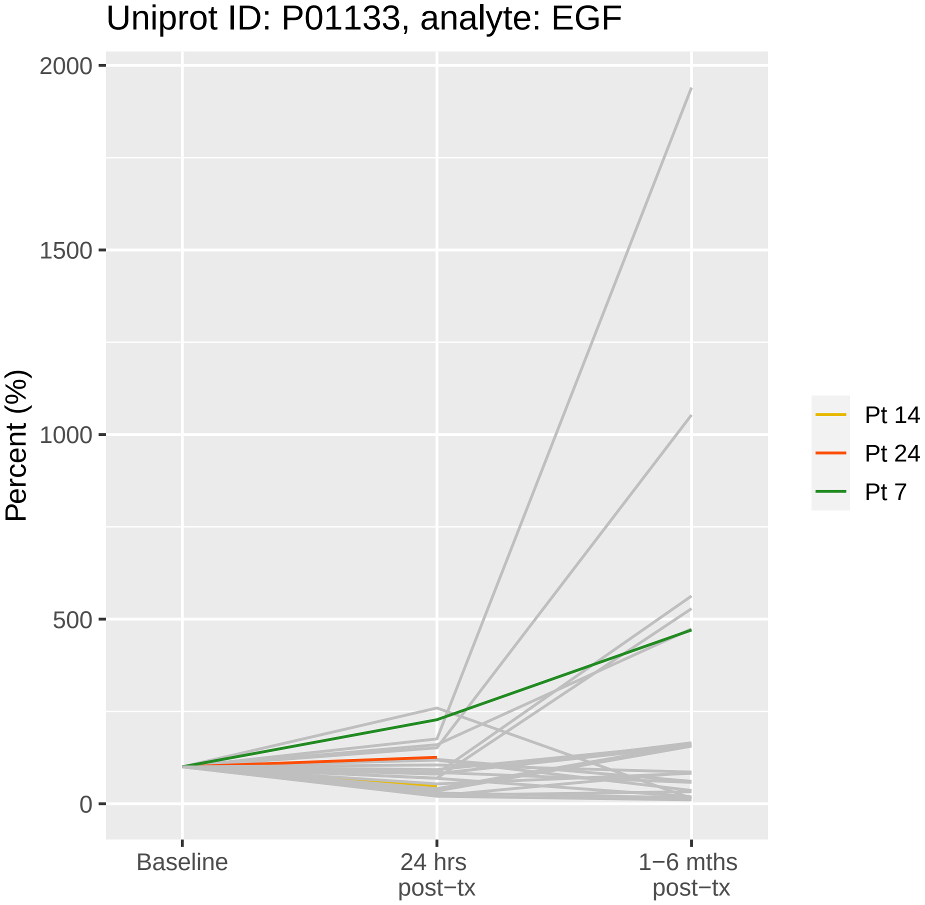

Uniprot ID: P01133, analyte: EGF
Percent (%)
2000
1500
1000
500
0
Baseline
24 hrs post−tx
1−6 mths post−tx
Pt 14
Pt 24
Pt 7

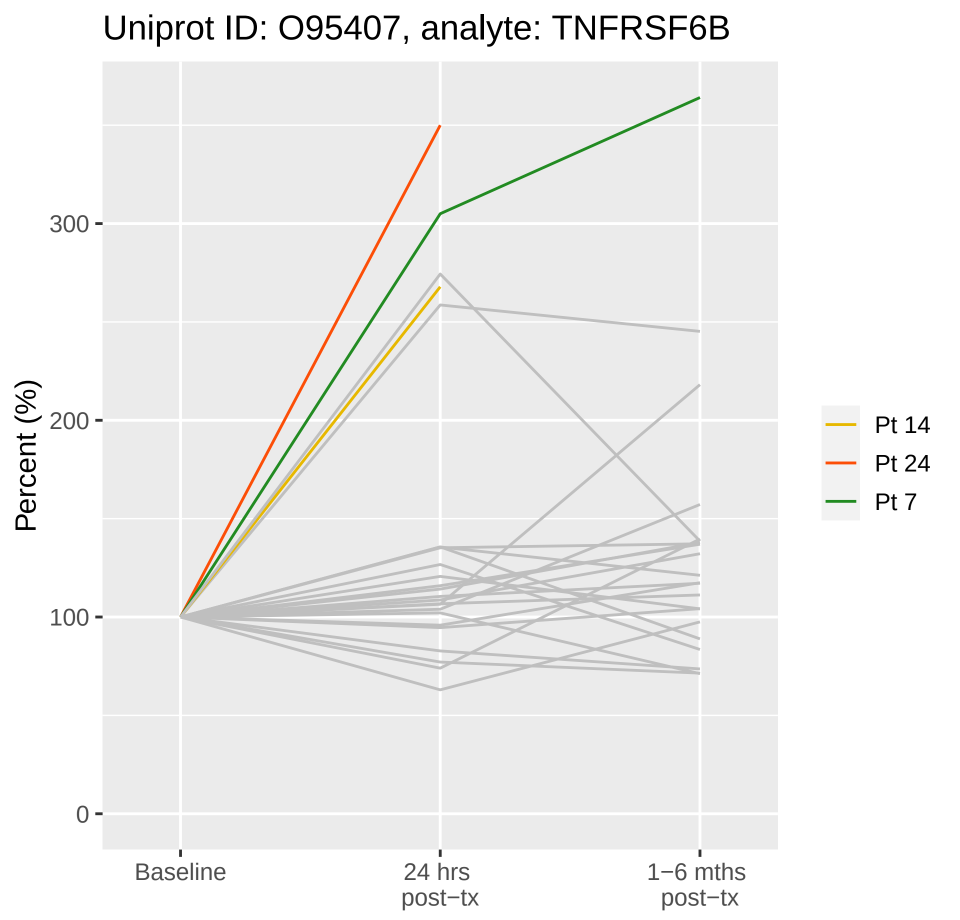
Uniprot ID: O95407, analyte: TNFRSF6B
Percent (%)
300
200
100
0
Baseline
24 hrs post−tx
1−6 mths post−tx
Pt 14
Pt 24
Pt 7

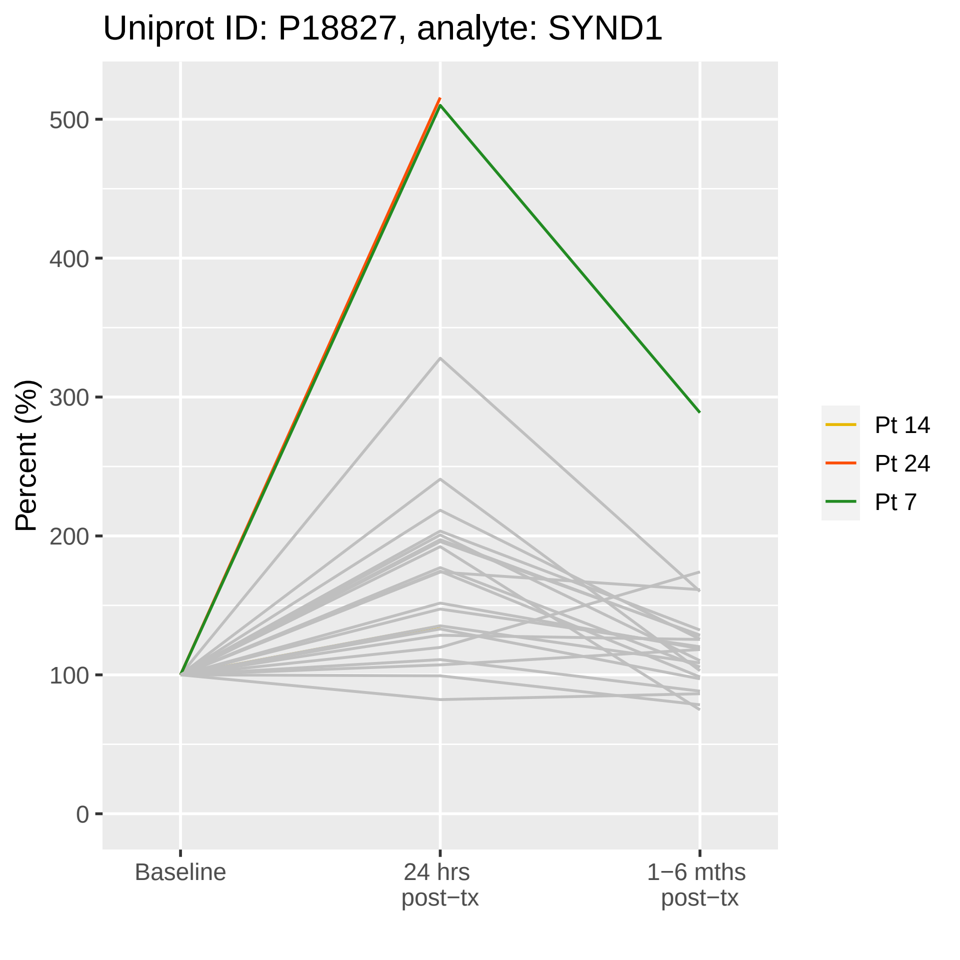
Uniprot ID: P18827, analyte: SYND1
Percent (%)
0
100
200
300
400
500
Baseline
24 hrs
post−tx
1−6 mths
post−tx
Pt 14
Pt 24
Pt 7

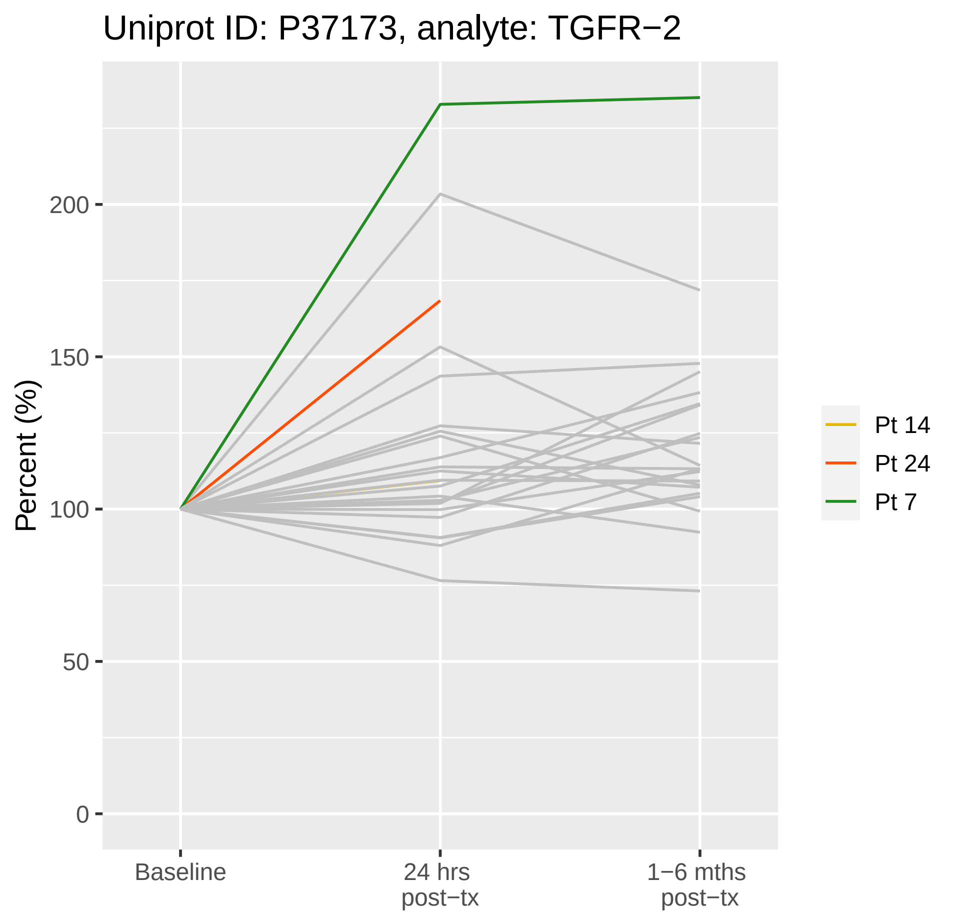
Uniprot ID: P37173, analyte: TGFR−2
Percent (%)
0
50
100
150
200
Baseline
24 hrs post−tx
1−6 mths post−tx
Pt 14
Pt 24
Pt 7

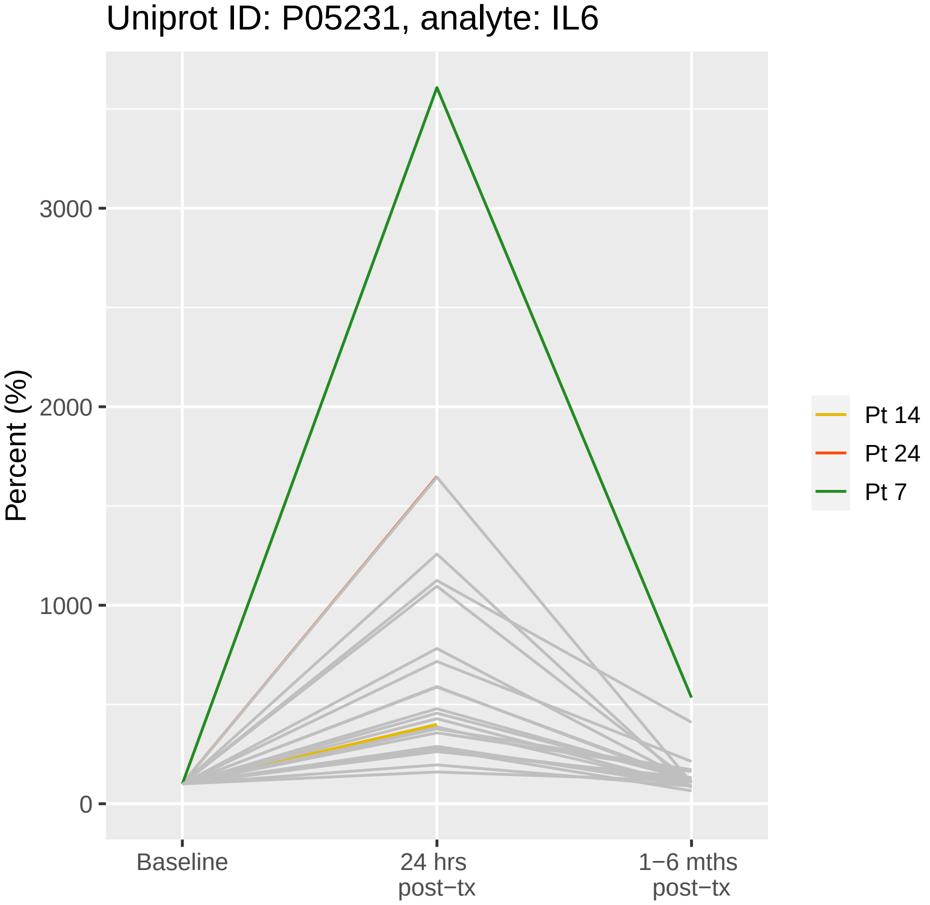

Uniprot ID: P05231, analyte: IL6
Percent (%)
3000
2000
1000
0
Baseline
24 hrs post−tx
1−6 mths post−tx
Pt 14
Pt 24
Pt 7

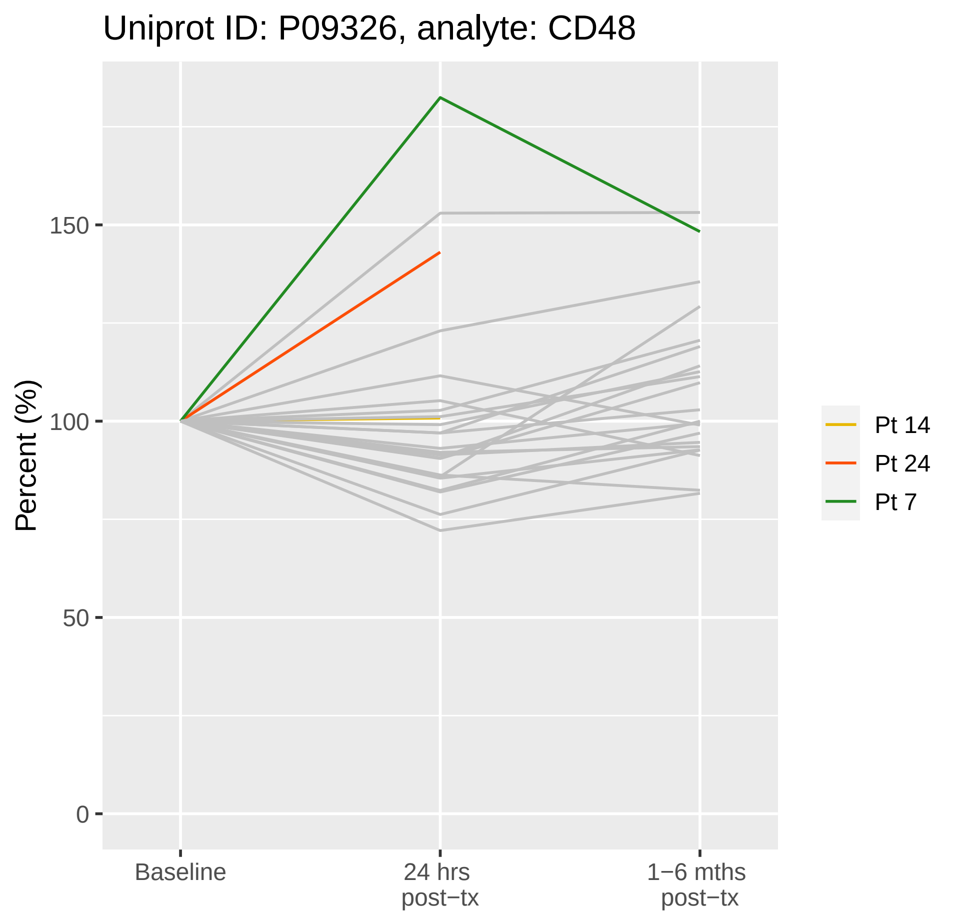
Uniprot ID: P09326, analyte: CD48
Percent (%)
150
100
50
0
Baseline
24 hrs post−tx
1−6 mths post−tx
Pt 14
Pt 24
Pt 7

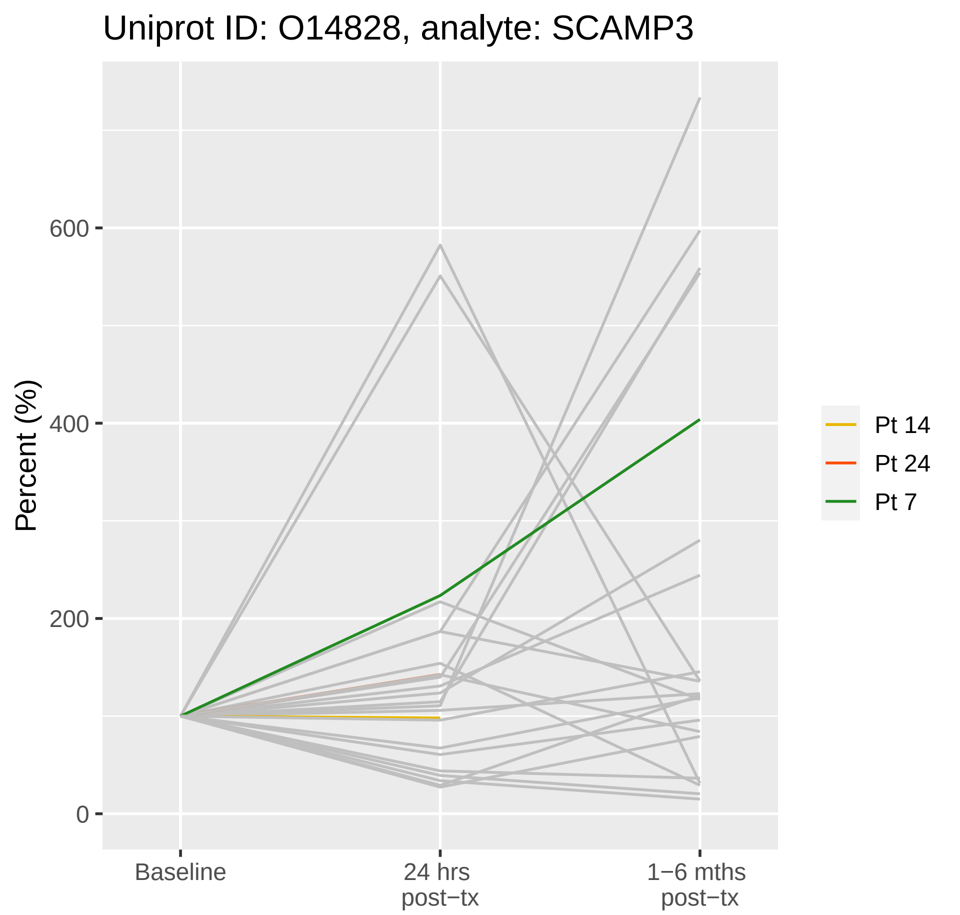

Uniprot ID: O14828, analyte: SCAMP3
Percent (%)
0
200
400
600
Baseline
24 hrs
post−tx
1−6 mths
post−tx
Pt 14
Pt 24
Pt 7

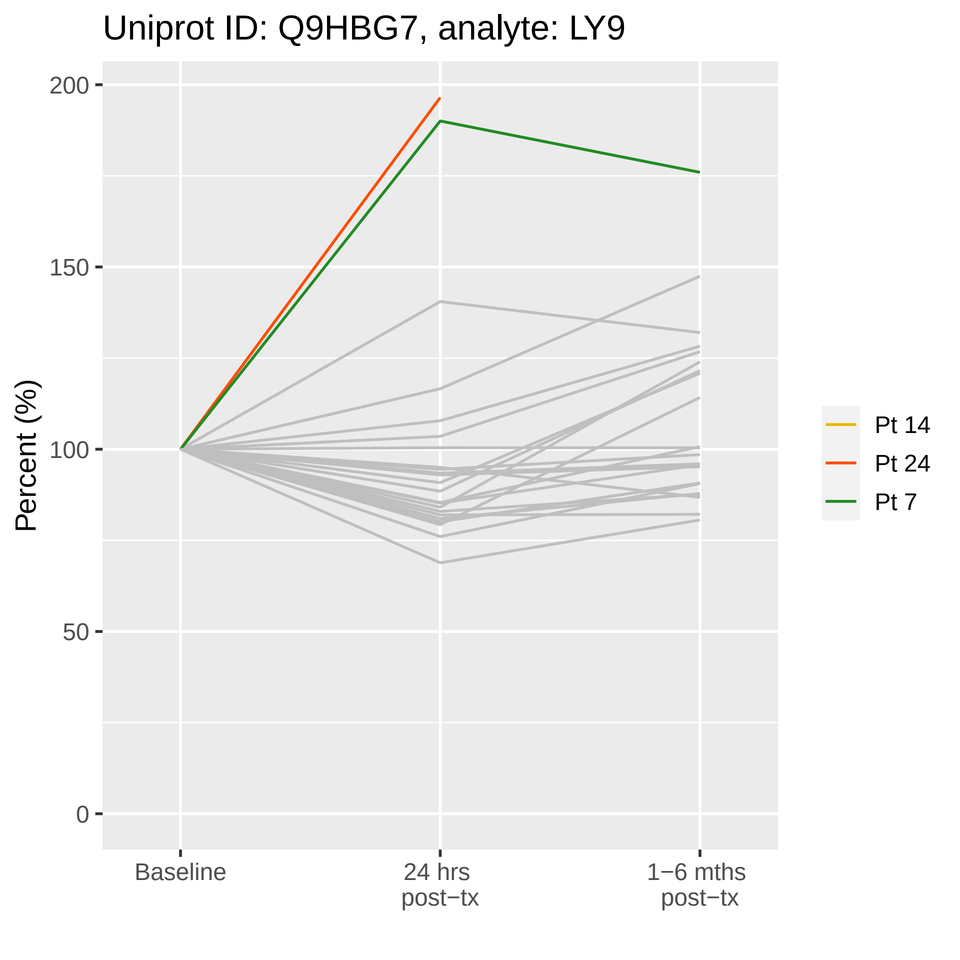

Uniprot ID: Q9HBG7, analyte: LY9
Percent (%)
200
150
100
50
0
Baseline
24 hrs post−tx
1−6 mths post−tx
Pt 14
Pt 24
Pt 7

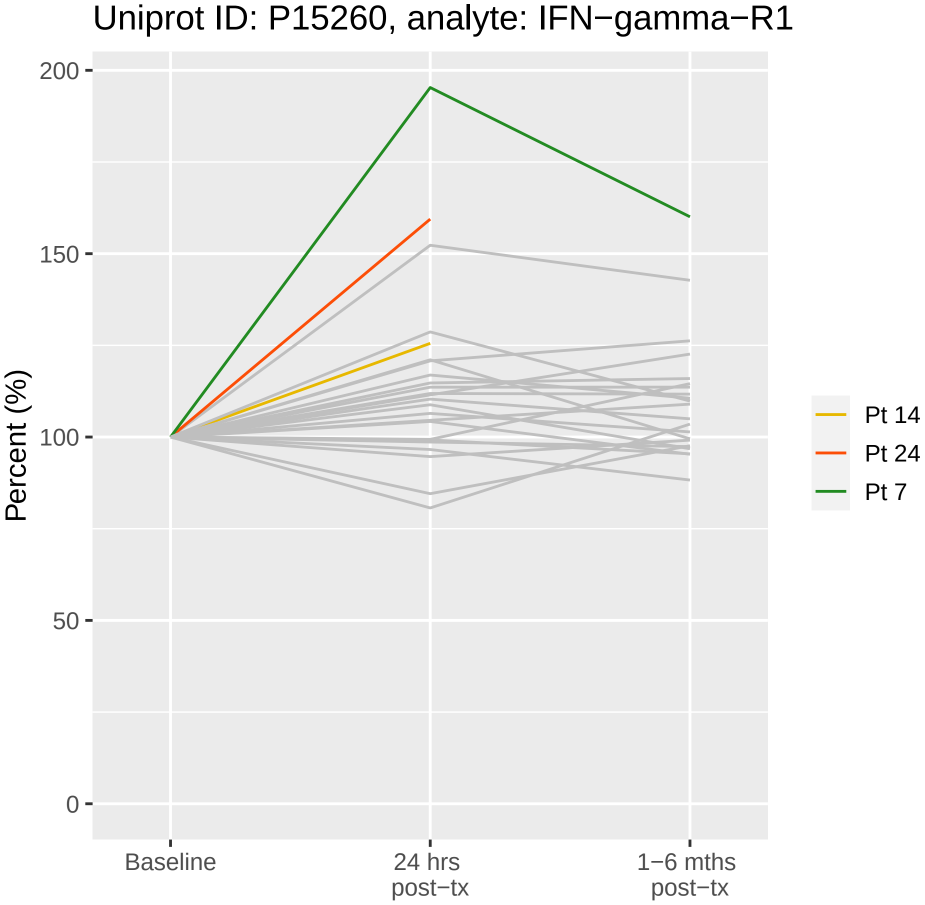
Uniprot ID: P15260, analyte: IFN−gamma−R1
Percent (%)
200
150
100
50
0
Baseline
24 hrs post−tx
1−6 mths post−tx
Pt 14
Pt 24
Pt 7

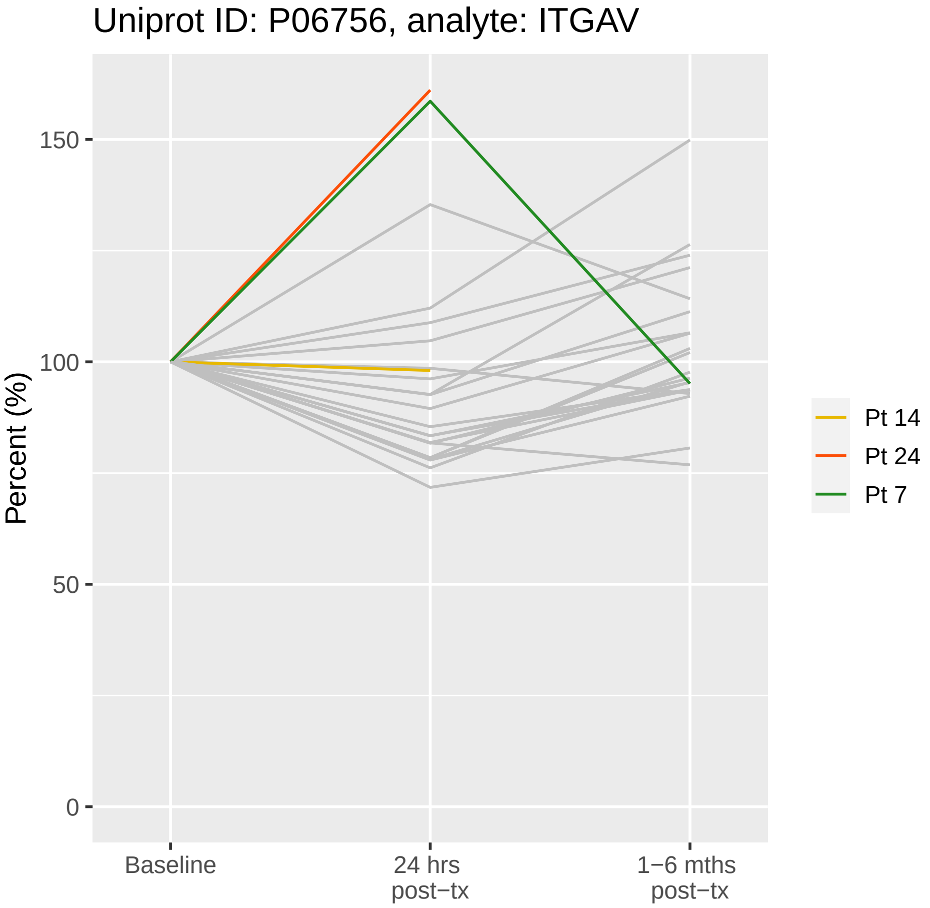

Uniprot ID: P06756, analyte: ITGAV
Percent (%)
150
100
50
0
Baseline
24 hrs post−tx
1−6 mths post−tx
Pt 14
Pt 24
Pt 7

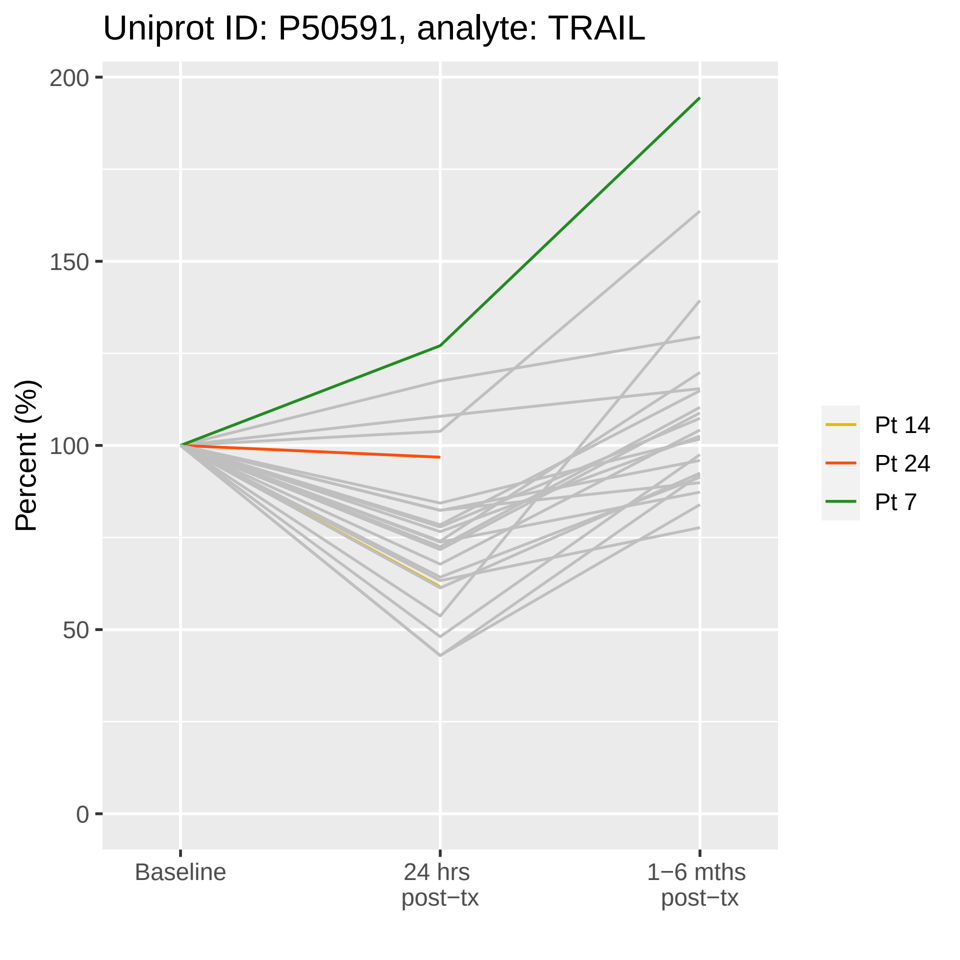
Uniprot ID: P50591, analyte: TRAIL
Percent (%)
200
150
100
50
0
Baseline
24 hrs post−tx
1−6 mths post−tx
Pt 14
Pt 24
Pt 7

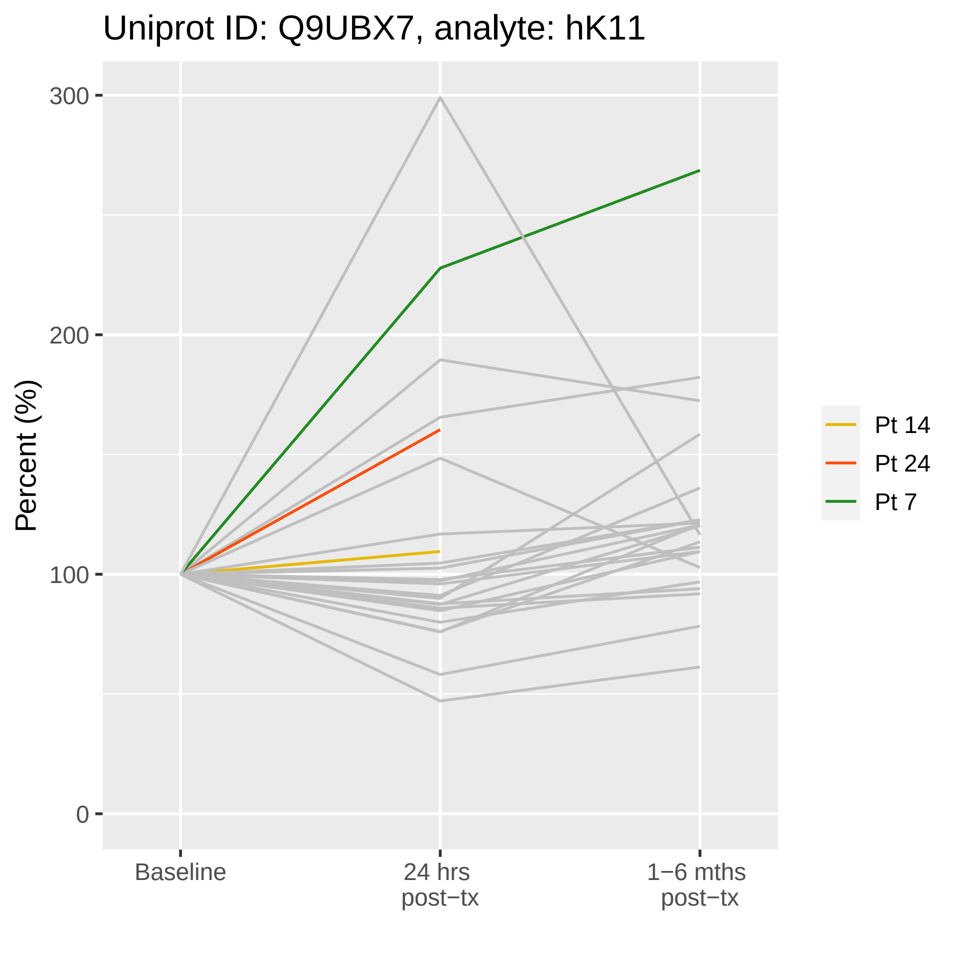

Uniprot ID: Q9UBX7, analyte: hK11
Percent (%)
300
200
100
0
Baseline
24 hrs
post−tx
1−6 mths
post−tx
Pt 14
Pt 24
Pt 7

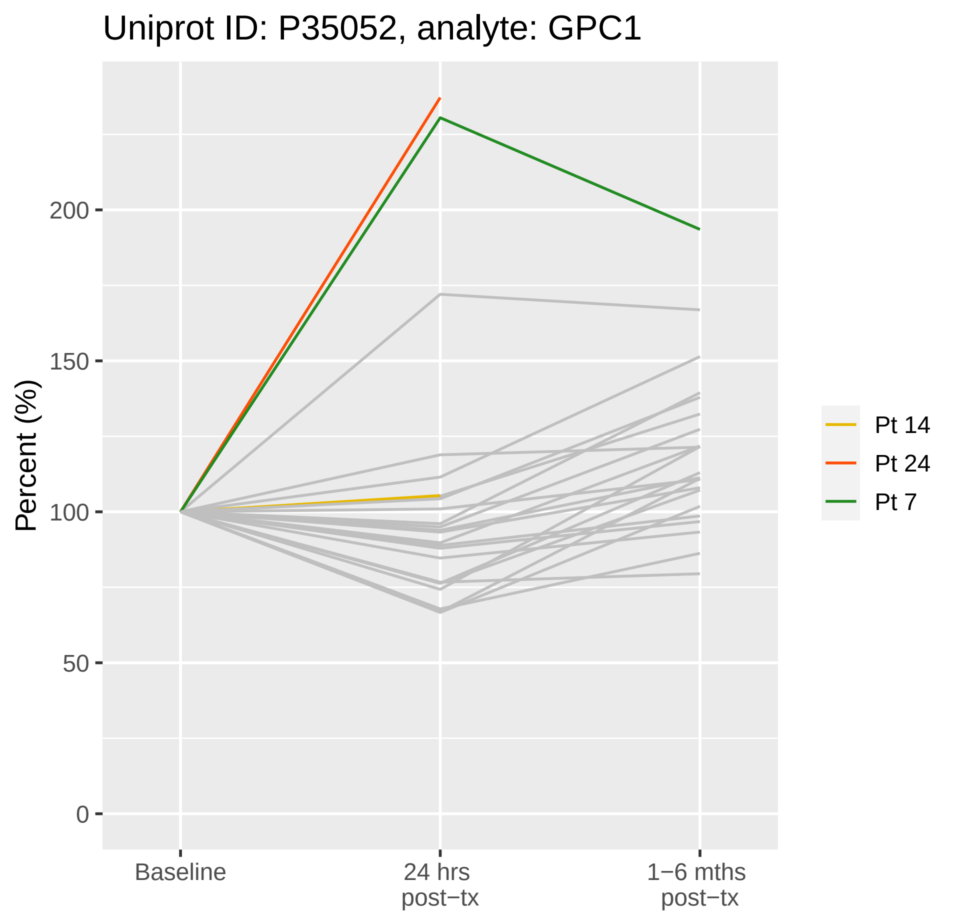
Uniprot ID: P35052, analyte: GPC1
Percent (%)
200
150
100
50
0
Baseline
24 hrs post−tx
1−6 mths post−tx
Pt 14
Pt 24
Pt 7

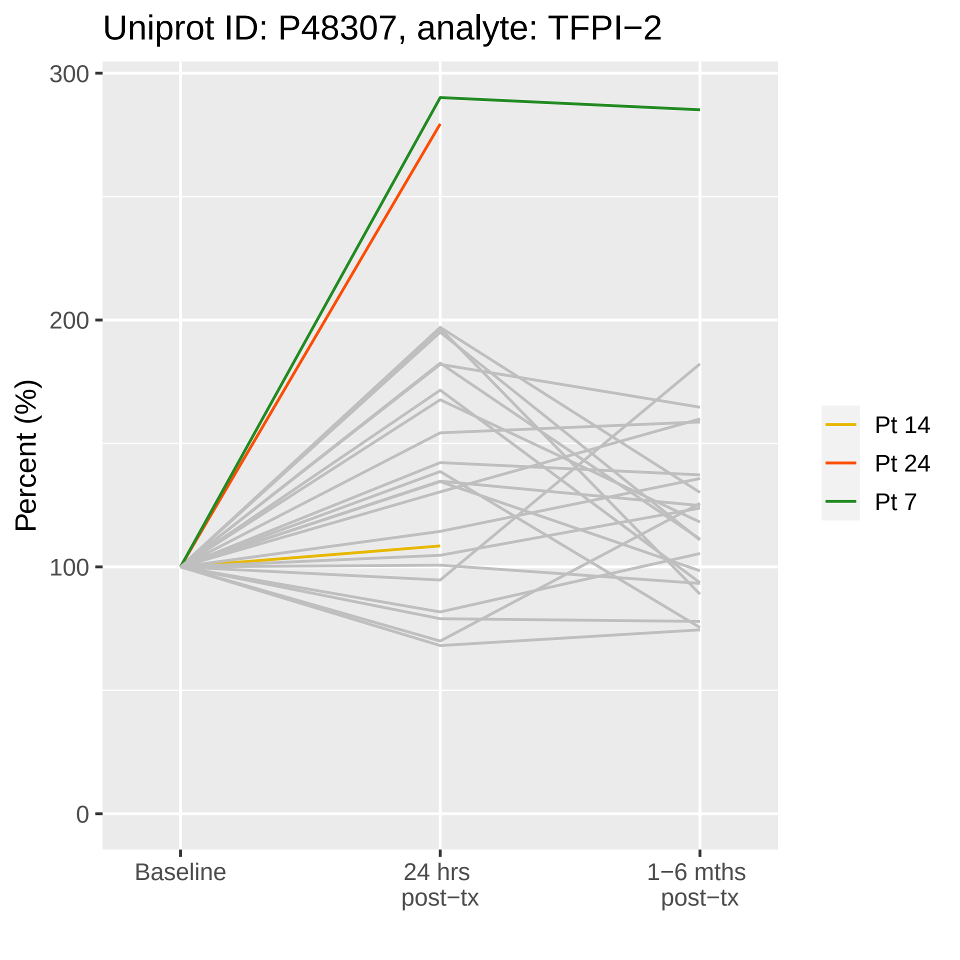

Uniprot ID: P48307, analyte: TFPI−2
Percent (%)
300
200
100
0
Baseline
24 hrs
post−tx
1−6 mths
post−tx
Pt 14
Pt 24
Pt 7

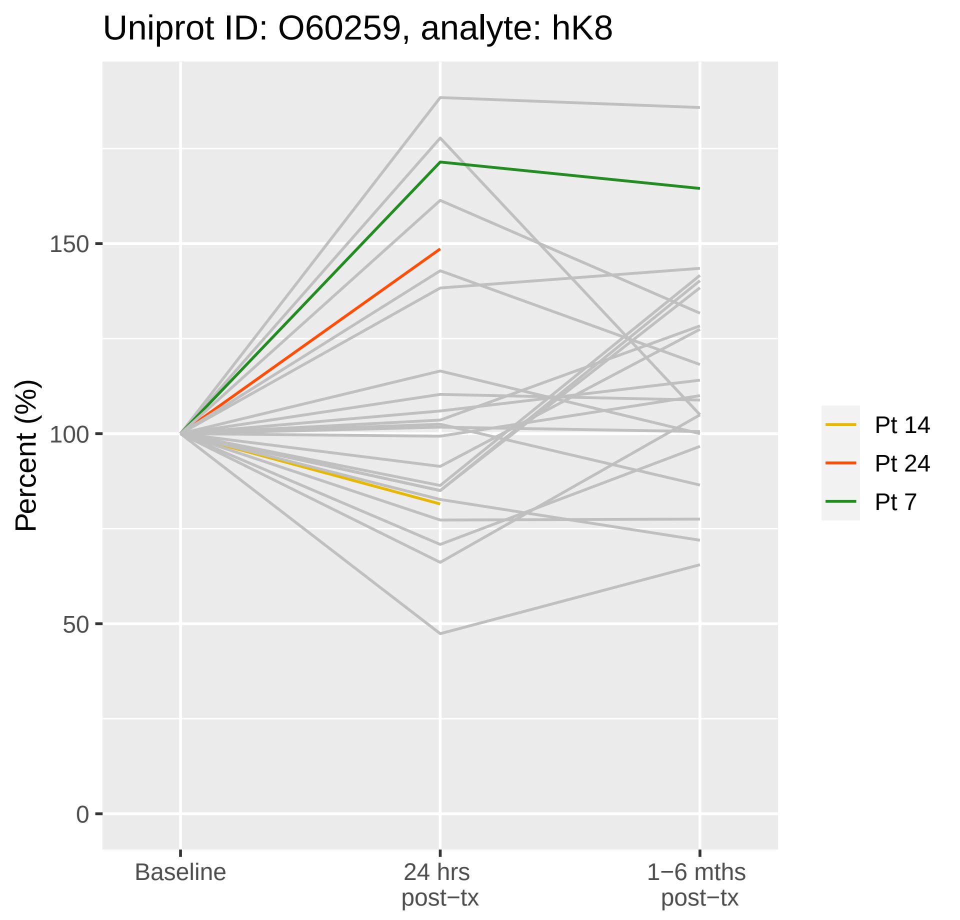
Uniprot ID: O60259, analyte: hK8
Percent (%)
150
100
50
0
Baseline
24 hrs post−tx
1−6 mths post−tx
Pt 14
Pt 24
Pt 7

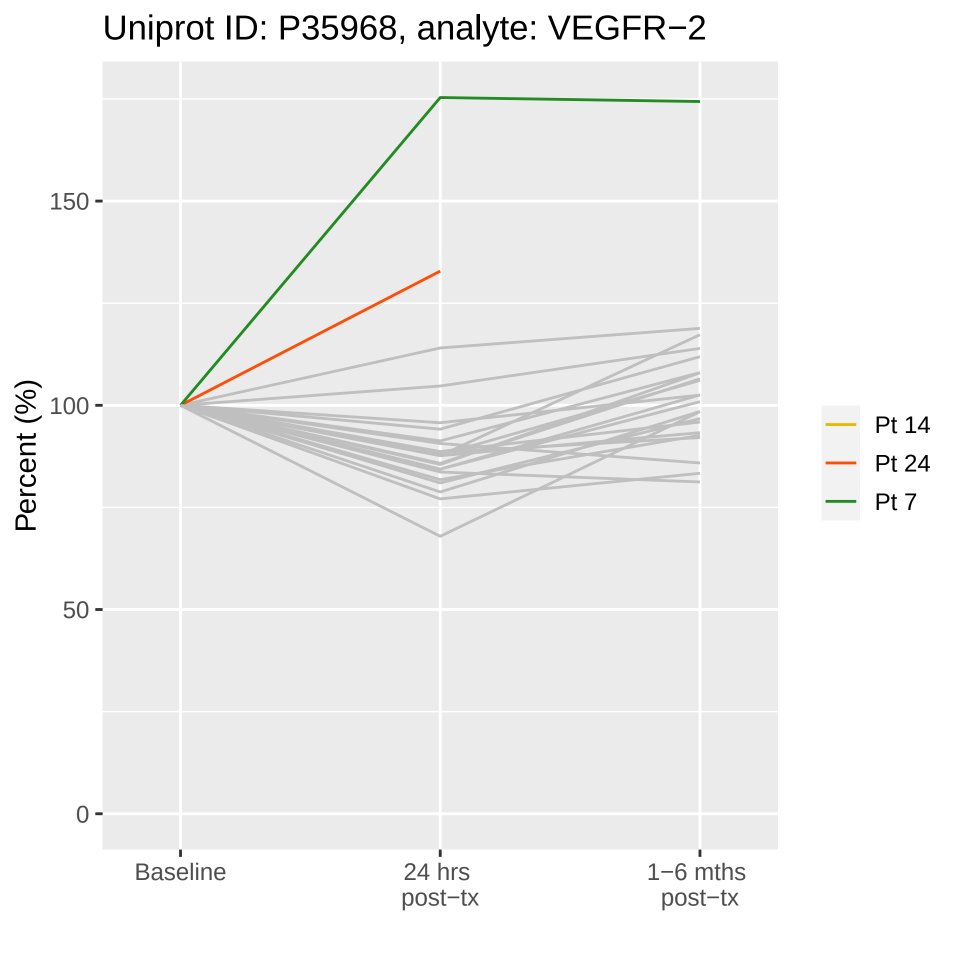

Uniprot ID: P35968, analyte: VEGFR−2
Percent (%)
150
100
50
0
Baseline
24 hrs post−tx
1−6 mths post−tx
Pt 14
Pt 24
Pt 7

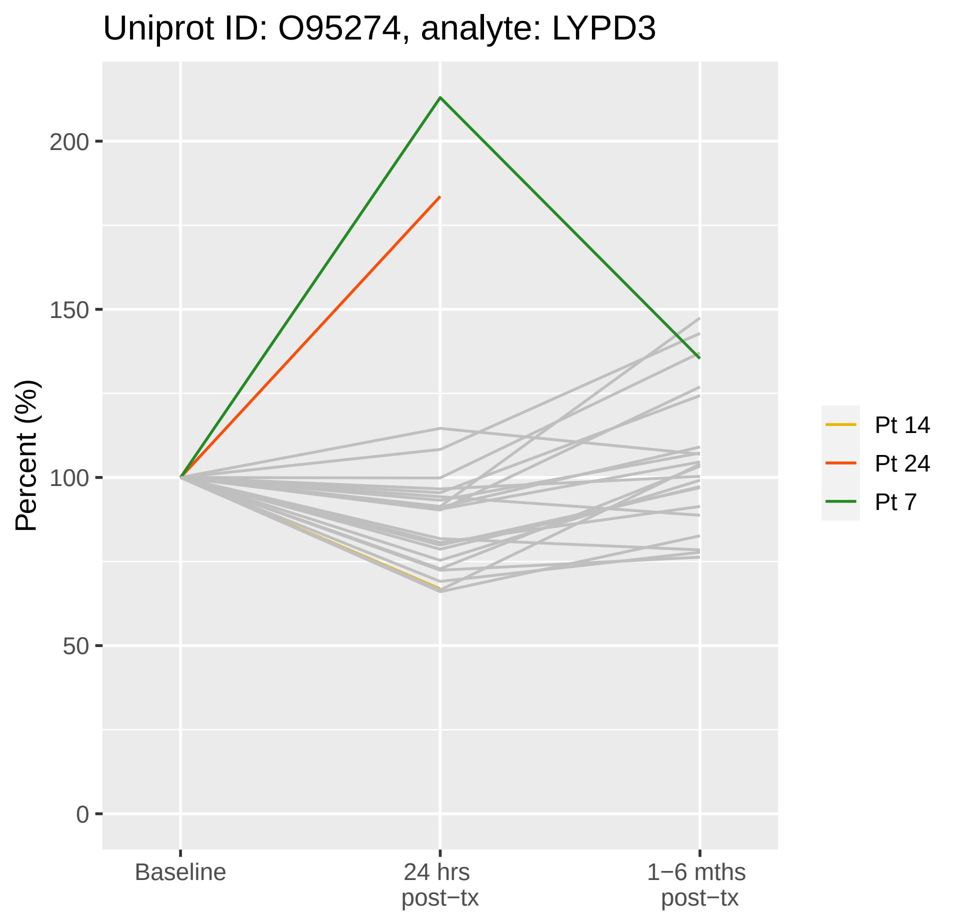
Uniprot ID: O95274, analyte: LYPD3
Percent (%)
200
150
100
50
0
Baseline
24 hrs post−tx
1−6 mths post−tx
Pt 14
Pt 24
Pt 7

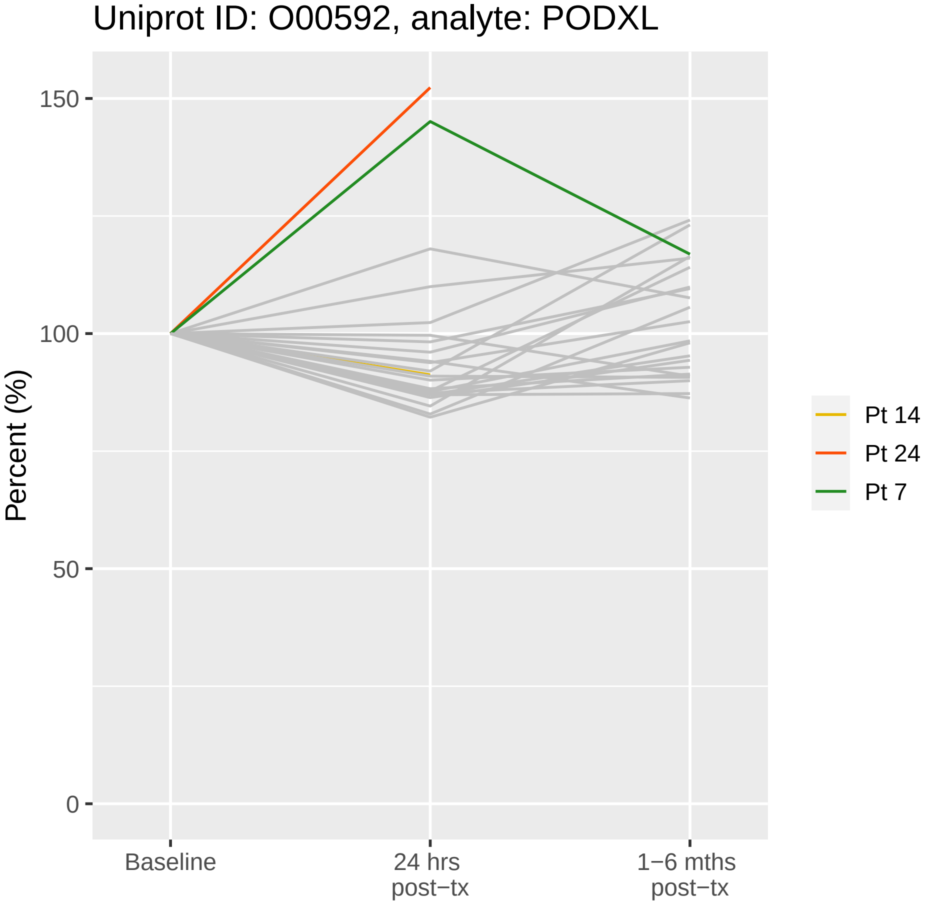
Uniprot ID: O00592, analyte: PODXL
Percent (%)
150
100
50
0
Baseline
24 hrs
post−tx
1−6 mths
post−tx
Pt 14
Pt 24
Pt 7

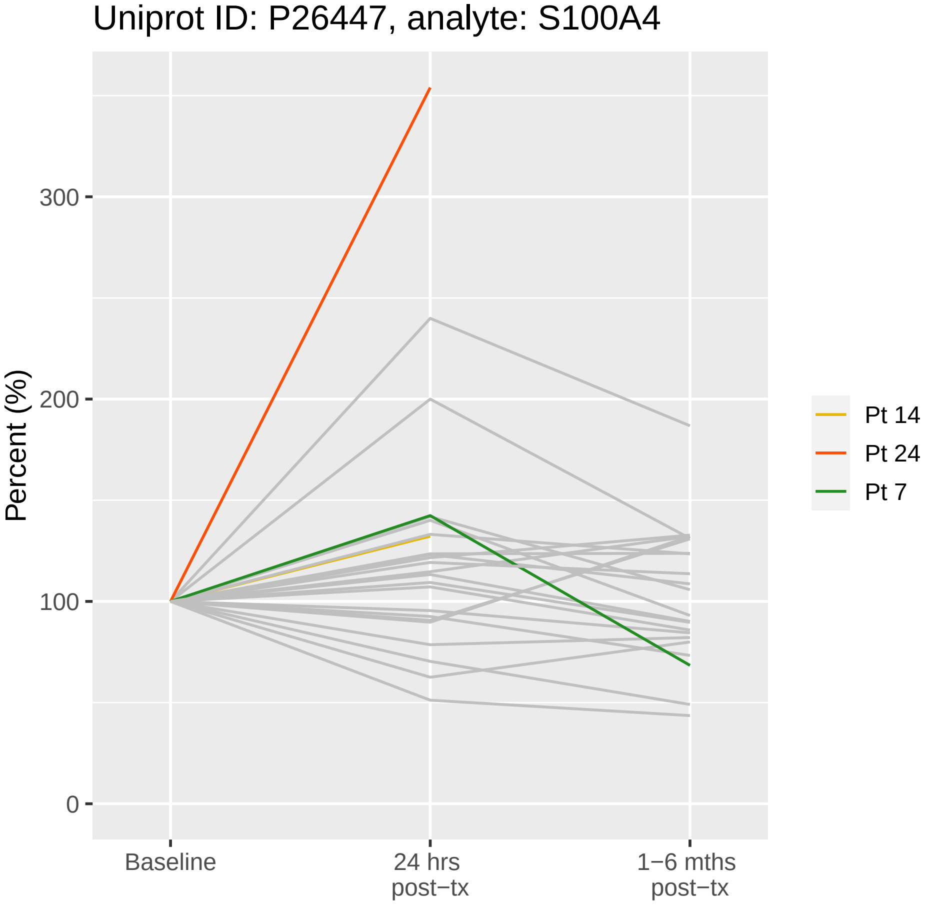

Uniprot ID: P26447, analyte: S100A4
Percent (%)
300
200
100
0
Baseline
24 hrs post−tx
1−6 mths post−tx
Pt 14
Pt 24
Pt 7

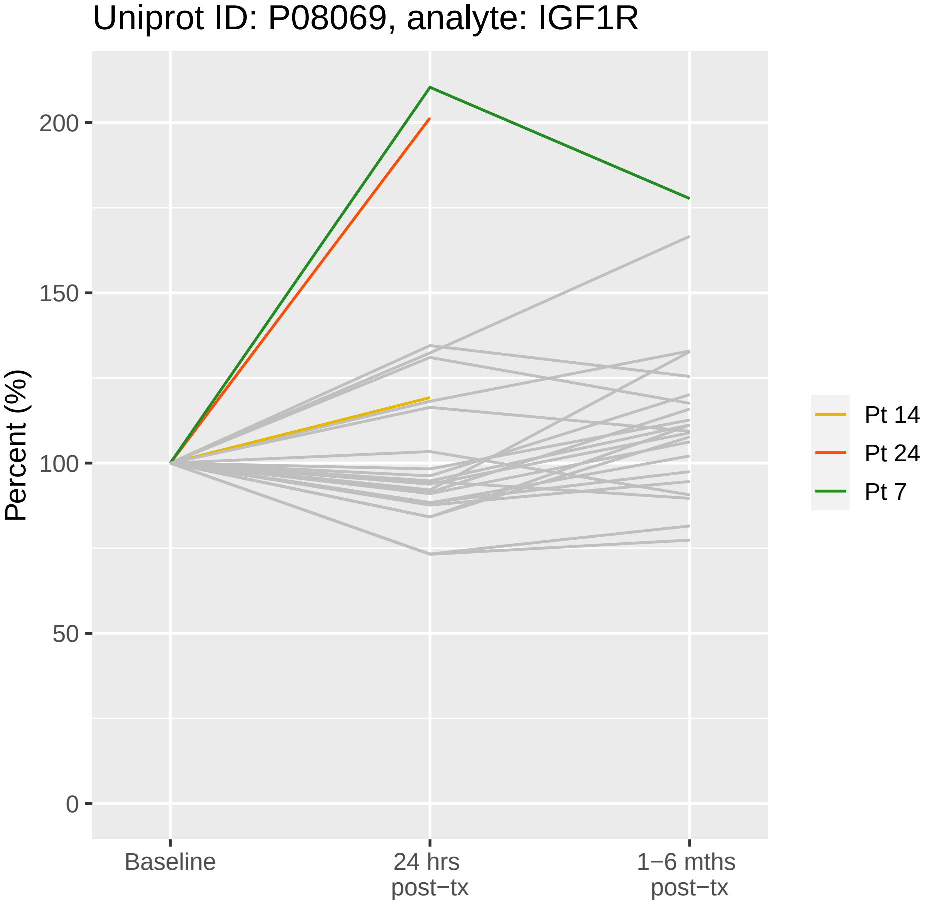
Uniprot ID: P08069, analyte: IGF1R
Percent (%)
0
50
100
150
200
Baseline
24 hrs
post−tx
1−6 mths
post−tx
Pt 14
Pt 24
Pt 7

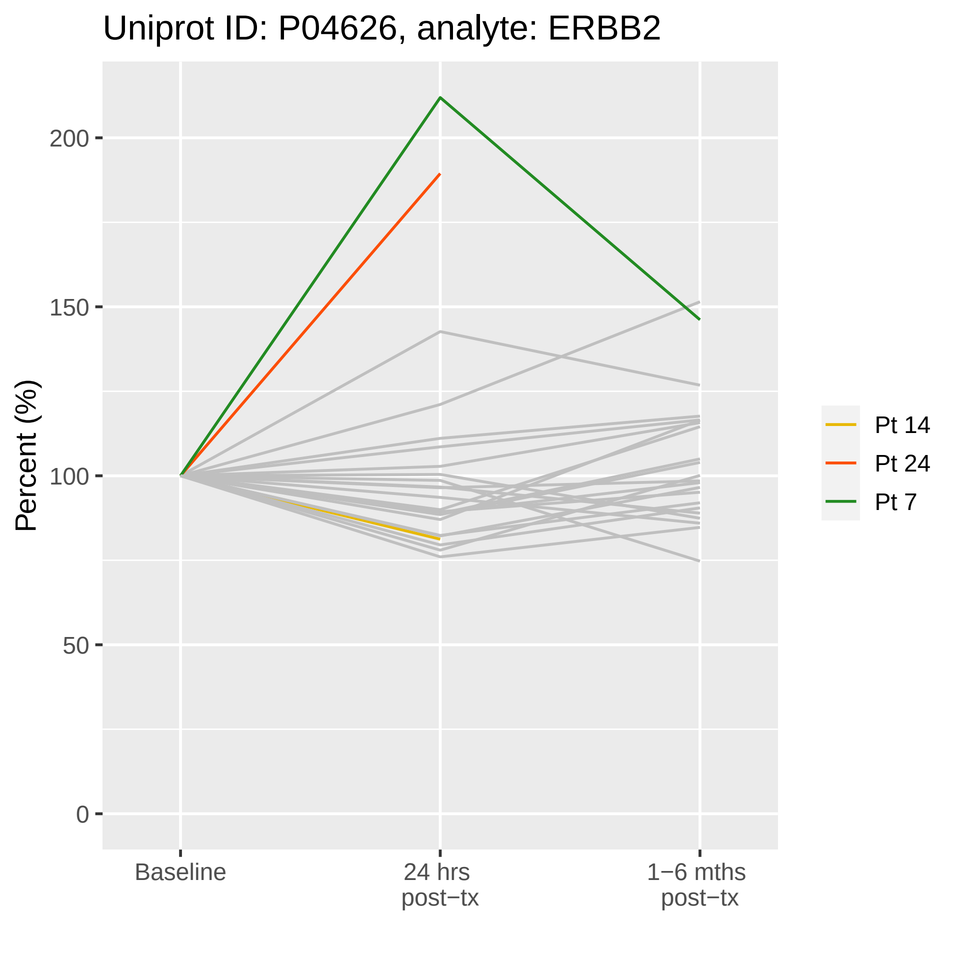
Uniprot ID: P04626, analyte: ERBB2
Percent (%)
200
150
100
50
0
Baseline
24 hrs post−tx
1−6 mths post−tx
Pt 14
Pt 24
Pt 7

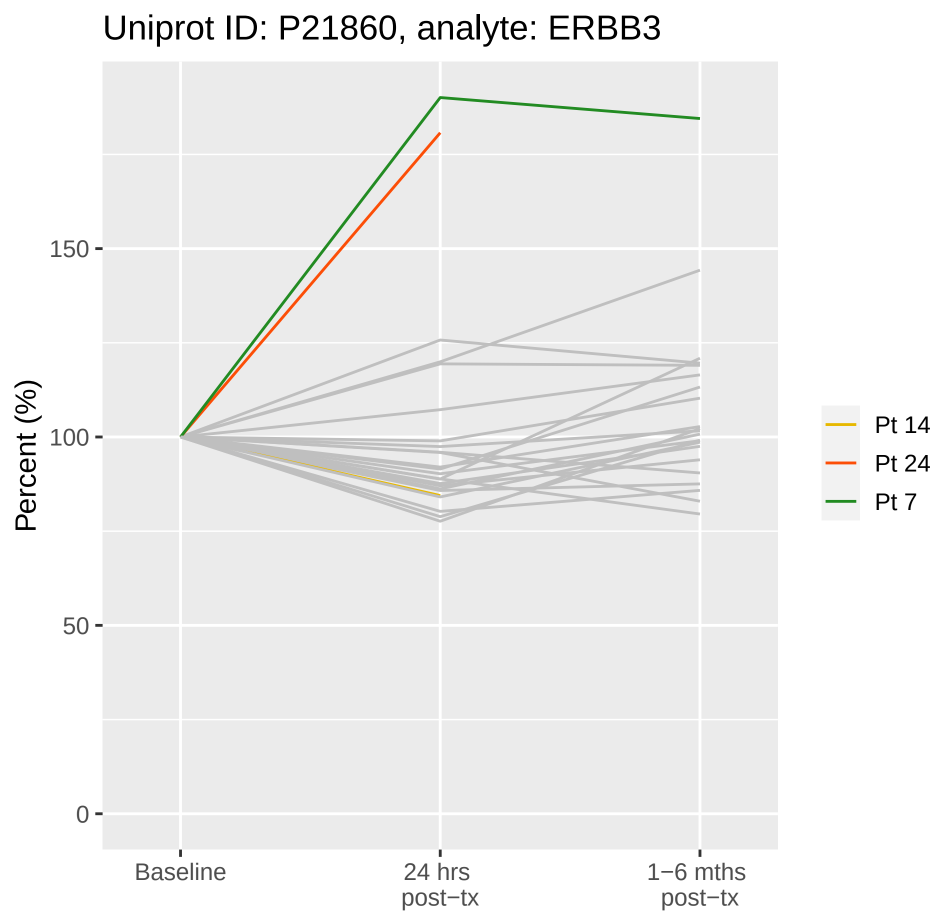
Uniprot ID: P21860, analyte: ERBB3
Percent (%)
150
100
50
0
Baseline
24 hrs
post−tx
1−6 mths
post−tx
Pt 14
Pt 24
Pt 7

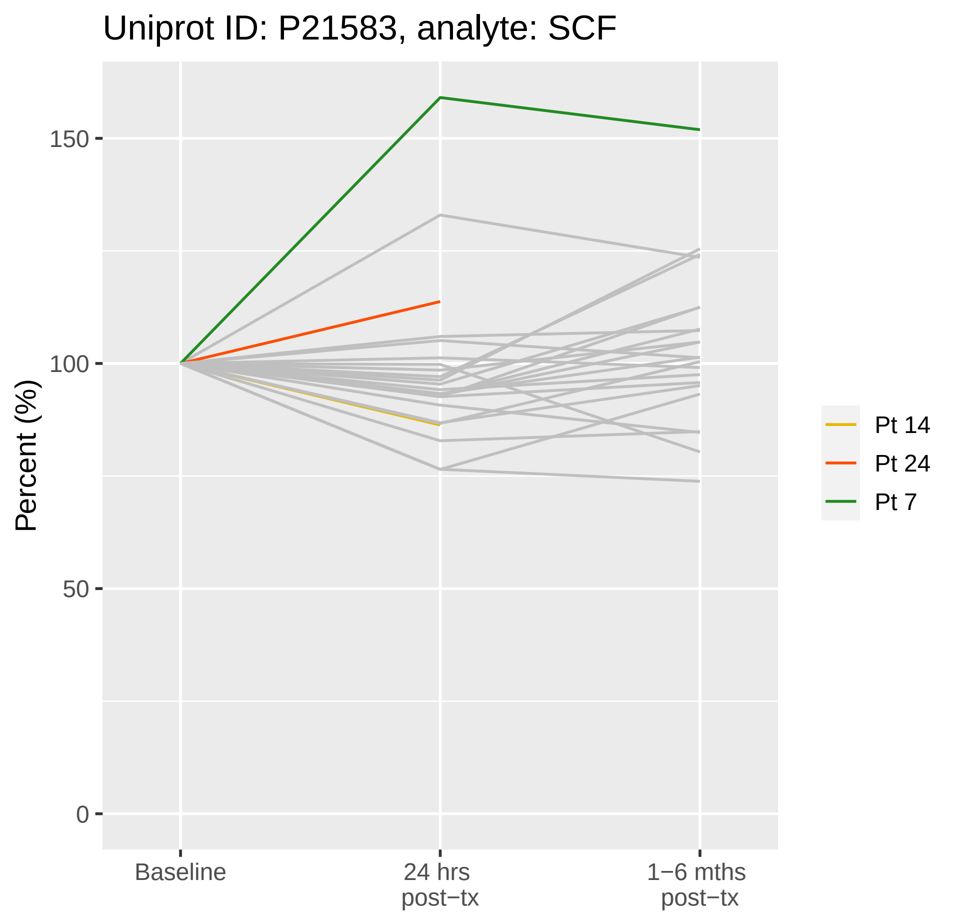
Uniprot ID: P21583, analyte: SCF
Percent (%)
150
100
50
0
Baseline
24 hrs
post−tx
1−6 mths
post−tx
Pt 14
Pt 24
Pt 7

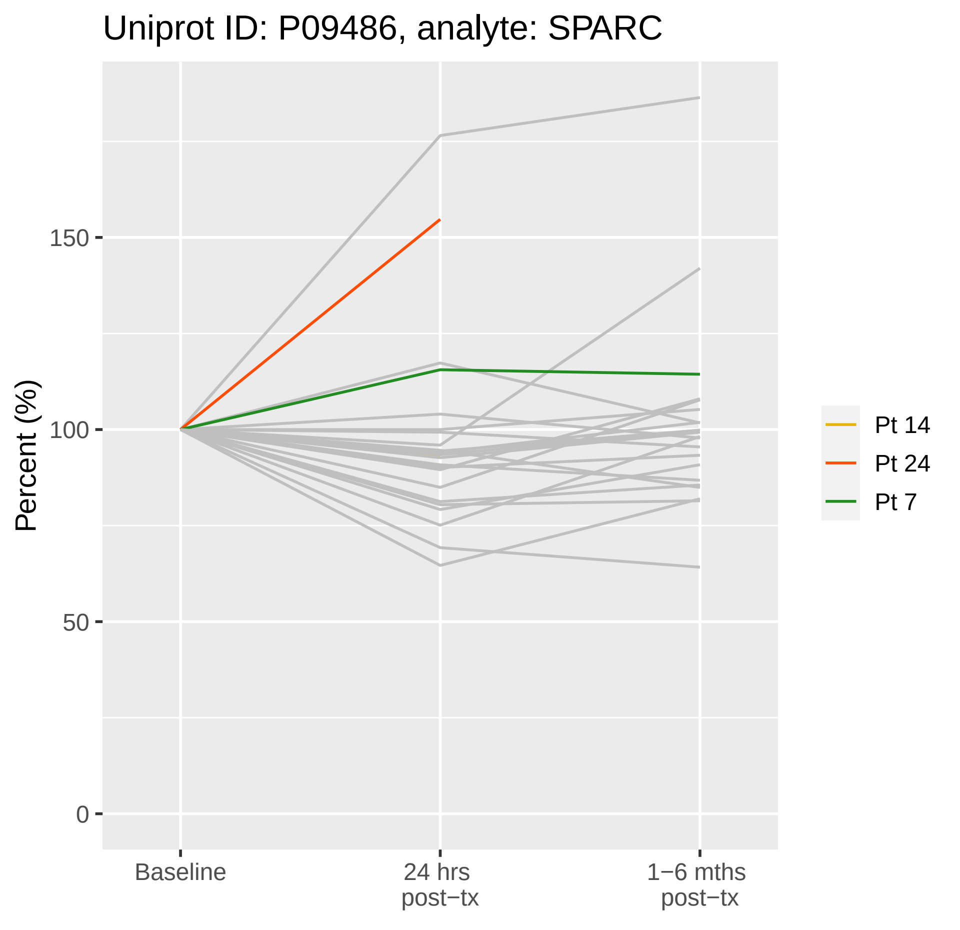

Uniprot ID: P09486, analyte: SPARC
Percent (%)
150
100
50
0
Baseline
24 hrs
post−tx
1−6 mths
post−tx
Pt 14
Pt 24
Pt 7

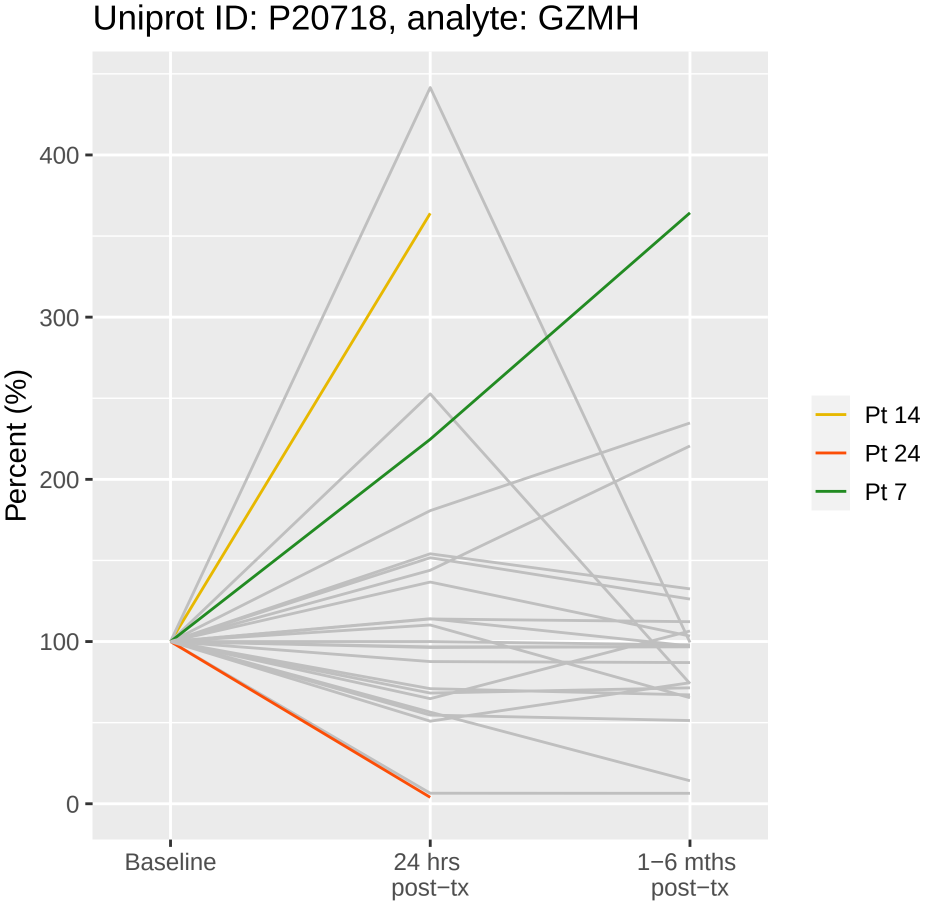
Uniprot ID: P20718, analyte: GZMH
Percent (%)
400
300
200
100
0
Baseline
24 hrs post−tx
1−6 mths post−tx
Pt 14
Pt 24
Pt 7

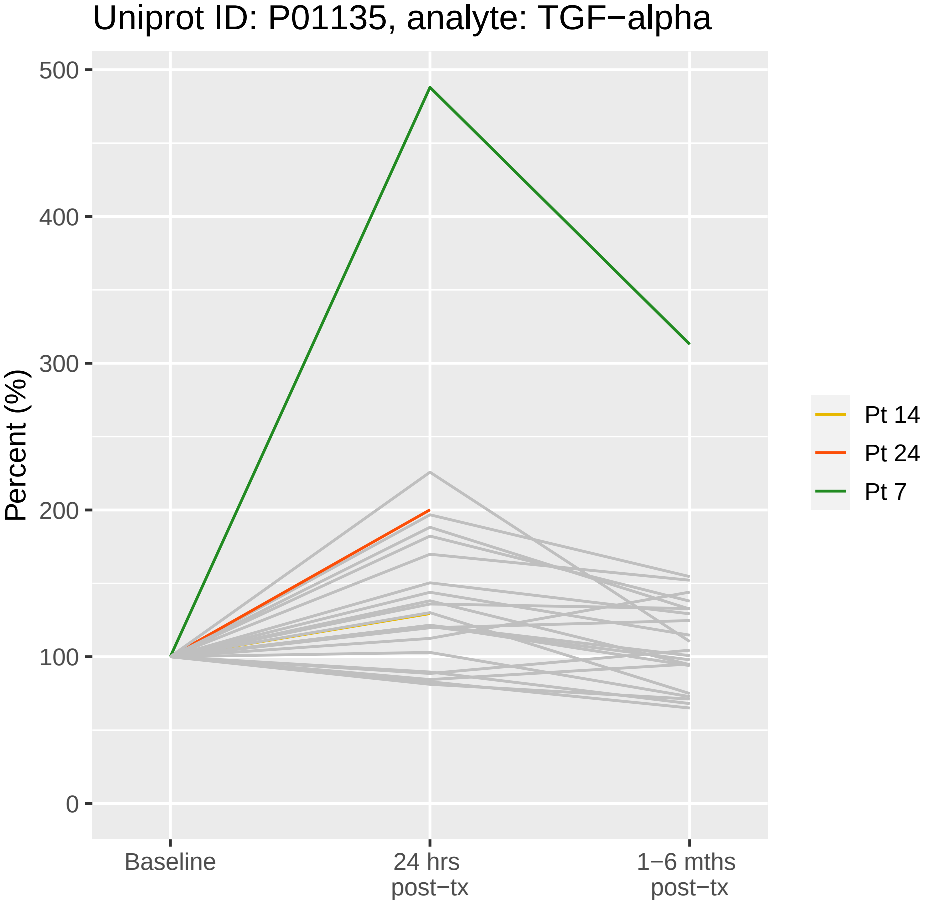
Uniprot ID: P01135, analyte: TGF−alpha
Percent (%)
500
400
300
200
100
0
Baseline
24 hrs post−tx
1−6 mths post−tx
Pt 14
Pt 24
Pt 7

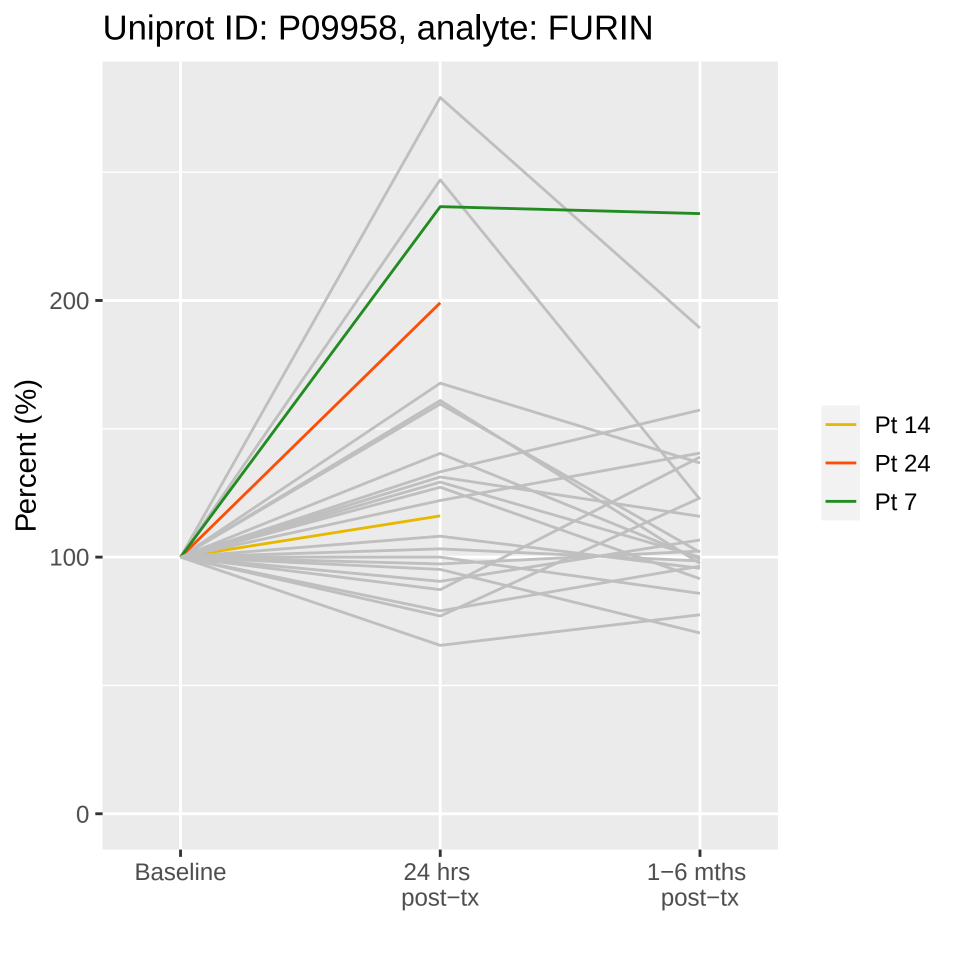

Uniprot ID: P09958, analyte: FURIN
Percent (%)
200
100
0
Baseline
24 hrs
post−tx
1−6 mths
post−tx
Pt 14
Pt 24
Pt 7

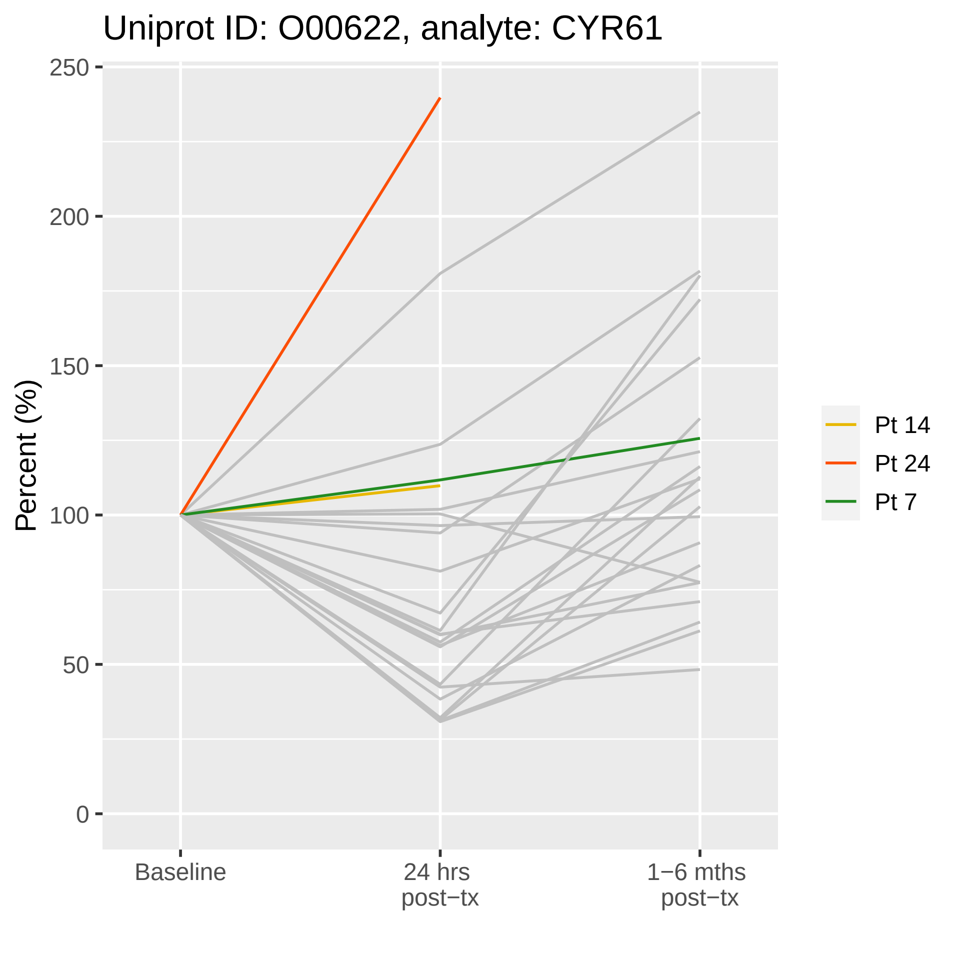
Uniprot ID: O00622, analyte: CYR61
Percent (%)
250
200
150
100
50
0
Baseline
24 hrs
post−tx
1−6 mths
post−tx
Pt 14
Pt 24
Pt 7

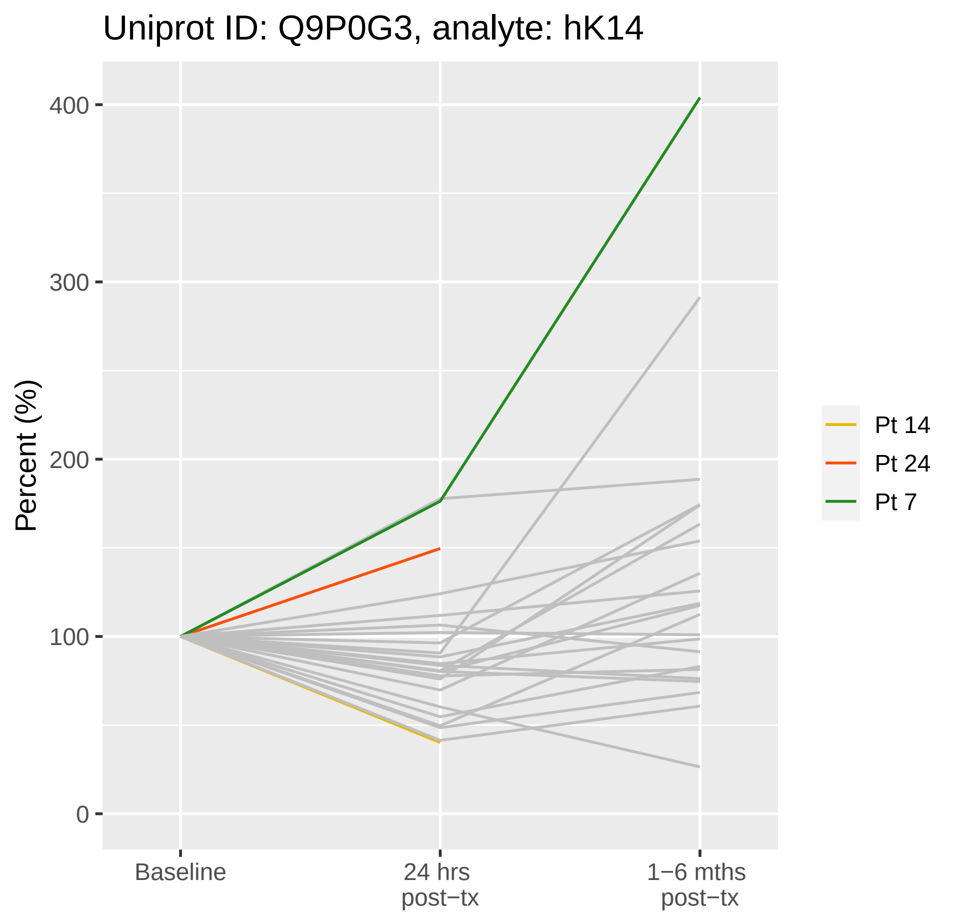
Uniprot ID: Q9P0G3, analyte: hK14
Percent (%)
400
300
200
100
0
Baseline
24 hrs post−tx
1−6 mths post−tx
Pt 14
Pt 24
Pt 7

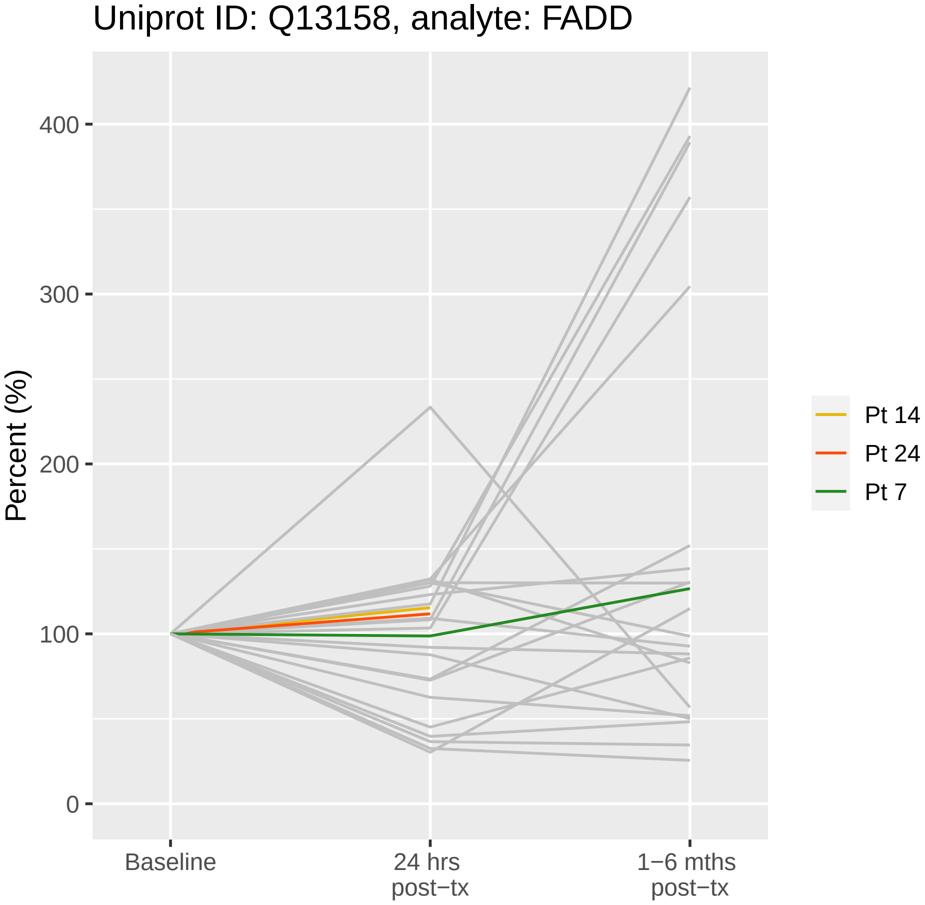

Uniprot ID: Q13158, analyte: FADD
Percent (%)
400
300
200
100
0
Baseline
24 hrs post−tx
1−6 mths post−tx
Pt 14
Pt 24
Pt 7

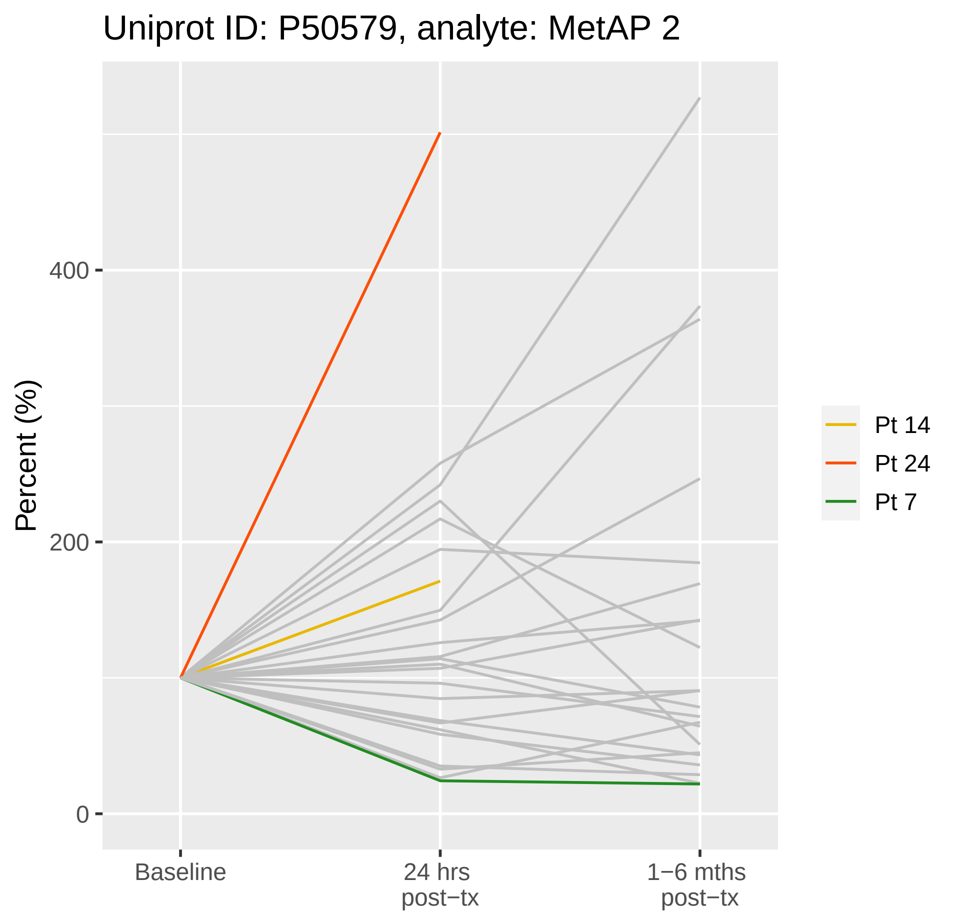
Uniprot ID: P50579, analyte: MetAP 2
Percent (%)
400
200
0
Baseline
24 hrs post−tx
1−6 mths post−tx
Pt 14
Pt 24
Pt 7

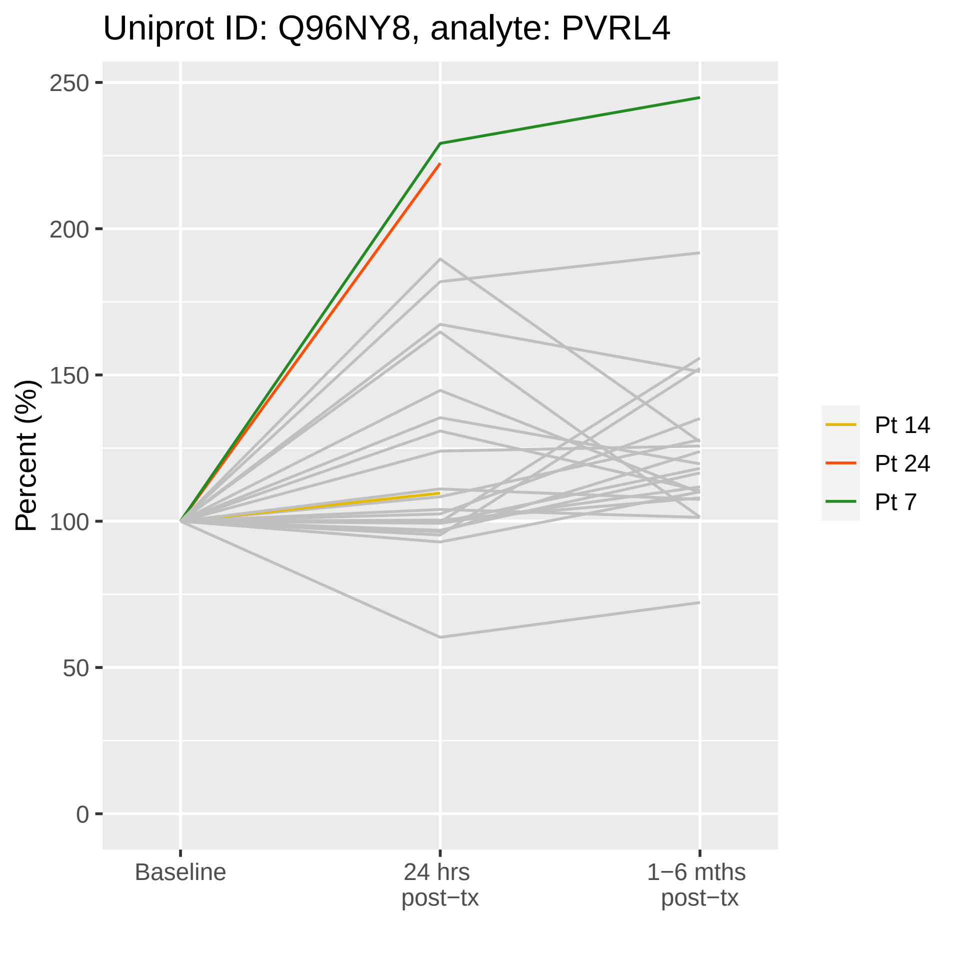

Uniprot ID: Q96NY8, analyte: PVRL4
Percent (%)
250
200
150
100
50
0
Baseline
24 hrs post−tx
1−6 mths post−tx
Pt 14
Pt 24
Pt 7

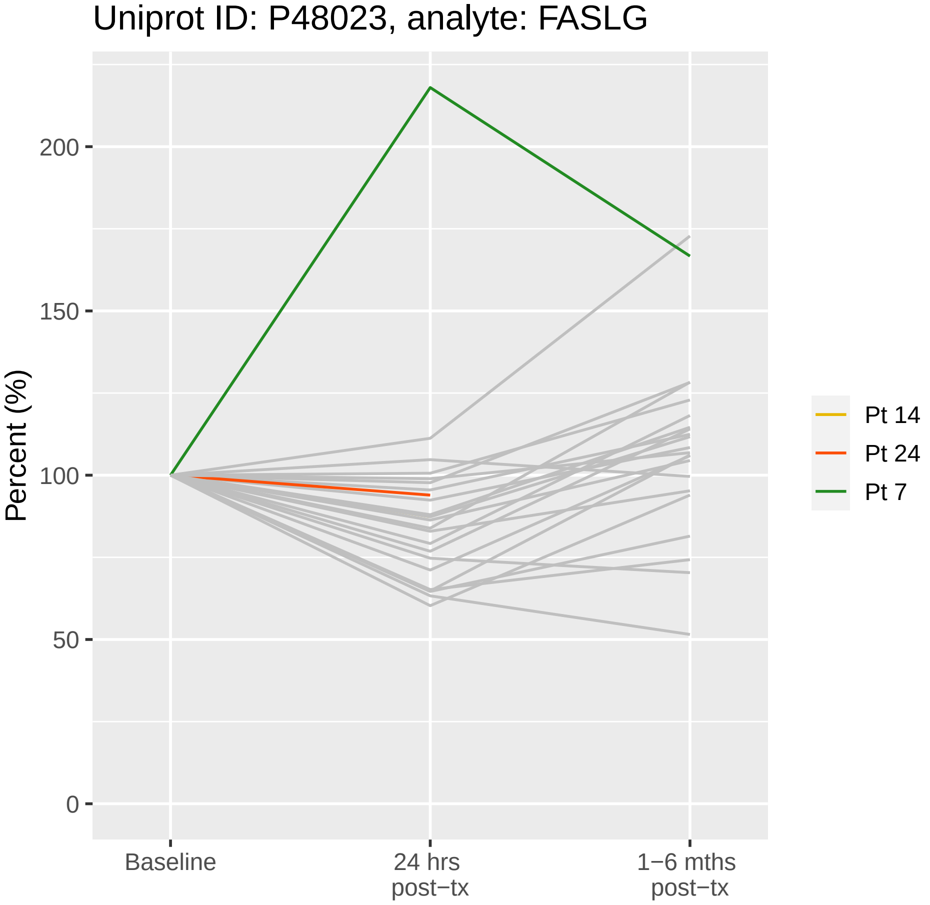
Uniprot ID: P48023, analyte: FASLG
Percent (%)
200
150
100
50
0
Baseline
24 hrs post−tx
1−6 mths post−tx
Pt 14
Pt 24
Pt 7

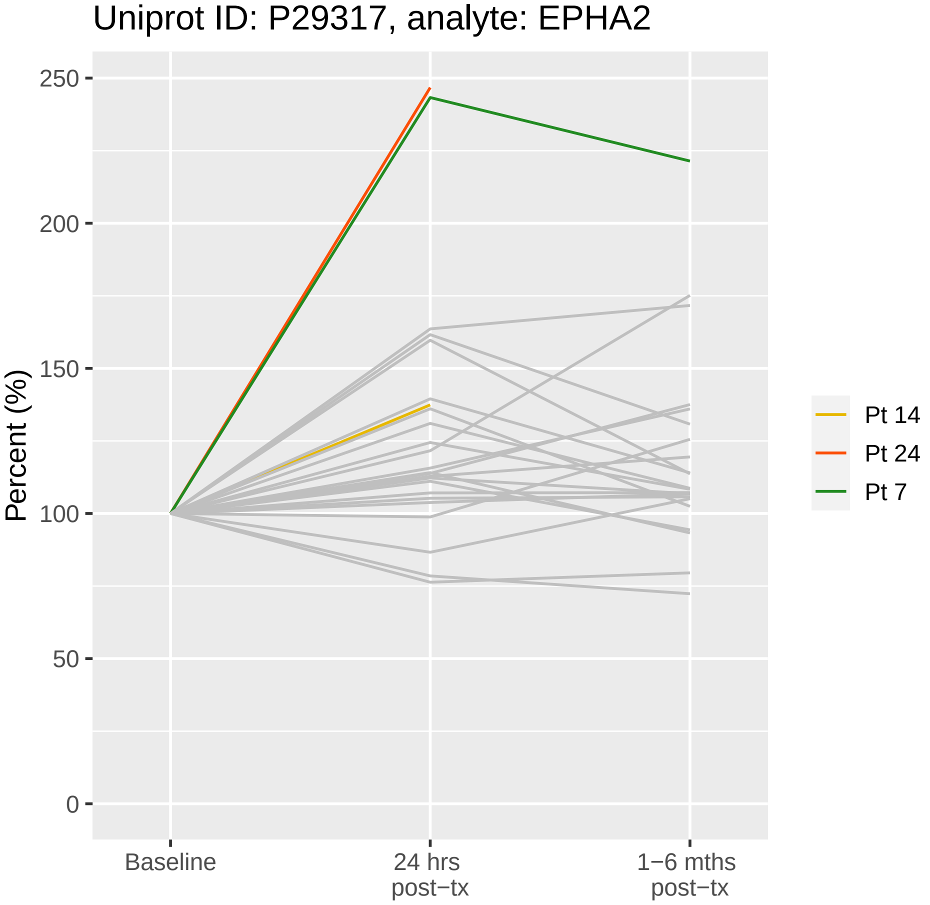
Uniprot ID: P29317, analyte: EPHA2
Percent (%)
250
200
150
100
50
0
Baseline
24 hrs post−tx
1−6 mths post−tx
Pt 14
Pt 24
Pt 7

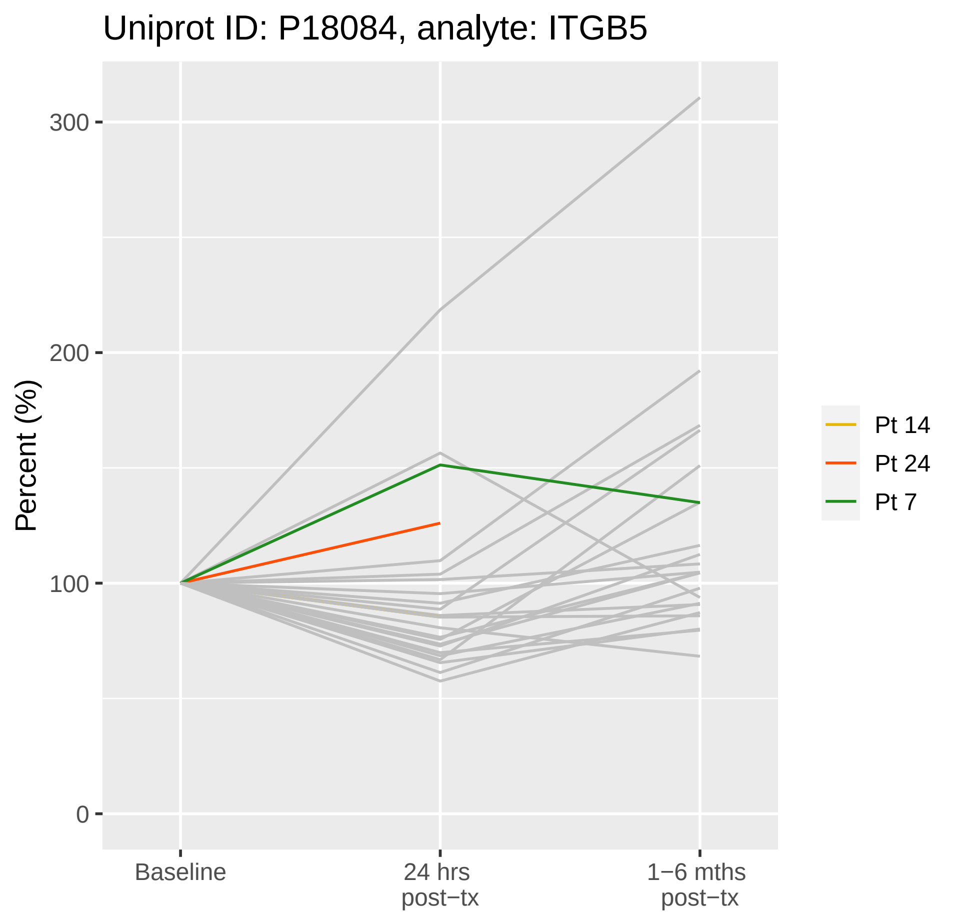

Uniprot ID: P18084, analyte: ITGB5
Percent (%)
300
200
100
0
Baseline
24 hrs
post−tx
1−6 mths
post−tx
Pt 14
Pt 24
Pt 7

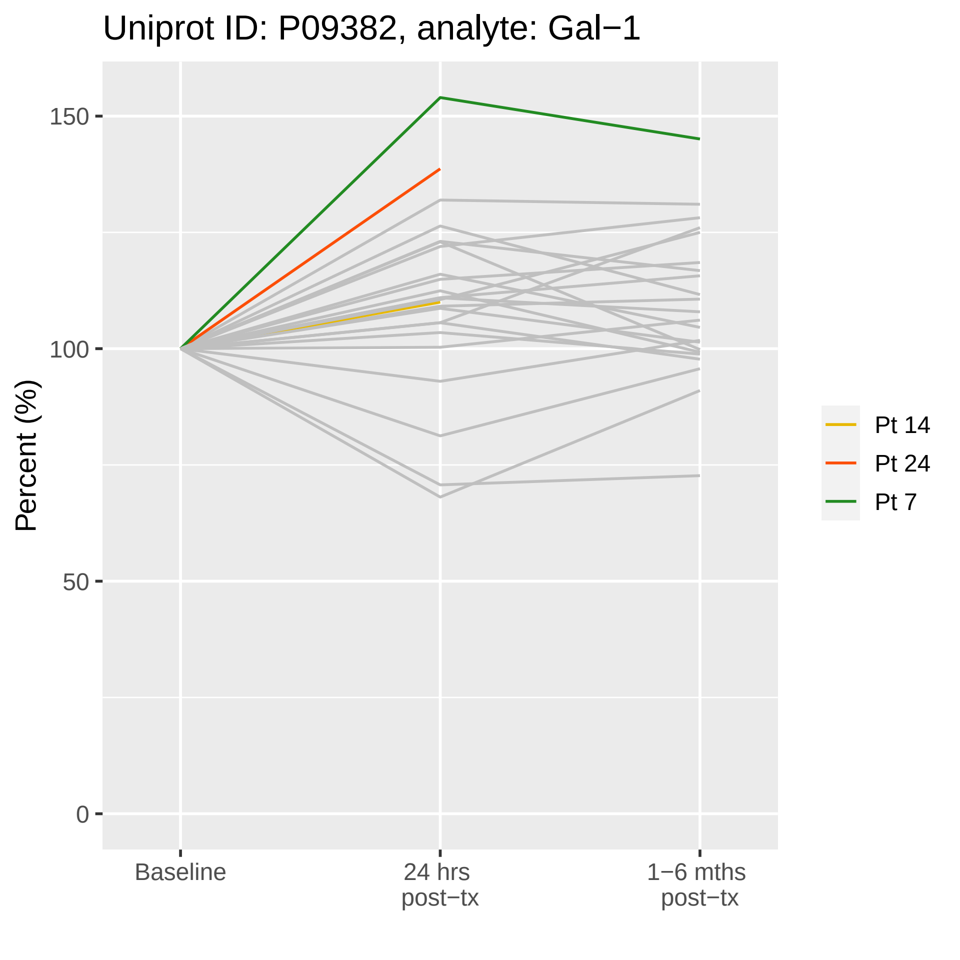
Uniprot ID: P09382, analyte: Gal−1
Percent (%)
150
100
50
0
Baseline
24 hrs post−tx
1−6 mths post−tx
Pt 14
Pt 24
Pt 7

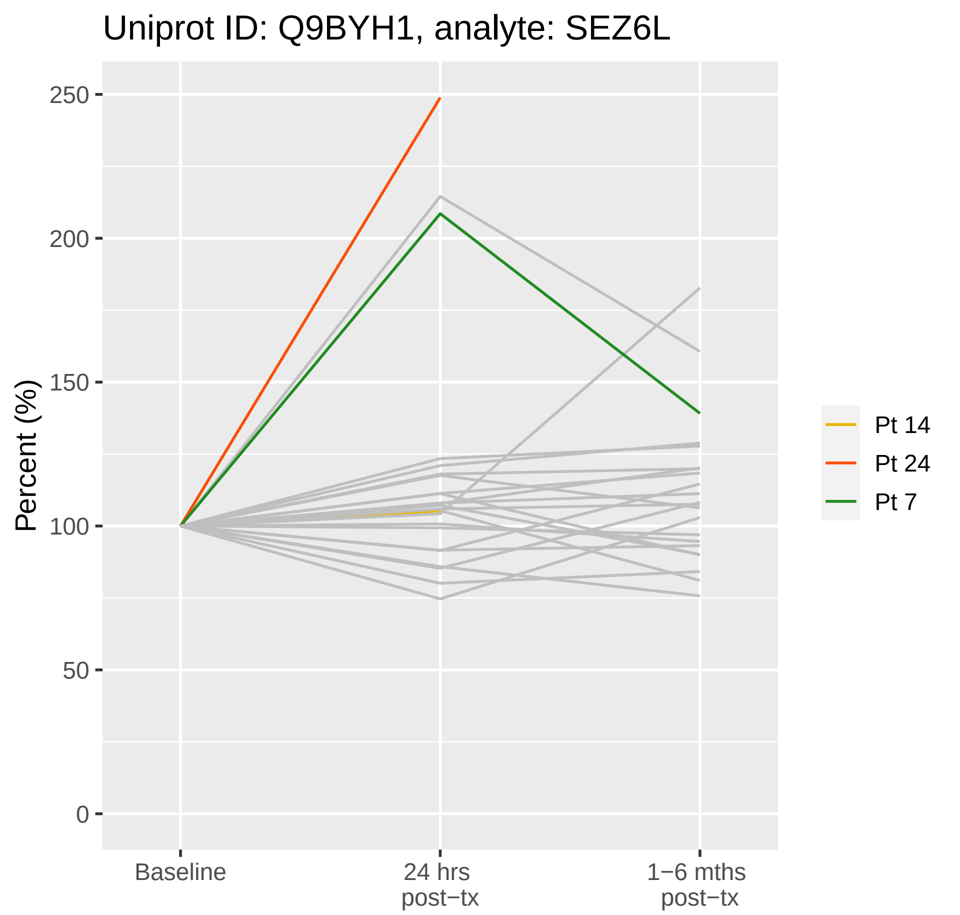
Uniprot ID: Q9BYH1, analyte: SEZ6L
Percent (%)
250
200
150
100
50
0
Baseline
24 hrs
post−tx
1−6 mths
post−tx
Pt 14
Pt 24
Pt 7

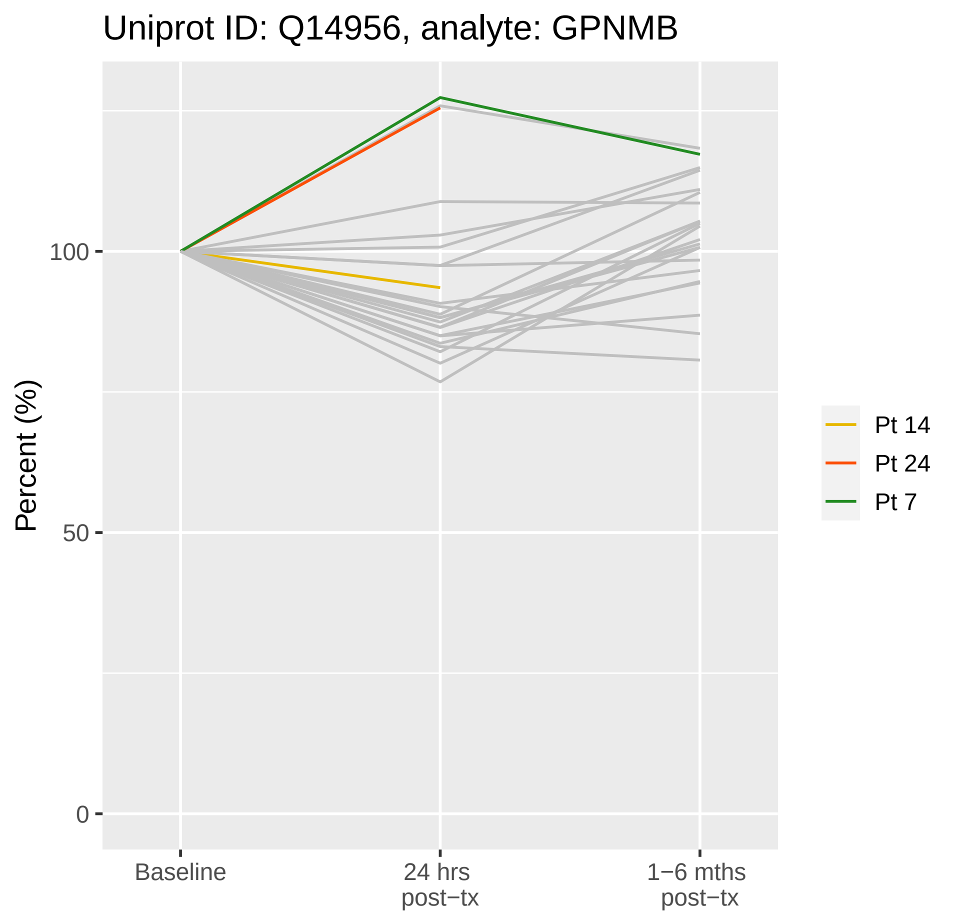

Uniprot ID: Q14956, analyte: GPNMB
Percent (%)
100
50
0
Baseline
24 hrs
post−tx
1−6 mths
post−tx
Pt 14
Pt 24
Pt 7

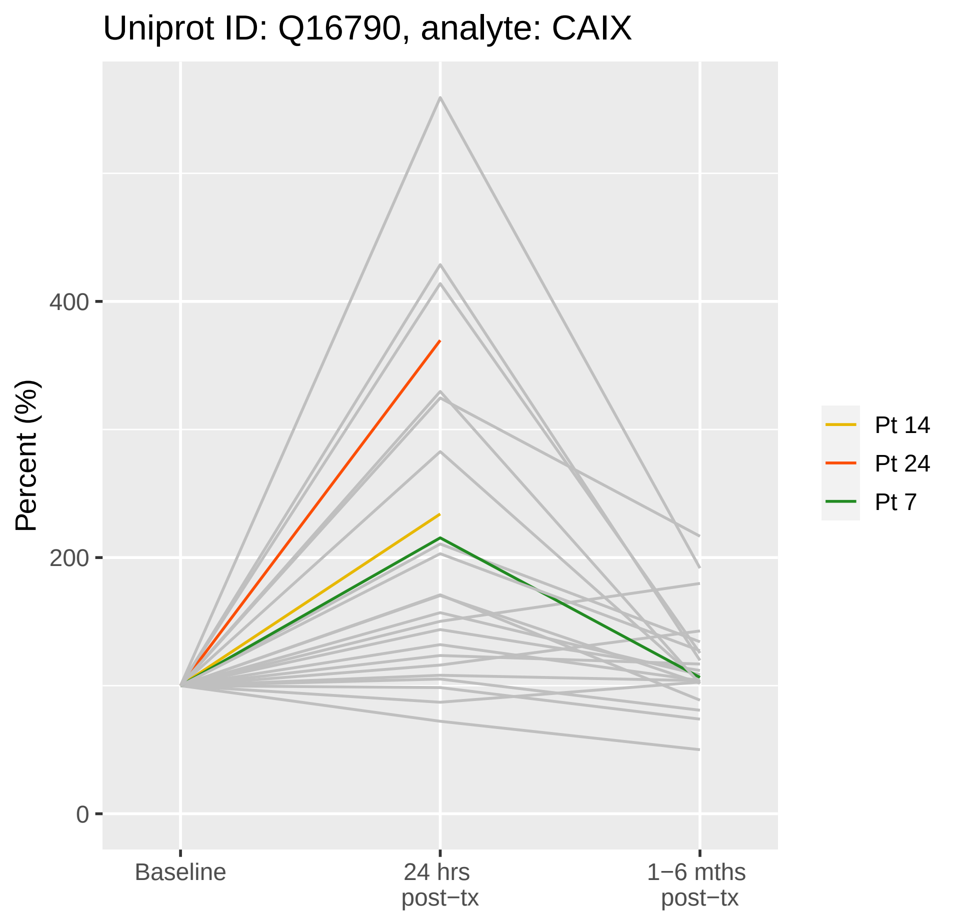
Uniprot ID: Q16790, analyte: CAIX
Percent (%)
400
200
0
Baseline
24 hrs
post−tx
1−6 mths
post−tx
Pt 14
Pt 24
Pt 7

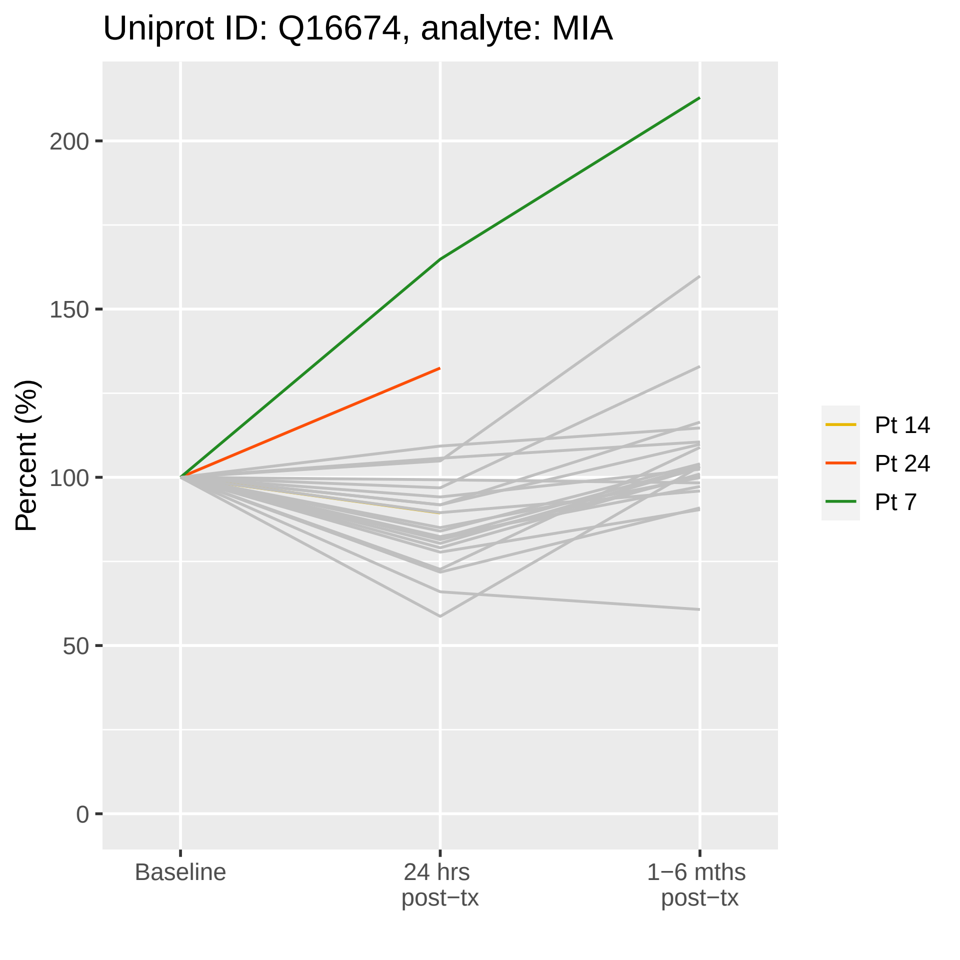
Uniprot ID: Q16674, analyte: MIA
Percent (%)
0
50
100
150
200
Baseline
24 hrs post−tx
1−6 mths post−tx
Pt 14
Pt 24
Pt 7

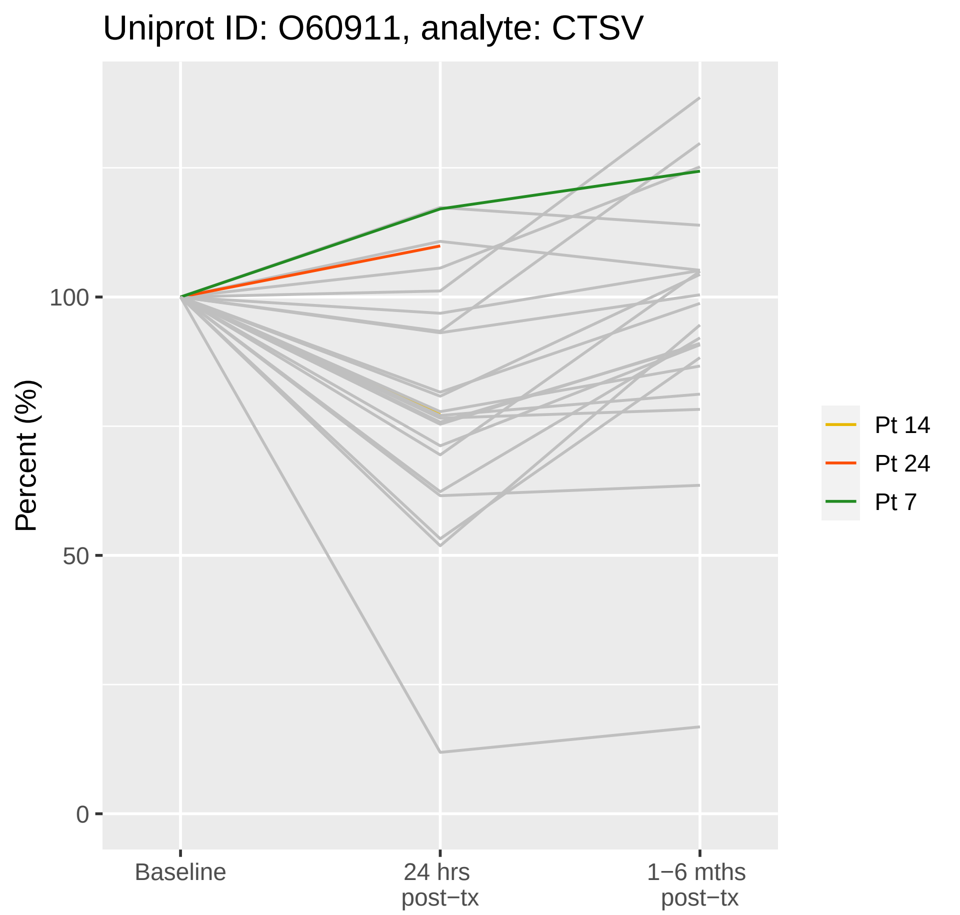

Uniprot ID: O60911, analyte: CTSV
Percent (%)
100
50
0
Baseline
24 hrs post−tx
1−6 mths post−tx
Pt 14
Pt 24
Pt 7

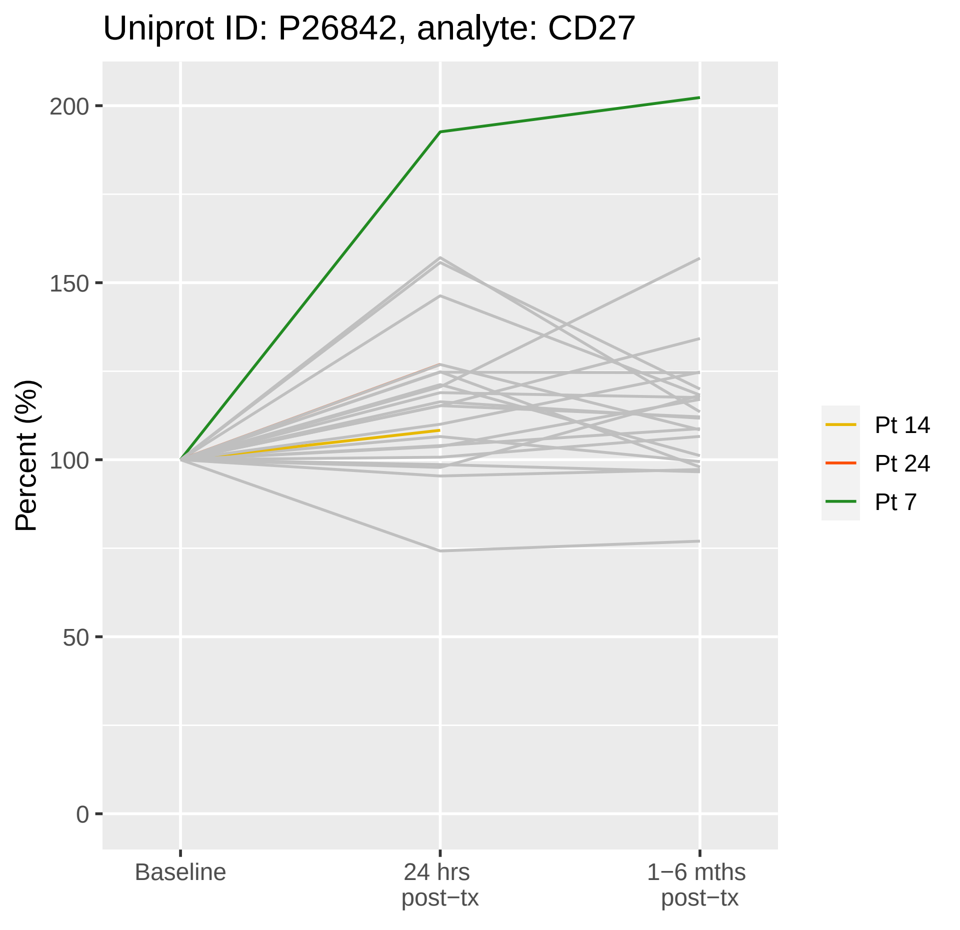

Uniprot ID: P26842, analyte: CD27
Percent (%)
200
150
100
50
0
Baseline
24 hrs
post−tx
1−6 mths
post−tx
Pt 14
Pt 24
Pt 7

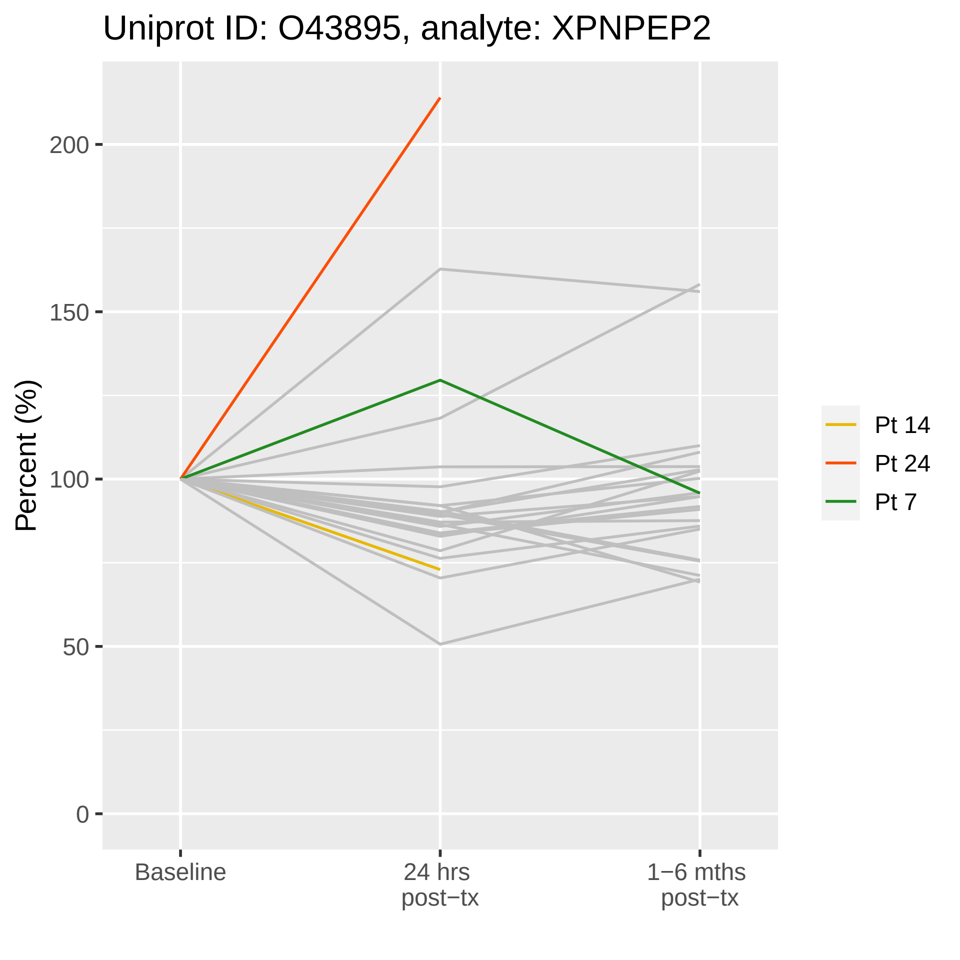
Uniprot ID: O43895, analyte: XPNPEP2
Percent (%)
0
50
100
150
200
Baseline
24 hrs post−tx
1−6 mths post−tx
Pt 14
Pt 24
Pt 7

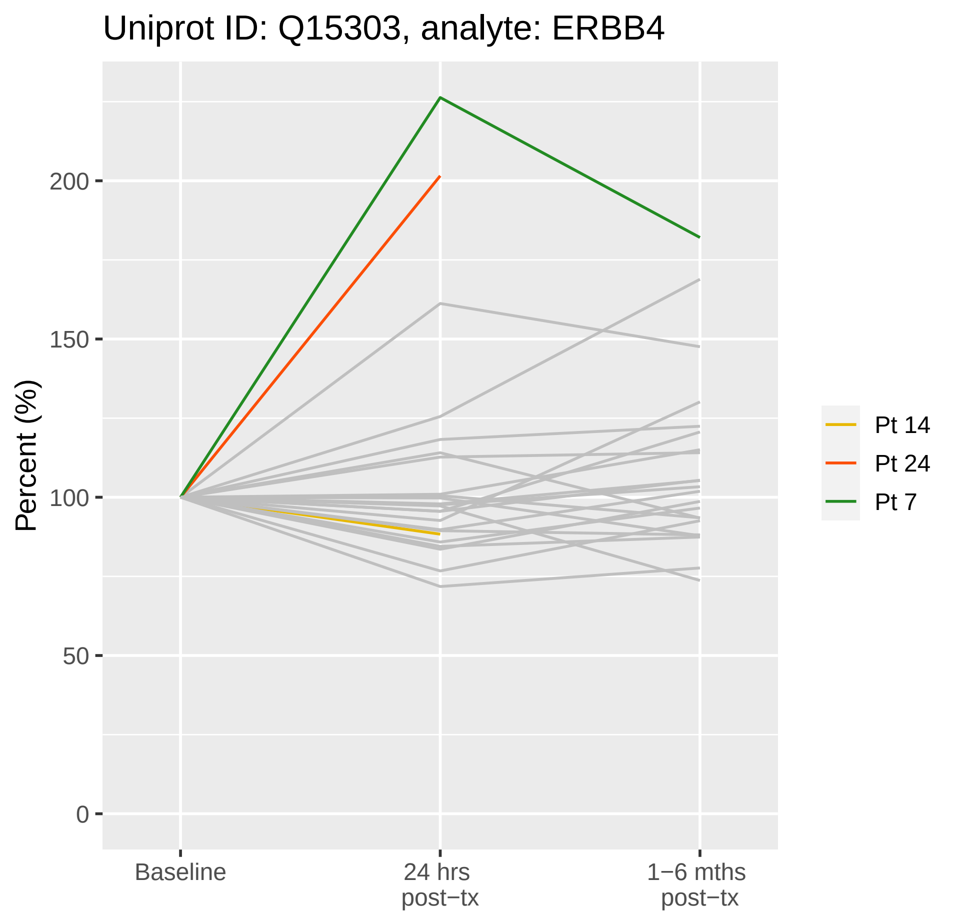
Uniprot ID: Q15303, analyte: ERBB4
Percent (%)
200
150
100
50
0
Baseline
24 hrs post−tx
1−6 mths post−tx
Pt 14
Pt 24
Pt 7

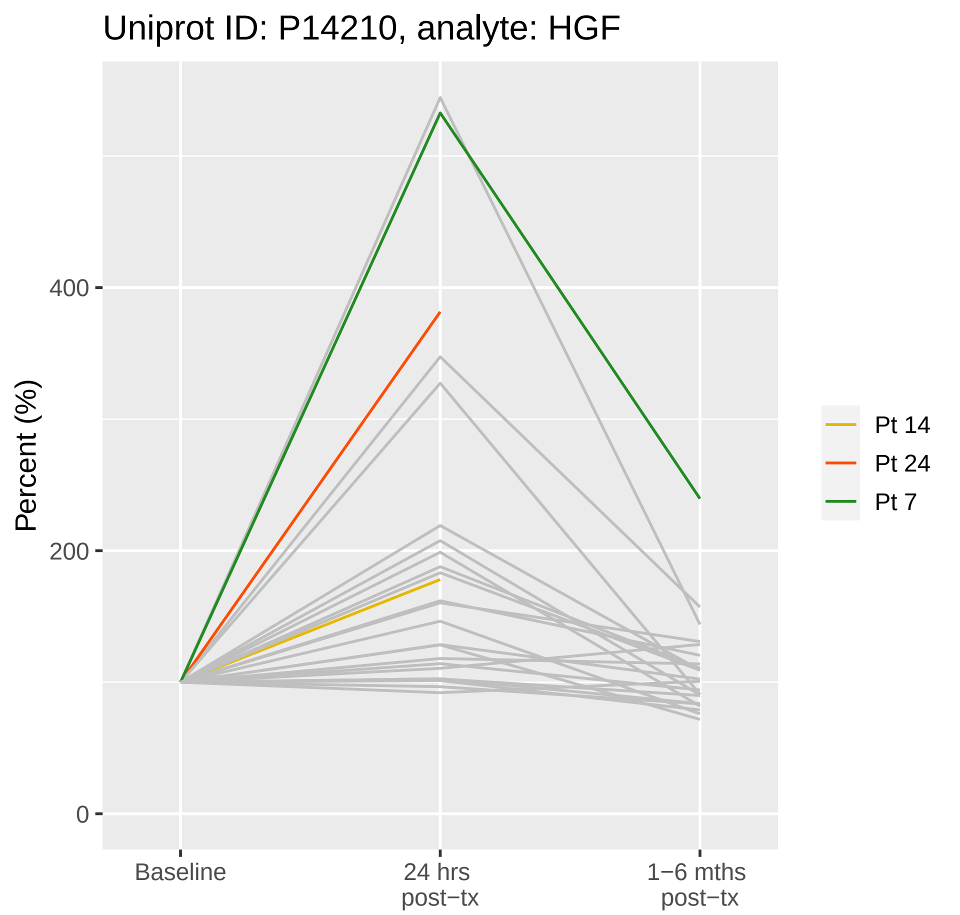

Uniprot ID: P14210, analyte: HGF
Percent (%)
400
200
0
Baseline
24 hrs
post−tx
1−6 mths
post−tx
Pt 14
Pt 24
Pt 7

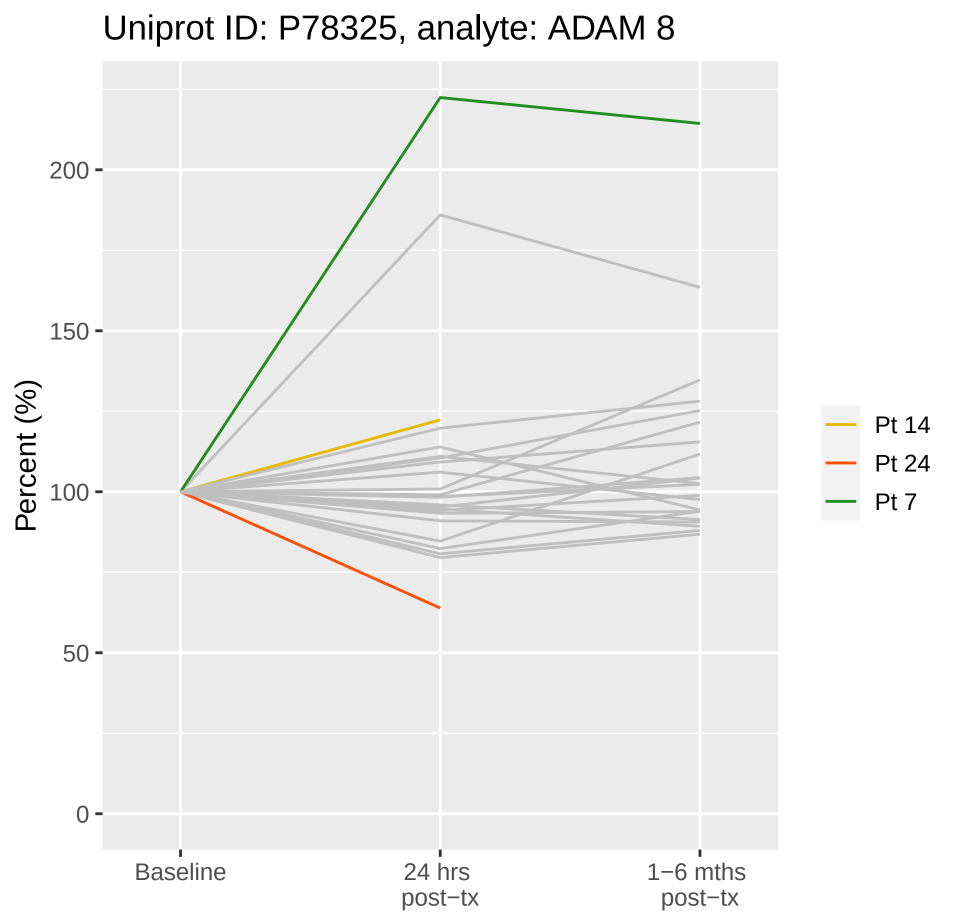
Uniprot ID: P78325, analyte: ADAM 8
Percent (%)
200
150
100
50
0
Baseline
24 hrs post−tx
1−6 mths post−tx
Pt 14
Pt 24
Pt 7

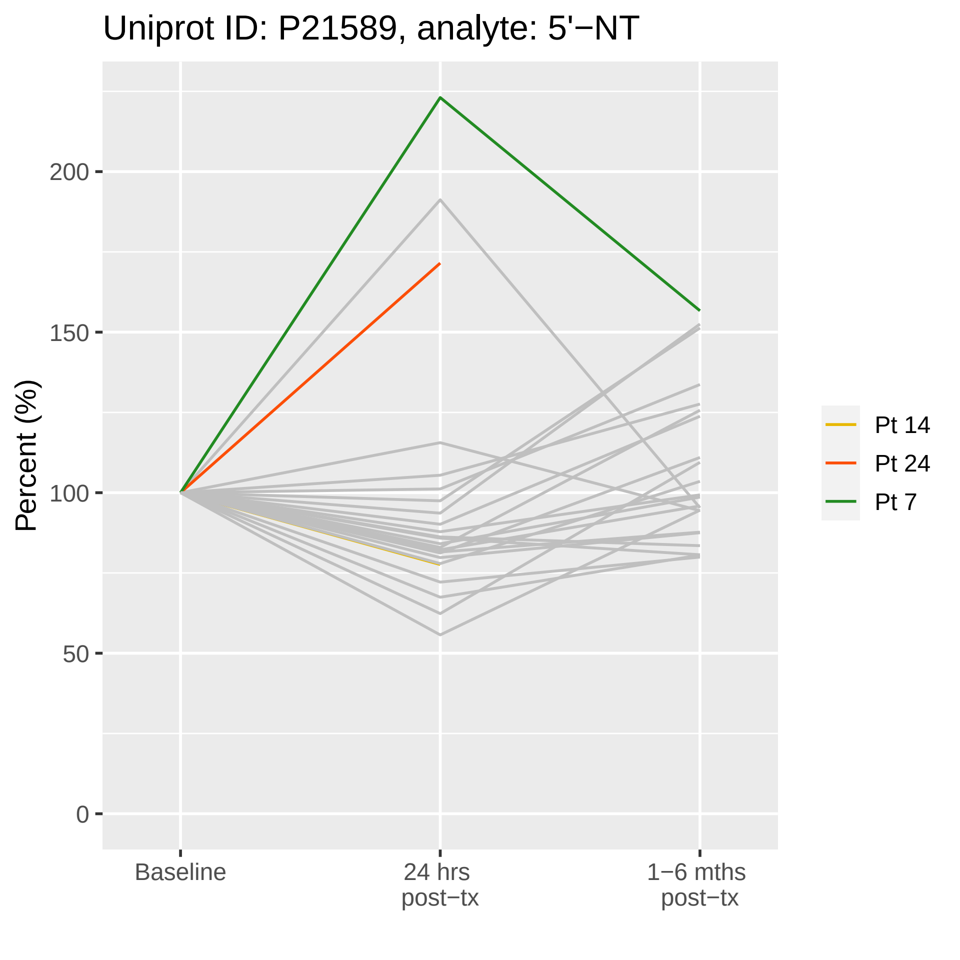

Uniprot ID: P21589, analyte: 5'−NT
Percent (%)
200
150
100
50
0
Baseline
24 hrs
post−tx
1−6 mths
post−tx
Pt 14
Pt 24
Pt 7

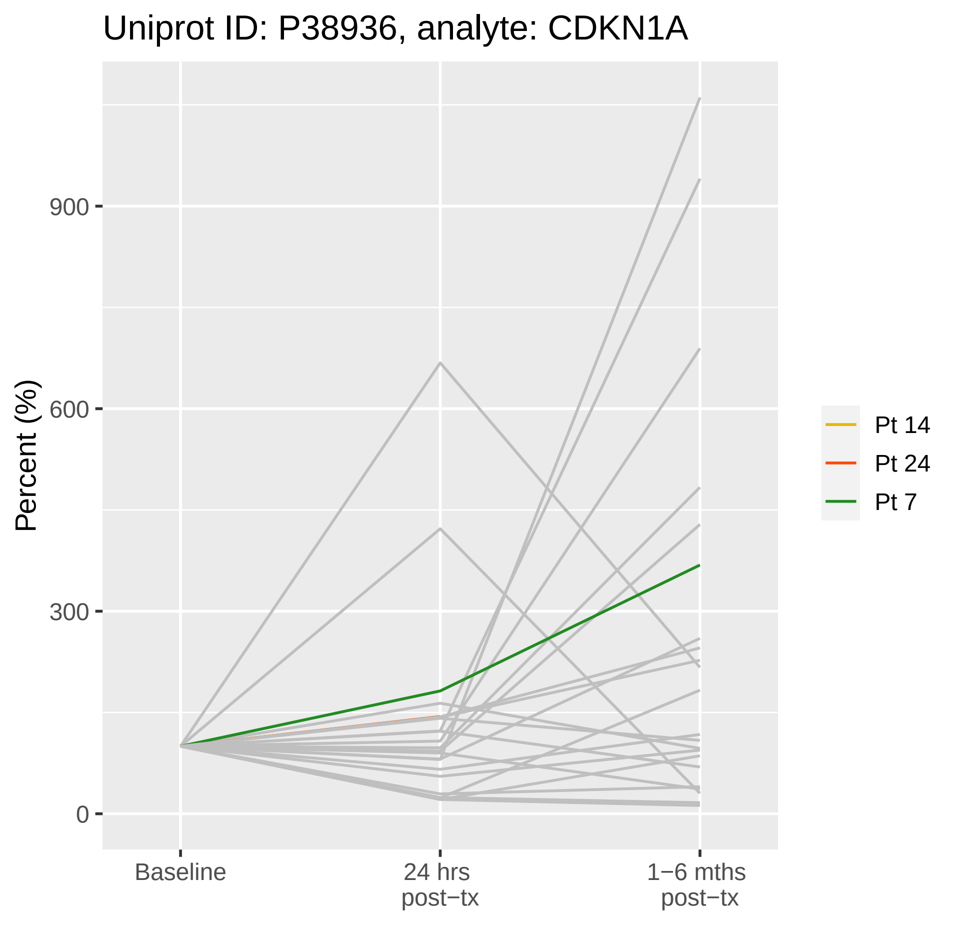

Uniprot ID: P38936, analyte: CDKN1A
Percent (%)
900
600
300
0
Baseline
24 hrs post−tx
1−6 mths post−tx
Pt 14
Pt 24
Pt 7

Uniprot ID: O00548, analyte: DLL1

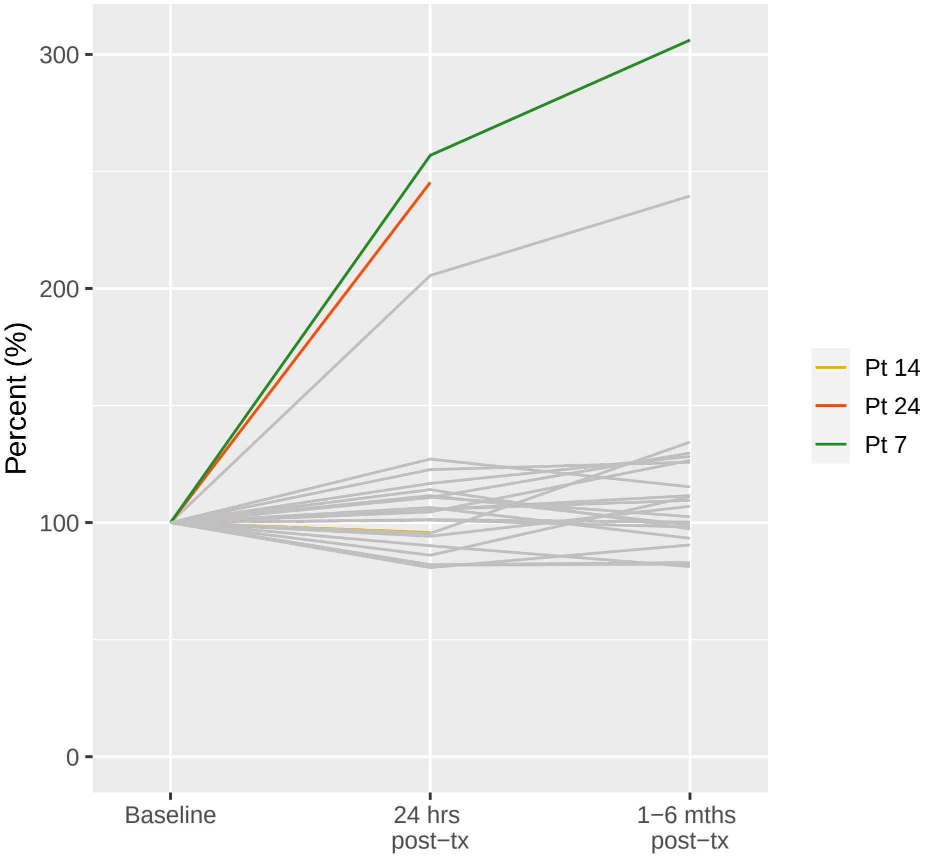

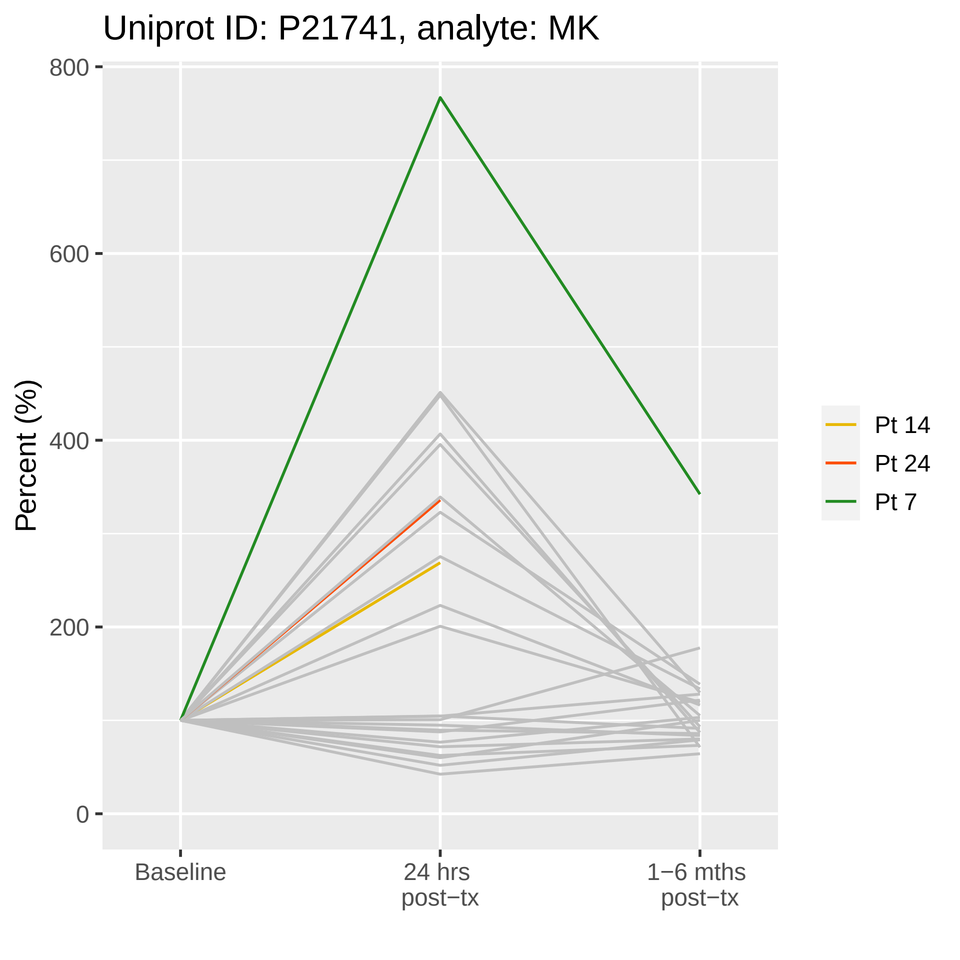
Uniprot ID: P21741, analyte: MK
Percent (%)
800
600
400
200
0
Baseline
24 hrs
post−tx
1−6 mths
post−tx
Pt 14
Pt 24
Pt 7

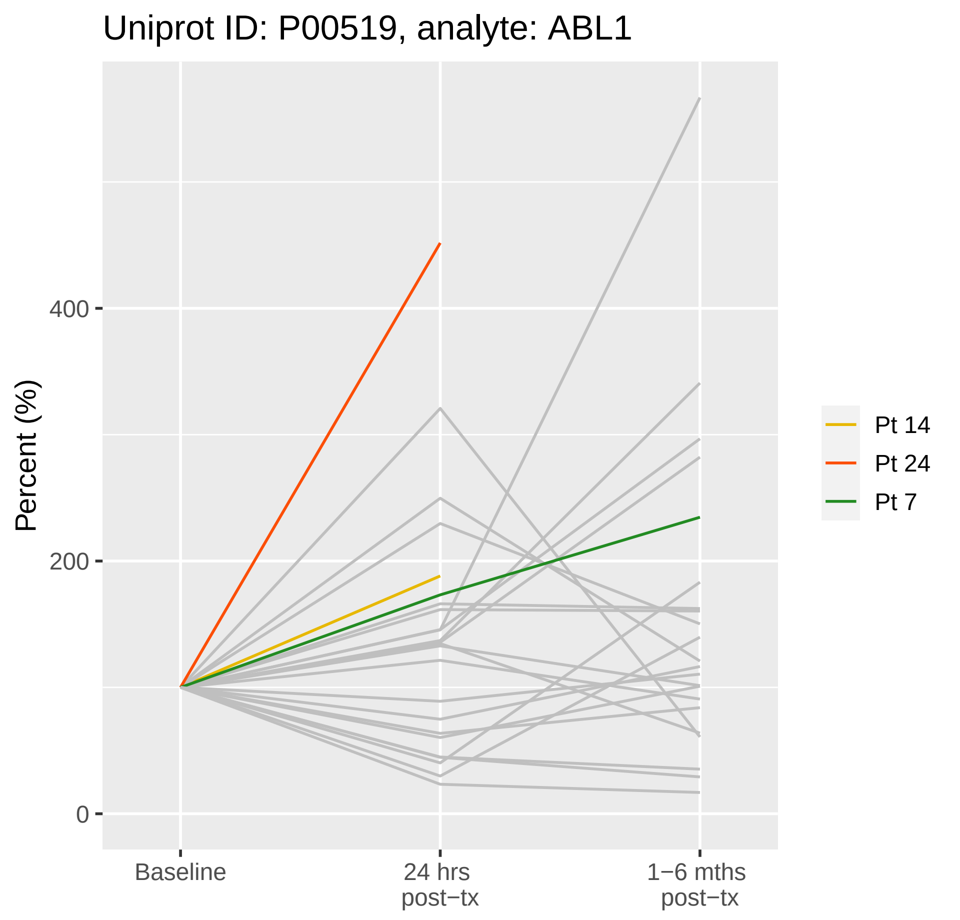

Uniprot ID: P00519, analyte: ABL1
Percent (%)
400
200
0
Baseline
24 hrs post−tx
1−6 mths post−tx
Pt 14
Pt 24
Pt 7

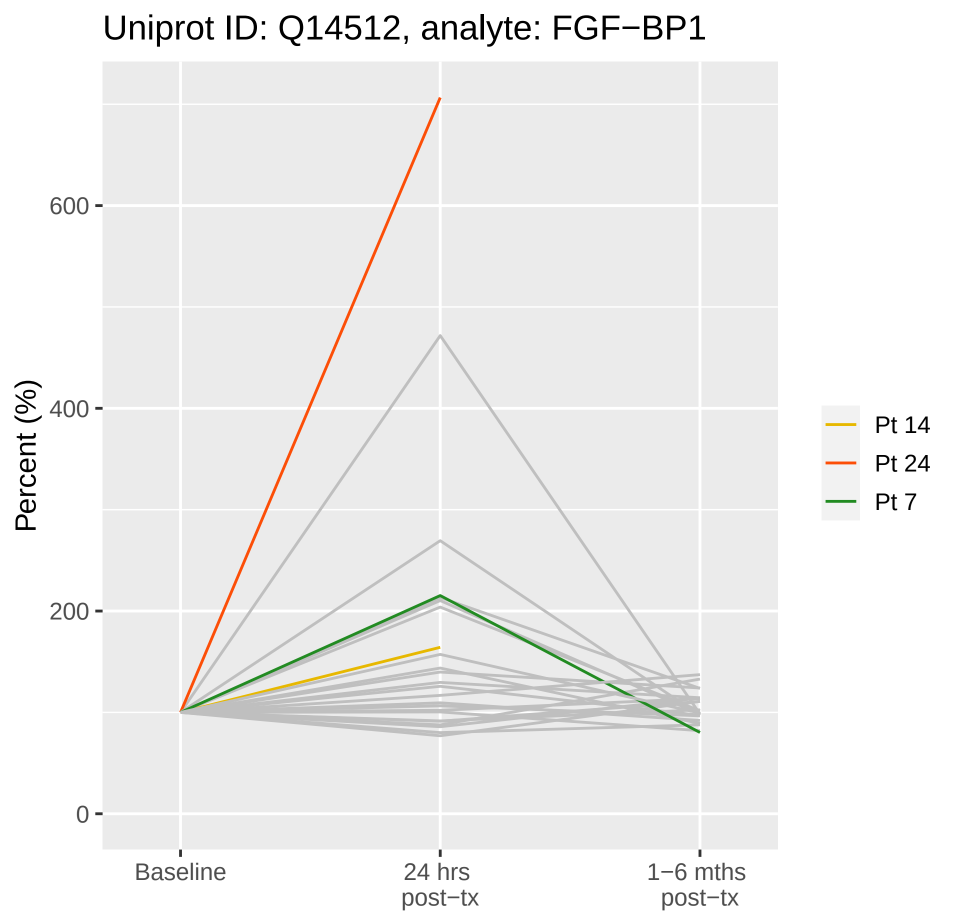
Uniprot ID: Q14512, analyte: FGF−BP1
Percent (%)
600
400
200
0
Baseline
24 hrs
post−tx
1−6 mths
post−tx
Pt 14
Pt 24
Pt 7

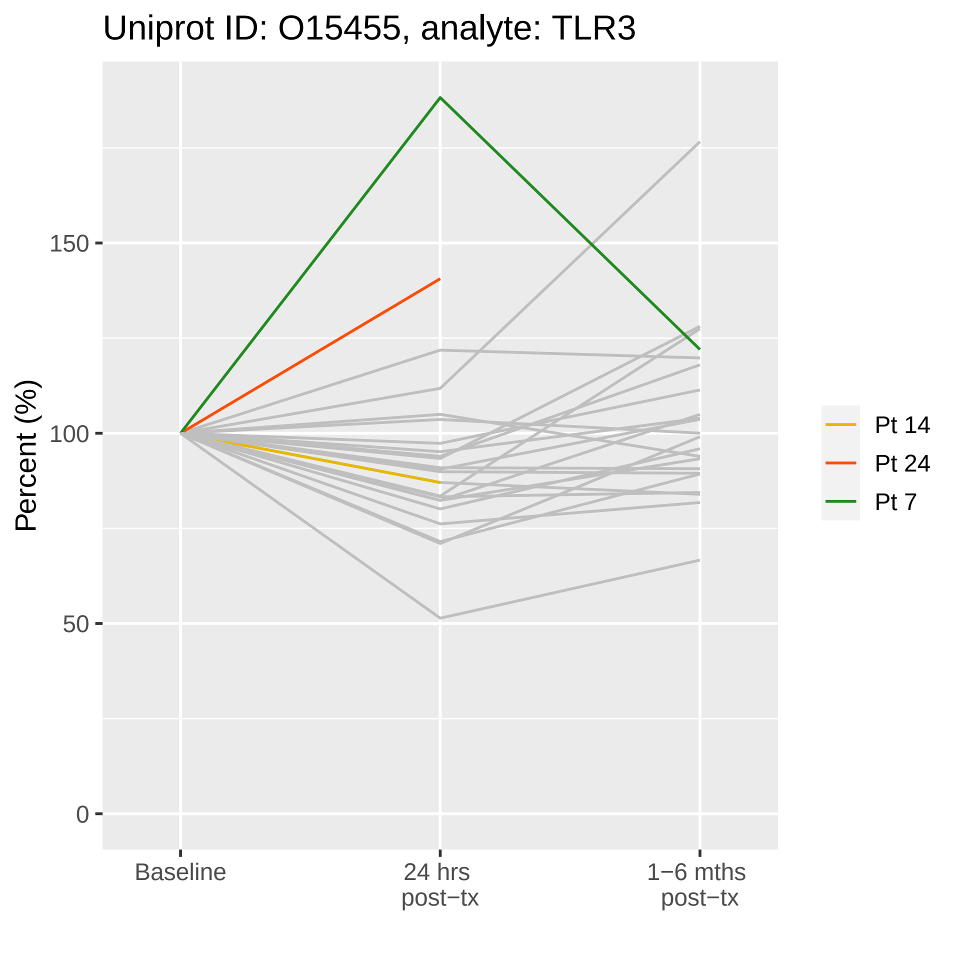

Uniprot ID: O15455, analyte: TLR3
Percent (%)
150
100
50
0
Baseline
24 hrs post−tx
1−6 mths post−tx
Pt 14
Pt 24
Pt 7

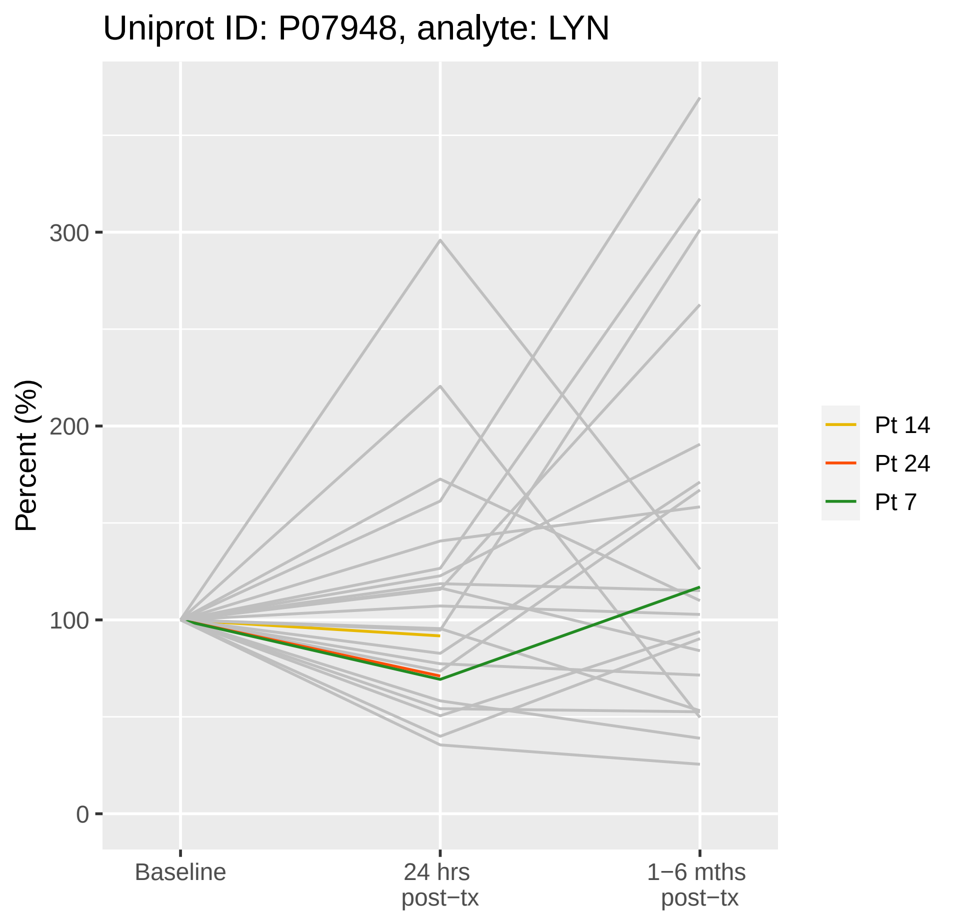

Uniprot ID: P07948, analyte: LYN
Percent (%)
300
200
100
0
Baseline
24 hrs post−tx
1−6 mths post−tx
Pt 14
Pt 24
Pt 7

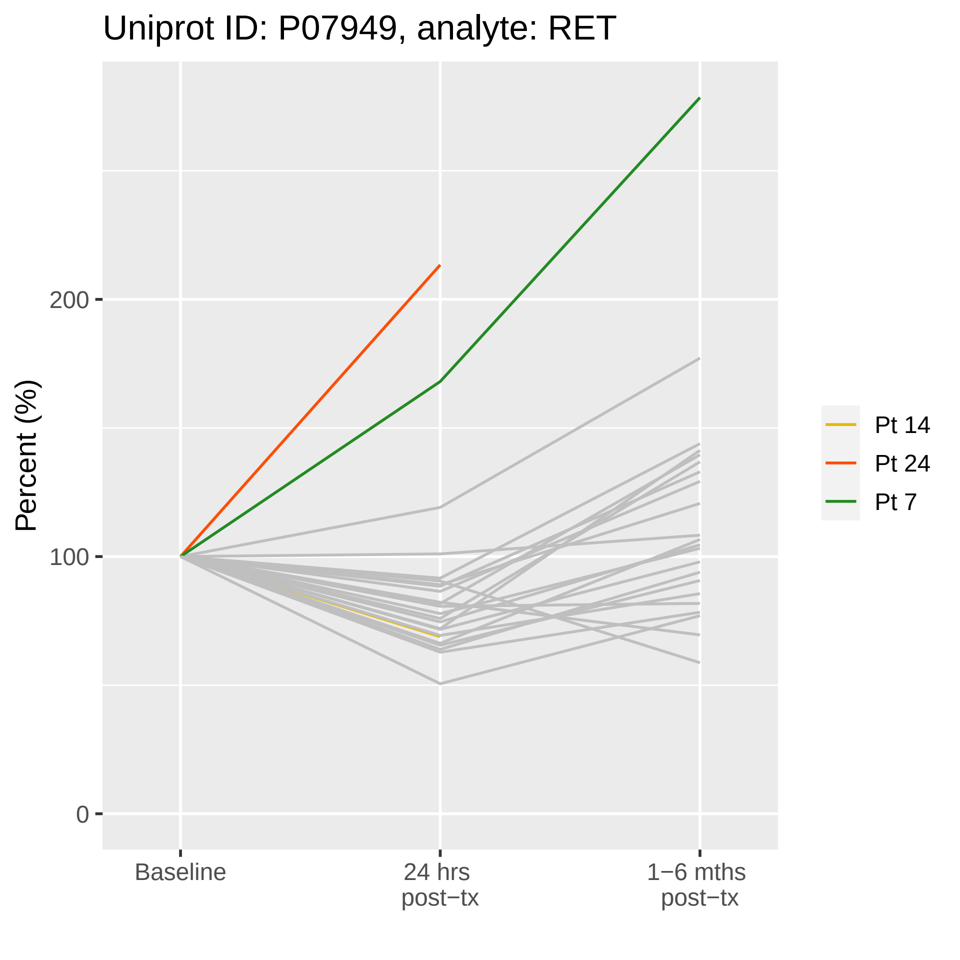
Uniprot ID: P07949, analyte: RET
Percent (%)
200
100
0
Baseline
24 hrs
post−tx
1−6 mths
post−tx
Pt 14
Pt 24
Pt 7

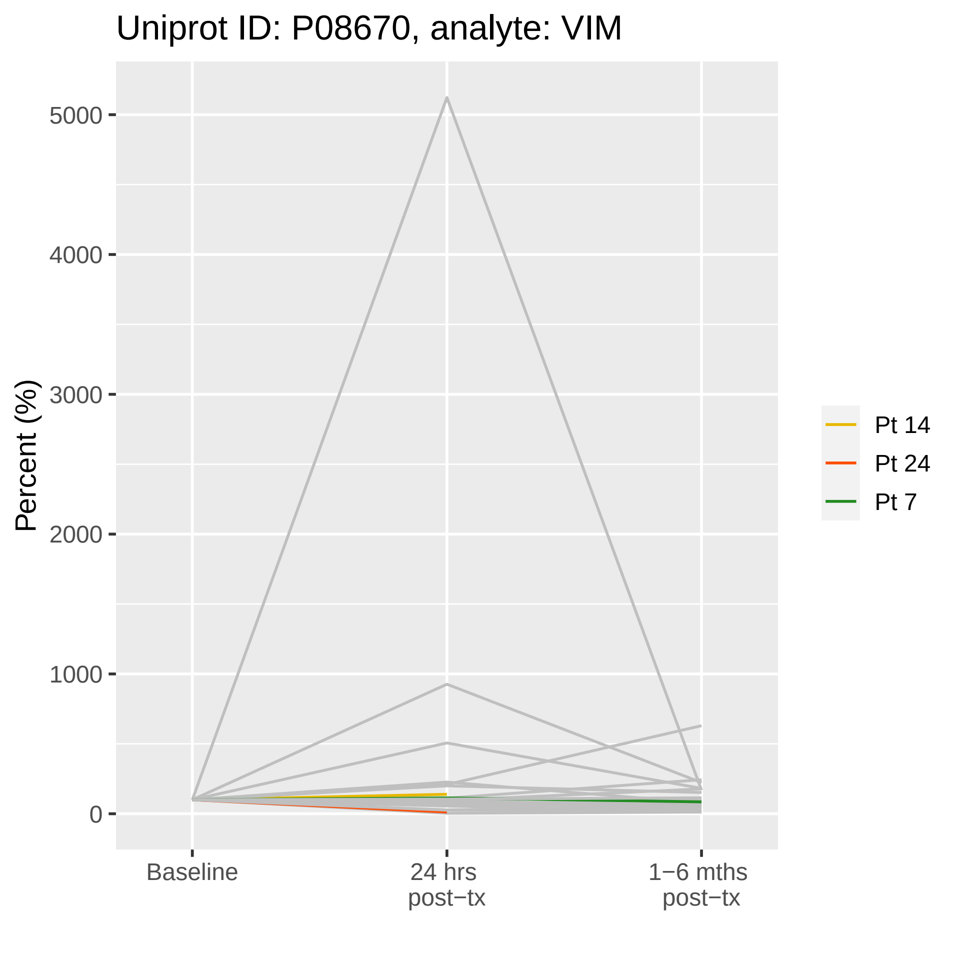

Uniprot ID: P08670, analyte: VIM
Percent (%)
5000
4000
3000
2000
1000
0
Baseline
24 hrs post−tx
1−6 mths post−tx
Pt 14
Pt 24
Pt 7

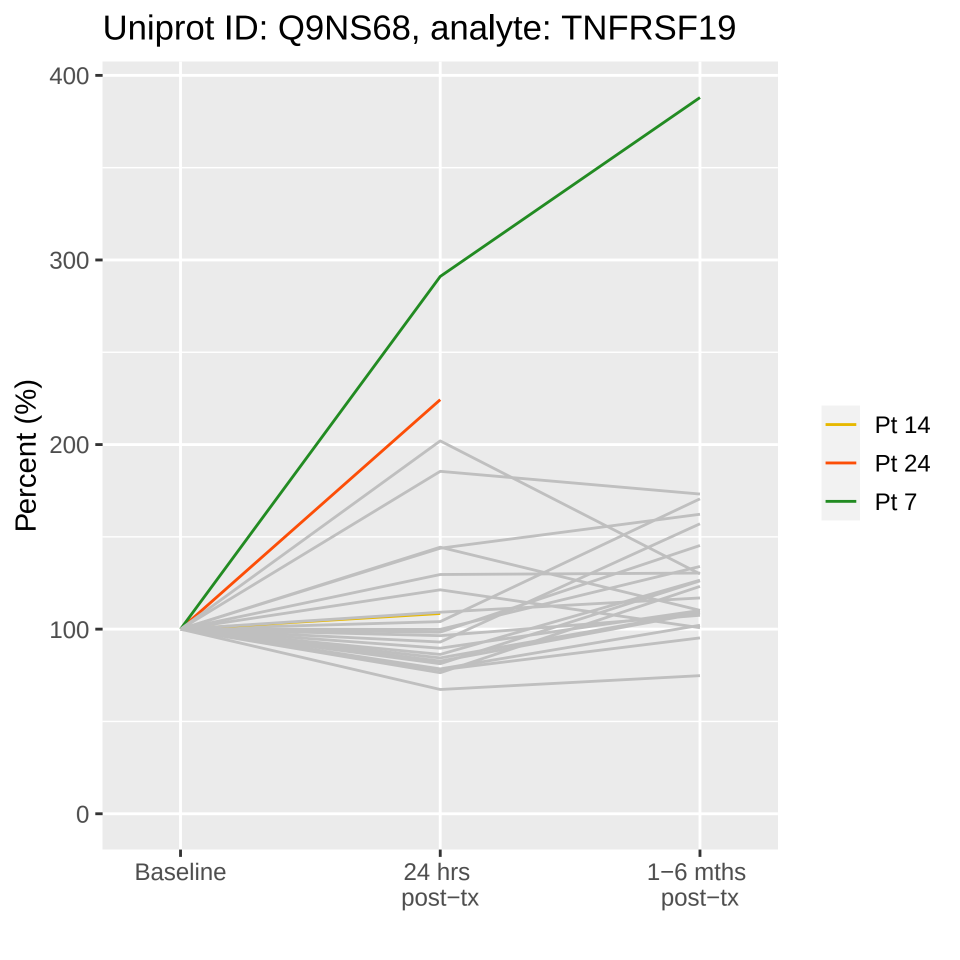
Uniprot ID: Q9NS68, analyte: TNFRSF19
Percent (%)
400
300
200
100
0
Baseline
24 hrs
post−tx
1−6 mths
post−tx
Pt 14
Pt 24
Pt 7

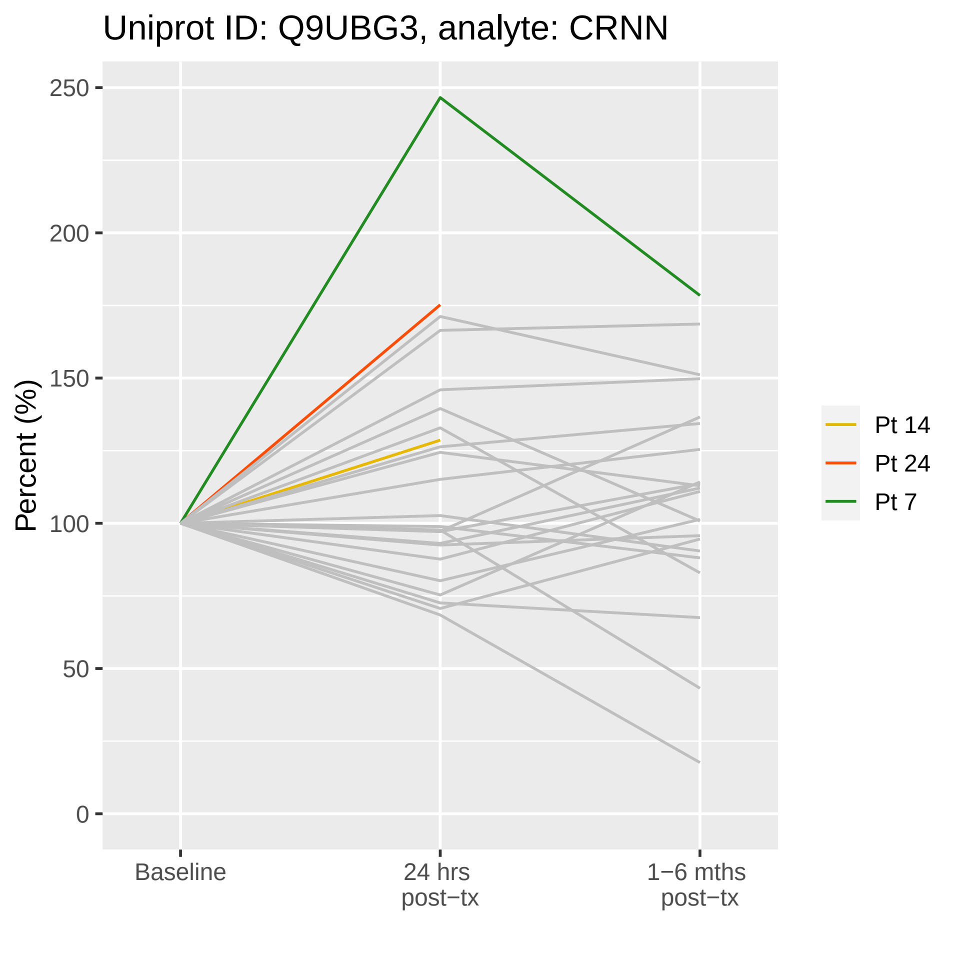

Uniprot ID: Q9UBG3, analyte: CRNN
Percent (%)
250
200
150
100
50
0
Baseline
24 hrs post−tx
1−6 mths post−tx
Pt 14
Pt 24
Pt 7

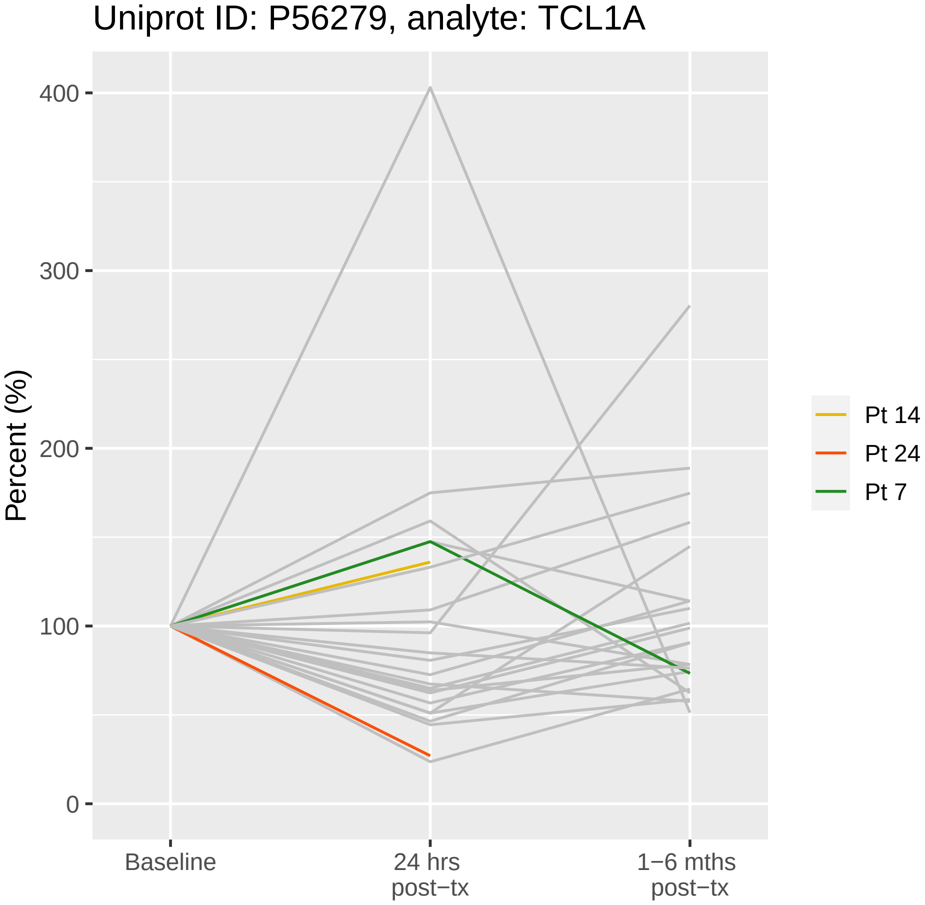

Uniprot ID: P56279, analyte: TCL1A
Percent (%)
400
300
200
100
0
Baseline
24 hrs
post−tx
1−6 mths
post−tx
Pt 14
Pt 24
Pt 7

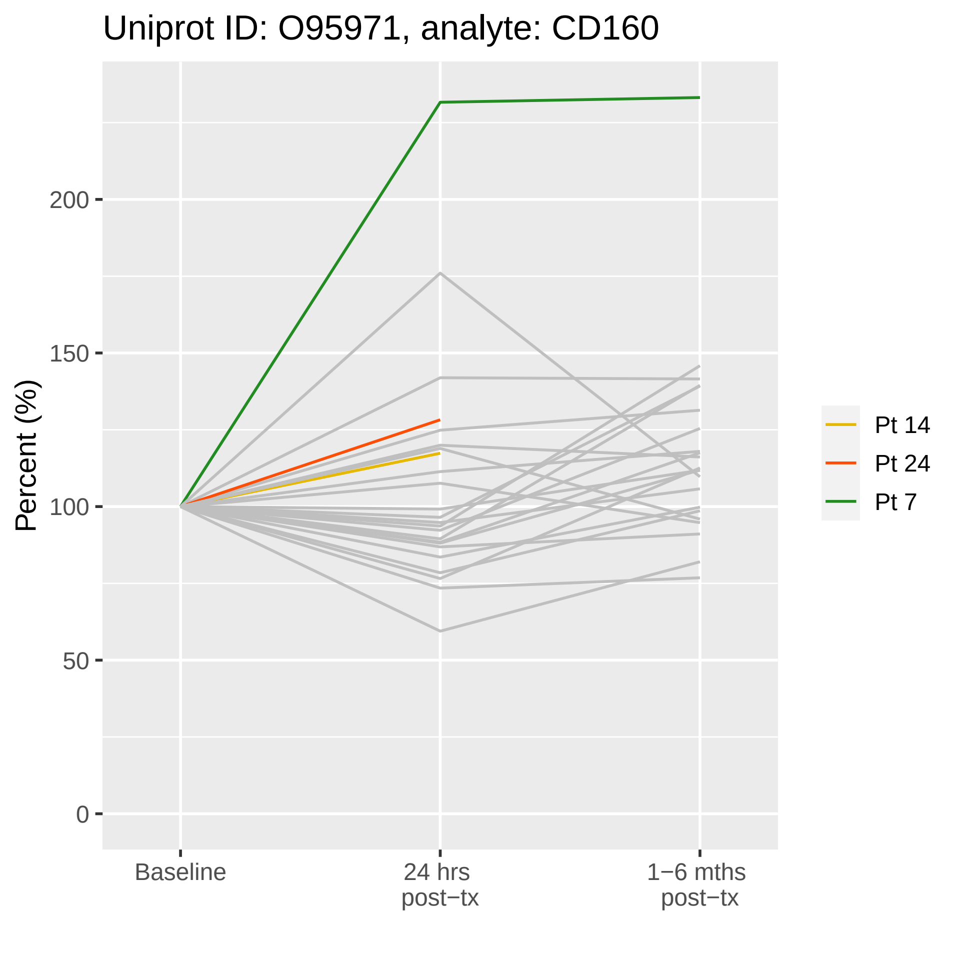

Uniprot ID: O95971, analyte: CD160
Percent (%)
200
150
100
50
0
Baseline
24 hrs post−tx
1−6 mths post−tx
Pt 14
Pt 24
Pt 7

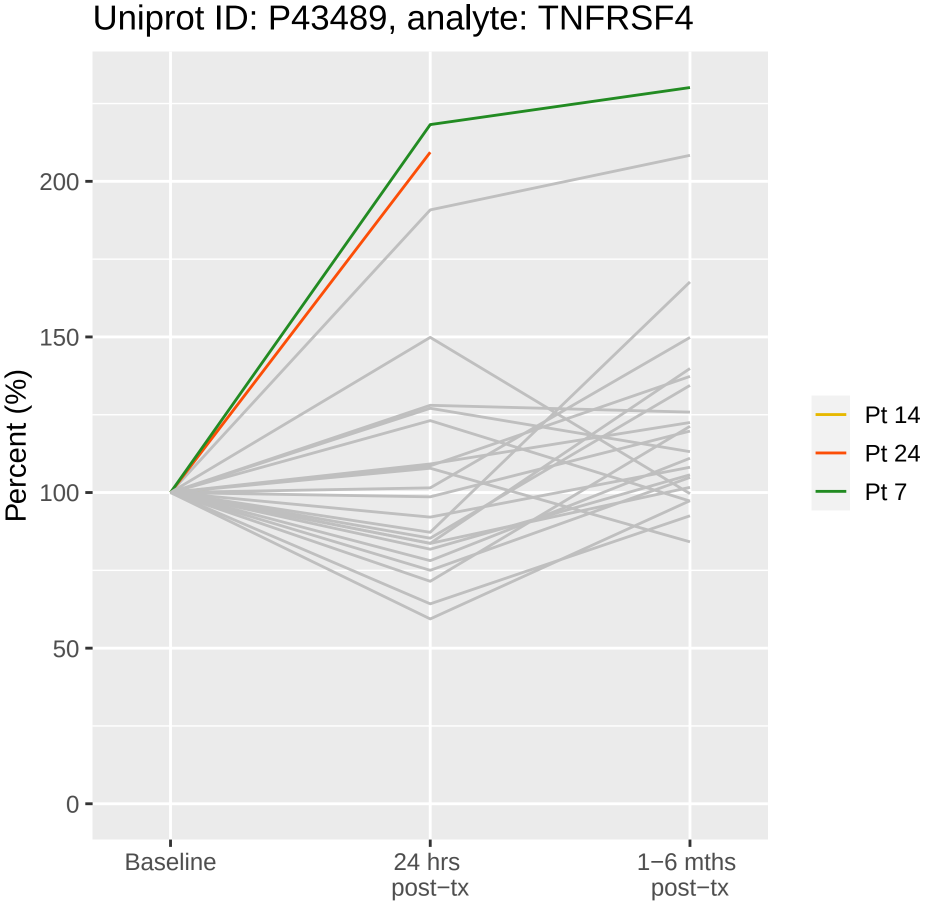

Uniprot ID: P43489, analyte: TNFRSF4
Percent (%)
200
150
100
50
0
Baseline
24 hrs post−tx
1−6 mths post−tx
Pt 14
Pt 24
Pt 7

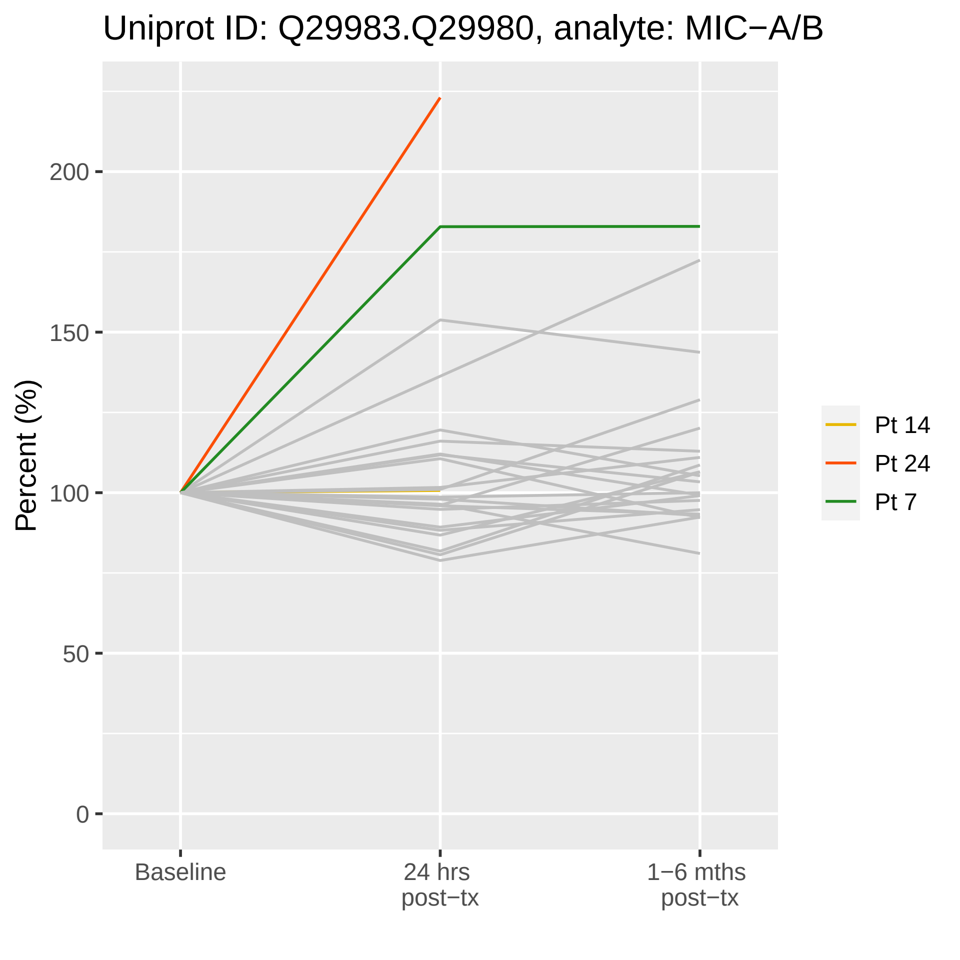

Uniprot ID: Q29983.Q29980, analyte: MIC−A/B
Percent (%)
0
50
100
150
200
Baseline
24 hrs post−tx
1−6 mths post−tx
Pt 14
Pt 24
Pt 7

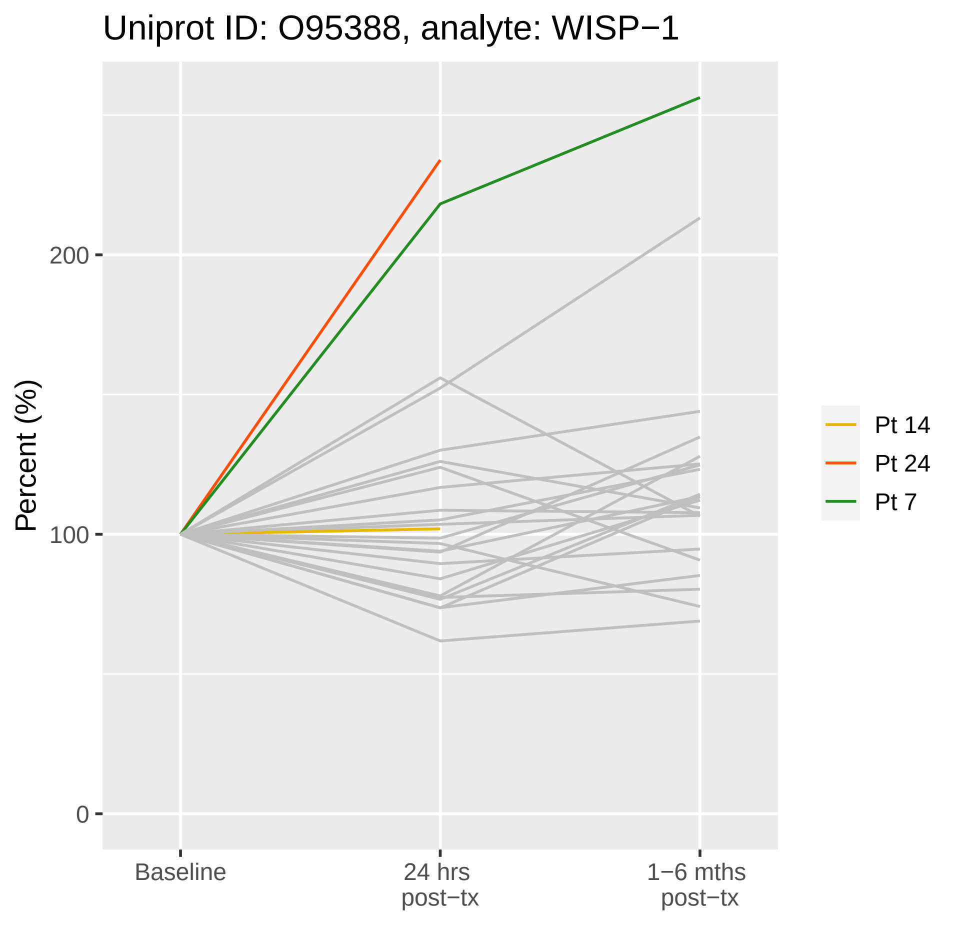
Uniprot ID: O95388, analyte: WISP−1
Percent (%)
200
100
0
Baseline
24 hrs post−tx
1−6 mths post−tx
Pt 14
Pt 24
Pt 7

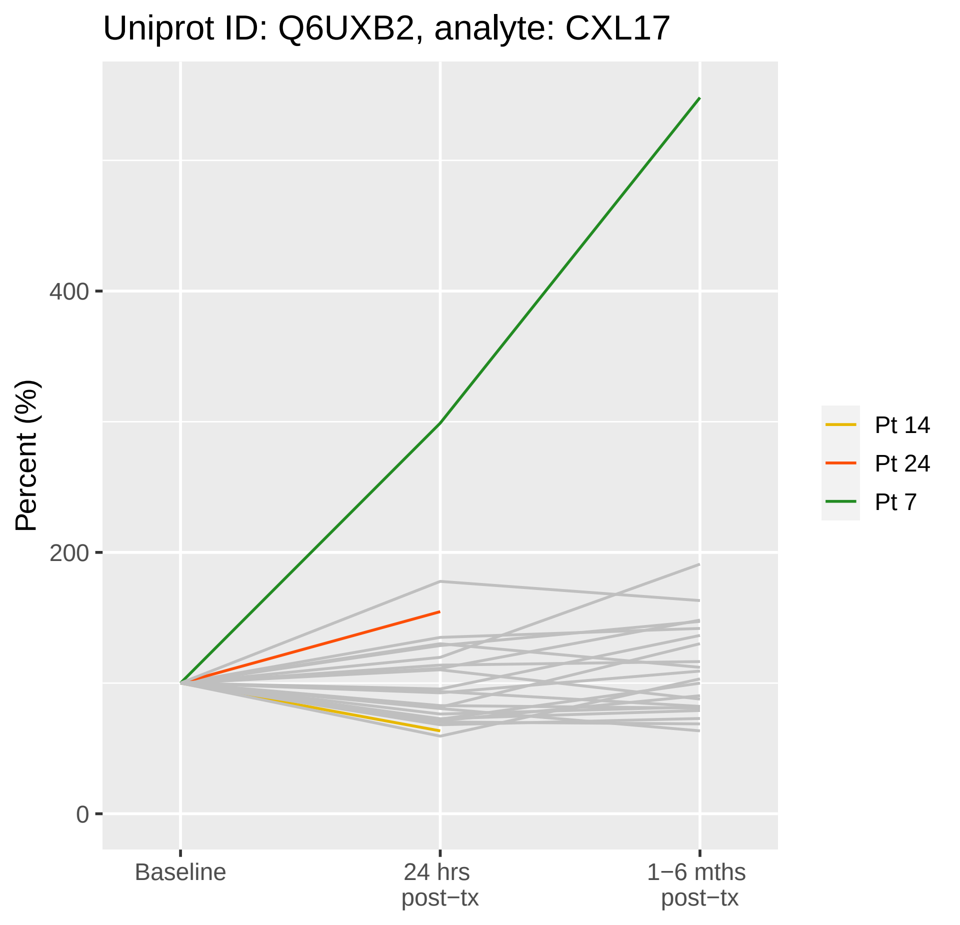
Uniprot ID: Q6UXB2, analyte: CXL17
Percent (%)
400
200
0
Baseline
24 hrs
post−tx
1−6 mths
post−tx
Pt 14
Pt 24
Pt 7

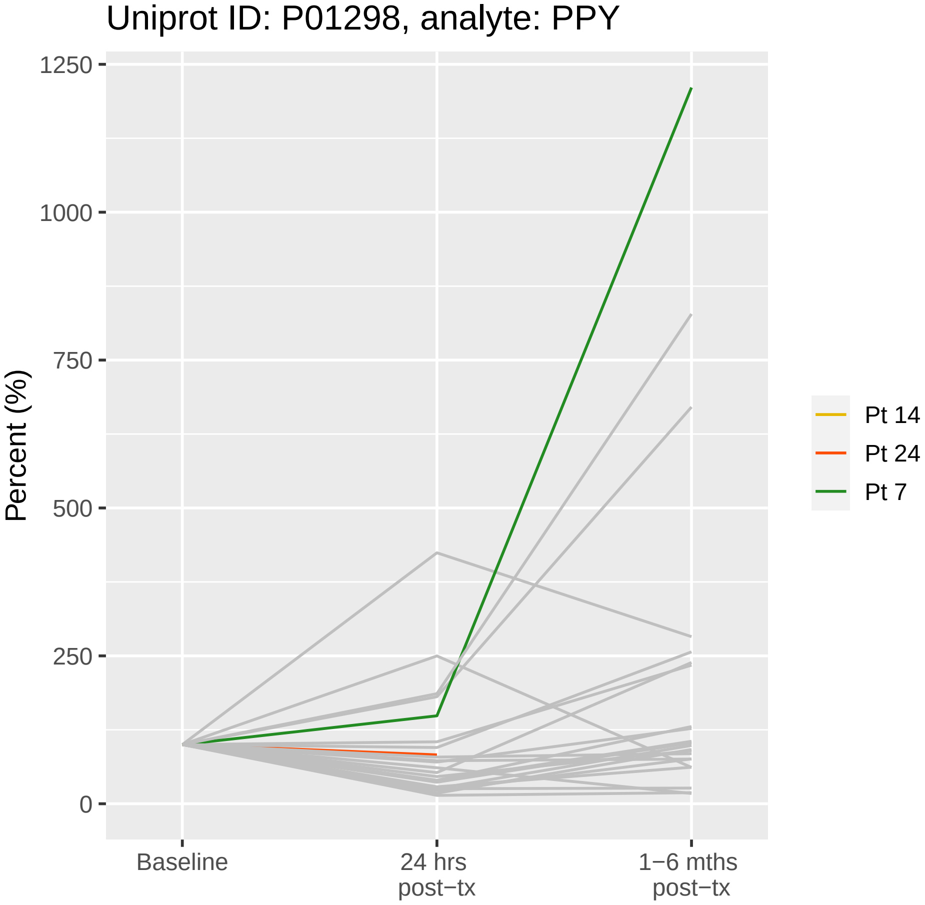
Uniprot ID: P01298, analyte: PPY
Percent (%)
1250
1000
750
500
250
0
Baseline
24 hrs
post−tx
1−6 mths
post−tx
Pt 14
Pt 24
Pt 7

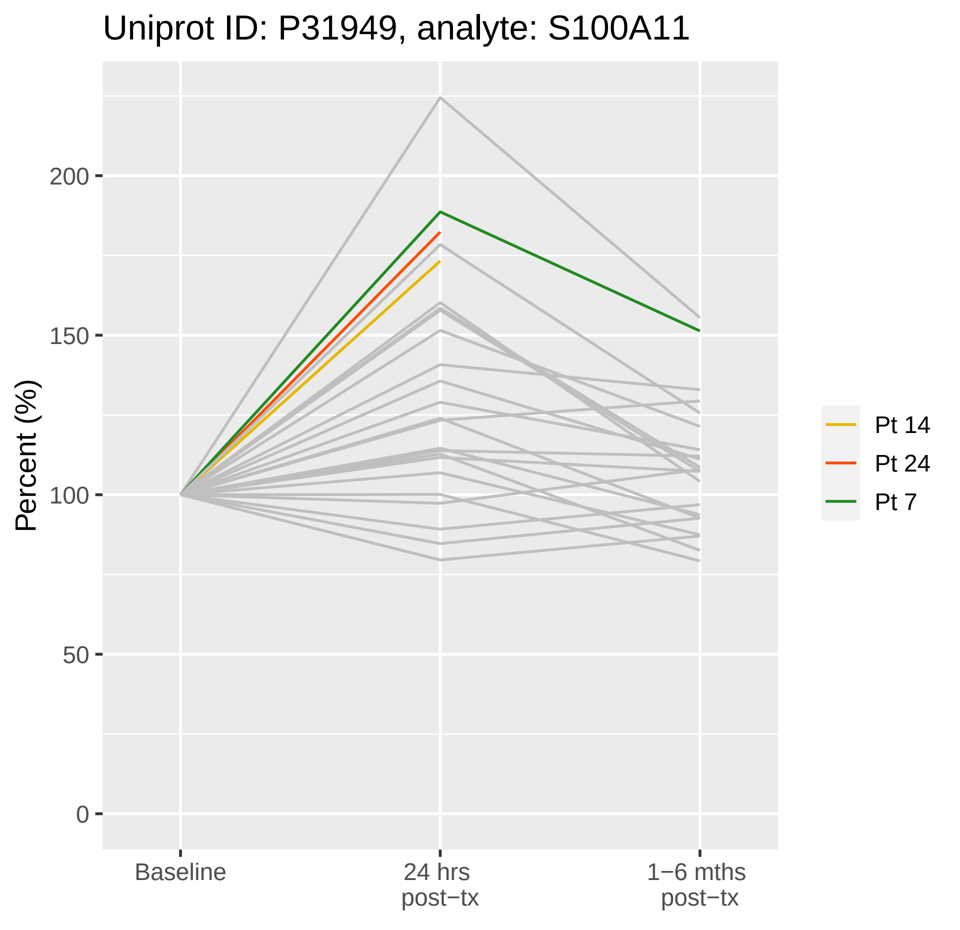
Uniprot ID: P31949, analyte: S100A11
Percent (%)
200
150
100
50
0
Baseline
24 hrs
post−tx
1−6 mths
post−tx
Pt 14
Pt 24
Pt 7

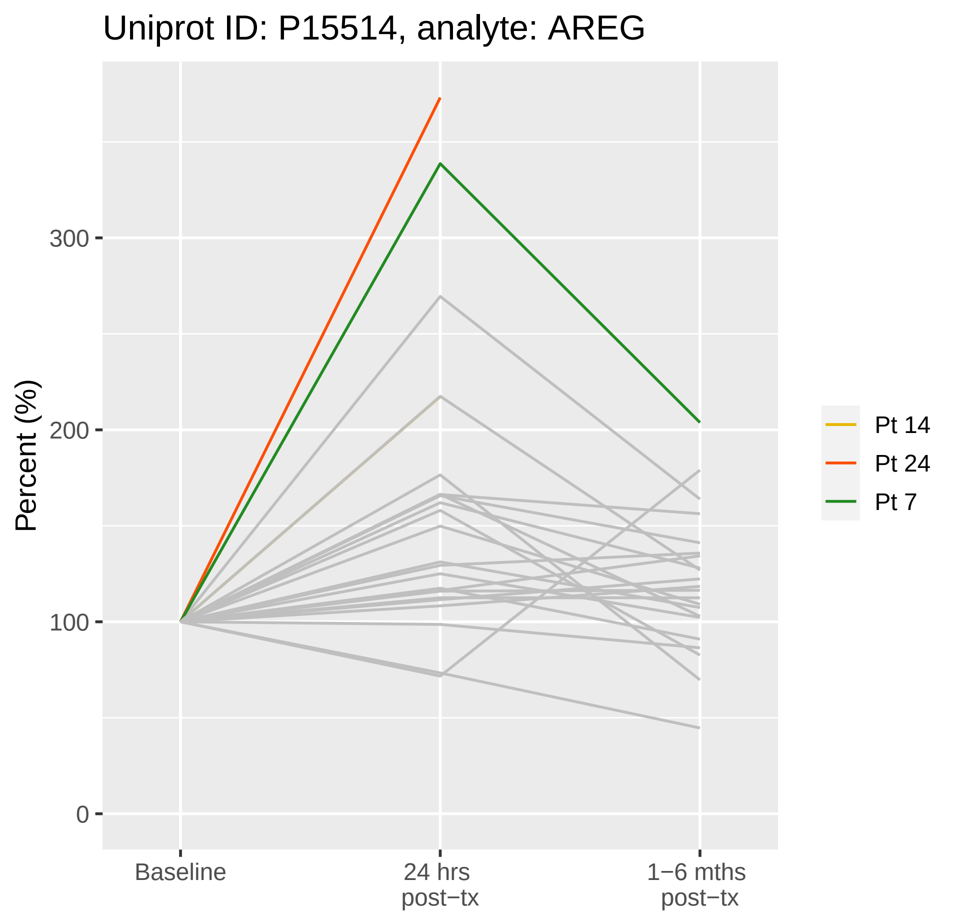

Uniprot ID: P15514, analyte: AREG
Percent (%)
300
200
100
0
Baseline
24 hrs post−tx
1−6 mths post−tx
Pt 14
Pt 24
Pt 7

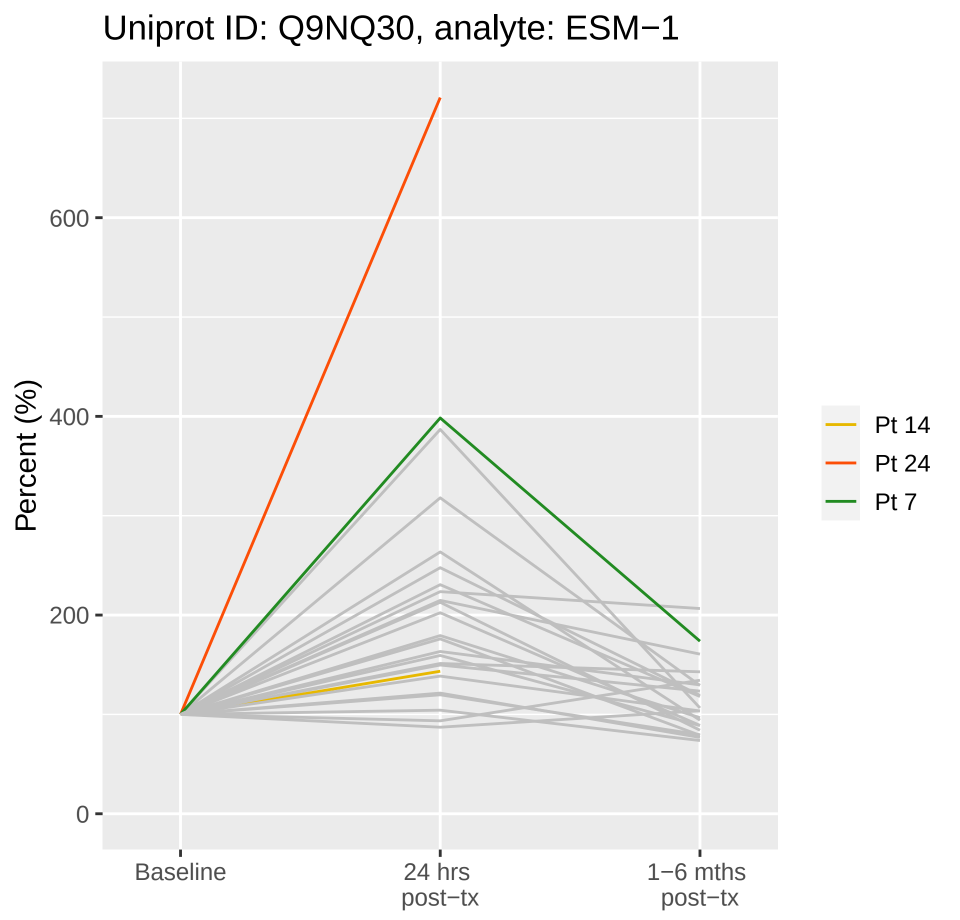
Uniprot ID: Q9NQ30, analyte: ESM−1
Percent (%)
600
400
200
0
Baseline
24 hrs post−tx
1−6 mths post−tx
Pt 14
Pt 24
Pt 7

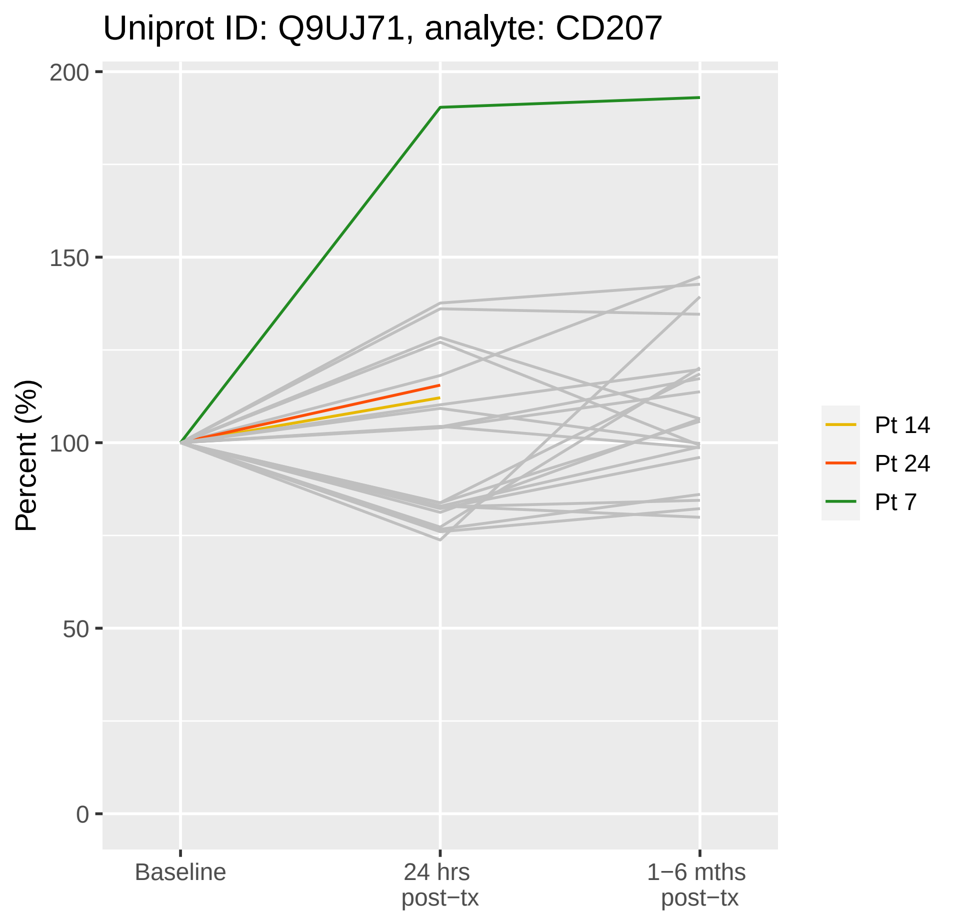
Uniprot ID: Q9UJ71, analyte: CD207
Percent (%)
200
150
100
50
0
Baseline
24 hrs post−tx
1−6 mths post−tx
Pt 14
Pt 24
Pt 7

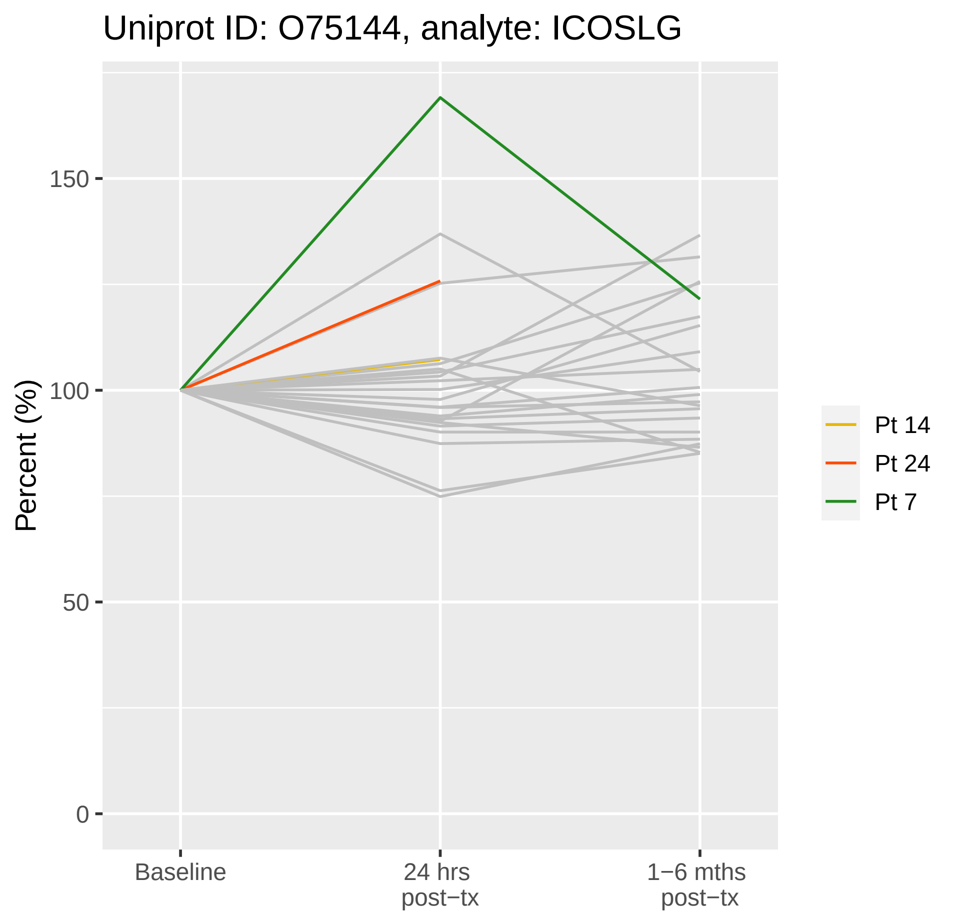
Uniprot ID: O75144, analyte: ICOSLG
Percent (%)
150
100
50
0
Baseline
24 hrs post−tx
1−6 mths post−tx
Pt 14
Pt 24
Pt 7

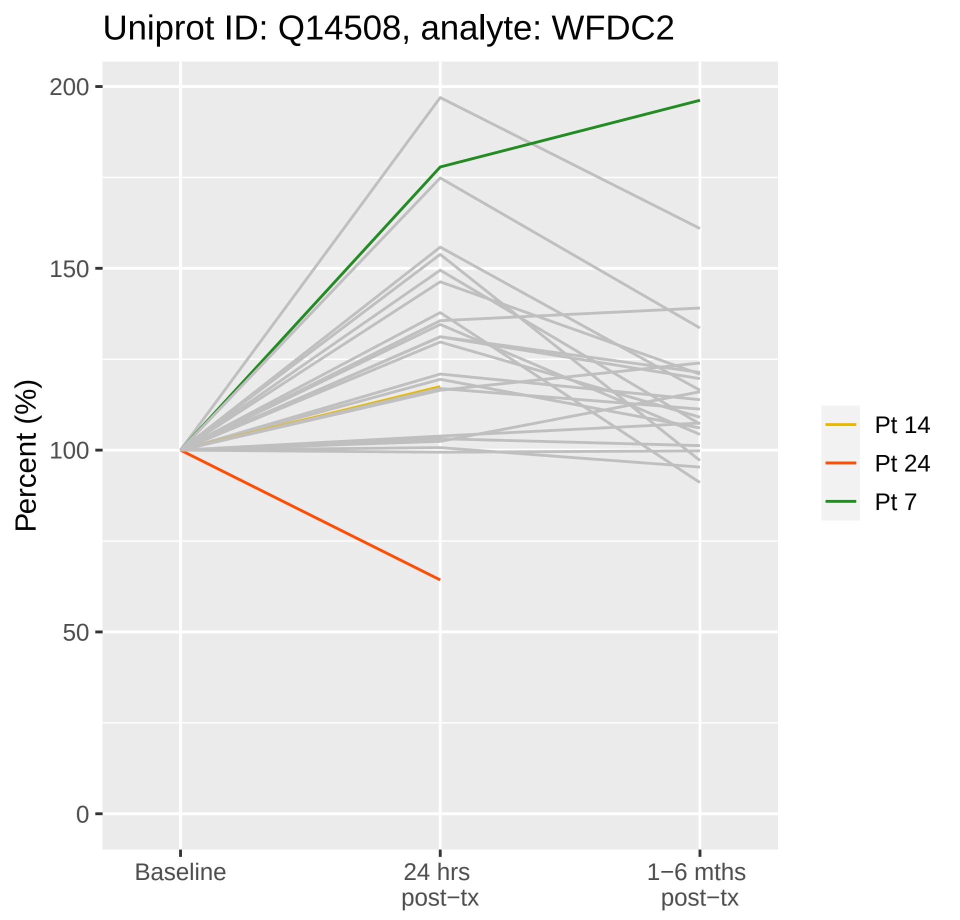
Uniprot ID: Q14508, analyte: WFDC2
Percent (%)
200
150
100
50
0
Baseline
24 hrs post−tx
1−6 mths post−tx
Pt 14
Pt 24
Pt 7

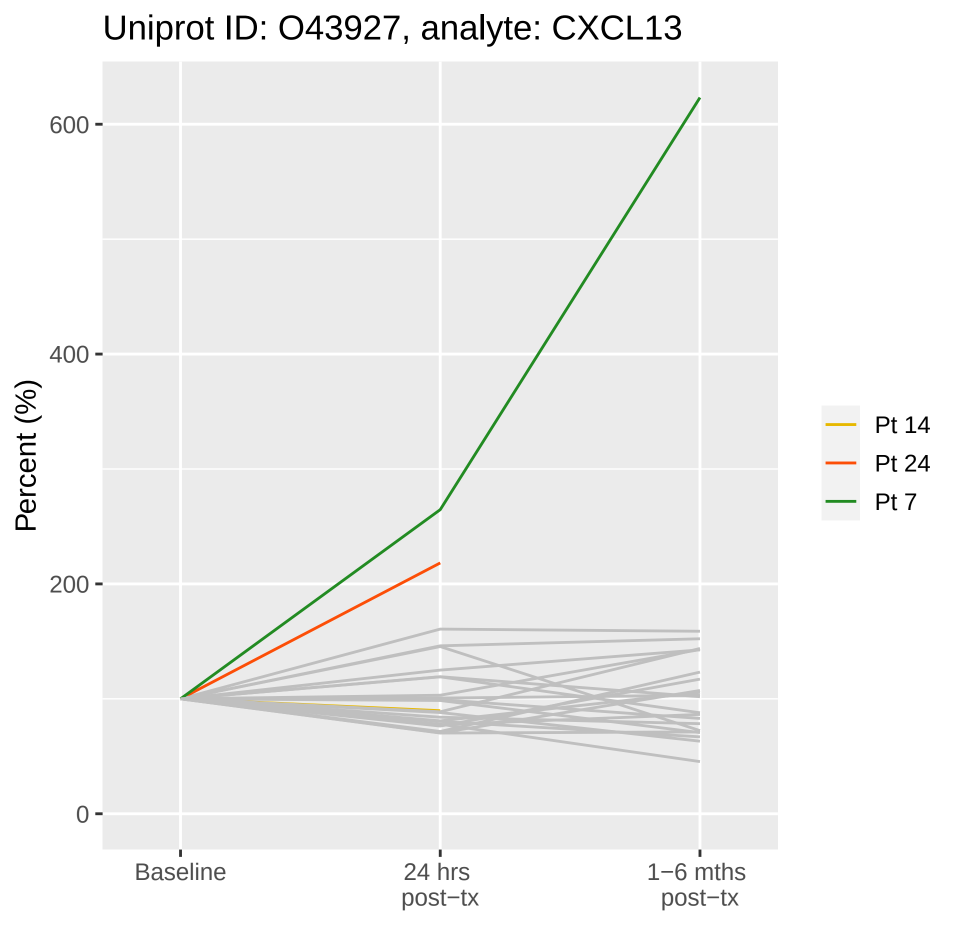

Uniprot ID: O43927, analyte: CXCL13
Percent (%)
600
400
200
0
Baseline
24 hrs post−tx
1−6 mths post−tx
Pt 14
Pt 24
Pt 7

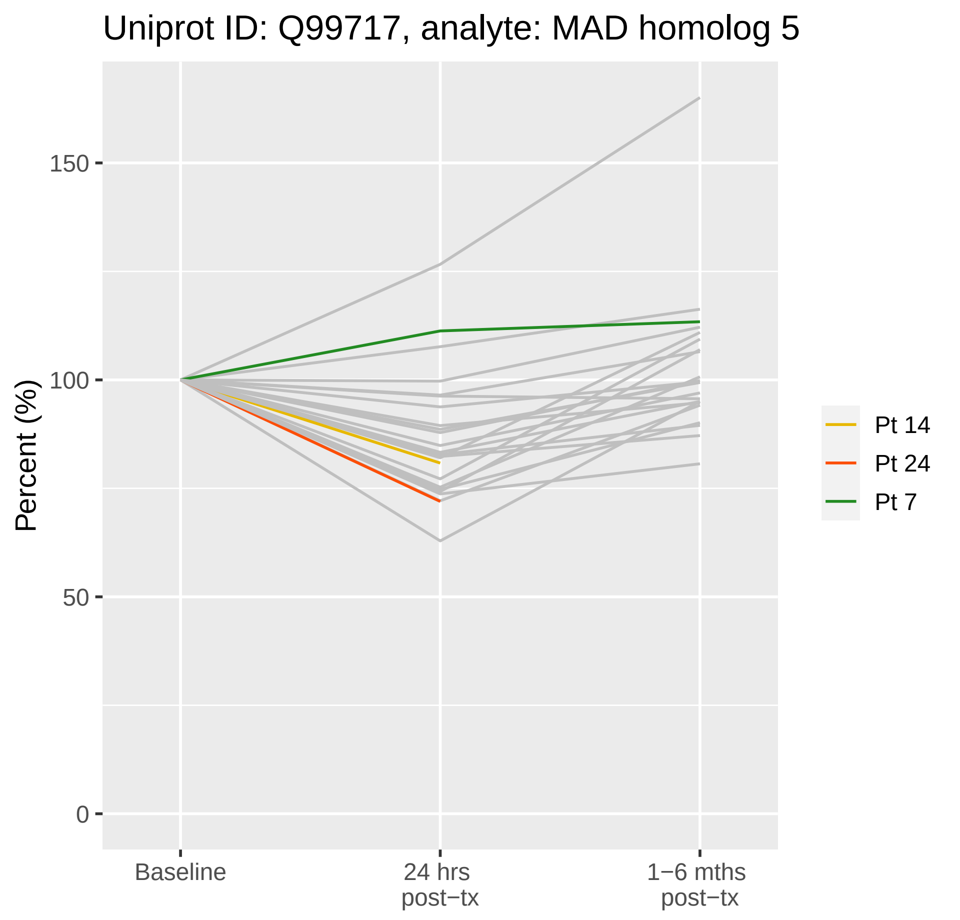
Uniprot ID: Q99717, analyte: MAD homolog 5
Percent (%)
150
100
50
0
Baseline
24 hrs post−tx
1−6 mths post−tx
Pt 14
Pt 24
Pt 7

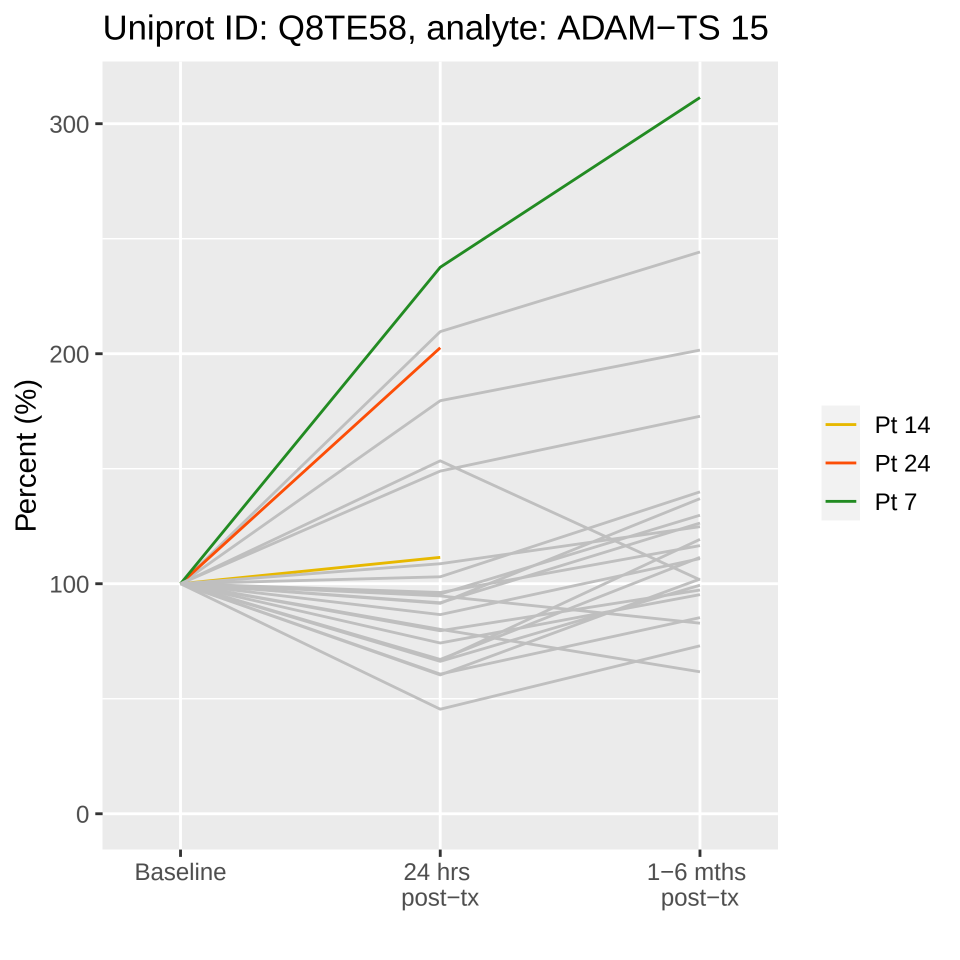

Uniprot ID: Q8TE58, analyte: ADAM−TS 15
Percent (%)
300
200
100
0
Baseline
24 hrs post−tx
1−6 mths post−tx
Pt 14
Pt 24
Pt 7

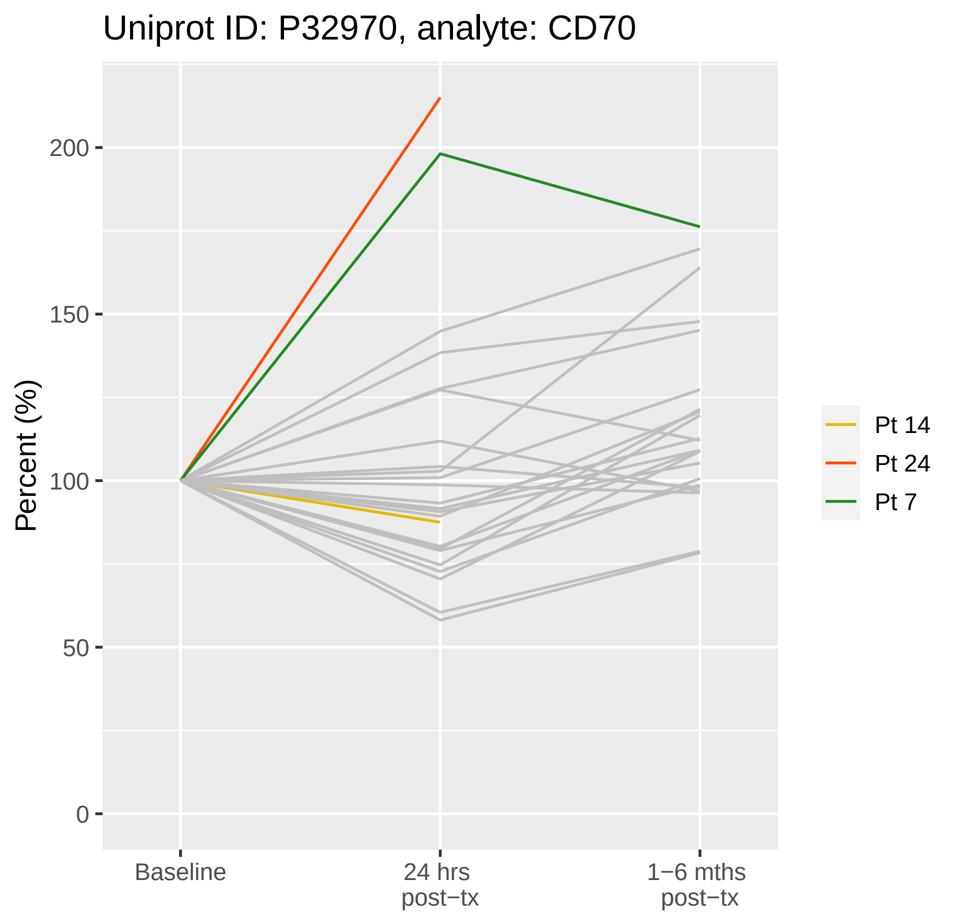

Uniprot ID: P32970, analyte: CD70
Percent (%)
200
150
100
50
0
Baseline
24 hrs post−tx
1−6 mths post−tx
Pt 14
Pt 24
Pt 7

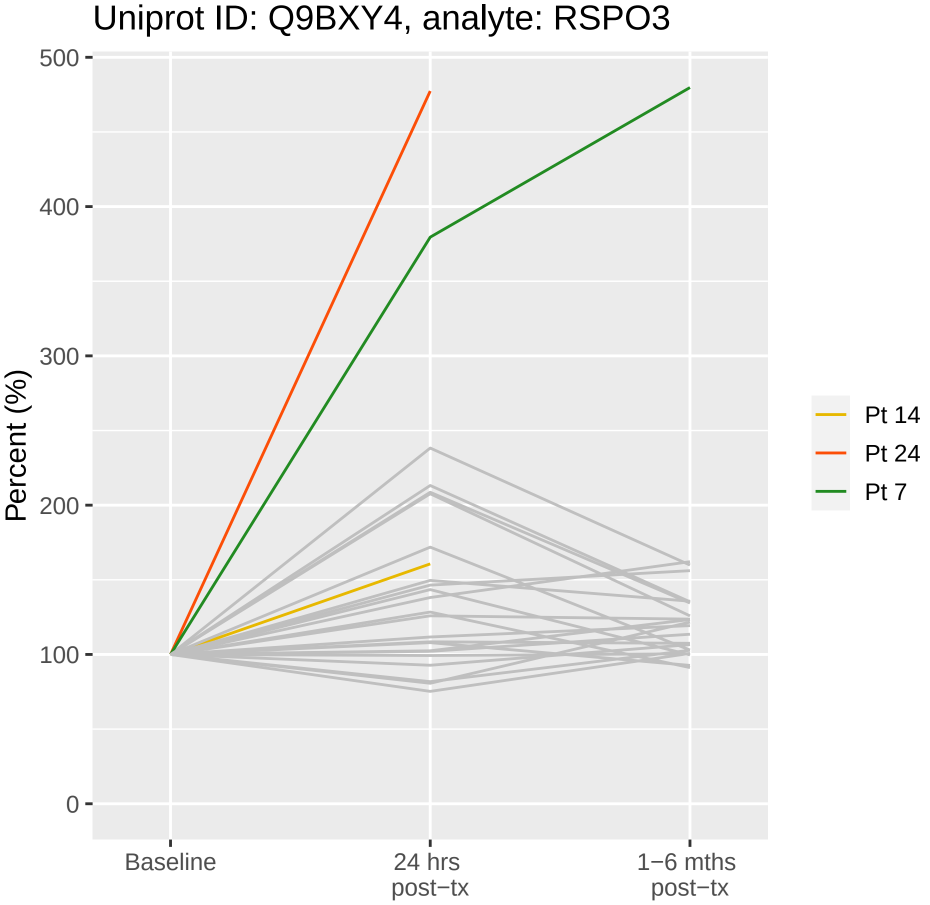

Uniprot ID: Q9BXY4, analyte: RSPO3
Percent (%)
500
400
300
200
100
0
Baseline
24 hrs
post−tx
1−6 mths
post−tx
Pt 14
Pt 24
Pt 7

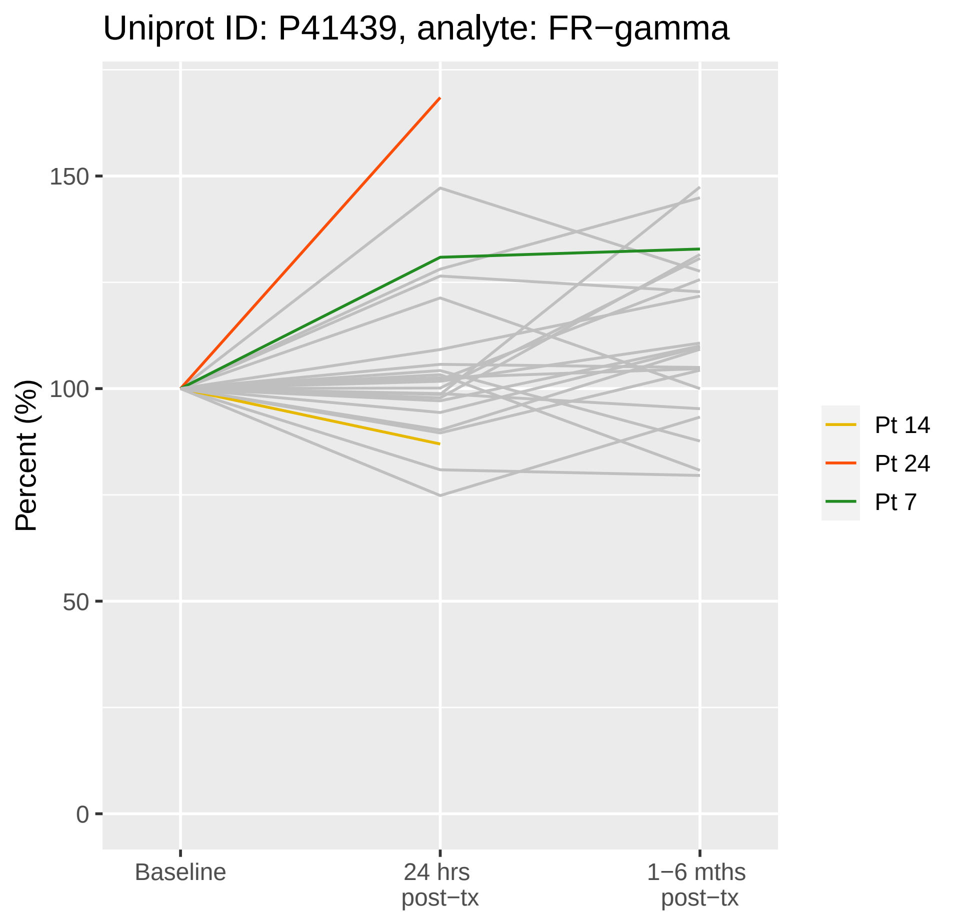

Uniprot ID: P41439, analyte: FR−gamma
Percent (%)
150
100
50
0
Baseline
24 hrs
post−tx
1−6 mths
post−tx
Pt 14
Pt 24
Pt 7

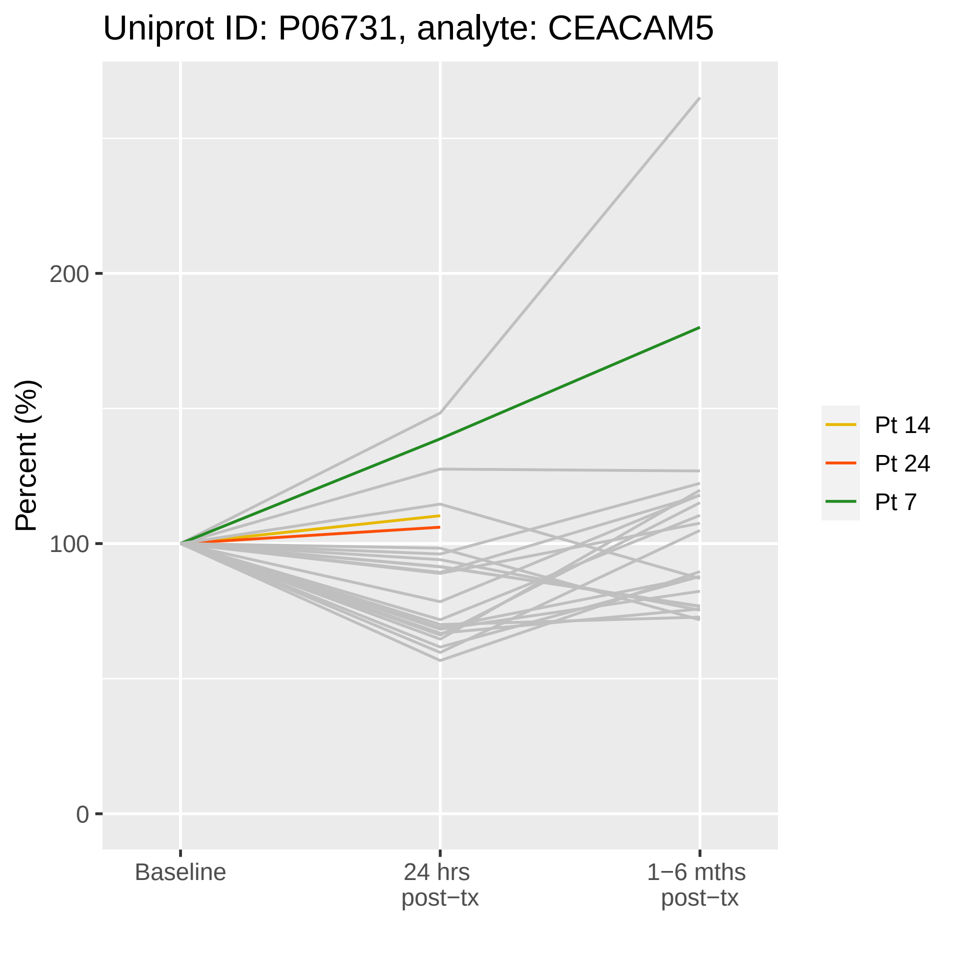

Uniprot ID: P06731, analyte: CEACAM5
Percent (%)
200
100
0
Baseline
24 hrs
post−tx
1−6 mths
post−tx
Pt 14
Pt 24
Pt 7

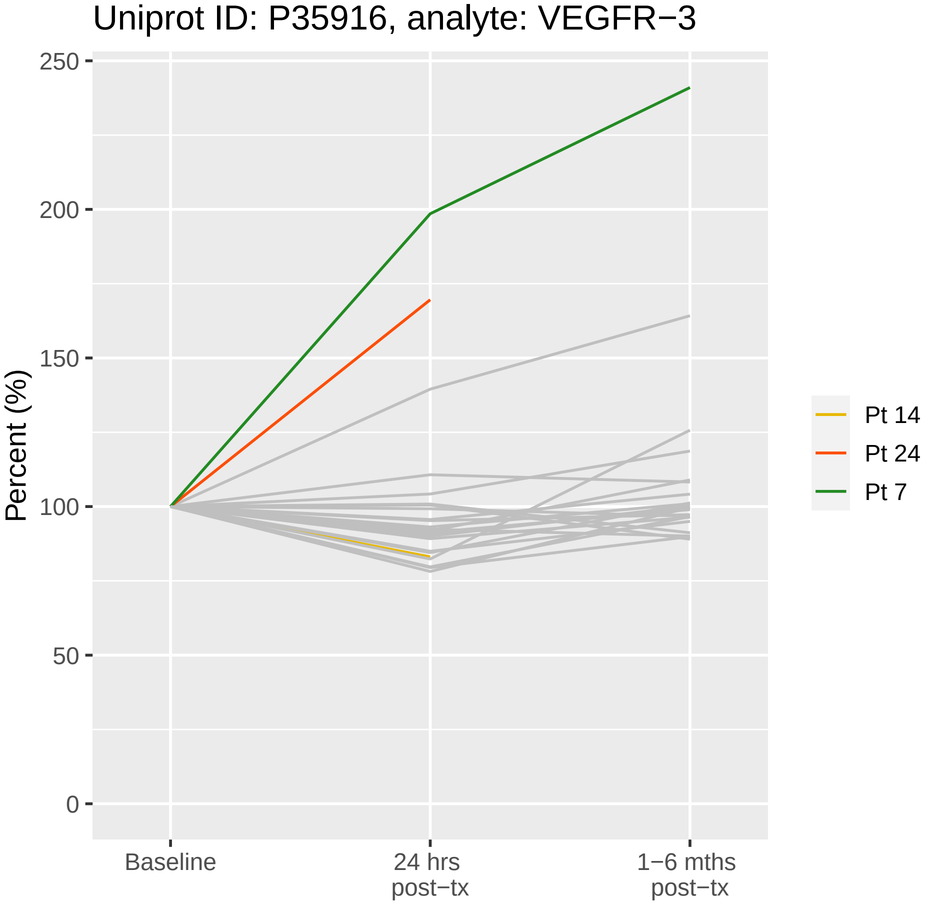

Uniprot ID: P35916, analyte: VEGFR−3
Percent (%)
250
200
150
100
50
0
Baseline
24 hrs post−tx
1−6 mths post−tx
Pt 14
Pt 24
Pt 7

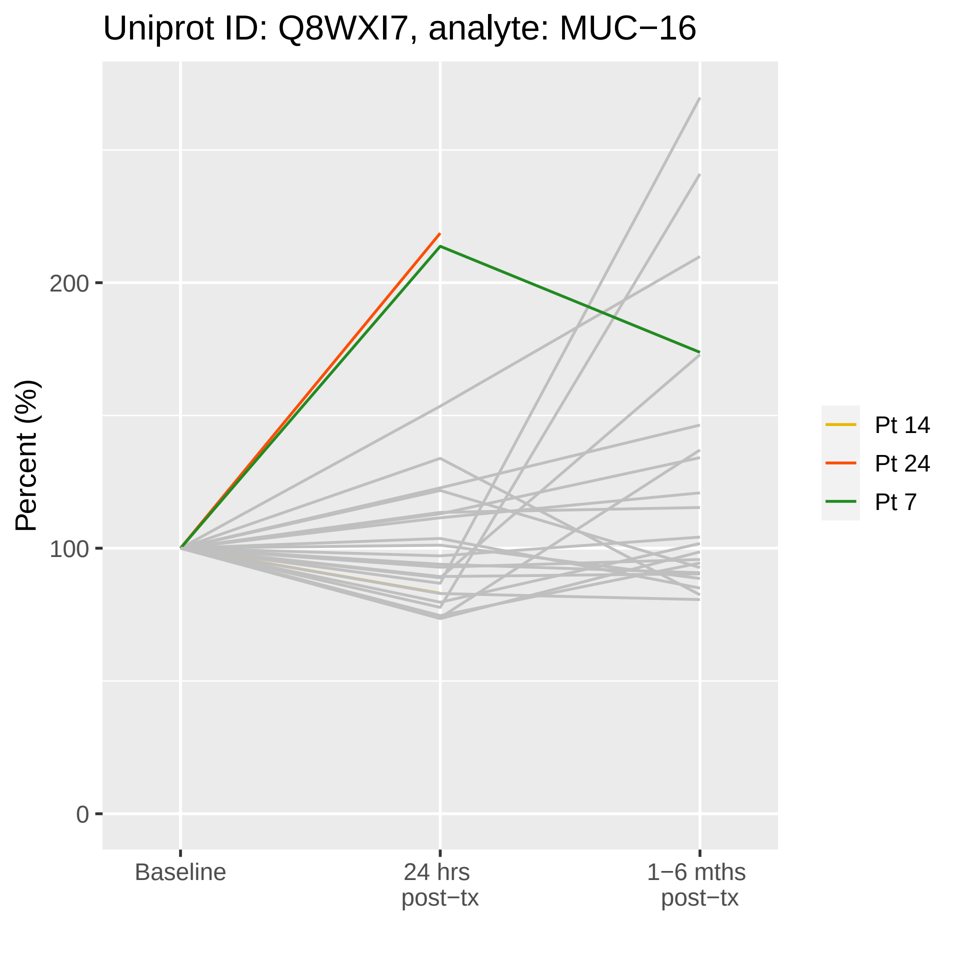
Uniprot ID: Q8WXI7, analyte: MUC−16
Percent (%)
200
100
0
Baseline
24 hrs
post−tx
1−6 mths
post−tx
Pt 14
Pt 24
Pt 7

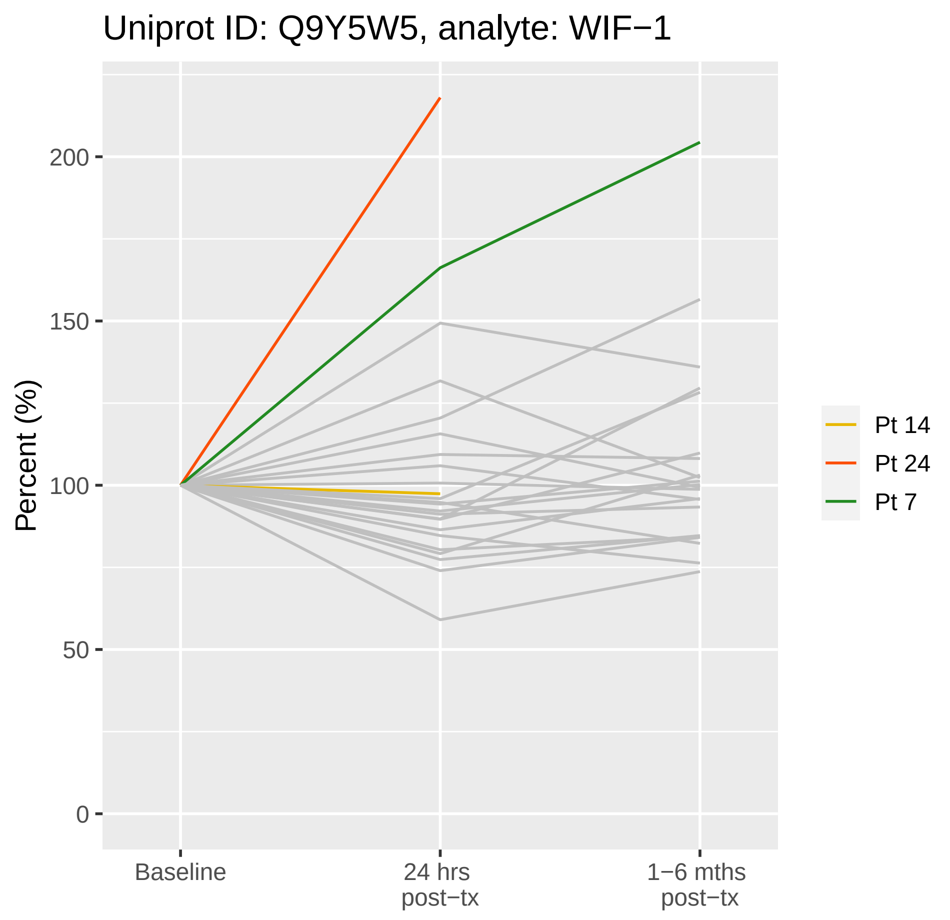

Uniprot ID: Q9Y5W5, analyte: WIF−1
Percent (%)
200
150
100
50
0
Baseline
24 hrs post−tx
1−6 mths post−tx
Pt 14
Pt 24
Pt 7

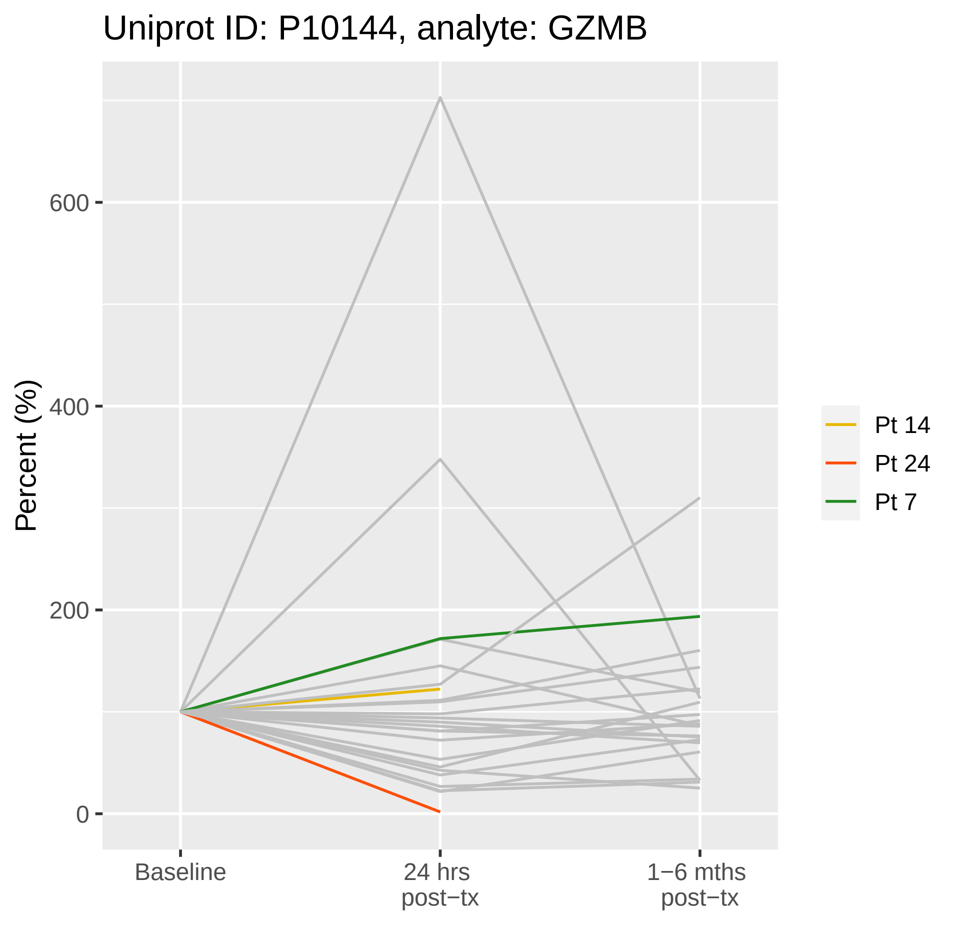
Uniprot ID: P10144, analyte: GZMB
Percent (%)
600
400
200
0
Baseline
24 hrs
post−tx
1−6 mths
post−tx
Pt 14
Pt 24
Pt 7

Uniprot ID: Q6BAA4, analyte: FCRLB

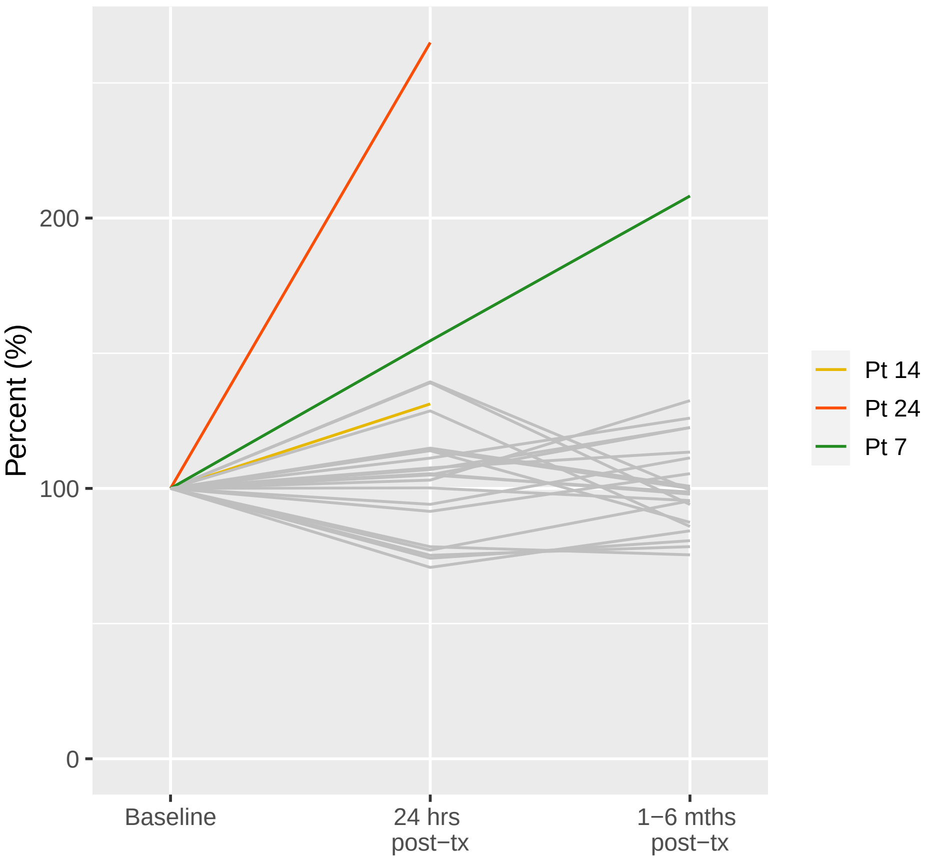

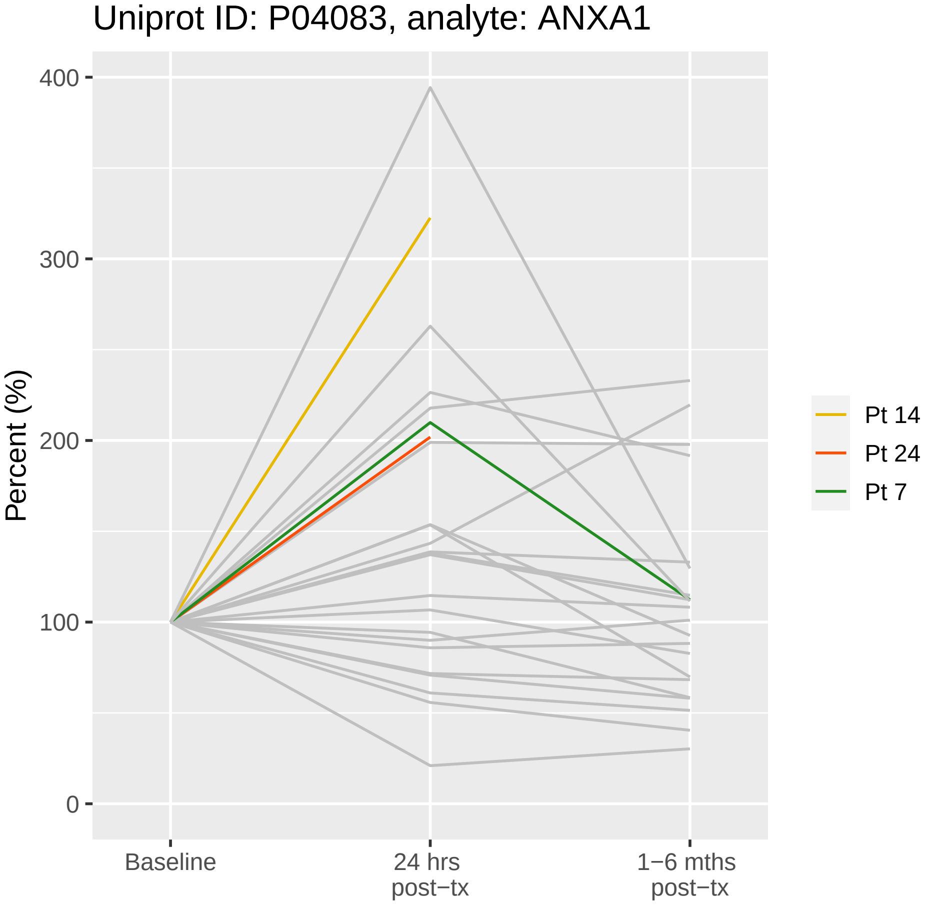
Uniprot ID: P04083, analyte: ANXA1
Percent (%)
400
300
200
100
0
Baseline
24 hrs post−tx
1−6 mths post−tx
Pt 14
Pt 24
Pt 7

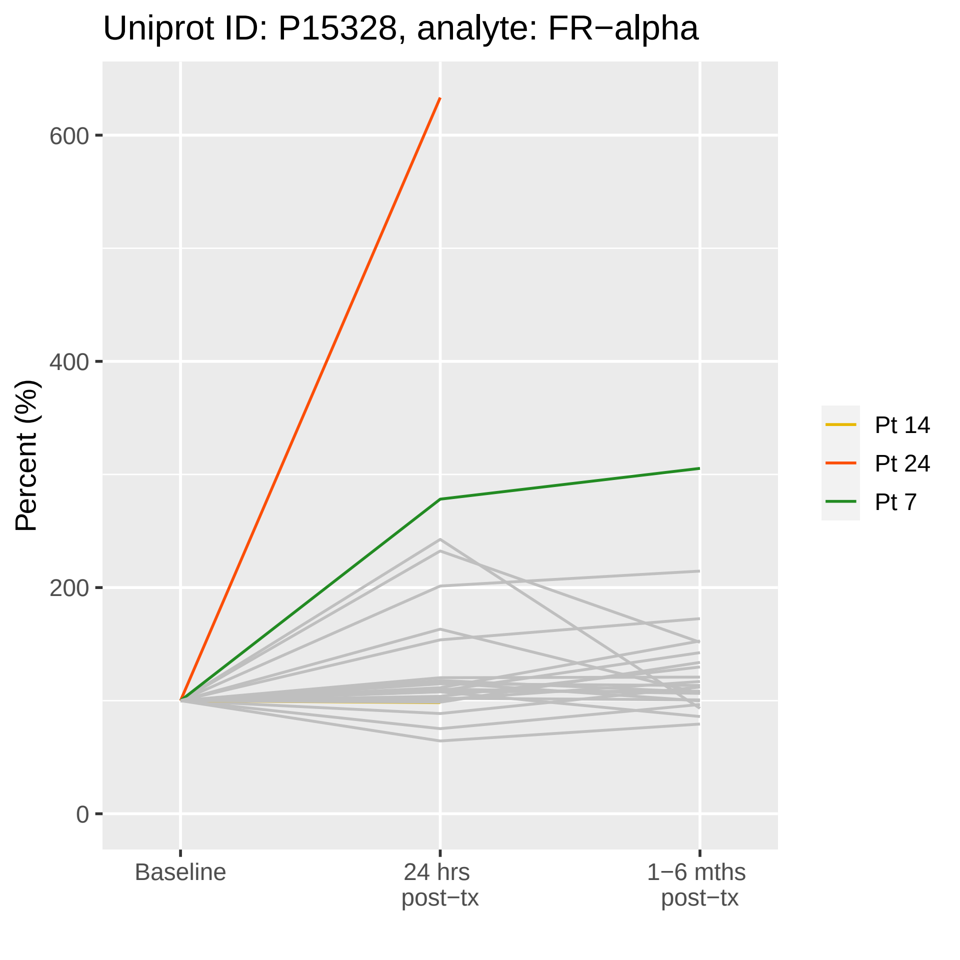
Uniprot ID: P15328, analyte: FR−alpha
Percent (%)
600
400
200
0
Baseline
24 hrs post−tx
1−6 mths post−tx
Pt 14
Pt 24
Pt 7
