# Supplementary material for: An Exploratory Analysis of Changes in Circulating Plasma Protein Profiles Following Image-Guided Ablation of Renal Tumours Provides Evidence for Effects on Multiple Biological Processes
Source: Cancers (Basel). 2021 Nov 30;13(23):6037. doi: 10.3390/cancers13236037 (PMC8656737; doi:10.3390/cancers13236037)
Supplement: Supplementary file 1 [file cancers-13-06037-s001.zip › Supplementary File/Figure S12.pdf]

Figure S12. Correlations between the 47 different proteins which changed significantly in patients 24 hours post-CRYO (n=24). a). Correlation matrix showing pair-wise correlations between the change in each protein with the scale bar gradation showing the correlation coefficients from 0 to 1 and blue and red indicating positive and negative correlations respectively; b). Examples of the most strongly correlated protein biomarkers which changed significantly post-CRYO

Figure S6a

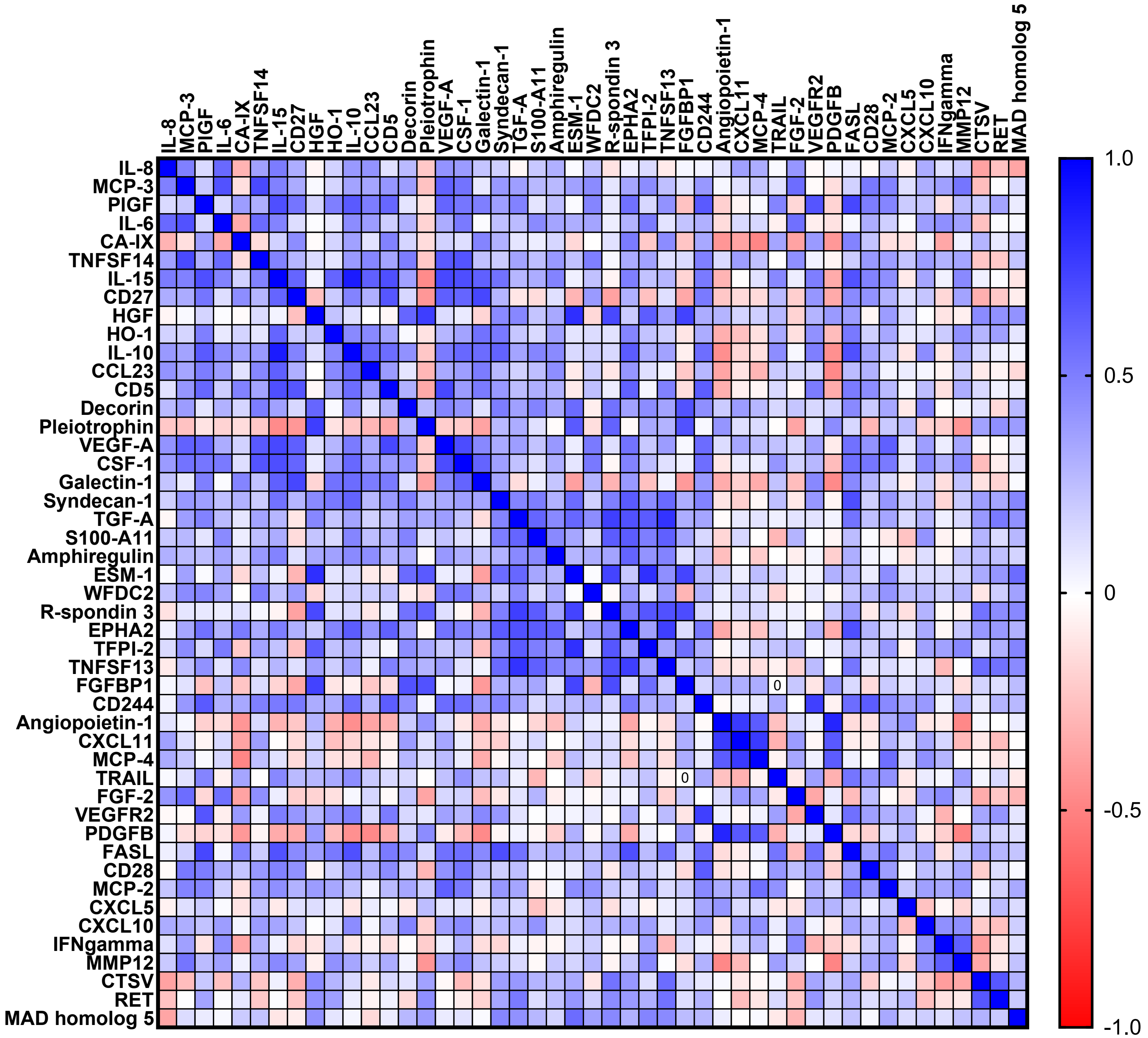
IL-8
MCP-3
PIGF
IL-6
CA-IX
TNFSF14
IL-15
CD27
HGF
HO-1
IL-10
CCL23
CD5
Decorin
Pleiotrophin
VEGF-A
CSF-1
Galectin-1
Syndecan-1
TGF-A
S100-A11
Amphiregulin
ESM-1
WFDC2
R-spondin 3
EPHA2
TFPI-2
TNFSF13
FGFBP1
CD244
Angiopoietin-1
CXCL11
MCP-4
TRAIL
FGF-2
VEGFR2
PDGFB
FASL
CD28
MCP-2
CXCL5
CXCL10
IFNgamma
MMP12
CTSV
RET
MAD homolog 5
0
0
1.0
0.5
0
-0.5
-1.0

Figure S6b

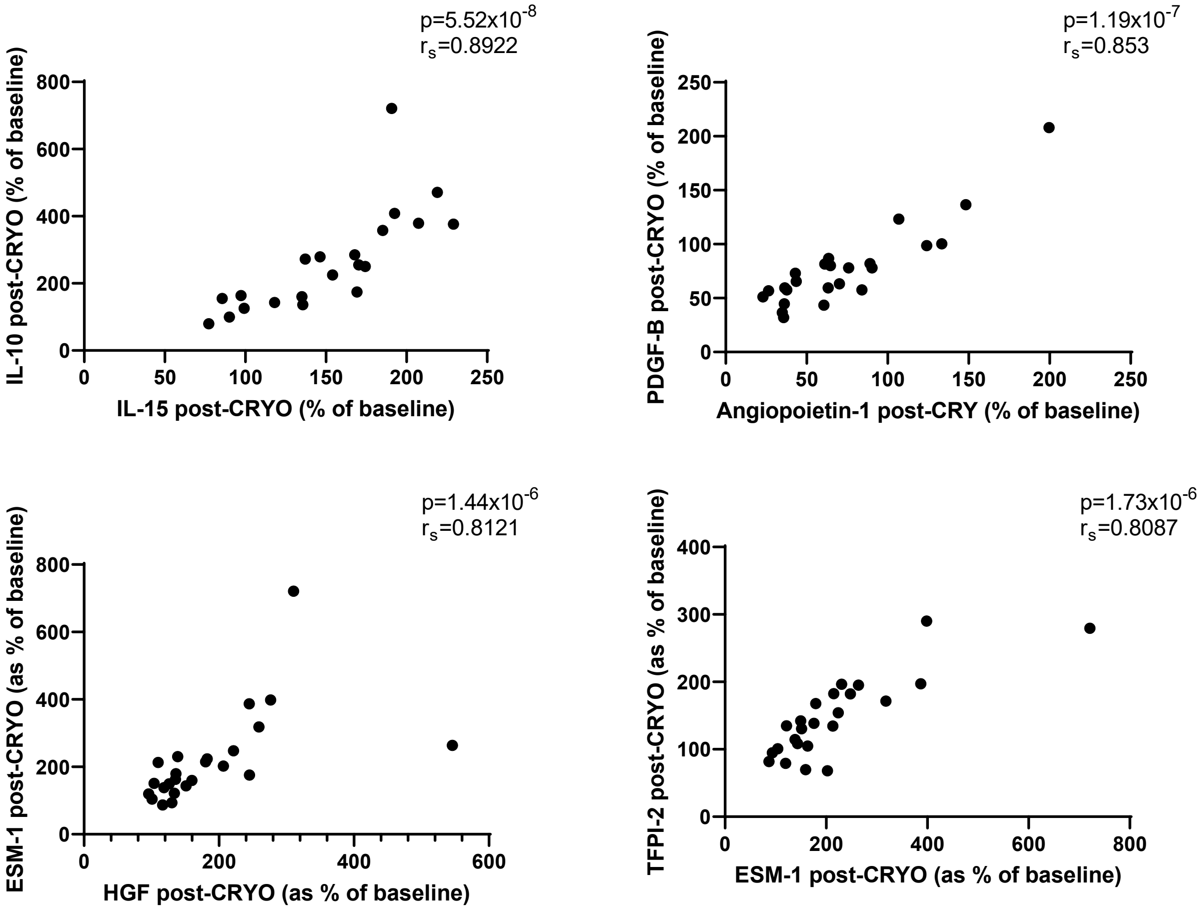

p=5.52x10-8
rs=0.8922
IL-10 post-CRYO (% of baseline)
IL-15 post-CRYO (% of baseline)
p=1.19x10-7
rs=0.853
PDGF-B post-CRYO (% of baseline)
Angiopoietin-1 post-CRY (% of baseline)
p=1.44x10-6
rs=0.8121
ESM-1 post-CRYO (as % of baseline)
HGF post-CRYO (as % of baseline)
p=1.73x10-6
rs=0.8087
TFPI-2 post-CRYO (as % of baseline)
ESM-1 post-CRYO (as % of baseline)
