# Supplementary material for: An Exploratory Analysis of Changes in Circulating Plasma Protein Profiles Following Image-Guided Ablation of Renal Tumours Provides Evidence for Effects on Multiple Biological Processes
Source: Cancers (Basel). 2021 Nov 30;13(23):6037. doi: 10.3390/cancers13236037 (PMC8656737; doi:10.3390/cancers13236037)

Figure S2. Longitudinal profiles of all plasma proteins on the Oncology panel, prior to and post-ablation. Results for each protein are shown as linear NPX values and are shown for patients receiving CRYO (n=25), MWA (n=2) and RFA (n=8) and as a single timepoint for healthy controls (n=11). Dotted line indicates lower limit of quantification

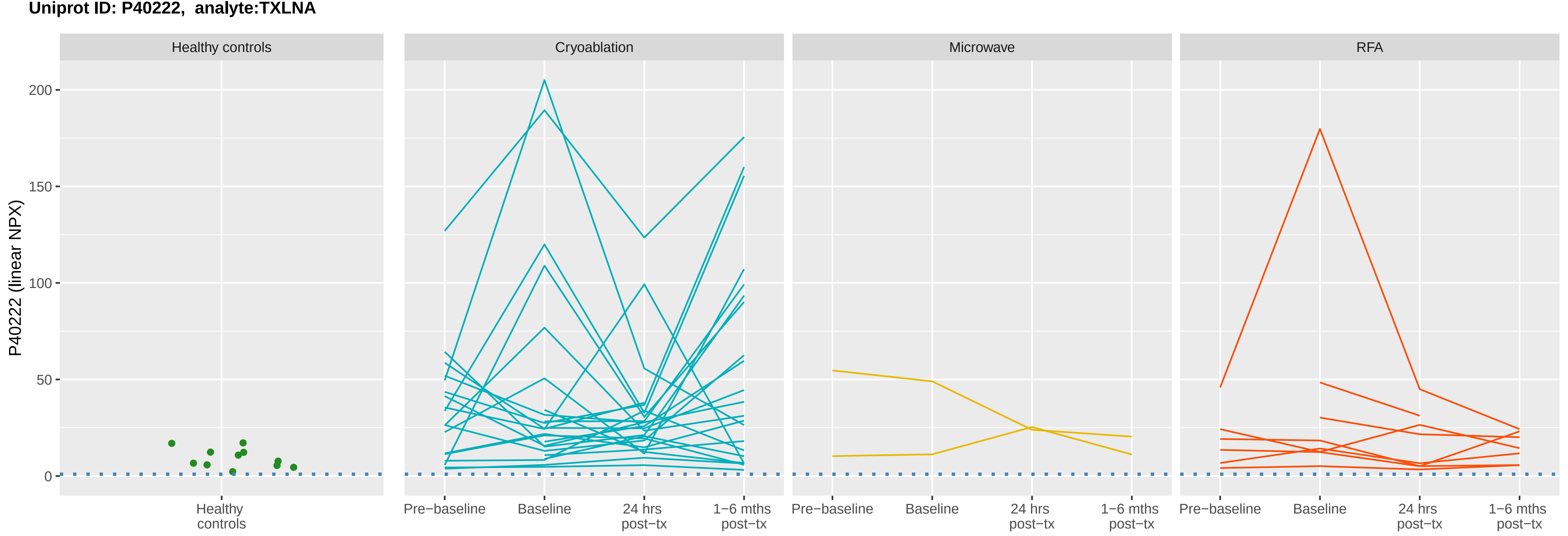

Uniprot ID: P40222, analyte:TXLNA
Healthy controls
Cryoablation
Microwave
RFA
P40222 (linear NPX)
200
150
100
50
0
Healthy controls
Pre−baseline
Baseline
24 hrs post−tx
1−6 mths post−tx

Uniprot ID: P15692, analyte:VEGFA

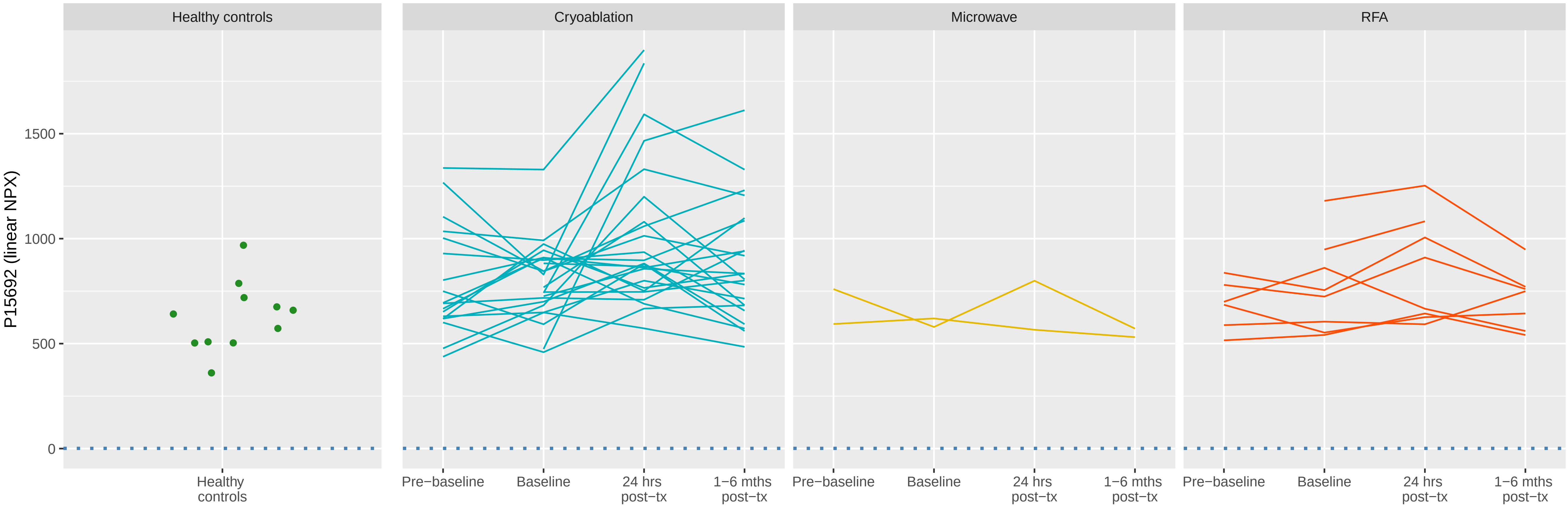

Uniprot ID: P16870, analyte:CPE

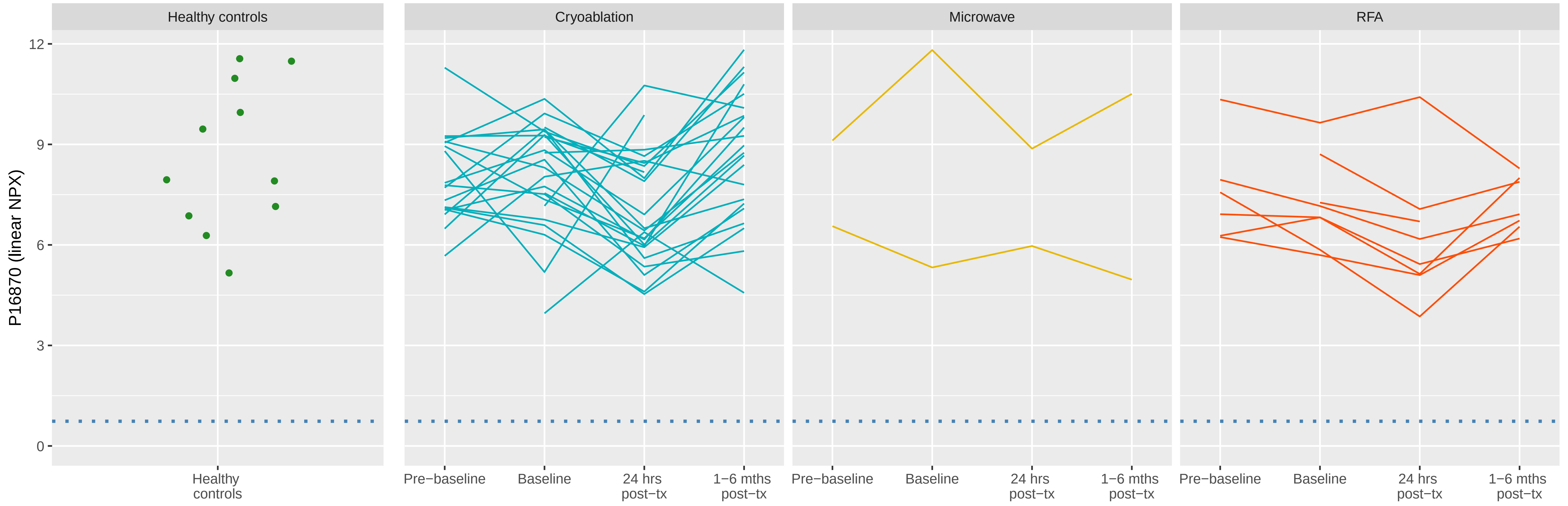

Uniprot ID: Q9UKR3, analyte:KLK13

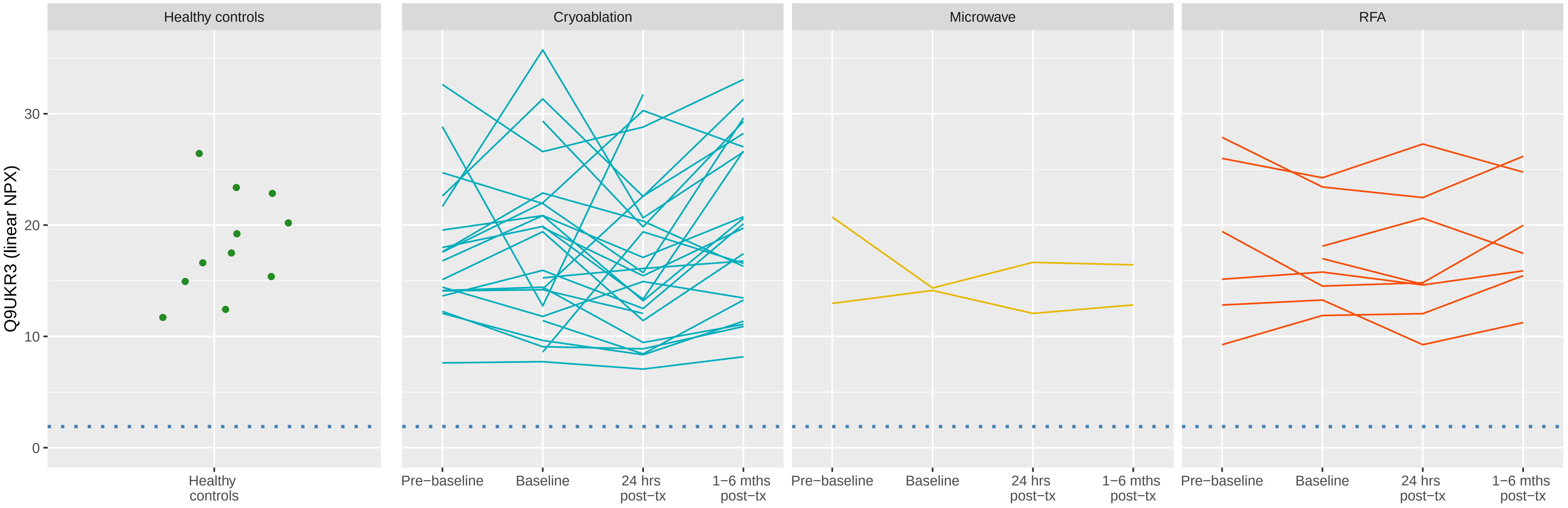

Uniprot ID: P13688, analyte:CEACAM1

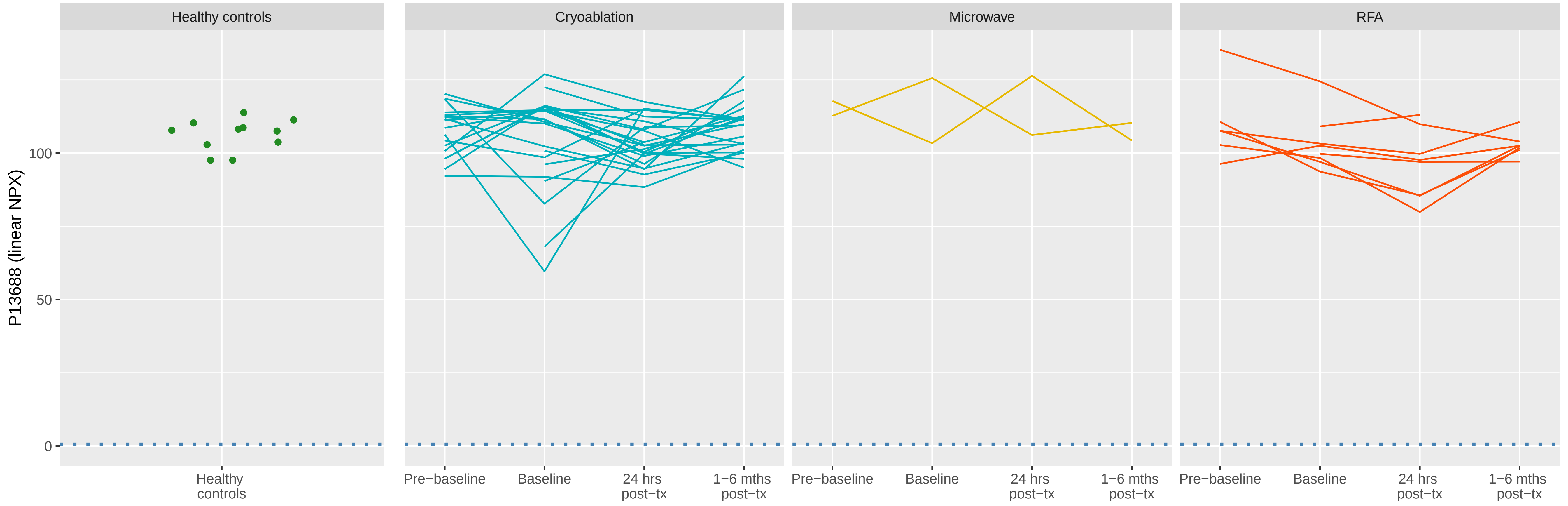

Uniprot ID: Q13421, analyte:MSLN

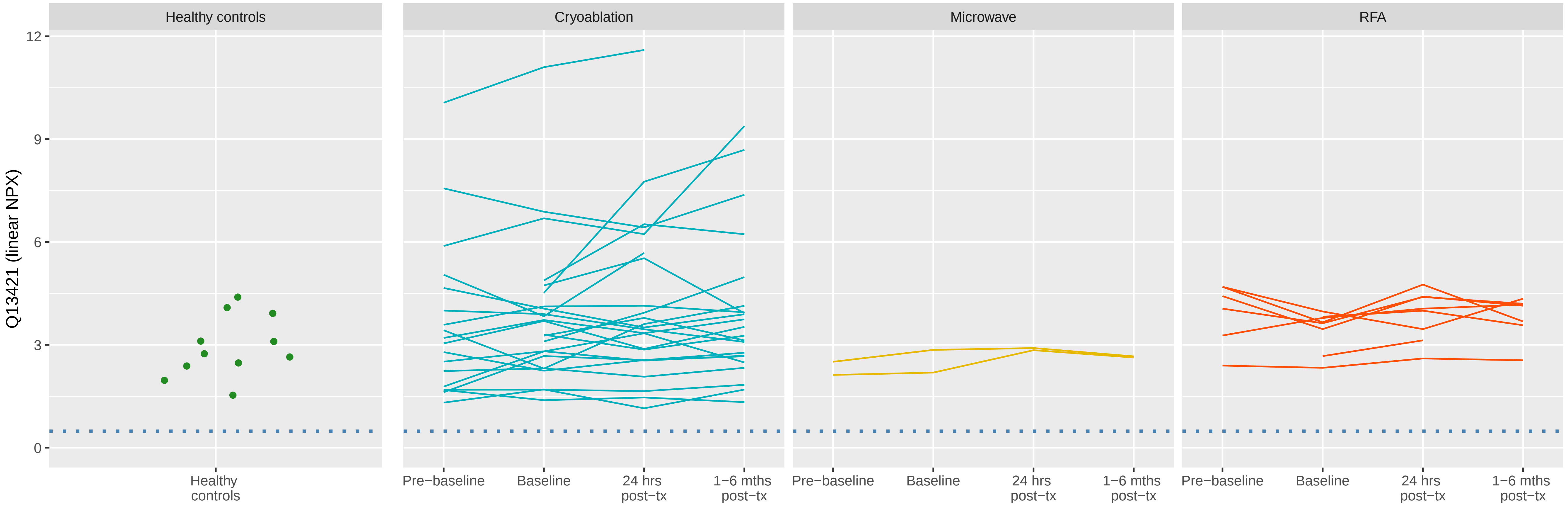

Uniprot ID: O75888, analyte:TNFSF13

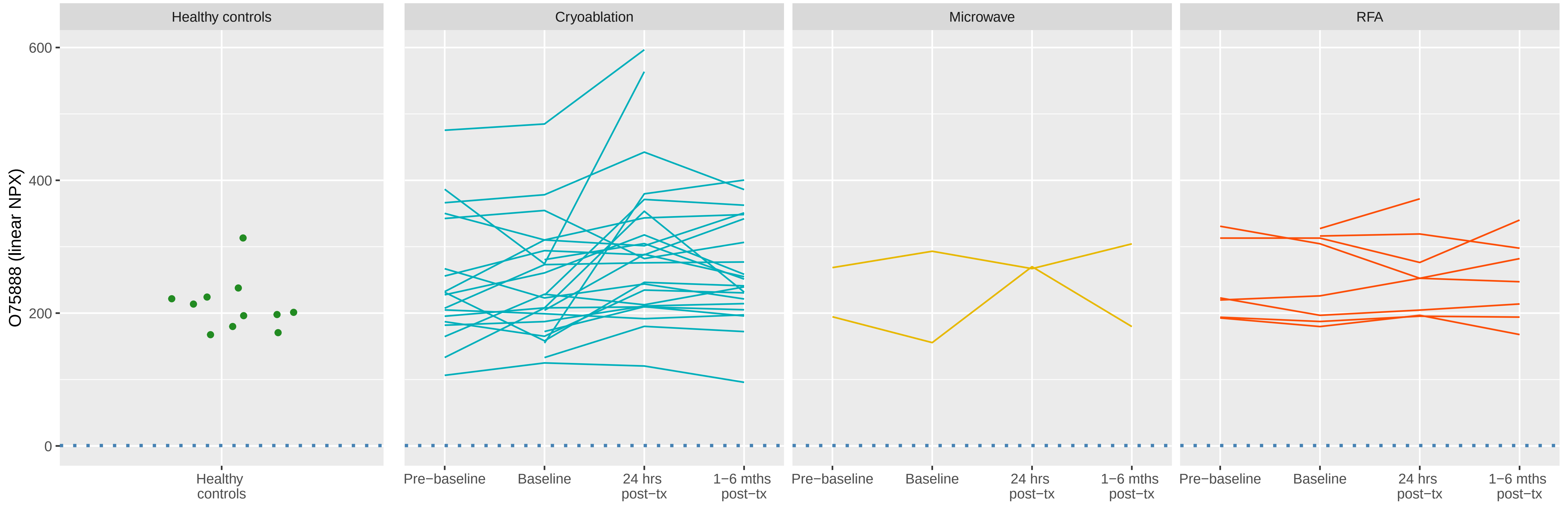

Uniprot ID: P01133, analyte:EGF

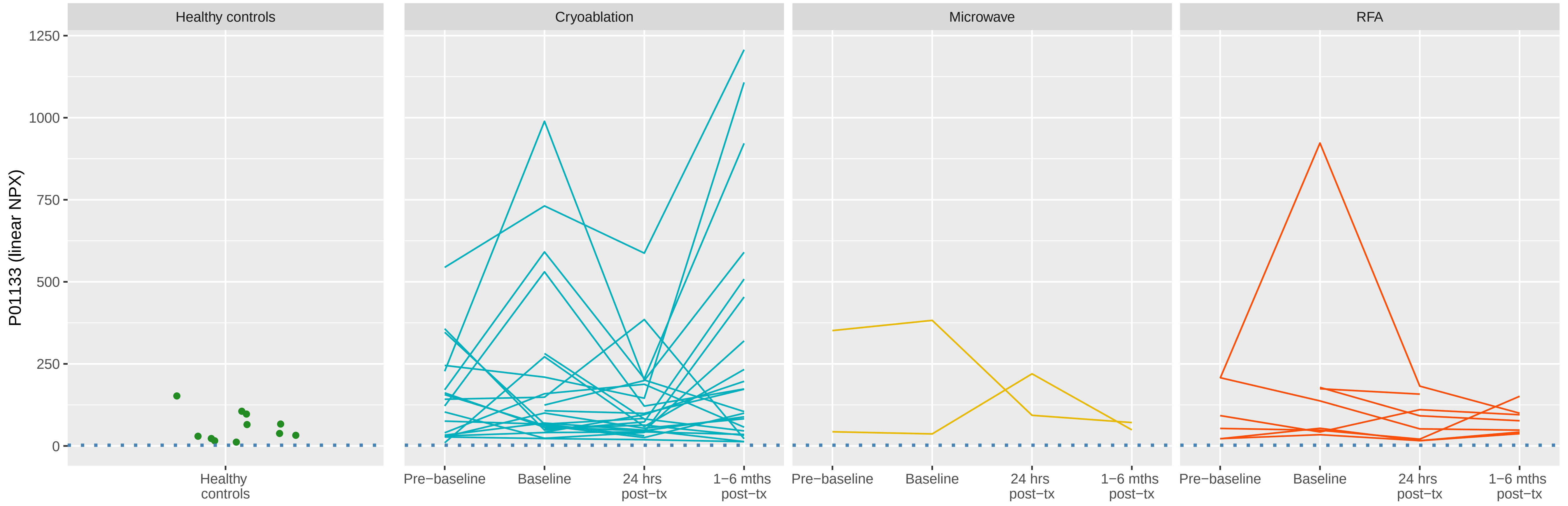

Uniprot ID: O95407, analyte:TNFRSF6B

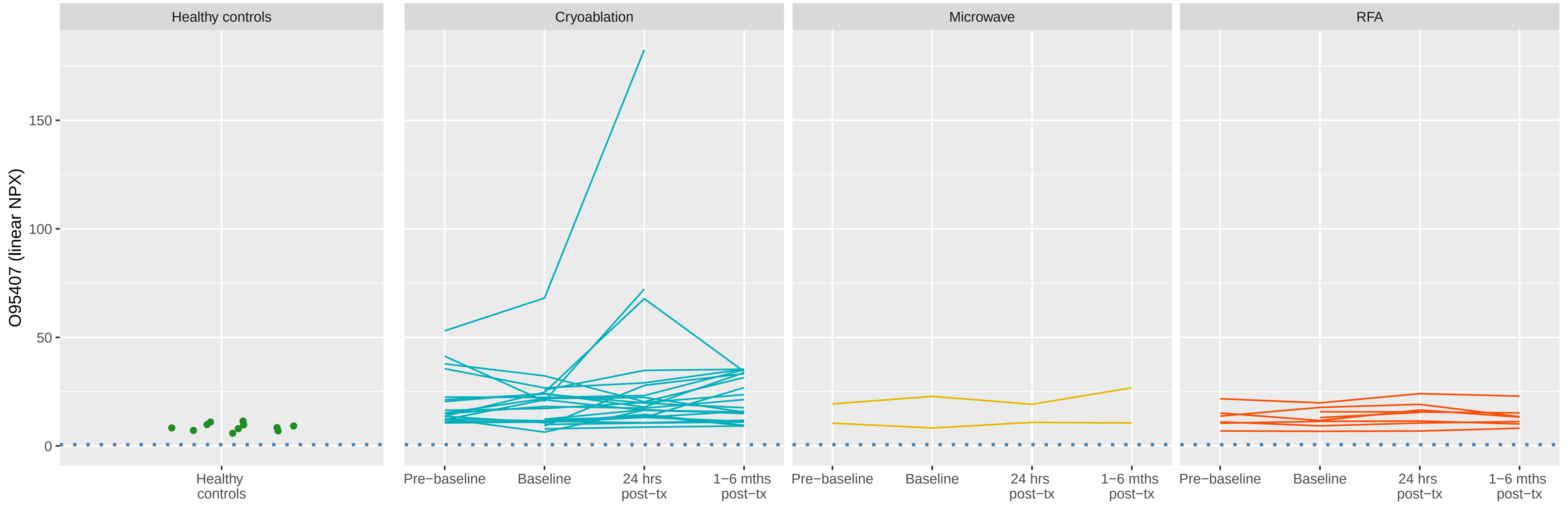

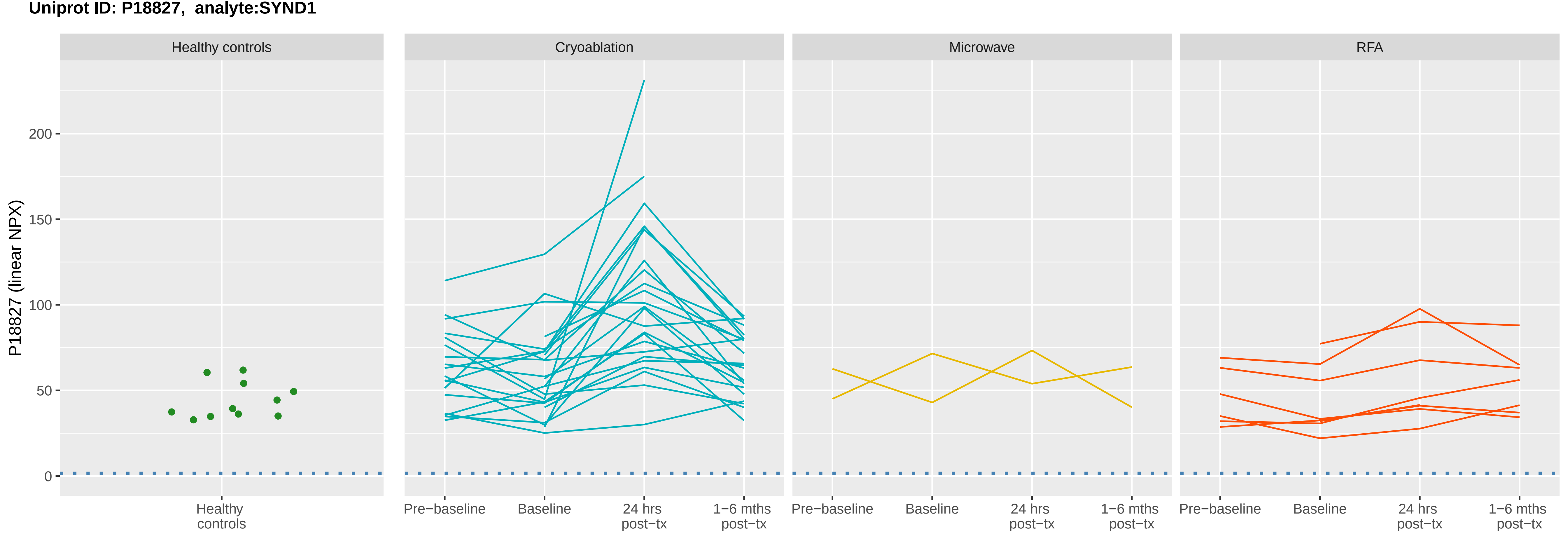
Uniprot ID: P18827, analyte:SYND1
Healthy controls
Cryoablation
Microwave
RFA
P18827 (linear NPX)
0
50
100
150
200
Healthy controls
Pre−baseline
Baseline
24 hrs post−tx
1−6 mths post−tx

Uniprot ID: P37173, analyte:TGFR−2

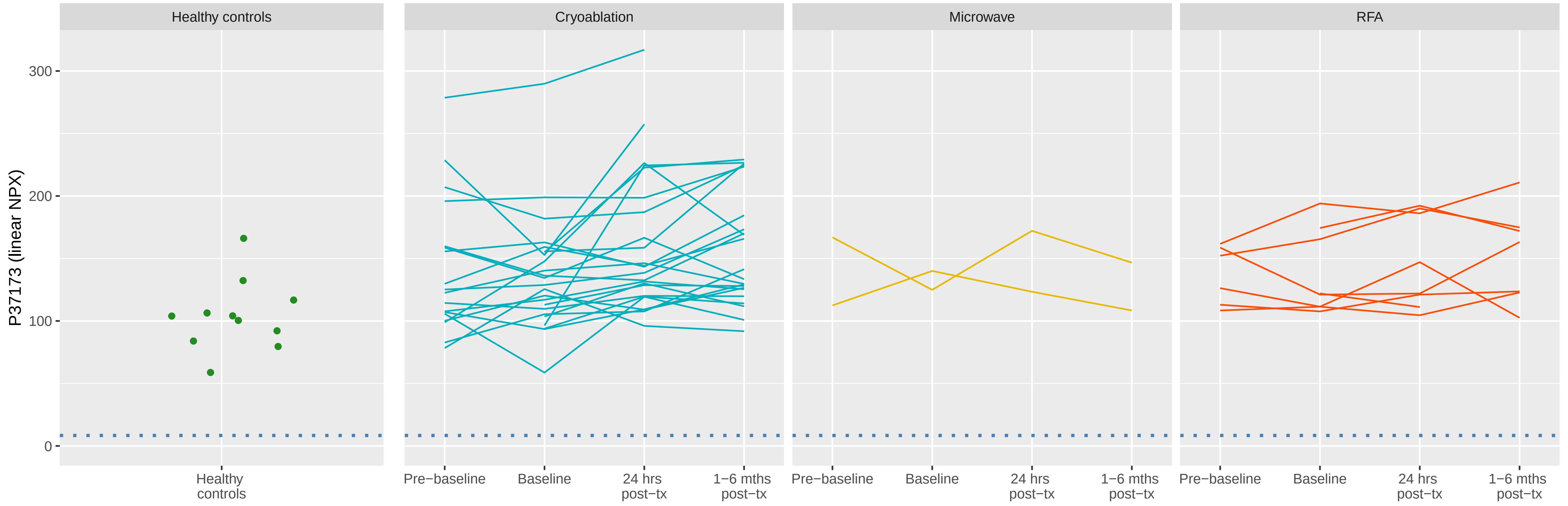

Uniprot ID: P05231, analyte:IL6

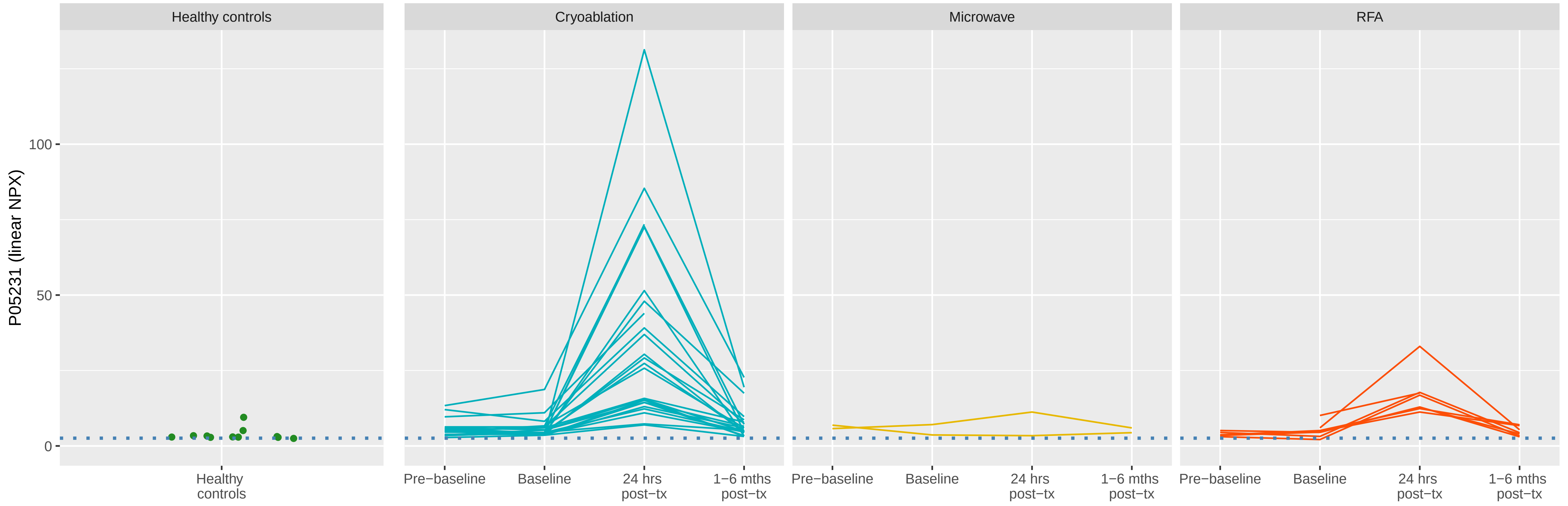

Uniprot ID: P09326, analyte:CD48

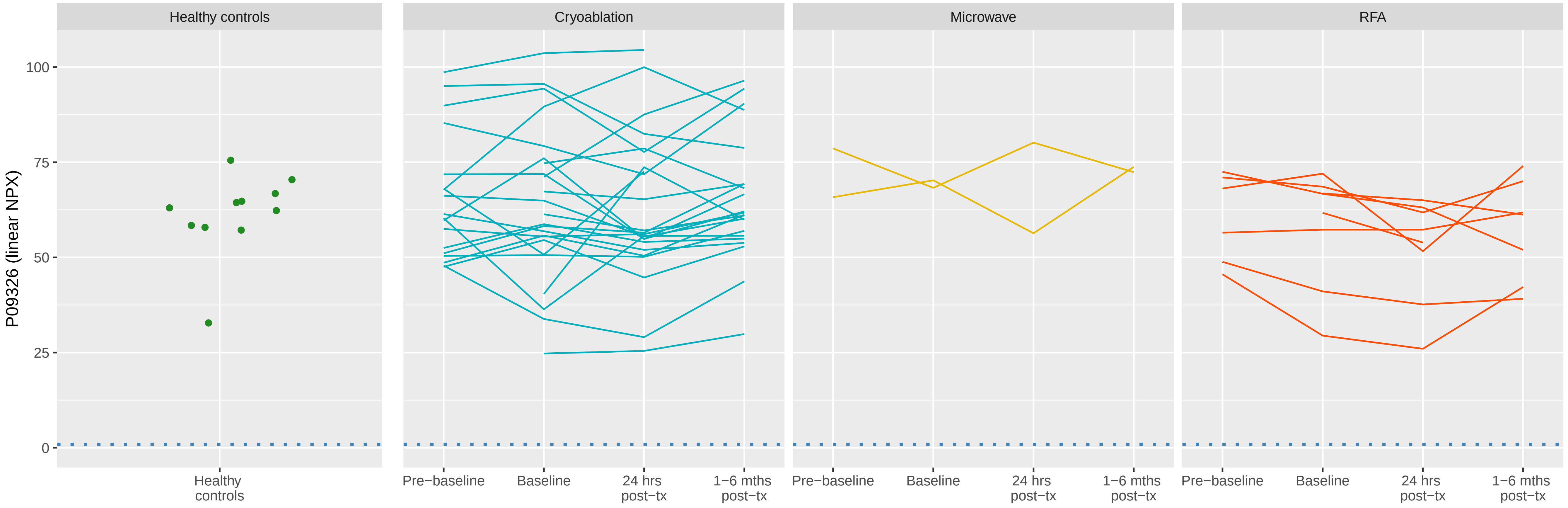

Uniprot ID: O14828, analyte:SCAMP3

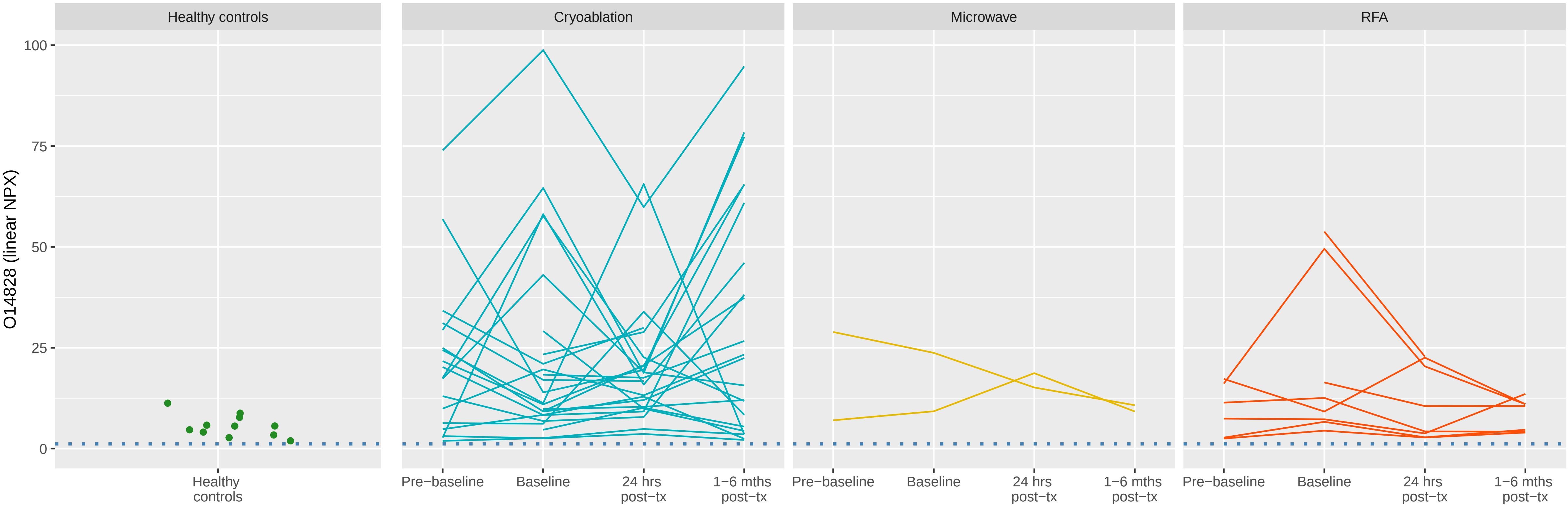

Uniprot ID: Q9HBG7, analyte:LY9

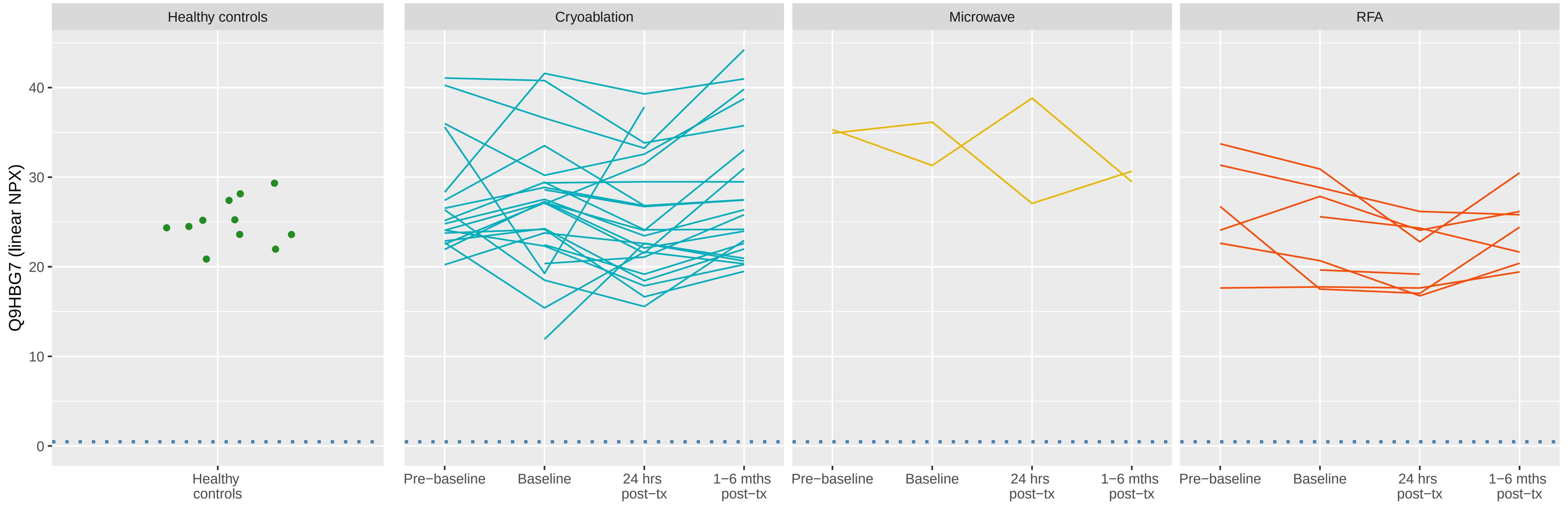

Uniprot ID: P15260, analyte:IFN−gamma−R1

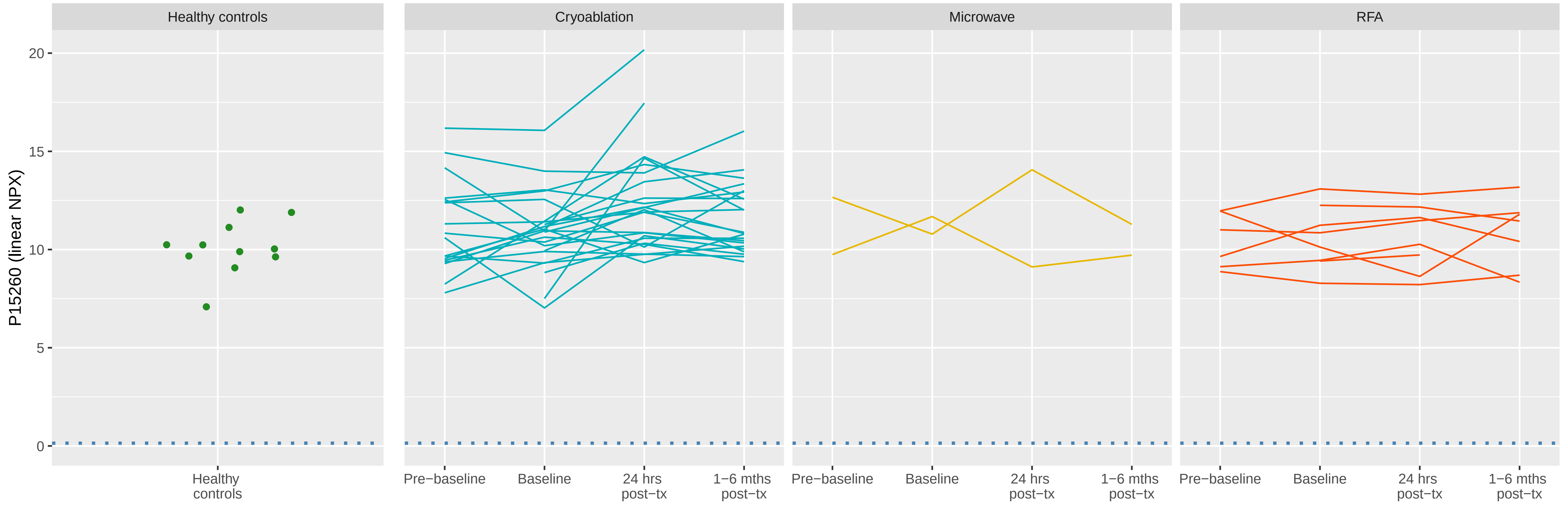

Uniprot ID: P06756, analyte:ITGAV

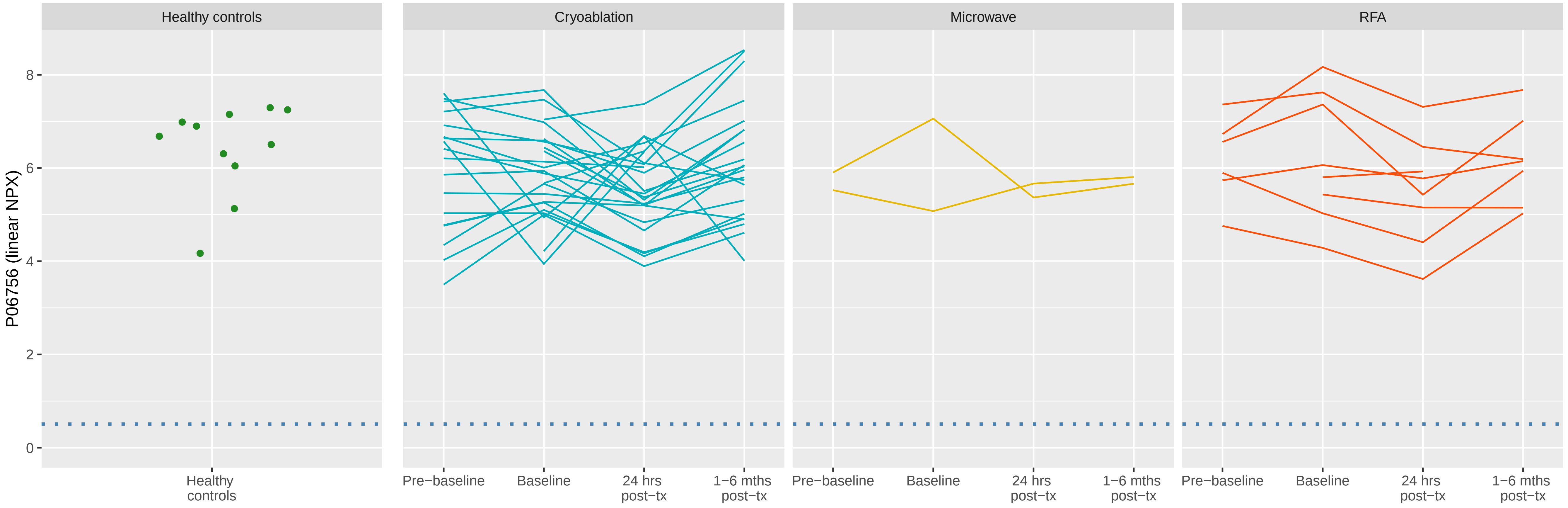

Uniprot ID: P50591, analyte:TRAIL

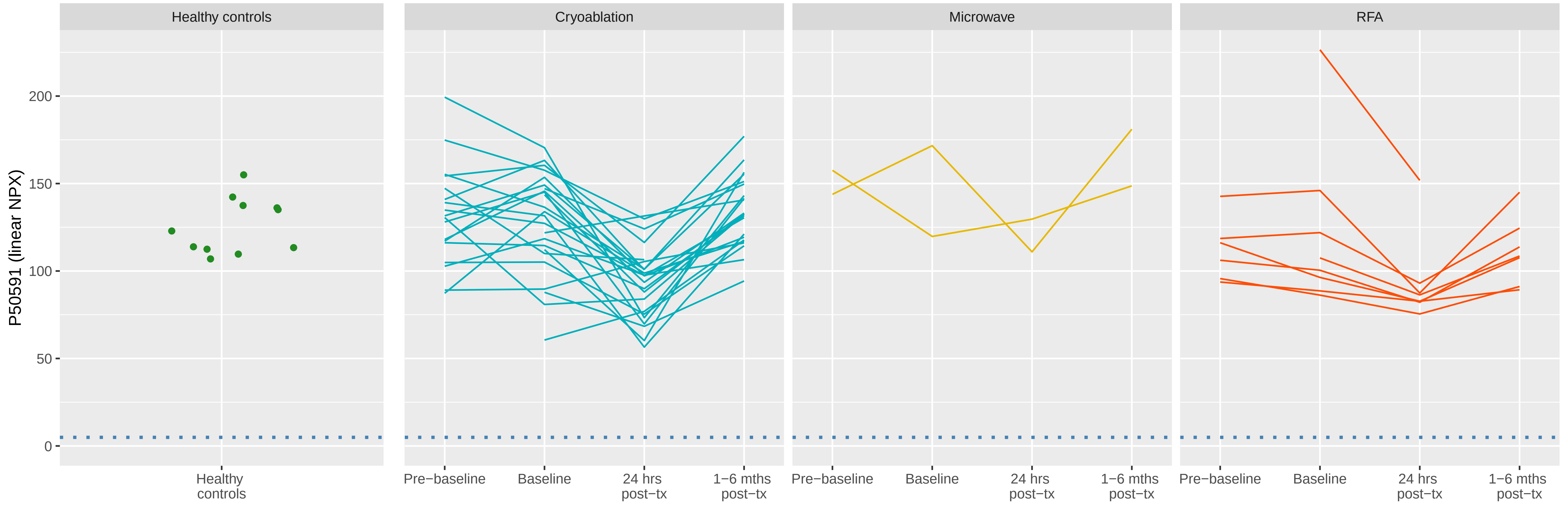

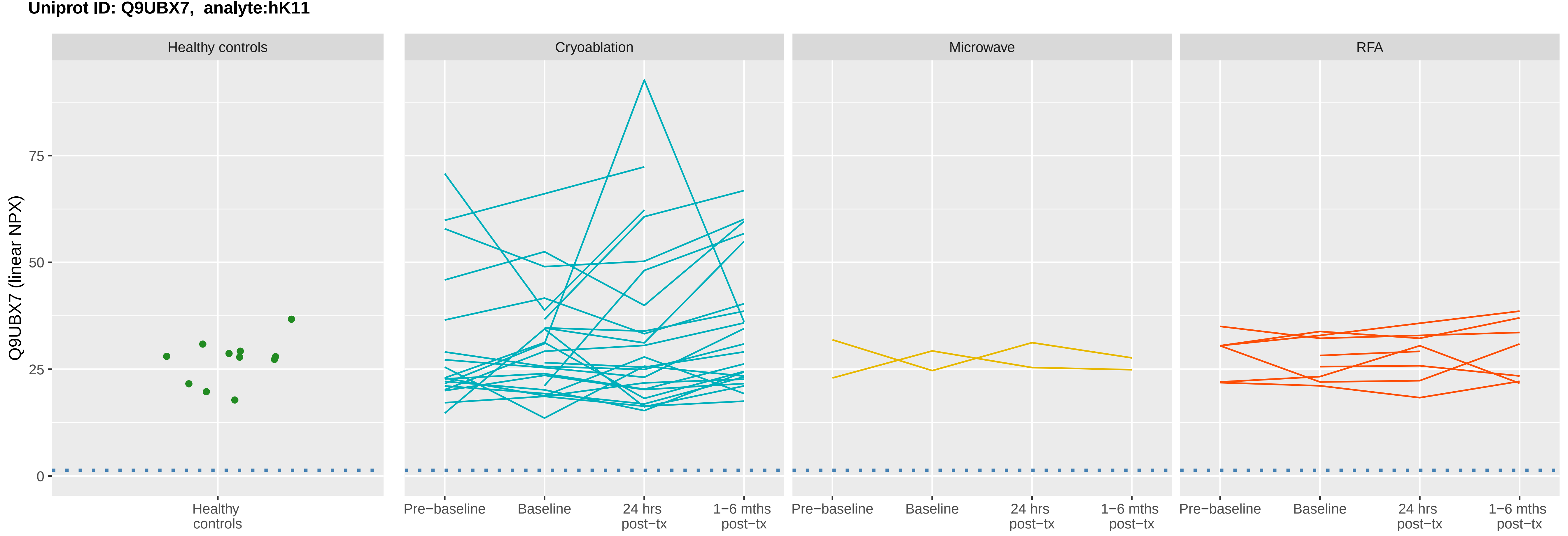

Uniprot ID: Q9UBX7, analyte:hK11
Healthy controls
Cryoablation
Microwave
RFA
Q9UBX7 (linear NPX)
75
50
25
0
Healthy controls
Pre−baseline
Baseline
24 hrs post−tx
1−6 mths post−tx

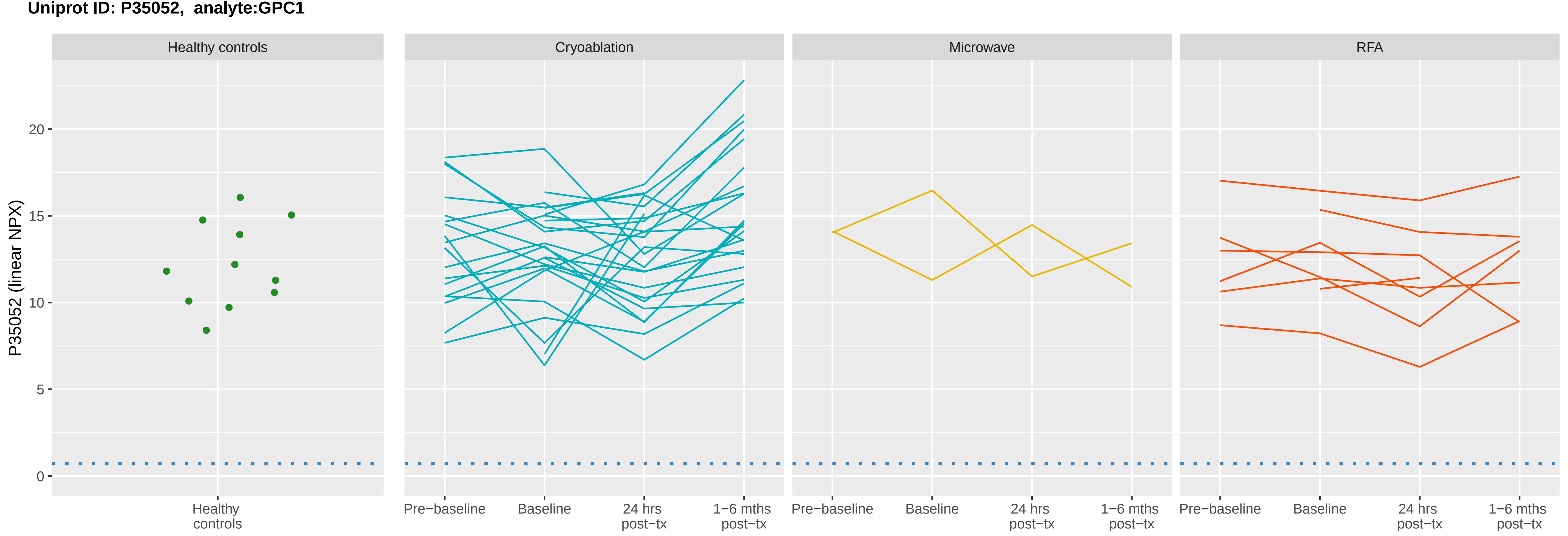

Uniprot ID: P35052, analyte:GPC1
Healthy controls
Cryoablation
Microwave
RFA
P35052 (linear NPX)
0
5
10
15
20
Healthy controls
Pre−baseline
Baseline
24 hrs post−tx
1−6 mths post−tx

Uniprot ID: P48307, analyte:TFPI−2

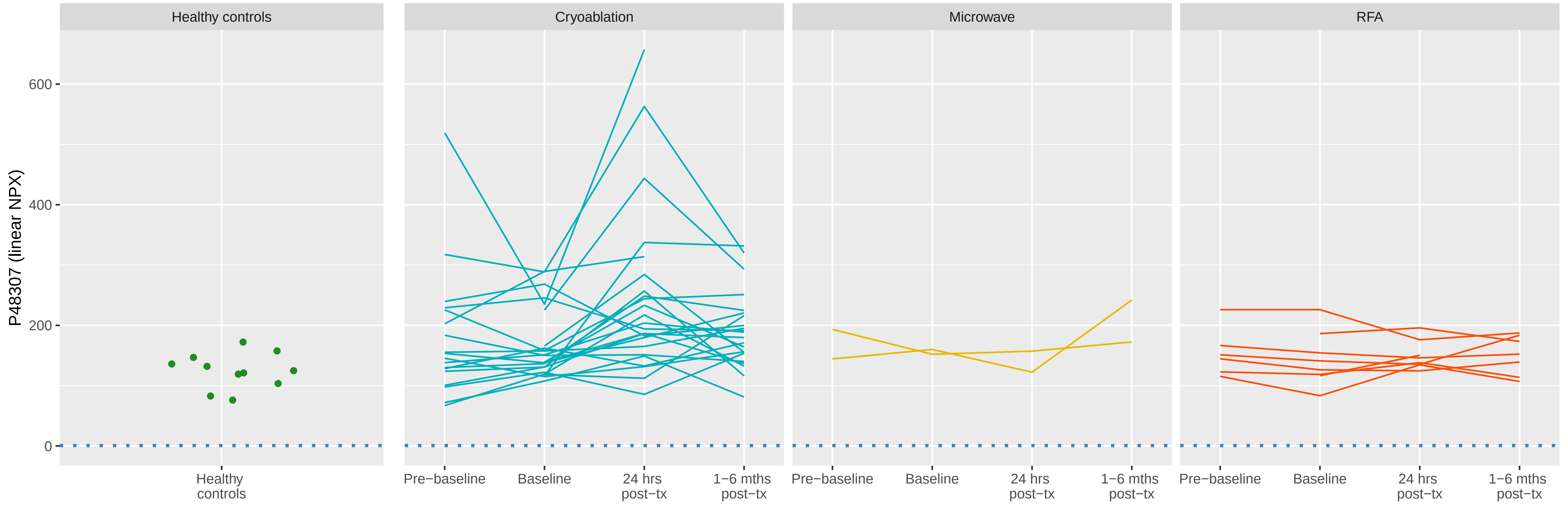

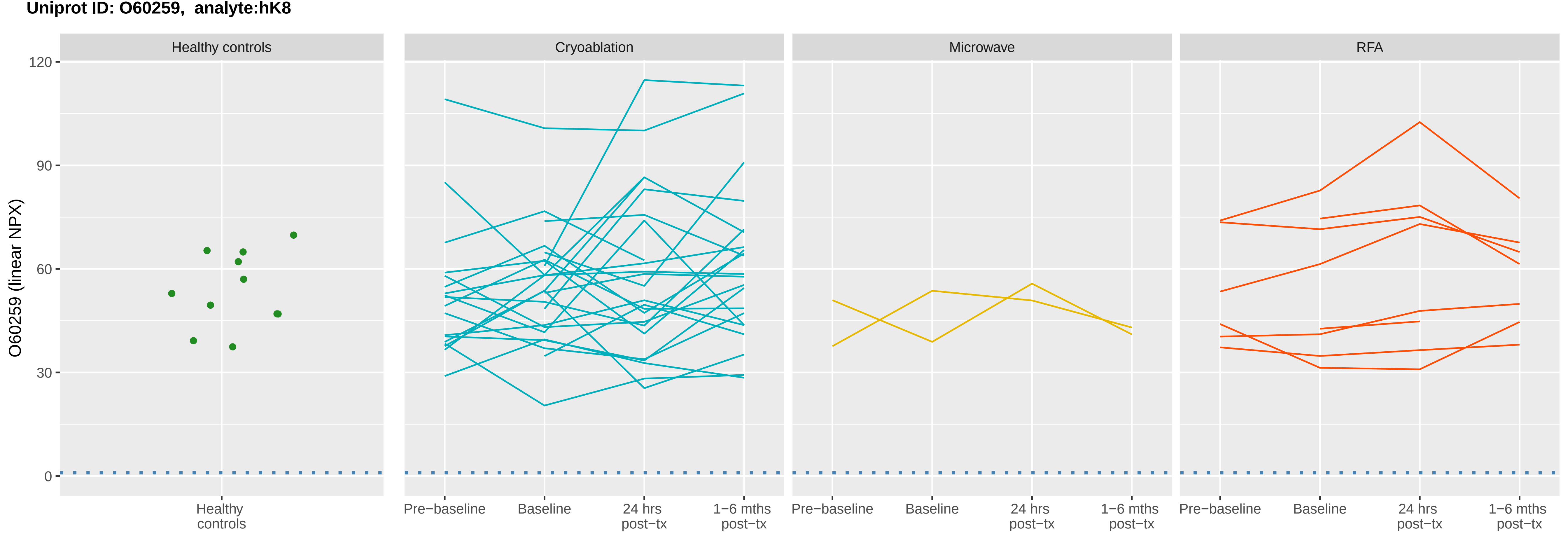
Uniprot ID: O60259, analyte:hK8
Healthy controls
Cryoablation
Microwave
RFA
O60259 (linear NPX)
120
90
60
30
0
Healthy controls
Pre−baseline
Baseline
24 hrs post−tx
1−6 mths post−tx

Uniprot ID: P35968, analyte:VEGFR−2

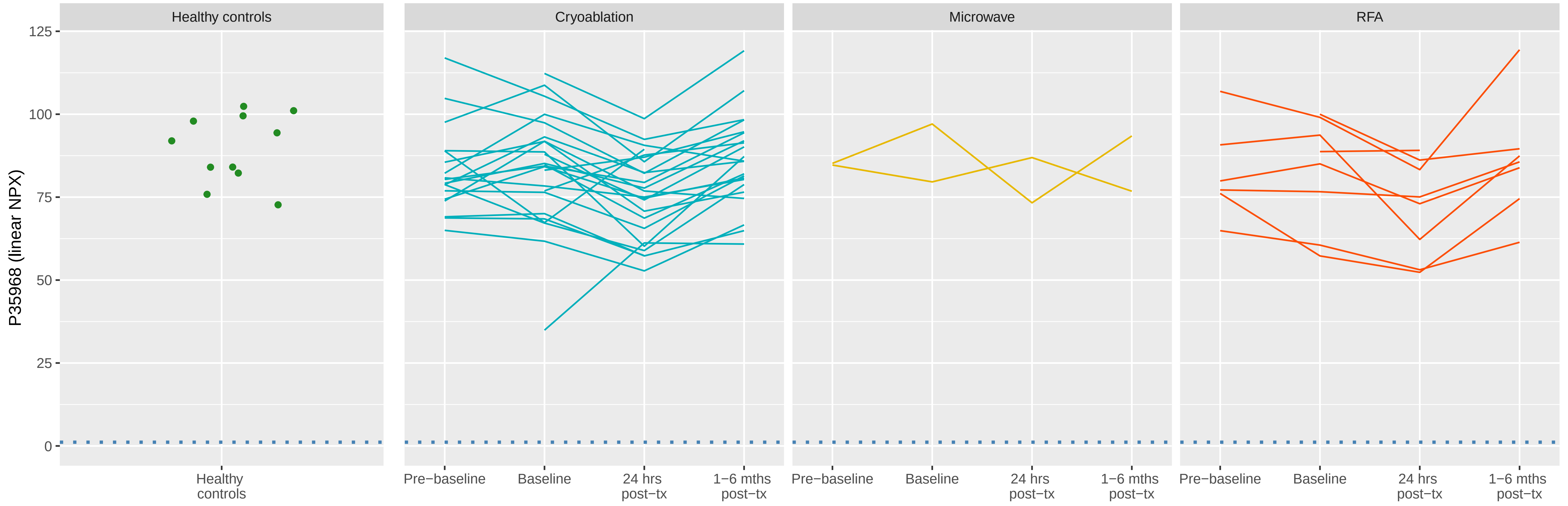

Uniprot ID: O95274, analyte:LYPD3

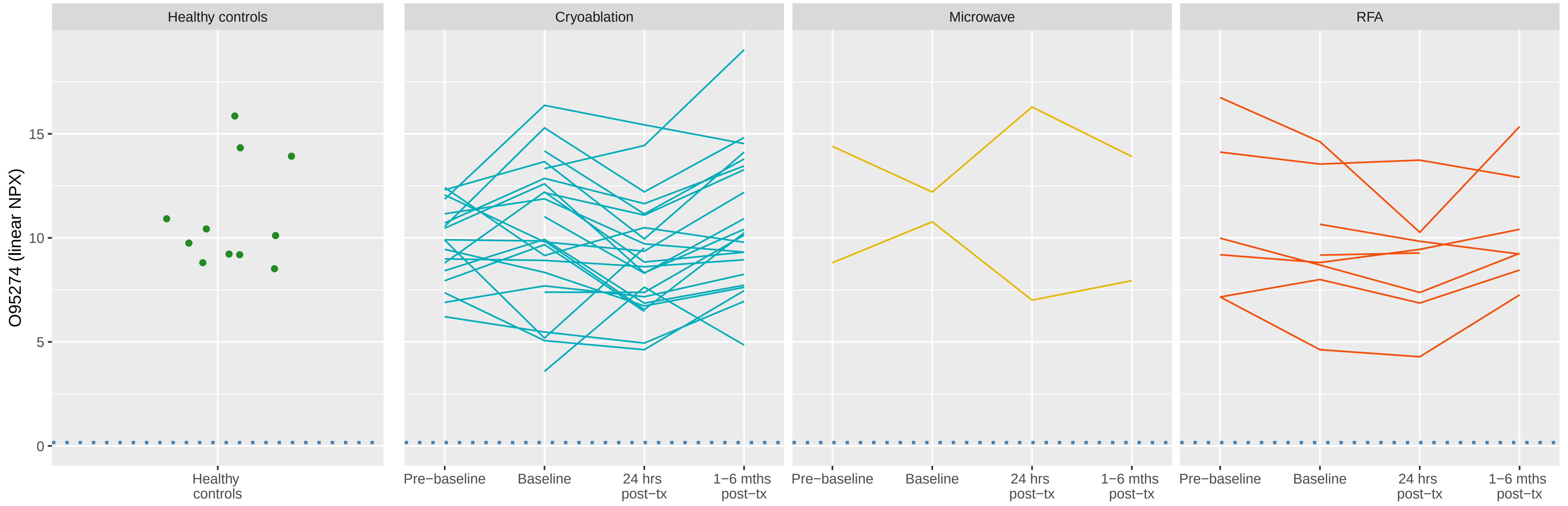

Uniprot ID: O00592, analyte:PODXL

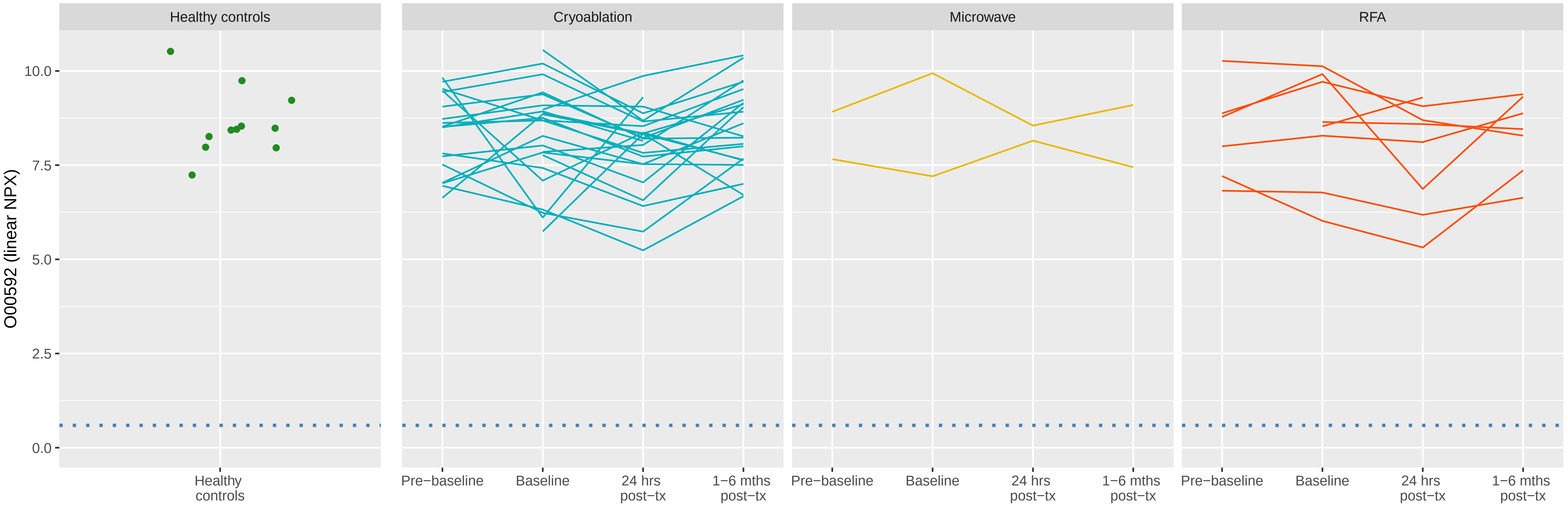

Uniprot ID: P26447, analyte:S100A4

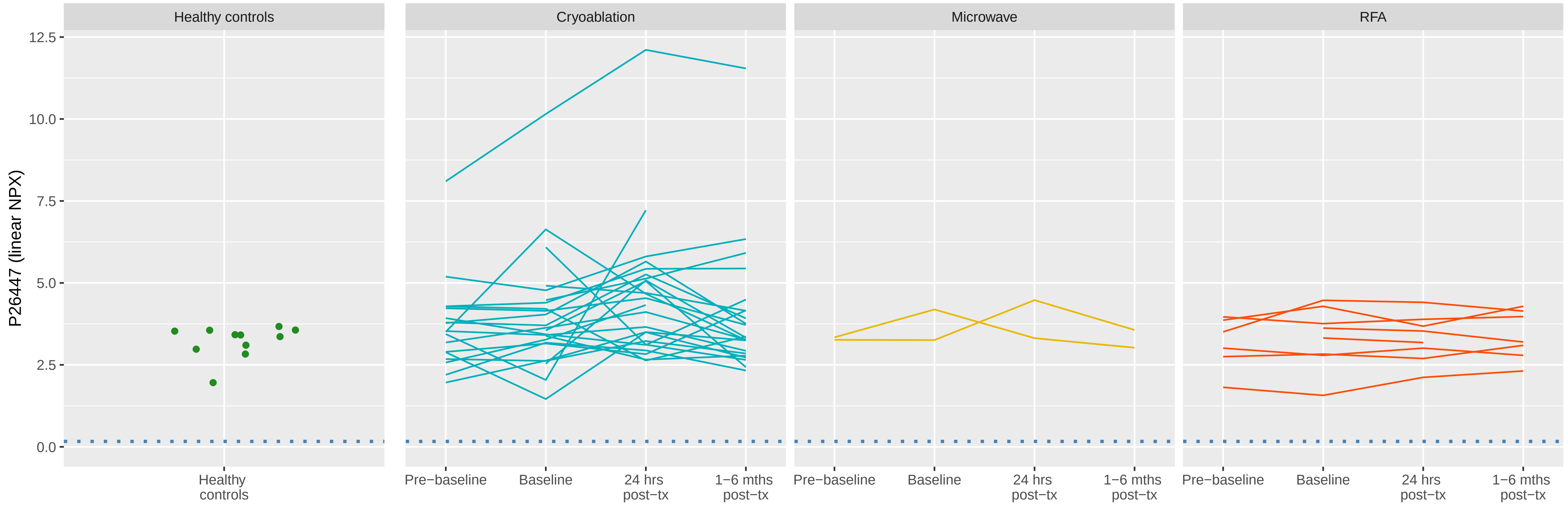

Uniprot ID: P08069, analyte:IGF1R

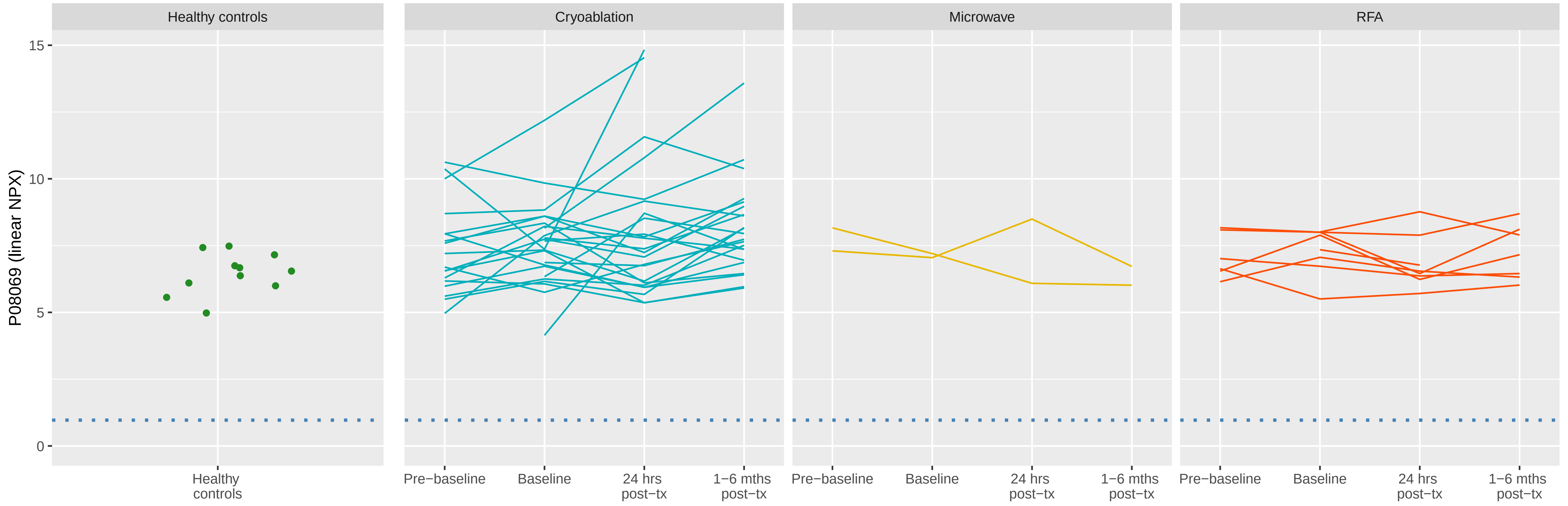

Uniprot ID: P04626, analyte:ERBB2

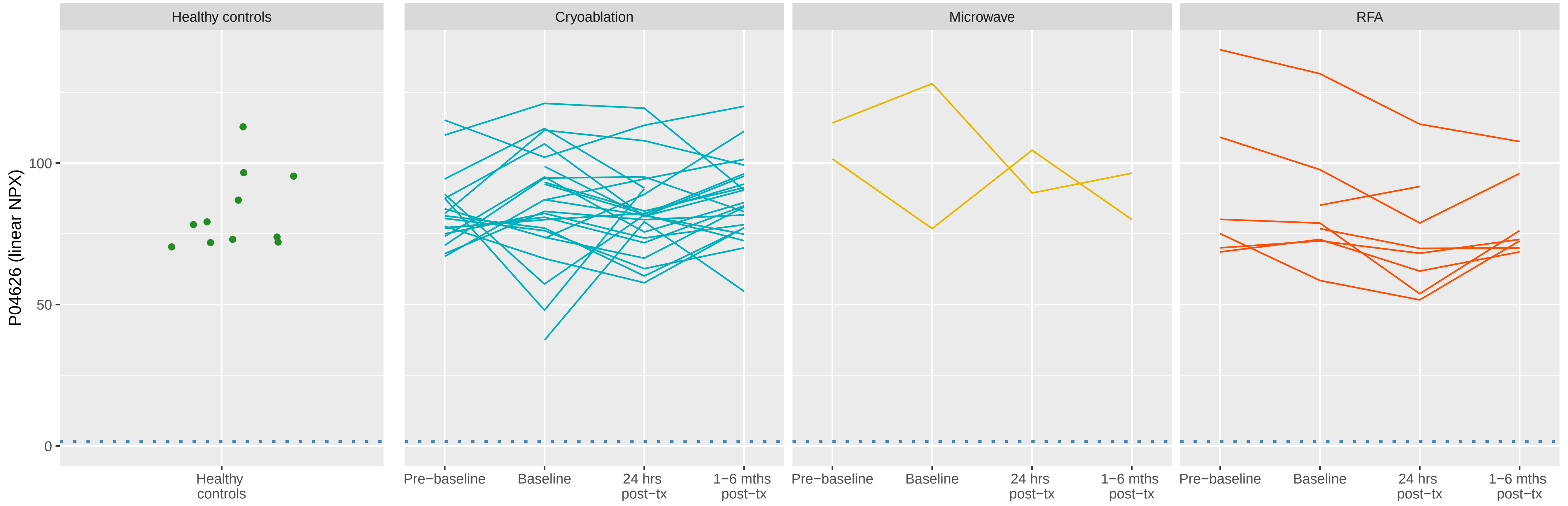

Uniprot ID: P21860, analyte:ERBB3

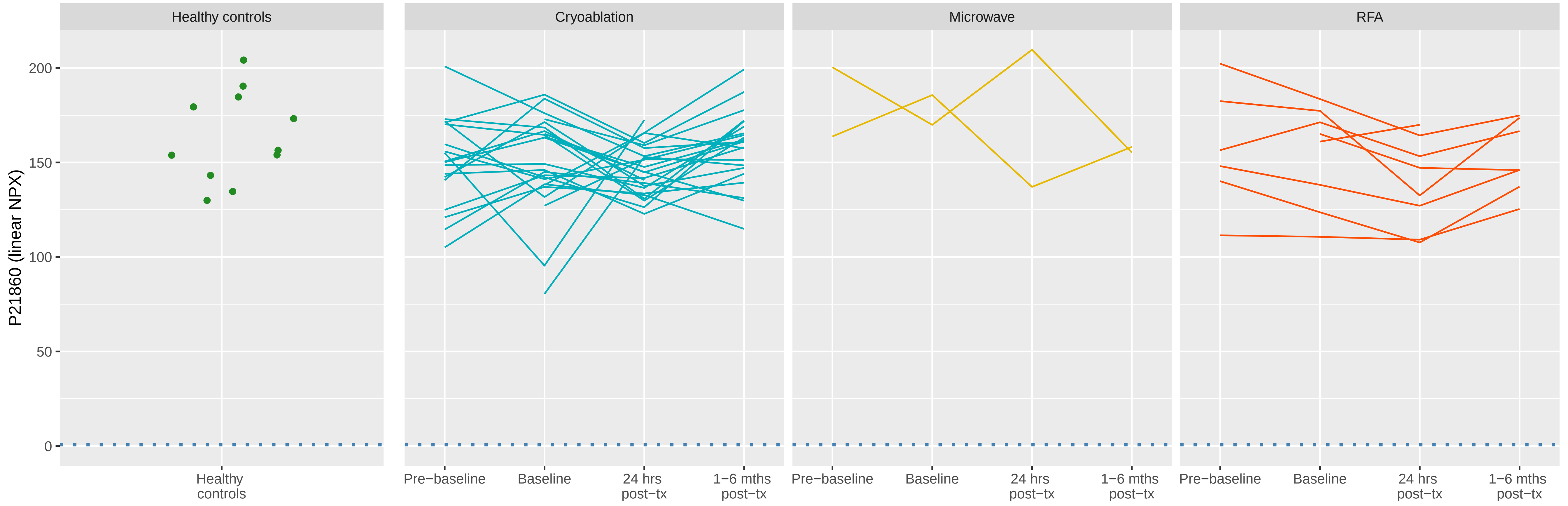

Uniprot ID: P21583, analyte:SCF

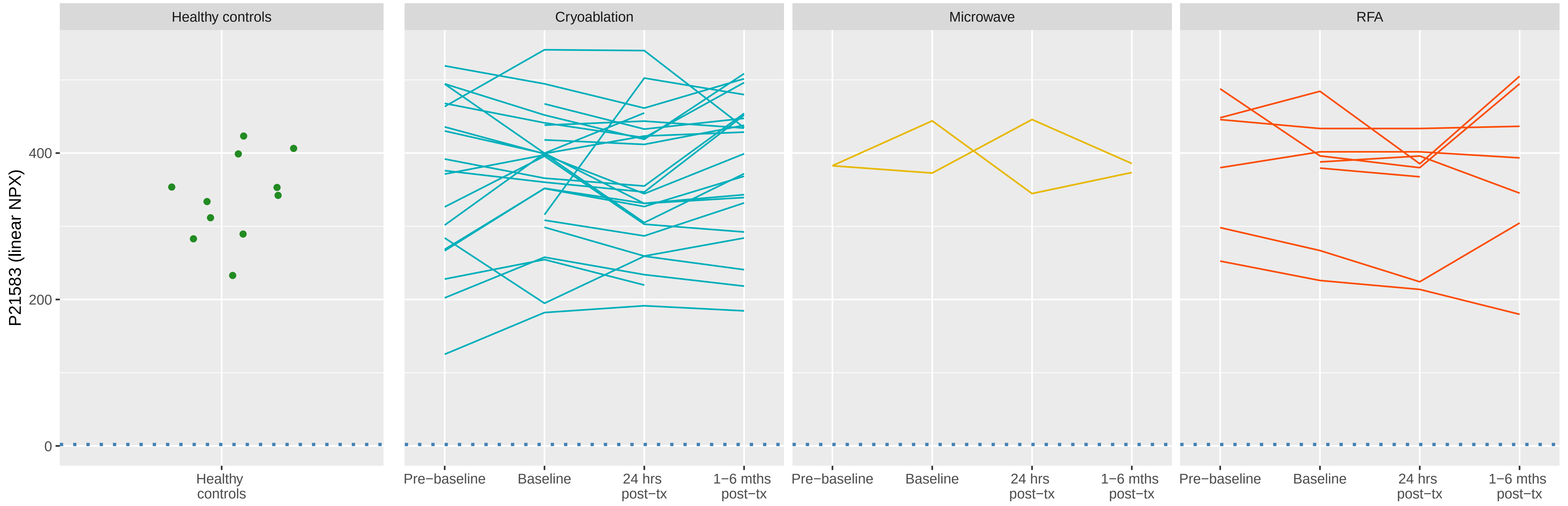

Uniprot ID: P09486, analyte:SPARC

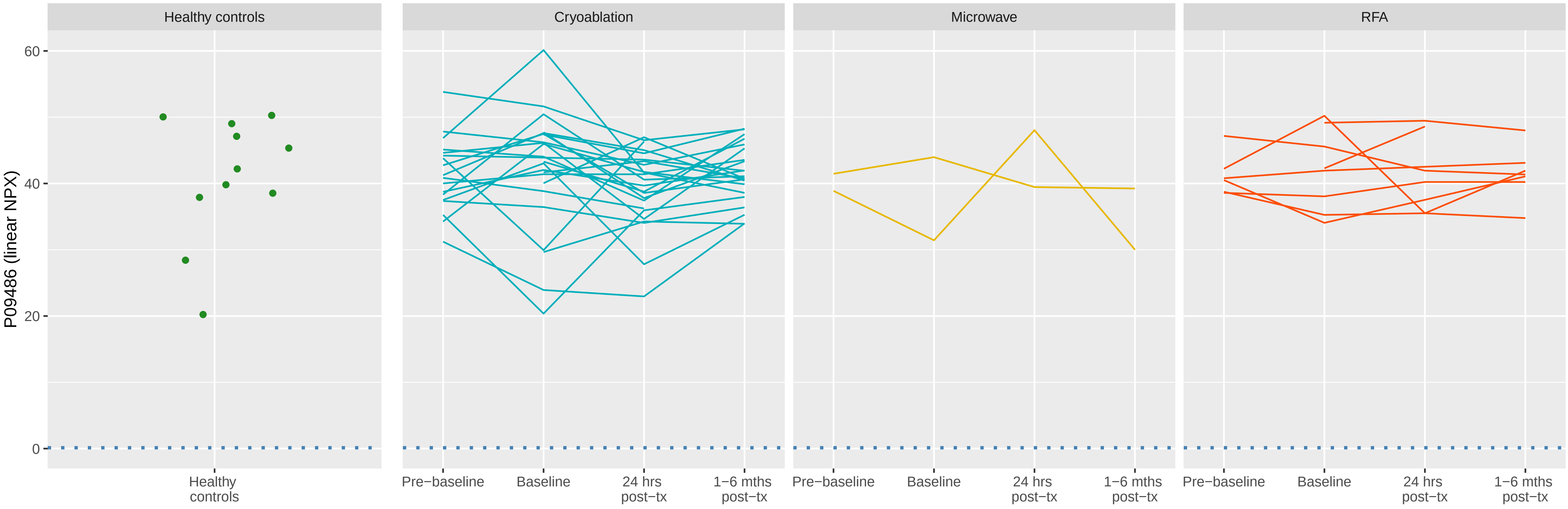

Uniprot ID: P20718, analyte:GZMH

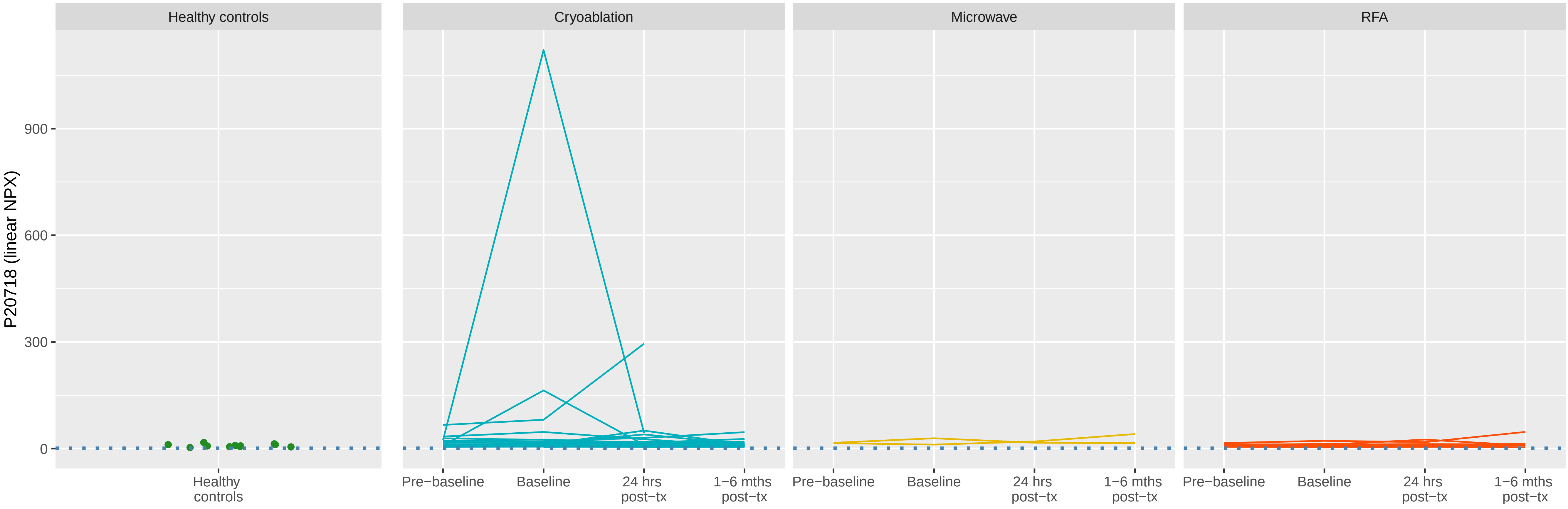

Uniprot ID: P01135, analyte:TGF−alpha

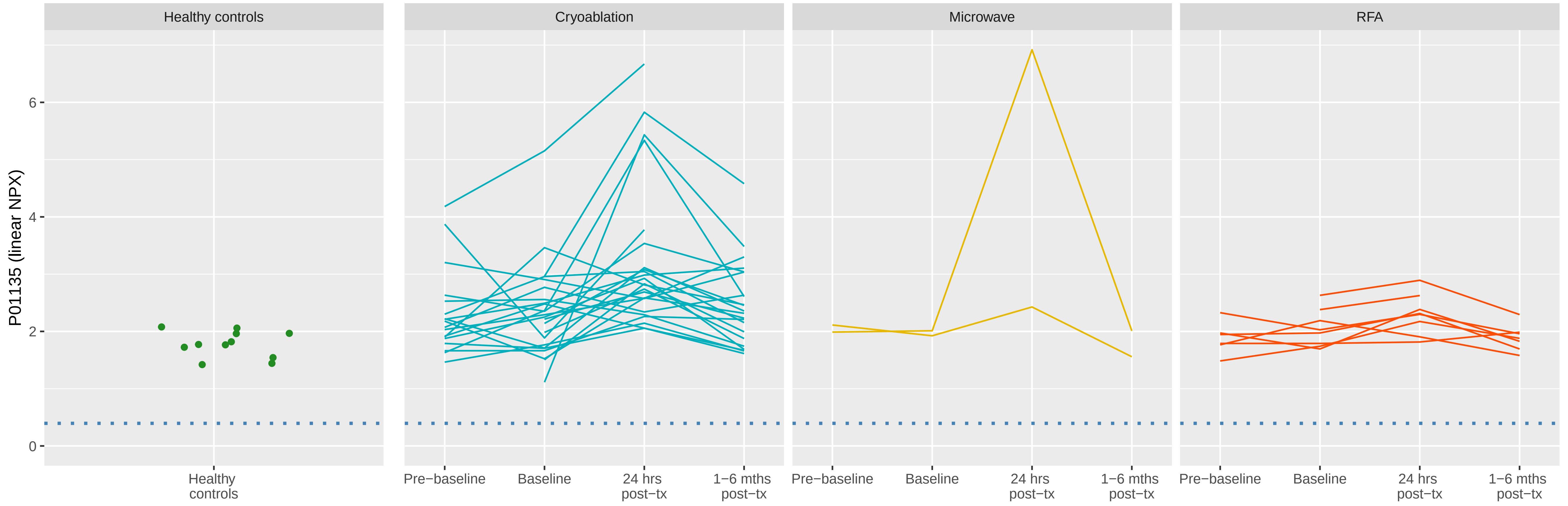

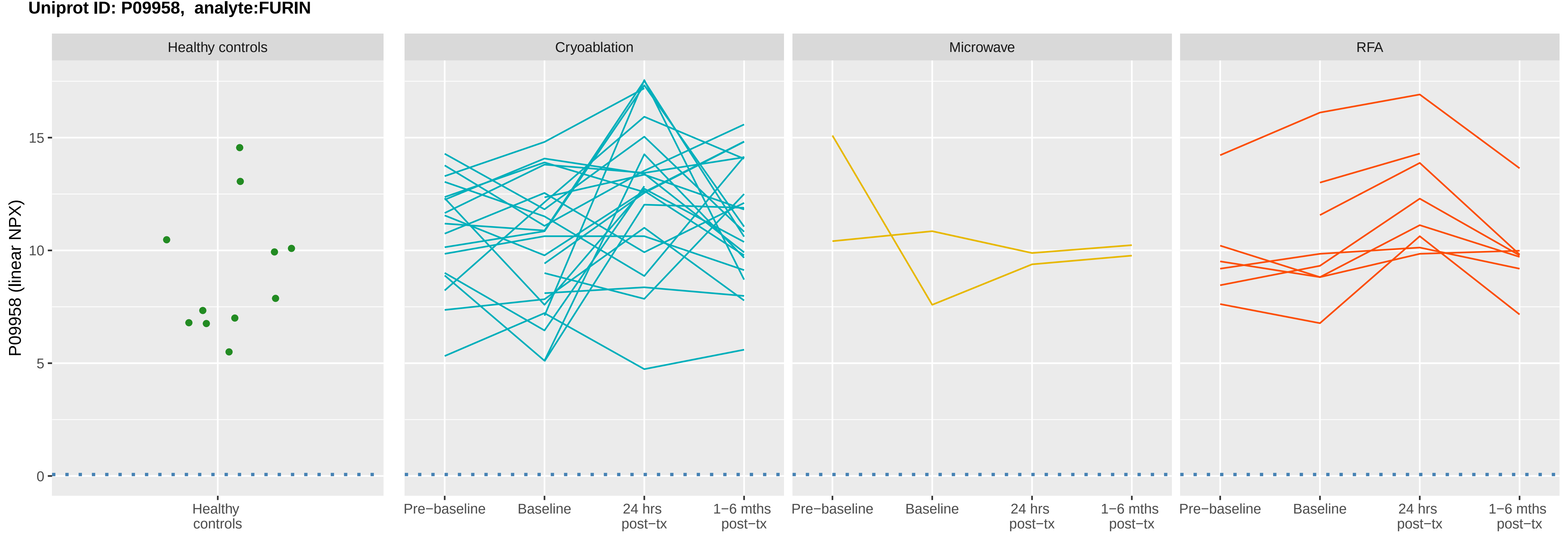
Uniprot ID: P09958, analyte:FURIN
Healthy controls
Cryoablation
Microwave
RFA
P09958 (linear NPX)
15
10
5
0
Healthy controls
Pre−baseline
Baseline
24 hrs post−tx
1−6 mths post−tx

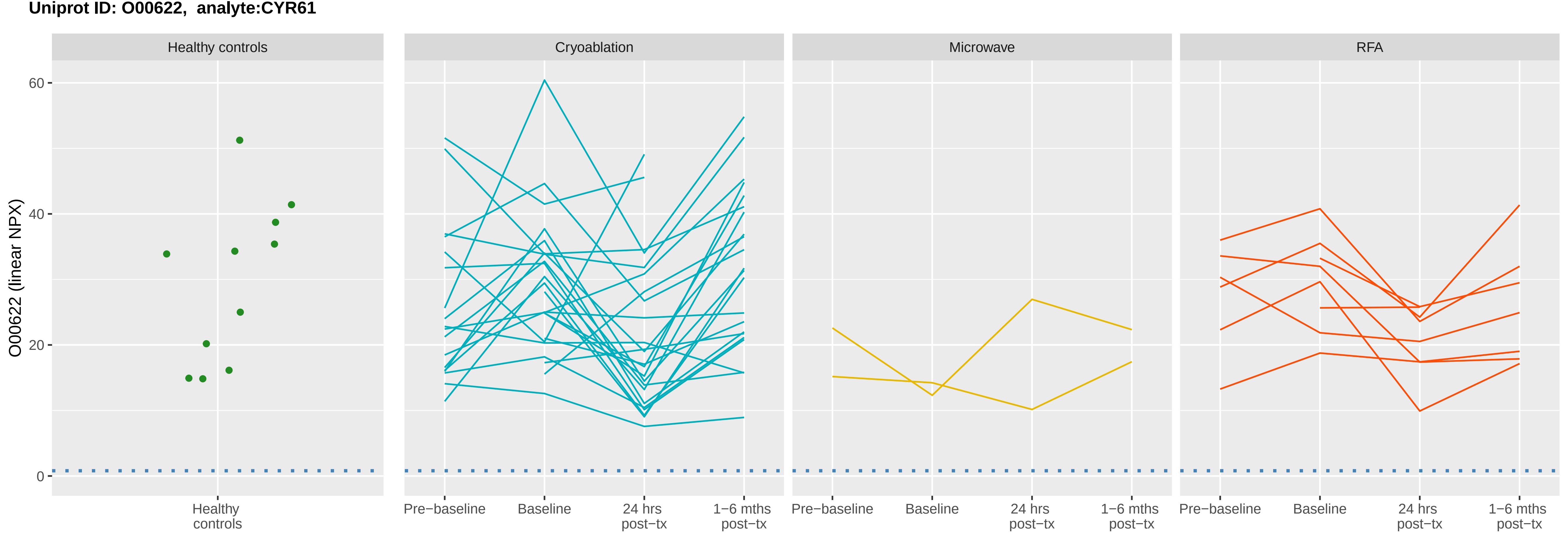
Uniprot ID: O00622, analyte:CYR61
Healthy controls
Cryoablation
Microwave
RFA
O00622 (linear NPX)
0
20
40
60
Healthy controls
Pre−baseline
Baseline
24 hrs post−tx
1−6 mths post−tx

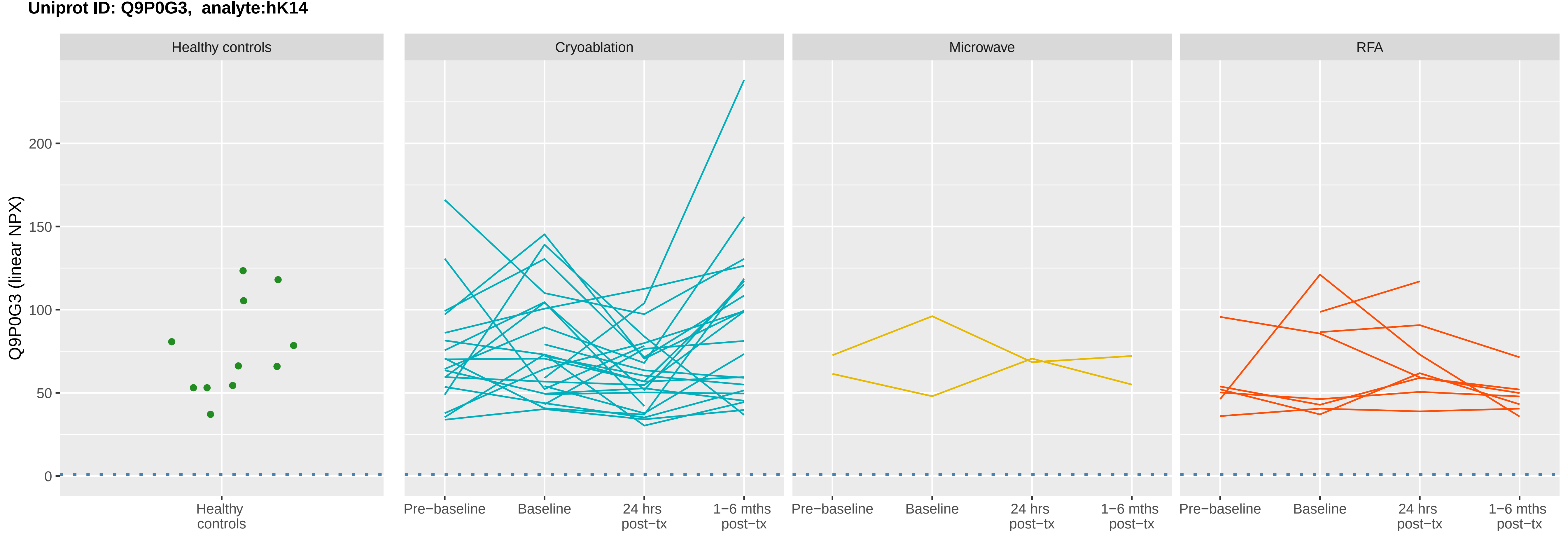

Uniprot ID: Q9P0G3, analyte:hK14
Healthy controls
Cryoablation
Microwave
RFA
Q9P0G3 (linear NPX)
200
150
100
50
0
Healthy controls
Pre−baseline
Baseline
24 hrs post−tx
1−6 mths post−tx

Uniprot ID: Q13158, analyte:FADD

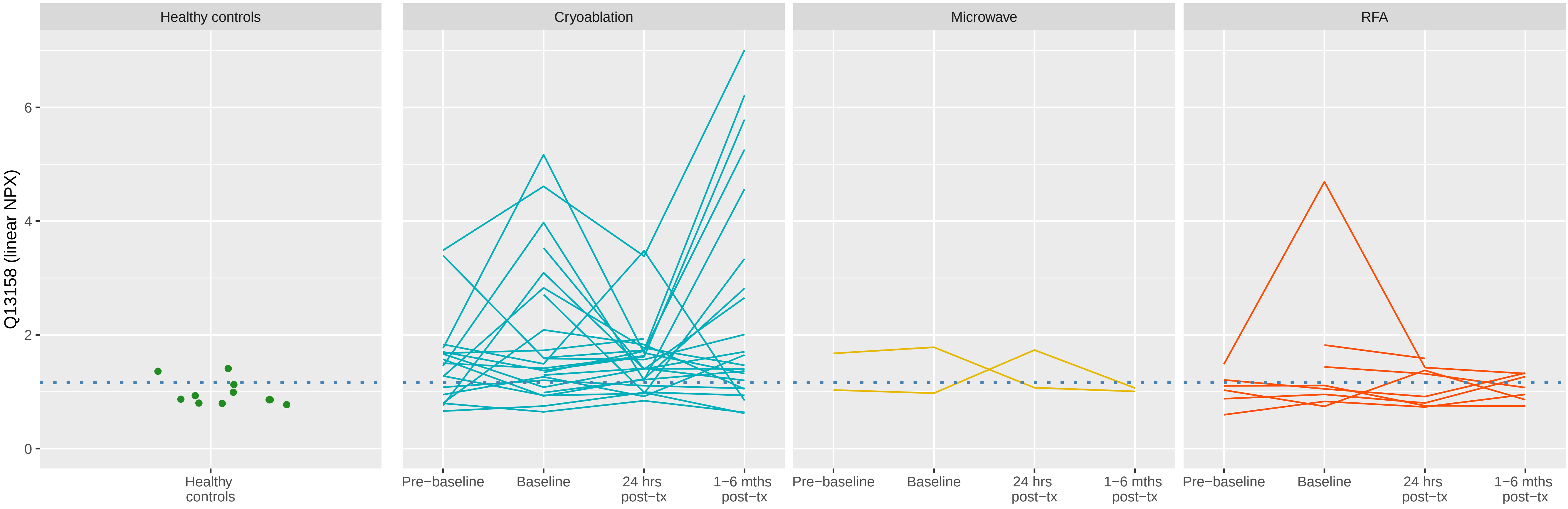

Uniprot ID: P50579, analyte:MetAP 2

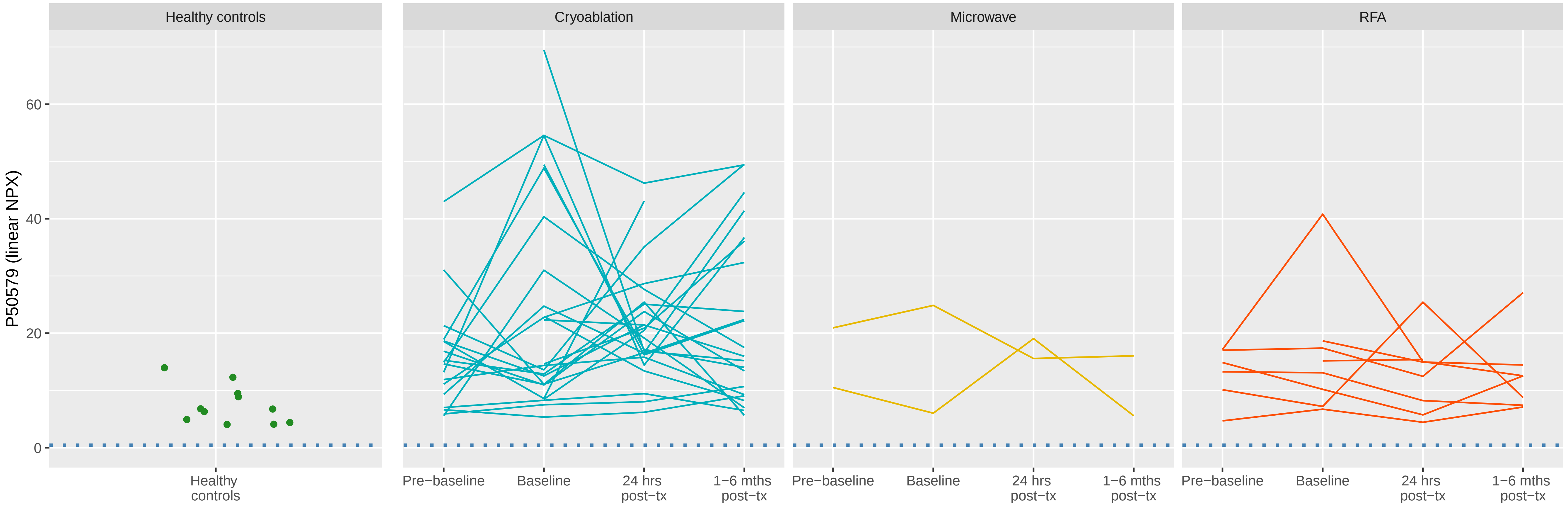

Uniprot ID: Q96NY8, analyte:PVRL4

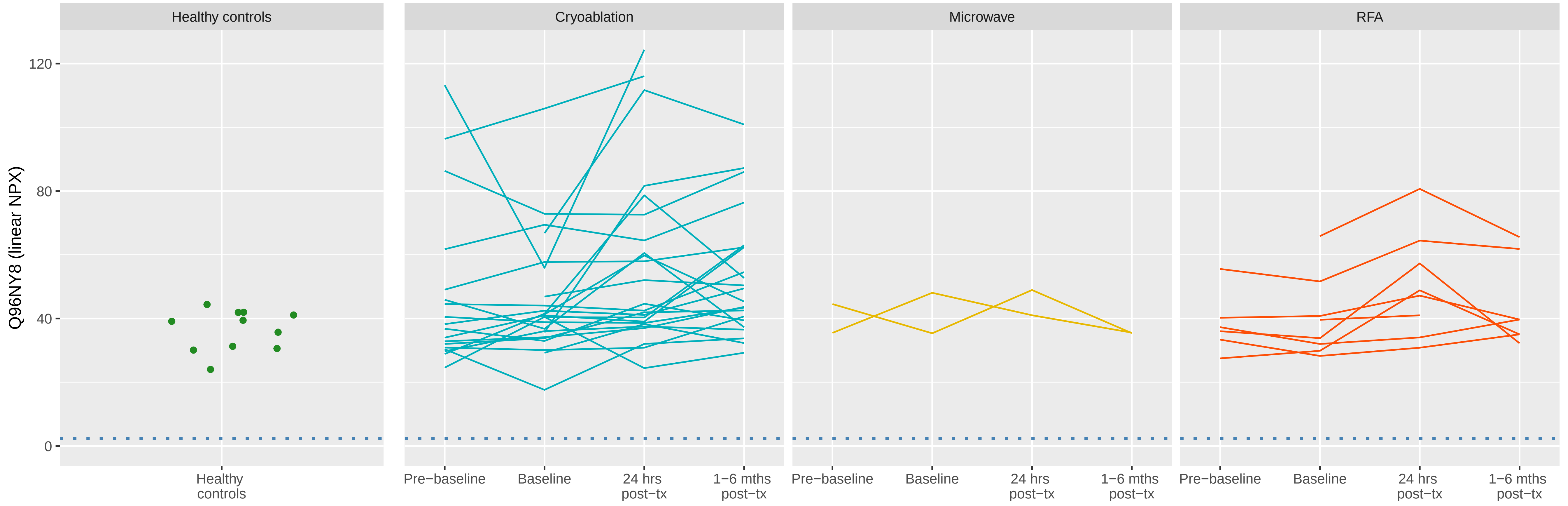

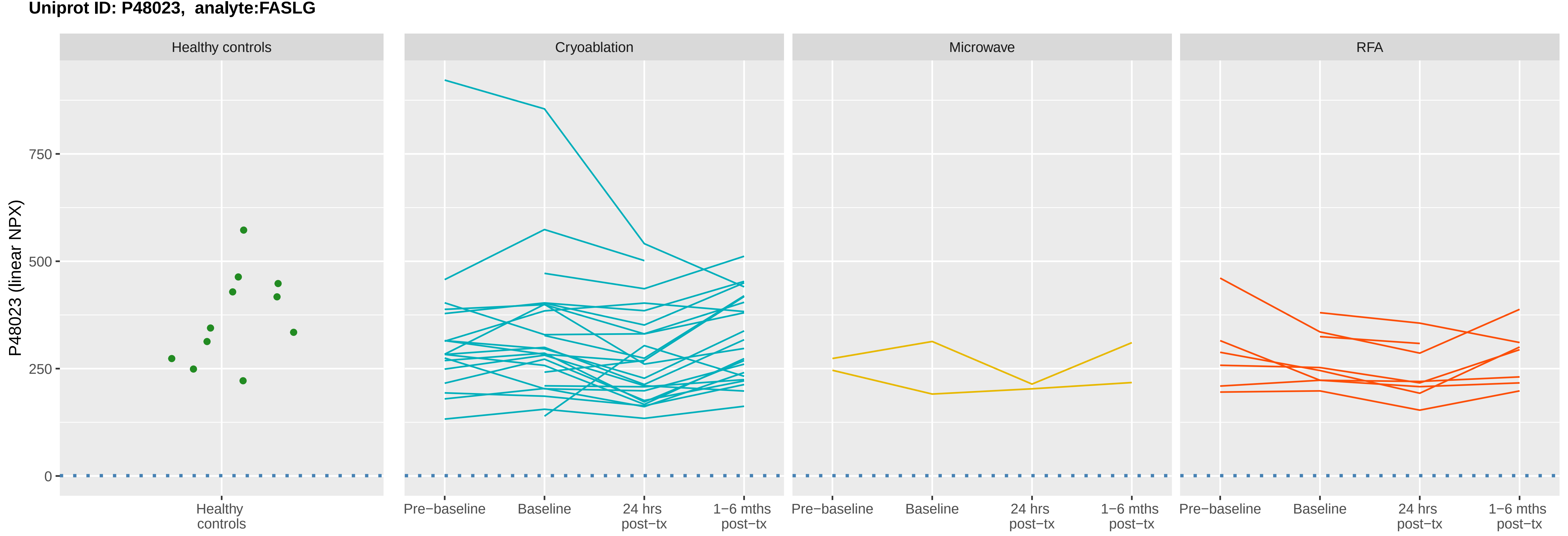
Uniprot ID: P48023, analyte:FASLG
Healthy controls
Cryoablation
Microwave
RFA
P48023 (linear NPX)
750
500
250
0
Healthy controls
Pre−baseline
Baseline
24 hrs post−tx
1−6 mths post−tx

Uniprot ID: P29317, analyte:EPHA2

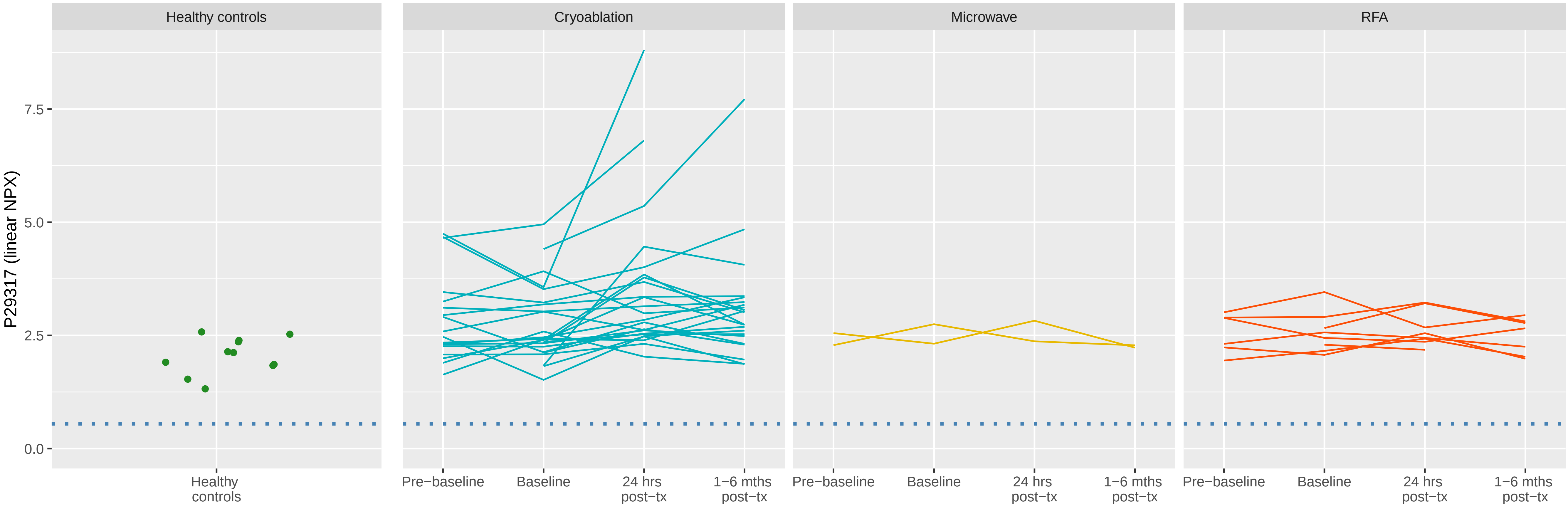

Uniprot ID: P18084, analyte:ITGB5

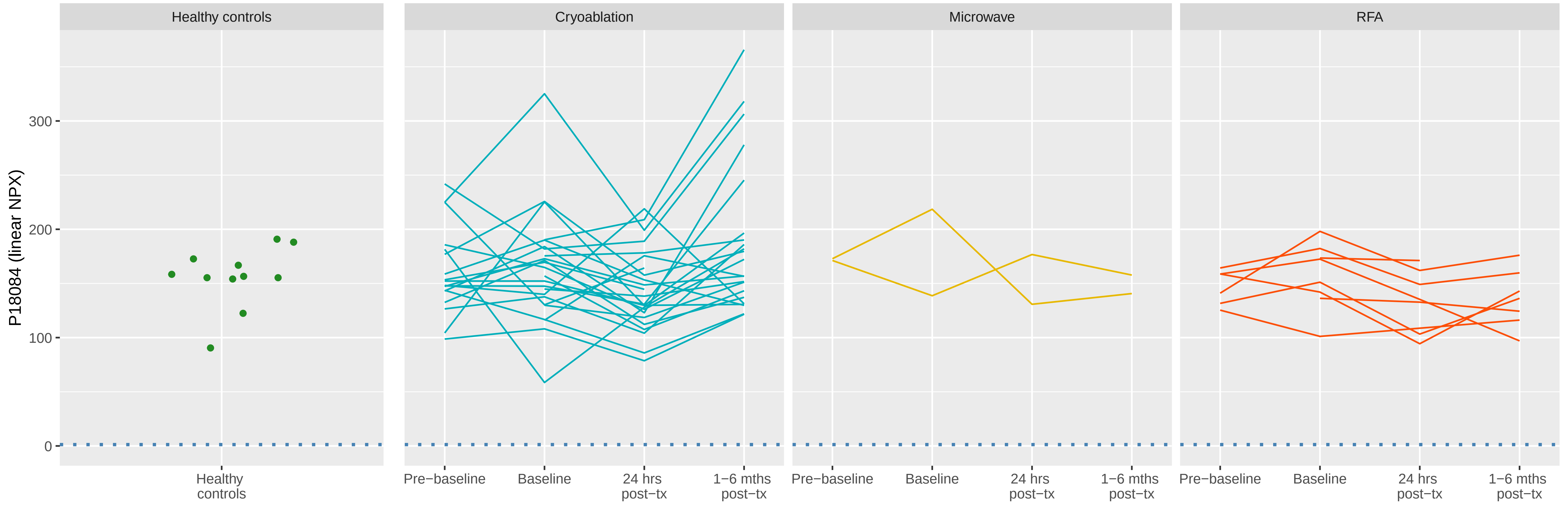

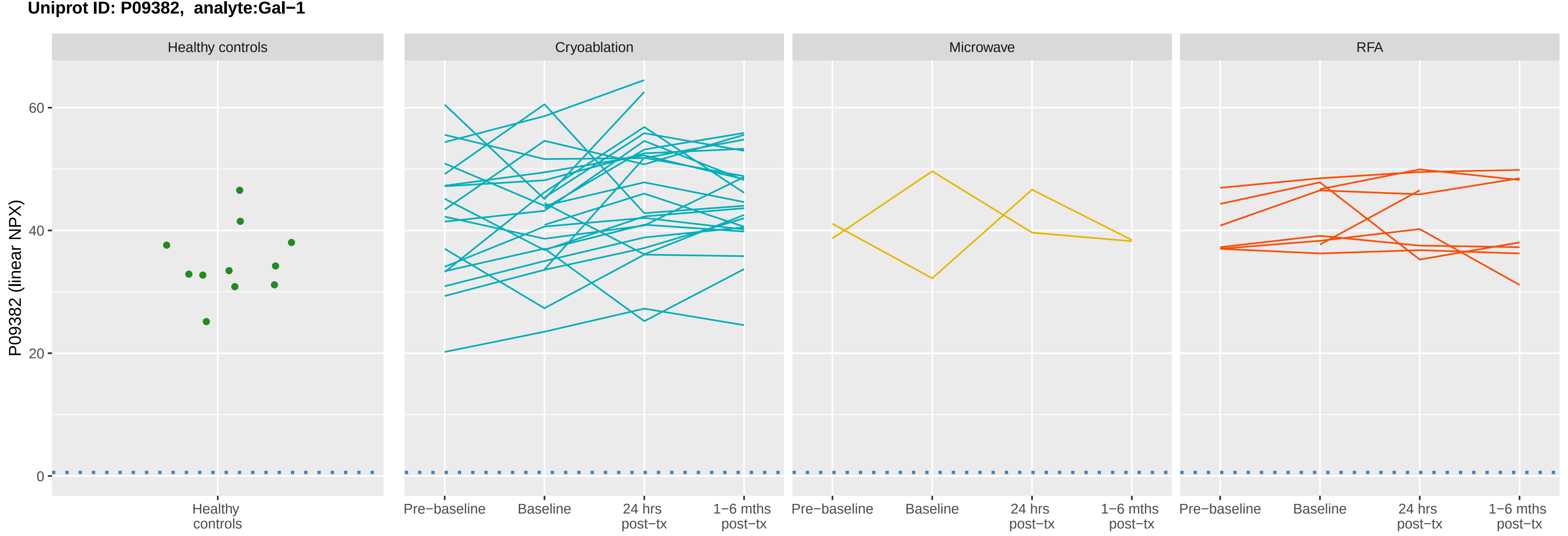
Uniprot ID: P09382, analyte:Gal−1
Healthy controls
Cryoablation
Microwave
RFA
P09382 (linear NPX)
60
40
20
0
Healthy controls
Pre−baseline
Baseline
24 hrs post−tx
1−6 mths post−tx

Uniprot ID: Q9BYH1, analyte:SEZ6L

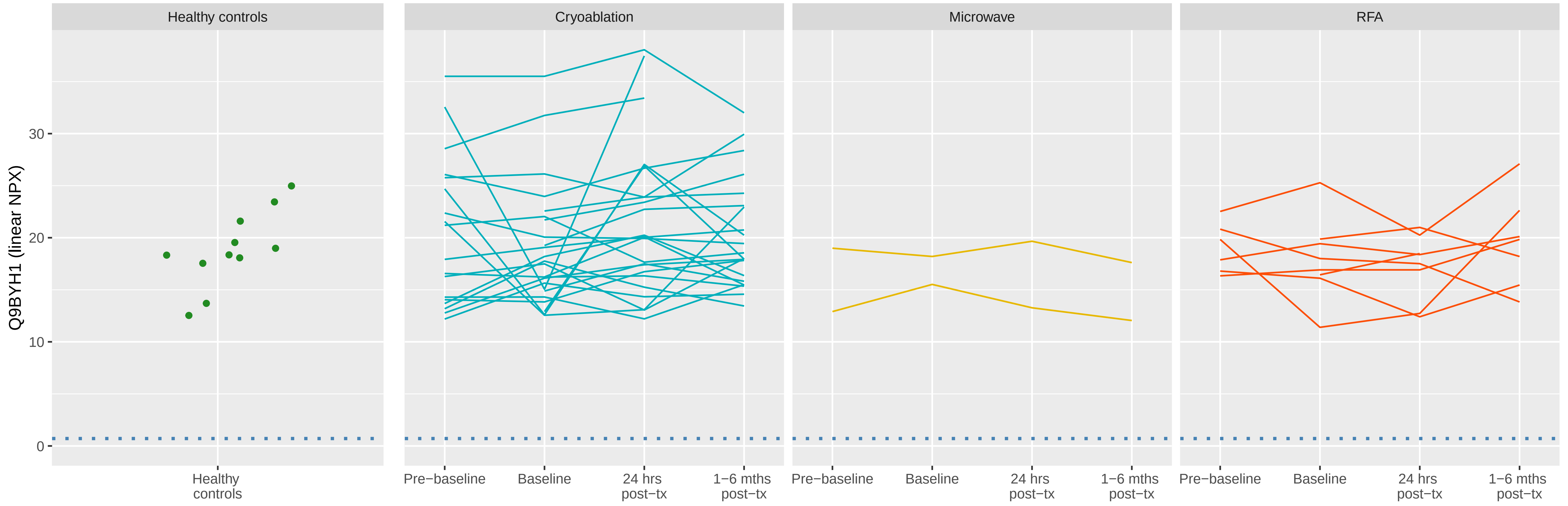

Uniprot ID: Q14956, analyte:GPNMB

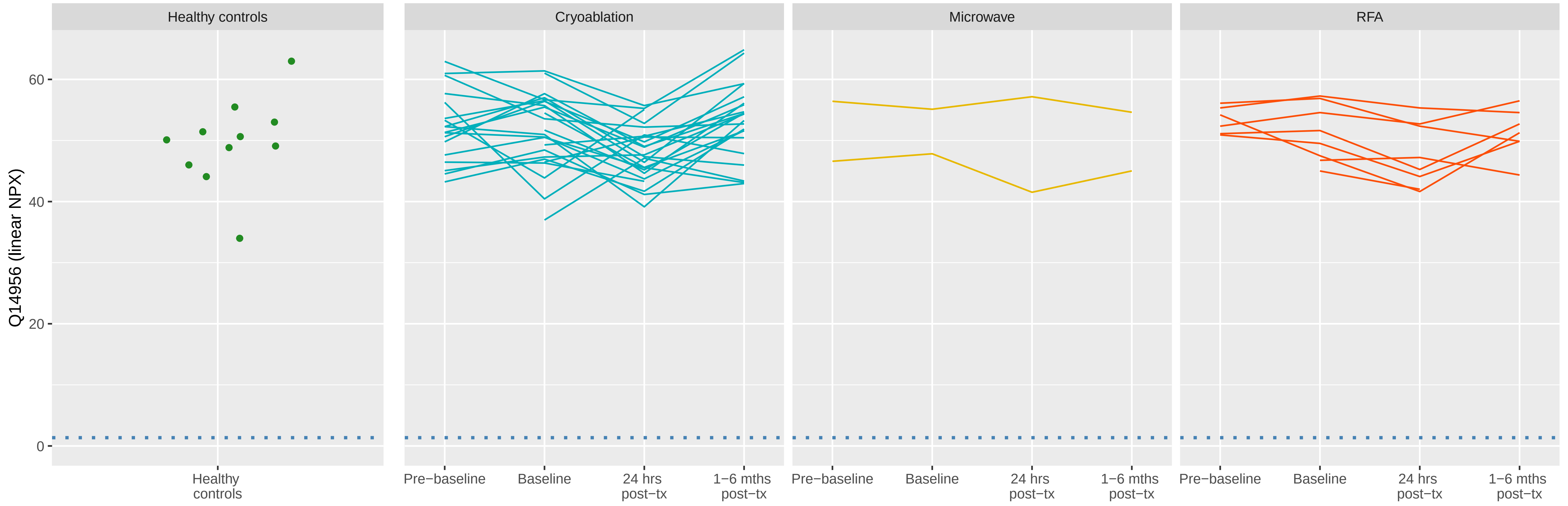

Uniprot ID: Q16790, analyte:CAIX

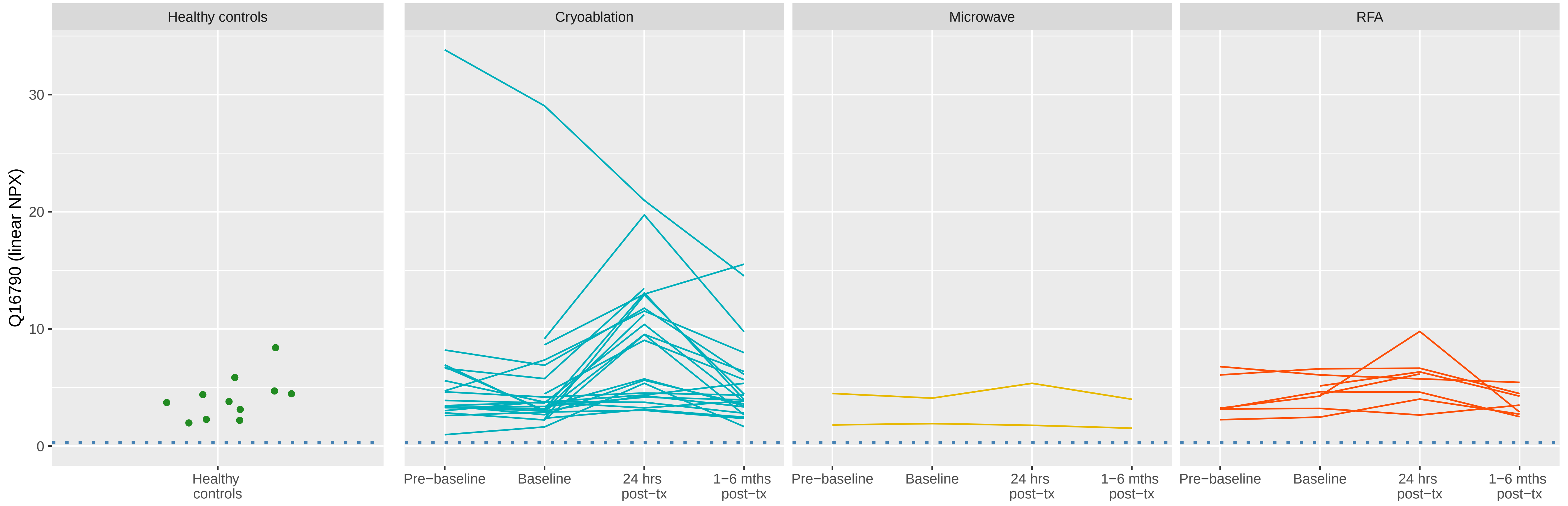

Uniprot ID: Q16674, analyte:MIA

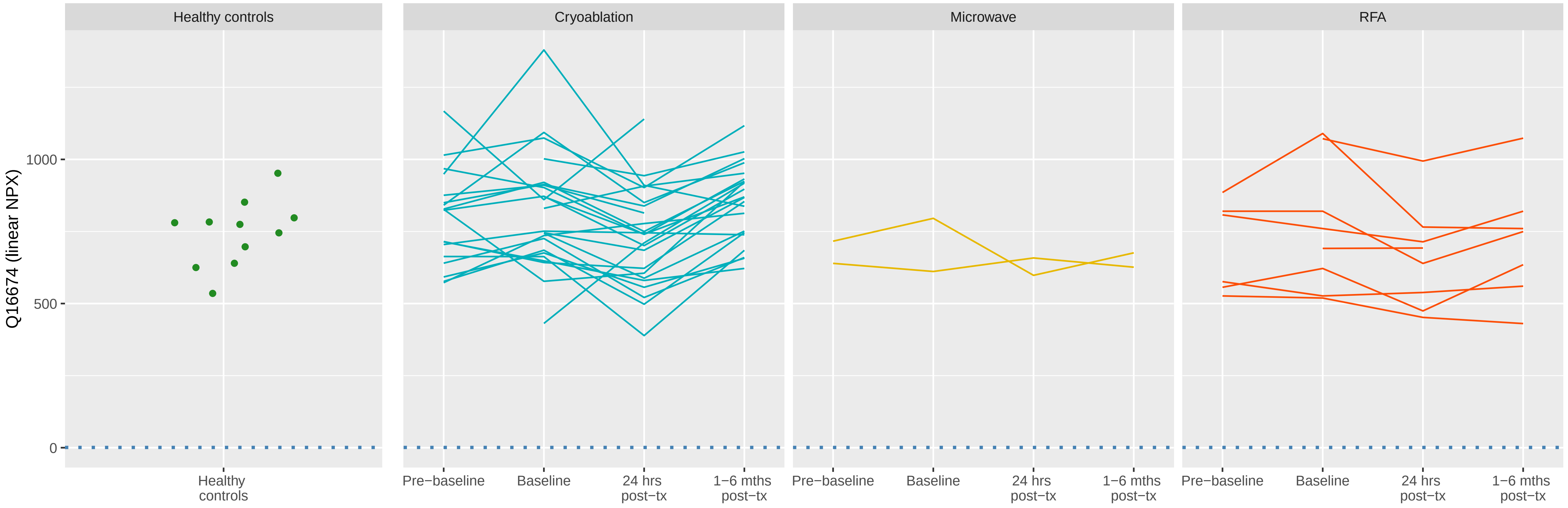

Uniprot ID: O60911, analyte:CTSV

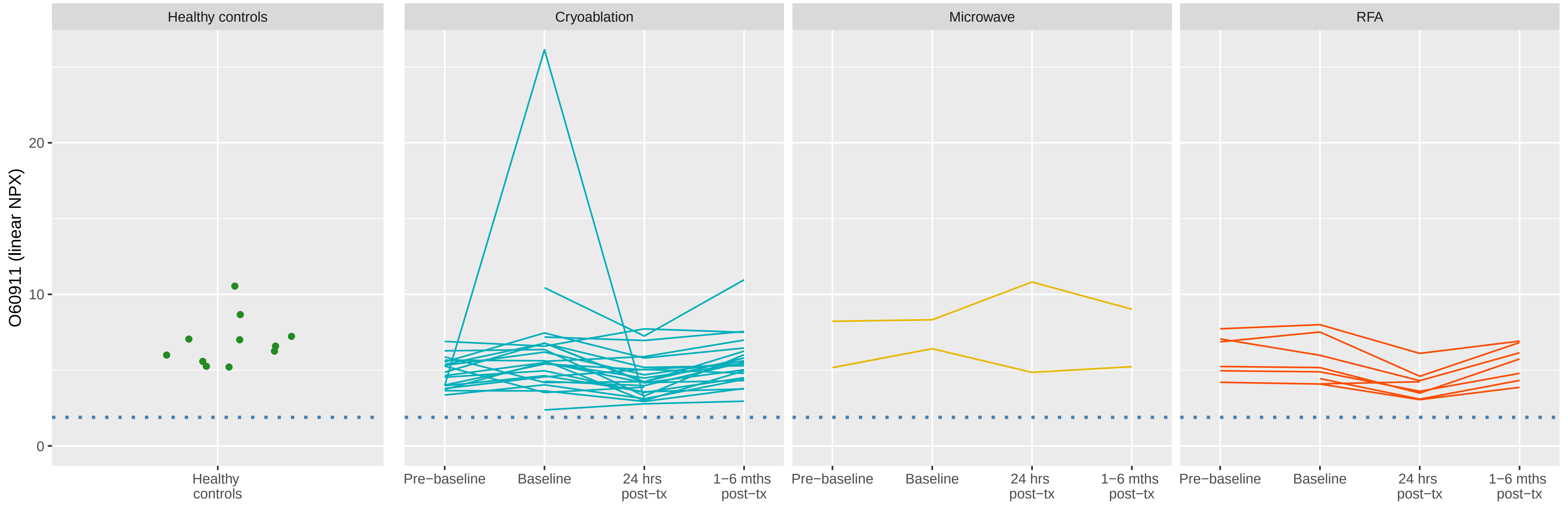

Uniprot ID: P26842, analyte:CD27

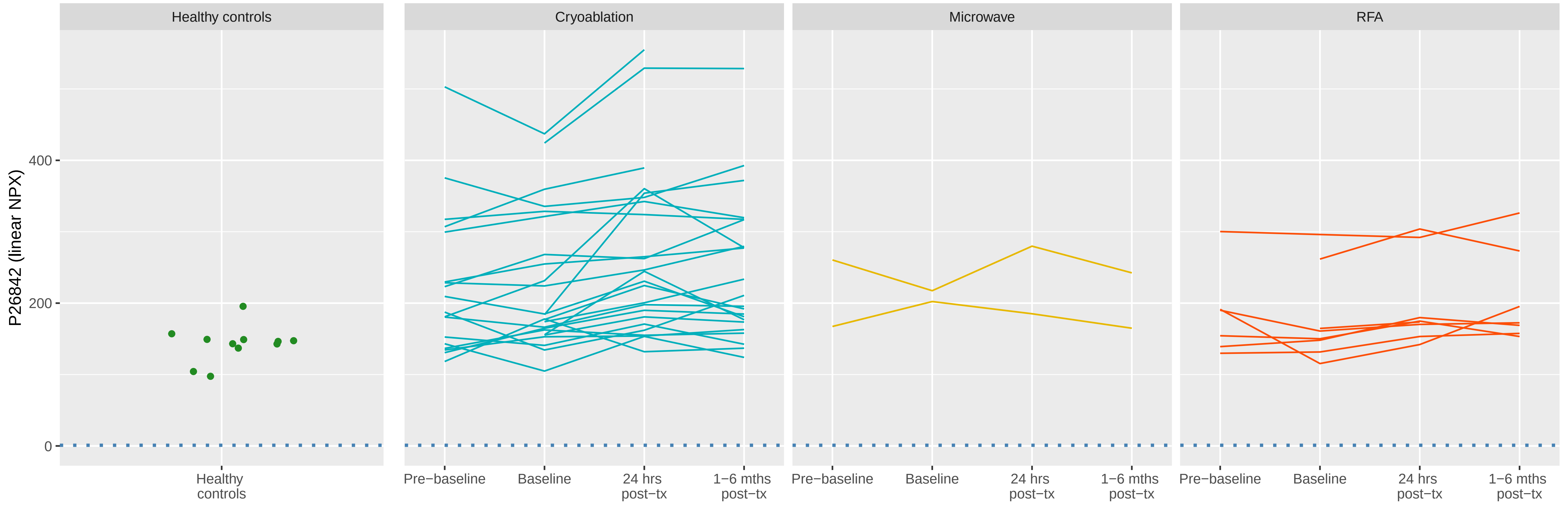

Uniprot ID: O43895, analyte:XPNPEP2

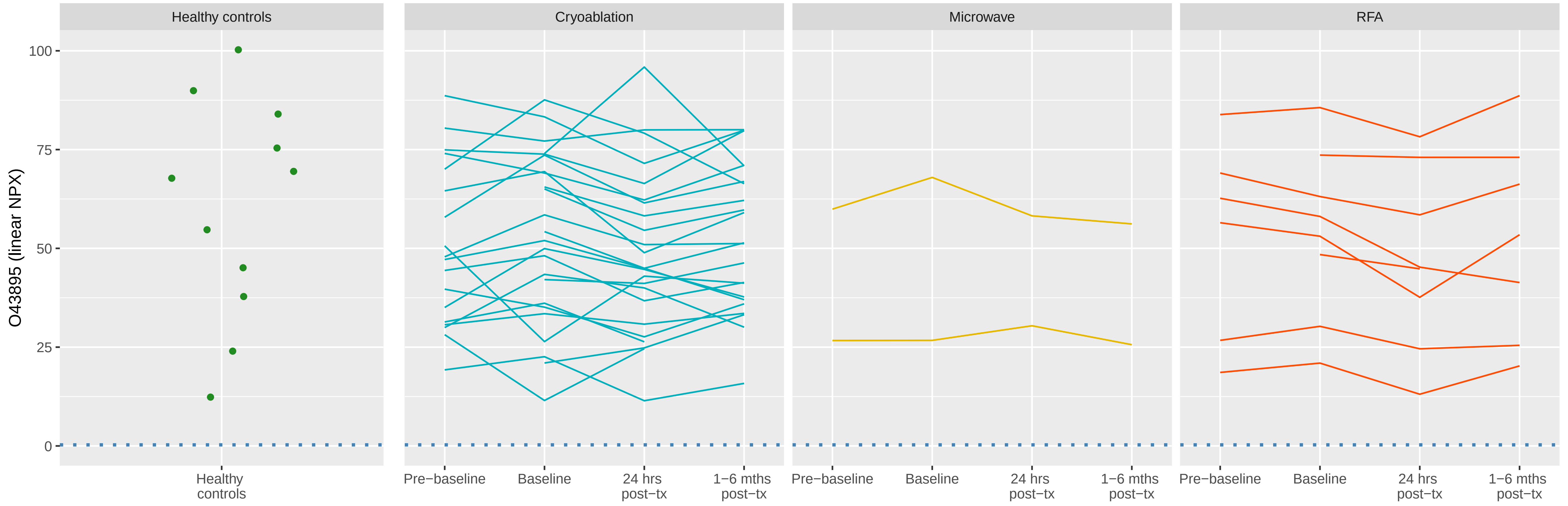

Uniprot ID: Q15303, analyte:ERBB4

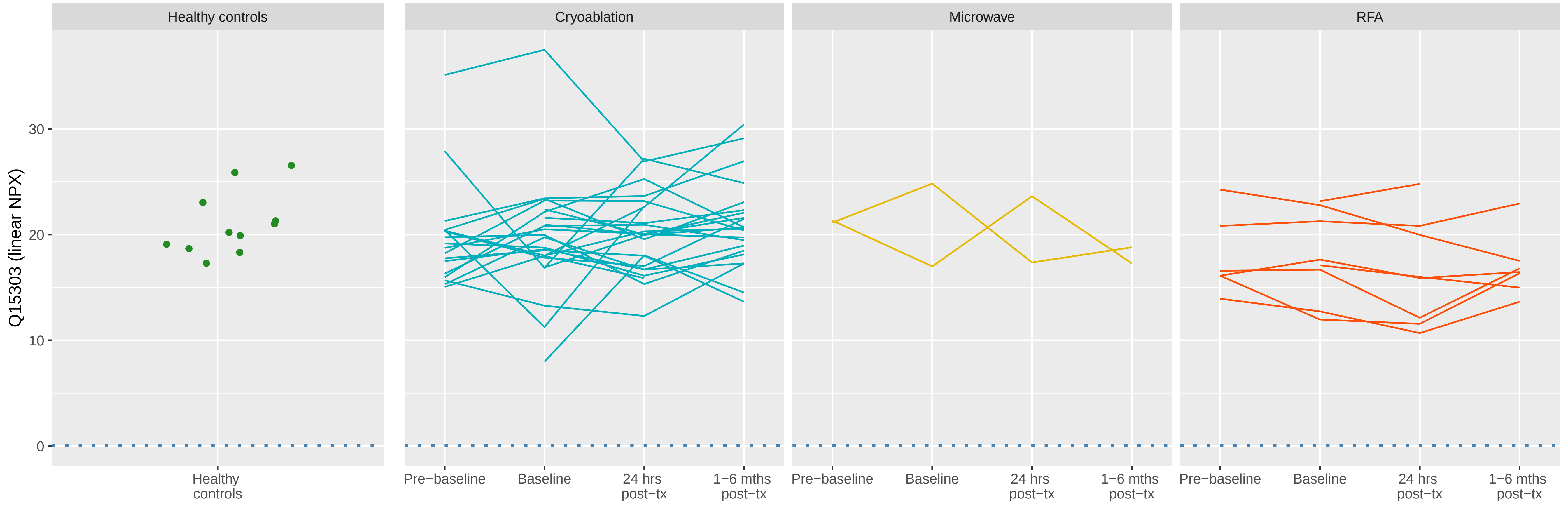

Uniprot ID: P14210, analyte:HGF

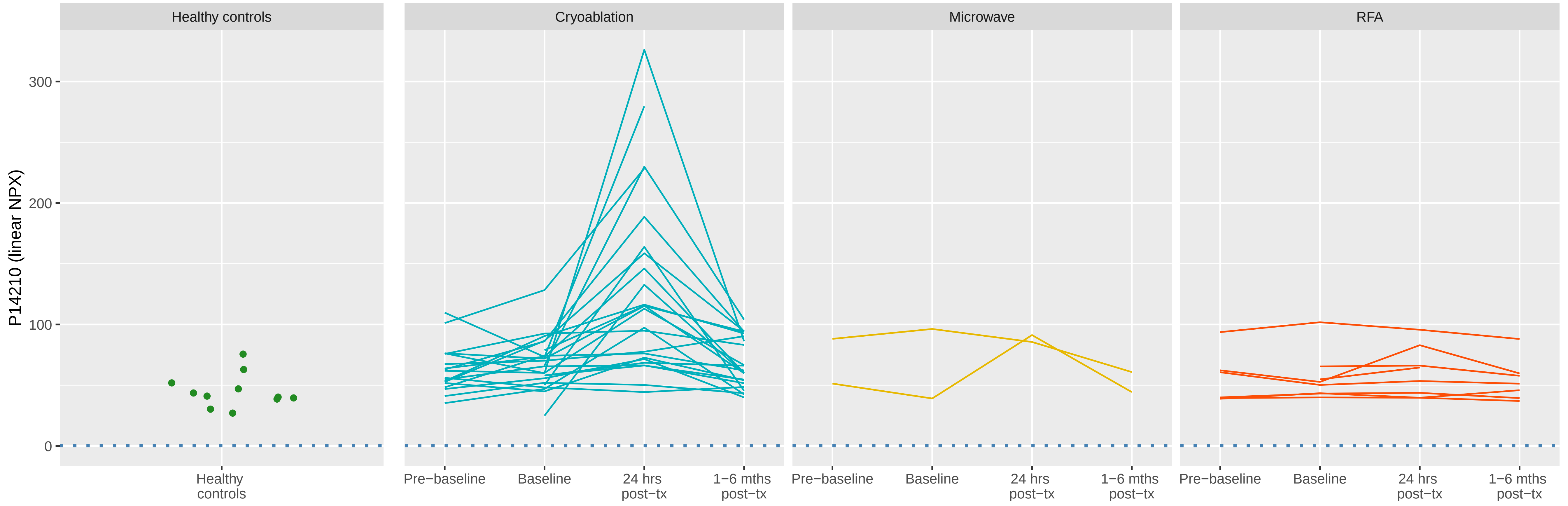

Uniprot ID: P78325, analyte:ADAM 8

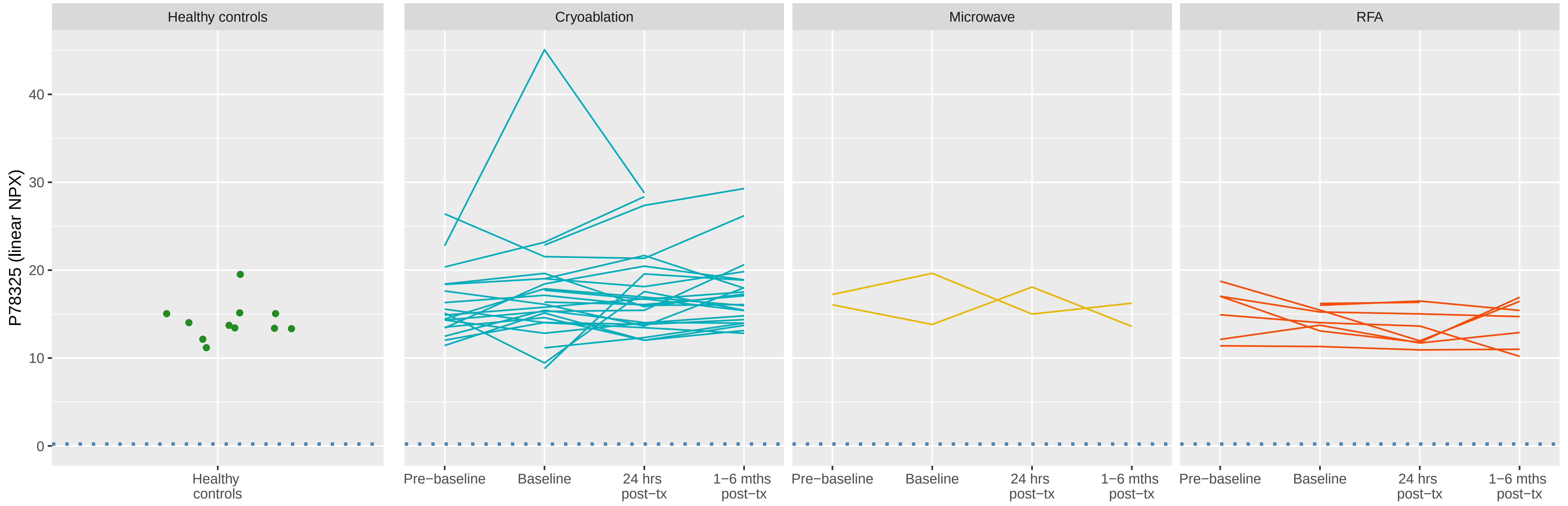

Uniprot ID: P21589, analyte:5'−NT

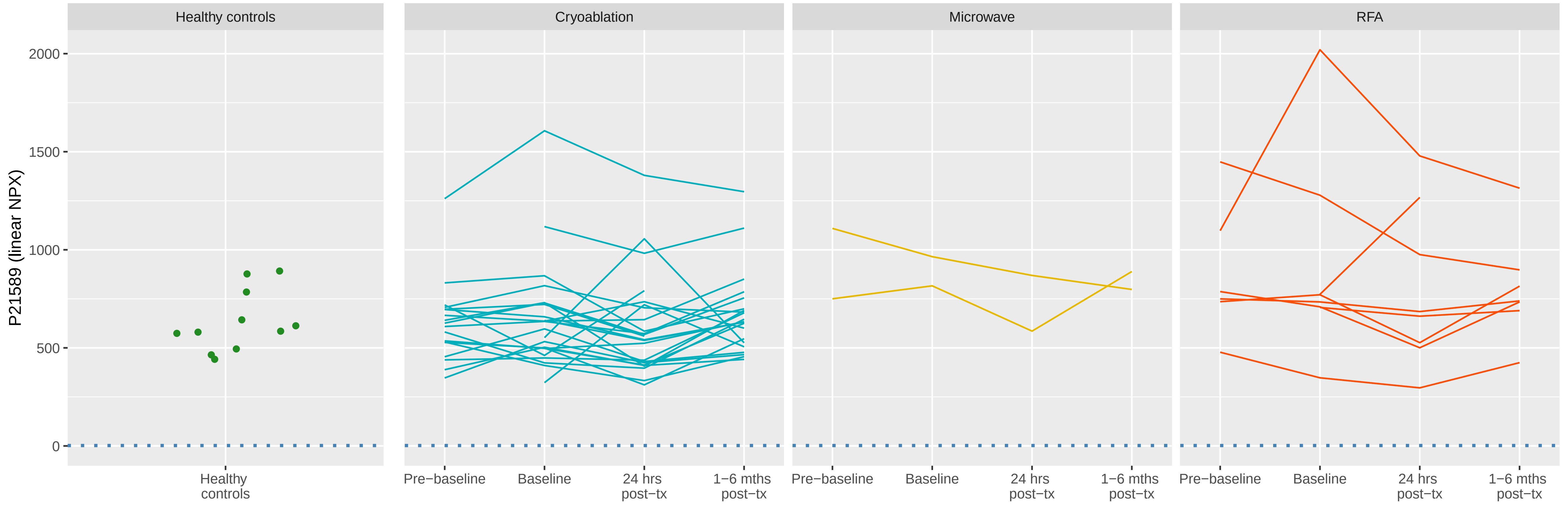

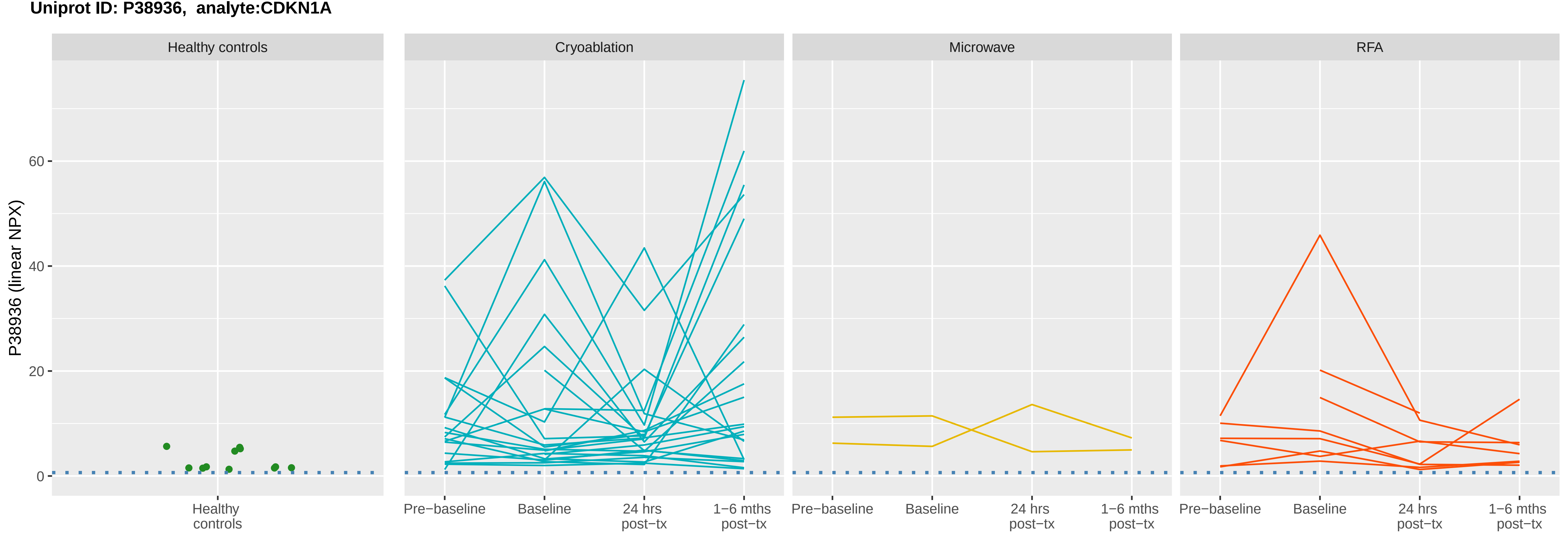
Uniprot ID: P38936, analyte:CDKN1A
P38936 (linear NPX)
0
20
40
60
Healthy controls
Cryoablation
Microwave
RFA
Healthy controls
Pre−baseline
Baseline
24 hrs post−tx
1−6 mths post−tx

Uniprot ID: O00548, analyte:DLL1

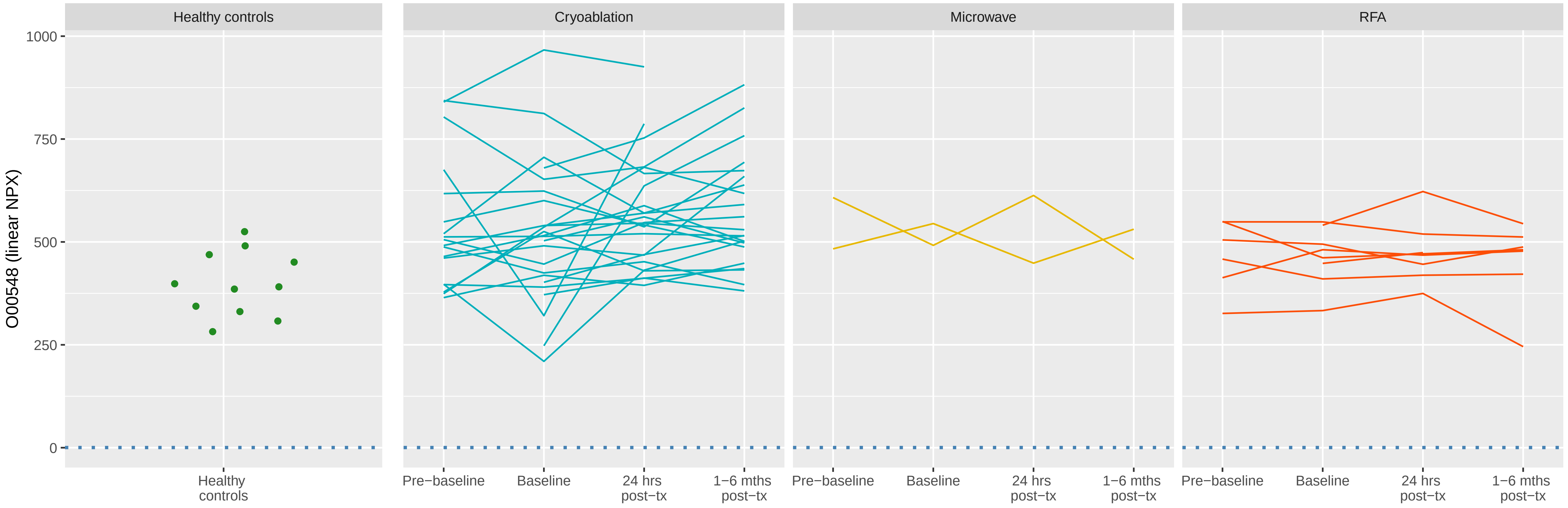

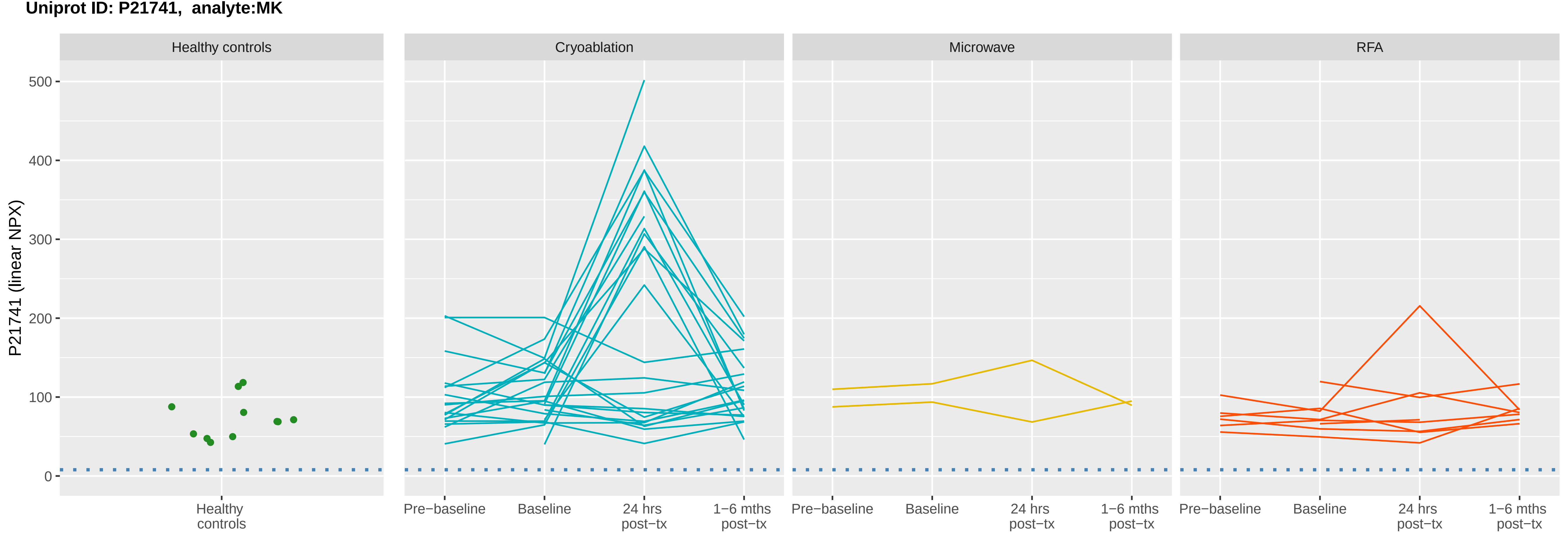
Uniprot ID: P21741, analyte:MK
Healthy controls
Cryoablation
Microwave
RFA
P21741 (linear NPX)
500
400
300
200
100
0
Healthy controls
Pre−baseline
Baseline
24 hrs post−tx
1−6 mths post−tx

Uniprot ID: P00519, analyte:ABL1

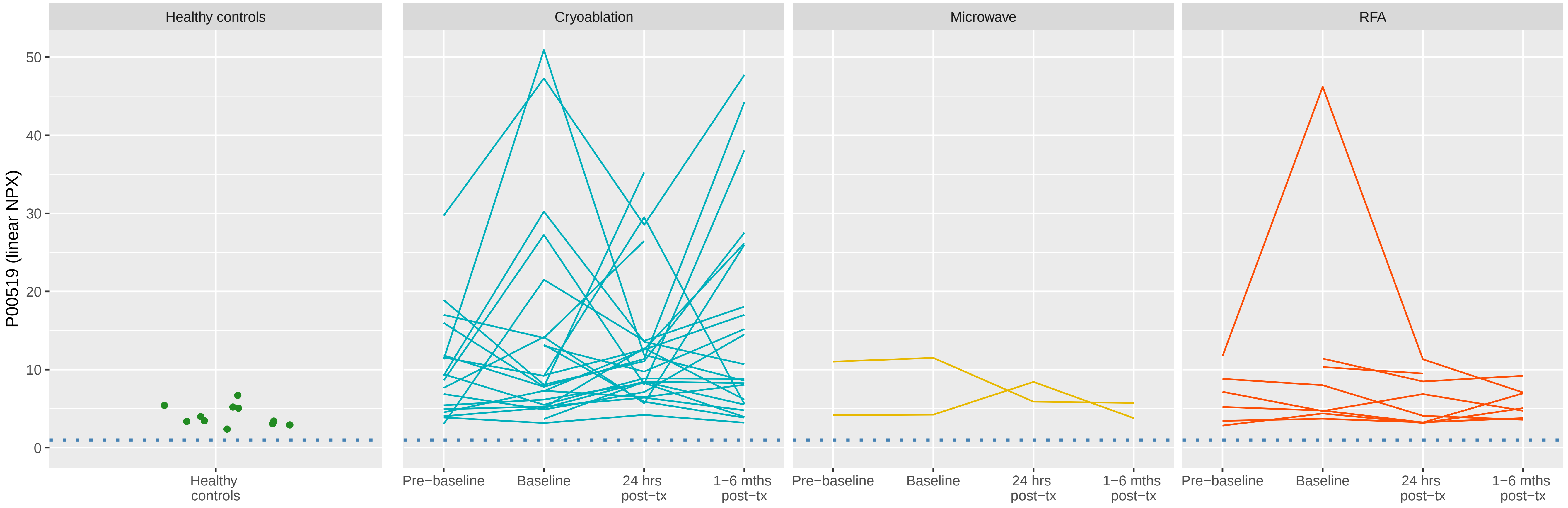

Uniprot ID: Q14512, analyte:FGF−BP1

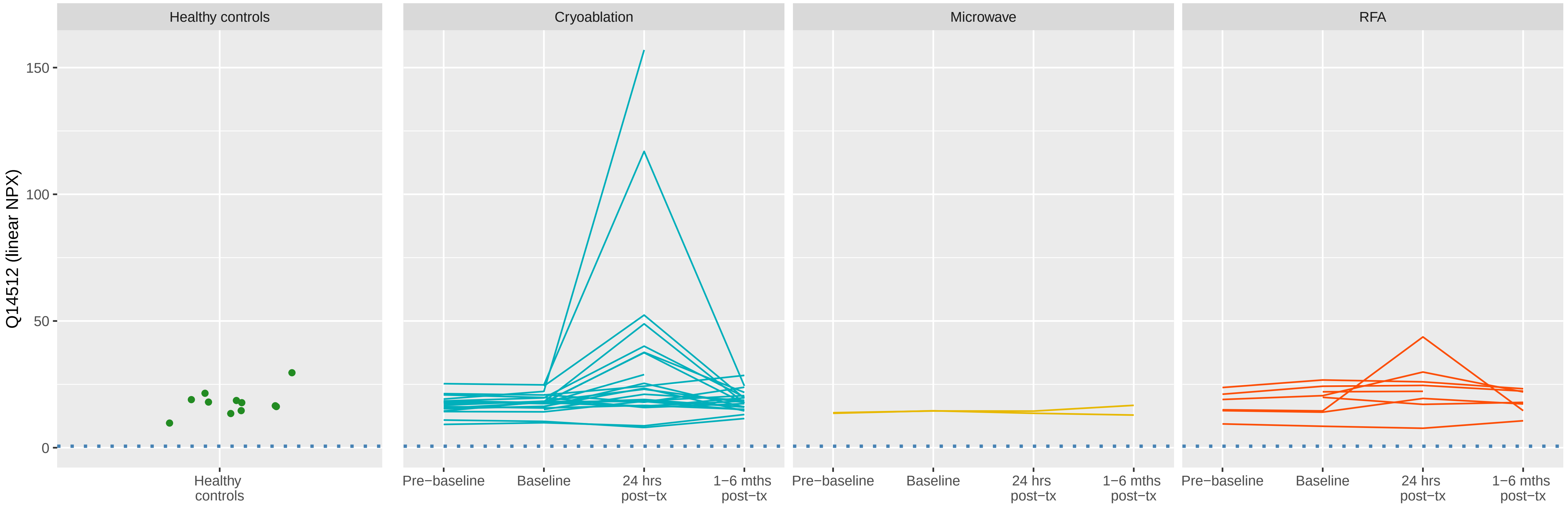

Uniprot ID: O15455, analyte:TLR3

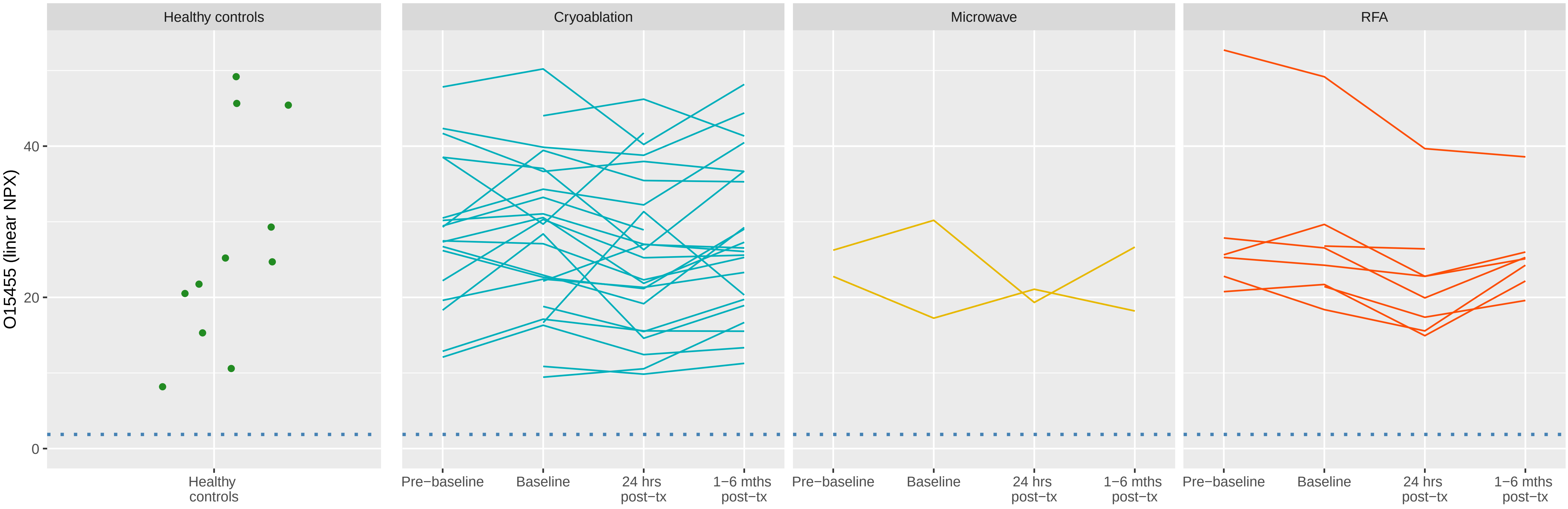

Uniprot ID: P07948, analyte:LYN

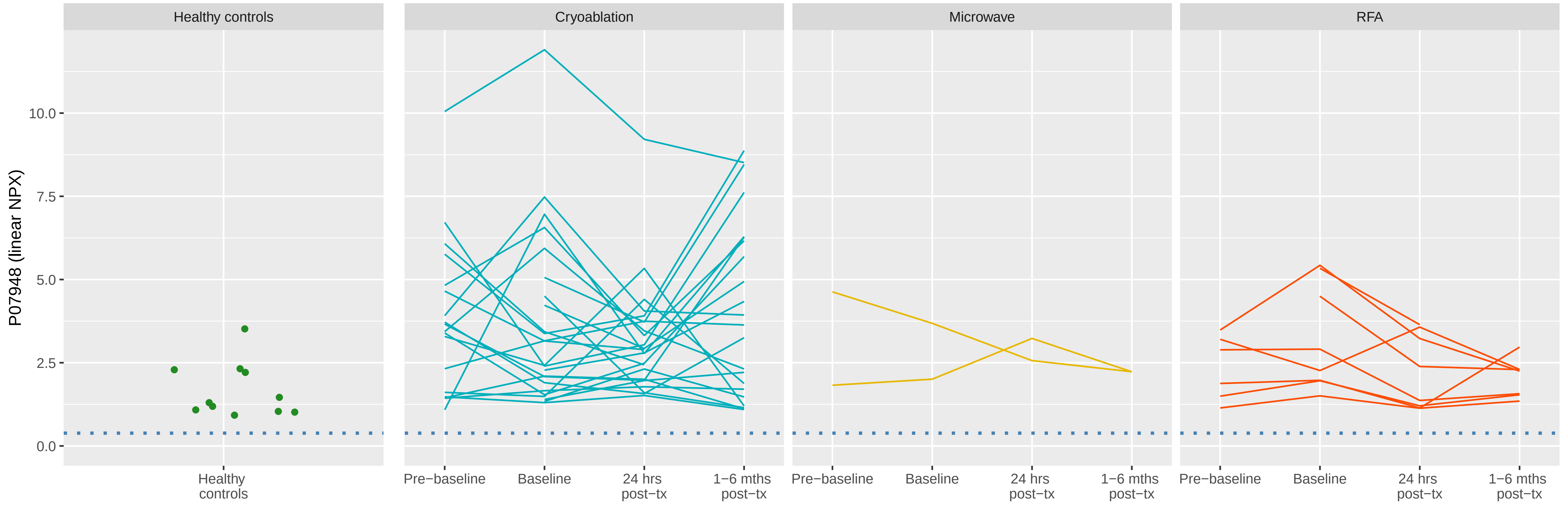

Uniprot ID: P07949, analyte:RET

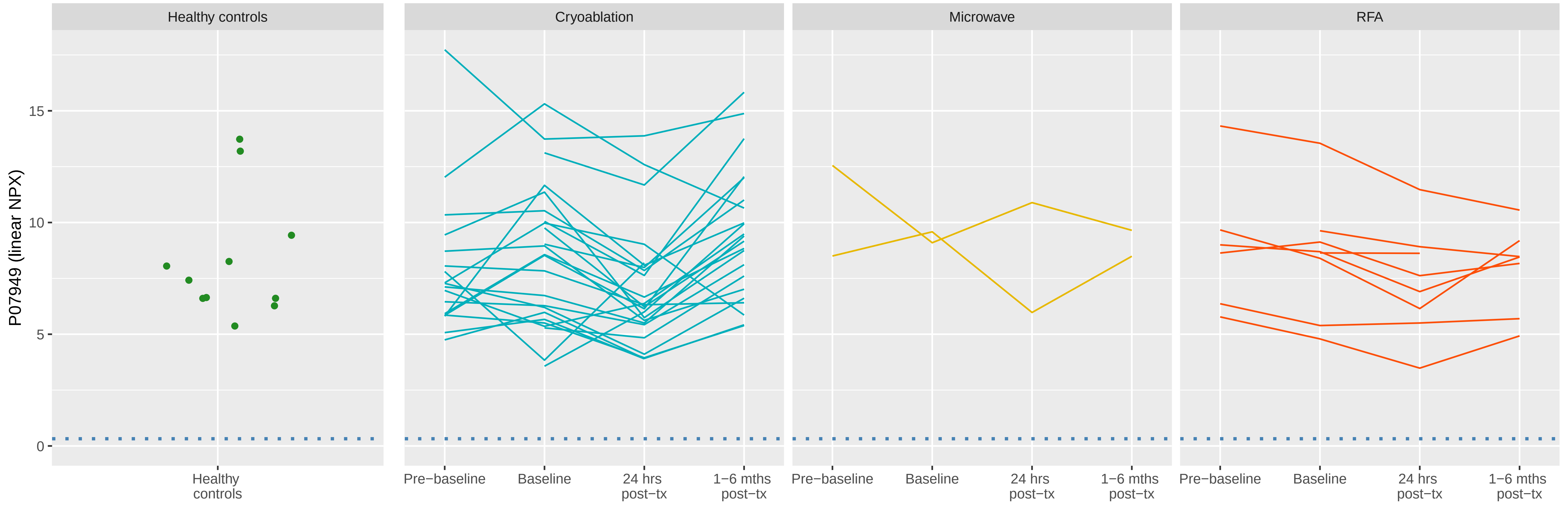

Uniprot ID: P08670, analyte:VIM

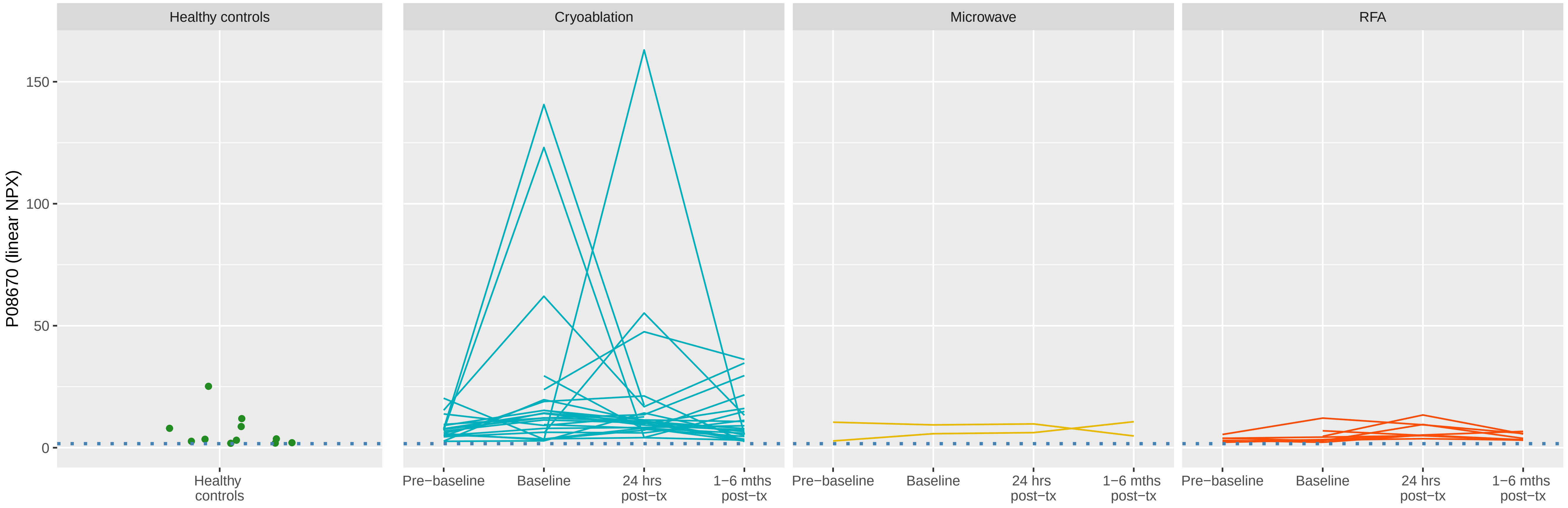

Uniprot ID: Q9NS68, analyte:TNFRSF19

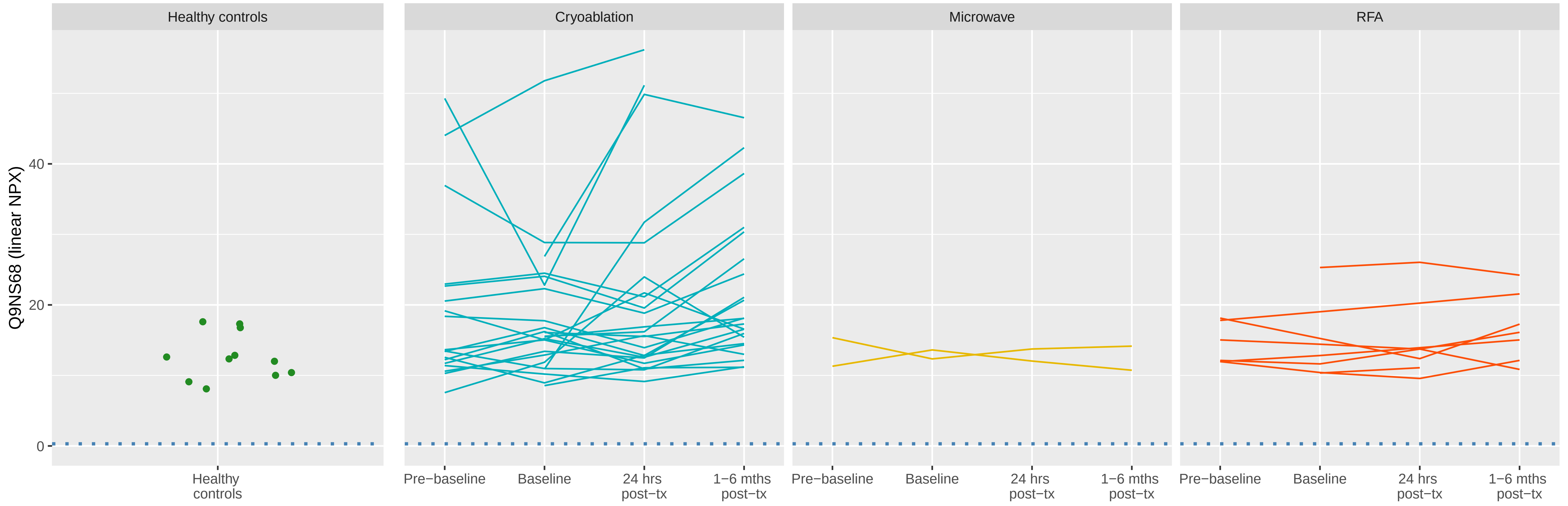

Uniprot ID: Q9UBG3, analyte:CRNN

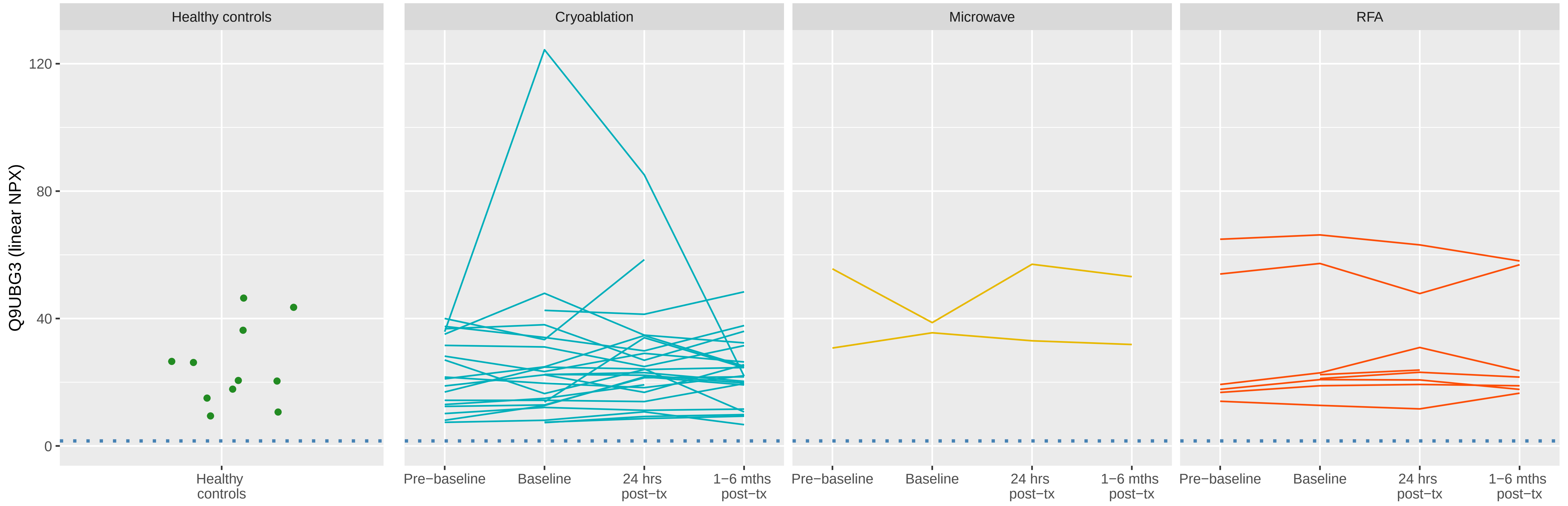

Uniprot ID: P56279, analyte:TCL1A

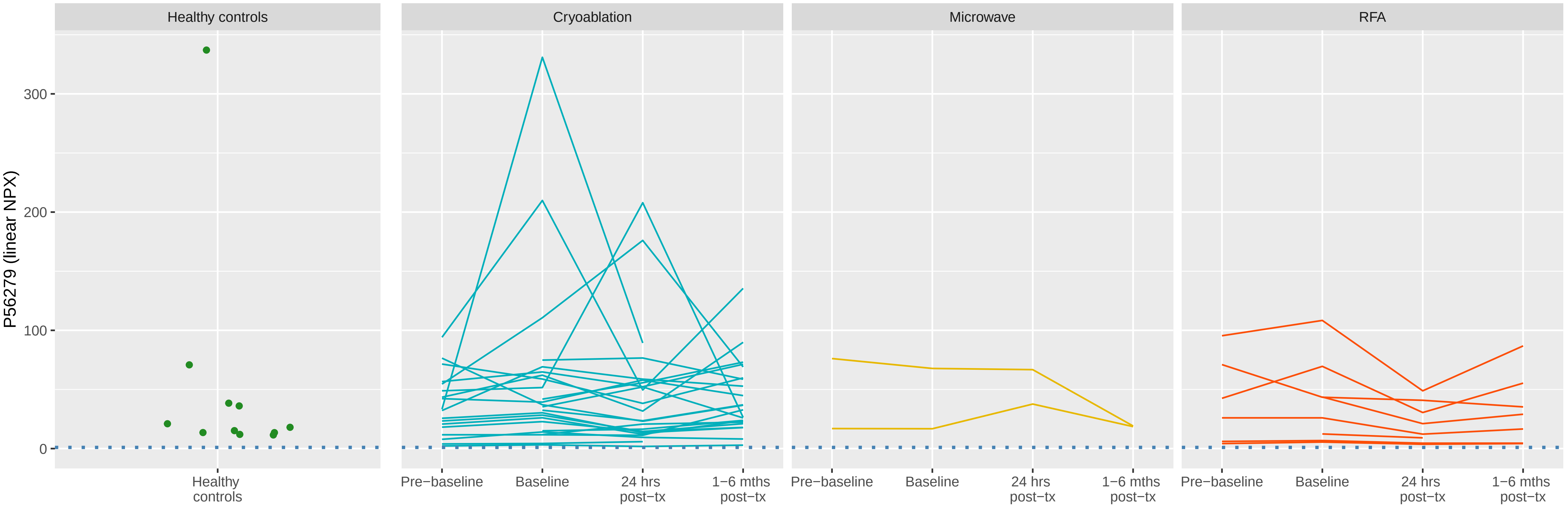

Uniprot ID: O95971, analyte:CD160

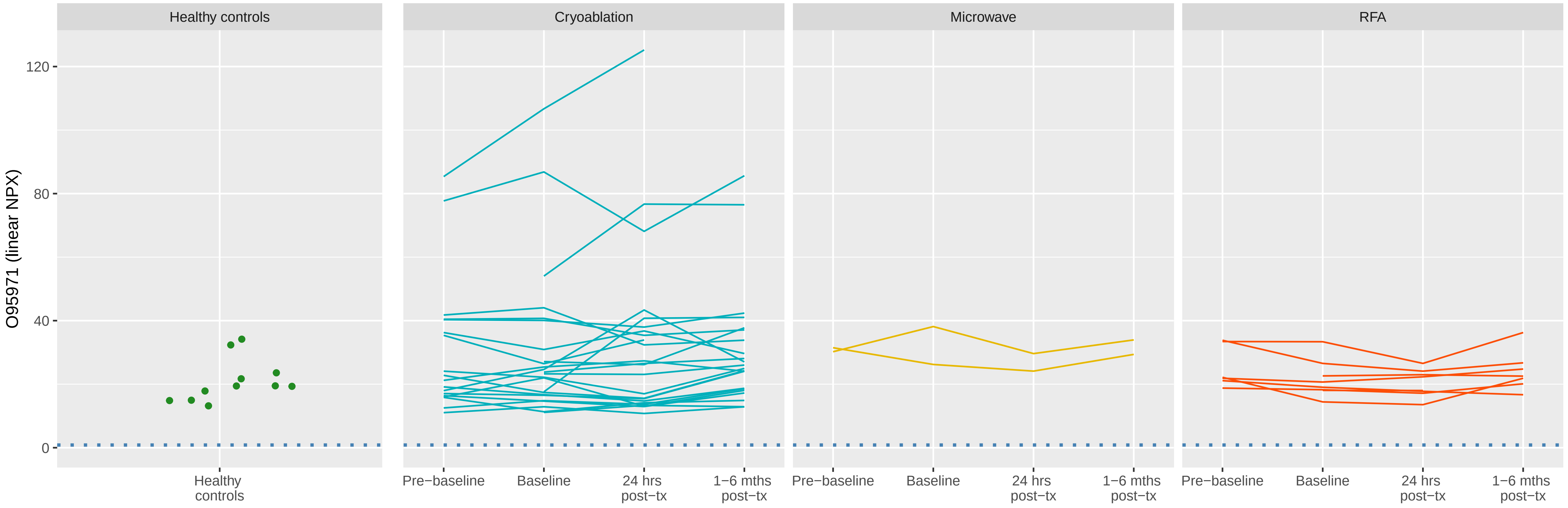

Uniprot ID: P43489, analyte:TNFRSF4

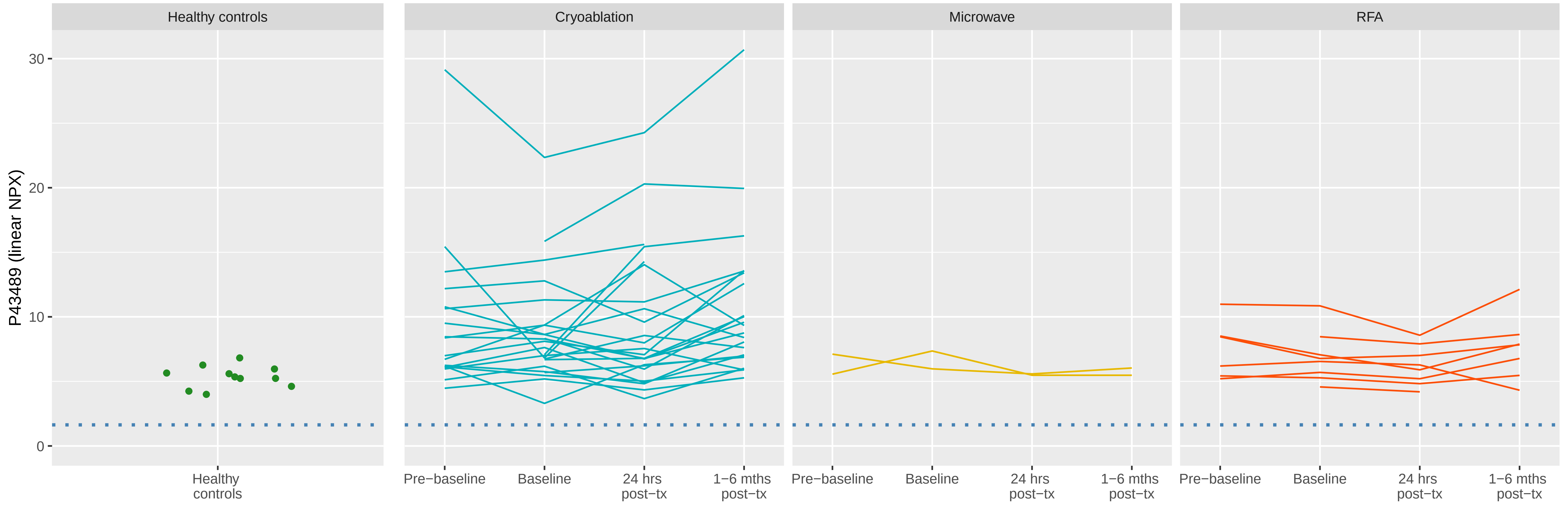

Uniprot ID: Q29983.Q29980, analyte:MIC−A/B

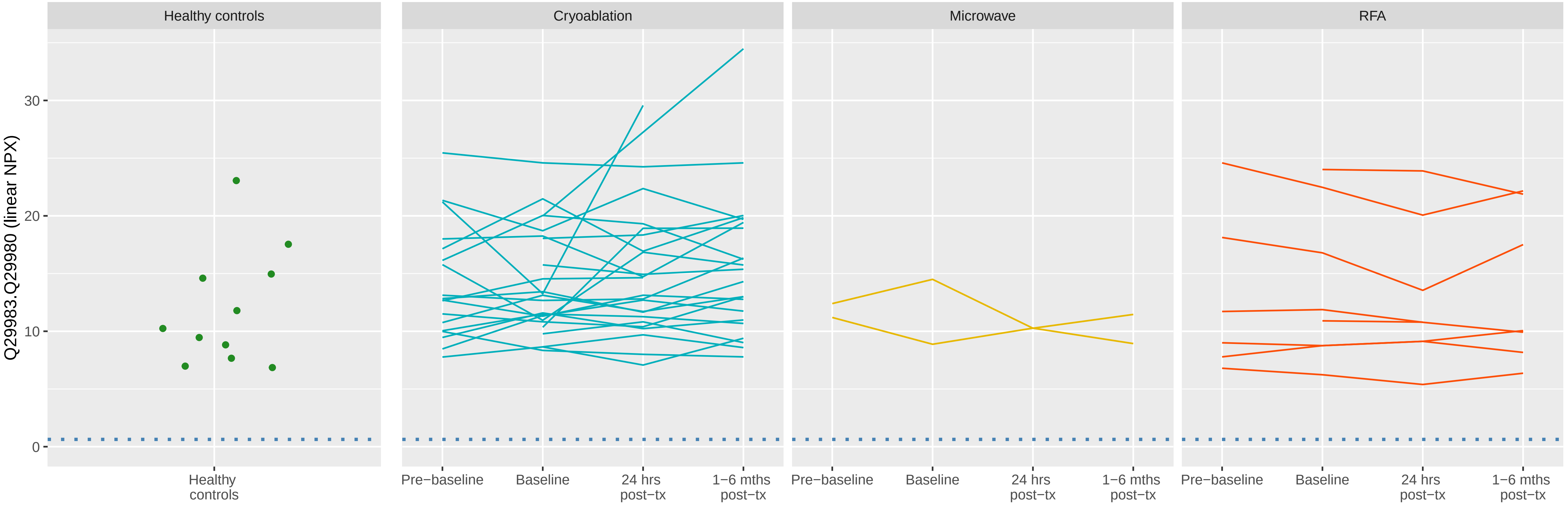

Uniprot ID: O95388, analyte:WISP−1

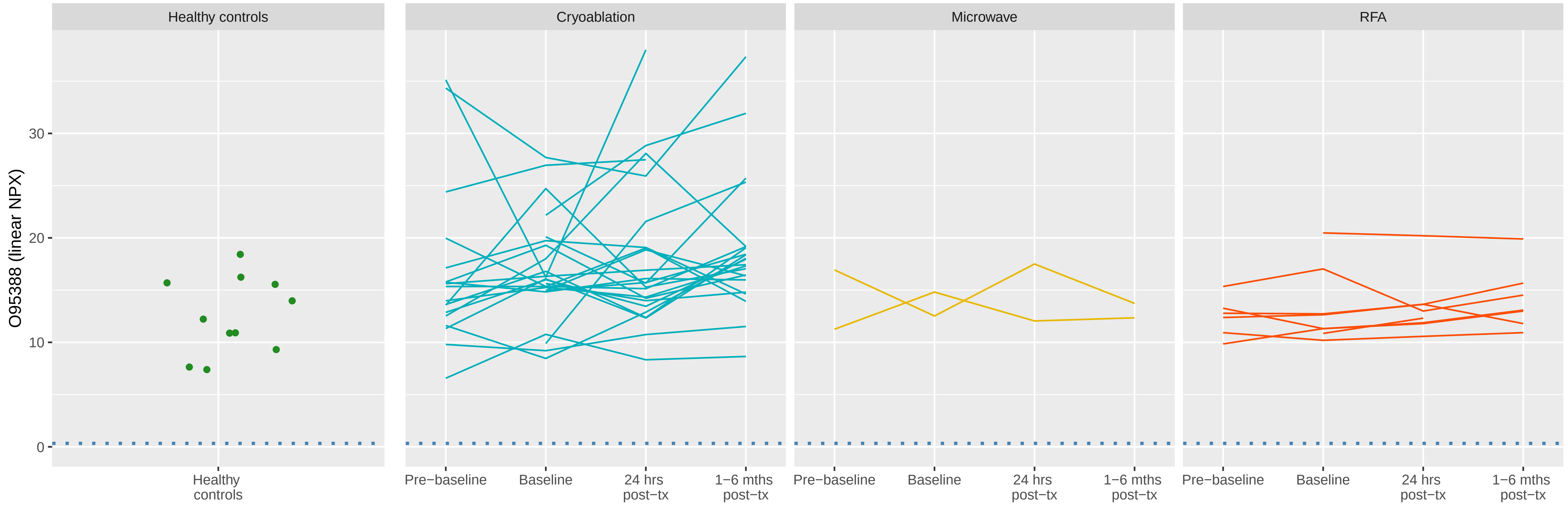

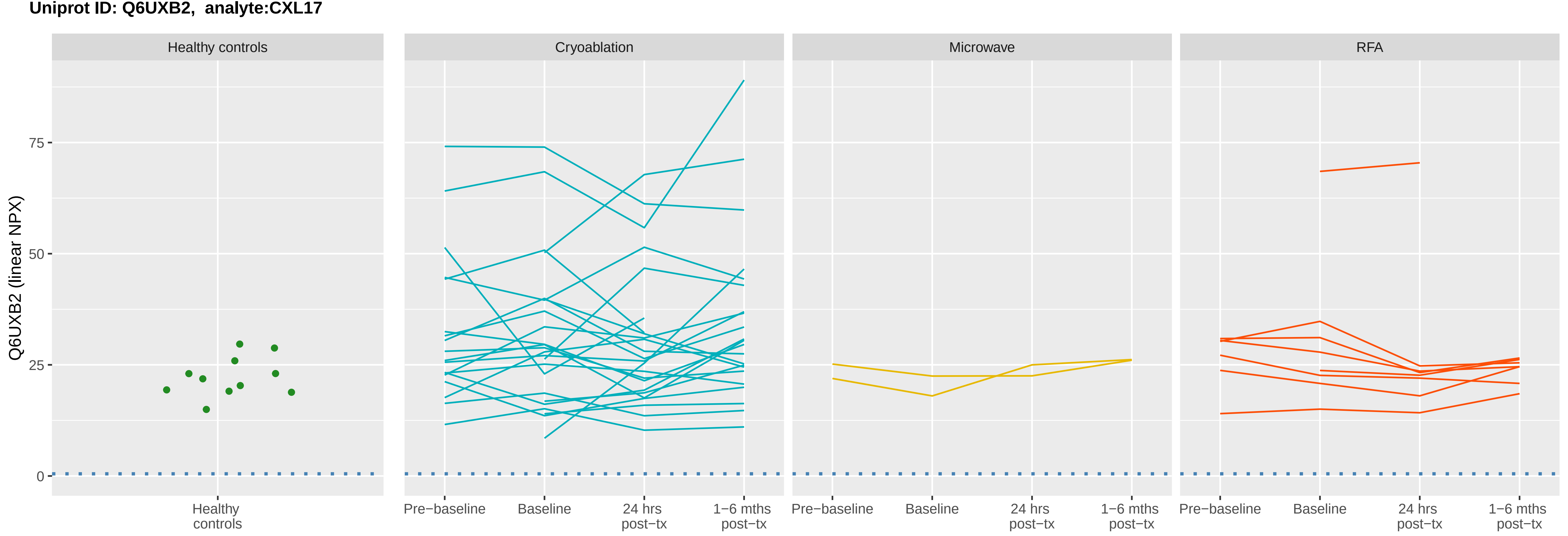

Uniprot ID: Q6UXB2, analyte:CXL17
Healthy controls
Cryoablation
Microwave
RFA
Q6UXB2 (linear NPX)
75
50
25
0
Healthy controls
Pre−baseline
Baseline
24 hrs post−tx
1−6 mths post−tx

Uniprot ID: P01298, analyte:PPY

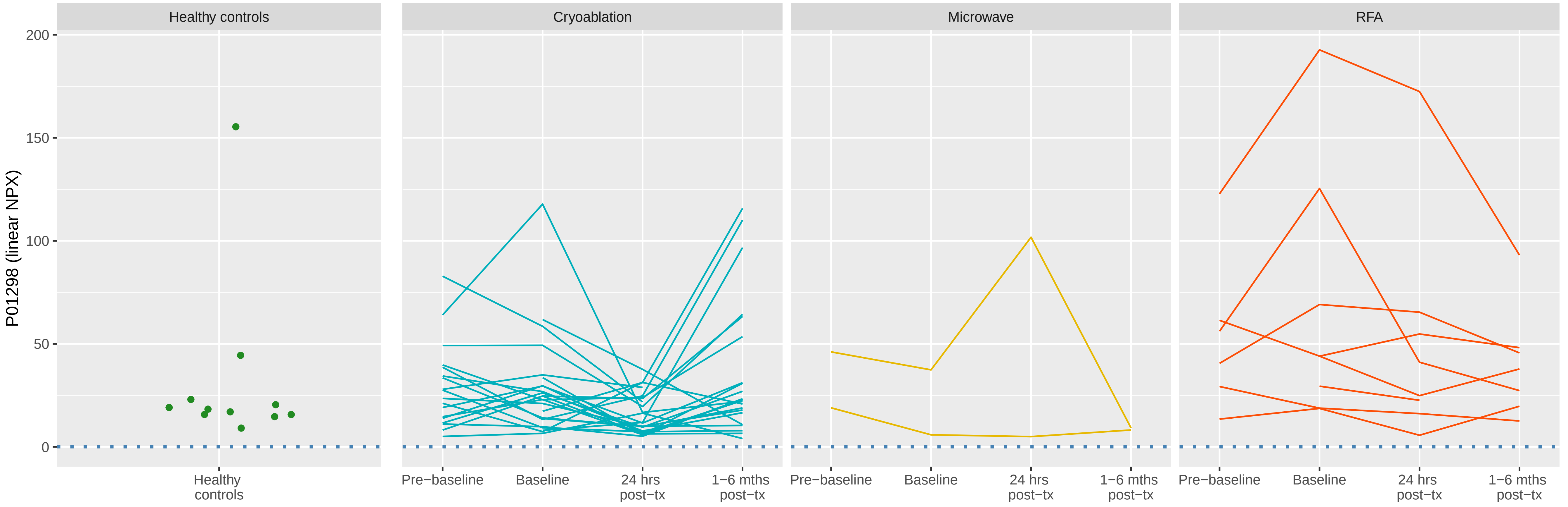

Uniprot ID: P31949, analyte:S100A11

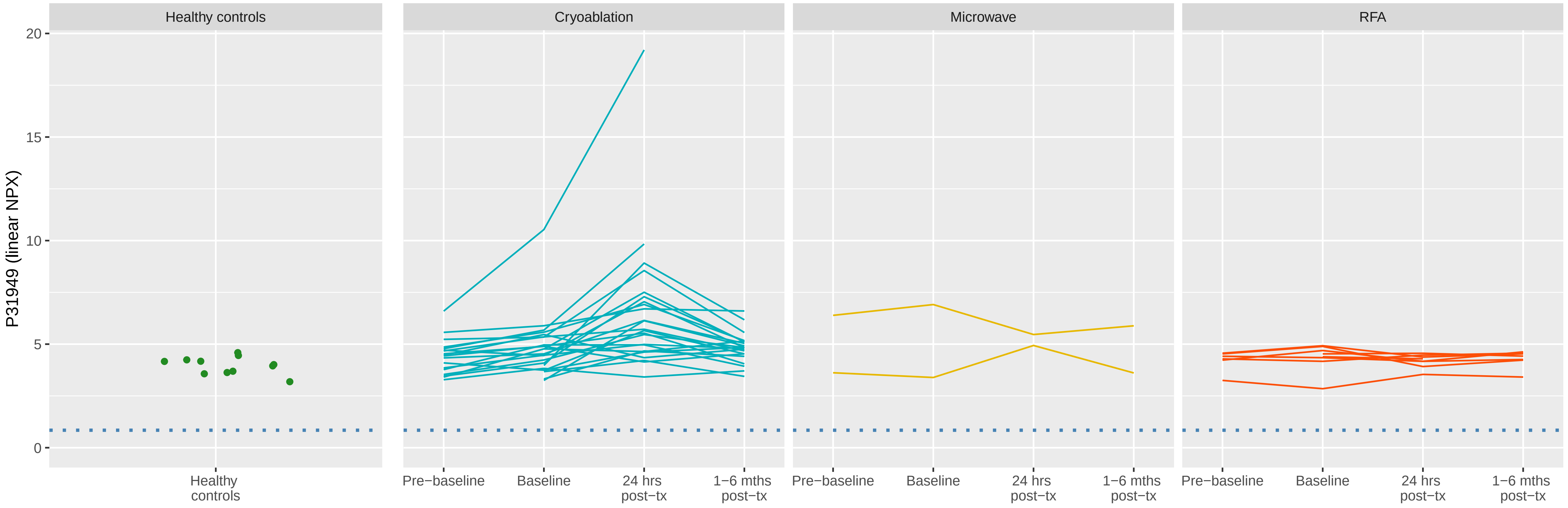

Uniprot ID: P15514, analyte:AREG

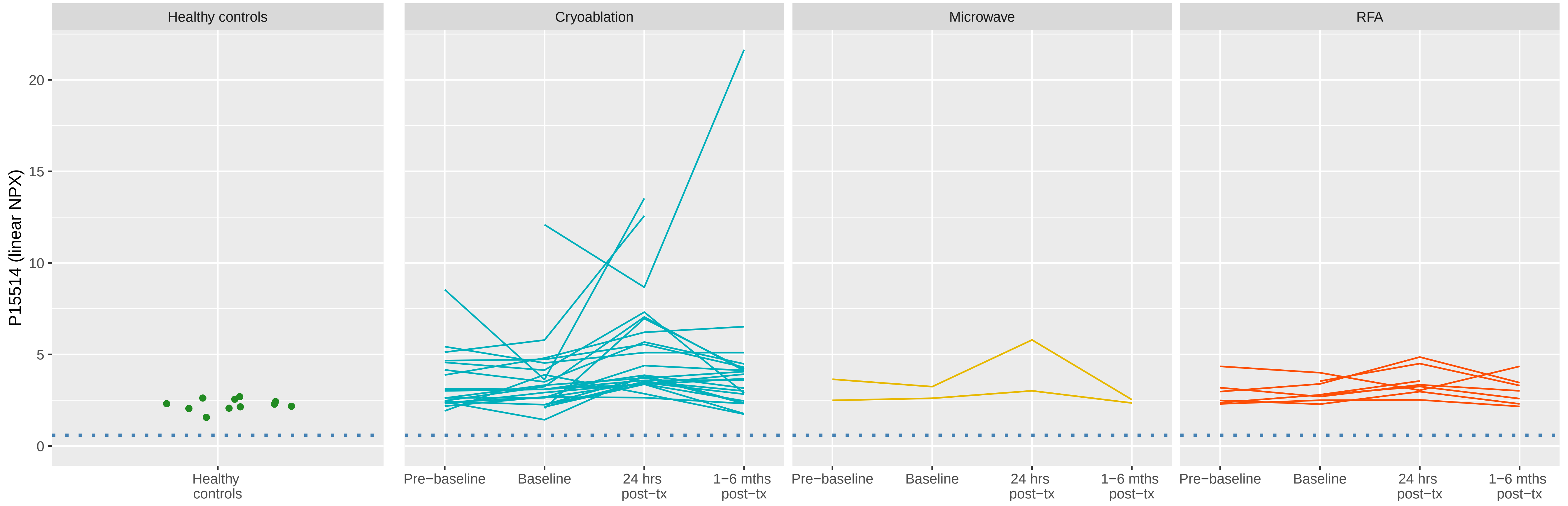

Uniprot ID: Q9NQ30, analyte:ESM−1

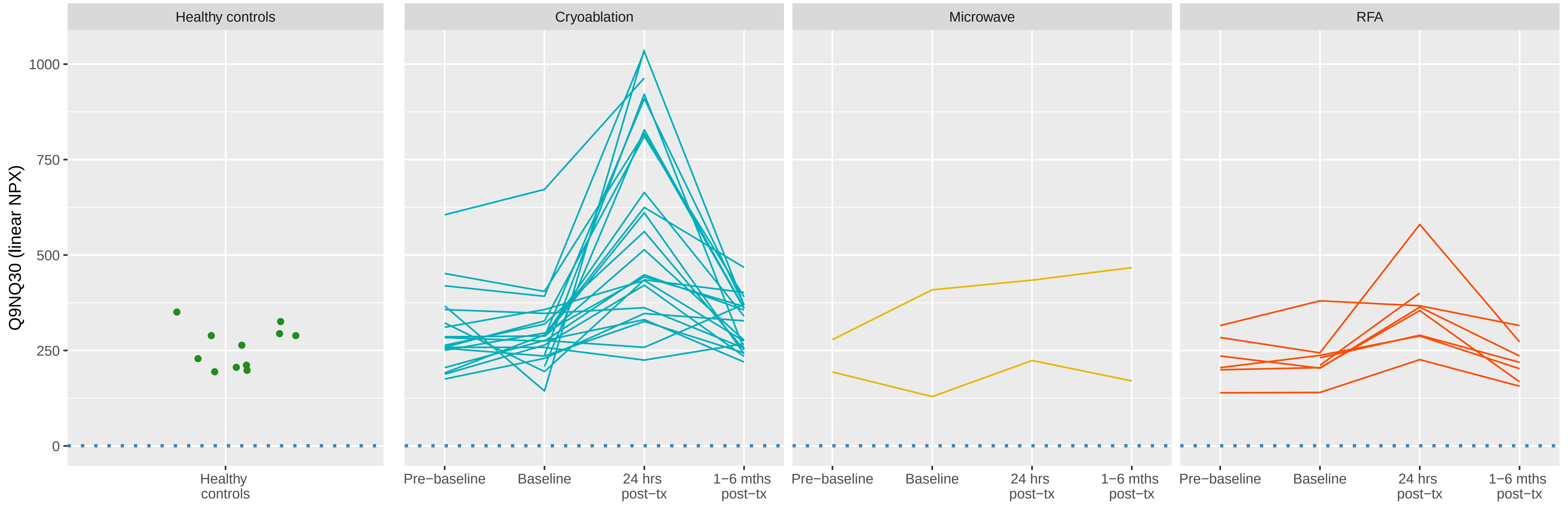

Uniprot ID: Q9UJ71, analyte:CD207

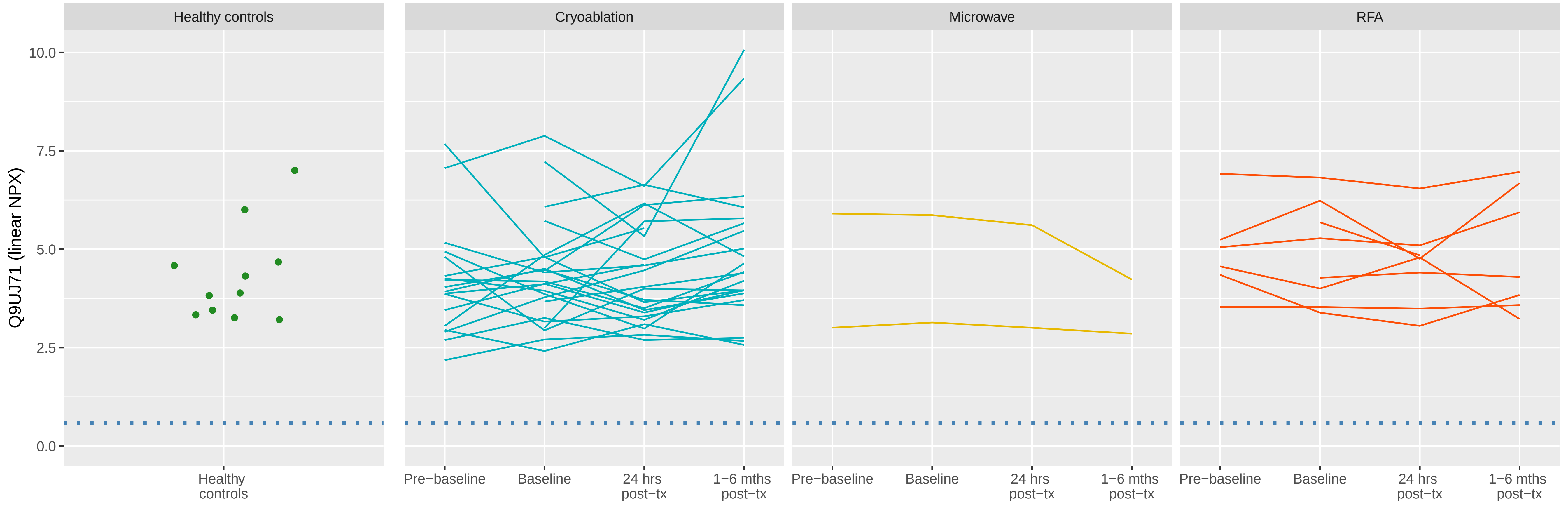

Uniprot ID: O75144, analyte:ICOSLG

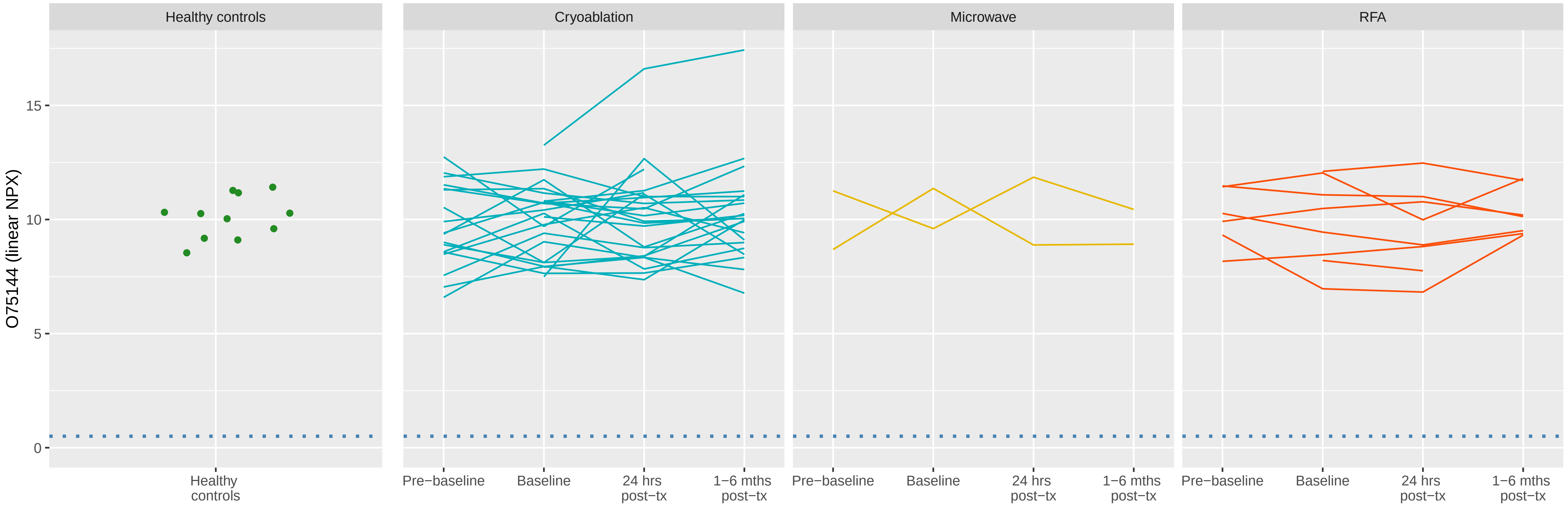

Uniprot ID: Q14508, analyte:WFDC2

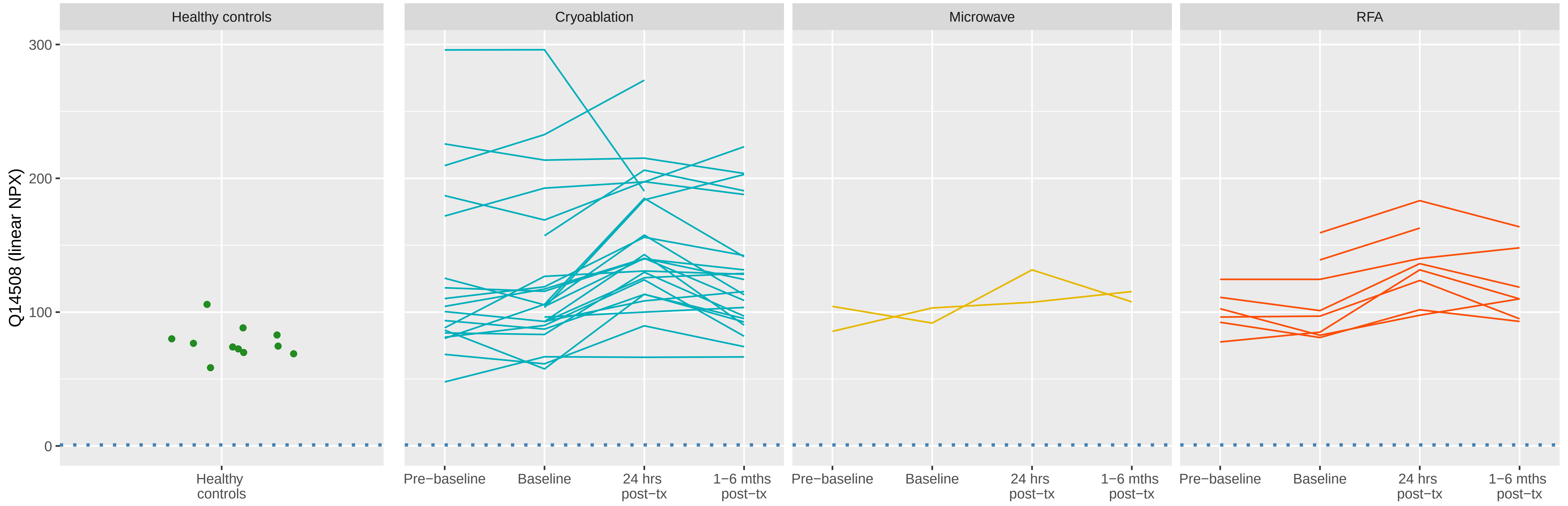

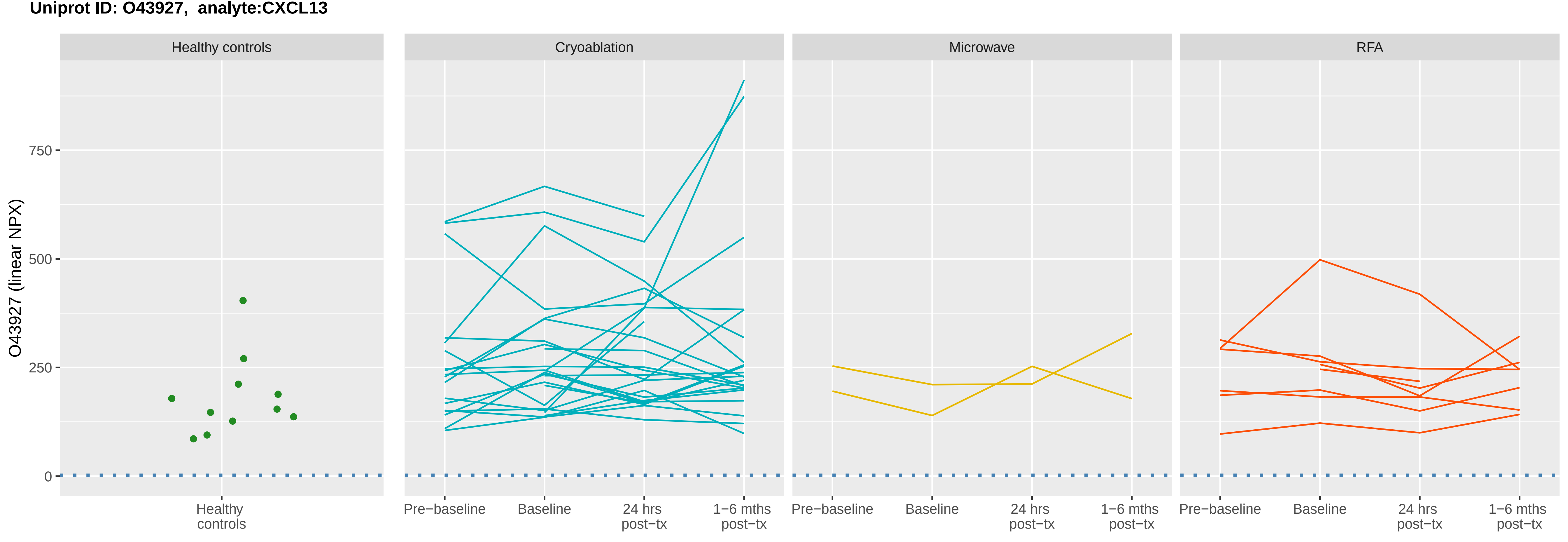
Uniprot ID: O43927, analyte:CXCL13
Healthy controls
Cryoablation
Microwave
RFA
O43927 (linear NPX)
750
500
250
0
Healthy controls
Pre−baseline
Baseline
24 hrs post−tx
1−6 mths post−tx

Uniprot ID: Q99717, analyte:MAD homolog 5

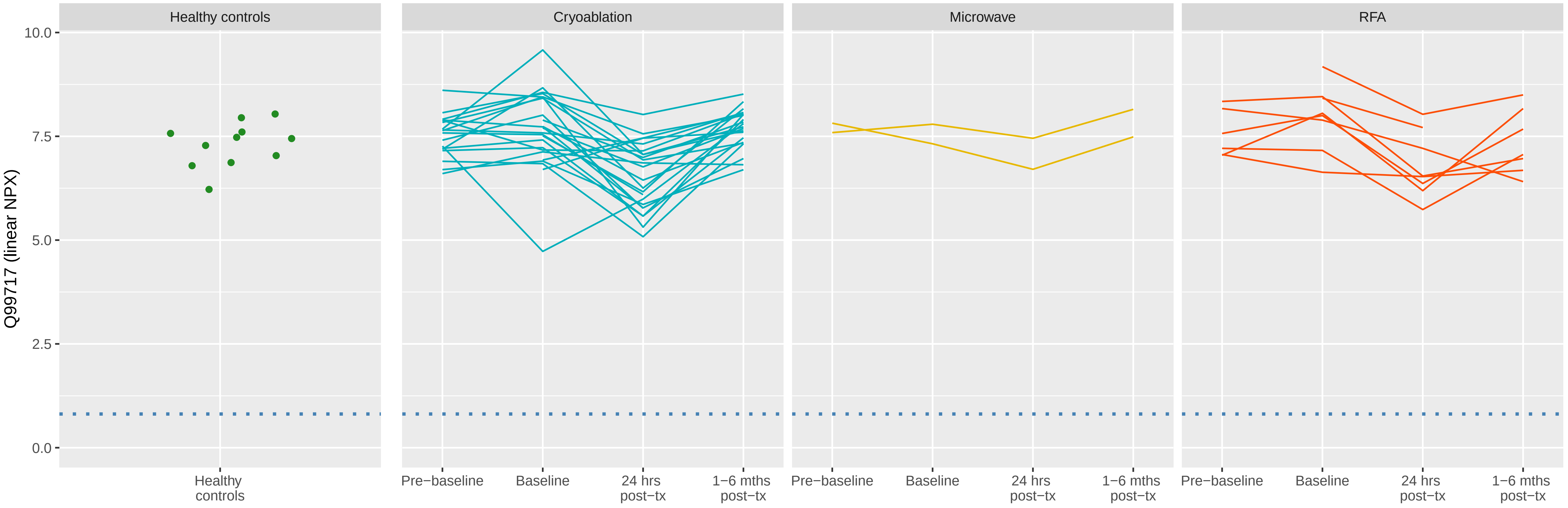

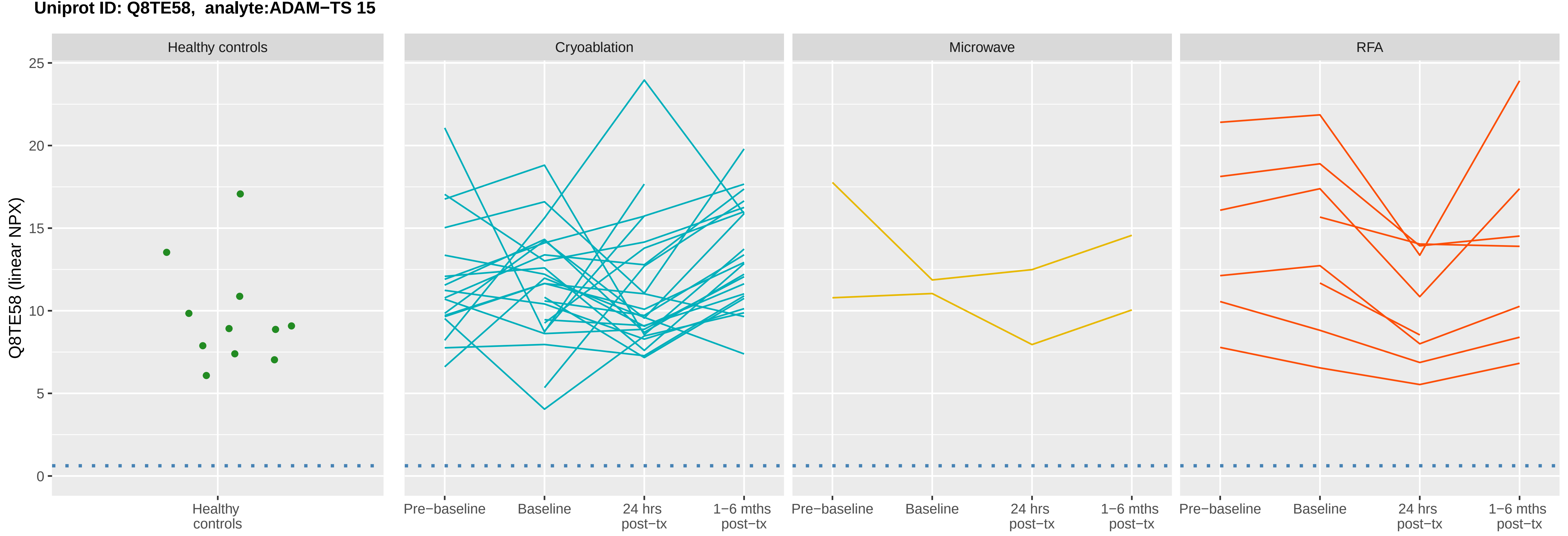

Uniprot ID: Q8TE58, analyte:ADAM−TS 15
Healthy controls
Cryoablation
Microwave
RFA
Q8TE58 (linear NPX)
25
20
15
10
5
0
Healthy controls
Pre−baseline
Baseline
24 hrs post−tx
1−6 mths post−tx

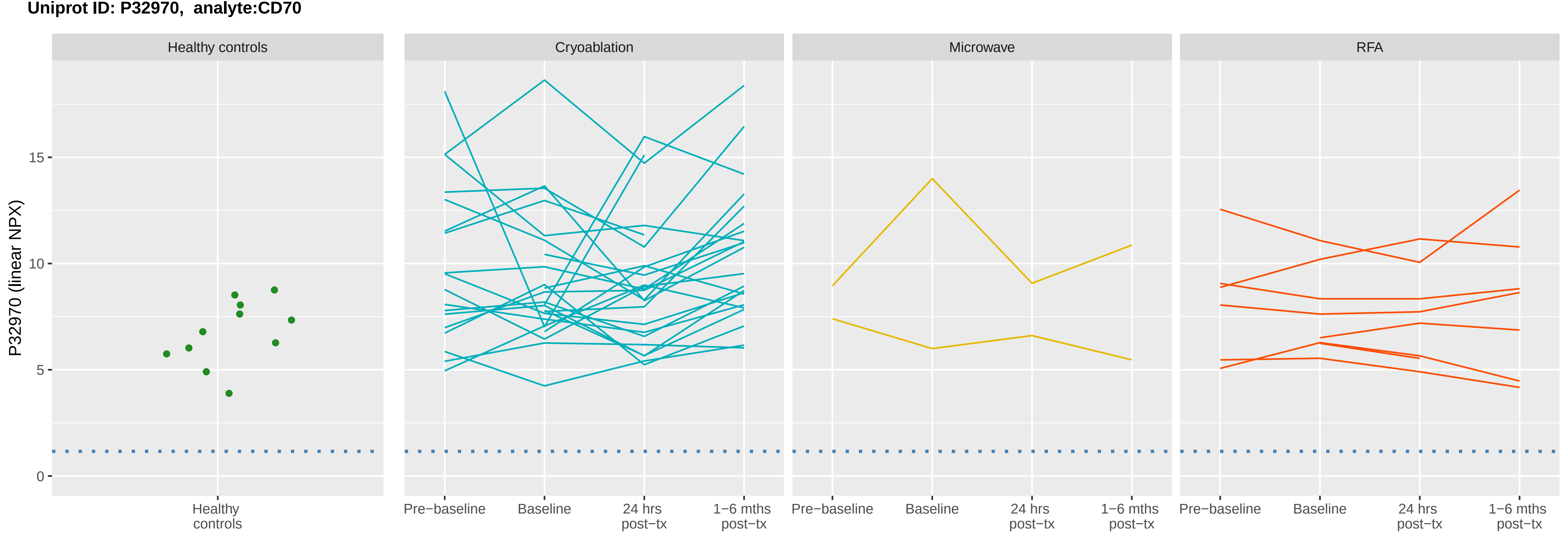
Uniprot ID: P32970, analyte:CD70
Healthy controls
Cryoablation
Microwave
RFA
P32970 (linear NPX)
15
10
5
0
Healthy controls
Pre−baseline
Baseline
24 hrs post−tx
1−6 mths post−tx

Uniprot ID: Q9BXY4, analyte:RSPO3

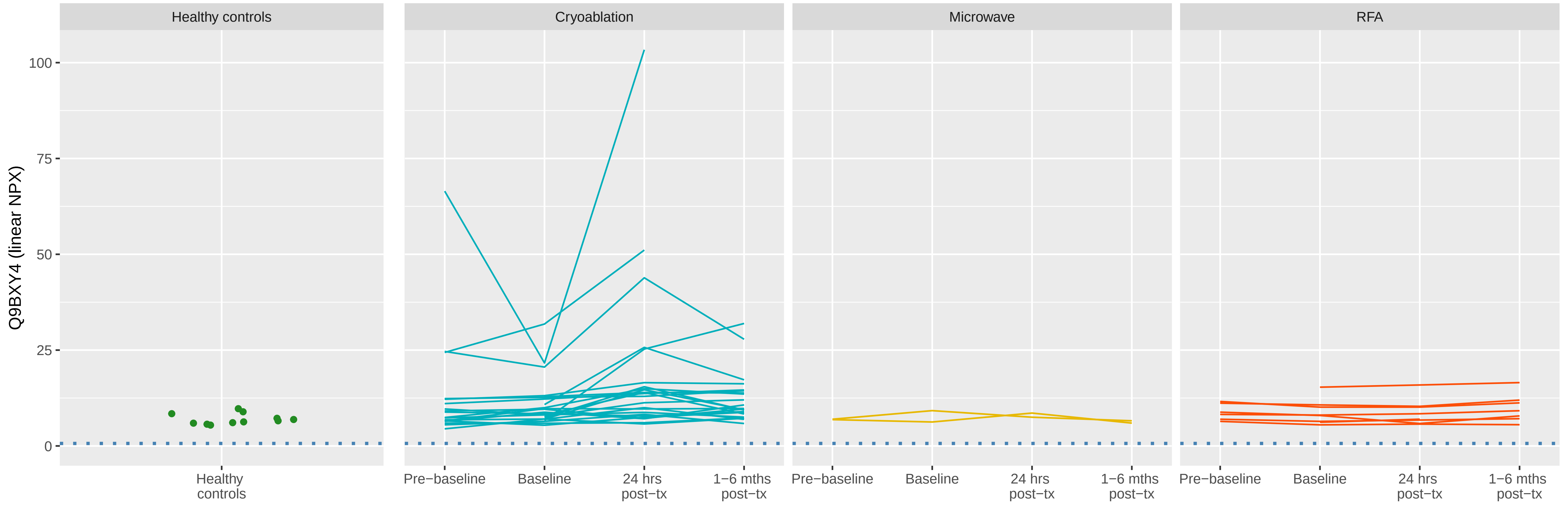

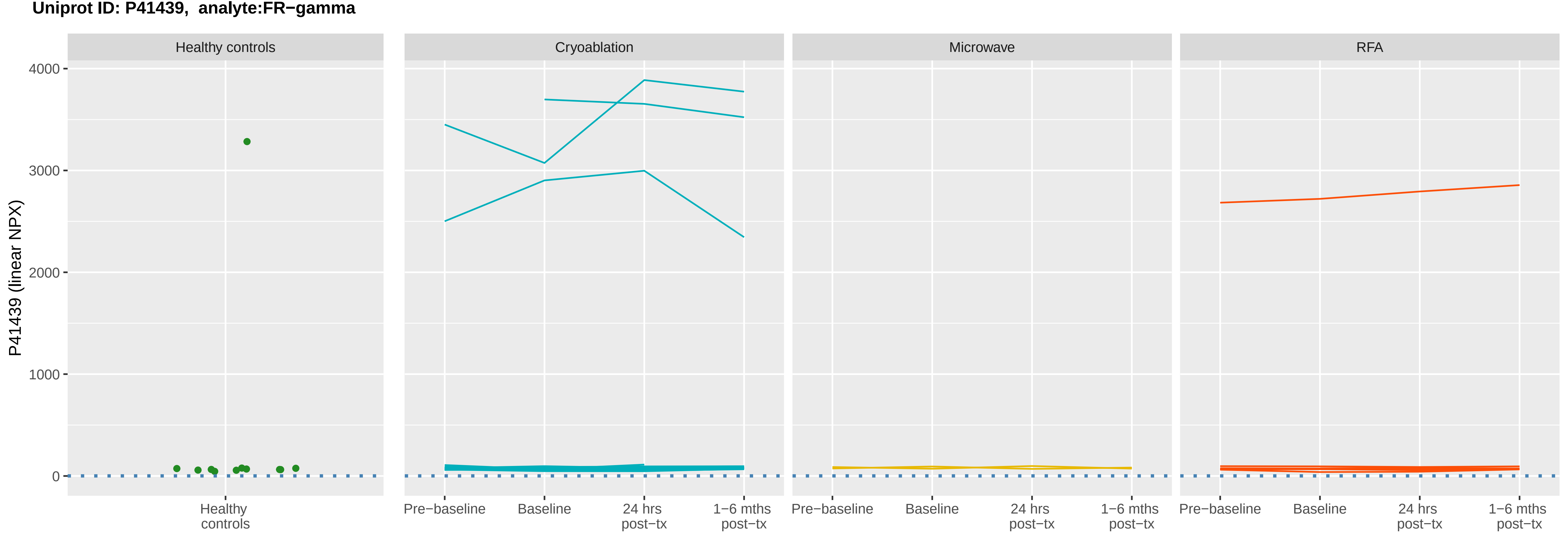
Uniprot ID: P41439, analyte:FR−gamma
Healthy controls
Cryoablation
Microwave
RFA
P41439 (linear NPX)
4000
3000
2000
1000
0
Healthy controls
Pre−baseline
Baseline
24 hrs post−tx
1−6 mths post−tx

Uniprot ID: P06731, analyte:CEACAM5

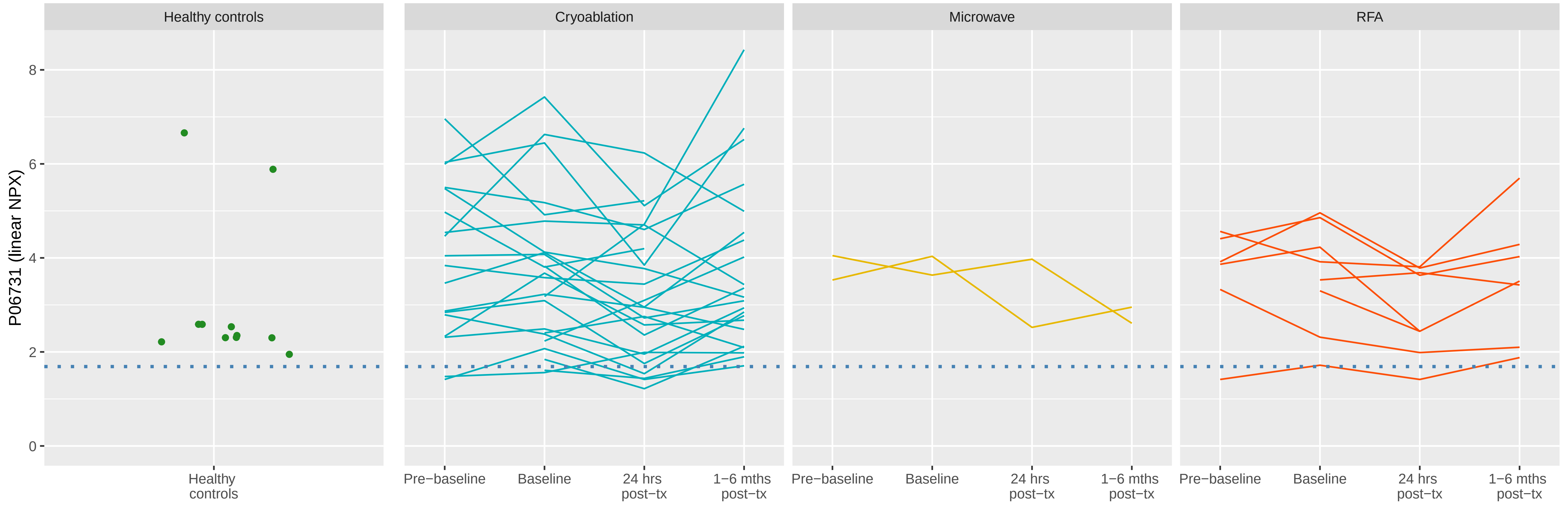

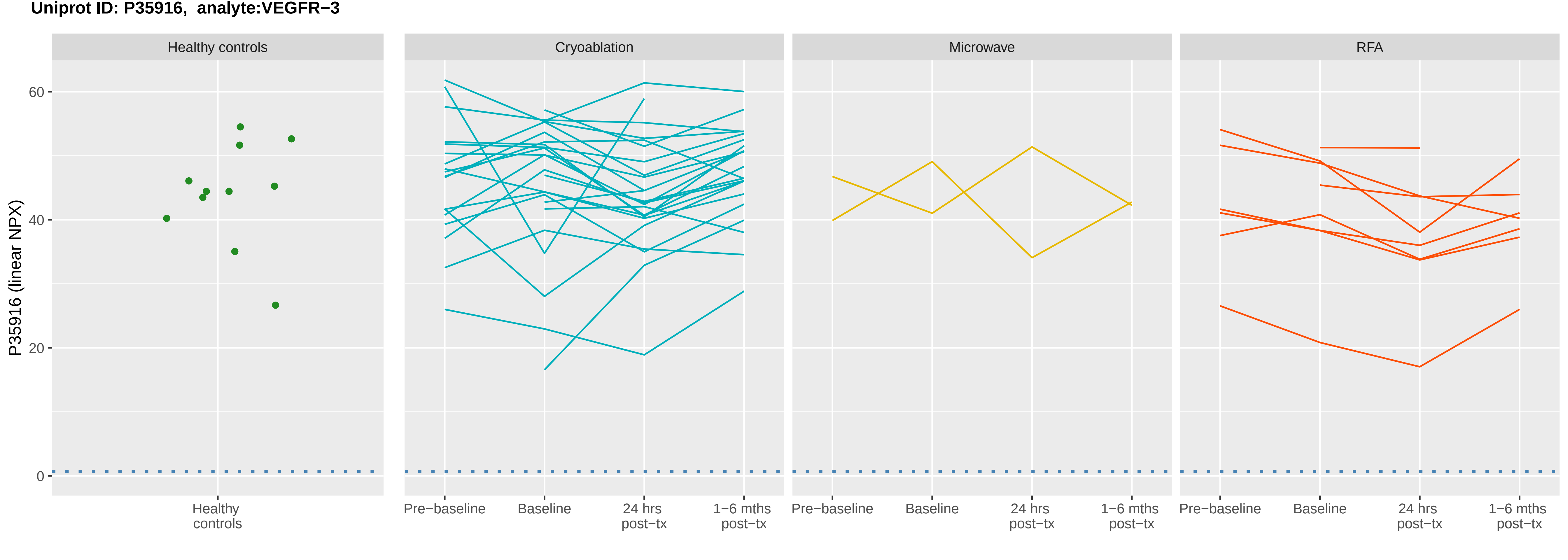

Uniprot ID: P35916, analyte:VEGFR−3
Healthy controls
Cryoablation
Microwave
RFA
P35916 (linear NPX)
60
40
20
0
Healthy controls
Pre−baseline
Baseline
24 hrs post−tx
1−6 mths post−tx

Uniprot ID: Q8WXI7, analyte:MUC−16

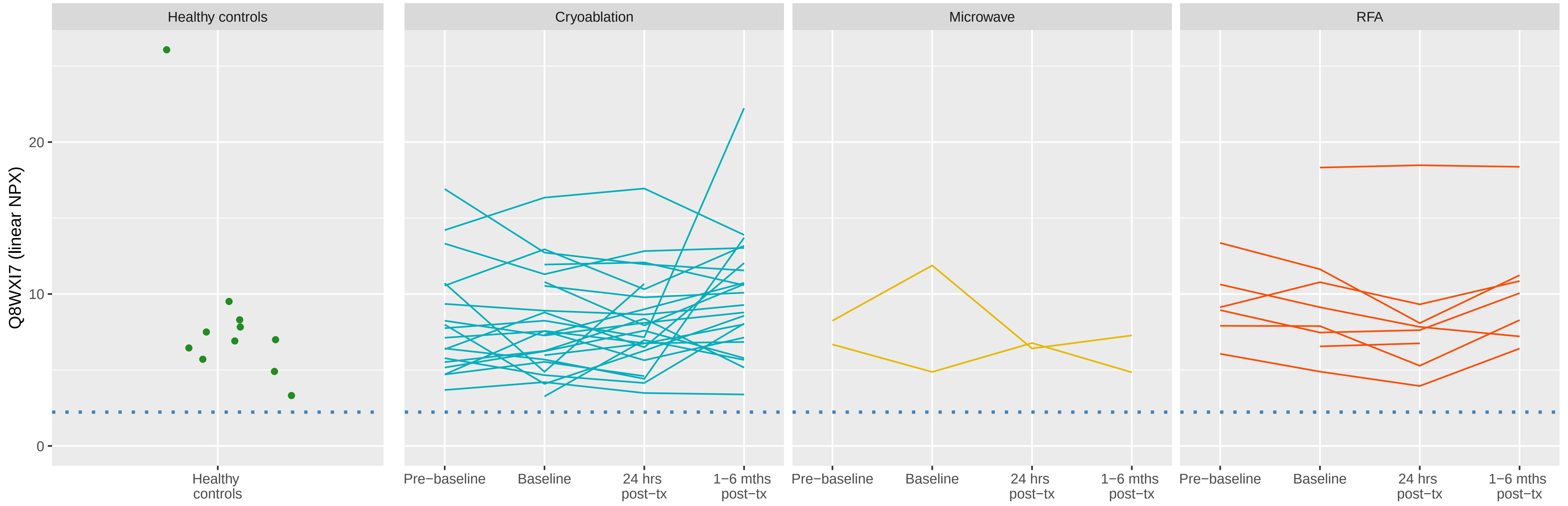

Uniprot ID: Q9Y5W5, analyte:WIF−1

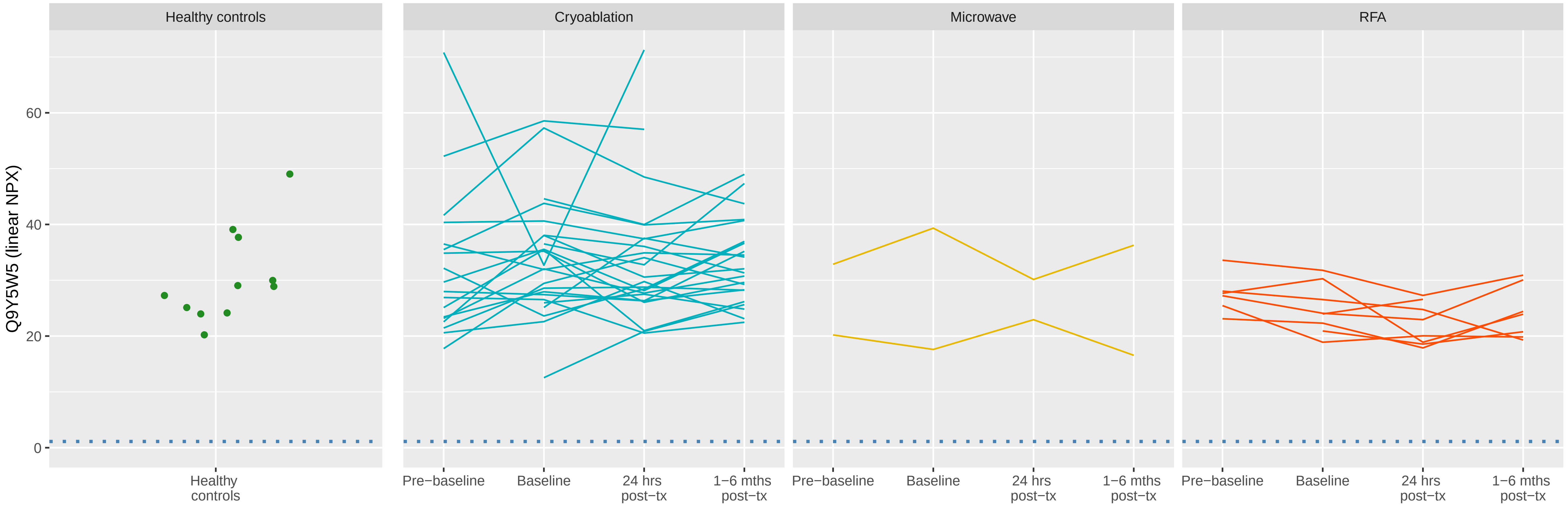

Uniprot ID: P10144, analyte:GZMB

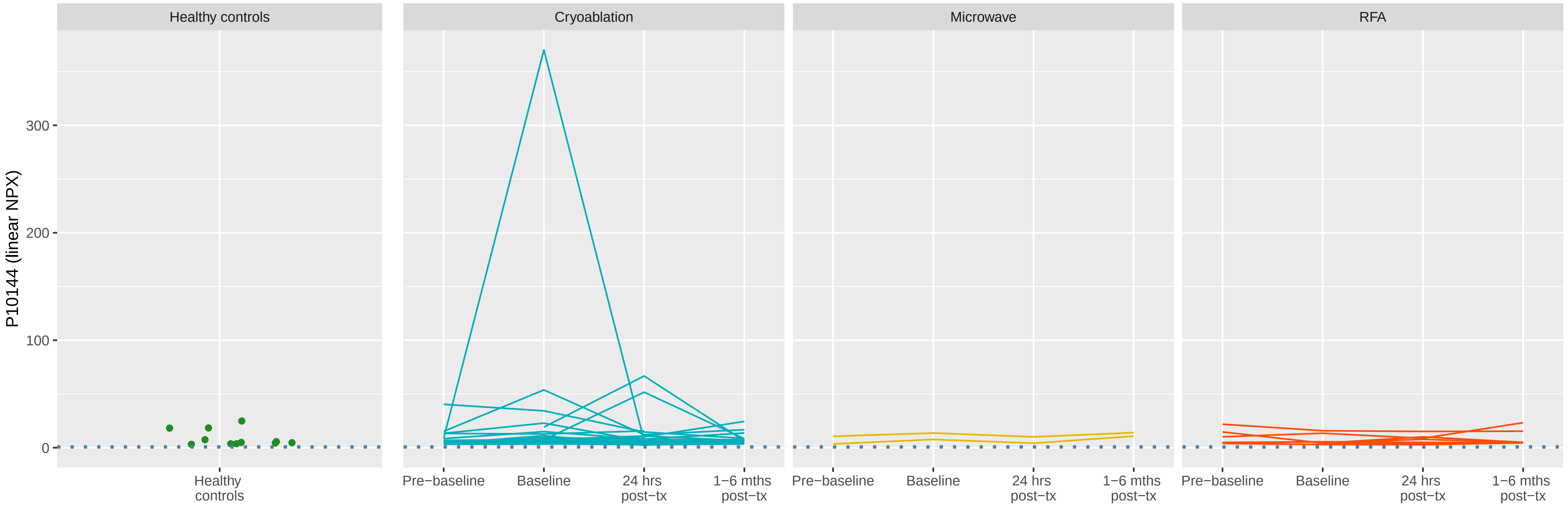

Uniprot ID: Q6BAA4, analyte:FCRLB

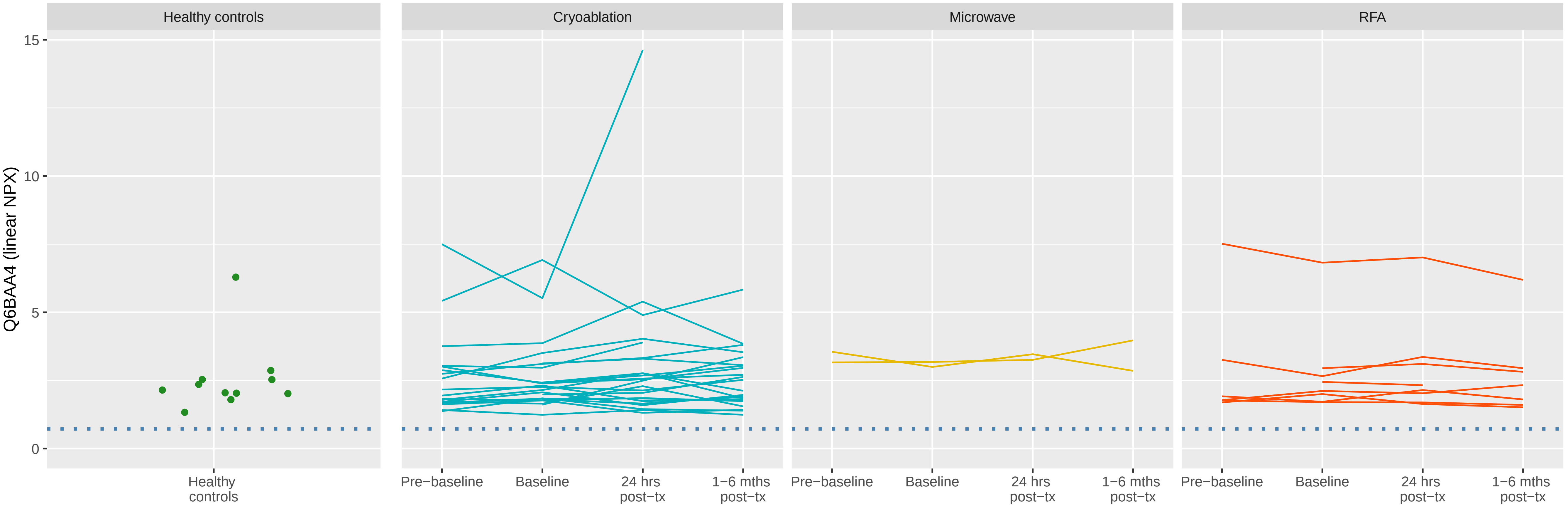

Uniprot ID: P04083, analyte:ANXA1

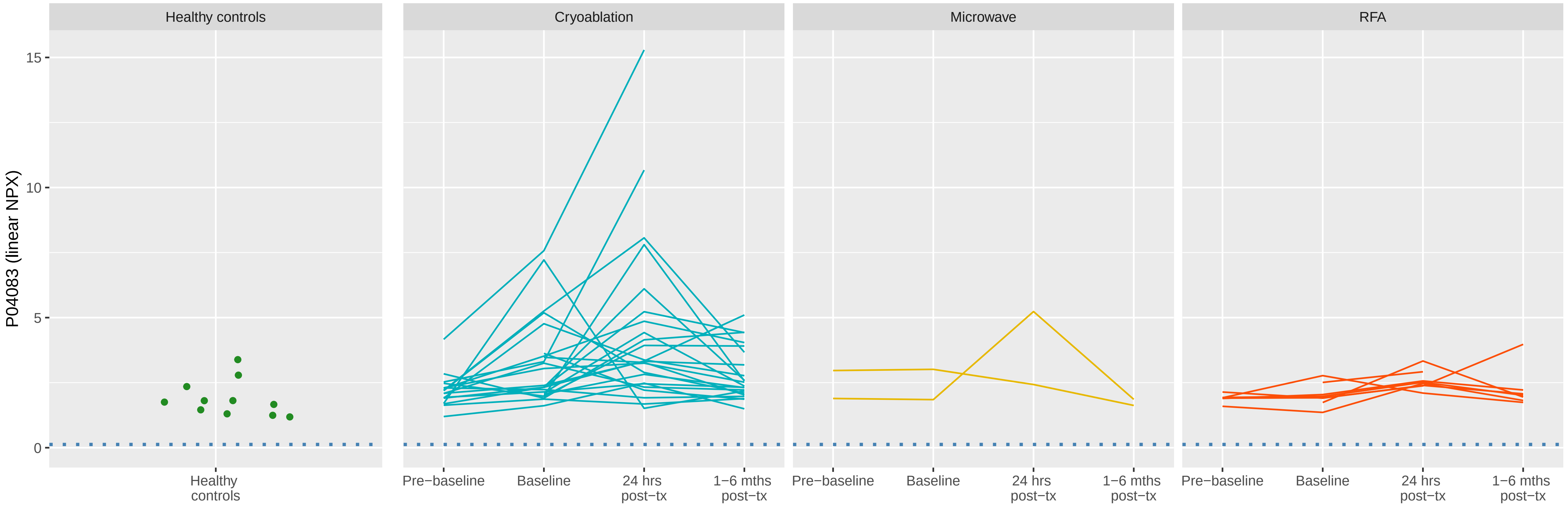

Uniprot ID: P15328, analyte:FR−alpha

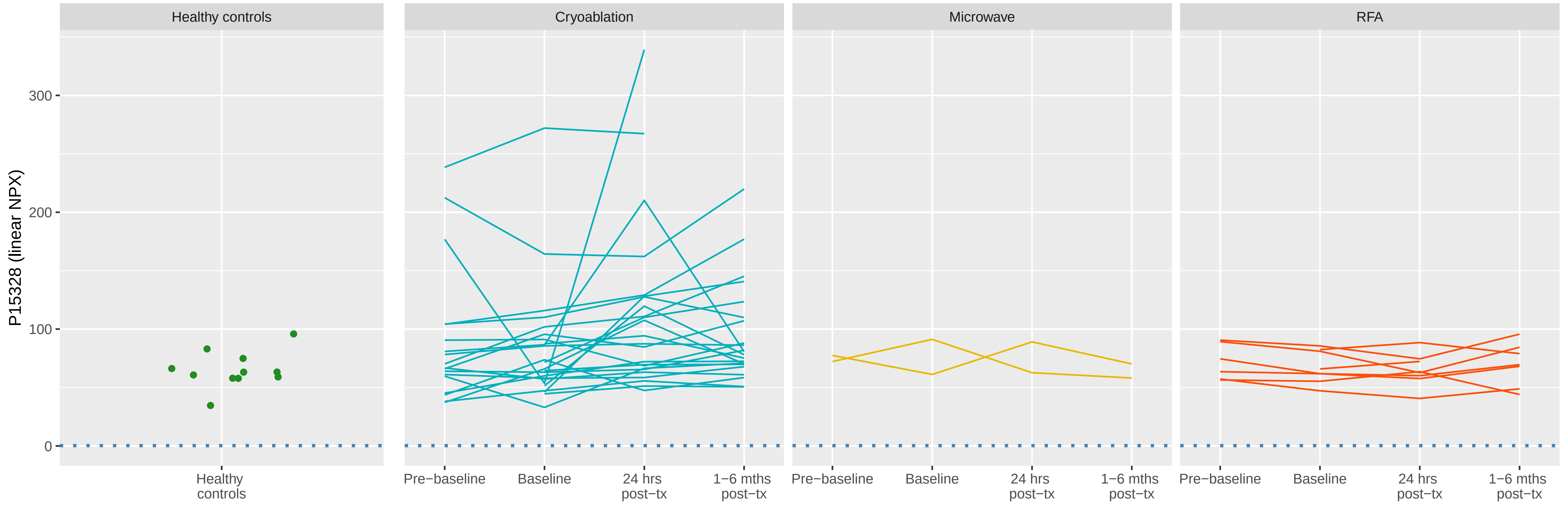

Supplement: Supplementary file 1 [file cancers-13-06037-s001.zip › Supplementary File/Figure S2.pdf]
