# Supplementary material for: An Exploratory Analysis of Changes in Circulating Plasma Protein Profiles Following Image-Guided Ablation of Renal Tumours Provides Evidence for Effects on Multiple Biological Processes
Source: Cancers (Basel). 2021 Nov 30;13(23):6037. doi: 10.3390/cancers13236037 (PMC8656737; doi:10.3390/cancers13236037)
Supplement: Supplementary file 1 [file cancers-13-06037-s001.zip › Supplementary File/Figure S3.pdf]

Figure S3. Longitudinal profiles of all plasma proteins on the Immunooncology panel, prior to and post-ablation. Results for each protein are expressed as a % of the reference baseline (TP2; 100%) and are shown for patients receiving CRYO (n=25), MWA (n=2) and RFA (n=8).

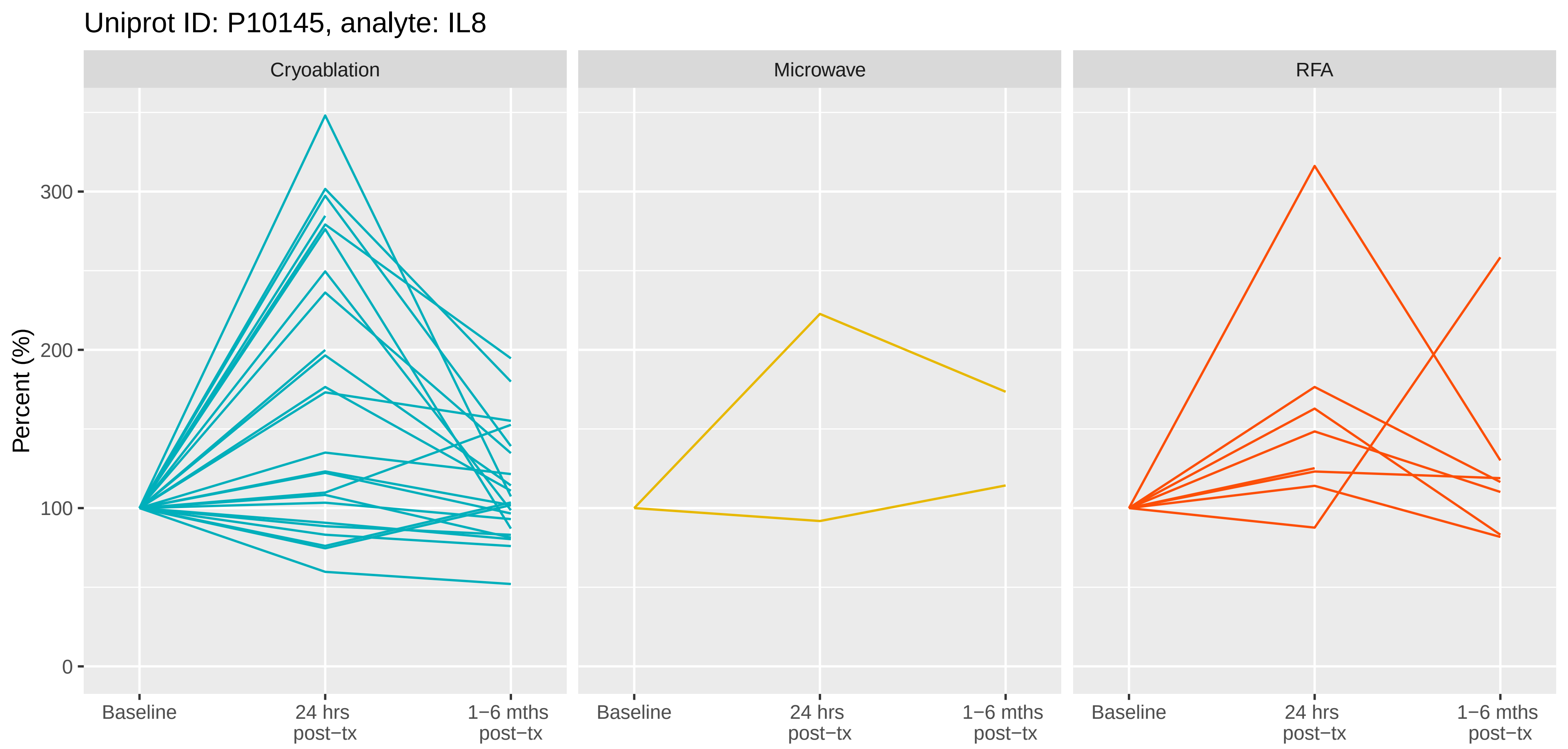

Uniprot ID: P10145, analyte: IL8
Cryoablation
Microwave
RFA
Percent (%)
300
200
100
0
Baseline
24 hrs post−tx
1−6 mths post−tx

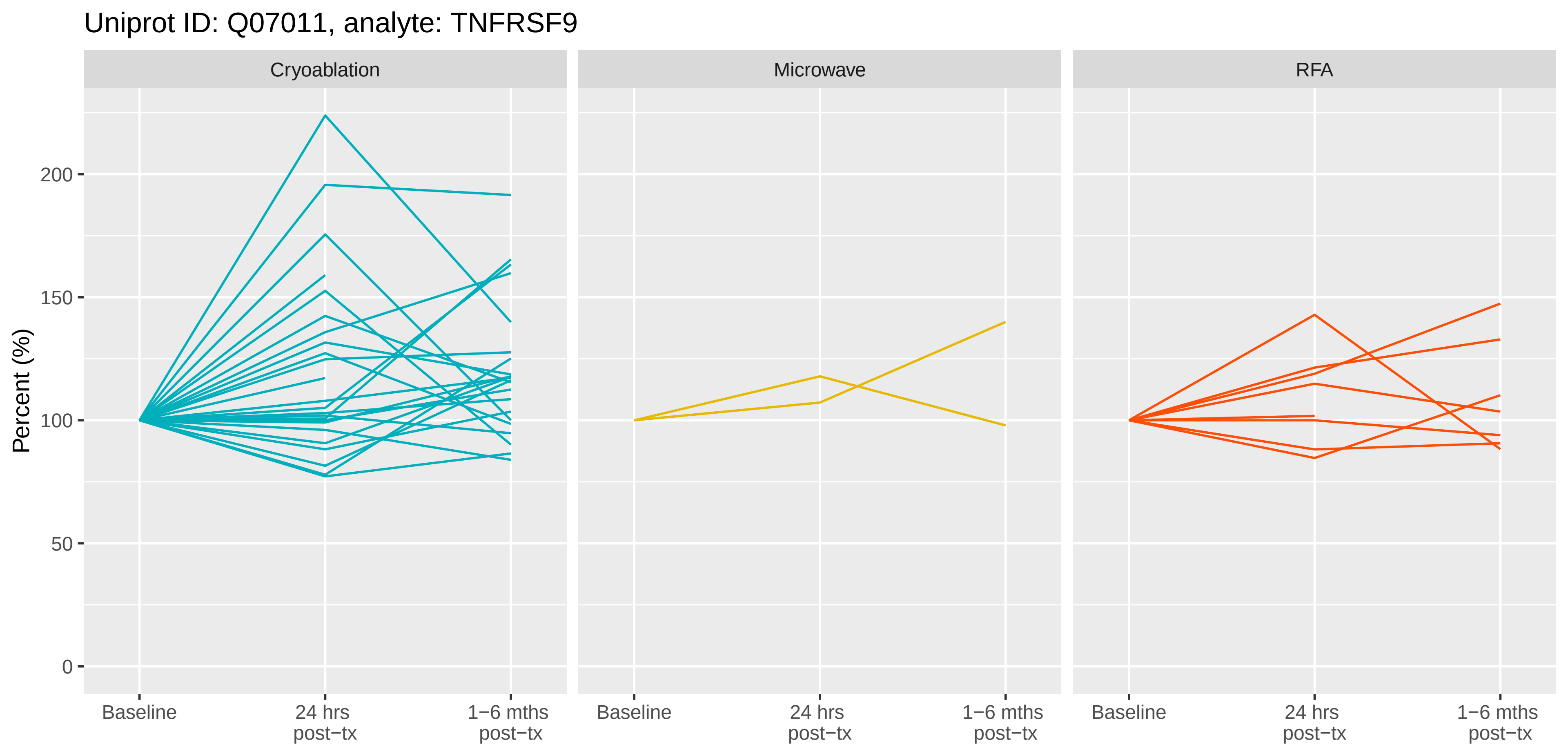

Uniprot ID: Q07011, analyte: TNFRSF9
Cryoablation
Microwave
RFA
Percent (%)
200
150
100
50
0
Baseline
24 hrs post−tx
1−6 mths post−tx

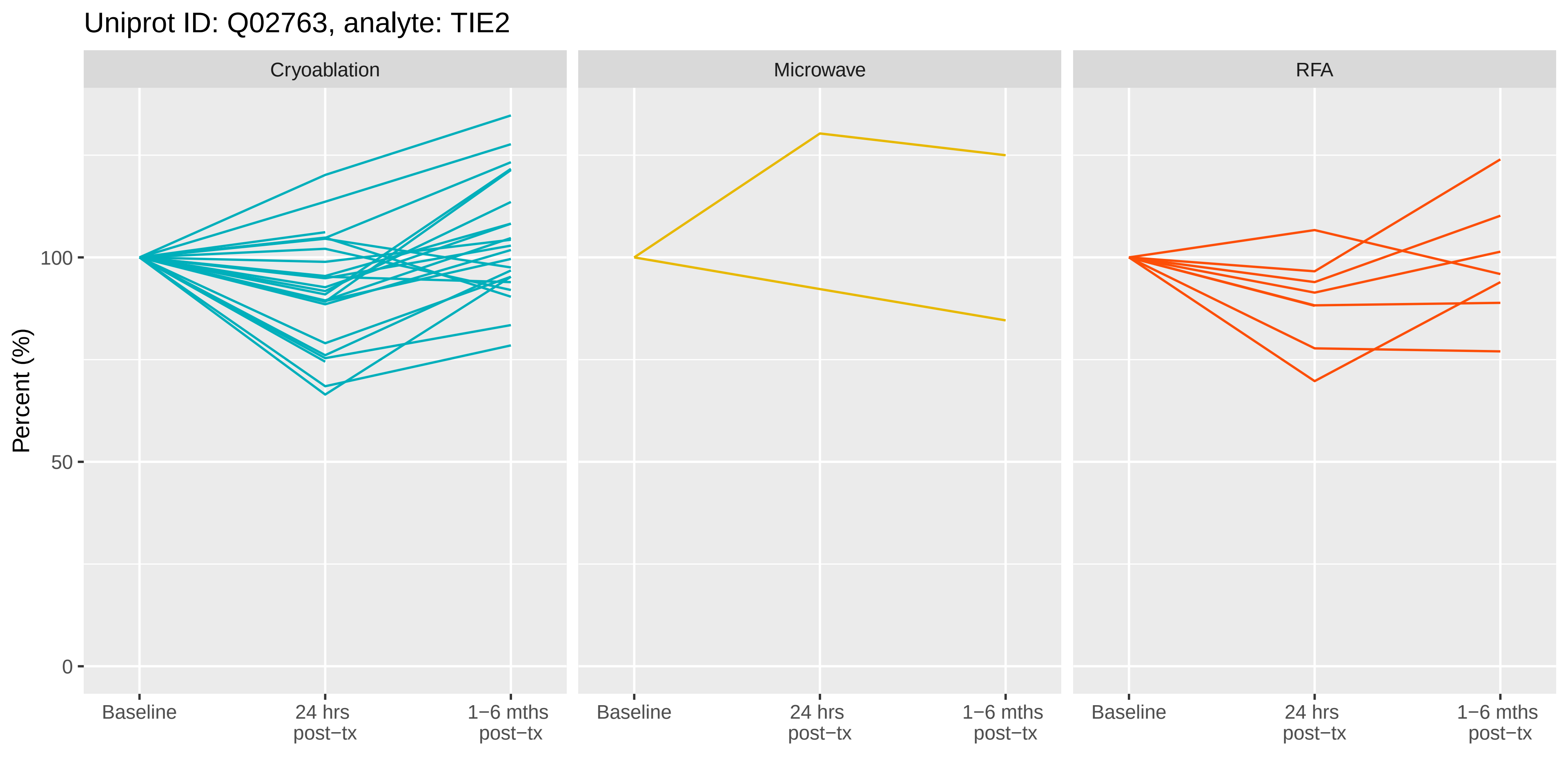
Uniprot ID: Q02763, analyte: TIE2
Cryoablation
Microwave
RFA
Percent (%)
100
50
0
Baseline
24 hrs post−tx
1−6 mths post−tx

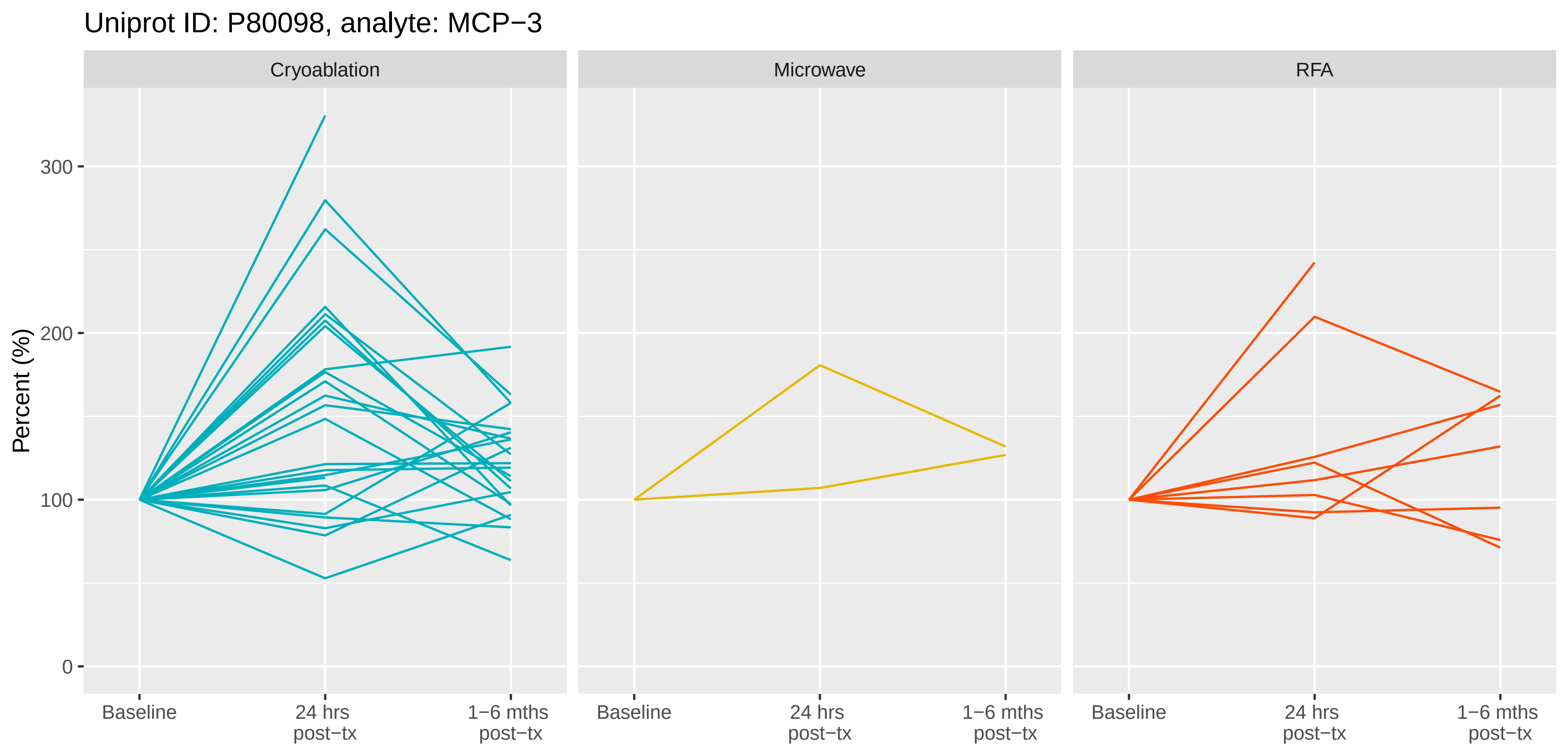

Uniprot ID: P80098, analyte: MCP−3
Cryoablation
Microwave
RFA
Percent (%)
300
200
100
0
Baseline
24 hrs post−tx
1−6 mths post−tx

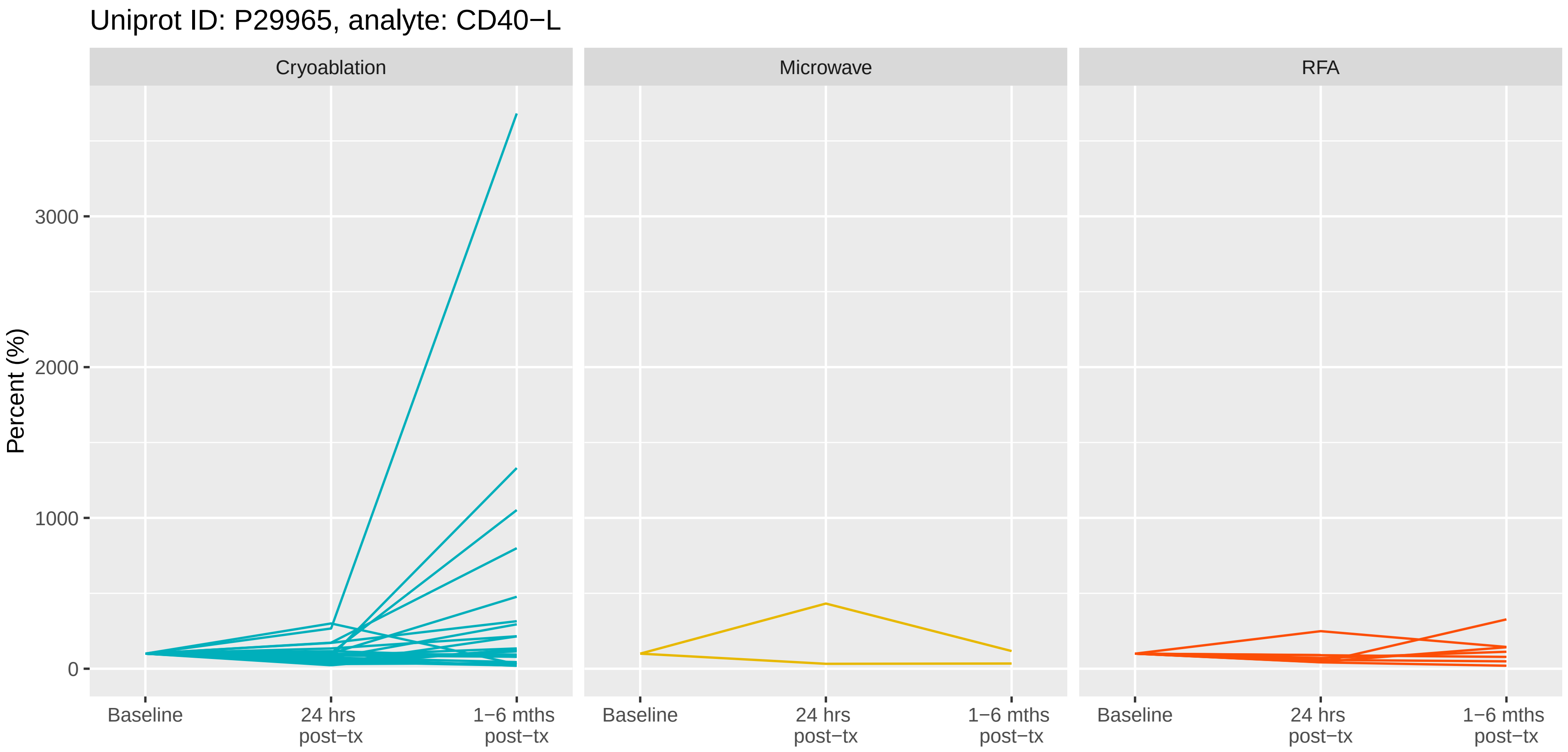

Uniprot ID: P29965, analyte: CD40−L
Cryoablation
Microwave
RFA
Percent (%)
3000
2000
1000
0
Baseline
24 hrs post−tx
1−6 mths post−tx

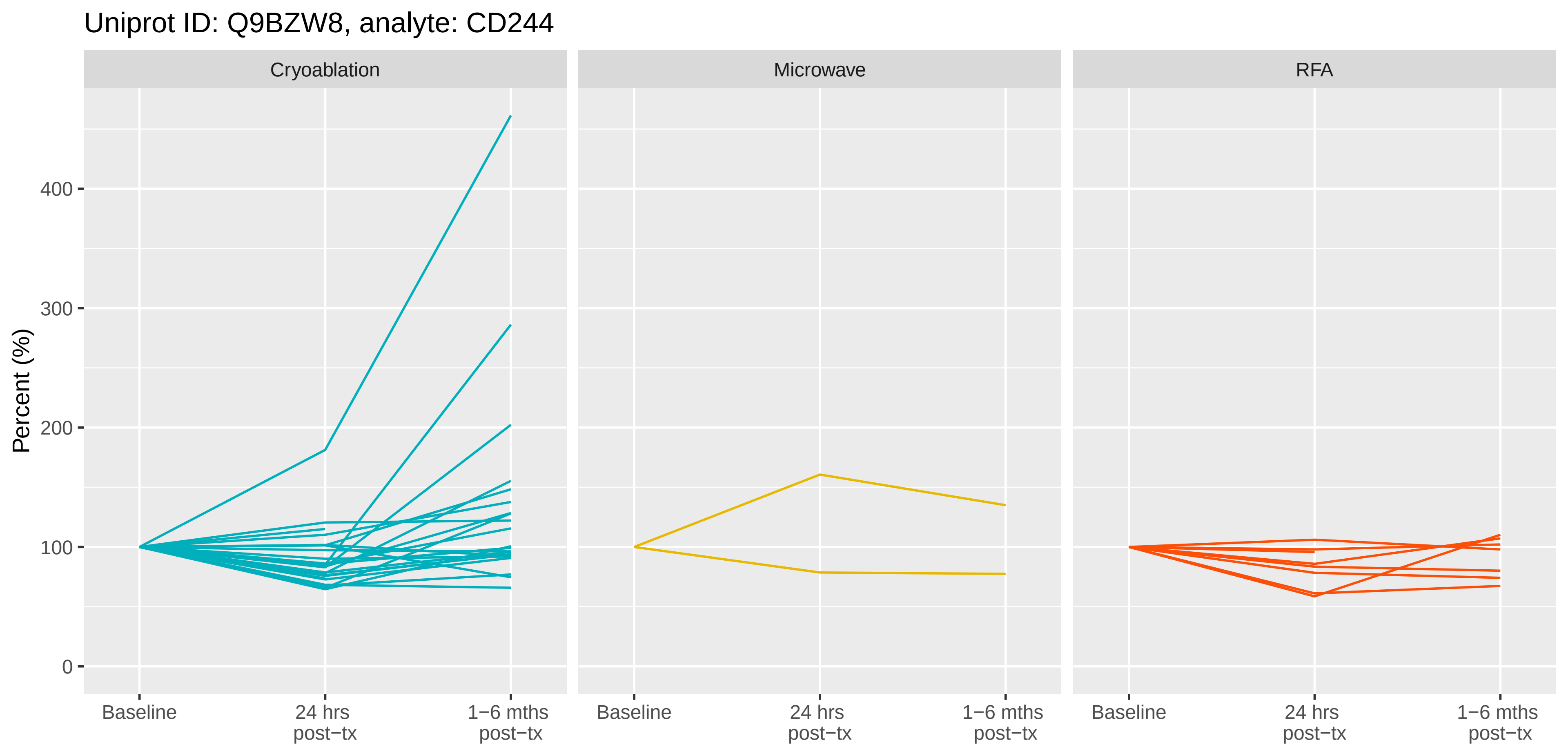

Uniprot ID: Q9BZW8, analyte: CD244
Cryoablation
Microwave
RFA
Percent (%)
0
100
200
300
400
Baseline
24 hrs post−tx
1−6 mths post−tx

Uniprot ID: P01133, analyte: EGF

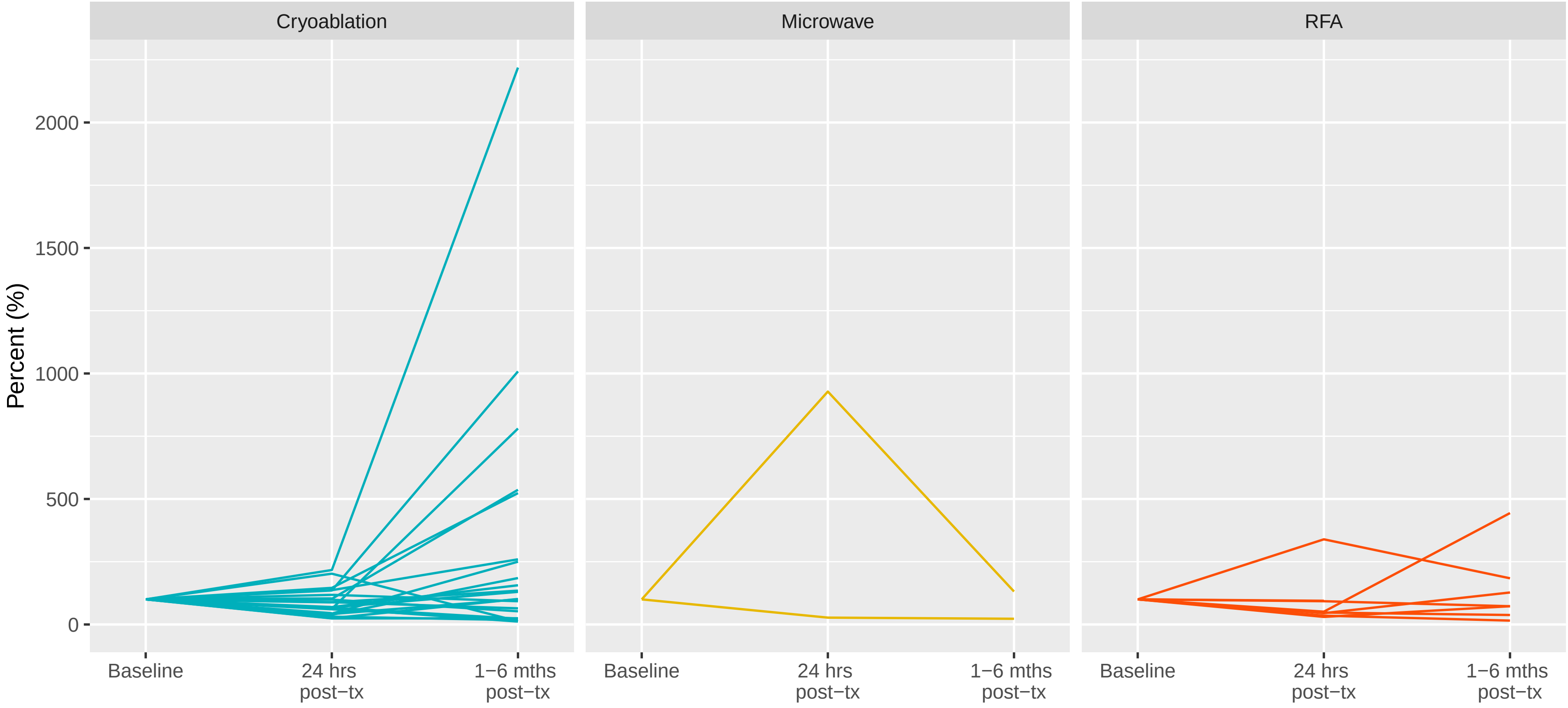

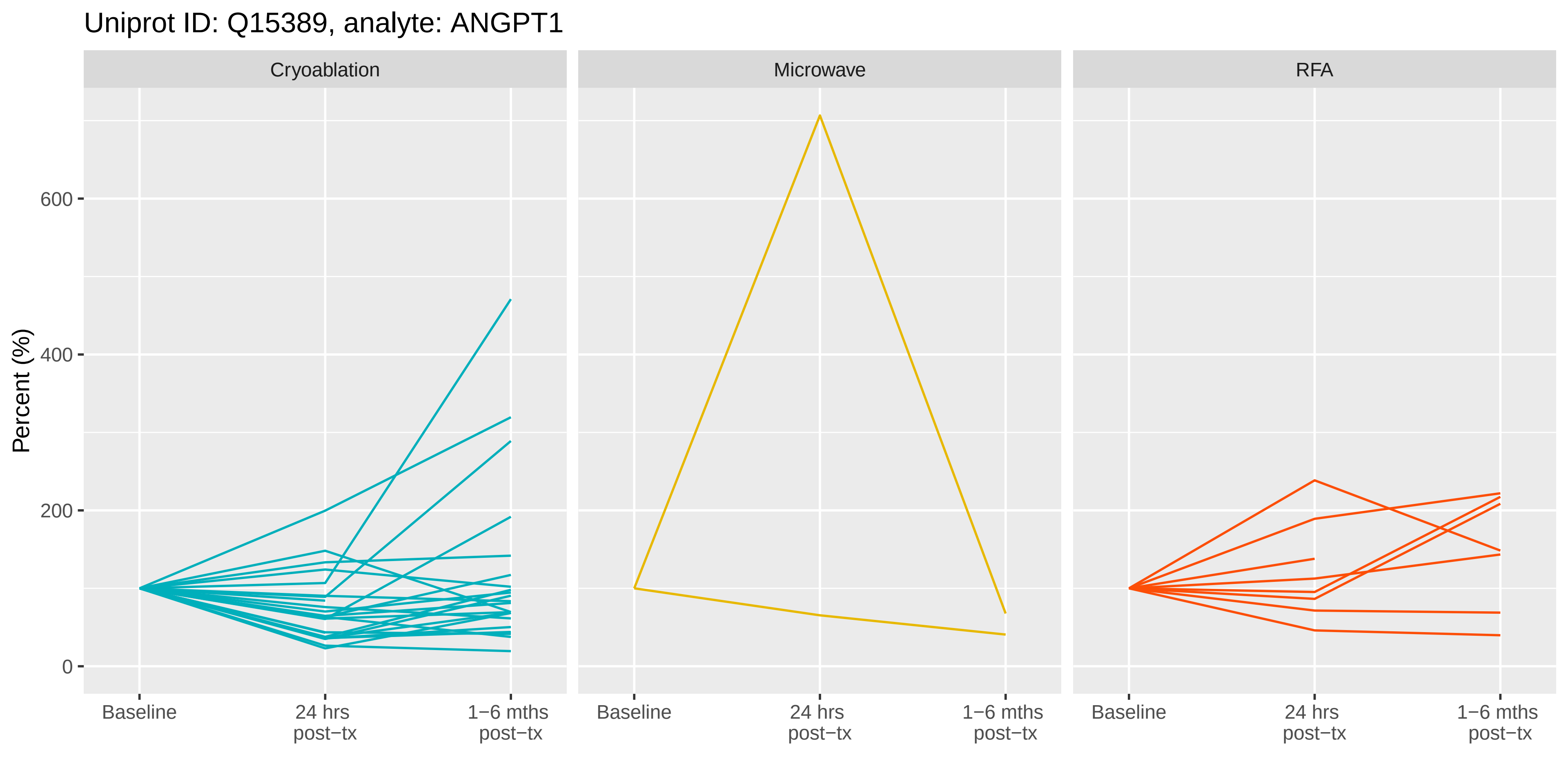
Uniprot ID: Q15389, analyte: ANGPT1
Cryoablation
Microwave
RFA
Percent (%)
600
400
200
0
Baseline
24 hrs post−tx
1−6 mths post−tx
Baseline
24 hrs post−tx
1−6 mths post−tx
Baseline
24 hrs post−tx
1−6 mths post−tx

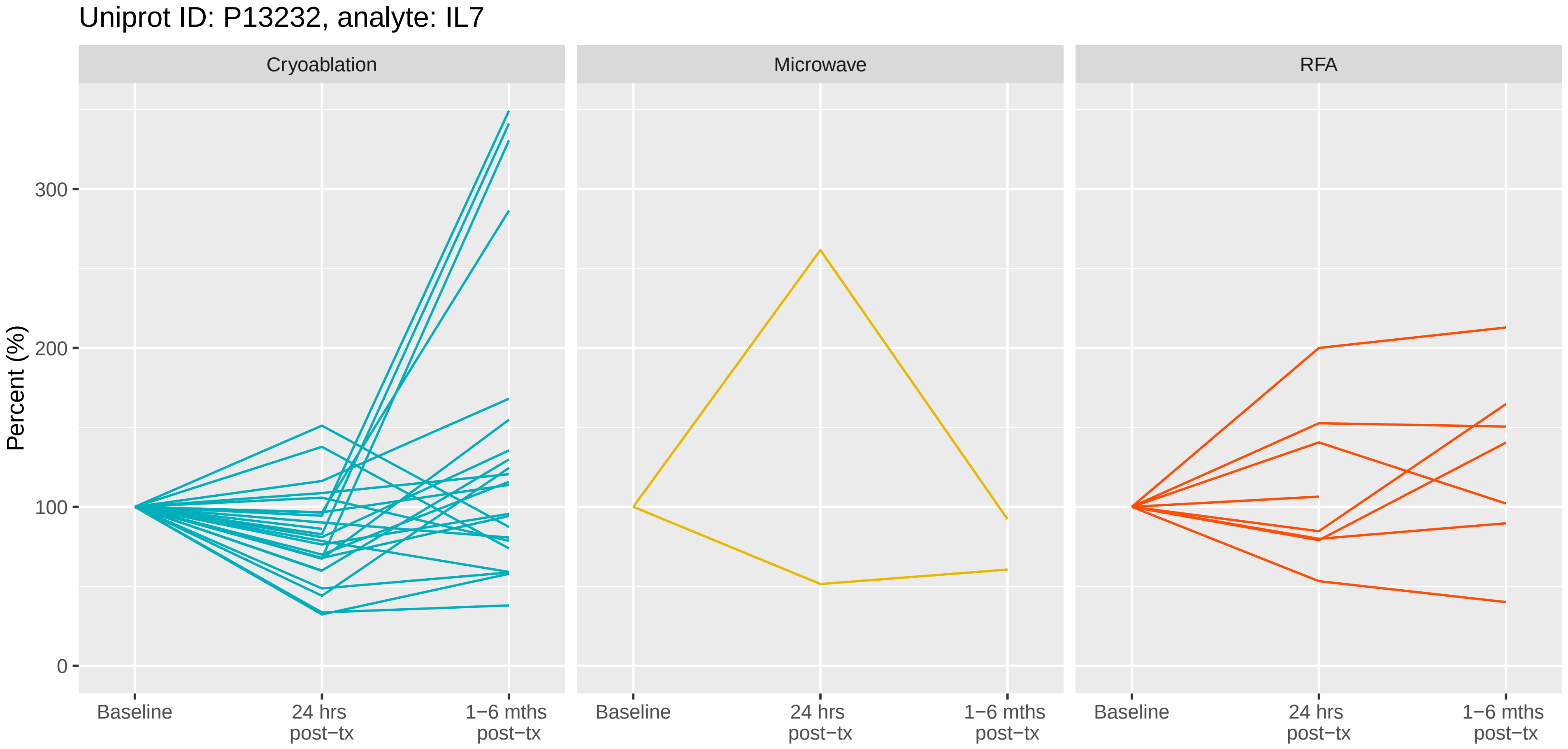

Uniprot ID: P13232, analyte: IL7
Cryoablation
Microwave
RFA
Percent (%)
300
200
100
0
Baseline
24 hrs post−tx
1−6 mths post−tx

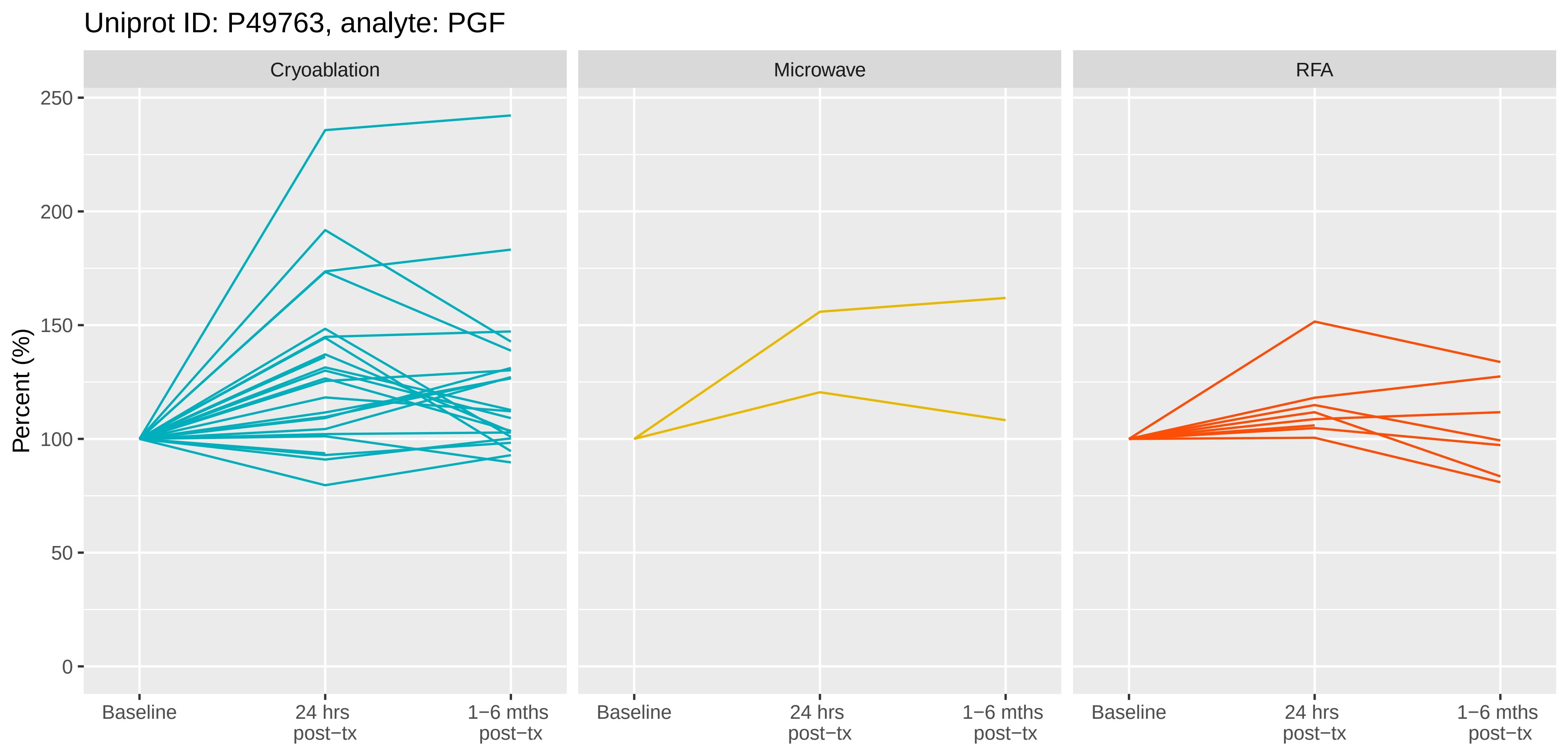

Uniprot ID: P49763, analyte: PGF
Cryoablation
Microwave
RFA
Percent (%)
250
200
150
100
50
0
Baseline
24 hrs post−tx
1−6 mths post−tx

Uniprot ID: P05231, analyte: IL6

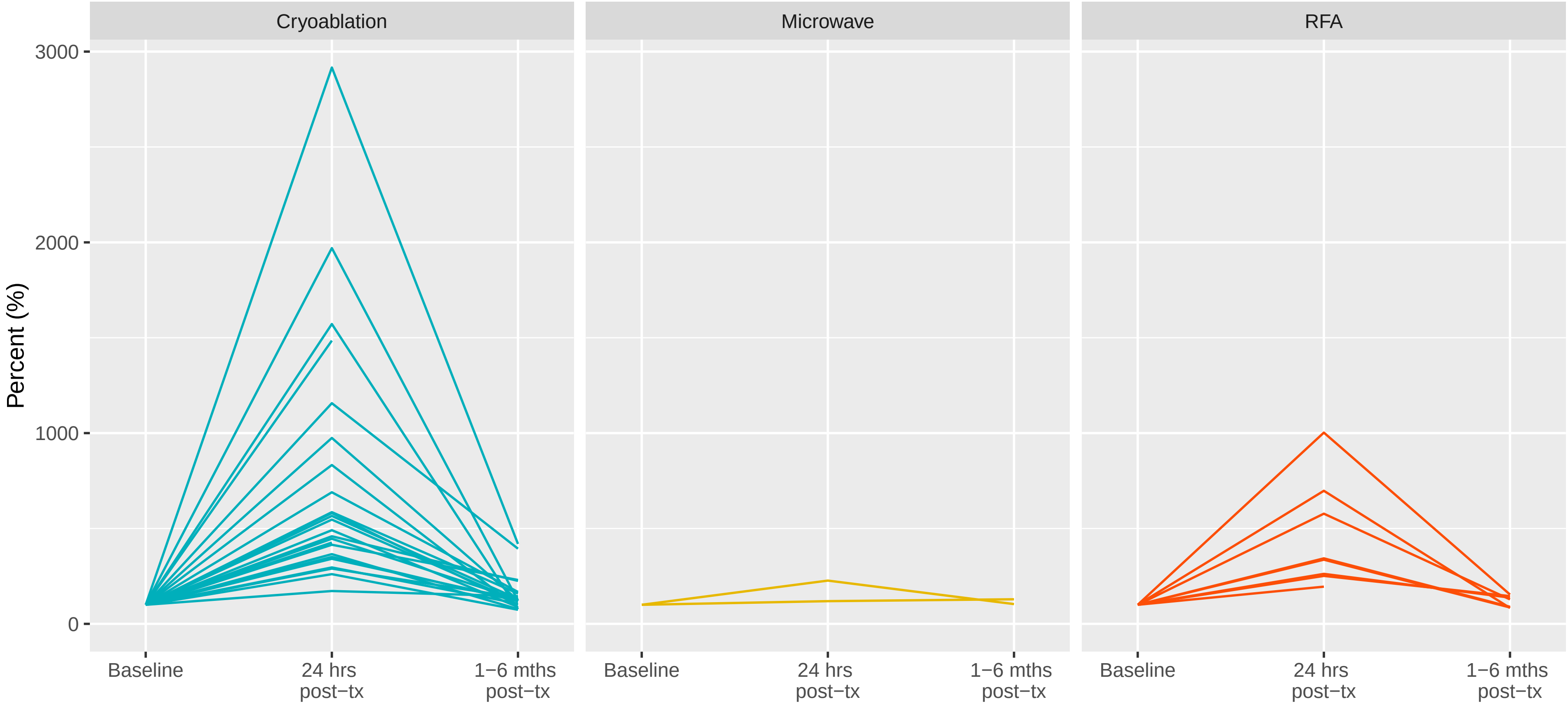

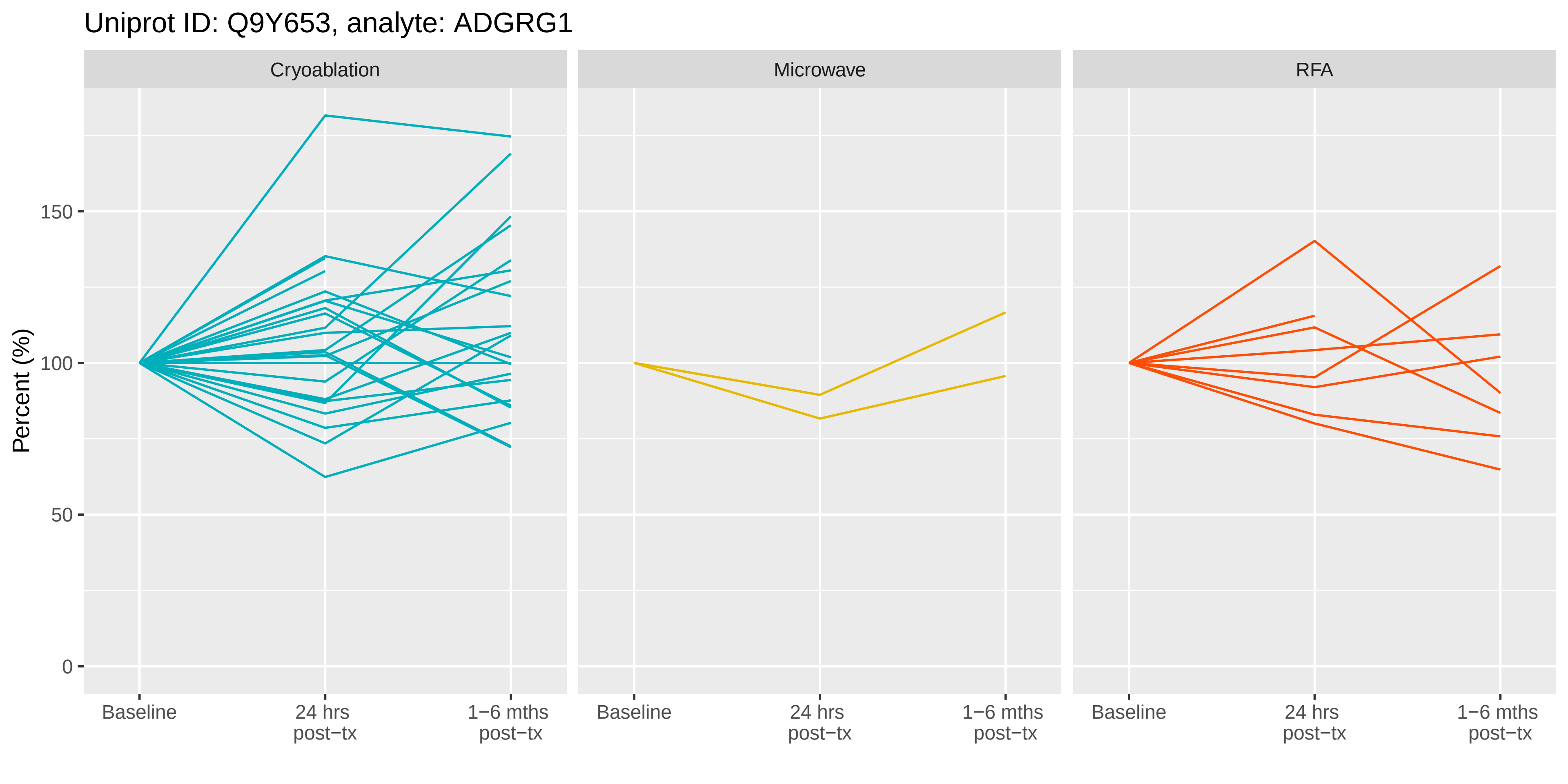
Uniprot ID: Q9Y653, analyte: ADGRG1
Cryoablation
Microwave
RFA
Percent (%)
150
100
50
0
Baseline
24 hrs post−tx
1−6 mths post−tx

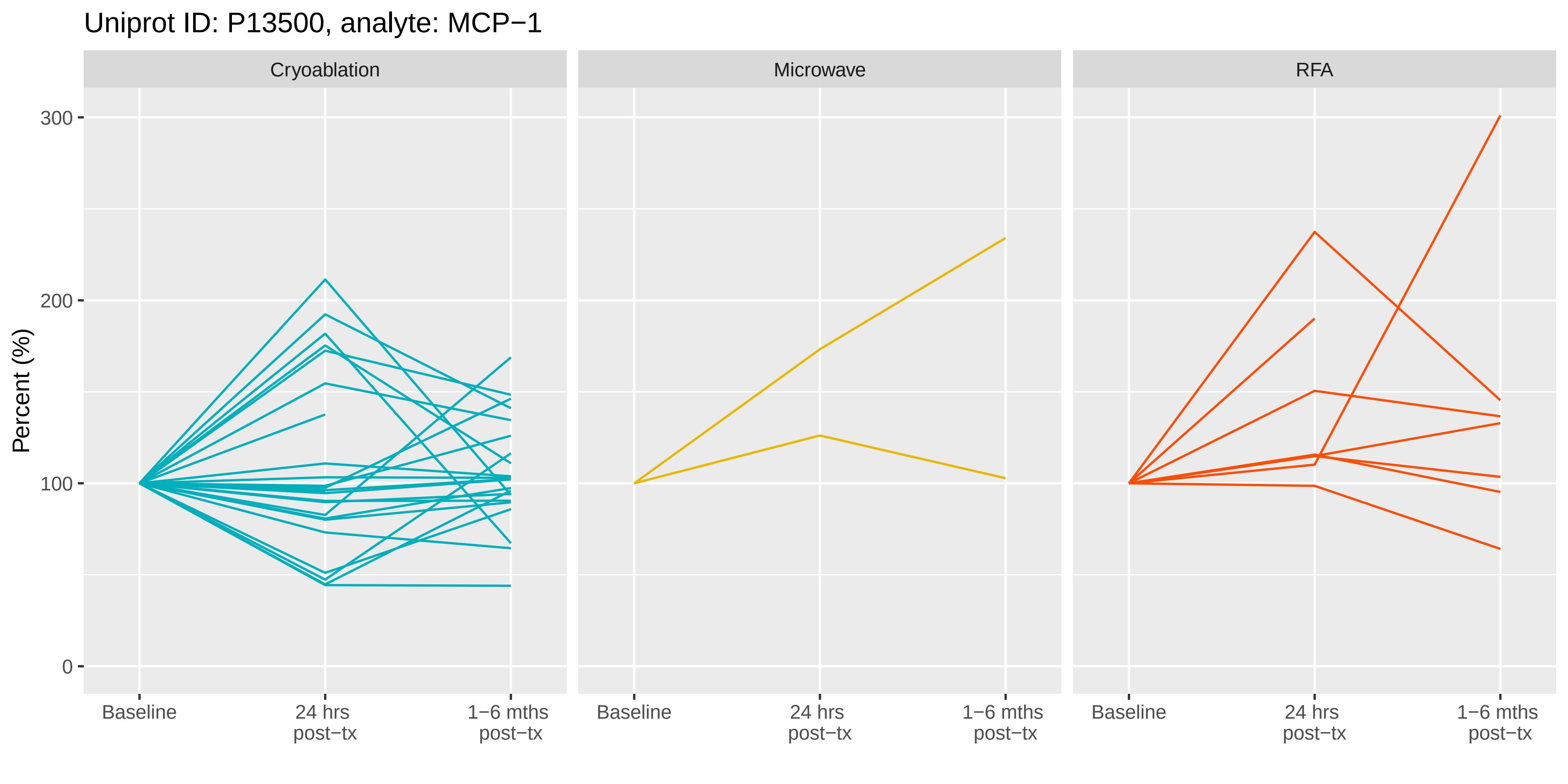

Uniprot ID: P13500, analyte: MCP−1
Cryoablation
Microwave
RFA
Percent (%)
300
200
100
0
Baseline
24 hrs post−tx
1−6 mths post−tx

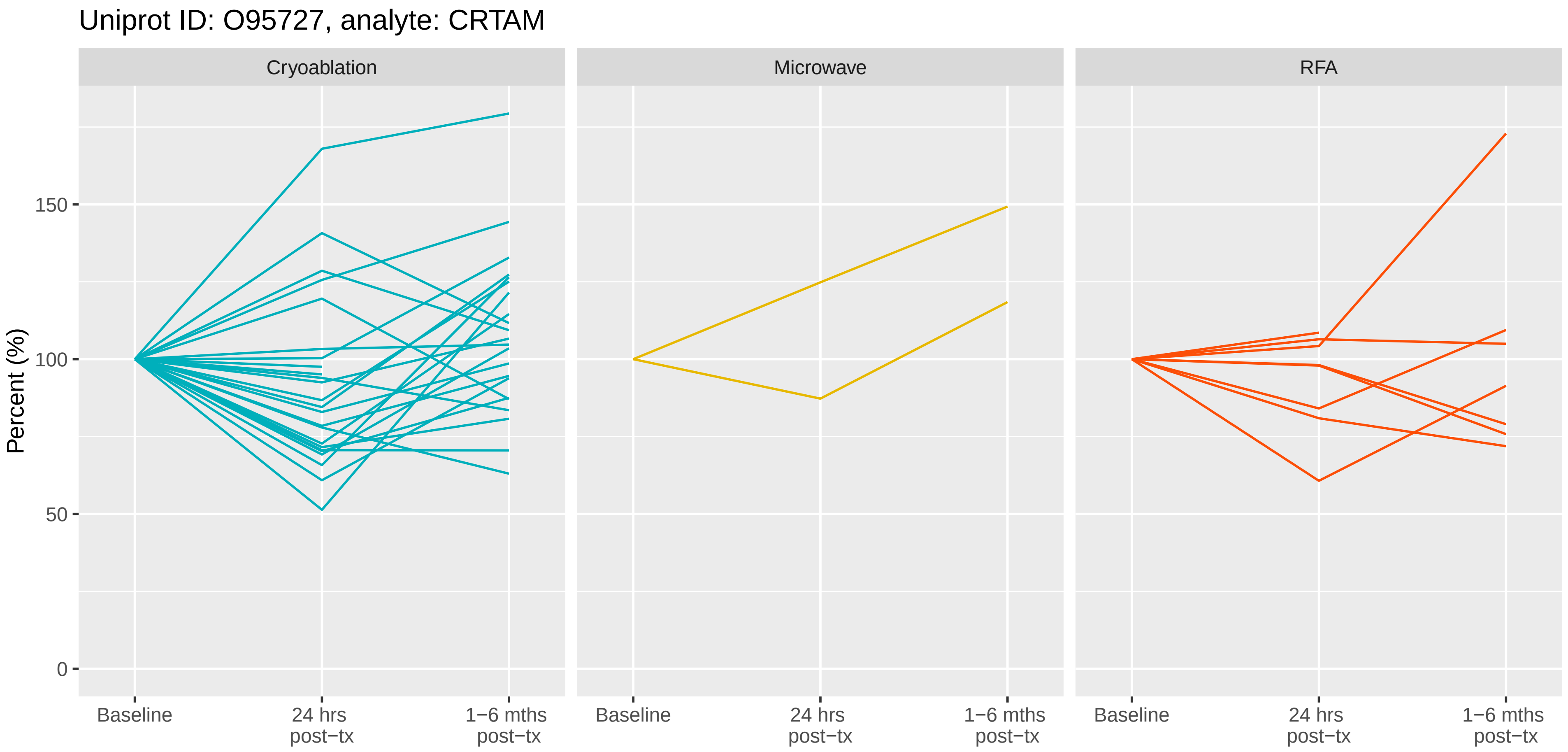

Uniprot ID: O95727, analyte: CRTAM
Cryoablation
Microwave
RFA
Percent (%)
150
100
50
0
Baseline
24 hrs post−tx
1−6 mths post−tx

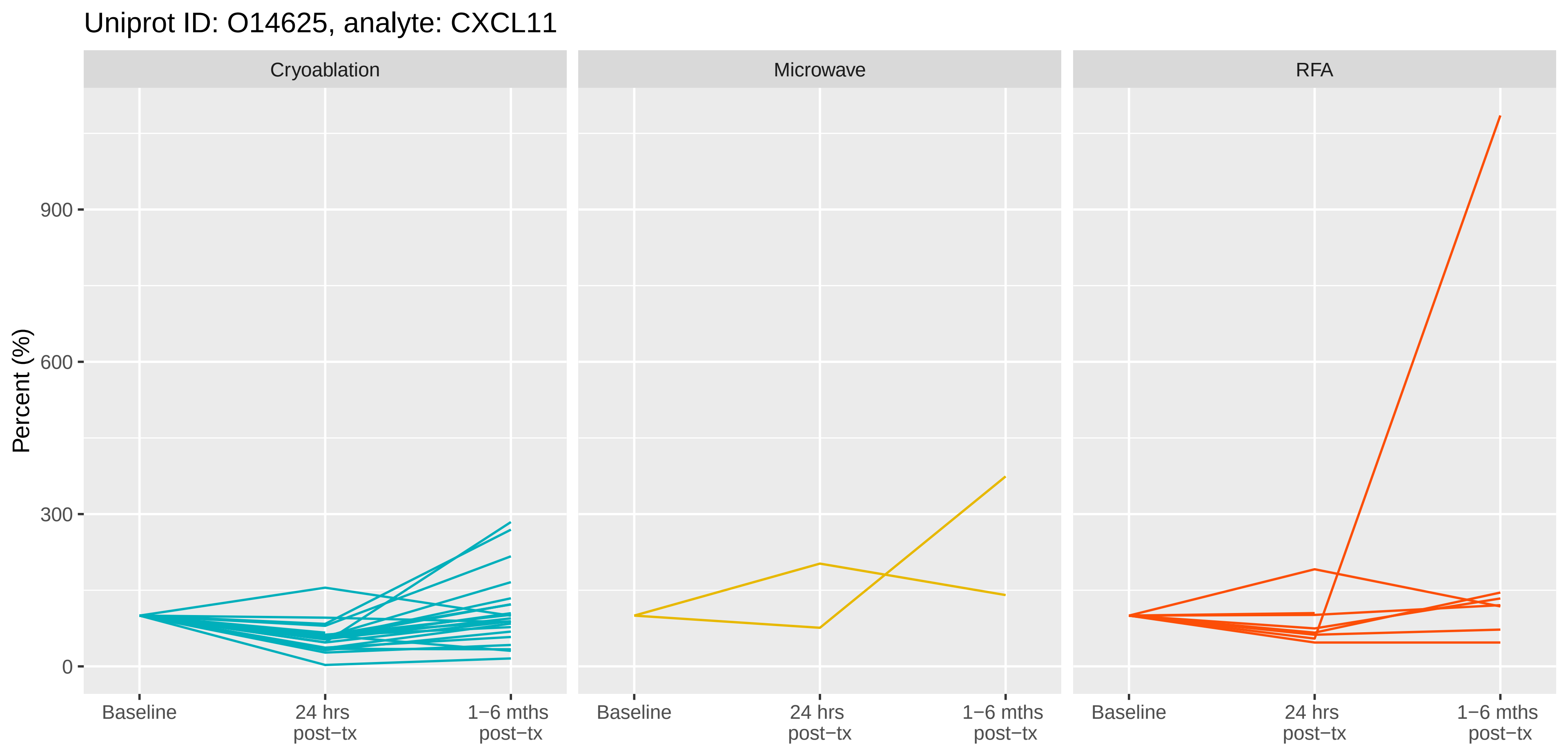
Uniprot ID: O14625, analyte: CXCL11
Cryoablation
Microwave
RFA
Percent (%)
900
600
300
0
Baseline
24 hrs post−tx
1−6 mths post−tx
Baseline
24 hrs post−tx
1−6 mths post−tx
Baseline
24 hrs post−tx
1−6 mths post−tx

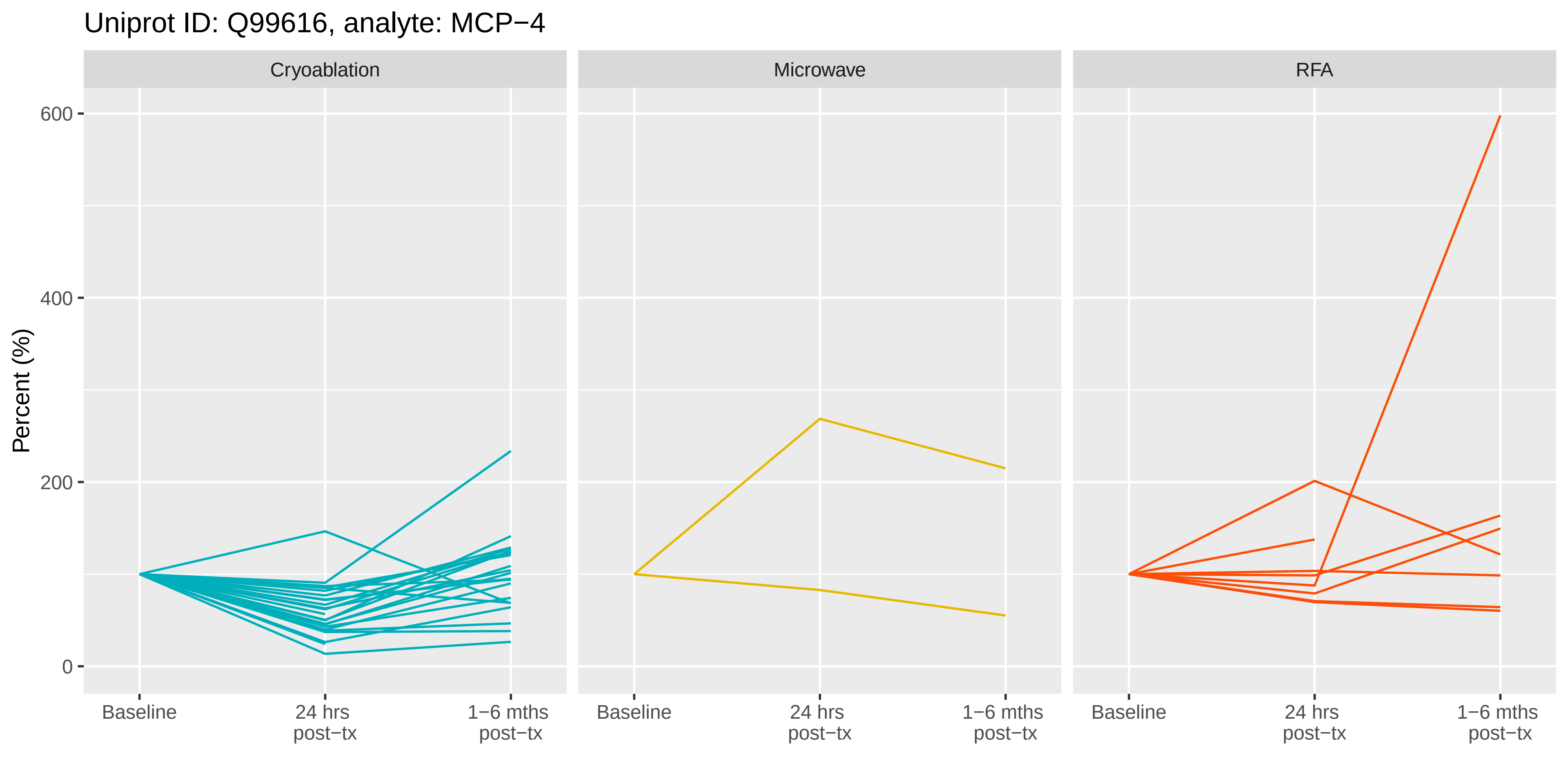
Uniprot ID: Q99616, analyte: MCP−4
Cryoablation
Microwave
RFA
Percent (%)
600
400
200
0
Baseline
24 hrs post−tx
1−6 mths post−tx

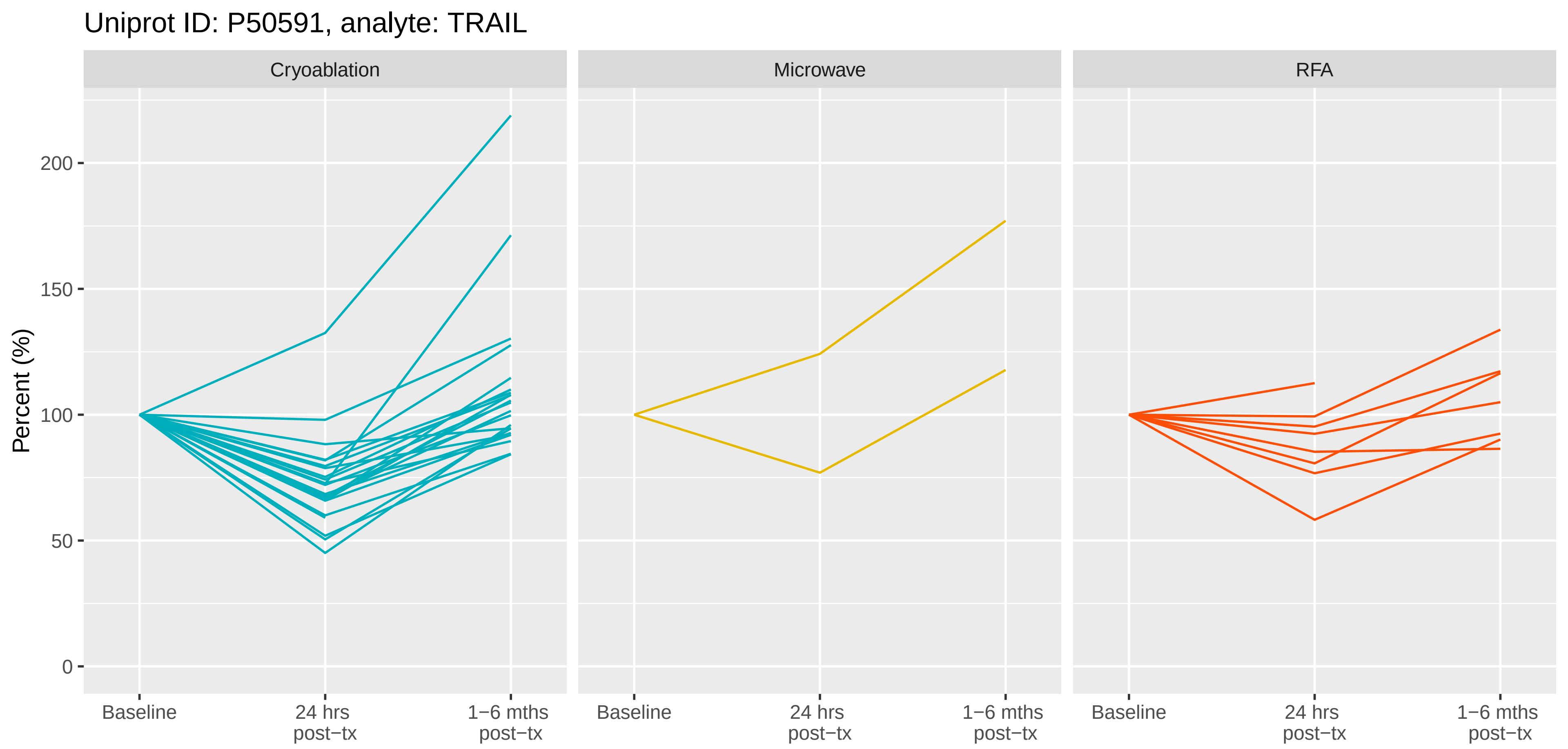

Uniprot ID: P50591, analyte: TRAIL
Cryoablation
Microwave
RFA
Percent (%)
200
150
100
50
0
Baseline
24 hrs post−tx
1−6 mths post−tx

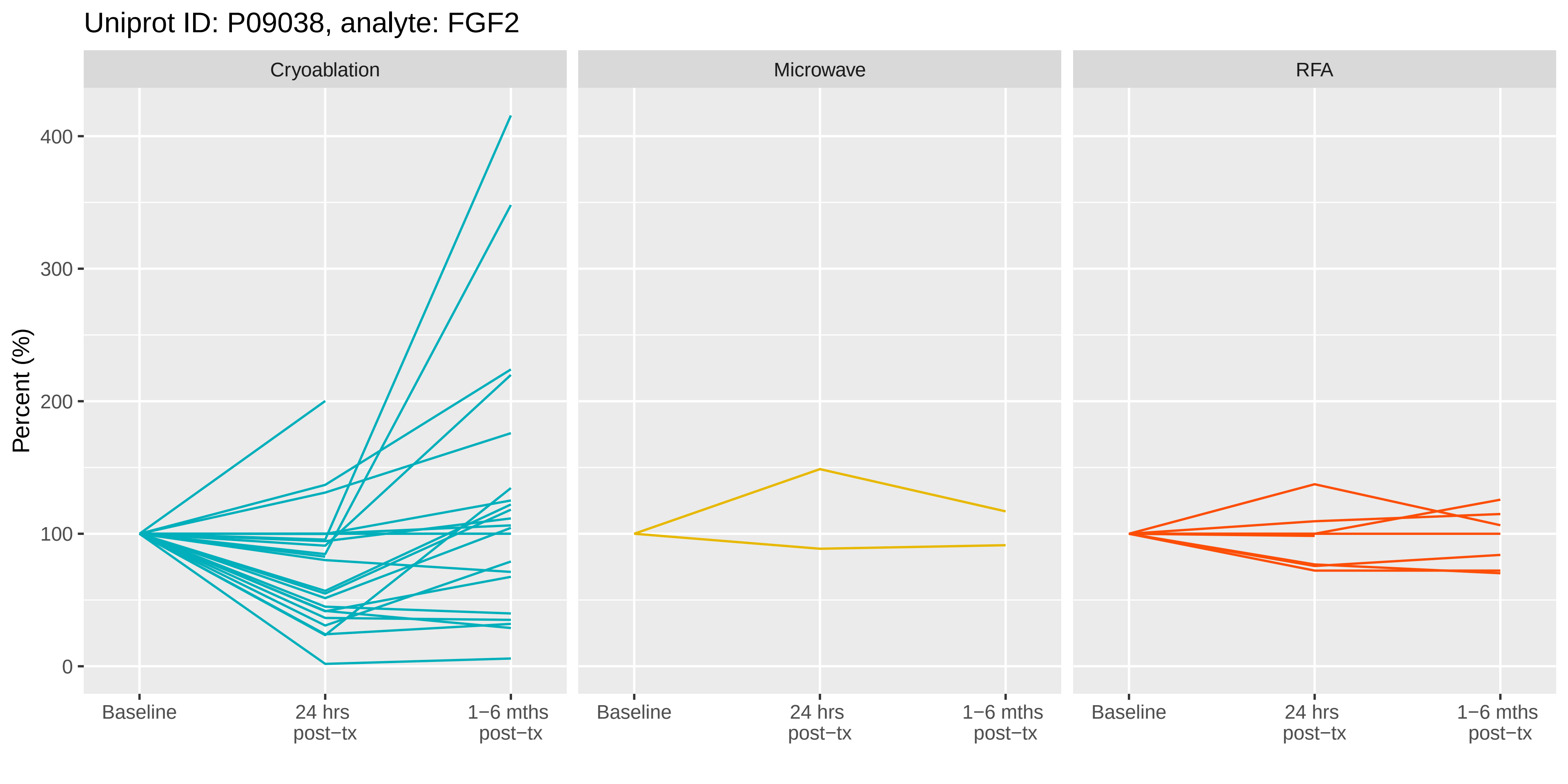
Uniprot ID: P09038, analyte: FGF2
Cryoablation
Microwave
RFA
Percent (%)
400
300
200
100
0
Baseline
24 hrs post−tx
1−6 mths post−tx
Baseline
24 hrs post−tx
1−6 mths post−tx
Baseline
24 hrs post−tx
1−6 mths post−tx

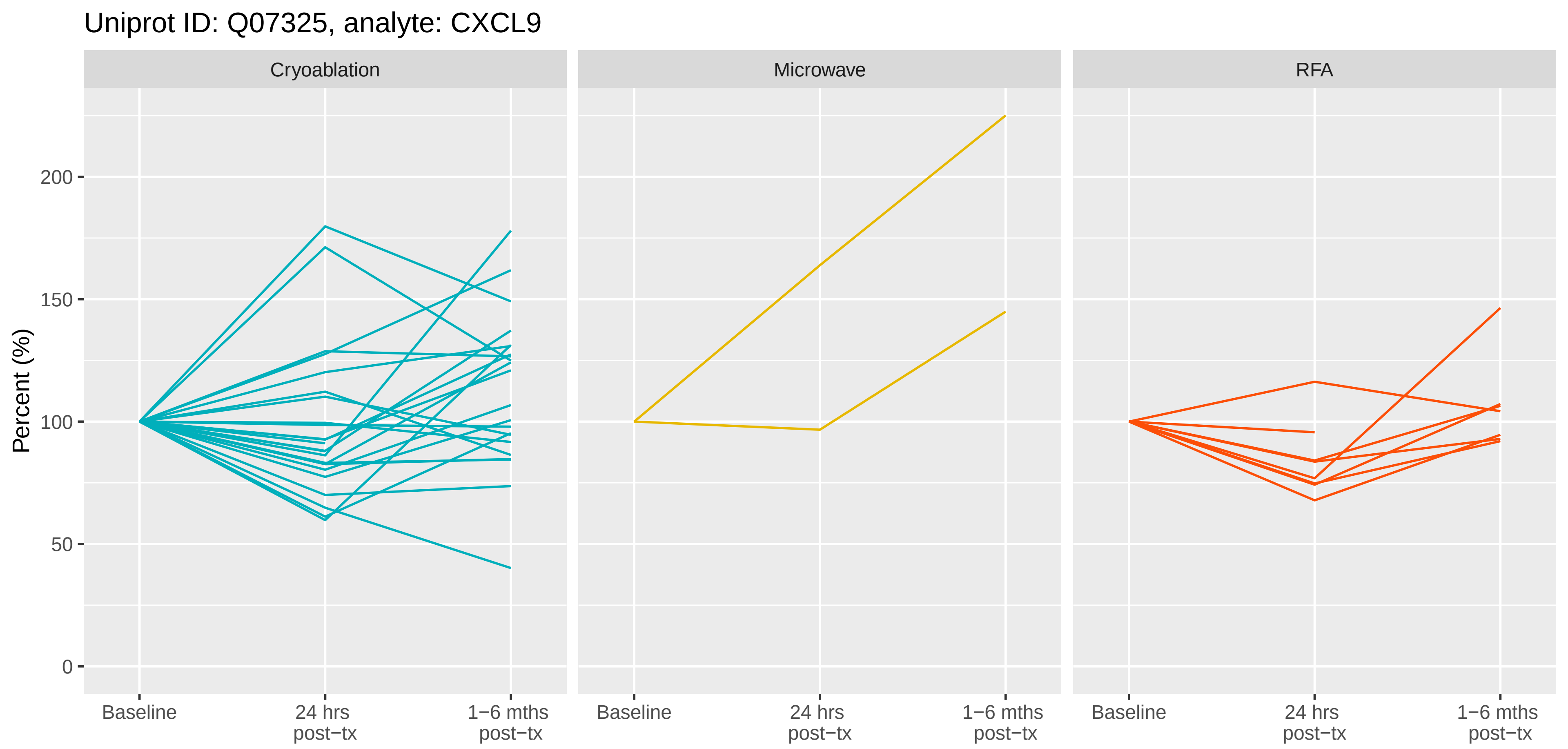
Uniprot ID: Q07325, analyte: CXCL9
Cryoablation
Microwave
RFA
Percent (%)
0
50
100
150
200
Baseline
24 hrs post−tx
1−6 mths post−tx

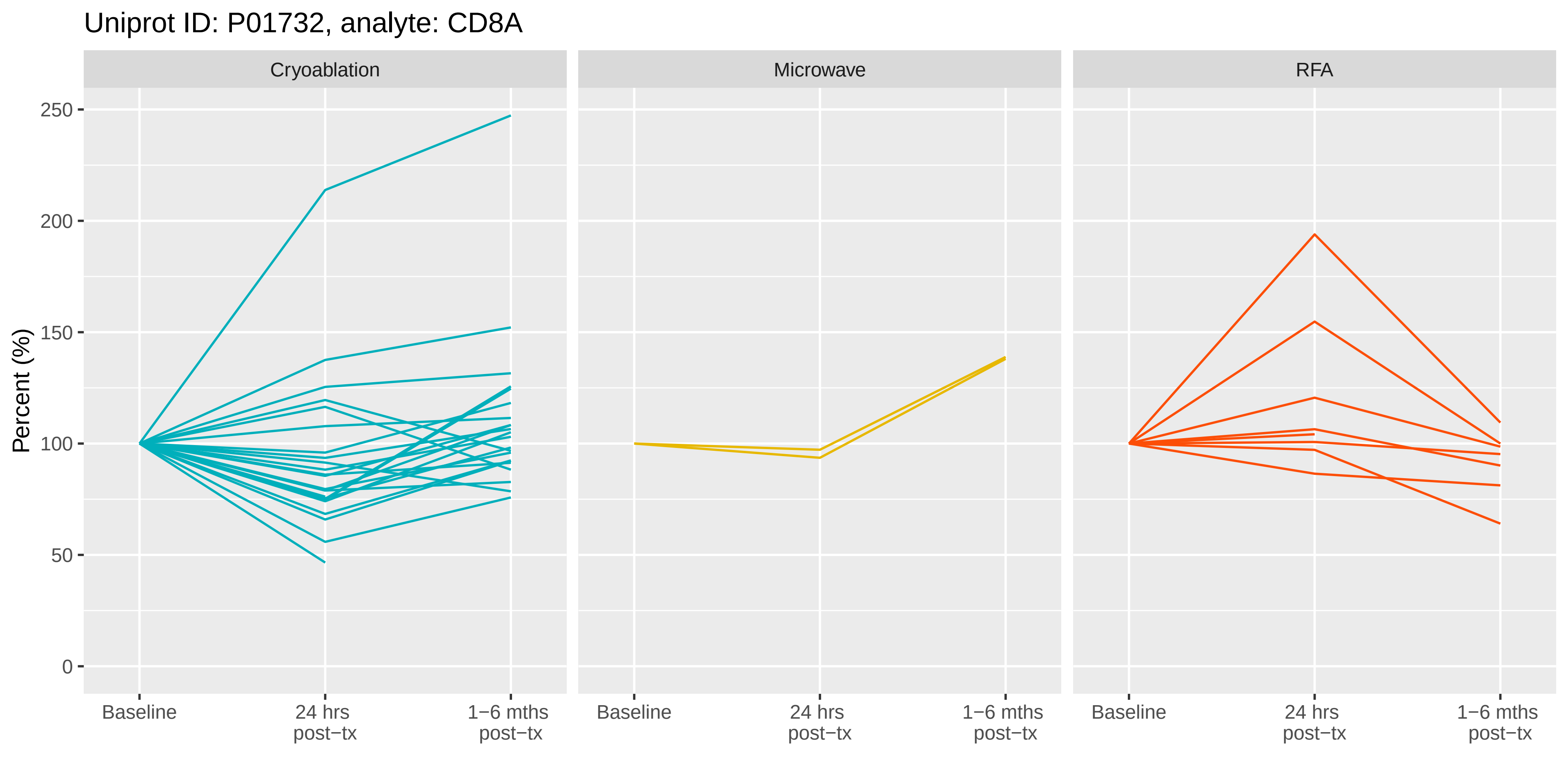

Uniprot ID: P01732, analyte: CD8A
Cryoablation
Microwave
RFA
Percent (%)
250
200
150
100
50
0
Baseline
24 hrs post−tx
1−6 mths post−tx

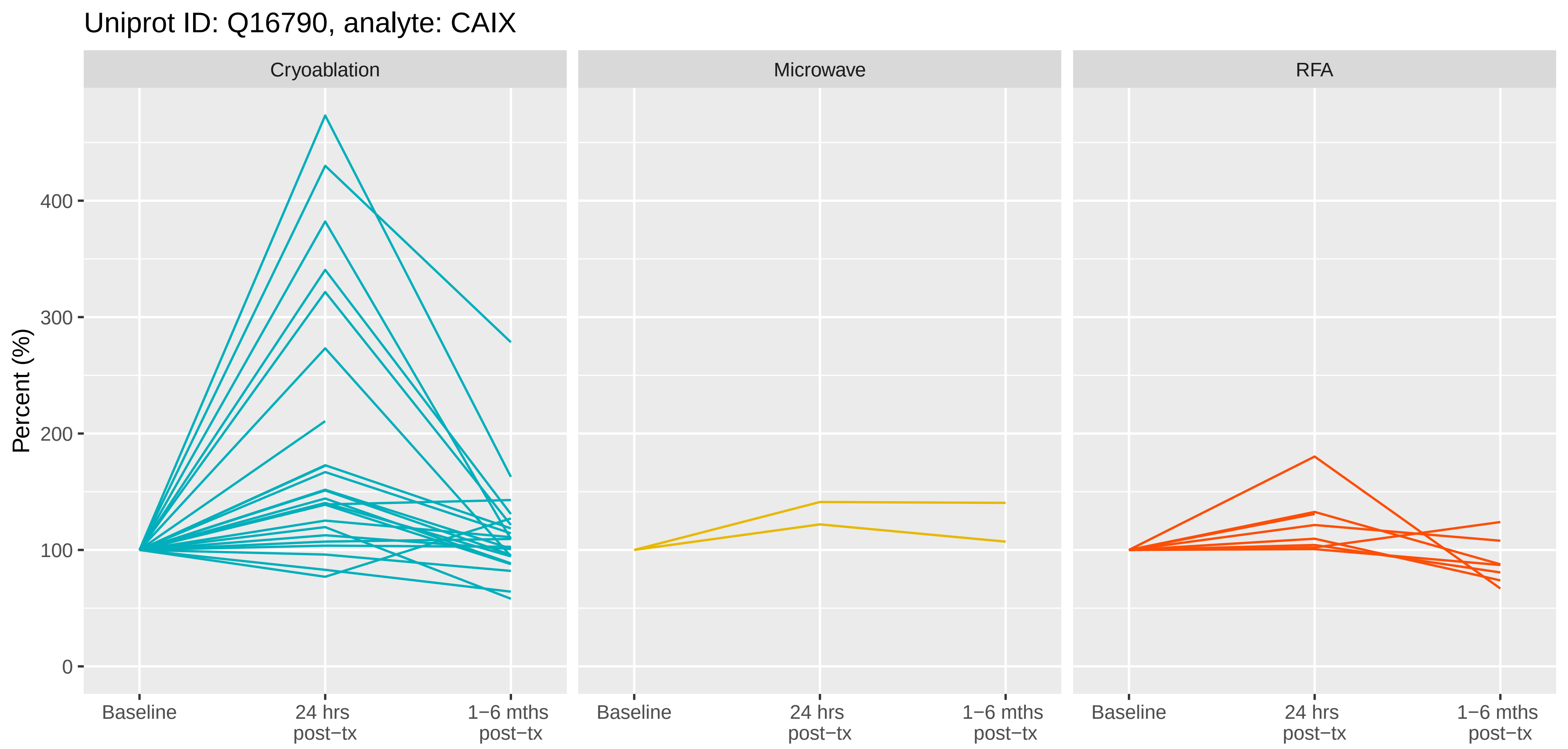

Uniprot ID: Q16790, analyte: CAIX
Cryoablation
Microwave
RFA
Percent (%)
0
100
200
300
400
Baseline
24 hrs post−tx
1−6 mths post−tx

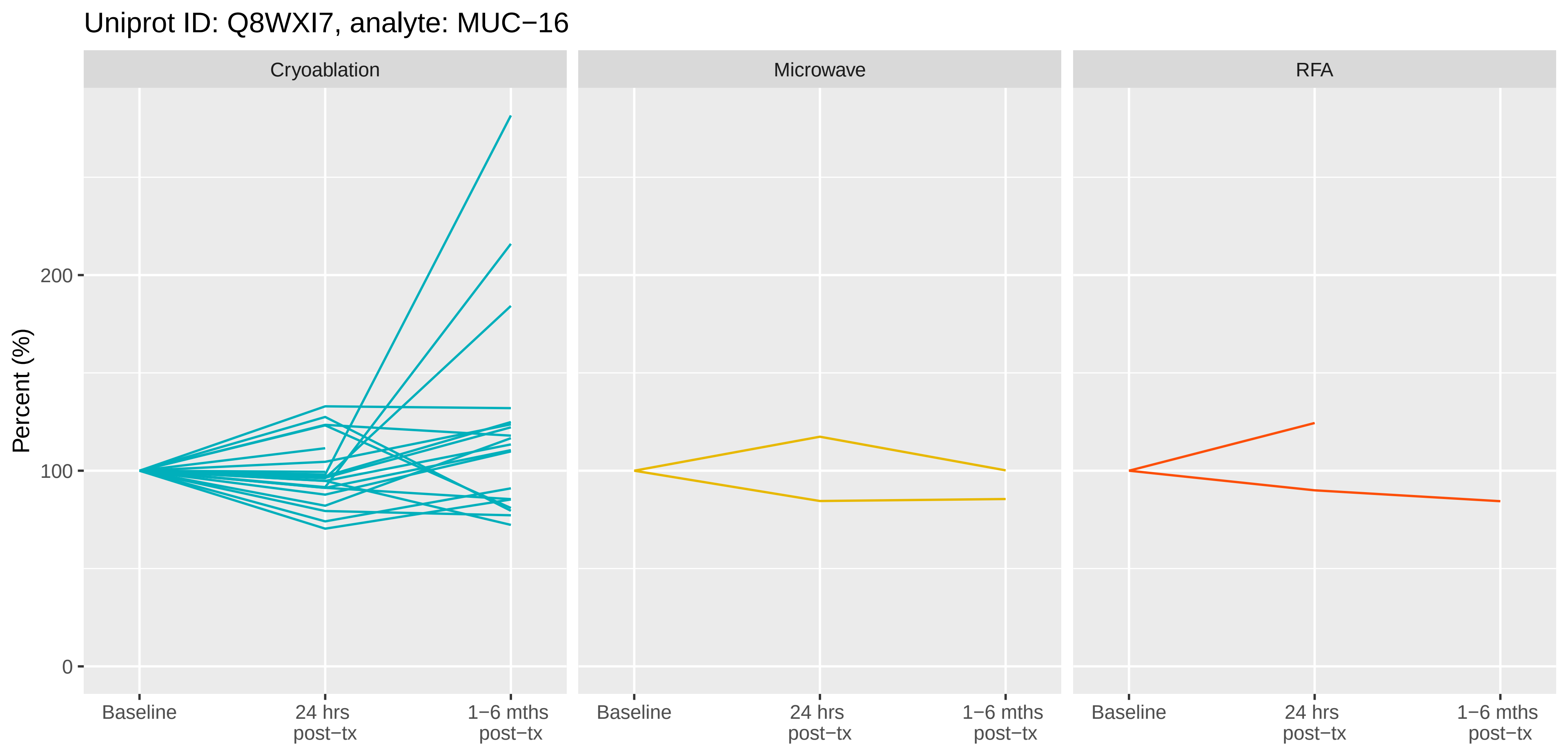

Uniprot ID: Q8WXI7, analyte: MUC−16
Cryoablation
Microwave
RFA
Percent (%)
200
100
0
Baseline
24 hrs post−tx
1−6 mths post−tx

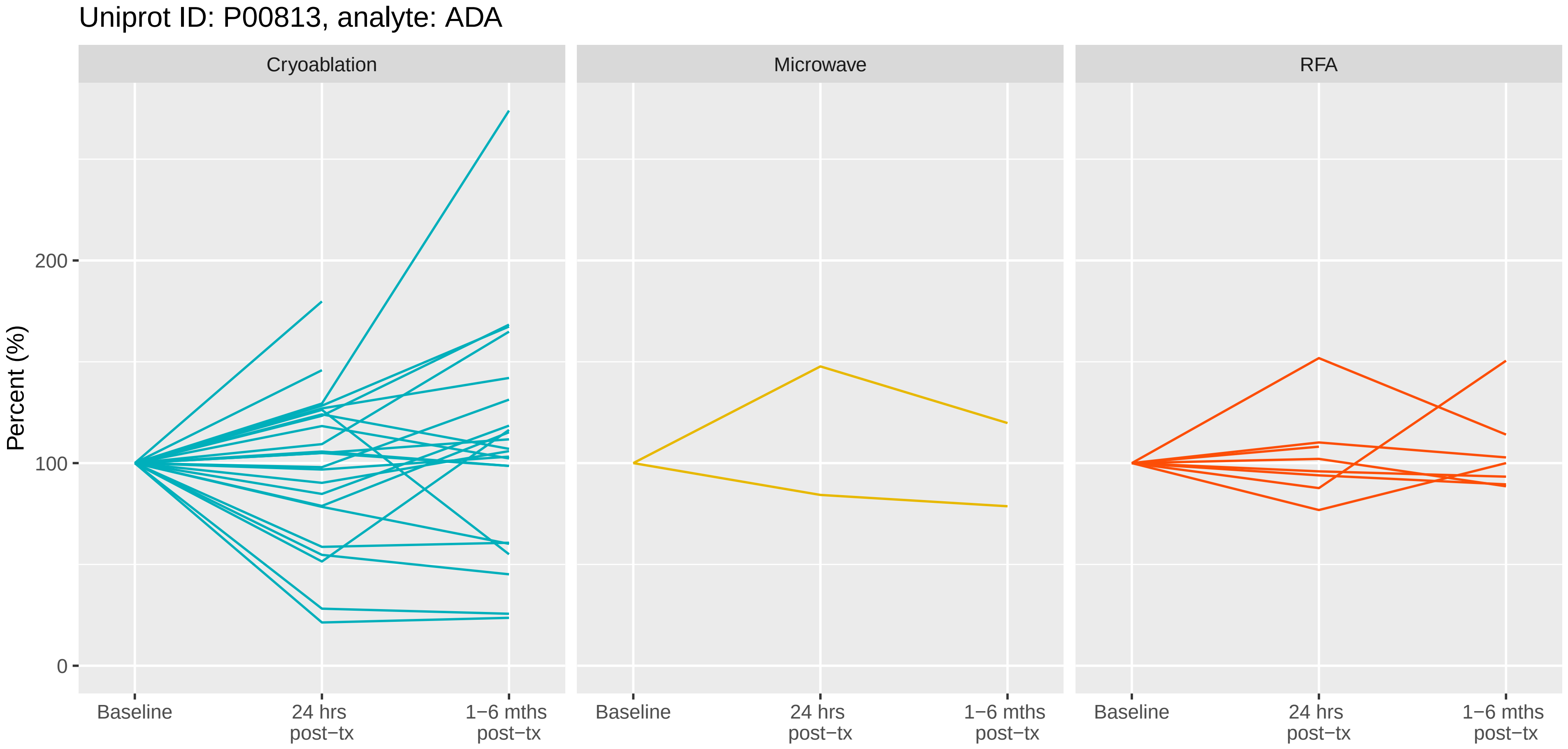

Uniprot ID: P00813, analyte: ADA
Cryoablation
Microwave
RFA
Percent (%)
200
100
0
Baseline
24 hrs post−tx
1−6 mths post−tx

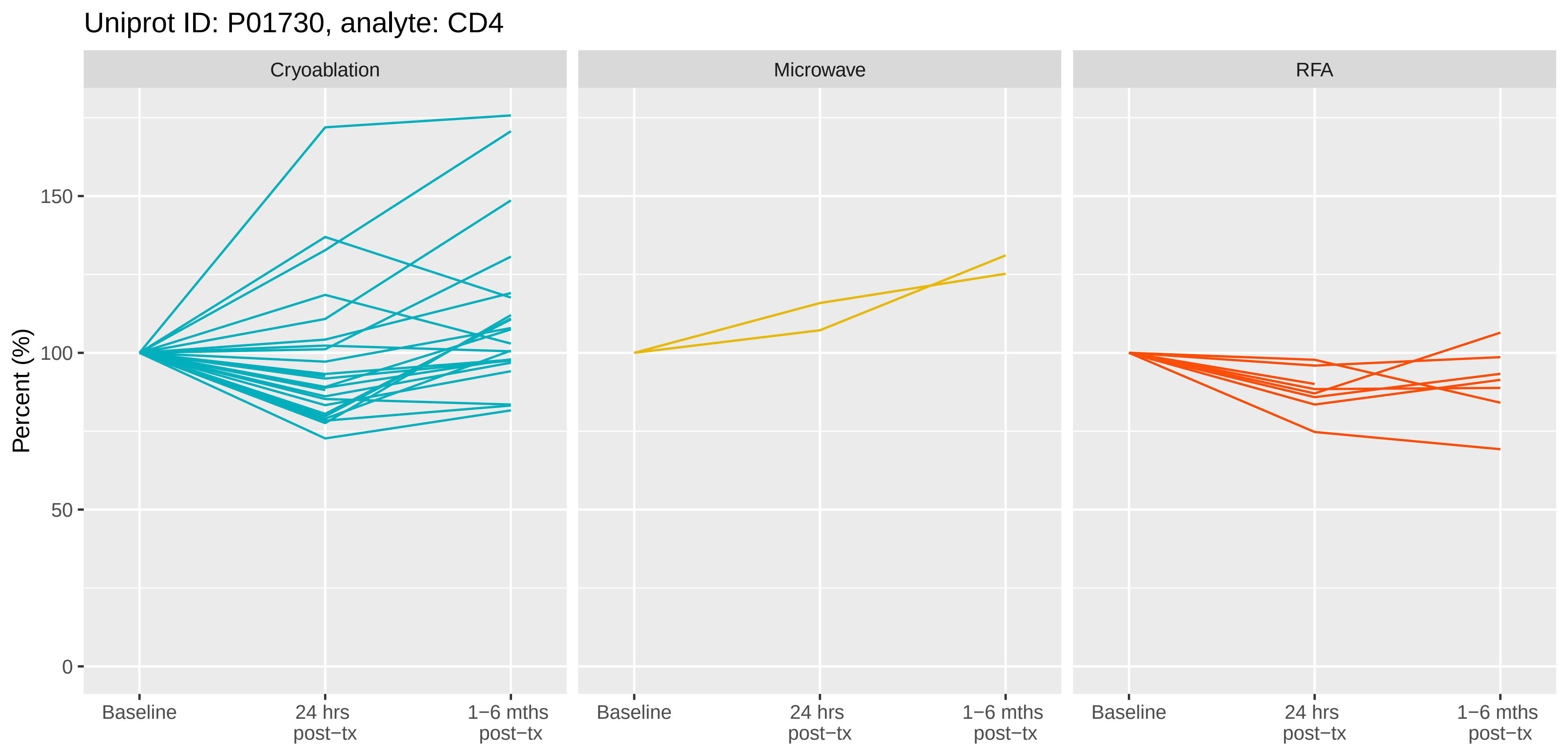

Uniprot ID: P01730, analyte: CD4
Cryoablation
Microwave
RFA
Percent (%)
150
100
50
0
Baseline
24 hrs post−tx
1−6 mths post−tx

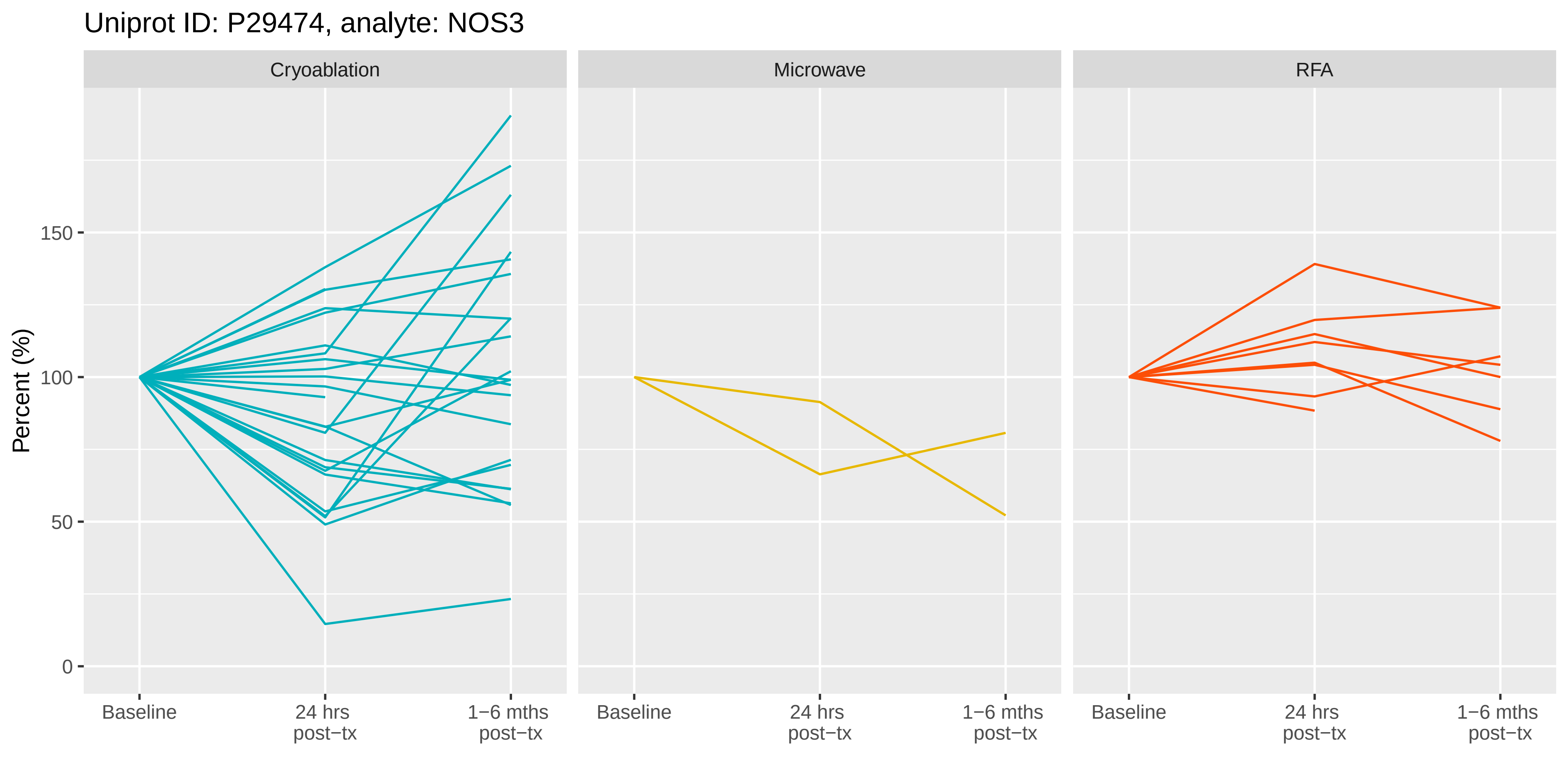
Uniprot ID: P29474, analyte: NOS3
Cryoablation
Microwave
RFA
Percent (%)
150
100
50
0
Baseline
24 hrs post−tx
1−6 mths post−tx
Baseline
24 hrs post−tx
1−6 mths post−tx
Baseline
24 hrs post−tx
1−6 mths post−tx

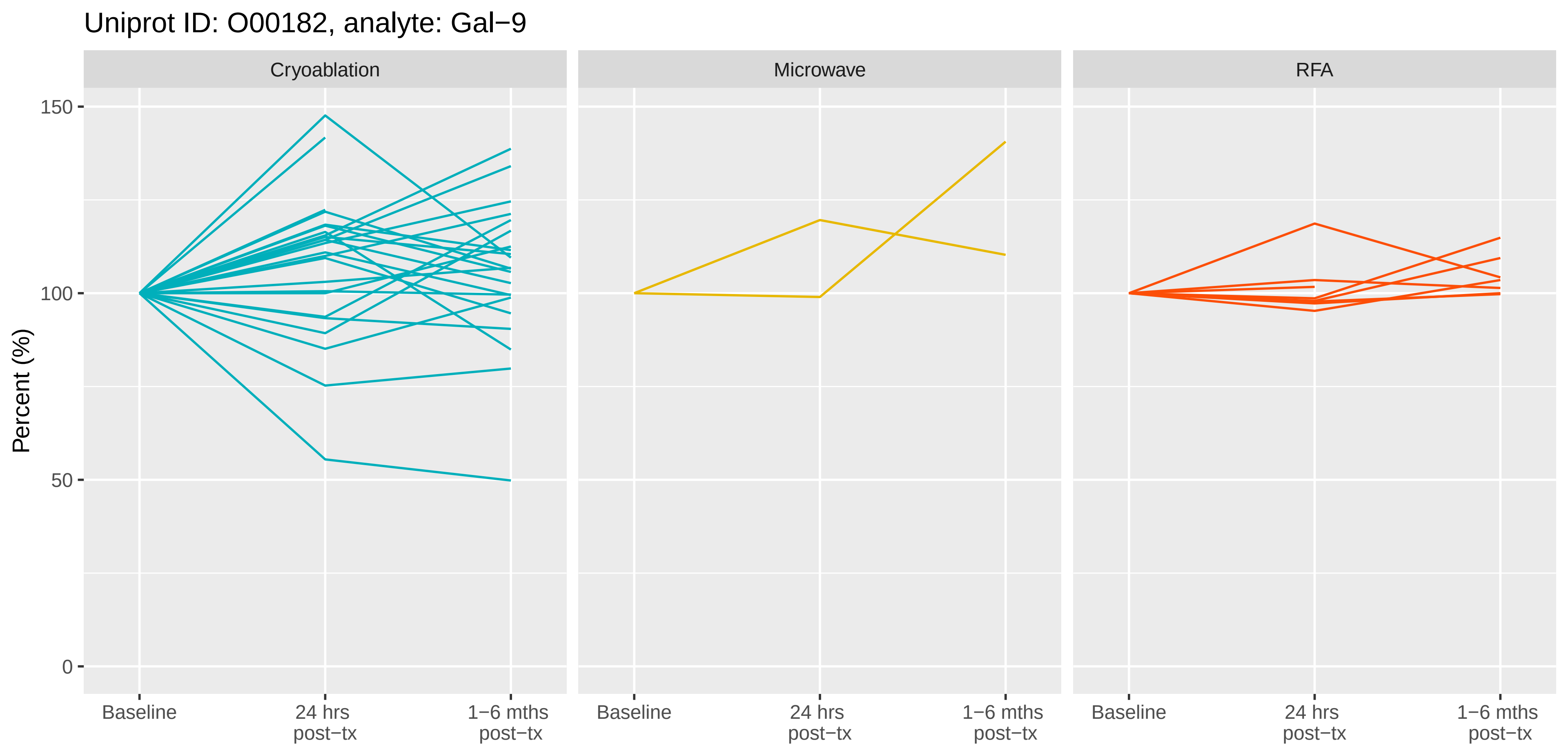

Uniprot ID: O00182, analyte: Gal−9
Cryoablation
Microwave
RFA
Percent (%)
150
100
50
0
Baseline
24 hrs post−tx
1−6 mths post−tx

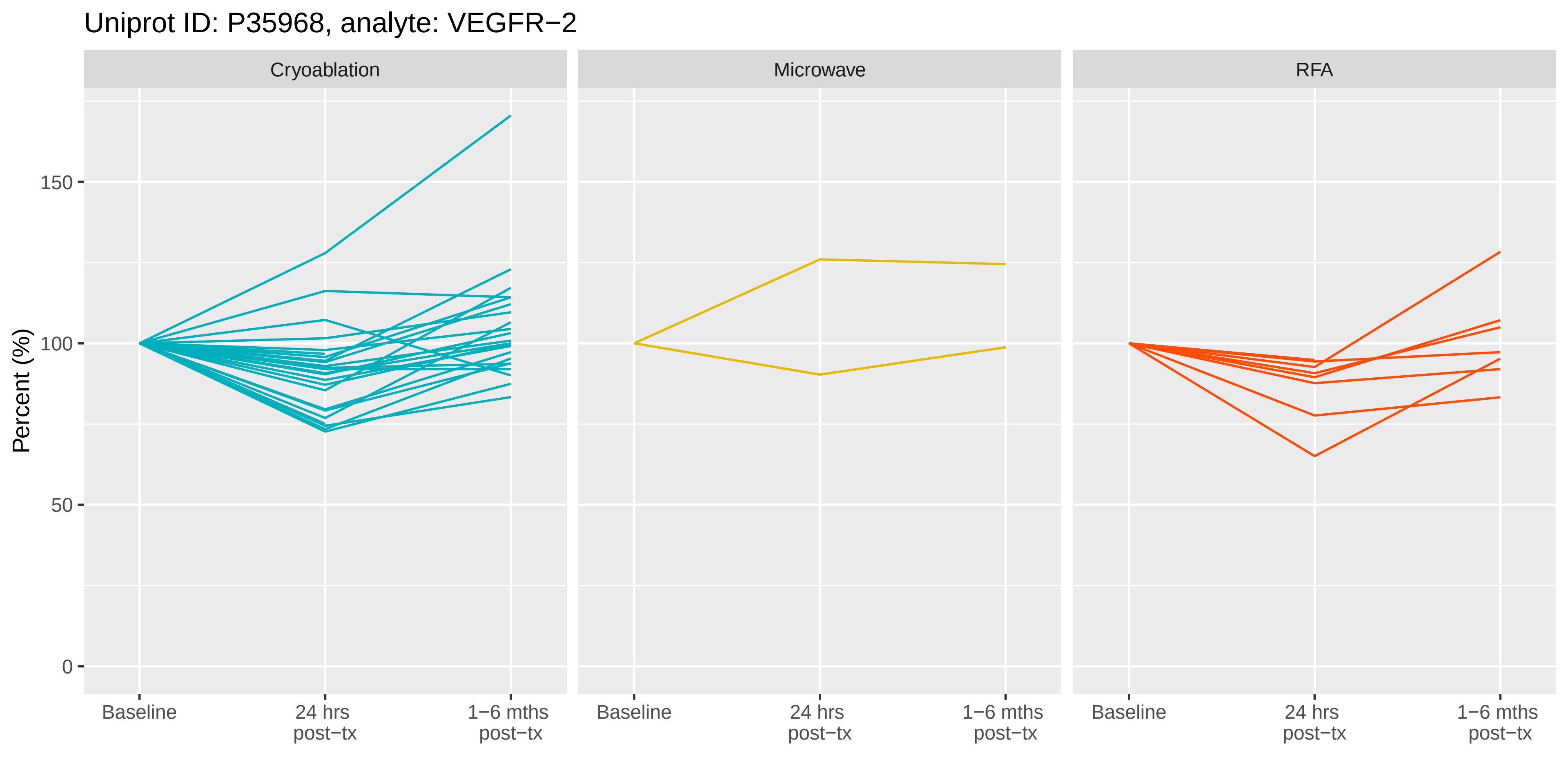

Uniprot ID: P35968, analyte: VEGFR−2
Cryoablation
Microwave
RFA
Percent (%)
150
100
50
0
Baseline
24 hrs post−tx
1−6 mths post−tx

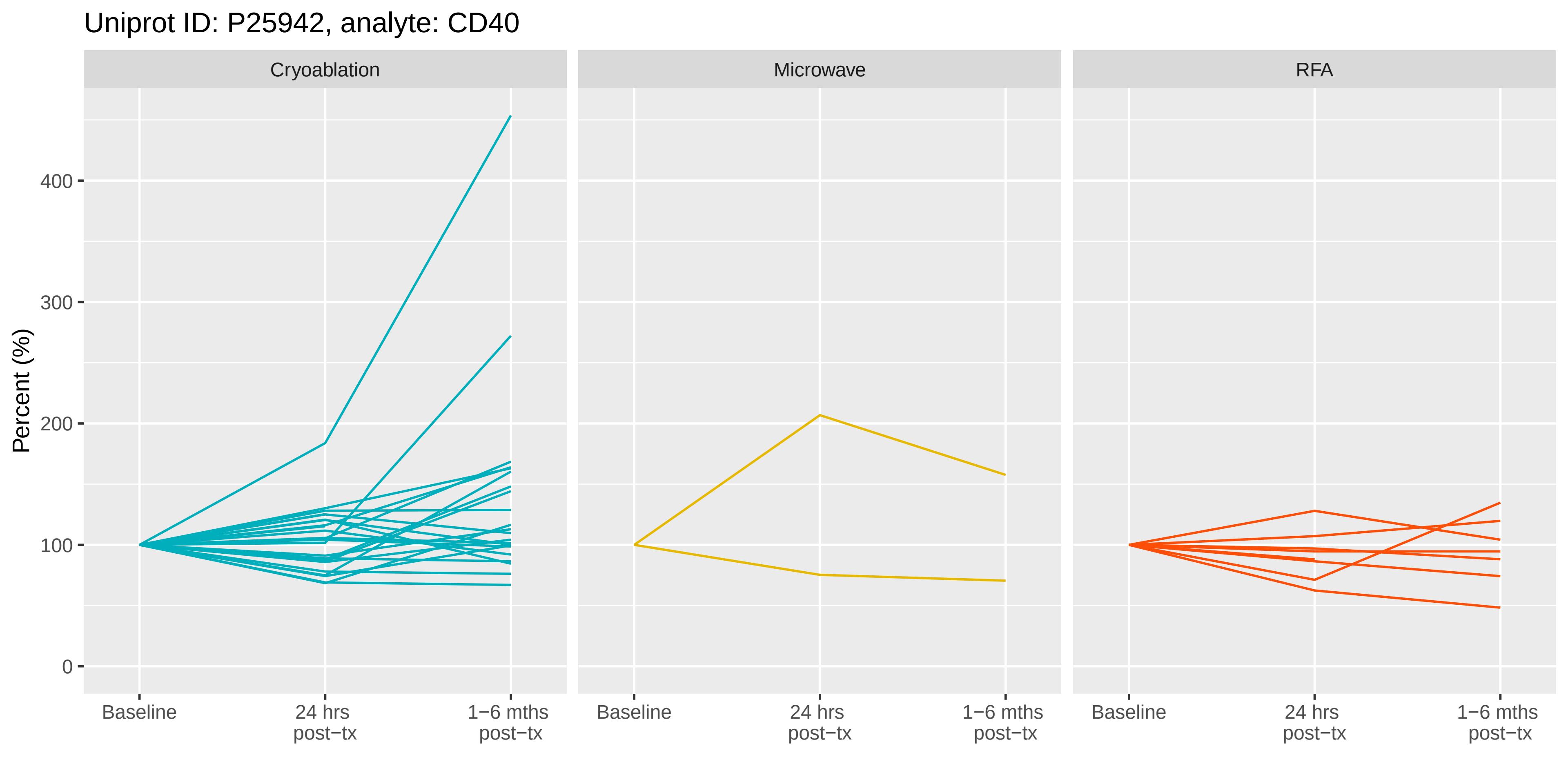

Uniprot ID: P25942, analyte: CD40
Cryoablation
Microwave
RFA
Percent (%)
400
300
200
100
0
Baseline
24 hrs post−tx
1−6 mths post−tx

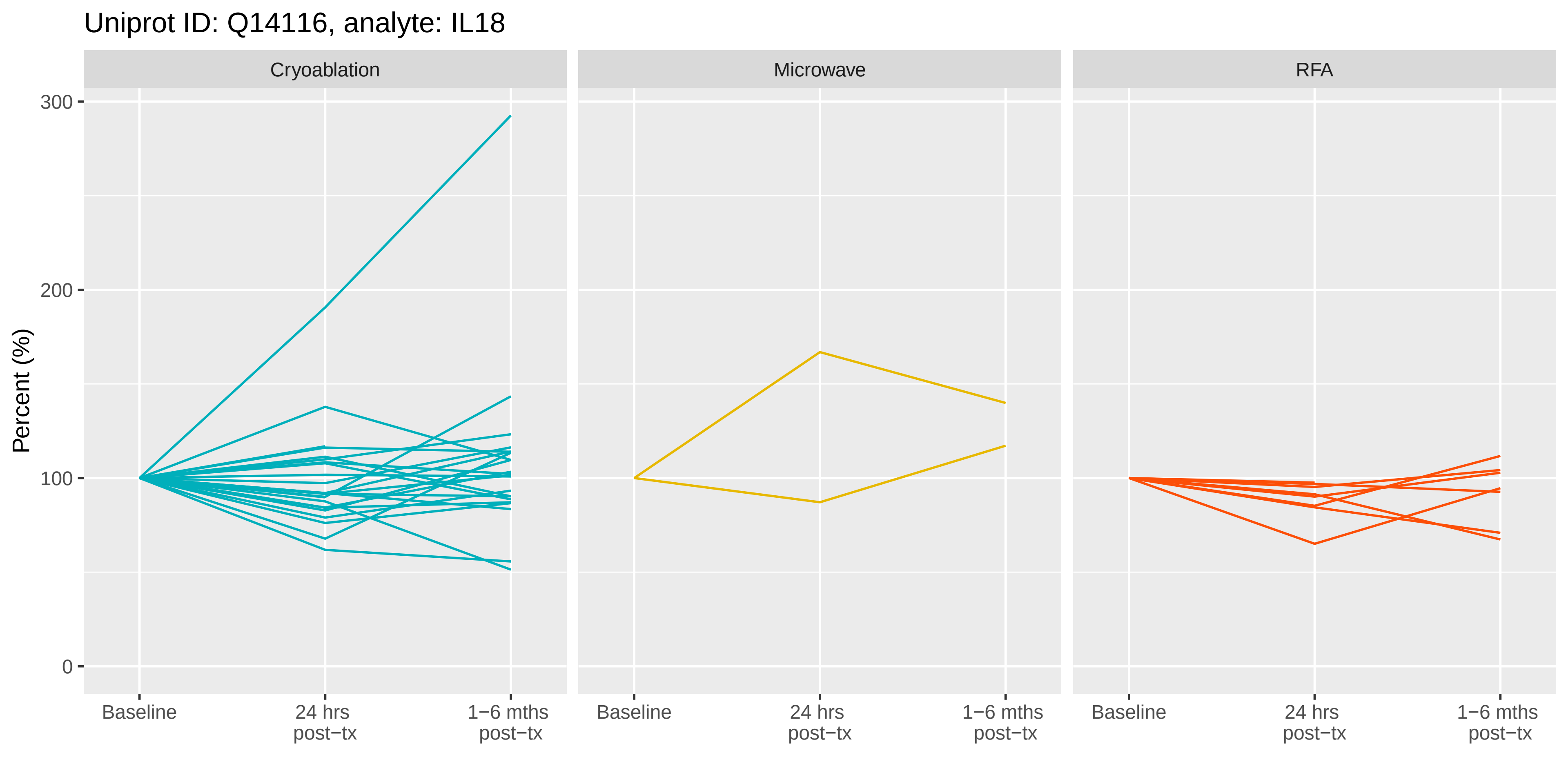

Uniprot ID: Q14116, analyte: IL18
Cryoablation
Microwave
RFA
Percent (%)
300
200
100
0
Baseline
24 hrs post−tx
1−6 mths post−tx

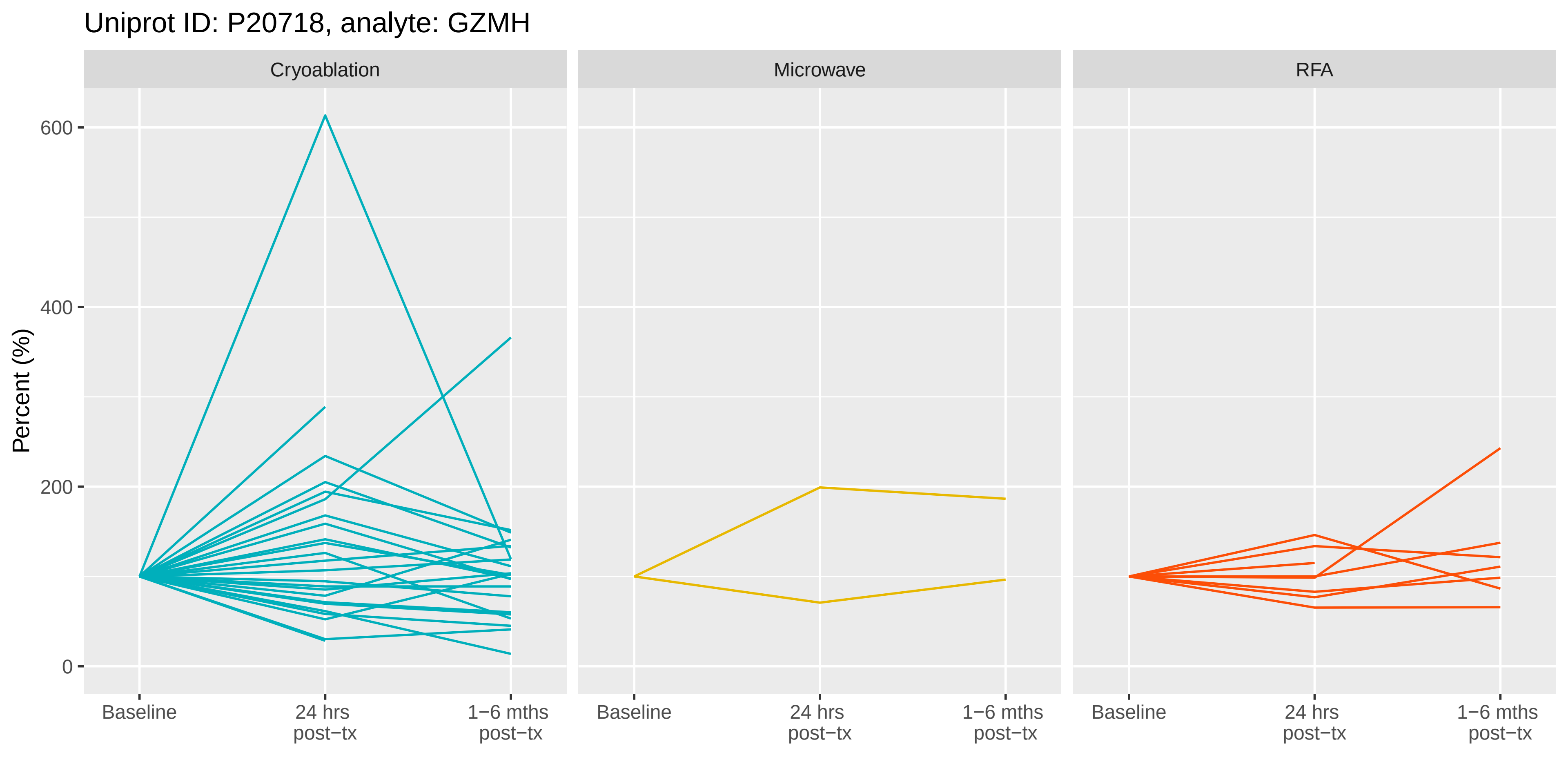

Uniprot ID: P20718, analyte: GZMH
Cryoablation
Microwave
RFA
Percent (%)
600
400
200
0
Baseline
24 hrs post−tx
1−6 mths post−tx

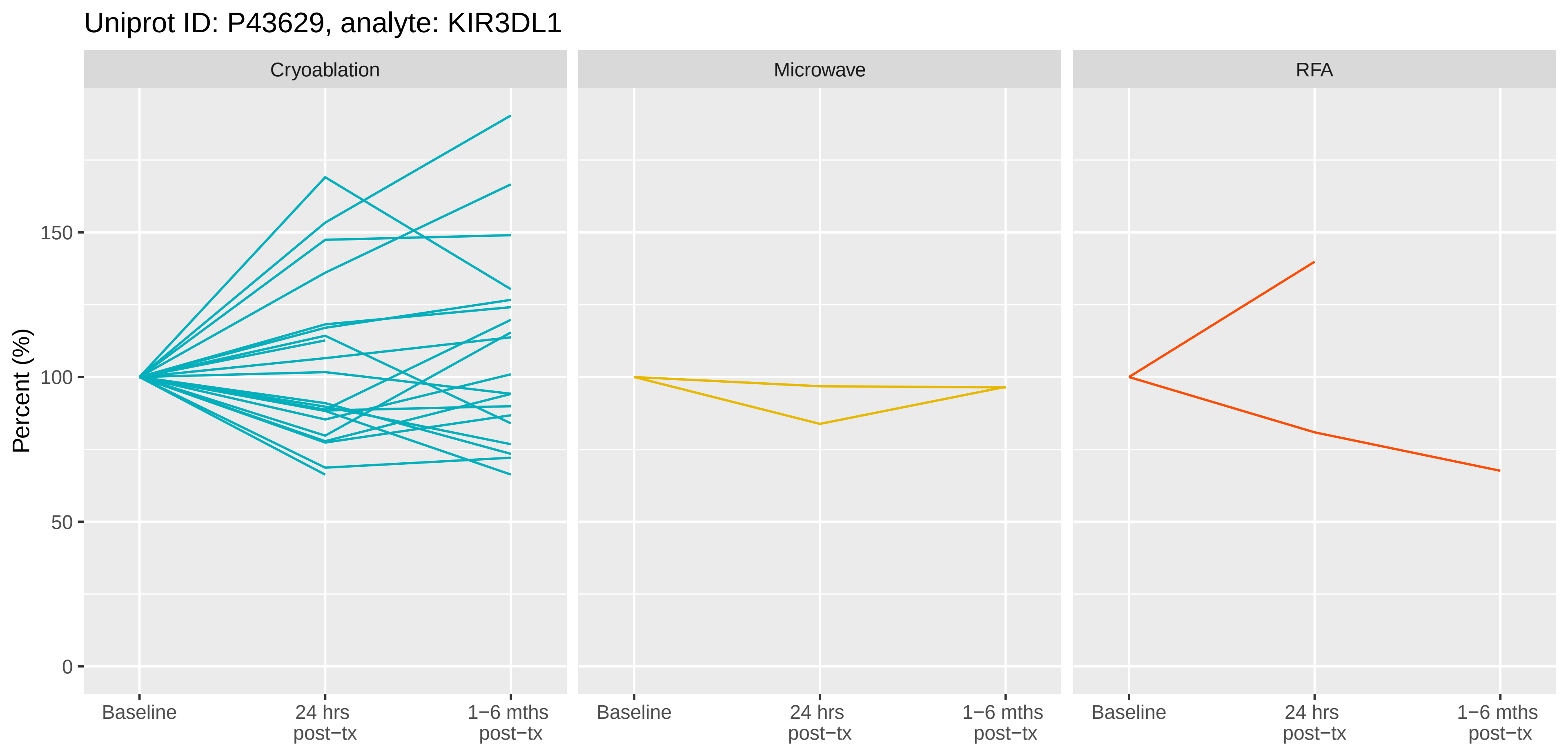
Uniprot ID: P43629, analyte: KIR3DL1
Cryoablation
Microwave
RFA
Percent (%)
0
50
100
150
Baseline
24 hrs post−tx
1−6 mths post−tx

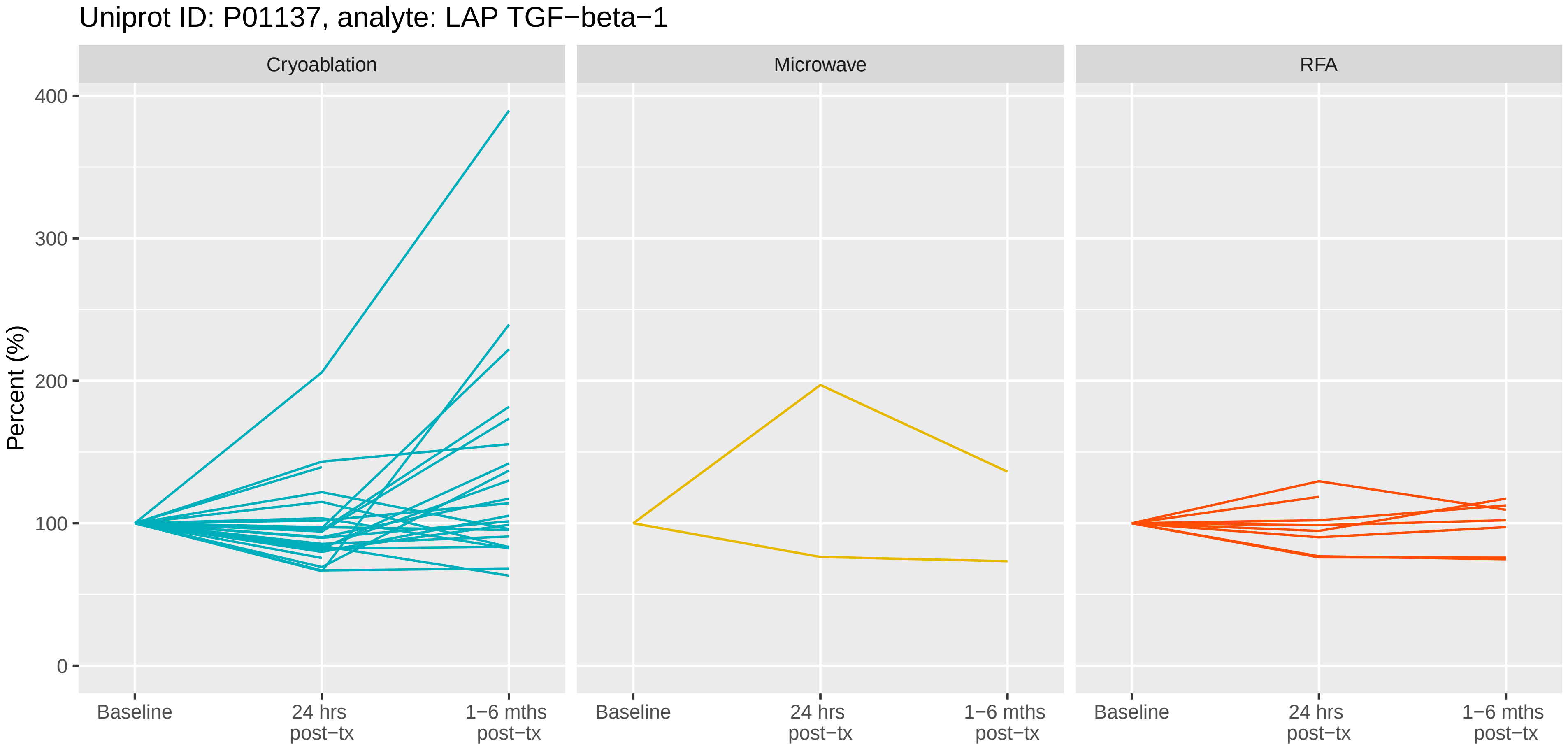

Uniprot ID: P01137, analyte: LAP TGF−beta−1
Cryoablation
Microwave
RFA
Percent (%)
400
300
200
100
0
Baseline
24 hrs post−tx
1−6 mths post−tx

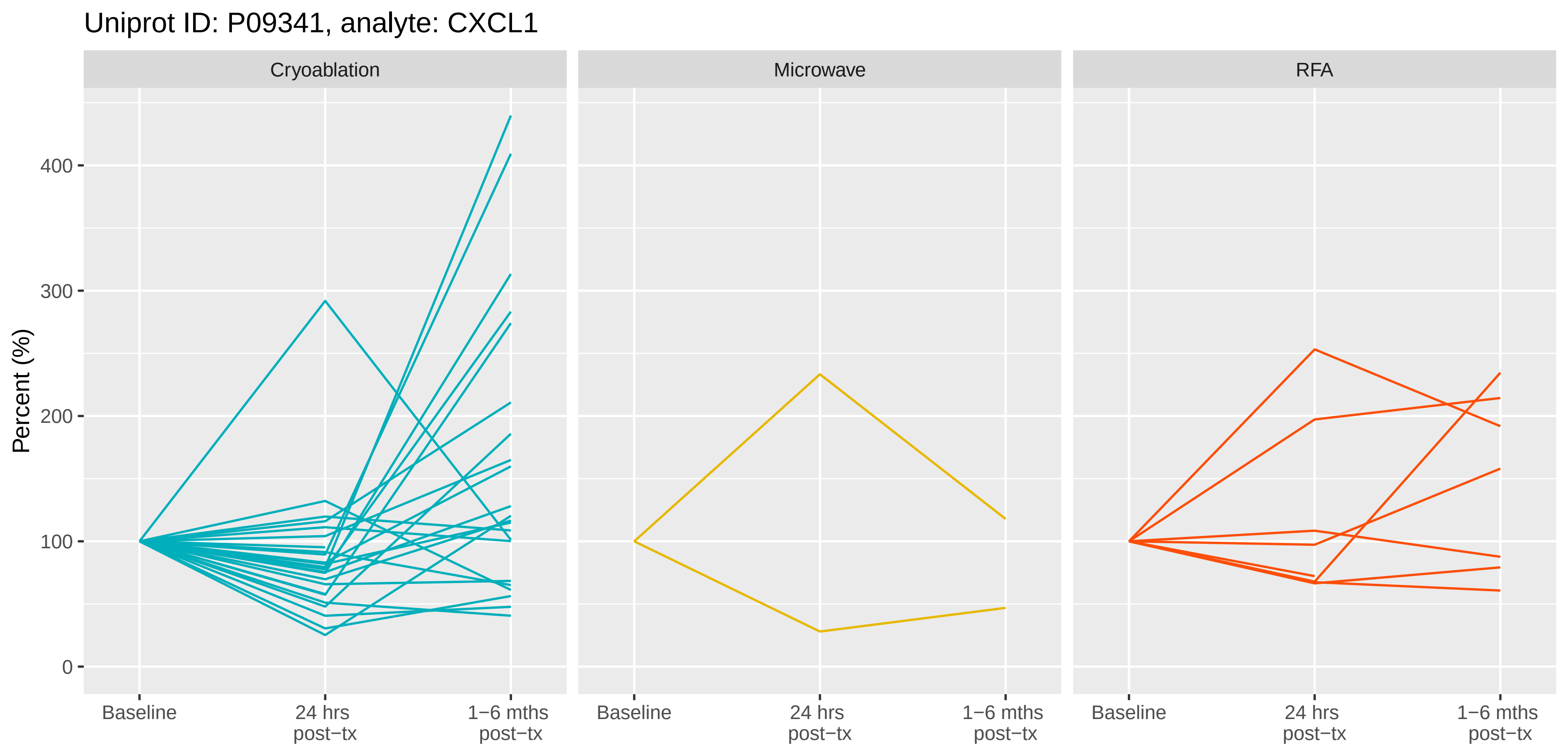
Uniprot ID: P09341, analyte: CXCL1
Cryoablation
Microwave
RFA
Percent (%)
0
100
200
300
400
Baseline
24 hrs post−tx
1−6 mths post−tx

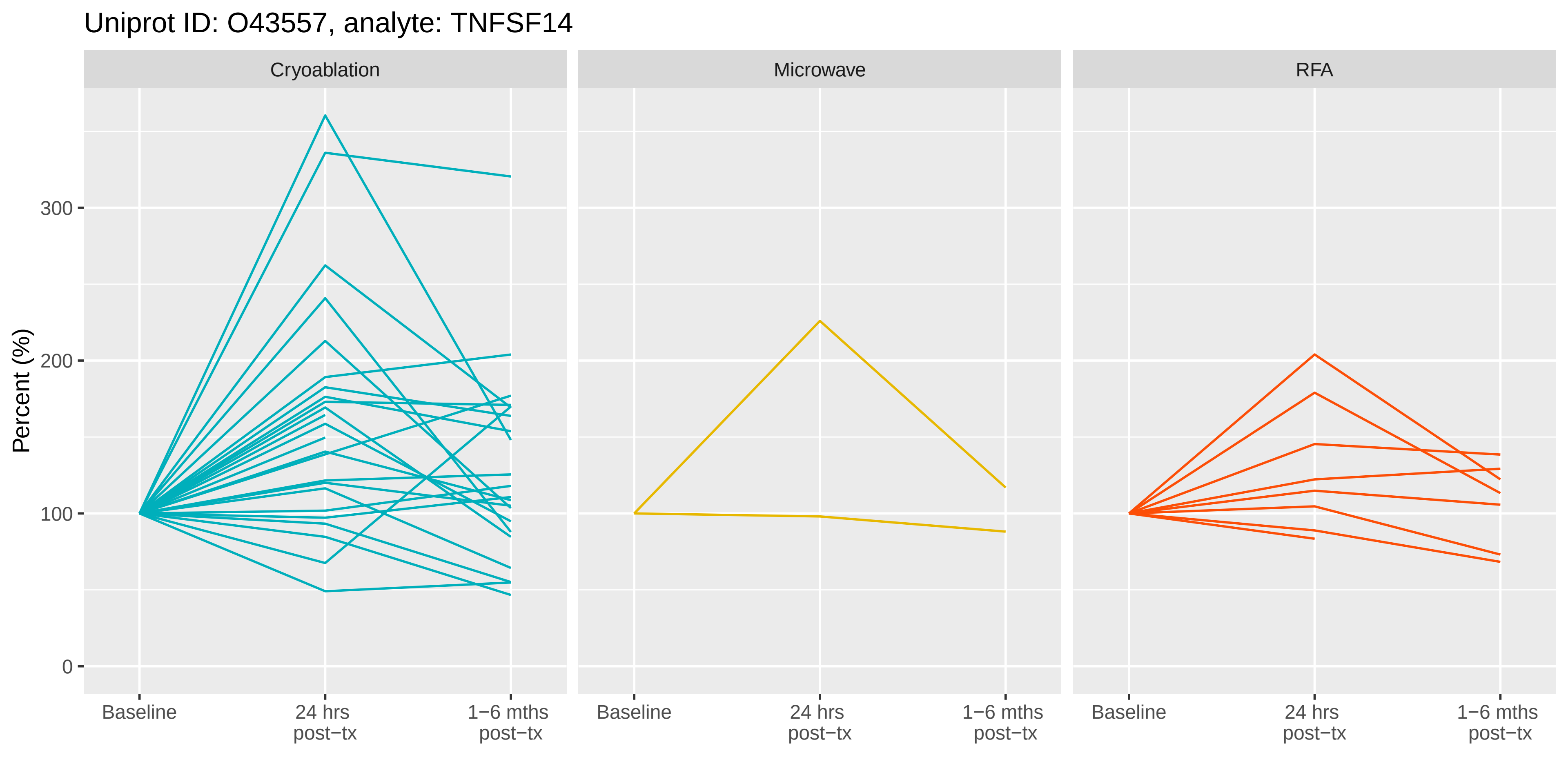

Uniprot ID: O43557, analyte: TNFSF14
Cryoablation
Microwave
RFA
Percent (%)
300
200
100
0
Baseline
24 hrs post−tx
1−6 mths post−tx

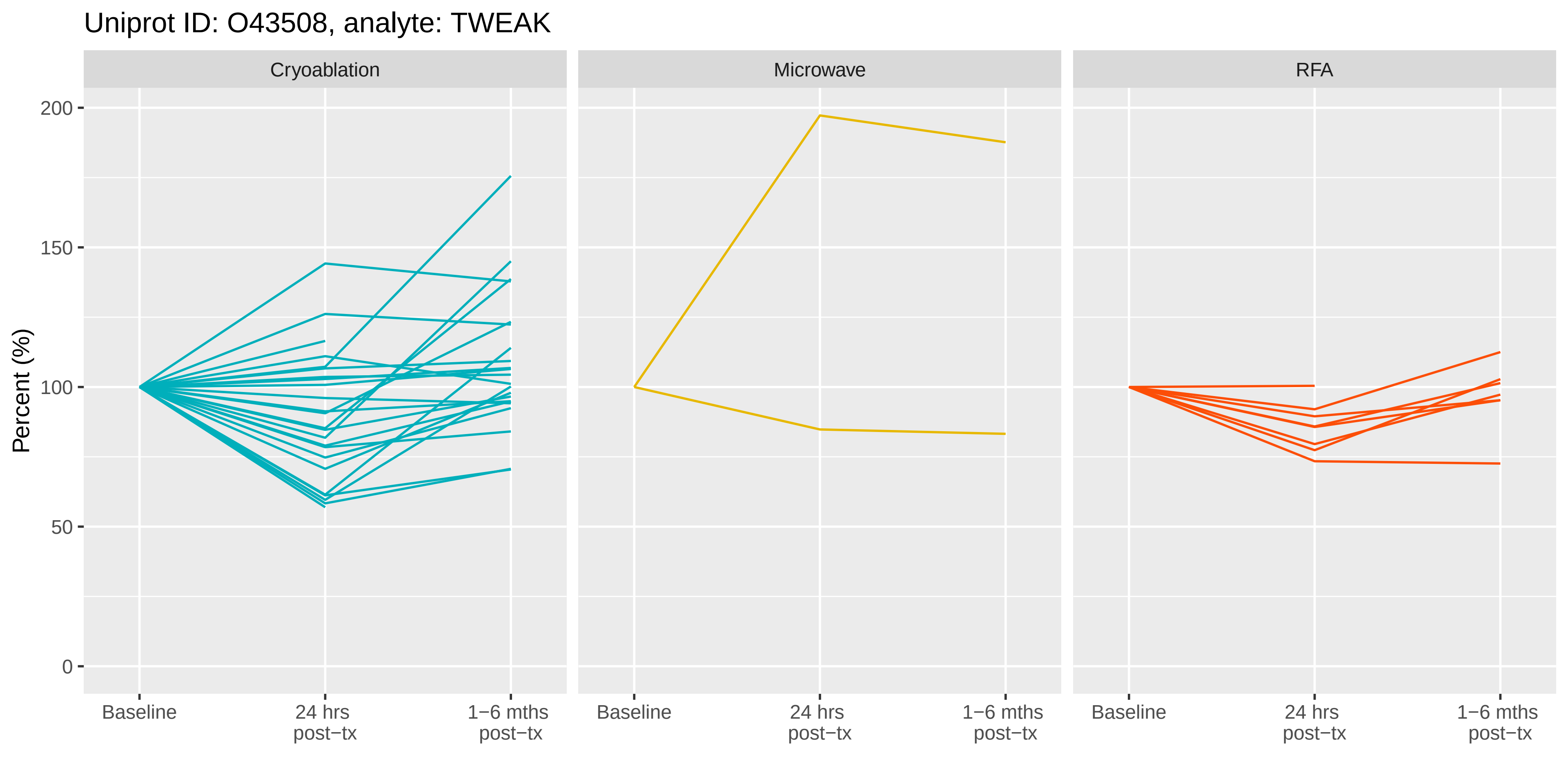
Uniprot ID: O43508, analyte: TWEAK
Cryoablation
Microwave
RFA
Percent (%)
200
150
100
50
0
Baseline
24 hrs post−tx
1−6 mths post−tx

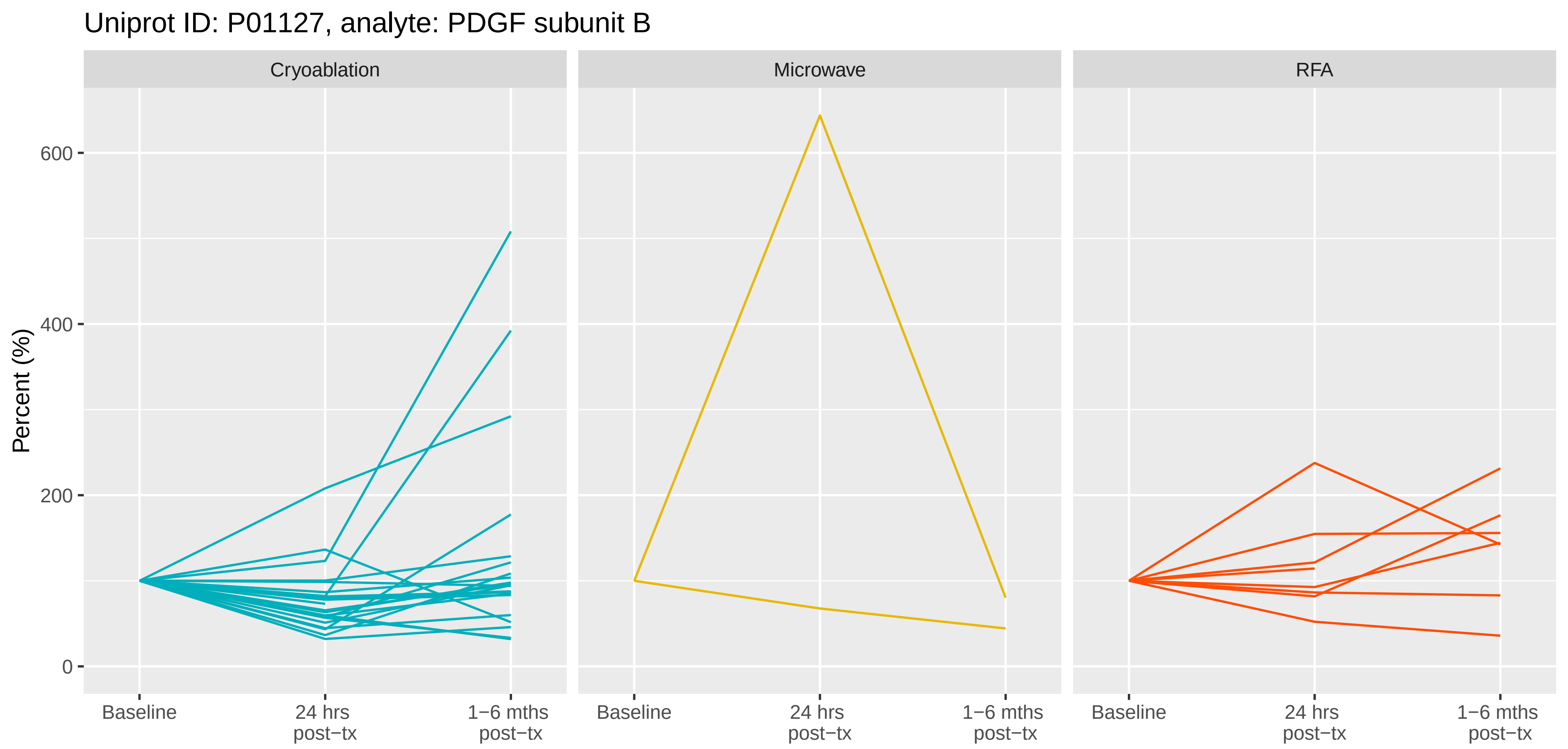
Uniprot ID: P01127, analyte: PDGF subunit B
Cryoablation
Microwave
RFA
Percent (%)
600
400
200
0
Baseline
24 hrs post−tx
1−6 mths post−tx

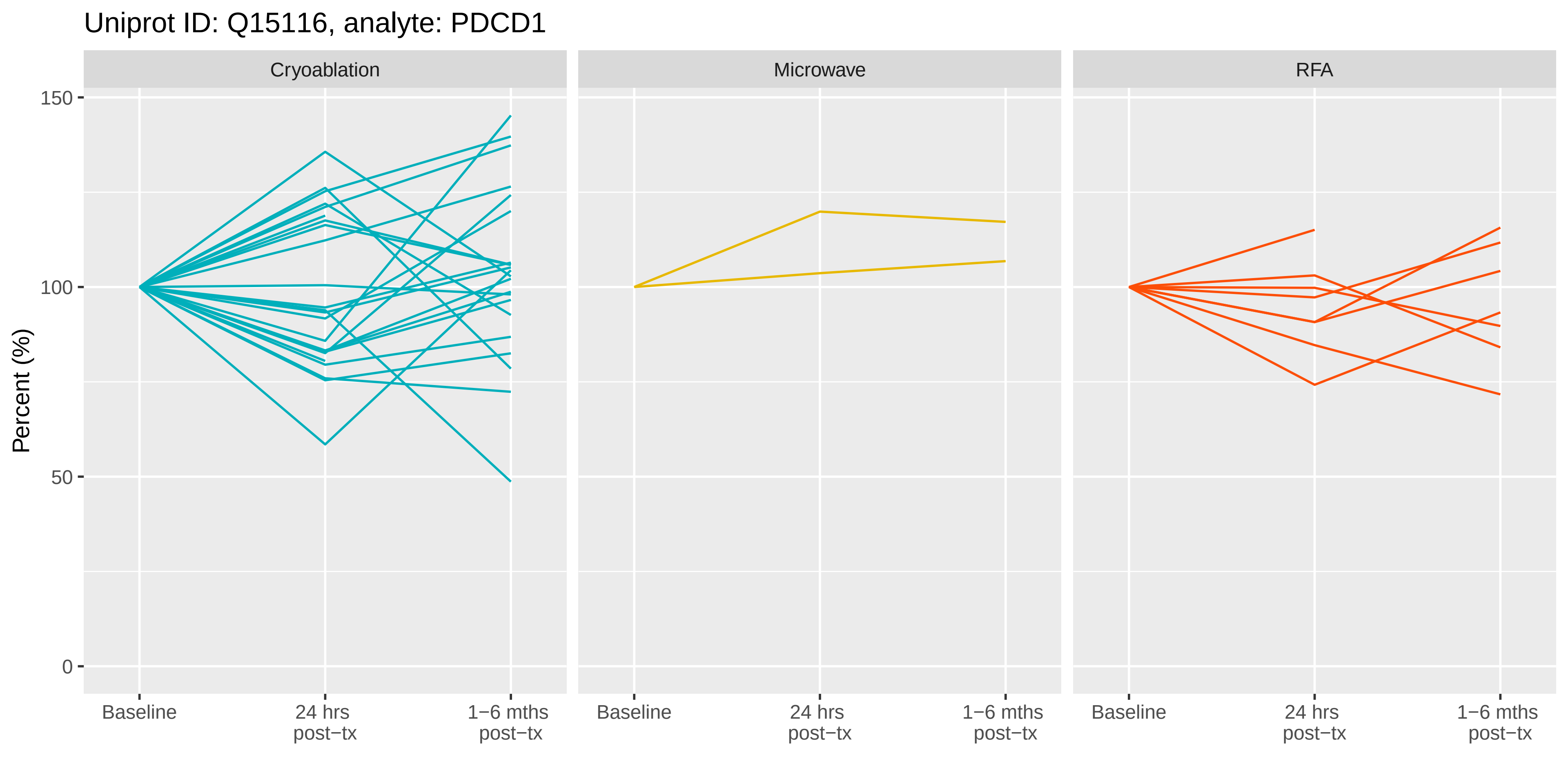
Uniprot ID: Q15116, analyte: PDCD1
Cryoablation
Microwave
RFA
Percent (%)
150
100
50
0
Baseline
24 hrs post−tx
1−6 mths post−tx

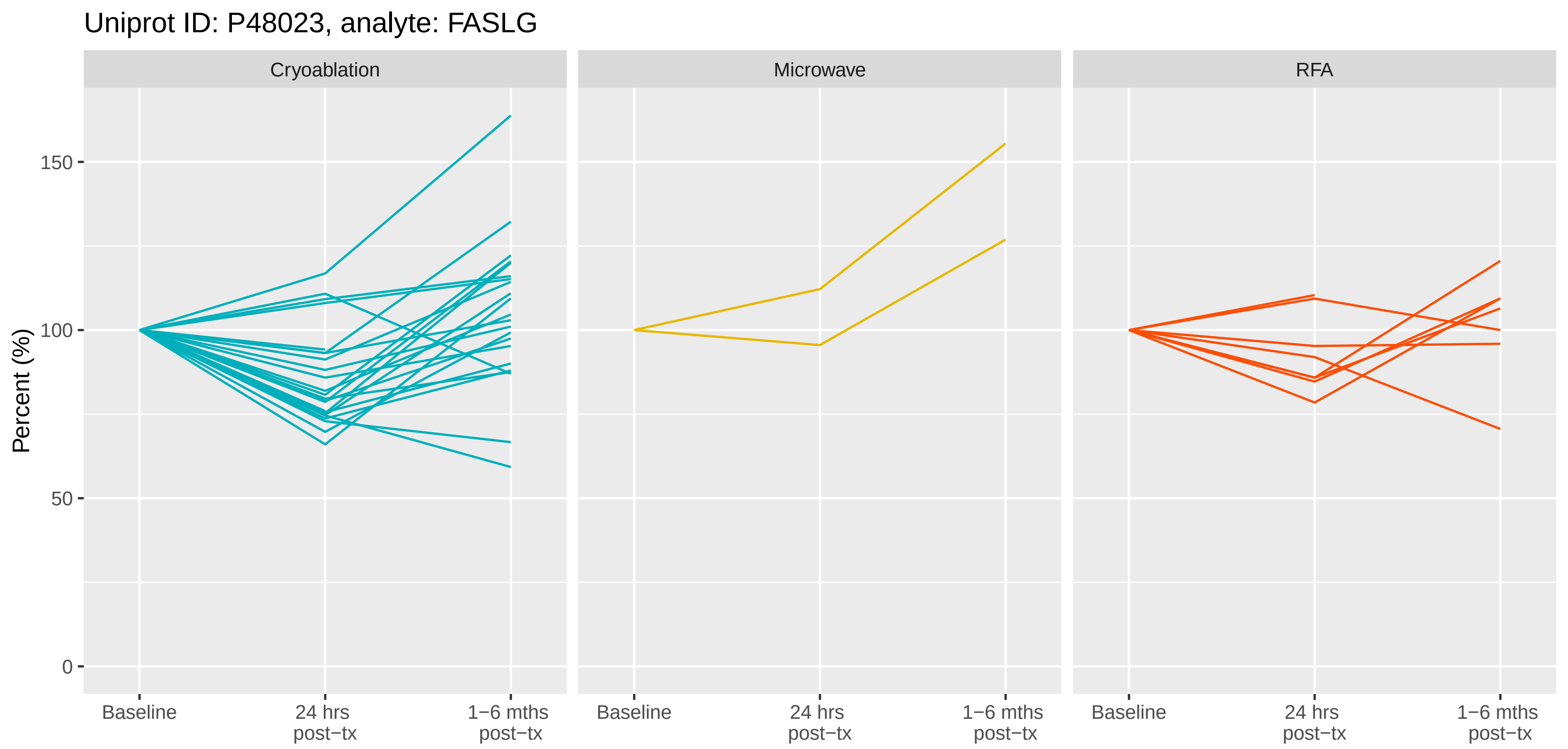

Uniprot ID: P48023, analyte: FASLG
Cryoablation
Microwave
RFA
Percent (%)
150
100
50
0
Baseline
24 hrs post−tx
1−6 mths post−tx

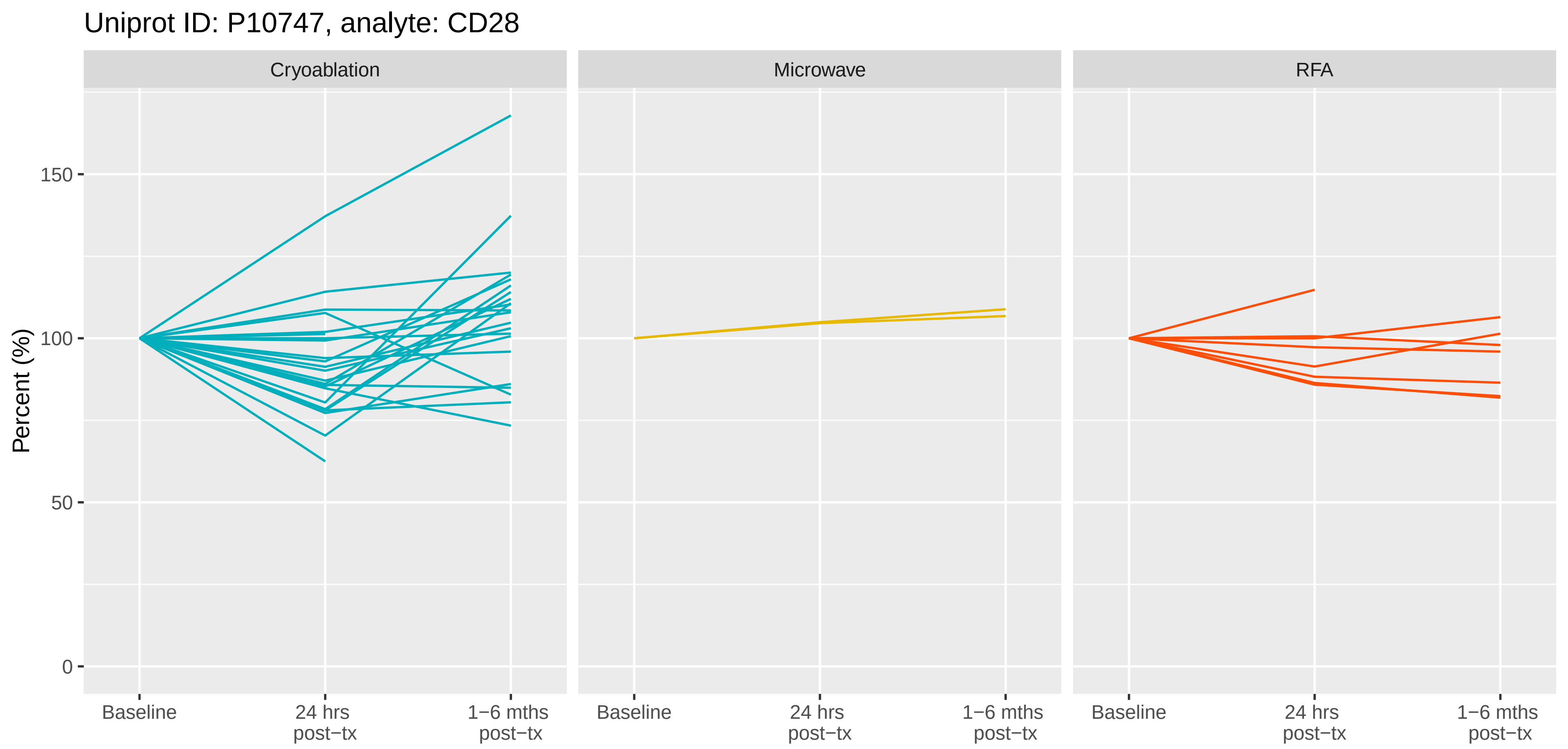

Uniprot ID: P10747, analyte: CD28
Cryoablation
Microwave
RFA
Percent (%)
150
100
50
0
Baseline
24 hrs post−tx
1−6 mths post−tx

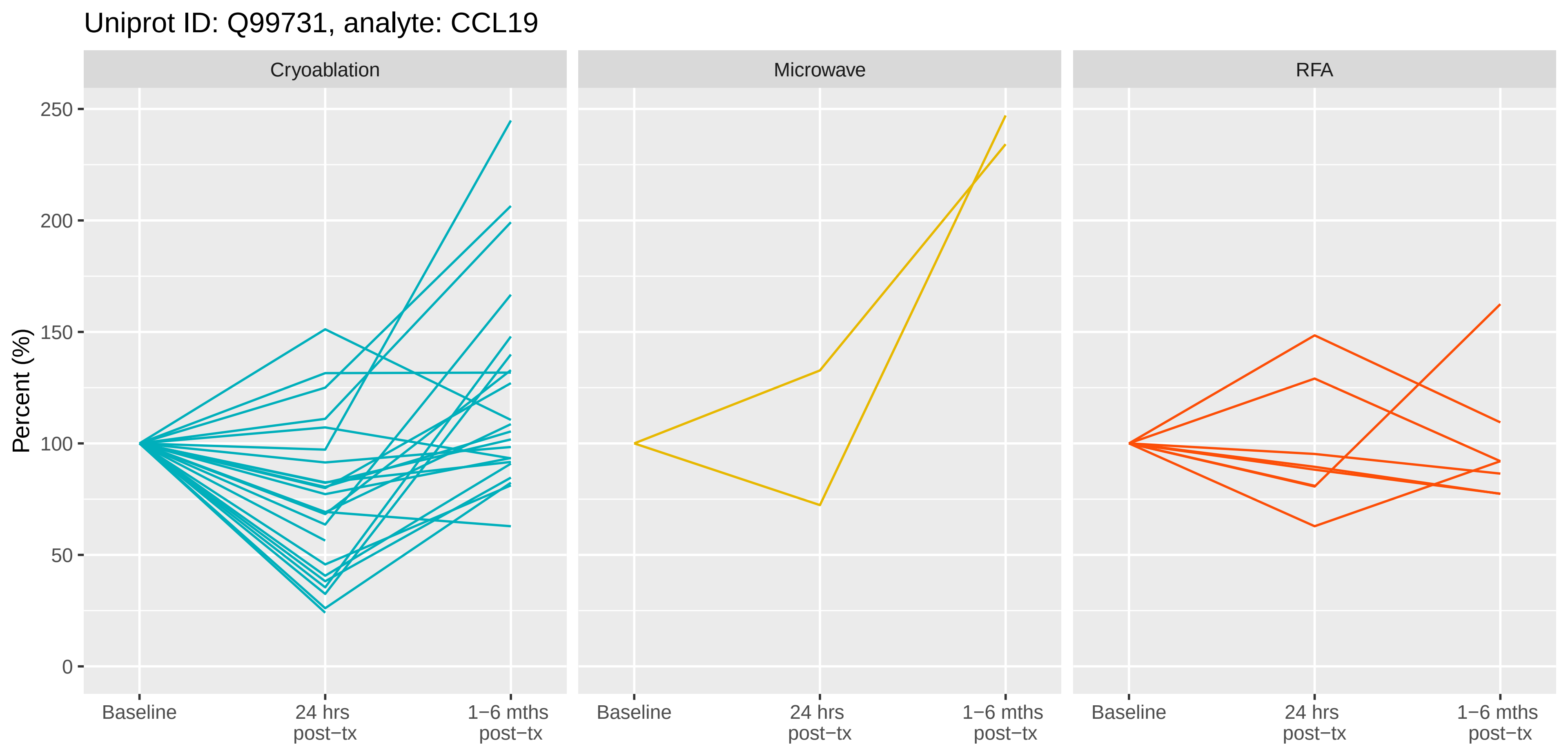
Uniprot ID: Q99731, analyte: CCL19
Cryoablation
Microwave
RFA
Percent (%)
0
50
100
150
200
250
Baseline
24 hrs post−tx
1−6 mths post−tx

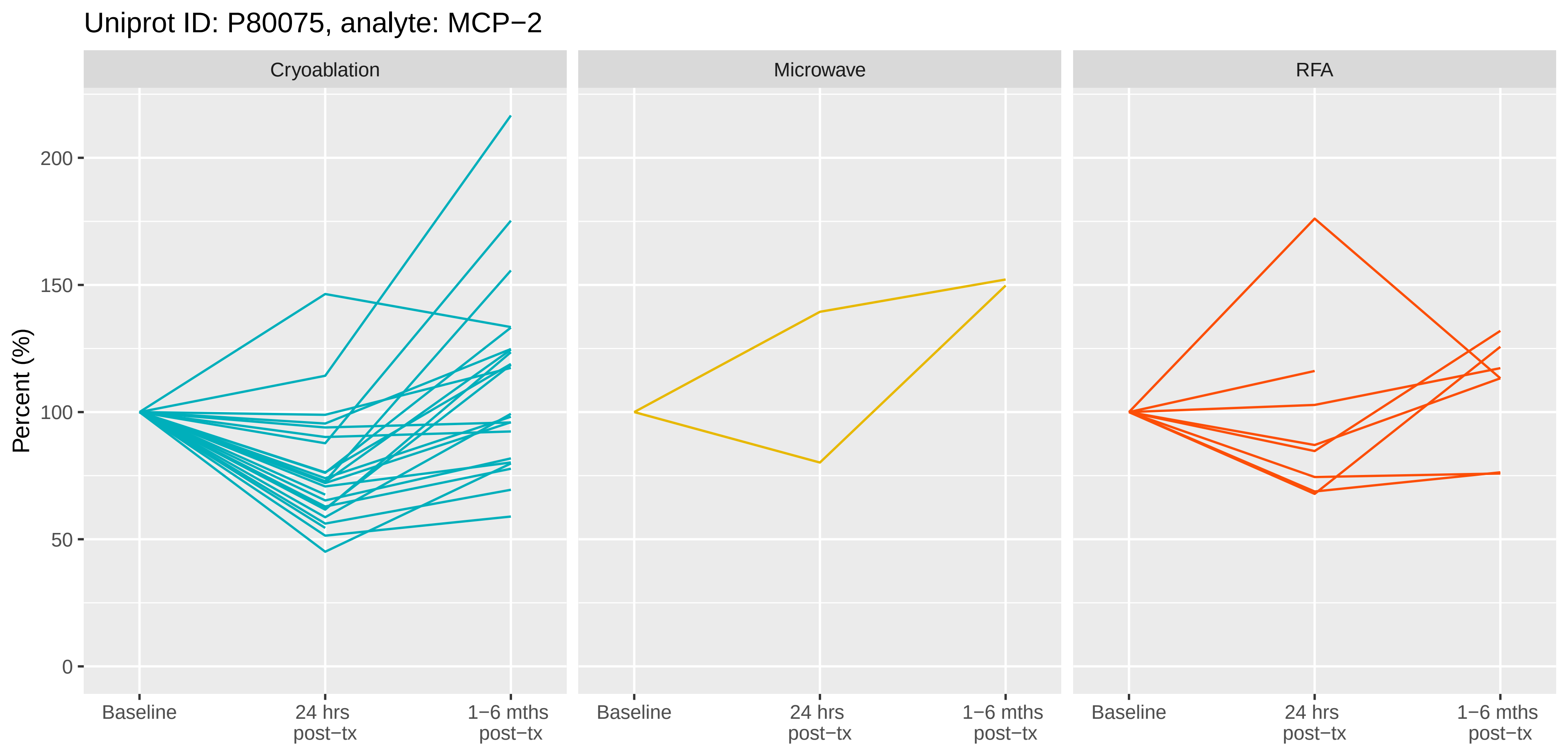
Uniprot ID: P80075, analyte: MCP−2
Cryoablation
Microwave
RFA
Percent (%)
200
150
100
50
0
Baseline
24 hrs post−tx
1−6 mths post−tx

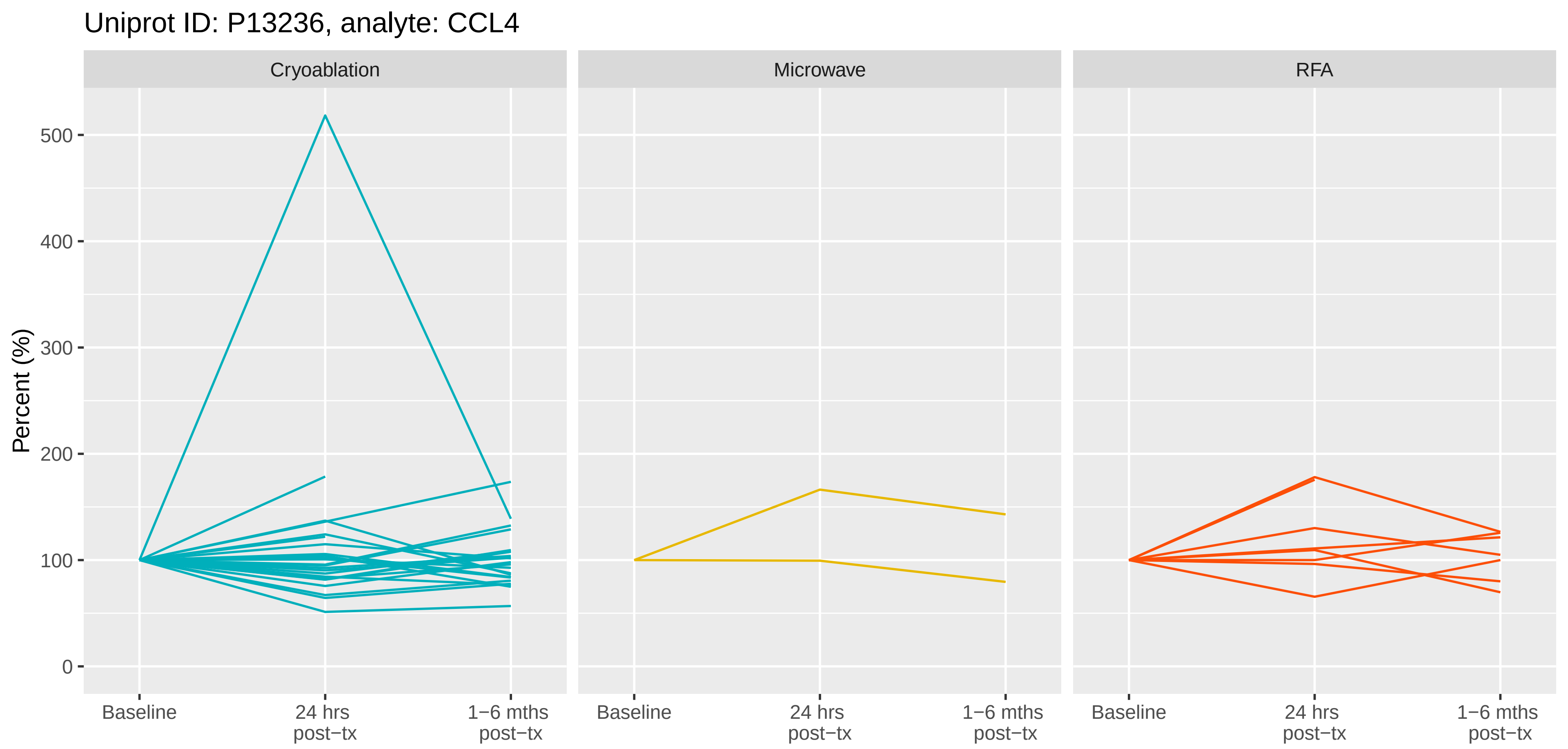
Uniprot ID: P13236, analyte: CCL4
Cryoablation
Microwave
RFA
Percent (%)
0
100
200
300
400
500
Baseline
24 hrs post−tx
1−6 mths post−tx

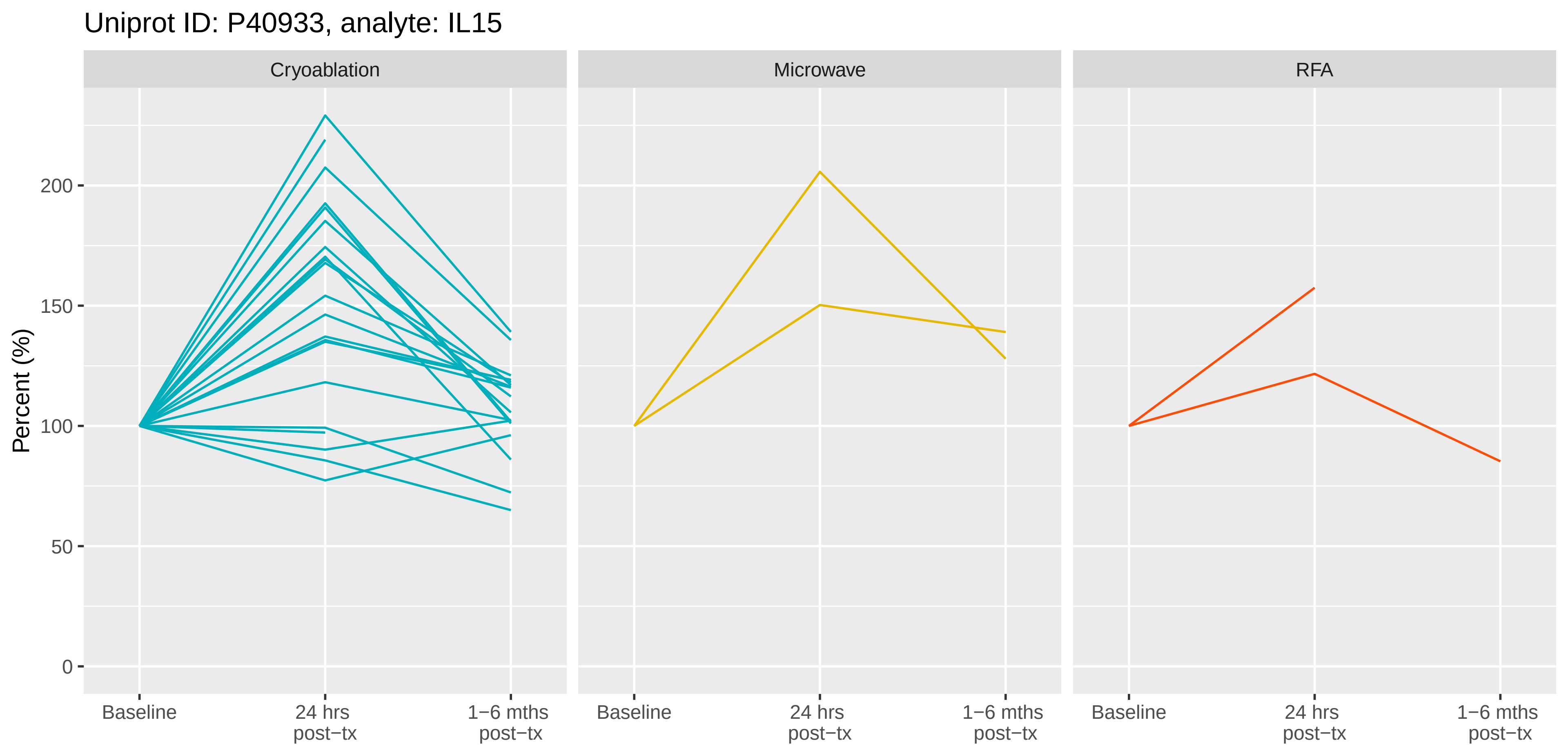
Uniprot ID: P40933, analyte: IL15
Cryoablation
Microwave
RFA
Percent (%)
0
50
100
150
200
Baseline
24 hrs post−tx
1−6 mths post−tx

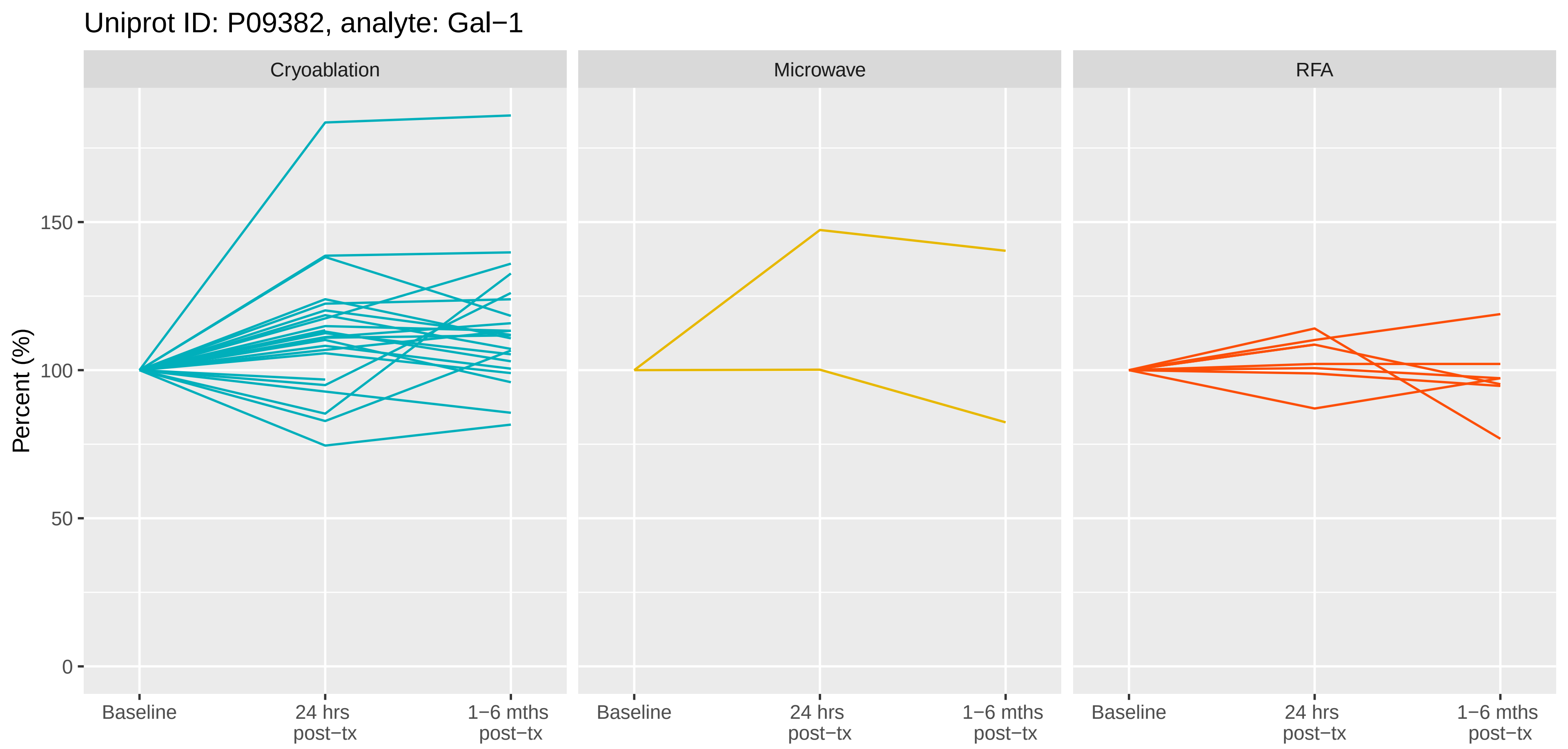

Uniprot ID: P09382, analyte: Gal−1
Cryoablation
Microwave
RFA
Percent (%)
150
100
50
0
Baseline
24 hrs post−tx
1−6 mths post−tx

Uniprot ID: Q9NZQ7, analyte: PD−L1

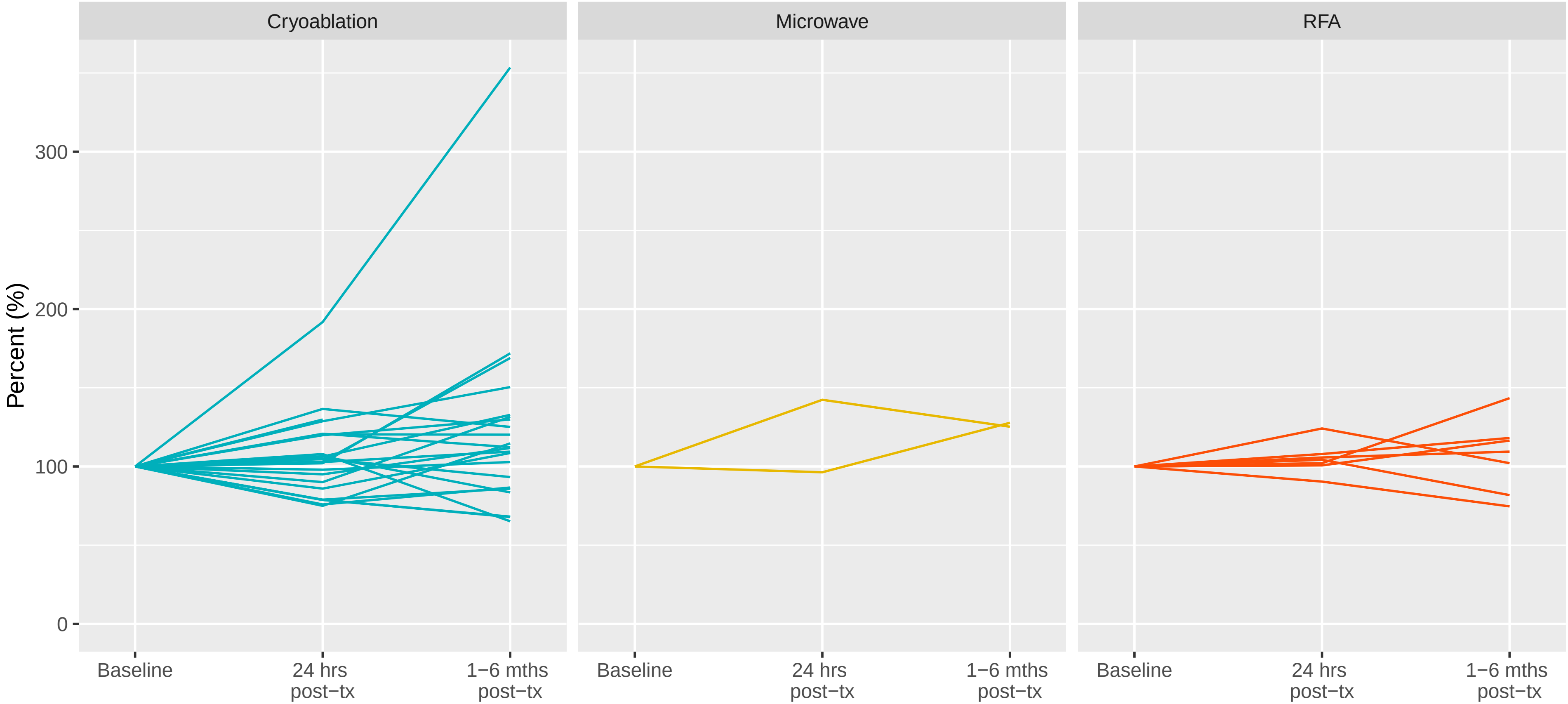

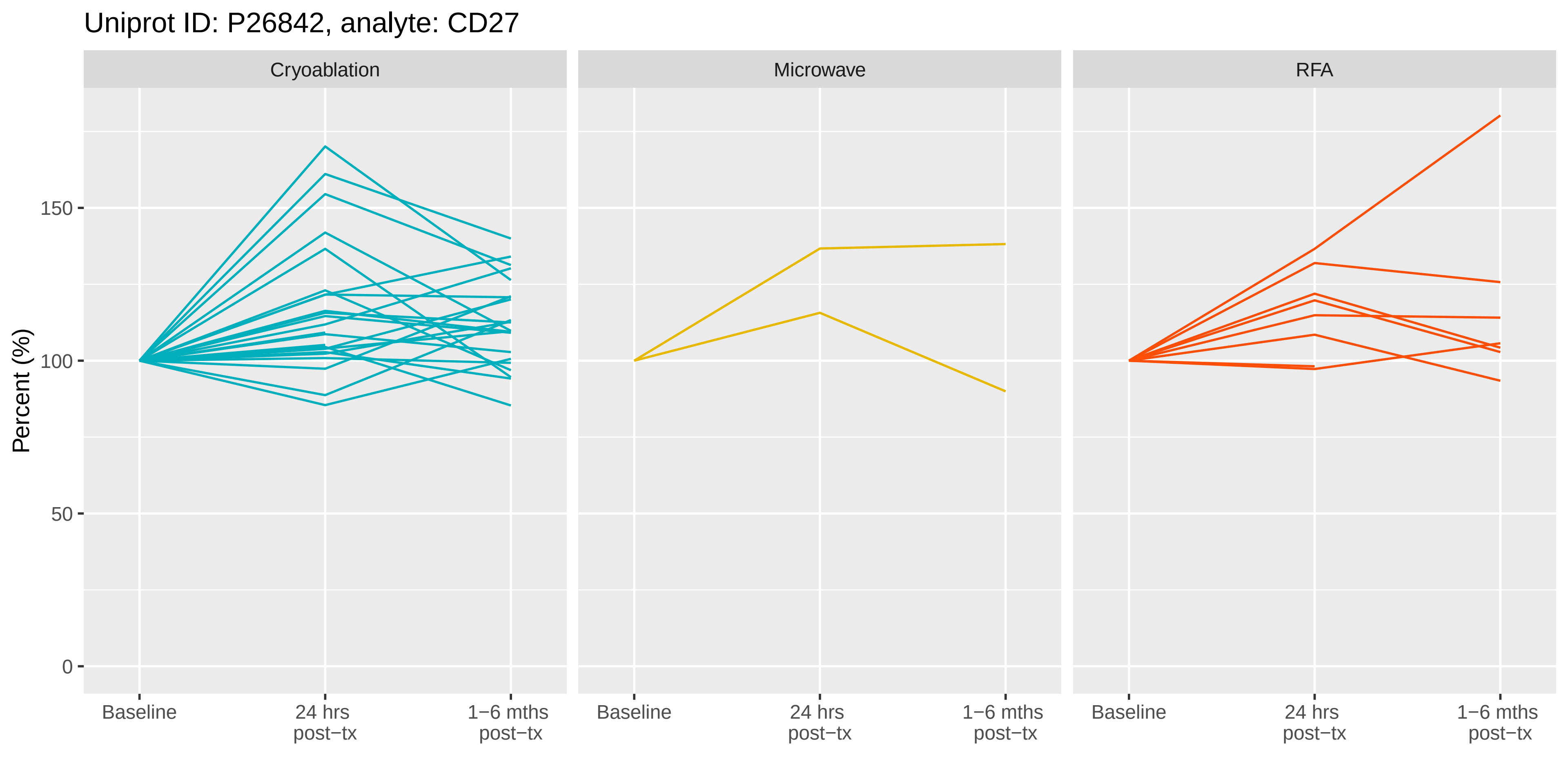

Uniprot ID: P26842, analyte: CD27
Cryoablation
Microwave
RFA
Percent (%)
150
100
50
0
Baseline
24 hrs post−tx
1−6 mths post−tx
Baseline
24 hrs post−tx
1−6 mths post−tx
Baseline
24 hrs post−tx
1−6 mths post−tx

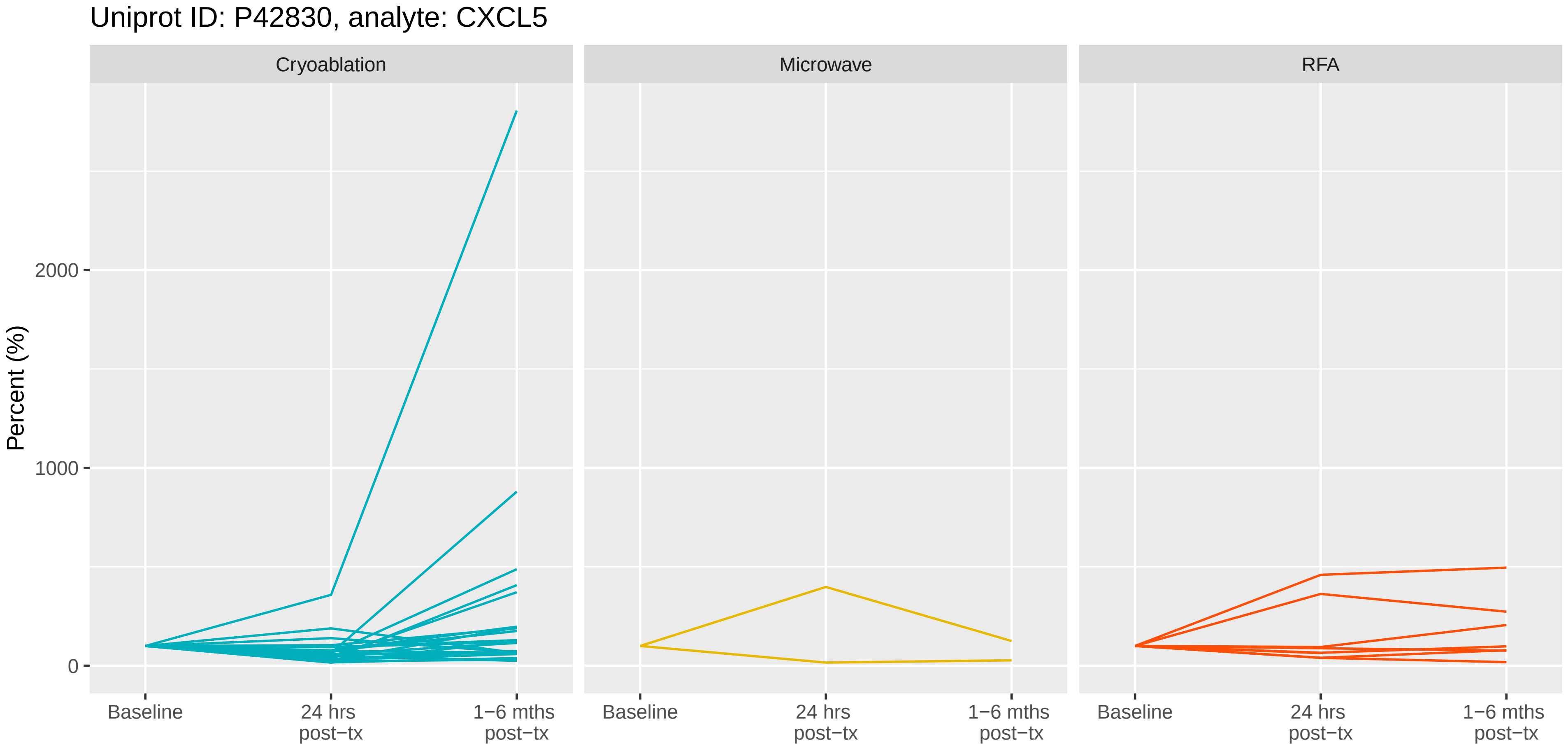
Uniprot ID: P42830, analyte: CXCL5
Cryoablation
Microwave
RFA
Percent (%)
0
1000
2000
Baseline
24 hrs post−tx
1−6 mths post−tx

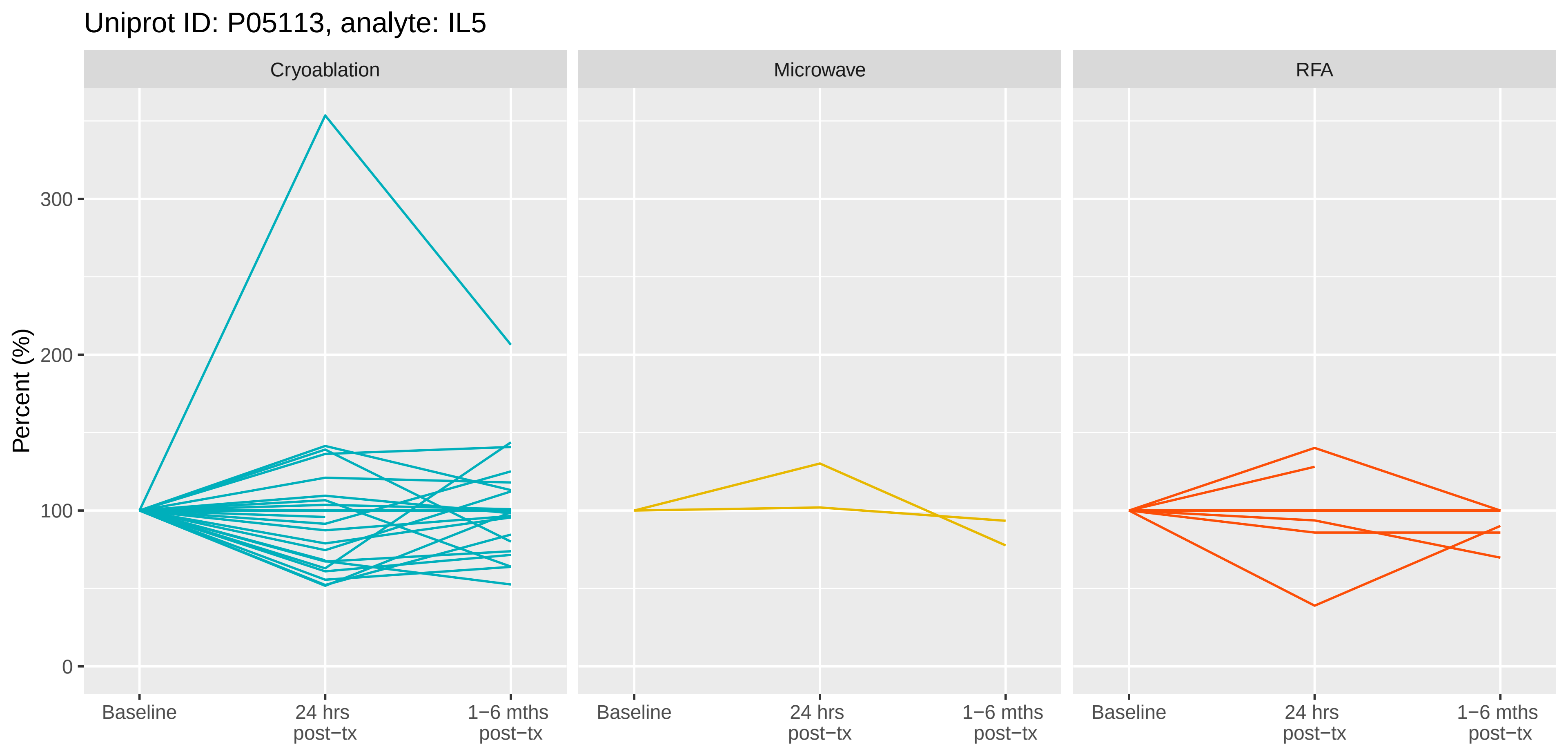

Uniprot ID: P05113, analyte: IL5
Cryoablation
Microwave
RFA
Percent (%)
300
200
100
0
Baseline
24 hrs post−tx
1−6 mths post−tx

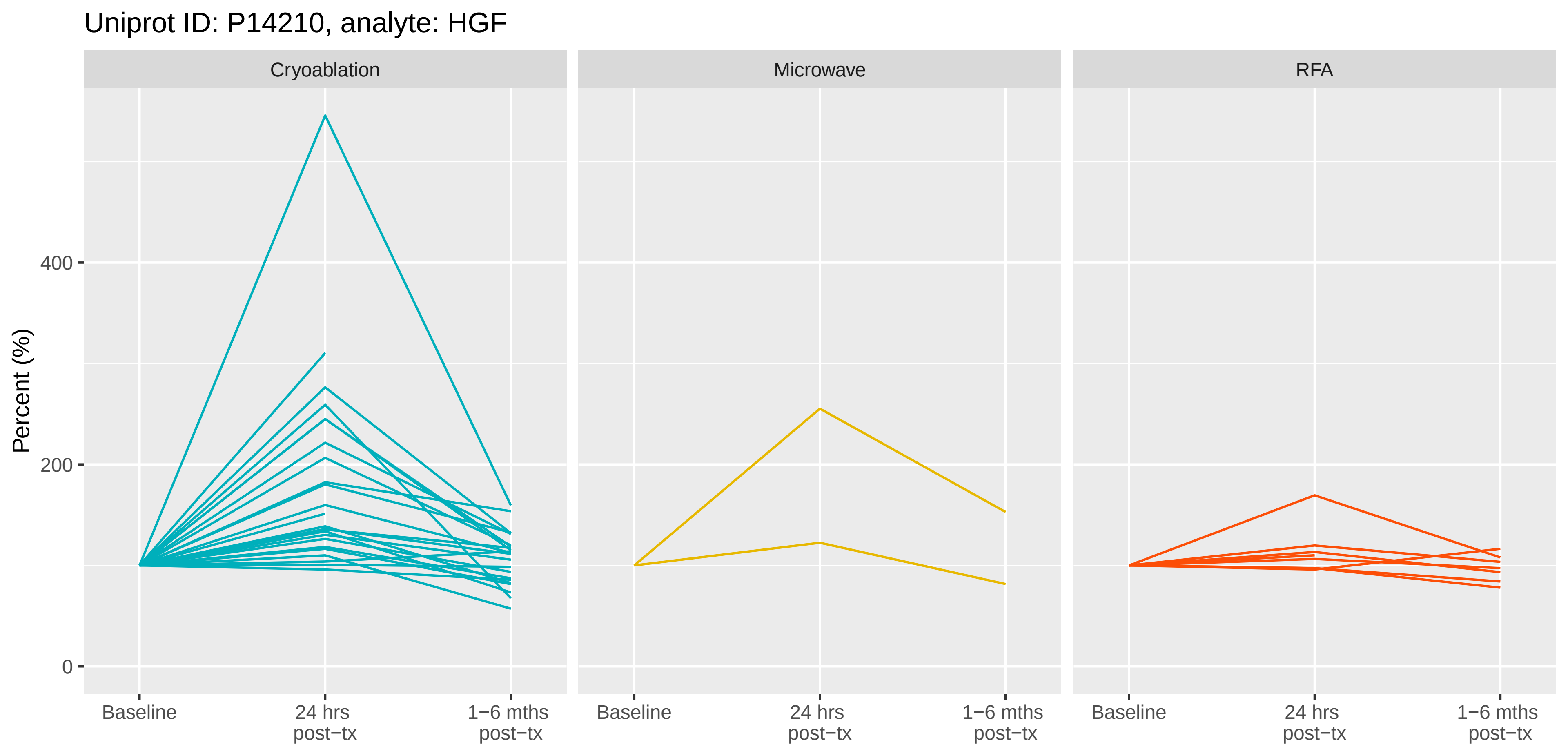

Uniprot ID: P14210, analyte: HGF
Cryoablation
Microwave
RFA
Percent (%)
400
200
0
Baseline
24 hrs post−tx
1−6 mths post−tx

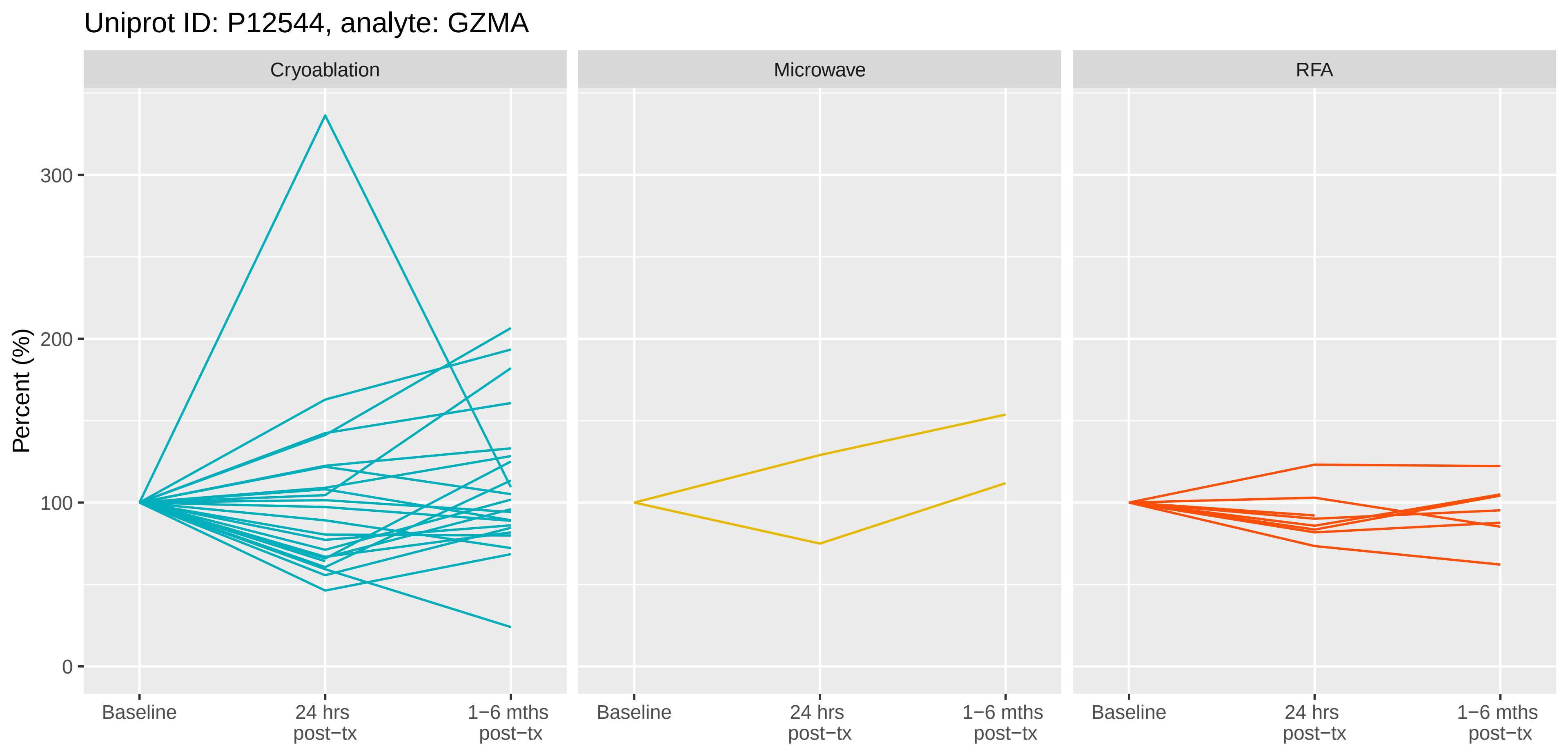

Uniprot ID: P12544, analyte: GZMA
Cryoablation
Microwave
RFA
Percent (%)
300
200
100
0
Baseline
24 hrs post−tx
1−6 mths post−tx

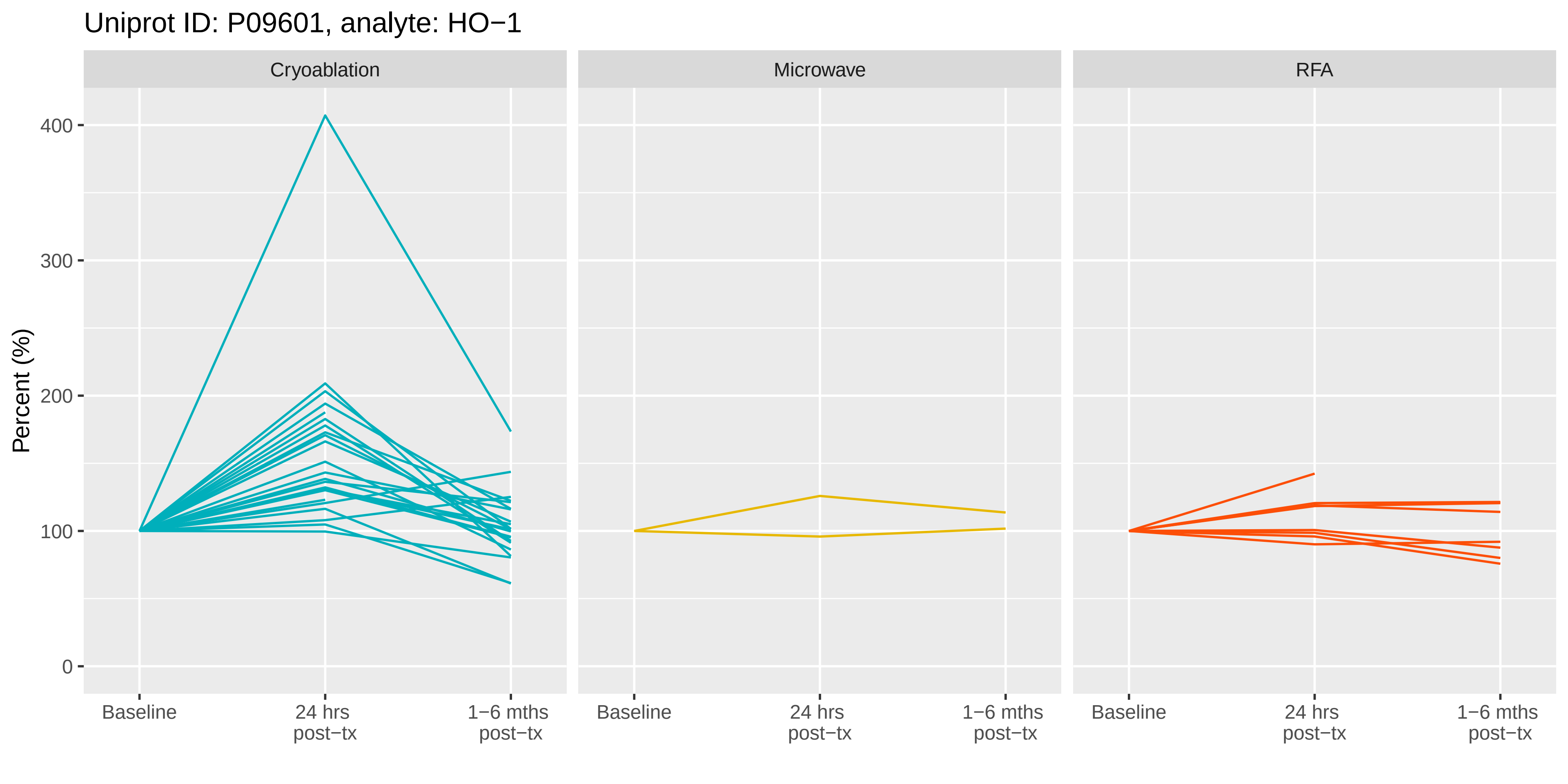
Uniprot ID: P09601, analyte: HO−1
Cryoablation
Microwave
RFA
Percent (%)
400
300
200
100
0
Baseline
24 hrs
post−tx
1−6 mths
post−tx

Uniprot ID: P78423, analyte: CX3CL1

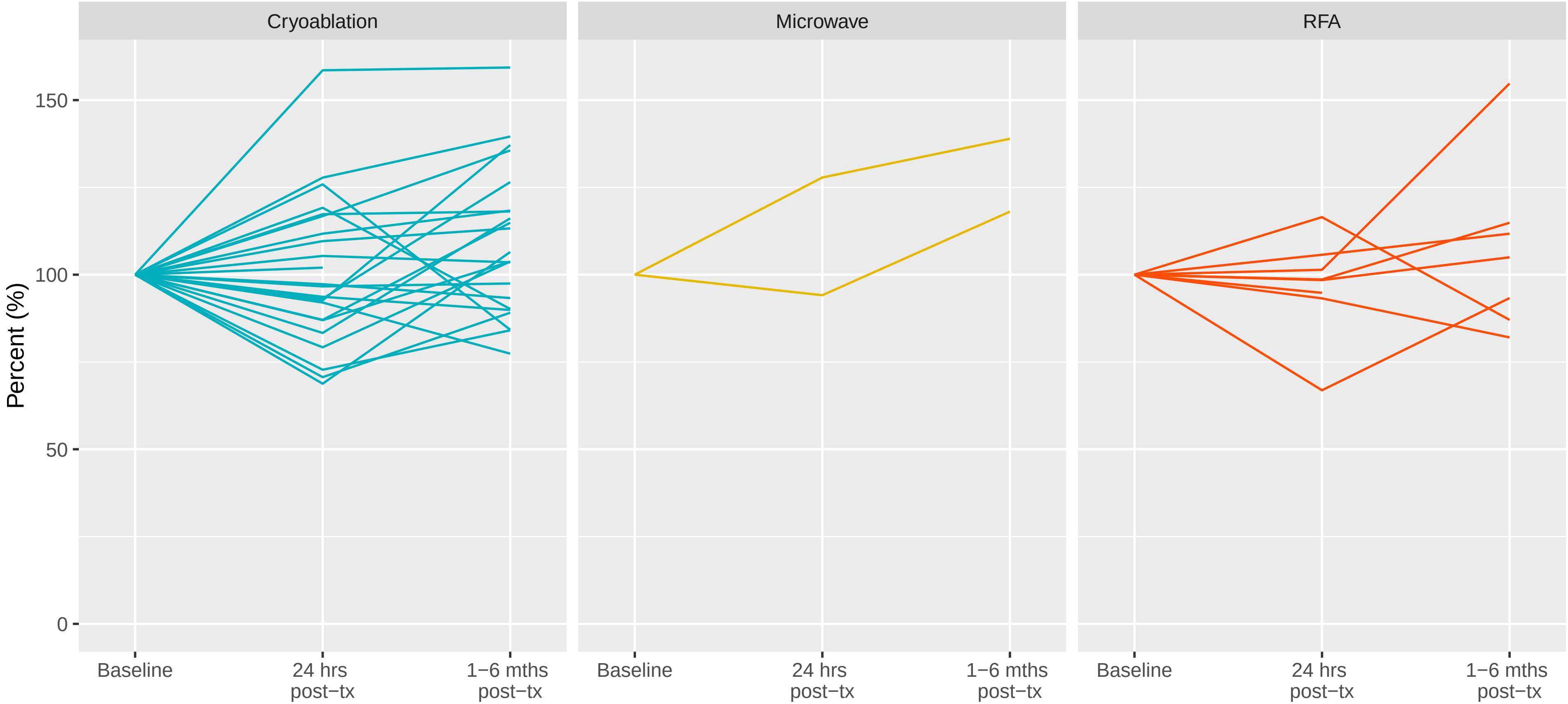

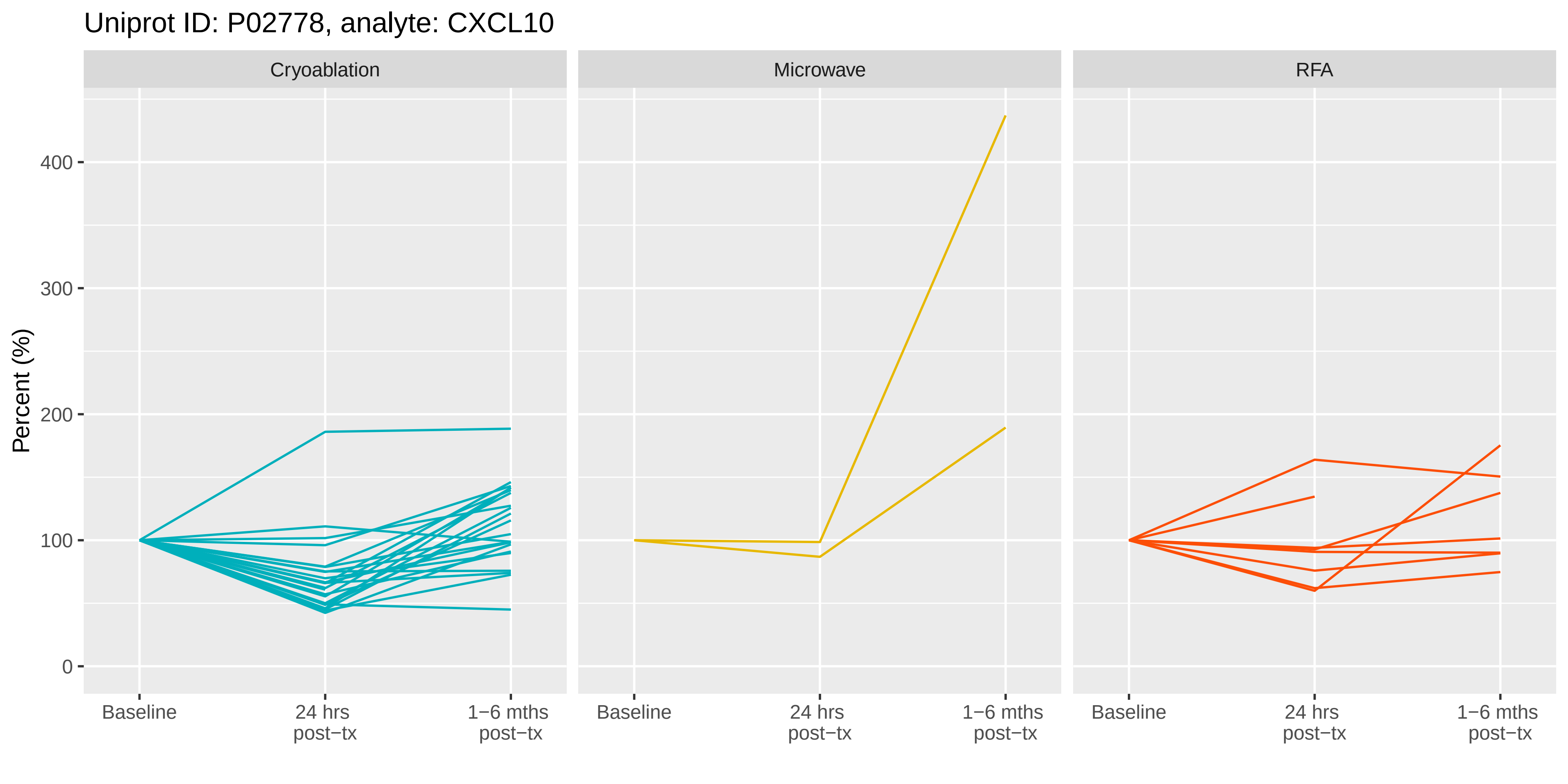

Uniprot ID: P02778, analyte: CXCL10
Cryoablation
Microwave
RFA
Percent (%)
0
100
200
300
400
Baseline
24 hrs post−tx
1−6 mths post−tx

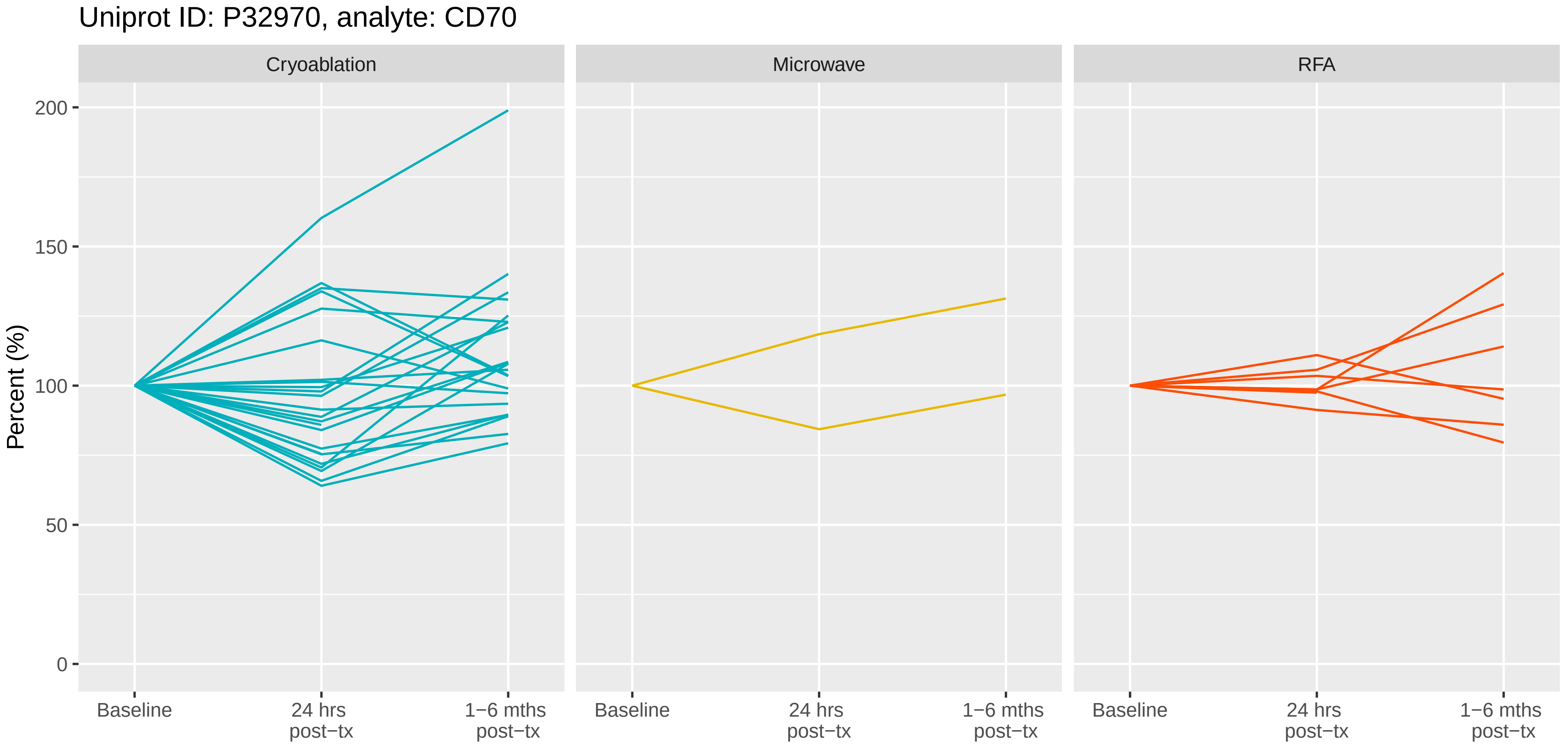
Uniprot ID: P32970, analyte: CD70
Cryoablation
Microwave
RFA
Percent (%)
200
150
100
50
0
Baseline
24 hrs post−tx
1−6 mths post−tx

Uniprot ID: P22301, analyte: IL10

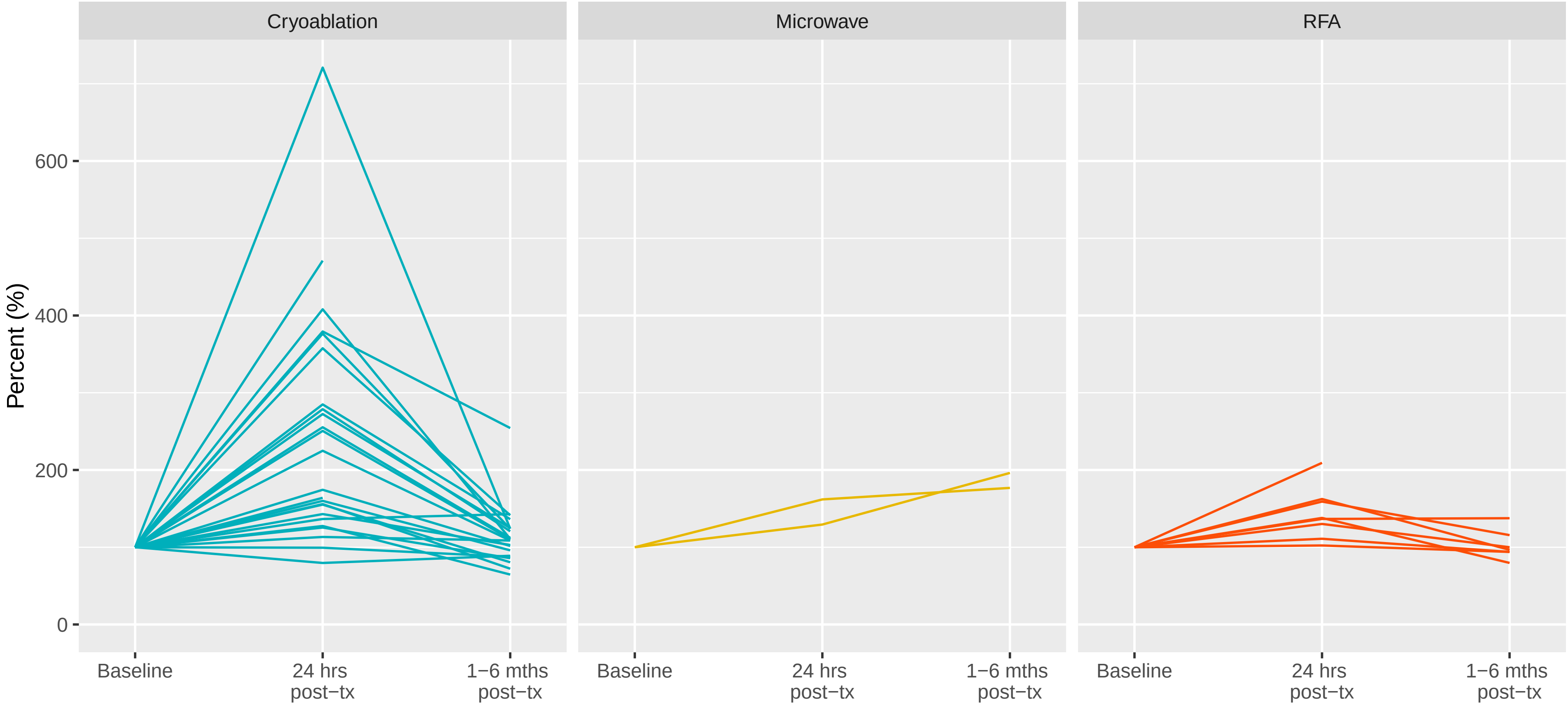

Uniprot ID: Q9NP84, analyte: TNFRSF12A

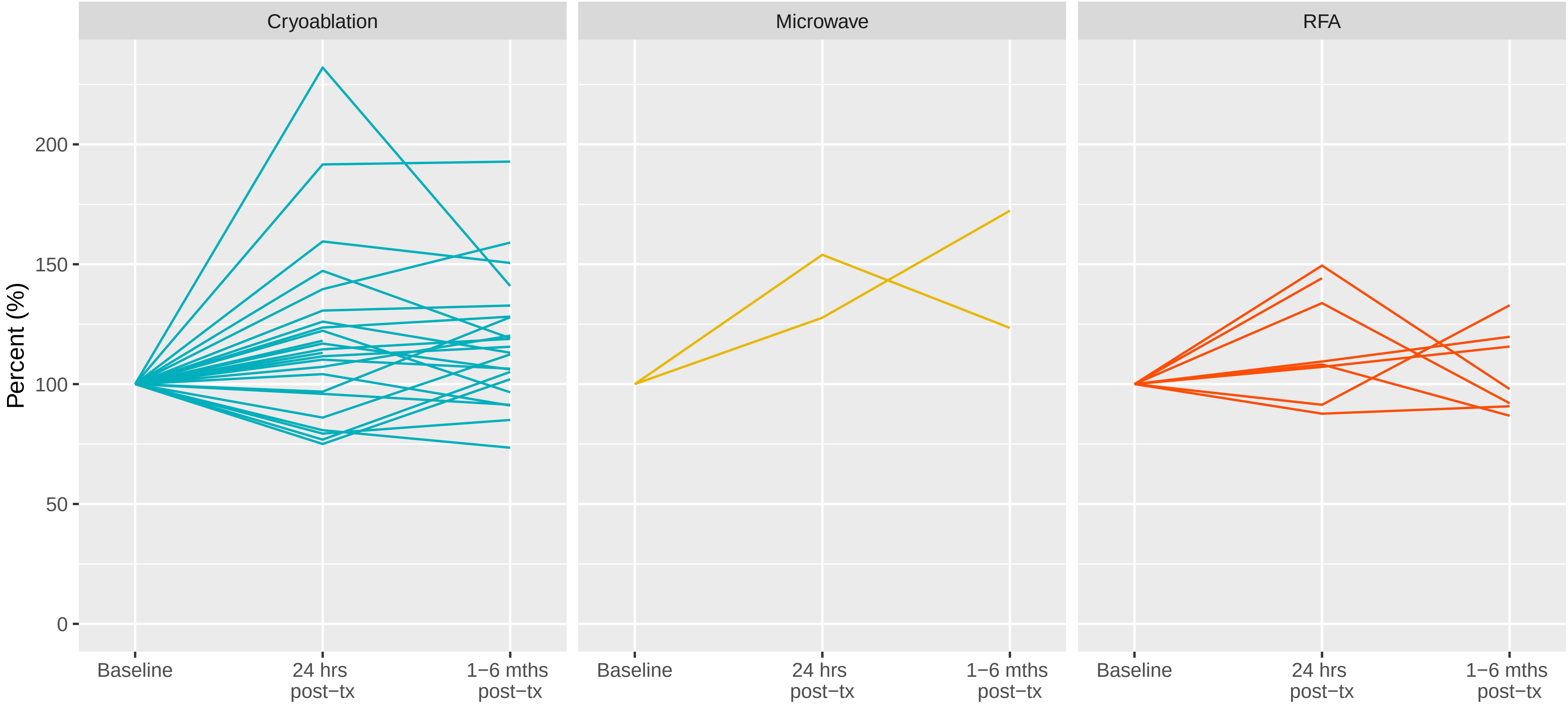

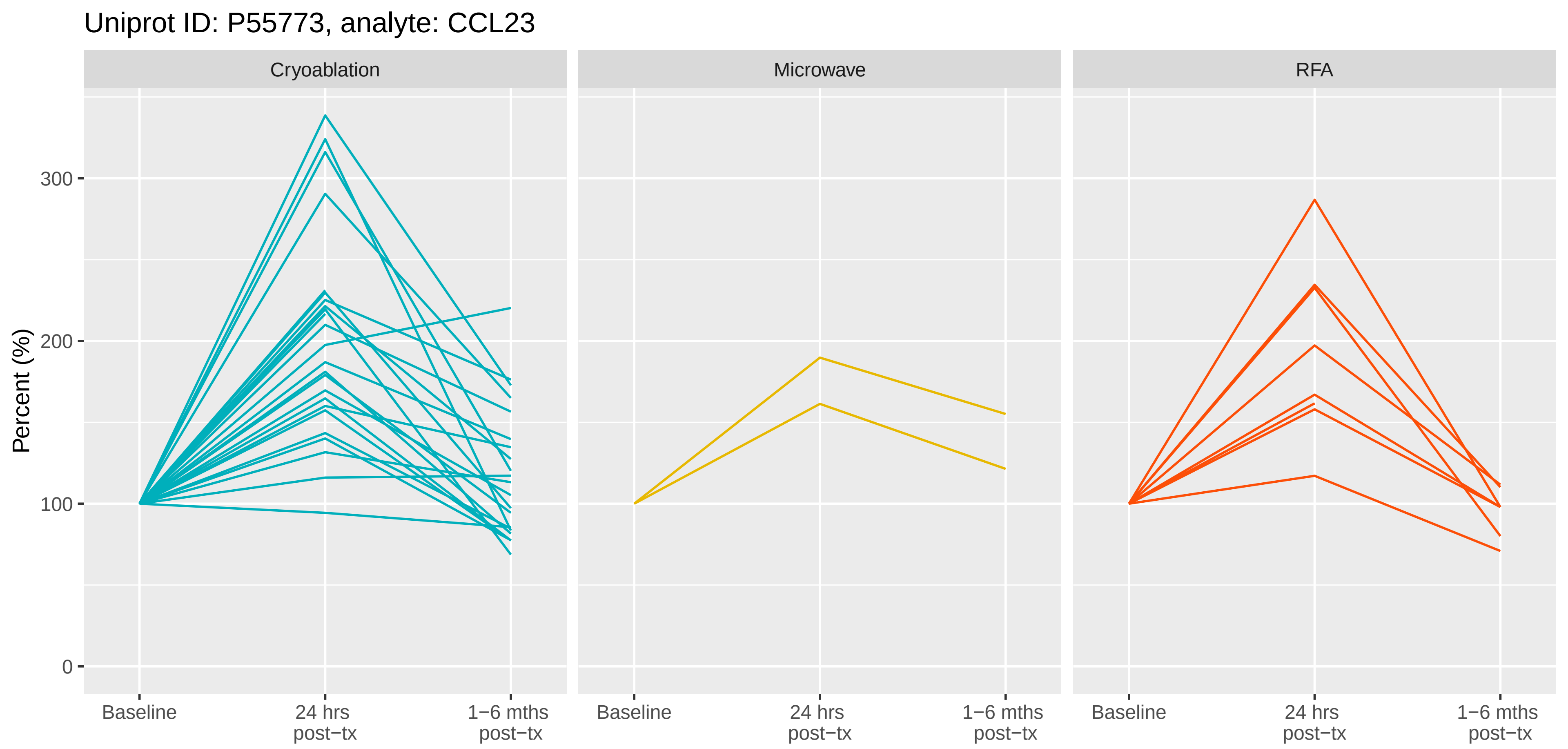
Uniprot ID: P55773, analyte: CCL23
Cryoablation
Microwave
RFA
Percent (%)
300
200
100
0
Baseline
24 hrs post−tx
1−6 mths post−tx

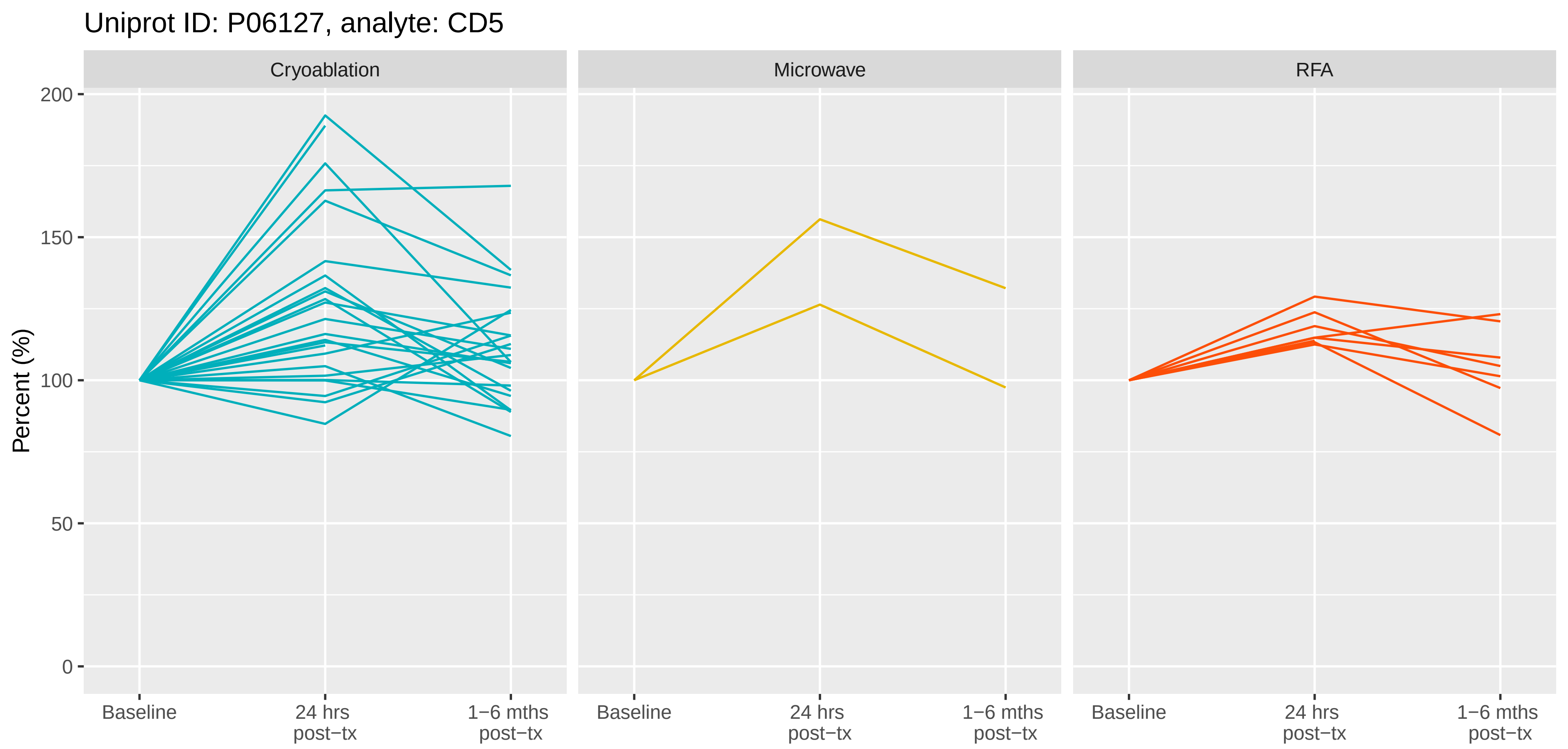

Uniprot ID: P06127, analyte: CD5
Cryoablation
Microwave
RFA
Percent (%)
200
150
100
50
0
Baseline
24 hrs post−tx
1−6 mths post−tx

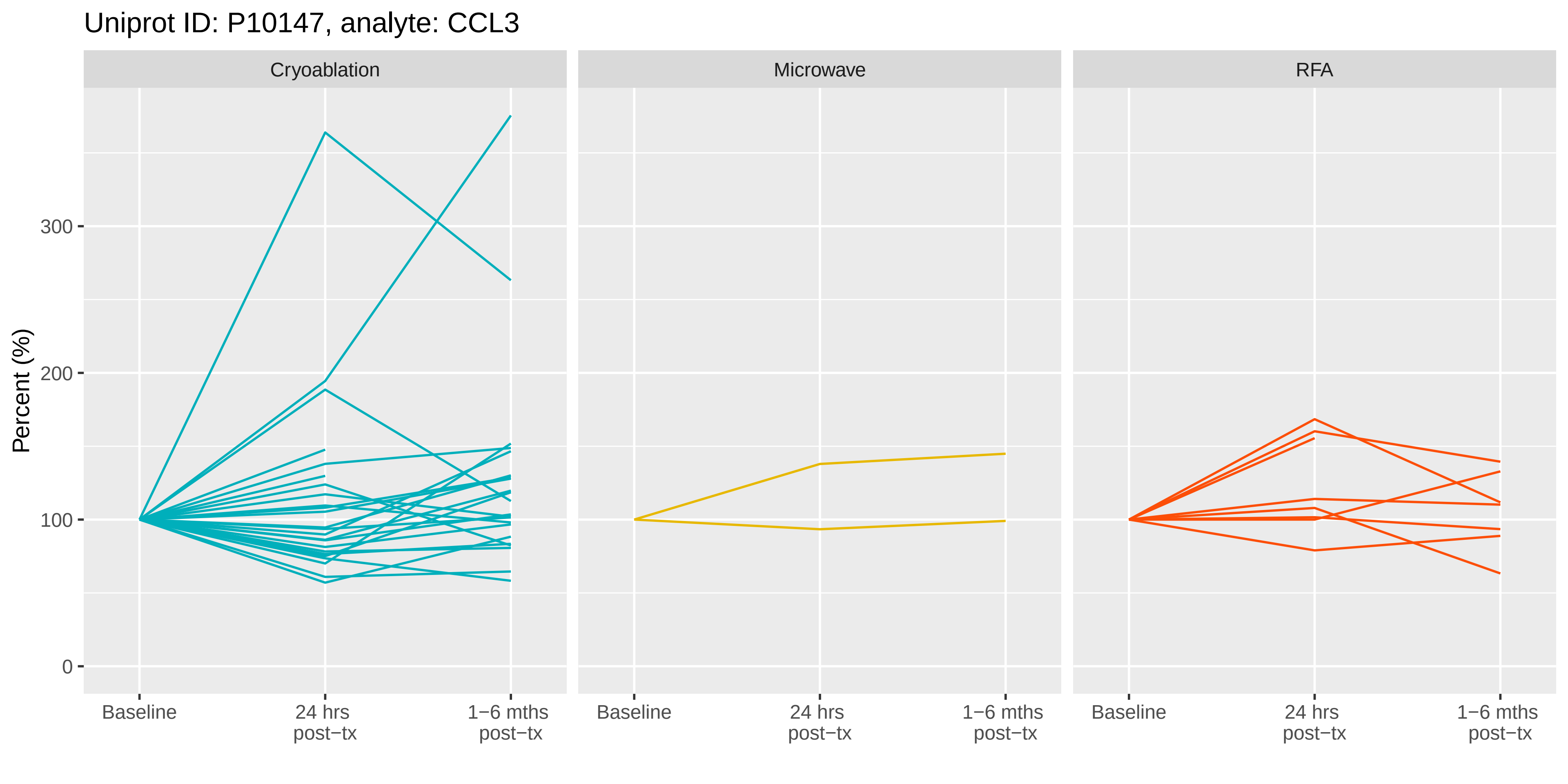

Uniprot ID: P10147, analyte: CCL3
Cryoablation
Microwave
RFA
Percent (%)
300
200
100
0
Baseline
24 hrs post−tx
1−6 mths post−tx

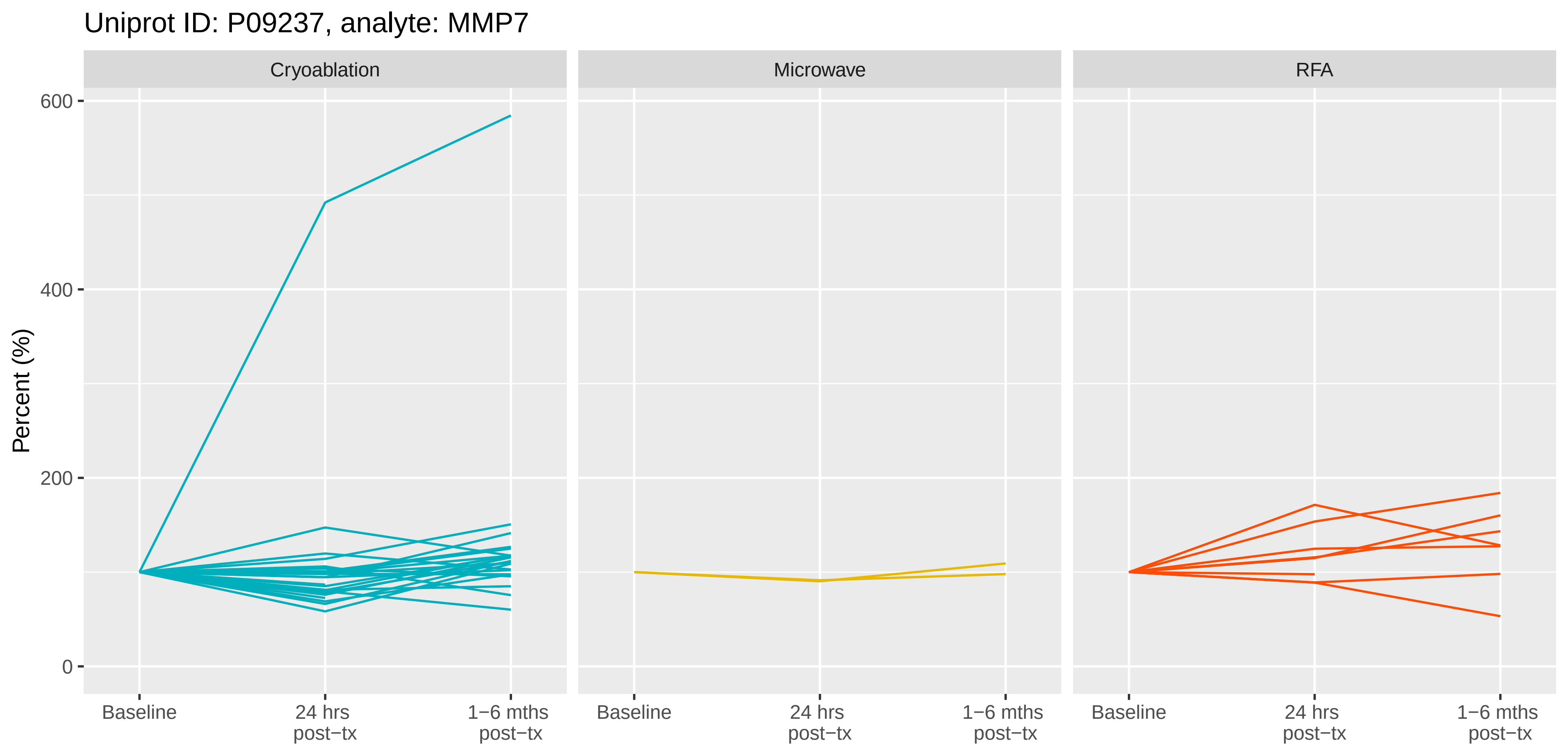
Uniprot ID: P09237, analyte: MMP7
Cryoablation
Microwave
RFA
600
400
200
0
Percent (%)
Baseline
24 hrs post−tx
1−6 mths post−tx

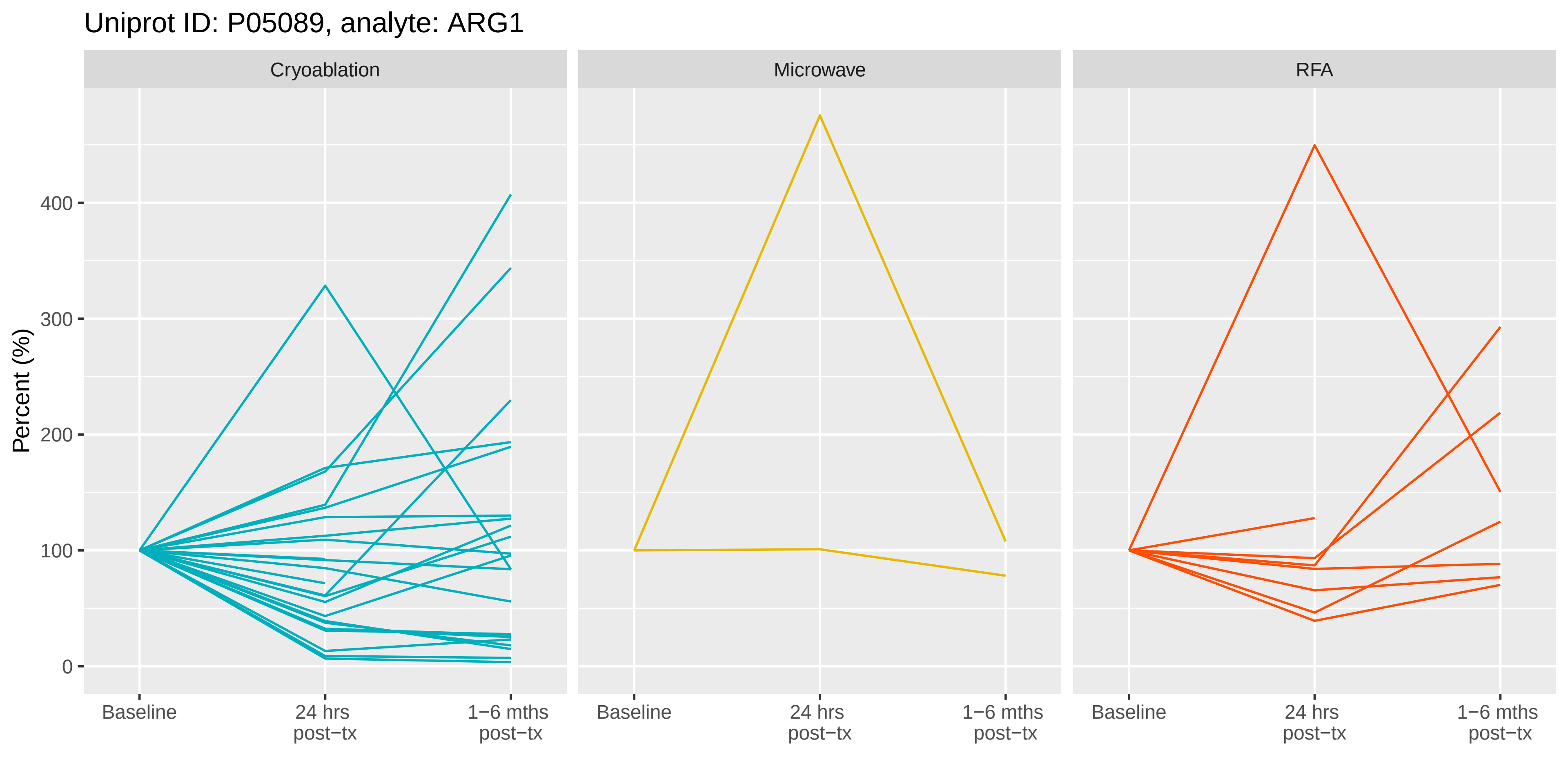

Uniprot ID: P05089, analyte: ARG1
Cryoablation
Microwave
RFA
Percent (%)
0
100
200
300
400
Baseline
24 hrs post−tx
1−6 mths post−tx

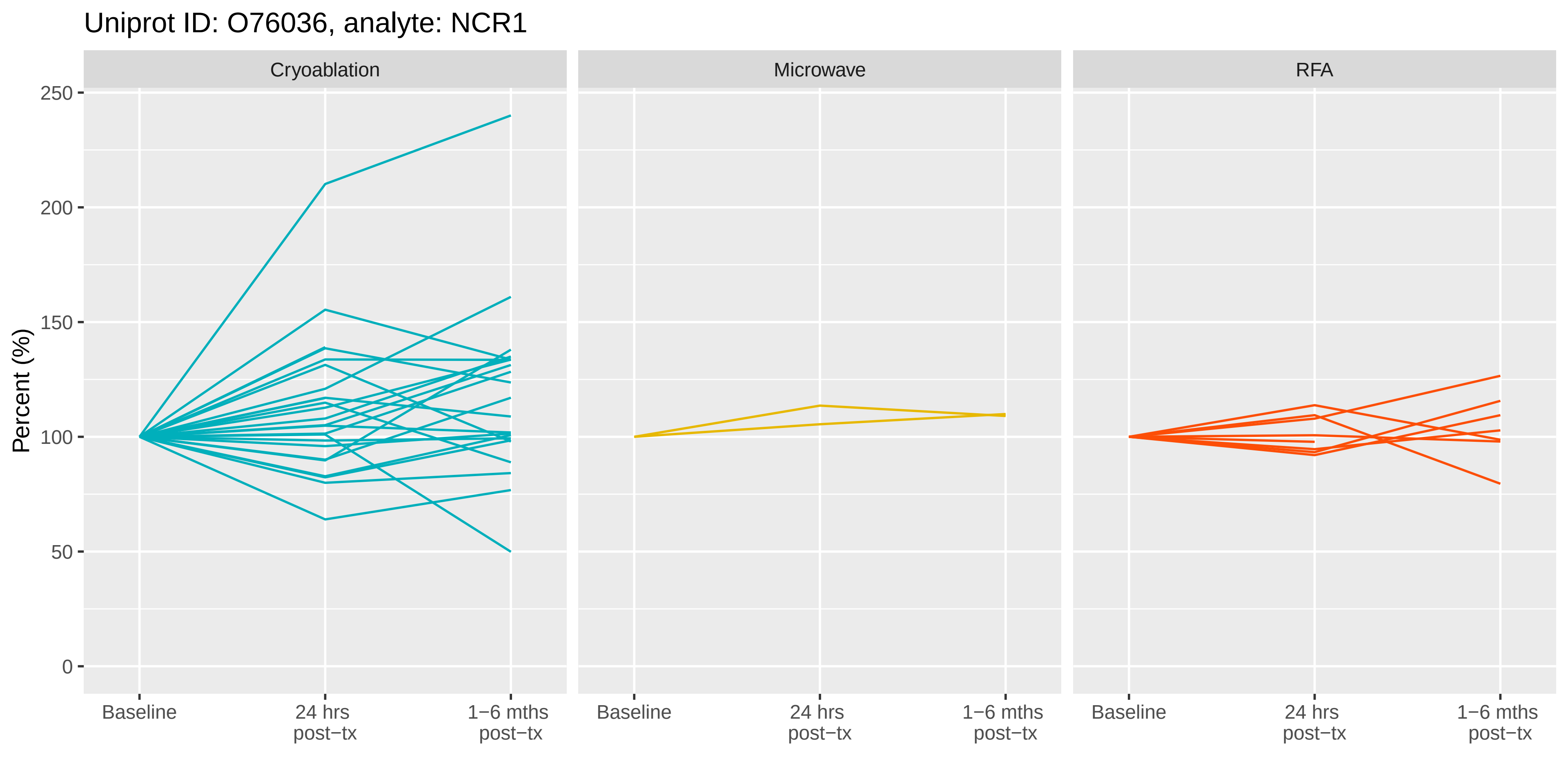

Uniprot ID: O76036, analyte: NCR1
Cryoablation
Microwave
RFA
Percent (%)
250
200
150
100
50
0
Baseline
24 hrs post−tx
1−6 mths post−tx

Uniprot ID: P07585, analyte: DCN

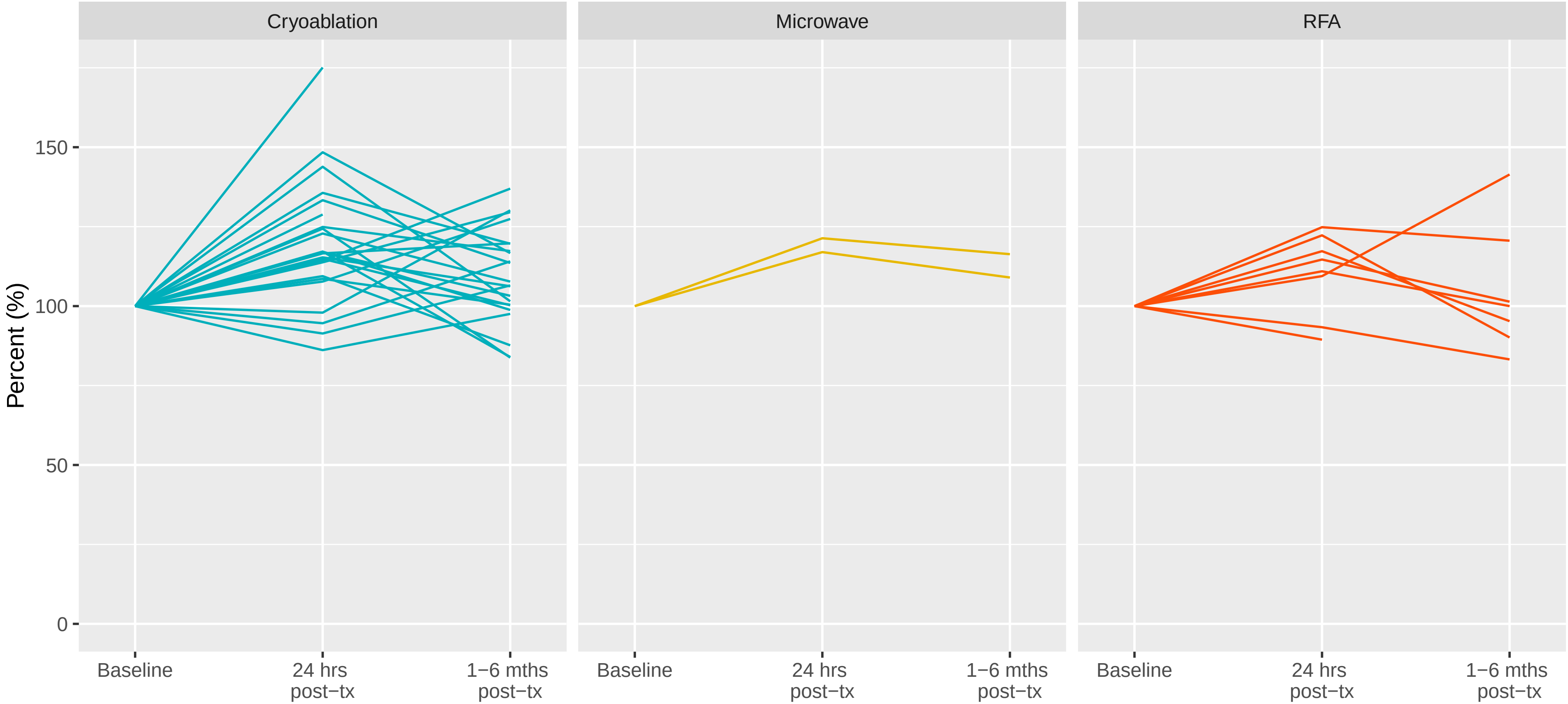

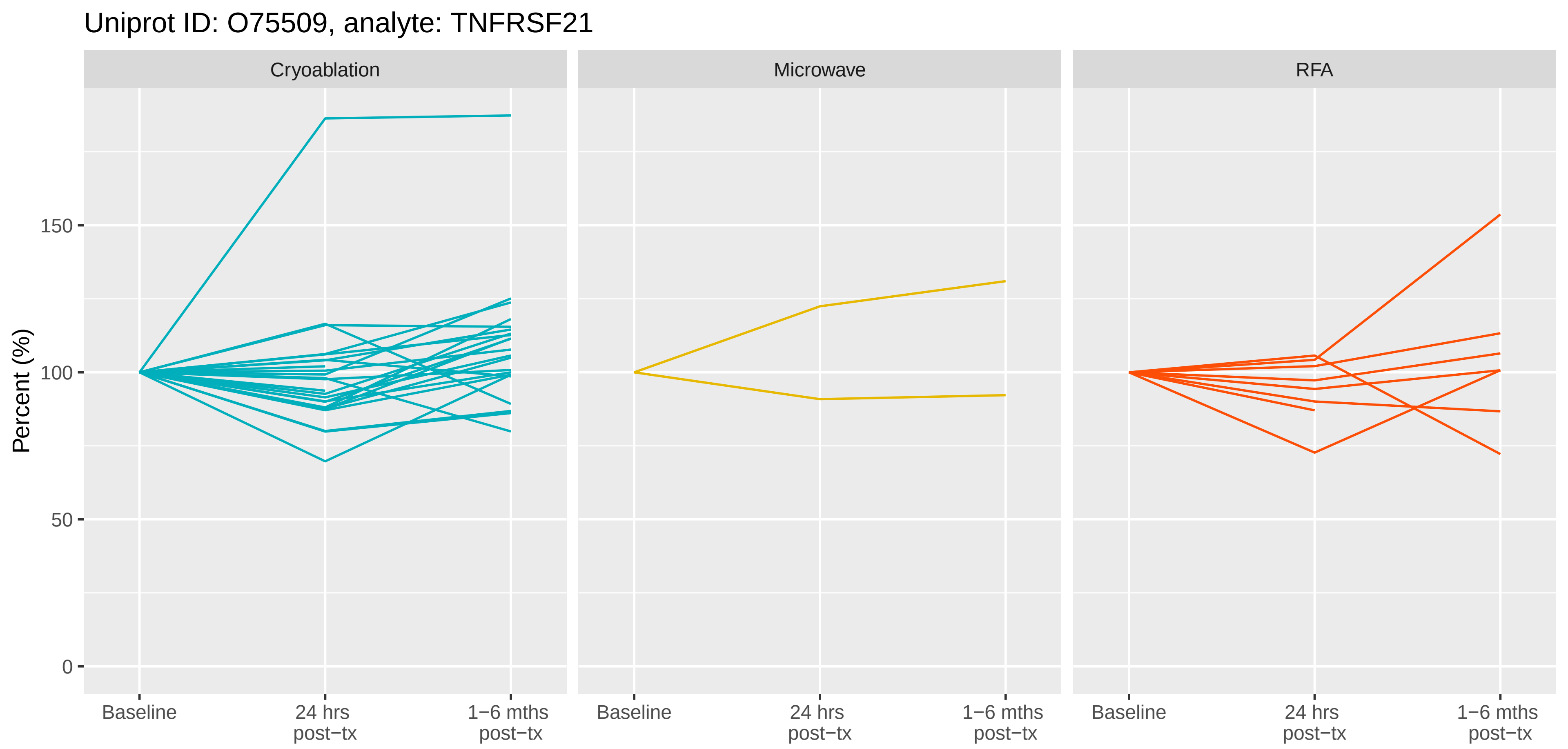

Uniprot ID: O75509, analyte: TNFRSF21
Cryoablation
Microwave
RFA
Percent (%)
150
100
50
0
Baseline
24 hrs post−tx
1−6 mths post−tx

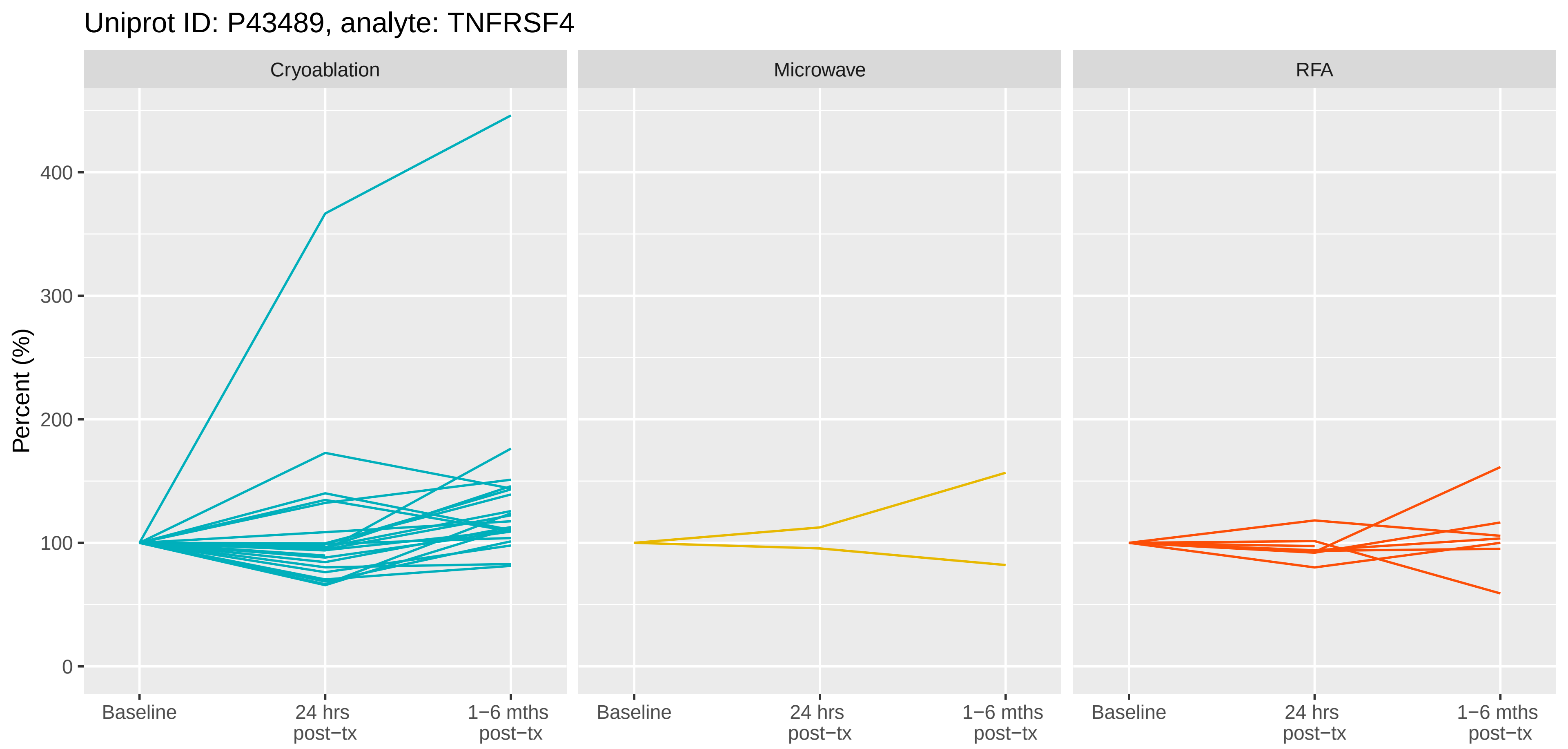
Uniprot ID: P43489, analyte: TNFRSF4
Cryoablation
Microwave
RFA
Percent (%)
0
100
200
300
400
Baseline
24 hrs post−tx
1−6 mths post−tx

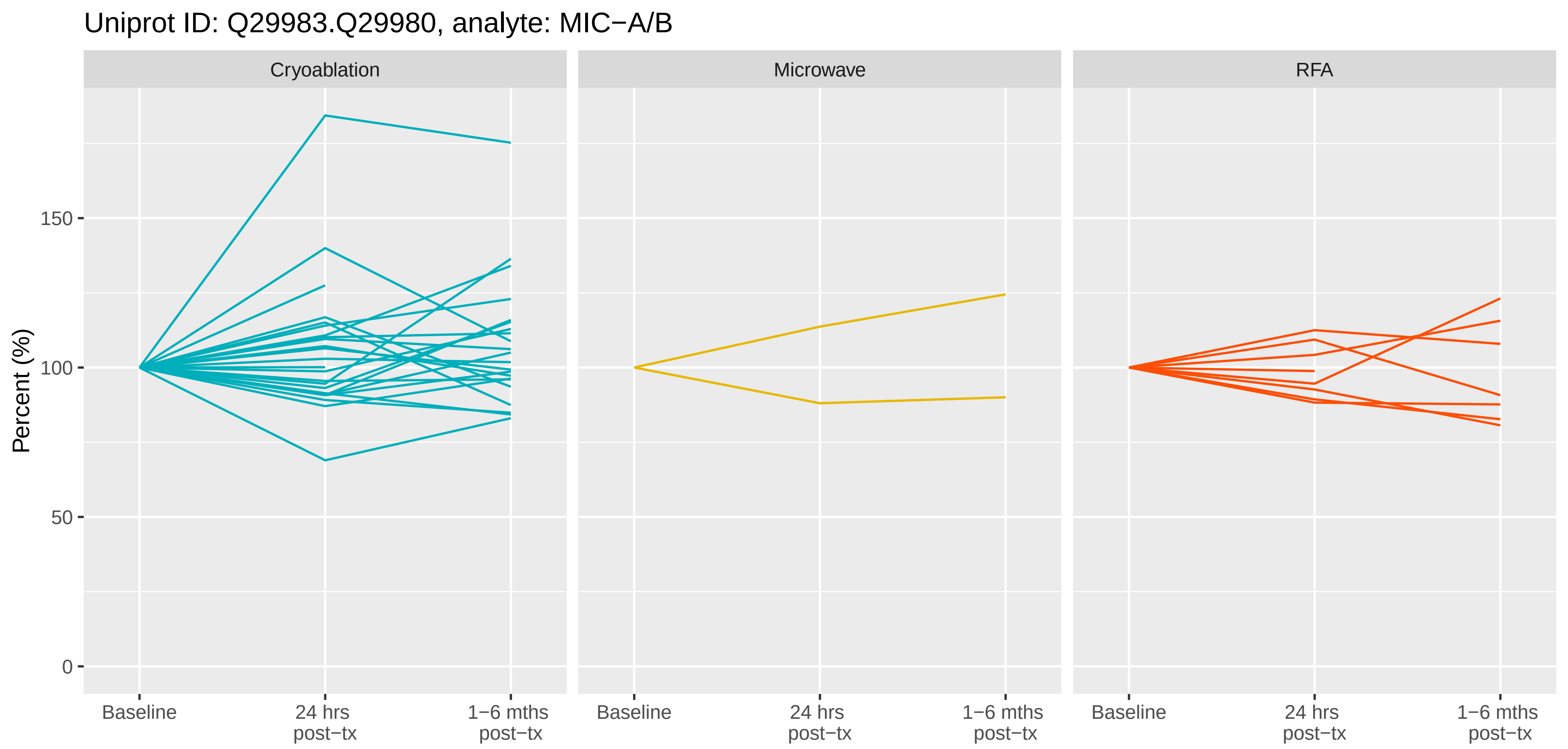

Uniprot ID: Q29983.Q29980, analyte: MIC−A/B
Cryoablation
Microwave
RFA
Percent (%)
150
100
50
0
Baseline
24 hrs post−tx
1−6 mths post−tx

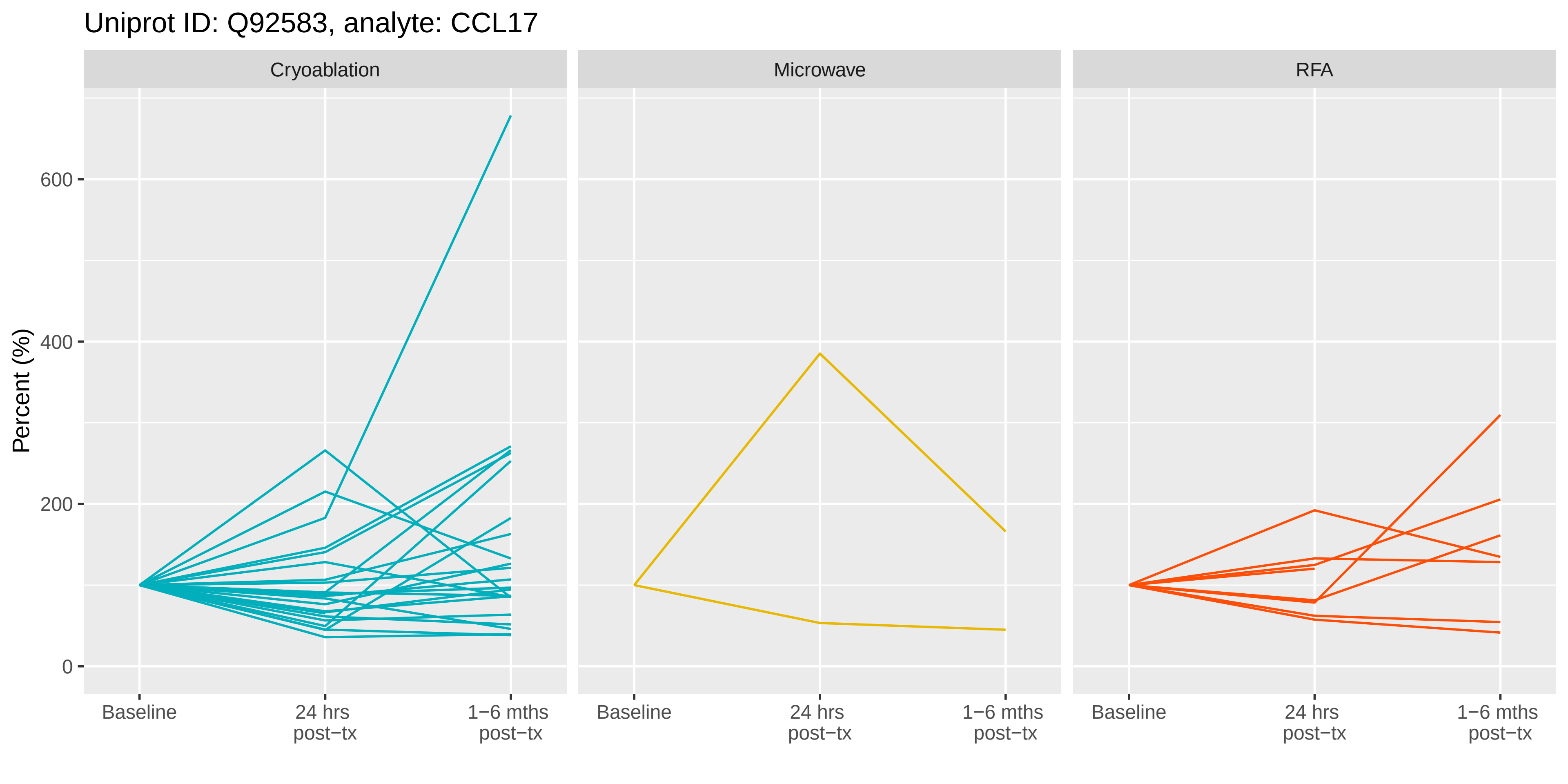

Uniprot ID: Q92583, analyte: CCL17
Cryoablation
Microwave
RFA
Percent (%)
600
400
200
0
Baseline
24 hrs post−tx
1−6 mths post−tx

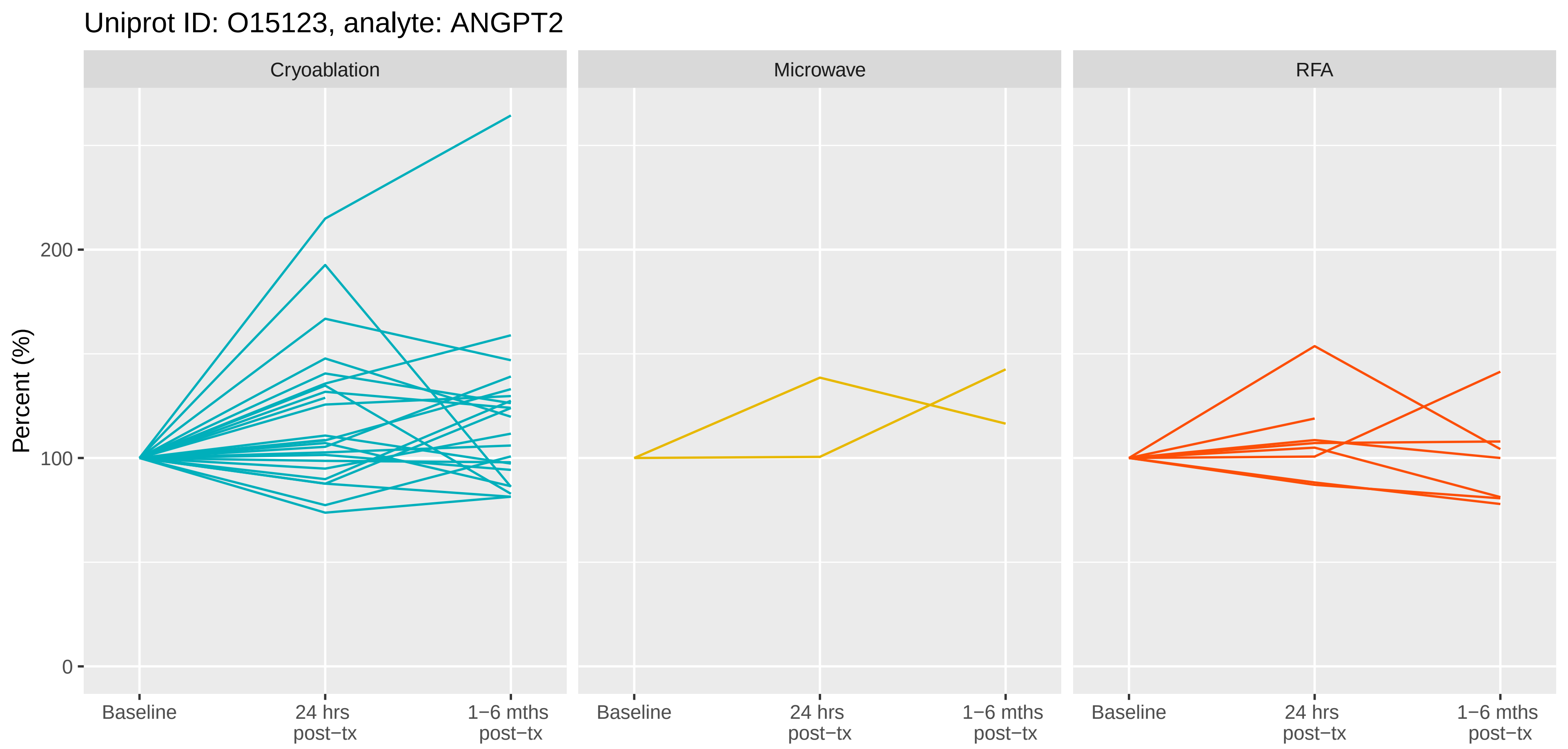
Uniprot ID: O15123, analyte: ANGPT2
Cryoablation
Microwave
RFA
Percent (%)
200
100
0
Baseline
24 hrs post−tx
1−6 mths post−tx
Baseline
24 hrs post−tx
1−6 mths post−tx
Baseline
24 hrs post−tx
1−6 mths post−tx

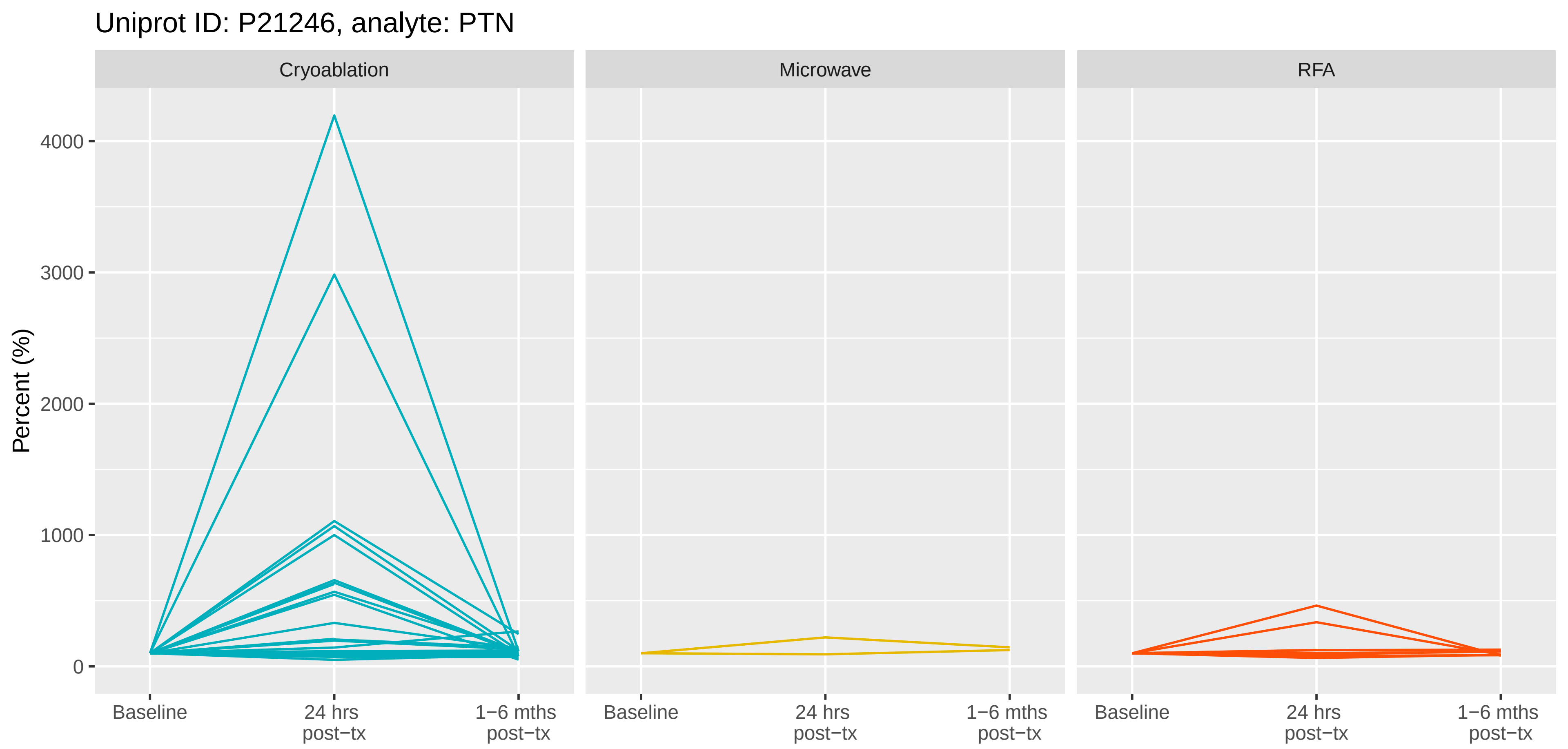
Uniprot ID: P21246, analyte: PTN
Cryoablation
Microwave
RFA
Percent (%)
4000
3000
2000
1000
0
Baseline
24 hrs post−tx
1−6 mths post−tx

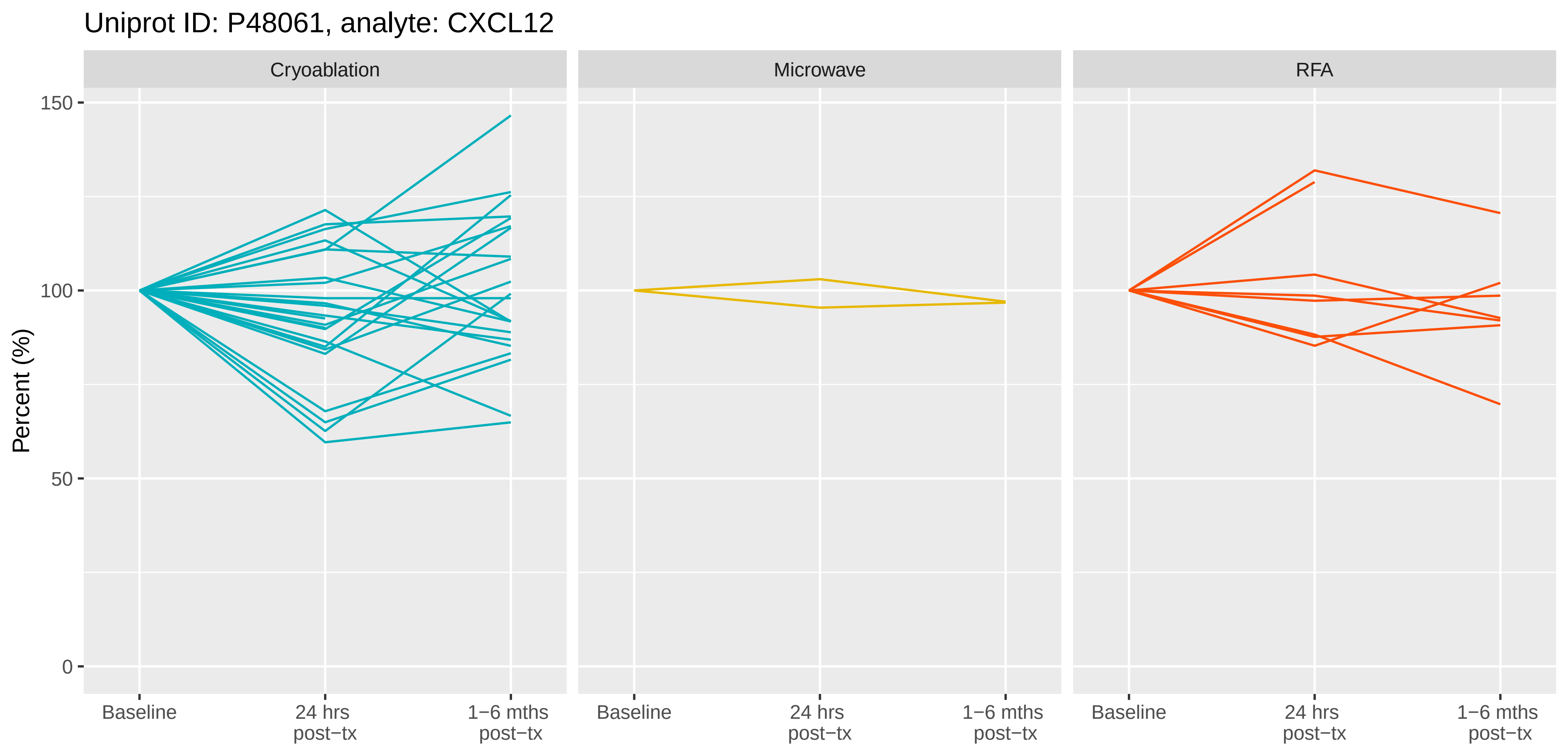

Uniprot ID: P48061, analyte: CXCL12
Cryoablation
Microwave
RFA
Percent (%)
150
100
50
0
Baseline
24 hrs post−tx
1−6 mths post−tx

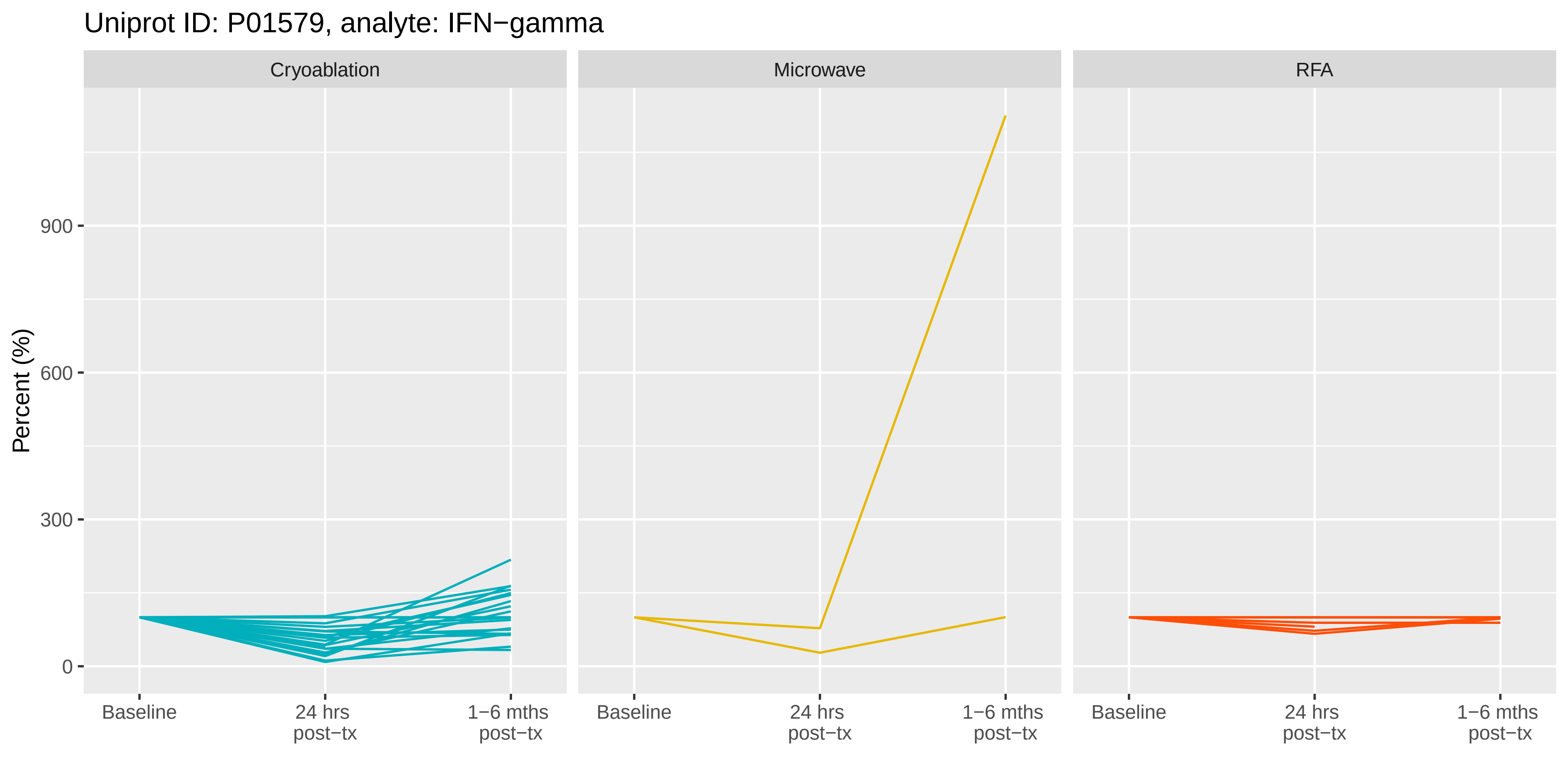

Uniprot ID: P01579, analyte: IFN−gamma
Cryoablation
Microwave
RFA
Percent (%)
900
600
300
0
Baseline
24 hrs post−tx
1−6 mths post−tx

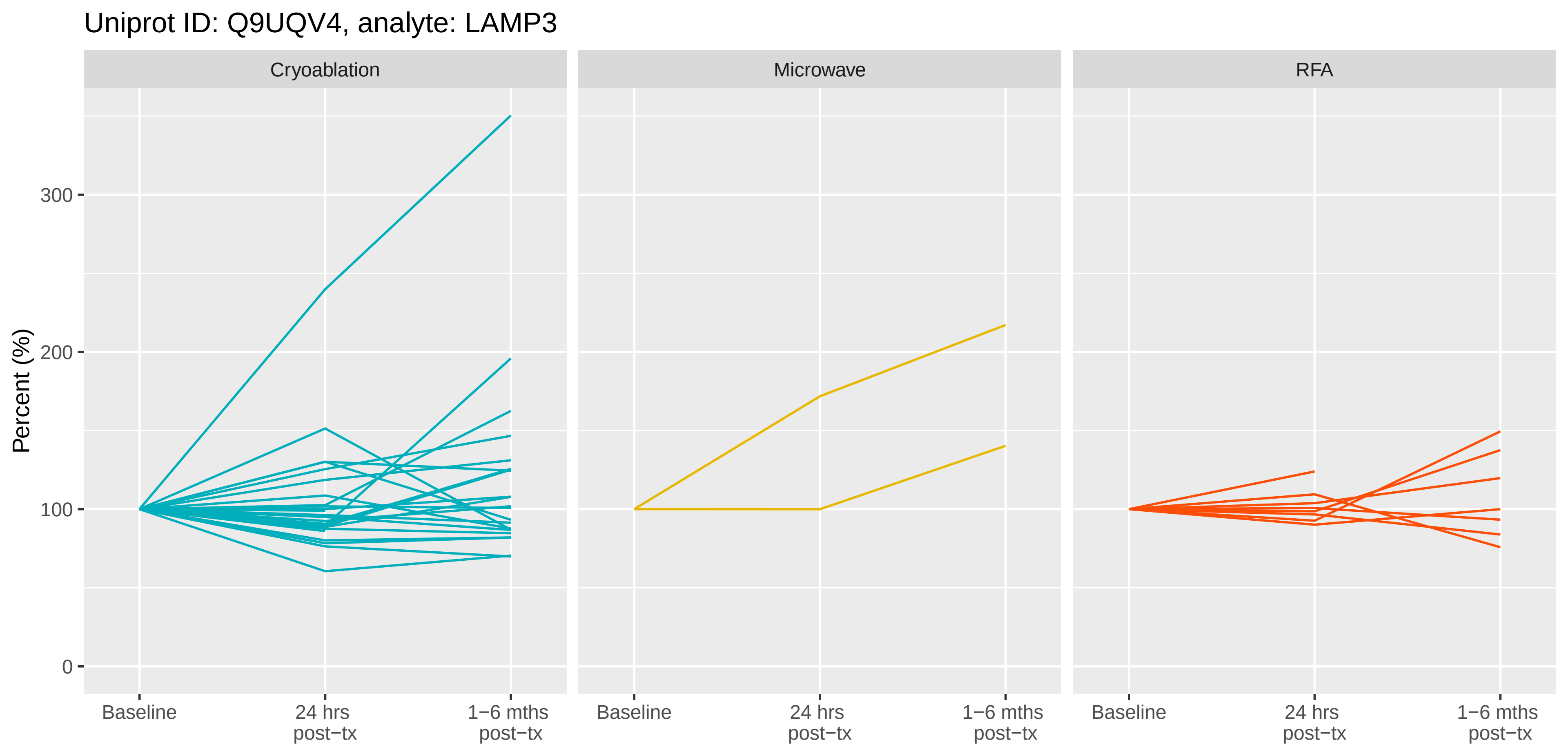

Uniprot ID: Q9UQV4, analyte: LAMP3
Cryoablation
Microwave
RFA
Percent (%)
300
200
100
0
Baseline
24 hrs post−tx
1−6 mths post−tx

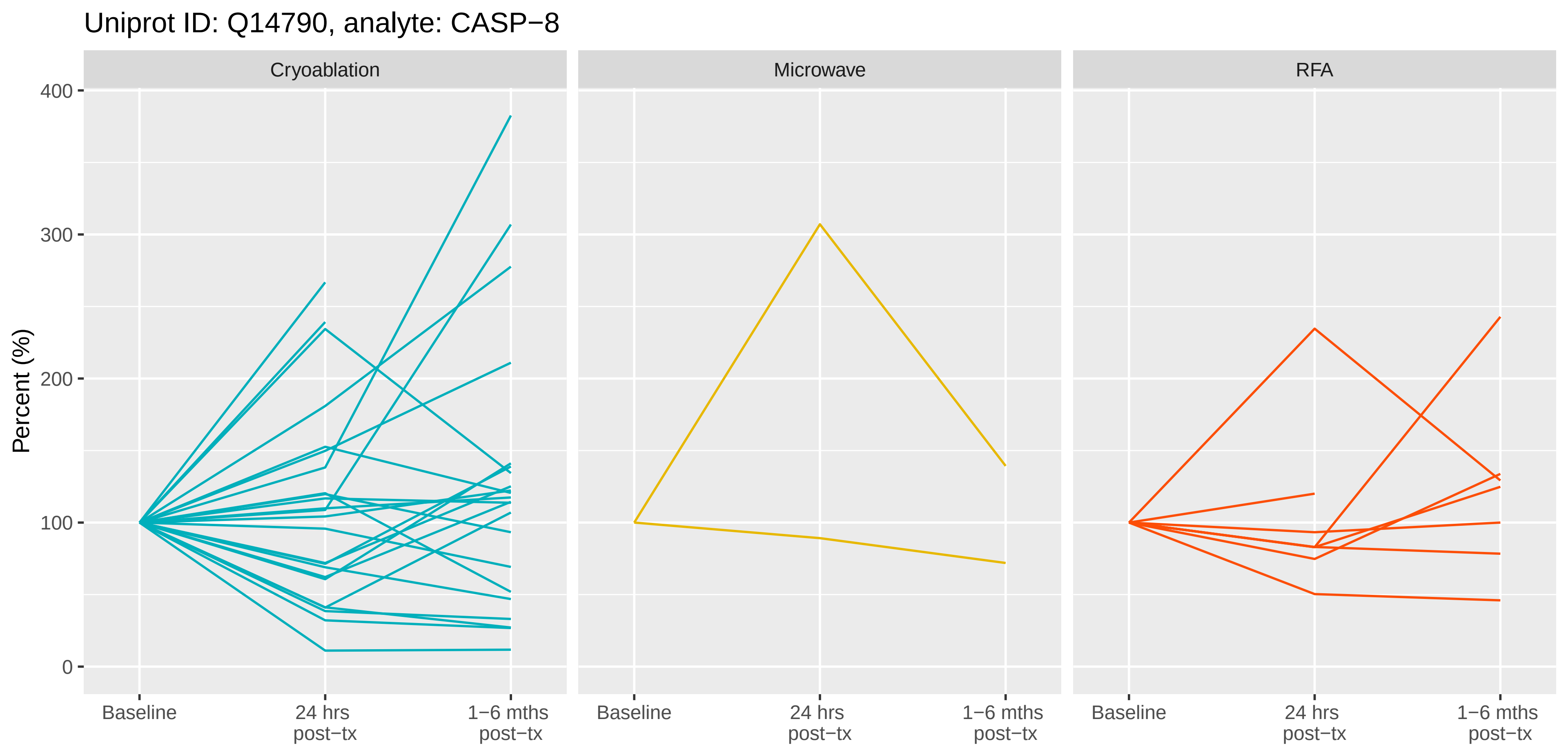
Uniprot ID: Q14790, analyte: CASP−8
Cryoablation
Microwave
RFA
Percent (%)
0
100
200
300
400
Baseline
24 hrs post−tx
1−6 mths post−tx

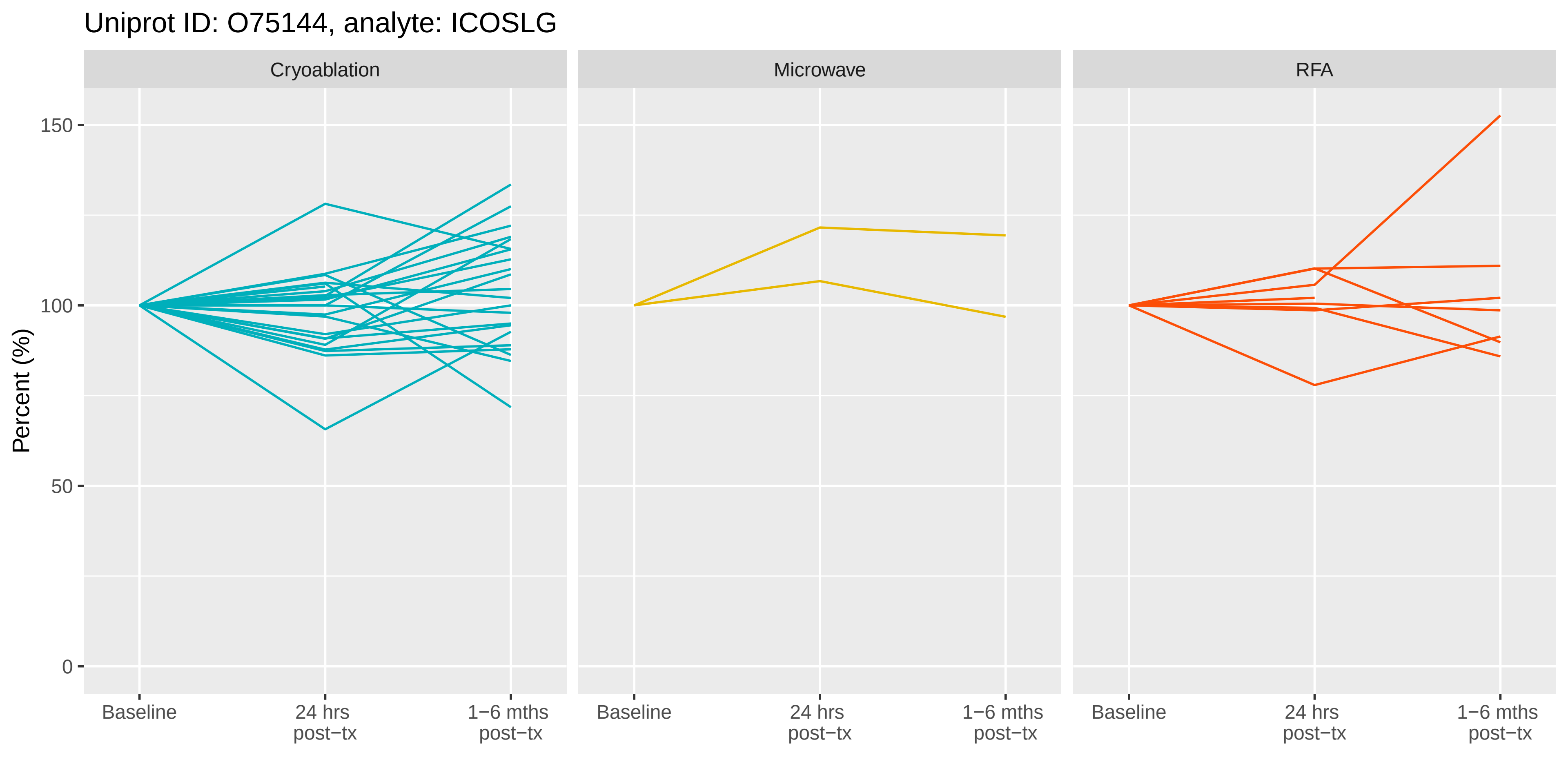
Uniprot ID: O75144, analyte: ICOSLG
Cryoablation
Microwave
RFA
Percent (%)
150
100
50
0
Baseline
24 hrs post−tx
1−6 mths post−tx

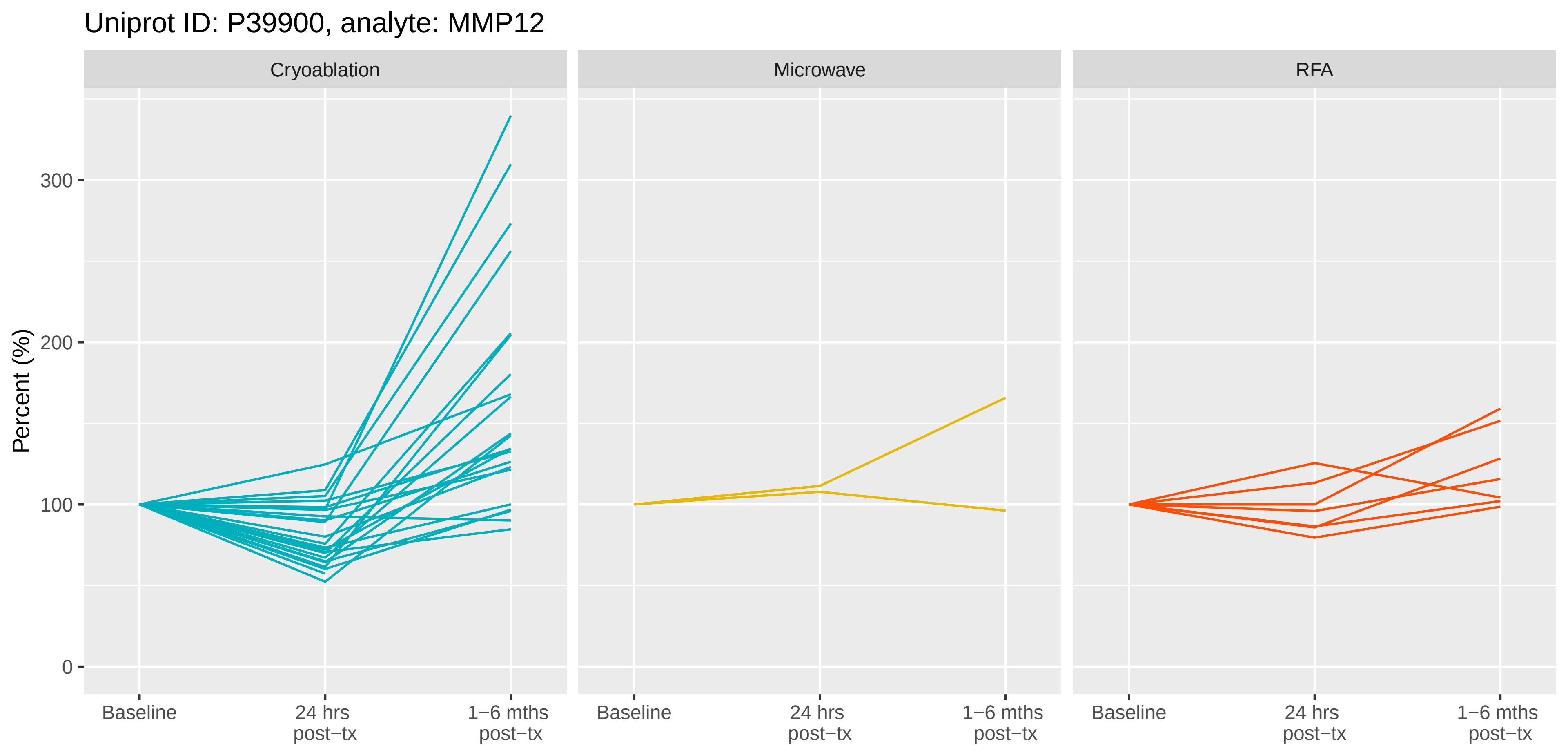

Uniprot ID: P39900, analyte: MMP12
Cryoablation
Microwave
RFA
Percent (%)
0
100
200
300
Baseline
24 hrs post−tx
1−6 mths post−tx

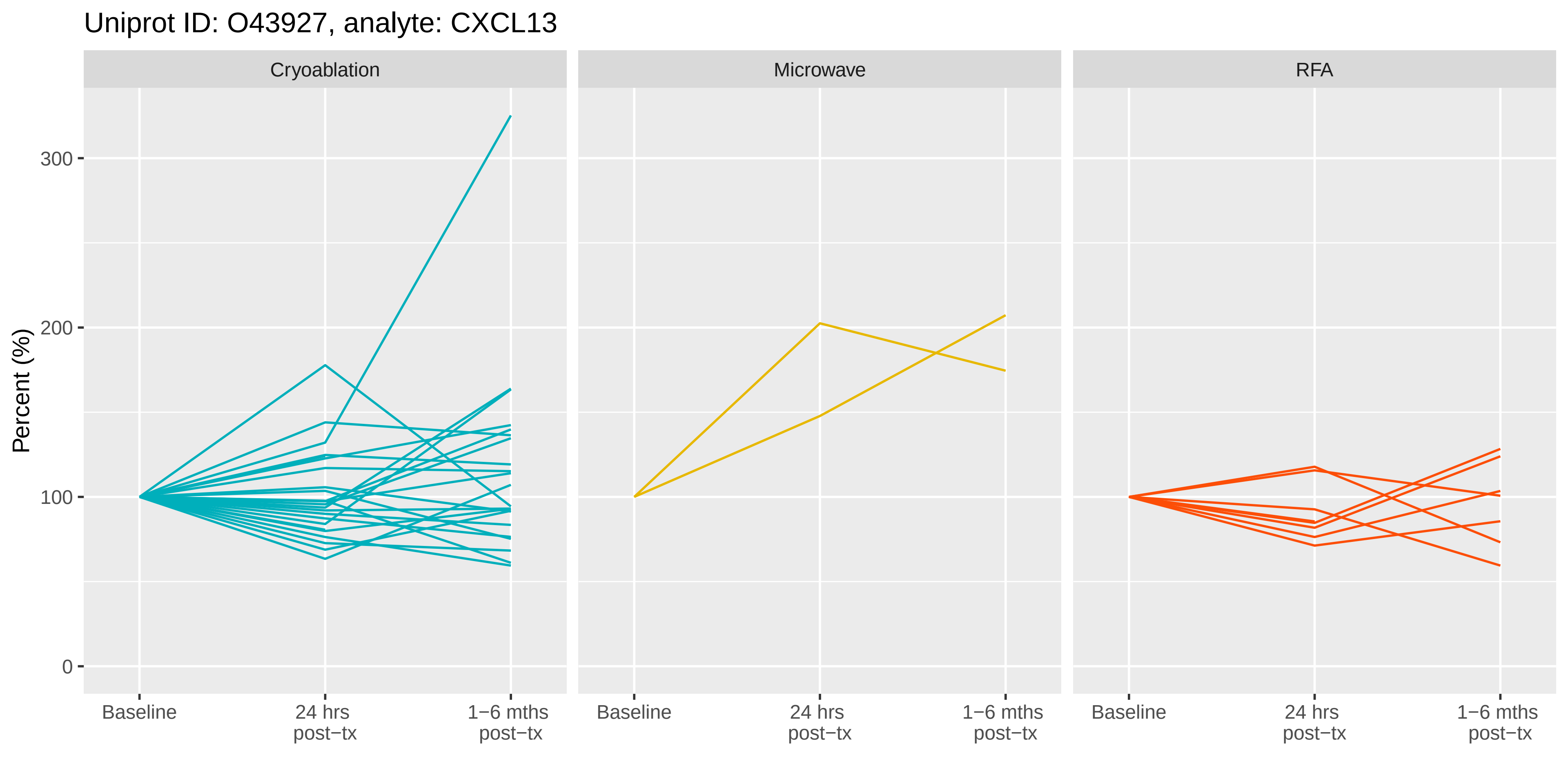
Uniprot ID: O43927, analyte: CXCL13
Cryoablation
Microwave
RFA
Percent (%)
300
200
100
0
Baseline
24 hrs post−tx
1−6 mths post−tx

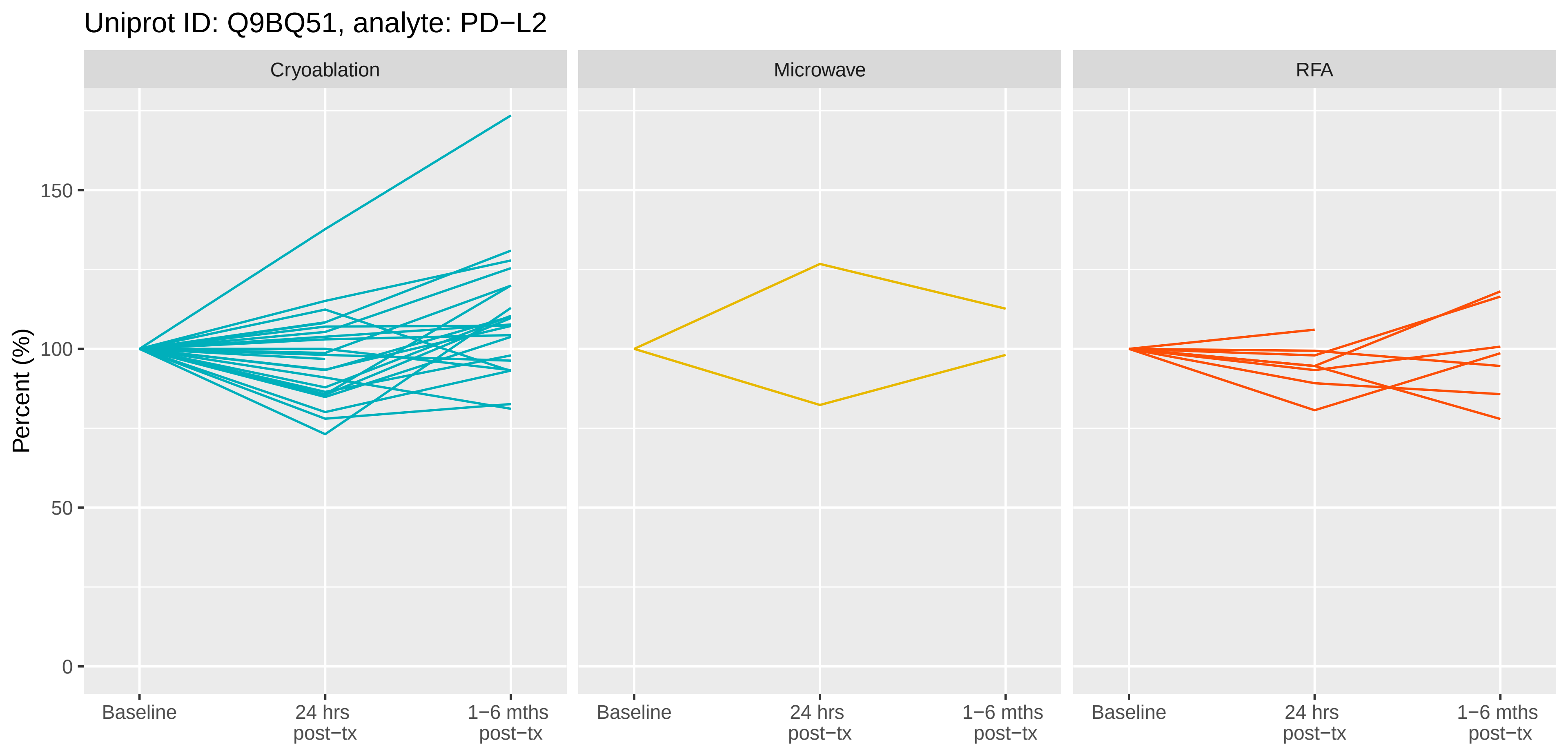
Uniprot ID: Q9BQ51, analyte: PD−L2
Cryoablation
Microwave
RFA
Percent (%)
150
100
50
0
Baseline
24 hrs post−tx
1−6 mths post−tx

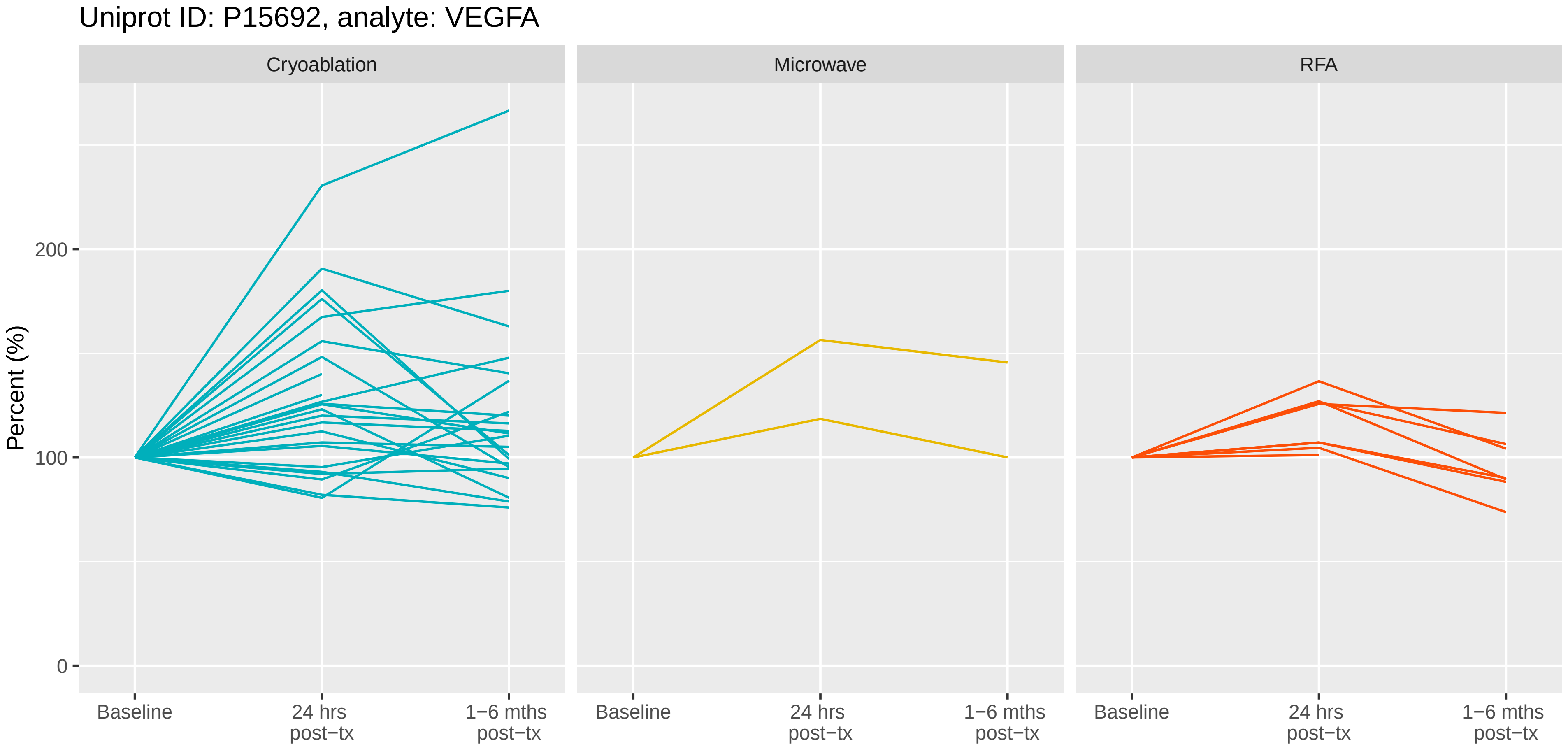

Uniprot ID: P15692, analyte: VEGFA
Cryoablation
Microwave
RFA
Percent (%)
200
100
0
Baseline
24 hrs post−tx
1−6 mths post−tx

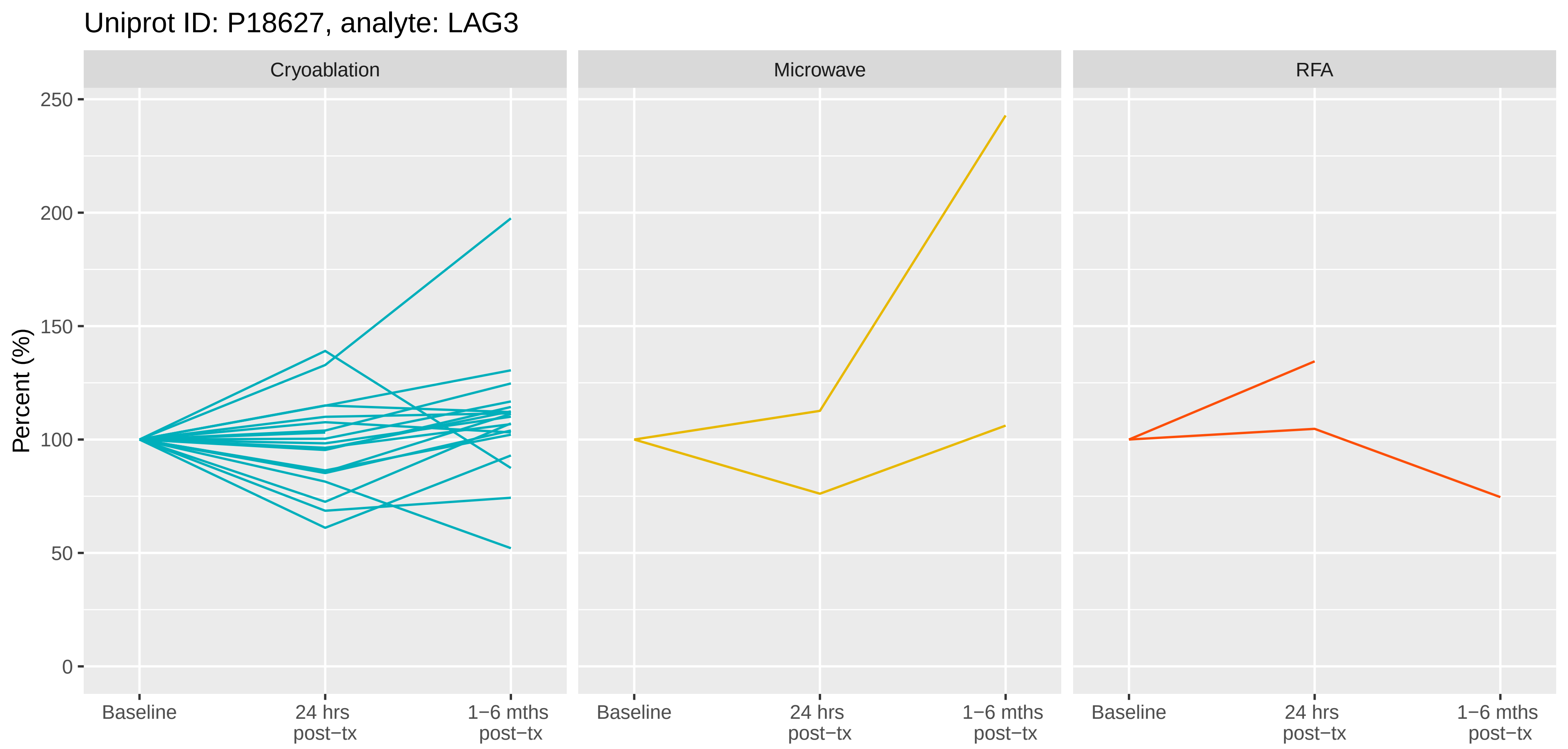
Uniprot ID: P18627, analyte: LAG3
Cryoablation
Microwave
RFA
Percent (%)
250
200
150
100
50
0
Baseline
24 hrs post−tx
1−6 mths post−tx

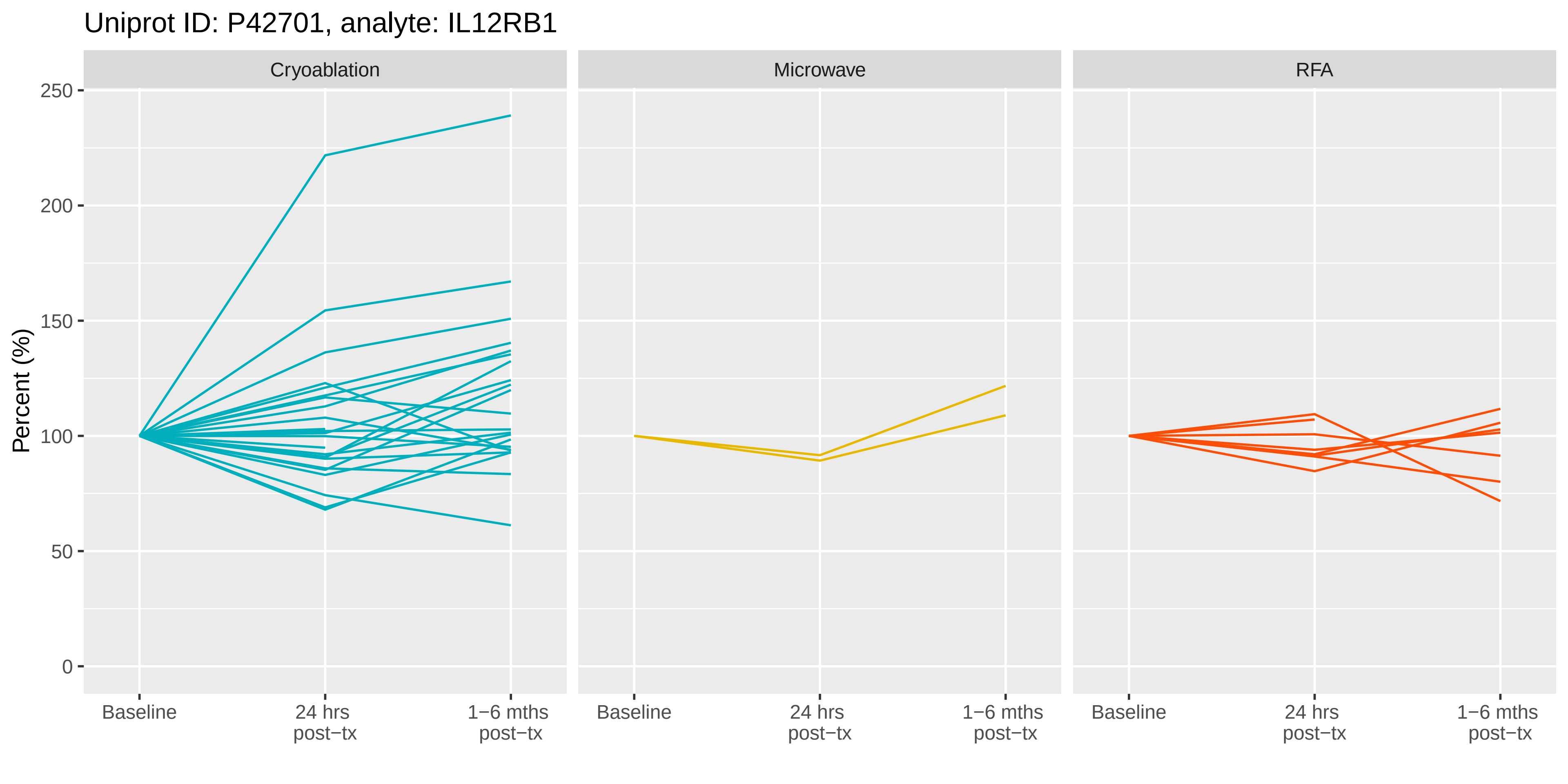
Uniprot ID: P42701, analyte: IL12RB1
Cryoablation
Microwave
RFA
Percent (%)
250
200
150
100
50
0
Baseline
24 hrs post−tx
1−6 mths post−tx
Baseline
24 hrs post−tx
1−6 mths post−tx
Baseline
24 hrs post−tx
1−6 mths post−tx

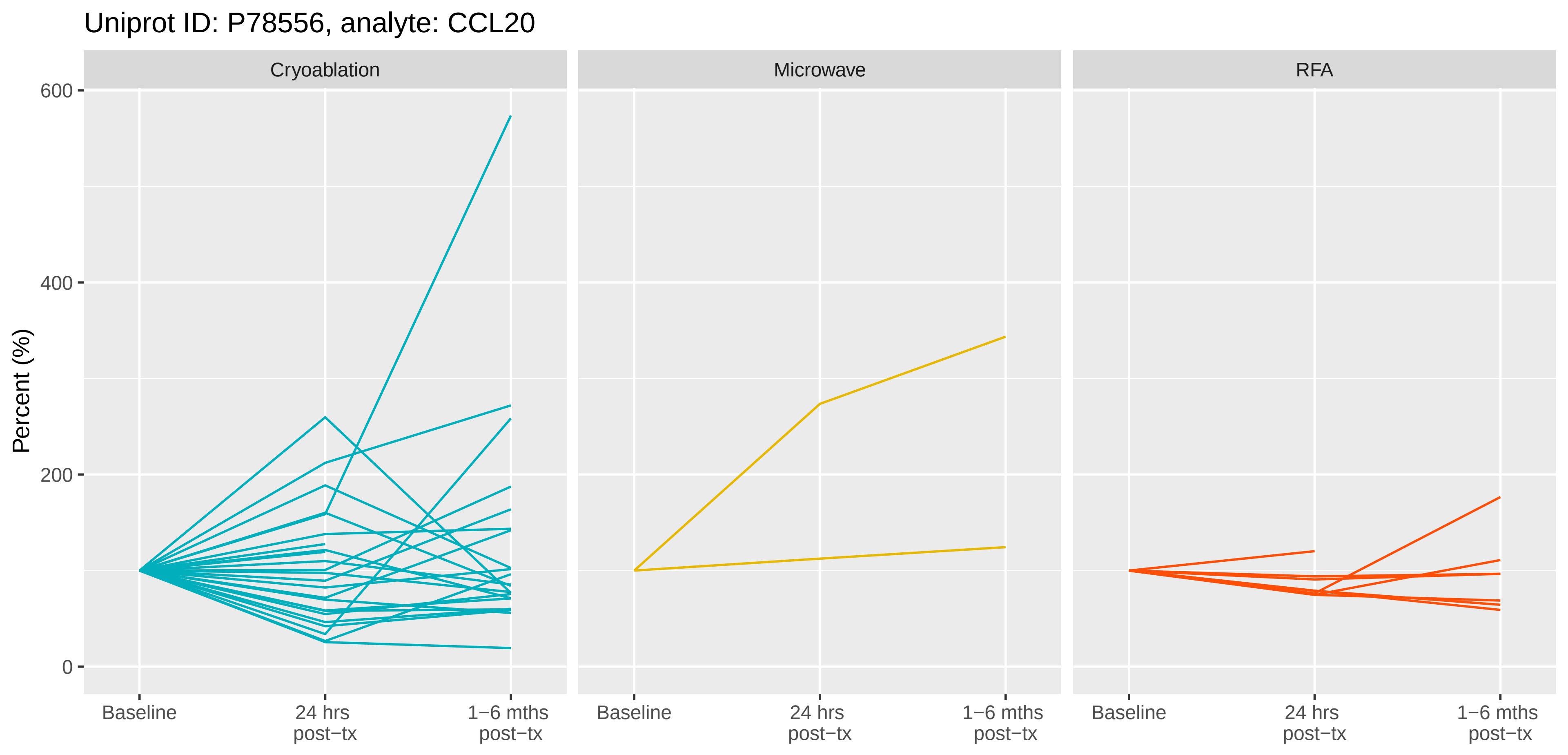

Uniprot ID: P78556, analyte: CCL20
Cryoablation
Microwave
RFA
Percent (%)
600
400
200
0
Baseline
24 hrs post−tx
1−6 mths post−tx

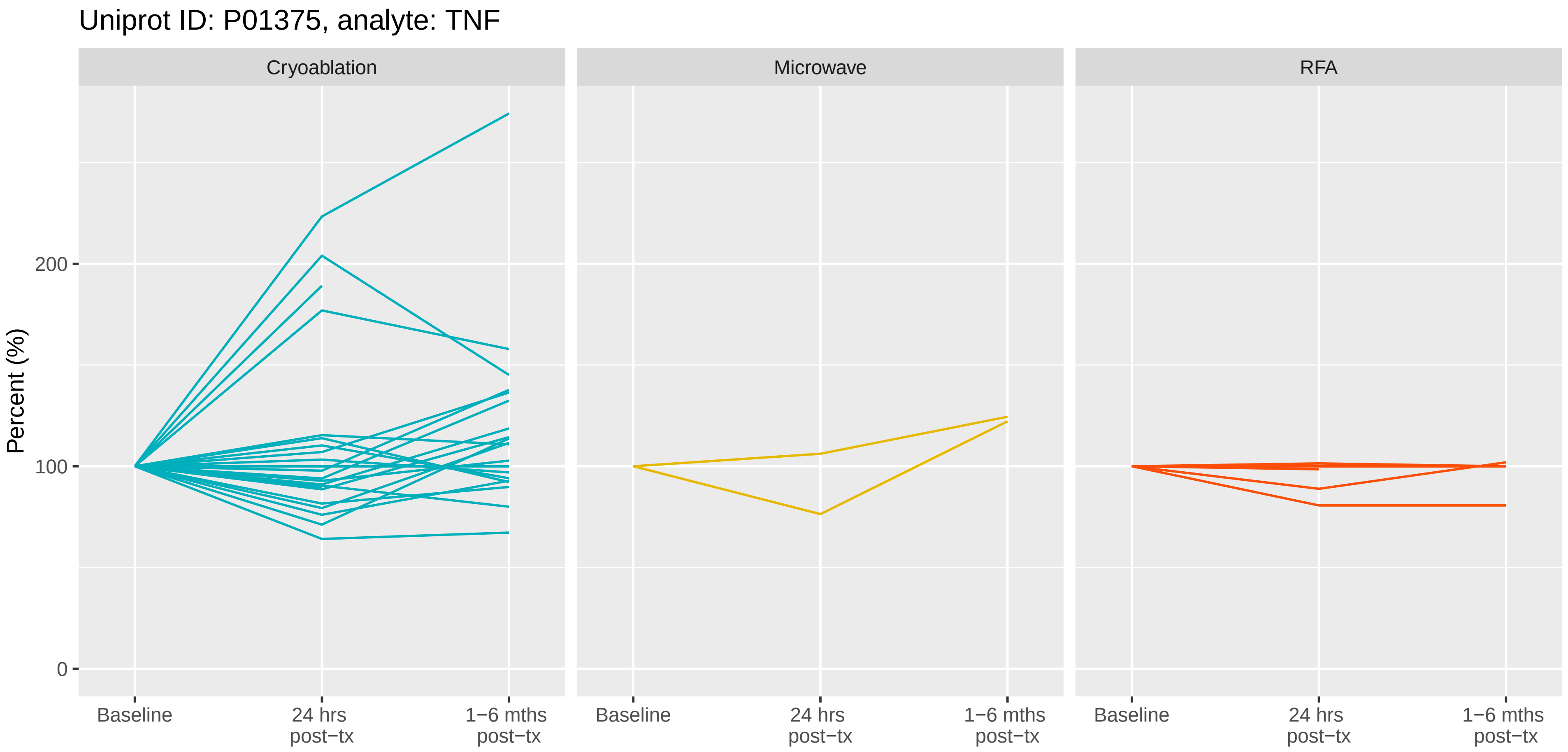

Uniprot ID: P01375, analyte: TNF
Cryoablation
Microwave
RFA
Percent (%)
0
100
200
Baseline
24 hrs post−tx
1−6 mths post−tx

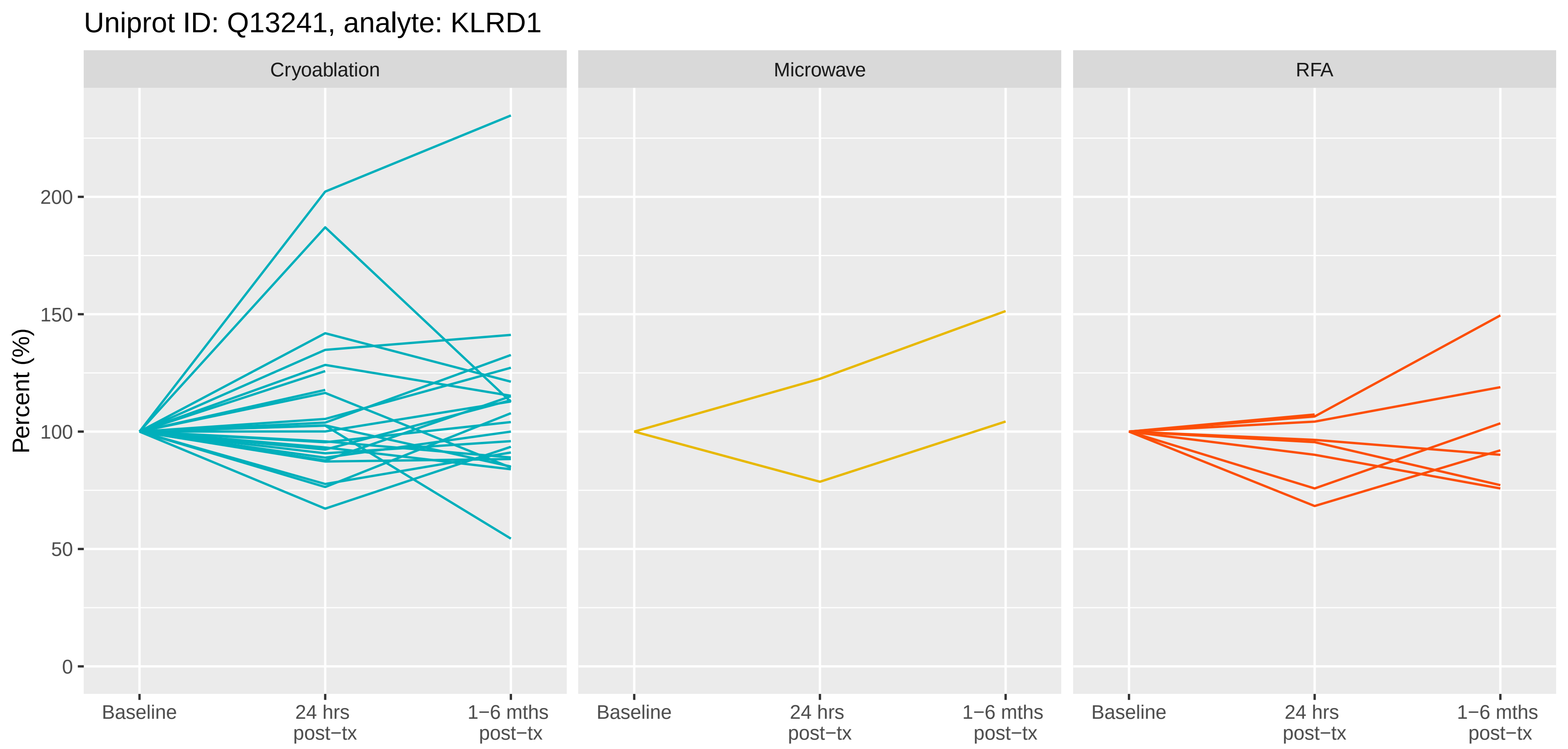

Uniprot ID: Q13241, analyte: KLRD1
Cryoablation
Microwave
RFA
Percent (%)
0
50
100
150
200
Baseline
24 hrs post−tx
1−6 mths post−tx

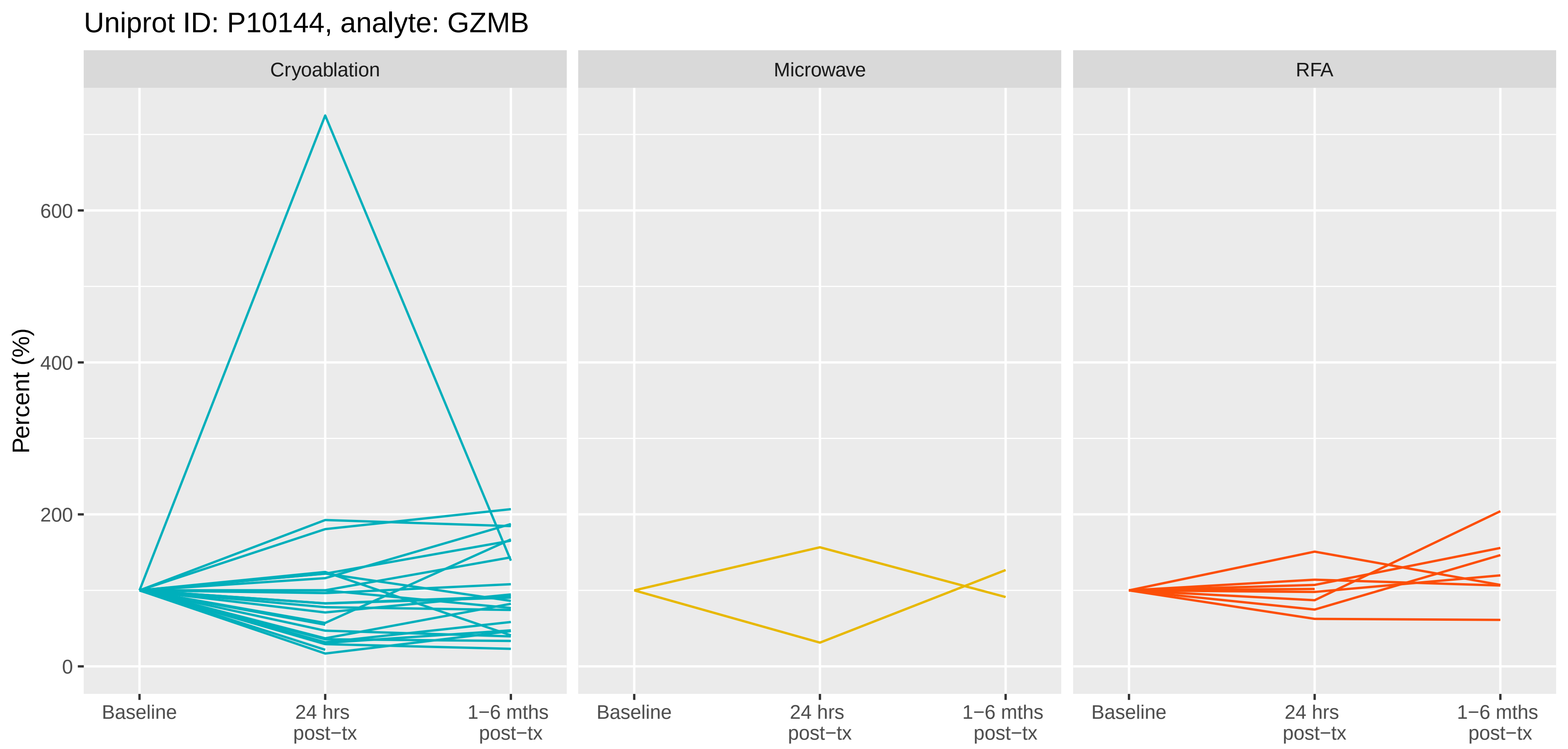

Uniprot ID: P10144, analyte: GZMB
Cryoablation
Microwave
RFA
Percent (%)
600
400
200
0
Baseline
24 hrs post−tx
1−6 mths post−tx

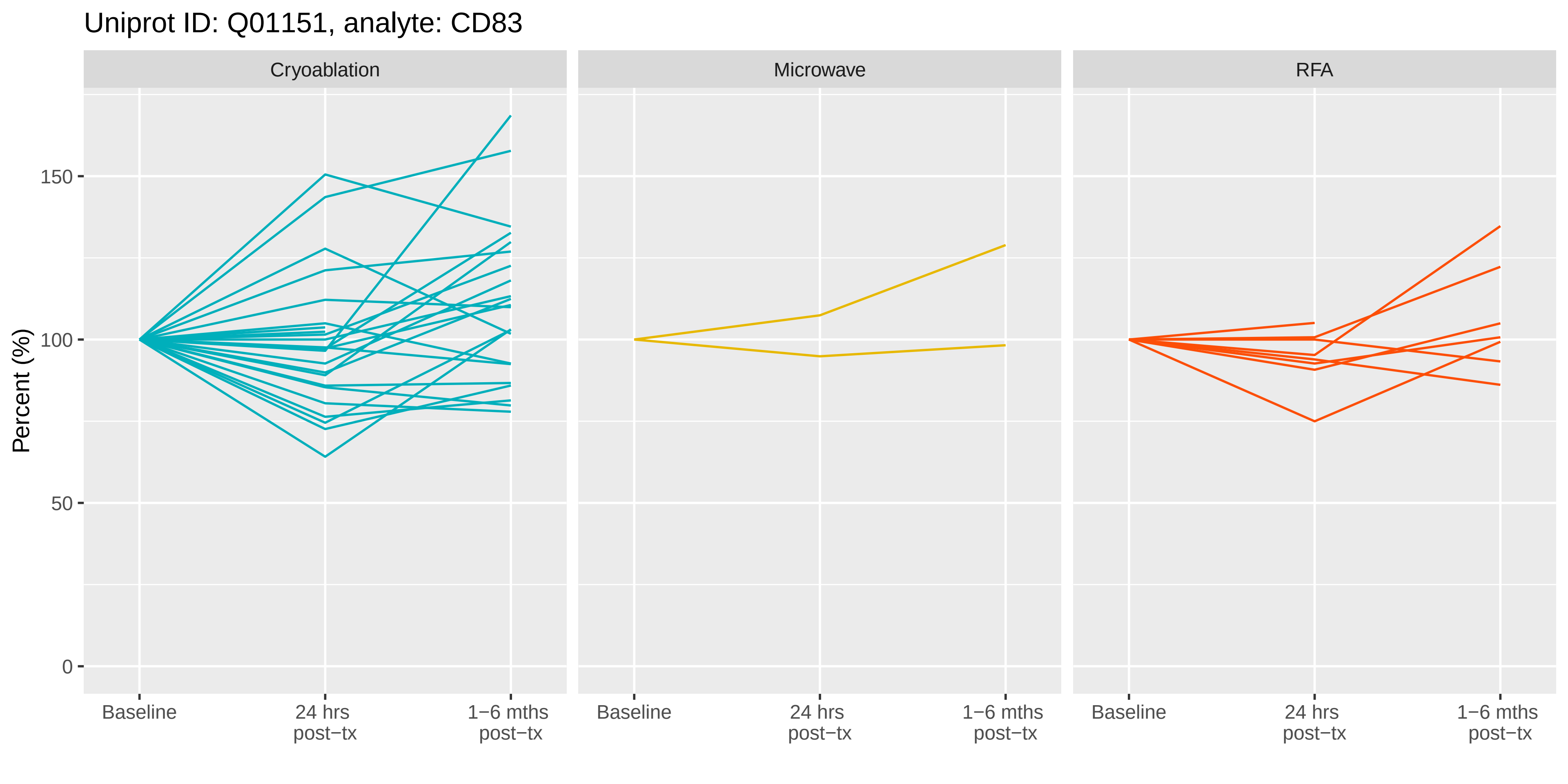

Uniprot ID: Q01151, analyte: CD83
Cryoablation
Microwave
RFA
Percent (%)
150
100
50
0
Baseline
24 hrs post−tx
1−6 mths post−tx
Baseline
24 hrs post−tx
1−6 mths post−tx
Baseline
24 hrs post−tx
1−6 mths post−tx

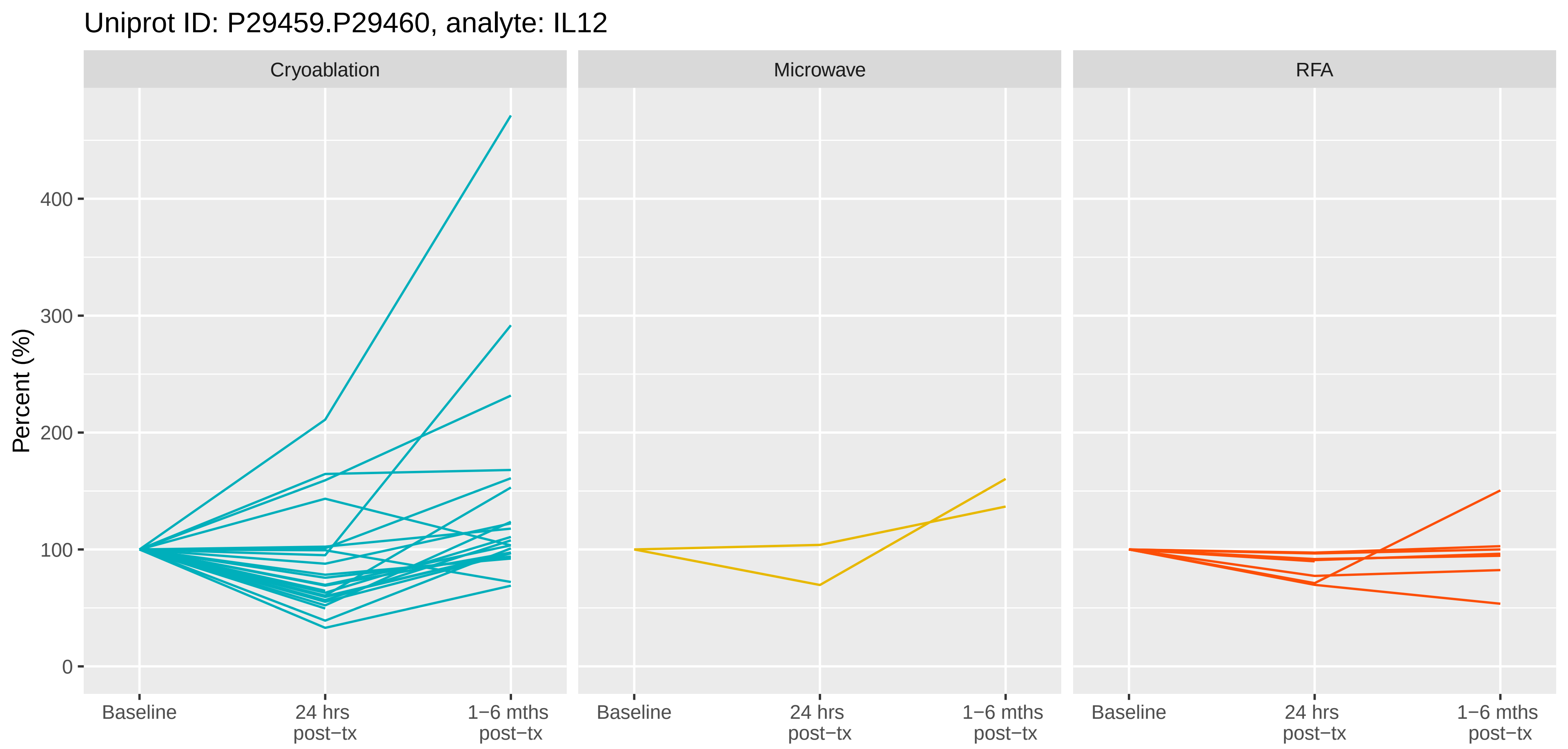

Uniprot ID: P29459.P29460, analyte: IL12
Cryoablation
Microwave
RFA
Percent (%)
0
100
200
300
400
Baseline
24 hrs post−tx
1−6 mths post−tx

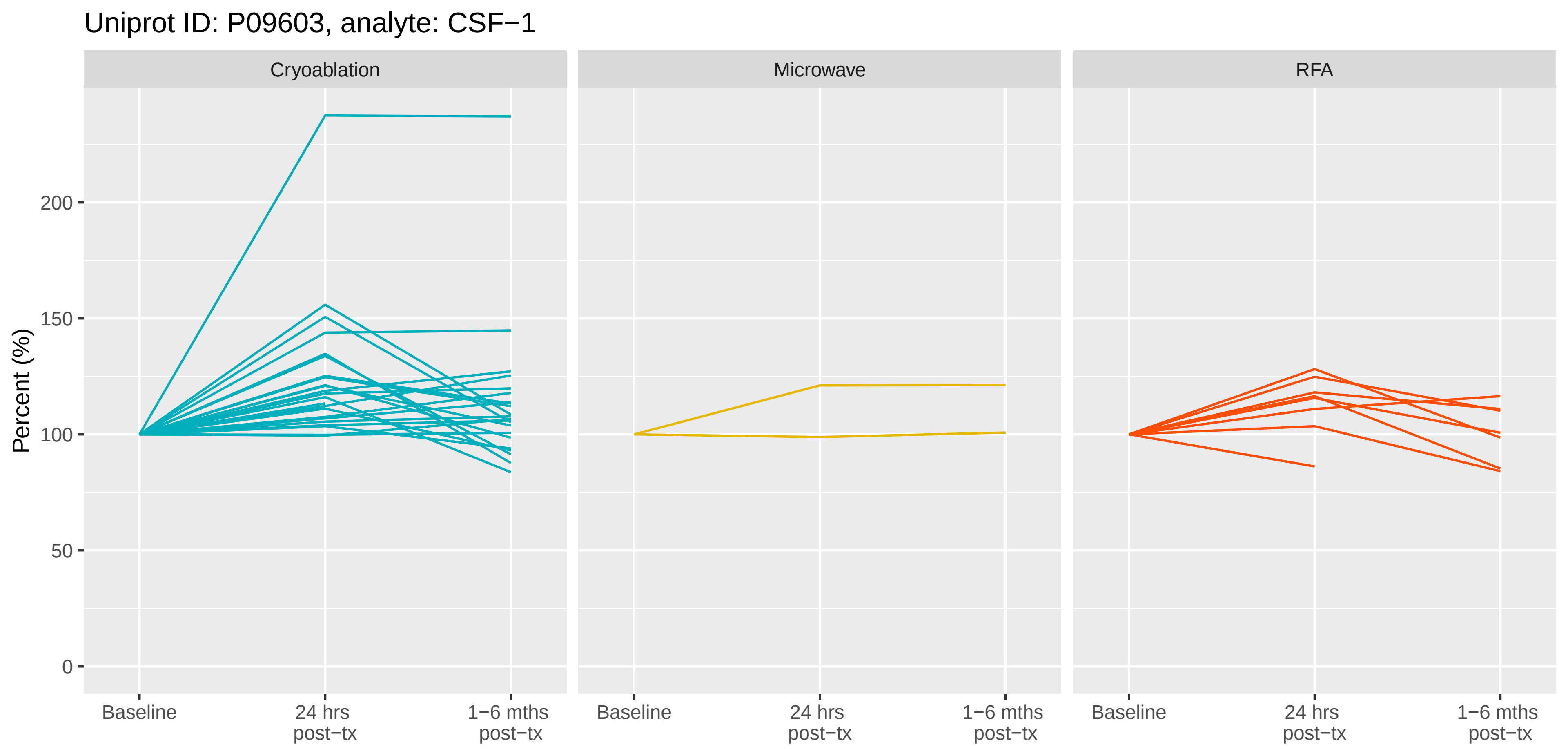

Uniprot ID: P09603, analyte: CSF−1
Cryoablation
Microwave
RFA
Percent (%)
200
150
100
50
0
Baseline
24 hrs post−tx
1−6 mths post−tx
