# Supplementary material for: An Exploratory Analysis of Changes in Circulating Plasma Protein Profiles Following Image-Guided Ablation of Renal Tumours Provides Evidence for Effects on Multiple Biological Processes
Source: Cancers (Basel). 2021 Nov 30;13(23):6037. doi: 10.3390/cancers13236037 (PMC8656737; doi:10.3390/cancers13236037)
Supplement: Supplementary file 1 [file cancers-13-06037-s001.zip › Supplementary File/Figure S4.pdf]

**Figure S4.** Longitudinal profiles of all plasma proteins on the Oncology II panel, prior to and post-ablation. Results for each protein are expressed as a % of the reference baseline (TP2; 100%) and are shown for patients receiving CRYO (n=25), MWA (n=2) and RFA (n=8).

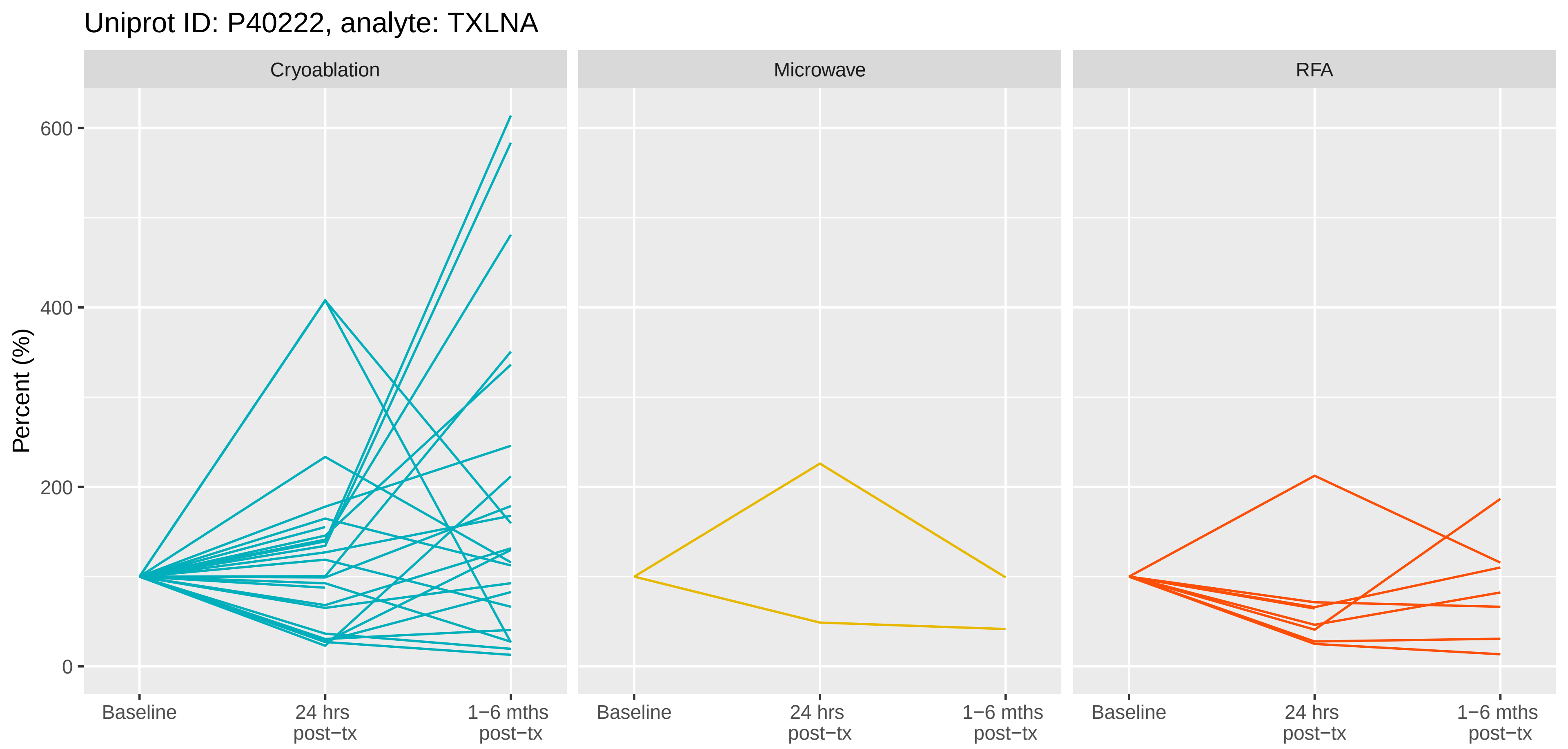

Uniprot ID: P40222, analyte: TXLNA
Cryoablation
Microwave
RFA
Percent (%)
0
200
400
600
Baseline
24 hrs post−tx
1−6 mths post−tx

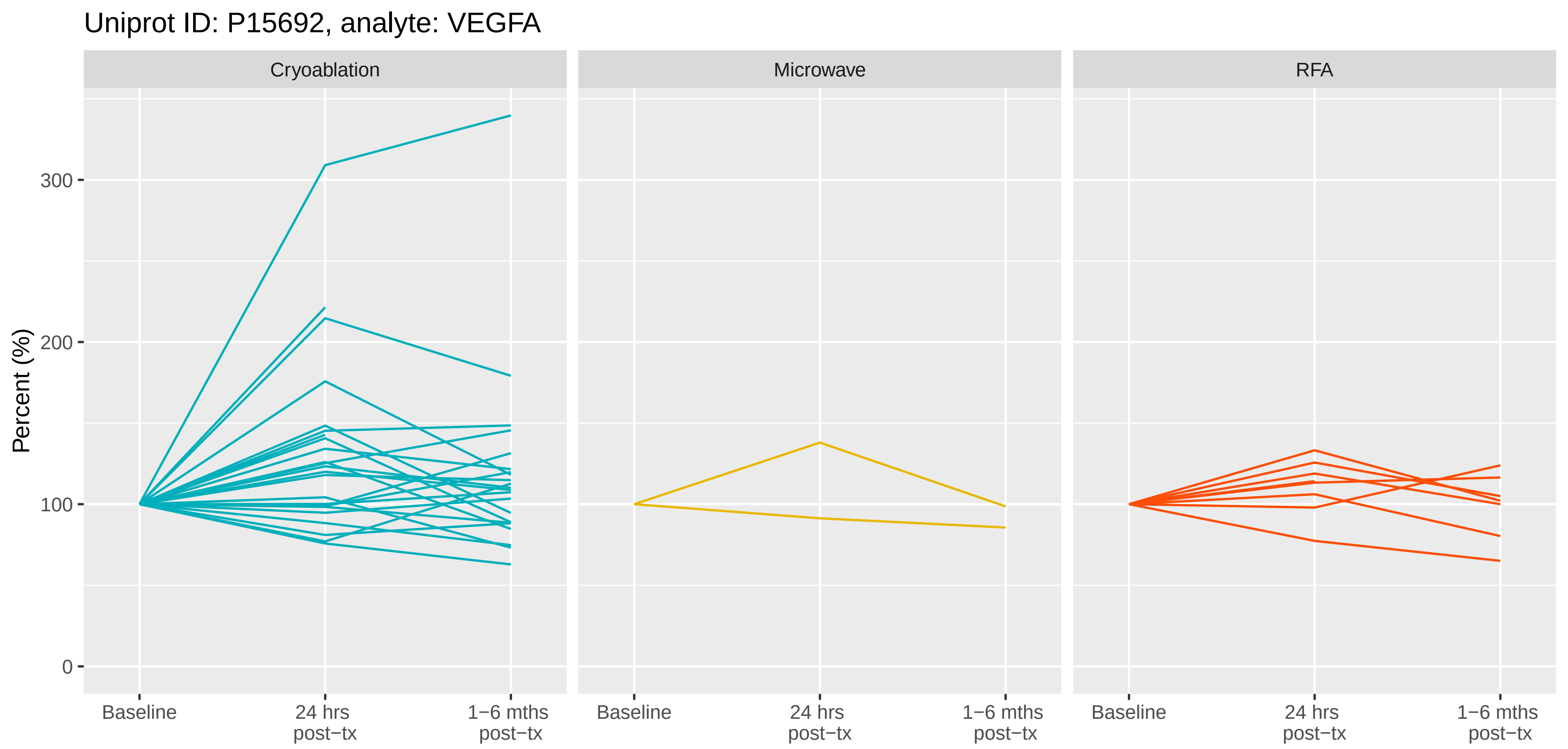

Uniprot ID: P15692, analyte: VEGFA
Cryoablation
Microwave
RFA
Percent (%)
300
200
100
0
Baseline
24 hrs post−tx
1−6 mths post−tx

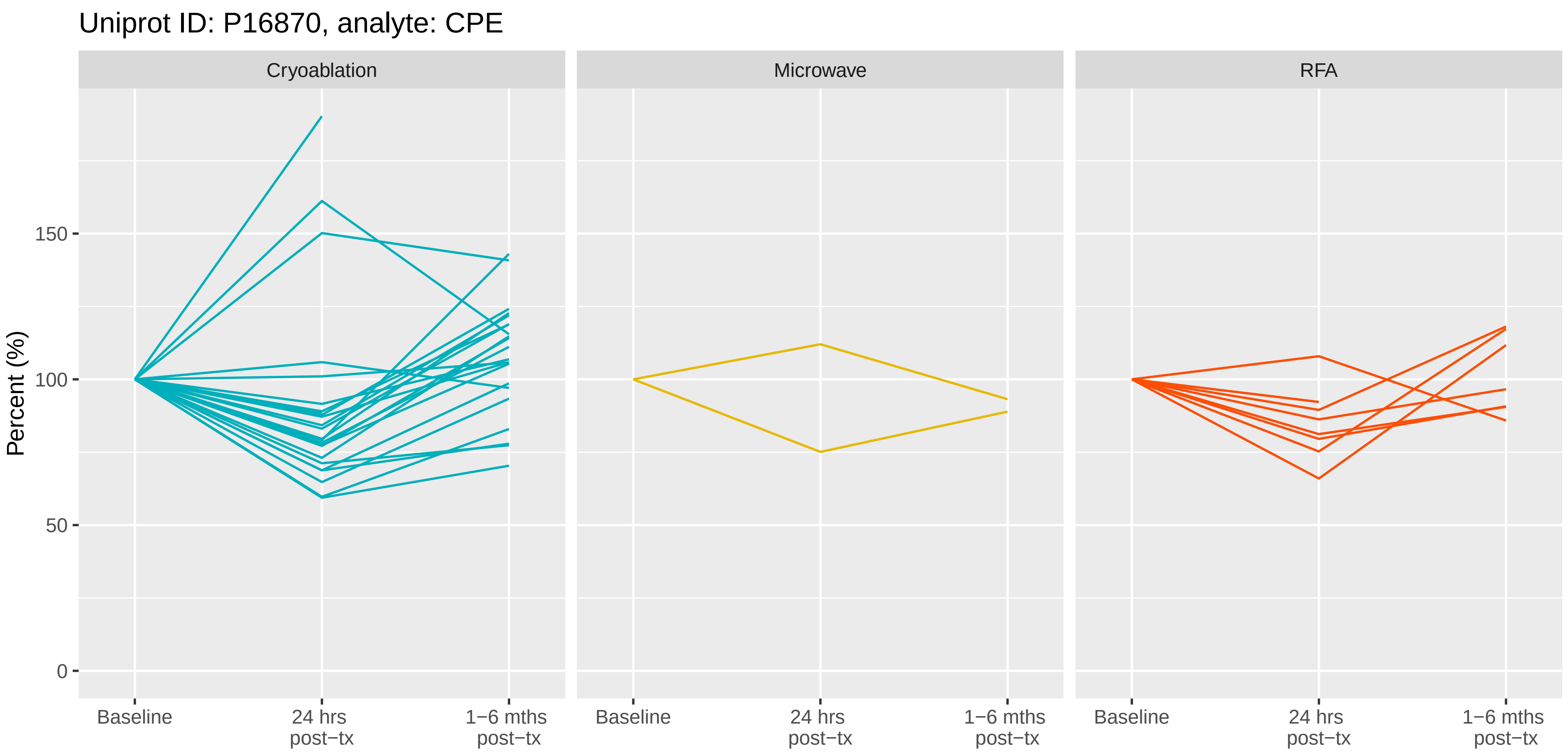
Uniprot ID: P16870, analyte: CPE
Cryoablation
Microwave
RFA
Percent (%)
150
100
50
0
Baseline
24 hrs post−tx
1−6 mths post−tx

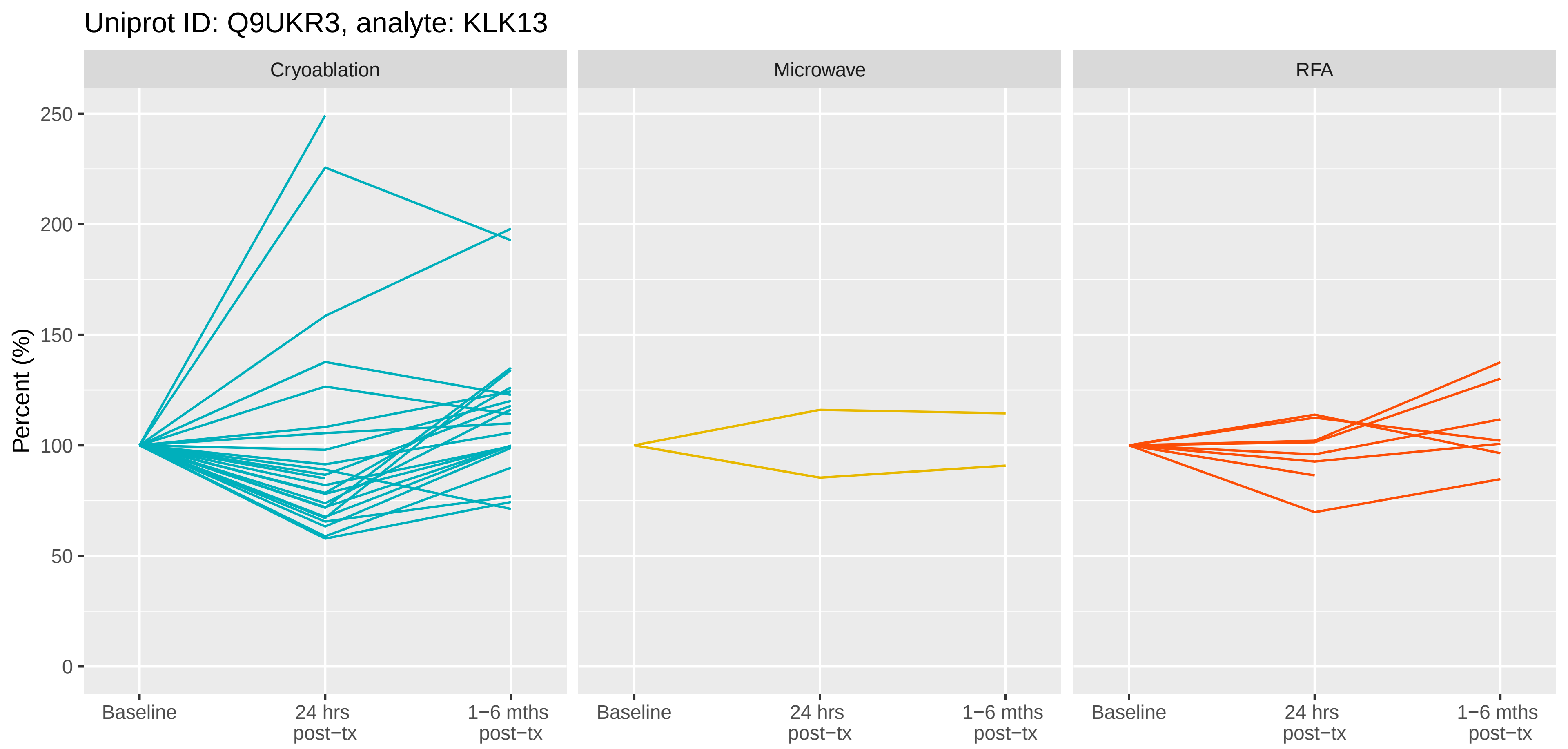
Uniprot ID: Q9UKR3, analyte: KLK13
Cryoablation
Microwave
RFA
Percent (%)
0
50
100
150
200
250
Baseline
24 hrs post−tx
1−6 mths post−tx

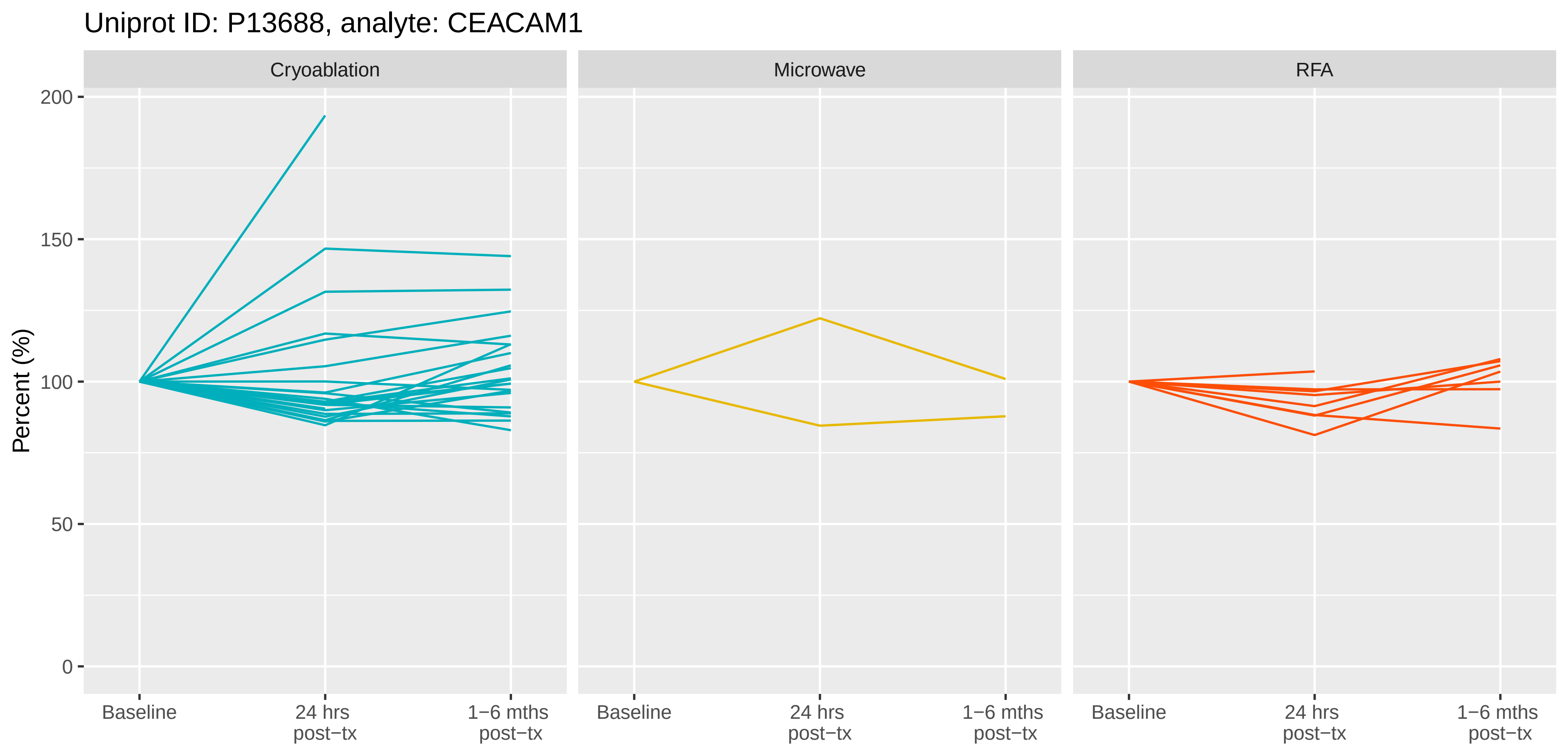

Uniprot ID: P13688, analyte: CEACAM1
Cryoablation
Microwave
RFA
Percent (%)
200
150
100
50
0
Baseline
24 hrs post−tx
1−6 mths post−tx

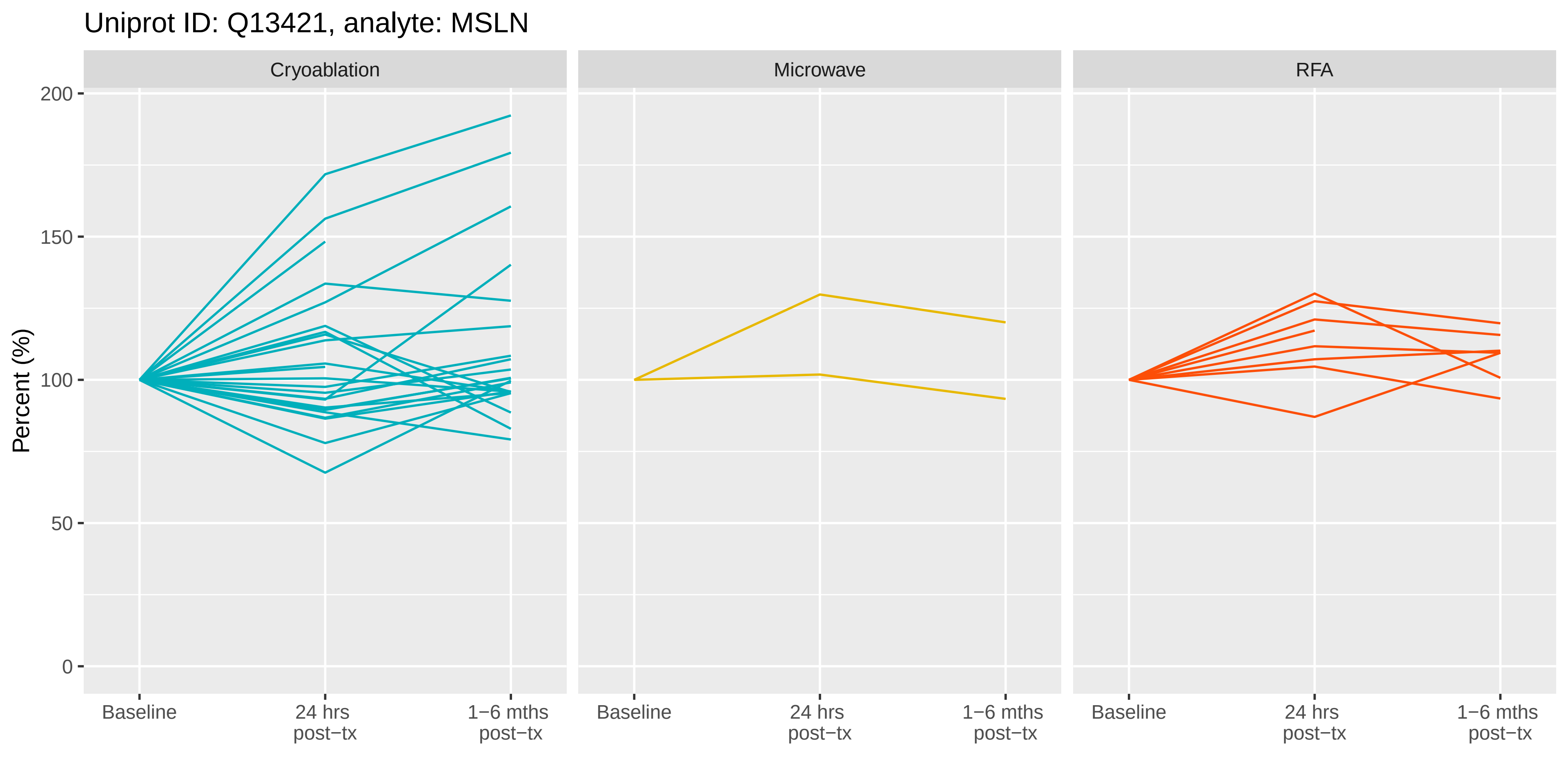

Uniprot ID: Q13421, analyte: MSLN
Cryoablation
Microwave
RFA
Percent (%)
200
150
100
50
0
Baseline
24 hrs post−tx
1−6 mths post−tx

Uniprot ID: O75888, analyte: TNFSF13

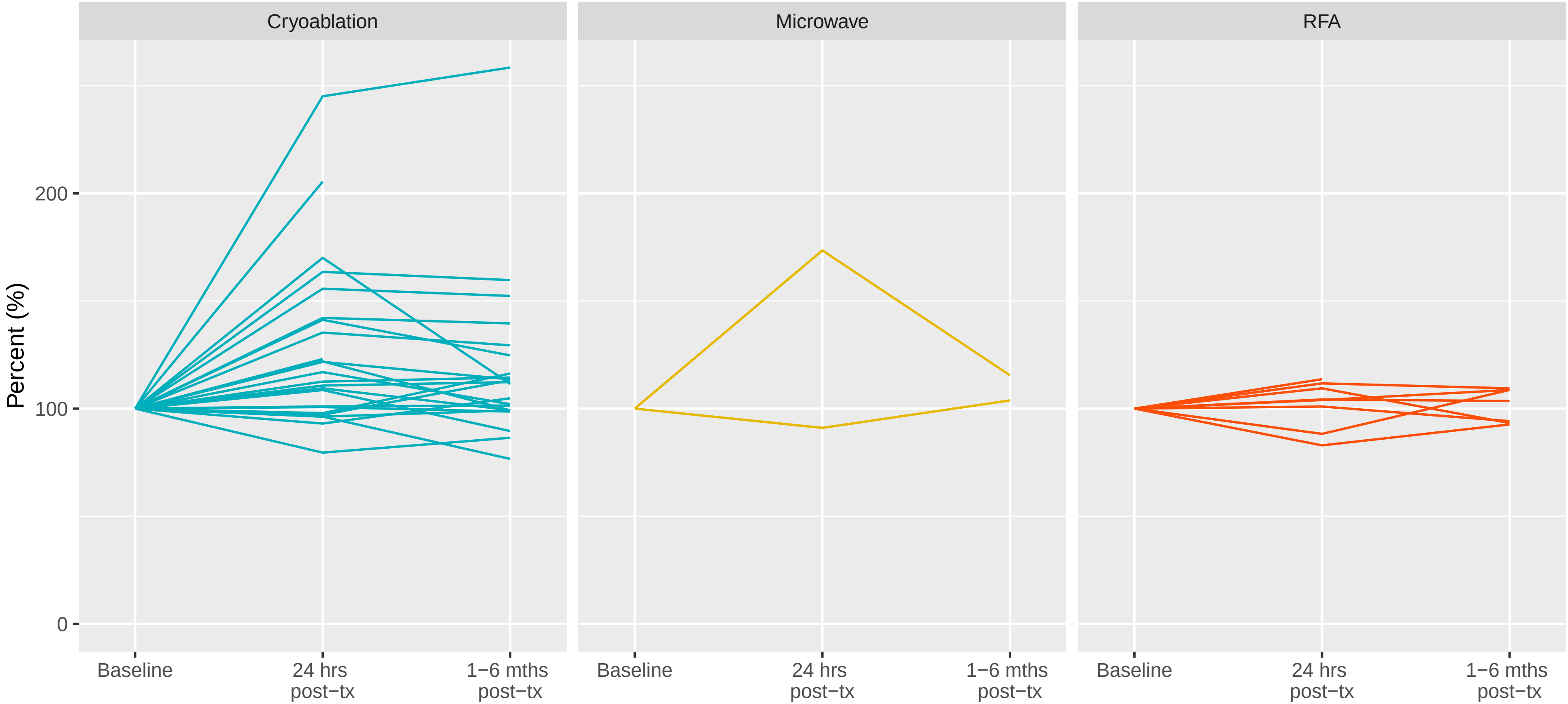

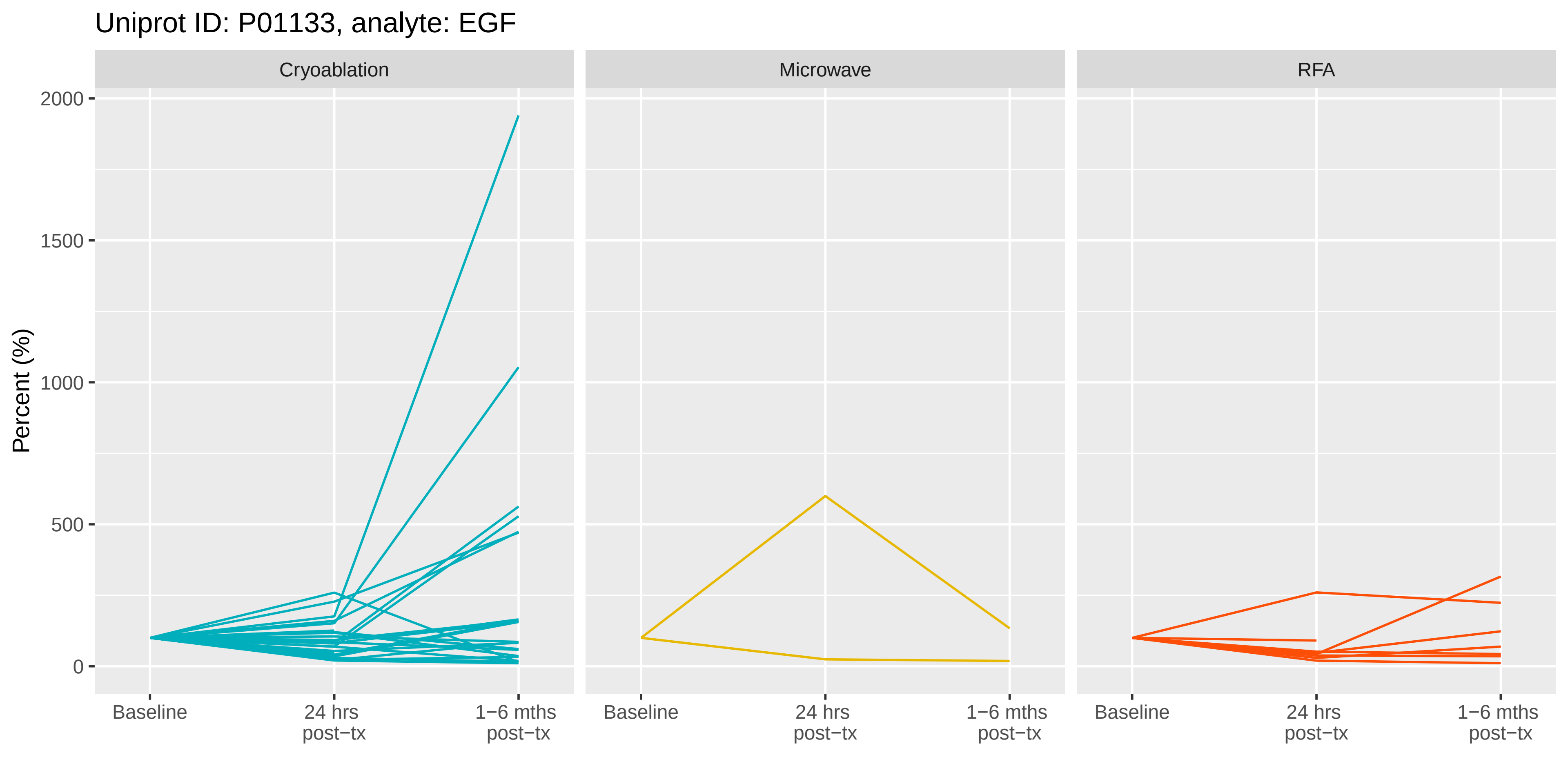

Uniprot ID: P01133, analyte: EGF
Cryoablation
Microwave
RFA
Percent (%)
2000
1500
1000
500
0
Baseline
24 hrs post−tx
1−6 mths post−tx

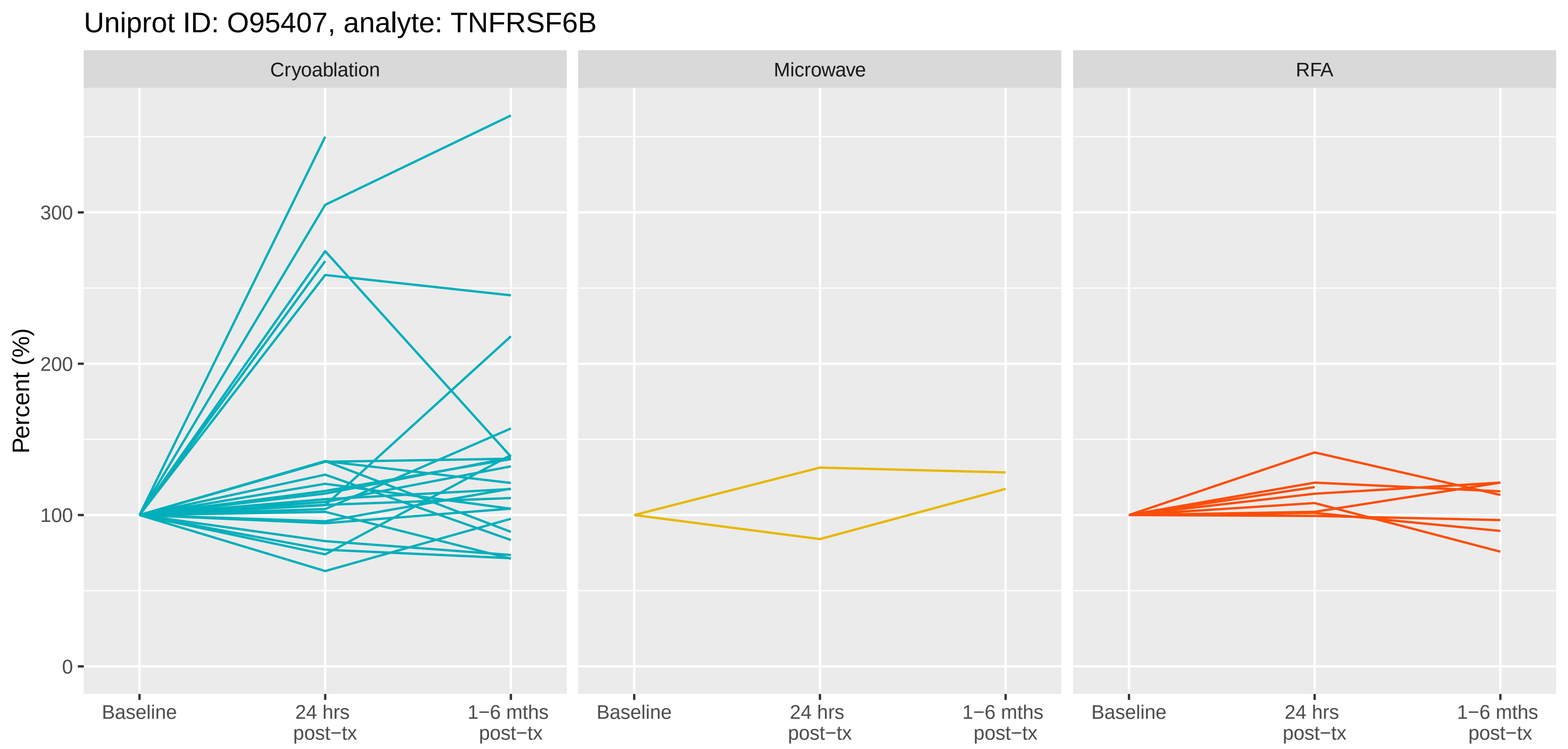

Uniprot ID: O95407, analyte: TNFRSF6B
Cryoablation
Microwave
RFA
Percent (%)
300
200
100
0
Baseline
24 hrs post−tx
1−6 mths post−tx

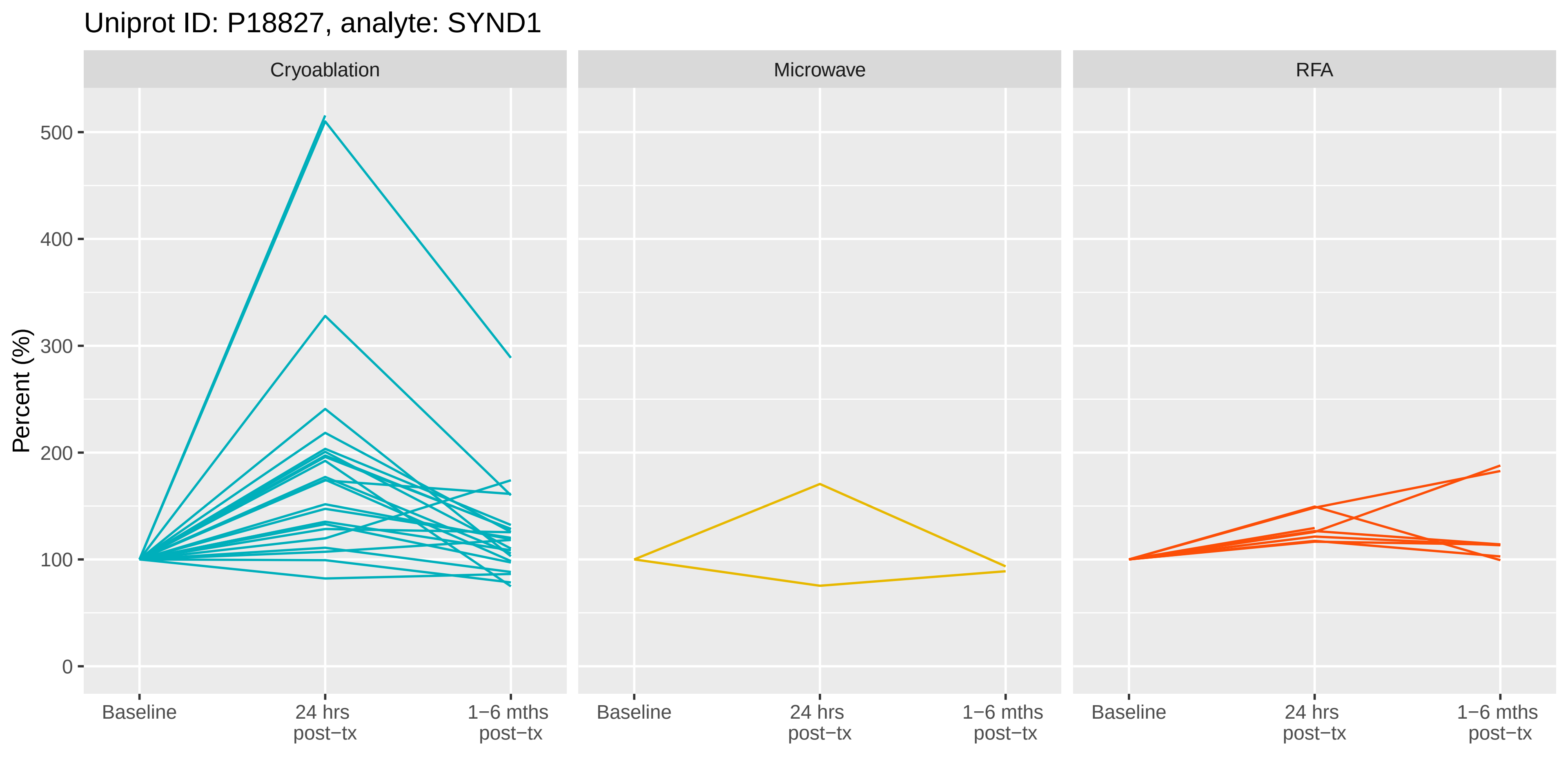

Uniprot ID: P18827, analyte: SYND1
Cryoablation
Microwave
RFA
Percent (%)
500
400
300
200
100
0
Baseline
24 hrs post−tx
1−6 mths post−tx
Baseline
24 hrs post−tx
1−6 mths post−tx
Baseline
24 hrs post−tx
1−6 mths post−tx

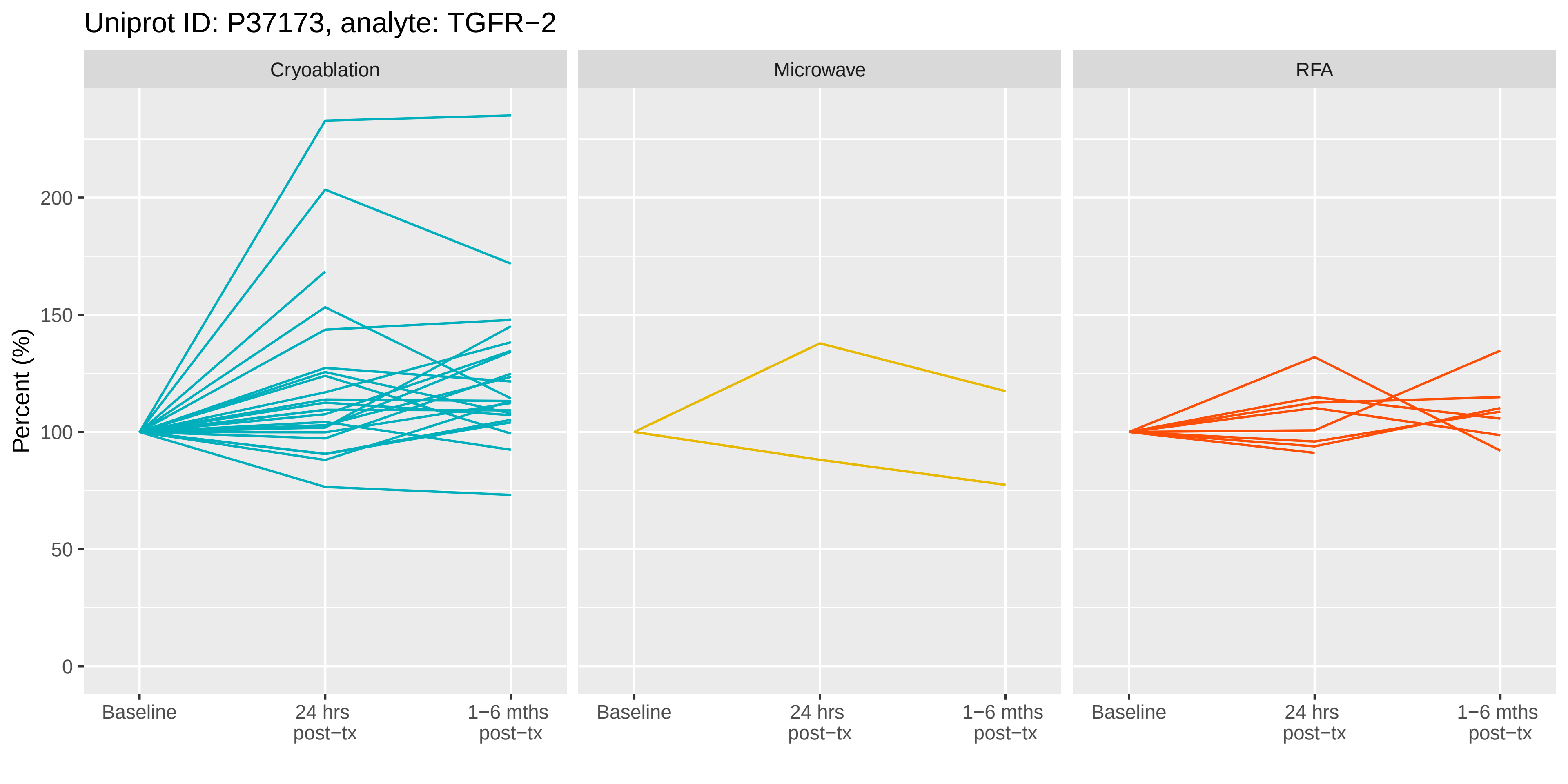

Uniprot ID: P37173, analyte: TGFR−2
Cryoablation
Microwave
RFA
Percent (%)
0
50
100
150
200
Baseline
24 hrs post−tx
1−6 mths post−tx

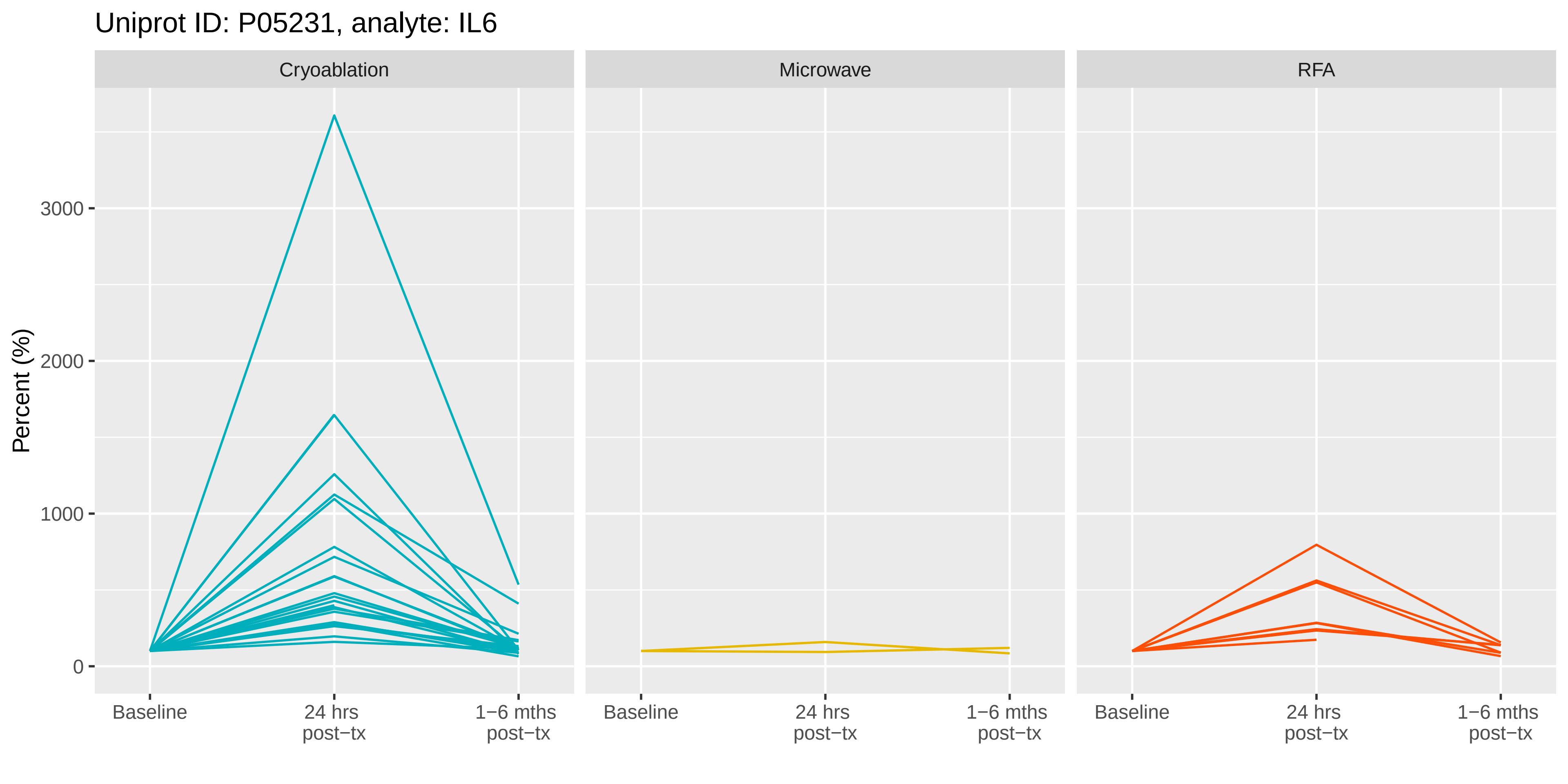

Uniprot ID: P05231, analyte: IL6
Cryoablation
Microwave
RFA
Percent (%)
3000
2000
1000
0
Baseline
24 hrs post−tx
1−6 mths post−tx

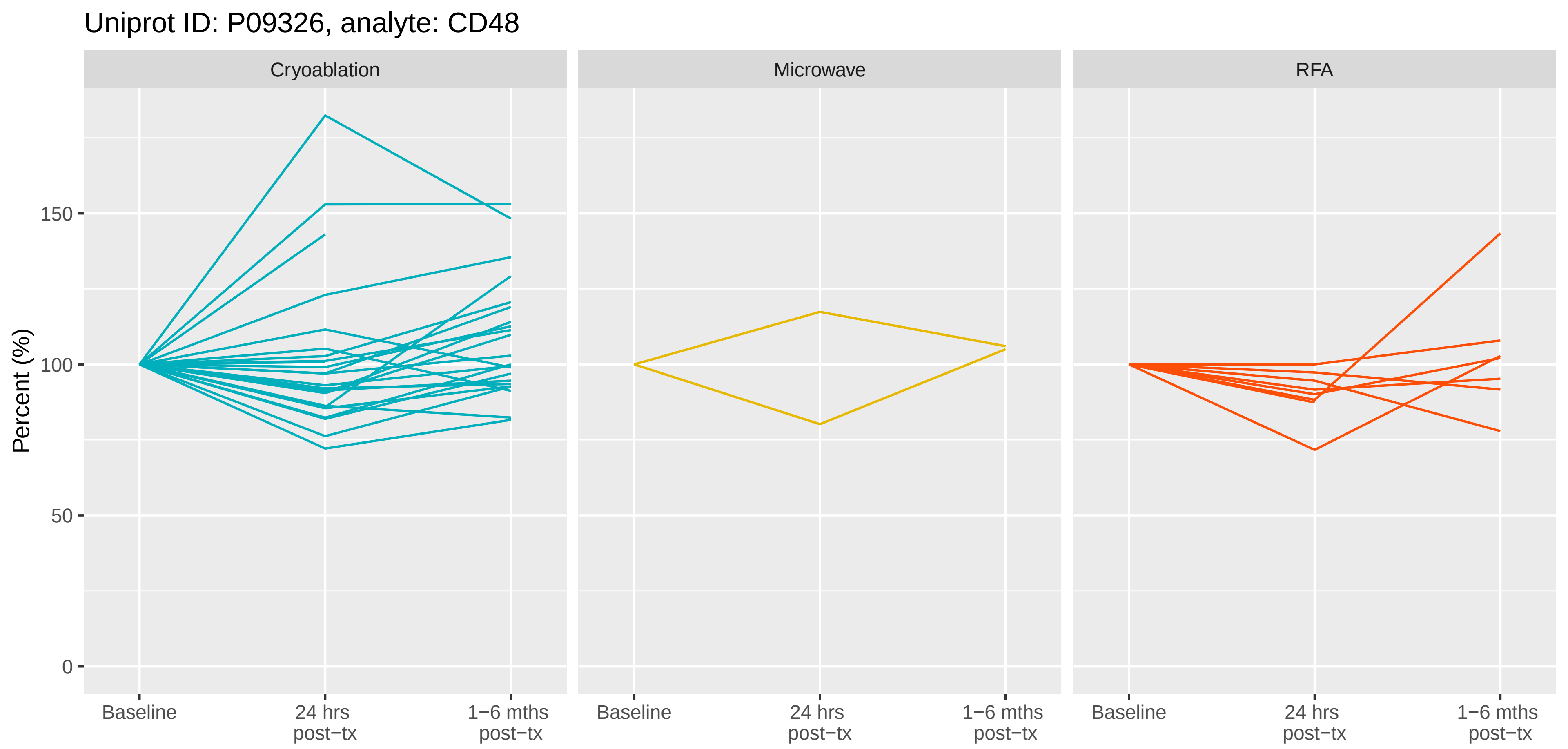
Uniprot ID: P09326, analyte: CD48
Cryoablation
Microwave
RFA
Percent (%)
150
100
50
0
Baseline
24 hrs post−tx
1−6 mths post−tx

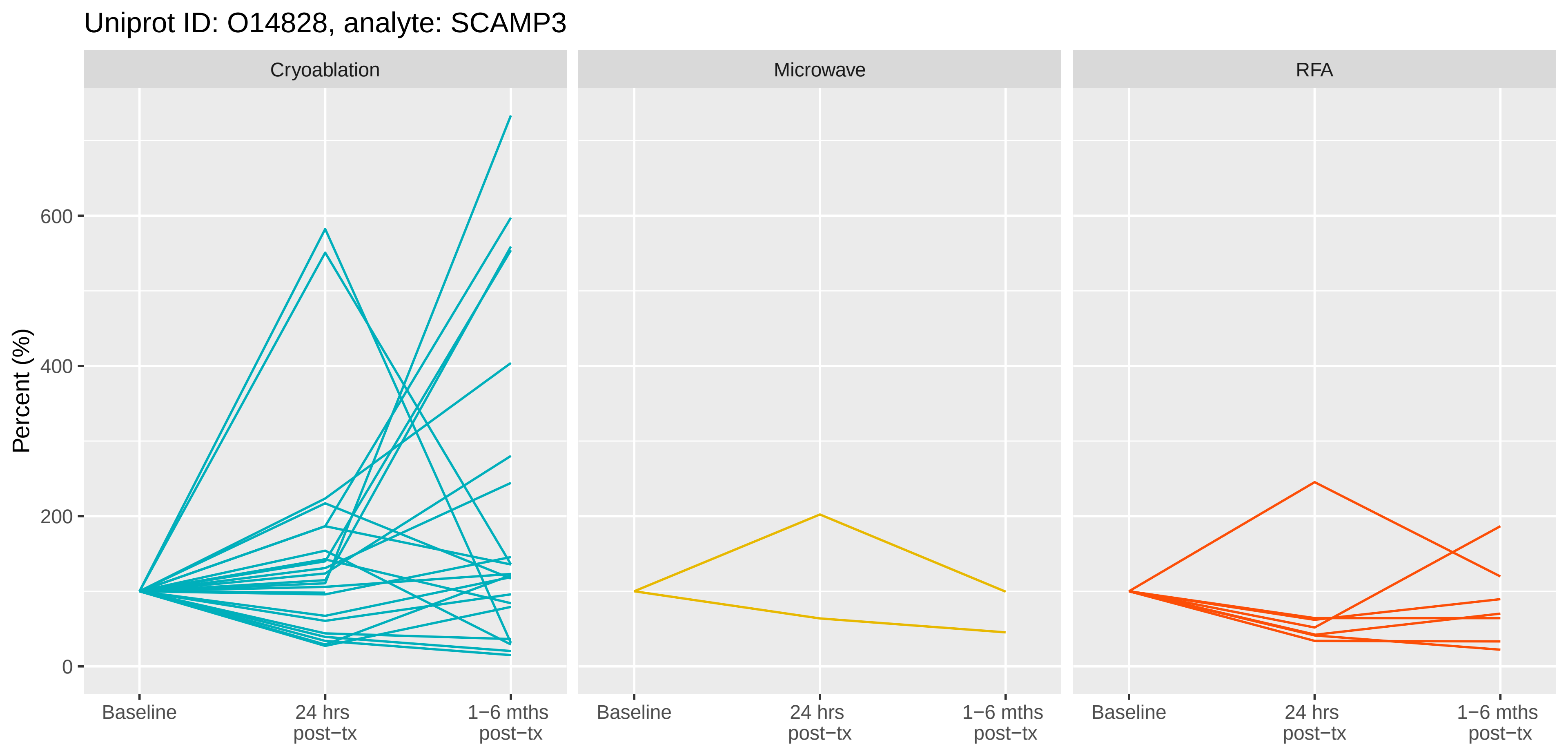

Uniprot ID: O14828, analyte: SCAMP3
Cryoablation
Microwave
RFA
Percent (%)
0
200
400
600
Baseline
24 hrs post−tx
1−6 mths post−tx

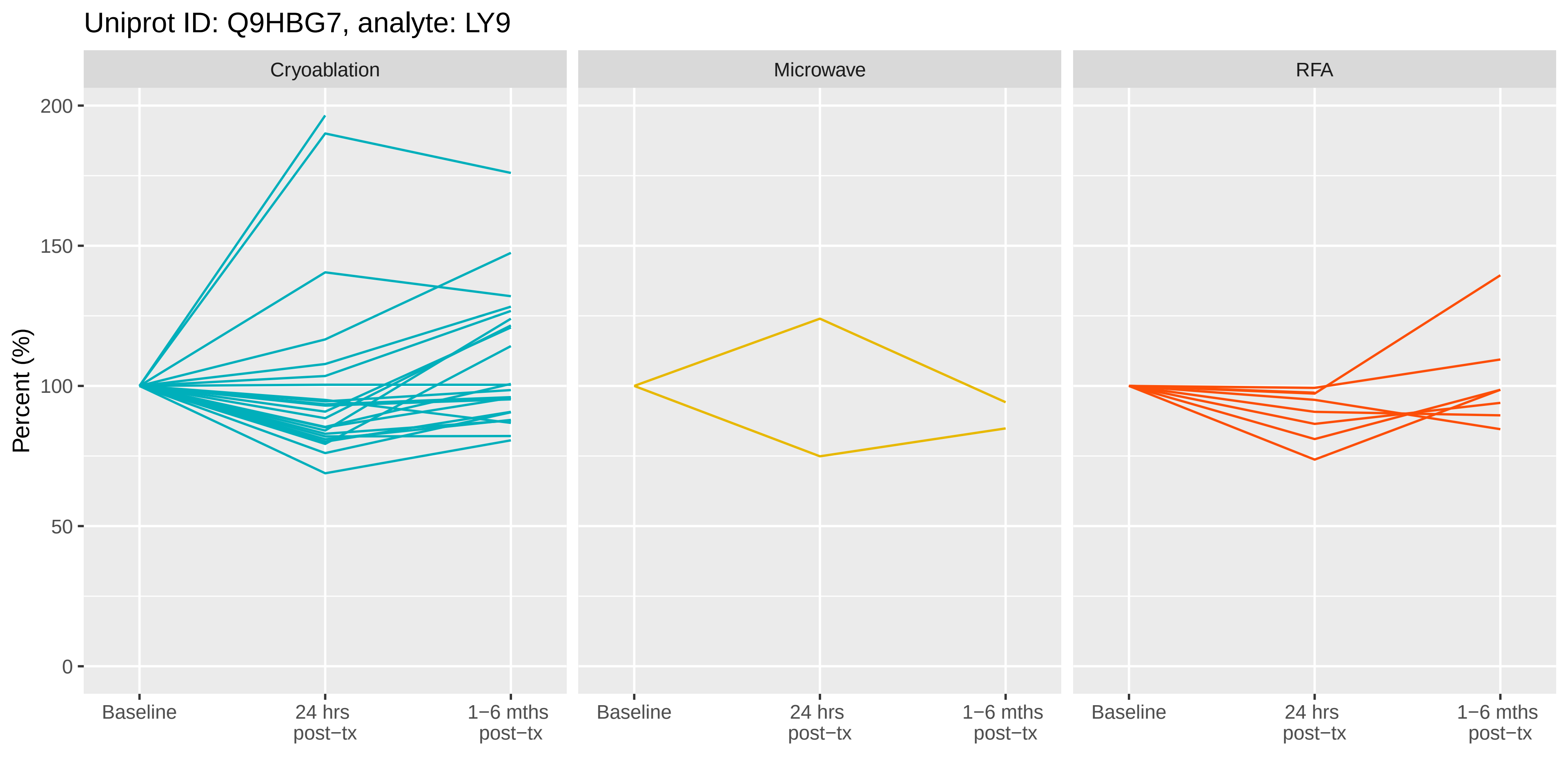

Uniprot ID: Q9HBG7, analyte: LY9
Cryoablation
Microwave
RFA
Percent (%)
200
150
100
50
0
Baseline
24 hrs post−tx
1−6 mths post−tx

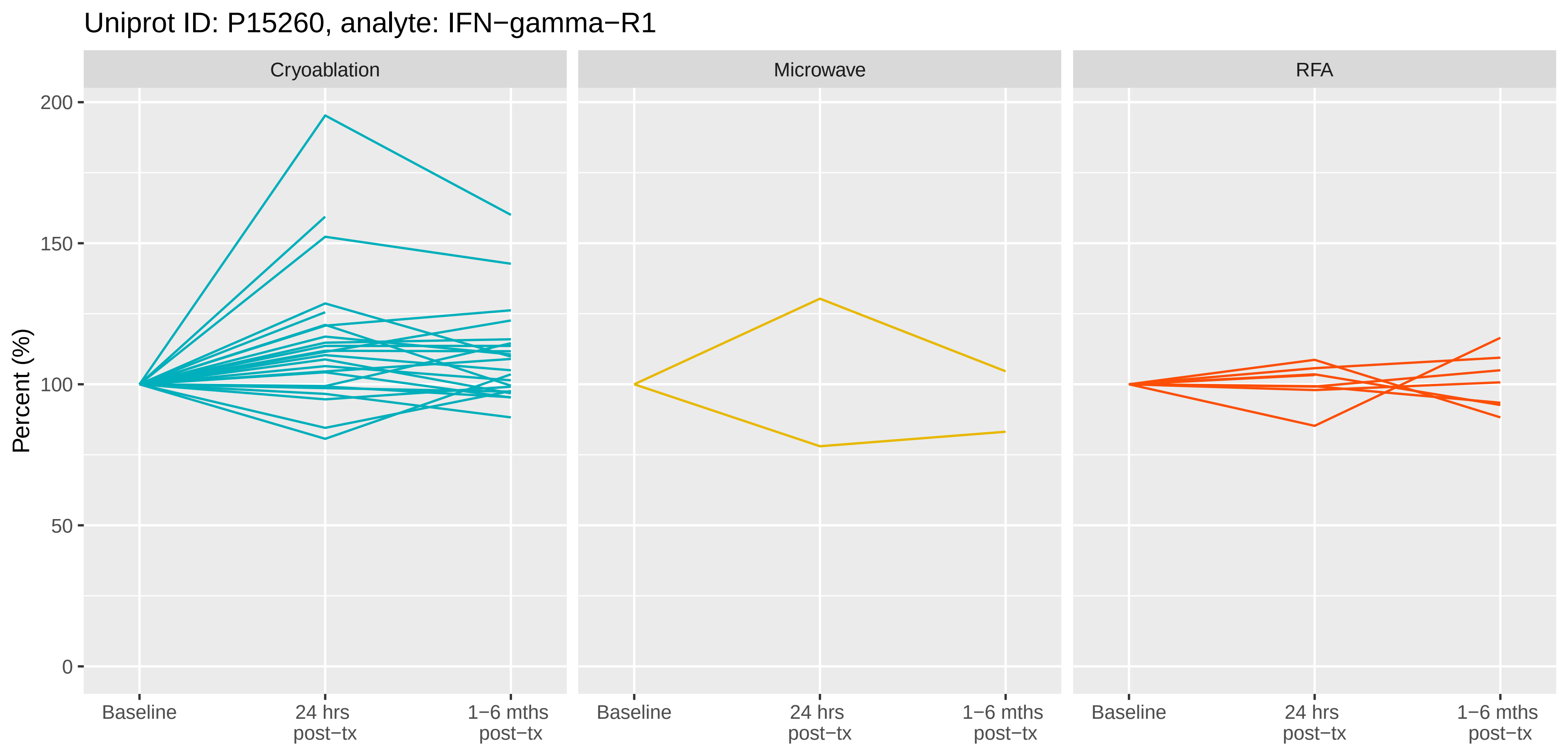
Uniprot ID: P15260, analyte: IFN−gamma−R1
Cryoablation
Microwave
RFA
Percent (%)
200
150
100
50
0
Baseline
24 hrs post−tx
1−6 mths post−tx

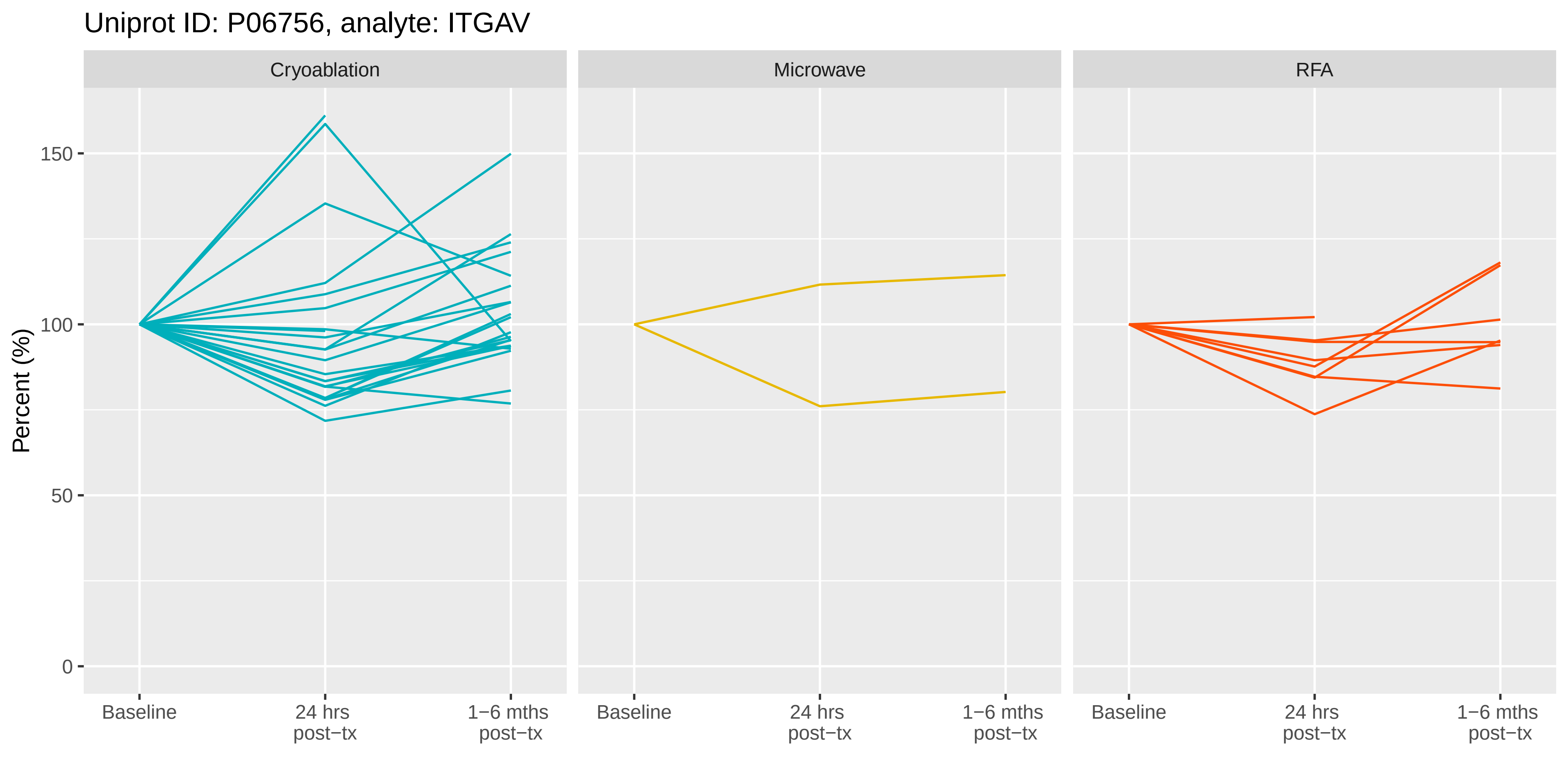
Uniprot ID: P06756, analyte: ITGAV
Cryoablation
Microwave
RFA
Percent (%)
150
100
50
0
Baseline
24 hrs post−tx
1−6 mths post−tx

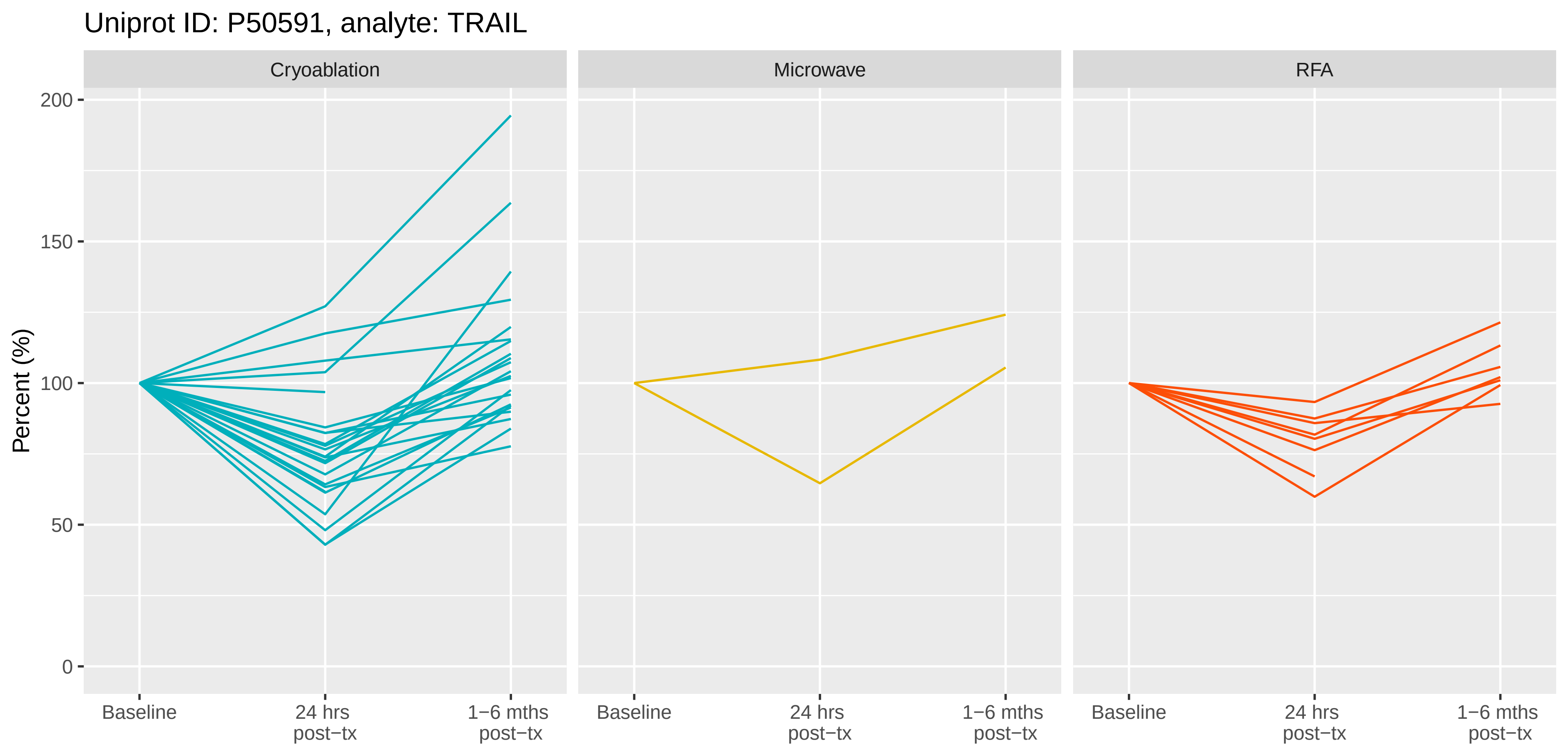
Uniprot ID: P50591, analyte: TRAIL
Cryoablation
Microwave
RFA
Percent (%)
0
50
100
150
200
Baseline
24 hrs post−tx
1−6 mths post−tx

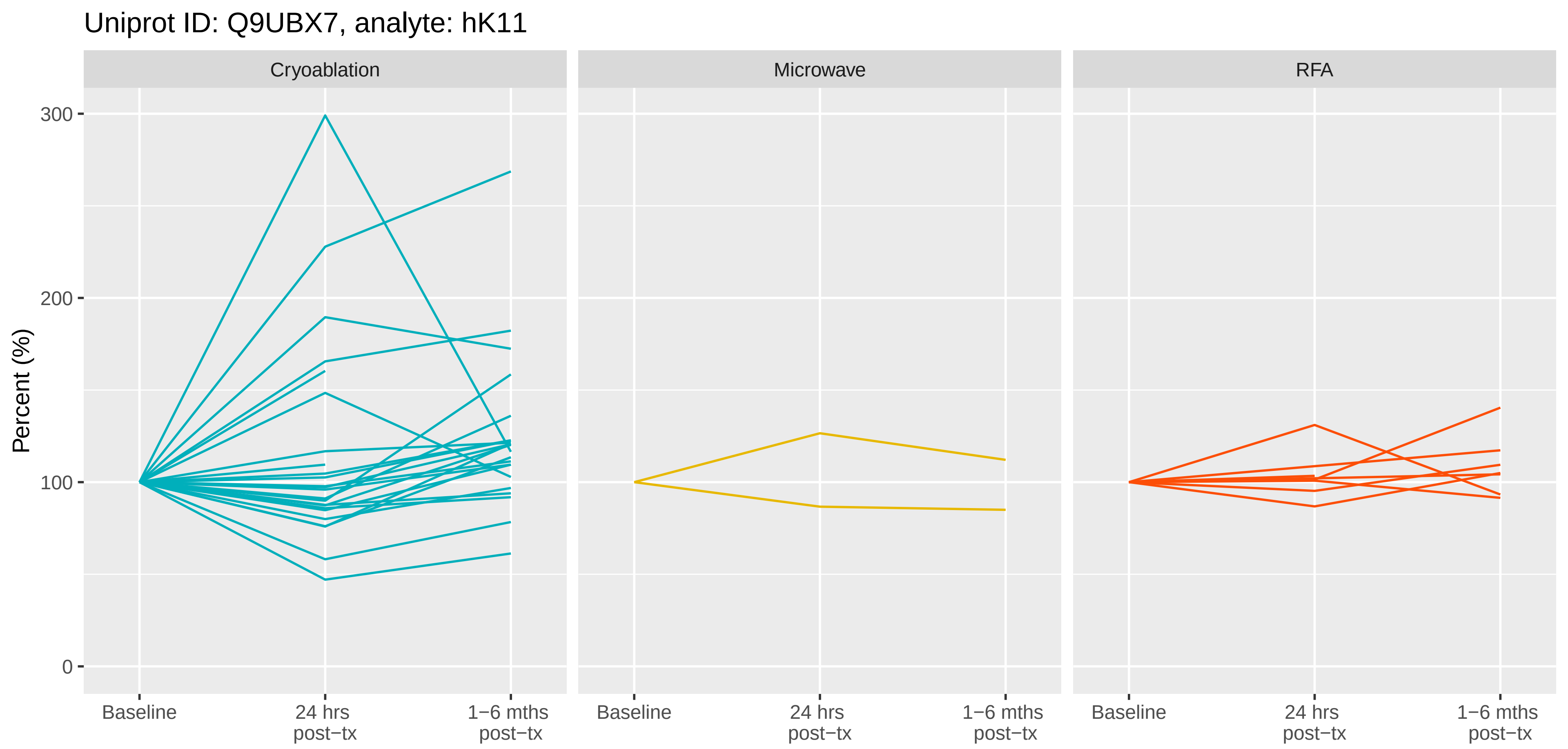
Uniprot ID: Q9UBX7, analyte: hK11
Cryoablation
Microwave
RFA
Percent (%)
300
200
100
0
Baseline
24 hrs post−tx
1−6 mths post−tx

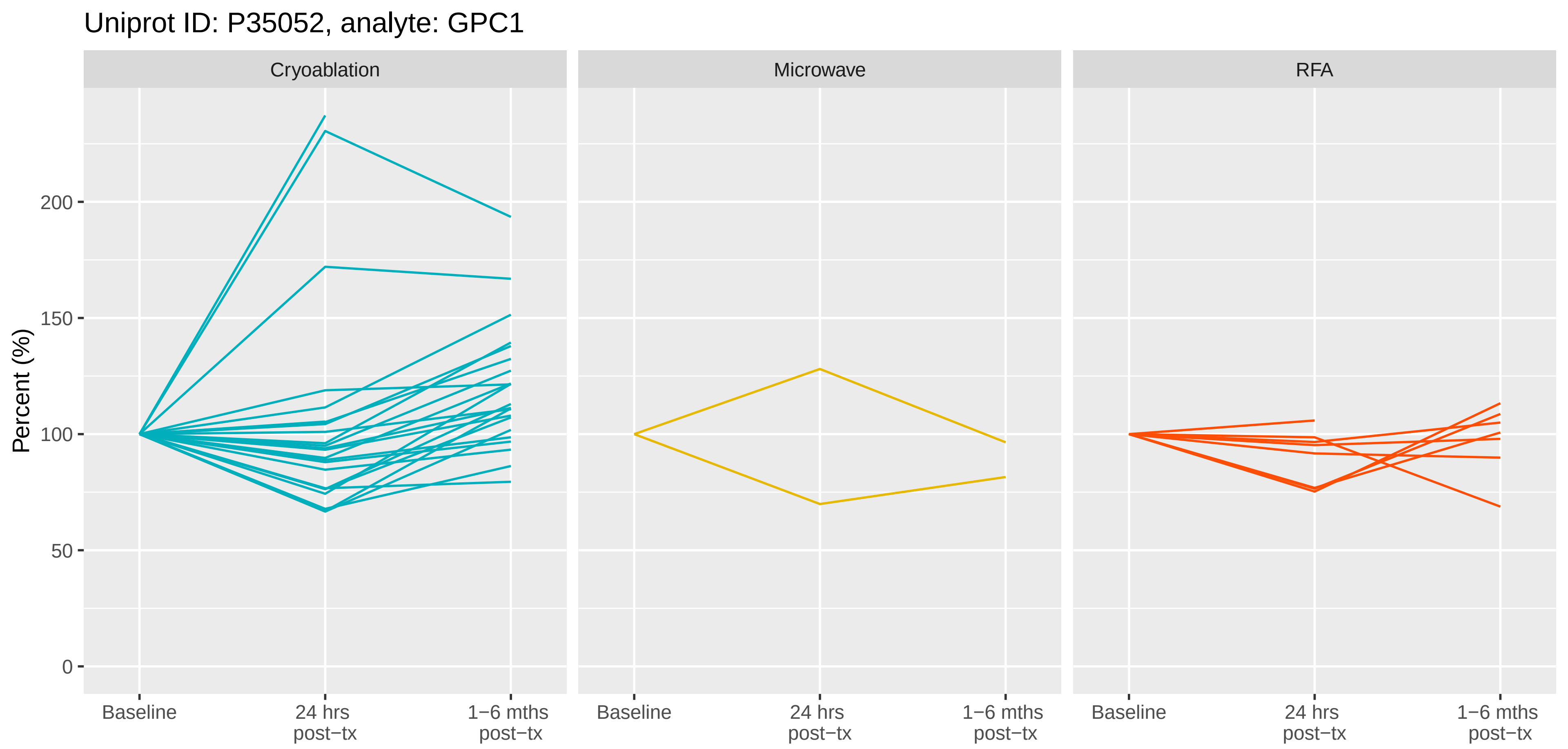

Uniprot ID: P35052, analyte: GPC1
Cryoablation
Microwave
RFA
Percent (%)
200
150
100
50
0
Baseline
24 hrs post−tx
1−6 mths post−tx

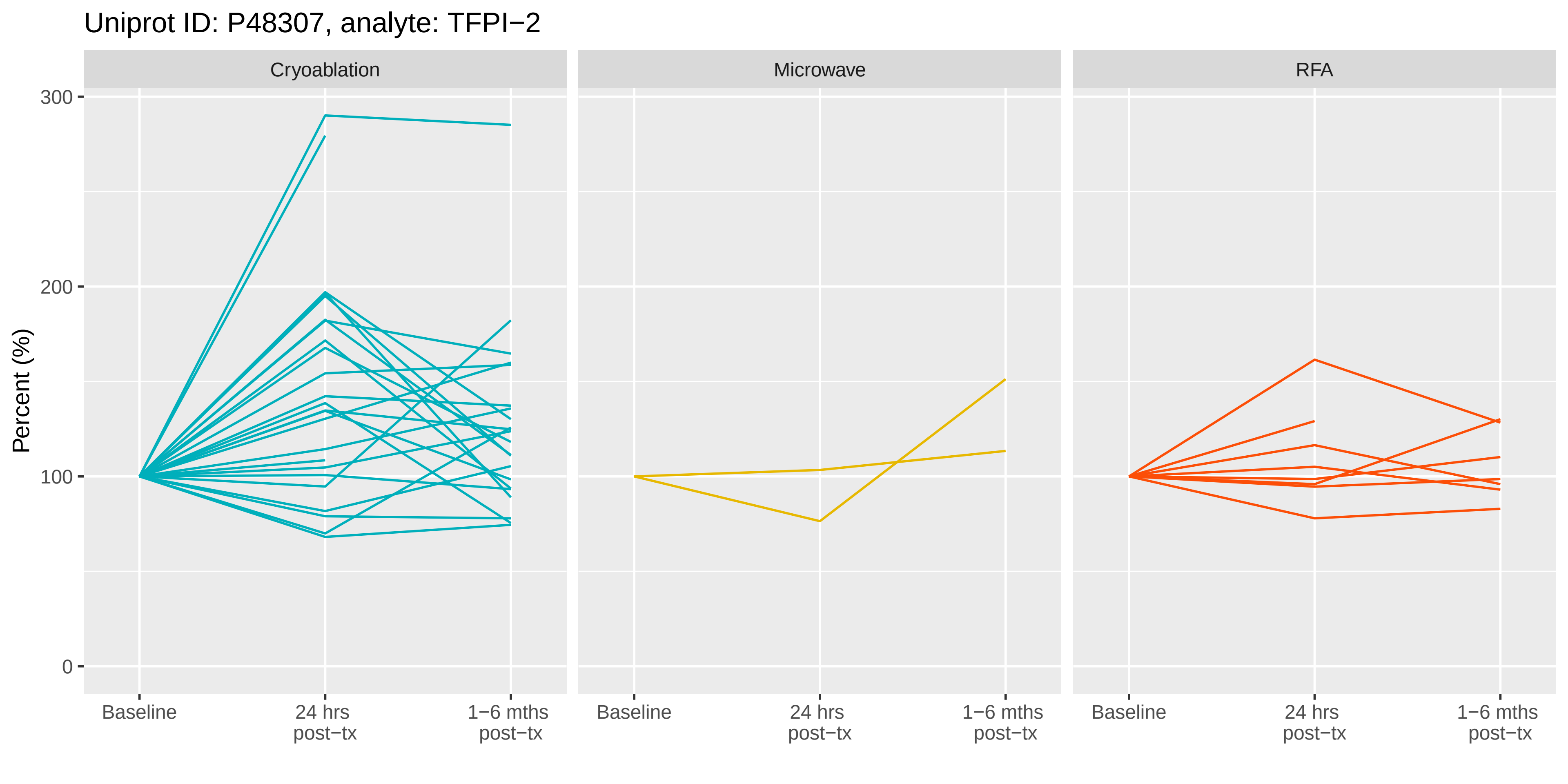

Uniprot ID: P48307, analyte: TFPI−2
Cryoablation
Microwave
RFA
Percent (%)
300
200
100
0
Baseline
24 hrs post−tx
1−6 mths post−tx

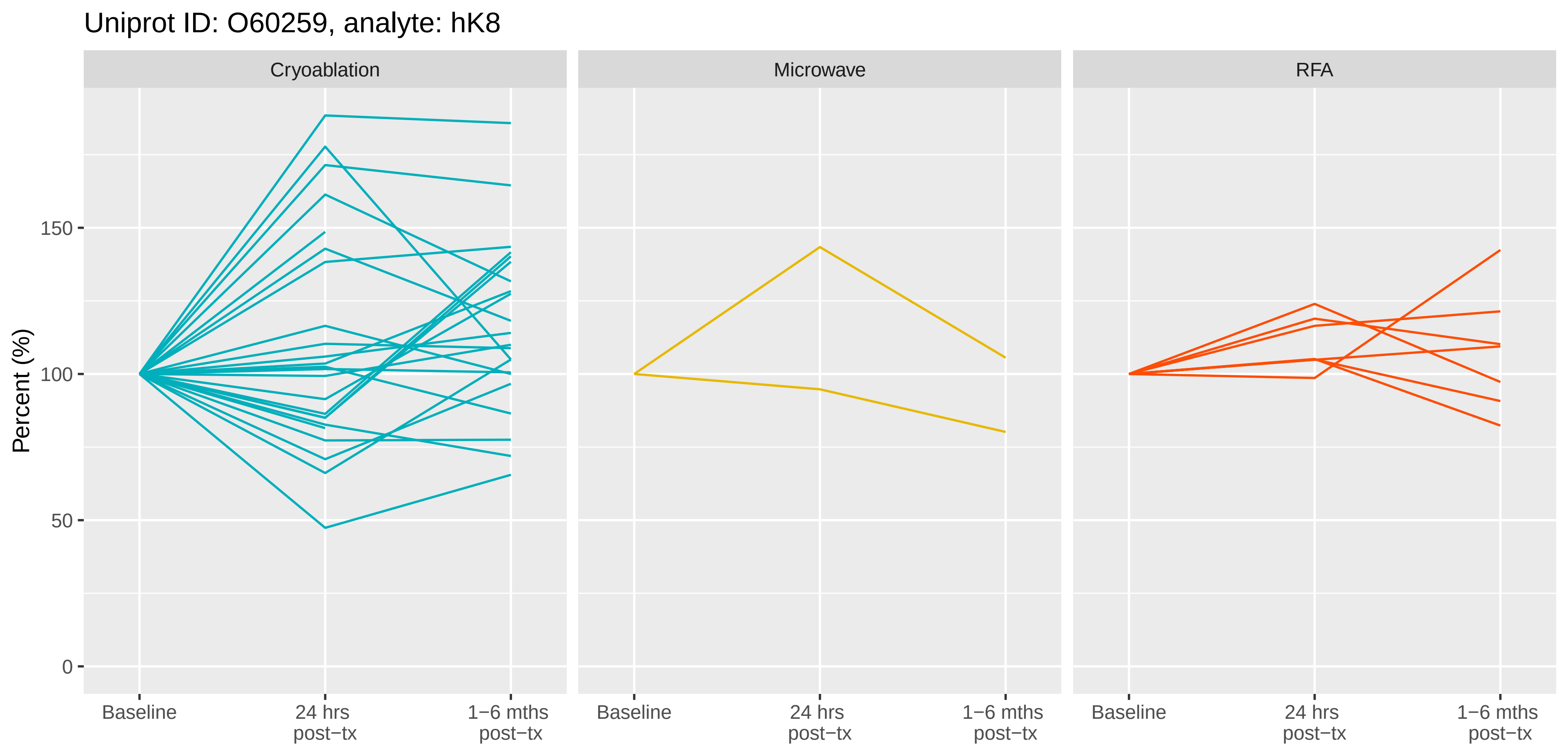

Uniprot ID: O60259, analyte: hK8
Cryoablation
Microwave
RFA
Percent (%)
150
100
50
0
Baseline
24 hrs post−tx
1−6 mths post−tx

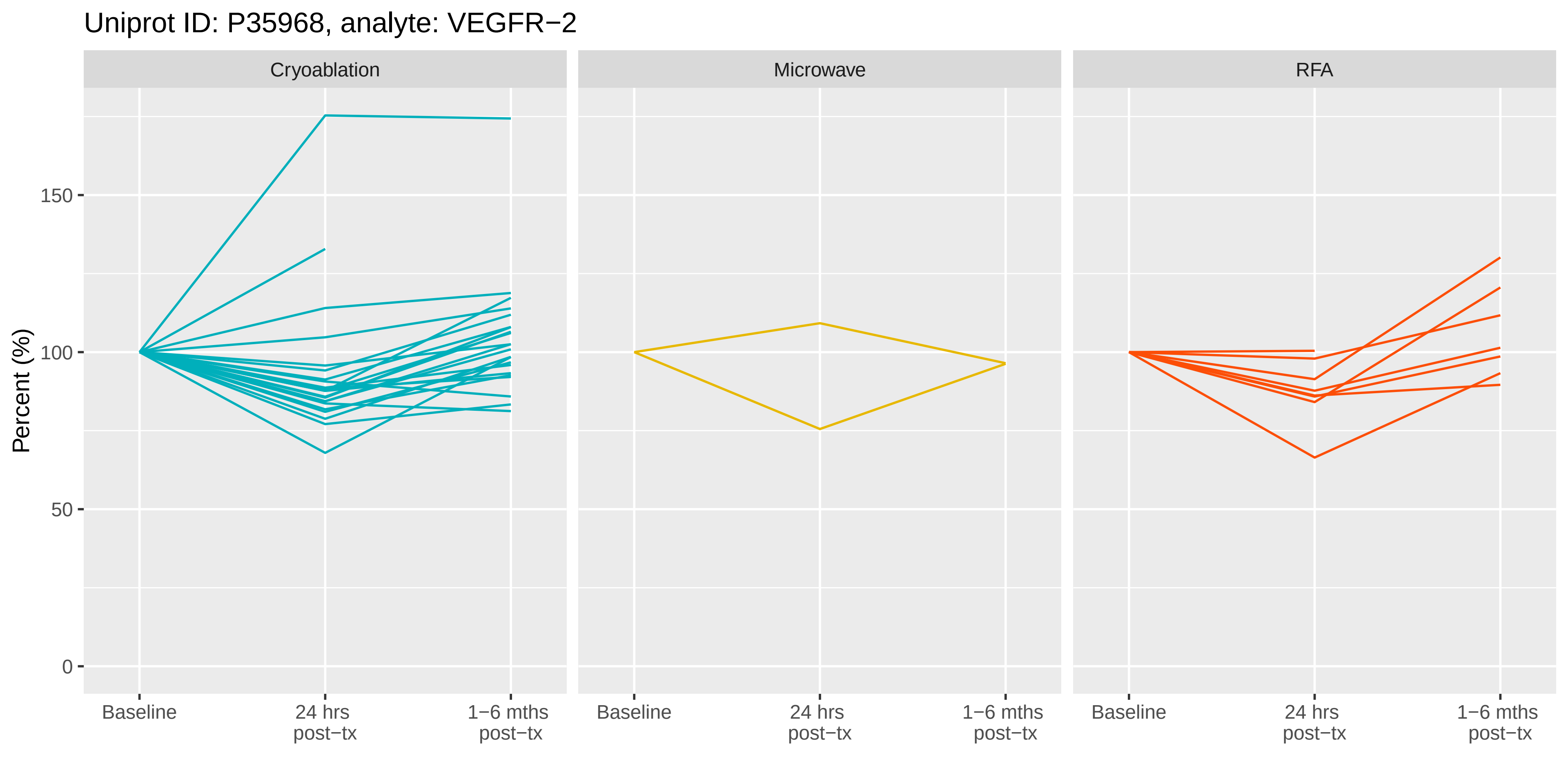

Uniprot ID: P35968, analyte: VEGFR−2
Cryoablation
Microwave
RFA
Percent (%)
150
100
50
0
Baseline
24 hrs post−tx
1−6 mths post−tx

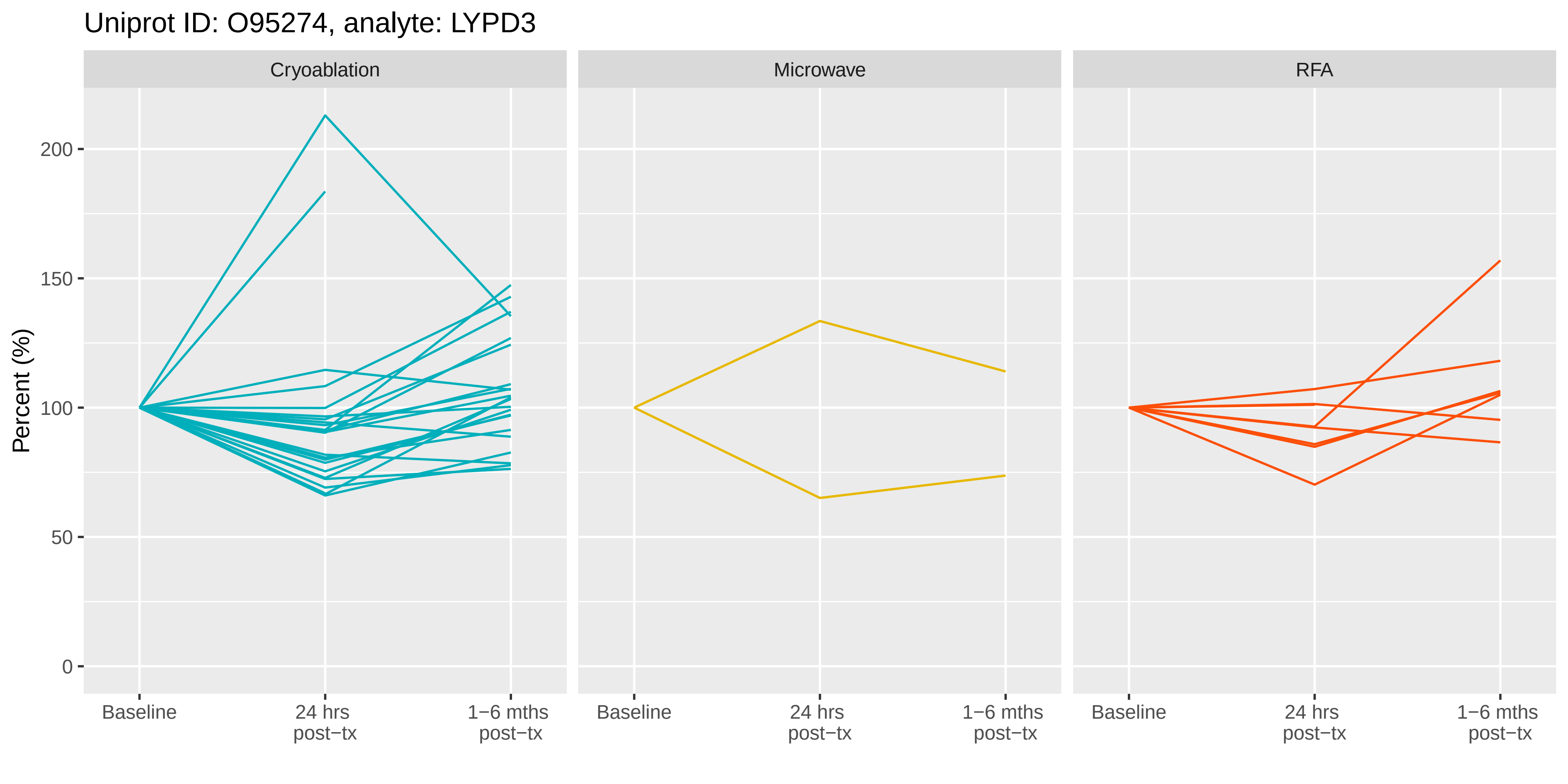

Uniprot ID: O95274, analyte: LYPD3
Cryoablation
Microwave
RFA
Percent (%)
200
150
100
50
0
Baseline
24 hrs post−tx
1−6 mths post−tx

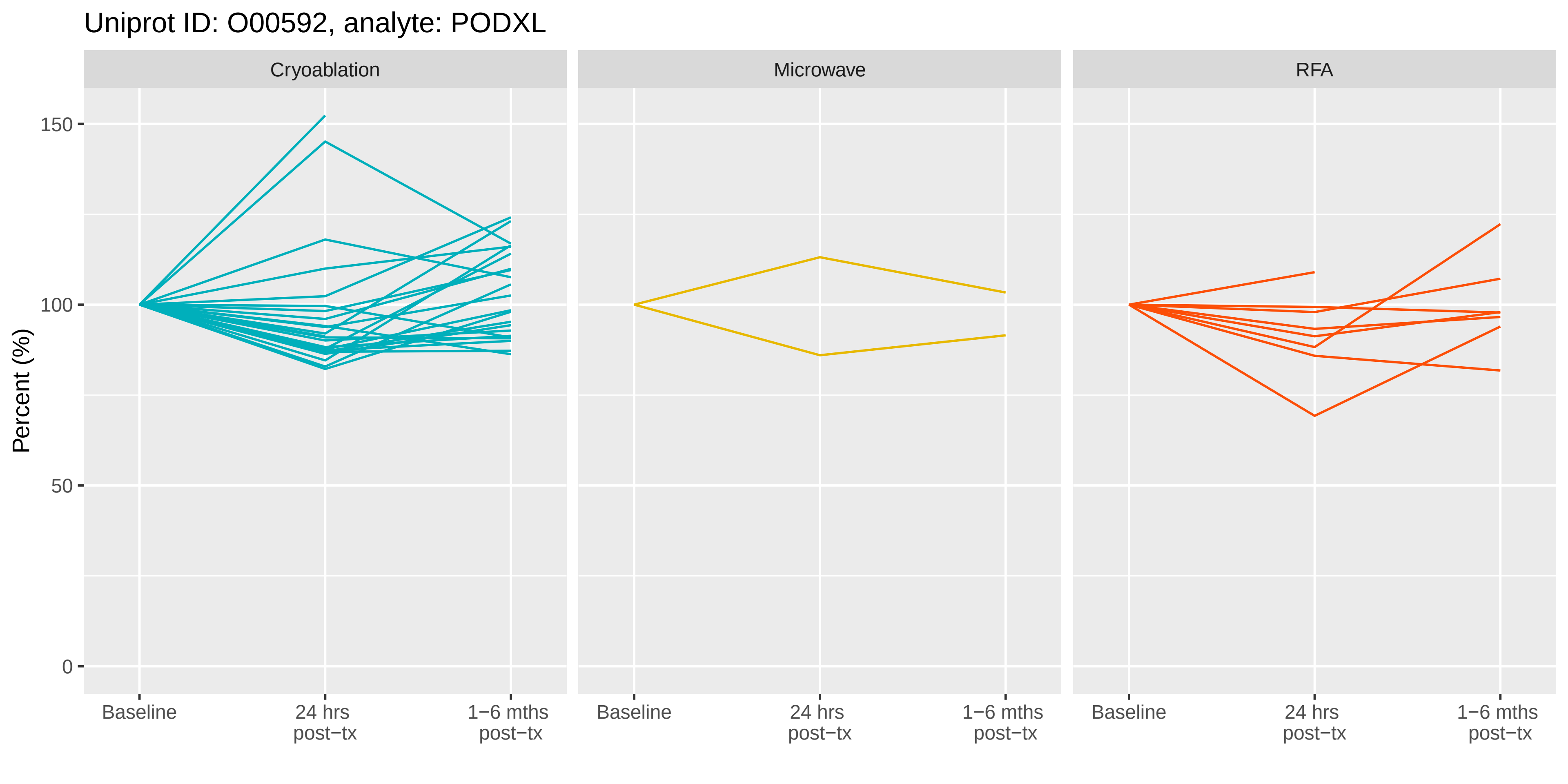
Uniprot ID: O00592, analyte: PODXL
Cryoablation
Microwave
RFA
Percent (%)
150
100
50
0
Baseline
24 hrs post−tx
1−6 mths post−tx

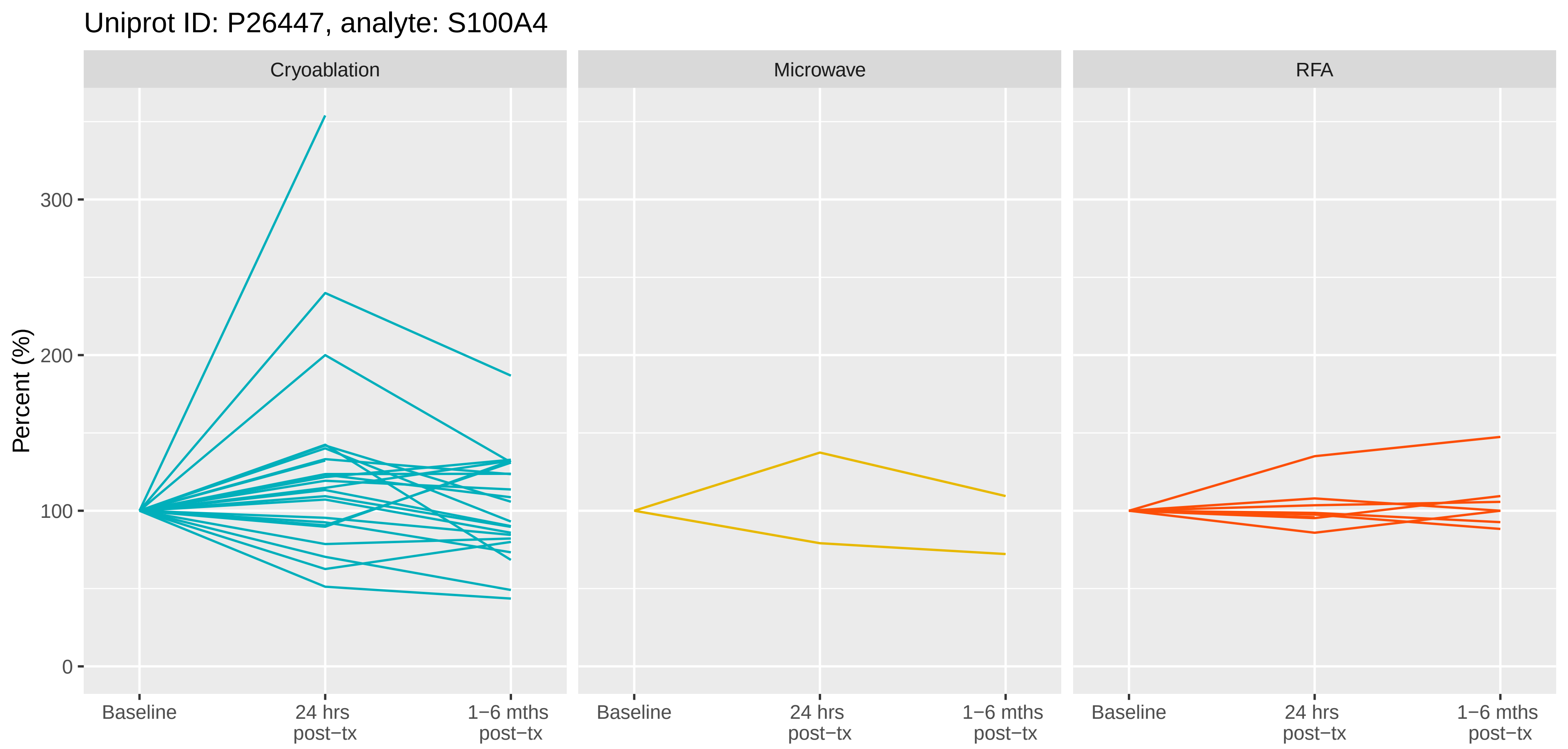

Uniprot ID: P26447, analyte: S100A4
Cryoablation
Microwave
RFA
Percent (%)
300
200
100
0
Baseline
24 hrs post−tx
1−6 mths post−tx

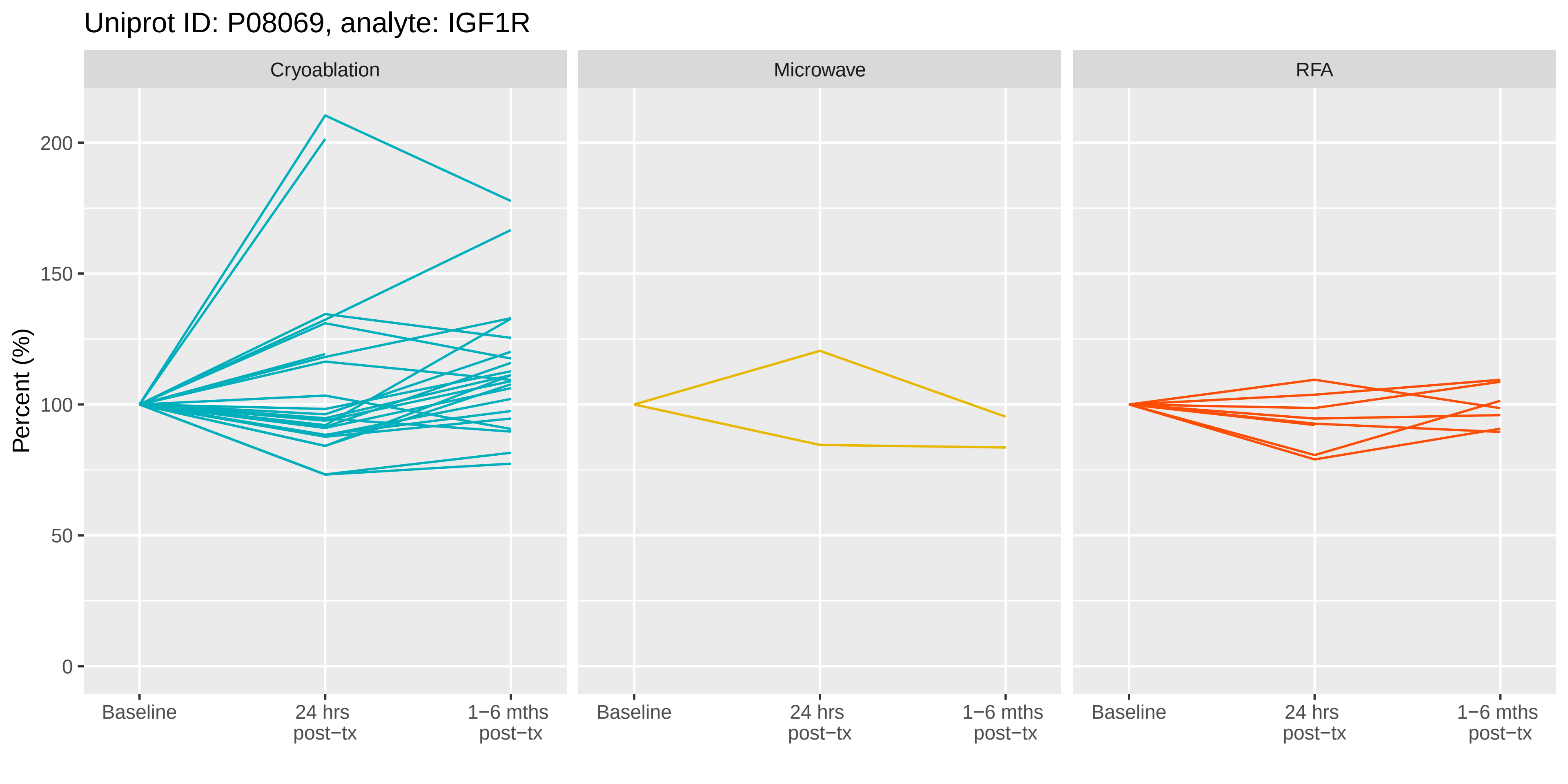
Uniprot ID: P08069, analyte: IGF1R
Cryoablation
Microwave
RFA
Percent (%)
0
50
100
150
200
Baseline
24 hrs post−tx
1−6 mths post−tx

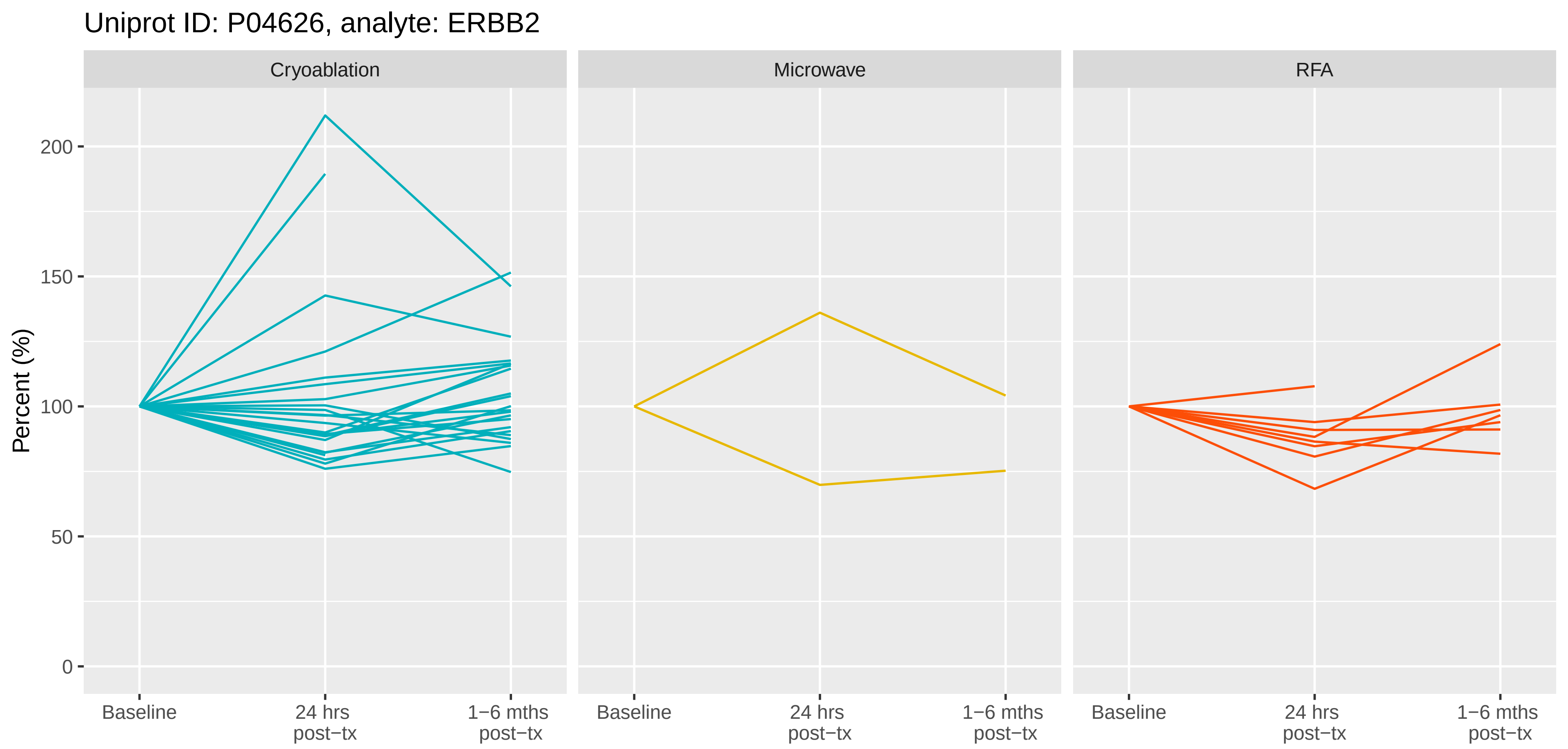
Uniprot ID: P04626, analyte: ERBB2
Cryoablation
Microwave
RFA
Percent (%)
200
150
100
50
0
Baseline
24 hrs post−tx
1−6 mths post−tx

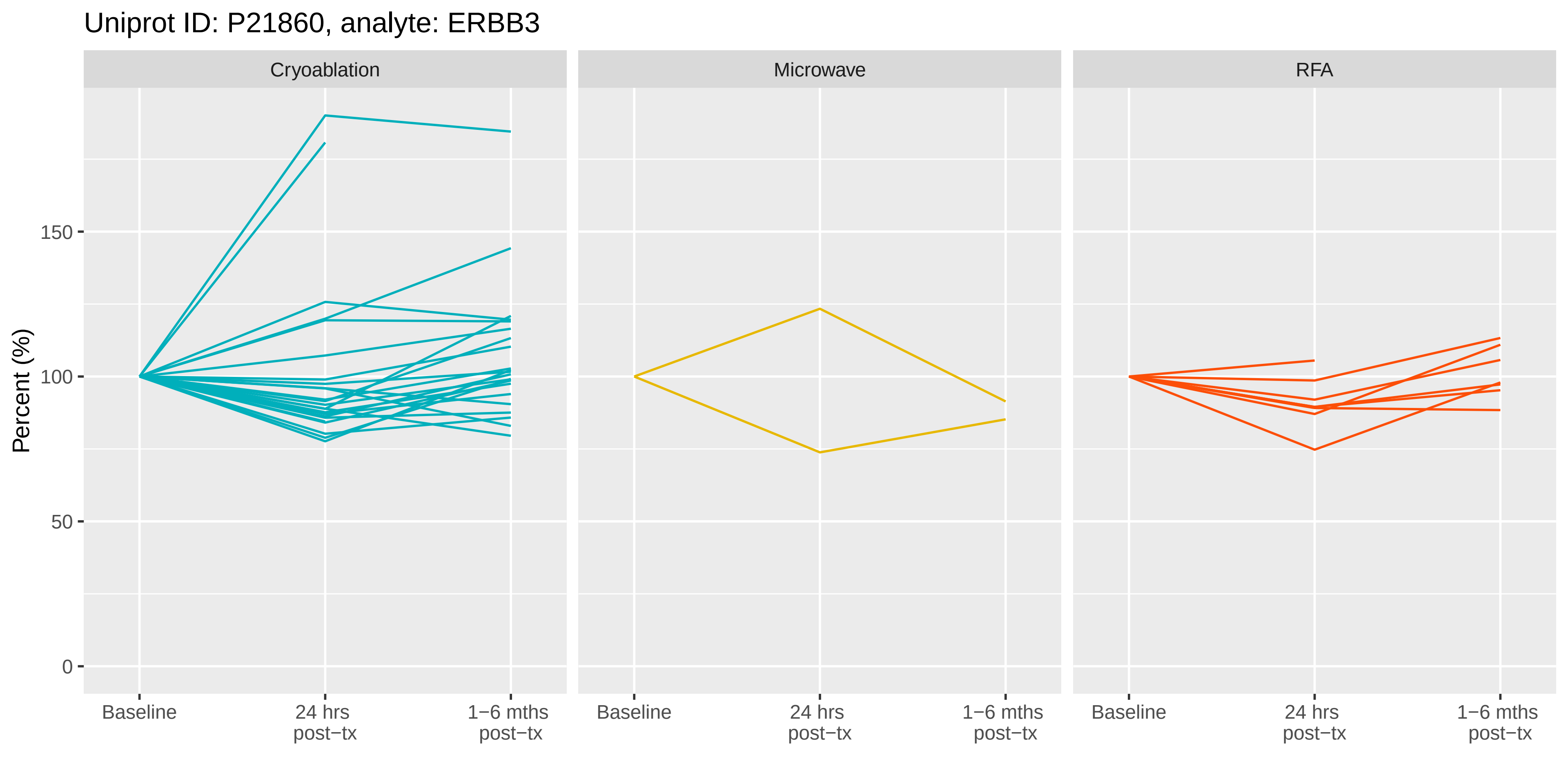

Uniprot ID: P21860, analyte: ERBB3
Cryoablation
Microwave
RFA
Percent (%)
150
100
50
0
Baseline
24 hrs post−tx
1−6 mths post−tx

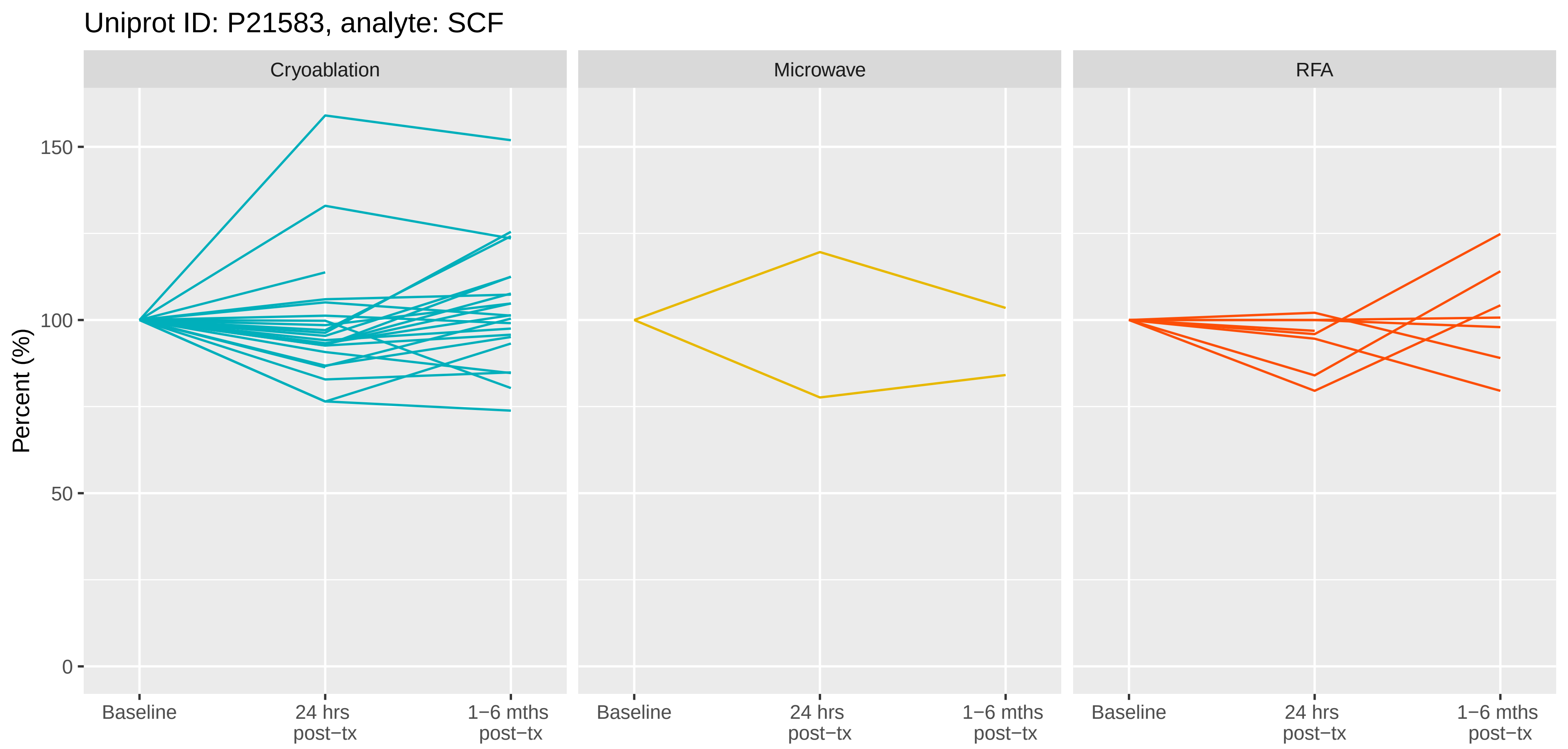
Uniprot ID: P21583, analyte: SCF
Cryoablation
Microwave
RFA
Percent (%)
150
100
50
0
Baseline
24 hrs post−tx
1−6 mths post−tx

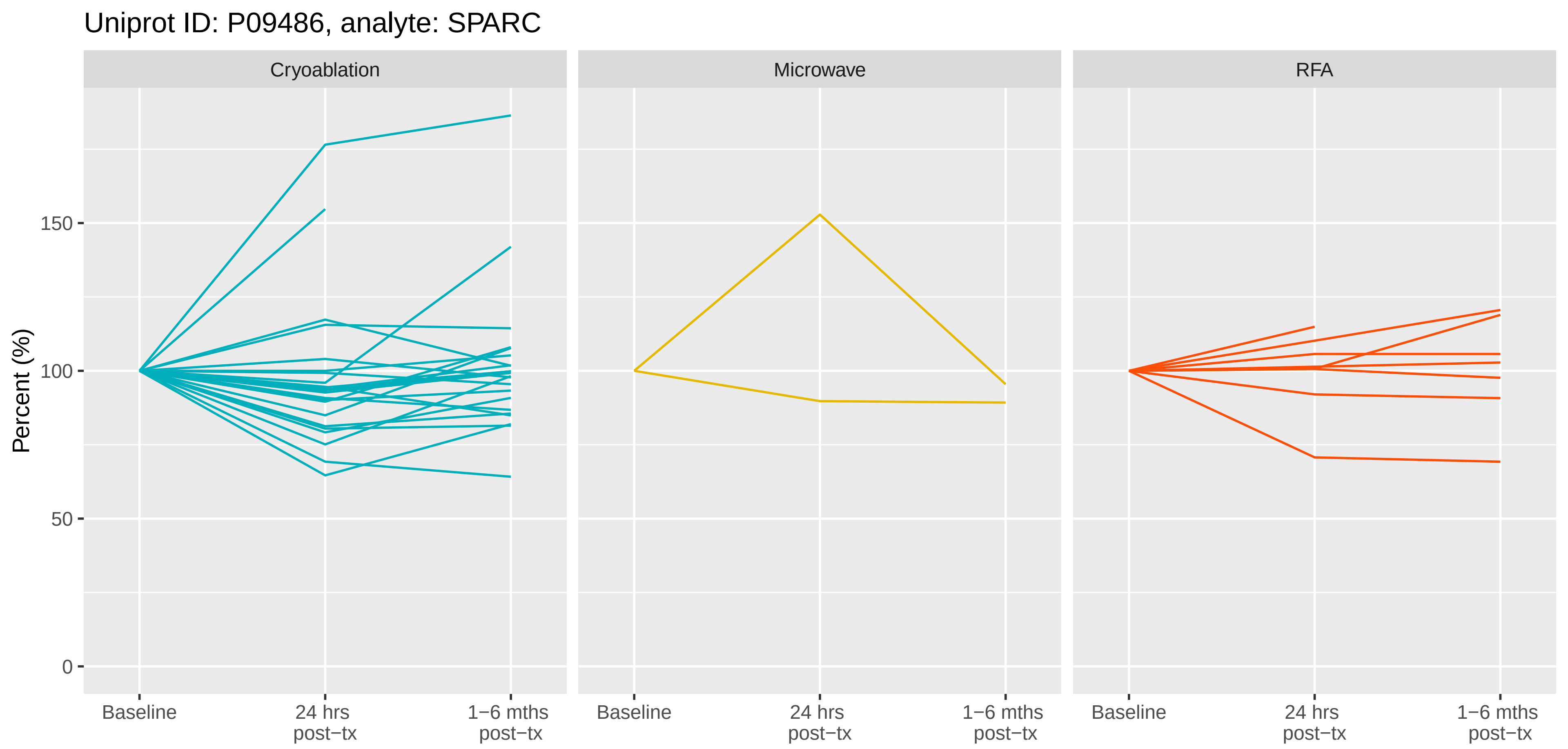

Uniprot ID: P09486, analyte: SPARC
Cryoablation
Microwave
RFA
Percent (%)
150
100
50
0
Baseline
24 hrs post−tx
1−6 mths post−tx

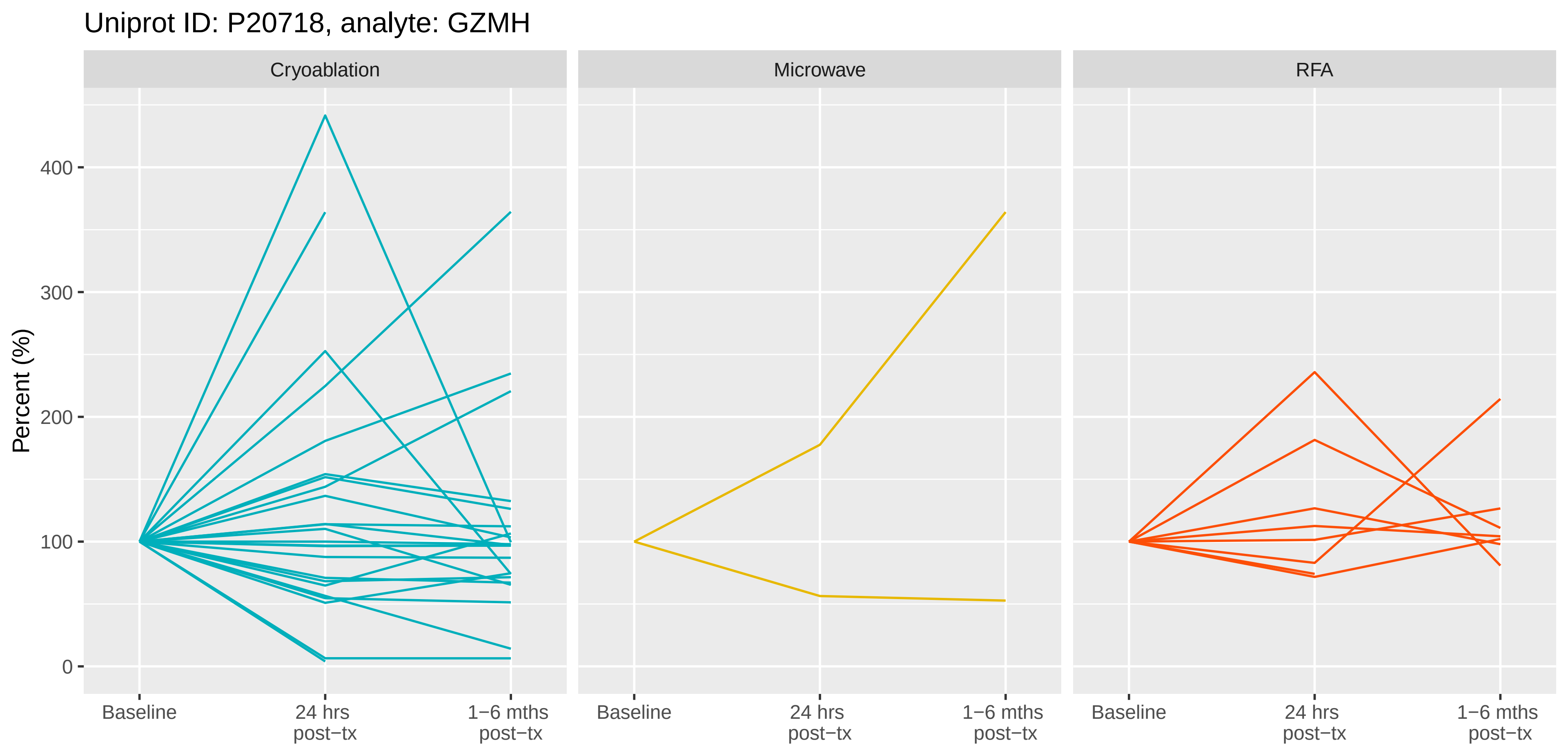

Uniprot ID: P20718, analyte: GZMH
Cryoablation
Microwave
RFA
Percent (%)
400
300
200
100
0
Baseline
24 hrs post−tx
1−6 mths post−tx

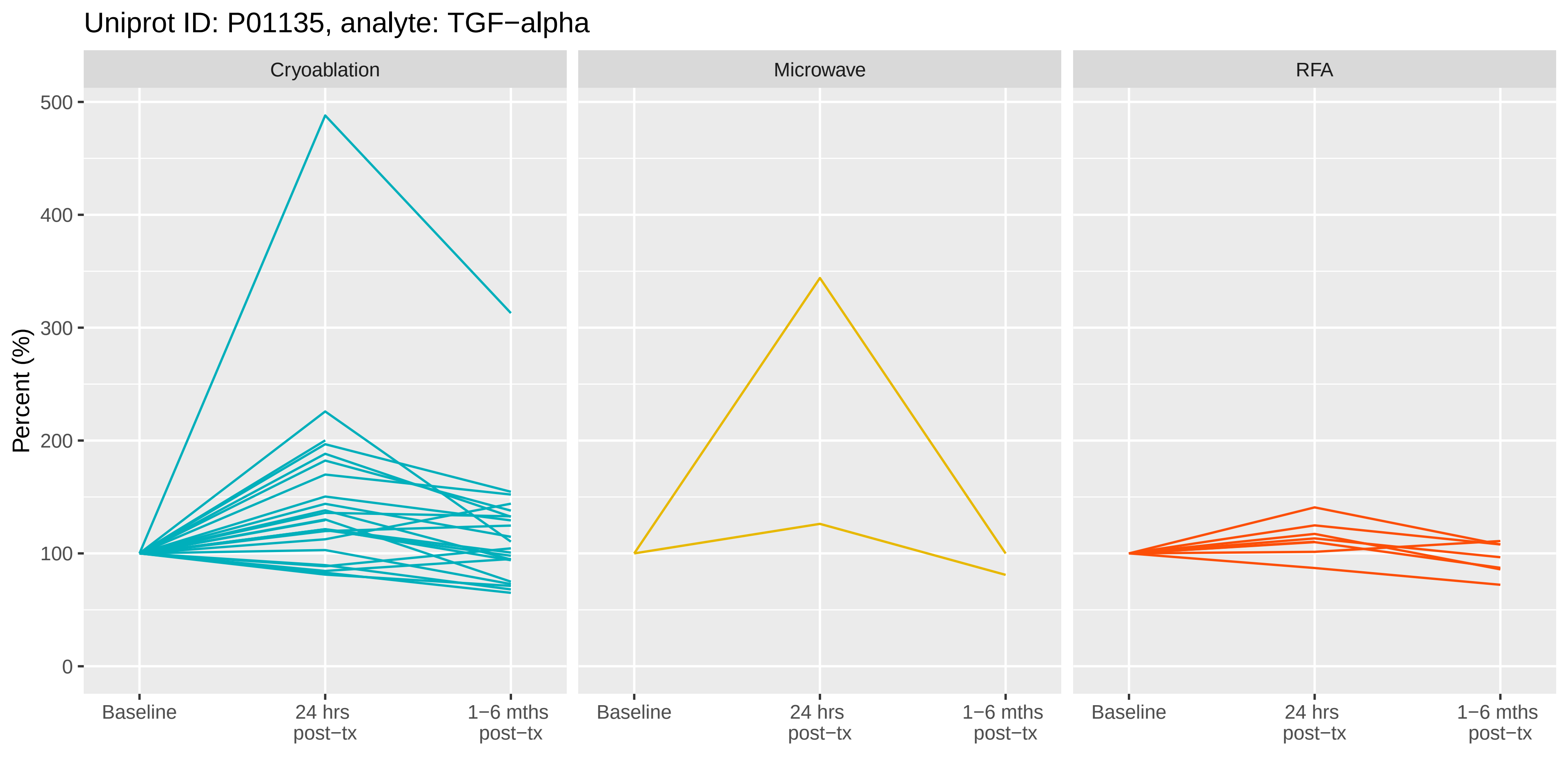
Uniprot ID: P01135, analyte: TGF−alpha
Cryoablation
Microwave
RFA
Percent (%)
500
400
300
200
100
0
Baseline
24 hrs post−tx
1−6 mths post−tx

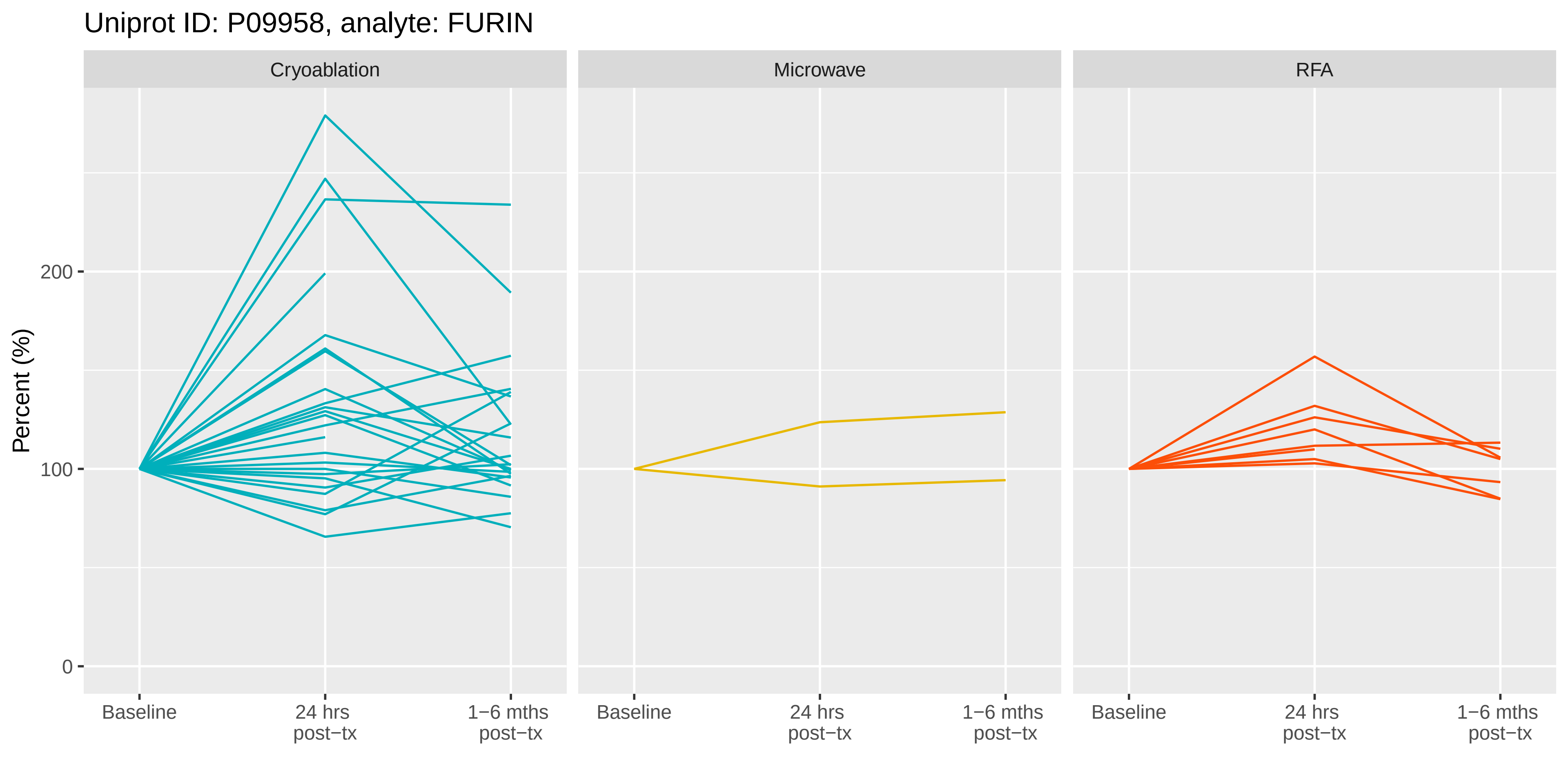
Uniprot ID: P09958, analyte: FURIN
Cryoablation
Microwave
RFA
Percent (%)
200
100
0
Baseline
24 hrs post−tx
1−6 mths post−tx

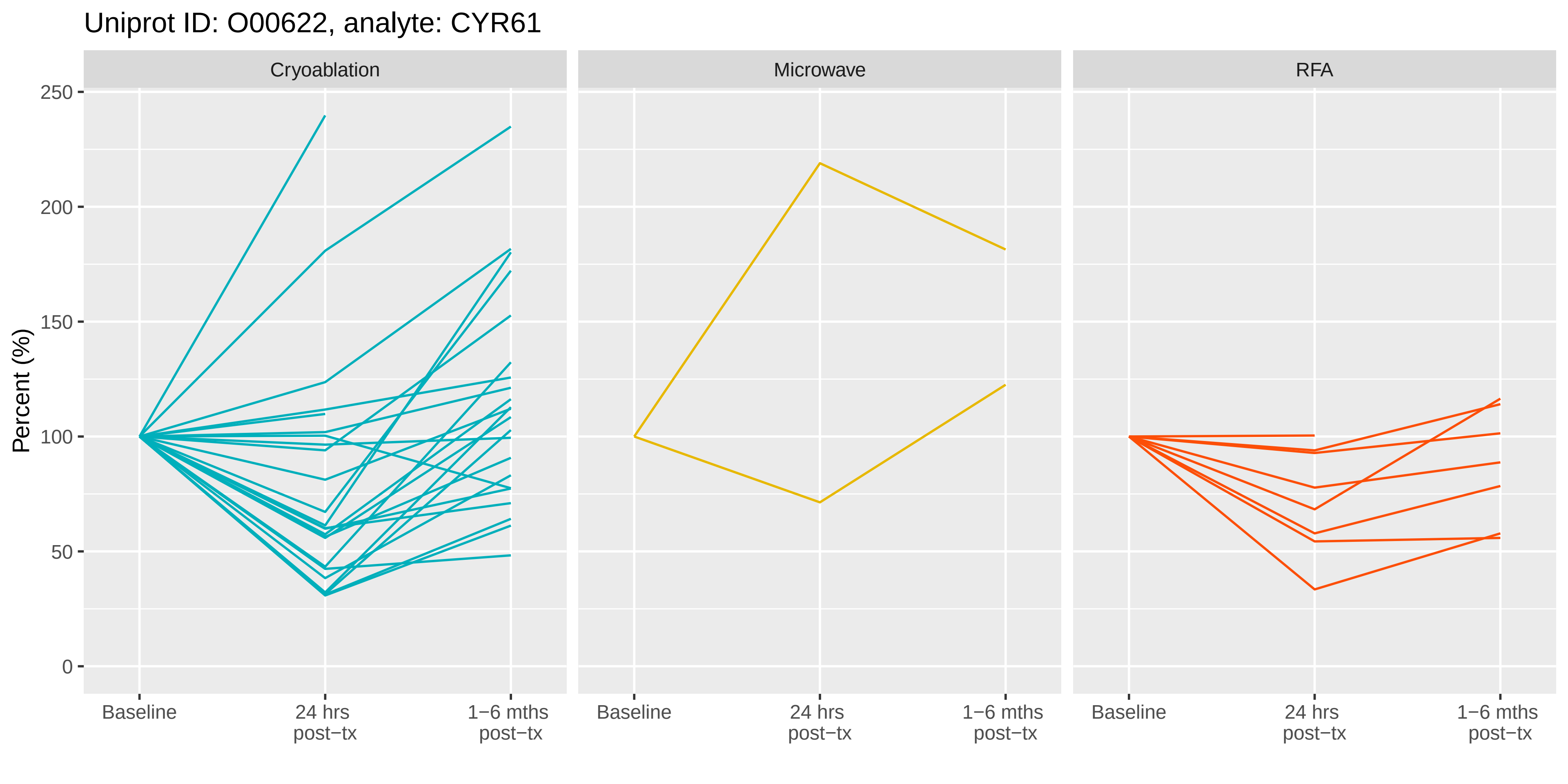

Uniprot ID: O00622, analyte: CYR61
Cryoablation
Microwave
RFA
Percent (%)
250
200
150
100
50
0
Baseline
24 hrs post−tx
1−6 mths post−tx

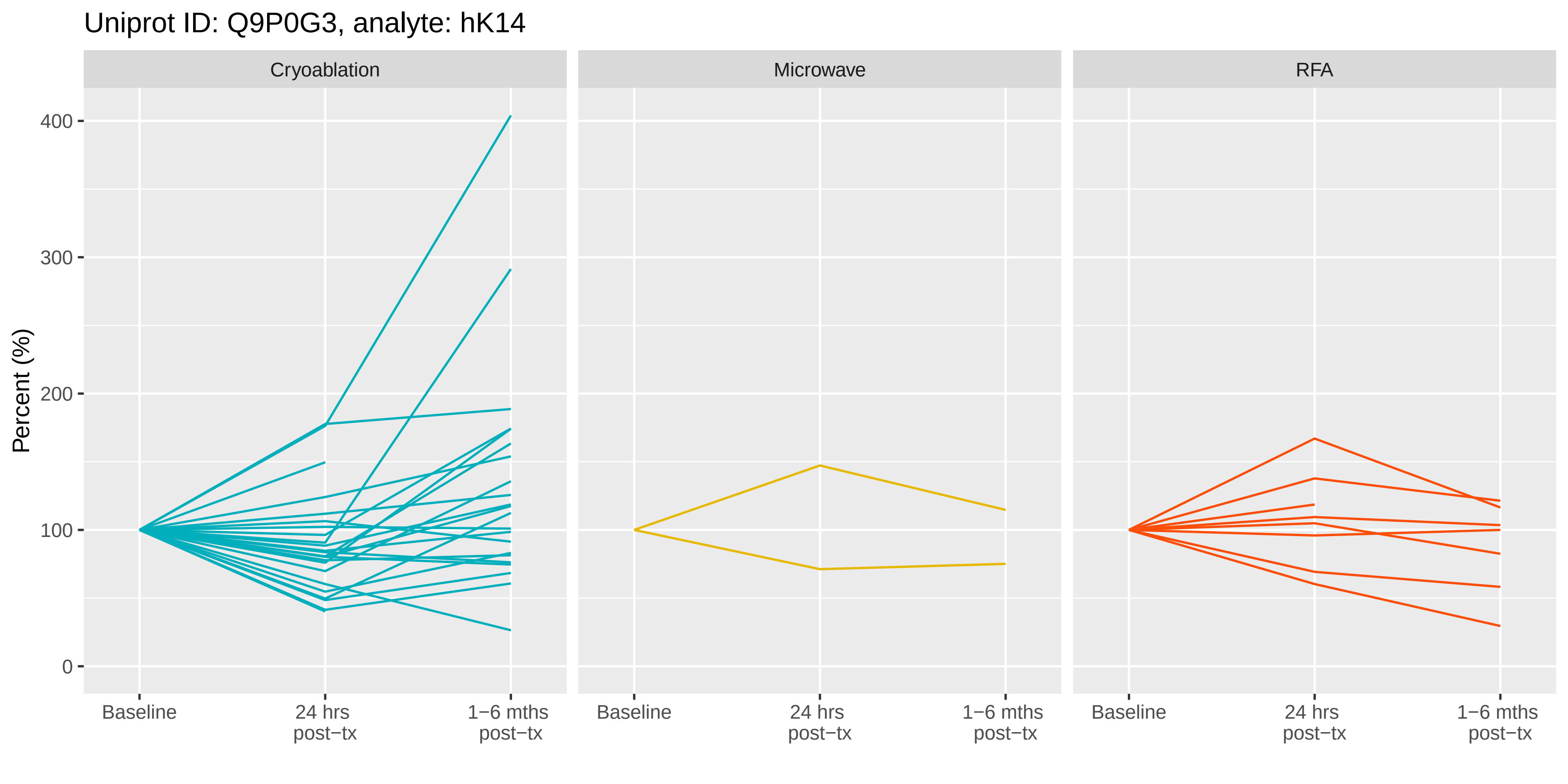
Uniprot ID: Q9P0G3, analyte: hK14
Cryoablation
Microwave
RFA
Percent (%)
400
300
200
100
0
Baseline
24 hrs post−tx
1−6 mths post−tx

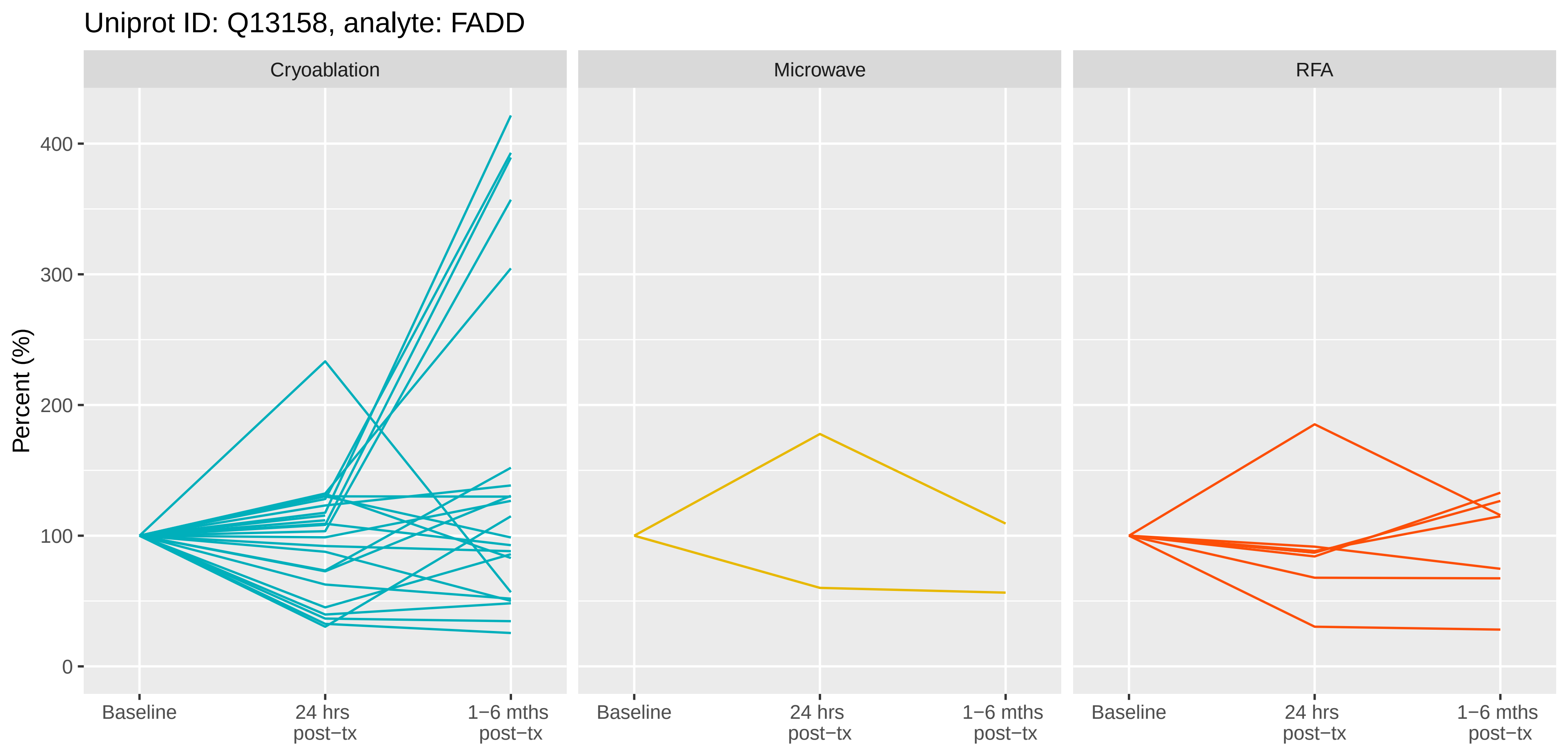

Uniprot ID: Q13158, analyte: FADD
Cryoablation
Microwave
RFA
Percent (%)
400
300
200
100
0
Baseline
24 hrs post−tx
1−6 mths post−tx

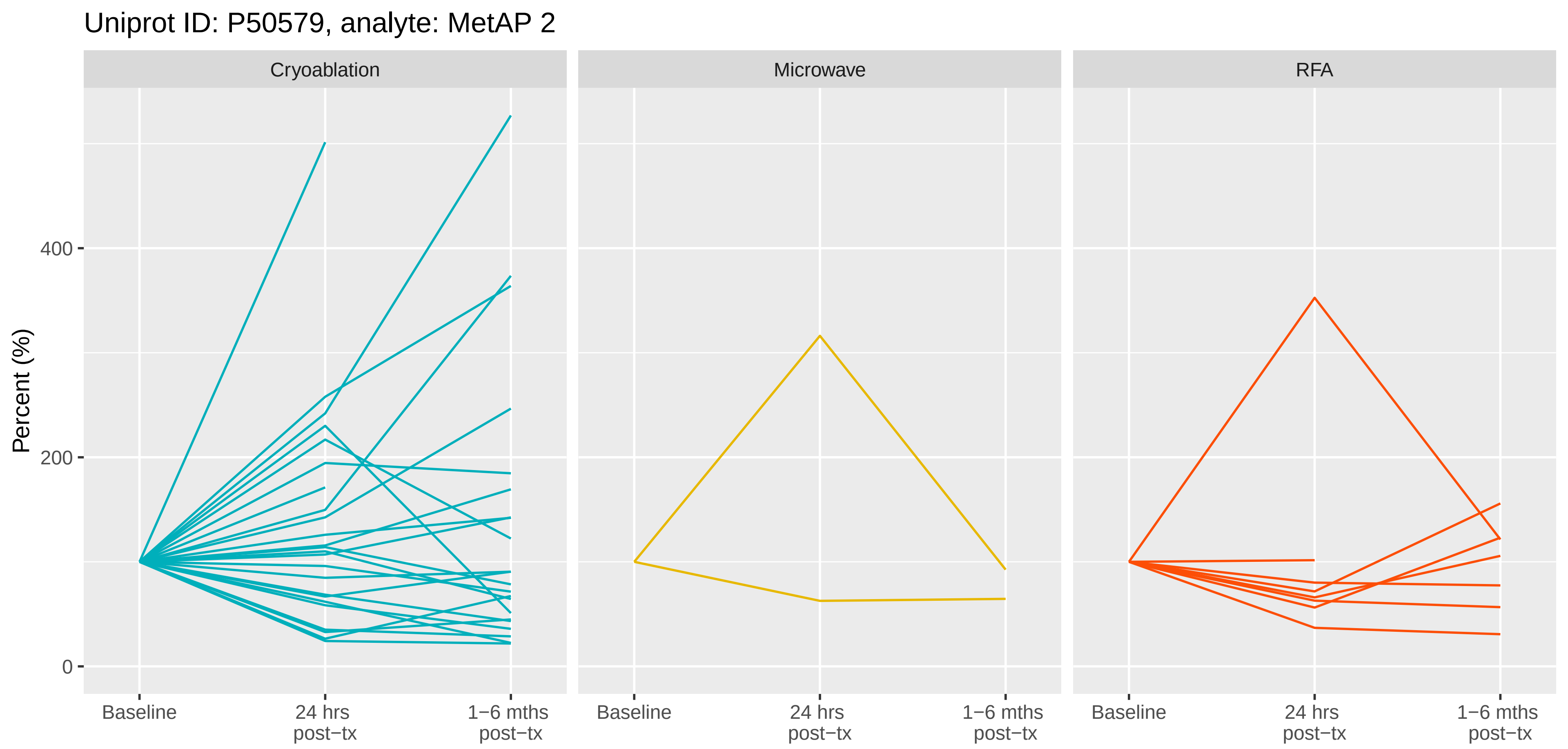
Uniprot ID: P50579, analyte: MetAP 2
Cryoablation
Microwave
RFA
Percent (%)
400
200
0
Baseline
24 hrs post−tx
1−6 mths post−tx

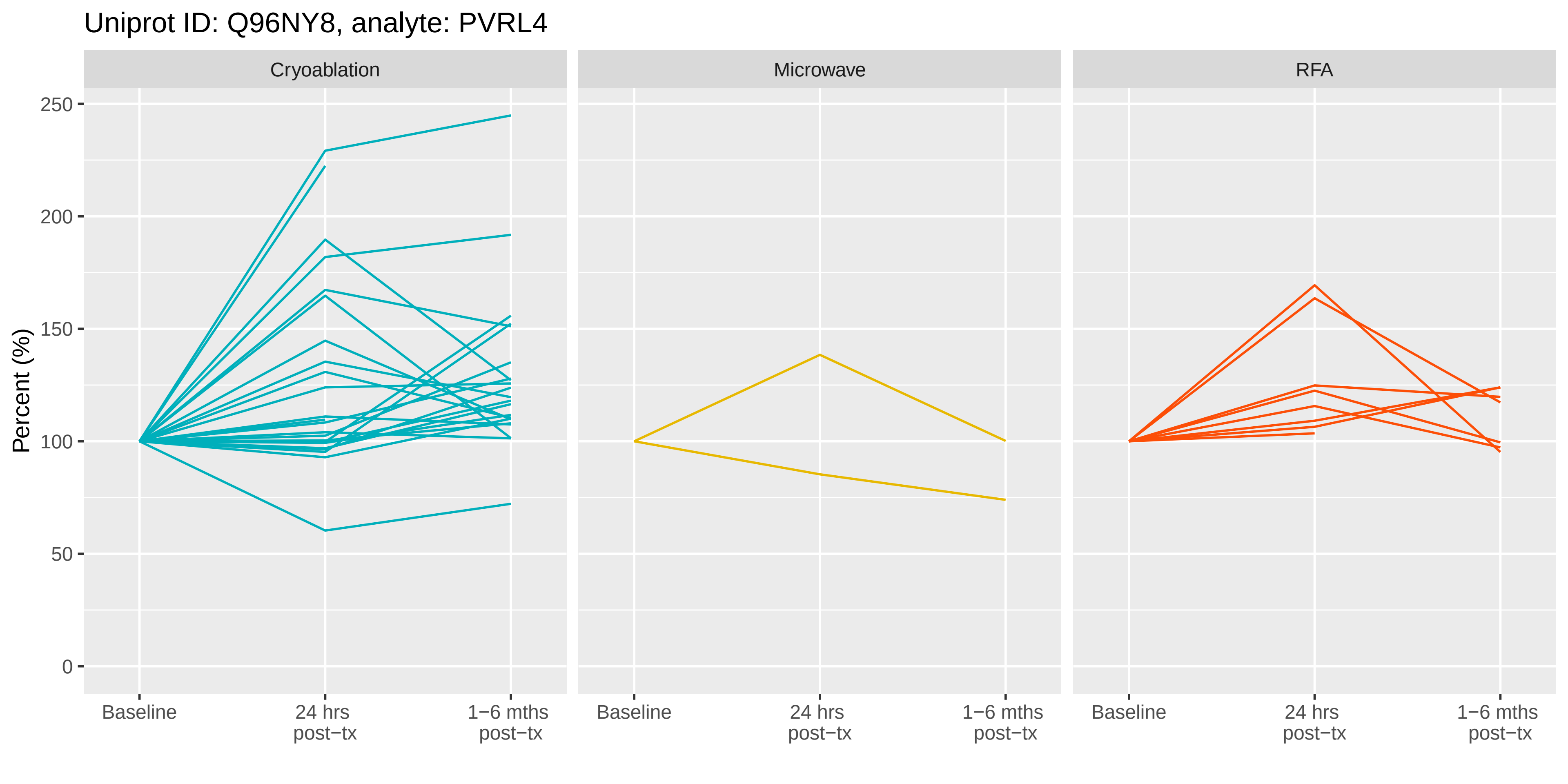
Uniprot ID: Q96NY8, analyte: PVRL4
Cryoablation
Microwave
RFA
Percent (%)
250
200
150
100
50
0
Baseline
24 hrs post−tx
1−6 mths post−tx

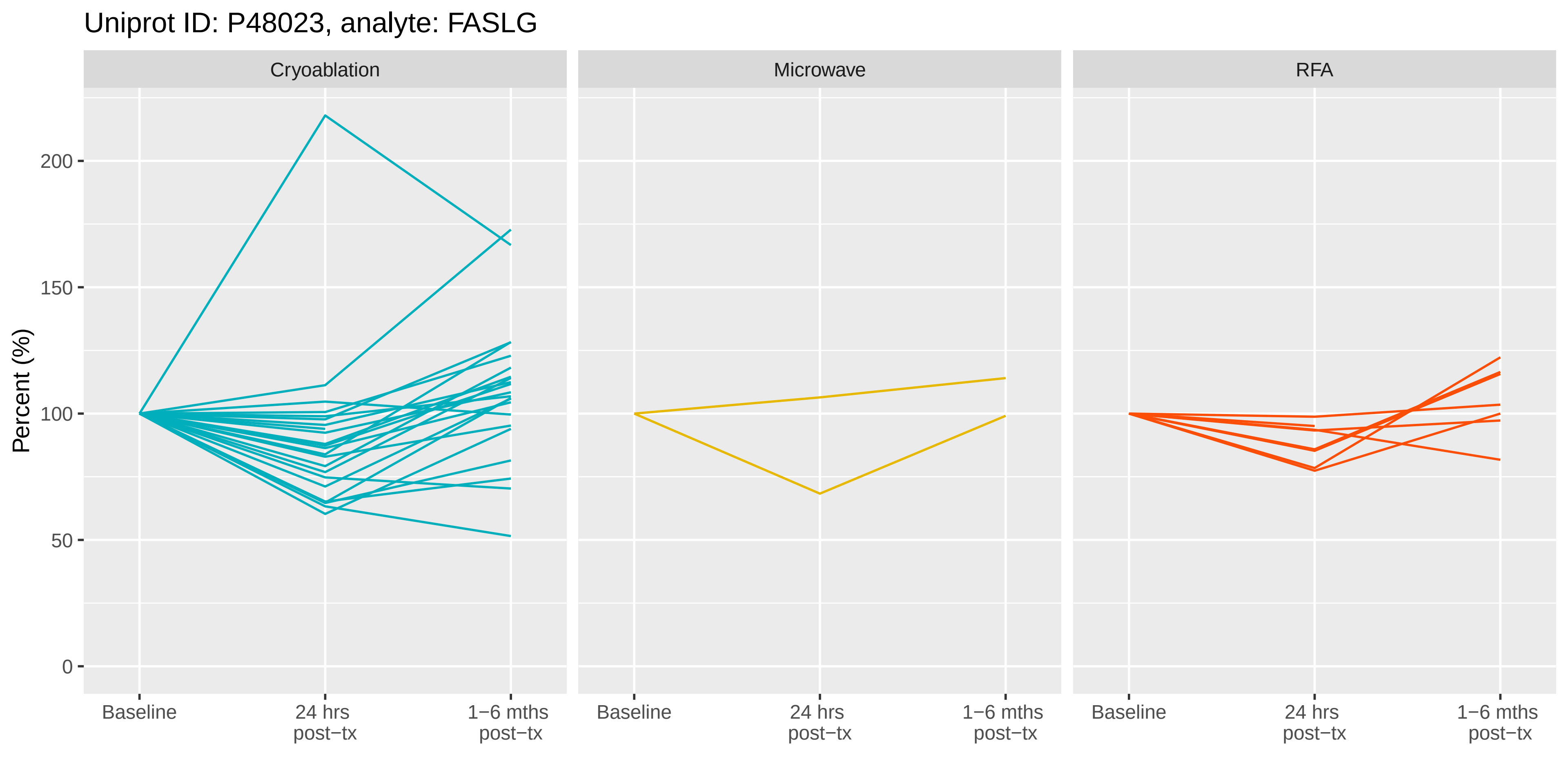

Uniprot ID: P48023, analyte: FASLG
Cryoablation
Microwave
RFA
Percent (%)
200
150
100
50
0
Baseline
24 hrs post−tx
1−6 mths post−tx

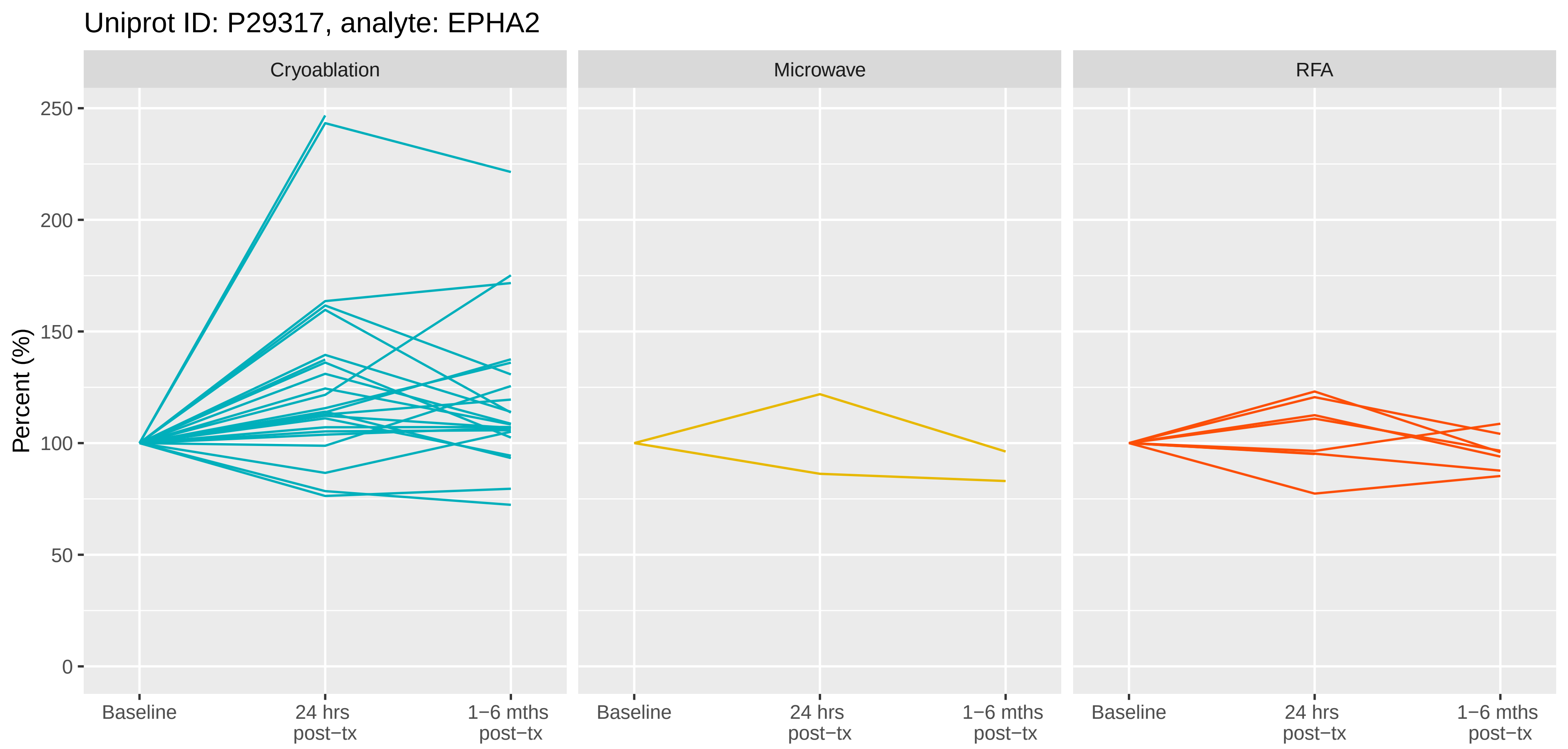

Uniprot ID: P29317, analyte: EPHA2
Cryoablation
Microwave
RFA
Percent (%)
250
200
150
100
50
0
Baseline
24 hrs post−tx
1−6 mths post−tx

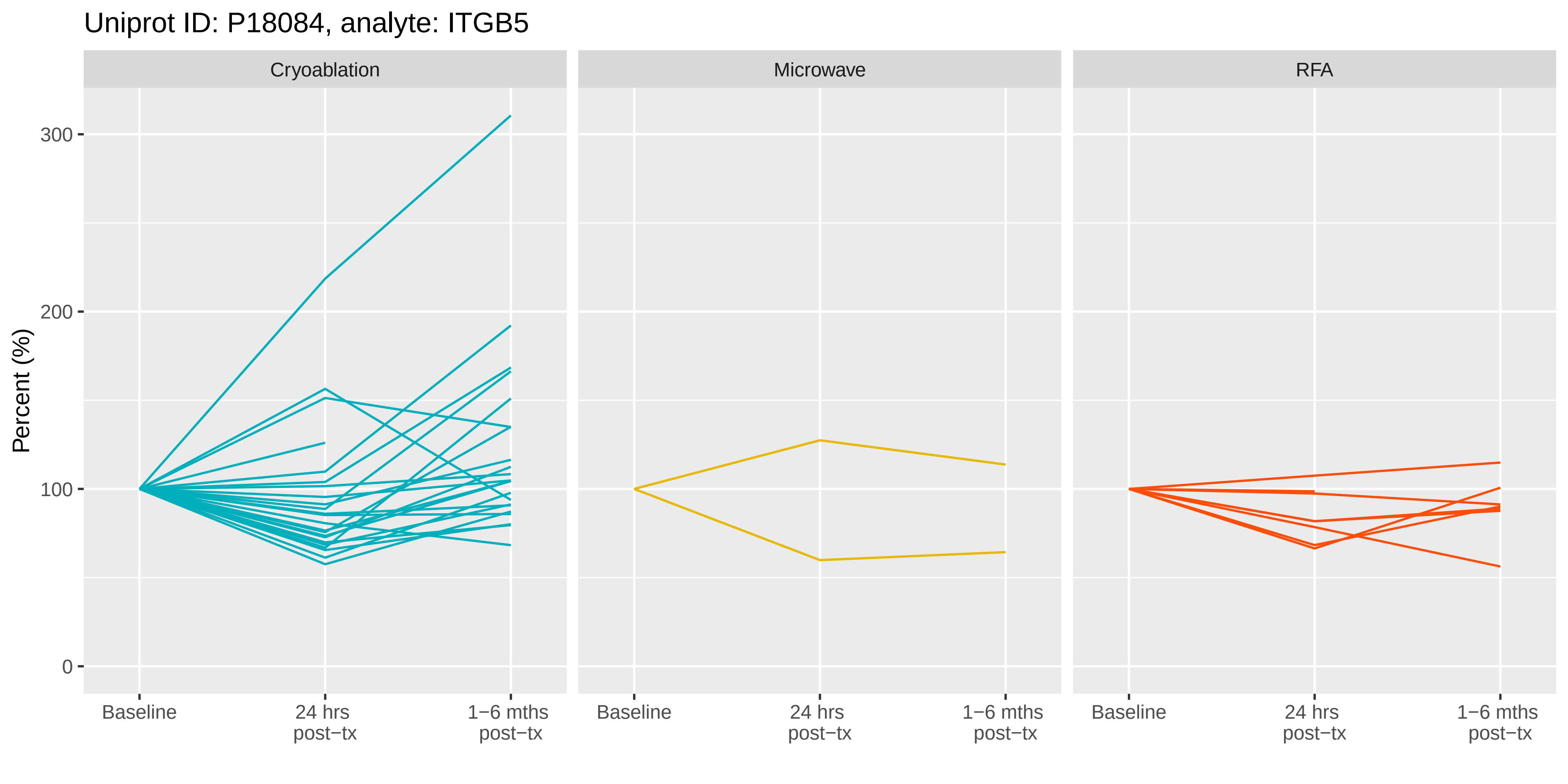

Uniprot ID: P18084, analyte: ITGB5
Cryoablation
Microwave
RFA
Percent (%)
300
200
100
0
Baseline
24 hrs post−tx
1−6 mths post−tx

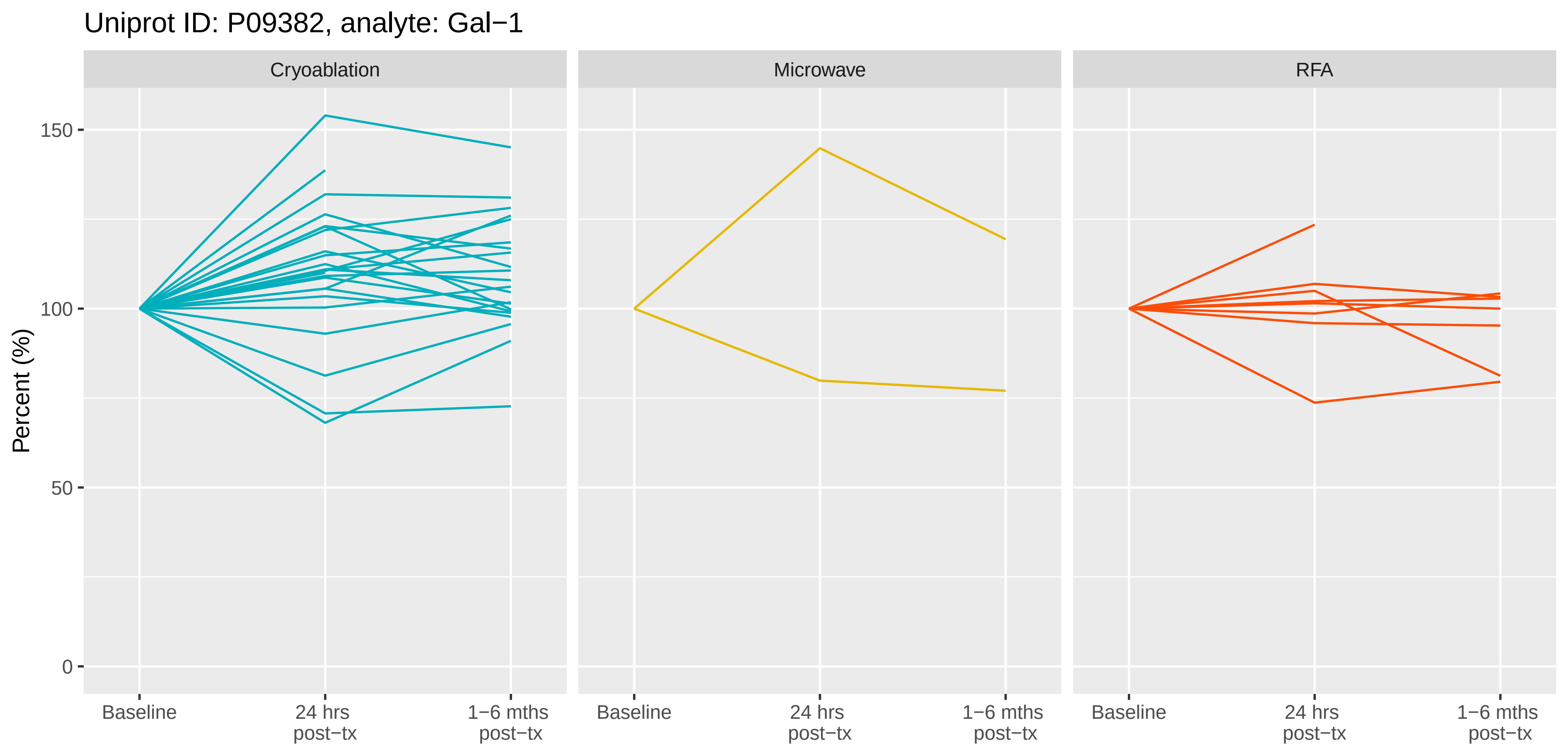
Uniprot ID: P09382, analyte: Gal−1
Cryoablation
Microwave
RFA
Percent (%)
150
100
50
0
Baseline
24 hrs post−tx
1−6 mths post−tx

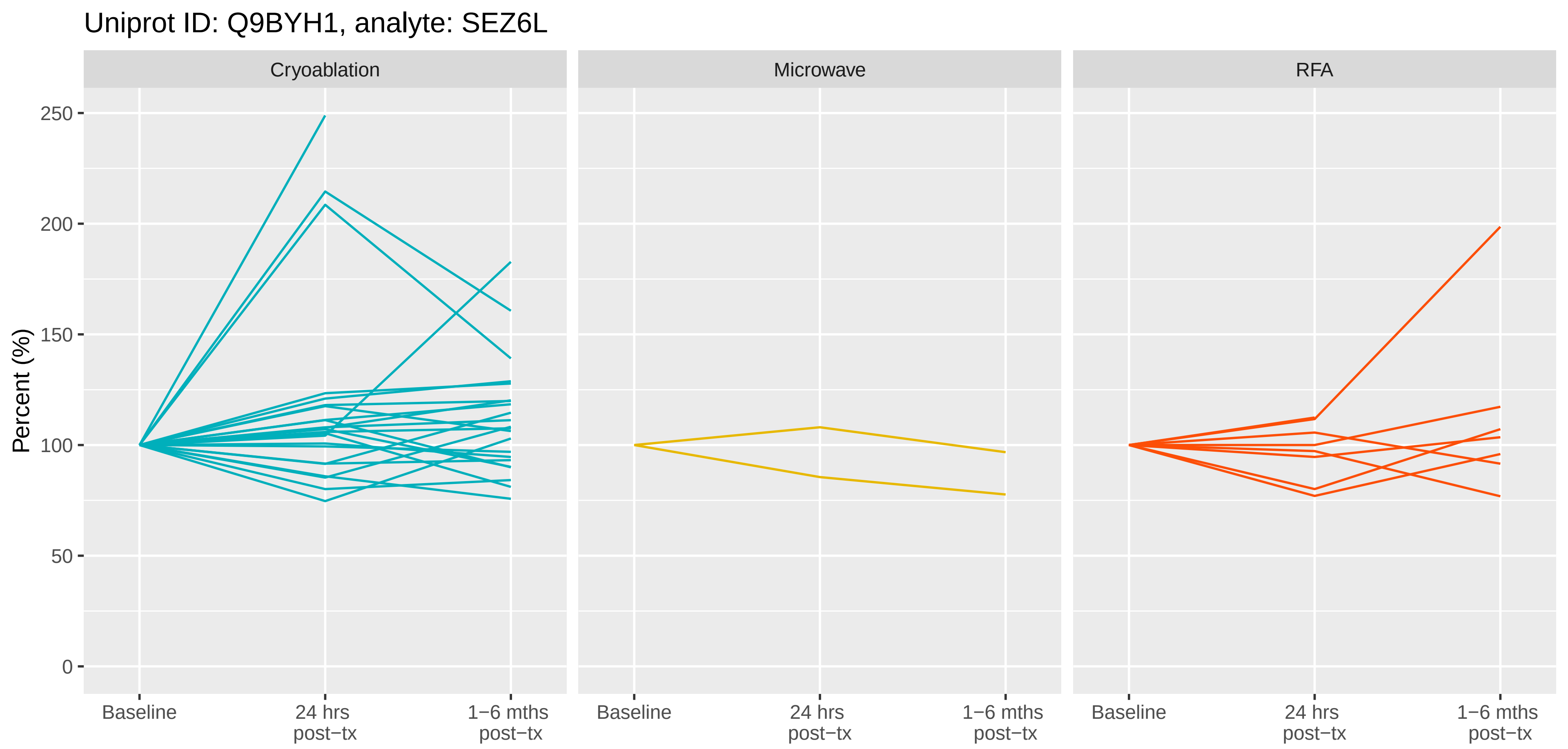

Uniprot ID: Q9BYH1, analyte: SEZ6L
Cryoablation
Microwave
RFA
Percent (%)
250
200
150
100
50
0
Baseline
24 hrs post−tx
1−6 mths post−tx

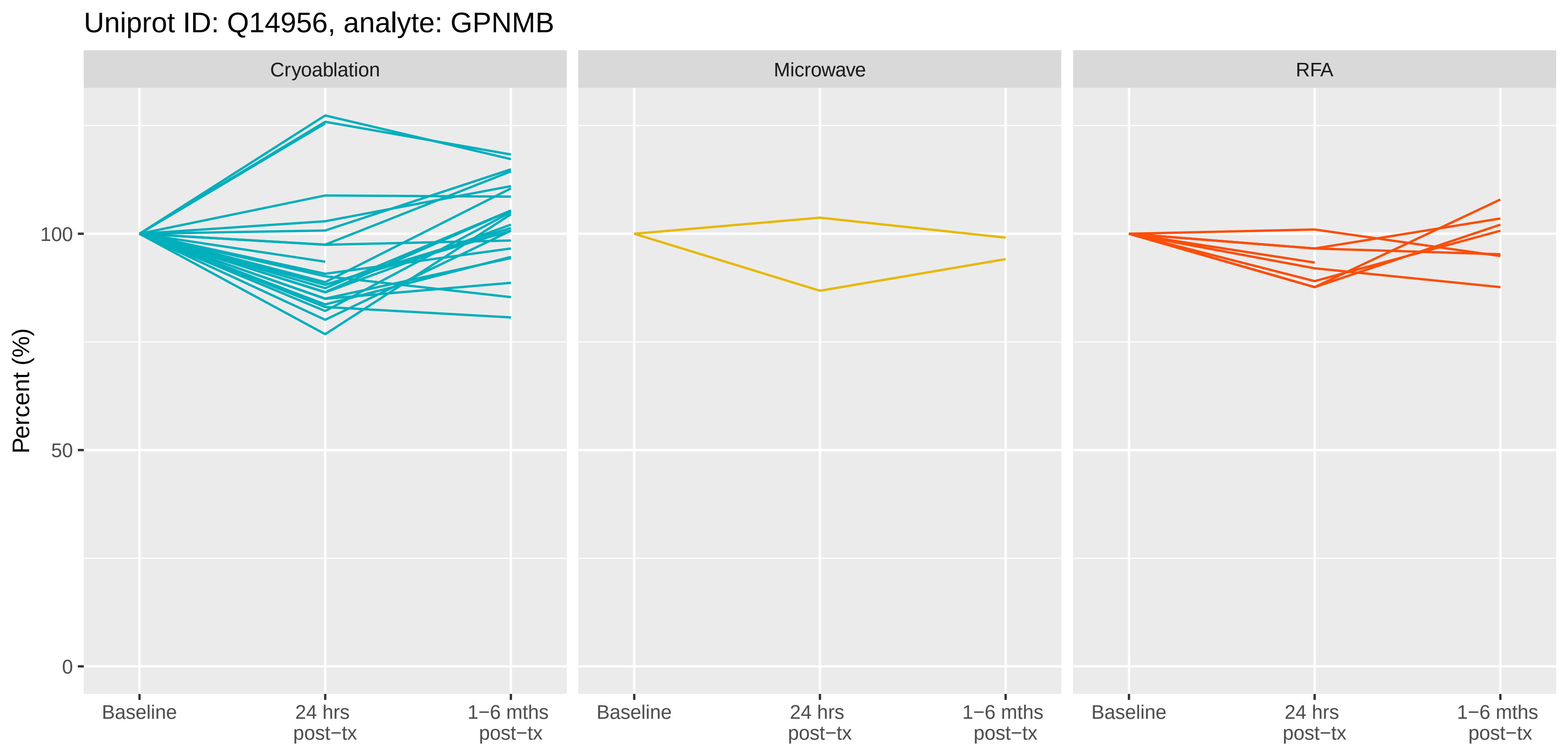

Uniprot ID: Q14956, analyte: GPNMB
Cryoablation
Microwave
RFA
Percent (%)
100
50
0
Baseline
24 hrs post−tx
1−6 mths post−tx

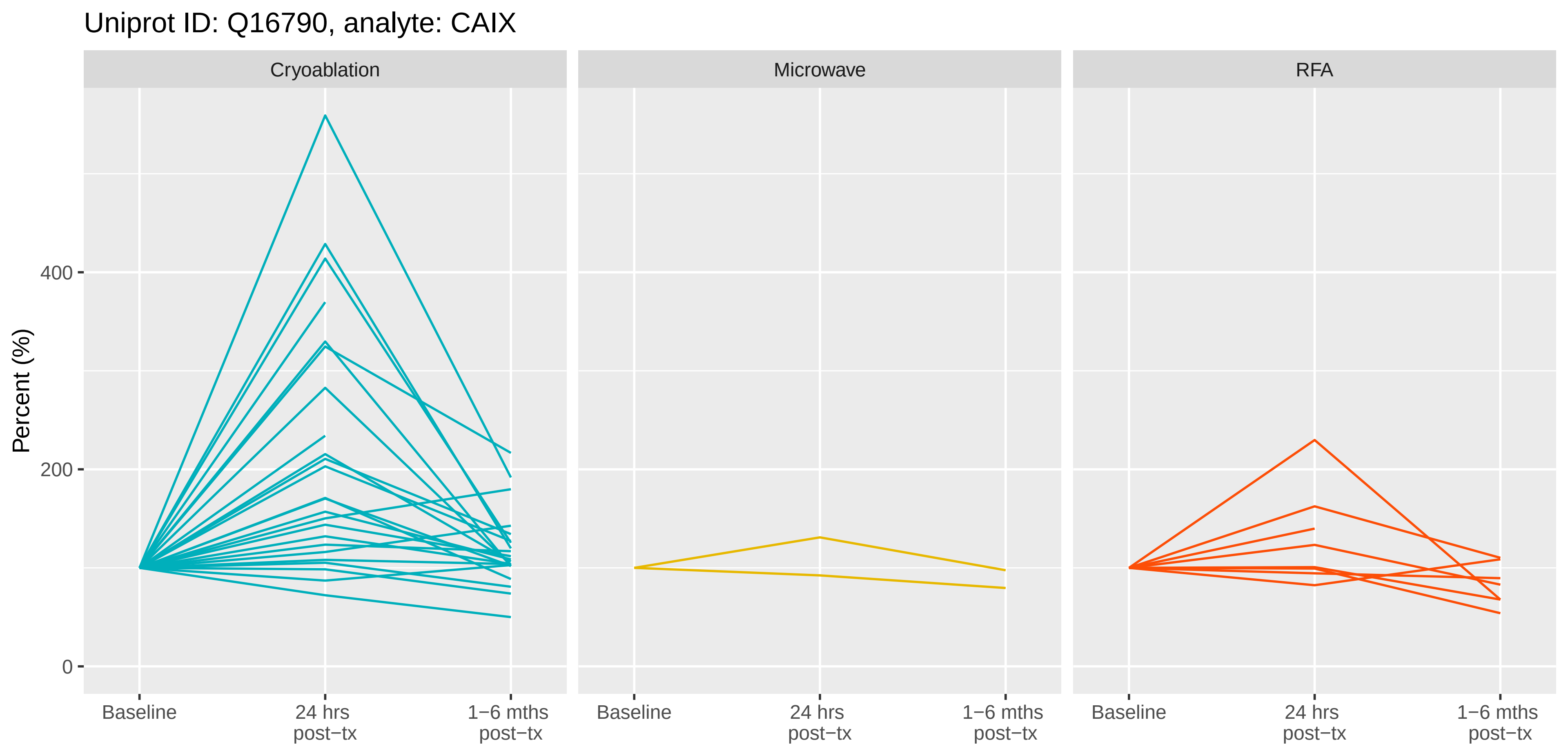

Uniprot ID: Q16790, analyte: CAIX
Cryoablation
Microwave
RFA
Percent (%)
400
200
0
Baseline
24 hrs post−tx
1−6 mths post−tx

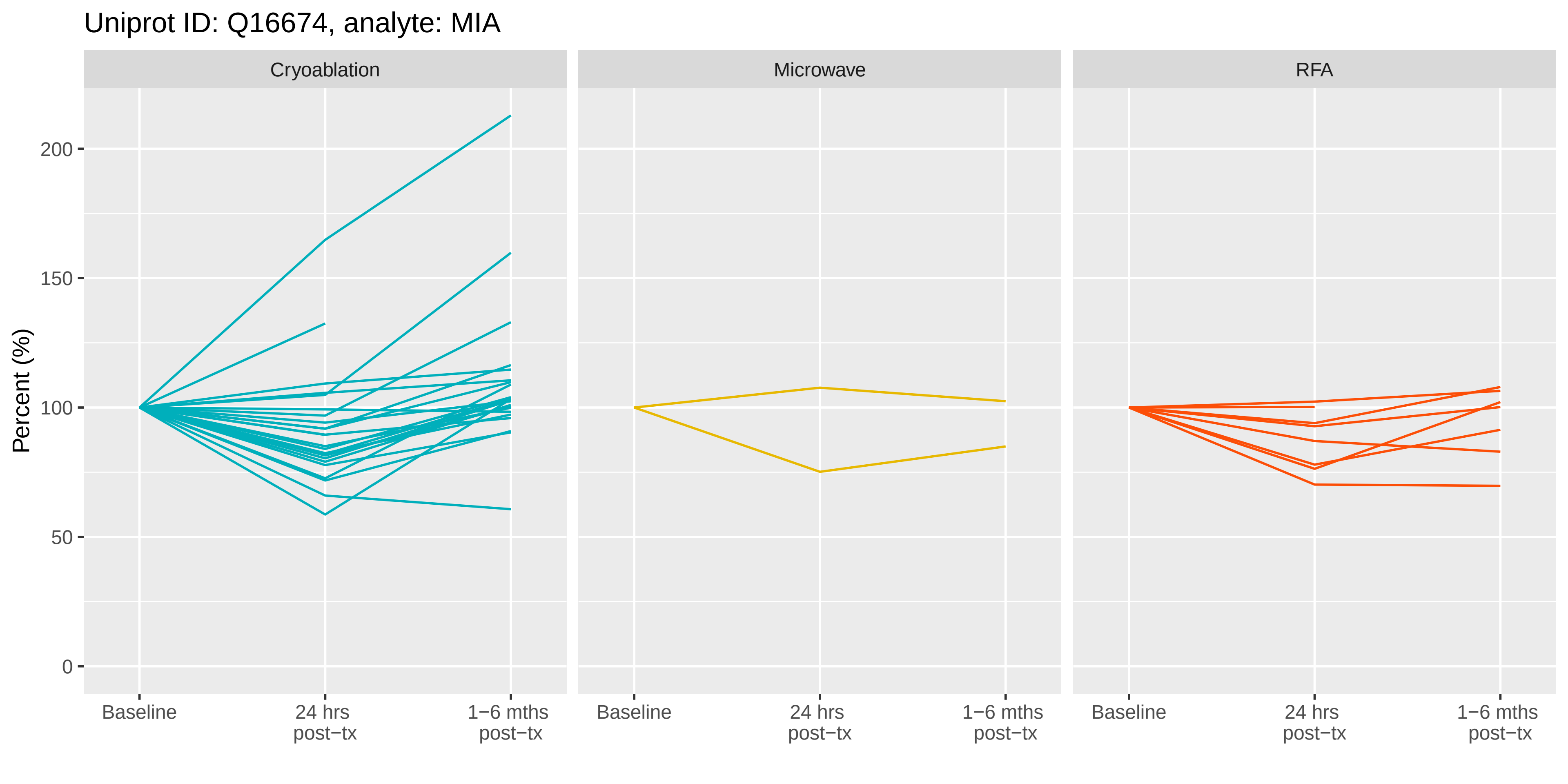
Uniprot ID: Q16674, analyte: MIA
Cryoablation
Microwave
RFA
Percent (%)
0
50
100
150
200
Baseline
24 hrs post−tx
1−6 mths post−tx

Uniprot ID: O60911, analyte: CTSV

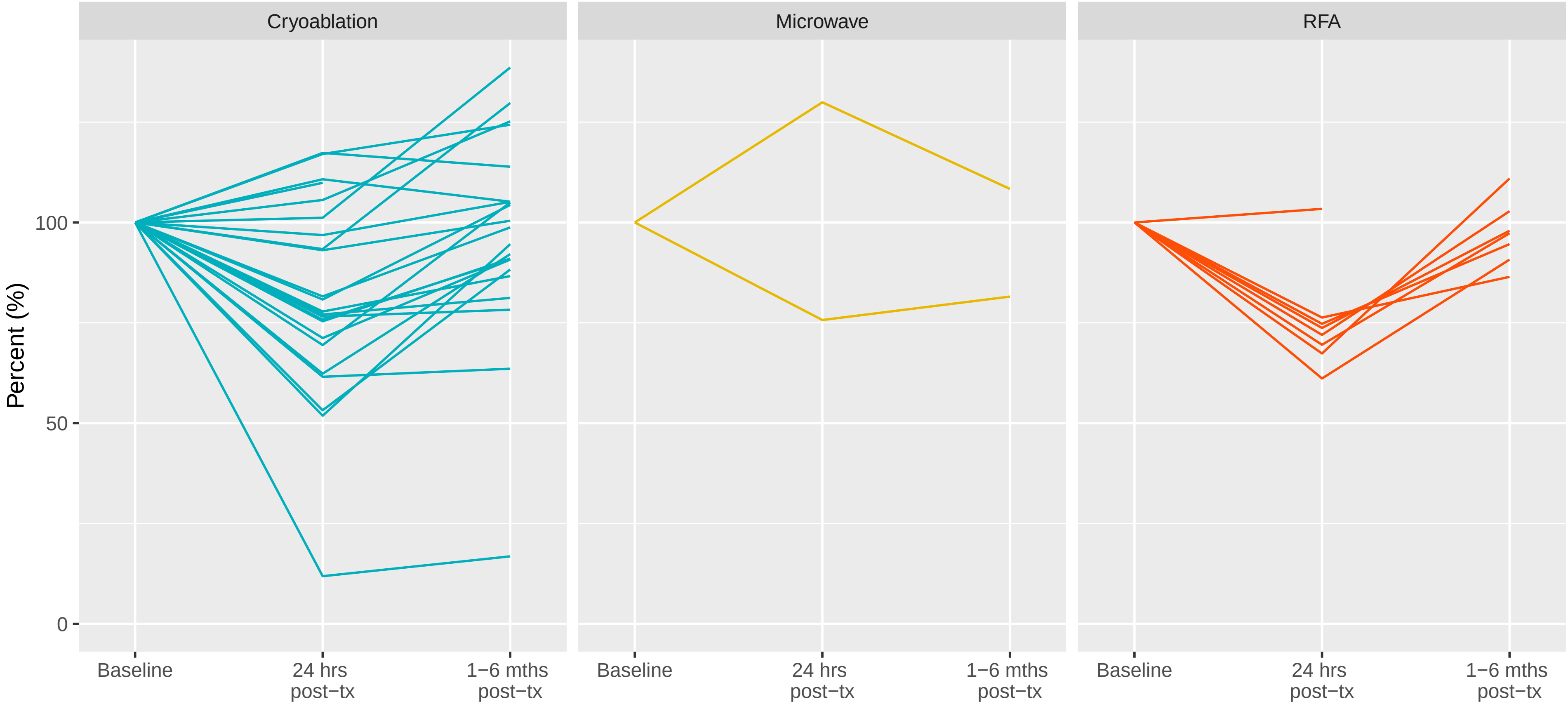

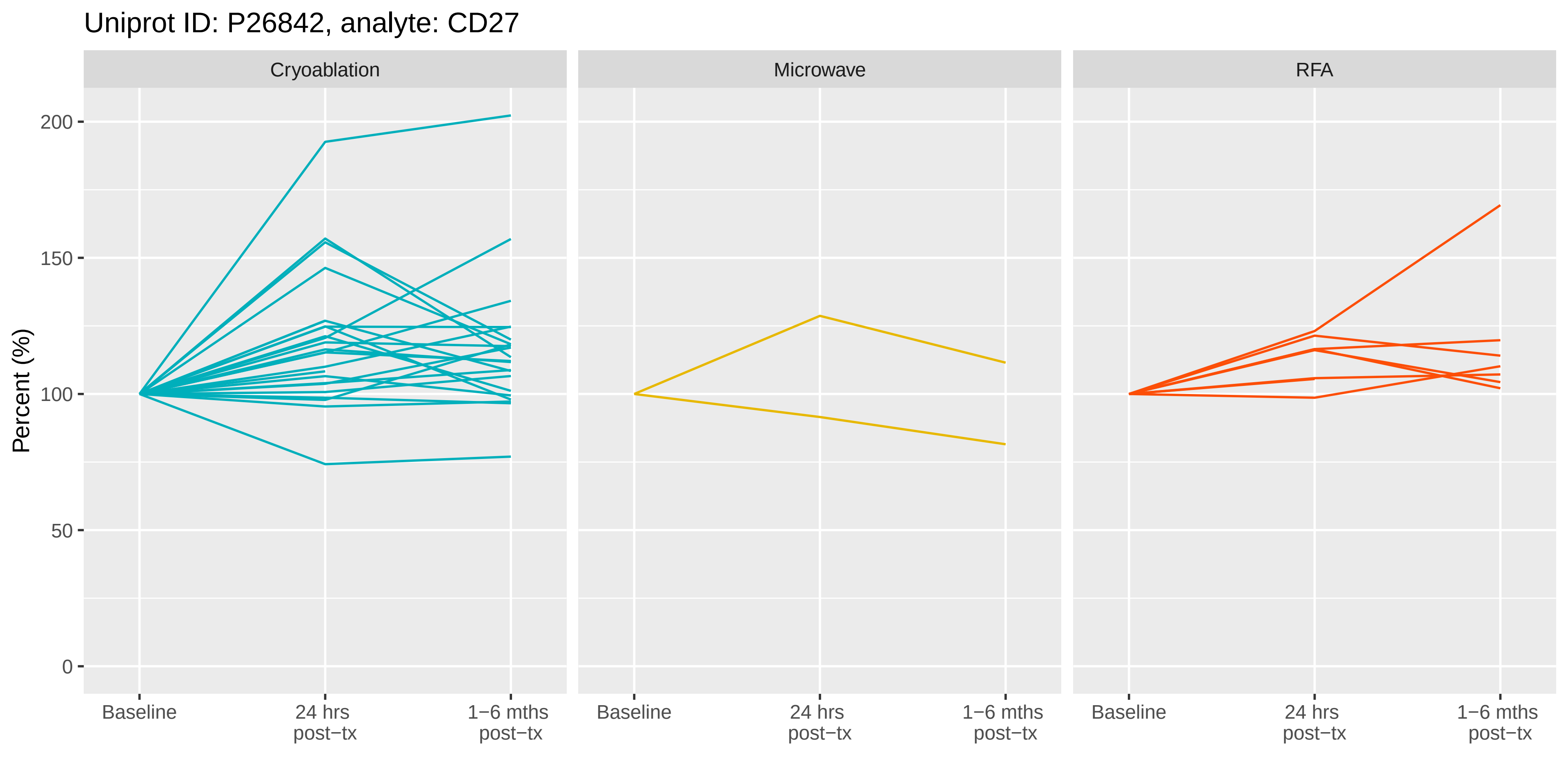
Uniprot ID: P26842, analyte: CD27
Cryoablation
Microwave
RFA
Percent (%)
200
150
100
50
0
Baseline
24 hrs post−tx
1−6 mths post−tx

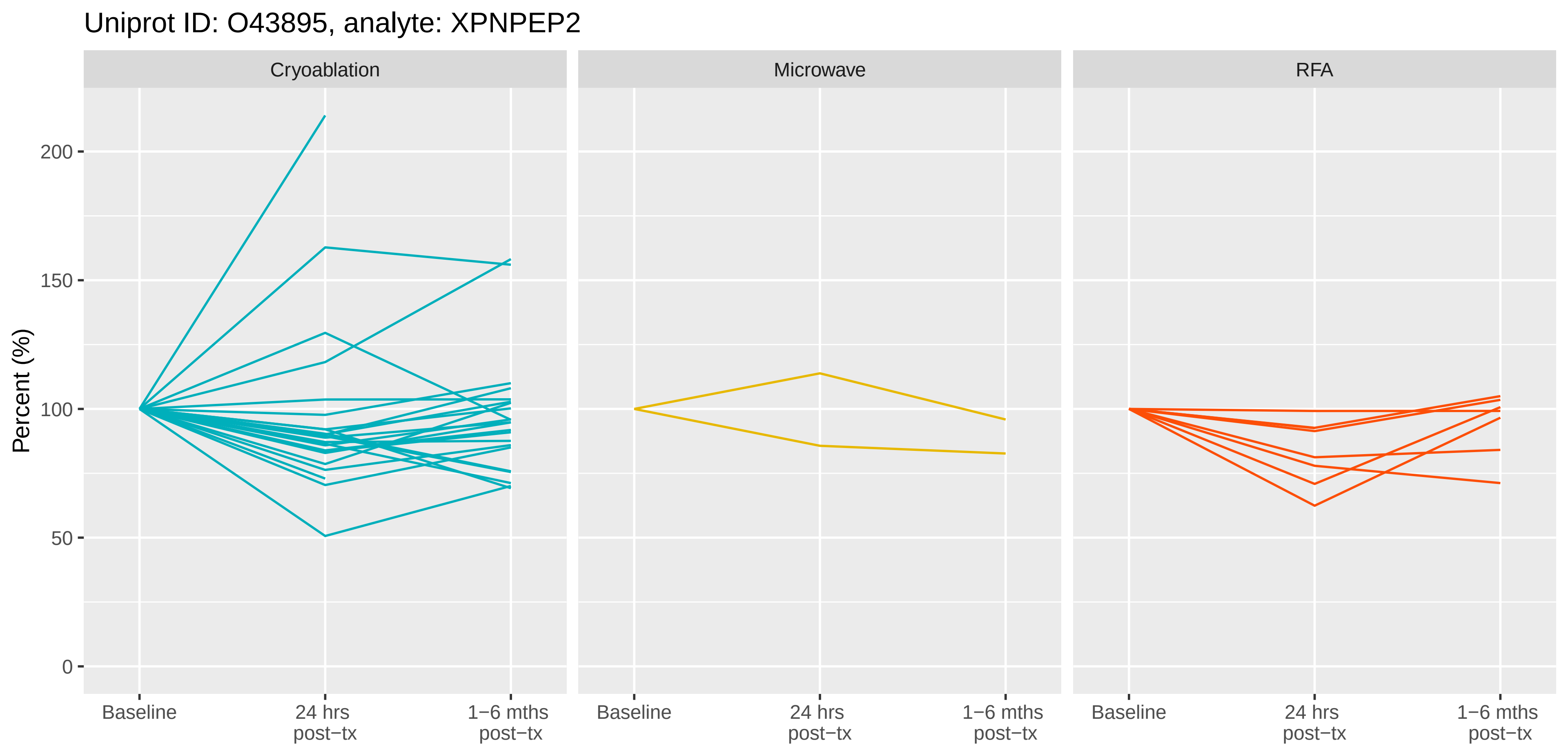
Uniprot ID: O43895, analyte: XPNPEP2
Cryoablation
Microwave
RFA
Percent (%)
200
150
100
50
0
Baseline
24 hrs post−tx
1−6 mths post−tx

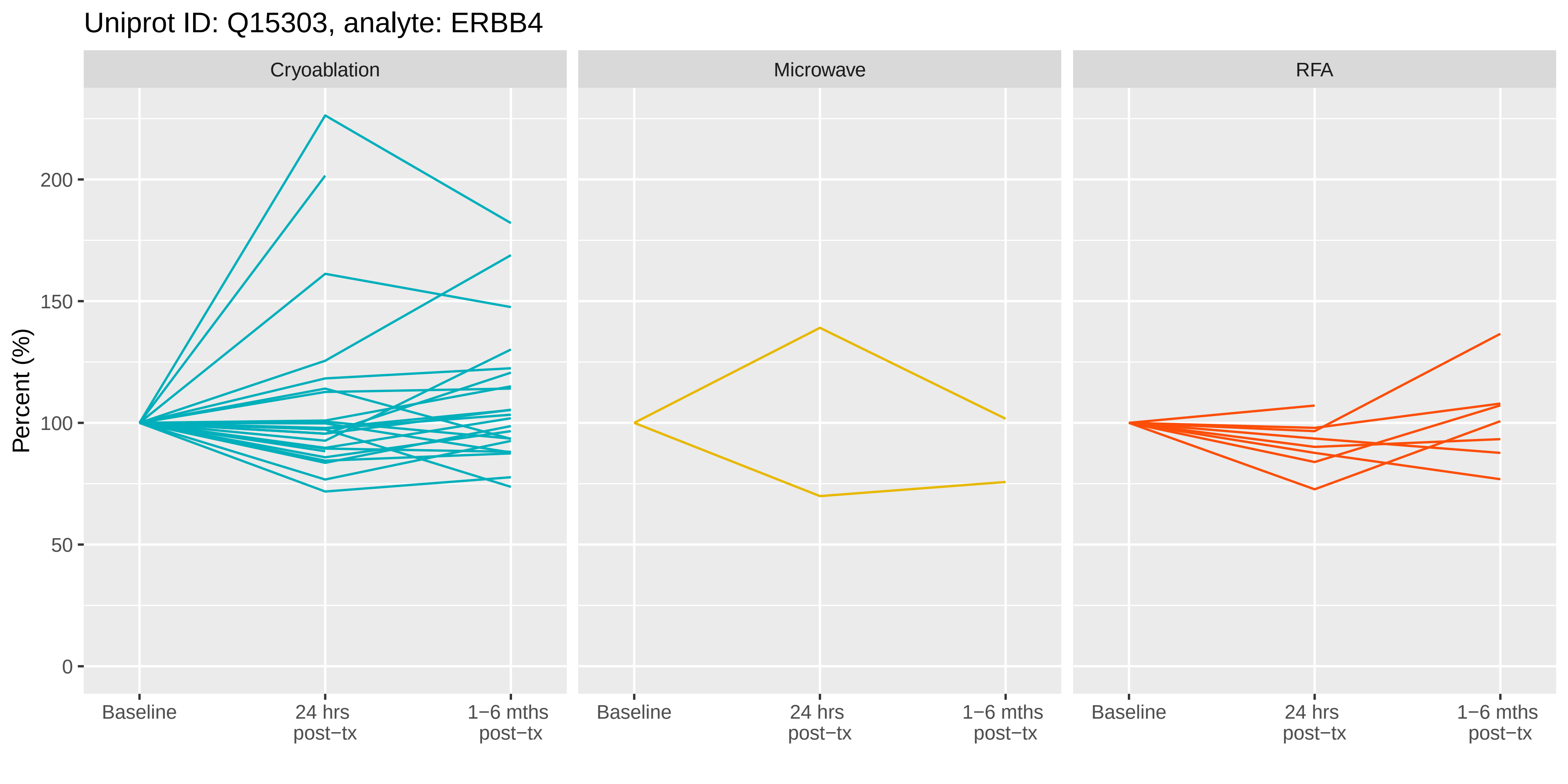

Uniprot ID: Q15303, analyte: ERBB4
Cryoablation
Microwave
RFA
Percent (%)
0
50
100
150
200
Baseline
24 hrs post−tx
1−6 mths post−tx

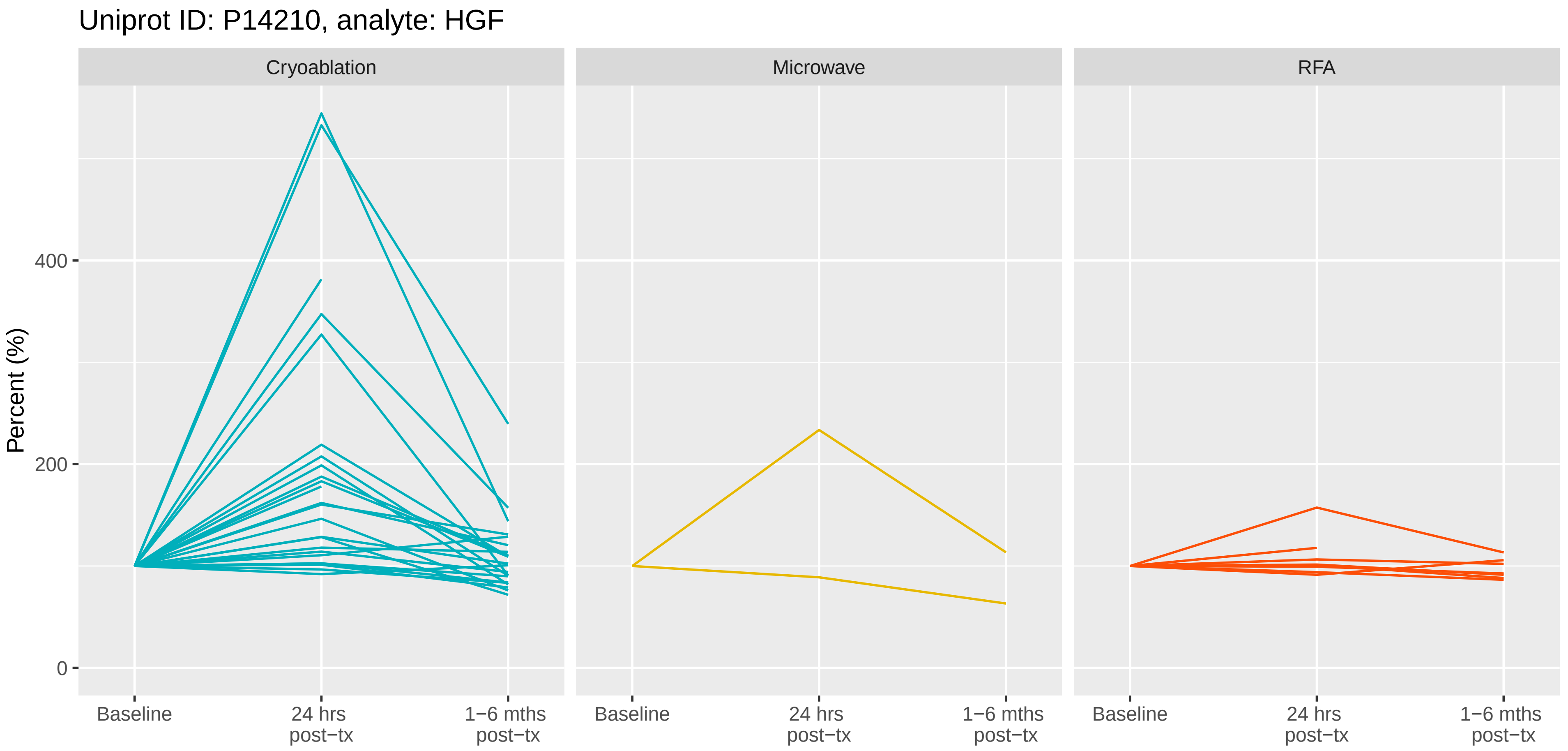

Uniprot ID: P14210, analyte: HGF
Cryoablation
Microwave
RFA
Percent (%)
400
200
0
Baseline
24 hrs post−tx
1−6 mths post−tx

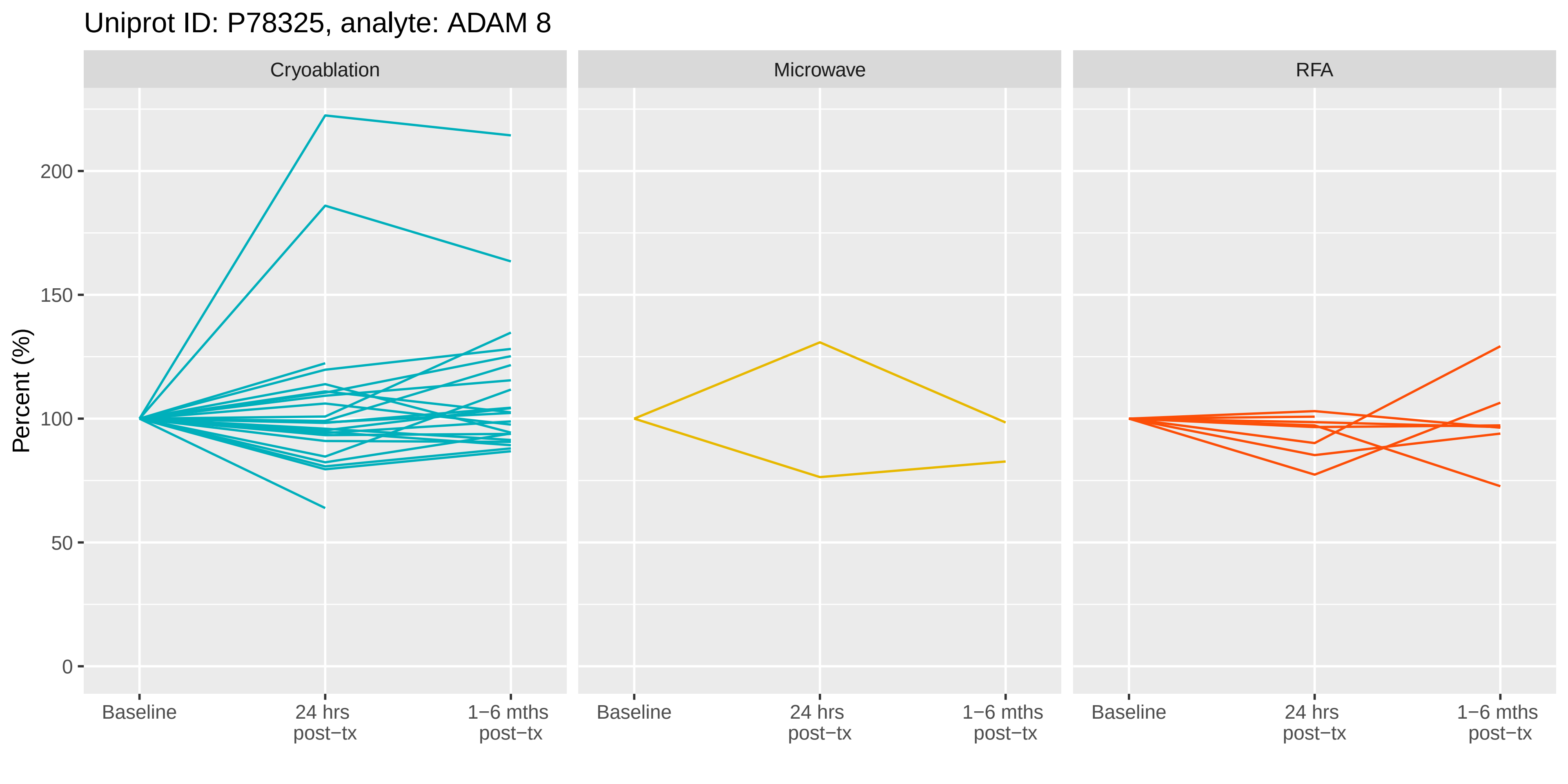

Uniprot ID: P78325, analyte: ADAM 8
Cryoablation
Microwave
RFA
Percent (%)
200
150
100
50
0
Baseline
24 hrs post−tx
1−6 mths post−tx

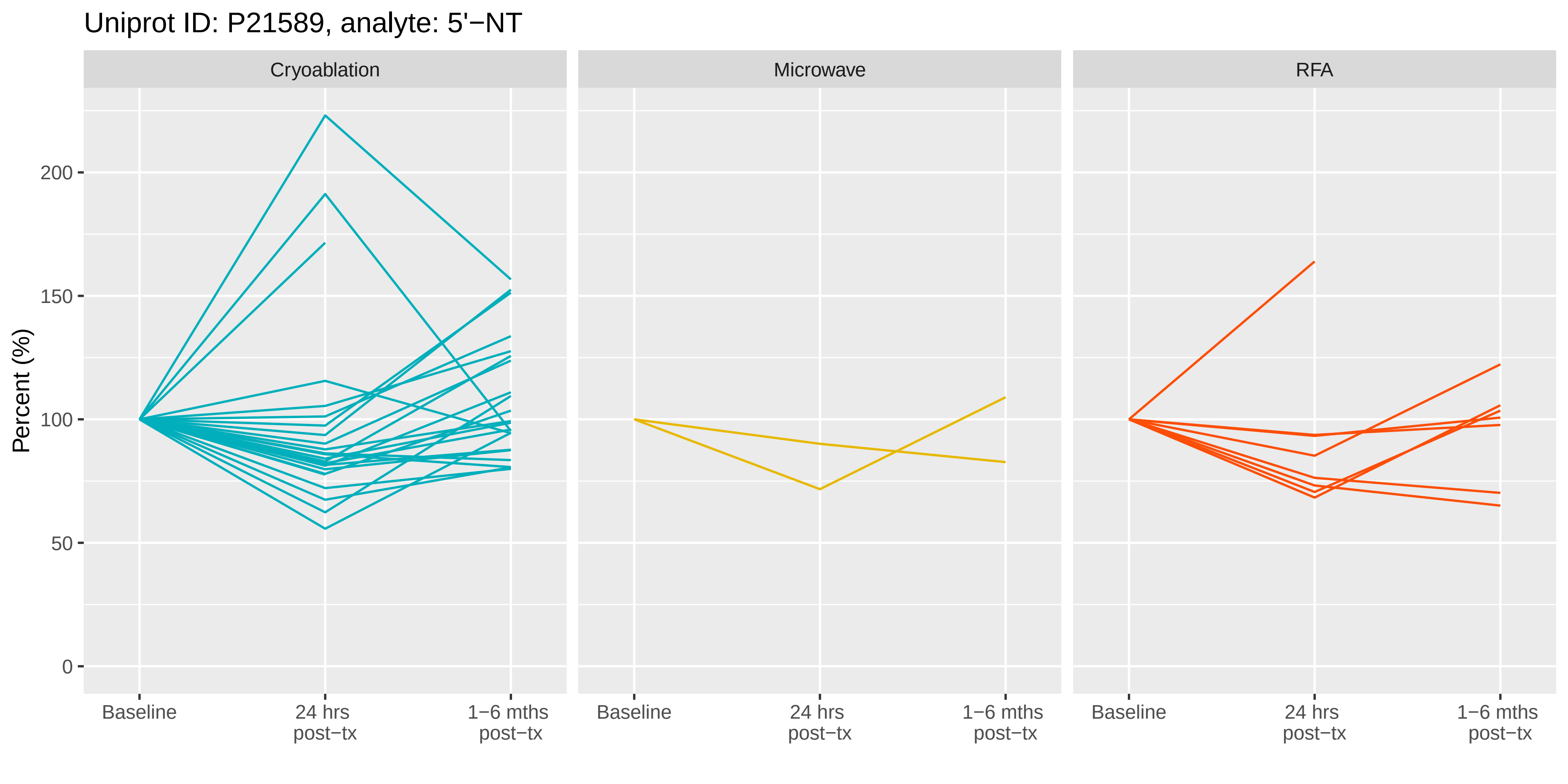

Uniprot ID: P21589, analyte: 5'−NT
Cryoablation
Microwave
RFA
Percent (%)
200
150
100
50
0
Baseline
24 hrs post−tx
1−6 mths post−tx

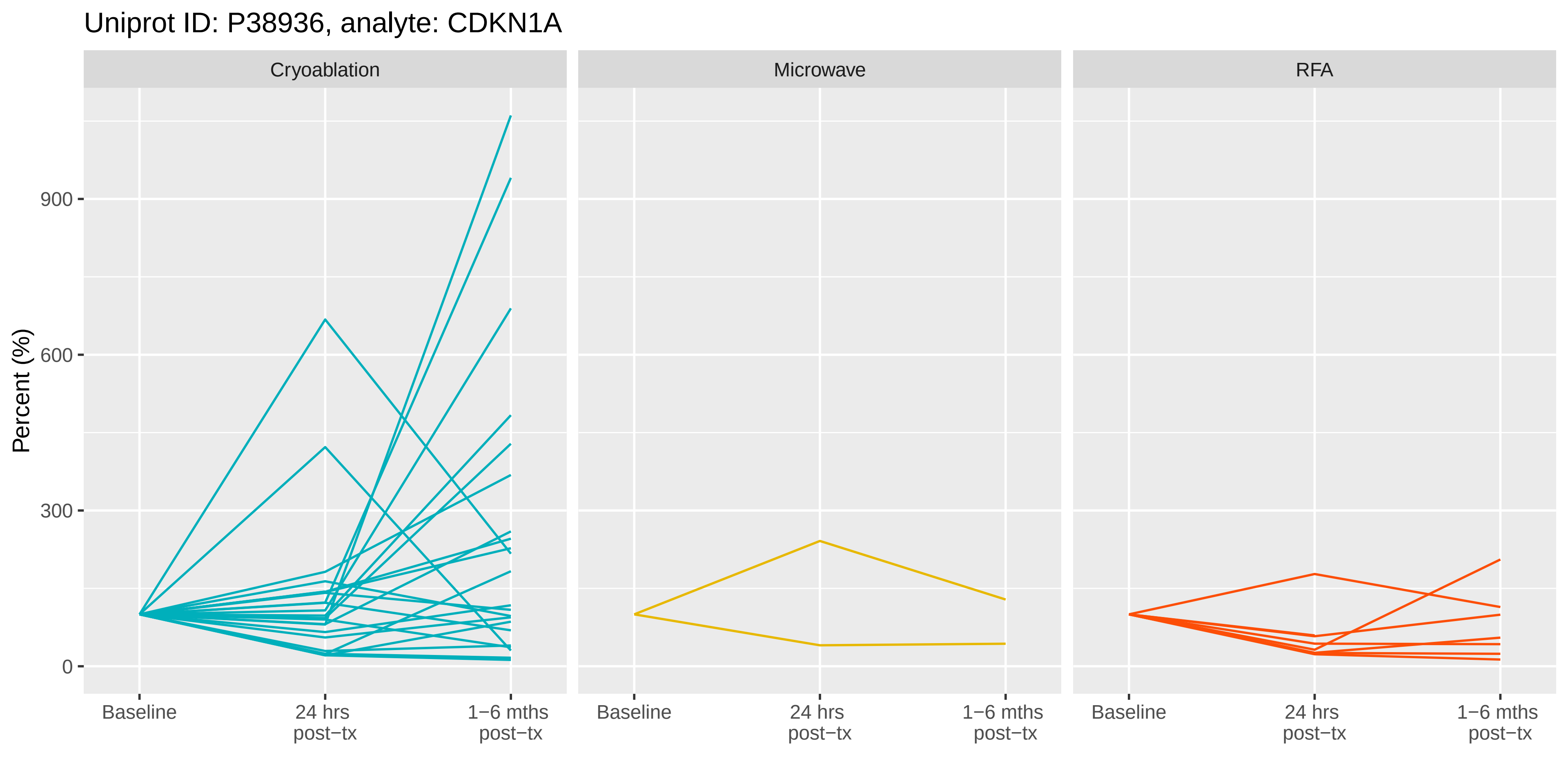

Uniprot ID: P38936, analyte: CDKN1A
Cryoablation
Microwave
RFA
Percent (%)
900
600
300
0
Baseline
24 hrs post−tx
1−6 mths post−tx

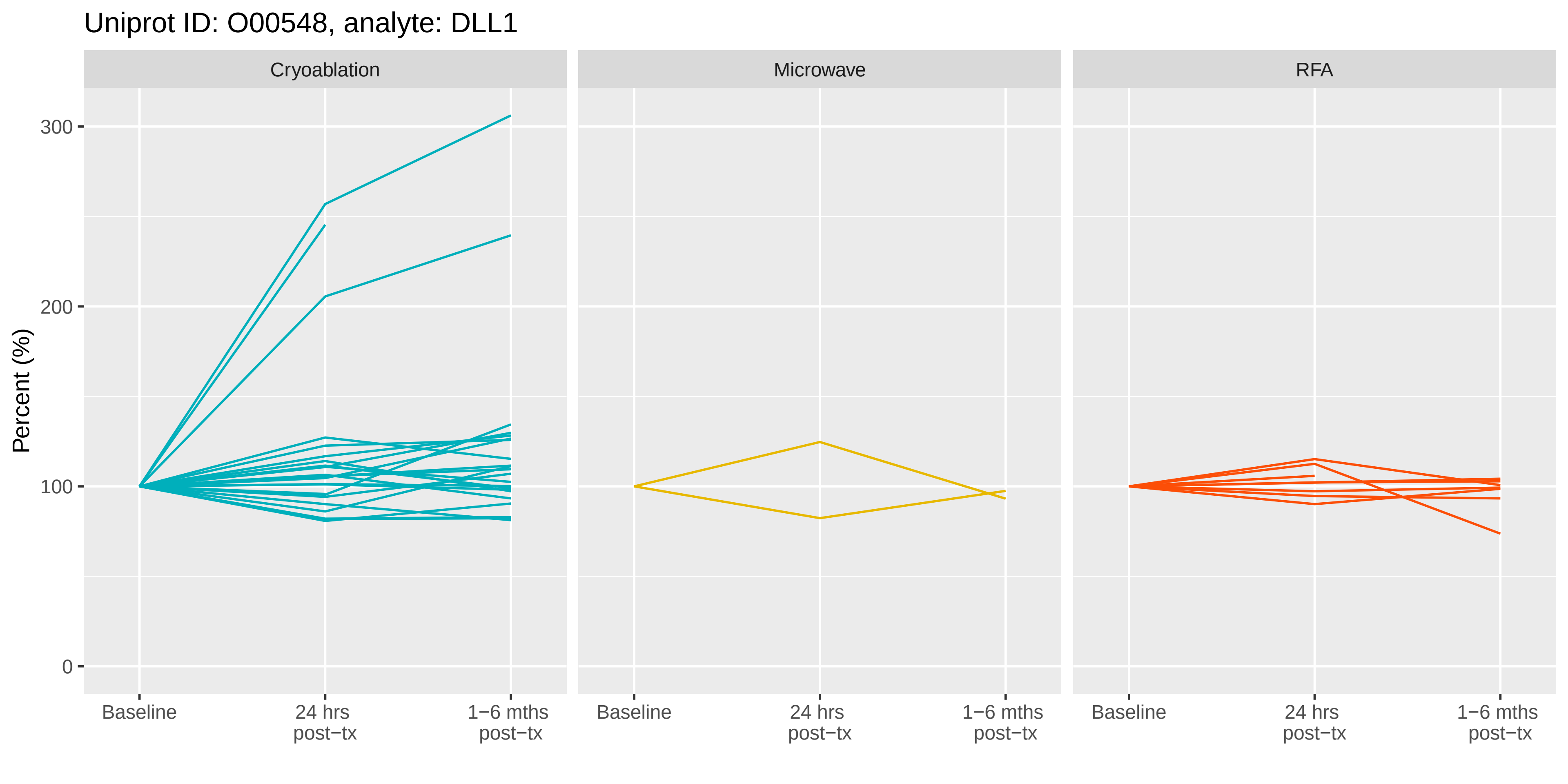

Uniprot ID: O00548, analyte: DLL1
Cryoablation
Microwave
RFA
Percent (%)
300
200
100
0
Baseline
24 hrs post−tx
1−6 mths post−tx

Uniprot ID: P21741, analyte: MK

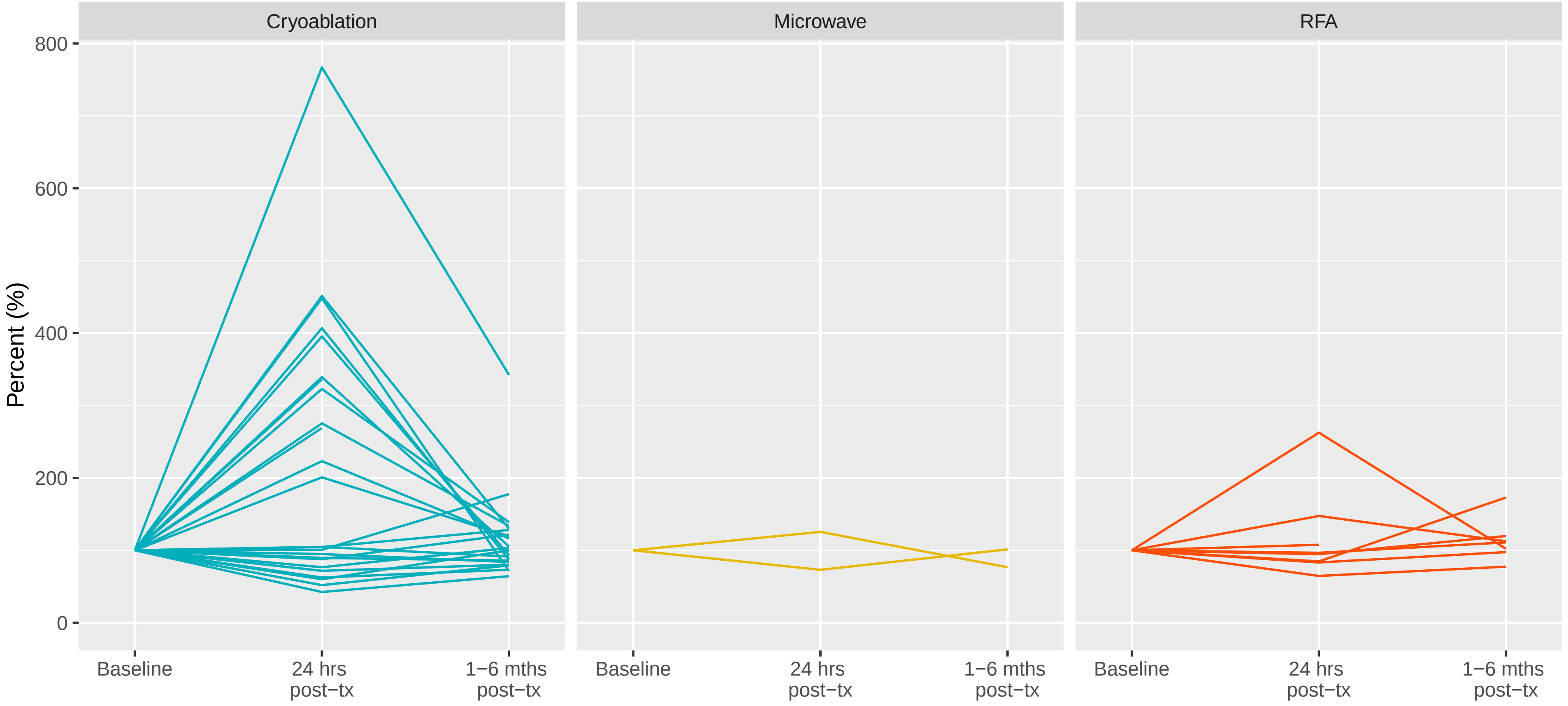

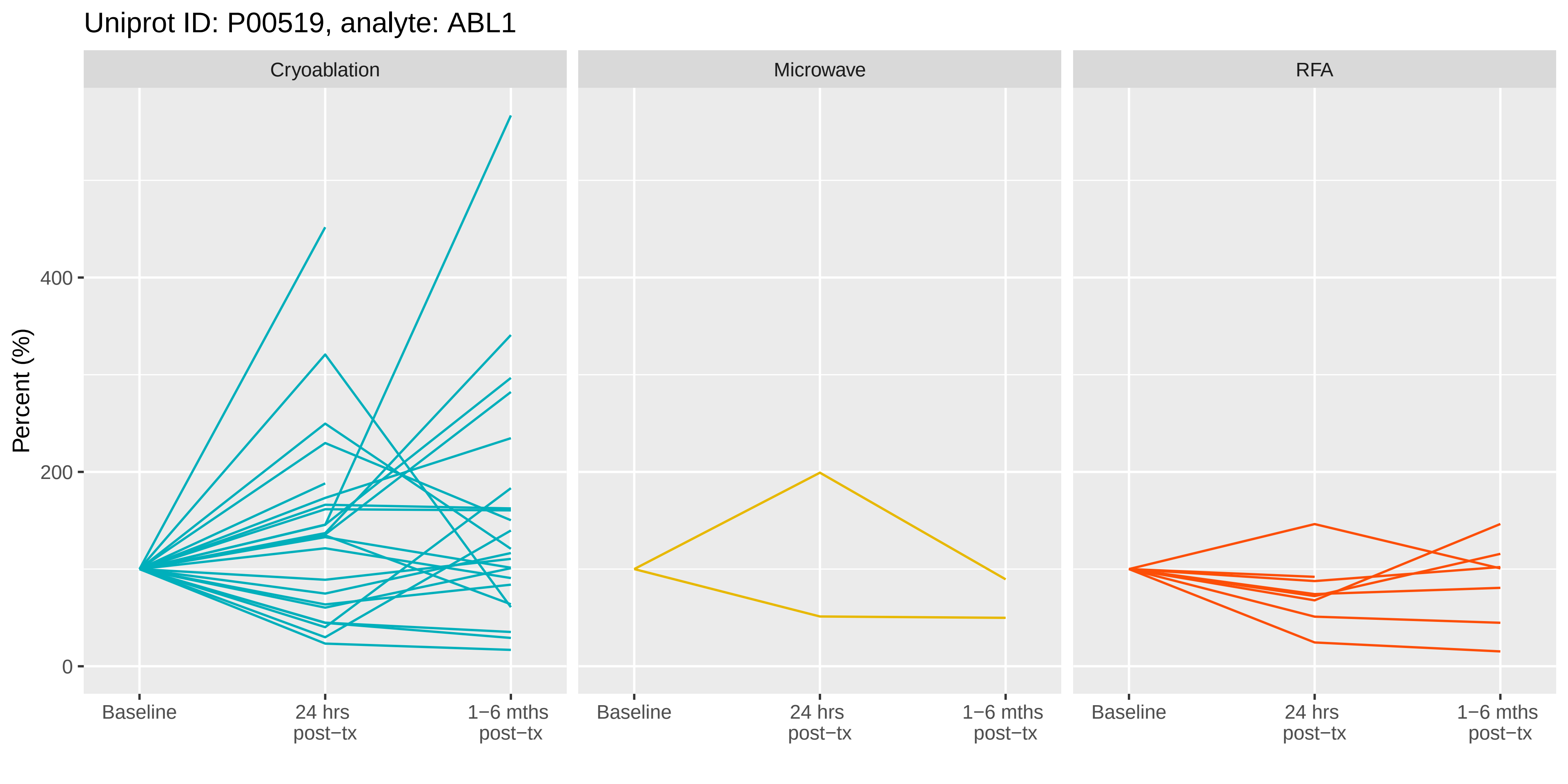

Uniprot ID: P00519, analyte: ABL1
Cryoablation
Microwave
RFA
Percent (%)
400
200
0
Baseline
24 hrs post−tx
1−6 mths post−tx

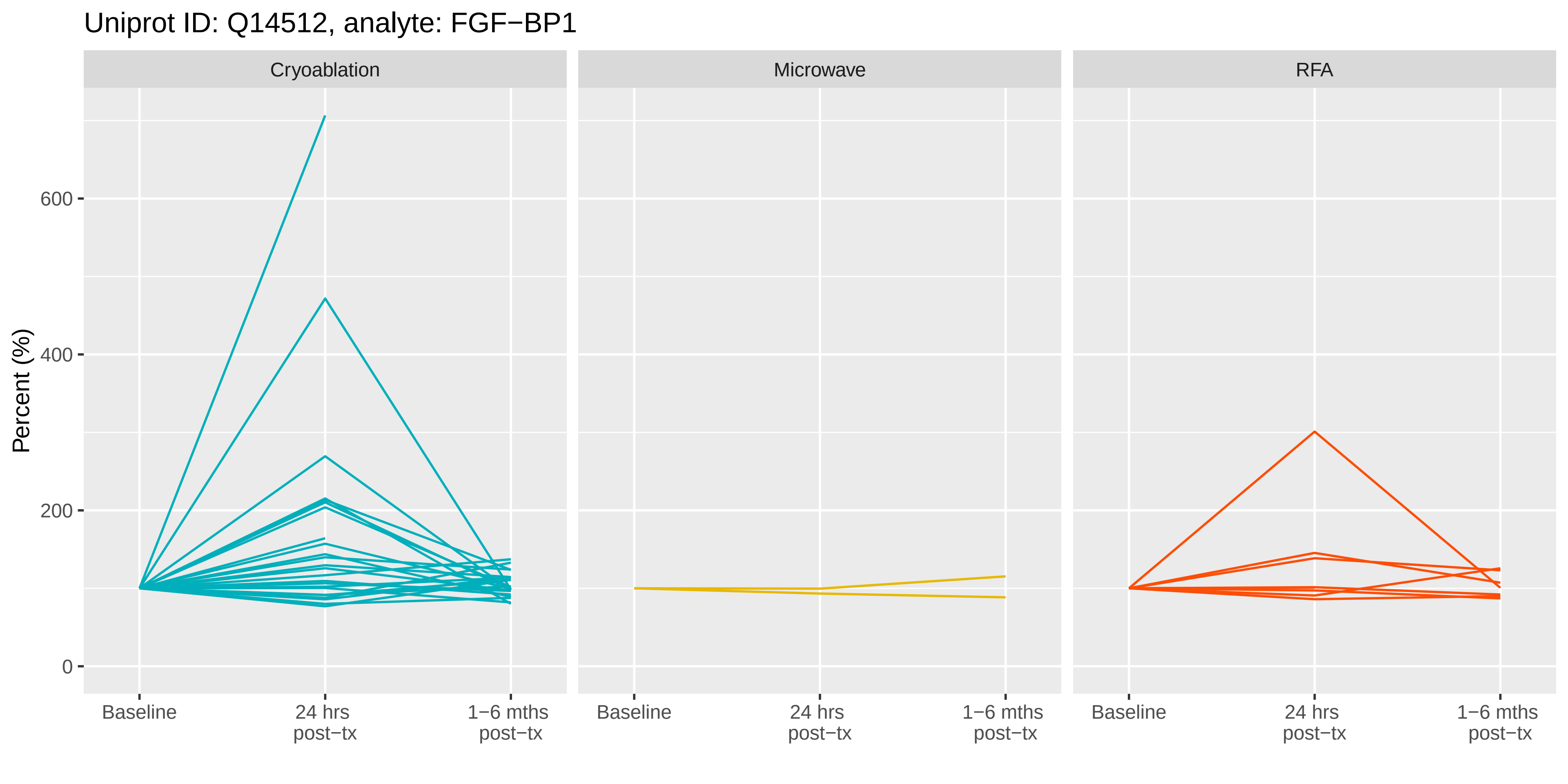

Uniprot ID: Q14512, analyte: FGF−BP1
Cryoablation
Microwave
RFA
Percent (%)
0
200
400
600
Baseline
24 hrs post−tx
1−6 mths post−tx

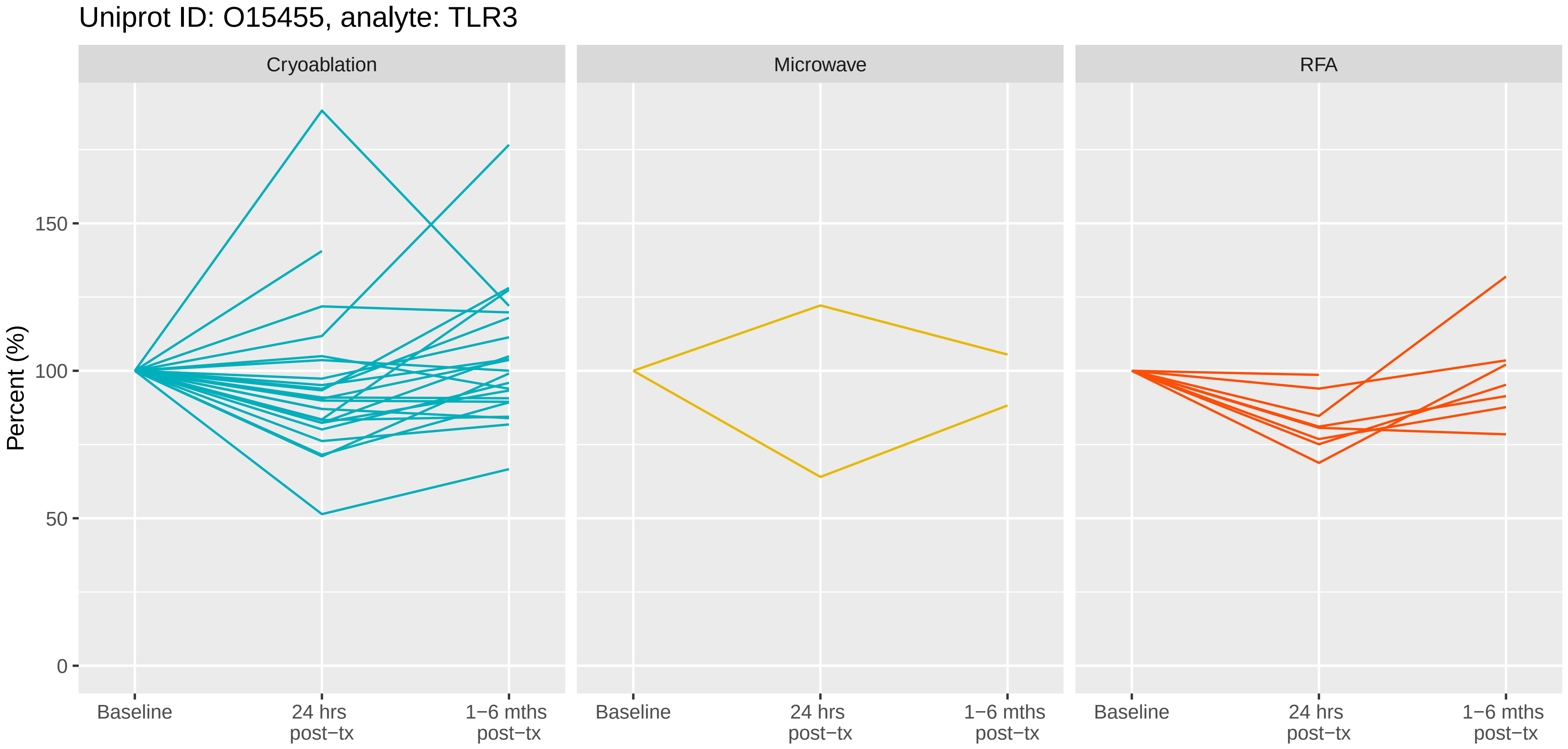
Uniprot ID: O15455, analyte: TLR3
Cryoablation
Microwave
RFA
Percent (%)
150
100
50
0
Baseline
24 hrs post−tx
1−6 mths post−tx

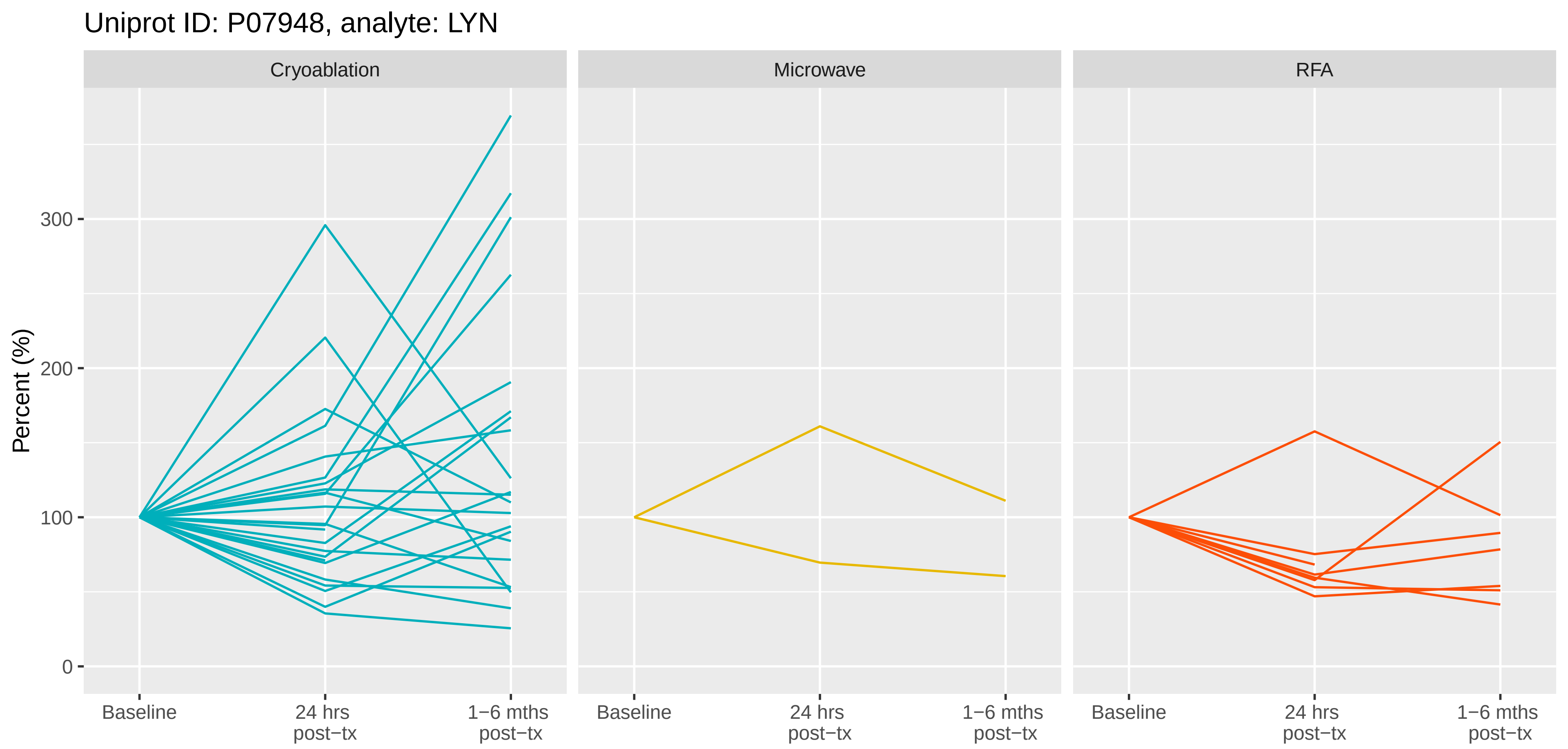

Uniprot ID: P07948, analyte: LYN
Cryoablation
Microwave
RFA
Percent (%)
300
200
100
0
Baseline
24 hrs post−tx
1−6 mths post−tx

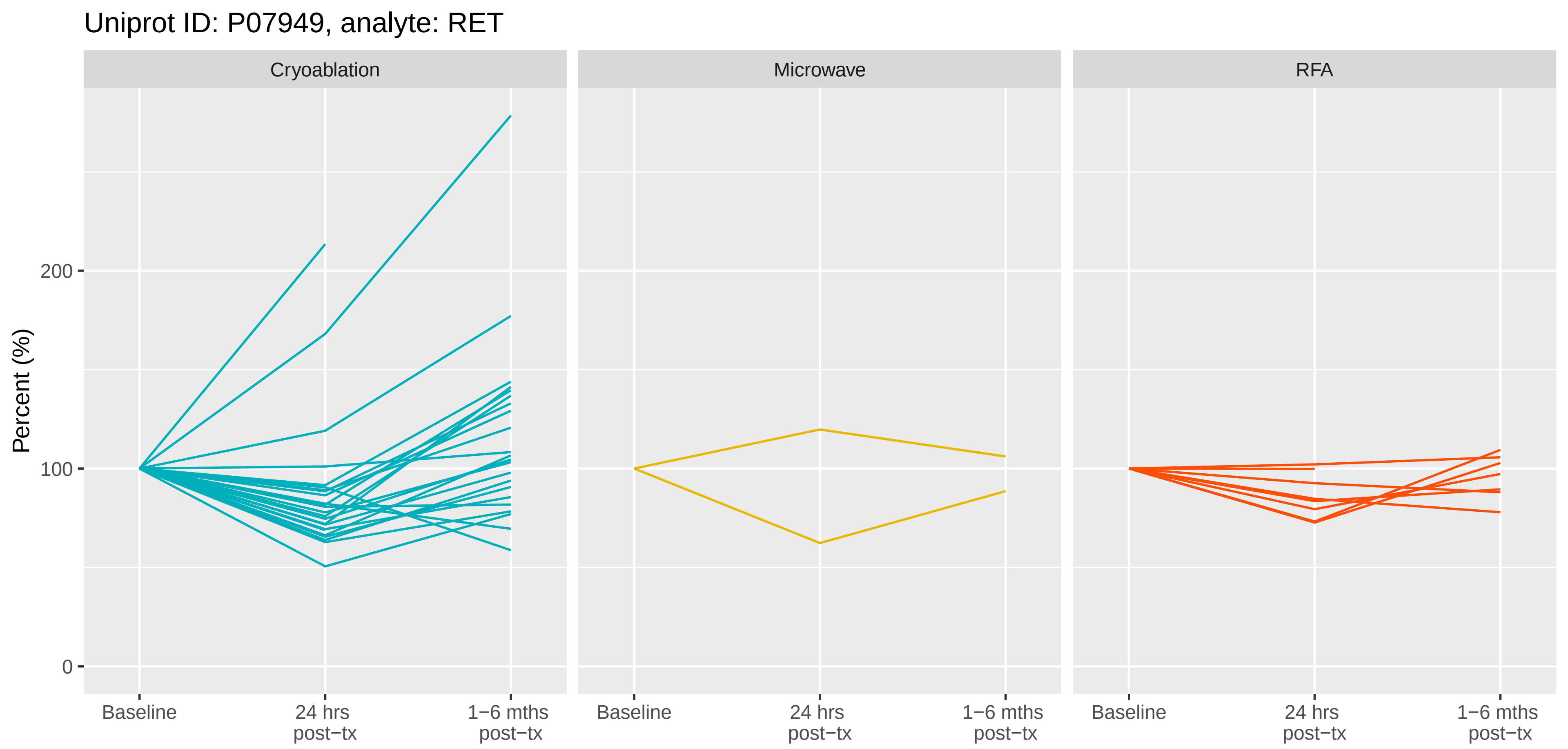

Uniprot ID: P07949, analyte: RET
Cryoablation
Microwave
RFA
Percent (%)
200
100
0
Baseline
24 hrs post−tx
1−6 mths post−tx

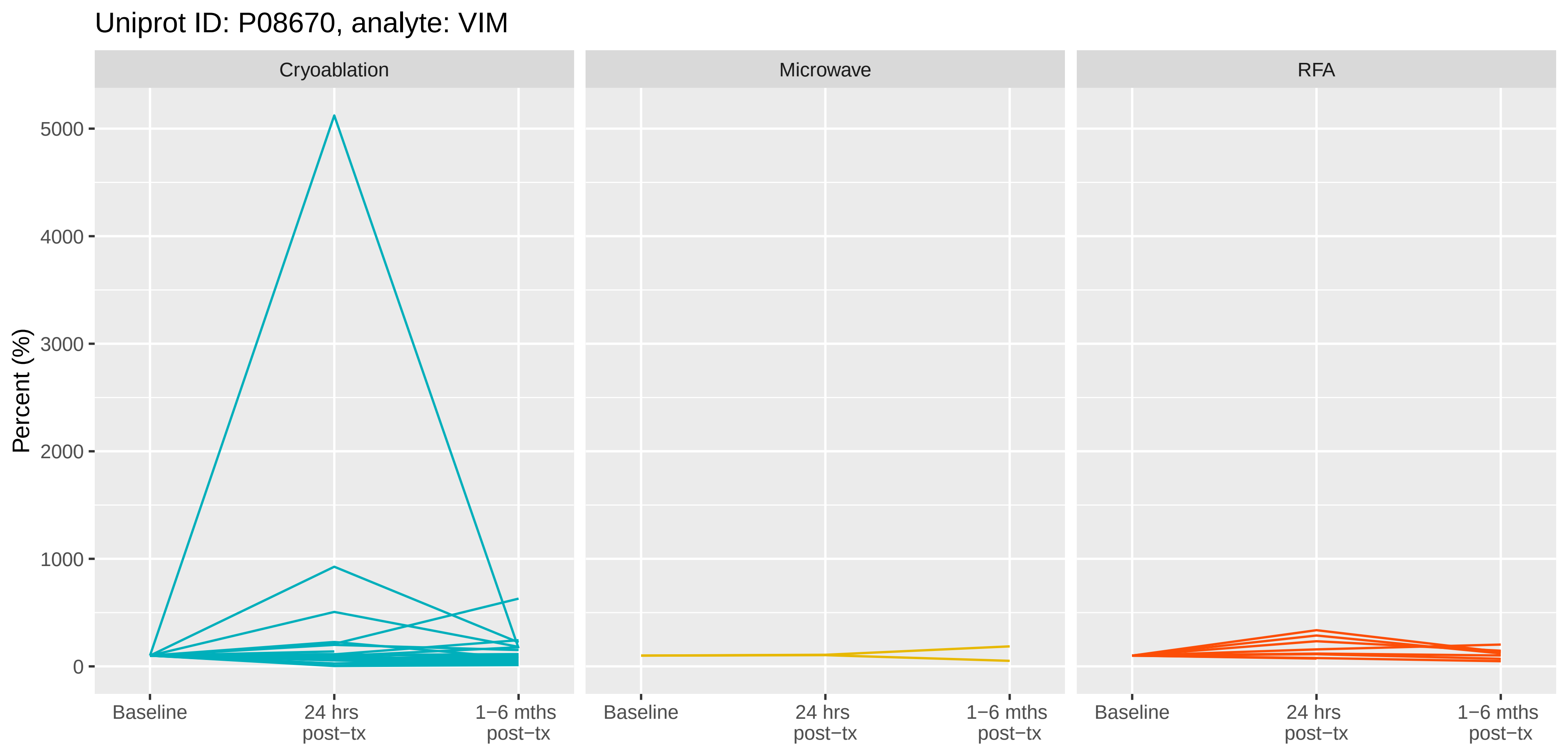

Uniprot ID: P08670, analyte: VIM
Cryoablation
Microwave
RFA
Percent (%)
5000
4000
3000
2000
1000
0
Baseline
24 hrs post−tx
1−6 mths post−tx

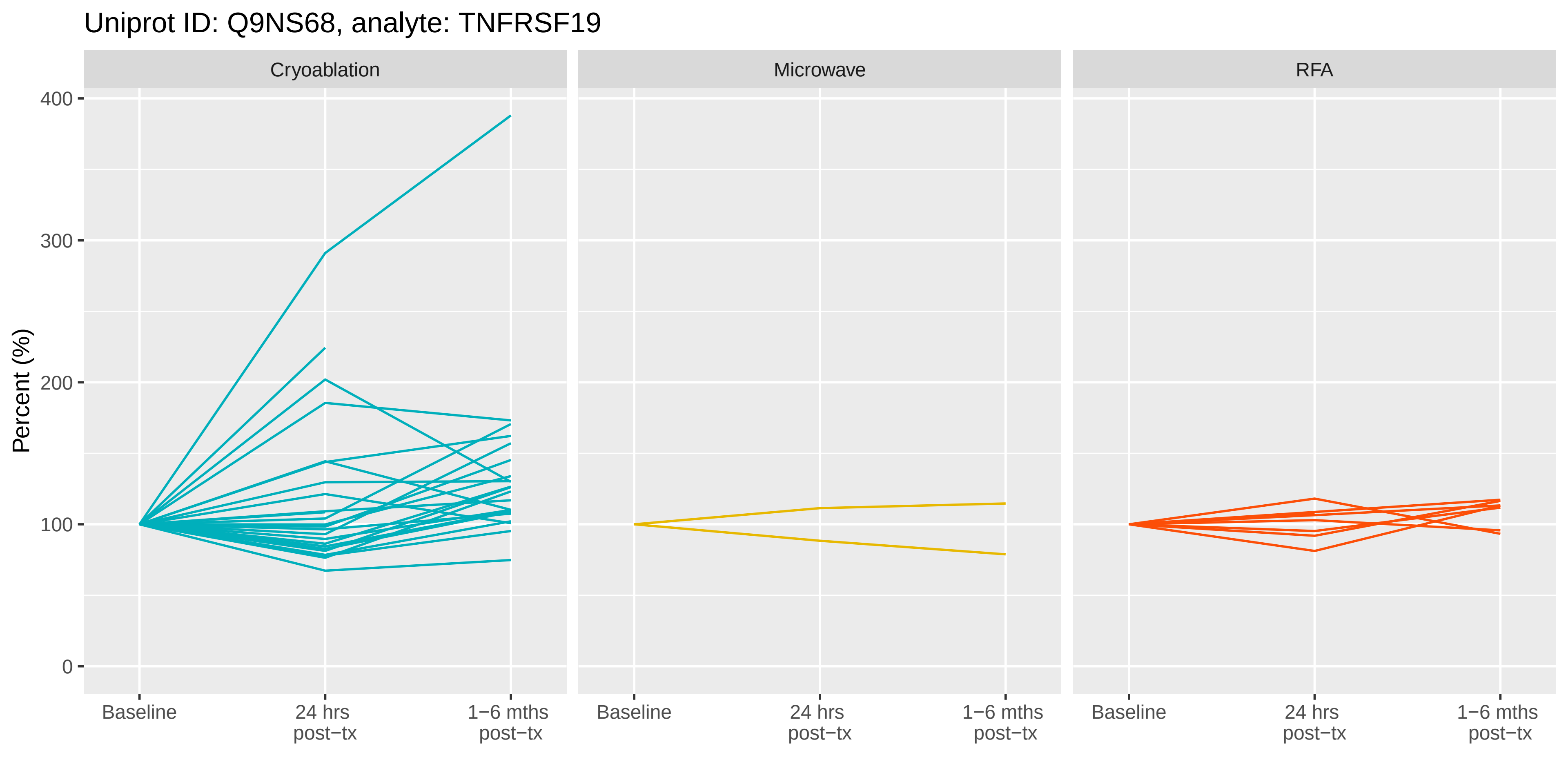

Uniprot ID: Q9NS68, analyte: TNFRSF19
Cryoablation
Microwave
RFA
Percent (%)
0
100
200
300
400
Baseline
24 hrs post−tx
1−6 mths post−tx

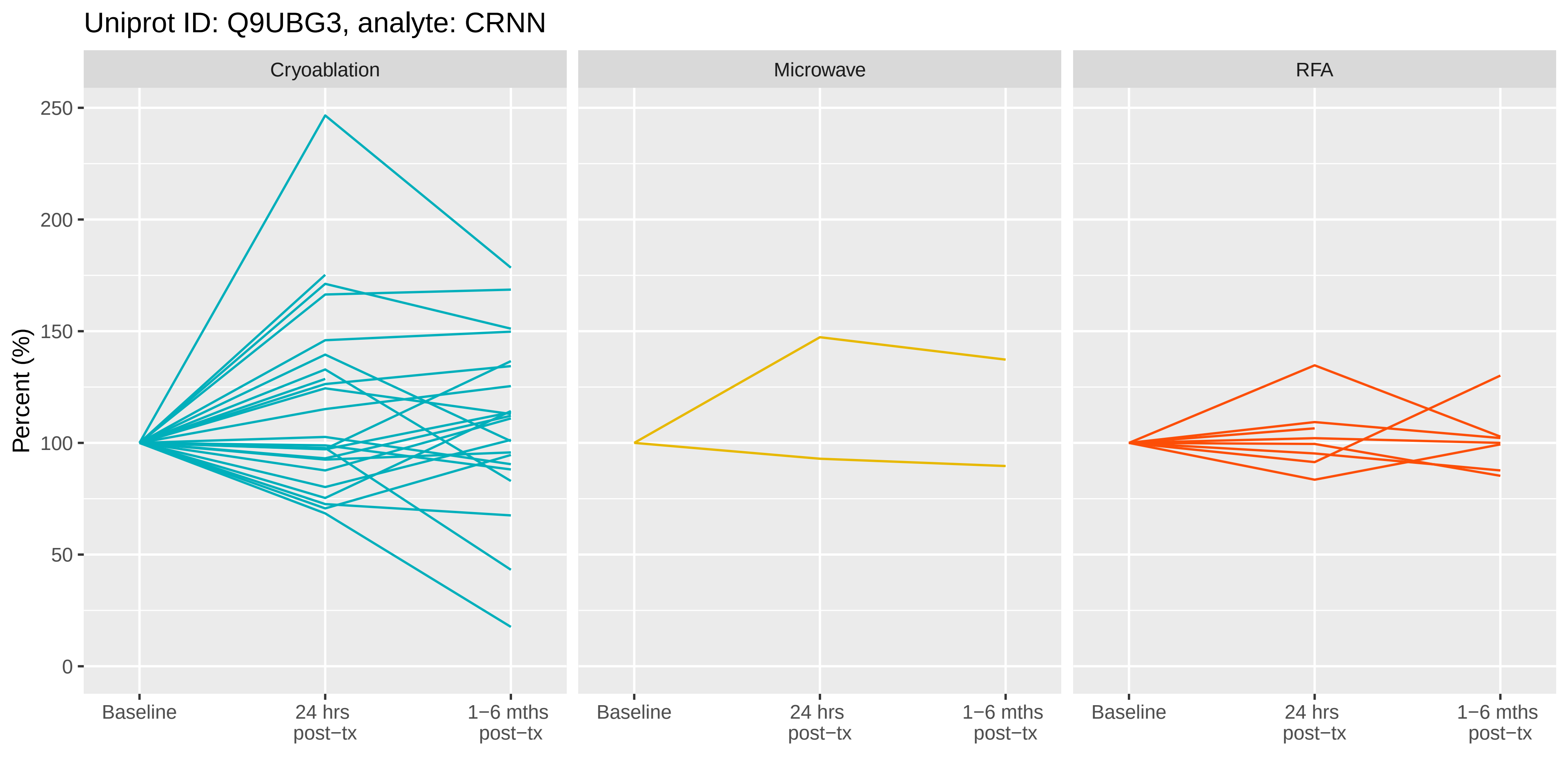

Uniprot ID: Q9UBG3, analyte: CRNN
Cryoablation
Microwave
RFA
Percent (%)
250
200
150
100
50
0
Baseline
24 hrs post−tx
1−6 mths post−tx

Uniprot ID: P56279, analyte: TCL1A

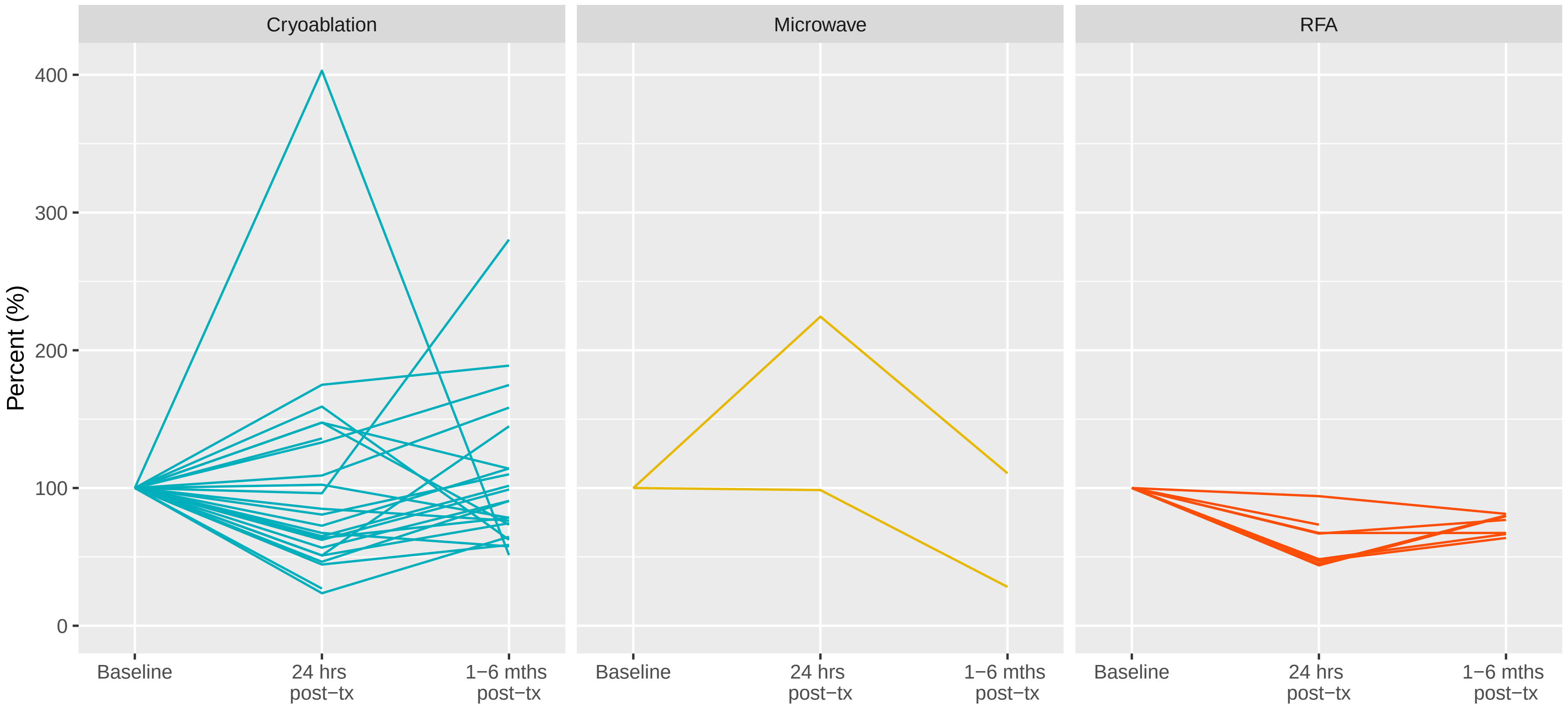

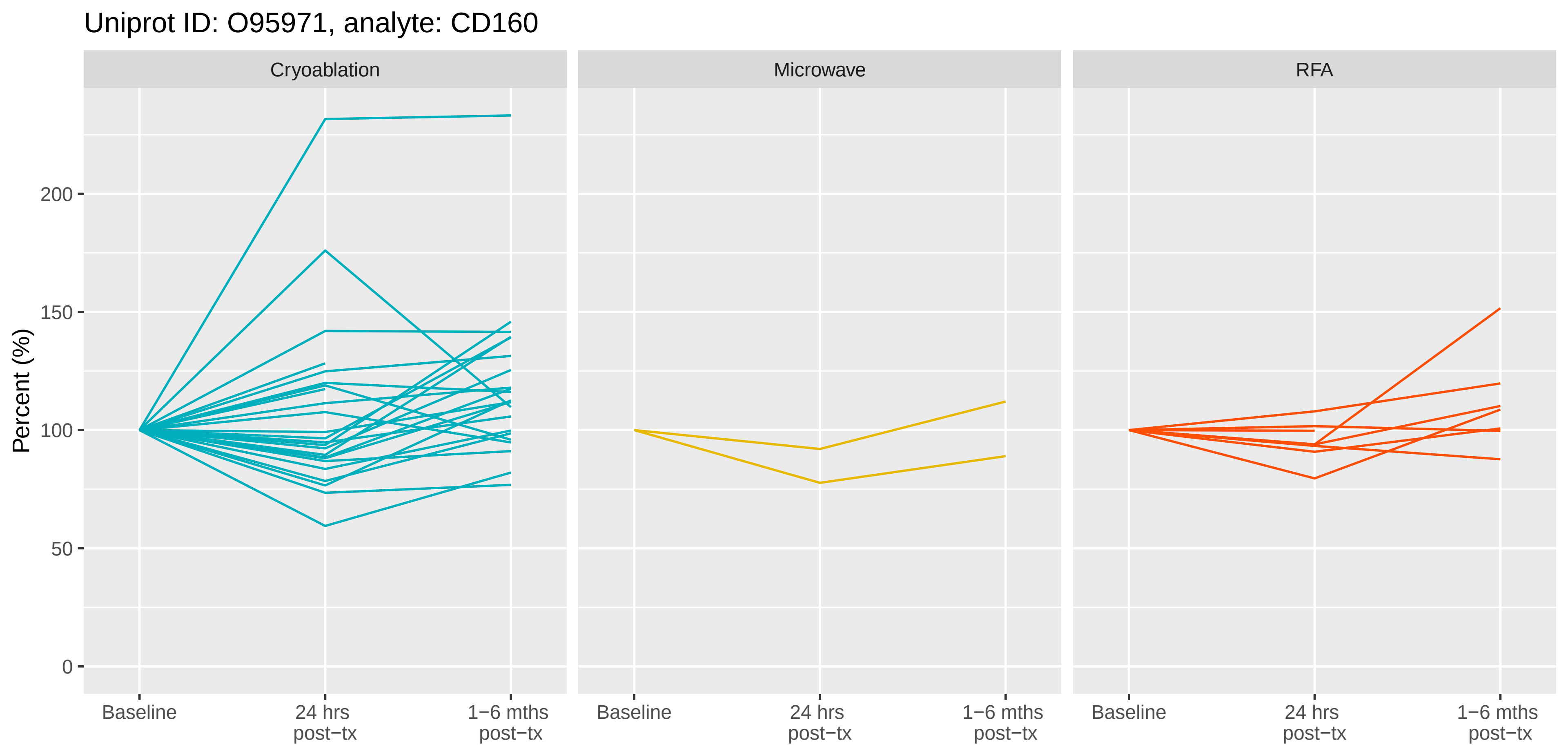
Uniprot ID: O95971, analyte: CD160
Cryoablation
Microwave
RFA
Percent (%)
200
150
100
50
0
Baseline
24 hrs post−tx
1−6 mths post−tx
Baseline
24 hrs post−tx
1−6 mths post−tx
Baseline
24 hrs post−tx
1−6 mths post−tx

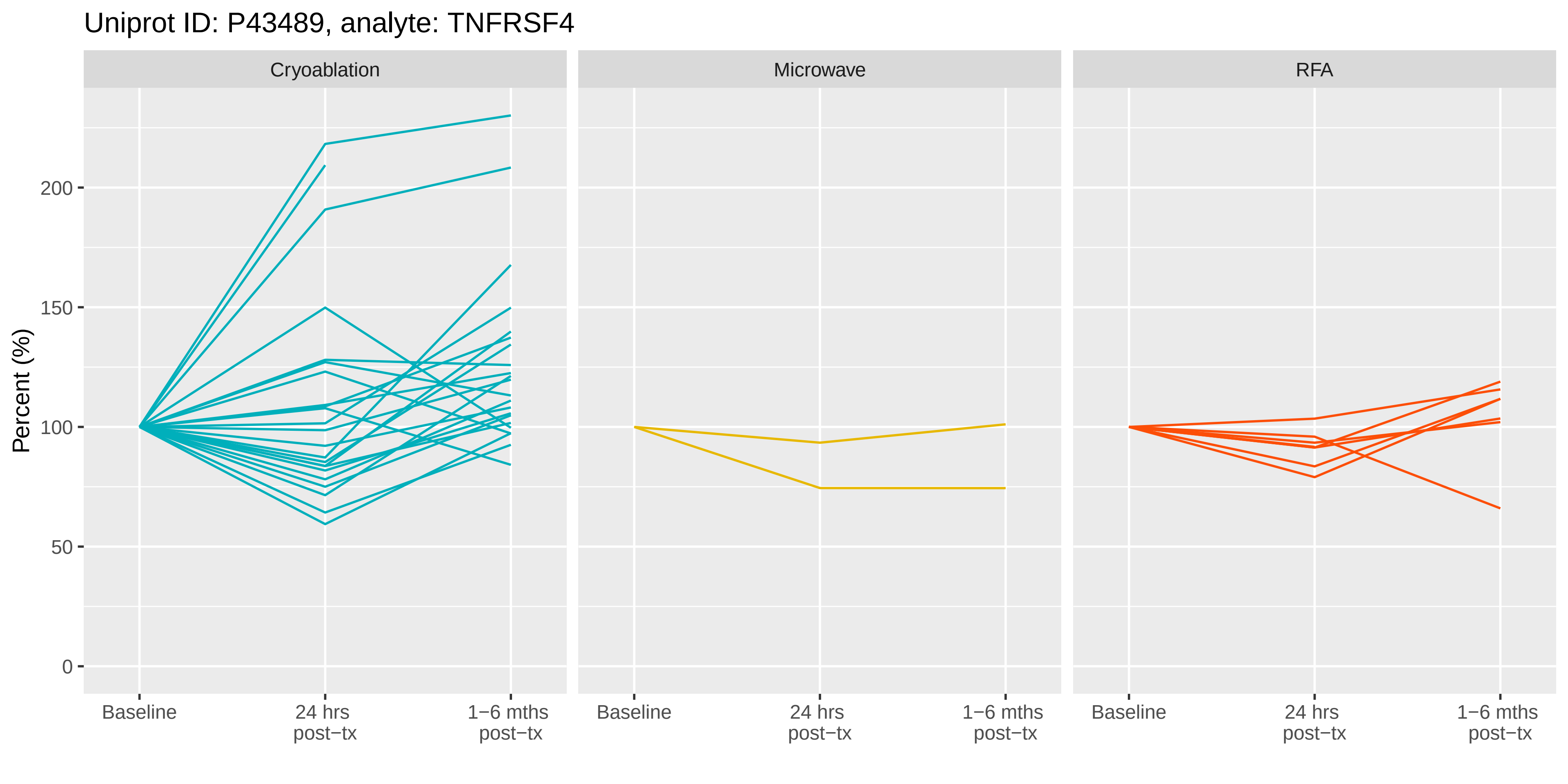

Uniprot ID: P43489, analyte: TNFRSF4
Cryoablation
Microwave
RFA
Percent (%)
200
150
100
50
0
Baseline
24 hrs post−tx
1−6 mths post−tx

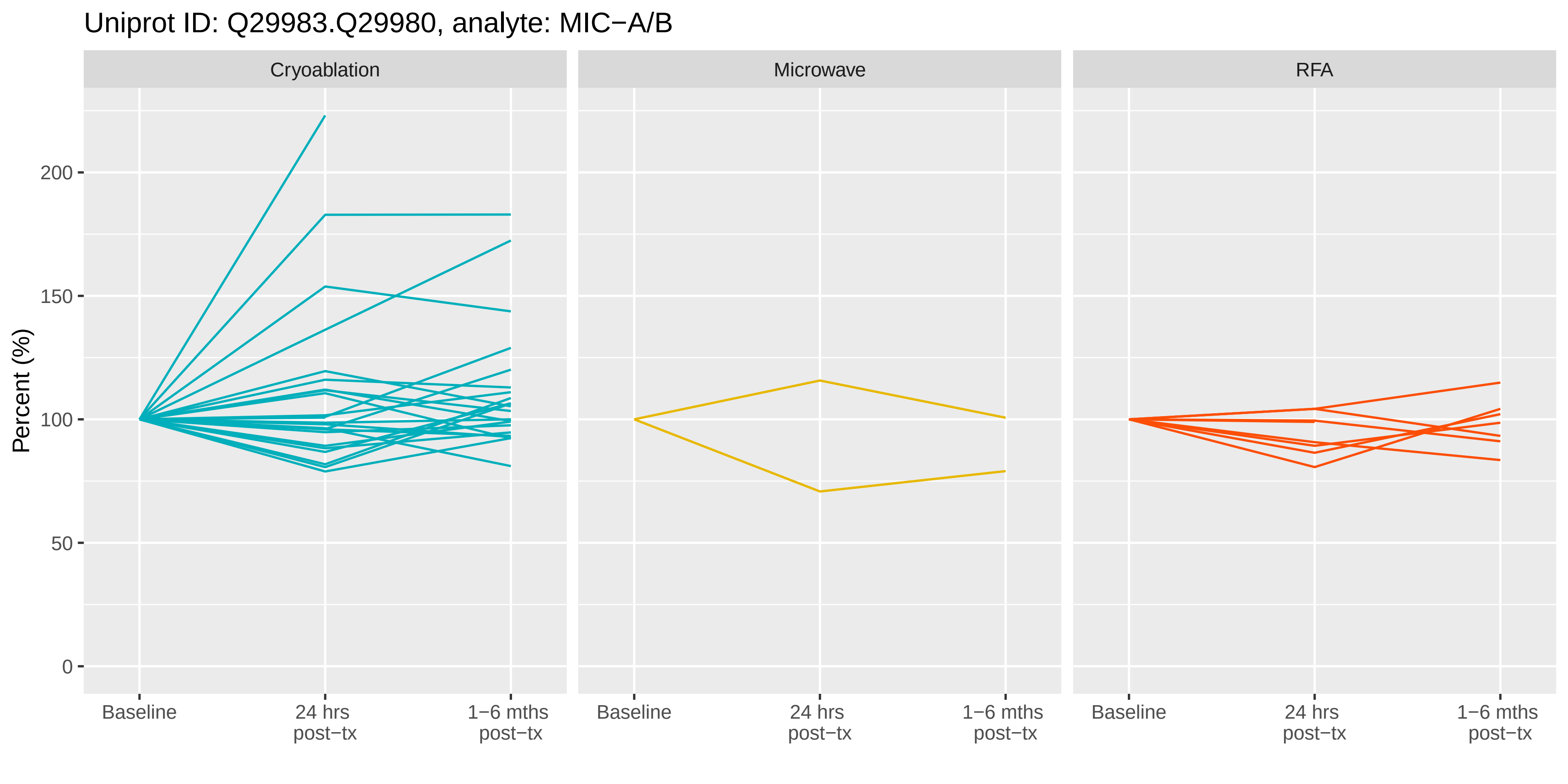

Uniprot ID: Q29983.Q29980, analyte: MIC−A/B
Cryoablation
Microwave
RFA
Percent (%)
200
150
100
50
0
Baseline
24 hrs post−tx
1−6 mths post−tx

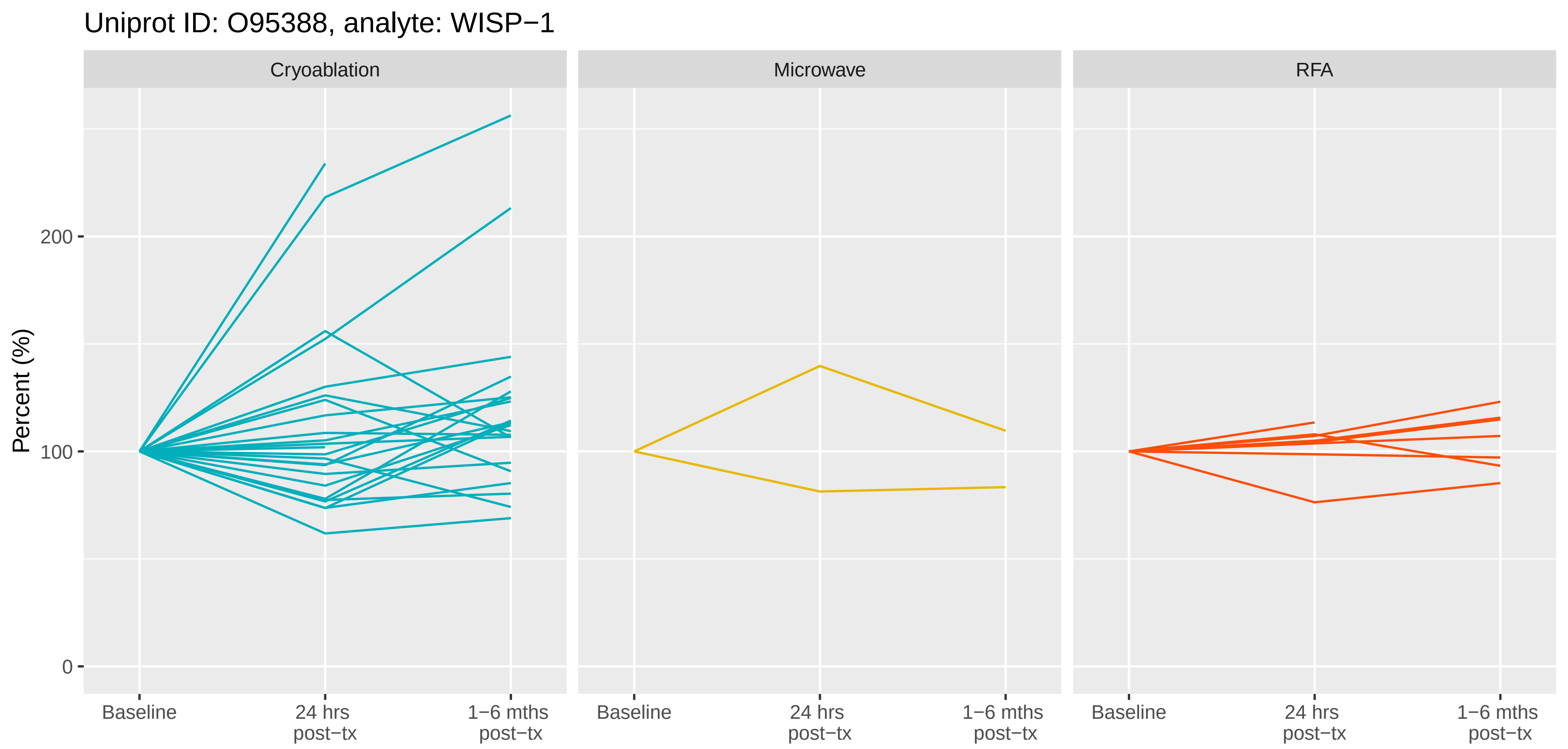
Uniprot ID: O95388, analyte: WISP−1
Cryoablation
Microwave
RFA
Percent (%)
0
100
200
Baseline
24 hrs post−tx
1−6 mths post−tx

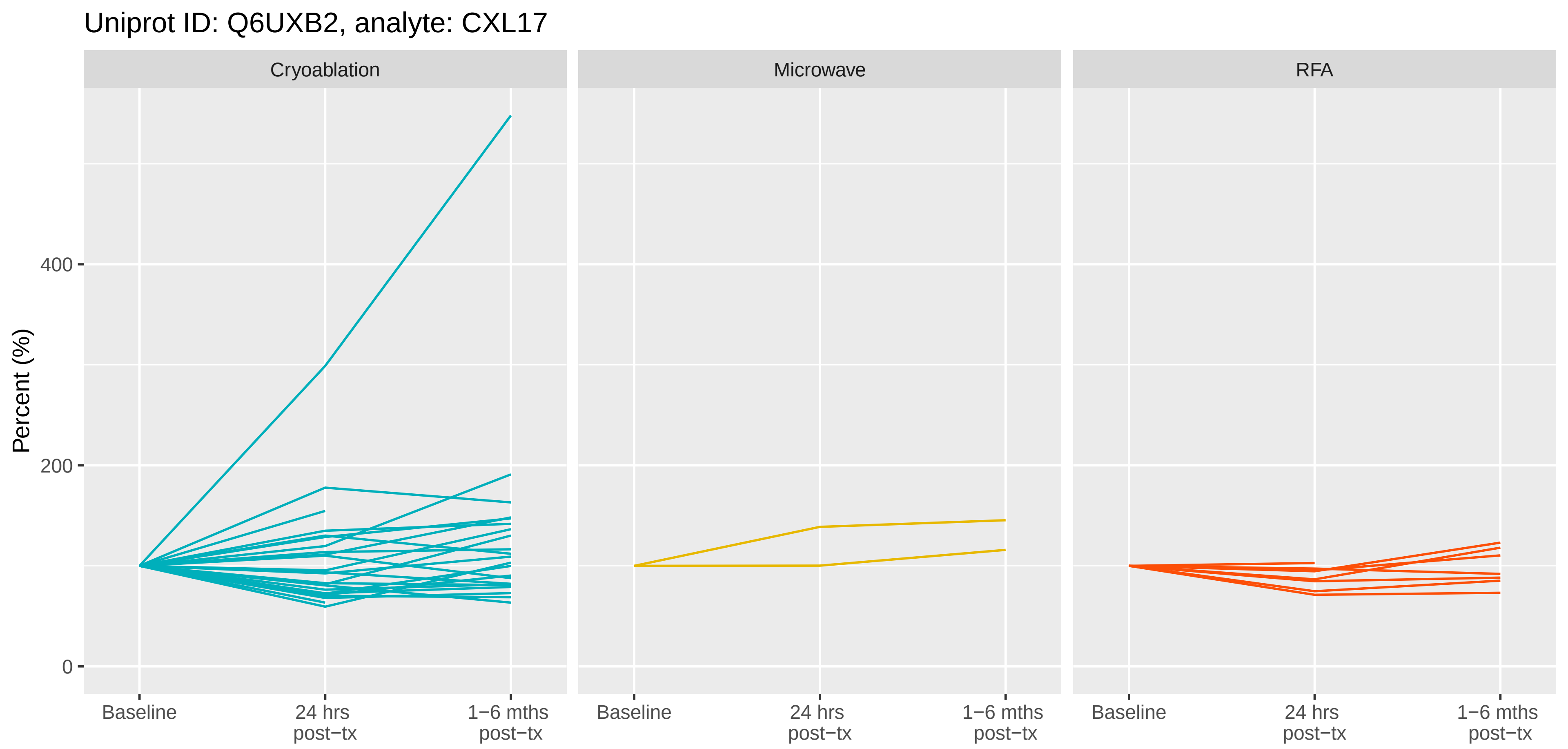

Uniprot ID: Q6UXB2, analyte: CXL17
Cryoablation
Microwave
RFA
Percent (%)
400
200
0
Baseline
24 hrs post−tx
1−6 mths post−tx

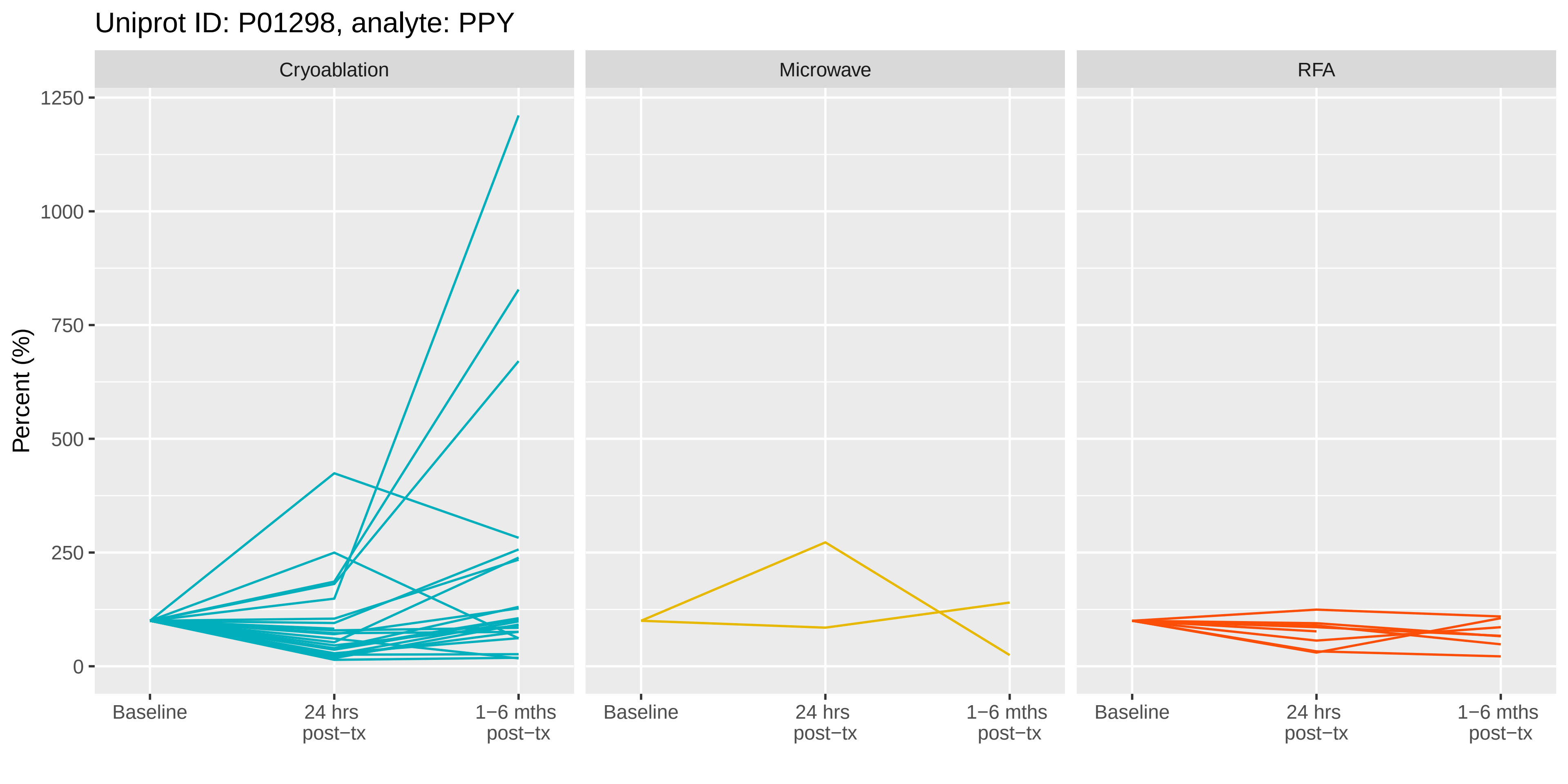
Uniprot ID: P01298, analyte: PPY
Cryoablation
Microwave
RFA
Percent (%)
1250
1000
750
500
250
0
Baseline
24 hrs post−tx
1−6 mths post−tx

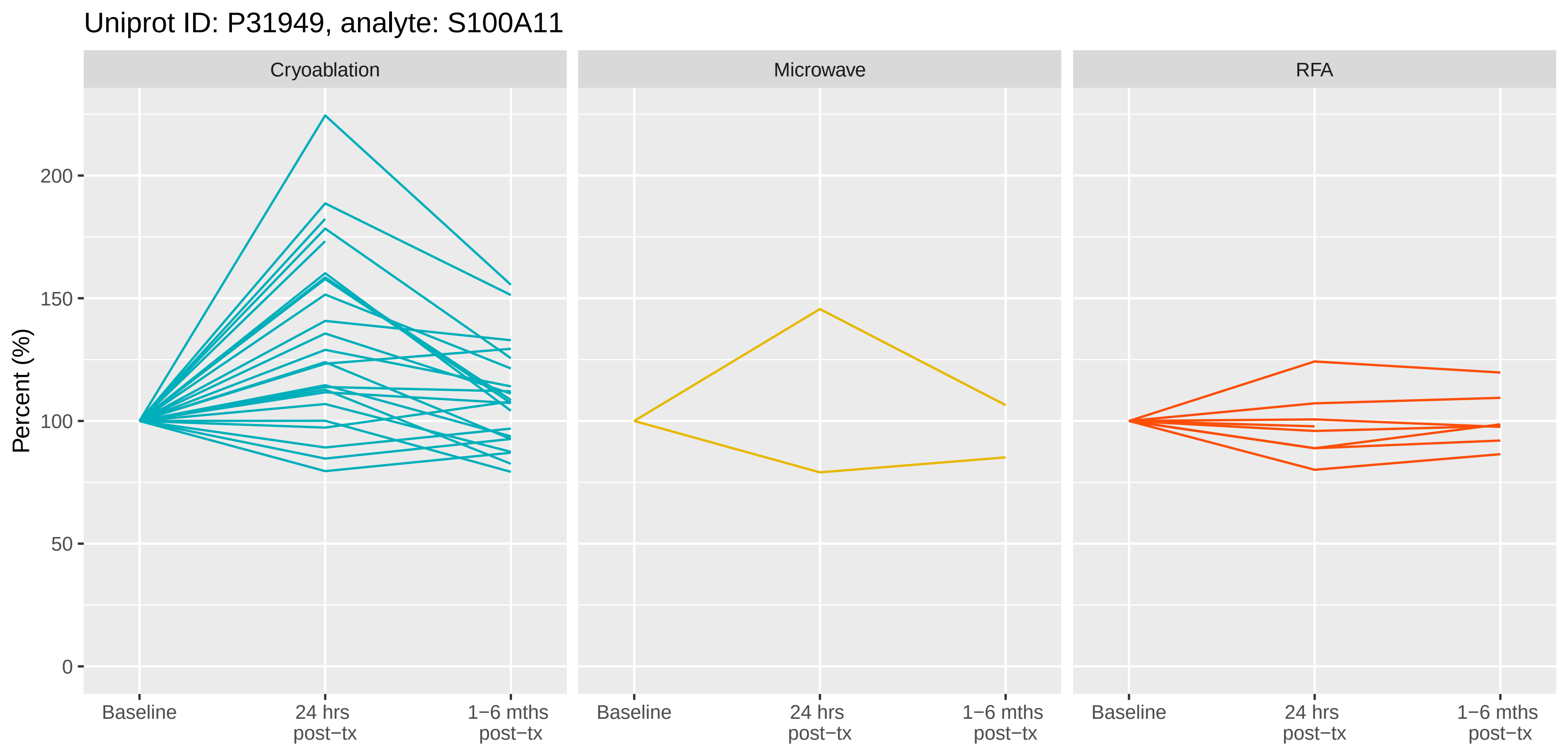

Uniprot ID: P31949, analyte: S100A11
Cryoablation
Microwave
RFA
Percent (%)
0
50
100
150
200
Baseline
24 hrs post−tx
1−6 mths post−tx

Uniprot ID: P15514, analyte: AREG

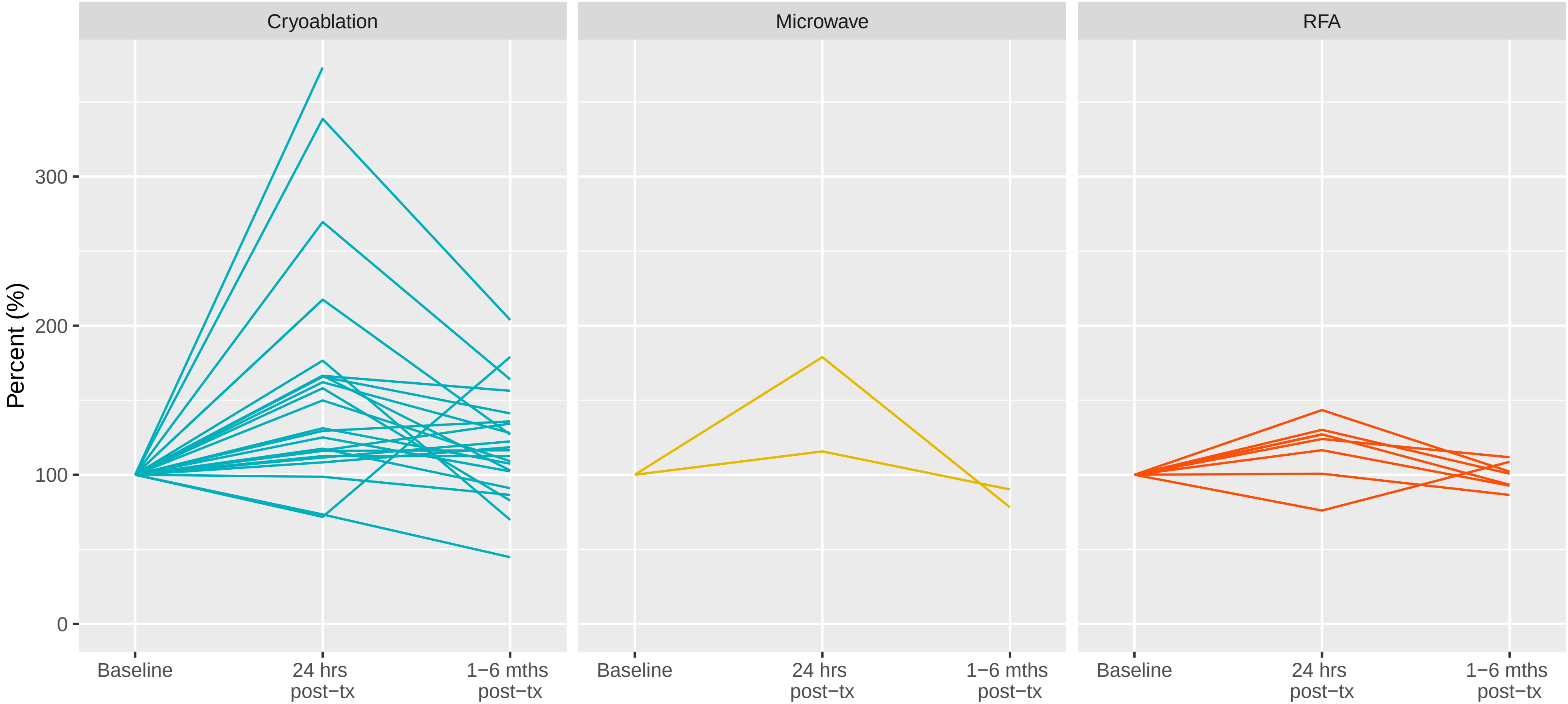

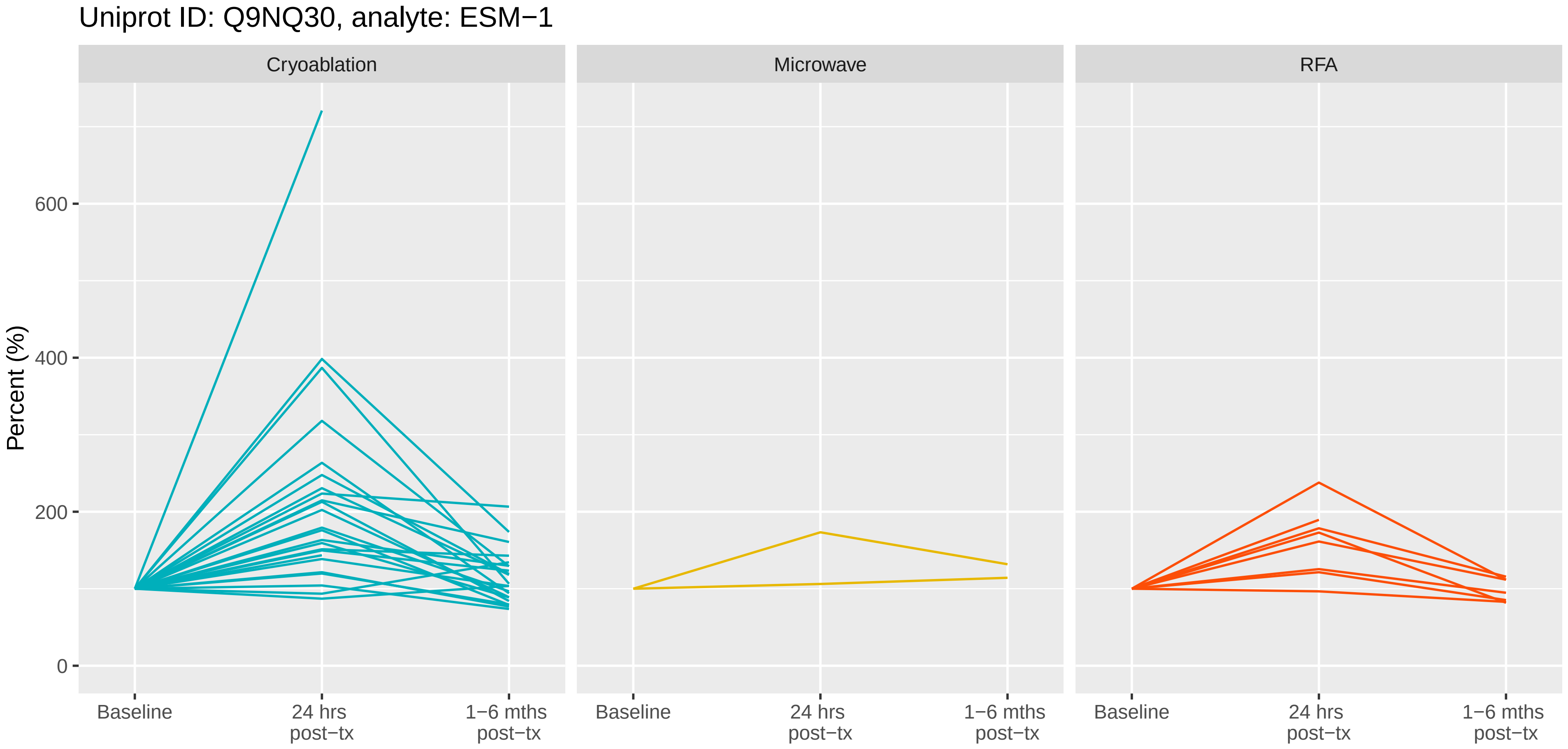

Uniprot ID: Q9NQ30, analyte: ESM−1
Cryoablation
Microwave
RFA
Percent (%)
0
200
400
600
Baseline
24 hrs post−tx
1−6 mths post−tx

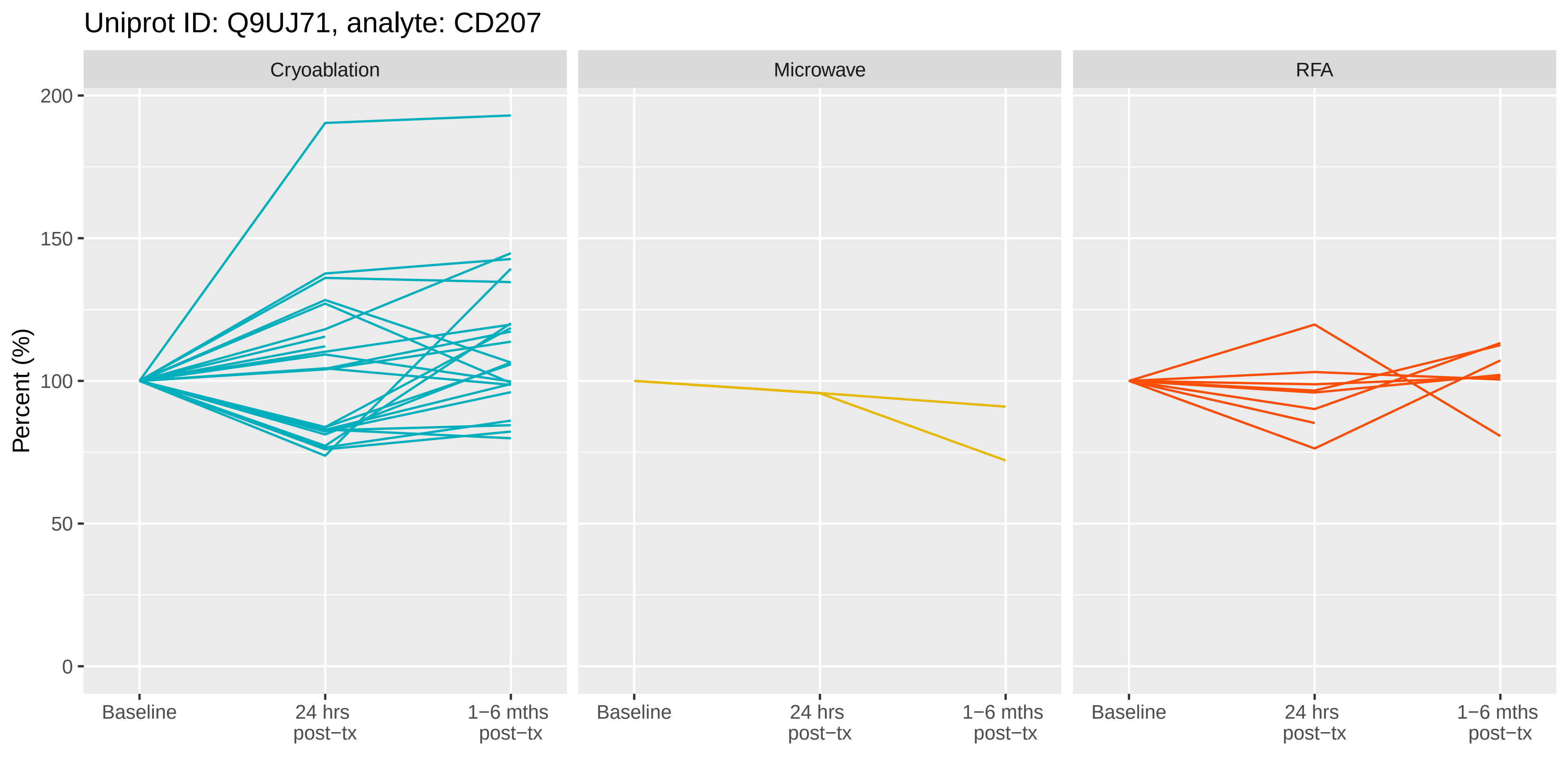

Uniprot ID: Q9UJ71, analyte: CD207
Cryoablation
Microwave
RFA
Percent (%)
200
150
100
50
0
Baseline
24 hrs post−tx
1−6 mths post−tx

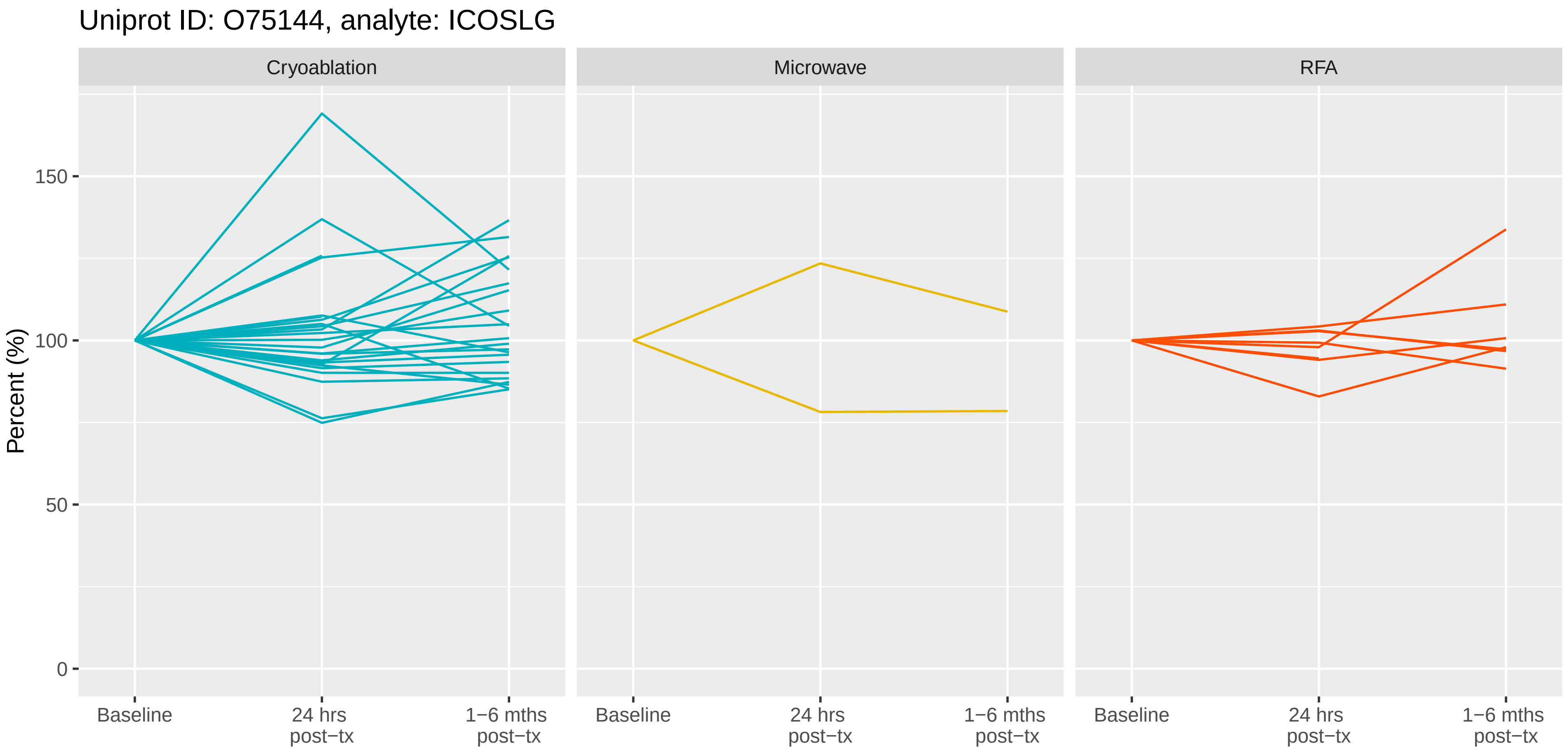

Uniprot ID: O75144, analyte: ICOSLG
Cryoablation
Microwave
RFA
Percent (%)
150
100
50
0
Baseline
24 hrs post−tx
1−6 mths post−tx

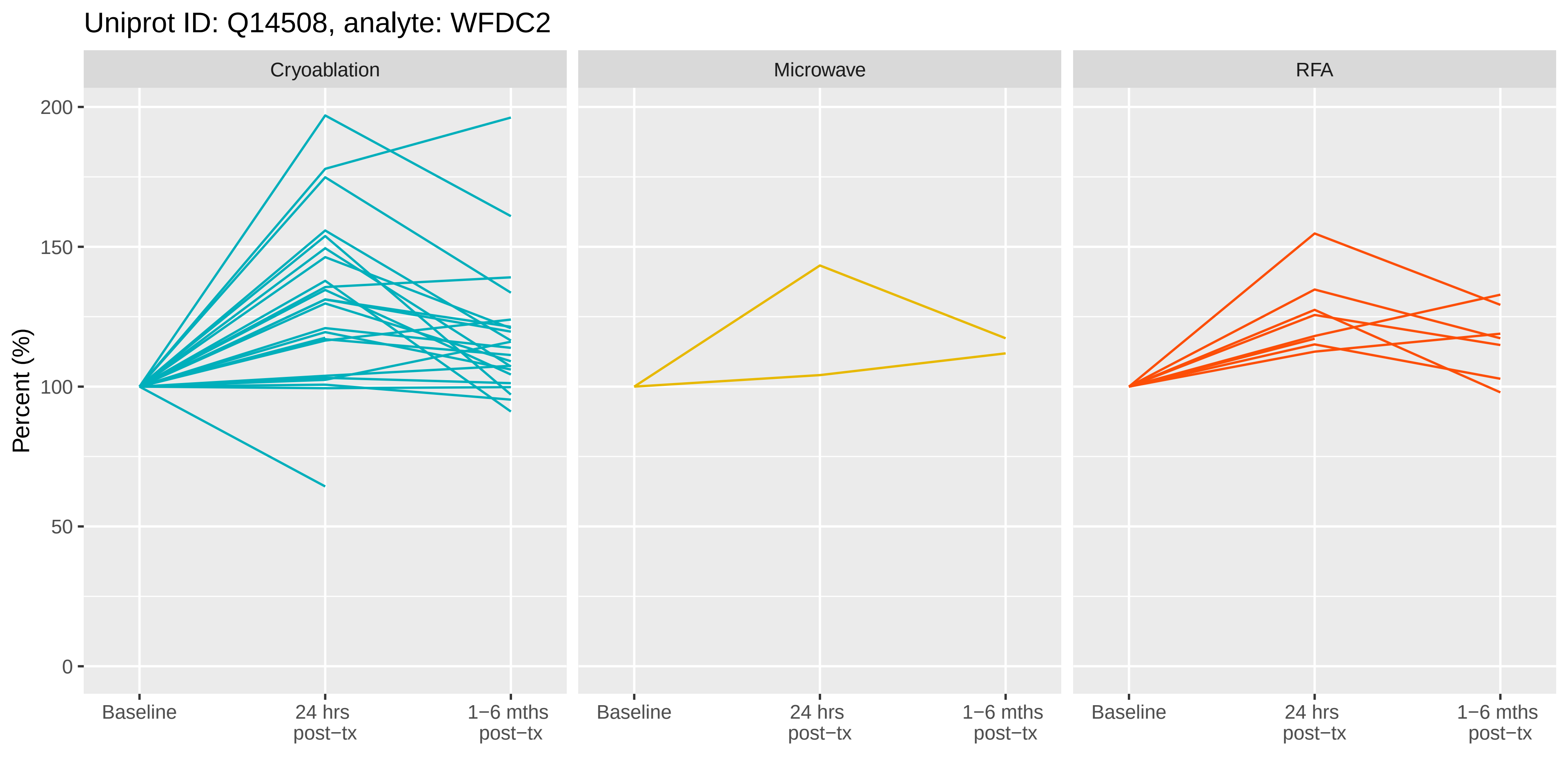

Uniprot ID: Q14508, analyte: WFDC2
Cryoablation
Microwave
RFA
Percent (%)
200
150
100
50
0
Baseline
24 hrs post−tx
1−6 mths post−tx

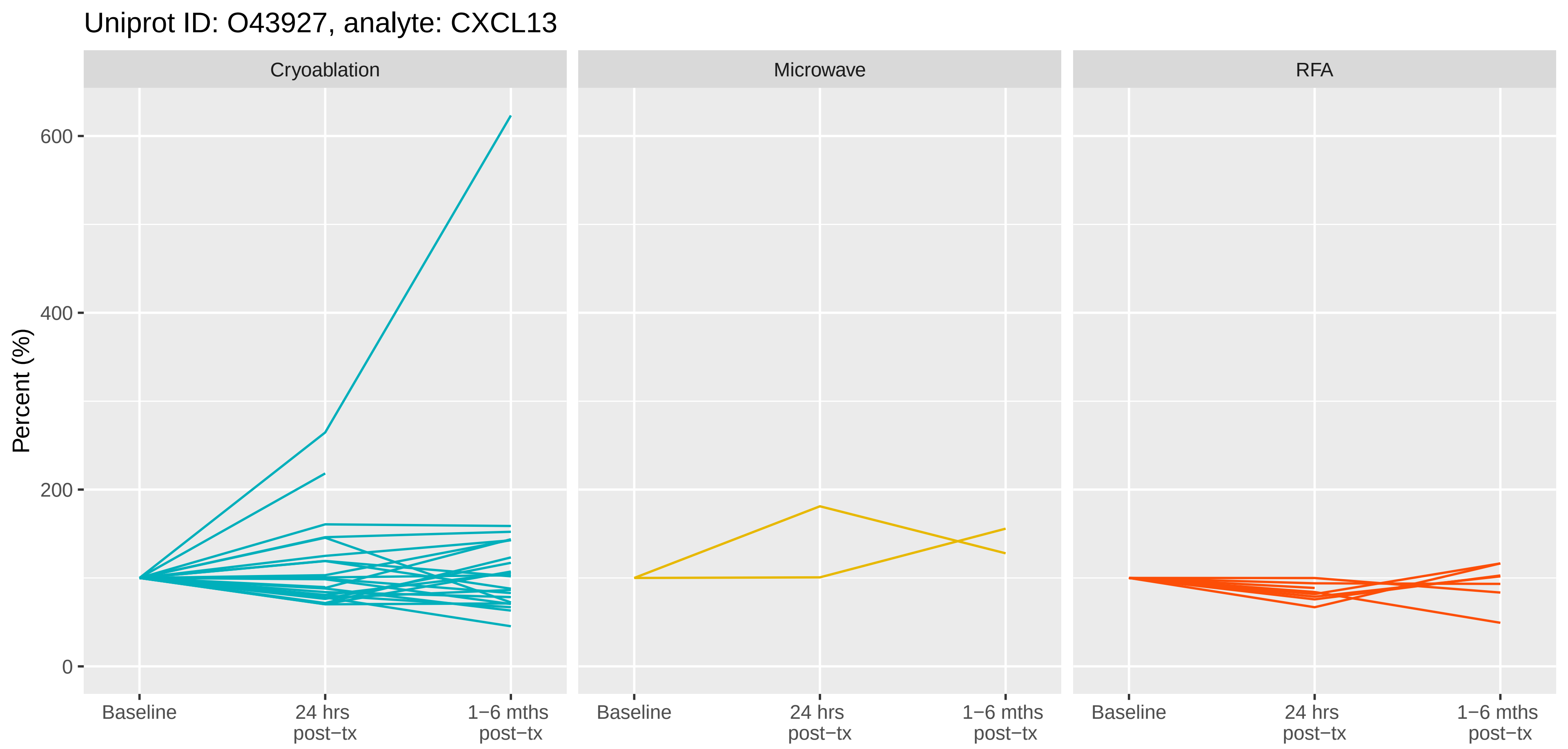

Uniprot ID: O43927, analyte: CXCL13
Cryoablation
Microwave
RFA
Percent (%)
600
400
200
0
Baseline
24 hrs post−tx
1−6 mths post−tx

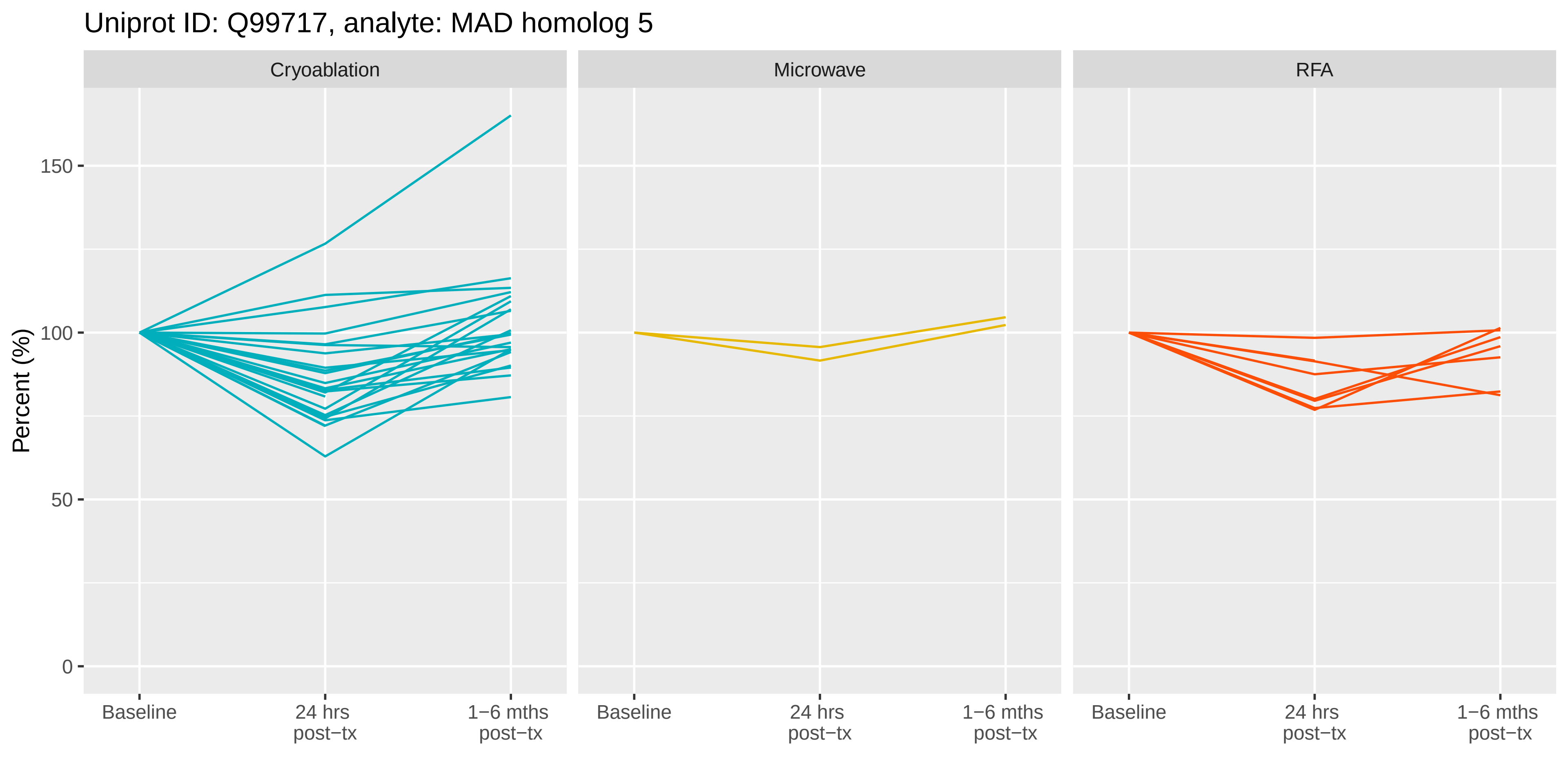

Uniprot ID: Q99717, analyte: MAD homolog 5
Cryoablation
Microwave
RFA
Percent (%)
0
50
100
150
Baseline
24 hrs post−tx
1−6 mths post−tx

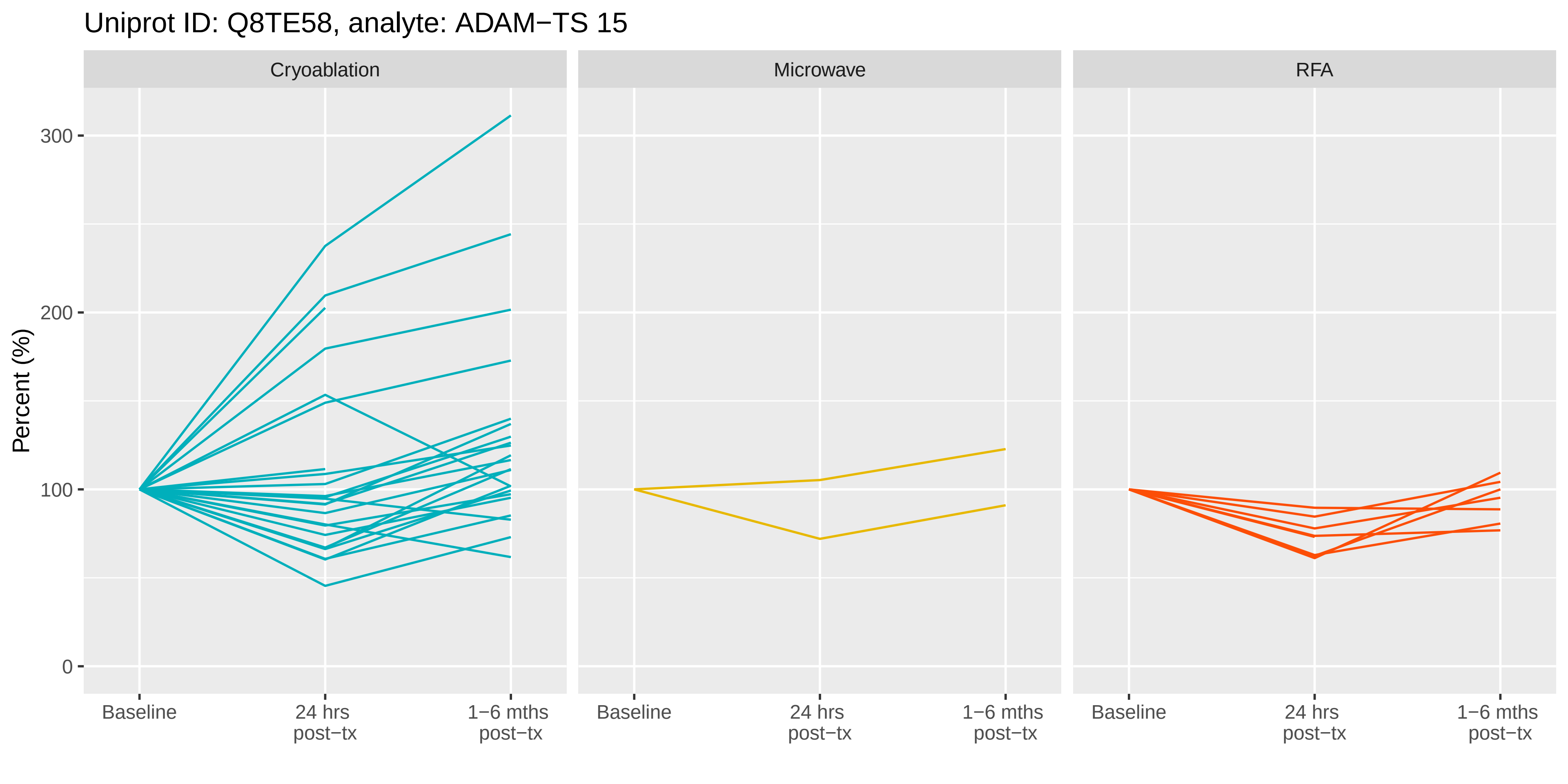

Uniprot ID: Q8TE58, analyte: ADAM−TS 15
Cryoablation
Microwave
RFA
Percent (%)
300
200
100
0
Baseline
24 hrs post−tx
1−6 mths post−tx

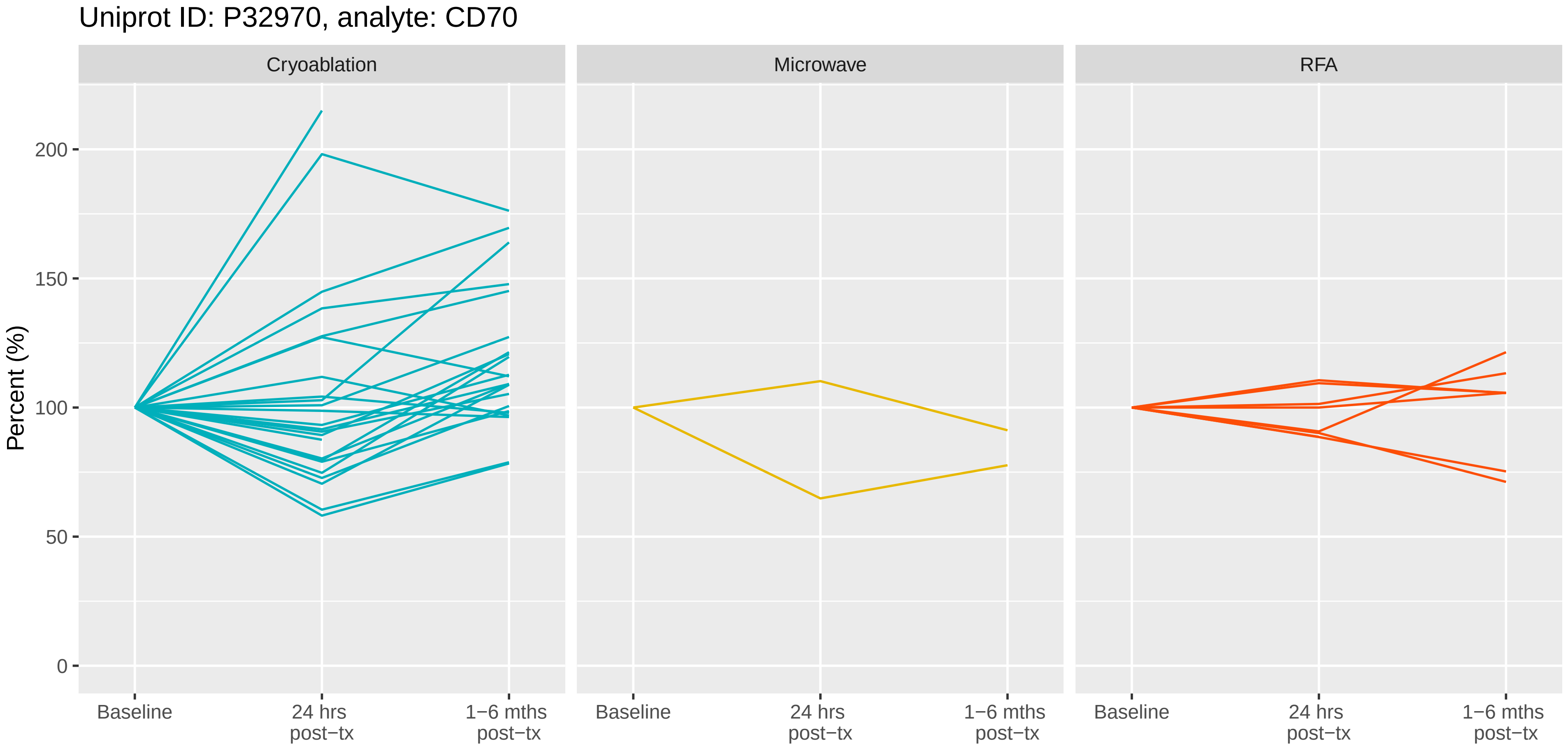
Uniprot ID: P32970, analyte: CD70
Cryoablation
Microwave
RFA
Percent (%)
0
50
100
150
200
Baseline
24 hrs post−tx
1−6 mths post−tx

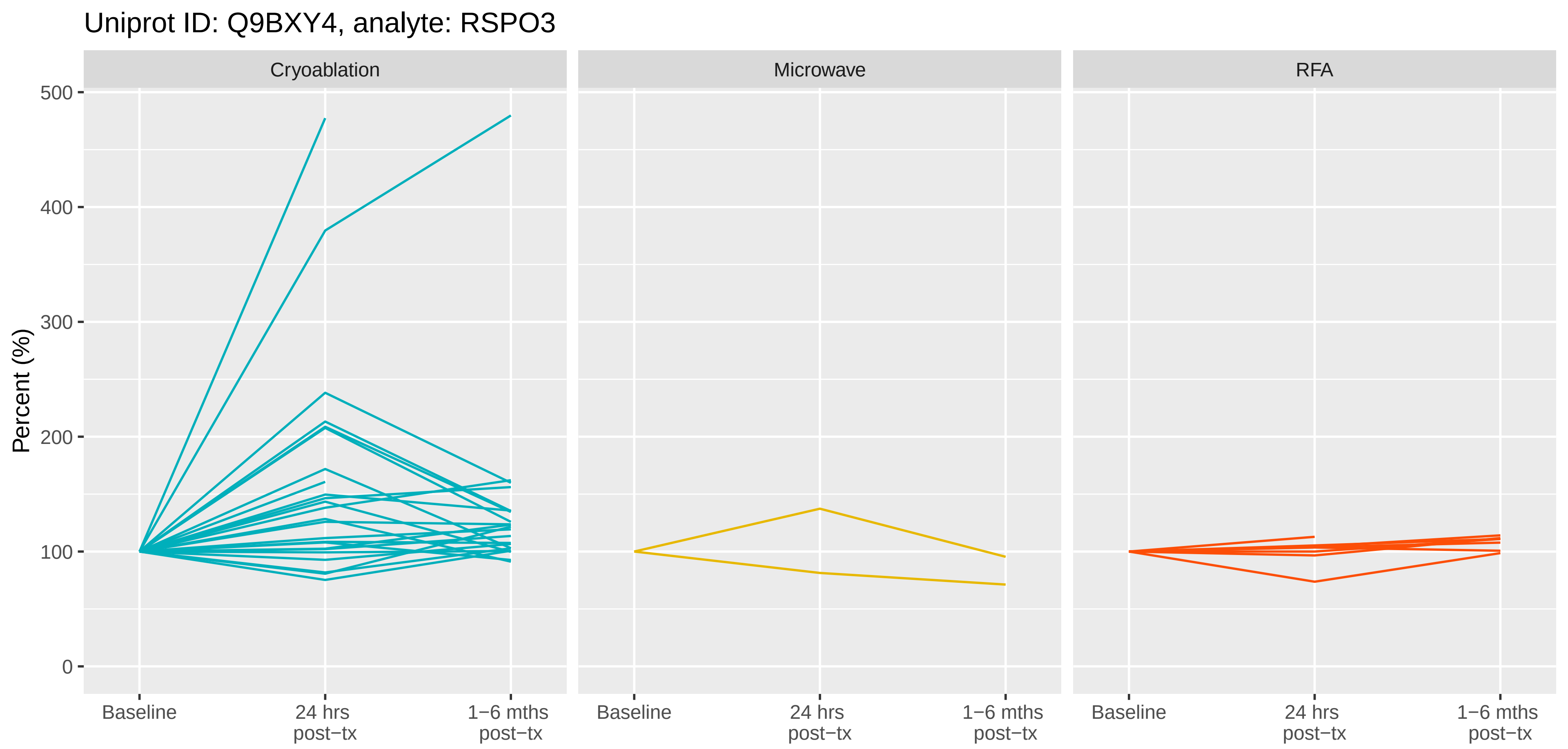
Uniprot ID: Q9BXY4, analyte: RSPO3
Cryoablation
Microwave
RFA
Percent (%)
500
400
300
200
100
0
Baseline
24 hrs post−tx
1−6 mths post−tx

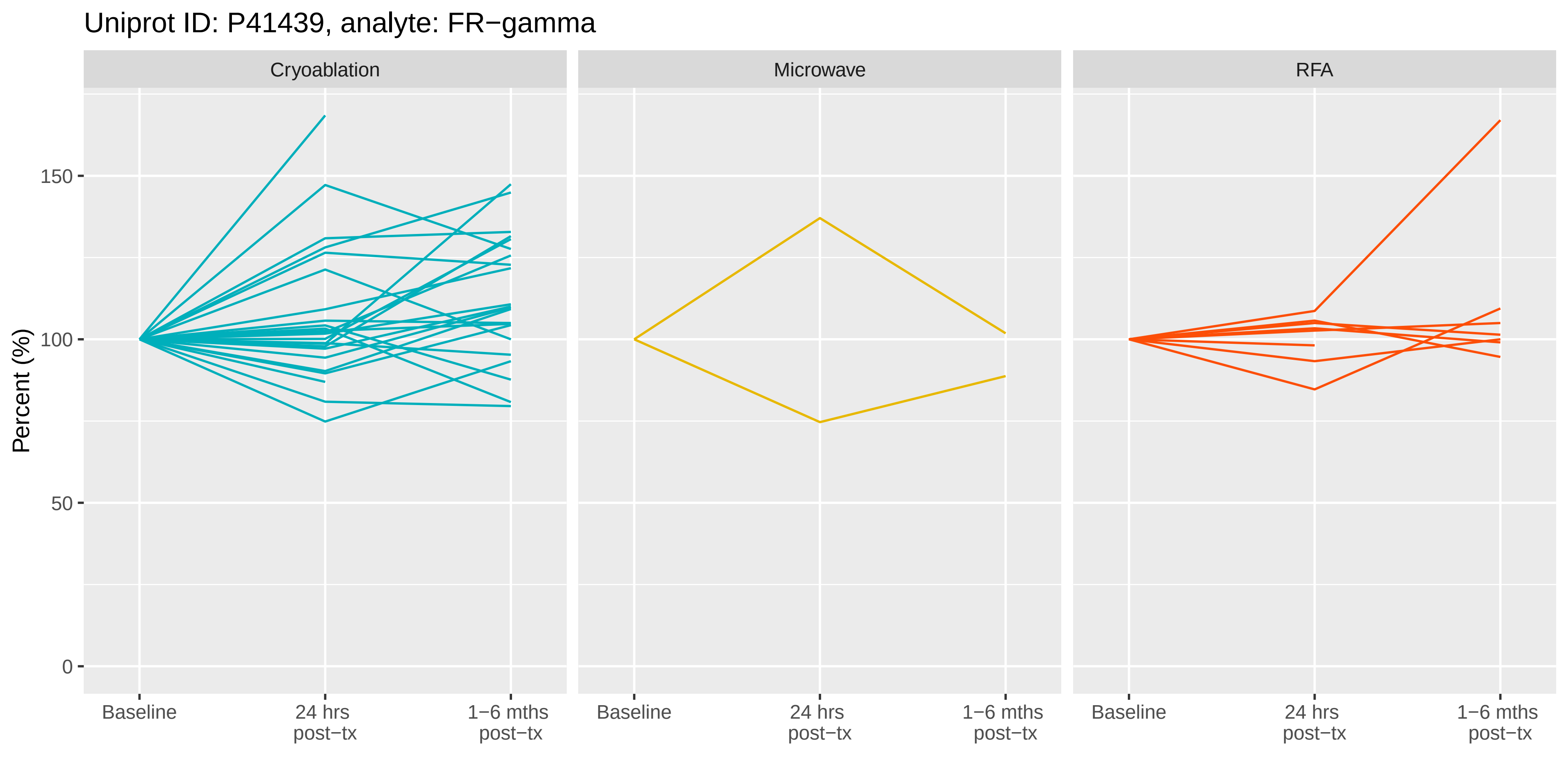
Uniprot ID: P41439, analyte: FR−gamma
Cryoablation
Microwave
RFA
Percent (%)
150
100
50
0
Baseline
24 hrs post−tx
1−6 mths post−tx

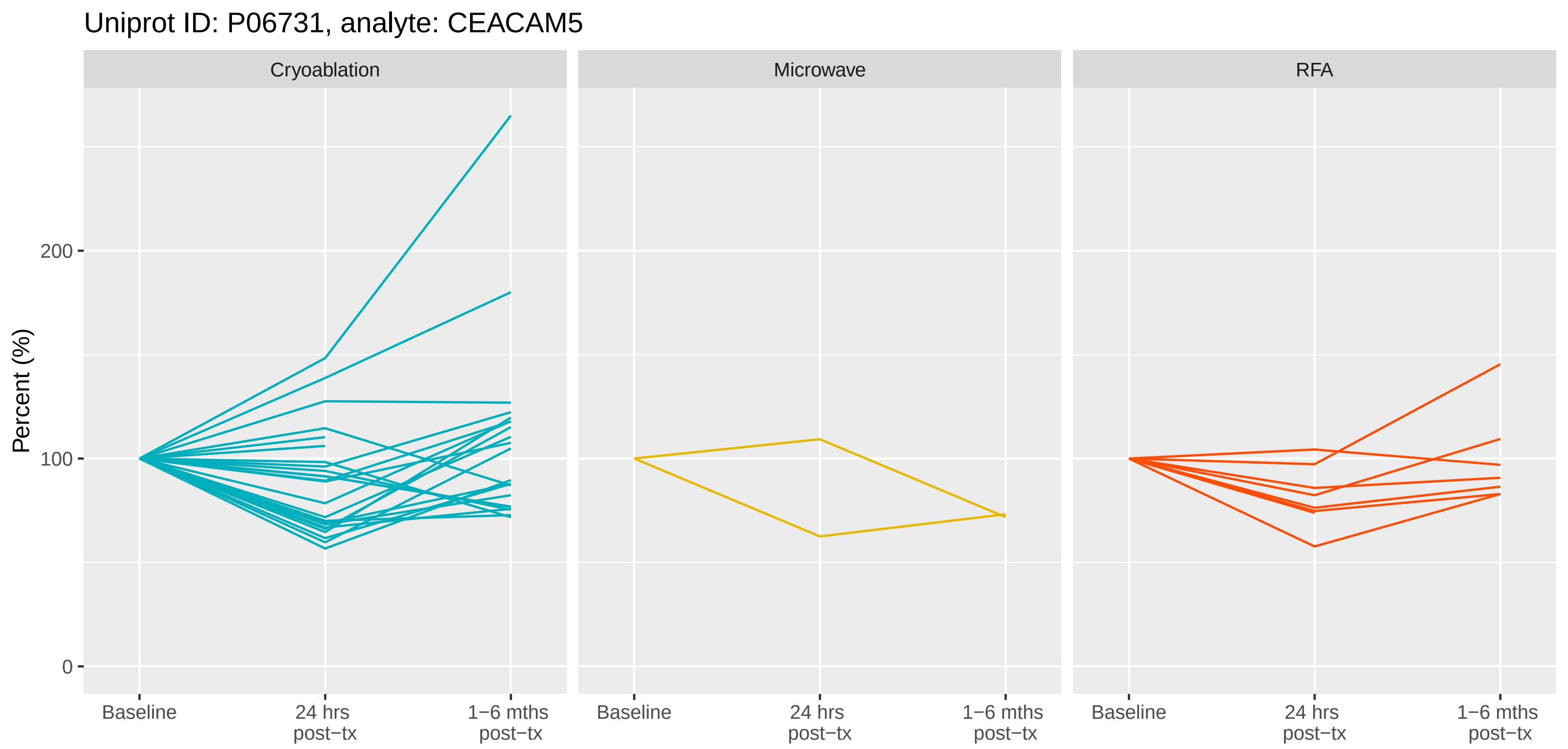

Uniprot ID: P06731, analyte: CEACAM5
Cryoablation
Microwave
RFA
Percent (%)
0
100
200
Baseline
24 hrs post−tx
1−6 mths post−tx

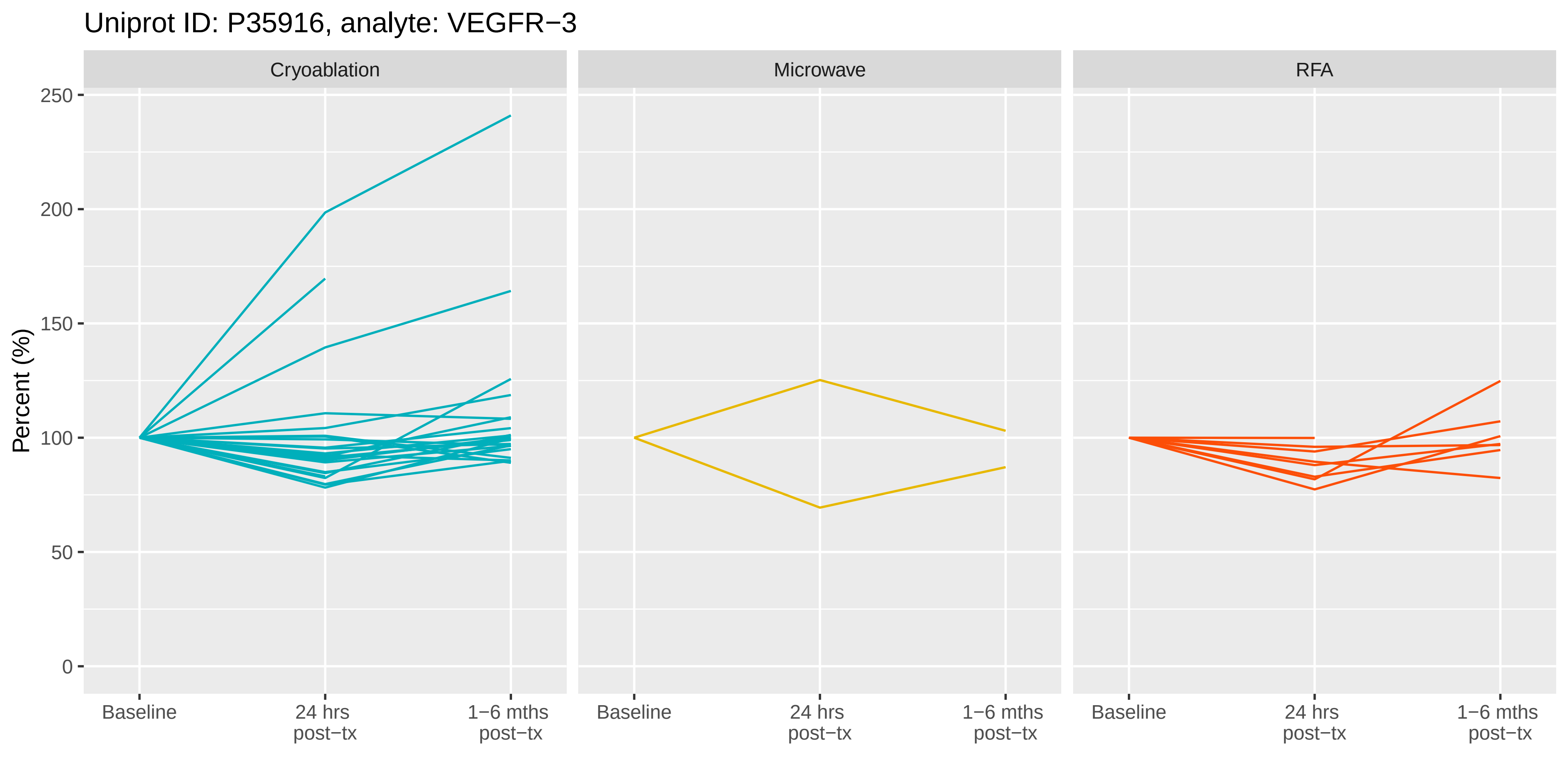

Uniprot ID: P35916, analyte: VEGFR−3
Cryoablation
Microwave
RFA
Percent (%)
250
200
150
100
50
0
Baseline
24 hrs post−tx
1−6 mths post−tx

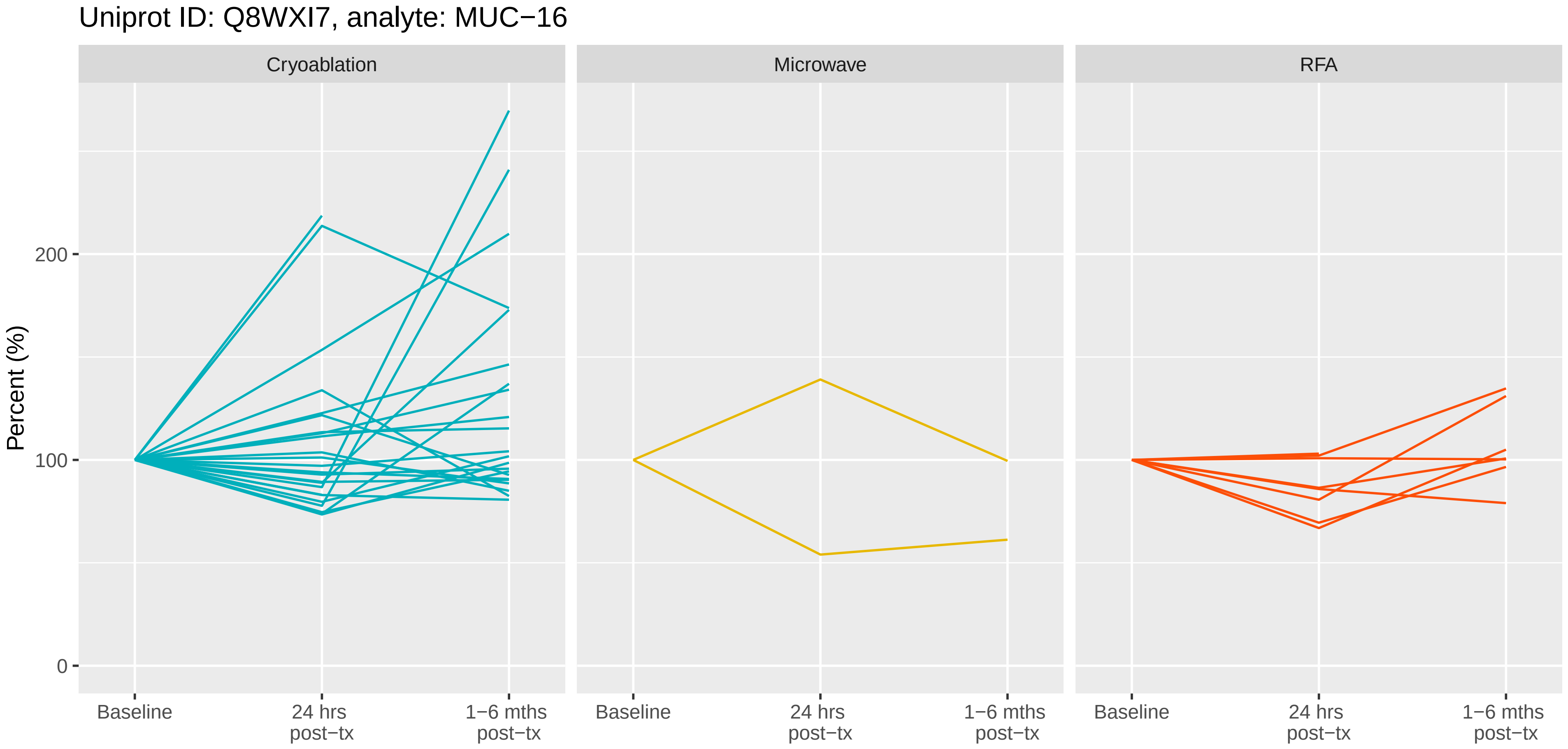

Uniprot ID: Q8WXI7, analyte: MUC−16
Cryoablation
Microwave
RFA
Percent (%)
0
100
200
Baseline
24 hrs post−tx
1−6 mths post−tx

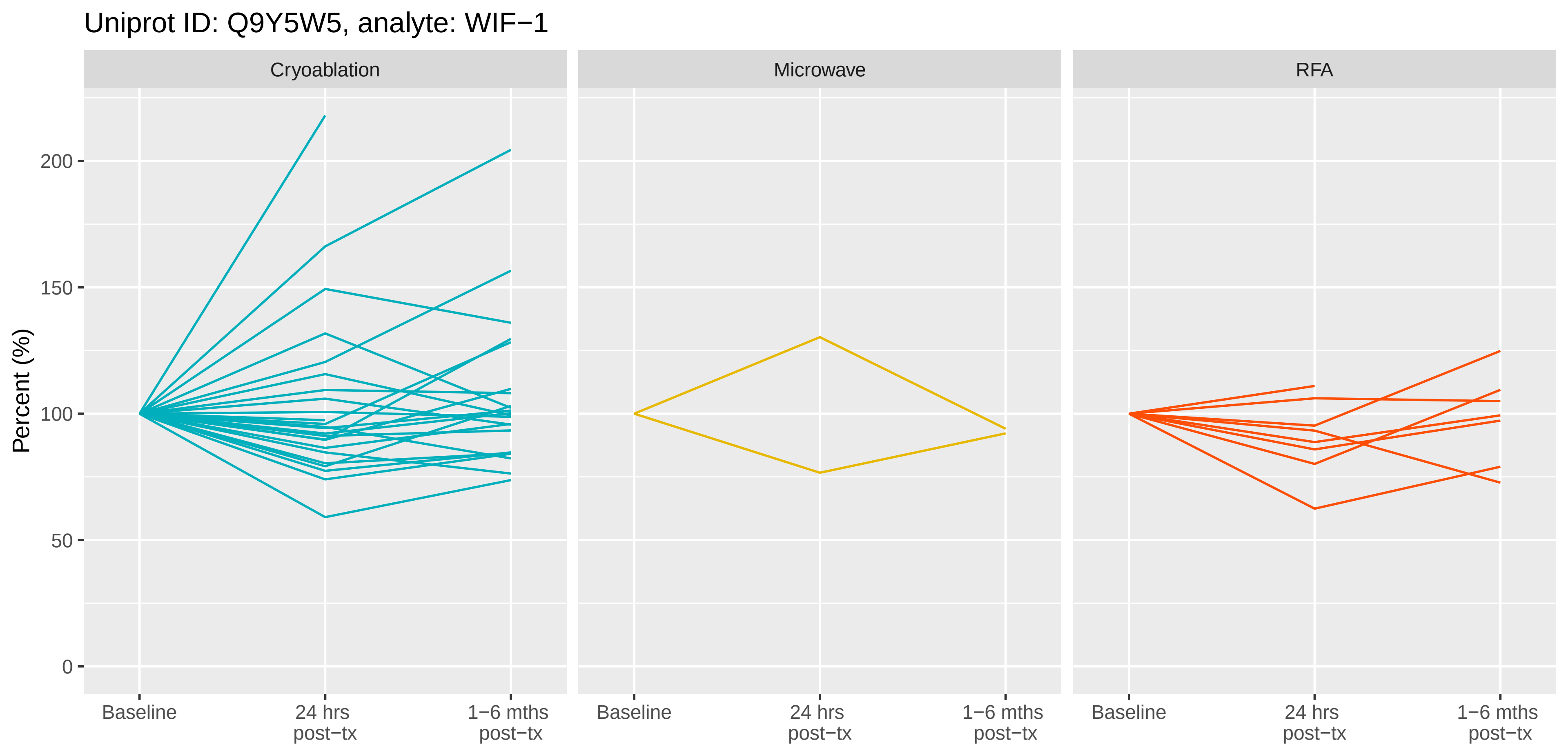

Uniprot ID: Q9Y5W5, analyte: WIF−1
Cryoablation
Microwave
RFA
Percent (%)
200
150
100
50
0
Baseline
24 hrs post−tx
1−6 mths post−tx

Uniprot ID: P10144, analyte: GZMB

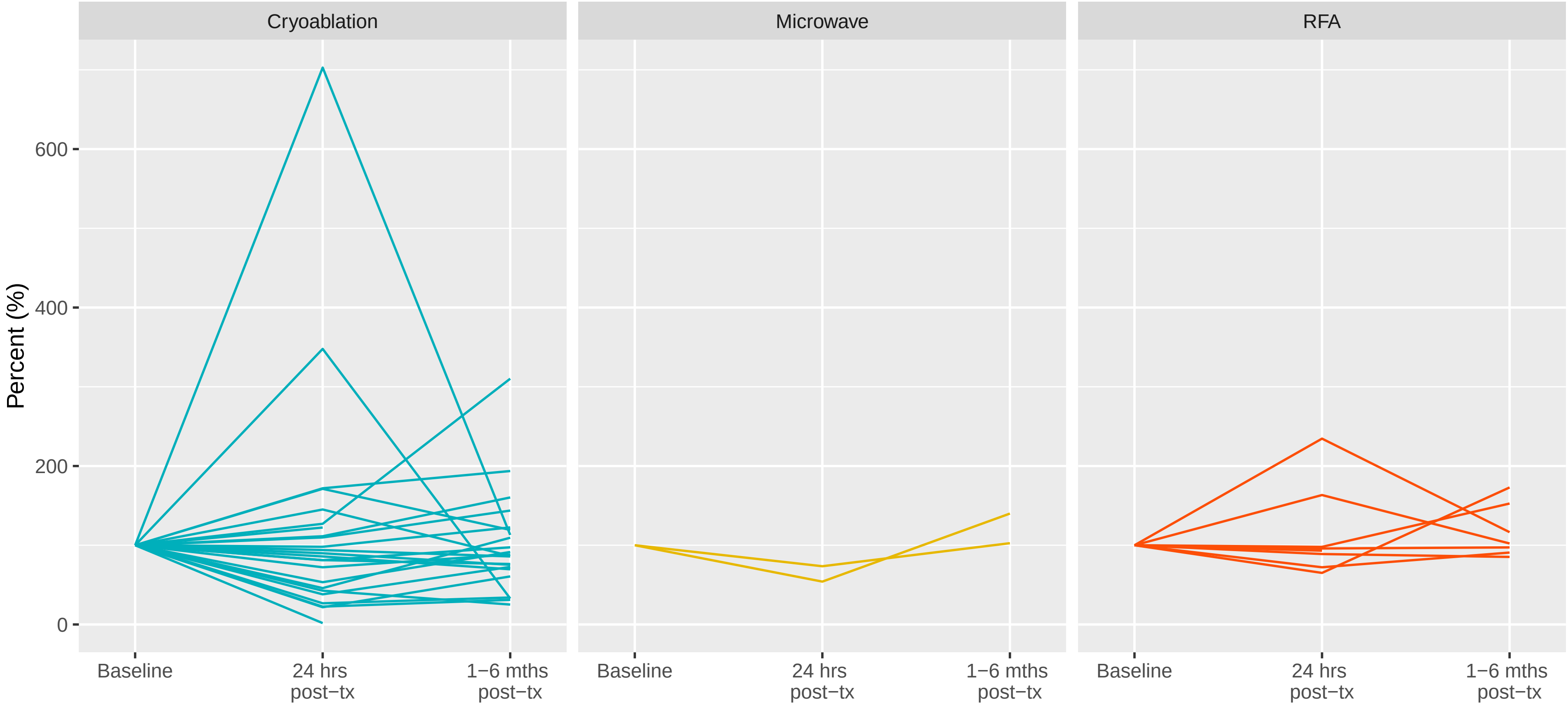

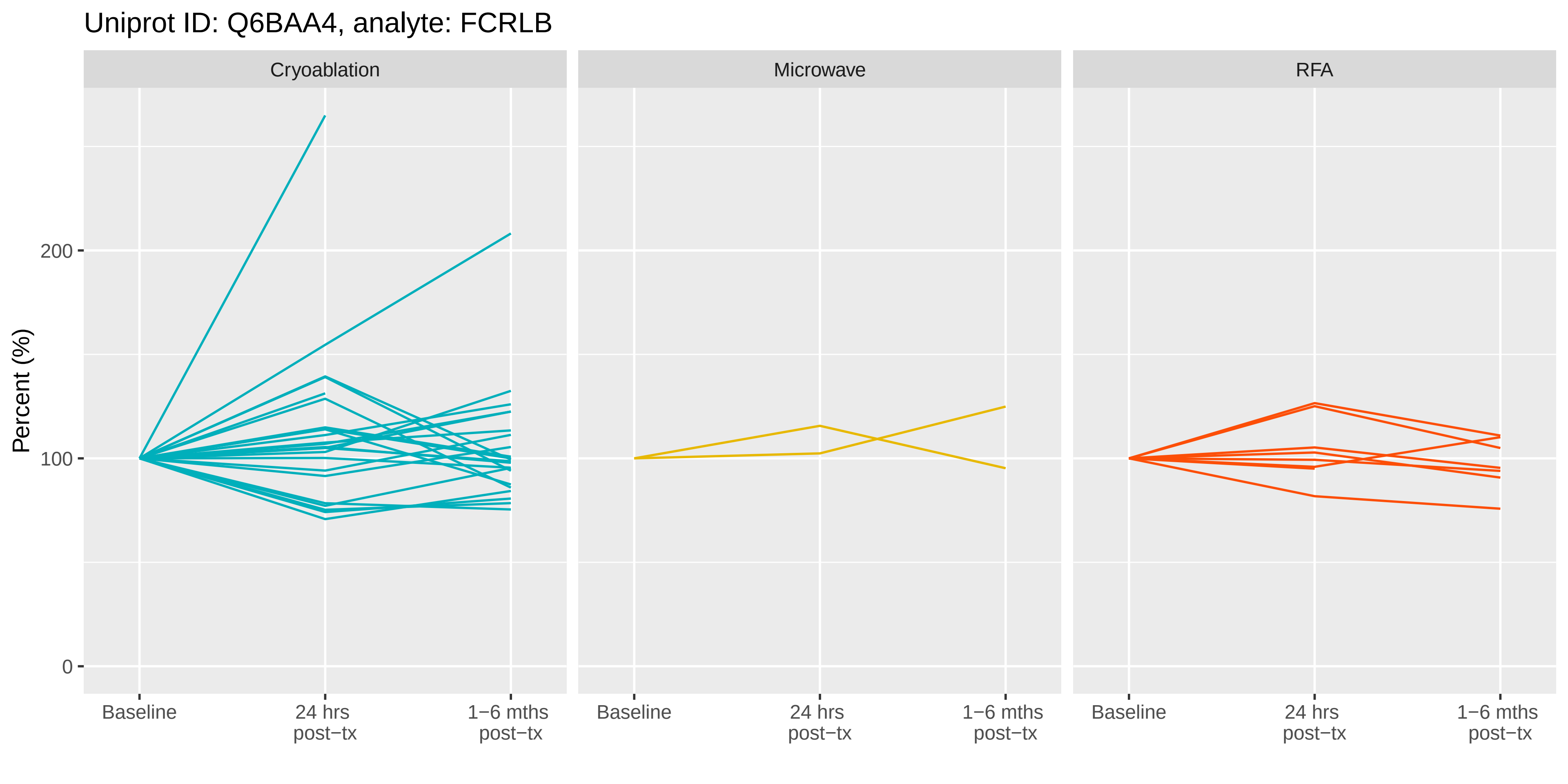
Uniprot ID: Q6BAA4, analyte: FCRLB
Cryoablation
Microwave
RFA
Percent (%)
200
100
0
Baseline
24 hrs post−tx
1−6 mths post−tx

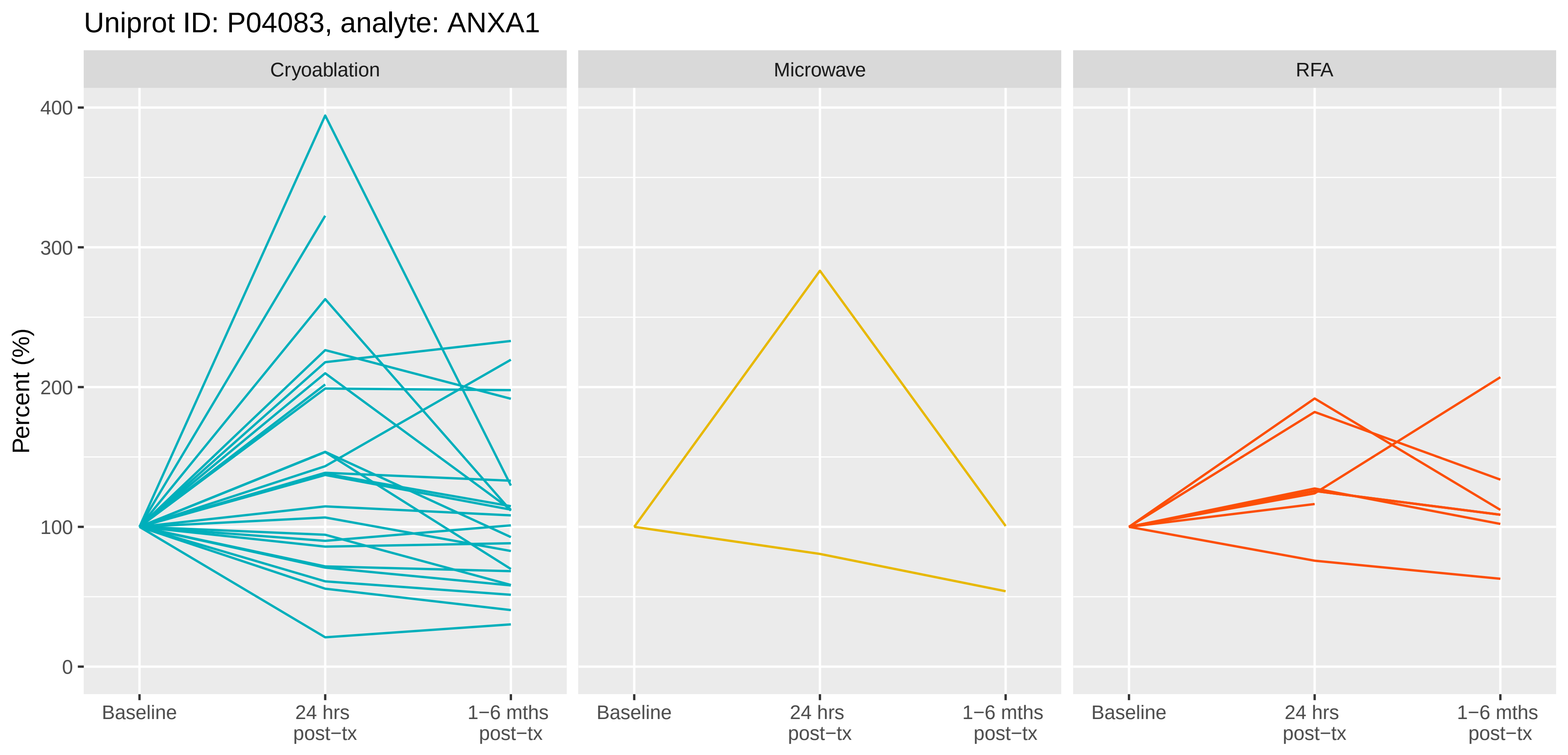
Uniprot ID: P04083, analyte: ANXA1
Cryoablation
Microwave
RFA
Percent (%)
400
300
200
100
0
Baseline
24 hrs post−tx
1−6 mths post−tx

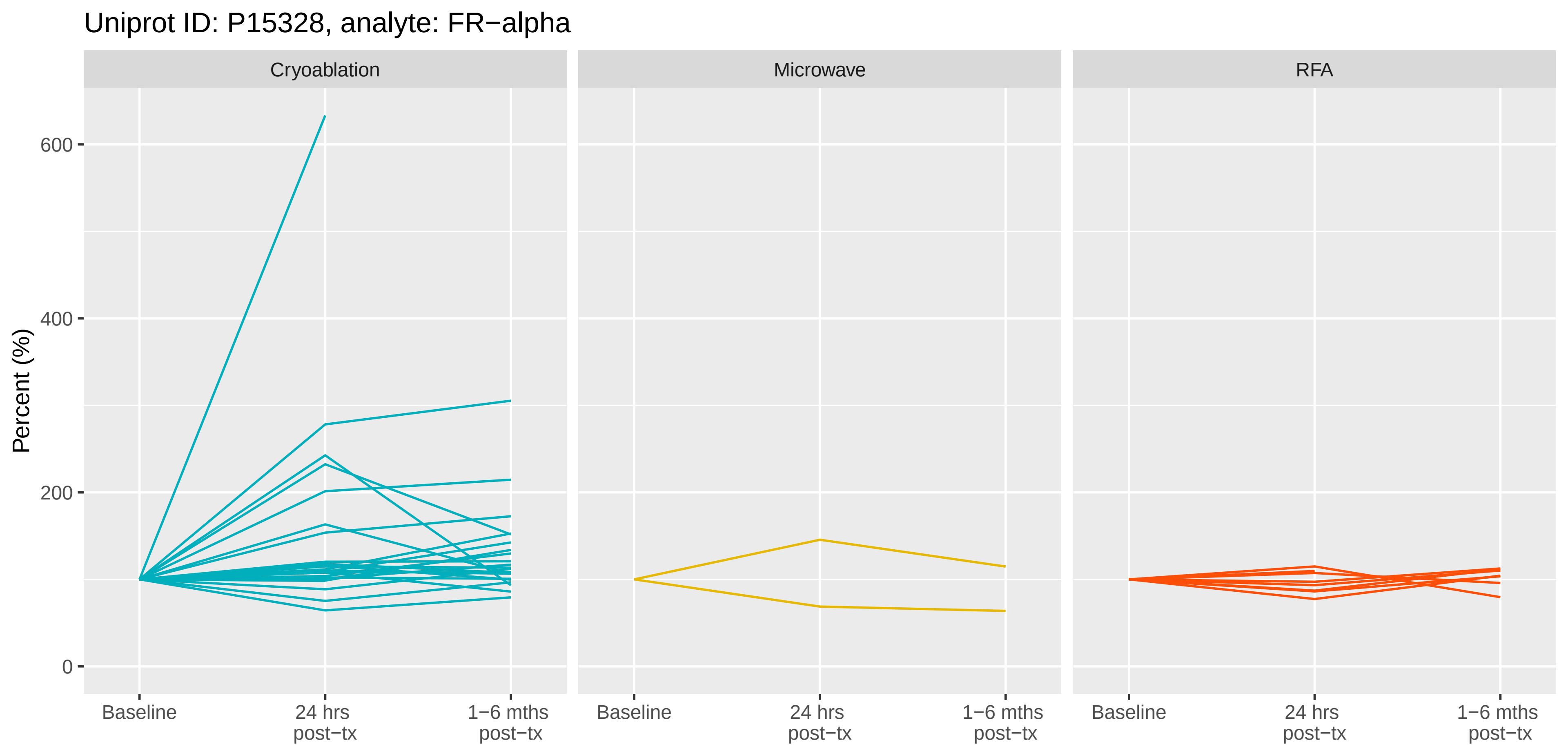
Uniprot ID: P15328, analyte: FR−alpha
Cryoablation
Microwave
RFA
Percent (%)
600
400
200
0
Baseline
24 hrs post−tx
1−6 mths post−tx
