# Supplementary material for: An Exploratory Analysis of Changes in Circulating Plasma Protein Profiles Following Image-Guided Ablation of Renal Tumours Provides Evidence for Effects on Multiple Biological Processes
Source: Cancers (Basel). 2021 Nov 30;13(23):6037. doi: 10.3390/cancers13236037 (PMC8656737; doi:10.3390/cancers13236037)
Supplement: Supplementary file 1 [file cancers-13-06037-s001.zip › Supplementary File/Figure S5.pdf]

Figure S5. Longitudinal profiles of plasma proteins which increased significantly ($p<0.05$ and $q<0.01$; TP3 vs TP2) in patients 24 hours post-CRYO (n=24 patients as 1 TP3 sample failed analytical QC). Results for each protein are expressed as a % of the reference baseline (TP2; 100%).

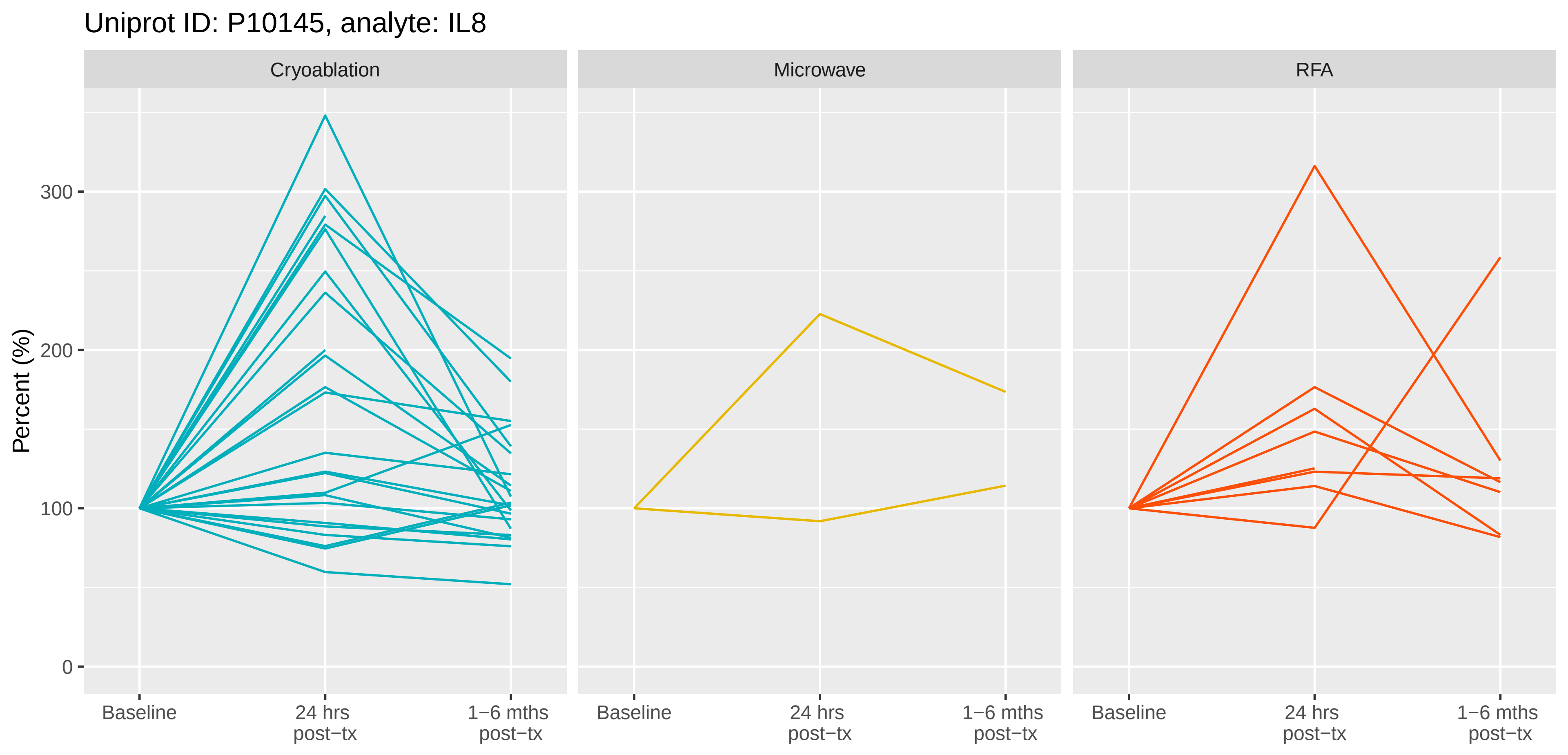

Uniprot ID: P10145, analyte: IL8
Cryoablation
Microwave
RFA
Percent (%)
0
100
200
300
Baseline
24 hrs post−tx
1−6 mths post−tx

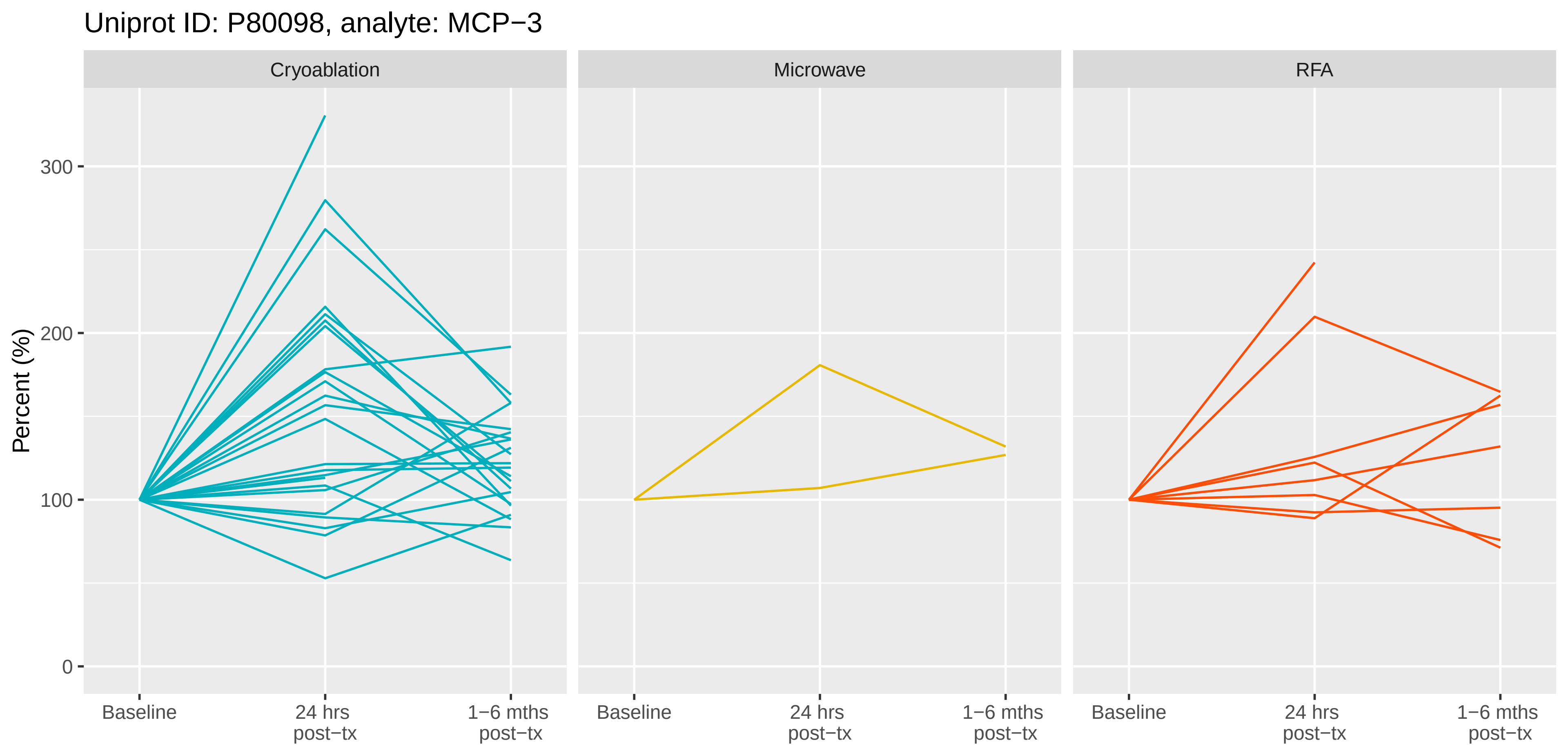

Uniprot ID: P80098, analyte: MCP−3
Cryoablation
Microwave
RFA
Percent (%)
0
100
200
300
Baseline
24 hrs post−tx
1−6 mths post−tx

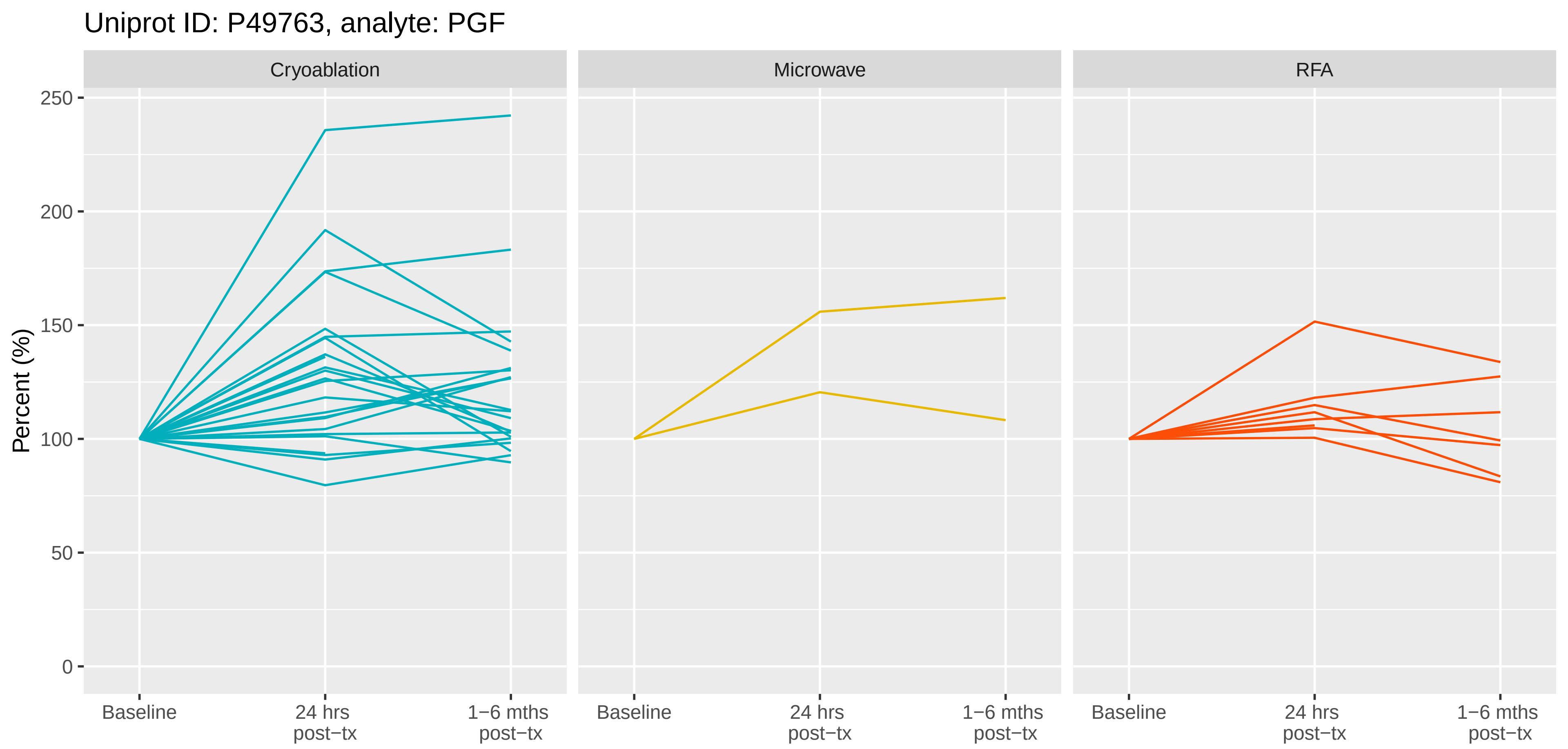

Uniprot ID: P49763, analyte: PGF
Cryoablation
Microwave
RFA
Percent (%)
250
200
150
100
50
0
Baseline
24 hrs post−tx
1−6 mths post−tx

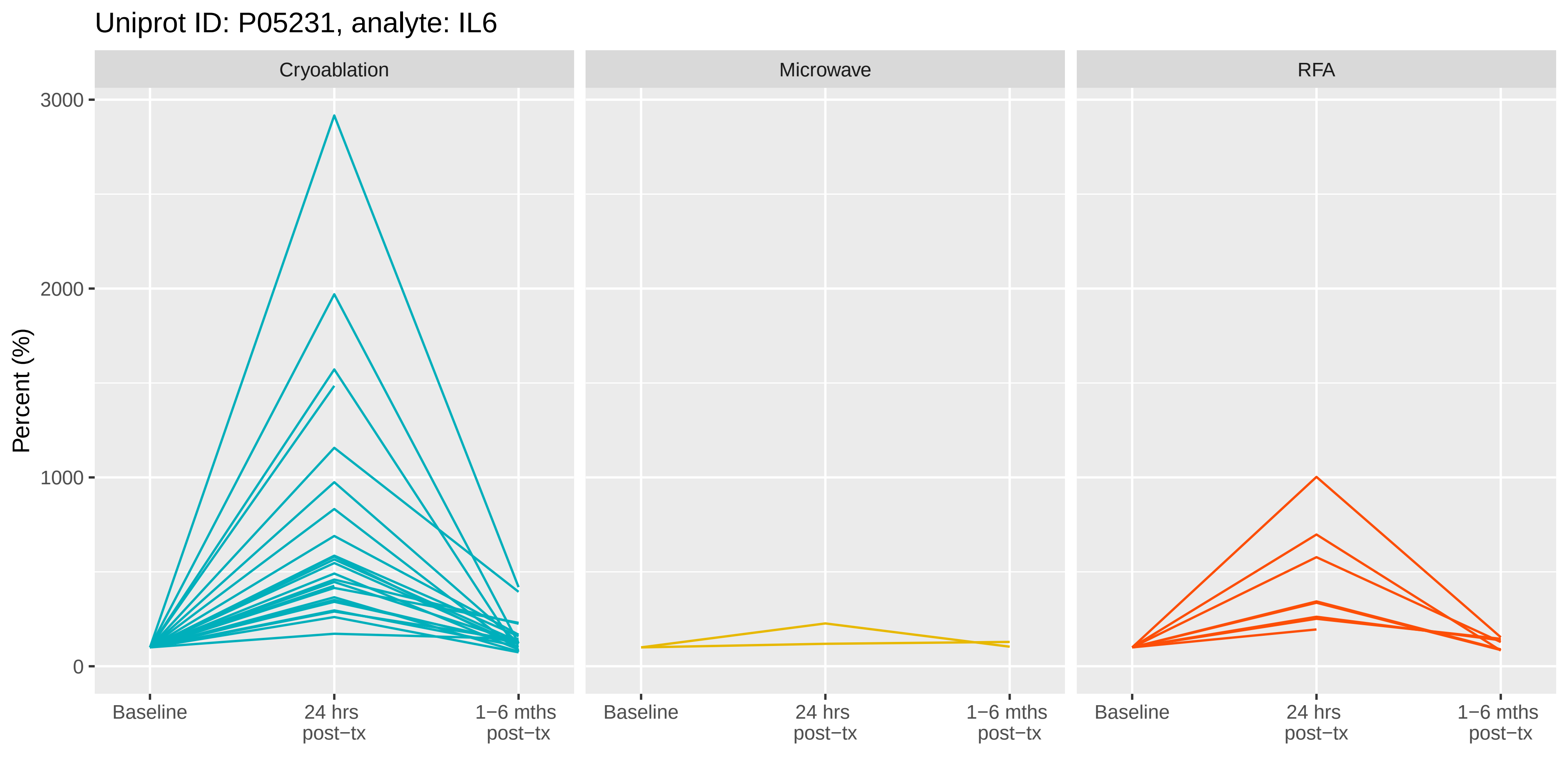

Uniprot ID: P05231, analyte: IL6
Cryoablation
Microwave
RFA
Percent (%)
3000
2000
1000
0
Baseline
24 hrs post−tx
1−6 mths post−tx

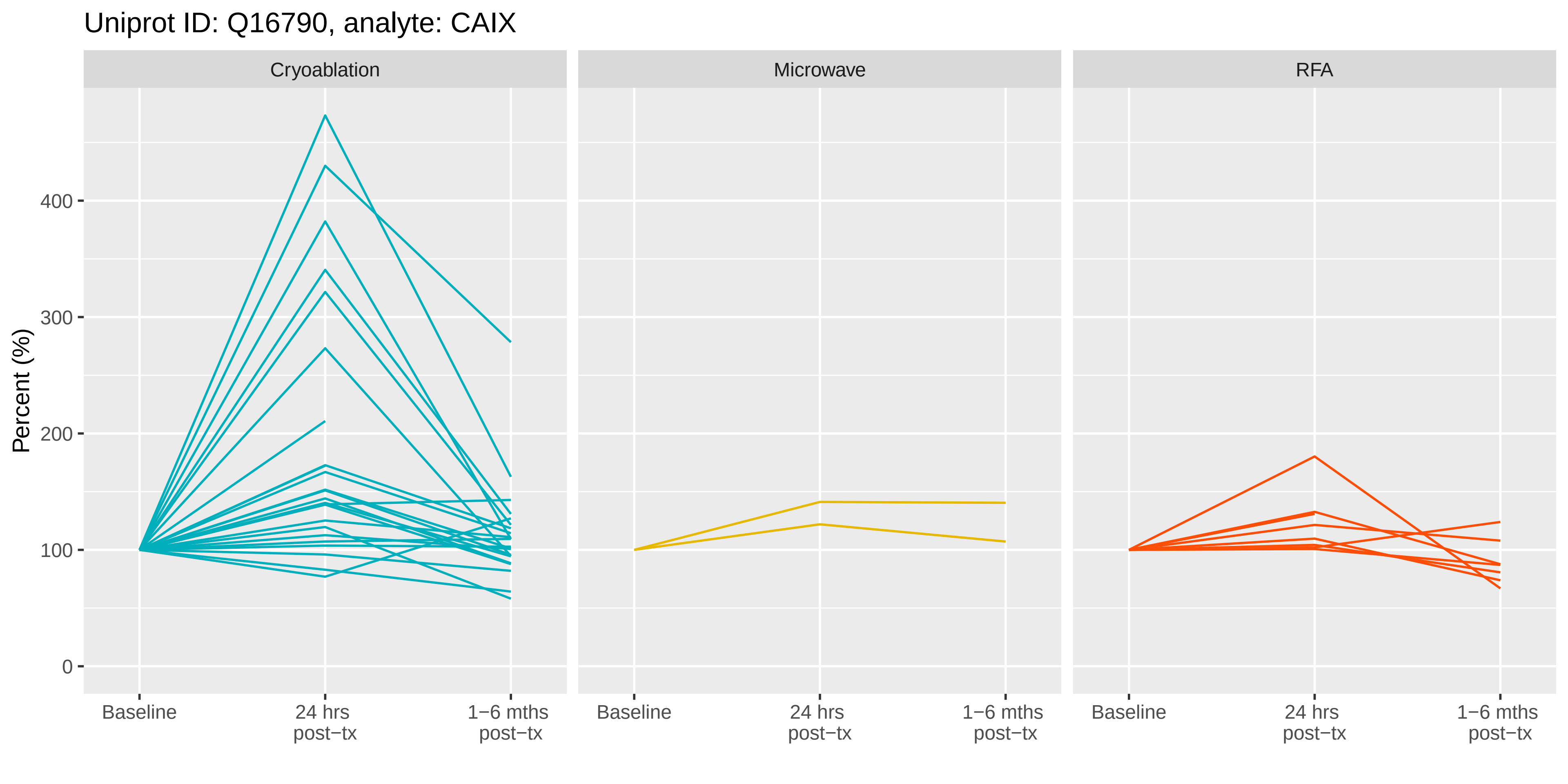

Uniprot ID: Q16790, analyte: CAIX
Cryoablation
Microwave
RFA
Percent (%)
0
100
200
300
400
Baseline
24 hrs
post−tx
1−6 mths
post−tx

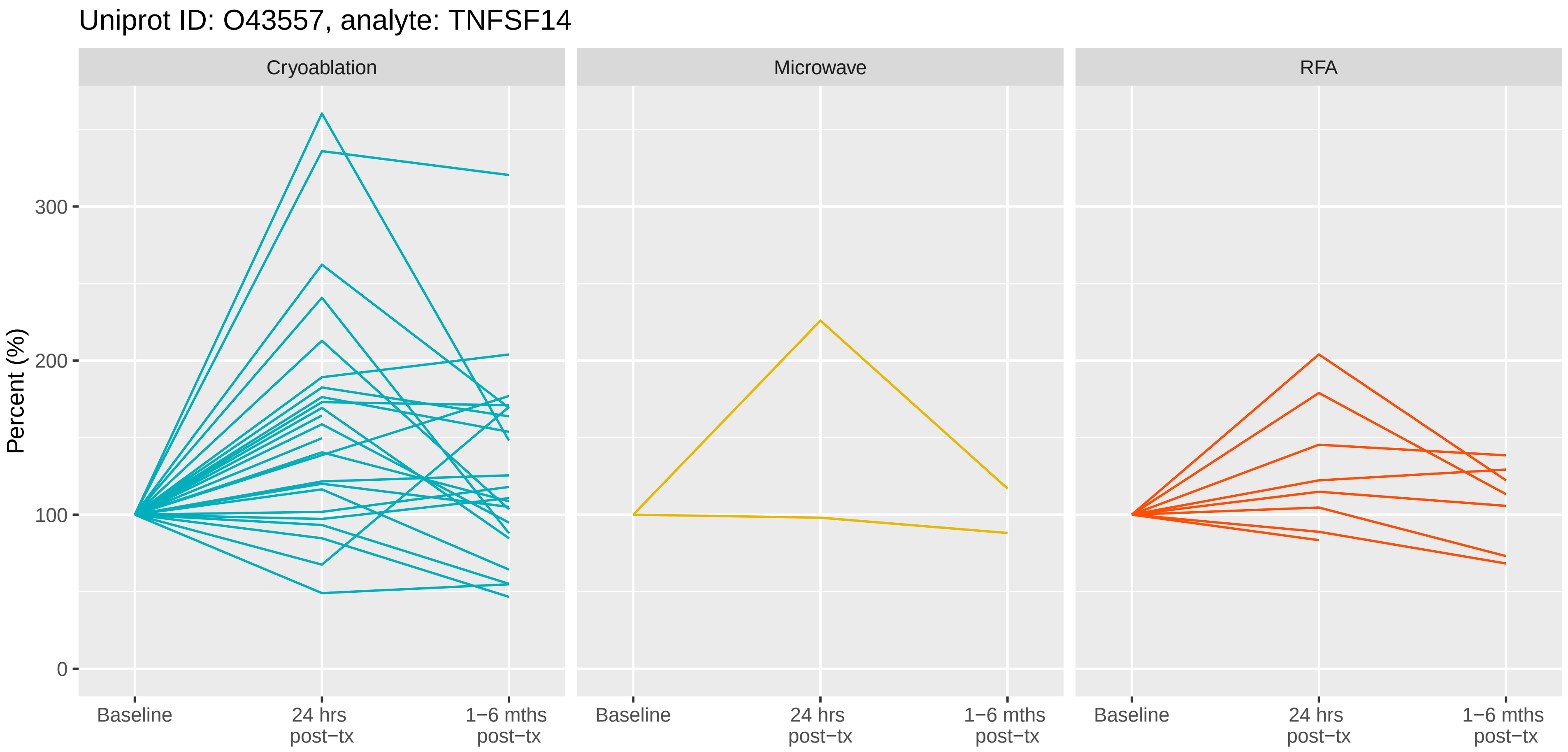

Uniprot ID: O43557, analyte: TNFSF14
Cryoablation
Microwave
RFA
Percent (%)
300
200
100
0
Baseline
24 hrs post−tx
1−6 mths post−tx

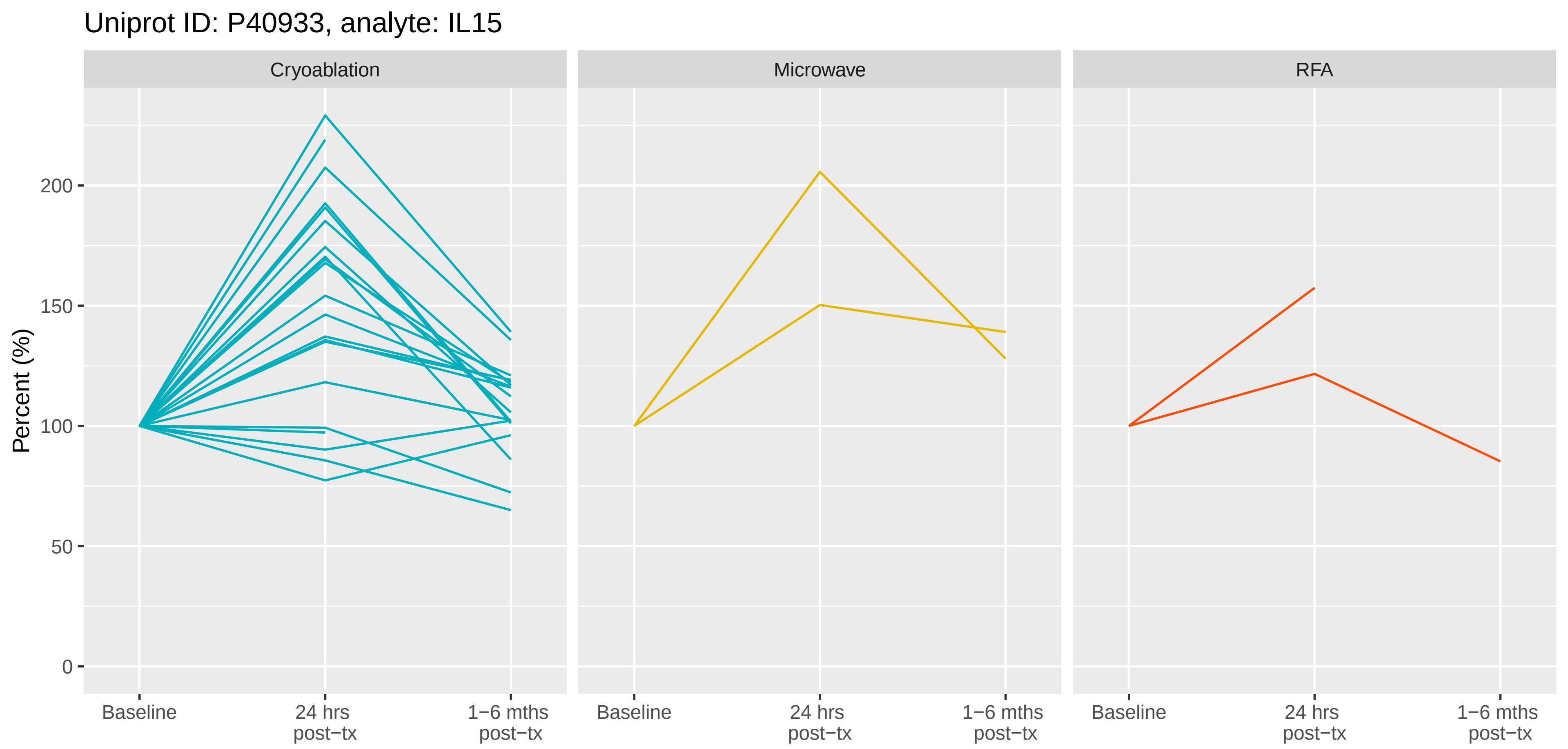
Uniprot ID: P40933, analyte: IL15
Cryoablation
Microwave
RFA
Percent (%)
0
50
100
150
200
Baseline
24 hrs post−tx
1−6 mths post−tx

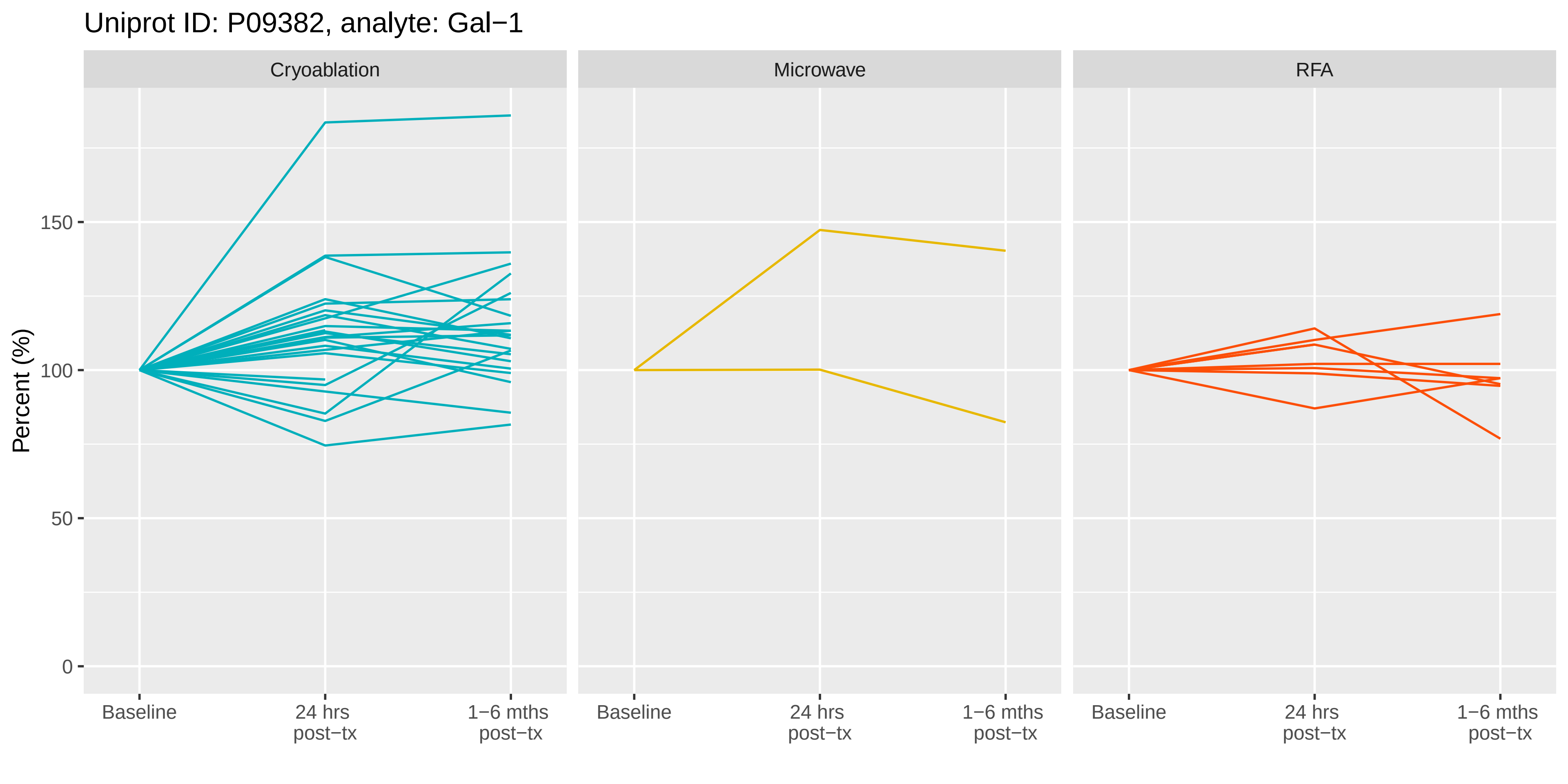
Uniprot ID: P09382, analyte: Gal−1
Cryoablation
Microwave
RFA
Percent (%)
150
100
50
0
Baseline
24 hrs post−tx
1−6 mths post−tx

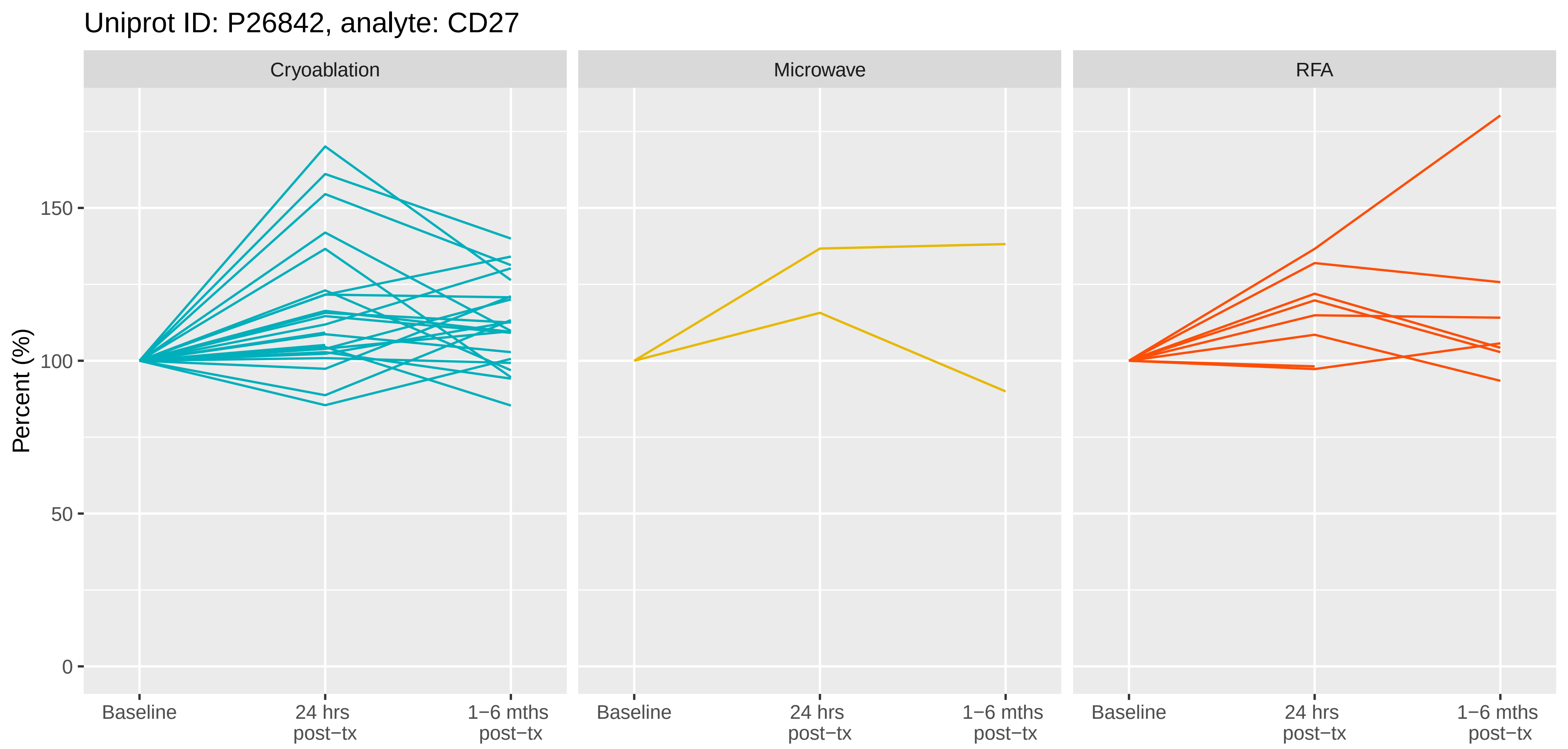
Uniprot ID: P26842, analyte: CD27
Cryoablation
Microwave
RFA
Percent (%)
150
100
50
0
Baseline
24 hrs post−tx
1−6 mths post−tx

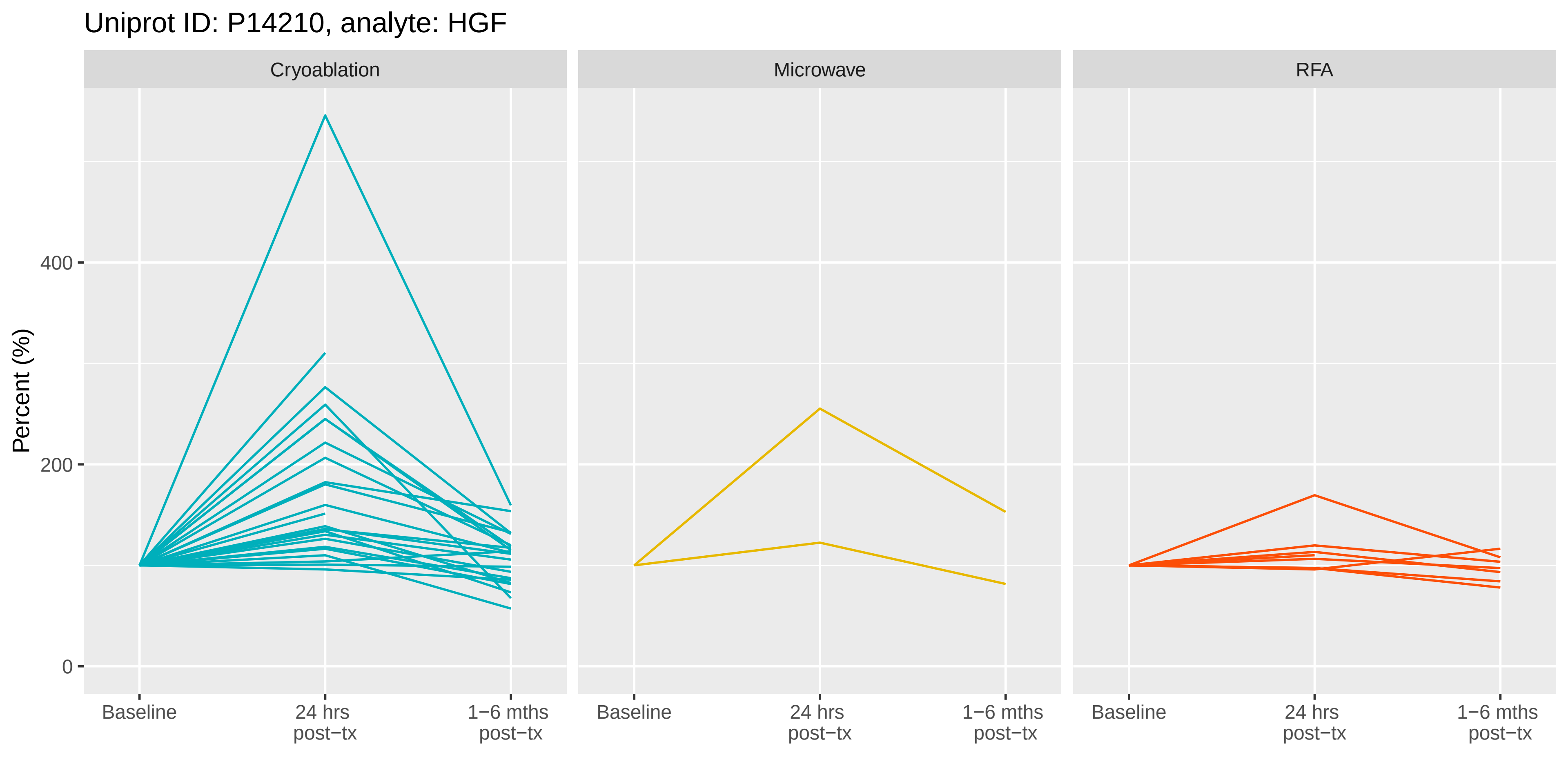
Uniprot ID: P14210, analyte: HGF
Cryoablation
Microwave
RFA
Percent (%)
400
200
0
Baseline
24 hrs post−tx
1−6 mths post−tx
Baseline
24 hrs post−tx
1−6 mths post−tx
Baseline
24 hrs post−tx
1−6 mths post−tx

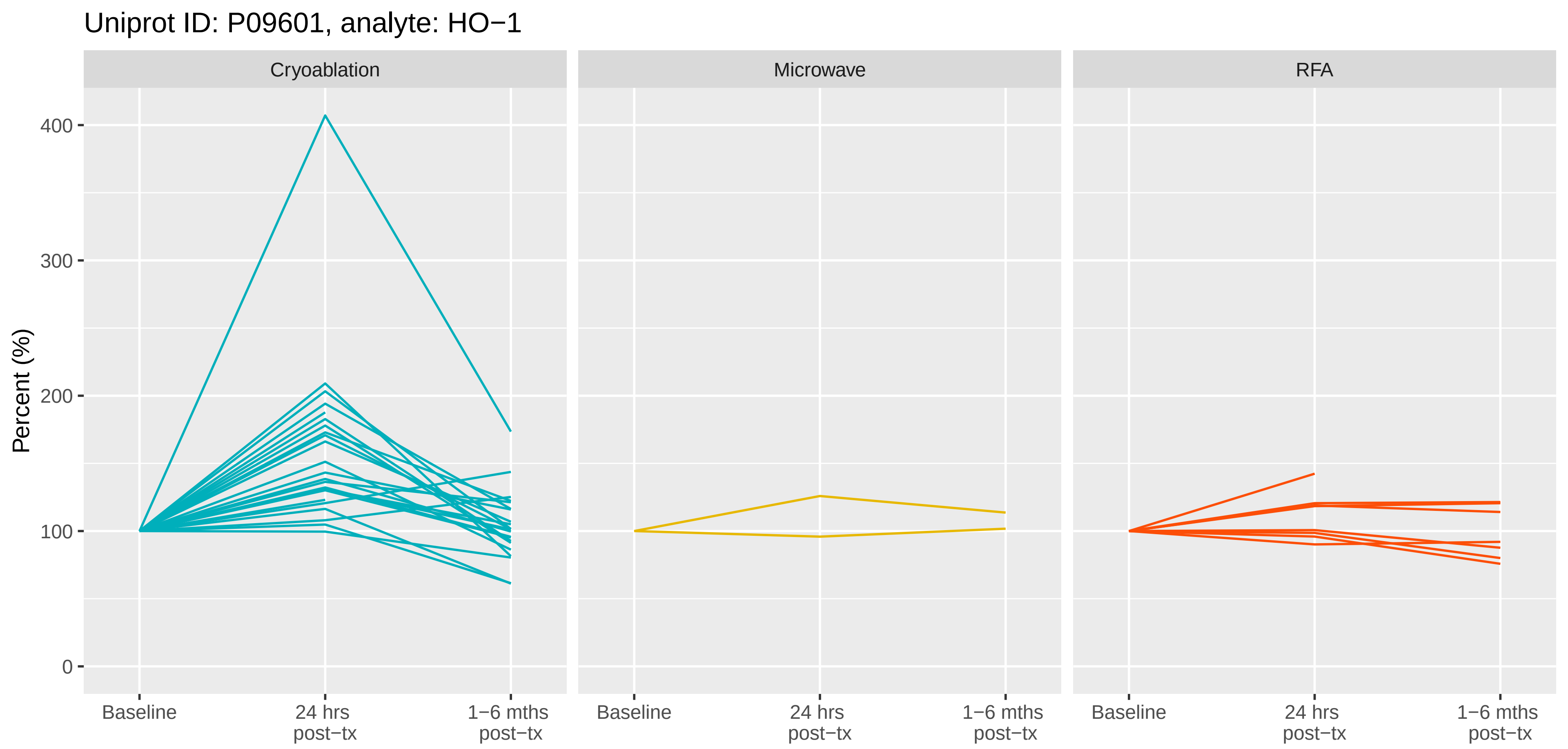

Uniprot ID: P09601, analyte: HO−1
Cryoablation
Microwave
RFA
Percent (%)
400
300
200
100
0
Baseline
24 hrs post−tx
1−6 mths post−tx
Baseline
24 hrs post−tx
1−6 mths post−tx
Baseline
24 hrs post−tx
1−6 mths post−tx

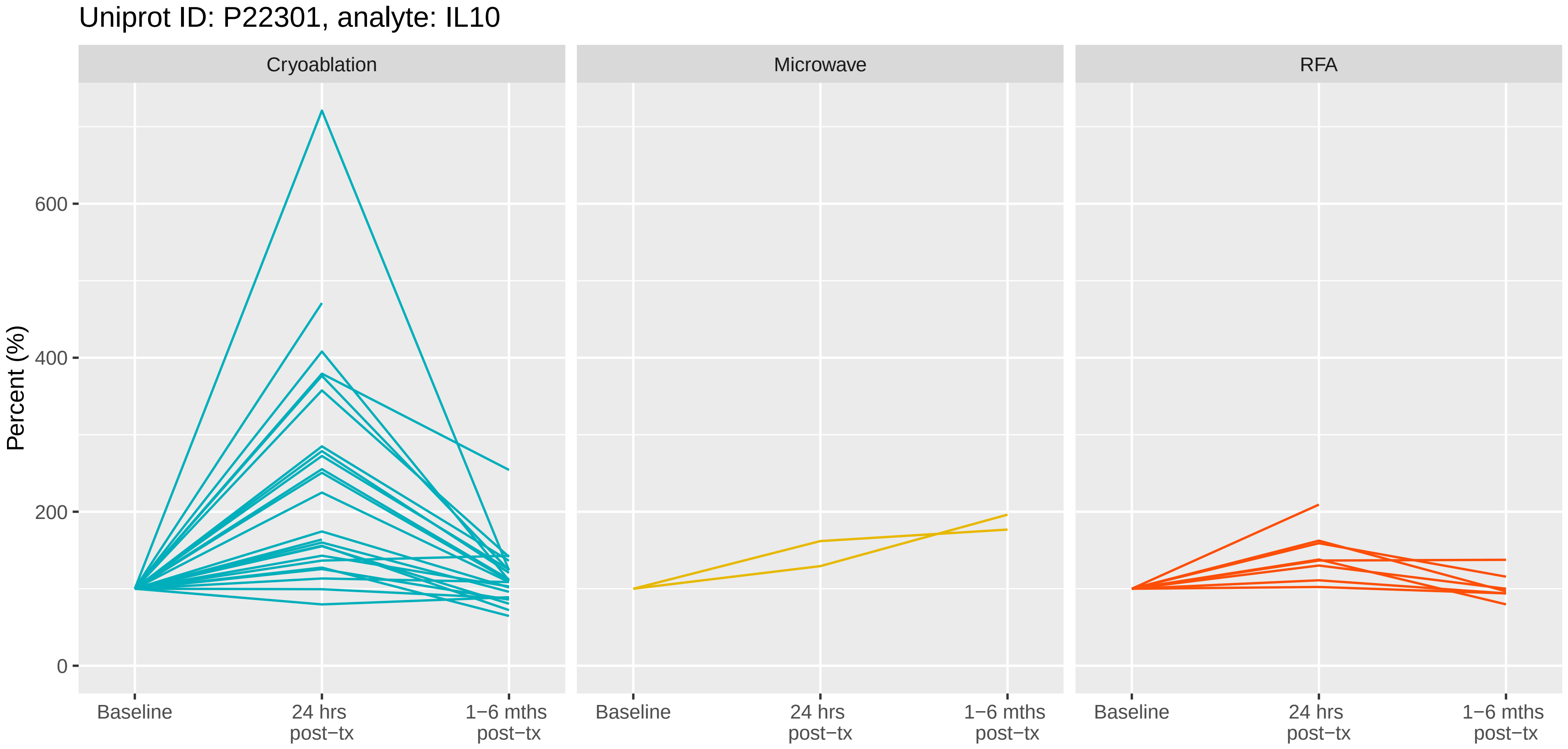

Uniprot ID: P22301, analyte: IL10
Cryoablation
Microwave
RFA
Percent (%)
600
400
200
0
Baseline
24 hrs post−tx
1−6 mths post−tx

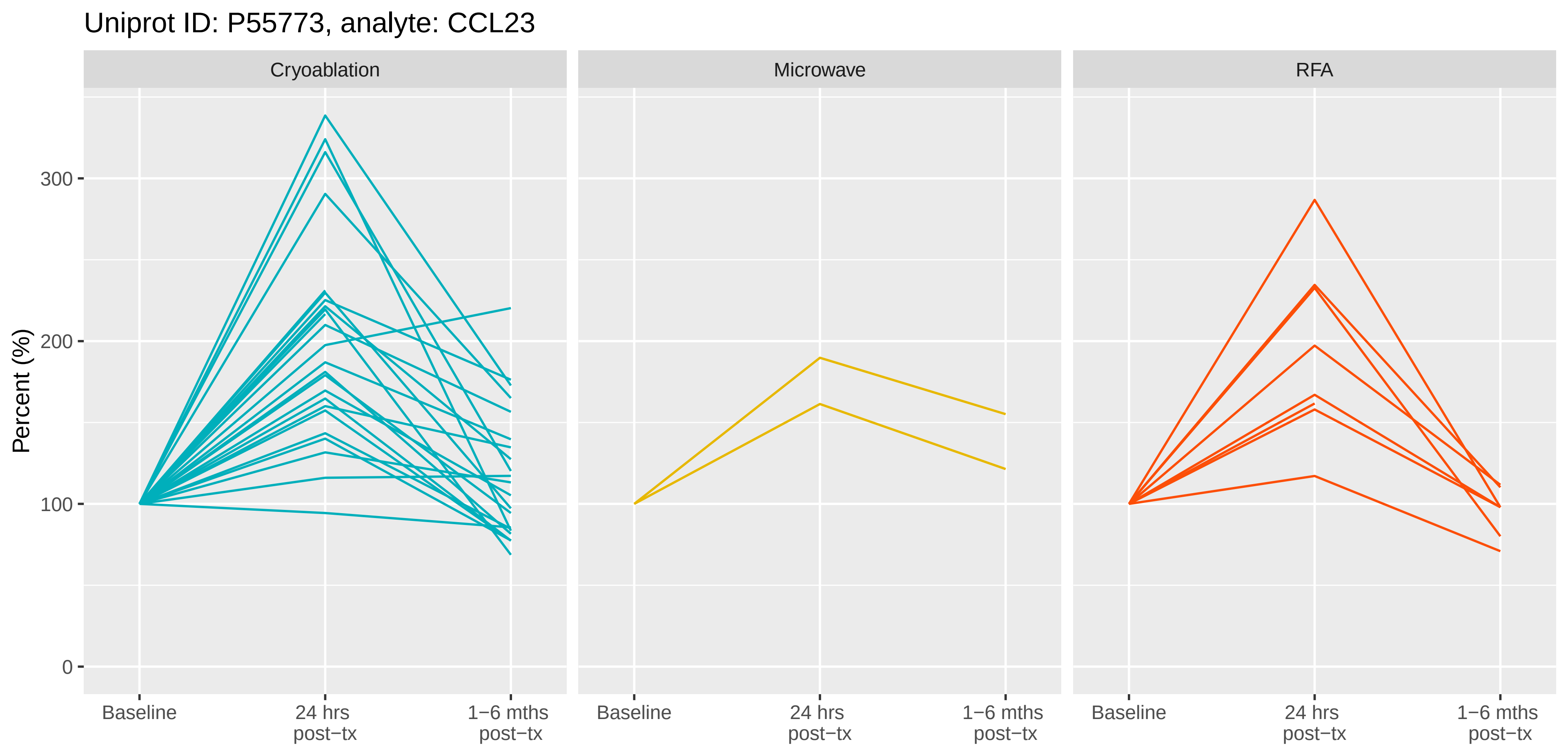

Uniprot ID: P55773, analyte: CCL23
Cryoablation
Microwave
RFA
Percent (%)
300
200
100
0
Baseline
24 hrs post−tx
1−6 mths post−tx

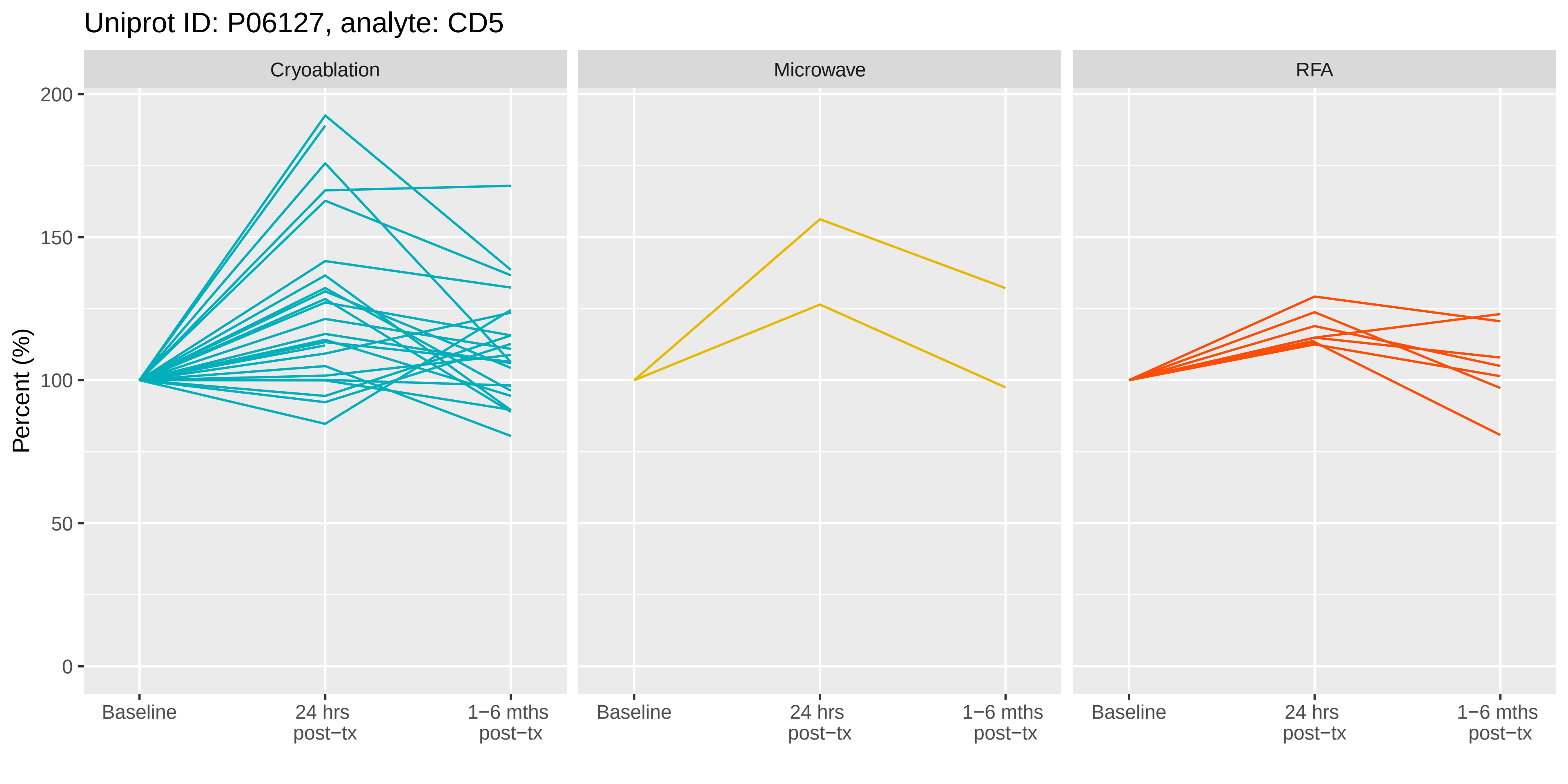

Uniprot ID: P06127, analyte: CD5
Cryoablation
Microwave
RFA
Percent (%)
200
150
100
50
0
Baseline
24 hrs post−tx
1−6 mths post−tx

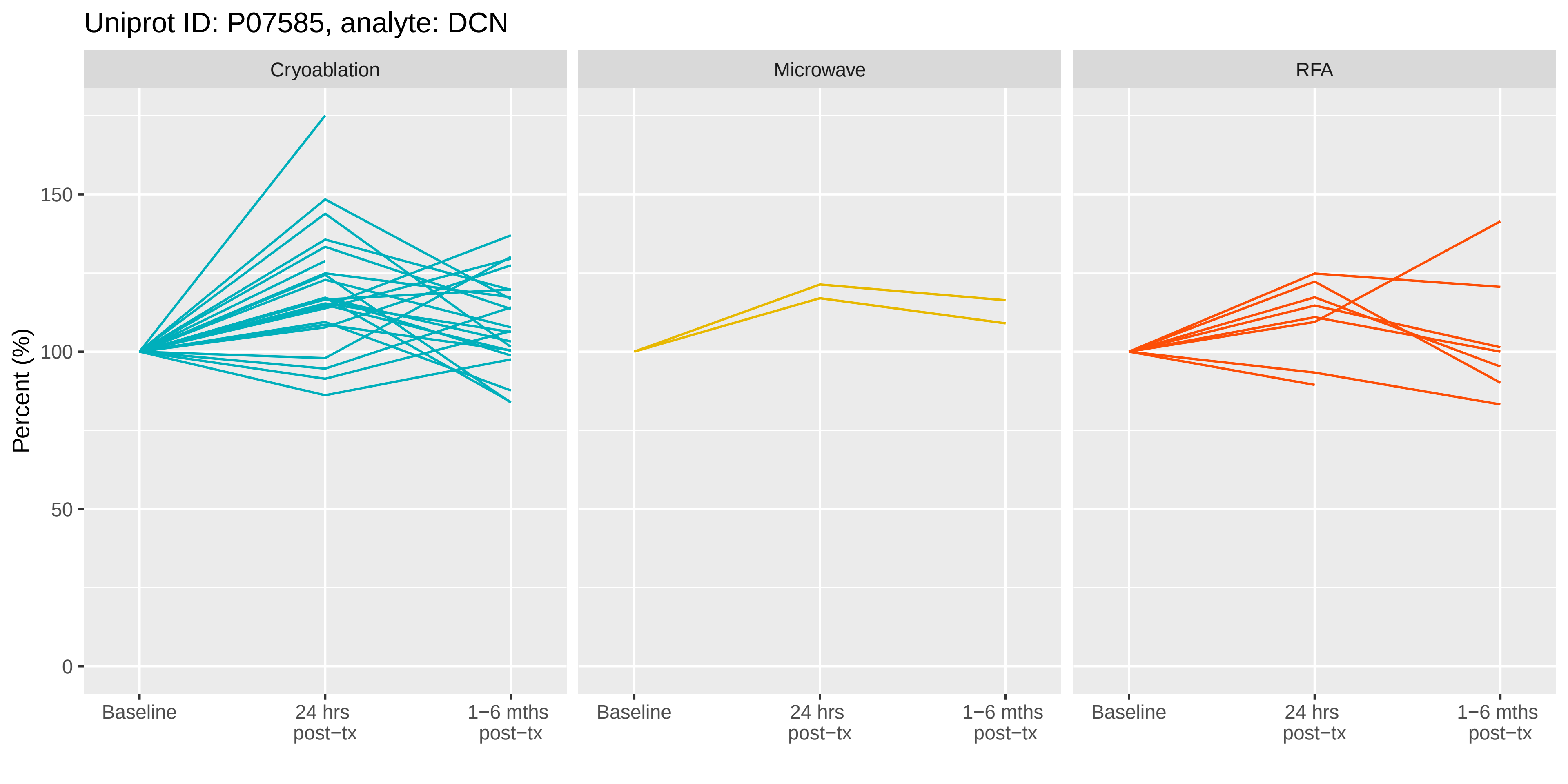

Uniprot ID: P07585, analyte: DCN
Cryoablation
Microwave
RFA
Percent (%)
150
100
50
0
Baseline
24 hrs post−tx
1−6 mths post−tx

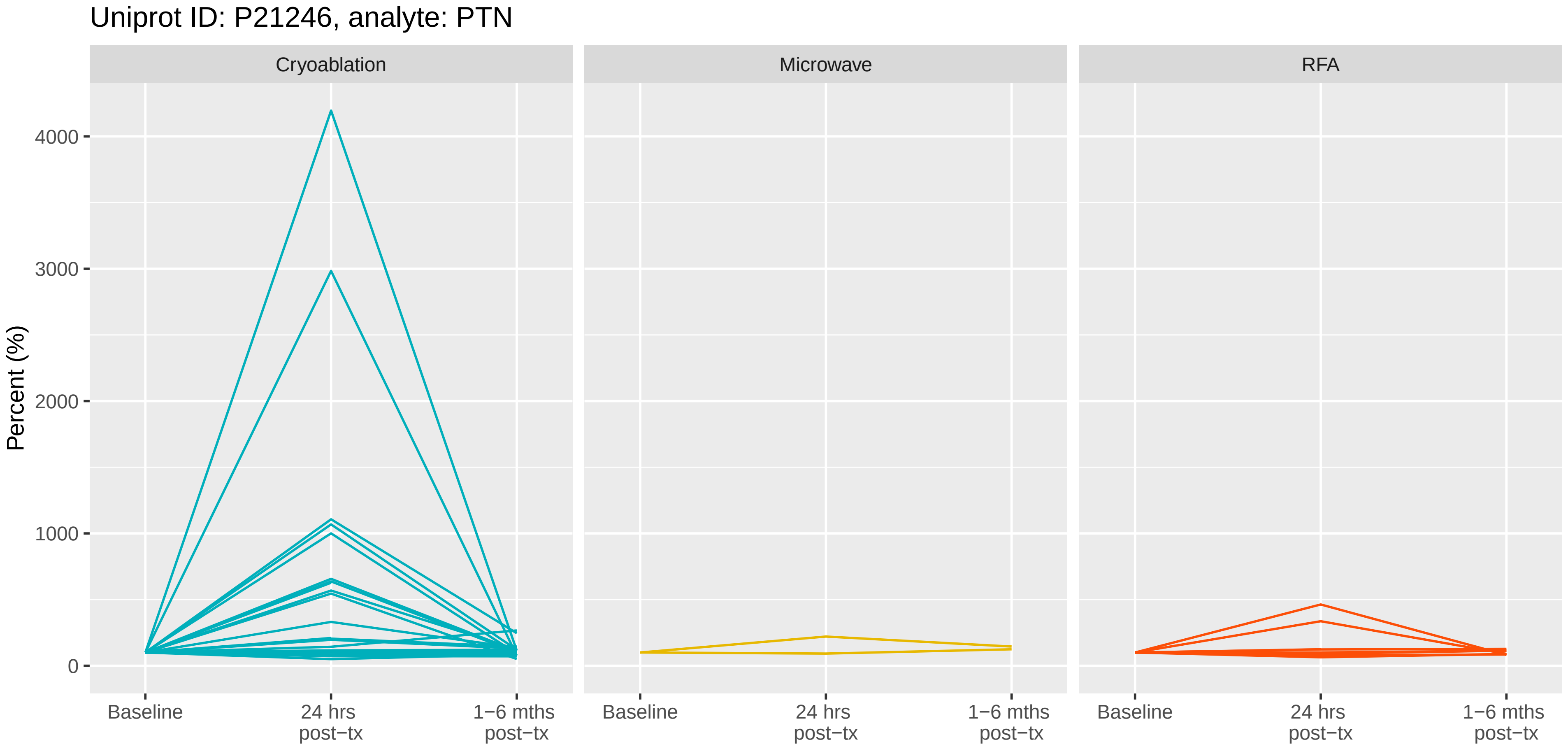
Uniprot ID: P21246, analyte: PTN
Cryoablation
Microwave
RFA
Percent (%)
4000
3000
2000
1000
0
Baseline
24 hrs post−tx
1−6 mths post−tx

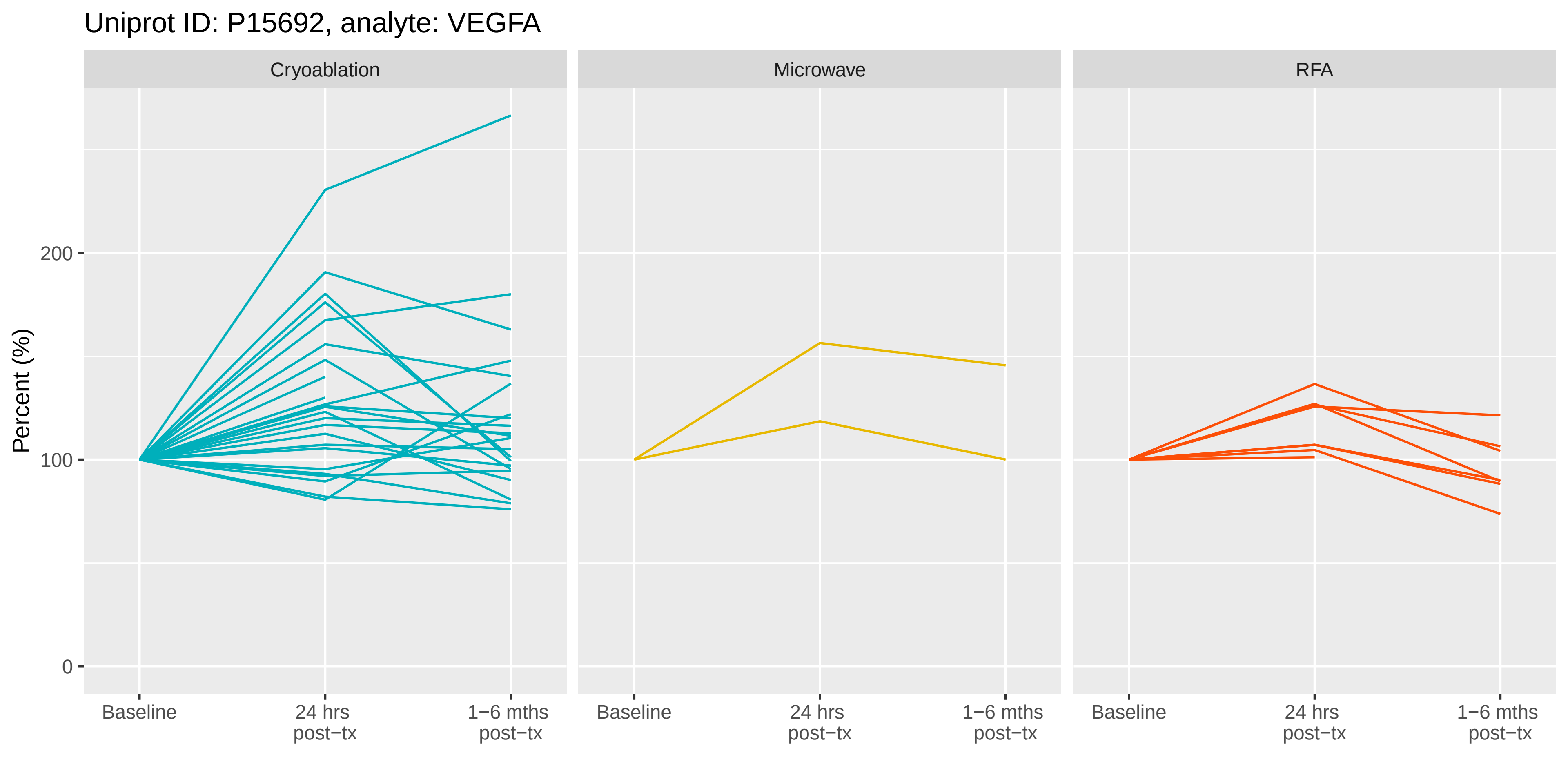

Uniprot ID: P15692, analyte: VEGFA
Cryoablation
Microwave
RFA
Percent (%)
200
100
0
Baseline
24 hrs post−tx
1−6 mths post−tx

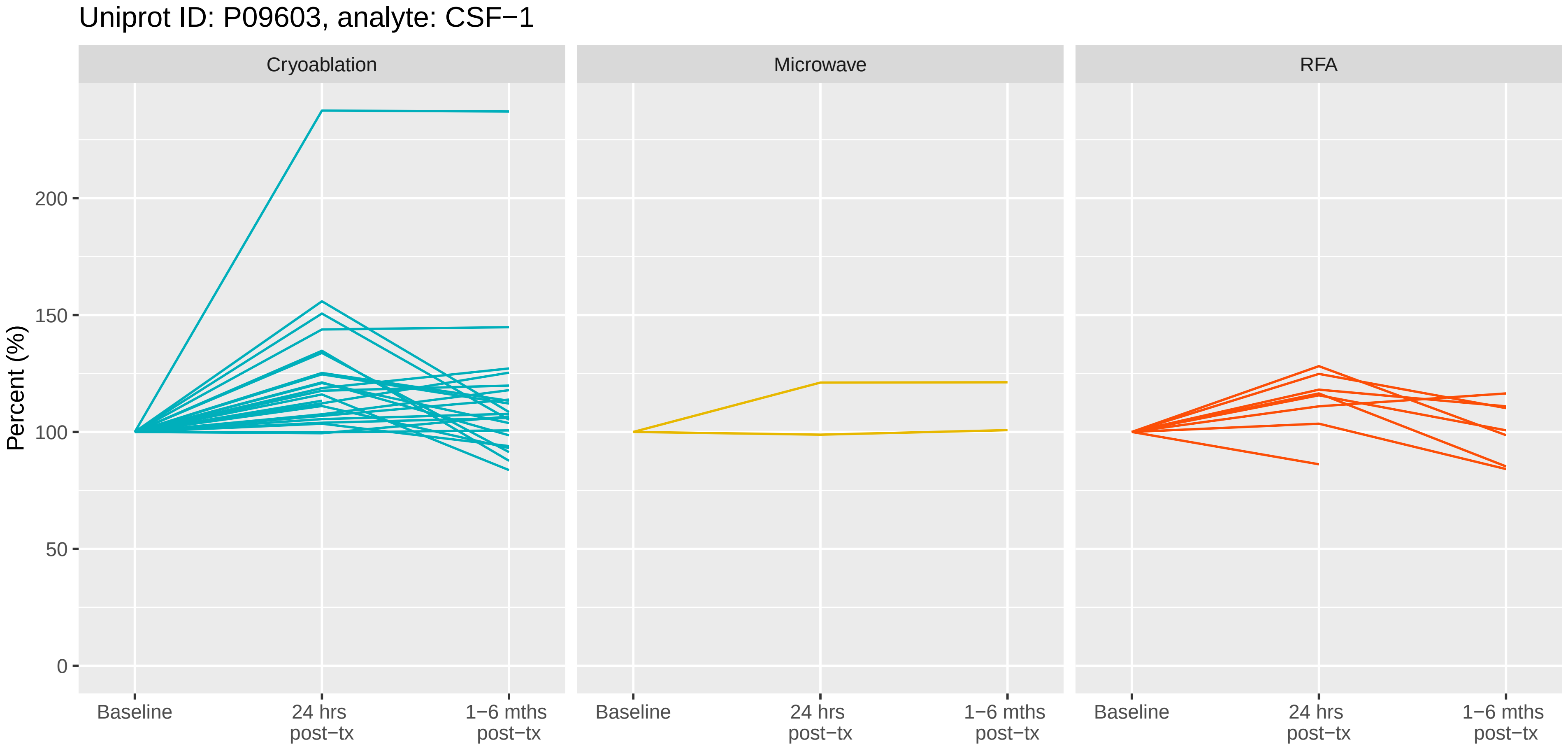
Uniprot ID: P09603, analyte: CSF−1
Cryoablation
Microwave
RFA
Percent (%)
200
150
100
50
0
Baseline
24 hrs post−tx
1−6 mths post−tx

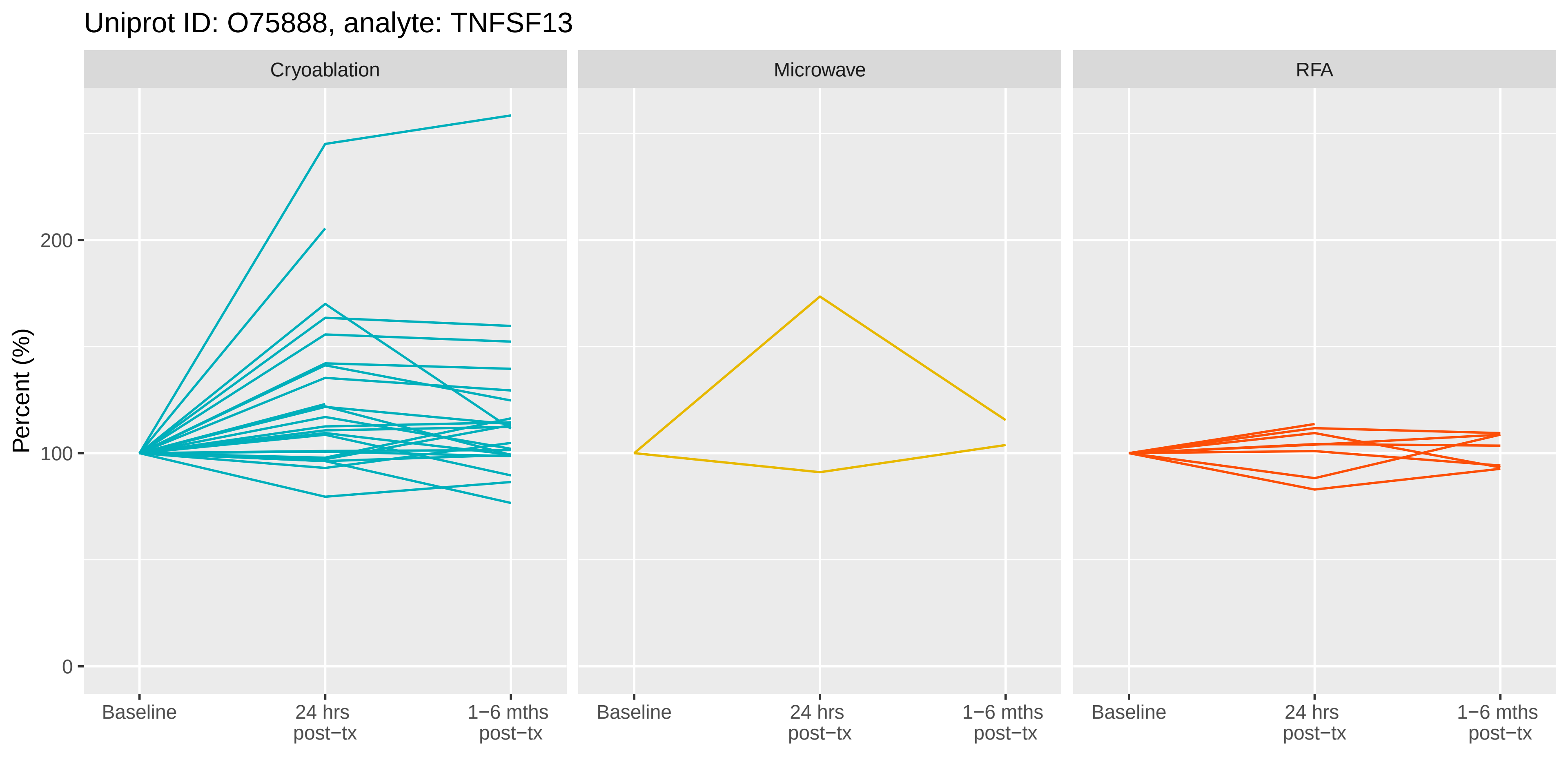
Uniprot ID: O75888, analyte: TNFSF13
Cryoablation
Microwave
RFA
Percent (%)
200
100
0
Baseline
24 hrs post−tx
1−6 mths post−tx
Baseline
24 hrs post−tx
1−6 mths post−tx
Baseline
24 hrs post−tx
1−6 mths post−tx

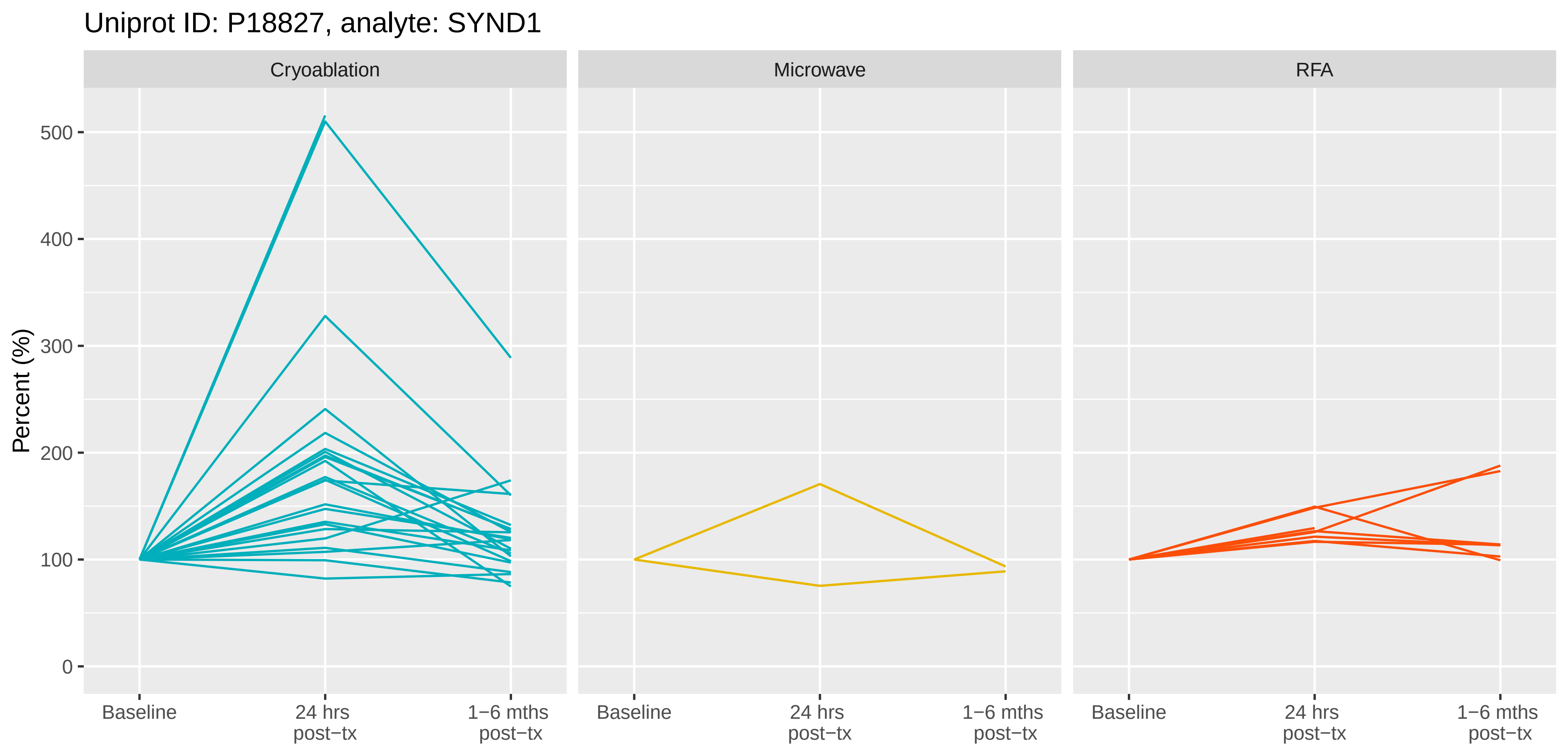

Uniprot ID: P18827, analyte: SYND1
Cryoablation
Microwave
RFA
Percent (%)
0
100
200
300
400
500
Baseline
24 hrs post−tx
1−6 mths post−tx

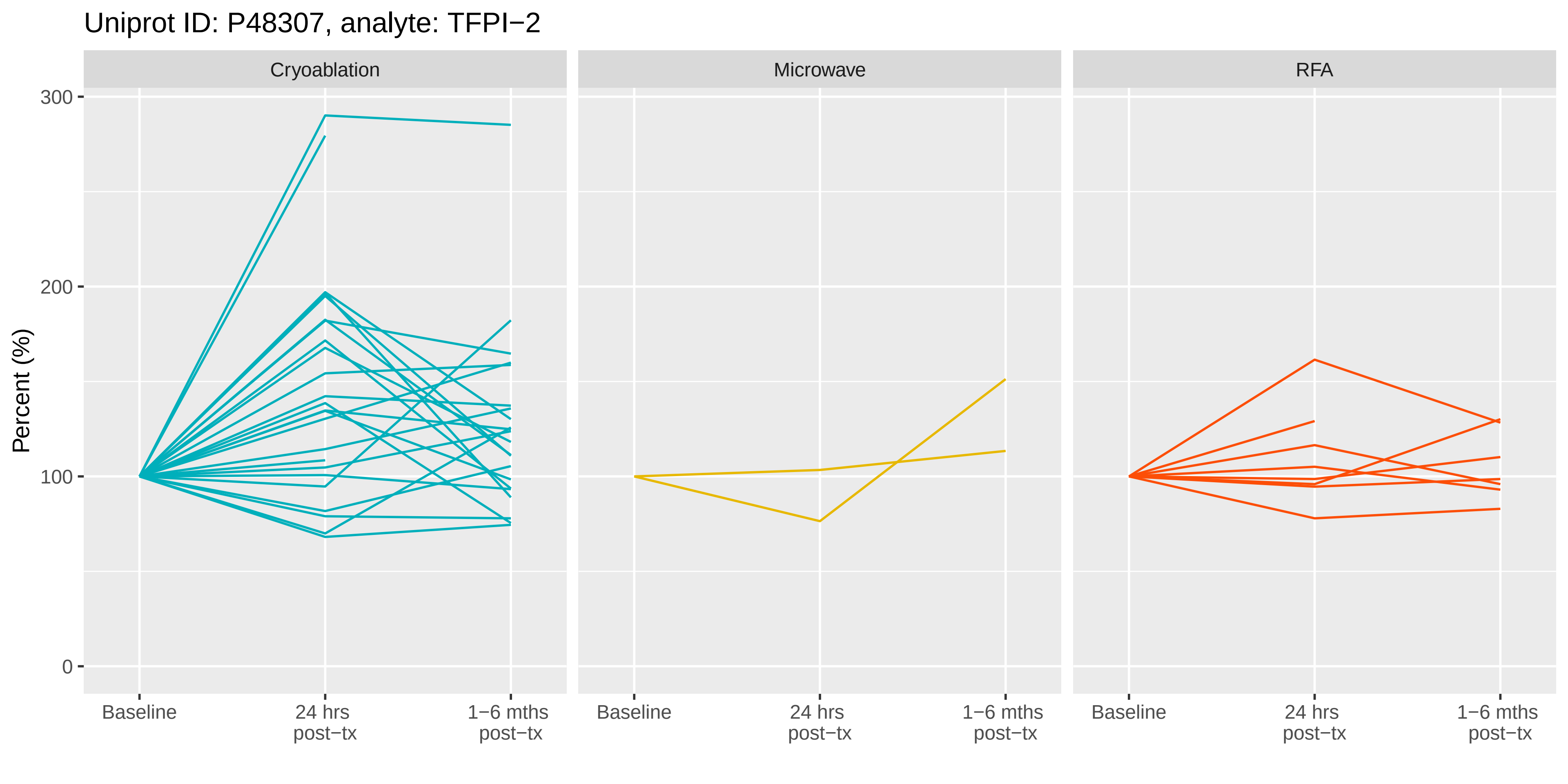

Uniprot ID: P48307, analyte: TFPI−2
Cryoablation
Microwave
RFA
Percent (%)
300
200
100
0
Baseline
24 hrs post−tx
1−6 mths post−tx

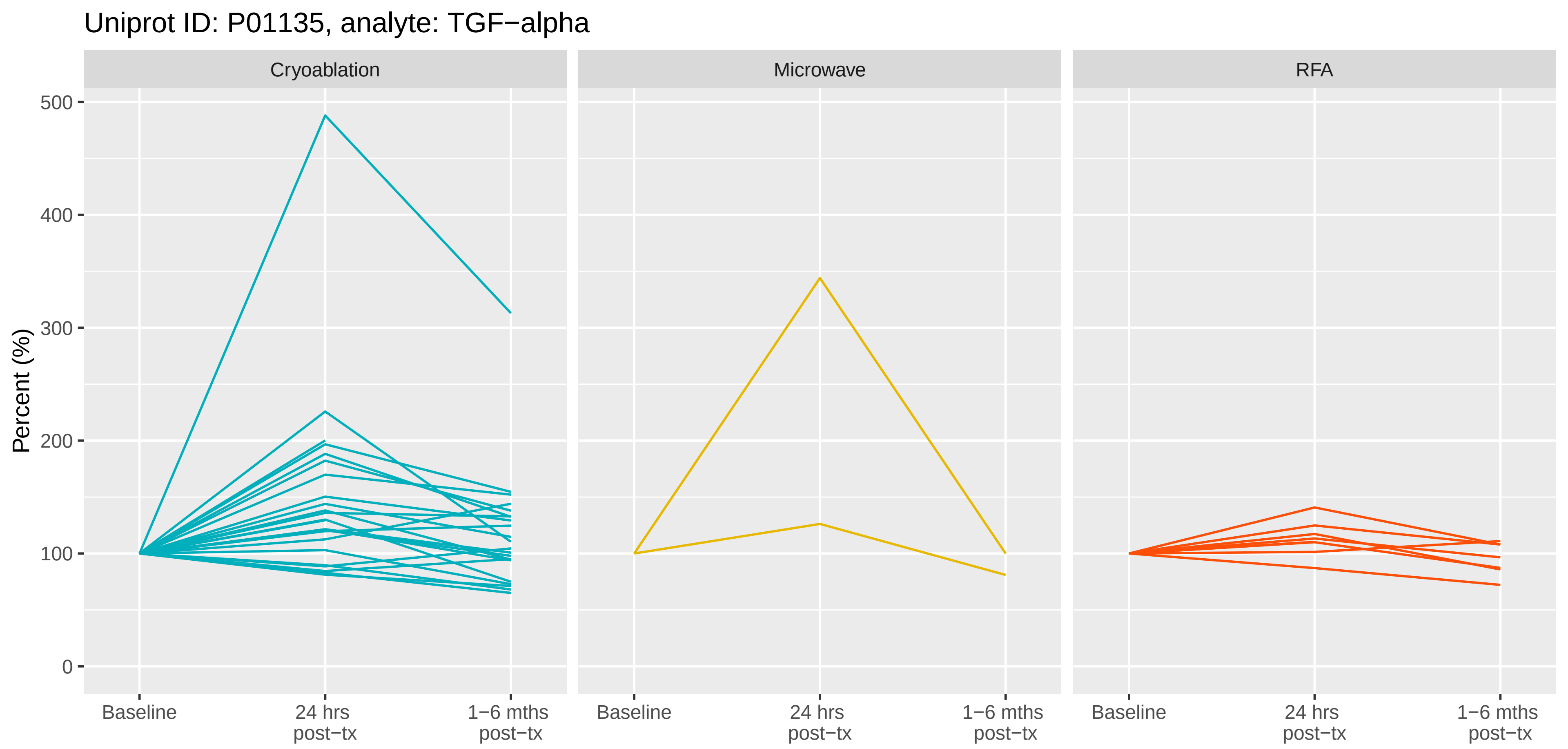

Uniprot ID: P01135, analyte: TGF−alpha
Cryoablation
Microwave
RFA
Percent (%)
500
400
300
200
100
0
Baseline
24 hrs post−tx
1−6 mths post−tx

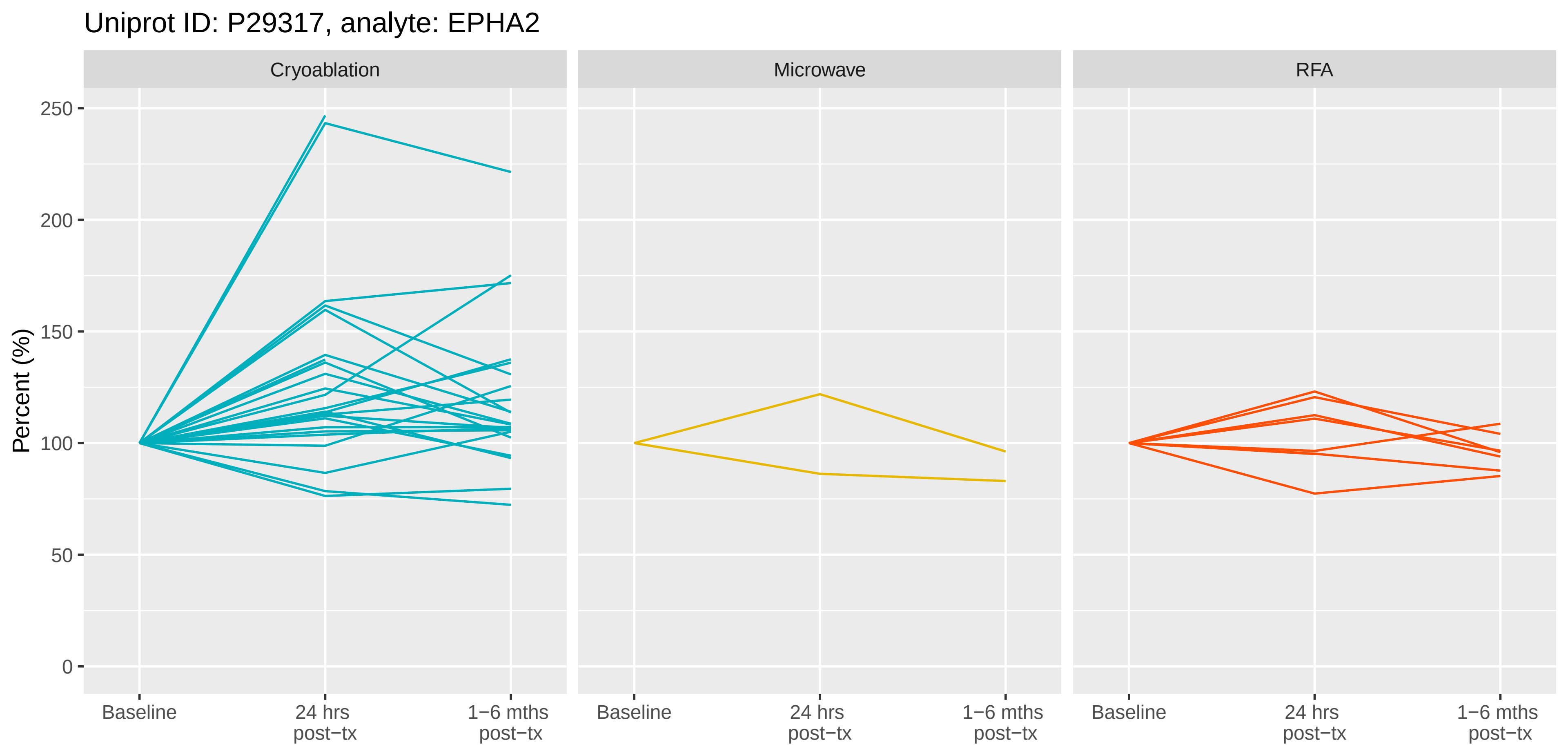

Uniprot ID: P29317, analyte: EPHA2
Cryoablation
Microwave
RFA
Percent (%)
250
200
150
100
50
0
Baseline
24 hrs post−tx
1−6 mths post−tx

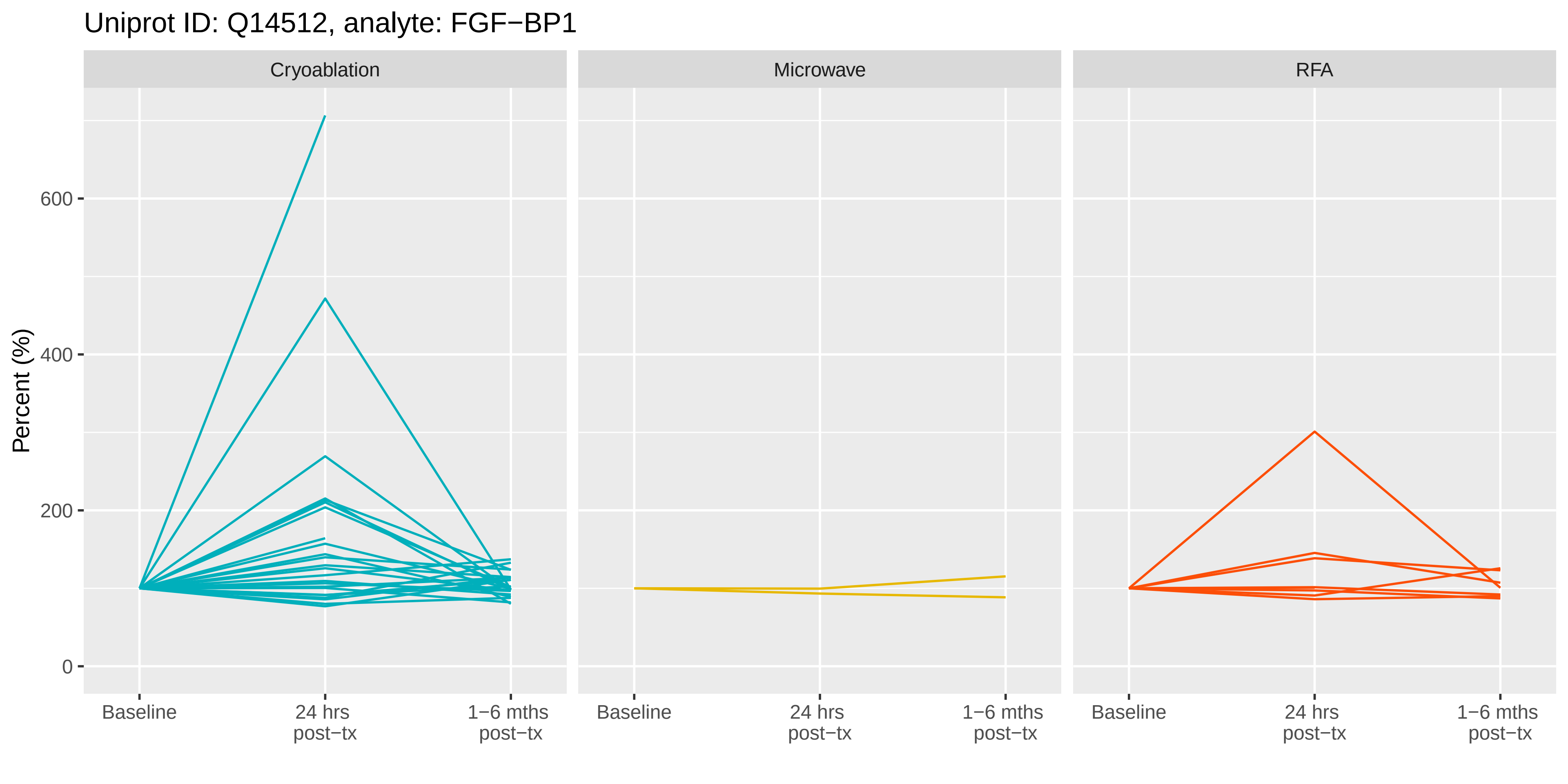
Uniprot ID: Q14512, analyte: FGF−BP1
Cryoablation
Microwave
RFA
Percent (%)
600
400
200
0
Baseline
24 hrs post−tx
1−6 mths post−tx

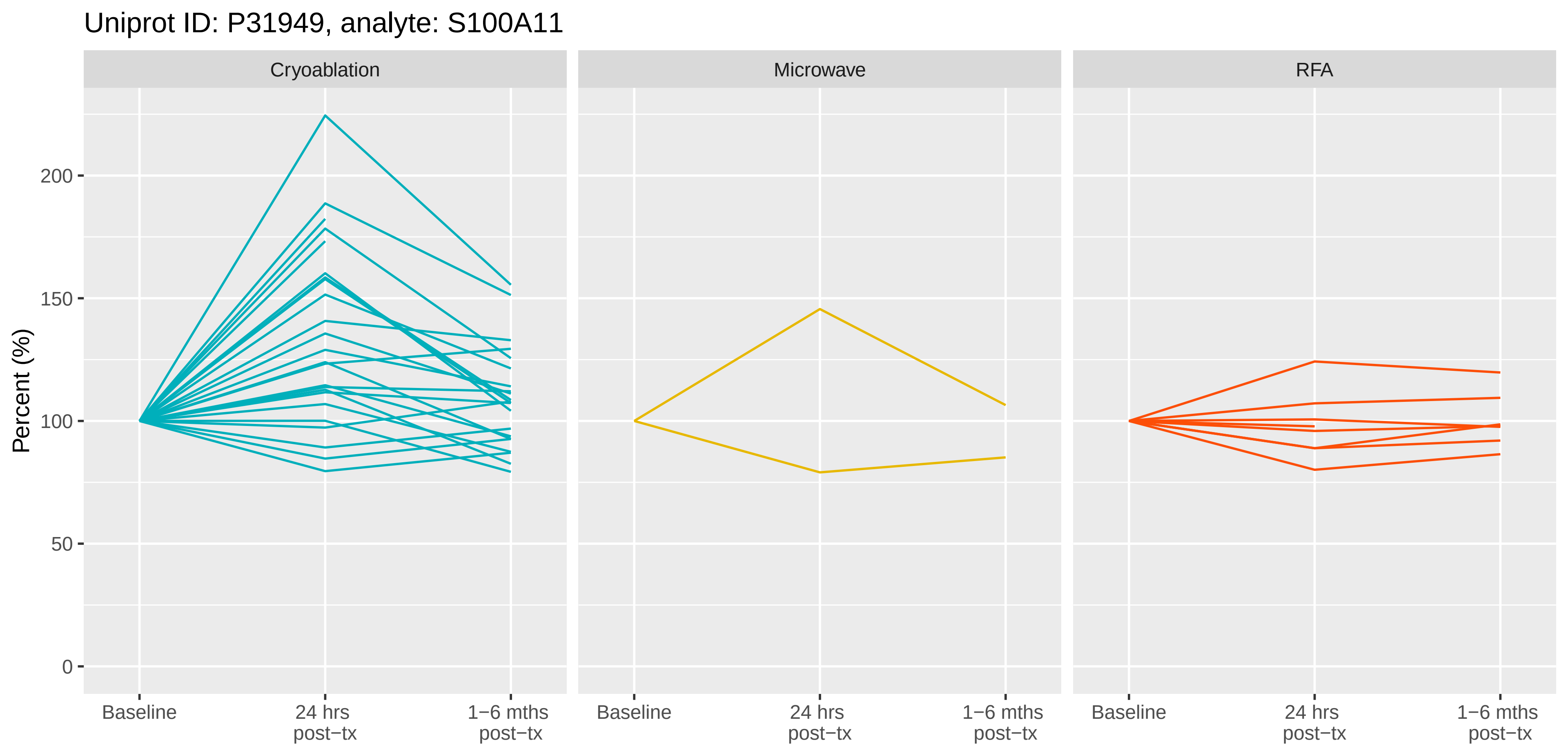

Uniprot ID: P31949, analyte: S100A11
Cryoablation
Microwave
RFA
Percent (%)
200
150
100
50
0
Baseline
24 hrs post−tx
1−6 mths post−tx

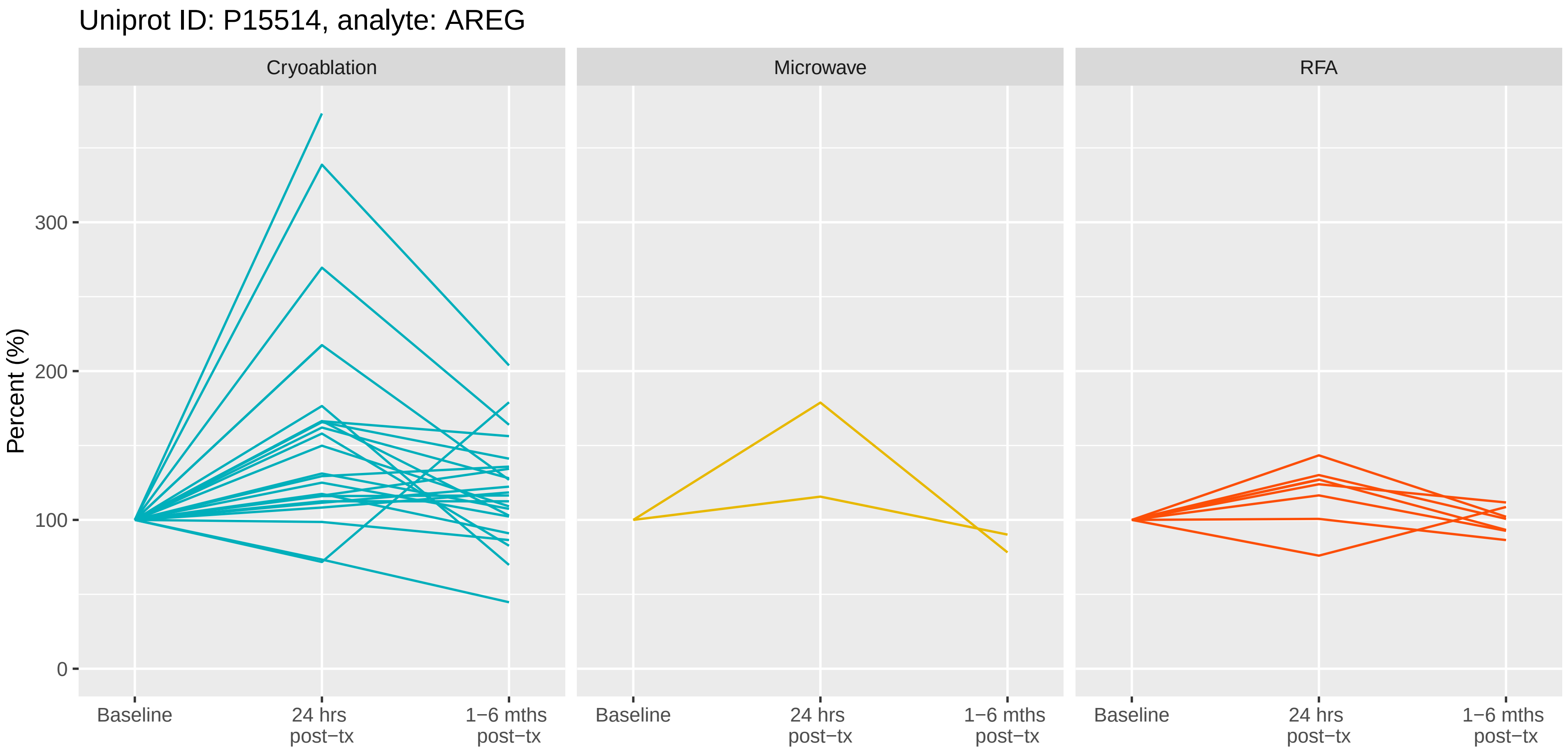

Uniprot ID: P15514, analyte: AREG
Cryoablation
Microwave
RFA
Percent (%)
300
200
100
0
Baseline
24 hrs post−tx
1−6 mths post−tx
Baseline
24 hrs post−tx
1−6 mths post−tx
Baseline
24 hrs post−tx
1−6 mths post−tx

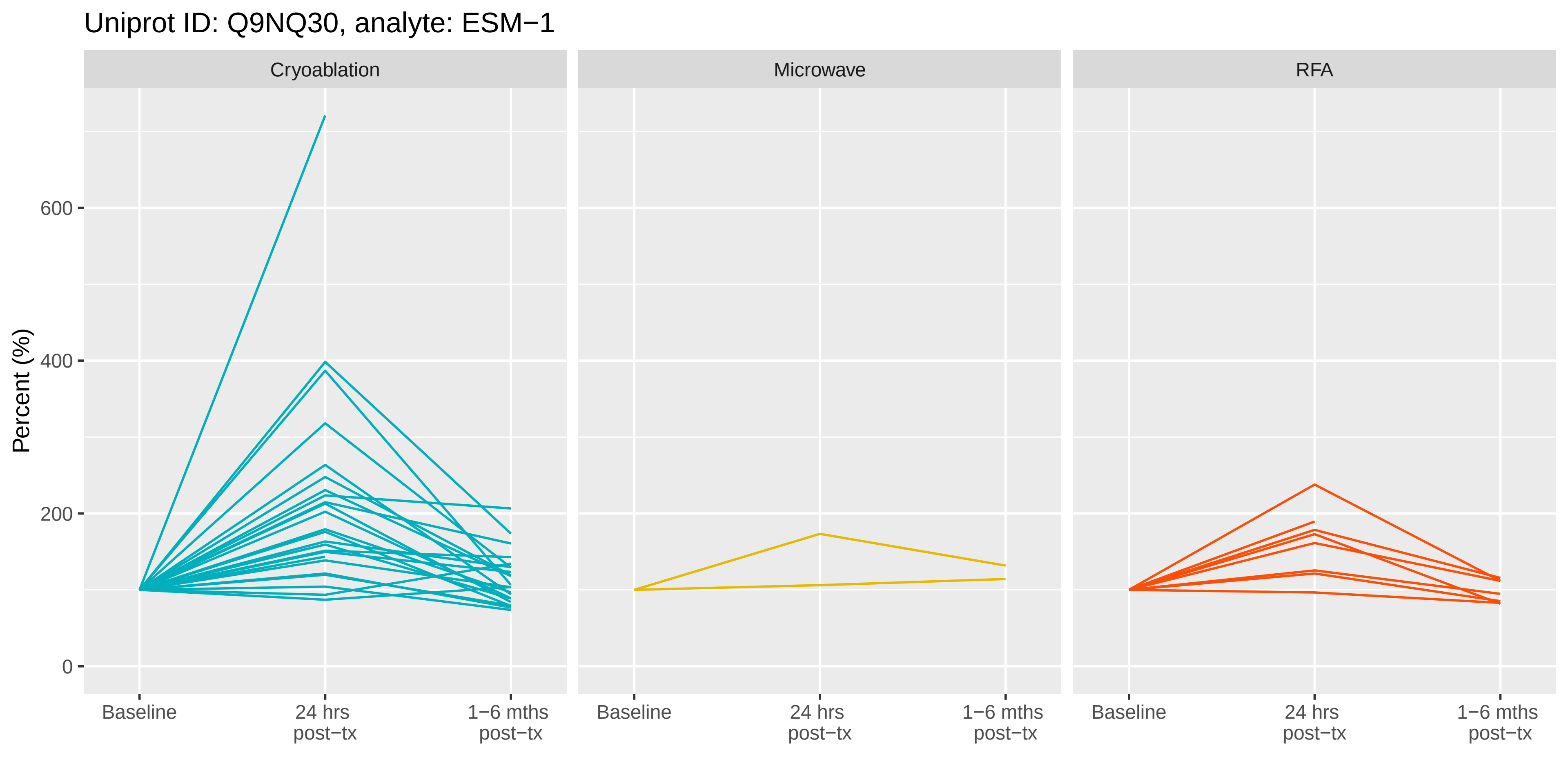
Uniprot ID: Q9NQ30, analyte: ESM−1
Cryoablation
Microwave
RFA
Percent (%)
0
200
400
600
Baseline
24 hrs post−tx
1−6 mths post−tx

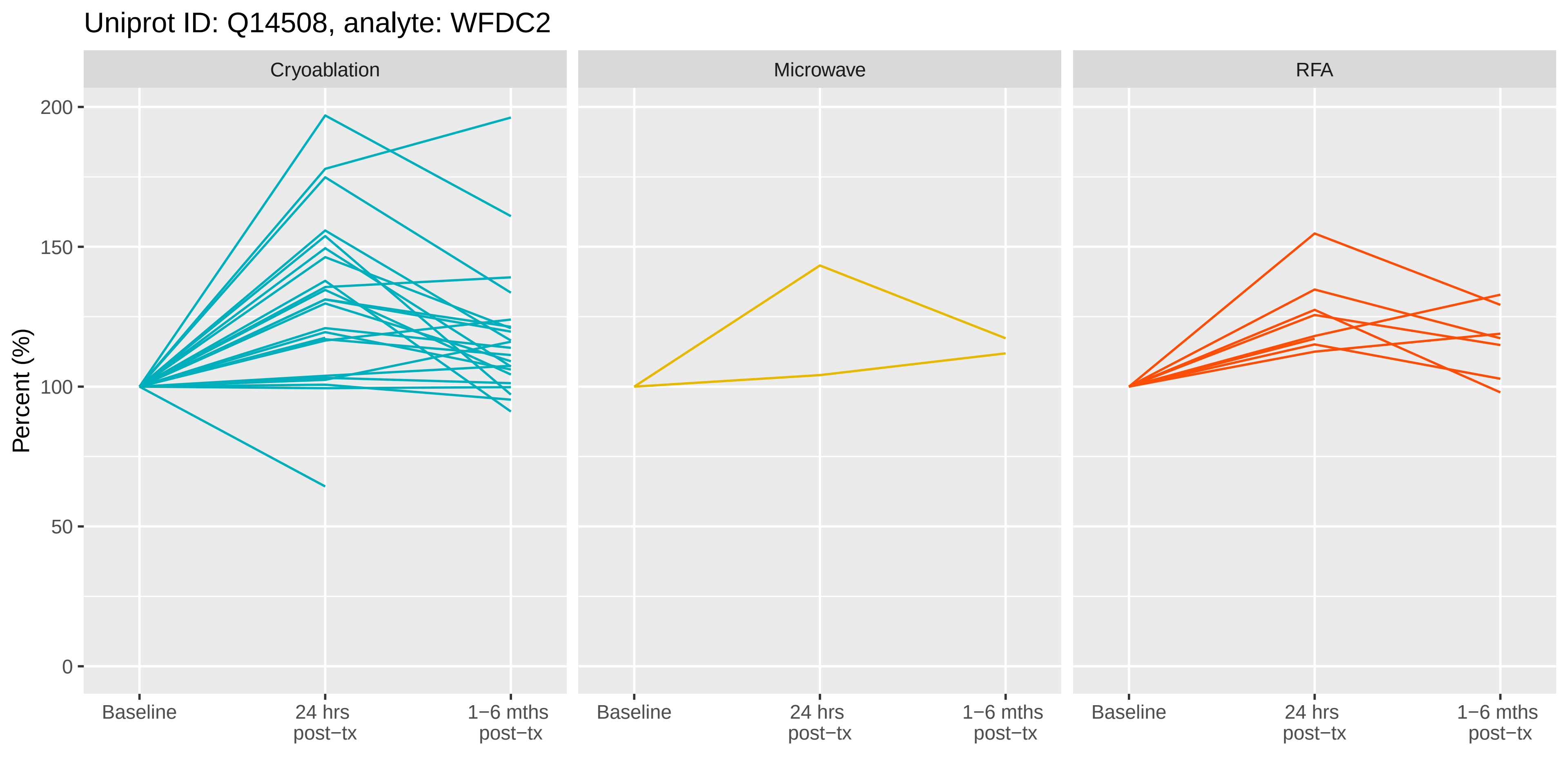

Uniprot ID: Q14508, analyte: WFDC2
Cryoablation
Microwave
RFA
Percent (%)
200
150
100
50
0
Baseline
24 hrs post−tx
1−6 mths post−tx

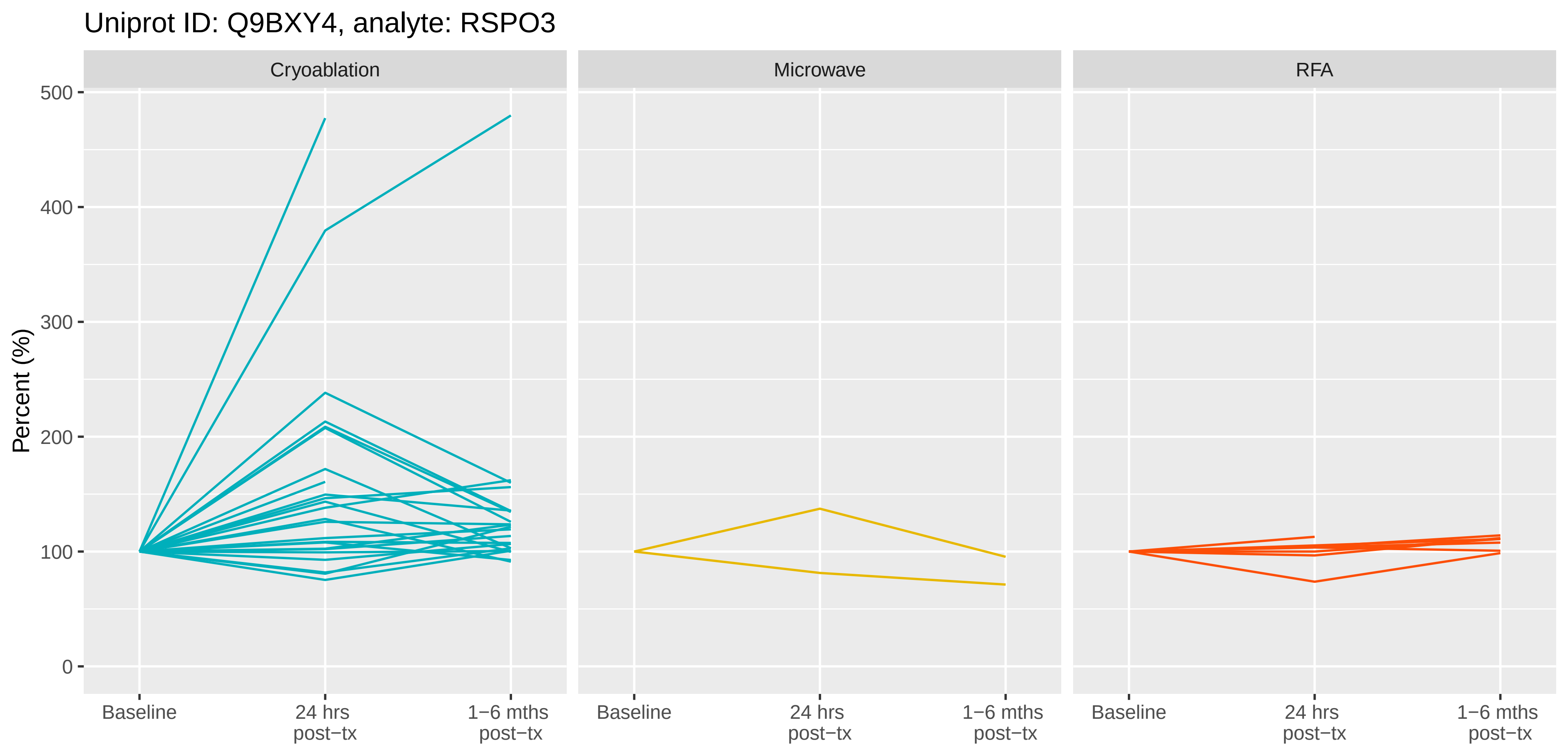
Uniprot ID: Q9BXY4, analyte: RSPO3
Cryoablation
Microwave
RFA
Percent (%)
500
400
300
200
100
0
Baseline
24 hrs post−tx
1−6 mths post−tx
