# Supplementary material for: An Exploratory Analysis of Changes in Circulating Plasma Protein Profiles Following Image-Guided Ablation of Renal Tumours Provides Evidence for Effects on Multiple Biological Processes
Source: Cancers (Basel). 2021 Nov 30;13(23):6037. doi: 10.3390/cancers13236037 (PMC8656737; doi:10.3390/cancers13236037)
Supplement: Supplementary file 1 [file cancers-13-06037-s001.zip › Supplementary File/Figure S6.pdf]

Figure S6. Longitudinal profiles of plasma proteins which decreased significantly ($p<0.05$ and $q<0.01$; TP3 vs TP2) in patients 24 hours post-CRYO ($n=24$ patients as one TP3 sample failed analytical QC). Results for each protein are expressed as a % of the reference baseline (TP2; 100%).

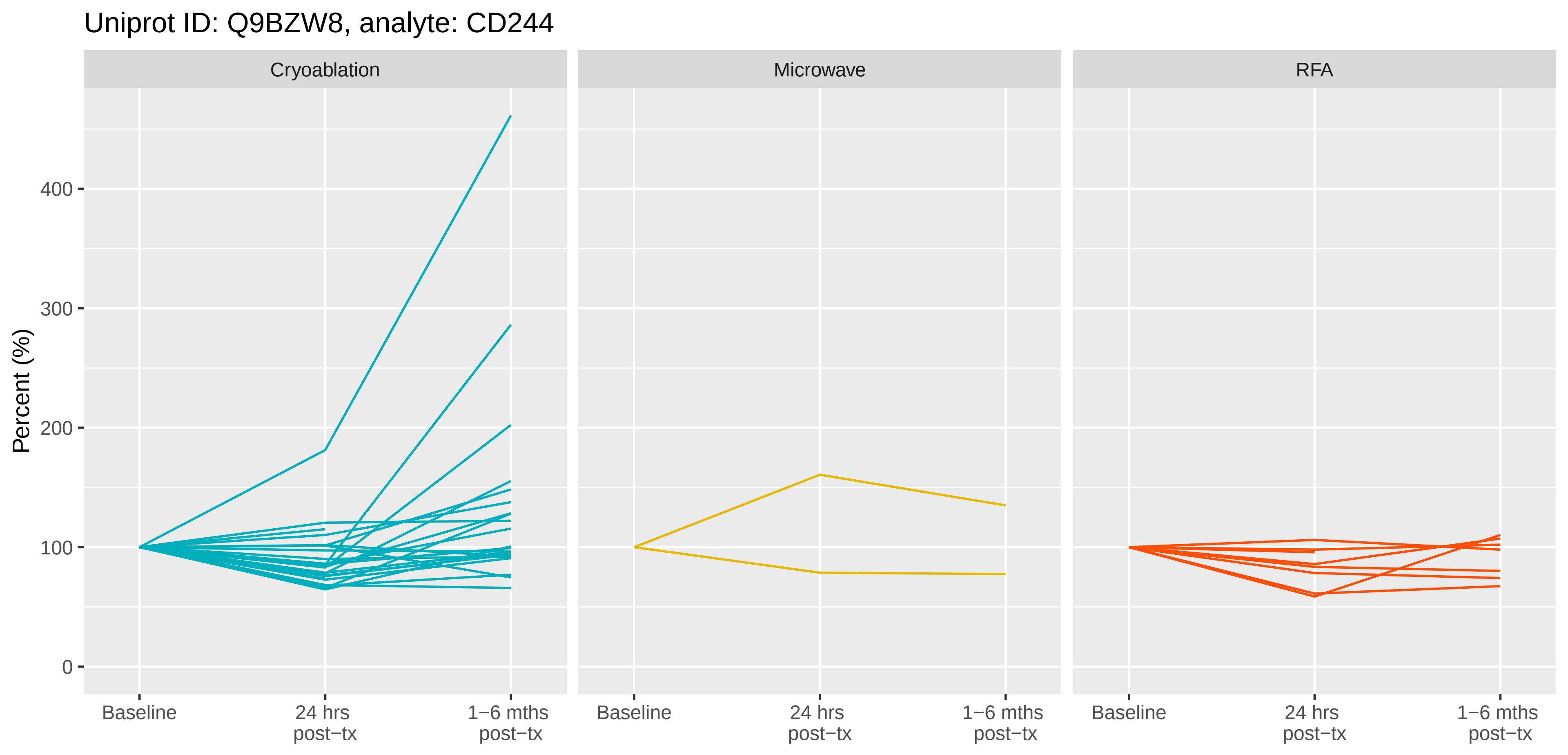

Uniprot ID: Q9BZW8, analyte: CD244
Cryoablation
Microwave
RFA
Percent (%)
0
100
200
300
400
Baseline
24 hrs post−tx
1−6 mths post−tx

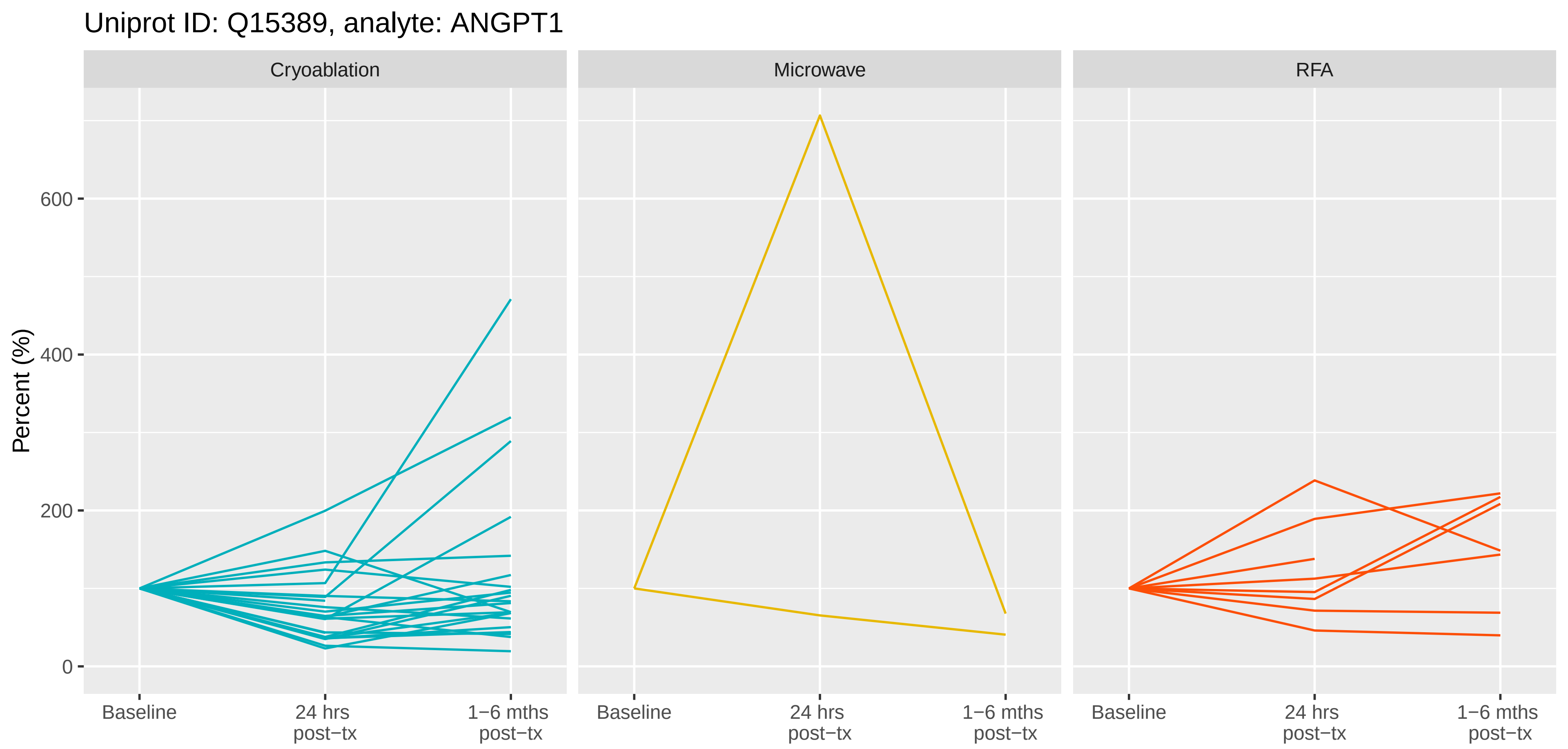

Uniprot ID: Q15389, analyte: ANGPT1
Cryoablation
Microwave
RFA
Percent (%)
600
400
200
0
Baseline
24 hrs post−tx
1−6 mths post−tx

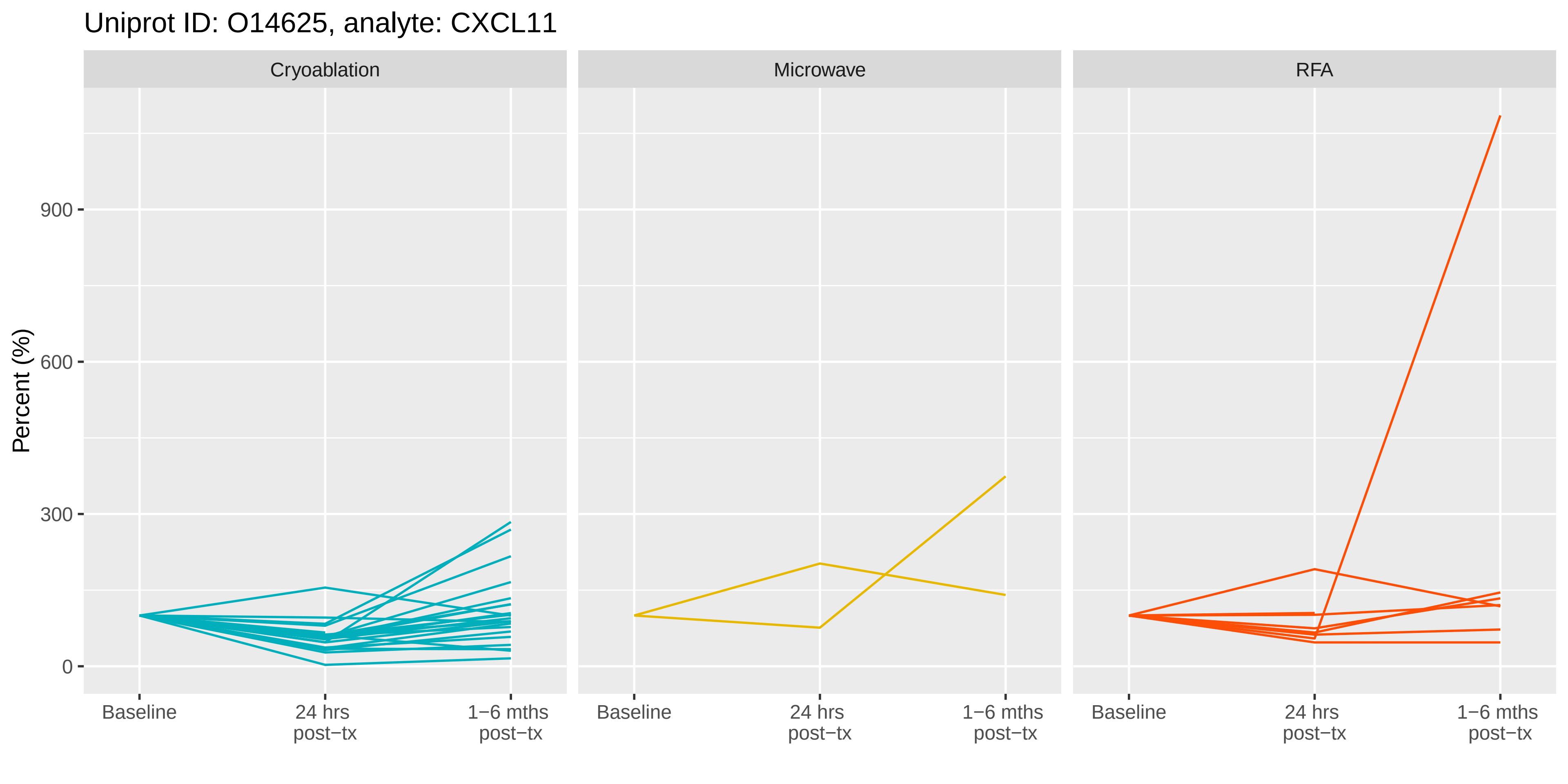

Uniprot ID: O14625, analyte: CXCL11
Cryoablation
Microwave
RFA
Percent (%)
900
600
300
0
Baseline
24 hrs post−tx
1−6 mths post−tx

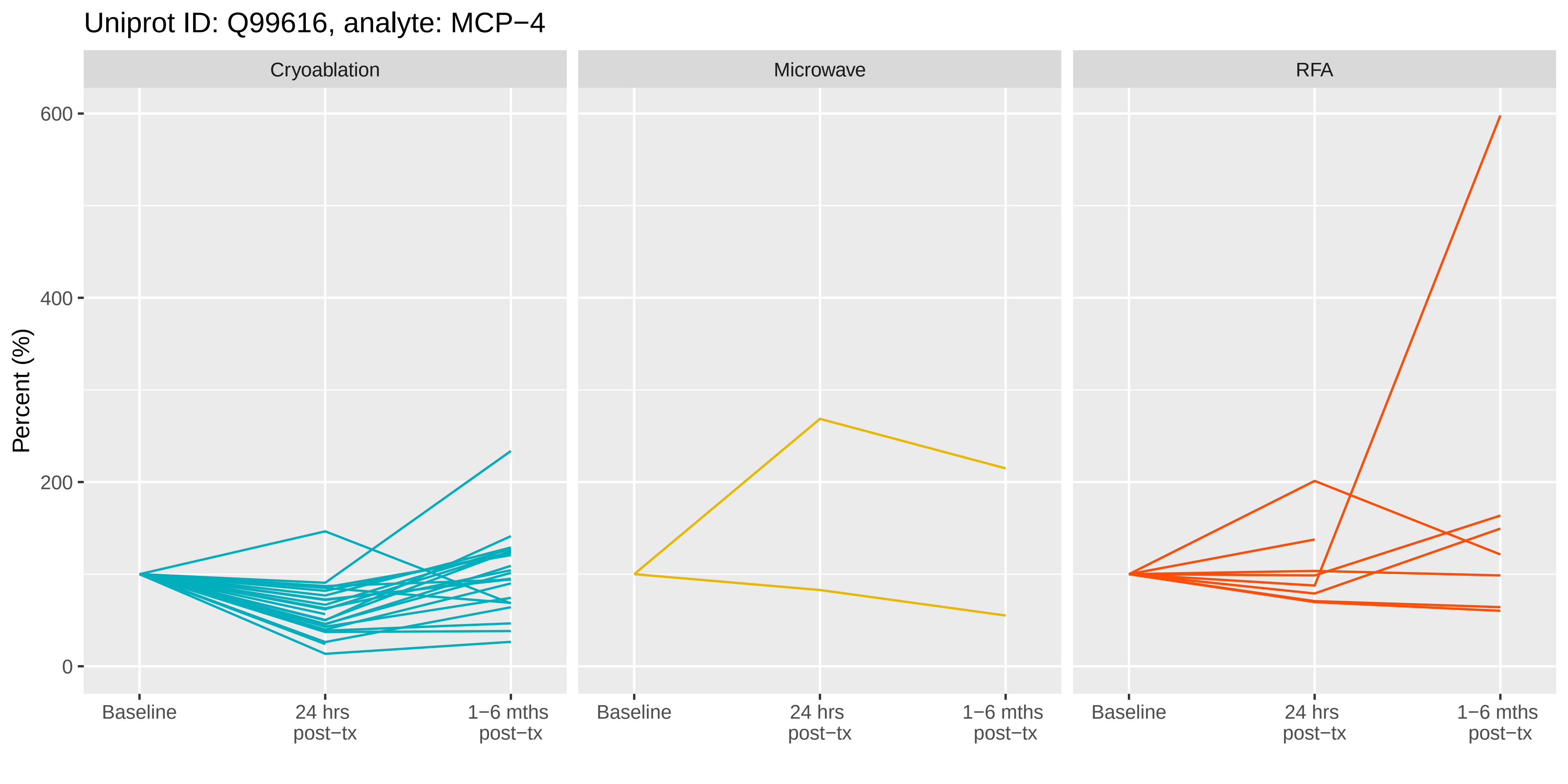

Uniprot ID: Q99616, analyte: MCP−4
Cryoablation
Microwave
RFA
Percent (%)
600
400
200
0
Baseline
24 hrs post−tx
1−6 mths post−tx

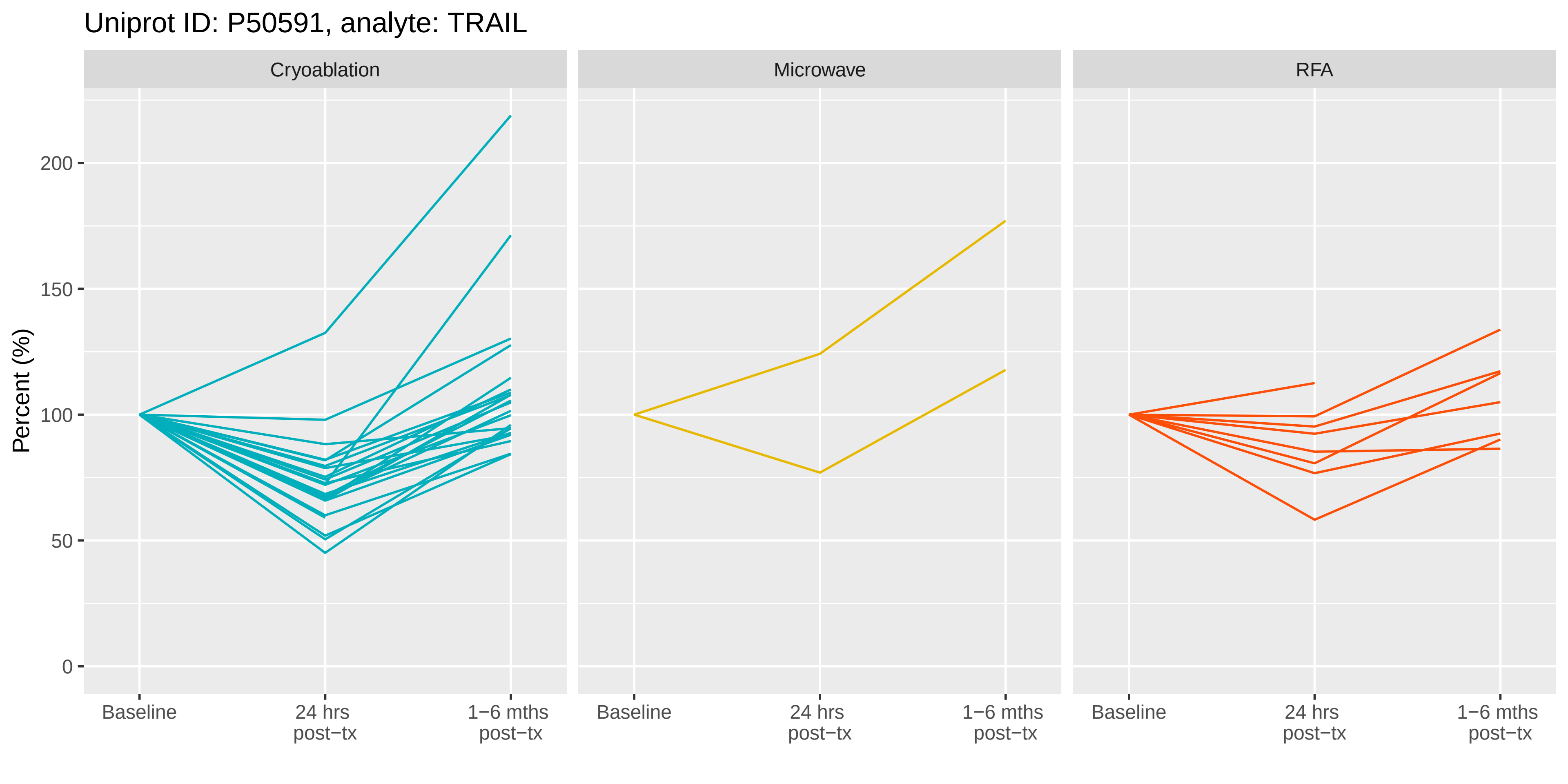

Uniprot ID: P50591, analyte: TRAIL
Cryoablation
Microwave
RFA
Percent (%)
0
50
100
150
200
Baseline
24 hrs post−tx
1−6 mths post−tx

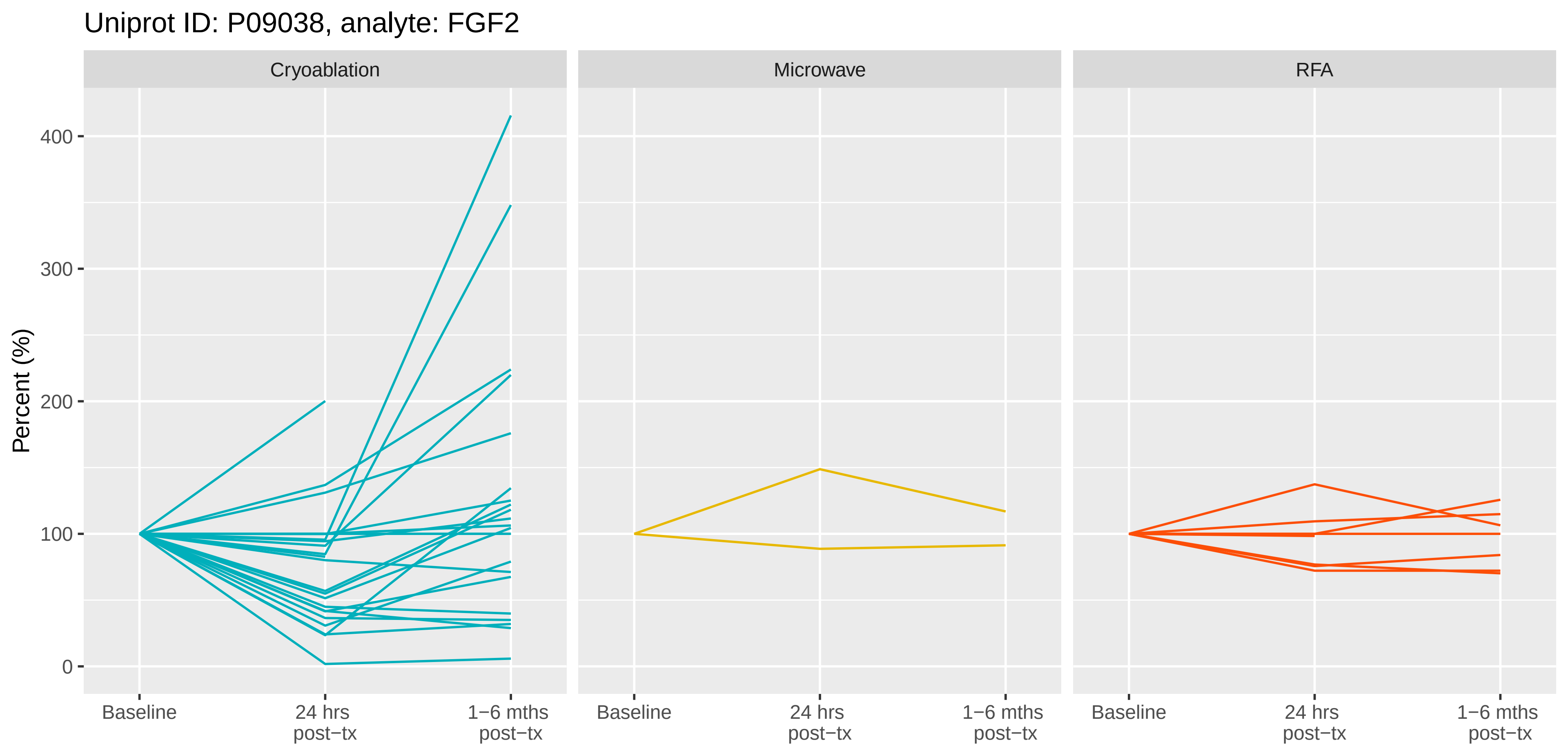

Uniprot ID: P09038, analyte: FGF2
Cryoablation
Microwave
RFA
Percent (%)
0
100
200
300
400
Baseline
24 hrs post−tx
1−6 mths post−tx

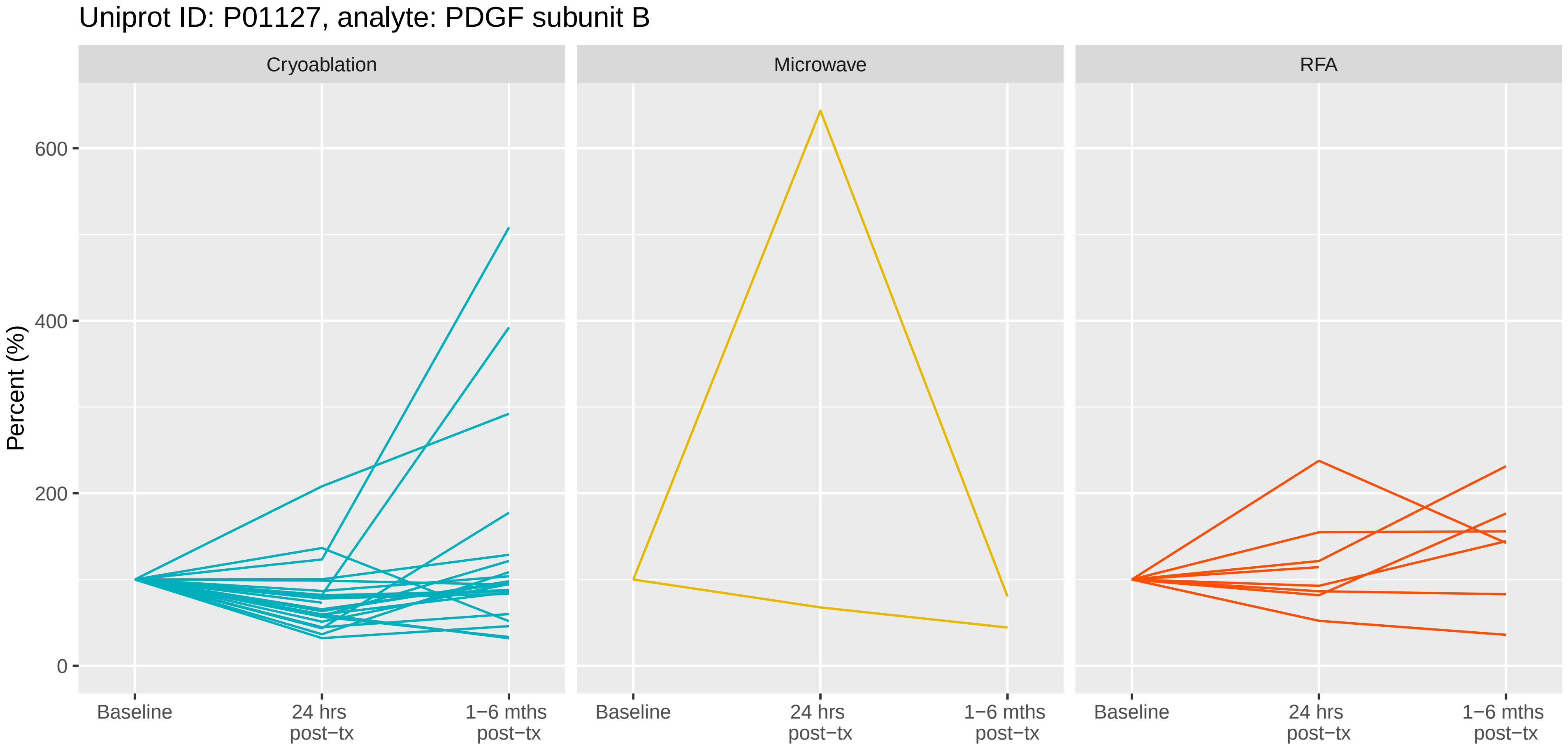

Uniprot ID: P01127, analyte: PDGF subunit B
Cryoablation
Microwave
RFA
Percent (%)
0
200
400
600
Baseline
24 hrs post−tx
1−6 mths post−tx

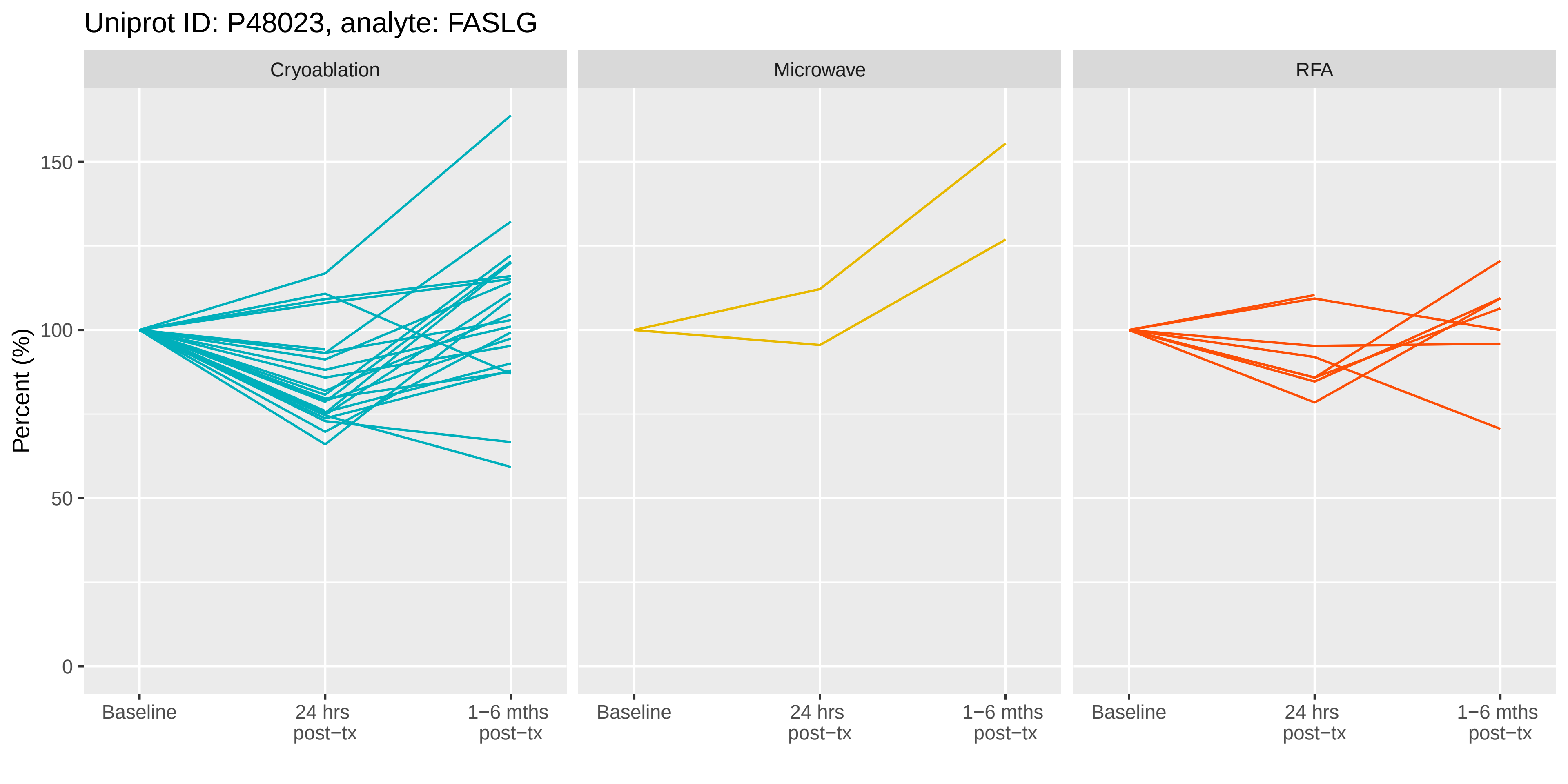

Uniprot ID: P48023, analyte: FASLG
Cryoablation
Microwave
RFA
Percent (%)
150
100
50
0
Baseline
24 hrs post−tx
1−6 mths post−tx

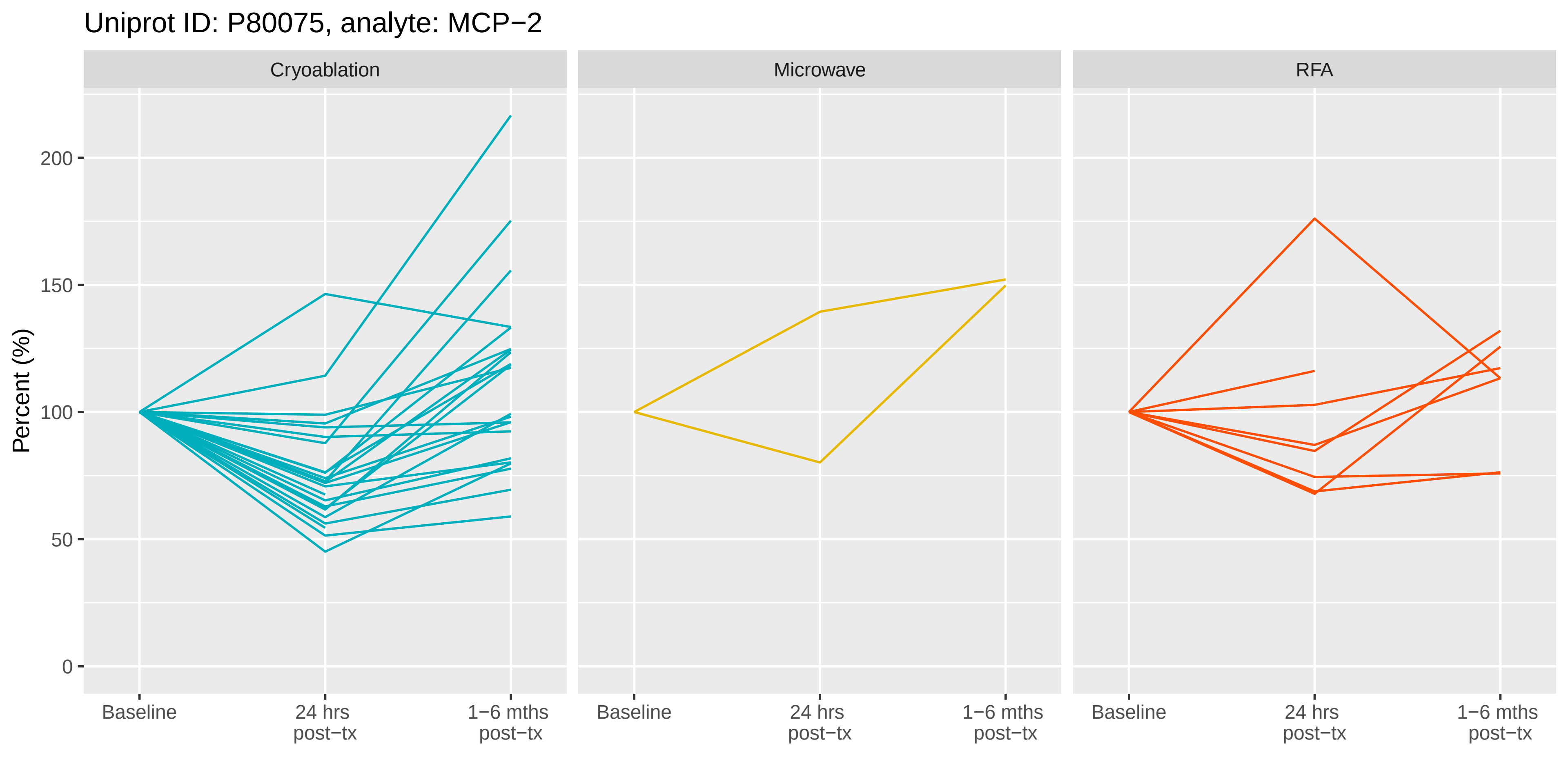

Uniprot ID: P80075, analyte: MCP−2
Cryoablation
Microwave
RFA
Percent (%)
200
150
100
50
0
Baseline
24 hrs post−tx
1−6 mths post−tx

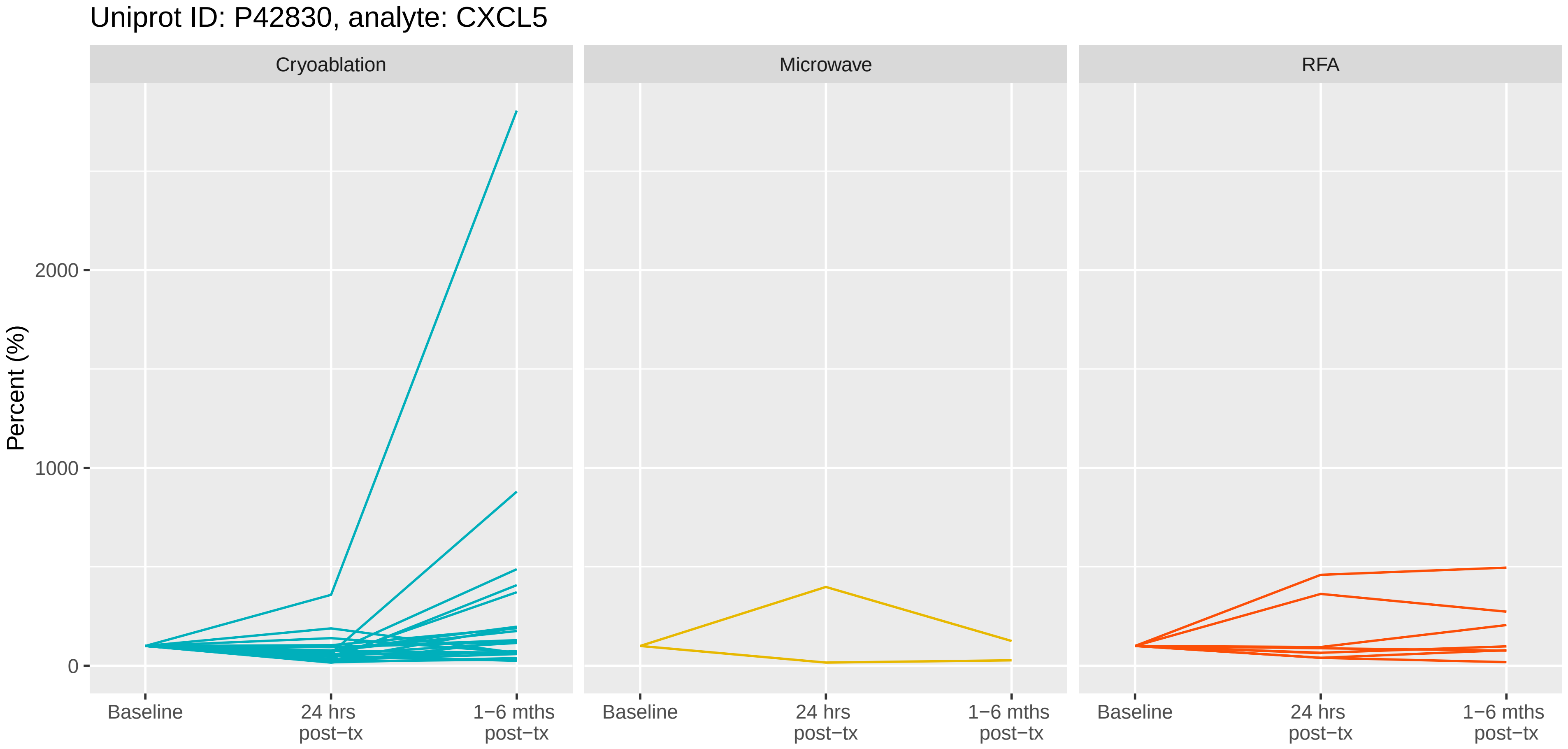

Uniprot ID: P42830, analyte: CXCL5
Cryoablation
Microwave
RFA
Percent (%)
2000
1000
0
Baseline
24 hrs post−tx
1−6 mths post−tx

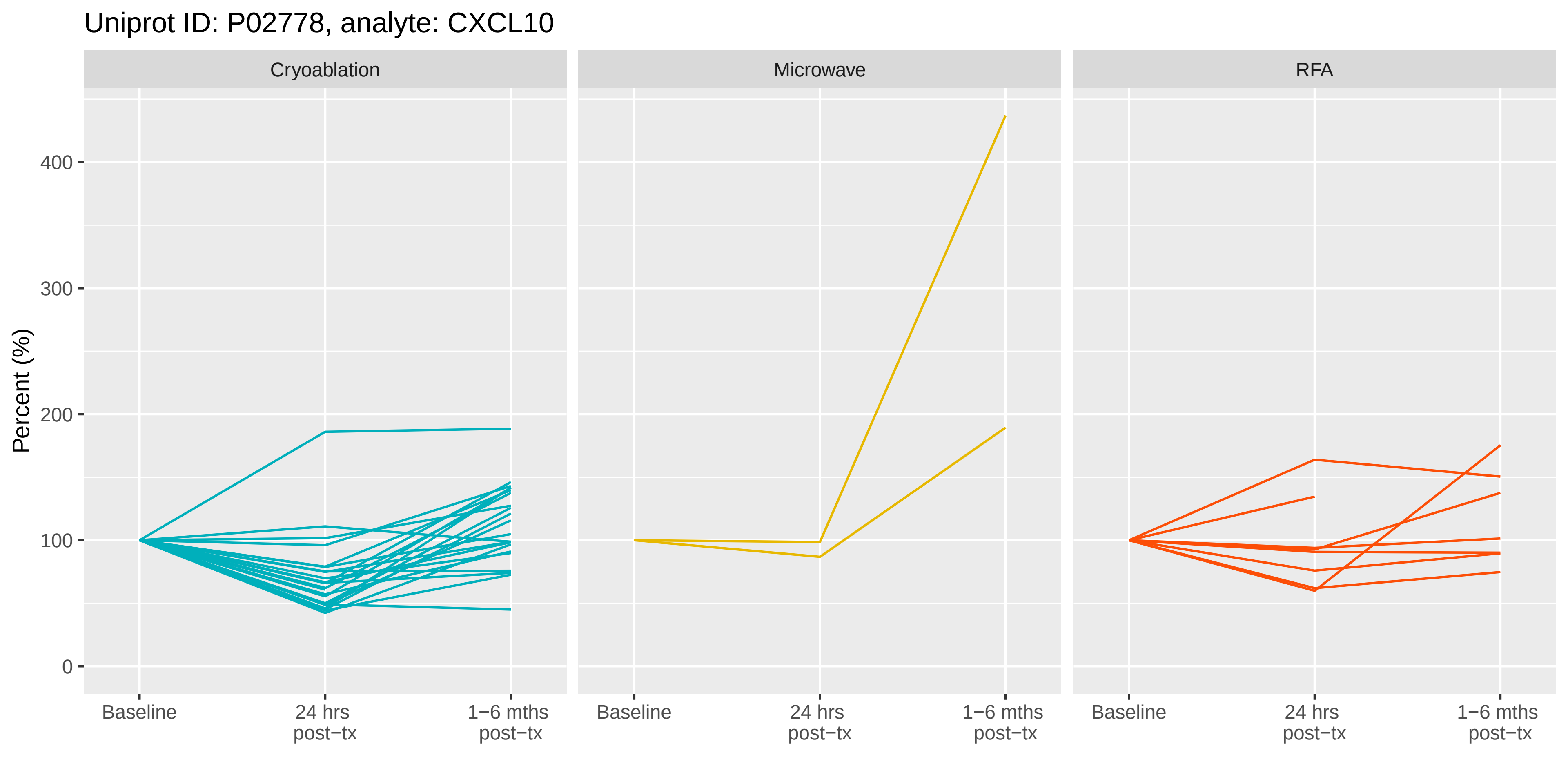

Uniprot ID: P02778, analyte: CXCL10
Cryoablation
Microwave
RFA
Percent (%)
400
300
200
100
0
Baseline
24 hrs post−tx
1−6 mths post−tx

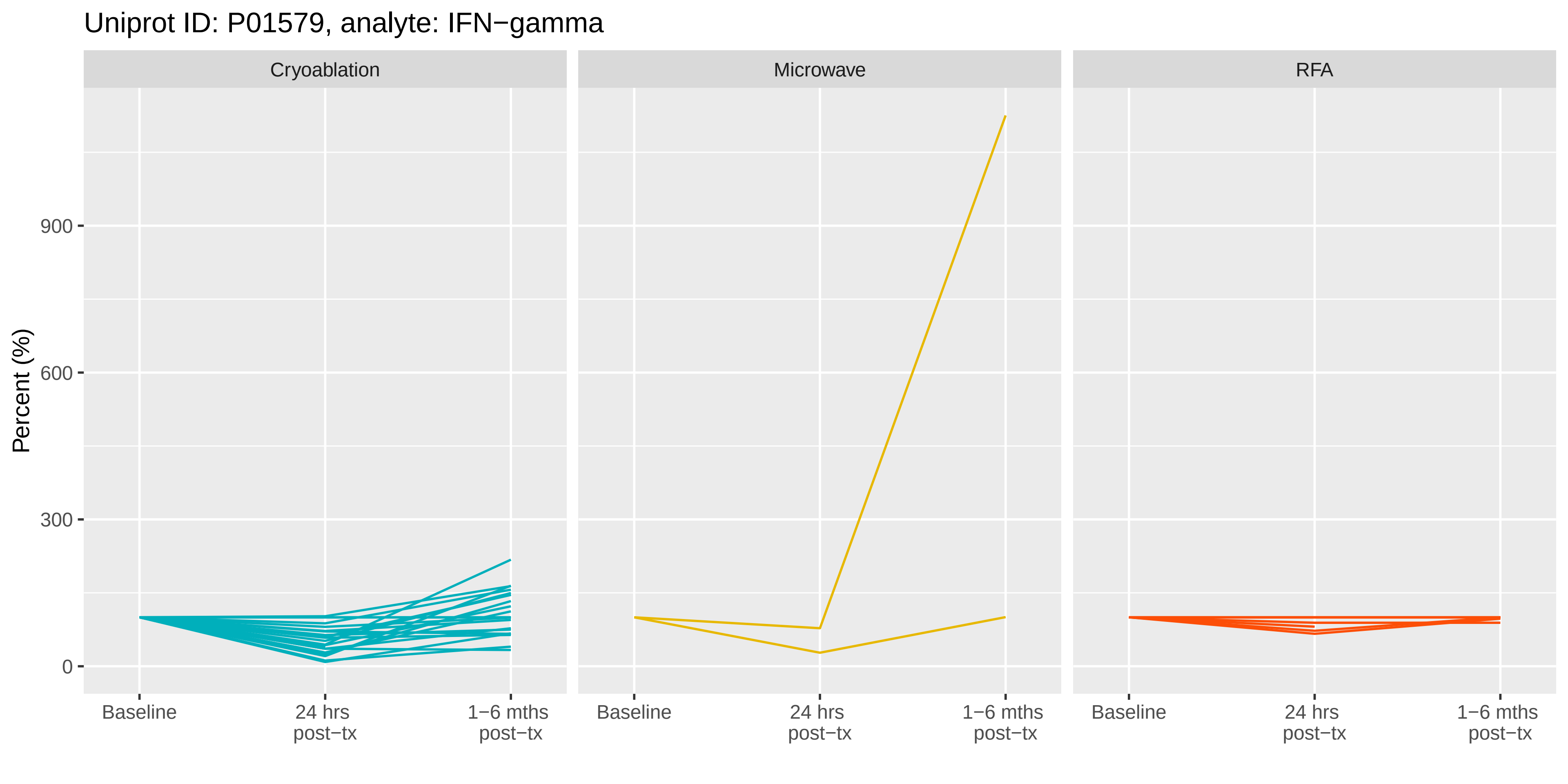

Uniprot ID: P01579, analyte: IFN−gamma
Cryoablation
Microwave
RFA
Percent (%)
900
600
300
0
Baseline
24 hrs post−tx
1−6 mths post−tx

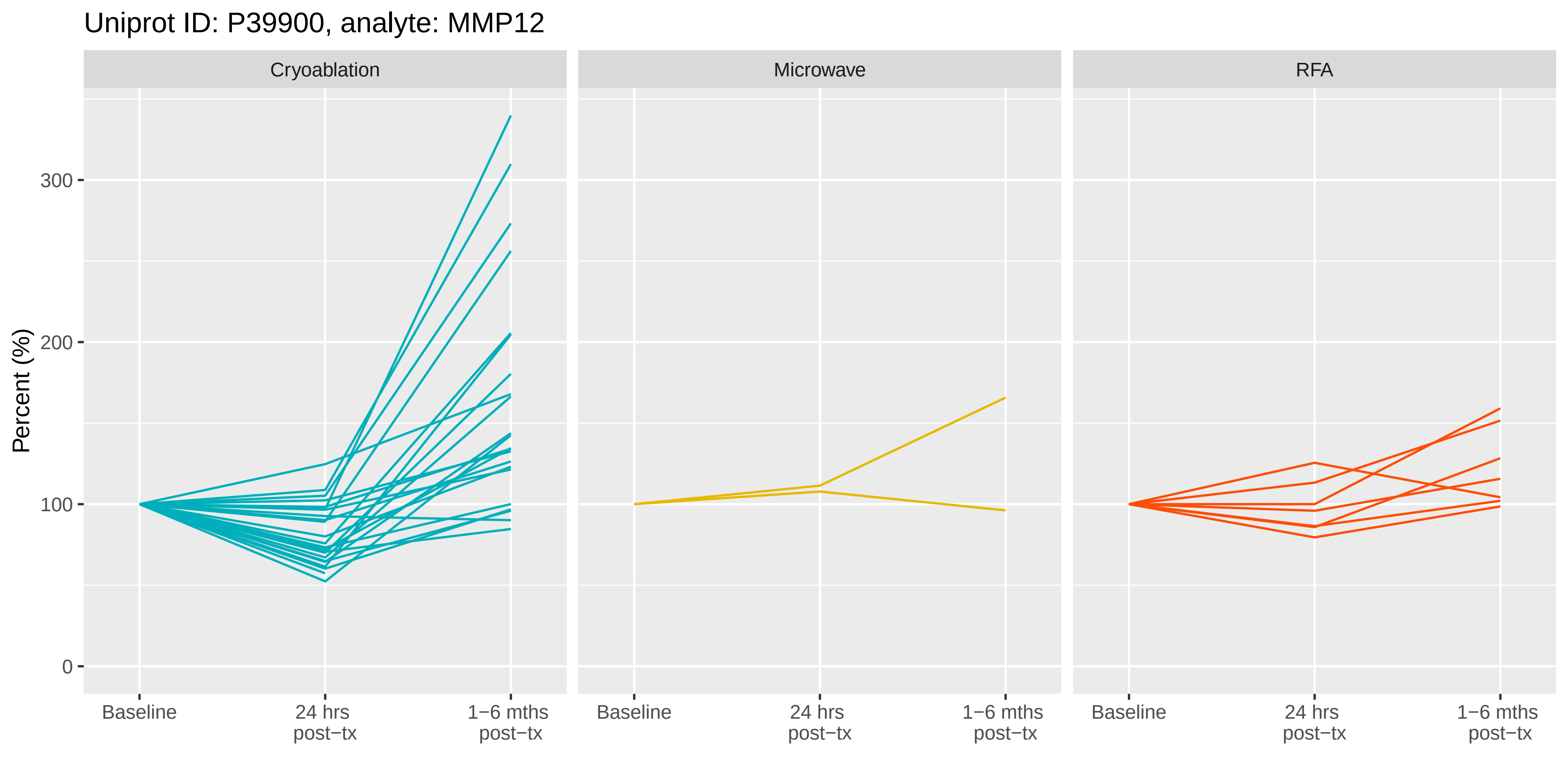

Uniprot ID: P39900, analyte: MMP12
Cryoablation
Microwave
RFA
Percent (%)
0
100
200
300
Baseline
24 hrs post−tx
1−6 mths post−tx

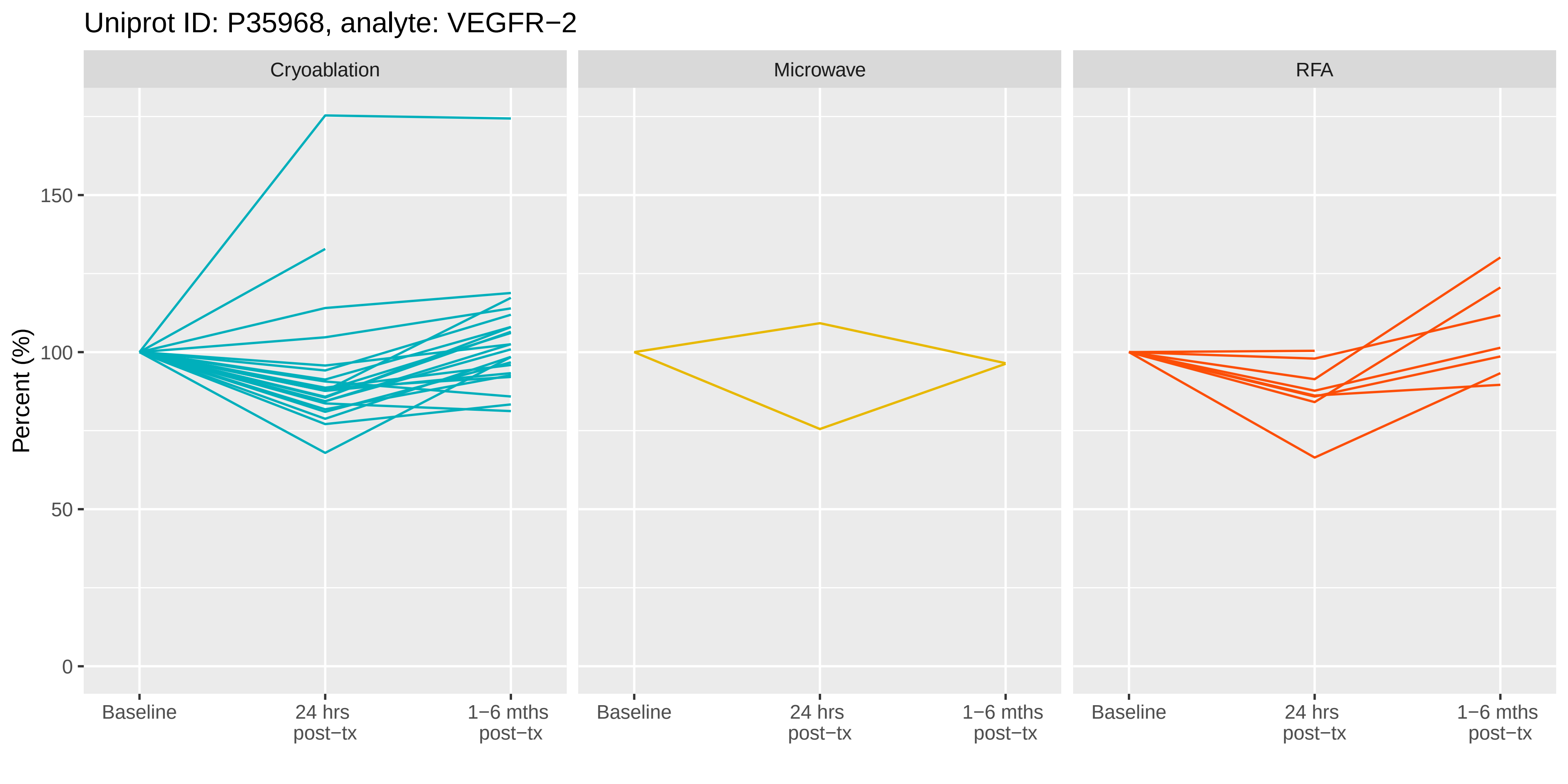

Uniprot ID: P35968, analyte: VEGFR−2
Cryoablation
Microwave
RFA
Percent (%)
0
50
100
150
Baseline
24 hrs post−tx
1−6 mths post−tx

Uniprot ID: O60911, analyte: CTSV

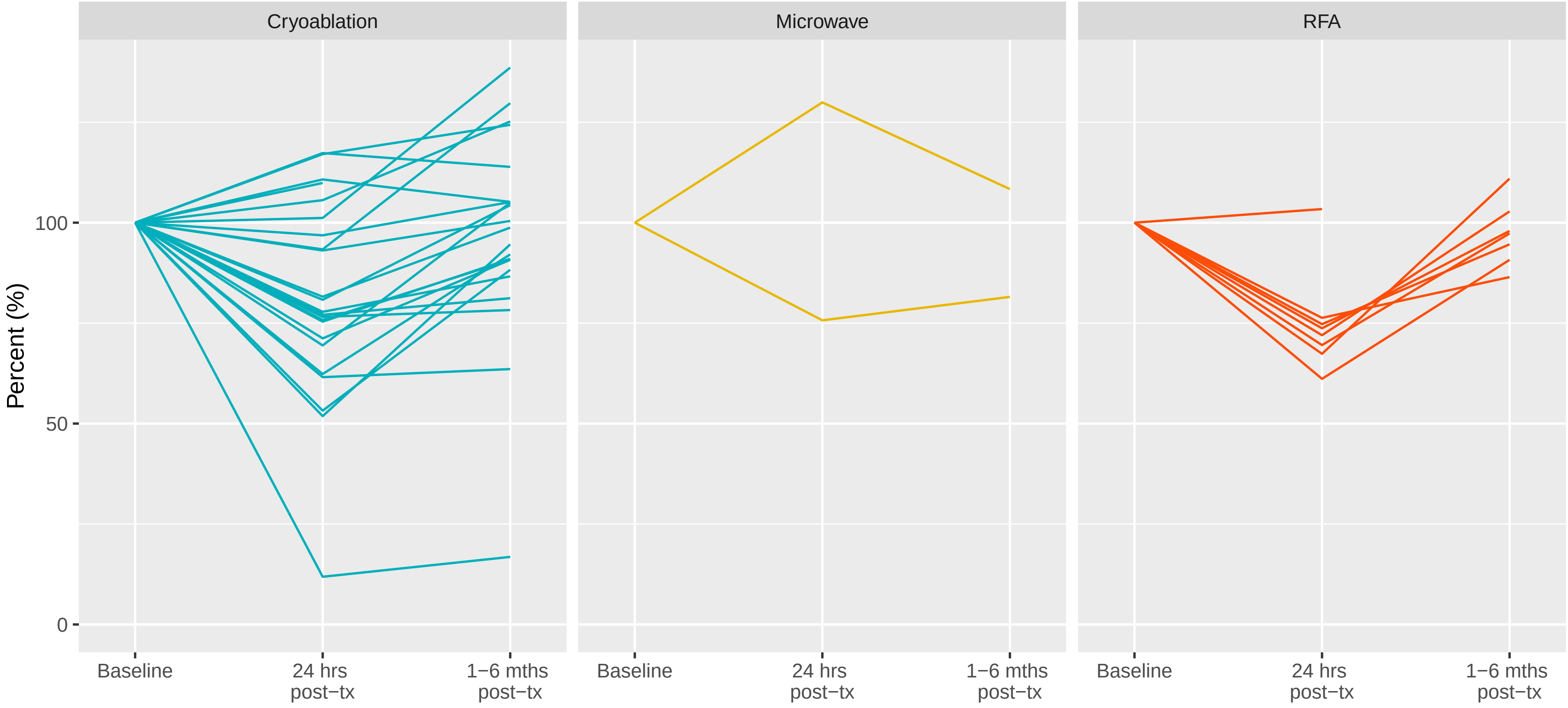

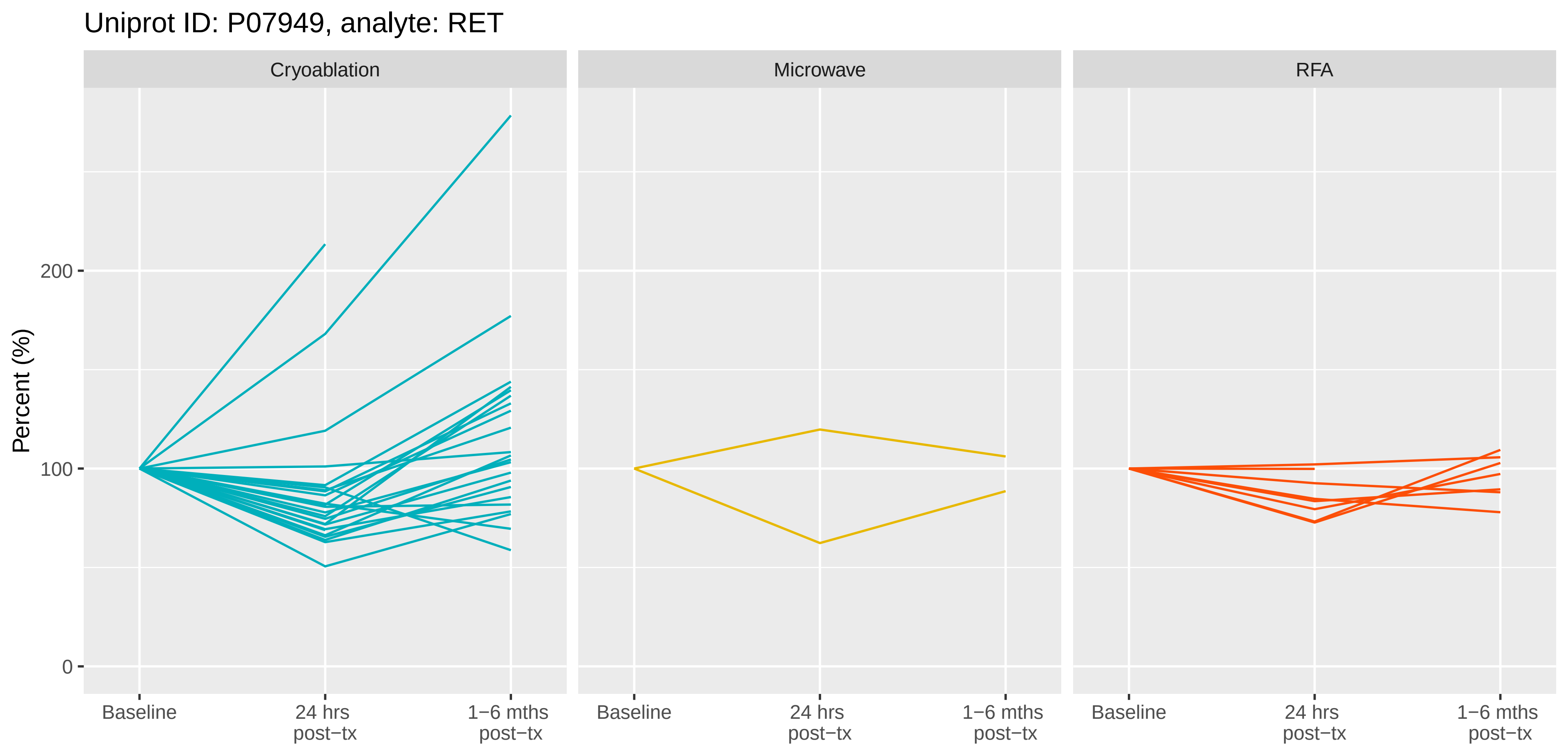
Uniprot ID: P07949, analyte: RET
Cryoablation
Microwave
RFA
Percent (%)
200
100
0
Baseline
24 hrs post−tx
1−6 mths post−tx

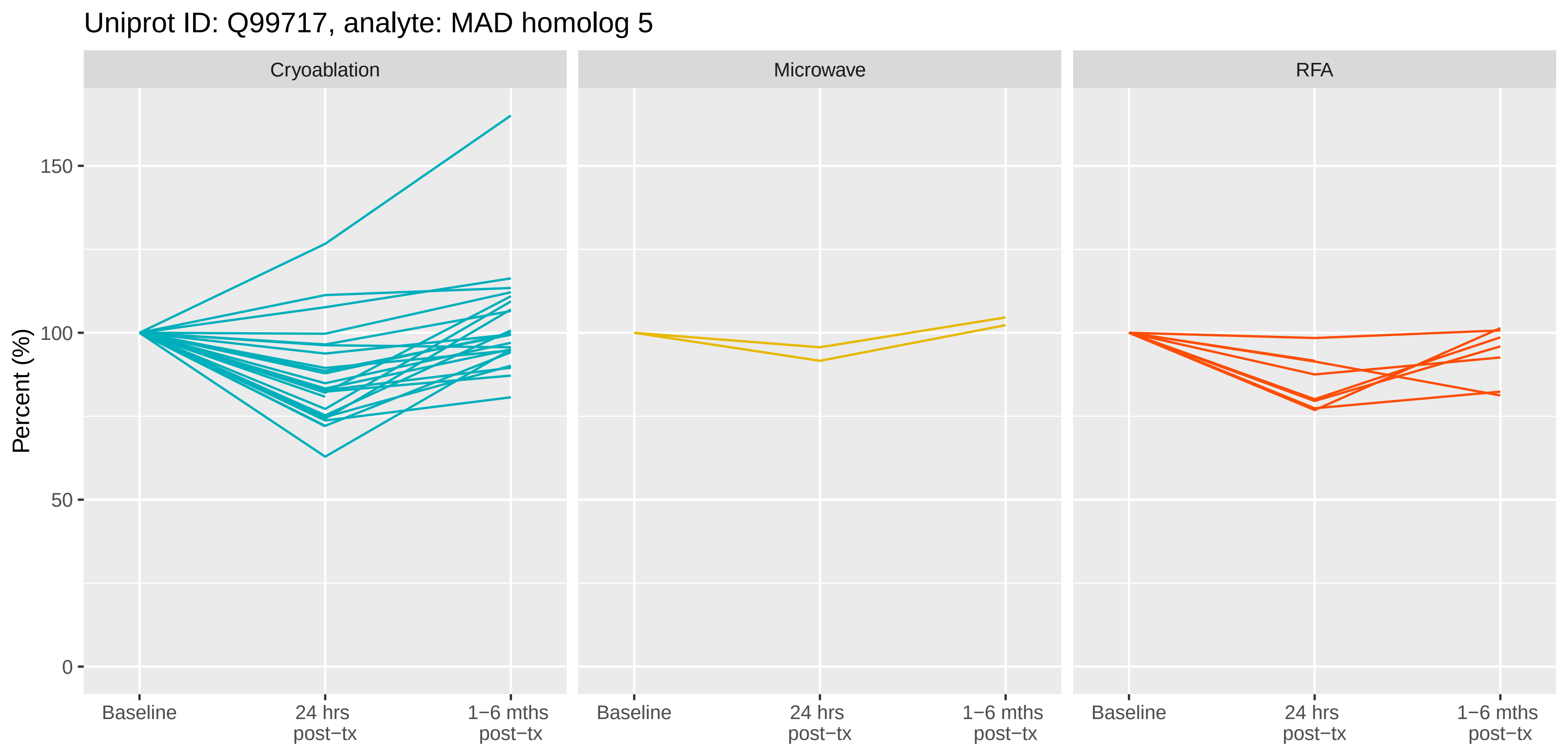

Uniprot ID: Q99717, analyte: MAD homolog 5
Cryoablation
Microwave
RFA
Percent (%)
0
50
100
150
Baseline
24 hrs post−tx
1−6 mths post−tx
