# Supplementary material for: An Exploratory Analysis of Changes in Circulating Plasma Protein Profiles Following Image-Guided Ablation of Renal Tumours Provides Evidence for Effects on Multiple Biological Processes
Source: Cancers (Basel). 2021 Nov 30;13(23):6037. doi: 10.3390/cancers13236037 (PMC8656737; doi:10.3390/cancers13236037)

Figure S7. Longitudinal profiles of all plasma proteins, prior to and post-CRYO (n=25 patients). Results for each protein are expressed as a % of the reference baseline (TP2; 100%) and are shown with different colours indicating the different histological subtypes of RCC.

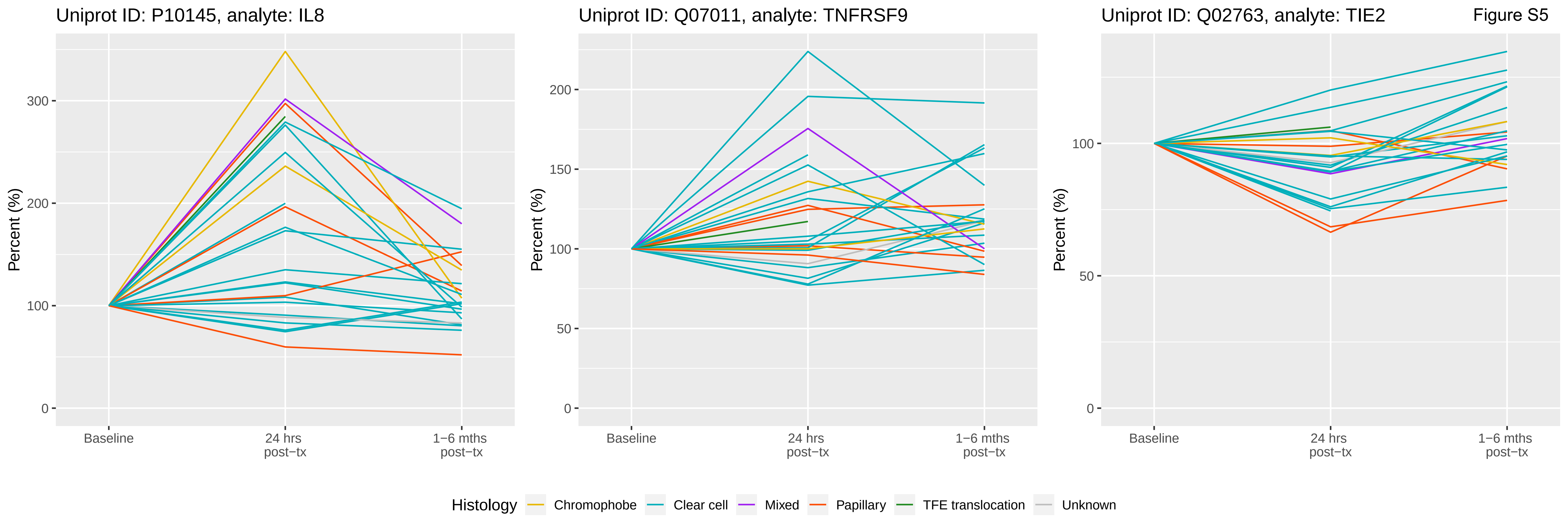
Uniprot ID: P10145, analyte: IL8
Uniprot ID: Q07011, analyte: TNFRSF9
Uniprot ID: Q02763, analyte: TIE2
Figure S5
Percent (%)
0
100
200
300
50
150
Baseline
24 hrs post−tx
1−6 mths post−tx
Histology
Chromophobe
Clear cell
Mixed
Papillary
TFE translocation
Unknown

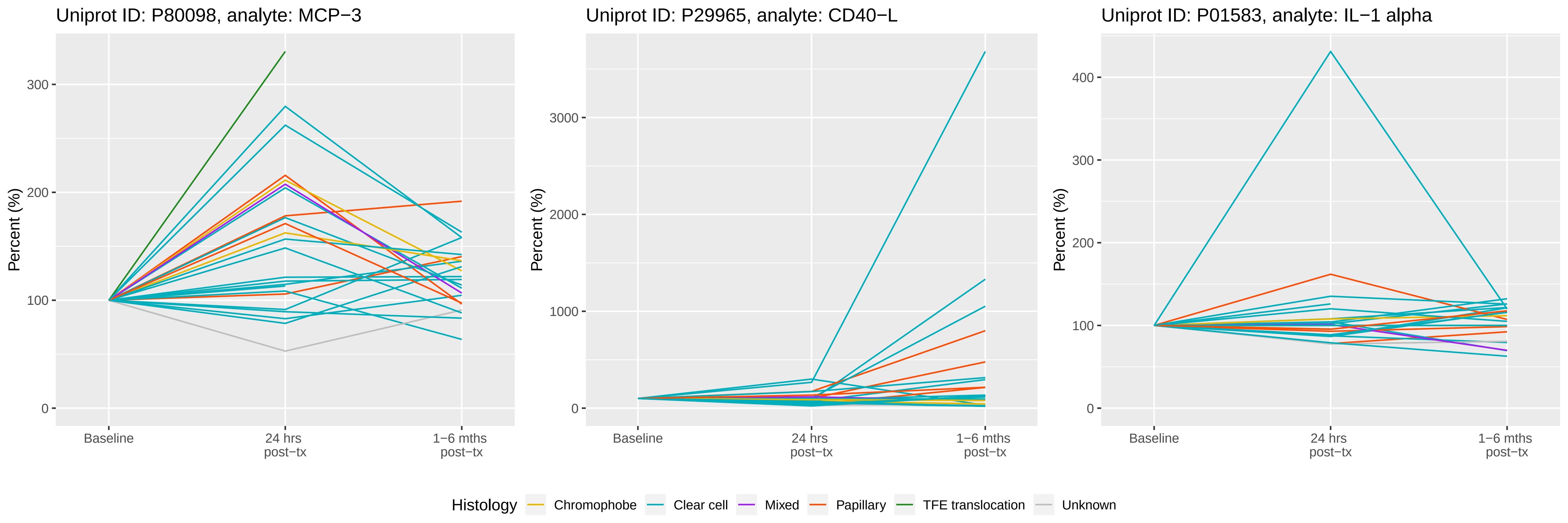

Uniprot ID: P80098, analyte: MCP−3
Uniprot ID: P29965, analyte: CD40−L
Uniprot ID: P01583, analyte: IL−1 alpha
Percent (%)
0
100
200
300
1000
2000
3000
400
Baseline
24 hrs post−tx
1−6 mths post−tx
Histology
Chromophobe
Clear cell
Mixed
Papillary
TFE translocation
Unknown

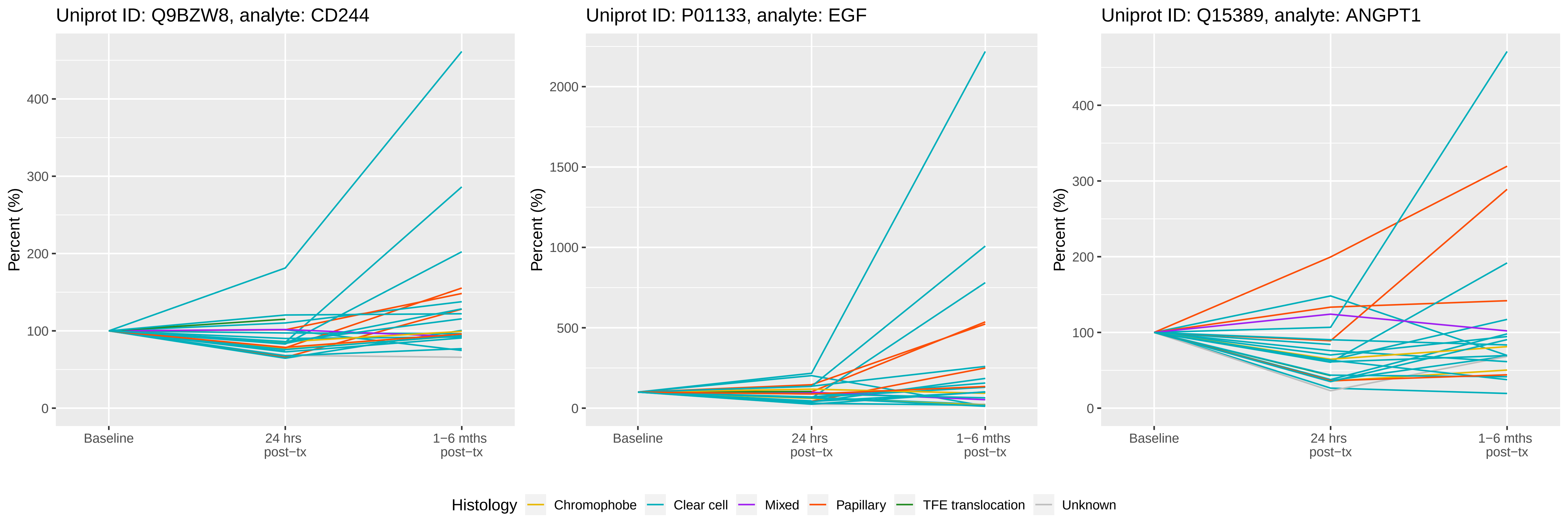

Uniprot ID: Q9BZW8, analyte: CD244
Uniprot ID: P01133, analyte: EGF
Uniprot ID: Q15389, analyte: ANGPT1
Percent (%)
Baseline
24 hrs post−tx
1−6 mths post−tx
Histology
Chromophobe
Clear cell
Mixed
Papillary
TFE translocation
Unknown

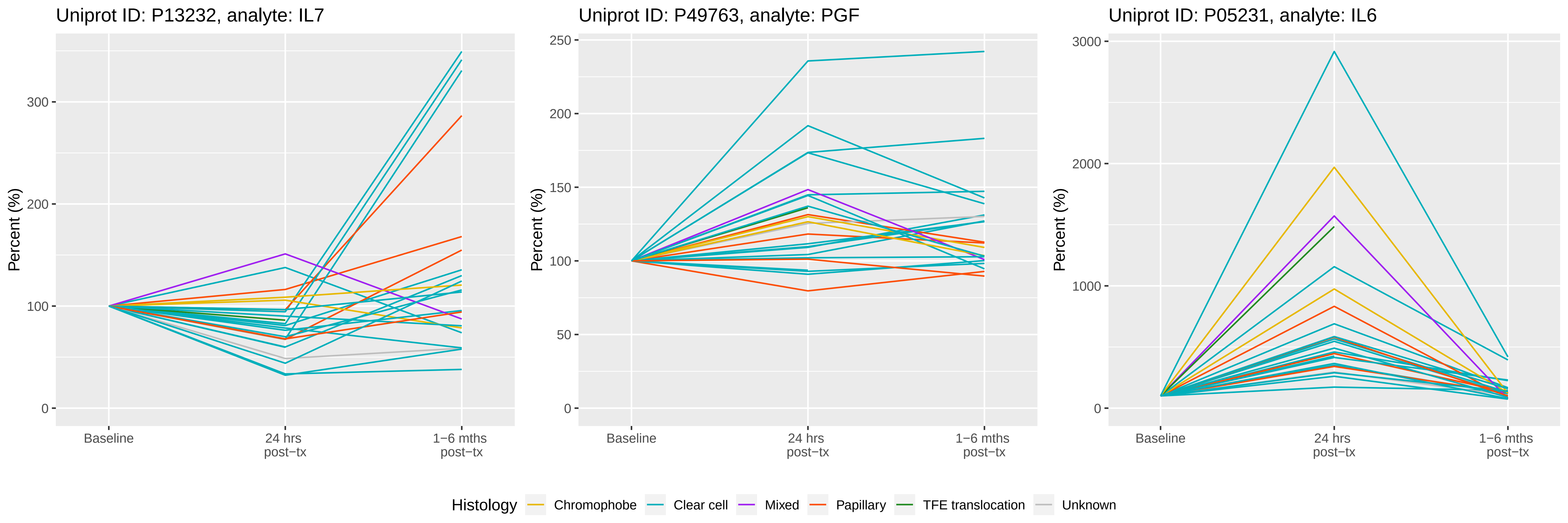

Uniprot ID: P13232, analyte: IL7
Uniprot ID: P49763, analyte: PGF
Uniprot ID: P05231, analyte: IL6
Percent (%)
Baseline
24 hrs post−tx
1−6 mths post−tx
Histology
Chromophobe
Clear cell
Mixed
Papillary
TFE translocation
Unknown

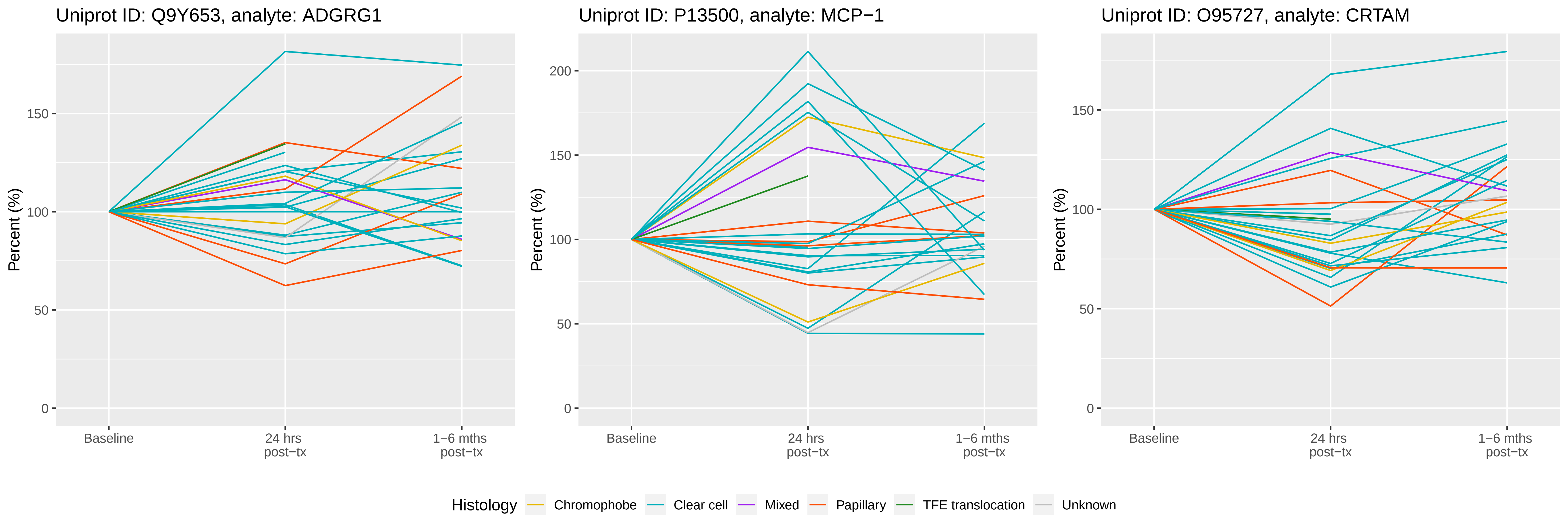
Uniprot ID: Q9Y653, analyte: ADGRG1
Uniprot ID: P13500, analyte: MCP−1
Uniprot ID: O95727, analyte: CRTAM
Percent (%)
0
50
100
150
200
Baseline
24 hrs post−tx
1−6 mths post−tx
Histology
Chromophobe
Clear cell
Mixed
Papillary
TFE translocation
Unknown

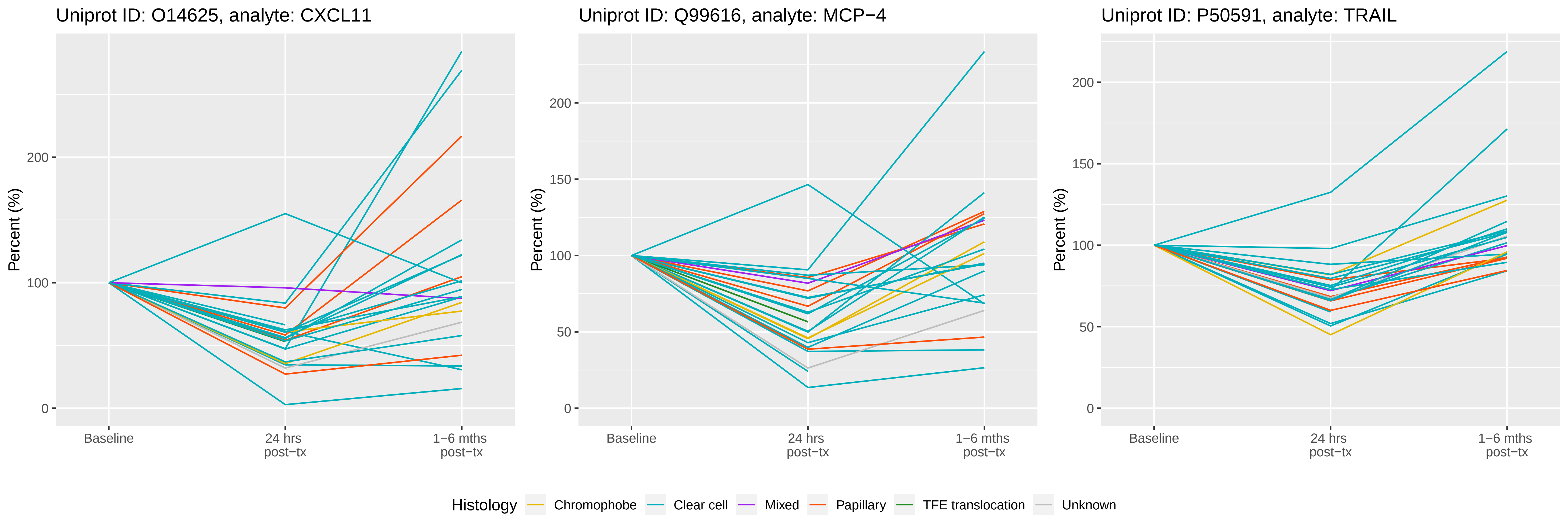

Uniprot ID: O14625, analyte: CXCL11
Uniprot ID: Q99616, analyte: MCP−4
Uniprot ID: P50591, analyte: TRAIL
Percent (%)
0
50
100
150
200
Baseline
24 hrs post−tx
1−6 mths post−tx
Histology
Chromophobe
Clear cell
Mixed
Papillary
TFE translocation
Unknown

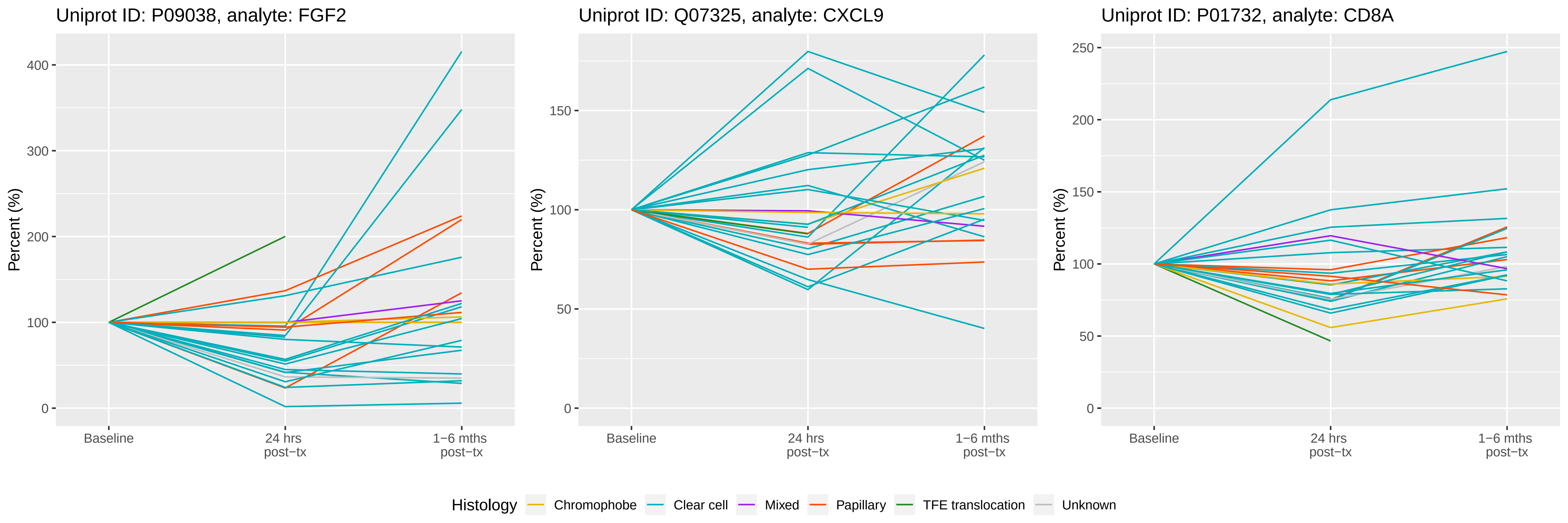

Uniprot ID: P09038, analyte: FGF2
Uniprot ID: Q07325, analyte: CXCL9
Uniprot ID: P01732, analyte: CD8A
Percent (%)
0
100
200
300
400
50
150
250
Baseline
24 hrs post−tx
1−6 mths post−tx
Histology
Chromophobe
Clear cell
Mixed
Papillary
TFE translocation
Unknown

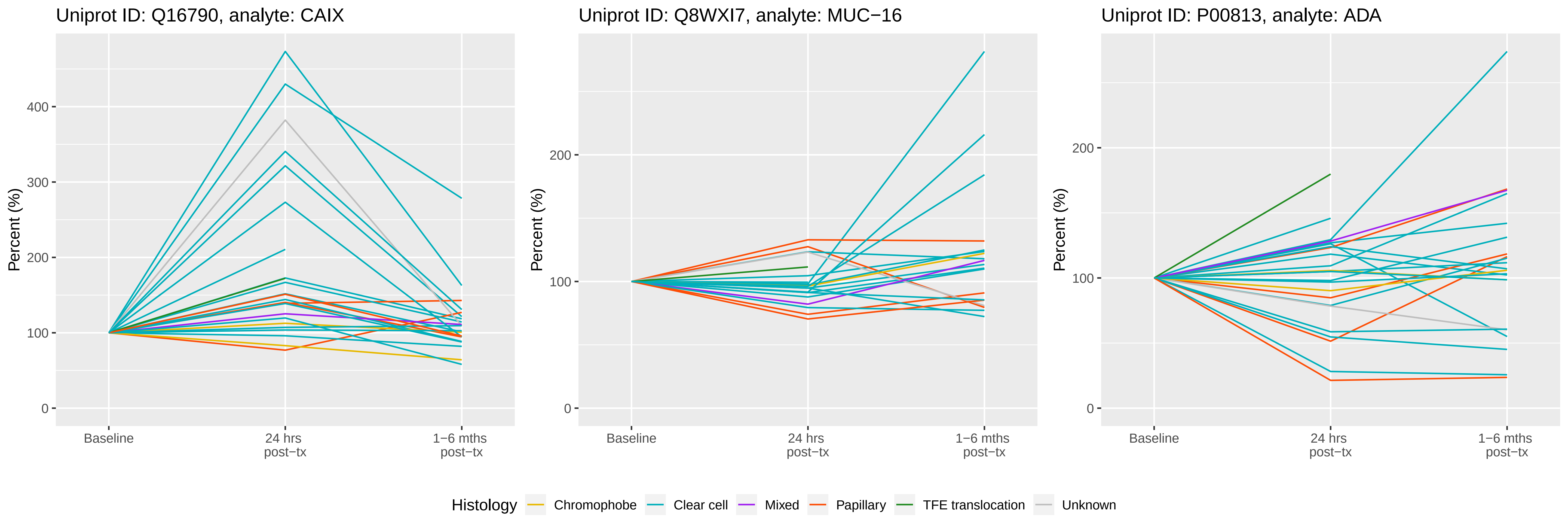

Uniprot ID: Q16790, analyte: CAIX
Uniprot ID: Q8WXI7, analyte: MUC−16
Uniprot ID: P00813, analyte: ADA
Percent (%)
0
100
200
300
400
Baseline
24 hrs post−tx
1−6 mths post−tx
Histology
Chromophobe
Clear cell
Mixed
Papillary
TFE translocation
Unknown

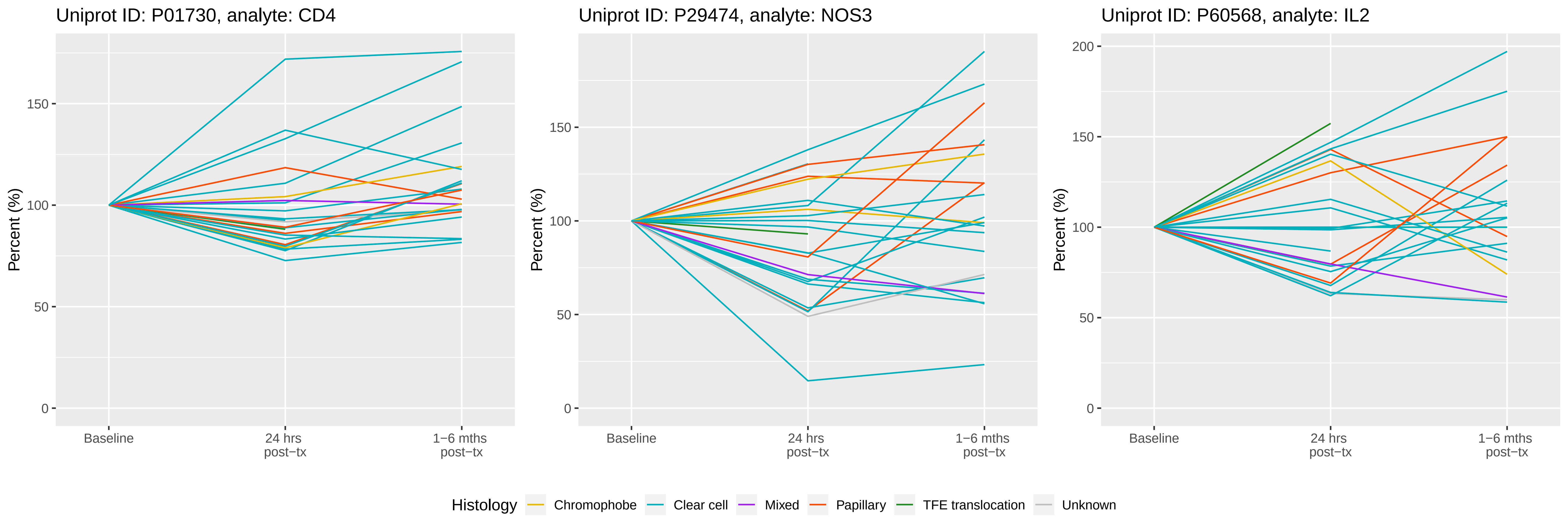

Uniprot ID: P01730, analyte: CD4
Uniprot ID: P29474, analyte: NOS3
Uniprot ID: P60568, analyte: IL2
Percent (%)
0
50
100
150
200
Baseline
24 hrs post−tx
1−6 mths post−tx
Histology
Chromophobe
Clear cell
Mixed
Papillary
TFE translocation
Unknown

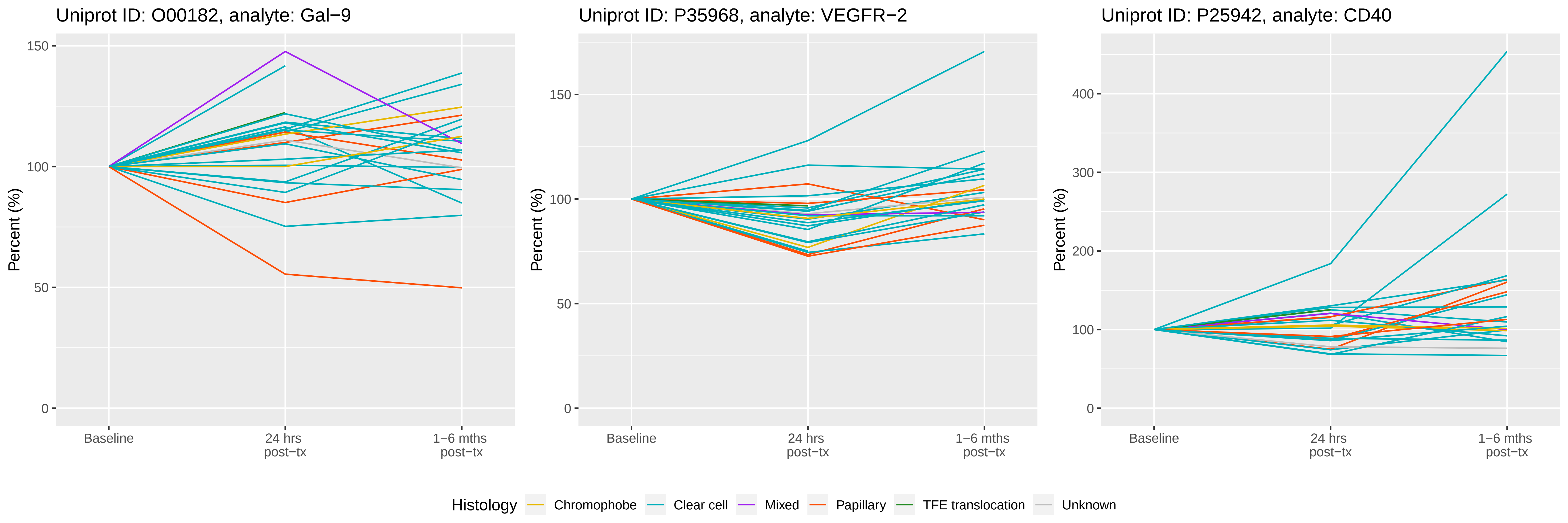
Uniprot ID: O00182, analyte: Gal−9
Uniprot ID: P35968, analyte: VEGFR−2
Uniprot ID: P25942, analyte: CD40
Percent (%)
0
50
100
150
200
300
400
Baseline
24 hrs post−tx
1−6 mths post−tx
Histology
Chromophobe
Clear cell
Mixed
Papillary
TFE translocation
Unknown

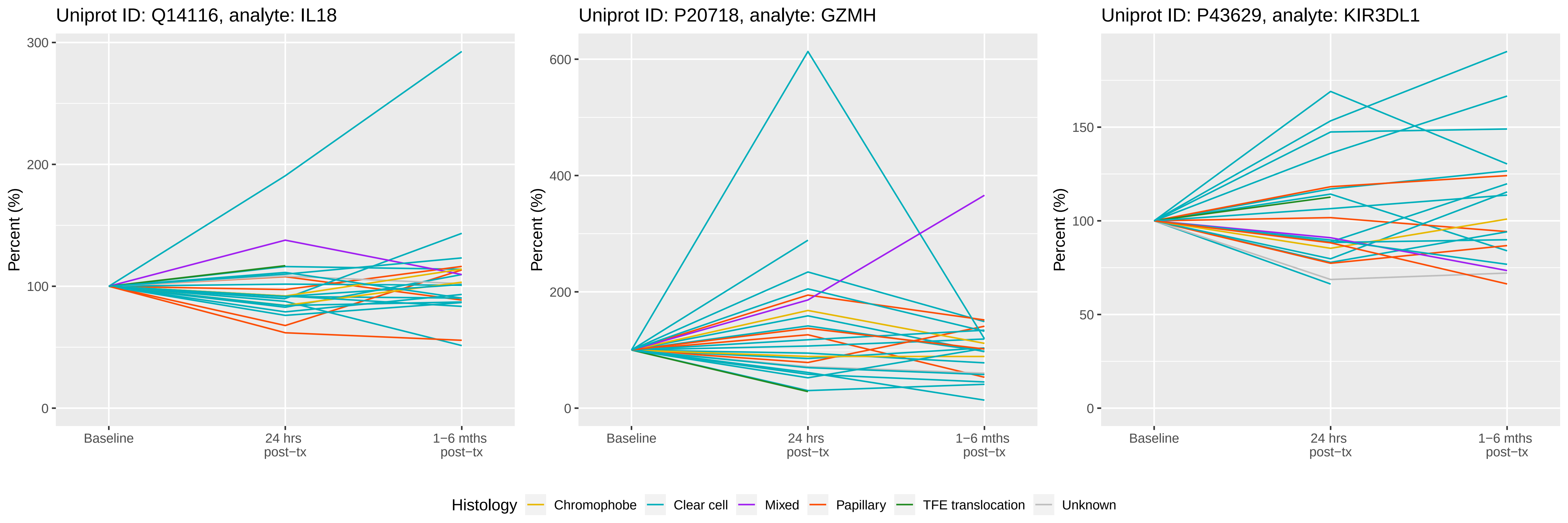

Uniprot ID: Q14116, analyte: IL18
Uniprot ID: P20718, analyte: GZMH
Uniprot ID: P43629, analyte: KIR3DL1
Percent (%)
0
100
200
300
400
600
50
150
Baseline
24 hrs post−tx
1−6 mths post−tx
Histology
Chromophobe
Clear cell
Mixed
Papillary
TFE translocation
Unknown

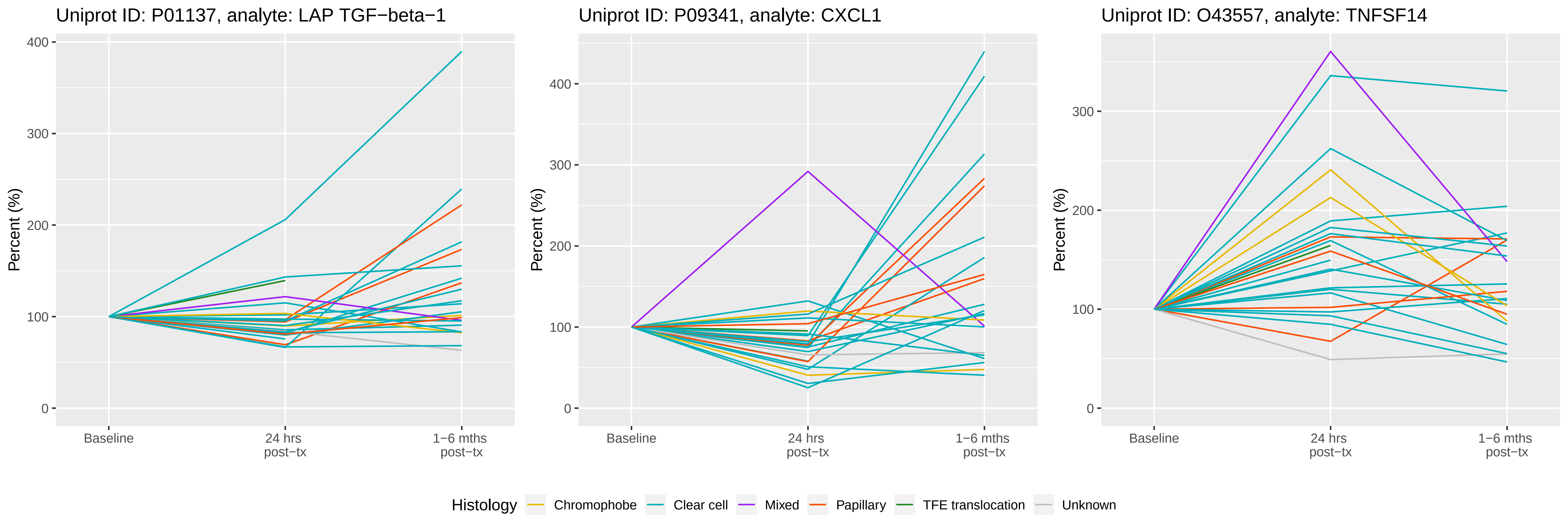

Uniprot ID: P01137, analyte: LAP TGF−beta−1
Uniprot ID: P09341, analyte: CXCL1
Uniprot ID: O43557, analyte: TNFSF14
Percent (%)
0
100
200
300
400
Baseline
24 hrs post−tx
1−6 mths post−tx
Histology
Chromophobe
Clear cell
Mixed
Papillary
TFE translocation
Unknown

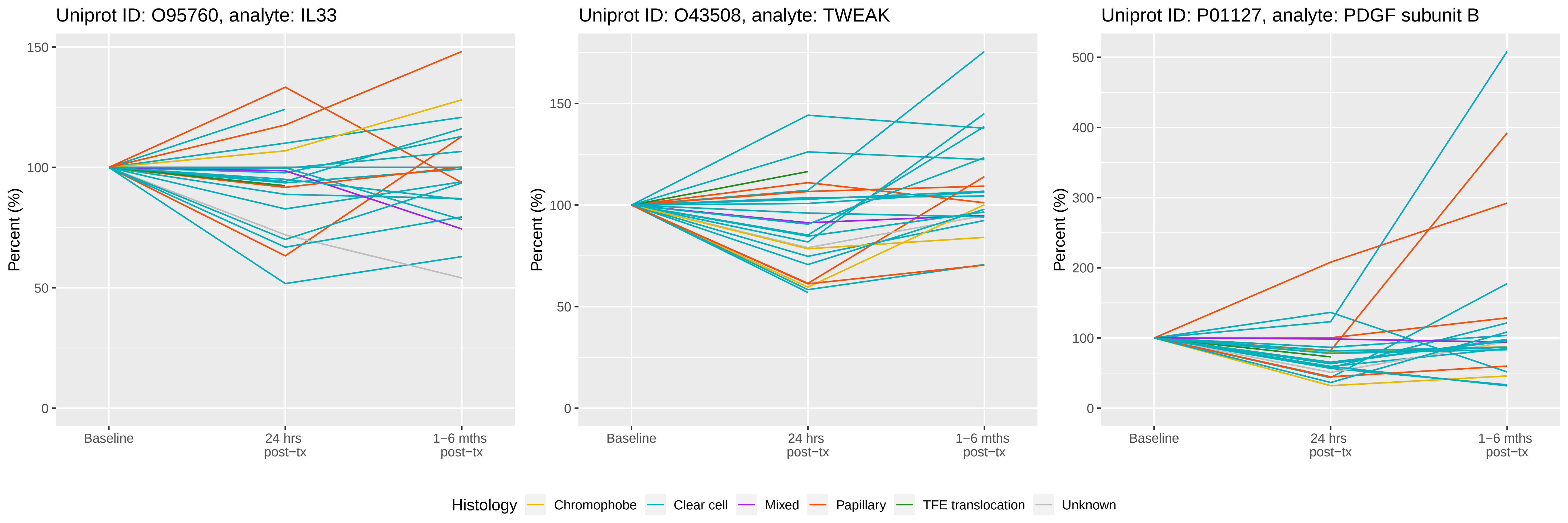
Uniprot ID: O95760, analyte: IL33
Uniprot ID: O43508, analyte: TWEAK
Uniprot ID: P01127, analyte: PDGF subunit B
Percent (%)
Baseline
24 hrs post−tx
1−6 mths post−tx
Histology
Chromophobe
Clear cell
Mixed
Papillary
TFE translocation
Unknown

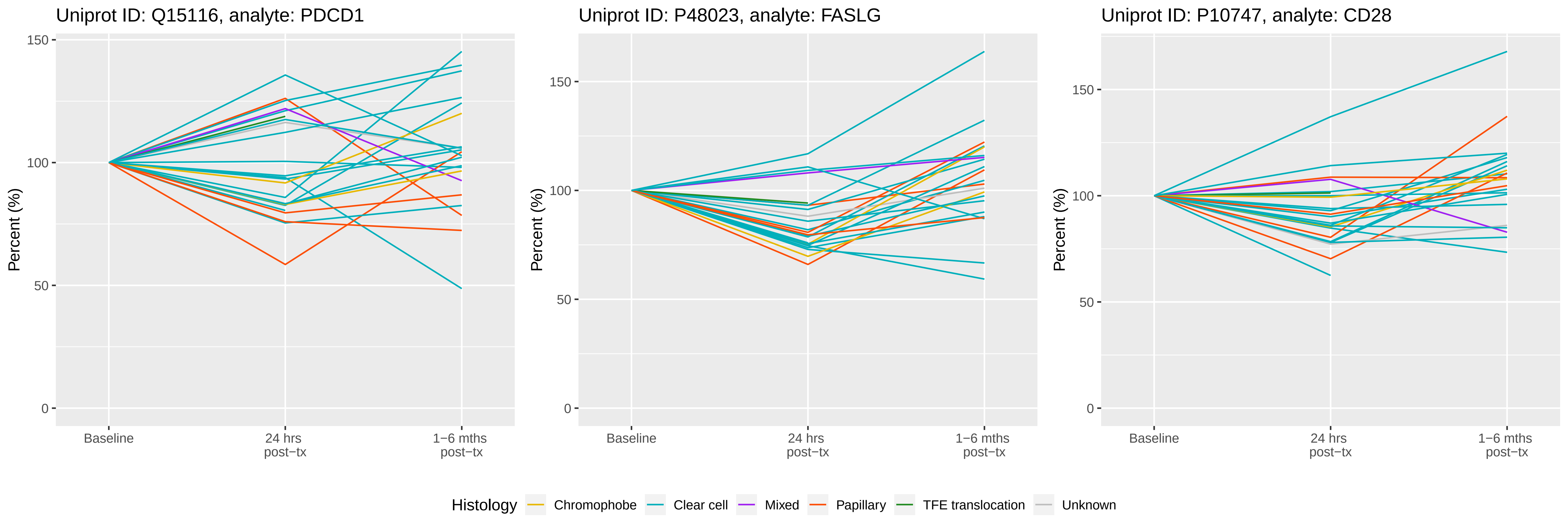

Uniprot ID: Q15116, analyte: PDCD1
Uniprot ID: P48023, analyte: FASLG
Uniprot ID: P10747, analyte: CD28
Percent (%)
0
50
100
150
Baseline
24 hrs post−tx
1−6 mths post−tx
Histology
Chromophobe
Clear cell
Mixed
Papillary
TFE translocation
Unknown

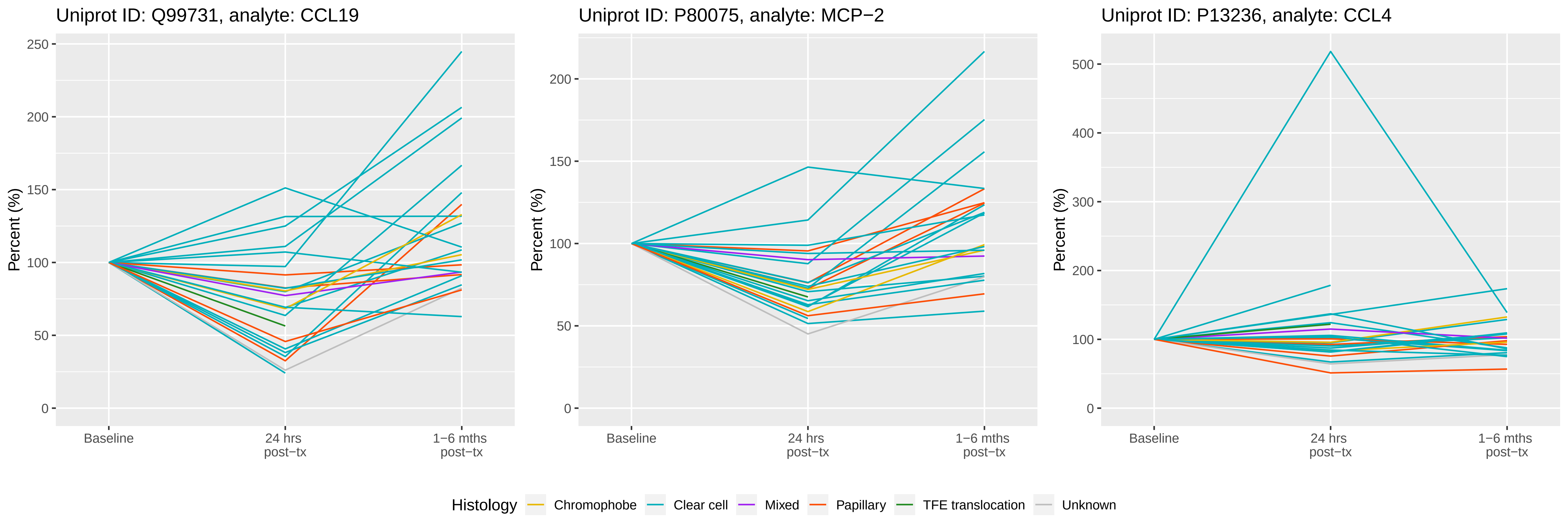
Uniprot ID: Q99731, analyte: CCL19
Uniprot ID: P80075, analyte: MCP−2
Uniprot ID: P13236, analyte: CCL4
Percent (%)
0
50
100
150
200
250
300
400
500
Baseline
24 hrs post−tx
1−6 mths post−tx
Histology
Chromophobe
Clear cell
Mixed
Papillary
TFE translocation
Unknown

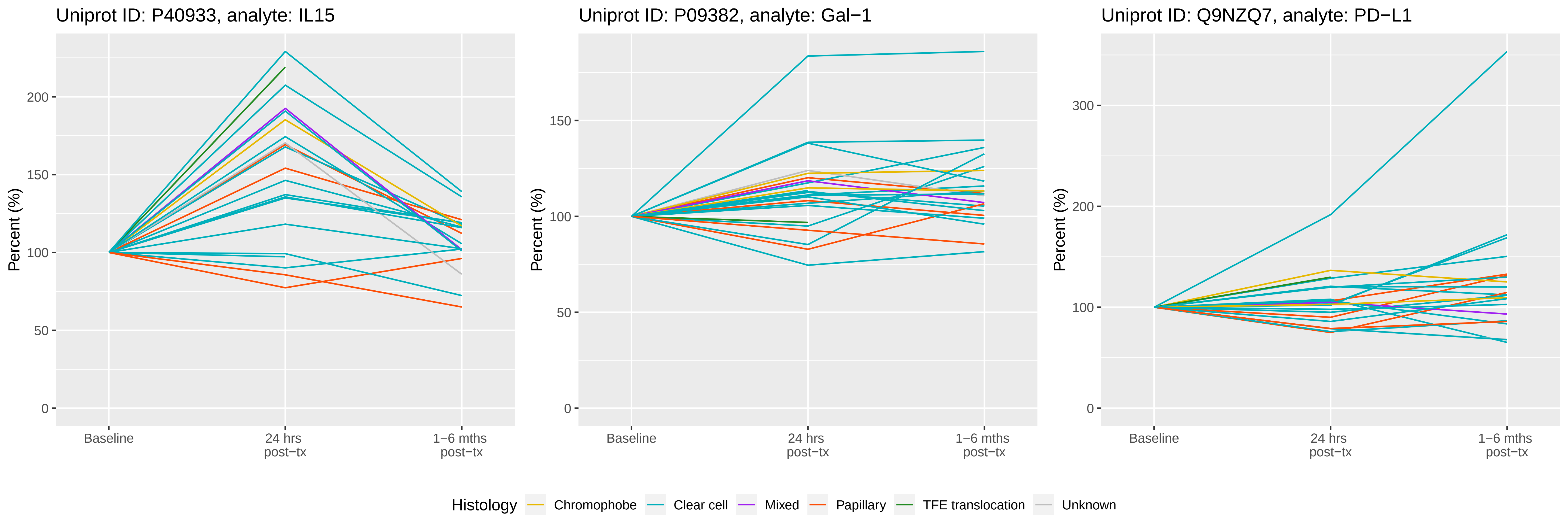
Uniprot ID: P40933, analyte: IL15
Uniprot ID: P09382, analyte: Gal−1
Uniprot ID: Q9NZQ7, analyte: PD−L1
Percent (%)
0
50
100
150
200
300
Baseline
24 hrs post−tx
1−6 mths post−tx
Histology
Chromophobe
Clear cell
Mixed
Papillary
TFE translocation
Unknown

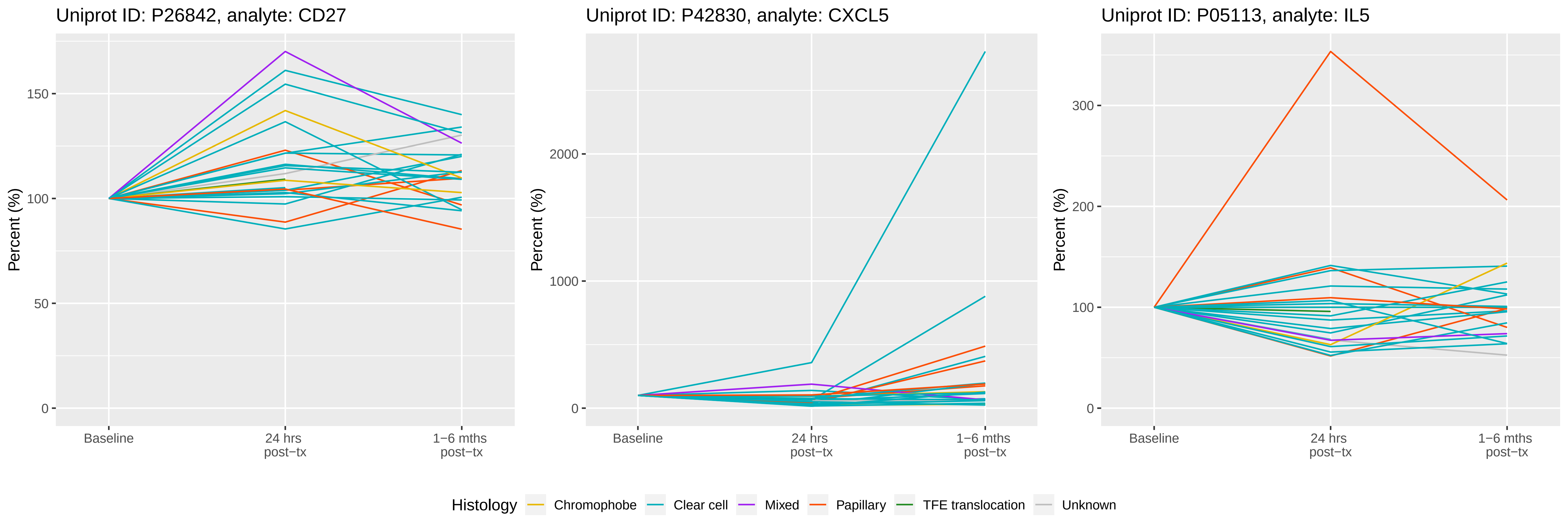
Uniprot ID: P26842, analyte: CD27
Uniprot ID: P42830, analyte: CXCL5
Uniprot ID: P05113, analyte: IL5
Percent (%)
Baseline
24 hrs post−tx
1−6 mths post−tx
Histology
Chromophobe
Clear cell
Mixed
Papillary
TFE translocation
Unknown

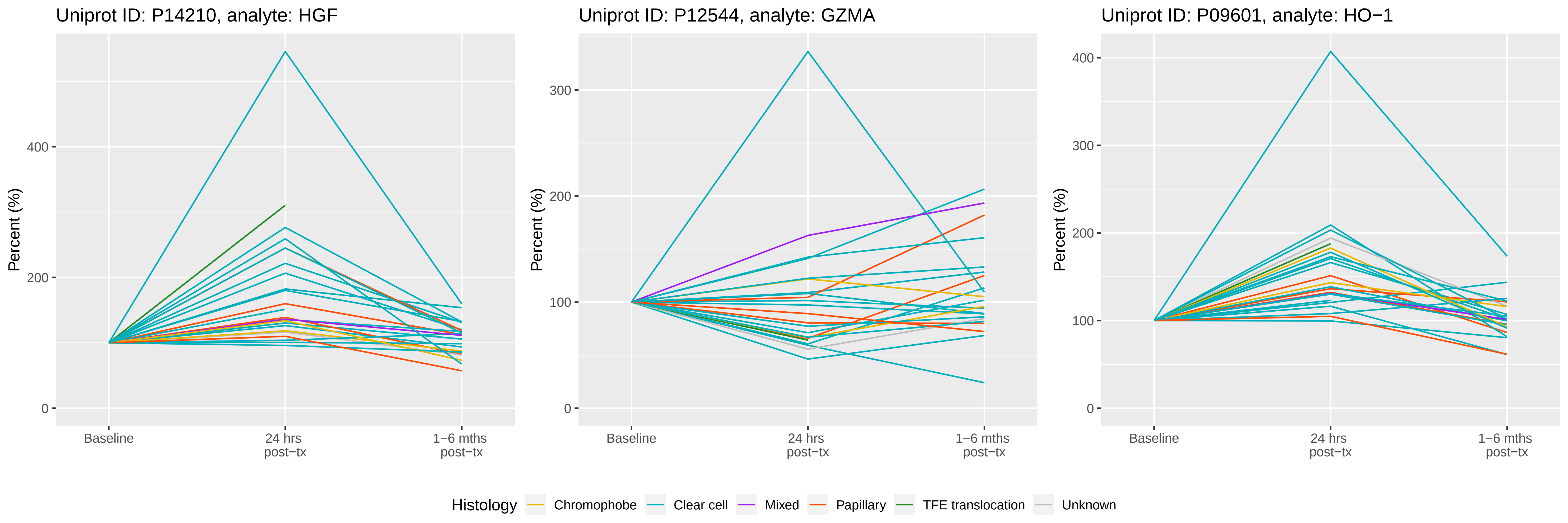

Uniprot ID: P14210, analyte: HGF
Uniprot ID: P12544, analyte: GZMA
Uniprot ID: P09601, analyte: HO−1
Percent (%)
0
200
400
100
300
Baseline
24 hrs post−tx
1−6 mths post−tx
Histology
Chromophobe
Clear cell
Mixed
Papillary
TFE translocation
Unknown

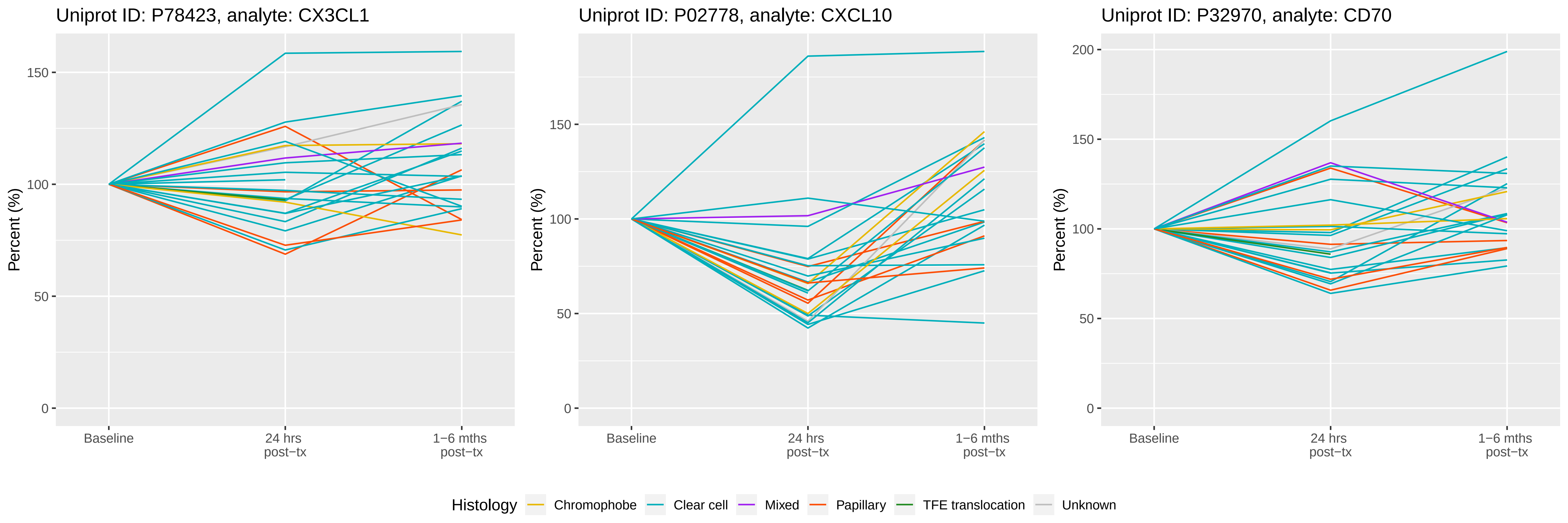

Uniprot ID: P78423, analyte: CX3CL1
Uniprot ID: P02778, analyte: CXCL10
Uniprot ID: P32970, analyte: CD70
Percent (%)
0
50
100
150
200
Baseline
24 hrs
post−tx
1−6 mths
post−tx
Histology
Chromophobe
Clear cell
Mixed
Papillary
TFE translocation
Unknown

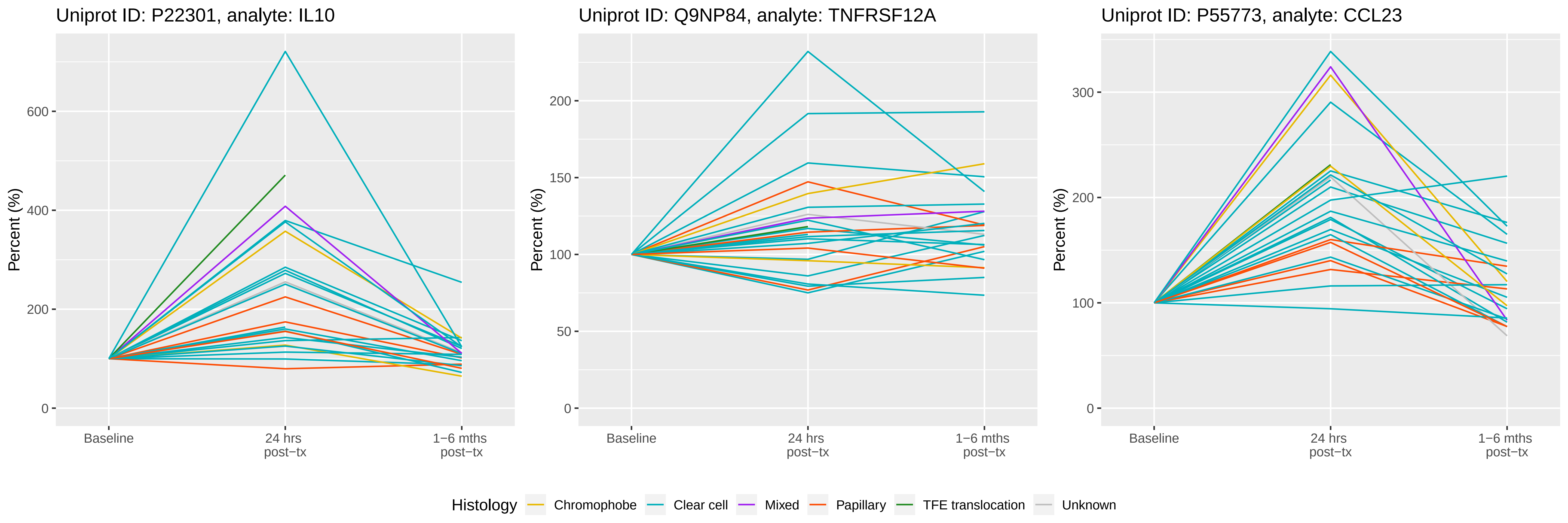

Uniprot ID: P22301, analyte: IL10
Uniprot ID: Q9NP84, analyte: TNFRSF12A
Uniprot ID: P55773, analyte: CCL23
Percent (%)
0
200
400
600
0
50
100
150
200
0
100
200
300
Baseline
24 hrs post−tx
1−6 mths post−tx
Histology
Chromophobe
Clear cell
Mixed
Papillary
TFE translocation
Unknown

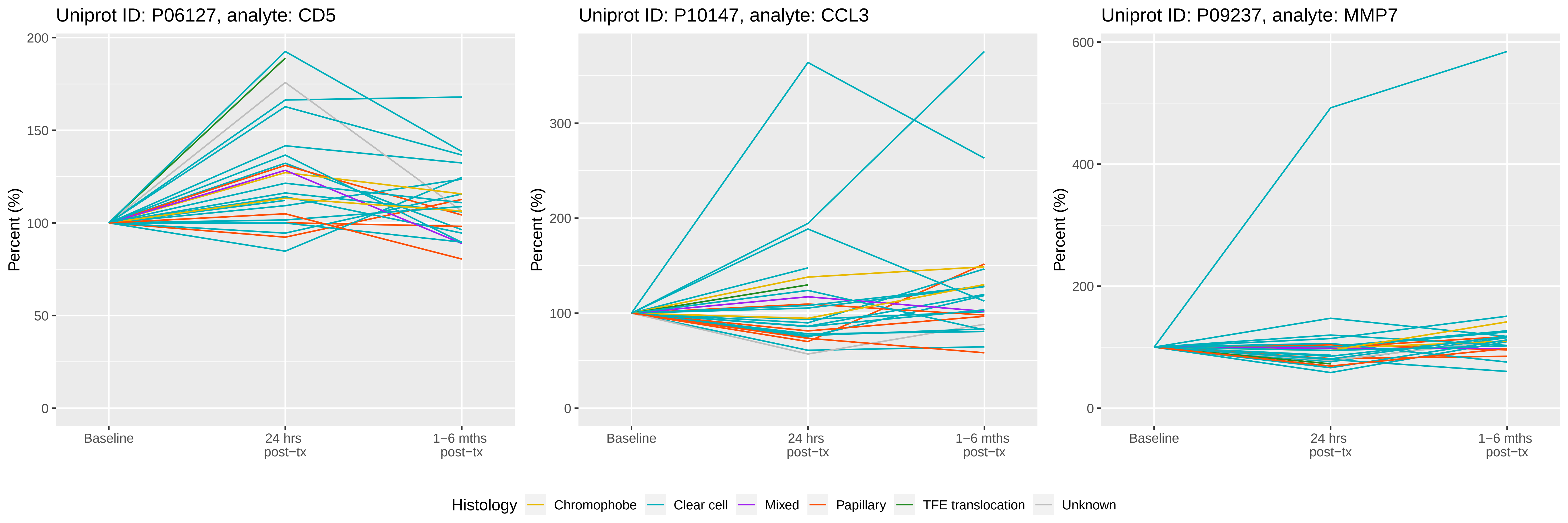
Uniprot ID: P06127, analyte: CD5
Uniprot ID: P10147, analyte: CCL3
Uniprot ID: P09237, analyte: MMP7
Percent (%)
Baseline
24 hrs post−tx
1−6 mths post−tx
Histology
Chromophobe
Clear cell
Mixed
Papillary
TFE translocation
Unknown

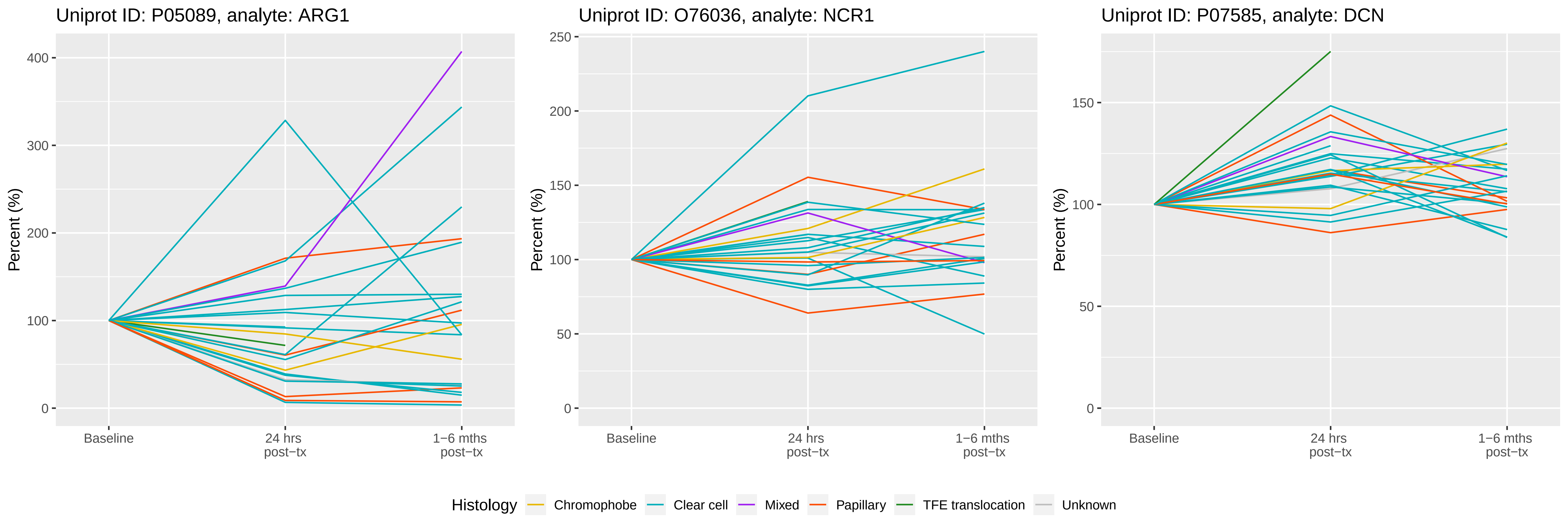

Uniprot ID: P05089, analyte: ARG1
Uniprot ID: O76036, analyte: NCR1
Uniprot ID: P07585, analyte: DCN
Percent (%)
Baseline
24 hrs post−tx
1−6 mths post−tx
Histology
Chromophobe
Clear cell
Mixed
Papillary
TFE translocation
Unknown

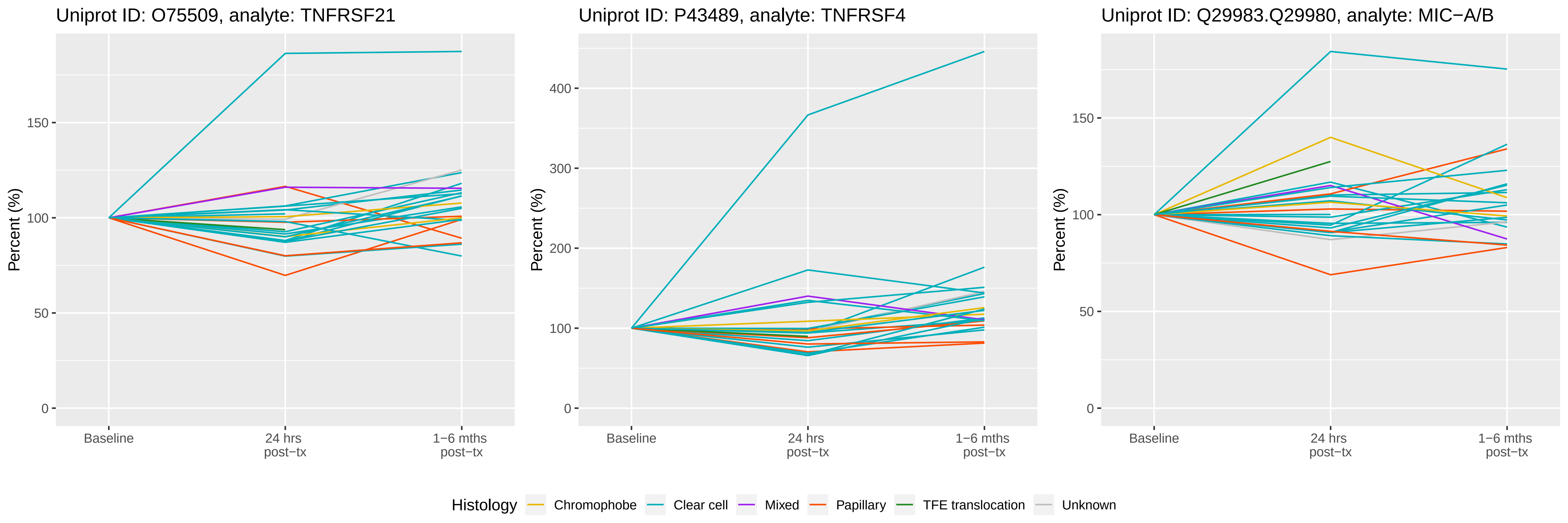

Uniprot ID: O75509, analyte: TNFRSF21
Uniprot ID: P43489, analyte: TNFRSF4
Uniprot ID: Q29983.Q29980, analyte: MIC−A/B
Percent (%)
Baseline
24 hrs post−tx
1−6 mths post−tx
Histology
Chromophobe
Clear cell
Mixed
Papillary
TFE translocation
Unknown

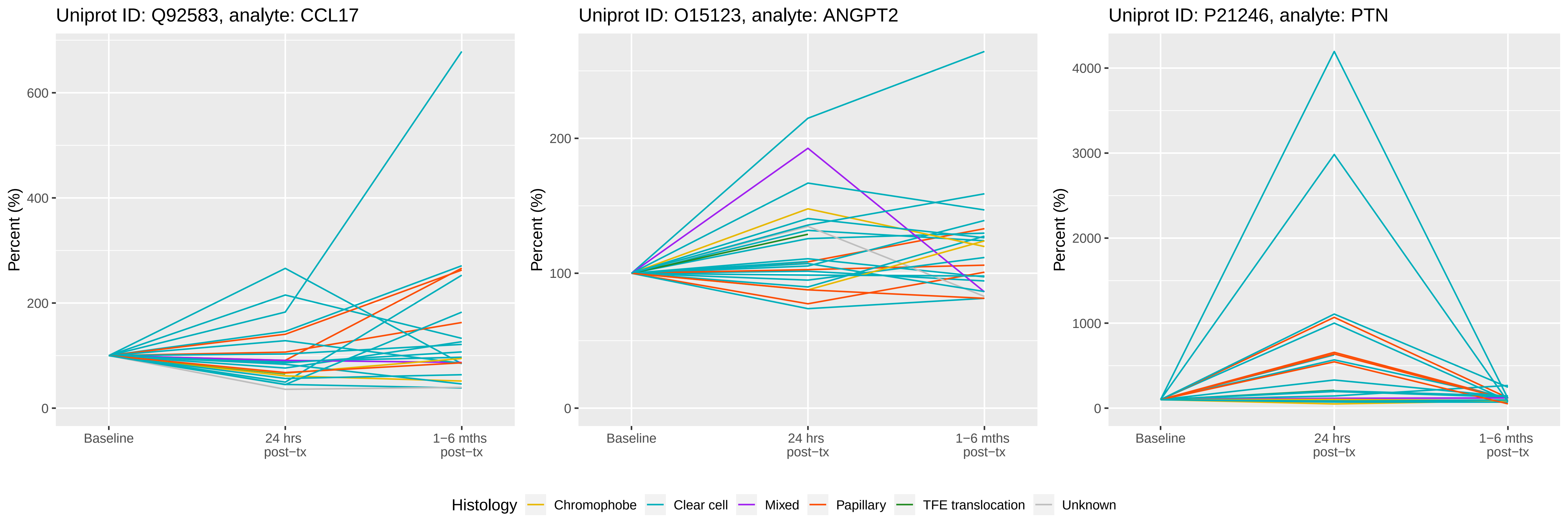

Uniprot ID: Q92583, analyte: CCL17
Uniprot ID: O15123, analyte: ANGPT2
Uniprot ID: P21246, analyte: PTN
Percent (%)
Baseline
24 hrs post−tx
1−6 mths post−tx
Histology
Chromophobe
Clear cell
Mixed
Papillary
TFE translocation
Unknown

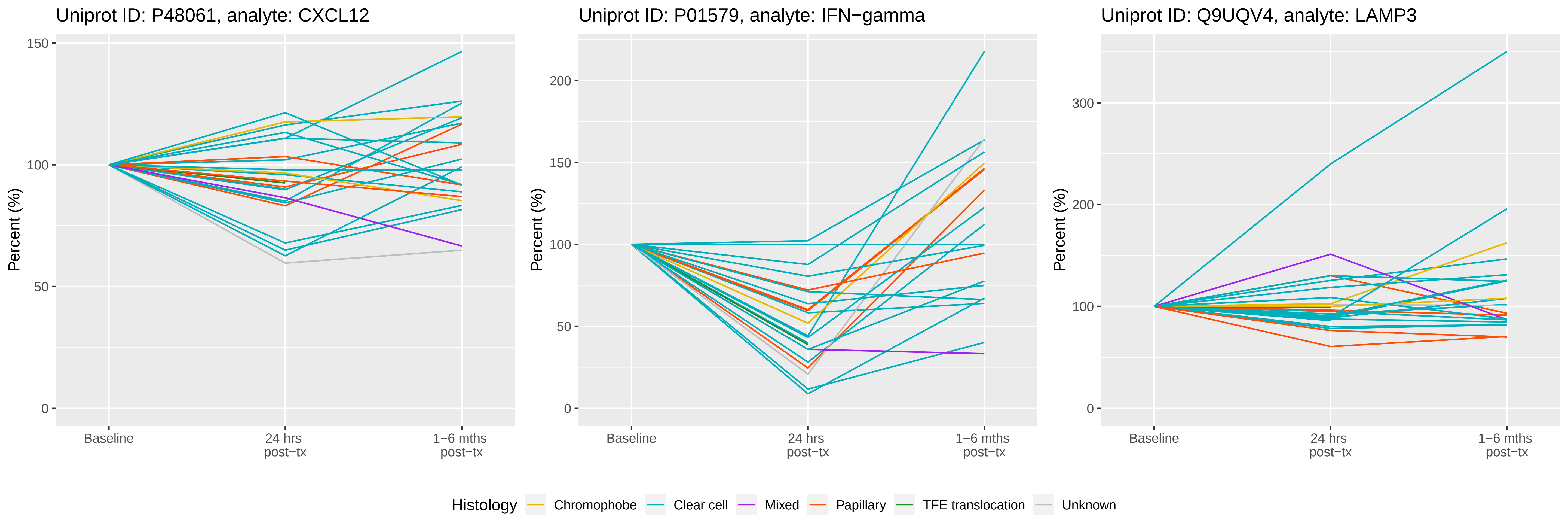

Uniprot ID: P48061, analyte: CXCL12
Uniprot ID: P01579, analyte: IFN−gamma
Uniprot ID: Q9UQV4, analyte: LAMP3
Percent (%)
0
50
100
150
200
300
Baseline
24 hrs post−tx
1−6 mths post−tx
Histology
Chromophobe
Clear cell
Mixed
Papillary
TFE translocation
Unknown

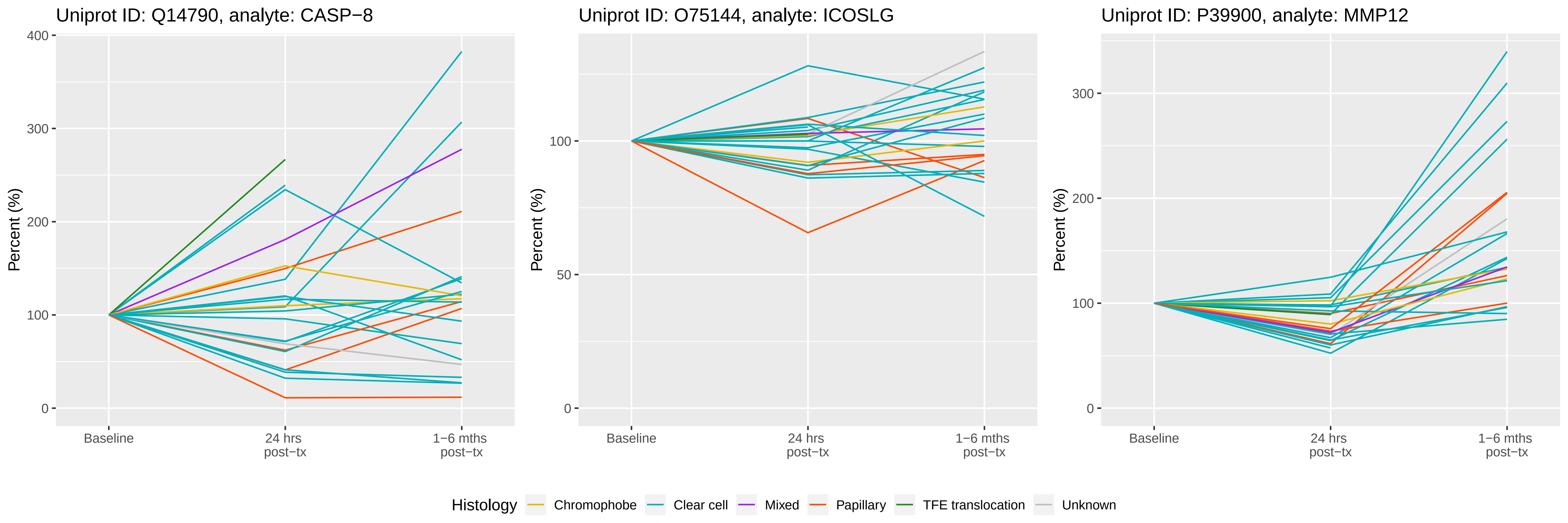

Uniprot ID: Q14790, analyte: CASP−8
Uniprot ID: O75144, analyte: ICOSLG
Uniprot ID: P39900, analyte: MMP12
Percent (%)
0
100
200
300
400
50
Baseline
24 hrs post−tx
1−6 mths post−tx
Histology
Chromophobe
Clear cell
Mixed
Papillary
TFE translocation
Unknown

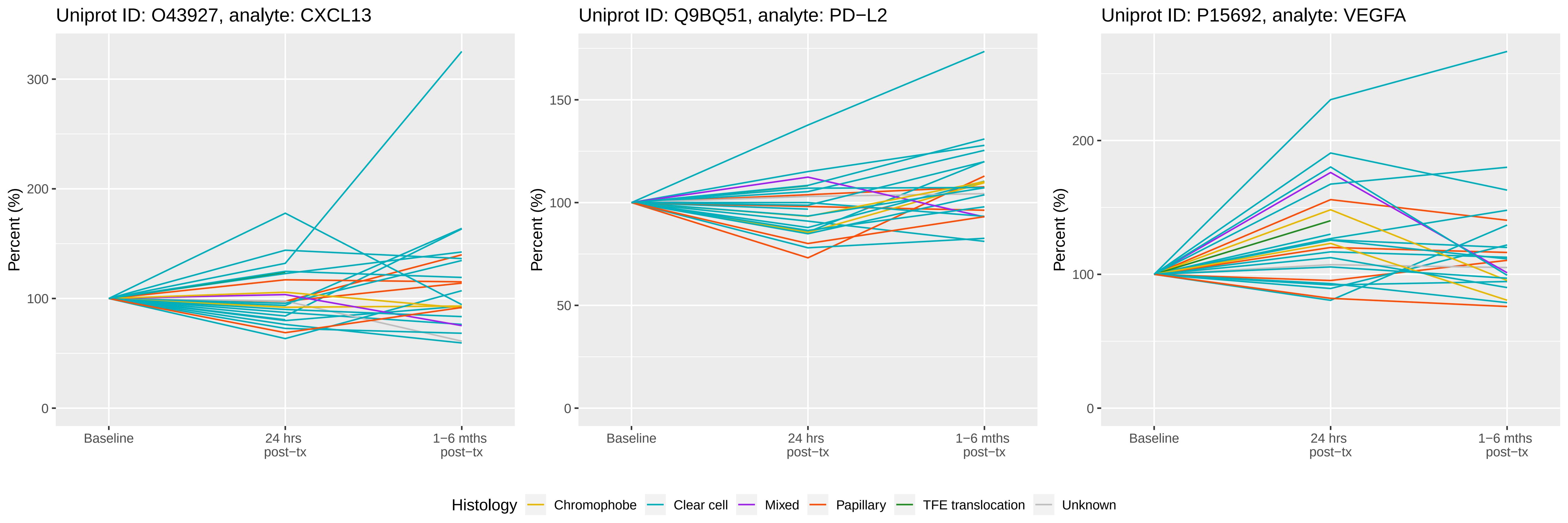
Uniprot ID: O43927, analyte: CXCL13
Uniprot ID: Q9BQ51, analyte: PD−L2
Uniprot ID: P15692, analyte: VEGFA
Percent (%)
Baseline
24 hrs post−tx
1−6 mths post−tx
Histology
Chromophobe
Clear cell
Mixed
Papillary
TFE translocation
Unknown

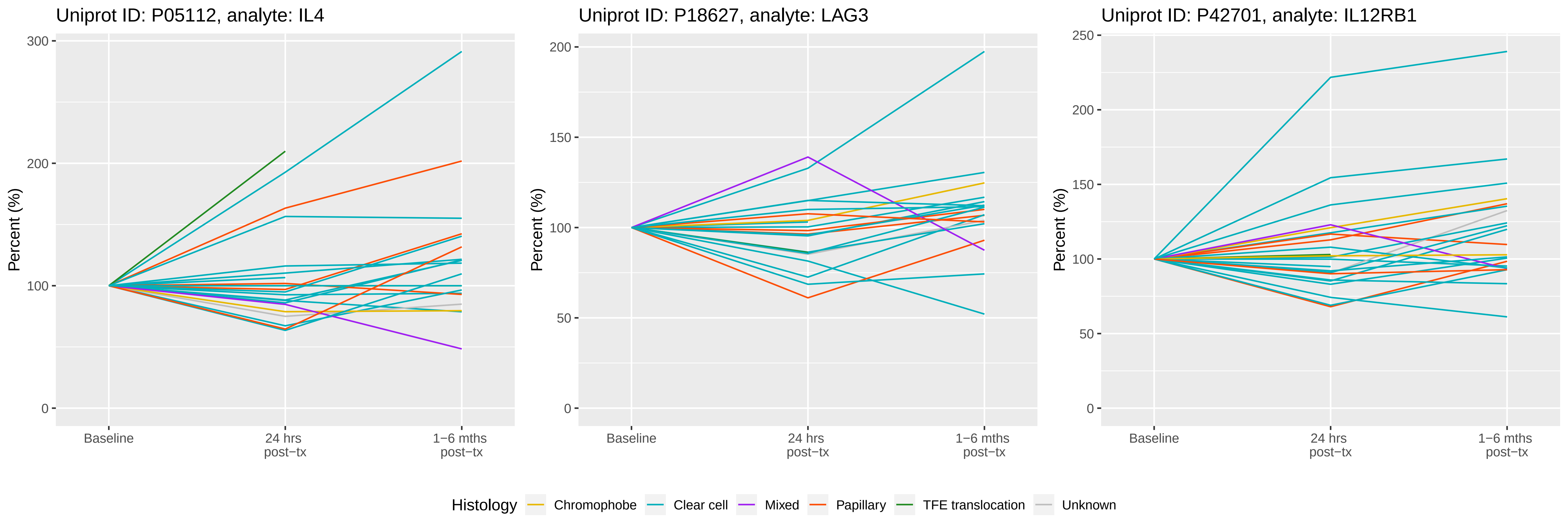
Uniprot ID: P05112, analyte: IL4
Uniprot ID: P18627, analyte: LAG3
Uniprot ID: P42701, analyte: IL12RB1
Percent (%)
0
100
200
300
0
50
100
150
200
0
50
100
150
200
250
Baseline
24 hrs post−tx
1−6 mths post−tx
Histology
Chromophobe
Clear cell
Mixed
Papillary
TFE translocation
Unknown

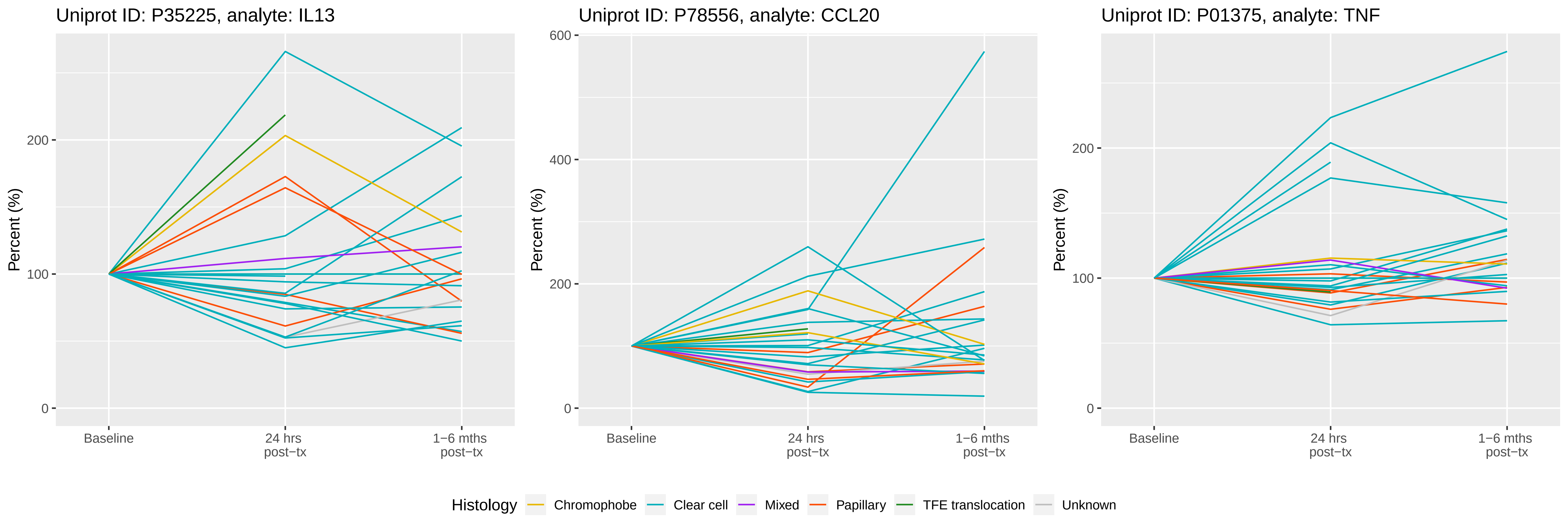

Uniprot ID: P35225, analyte: IL13
Uniprot ID: P78556, analyte: CCL20
Uniprot ID: P01375, analyte: TNF
Percent (%)
0
100
200
400
600
Baseline
24 hrs post−tx
1−6 mths post−tx
Histology
Chromophobe
Clear cell
Mixed
Papillary
TFE translocation
Unknown

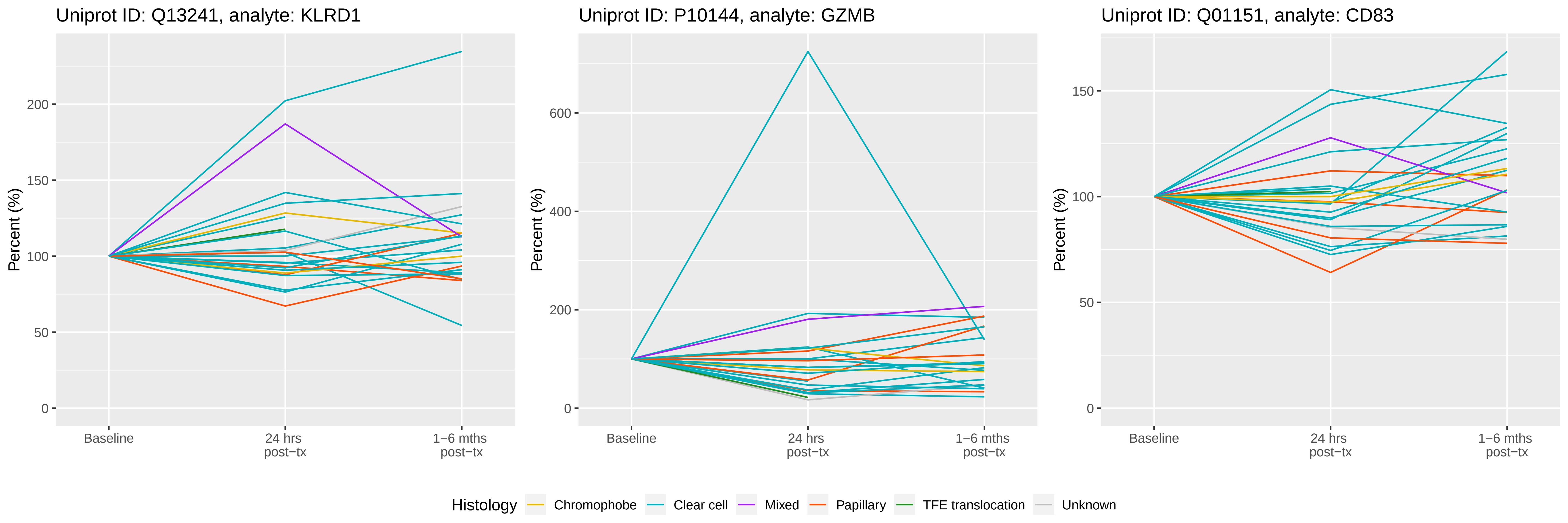

Uniprot ID: Q13241, analyte: KLRD1
Uniprot ID: P10144, analyte: GZMB
Uniprot ID: Q01151, analyte: CD83
Percent (%)
Baseline
24 hrs post−tx
1−6 mths post−tx
Histology
Chromophobe
Clear cell
Mixed
Papillary
TFE translocation
Unknown

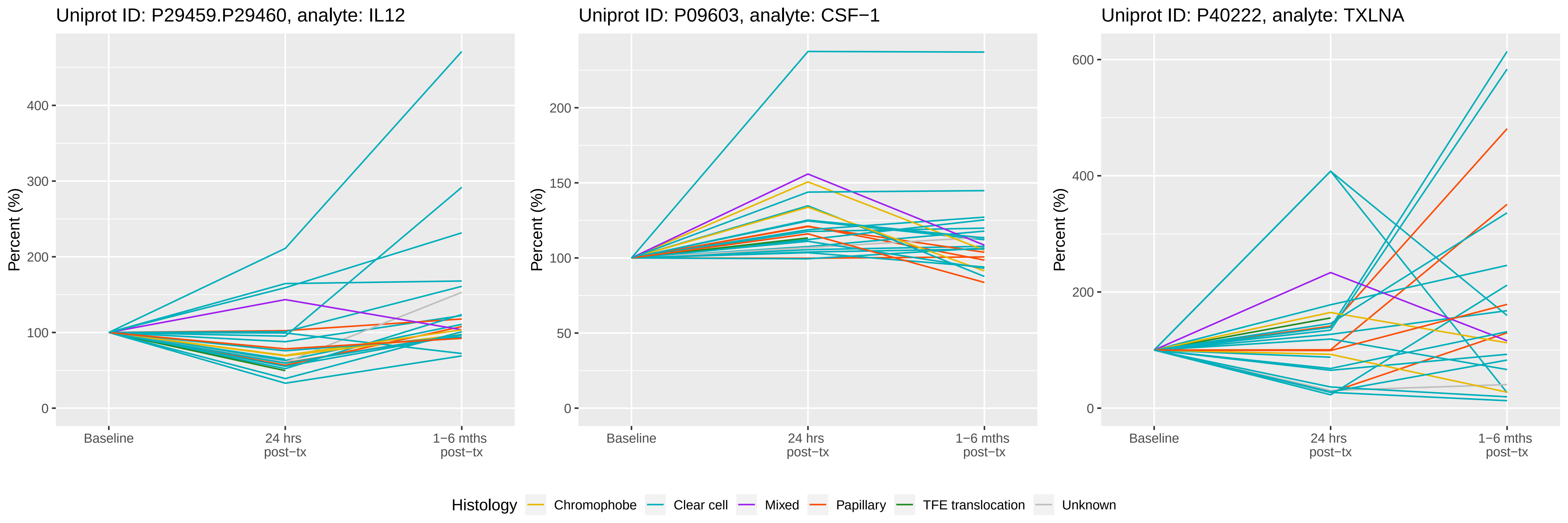

Uniprot ID: P29459.P29460, analyte: IL12
Uniprot ID: P09603, analyte: CSF−1
Uniprot ID: P40222, analyte: TXLNA
Percent (%)
Baseline
24 hrs post−tx
1−6 mths post−tx
Histology
Chromophobe
Clear cell
Mixed
Papillary
TFE translocation
Unknown

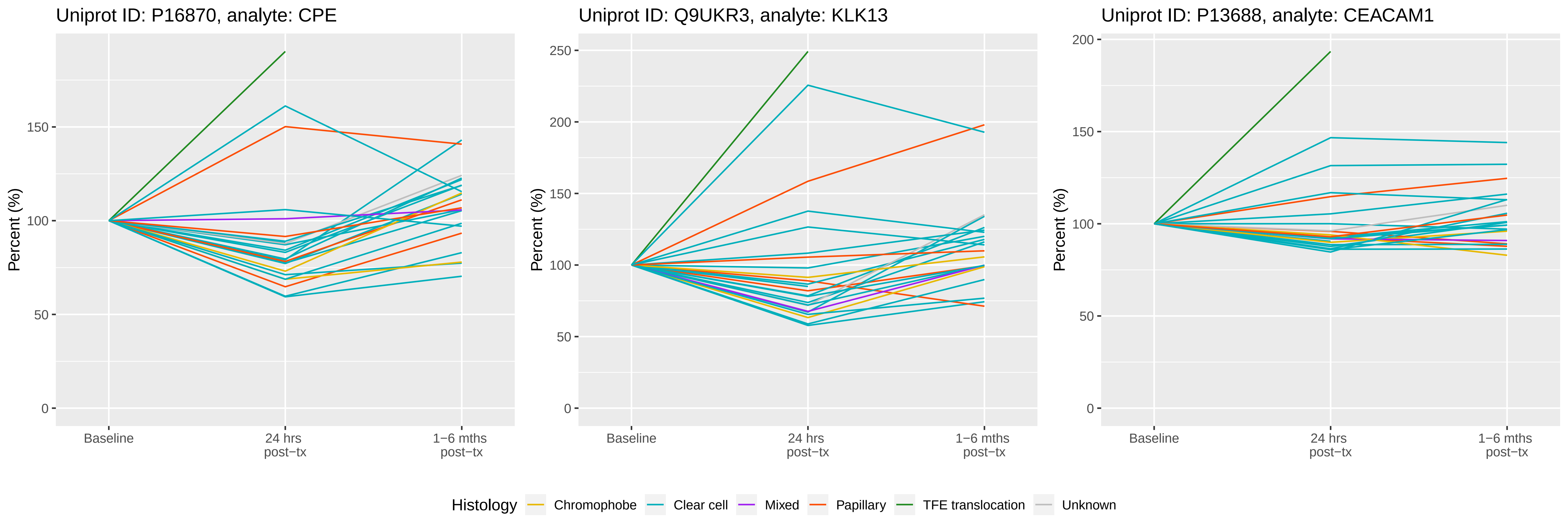
Uniprot ID: P16870, analyte: CPE
Uniprot ID: Q9UKR3, analyte: KLK13
Uniprot ID: P13688, analyte: CEACAM1
Percent (%)
0
50
100
150
200
250
Baseline
24 hrs post−tx
1−6 mths post−tx
Histology
Chromophobe
Clear cell
Mixed
Papillary
TFE translocation
Unknown

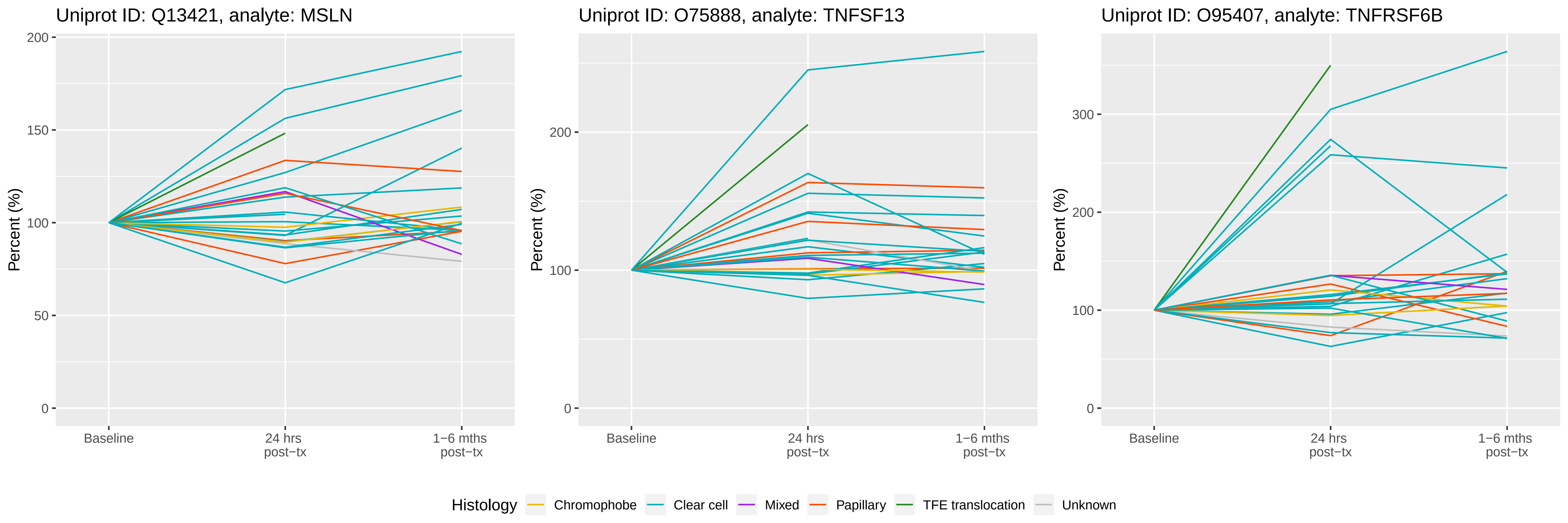

Uniprot ID: Q13421, analyte: MSLN
Uniprot ID: O75888, analyte: TNFSF13
Uniprot ID: O95407, analyte: TNFRSF6B
Percent (%)
0
50
100
150
200
300
Baseline
24 hrs post−tx
1−6 mths post−tx
Histology
Chromophobe
Clear cell
Mixed
Papillary
TFE translocation
Unknown

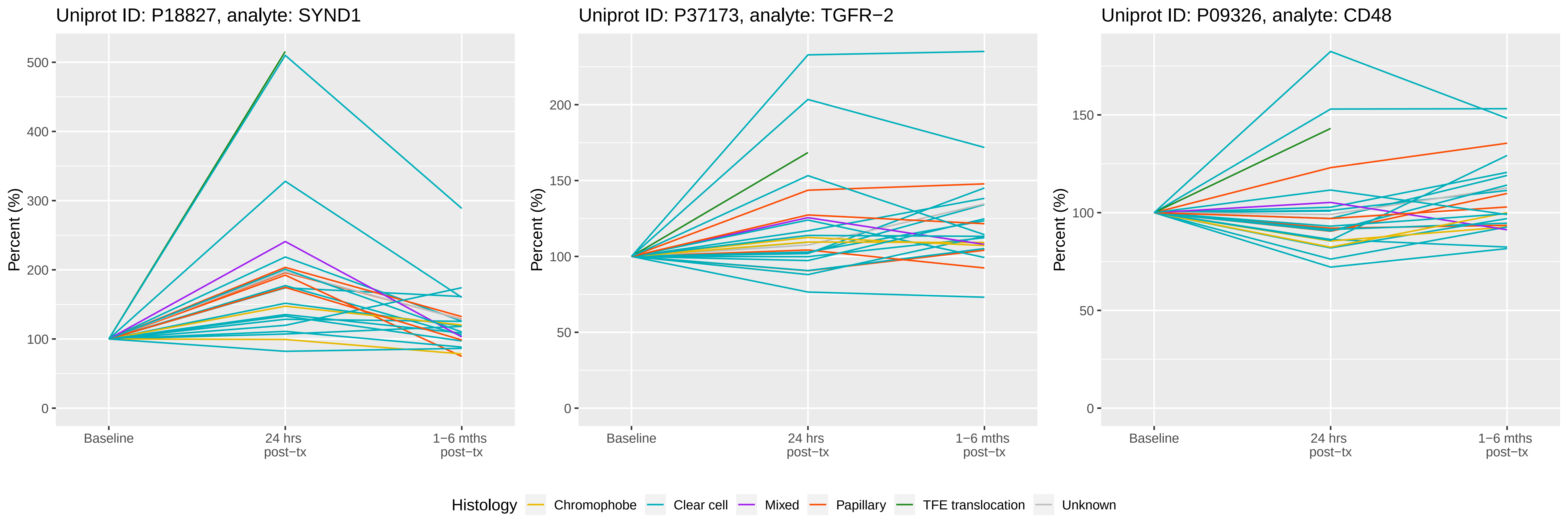

Uniprot ID: P18827, analyte: SYND1
Uniprot ID: P37173, analyte: TGFR−2
Uniprot ID: P09326, analyte: CD48
Percent (%)
0
100
200
300
400
500
50
150
Baseline
24 hrs post−tx
1−6 mths post−tx
Histology
Chromophobe
Clear cell
Mixed
Papillary
TFE translocation
Unknown

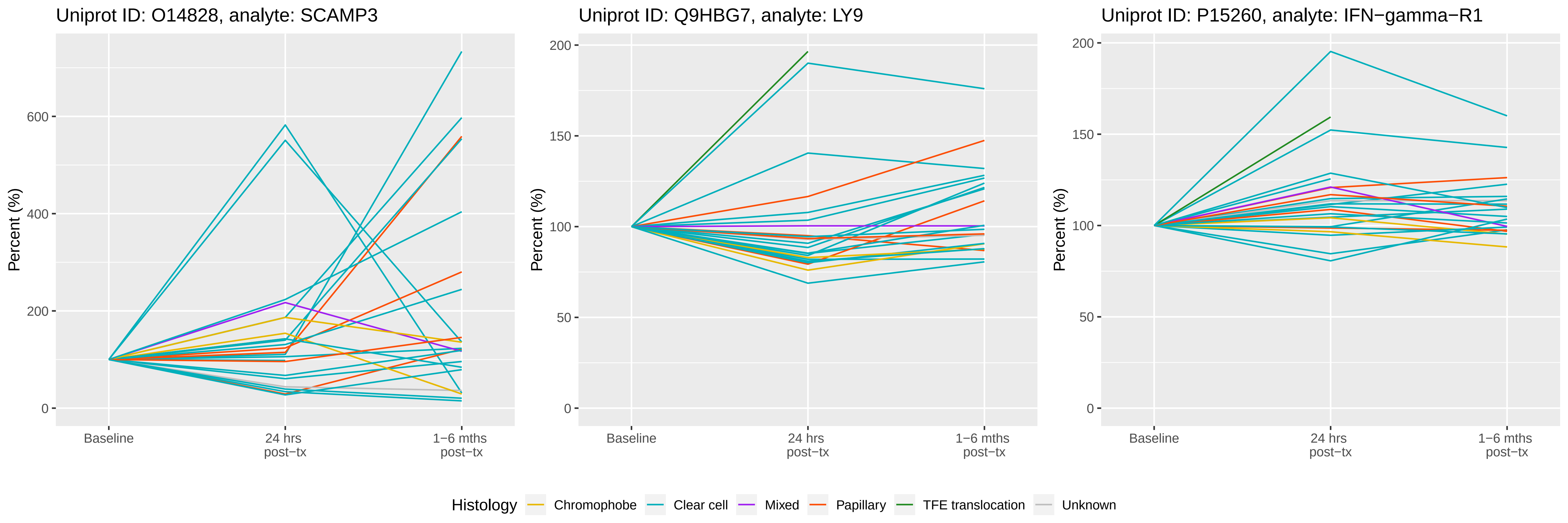
Uniprot ID: O14828, analyte: SCAMP3
Uniprot ID: Q9HBG7, analyte: LY9
Uniprot ID: P15260, analyte: IFN−gamma−R1
Percent (%)
Baseline
24 hrs post−tx
1−6 mths post−tx
0
200
400
600
50
100
150
200
Histology
Chromophobe
Clear cell
Mixed
Papillary
TFE translocation
Unknown

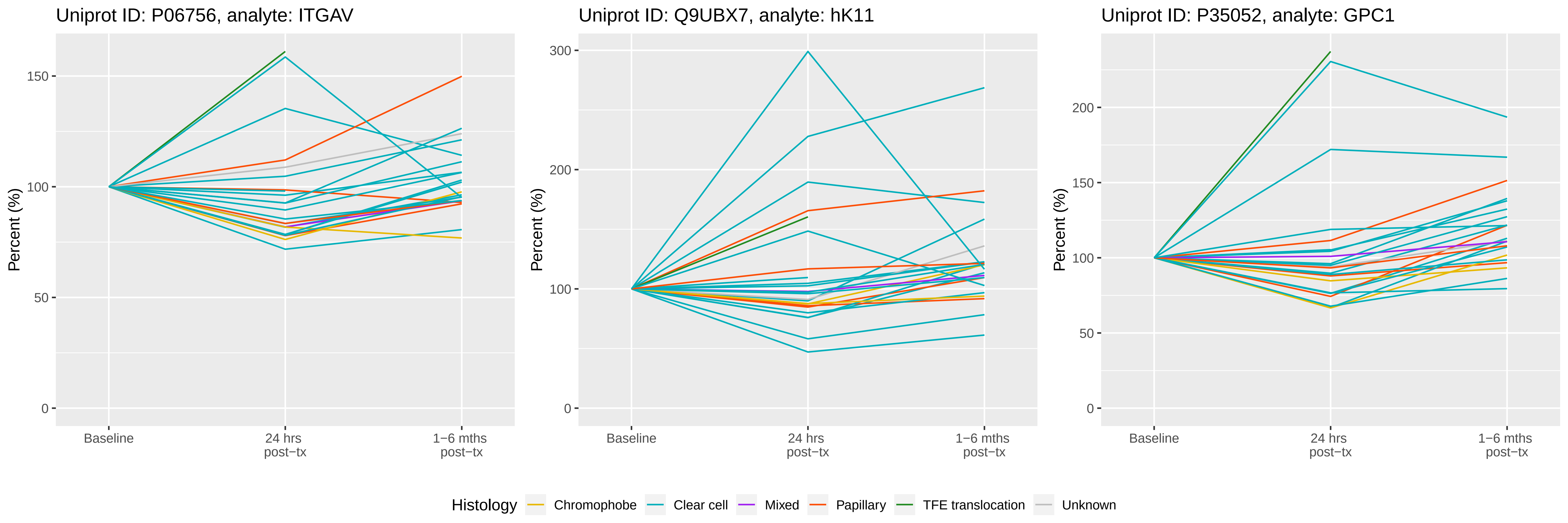

Uniprot ID: P06756, analyte: ITGAV
Uniprot ID: Q9UBX7, analyte: hK11
Uniprot ID: P35052, analyte: GPC1
Percent (%)
Baseline
24 hrs post−tx
1−6 mths post−tx
Histology
Chromophobe
Clear cell
Mixed
Papillary
TFE translocation
Unknown

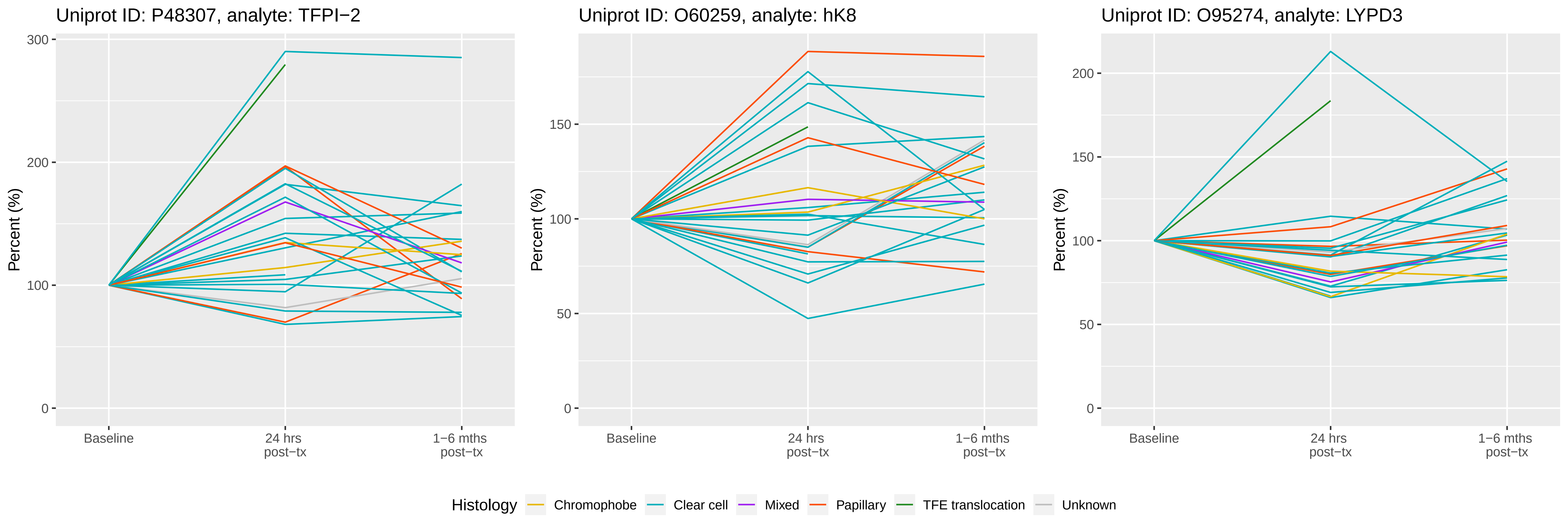
Uniprot ID: P48307, analyte: TFPI−2
Uniprot ID: O60259, analyte: hK8
Uniprot ID: O95274, analyte: LYPD3
Percent (%)
300
200
100
0
150
50
Baseline
24 hrs post−tx
1−6 mths post−tx
Histology
Chromophobe
Clear cell
Mixed
Papillary
TFE translocation
Unknown

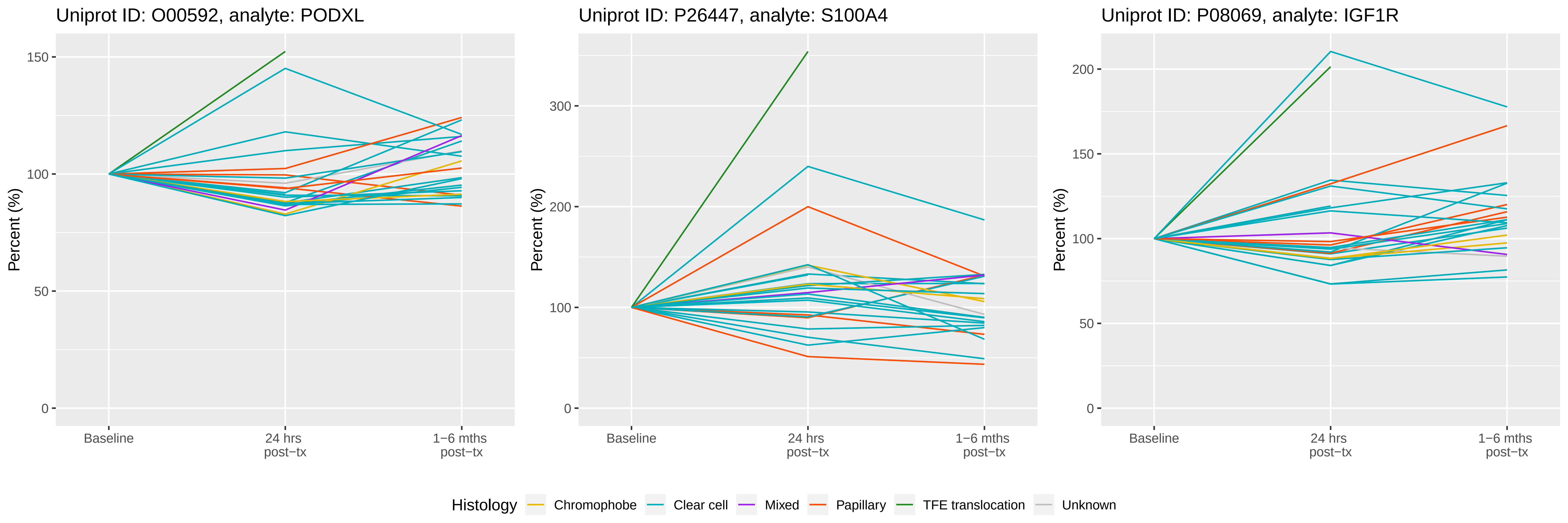

Uniprot ID: O00592, analyte: PODXL
Uniprot ID: P26447, analyte: S100A4
Uniprot ID: P08069, analyte: IGF1R
Percent (%)
Baseline
24 hrs post−tx
1−6 mths post−tx
Histology
Chromophobe
Clear cell
Mixed
Papillary
TFE translocation
Unknown

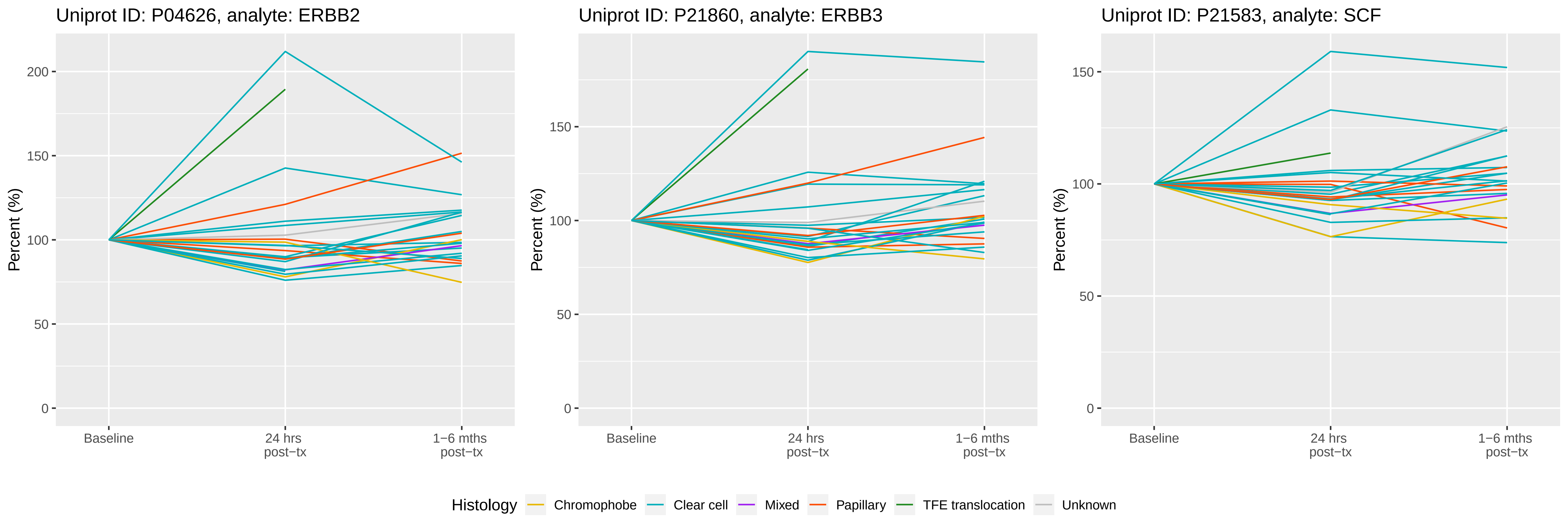
Uniprot ID: P04626, analyte: ERBB2
Uniprot ID: P21860, analyte: ERBB3
Uniprot ID: P21583, analyte: SCF
Percent (%)
Baseline
24 hrs post−tx
1−6 mths post−tx
Histology
Chromophobe
Clear cell
Mixed
Papillary
TFE translocation
Unknown

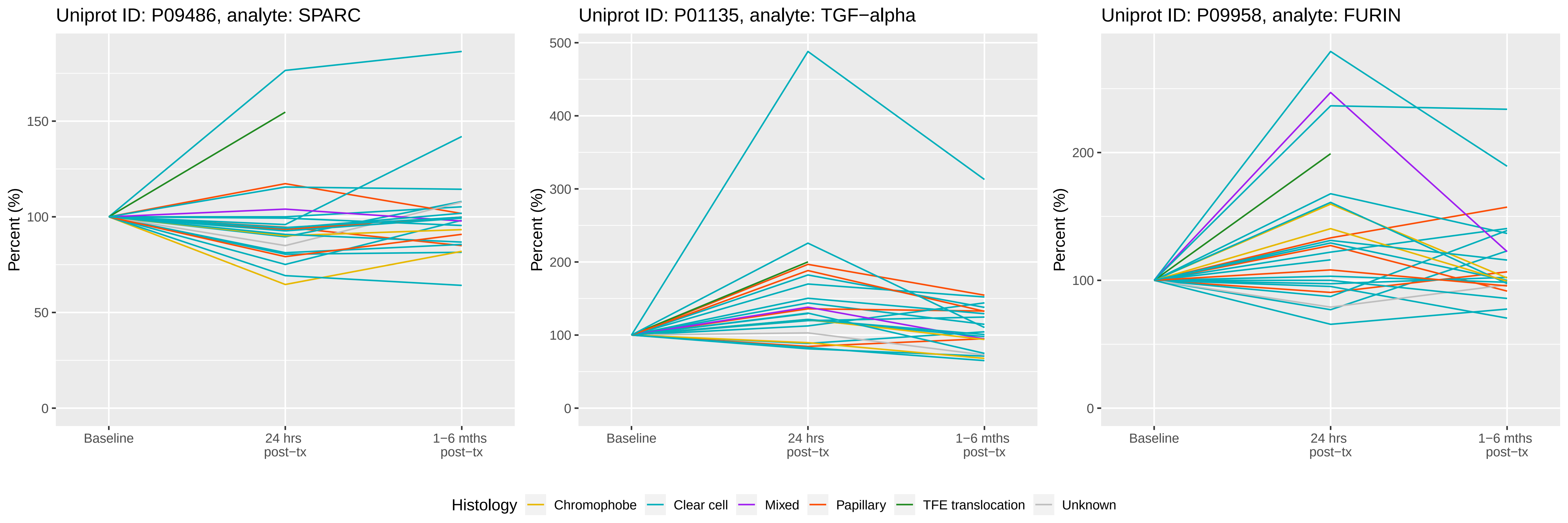
Uniprot ID: P09486, analyte: SPARC
Uniprot ID: P01135, analyte: TGF−alpha
Uniprot ID: P09958, analyte: FURIN
Percent (%)
0
50
100
150
0
100
200
300
400
500
0
100
200
Baseline
24 hrs post−tx
1−6 mths post−tx
Histology
Chromophobe
Clear cell
Mixed
Papillary
TFE translocation
Unknown

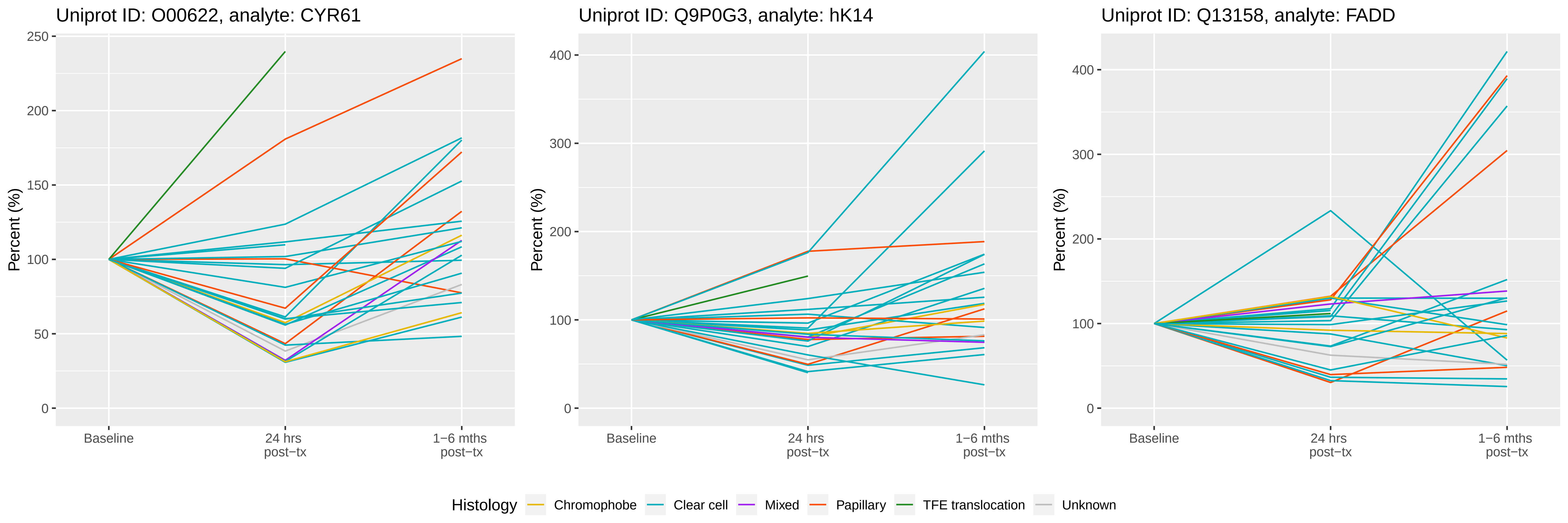

Uniprot ID: O00622, analyte: CYR61
Uniprot ID: Q9P0G3, analyte: hK14
Uniprot ID: Q13158, analyte: FADD
Percent (%)
0
50
100
150
200
250
0
100
200
300
400
Baseline
24 hrs post−tx
1−6 mths post−tx
Histology
Chromophobe
Clear cell
Mixed
Papillary
TFE translocation
Unknown

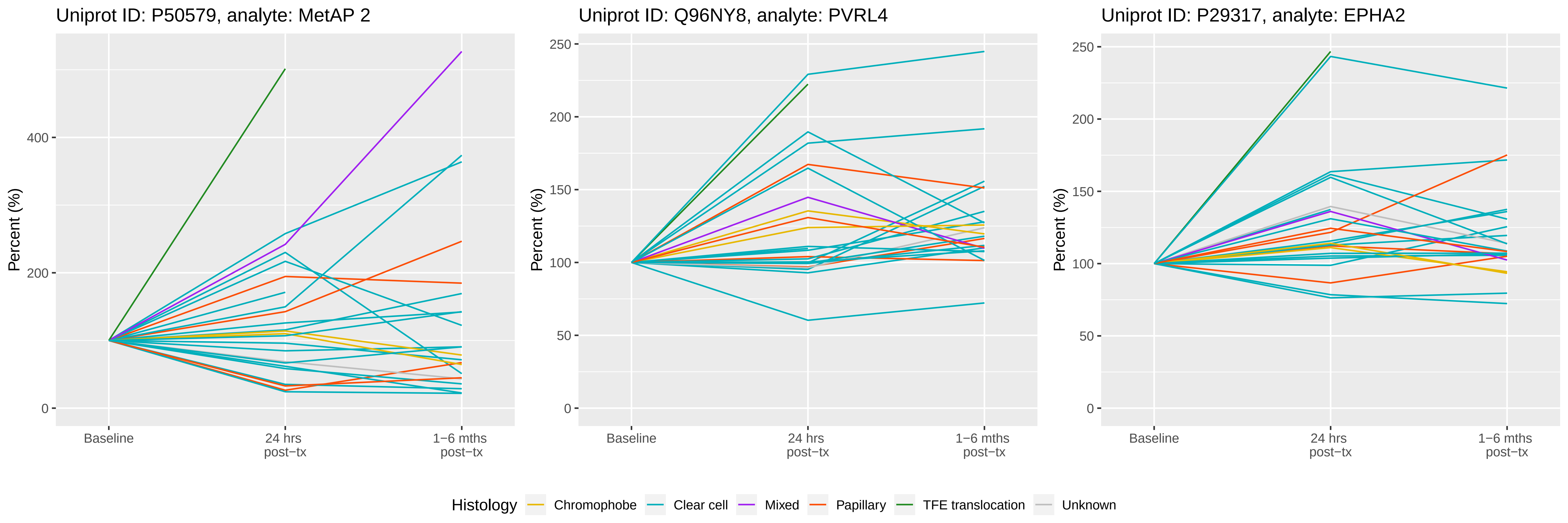
Uniprot ID: P50579, analyte: MetAP 2
Uniprot ID: Q96NY8, analyte: PVRL4
Uniprot ID: P29317, analyte: EPHA2
Percent (%)
0
200
400
0
50
100
150
200
250
Baseline
24 hrs post−tx
1−6 mths post−tx
Histology
Chromophobe
Clear cell
Mixed
Papillary
TFE translocation
Unknown

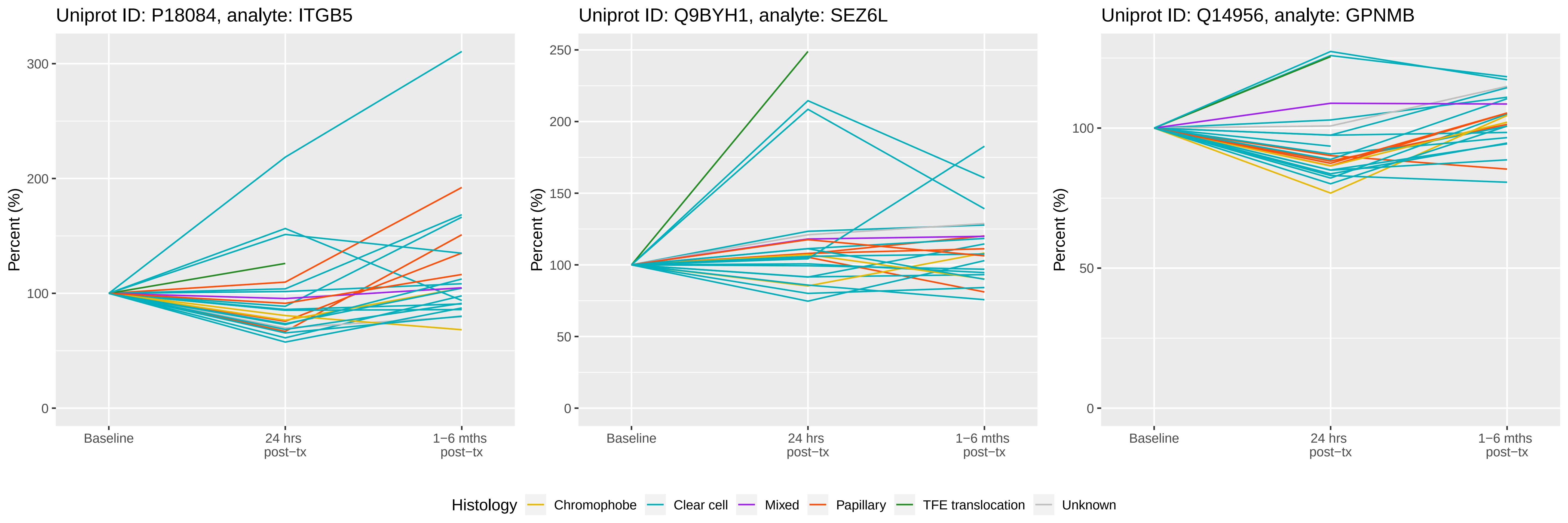

Uniprot ID: P18084, analyte: ITGB5
Uniprot ID: Q9BYH1, analyte: SEZ6L
Uniprot ID: Q14956, analyte: GPNMB
Percent (%)
0
100
200
300
0
50
100
150
200
250
0
50
100
Baseline
24 hrs post−tx
1−6 mths post−tx
Histology
Chromophobe
Clear cell
Mixed
Papillary
TFE translocation
Unknown

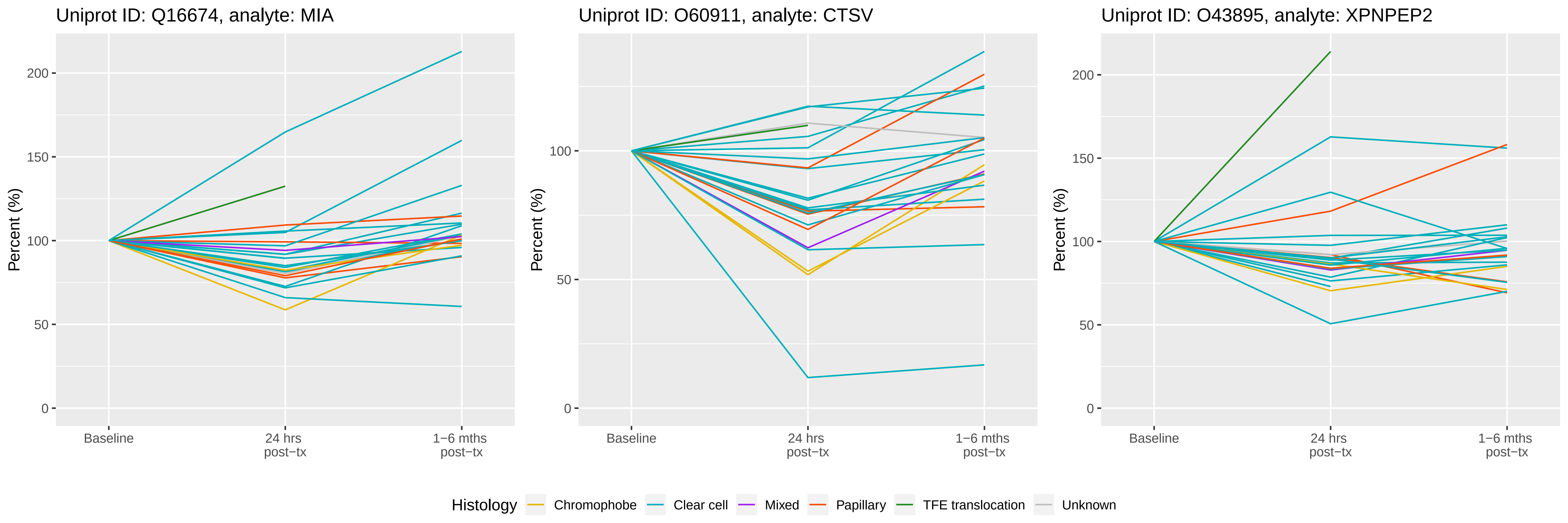
Uniprot ID: Q16674, analyte: MIA
Uniprot ID: O60911, analyte: CTSV
Uniprot ID: O43895, analyte: XPNPEP2
Percent (%)
0
50
100
150
200
Baseline
24 hrs post−tx
1−6 mths post−tx
Histology
Chromophobe
Clear cell
Mixed
Papillary
TFE translocation
Unknown

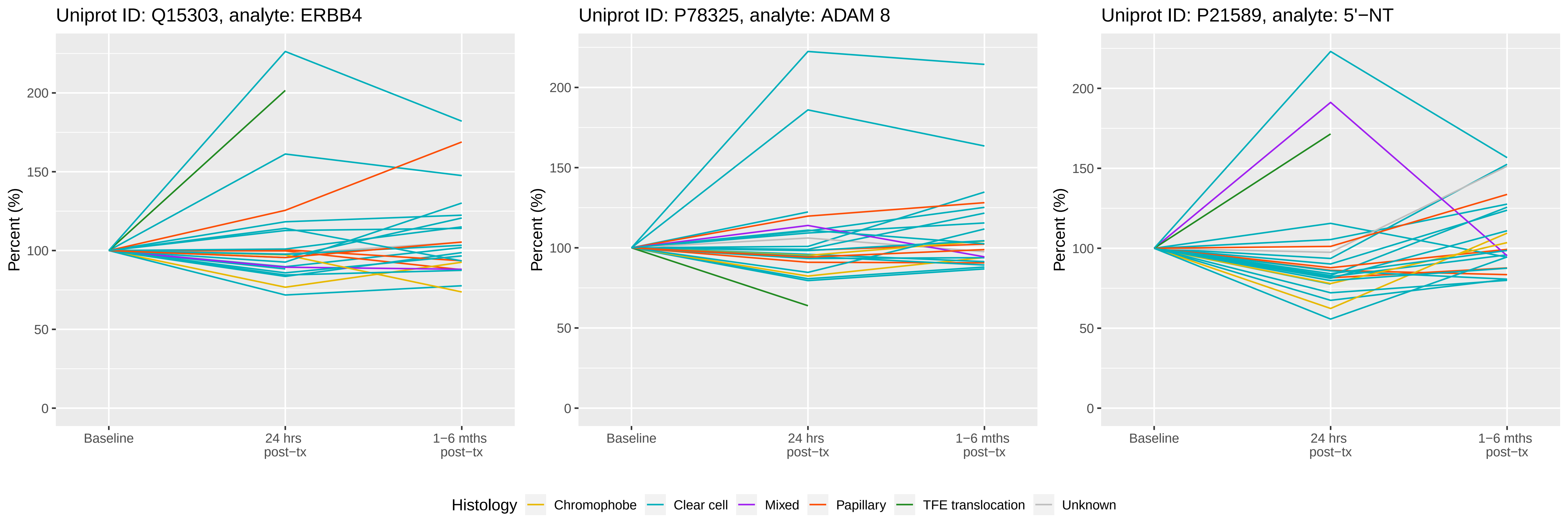
Uniprot ID: Q15303, analyte: ERBB4
Uniprot ID: P78325, analyte: ADAM 8
Uniprot ID: P21589, analyte: 5'−NT
Percent (%)
0
50
100
150
200
Baseline
24 hrs post−tx
1−6 mths post−tx
Histology
Chromophobe
Clear cell
Mixed
Papillary
TFE translocation
Unknown

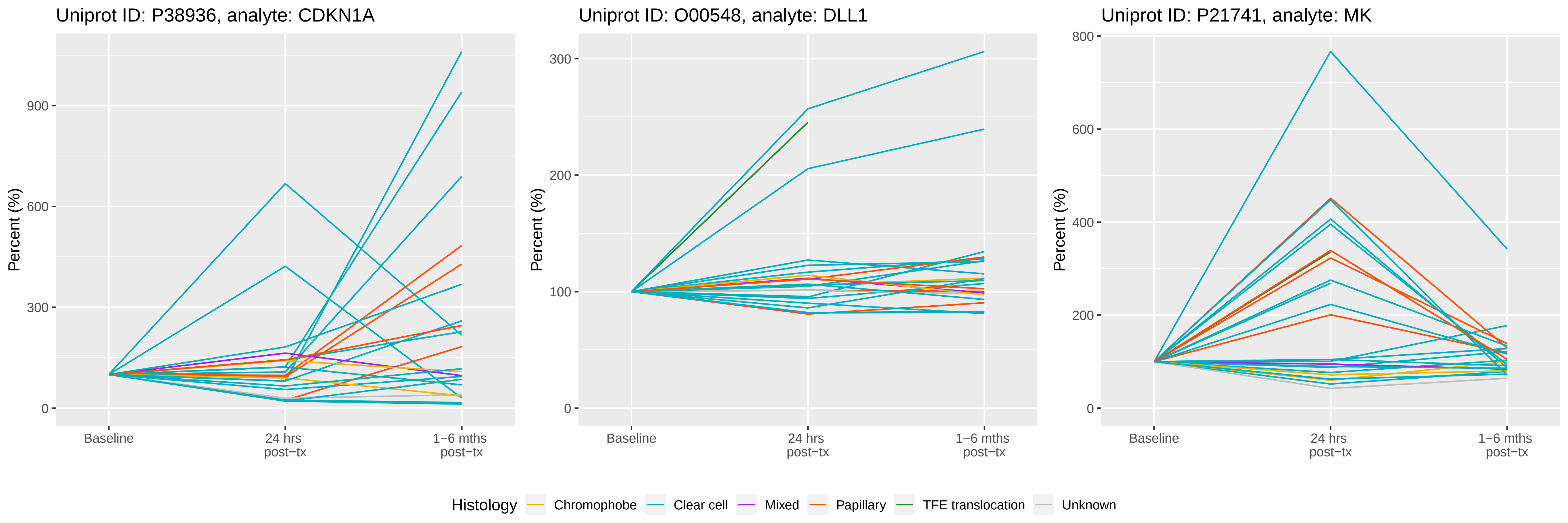
Uniprot ID: P38936, analyte: CDKN1A
Uniprot ID: O00548, analyte: DLL1
Uniprot ID: P21741, analyte: MK
Percent (%)
Baseline
24 hrs post−tx
1−6 mths post−tx
Histology
Chromophobe
Clear cell
Mixed
Papillary
TFE translocation
Unknown

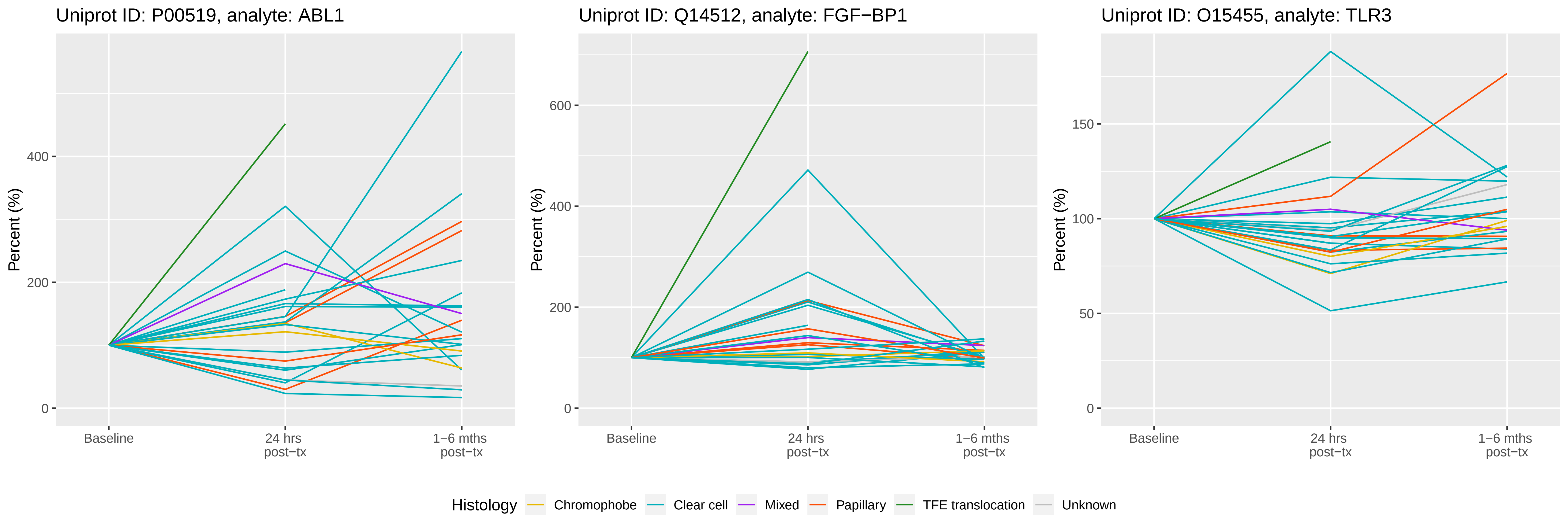

Uniprot ID: P00519, analyte: ABL1
Uniprot ID: Q14512, analyte: FGF−BP1
Uniprot ID: O15455, analyte: TLR3
Percent (%)
Baseline
24 hrs post−tx
1−6 mths post−tx
Histology
Chromophobe
Clear cell
Mixed
Papillary
TFE translocation
Unknown

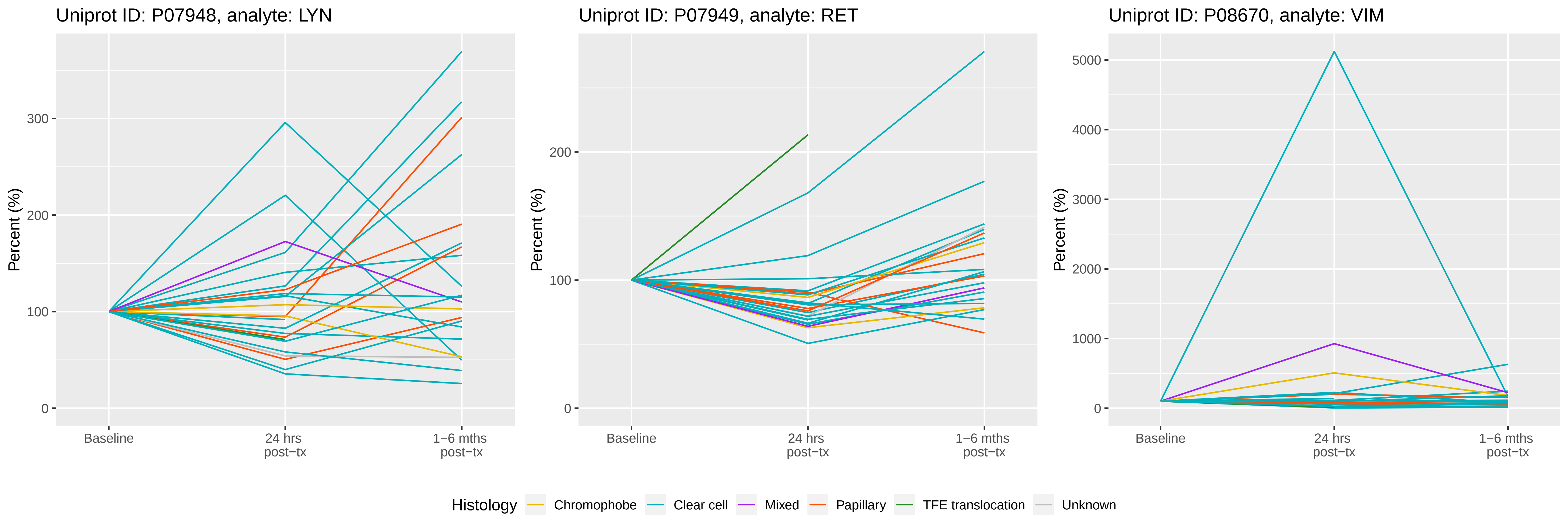
Uniprot ID: P07948, analyte: LYN
Uniprot ID: P07949, analyte: RET
Uniprot ID: P08670, analyte: VIM
Percent (%)
0
100
200
300
1000
2000
3000
4000
5000
Baseline
24 hrs post−tx
1−6 mths post−tx
Histology
Chromophobe
Clear cell
Mixed
Papillary
TFE translocation
Unknown

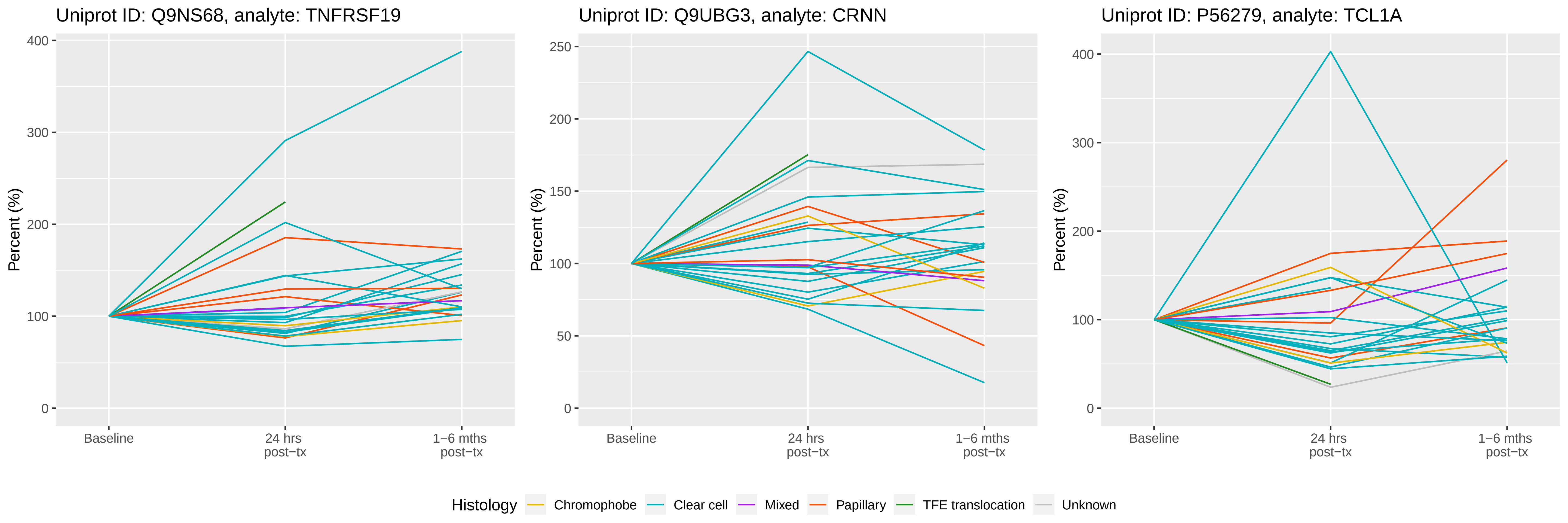

Uniprot ID: Q9NS68, analyte: TNFRSF19
Uniprot ID: Q9UBG3, analyte: CRNN
Uniprot ID: P56279, analyte: TCL1A
Percent (%)
0
100
200
300
400
0
50
100
150
200
250
Baseline
24 hrs post−tx
1−6 mths post−tx
Histology
Chromophobe
Clear cell
Mixed
Papillary
TFE translocation
Unknown

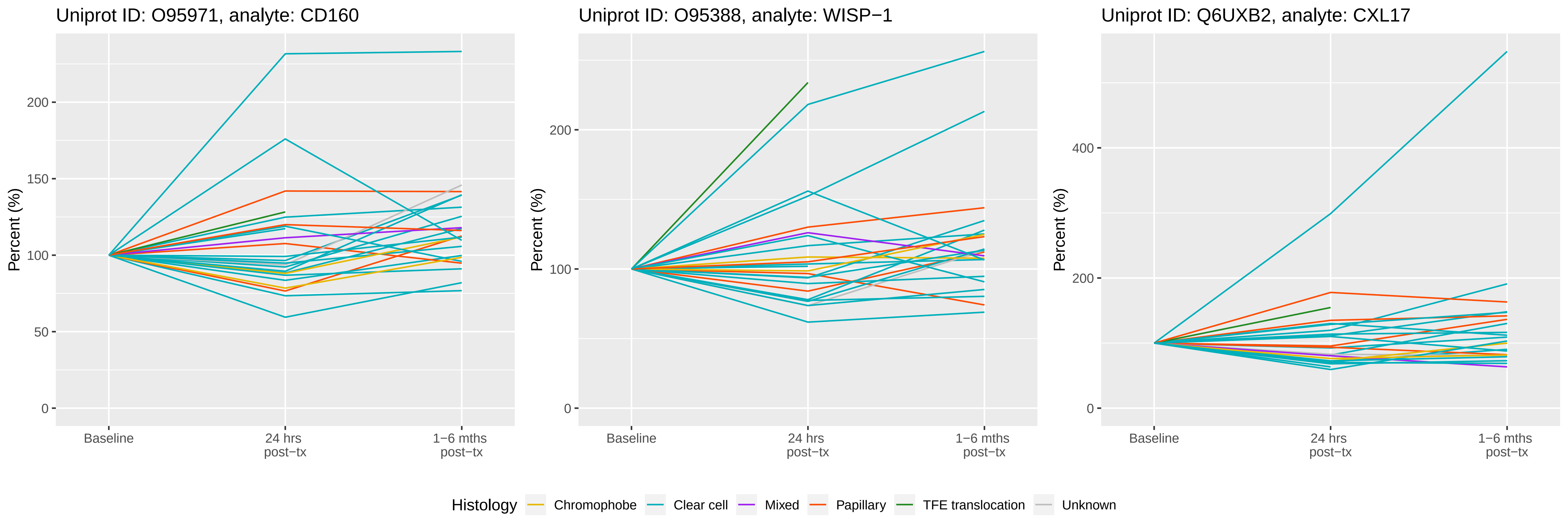

Uniprot ID: O95971, analyte: CD160
Uniprot ID: O95388, analyte: WISP−1
Uniprot ID: Q6UXB2, analyte: CXL17
Percent (%)
0
50
100
150
200
400
Baseline
24 hrs post−tx
1−6 mths post−tx
Histology
Chromophobe
Clear cell
Mixed
Papillary
TFE translocation
Unknown

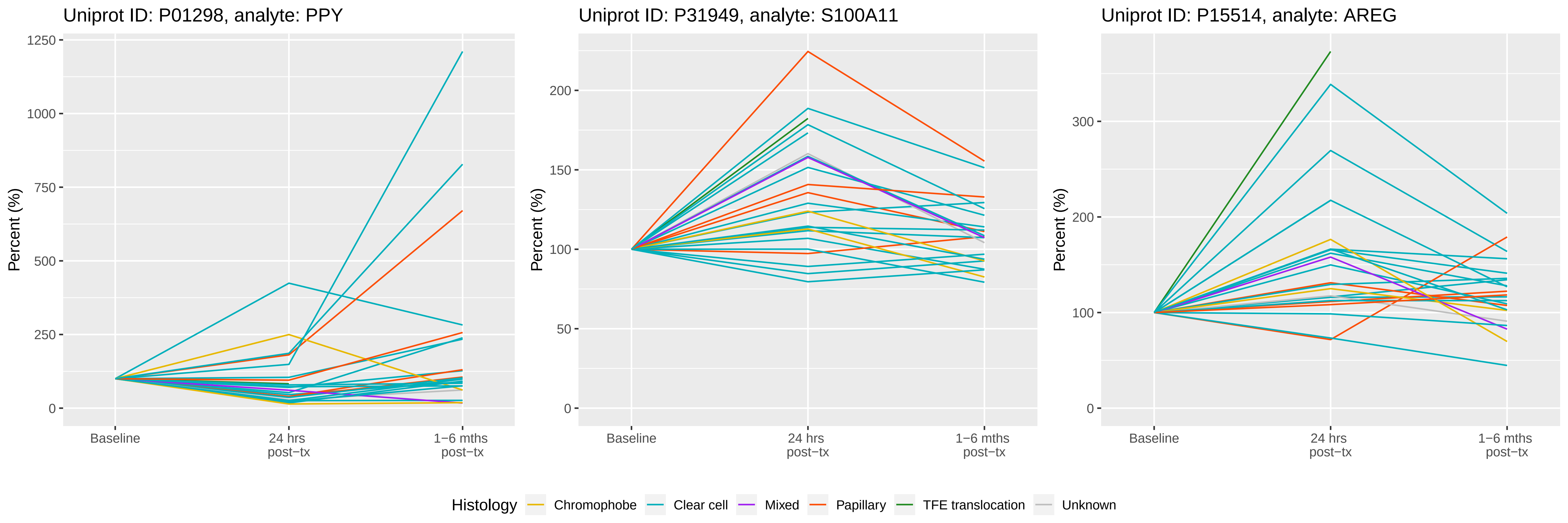
Uniprot ID: P01298, analyte: PPY
Uniprot ID: P31949, analyte: S100A11
Uniprot ID: P15514, analyte: AREG
Percent (%)
1250
1000
750
500
250
0
200
150
100
50
300
Baseline
24 hrs post−tx
1−6 mths post−tx
Histology
Chromophobe
Clear cell
Mixed
Papillary
TFE translocation
Unknown

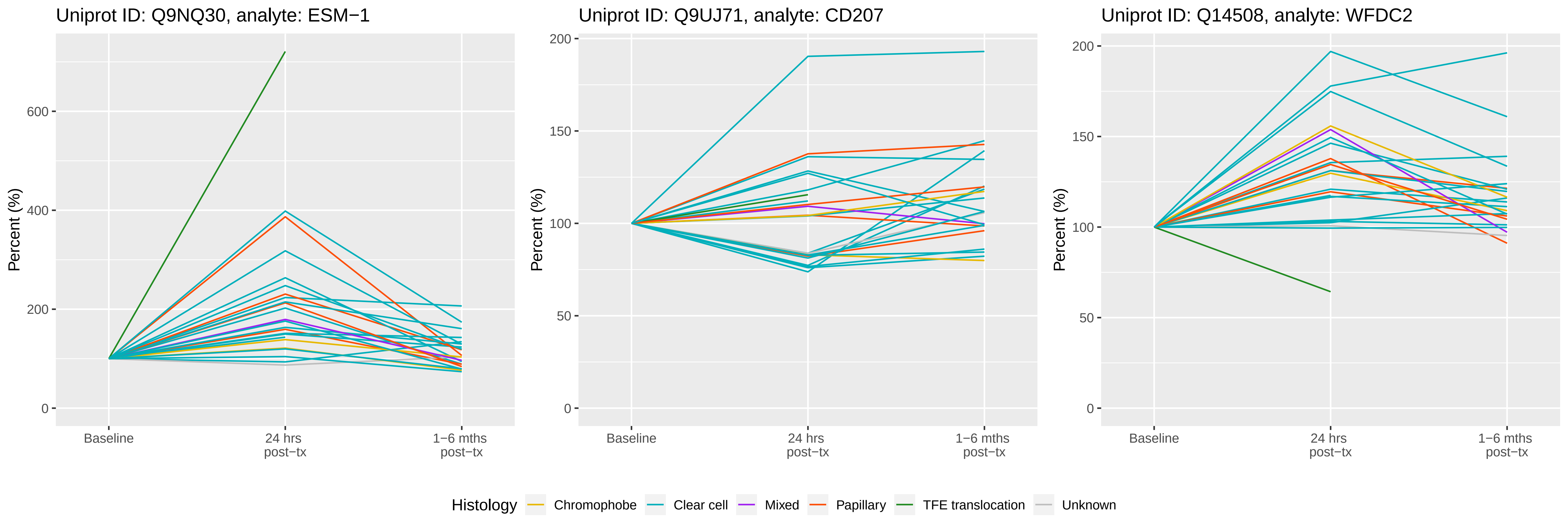

Uniprot ID: Q9NQ30, analyte: ESM−1
Uniprot ID: Q9UJ71, analyte: CD207
Uniprot ID: Q14508, analyte: WFDC2
Percent (%)
0
200
400
600
0
50
100
150
200
Baseline
24 hrs post−tx
1−6 mths post−tx
Histology
Chromophobe
Clear cell
Mixed
Papillary
TFE translocation
Unknown

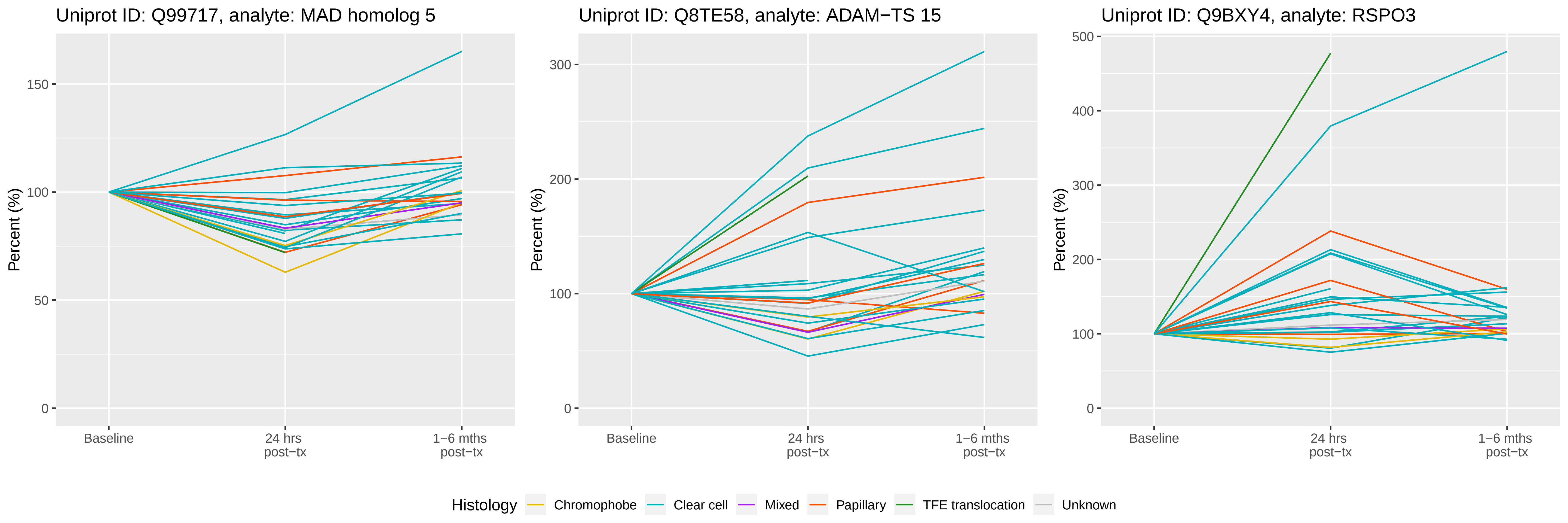

Uniprot ID: Q99717, analyte: MAD homolog 5
Uniprot ID: Q8TE58, analyte: ADAM−TS 15
Uniprot ID: Q9BXY4, analyte: RSPO3
Percent (%)
Baseline
24 hrs post−tx
1−6 mths post−tx
Histology
Chromophobe
Clear cell
Mixed
Papillary
TFE translocation
Unknown

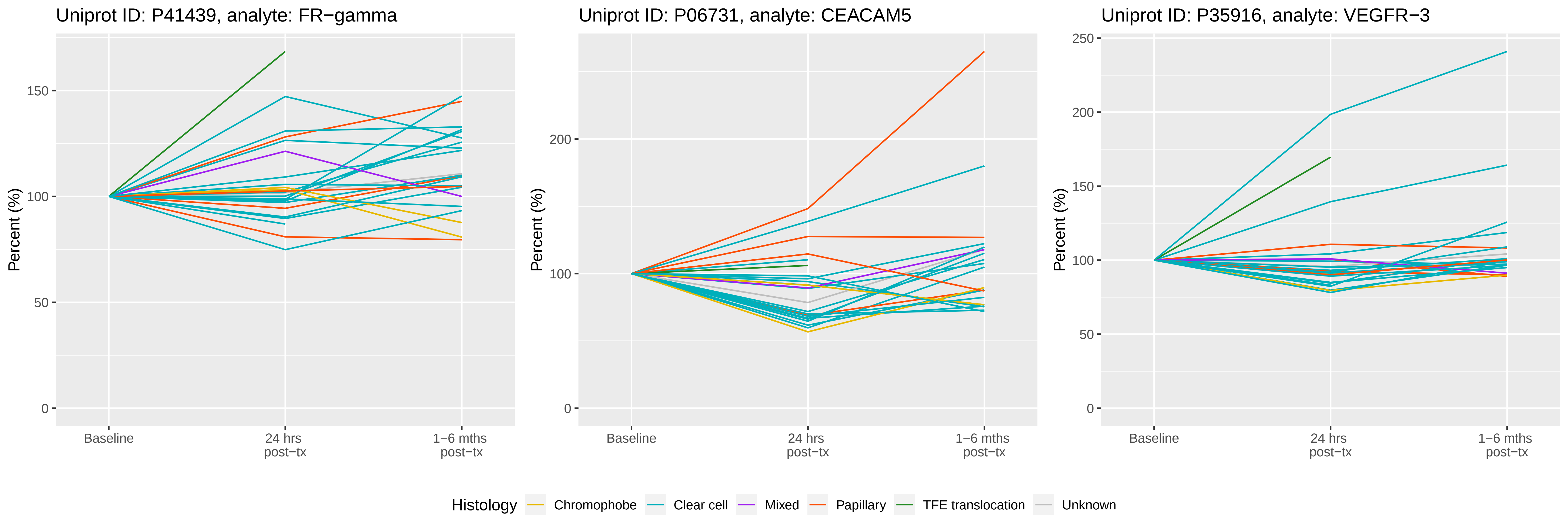
Uniprot ID: P41439, analyte: FR−gamma
Uniprot ID: P06731, analyte: CEACAM5
Uniprot ID: P35916, analyte: VEGFR−3
Percent (%)
0
50
100
150
200
250
Baseline
24 hrs post−tx
1−6 mths post−tx
Histology
Chromophobe
Clear cell
Mixed
Papillary
TFE translocation
Unknown

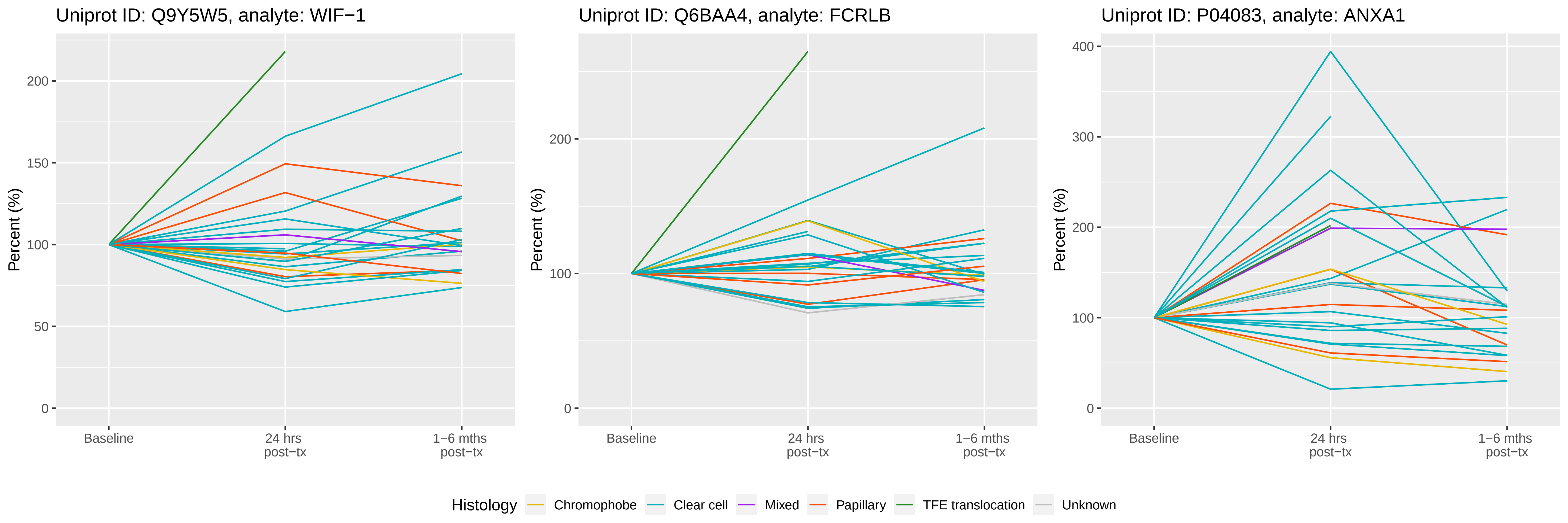

Uniprot ID: Q9Y5W5, analyte: WIF−1
Uniprot ID: Q6BAA4, analyte: FCRLB
Uniprot ID: P04083, analyte: ANXA1
Percent (%)
Baseline
24 hrs post−tx
1−6 mths post−tx
Histology
Chromophobe
Clear cell
Mixed
Papillary
TFE translocation
Unknown

Uniprot ID: P15328, analyte: FR−alpha

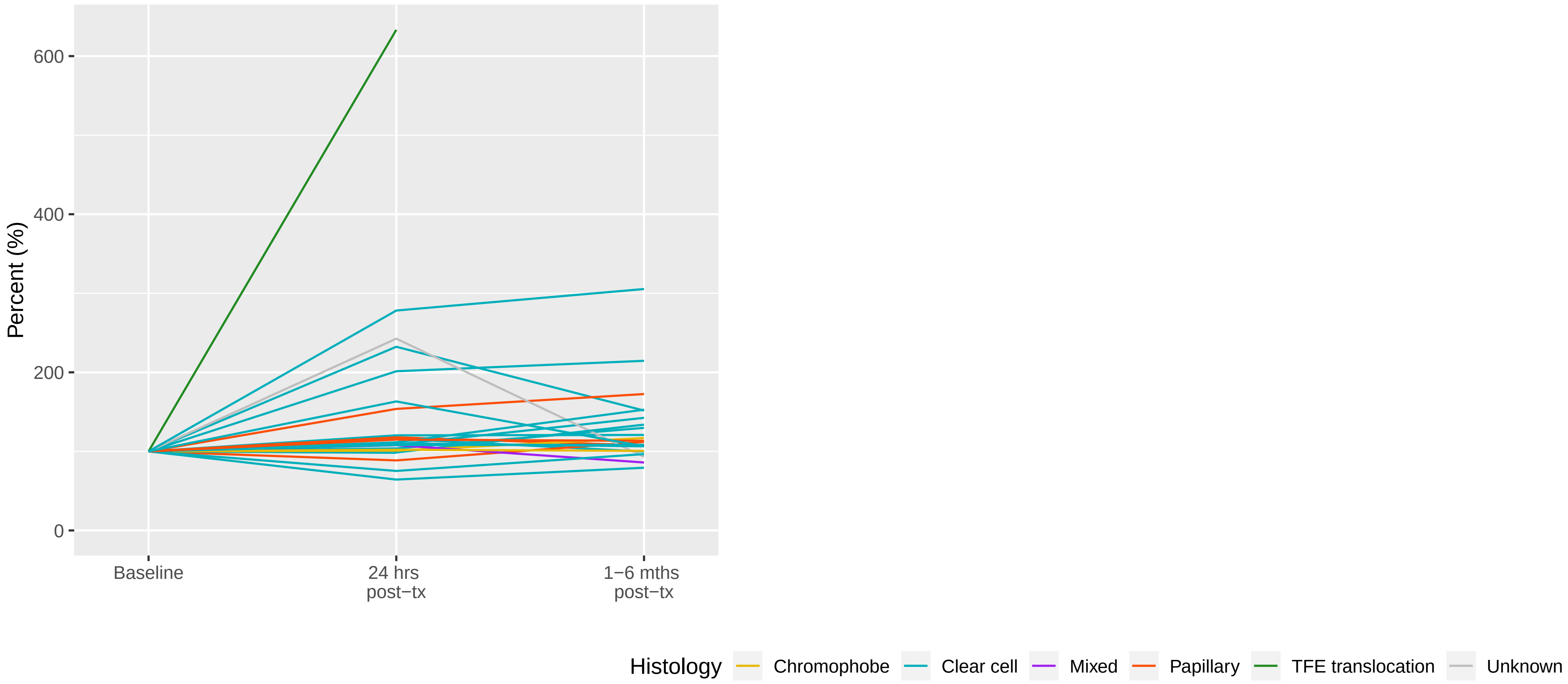

Supplement: Supplementary file 1 [file cancers-13-06037-s001.zip › Supplementary File/Figure S7.pdf]
