# Supplementary material for: An Exploratory Analysis of Changes in Circulating Plasma Protein Profiles Following Image-Guided Ablation of Renal Tumours Provides Evidence for Effects on Multiple Biological Processes
Source: Cancers (Basel). 2021 Nov 30;13(23):6037. doi: 10.3390/cancers13236037 (PMC8656737; doi:10.3390/cancers13236037)
Supplement: Supplementary file 1 [file cancers-13-06037-s001.zip › Supplementary File/Figure S8.pdf]

Figure S8. Longitudinal profiles of all plasma proteins on the Immunooncology panel, prior to and post-ablation. Results for each protein are shown as linear NPX values and are shown for patients receiving CRYO (n=25) with the three patients who developed acute complications post-CRYO highlighted. Dotted lines indicate the lower limit of quantitation.

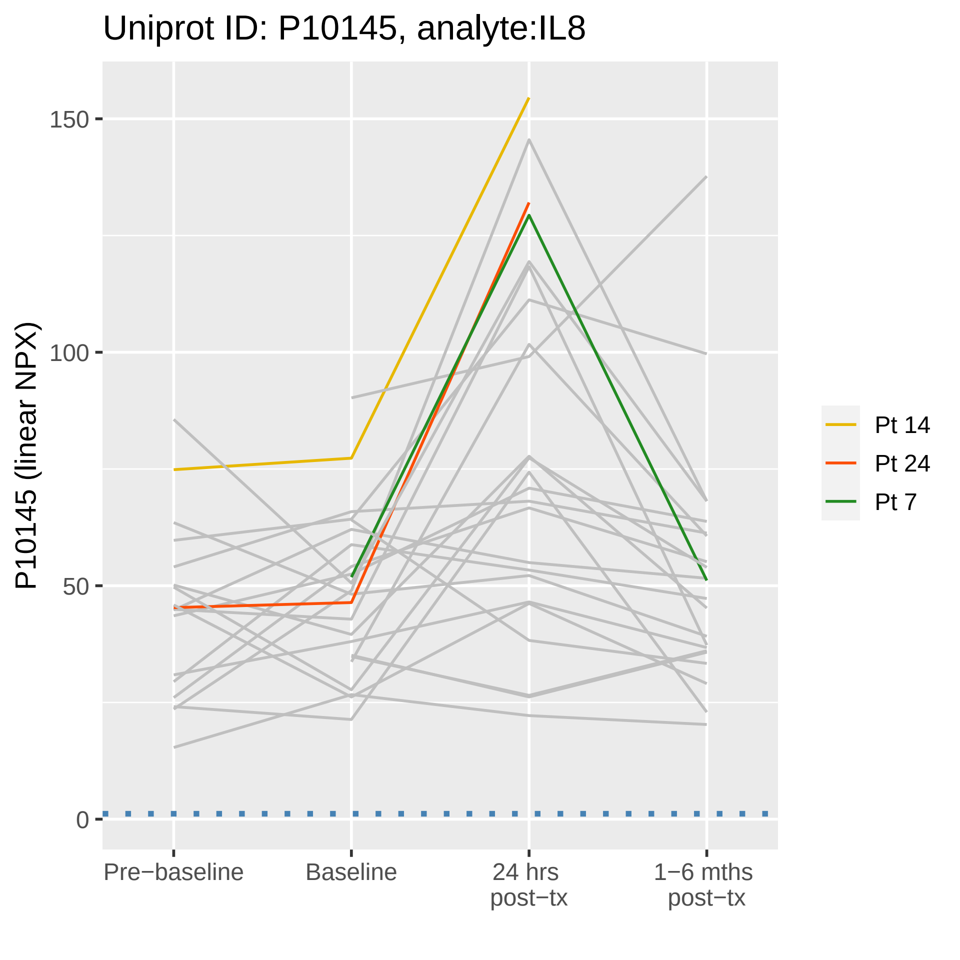

Uniprot ID: P10145, analyte:IL8
P10145 (linear NPX)
150
100
50
0
Pre−baseline
Baseline
24 hrs
post−tx
1−6 mths
post−tx
Pt 14
Pt 24
Pt 7

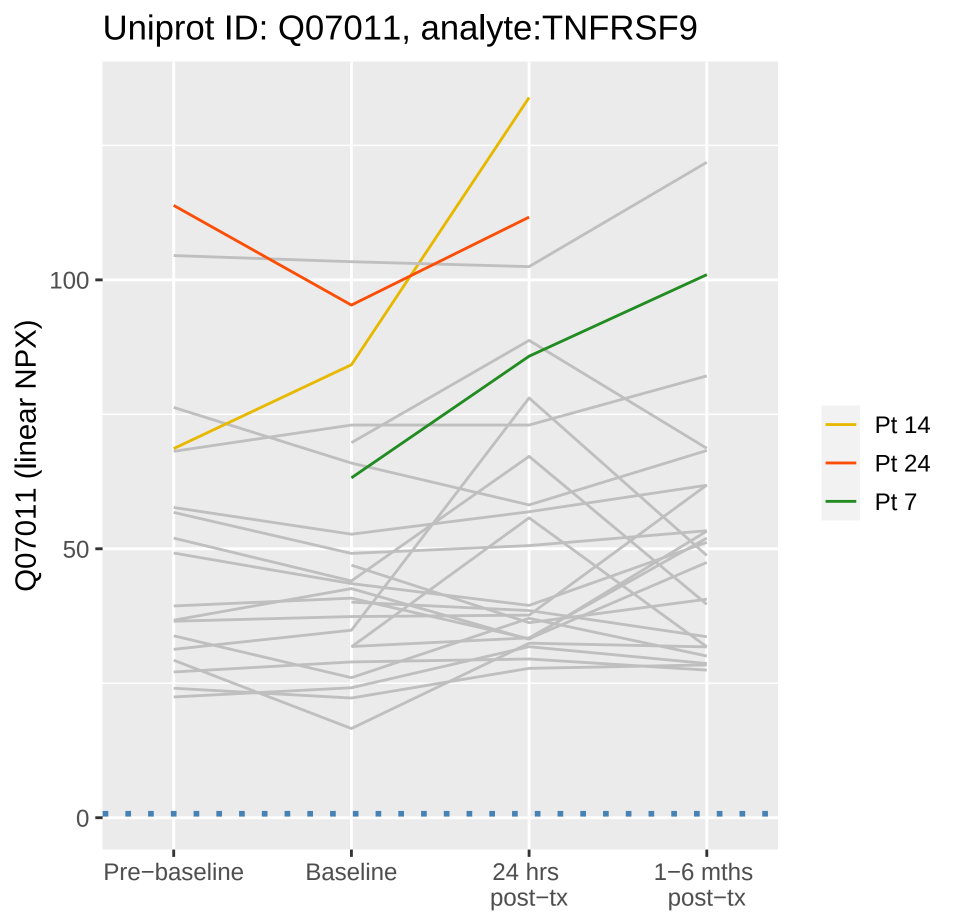
Uniprot ID: Q07011, analyte:TNFRSF9
Q07011 (linear NPX)
100
50
0
Pre−baseline
Baseline
24 hrs post−tx
1−6 mths post−tx
Pt 14
Pt 24
Pt 7

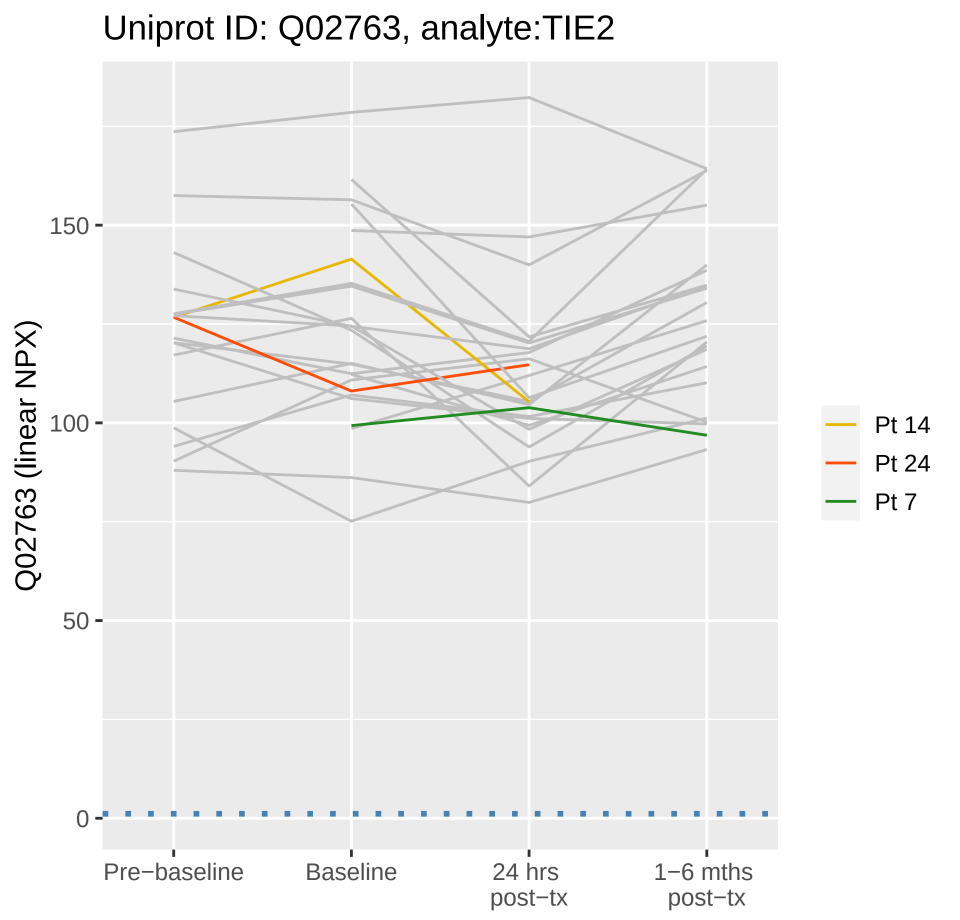
Uniprot ID: Q02763, analyte:TIE2
Q02763 (linear NPX)
150
100
50
0
Pre−baseline
Baseline
24 hrs post−tx
1−6 mths post−tx
Pt 14
Pt 24
Pt 7

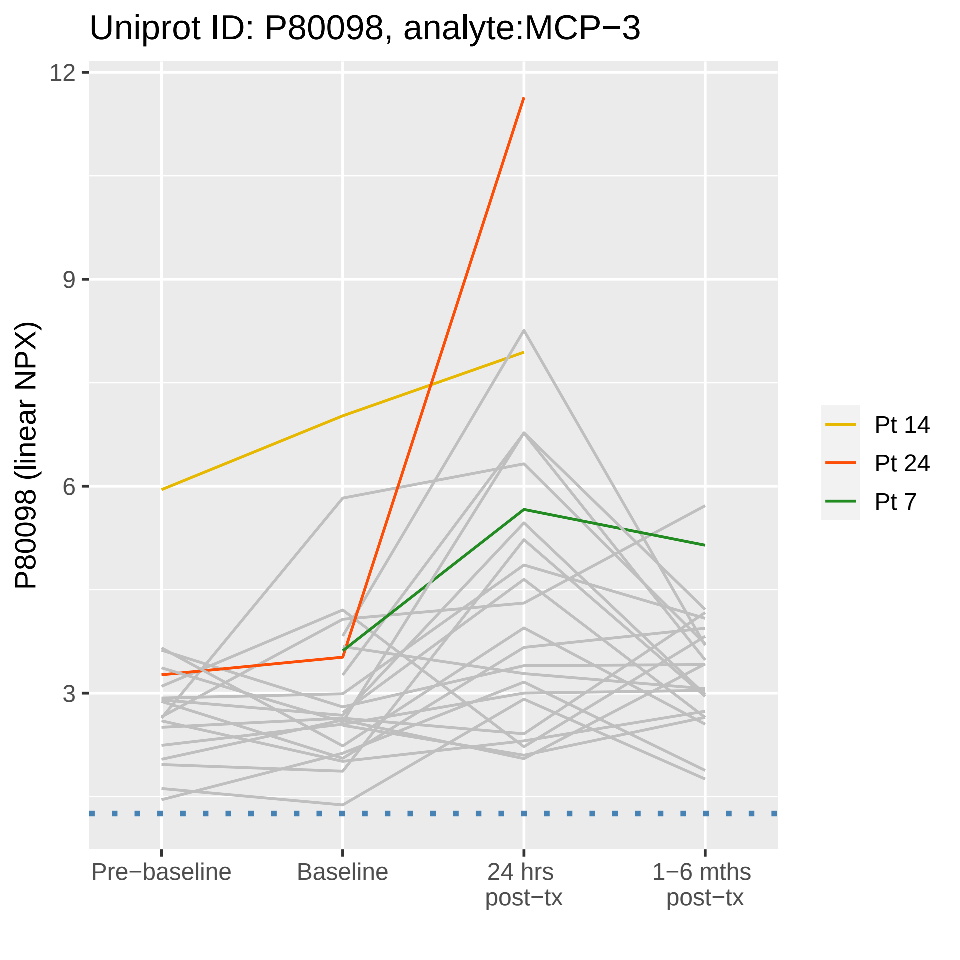
Uniprot ID: P80098, analyte:MCP−3
P80098 (linear NPX)
12
9
6
3
Pre−baseline
Baseline
24 hrs post−tx
1−6 mths post−tx
Pt 14
Pt 24
Pt 7

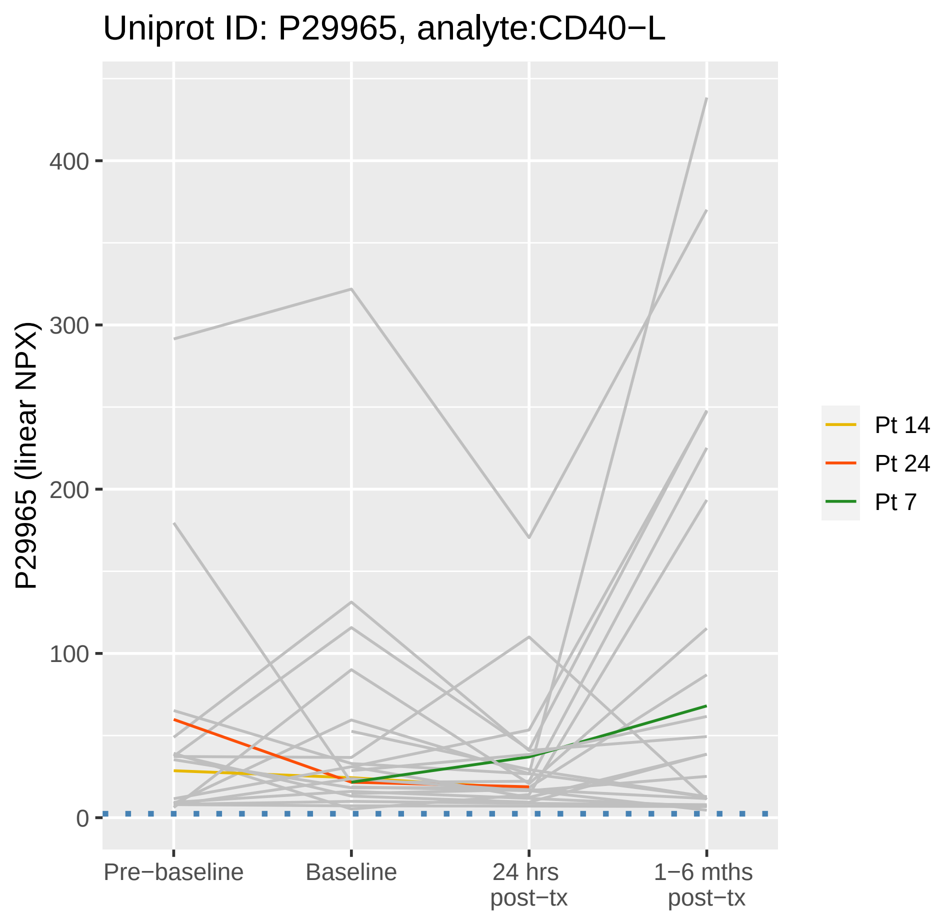
Uniprot ID: P29965, analyte:CD40−L
P29965 (linear NPX)
0
100
200
300
400
Pre−baseline
Baseline
24 hrs post−tx
1−6 mths post−tx
Pt 14
Pt 24
Pt 7

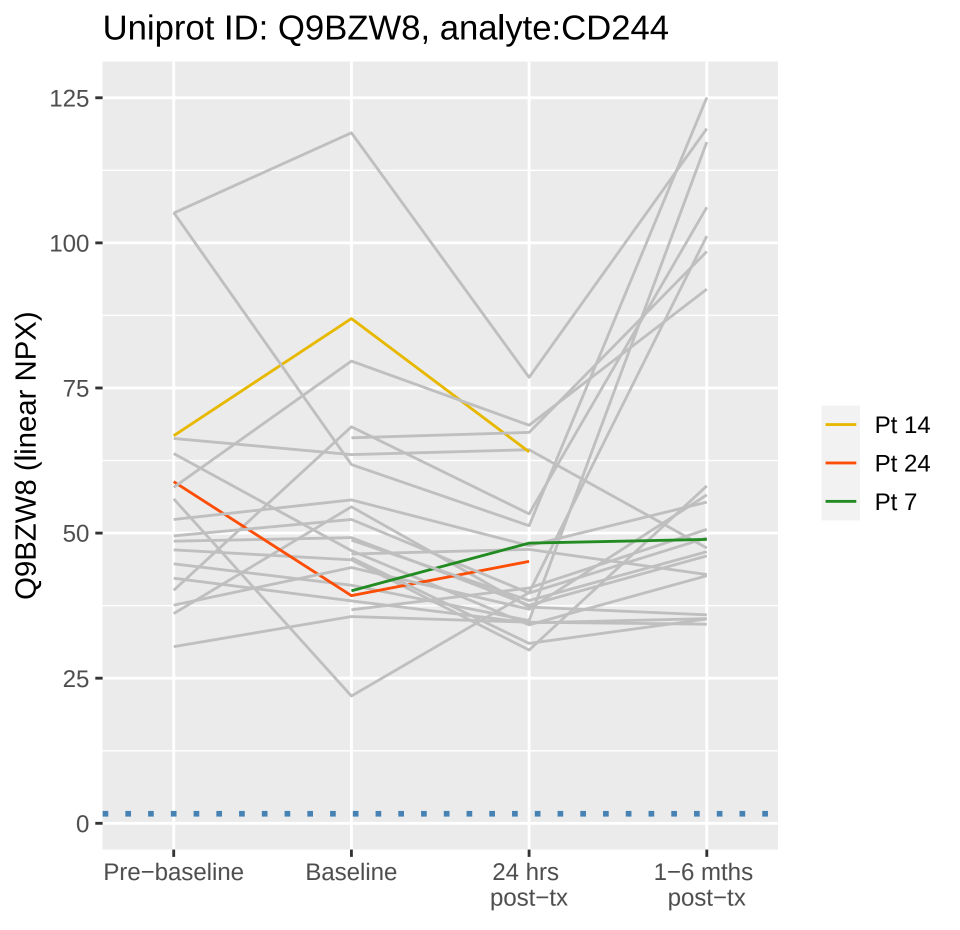

Uniprot ID: Q9BZW8, analyte:CD244
Q9BZW8 (linear NPX)
125
100
75
50
25
0
Pre−baseline
Baseline
24 hrs post−tx
1−6 mths post−tx
Pt 14
Pt 24
Pt 7

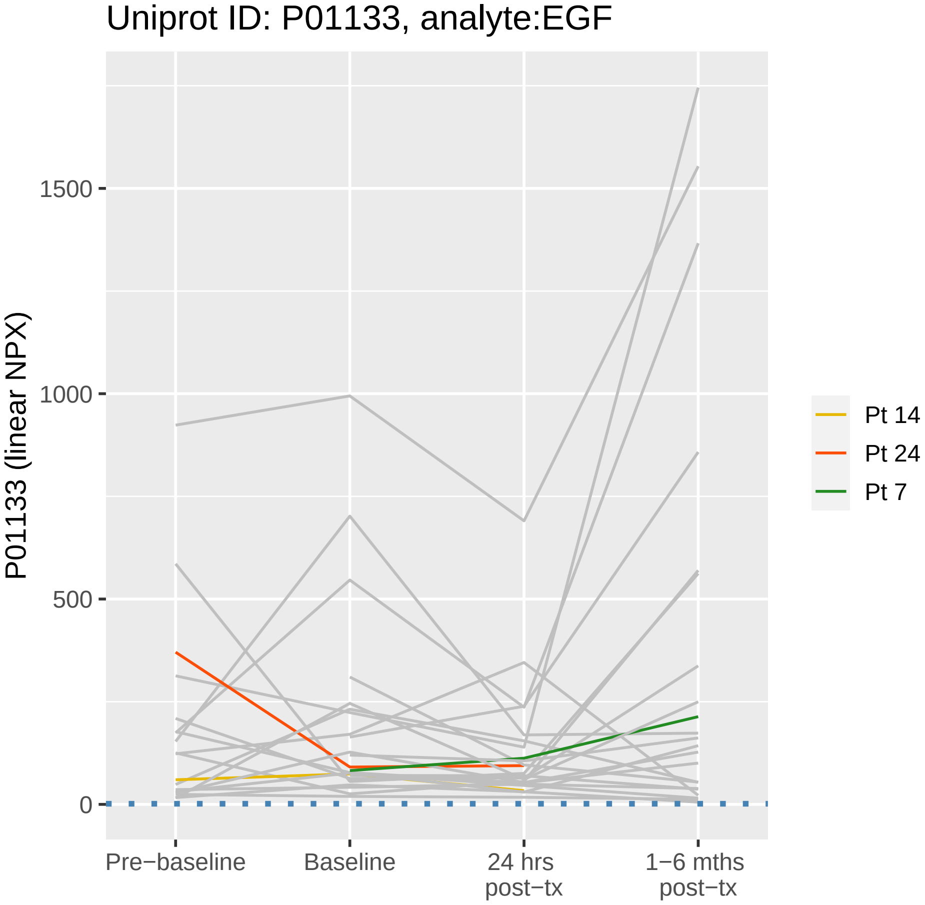

Uniprot ID: P01133, analyte:EGF
P01133 (linear NPX)
1500
1000
500
0
Pre−baseline
Baseline
24 hrs post−tx
1−6 mths post−tx
Pt 14
Pt 24
Pt 7

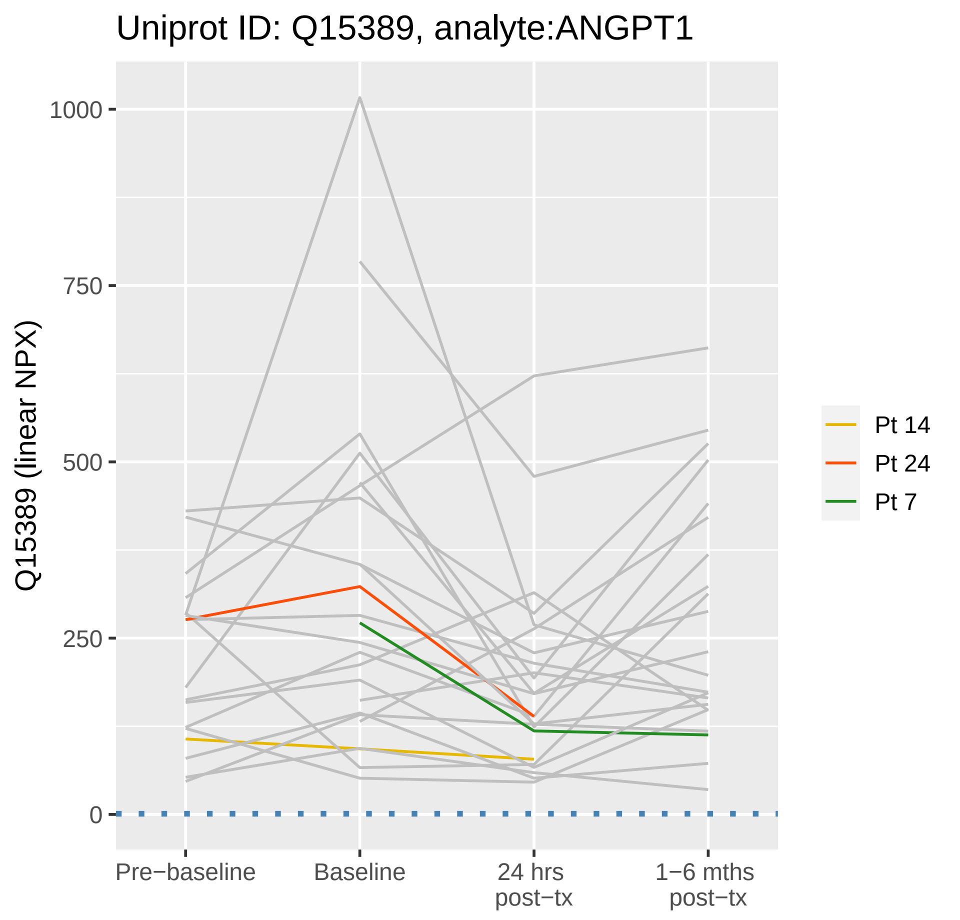

Uniprot ID: Q15389, analyte:ANGPT1
Q15389 (linear NPX)
1000
750
500
250
0
Pre−baseline
Baseline
24 hrs post−tx
1−6 mths post−tx
Pt 14
Pt 24
Pt 7

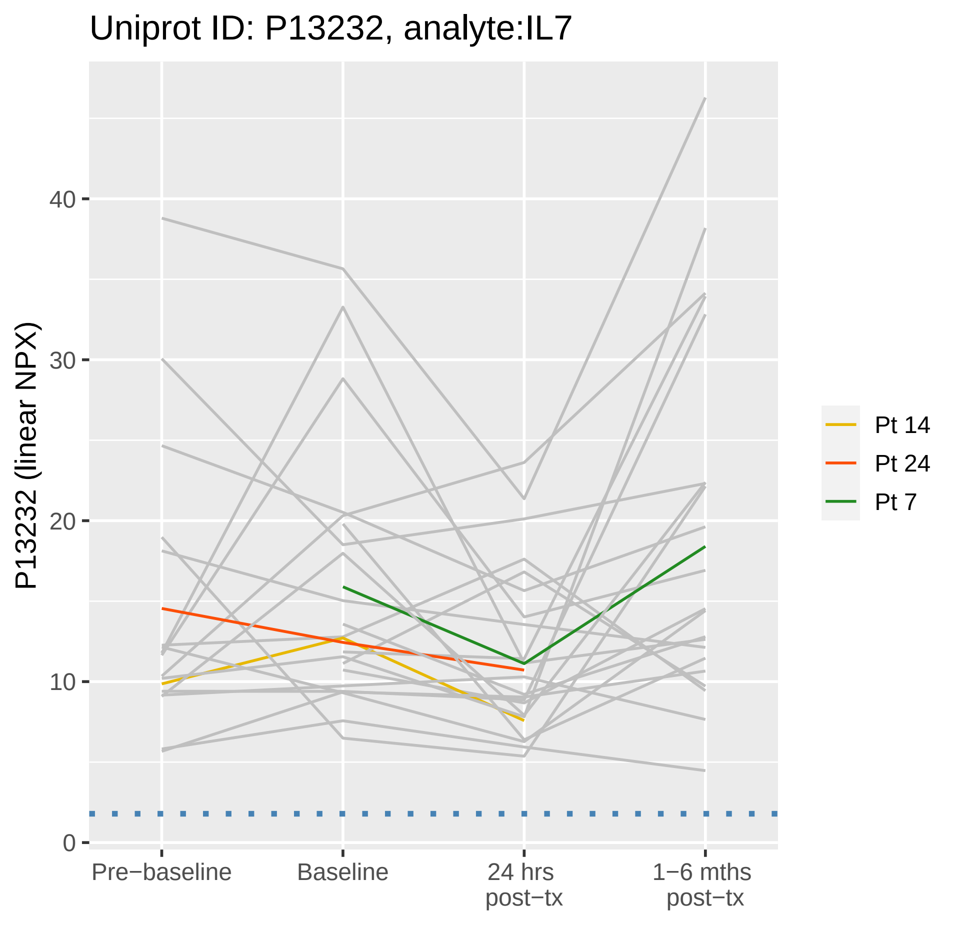

Uniprot ID: P13232, analyte:IL7
P13232 (linear NPX)
0
10
20
30
40
Pre−baseline
Baseline
24 hrs post−tx
1−6 mths post−tx
Pt 14
Pt 24
Pt 7

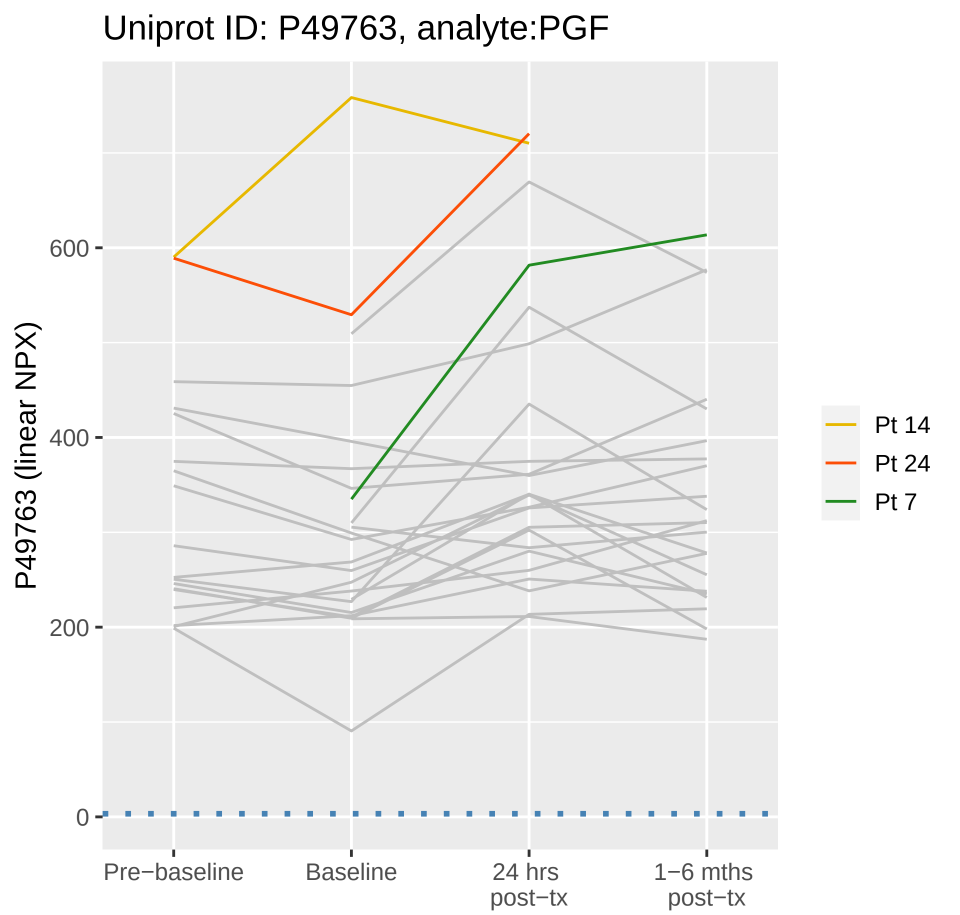

Uniprot ID: P49763, analyte:PGF
P49763 (linear NPX)
600
400
200
0
Pre−baseline
Baseline
24 hrs post−tx
1−6 mths post−tx
Pt 14
Pt 24
Pt 7

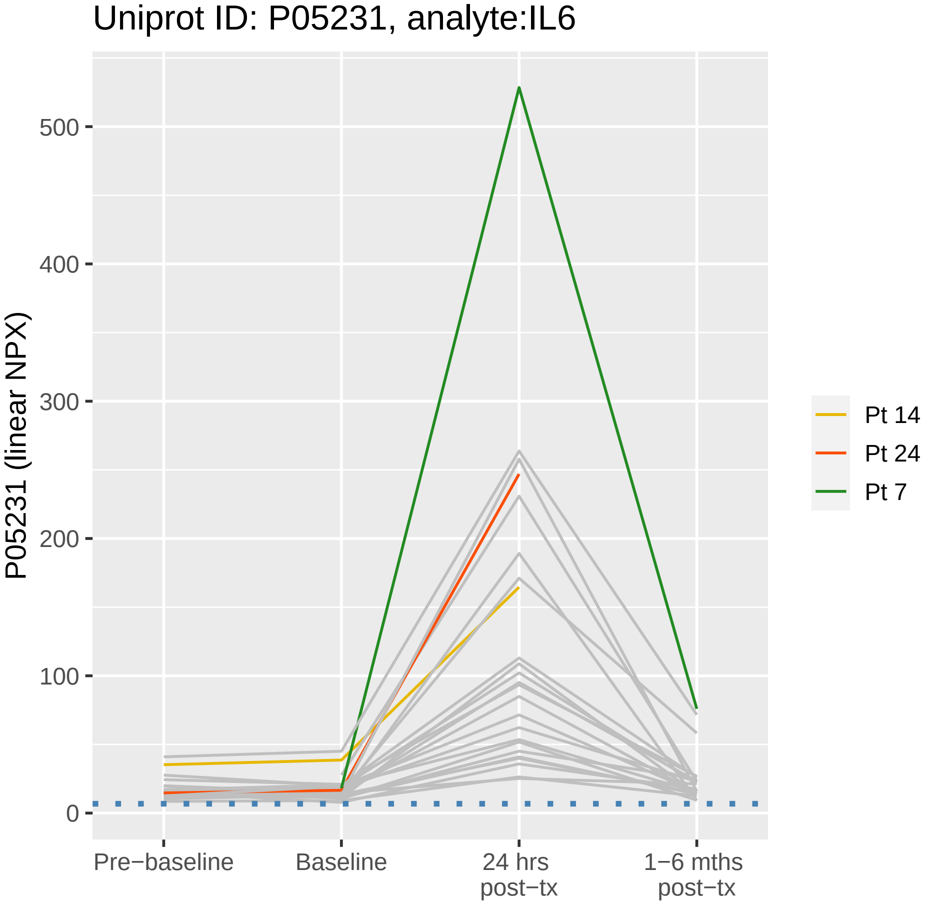

Uniprot ID: P05231, analyte:IL6
P05231 (linear NPX)
500
400
300
200
100
0
Pre−baseline
Baseline
24 hrs post−tx
1−6 mths post−tx
Pt 14
Pt 24
Pt 7

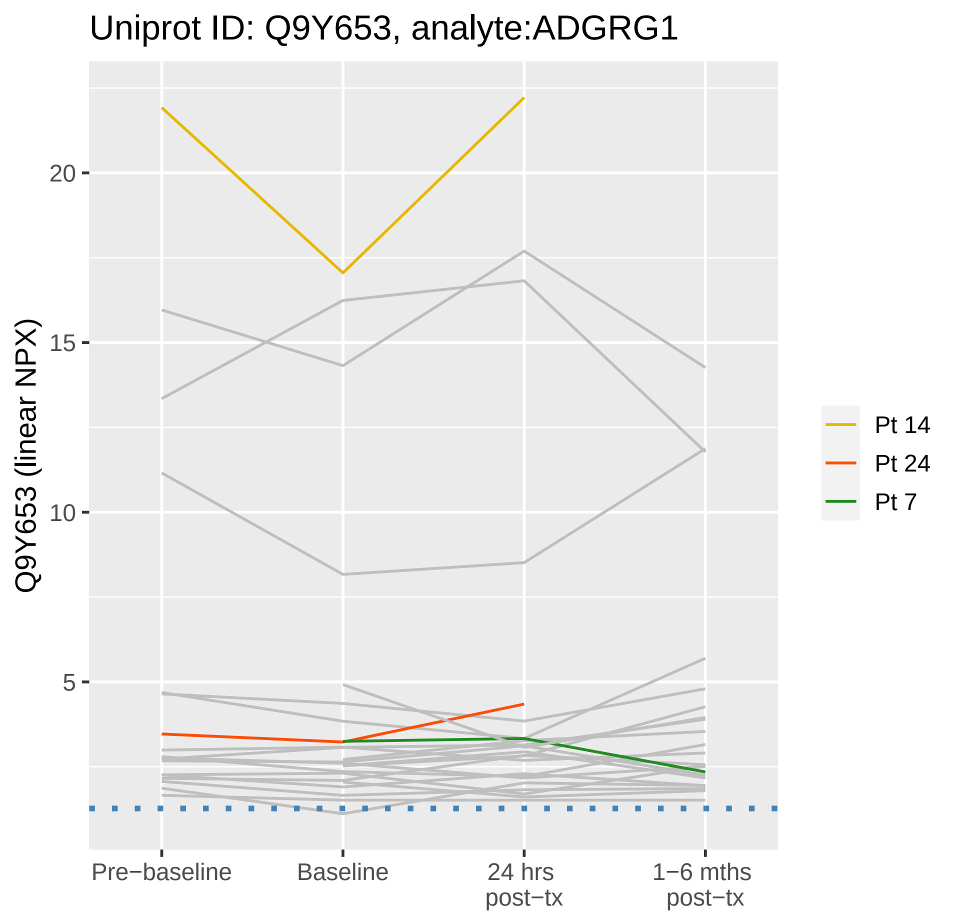
Uniprot ID: Q9Y653, analyte:ADGRG1
Q9Y653 (linear NPX)
20
15
10
5
Pre−baseline
Baseline
24 hrs post−tx
1−6 mths post−tx
Pt 14
Pt 24
Pt 7

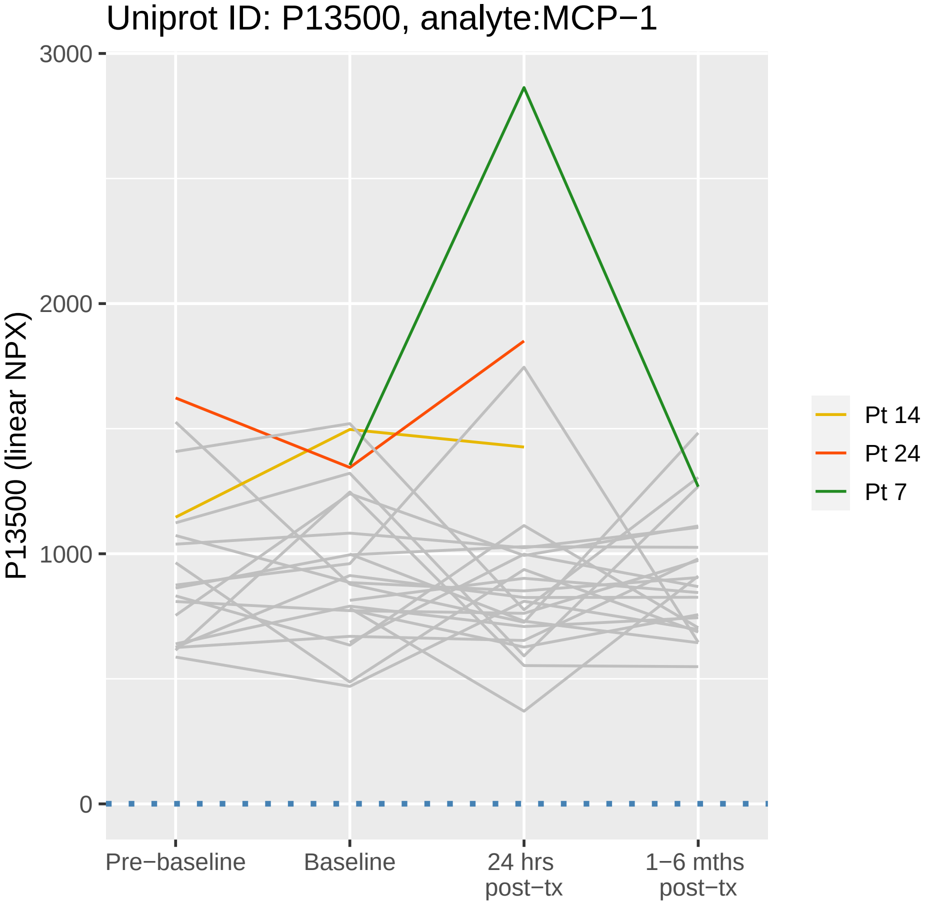

Uniprot ID: P13500, analyte:MCP−1
P13500 (linear NPX)
3000
2000
1000
0
Pre−baseline
Baseline
24 hrs post−tx
1−6 mths post−tx
Pt 14
Pt 24
Pt 7

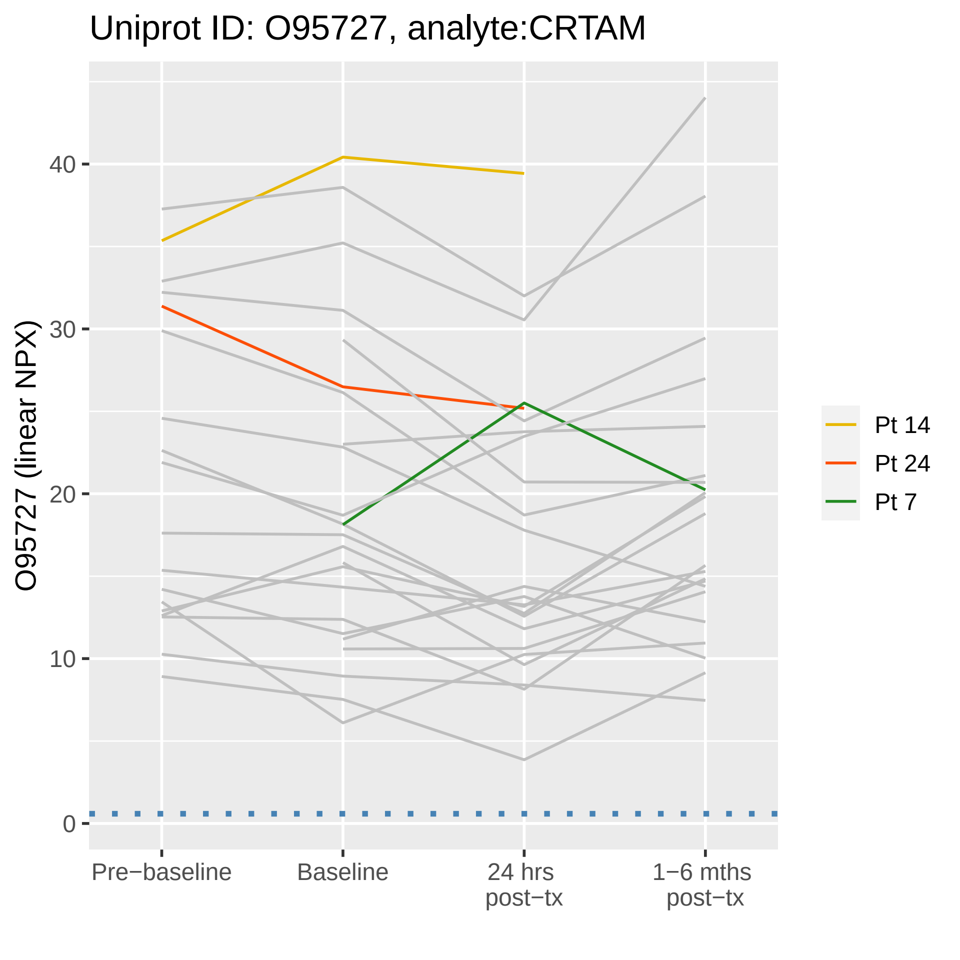

Uniprot ID: O95727, analyte:CRTAM
O95727 (linear NPX)
40
30
20
10
0
Pre−baseline
Baseline
24 hrs post−tx
1−6 mths post−tx
Pt 14
Pt 24
Pt 7

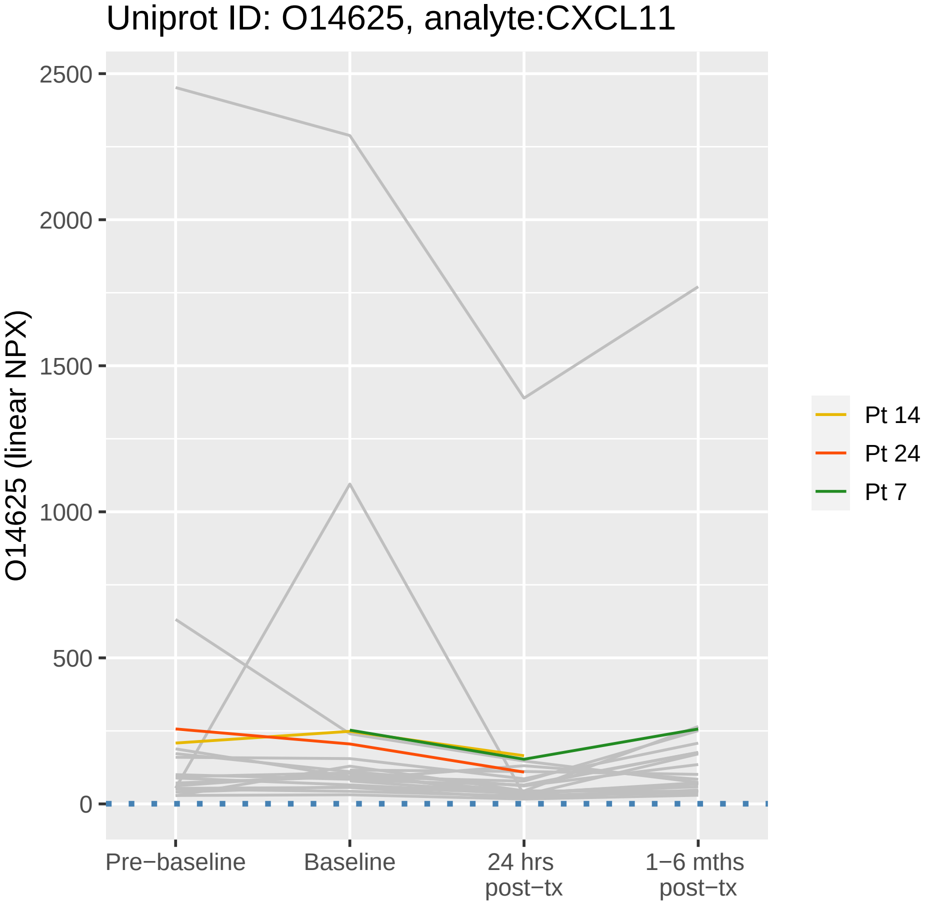

Uniprot ID: O14625, analyte:CXCL11
O14625 (linear NPX)
2500
2000
1500
1000
500
0
Pre−baseline
Baseline
24 hrs post−tx
1−6 mths post−tx
Pt 14
Pt 24
Pt 7

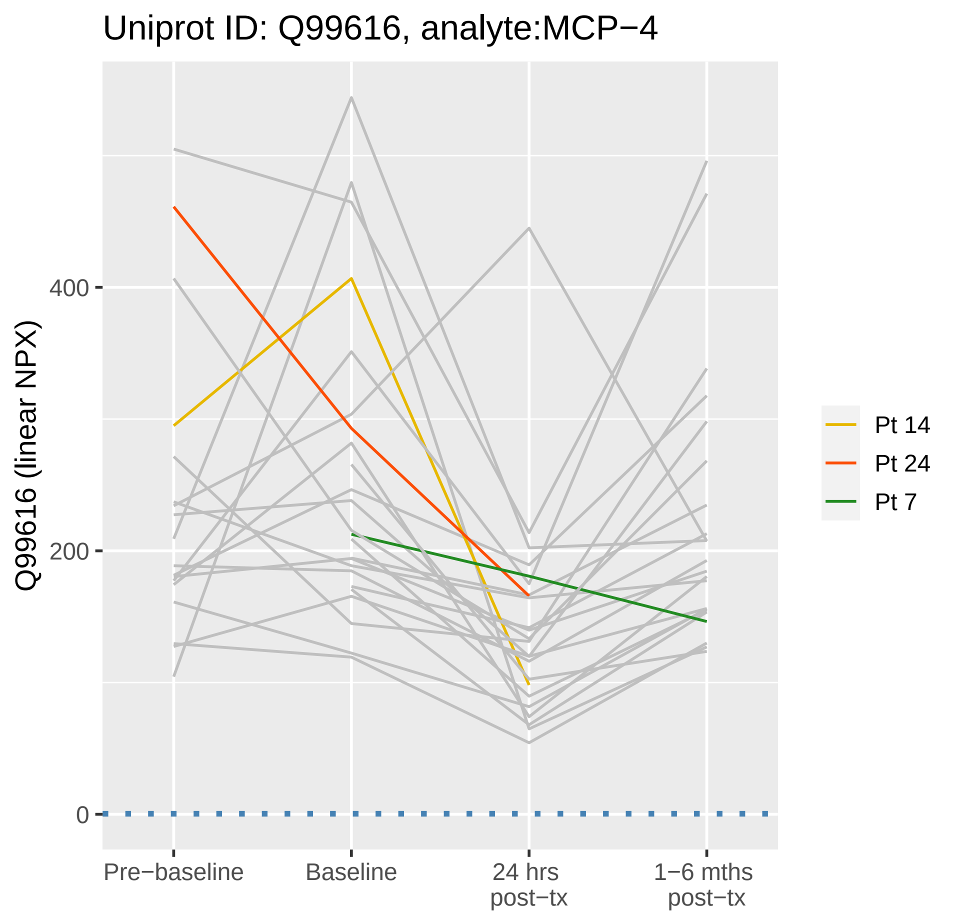
Uniprot ID: Q99616, analyte:MCP−4
Q99616 (linear NPX)
400
200
0
Pre−baseline
Baseline
24 hrs post−tx
1−6 mths post−tx
Pt 14
Pt 24
Pt 7

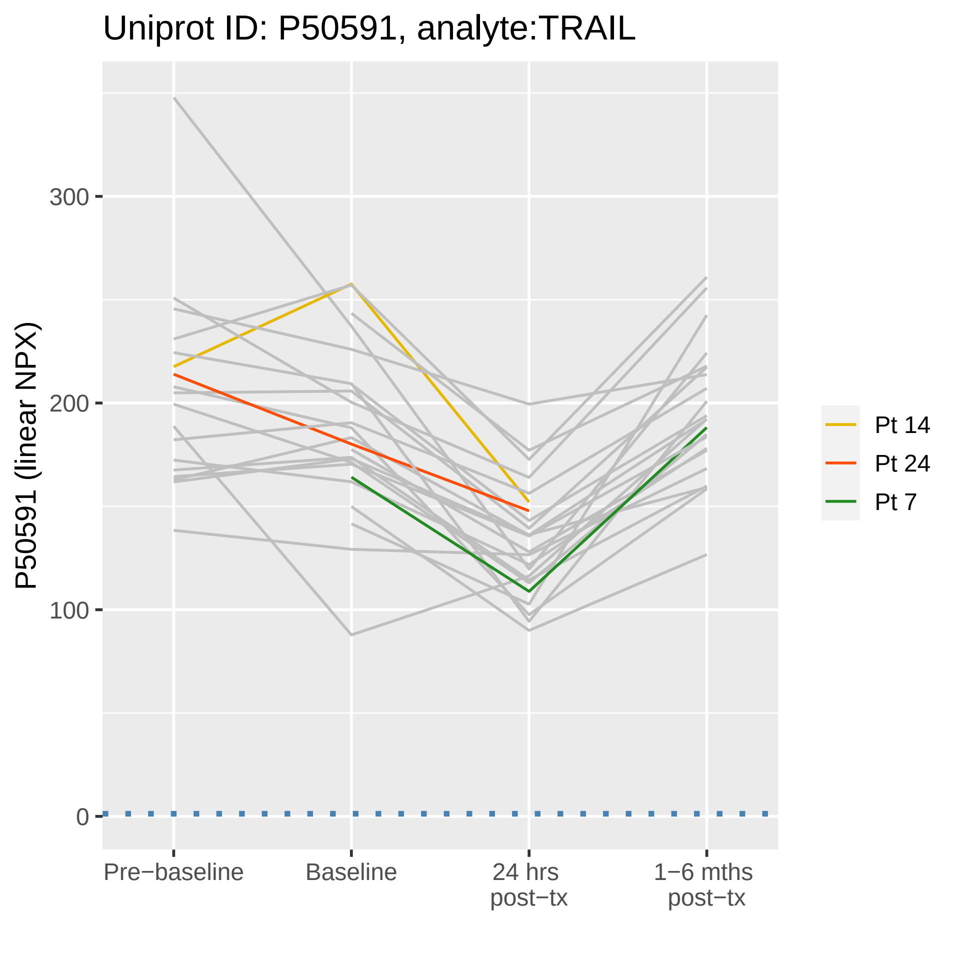
Uniprot ID: P50591, analyte:TRAIL
P50591 (linear NPX)
300
200
100
0
Pre−baseline
Baseline
24 hrs post−tx
1−6 mths post−tx
Pt 14
Pt 24
Pt 7

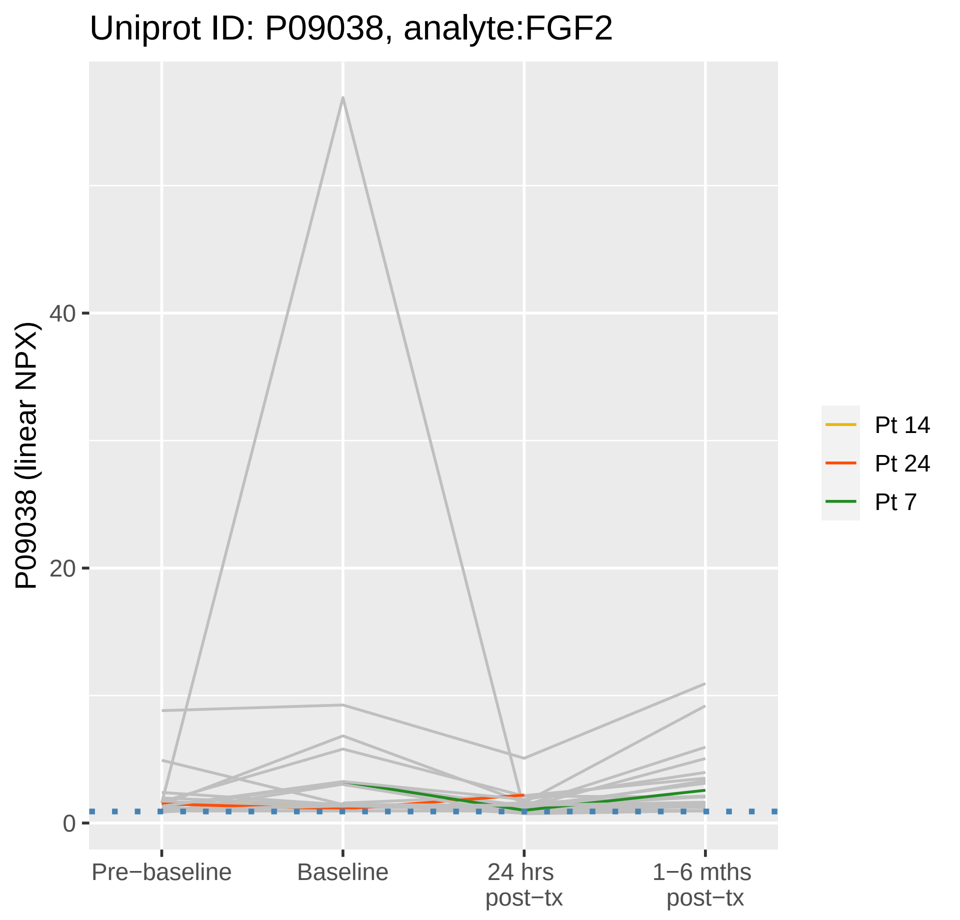
Uniprot ID: P09038, analyte:FGF2
P09038 (linear NPX)
40
20
0
Pre−baseline
Baseline
24 hrs
post−tx
1−6 mths
post−tx
Pt 14
Pt 24
Pt 7

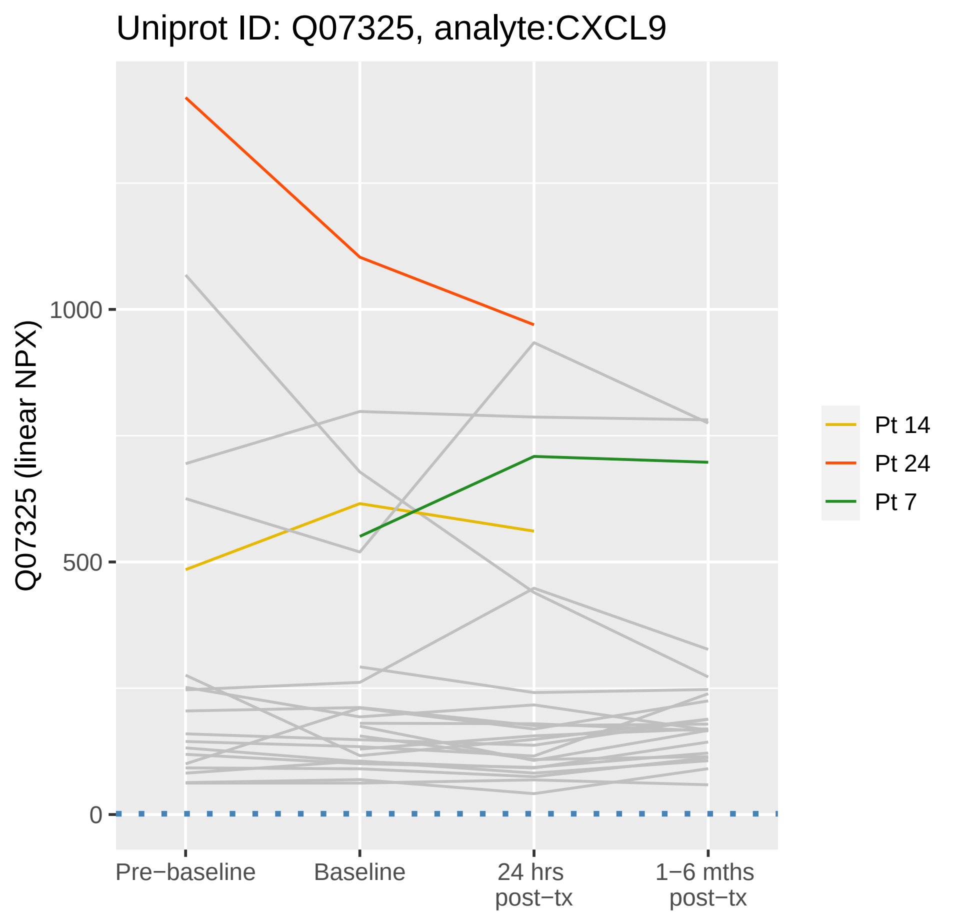

Uniprot ID: Q07325, analyte:CXCL9
Q07325 (linear NPX)
1000
500
0
Pre−baseline
Baseline
24 hrs post−tx
1−6 mths post−tx
Pt 14
Pt 24
Pt 7

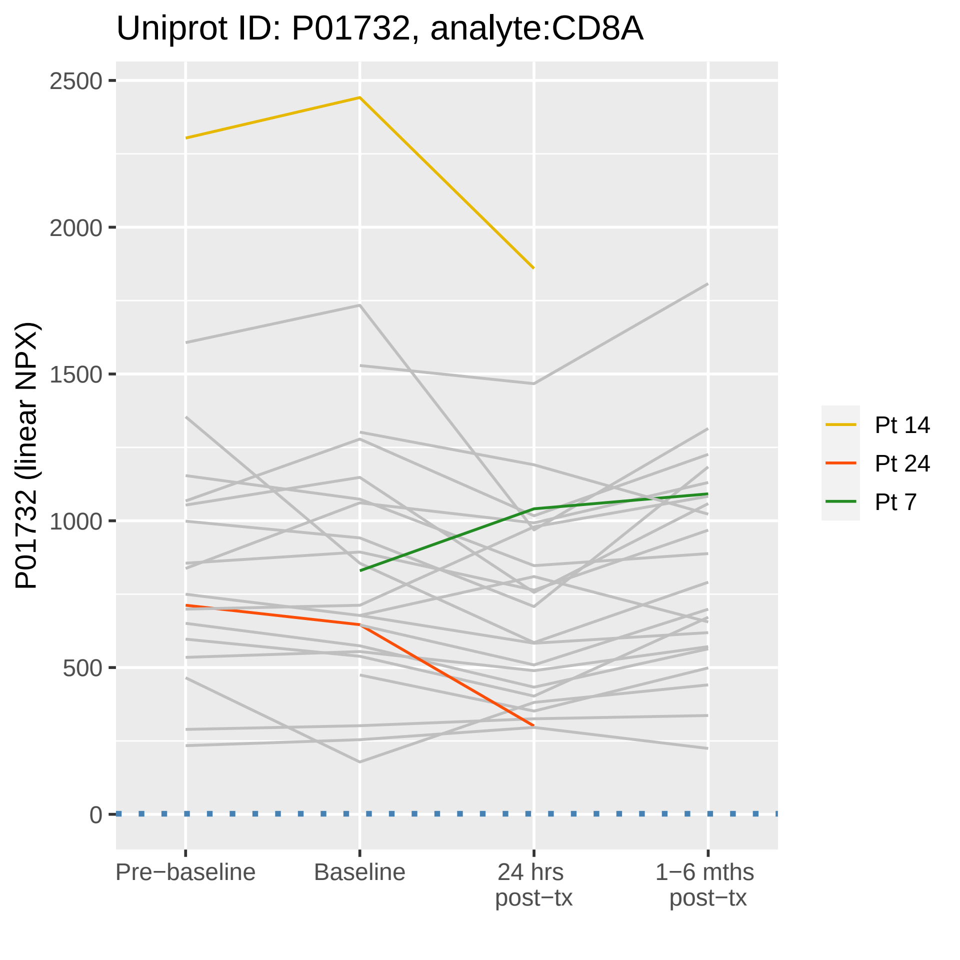
Uniprot ID: P01732, analyte:CD8A
P01732 (linear NPX)
2500
2000
1500
1000
500
0
Pre−baseline
Baseline
24 hrs post−tx
1−6 mths post−tx
Pt 14
Pt 24
Pt 7

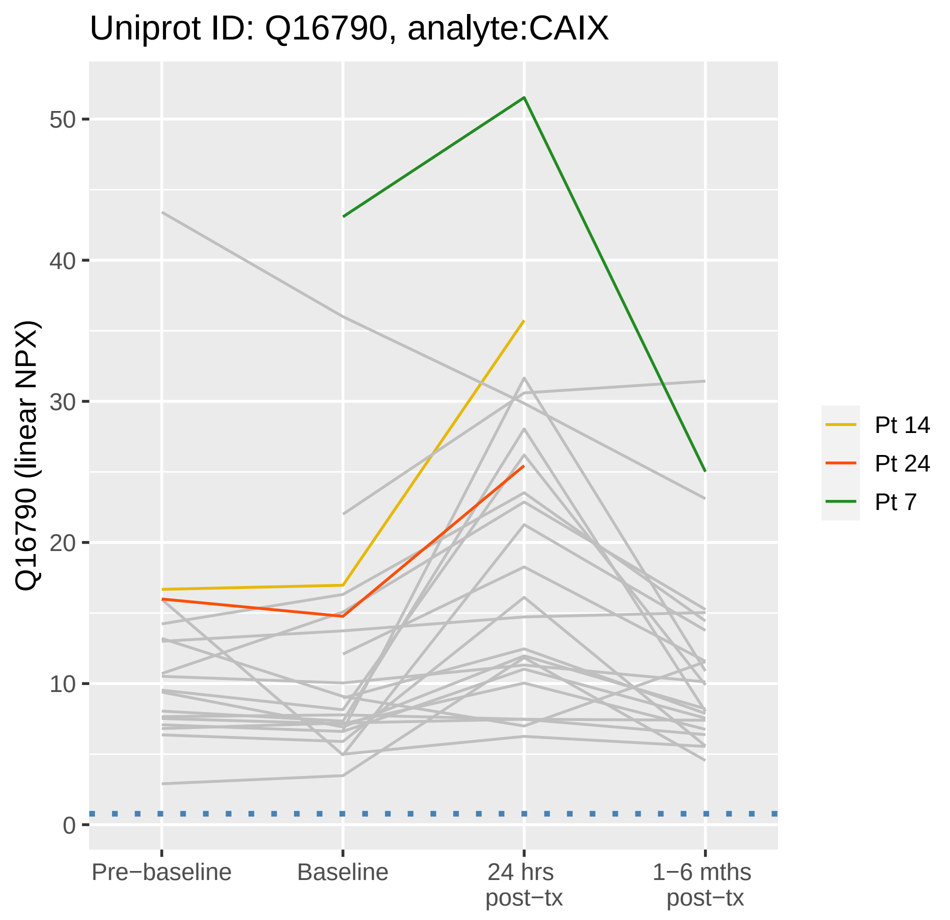

Uniprot ID: Q16790, analyte:CAIX
Q16790 (linear NPX)
50
40
30
20
10
0
Pre−baseline
Baseline
24 hrs post−tx
1−6 mths post−tx
Pt 14
Pt 24
Pt 7

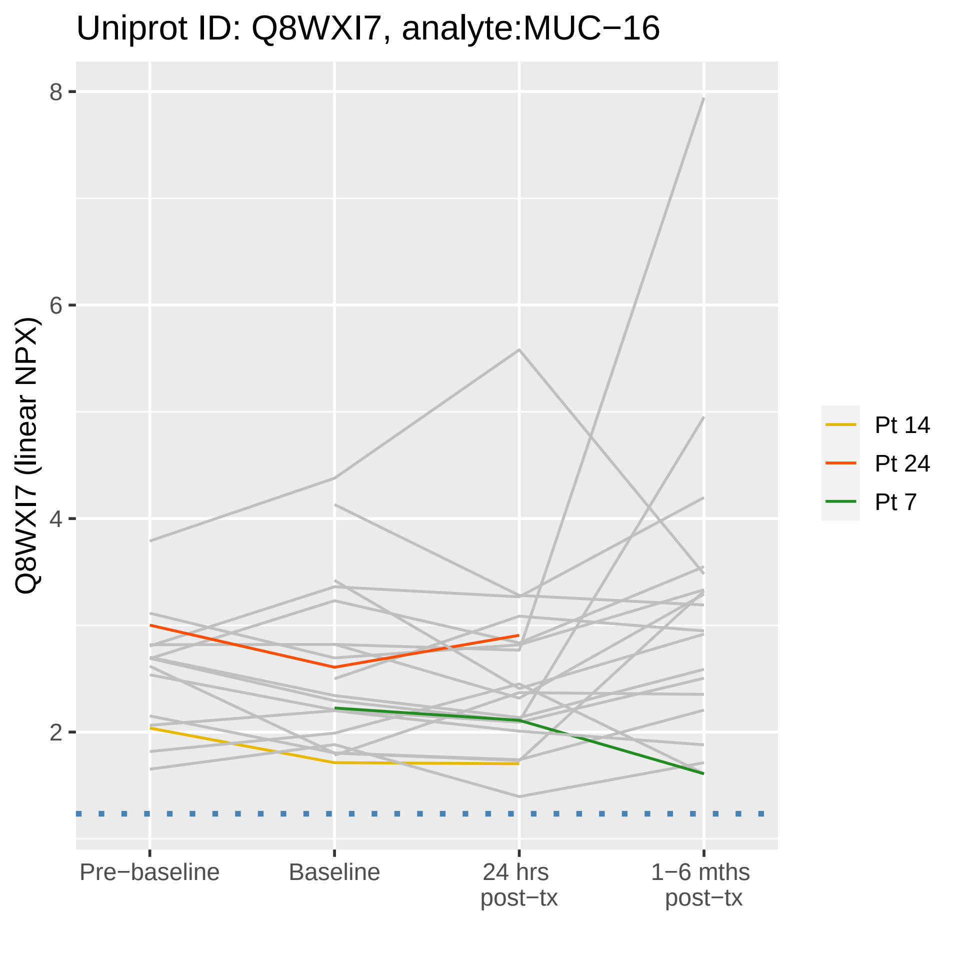

Uniprot ID: Q8WXI7, analyte:MUC−16
Q8WXI7 (linear NPX)
8
6
4
2
Pre−baseline
Baseline
24 hrs post−tx
1−6 mths post−tx
Pt 14
Pt 24
Pt 7

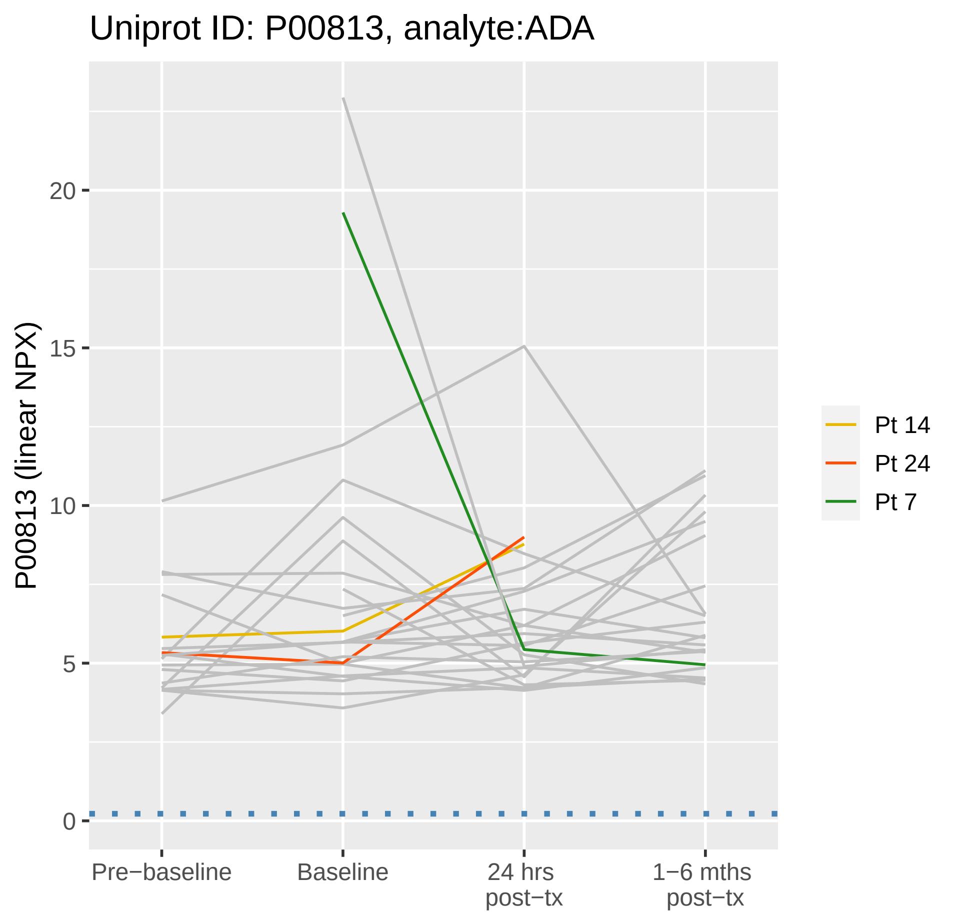

Uniprot ID: P00813, analyte:ADA
P00813 (linear NPX)
20
15
10
5
0
Pre−baseline
Baseline
24 hrs post−tx
1−6 mths post−tx
Pt 14
Pt 24
Pt 7

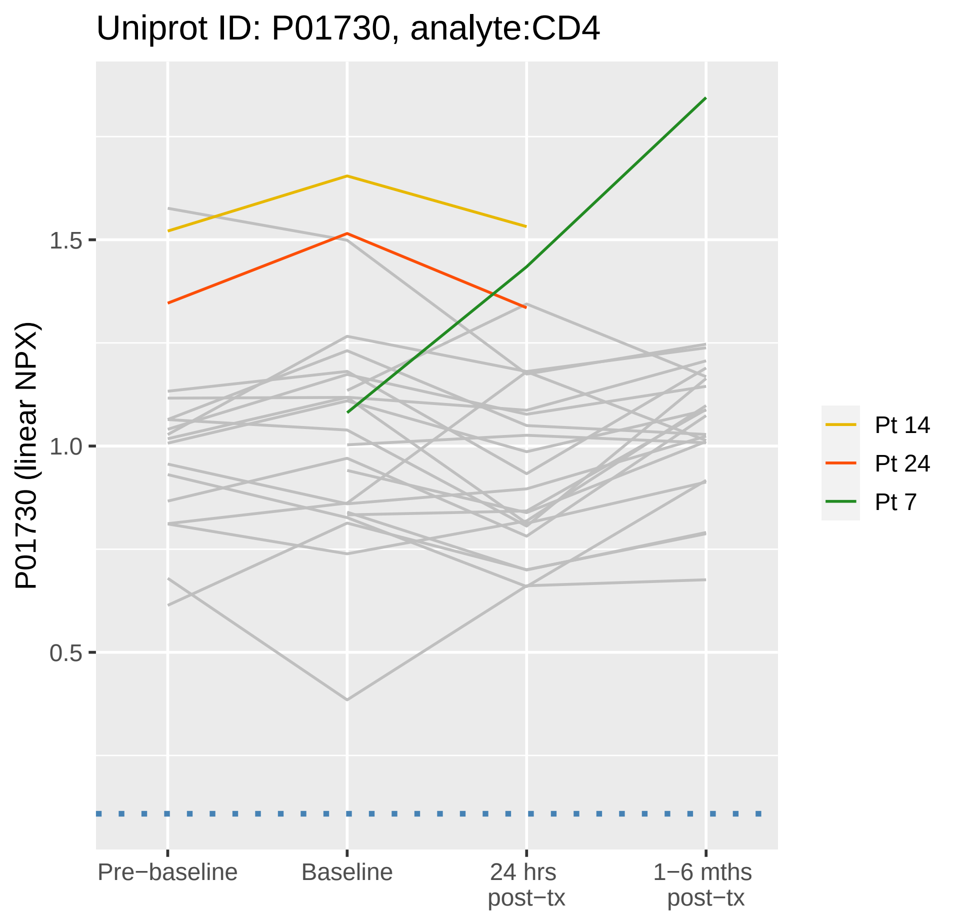

Uniprot ID: P01730, analyte:CD4
P01730 (linear NPX)
1.5
1.0
0.5
Pre−baseline
Baseline
24 hrs
post−tx
1−6 mths
post−tx
Pt 14
Pt 24
Pt 7

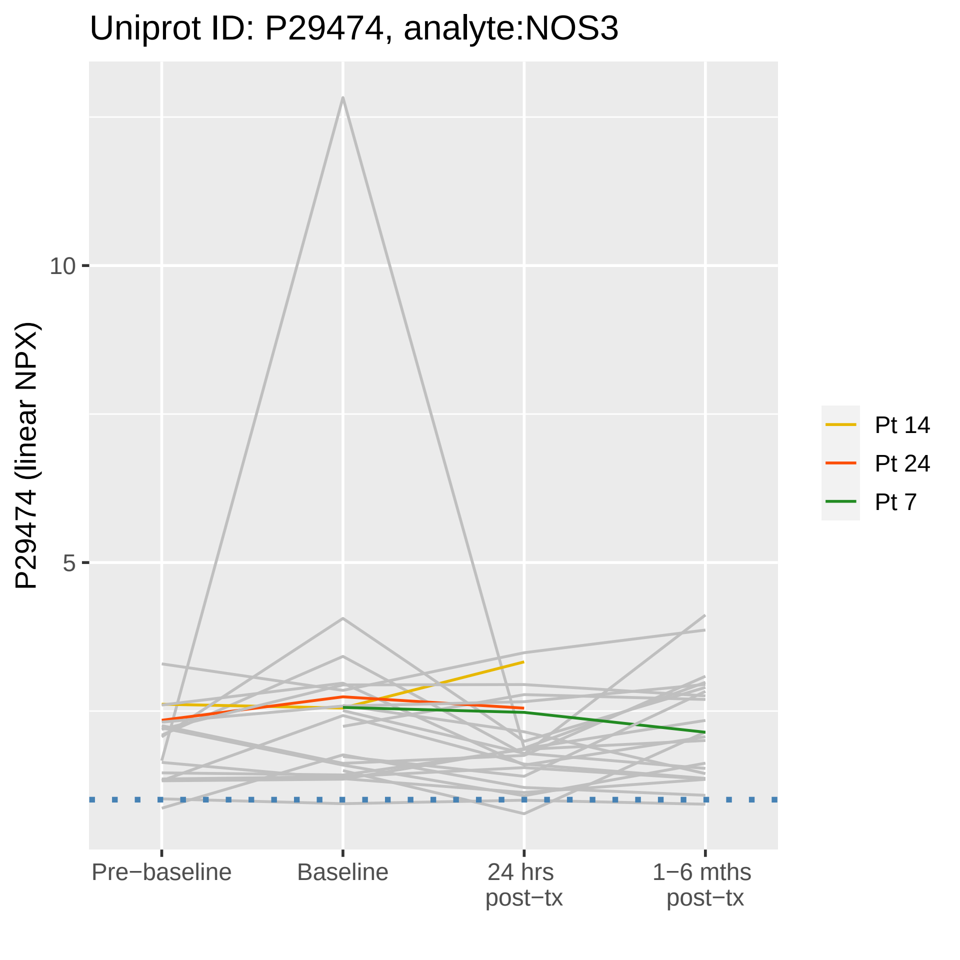

Uniprot ID: P29474, analyte:NOS3
P29474 (linear NPX)
10
5
Pre−baseline
Baseline
24 hrs post−tx
1−6 mths post−tx
Pt 14
Pt 24
Pt 7

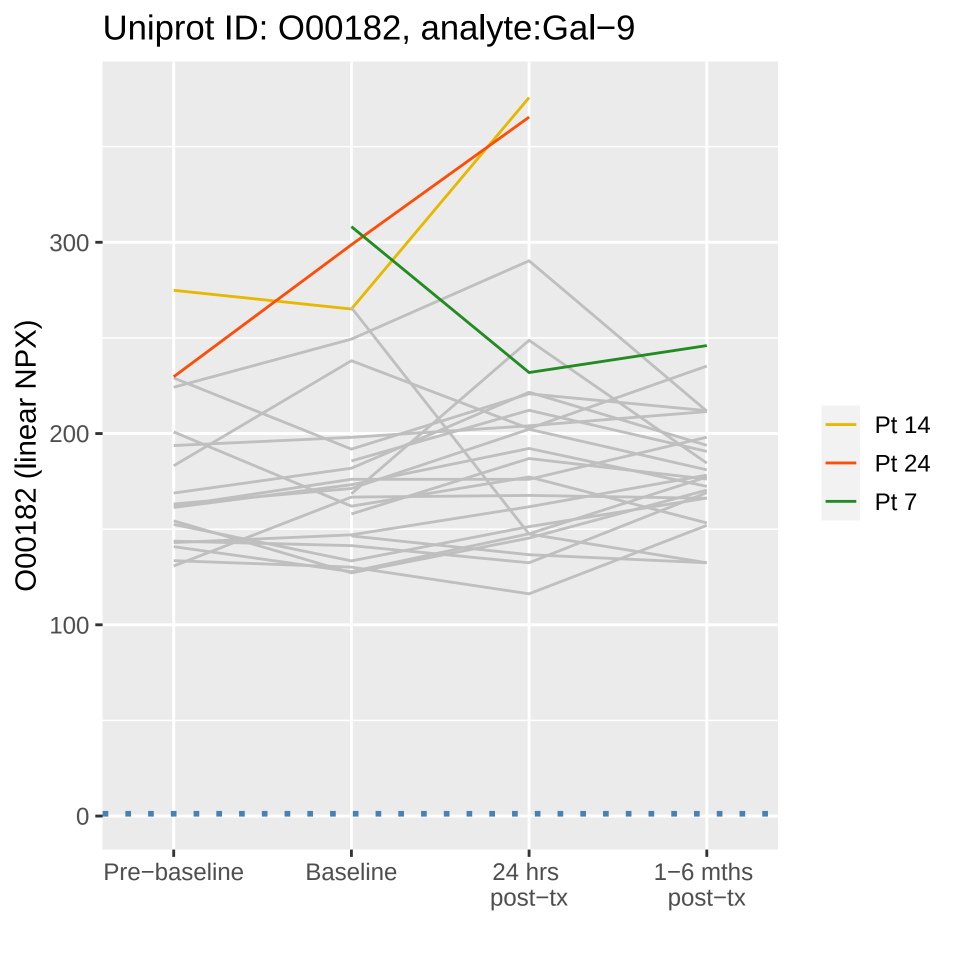

Uniprot ID: O00182, analyte:Gal−9
O00182 (linear NPX)
300
200
100
0
Pre−baseline
Baseline
24 hrs post−tx
1−6 mths post−tx
Pt 14
Pt 24
Pt 7

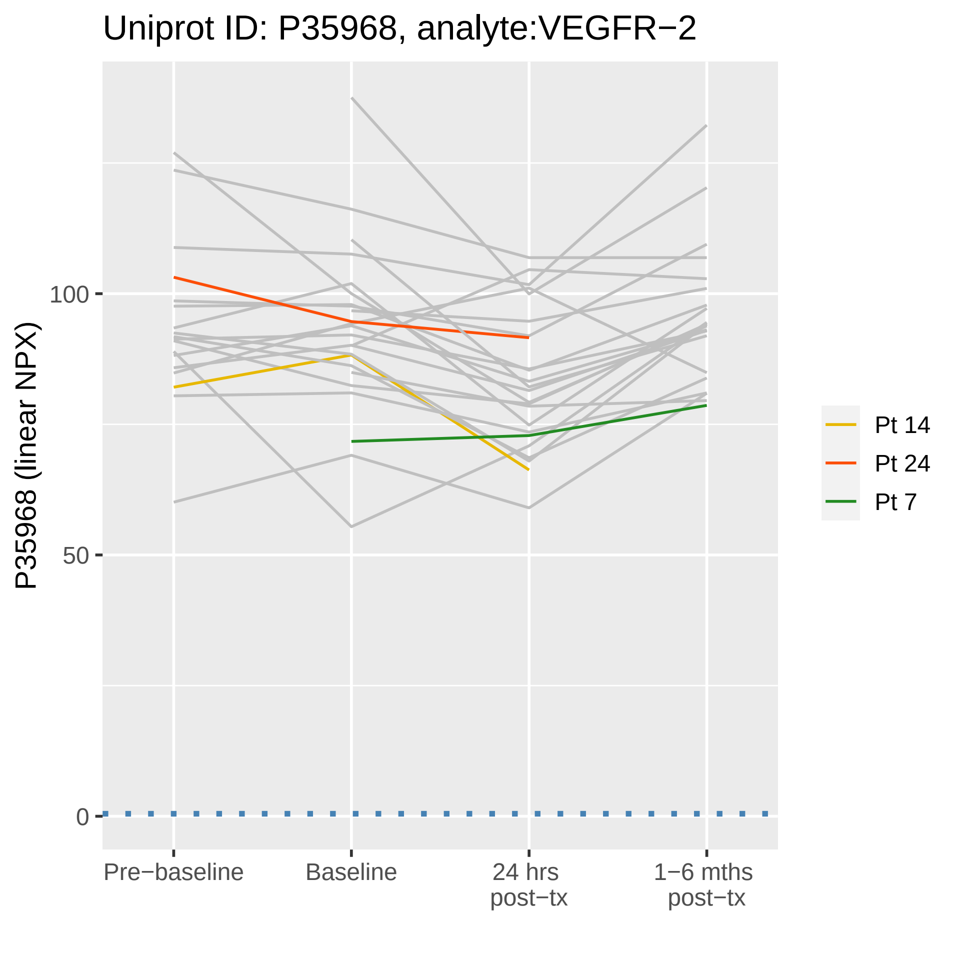
Uniprot ID: P35968, analyte:VEGFR−2
P35968 (linear NPX)
100
50
0
Pre−baseline
Baseline
24 hrs post−tx
1−6 mths post−tx
Pt 14
Pt 24
Pt 7

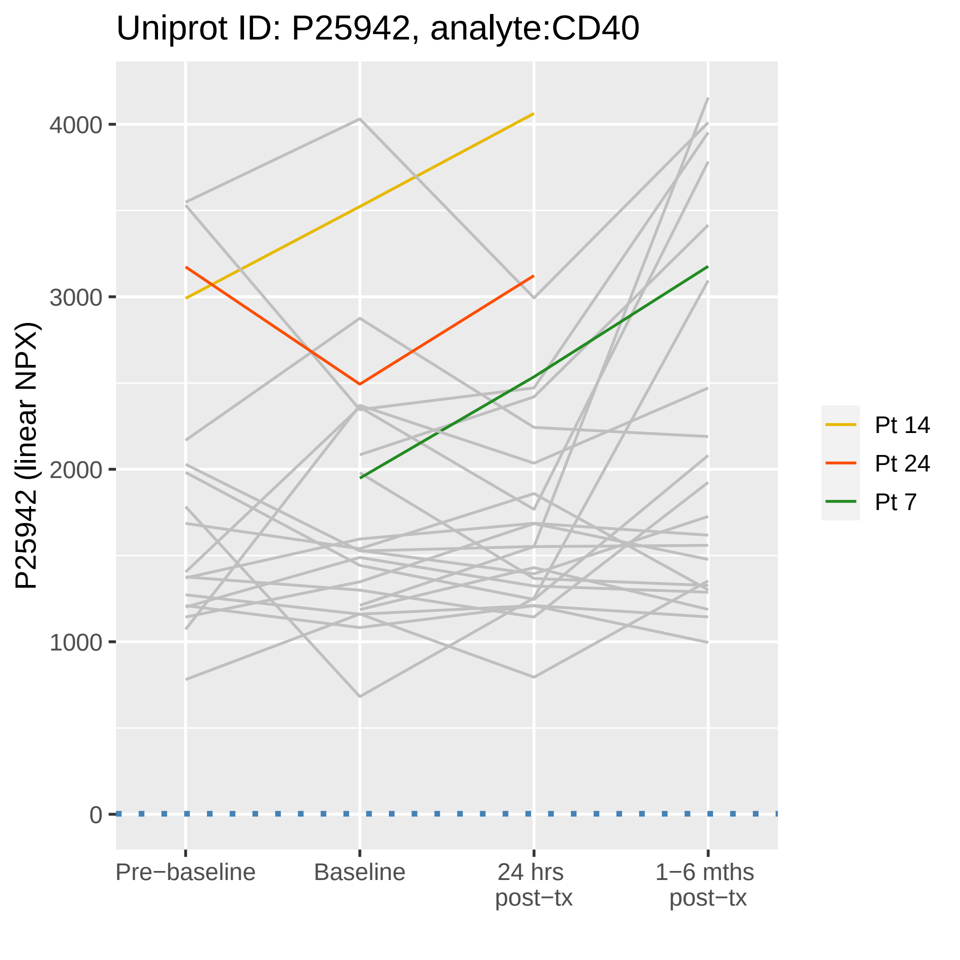
Uniprot ID: P25942, analyte:CD40
P25942 (linear NPX)
4000
3000
2000
1000
0
Pre−baseline
Baseline
24 hrs post−tx
1−6 mths post−tx
Pt 14
Pt 24
Pt 7

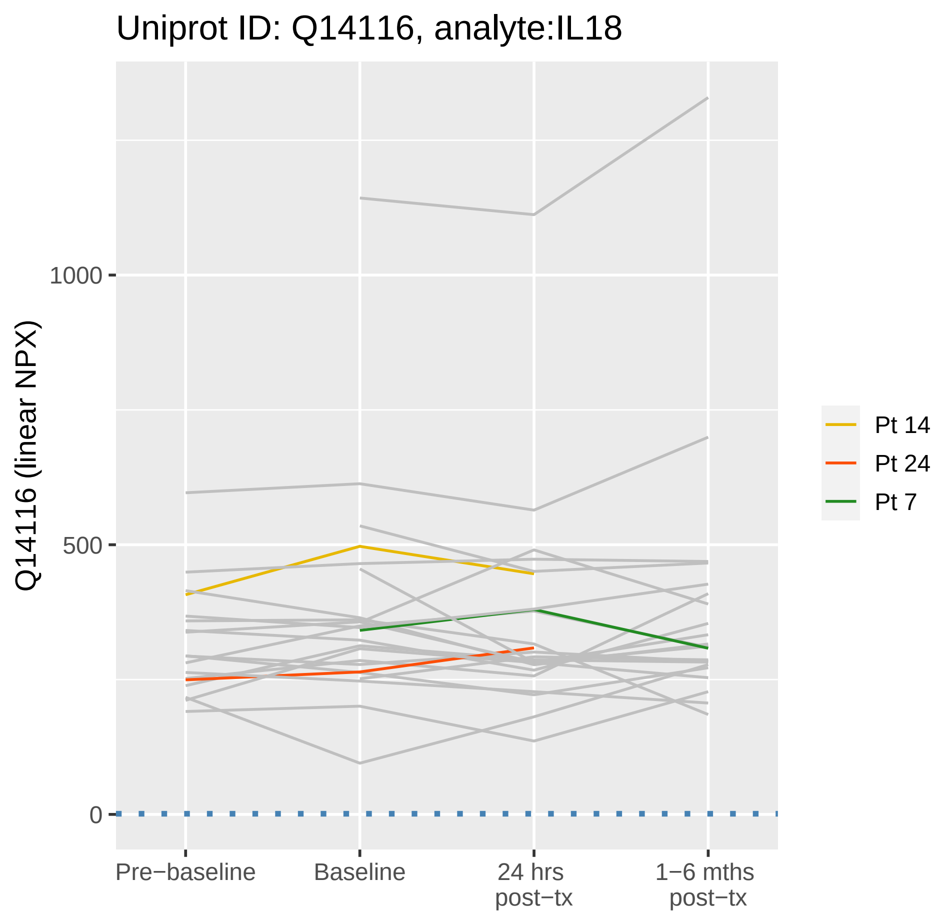

Uniprot ID: Q14116, analyte:IL18
Q14116 (linear NPX)
1000
500
0
Pre−baseline
Baseline
24 hrs post−tx
1−6 mths post−tx
Pt 14
Pt 24
Pt 7

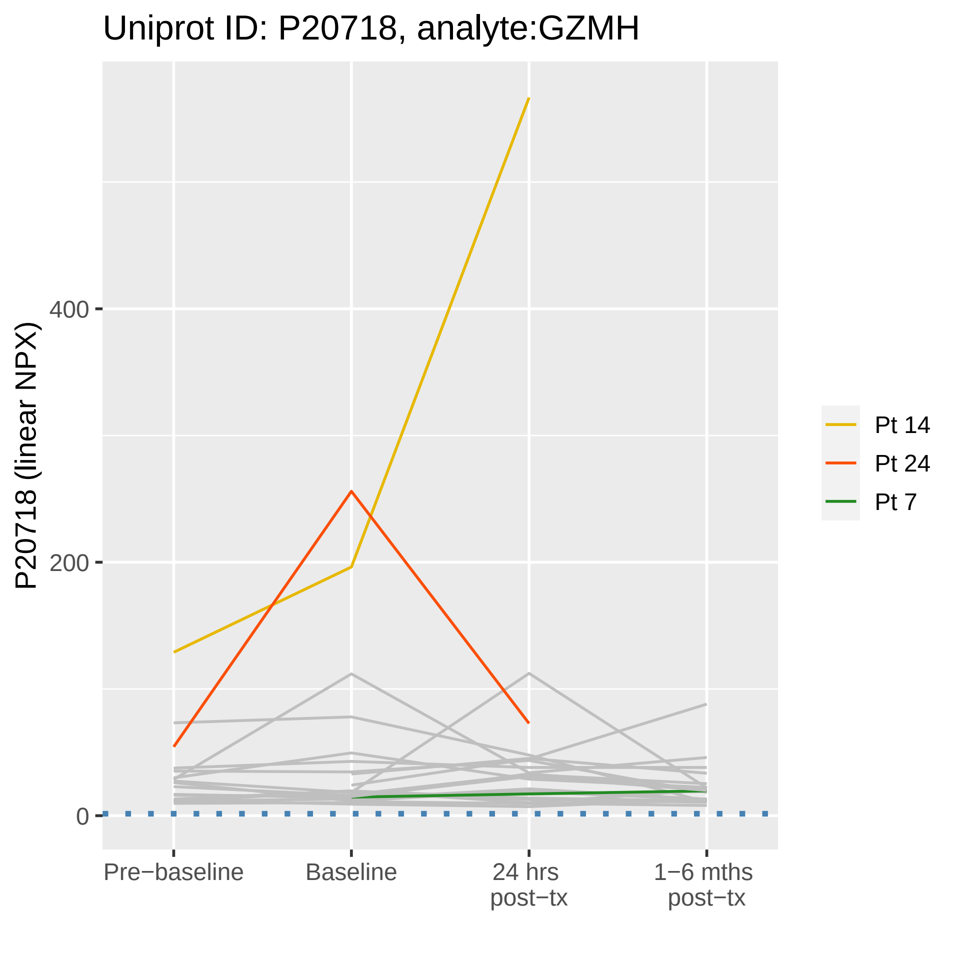

Uniprot ID: P20718, analyte:GZMH
P20718 (linear NPX)
400
200
0
Pre−baseline
Baseline
24 hrs post−tx
1−6 mths post−tx
Pt 14
Pt 24
Pt 7

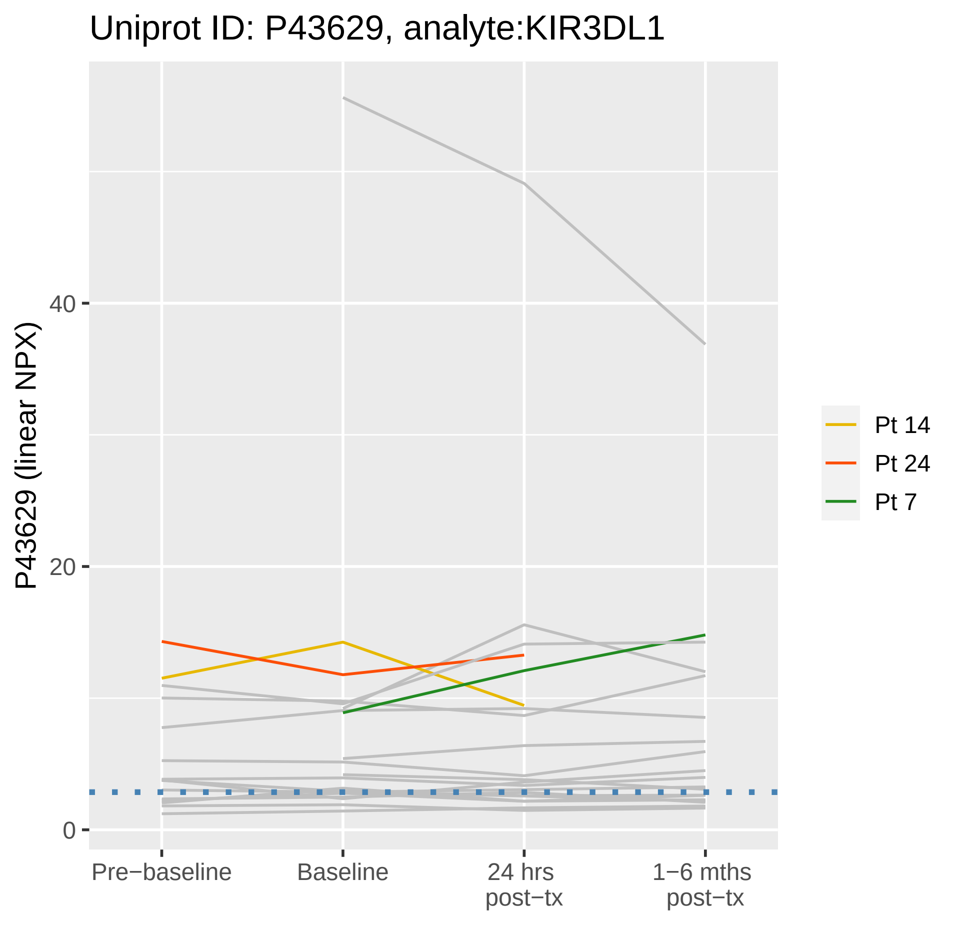

Uniprot ID: P43629, analyte:KIR3DL1
P43629 (linear NPX)
40
20
0
Pre−baseline
Baseline
24 hrs
post−tx
1−6 mths
post−tx
Pt 14
Pt 24
Pt 7

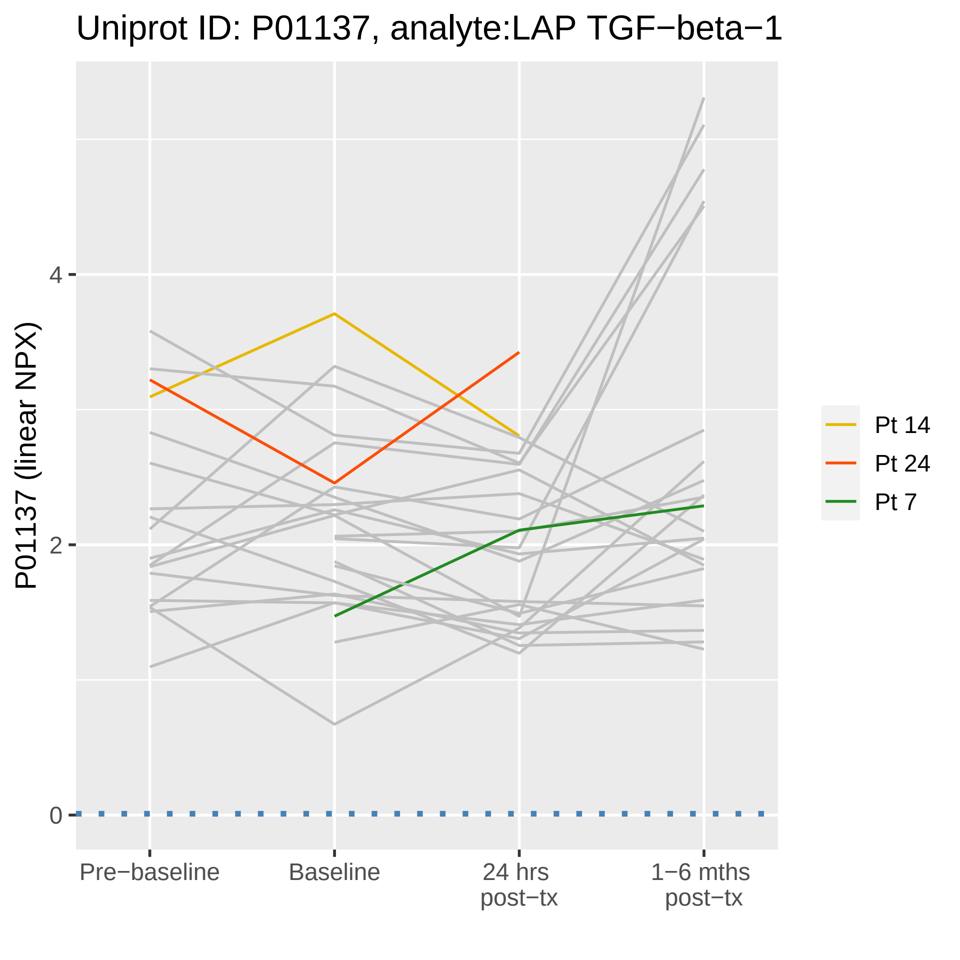

Uniprot ID: P01137, analyte:LAP TGF−beta−1
P01137 (linear NPX)
4
2
0
Pre−baseline
Baseline
24 hrs post−tx
1−6 mths post−tx
Pt 14
Pt 24
Pt 7

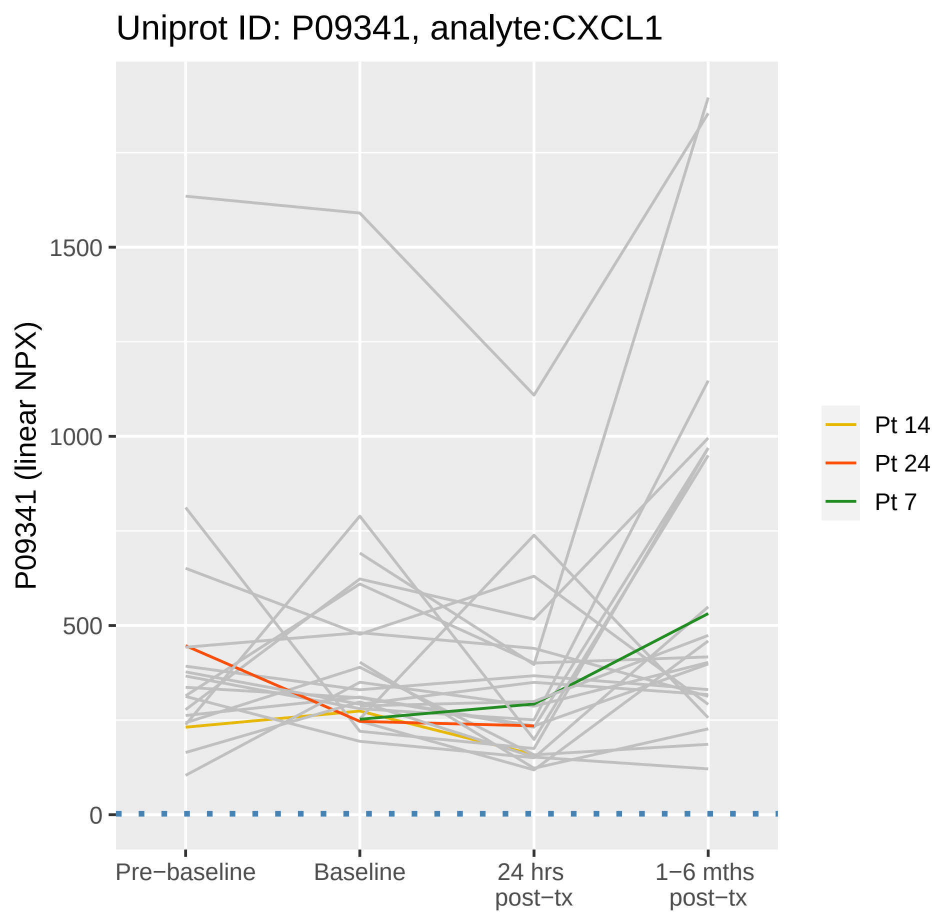
Uniprot ID: P09341, analyte:CXCL1
P09341 (linear NPX)
1500
1000
500
0
Pre−baseline
Baseline
24 hrs post−tx
1−6 mths post−tx
Pt 14
Pt 24
Pt 7

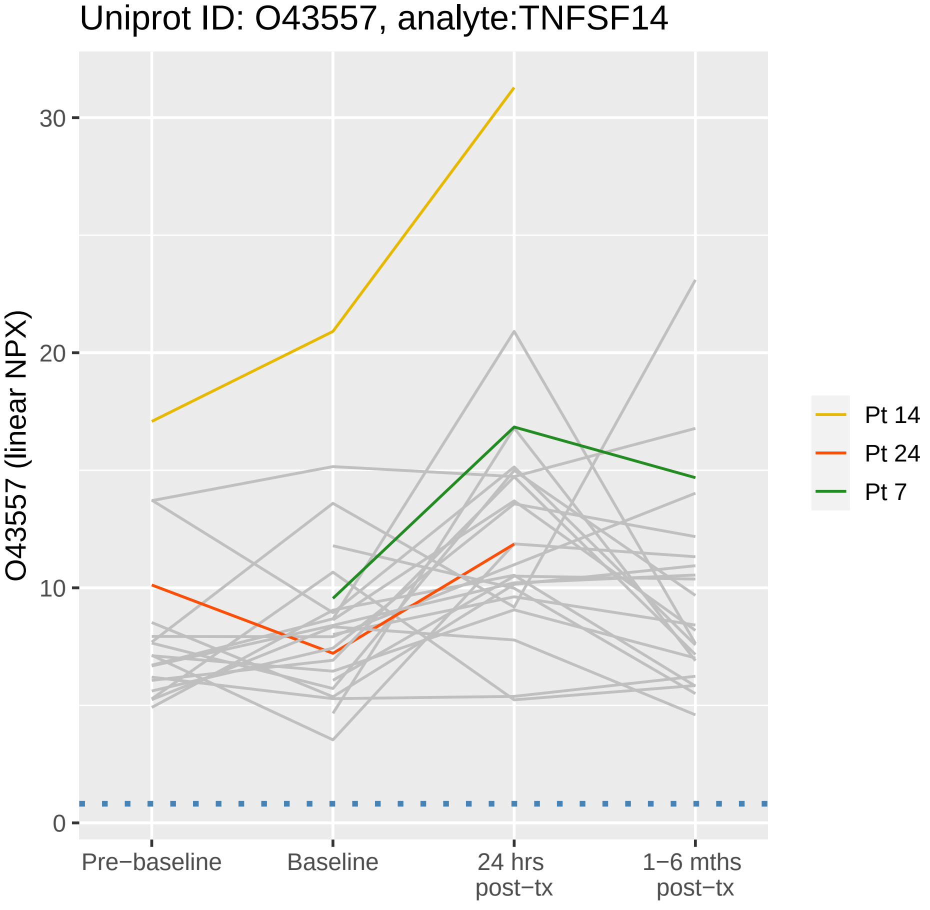

Uniprot ID: O43557, analyte:TNFSF14
O43557 (linear NPX)
30
20
10
0
Pre−baseline
Baseline
24 hrs post−tx
1−6 mths post−tx
Pt 14
Pt 24
Pt 7

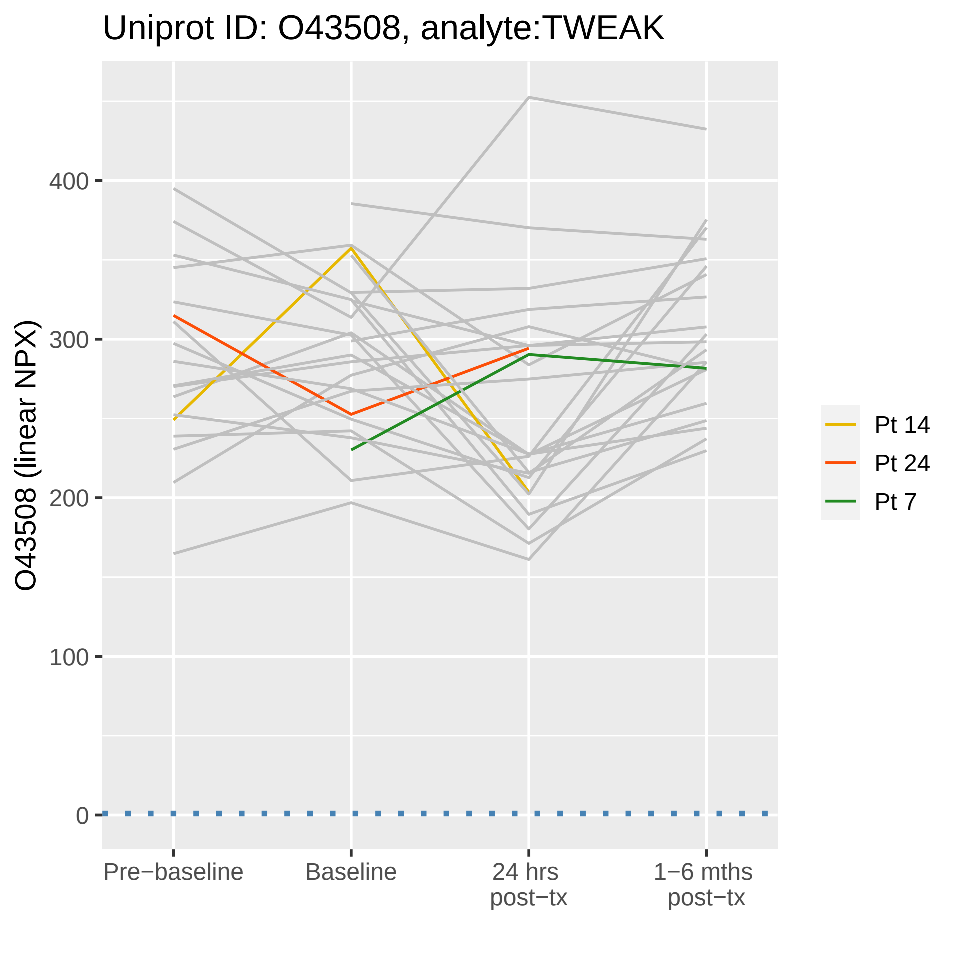
Uniprot ID: O43508, analyte:TWEAK
O43508 (linear NPX)
400
300
200
100
0
Pre−baseline
Baseline
24 hrs post−tx
1−6 mths post−tx
Pt 14
Pt 24
Pt 7

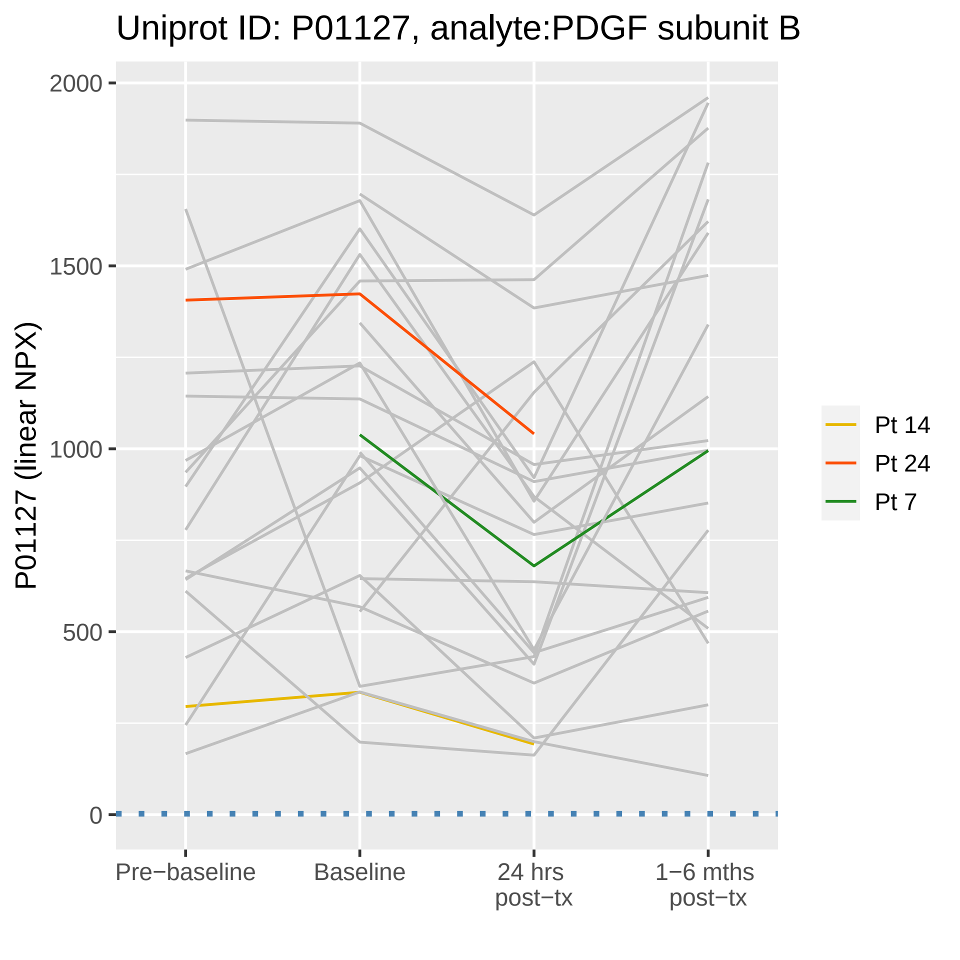
Uniprot ID: P01127, analyte:PDGF subunit B
P01127 (linear NPX)
2000
1500
1000
500
0
Pre−baseline
Baseline
24 hrs
post−tx
1−6 mths
post−tx
Pt 14
Pt 24
Pt 7

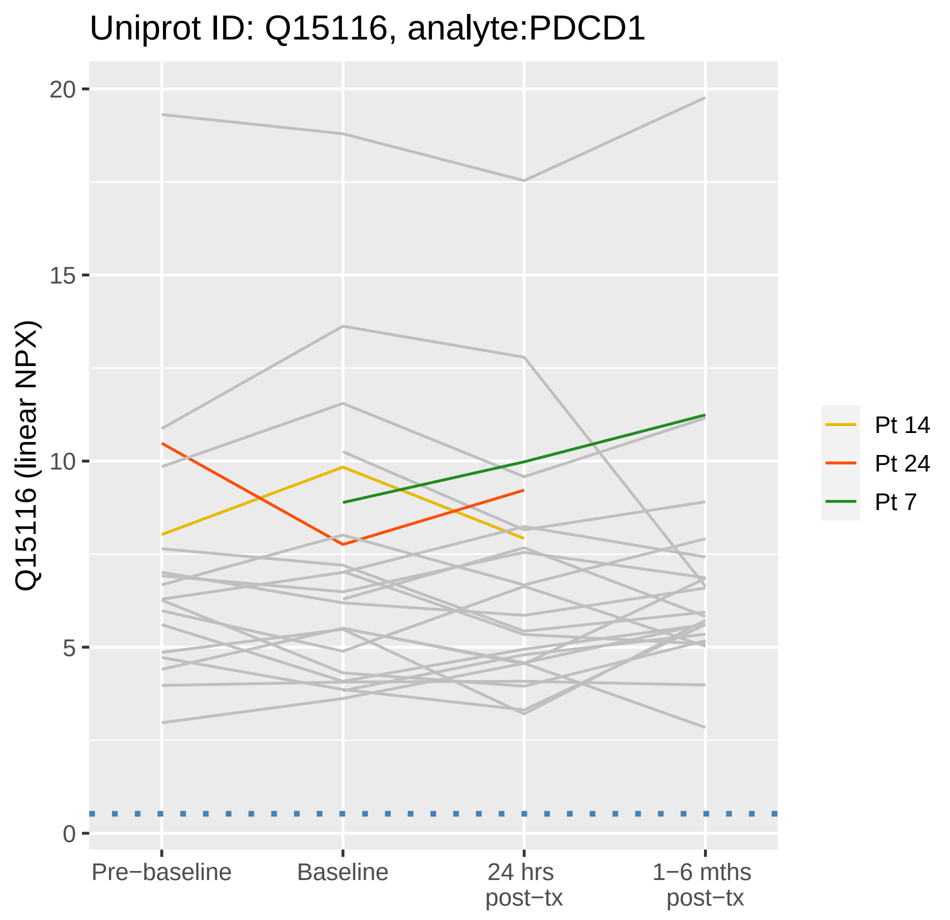

Uniprot ID: Q15116, analyte:PDCD1
Q15116 (linear NPX)
20
15
10
5
0
Pre−baseline
Baseline
24 hrs post−tx
1−6 mths post−tx
Pt 14
Pt 24
Pt 7

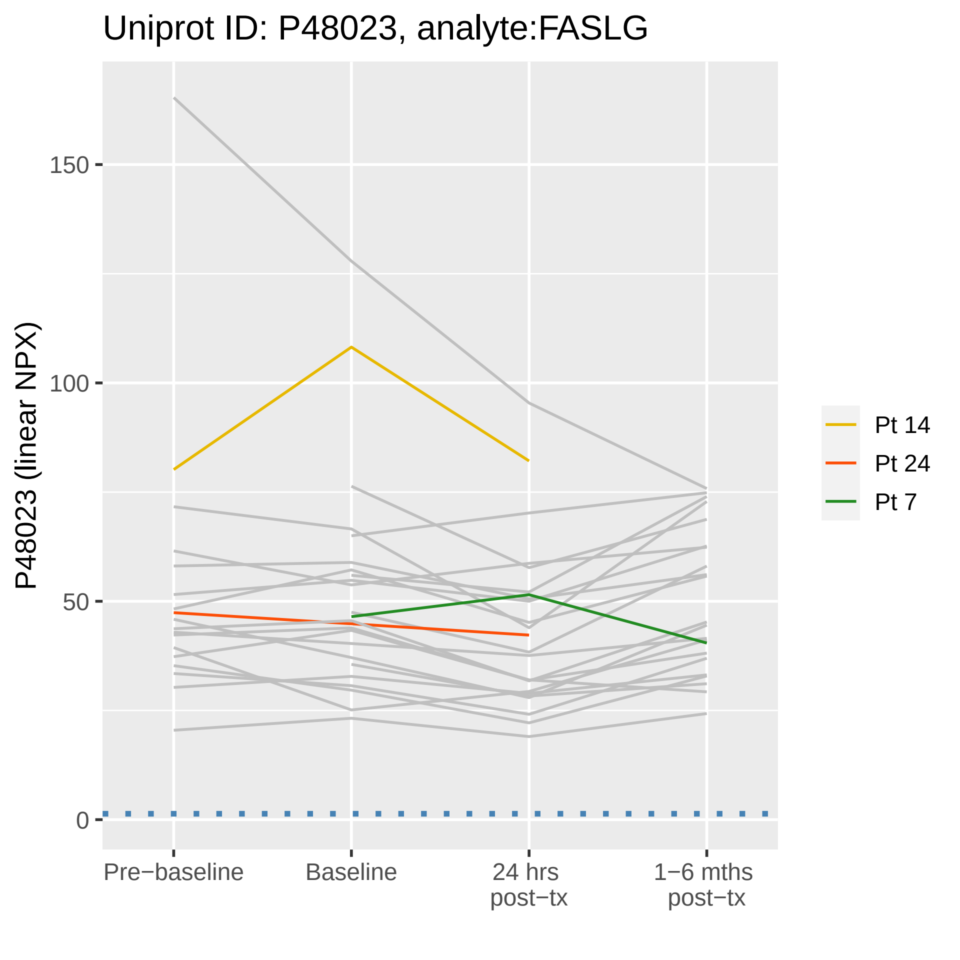

Uniprot ID: P48023, analyte:FASLG
P48023 (linear NPX)
150
100
50
0
Pre−baseline
Baseline
24 hrs post−tx
1−6 mths post−tx
Pt 14
Pt 24
Pt 7

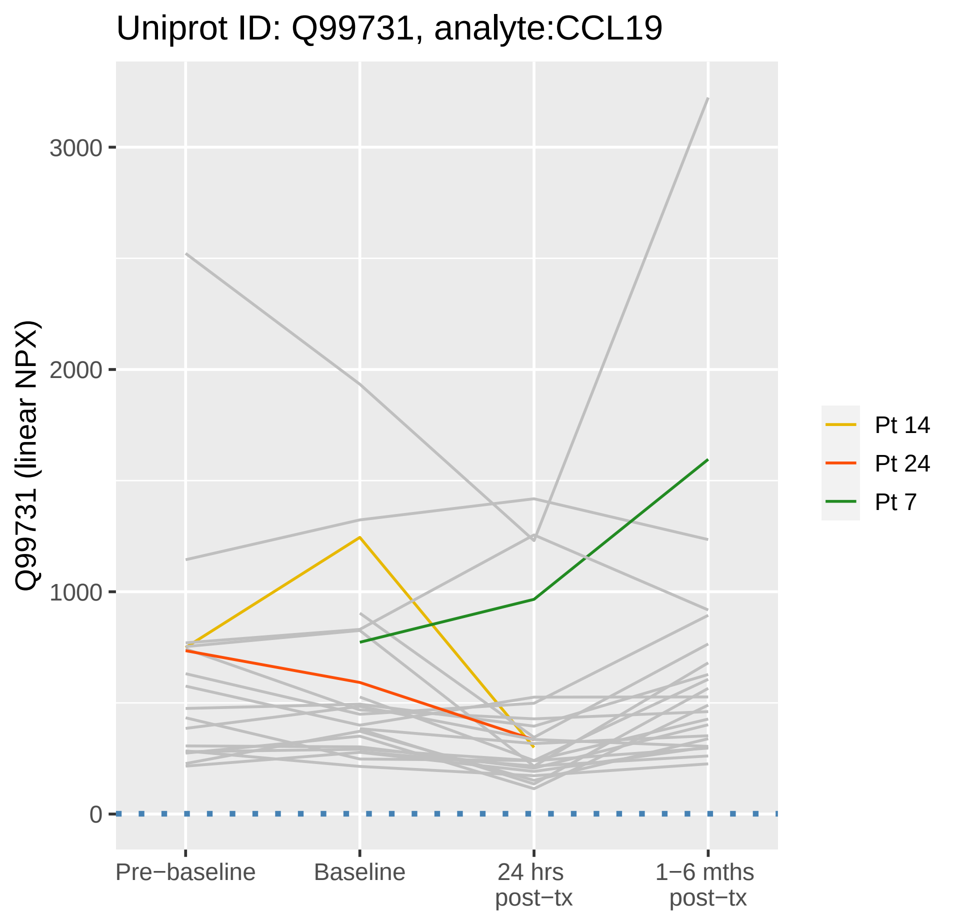

Uniprot ID: Q99731, analyte:CCL19
Q99731 (linear NPX)
3000
2000
1000
0
Pre−baseline
Baseline
24 hrs post−tx
1−6 mths post−tx
Pt 14
Pt 24
Pt 7

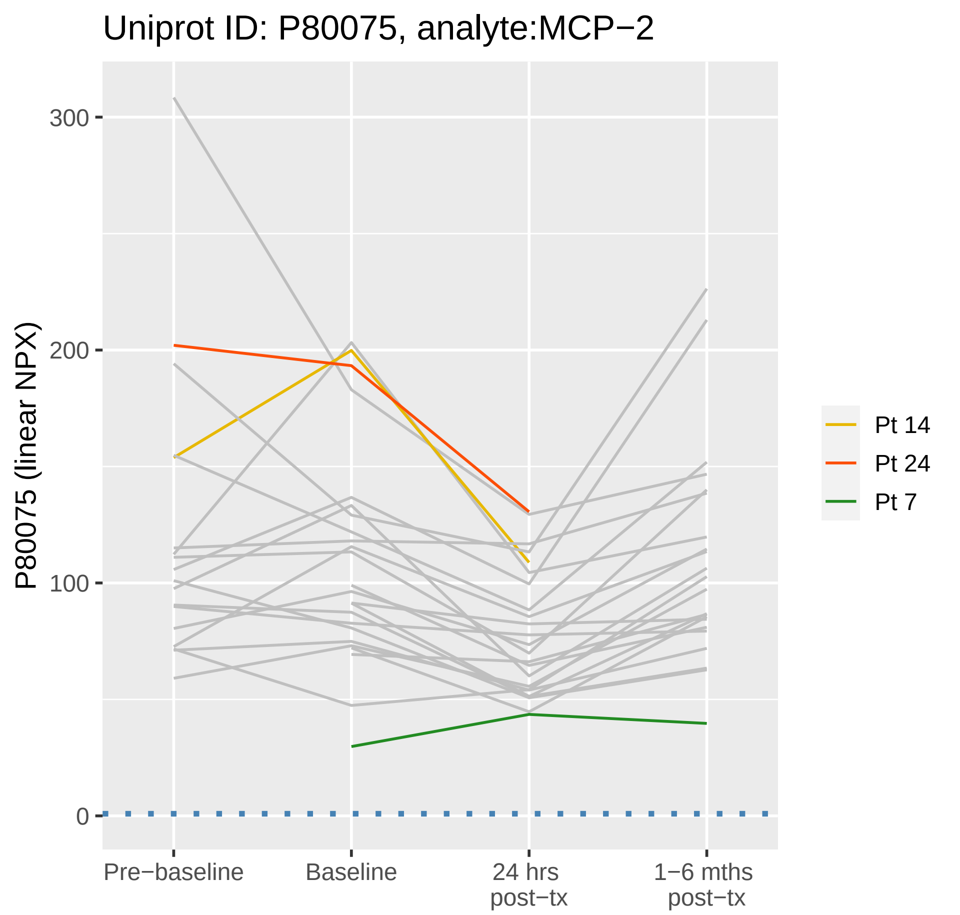
Uniprot ID: P80075, analyte:MCP−2
P80075 (linear NPX)
300
200
100
0
Pre−baseline
Baseline
24 hrs post−tx
1−6 mths post−tx
Pt 14
Pt 24
Pt 7

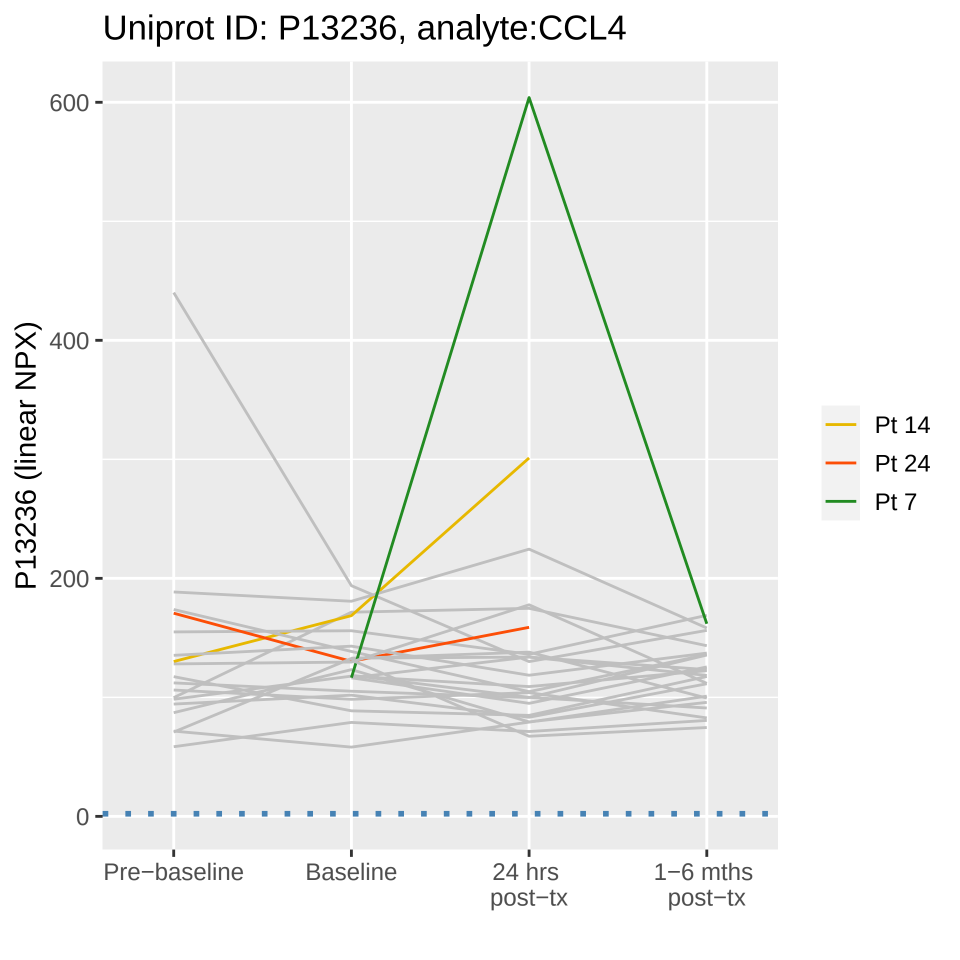

Uniprot ID: P13236, analyte:CCL4
P13236 (linear NPX)
600
400
200
0
Pre−baseline
Baseline
24 hrs post−tx
1−6 mths post−tx
Pt 14
Pt 24
Pt 7

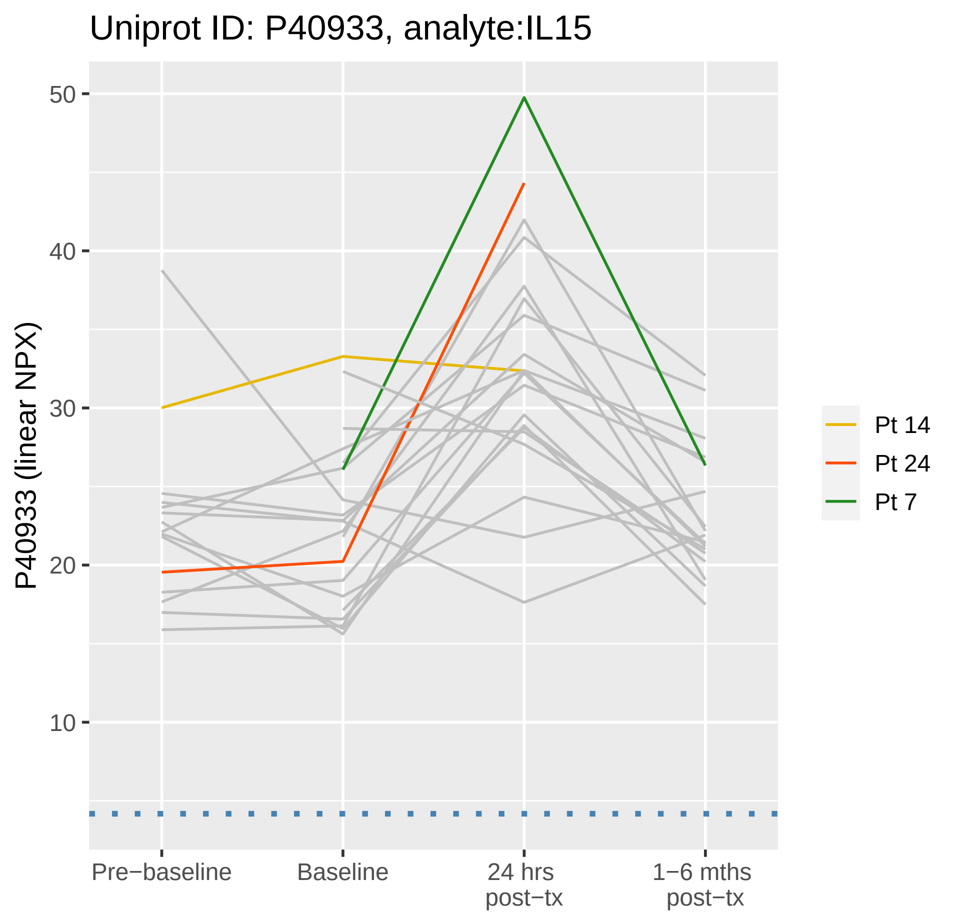

Uniprot ID: P40933, analyte:IL15
P40933 (linear NPX)
50
40
30
20
10
Pre−baseline
Baseline
24 hrs
post−tx
1−6 mths
post−tx
Pt 14
Pt 24
Pt 7

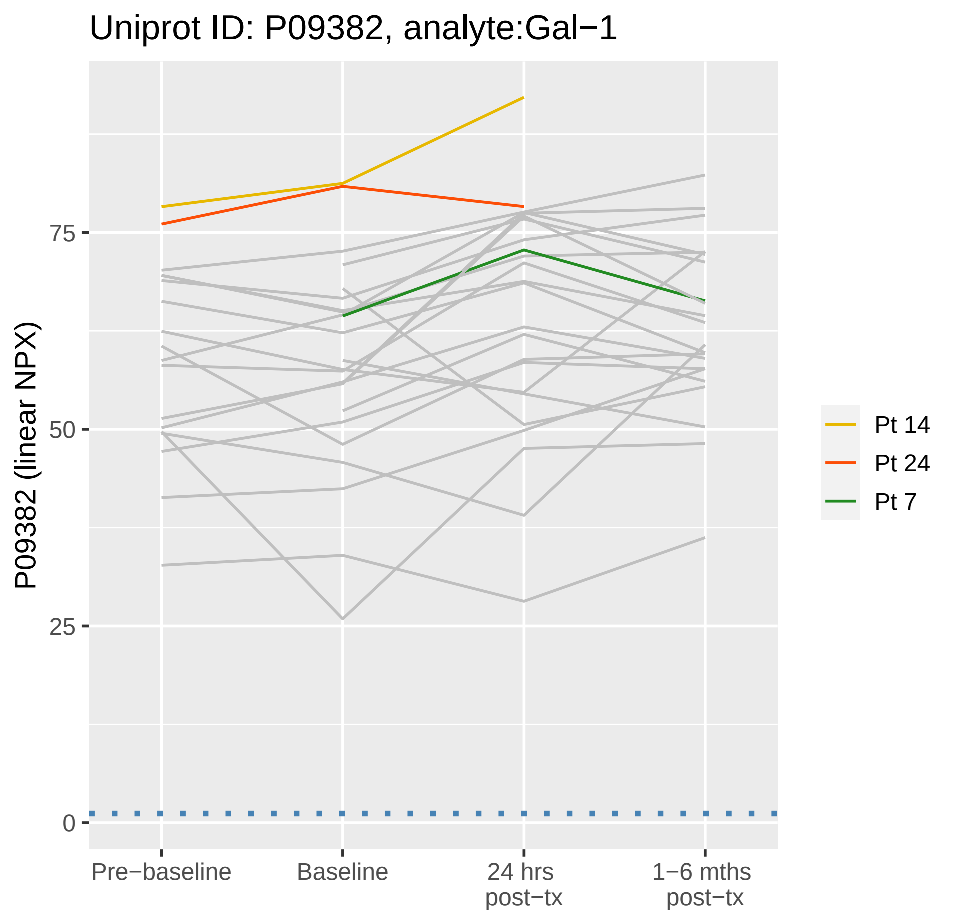

Uniprot ID: P09382, analyte:Gal−1
P09382 (linear NPX)
75
50
25
0
Pre−baseline
Baseline
24 hrs post−tx
1−6 mths post−tx
Pt 14
Pt 24
Pt 7

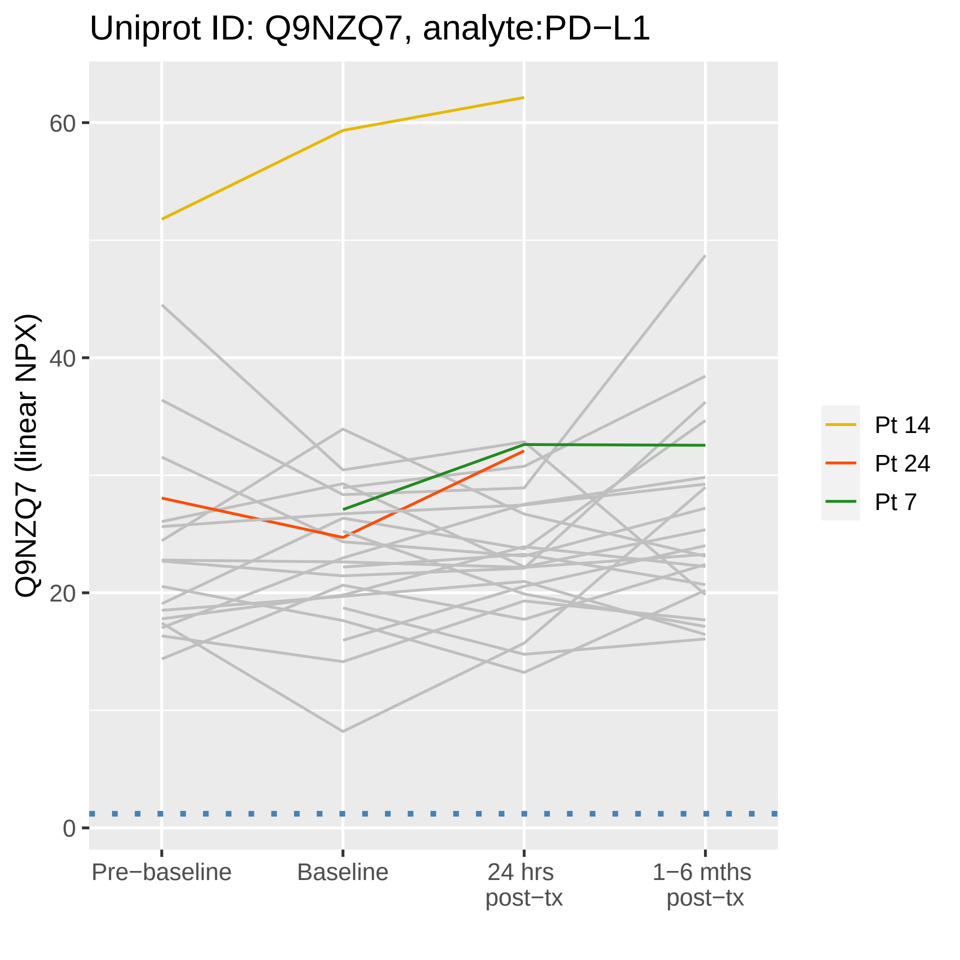

Uniprot ID: Q9NZQ7, analyte:PD−L1
Q9NZQ7 (linear NPX)
60
40
20
0
Pre−baseline
Baseline
24 hrs post−tx
1−6 mths post−tx
Pt 14
Pt 24
Pt 7

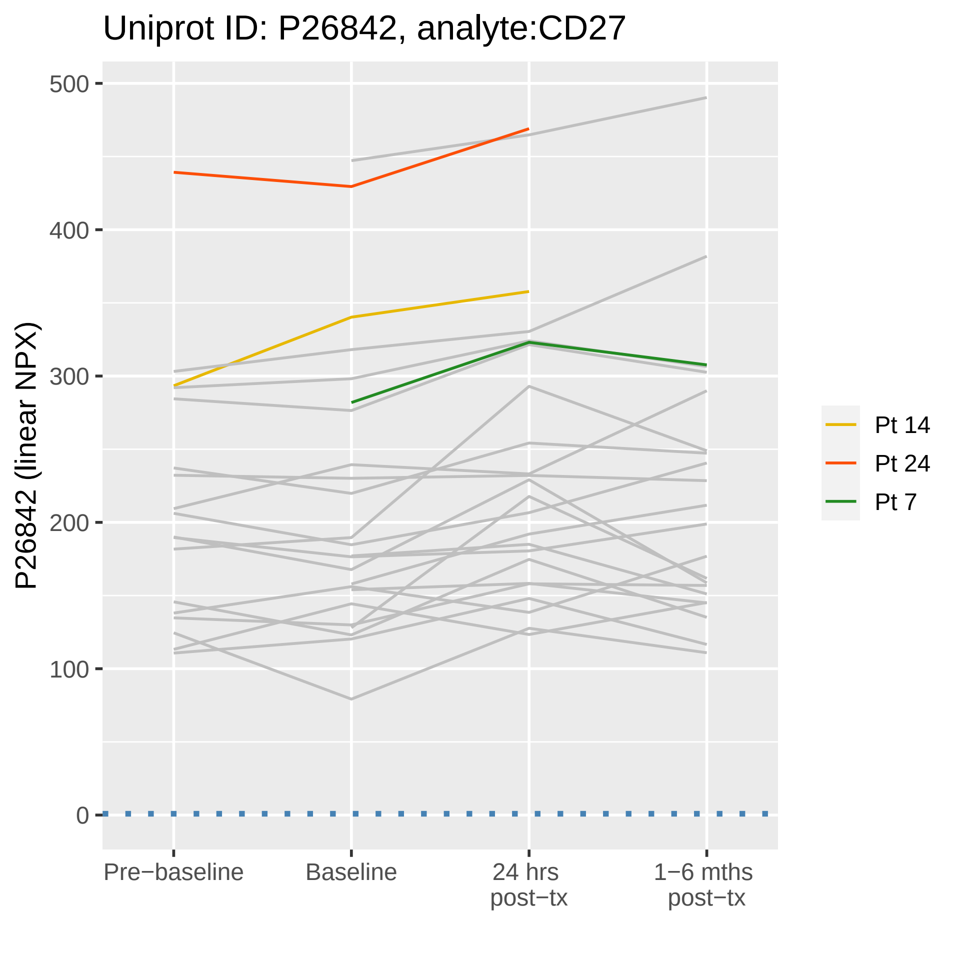
Uniprot ID: P26842, analyte:CD27
P26842 (linear NPX)
500
400
300
200
100
0
Pre−baseline
Baseline
24 hrs post−tx
1−6 mths post−tx
Pt 14
Pt 24
Pt 7

Uniprot ID: P42830, analyte:CXCL5

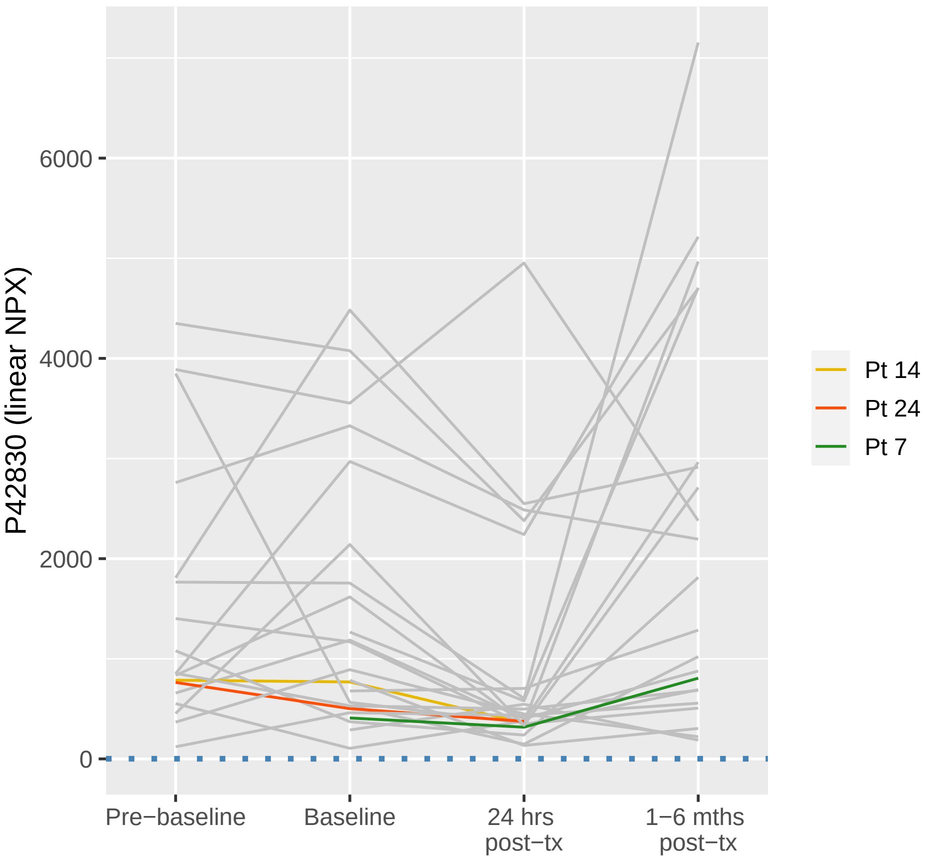

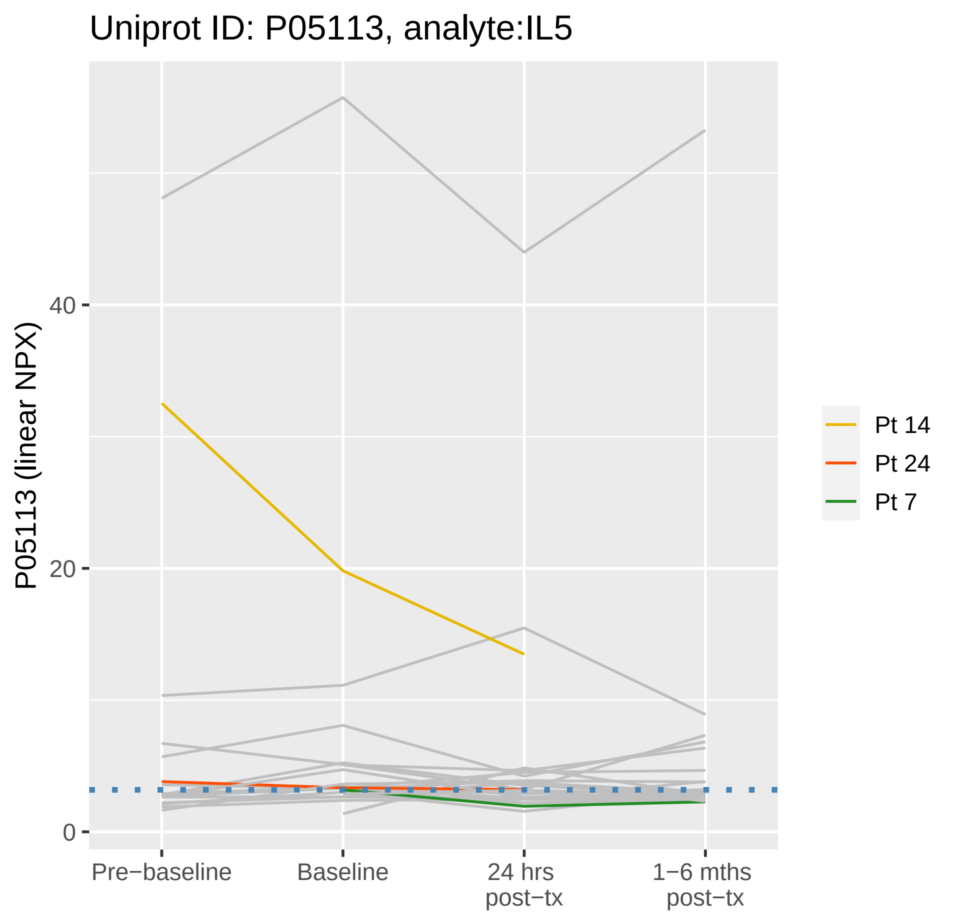

Uniprot ID: P05113, analyte:IL5
P05113 (linear NPX)
40
20
0
Pre−baseline
Baseline
24 hrs post−tx
1−6 mths post−tx
Pt 14
Pt 24
Pt 7

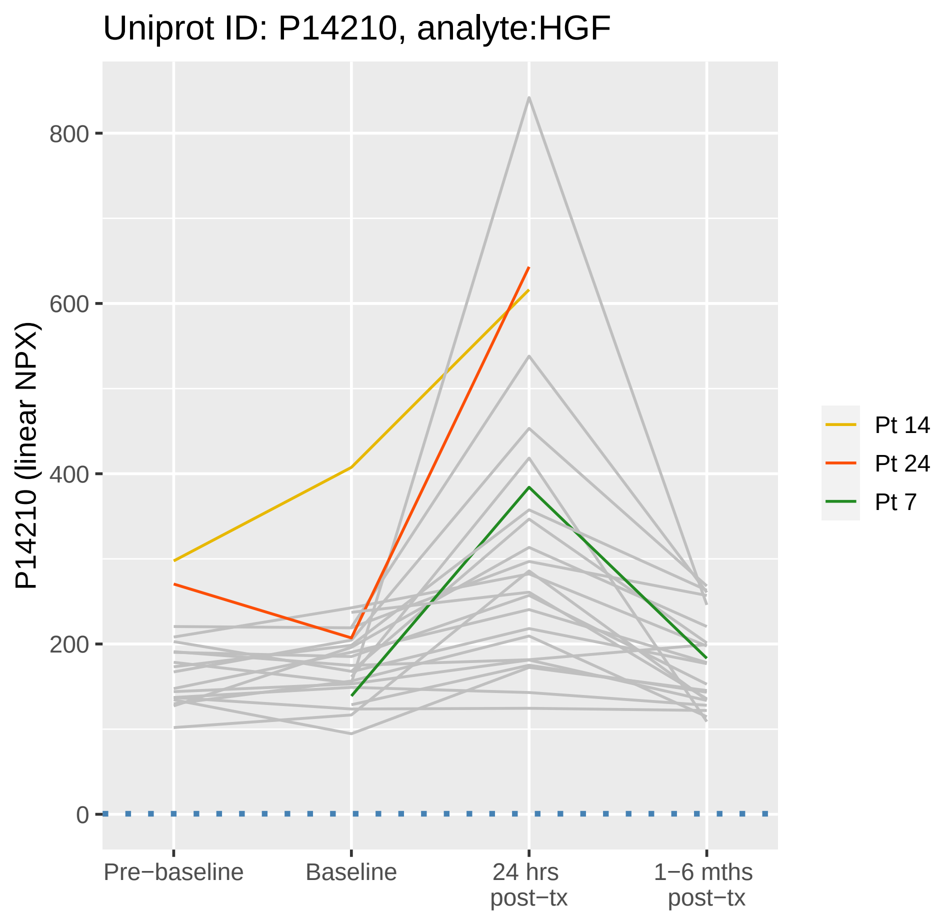
Uniprot ID: P14210, analyte:HGF
P14210 (linear NPX)
800
600
400
200
0
Pre−baseline
Baseline
24 hrs post−tx
1−6 mths post−tx
Pt 14
Pt 24
Pt 7

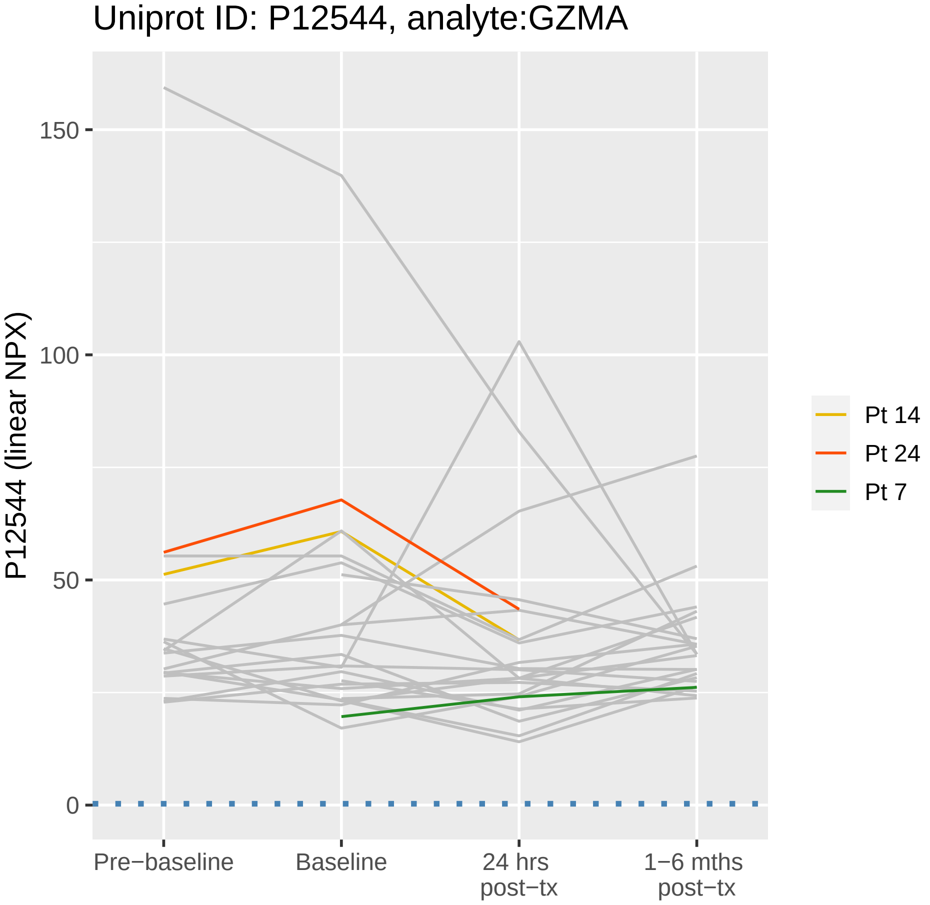

Uniprot ID: P12544, analyte:GZMA
P12544 (linear NPX)
150
100
50
0
Pre−baseline
Baseline
24 hrs post−tx
1−6 mths post−tx
Pt 14
Pt 24
Pt 7

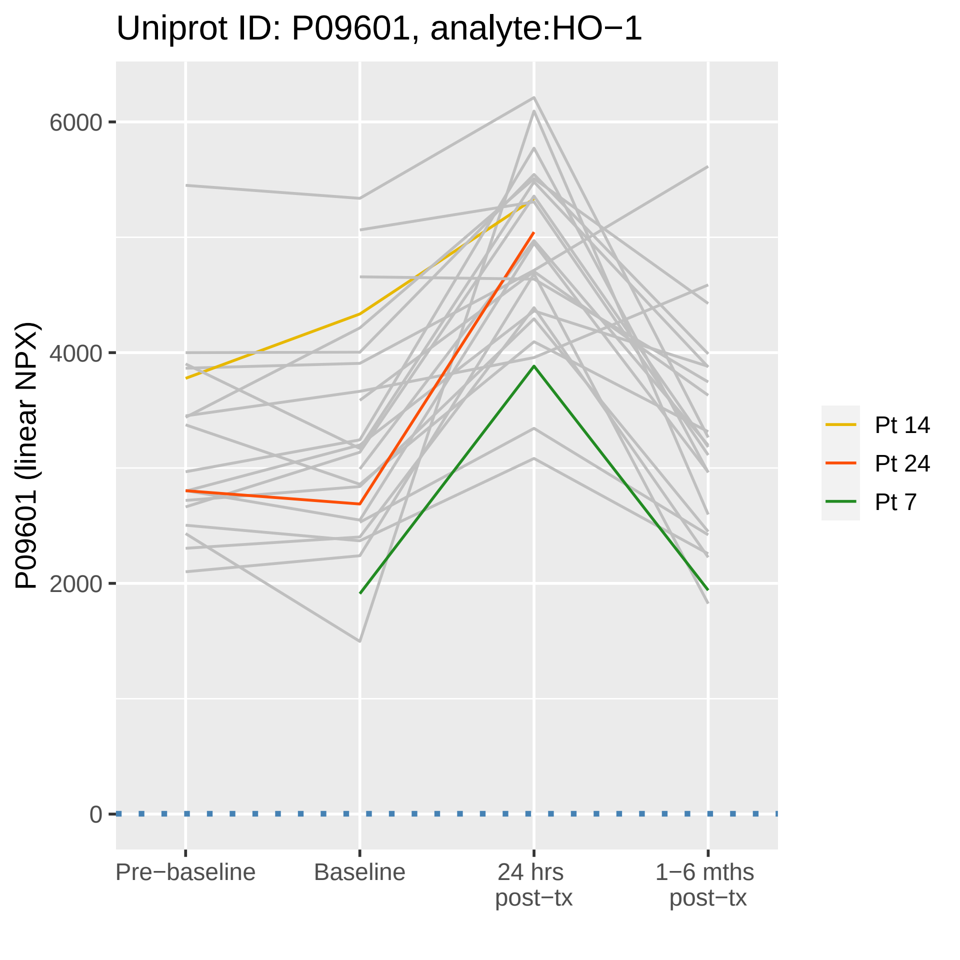

Uniprot ID: P09601, analyte:HO−1
P09601 (linear NPX)
6000
4000
2000
0
Pre−baseline
Baseline
24 hrs post−tx
1−6 mths post−tx
Pt 14
Pt 24
Pt 7

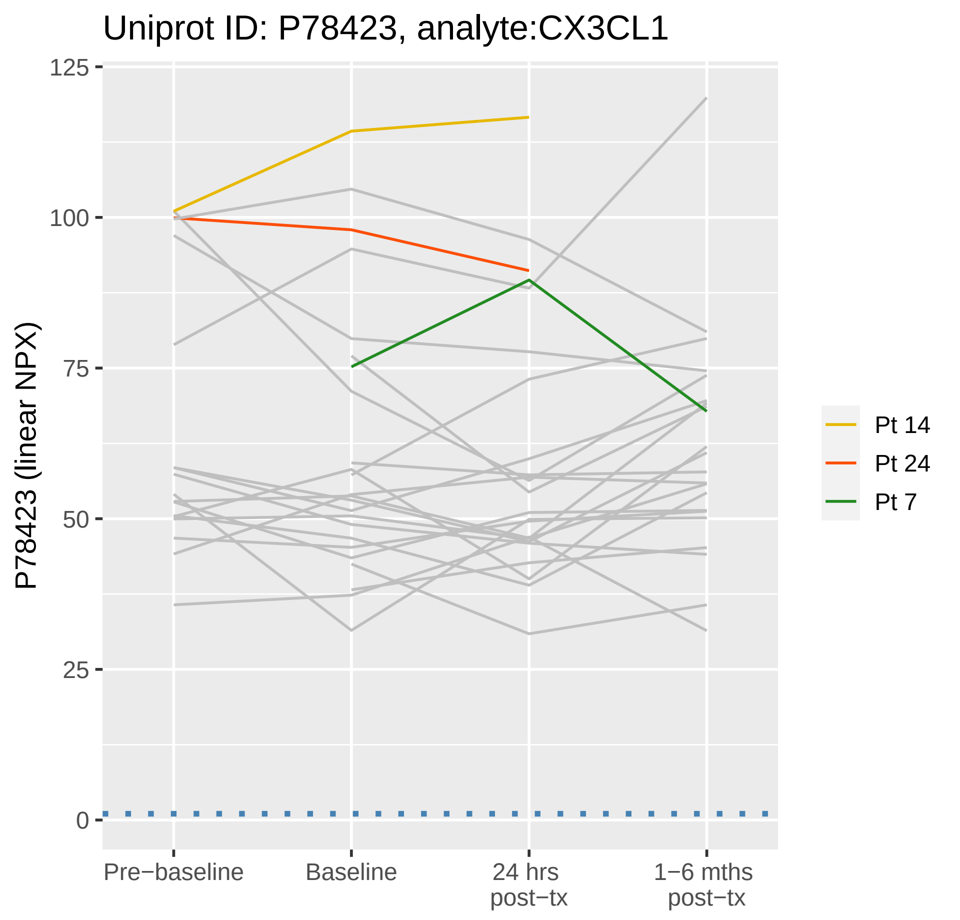

Uniprot ID: P78423, analyte:CX3CL1
P78423 (linear NPX)
125
100
75
50
25
0
Pre−baseline
Baseline
24 hrs post−tx
1−6 mths post−tx
Pt 14
Pt 24
Pt 7

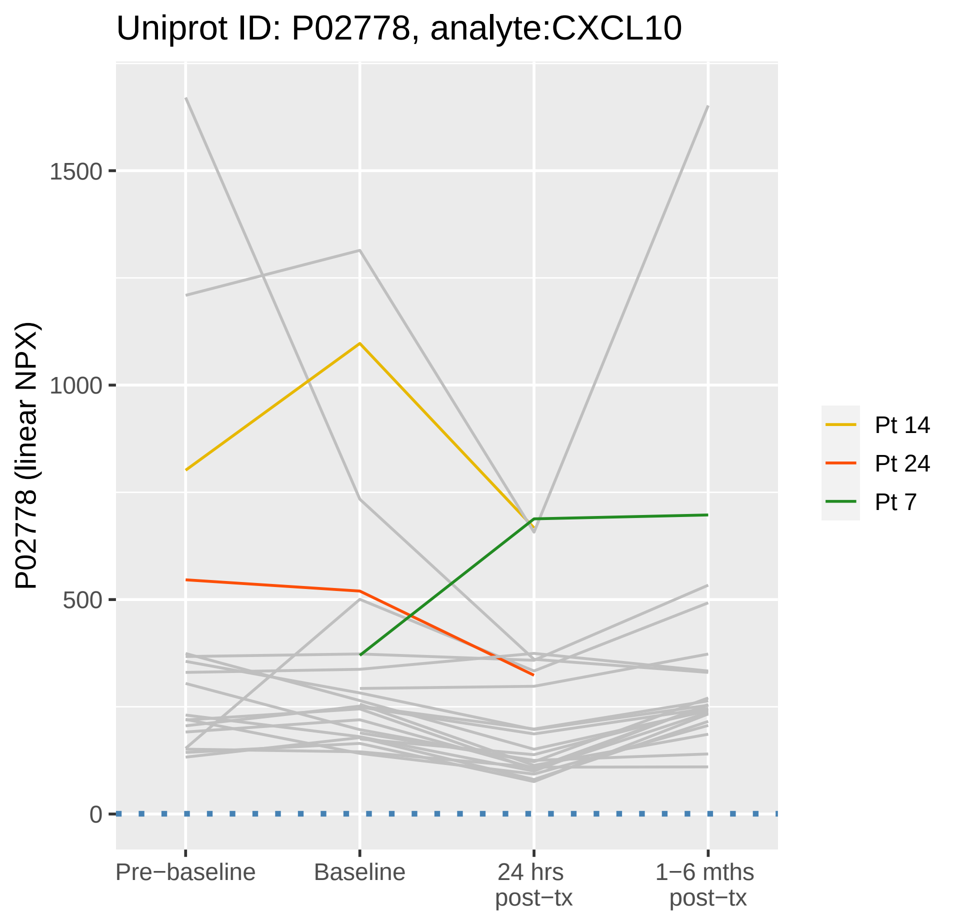

Uniprot ID: P02778, analyte:CXCL10
P02778 (linear NPX)
1500
1000
500
0
Pre−baseline
Baseline
24 hrs
post−tx
1−6 mths
post−tx
Pt 14
Pt 24
Pt 7

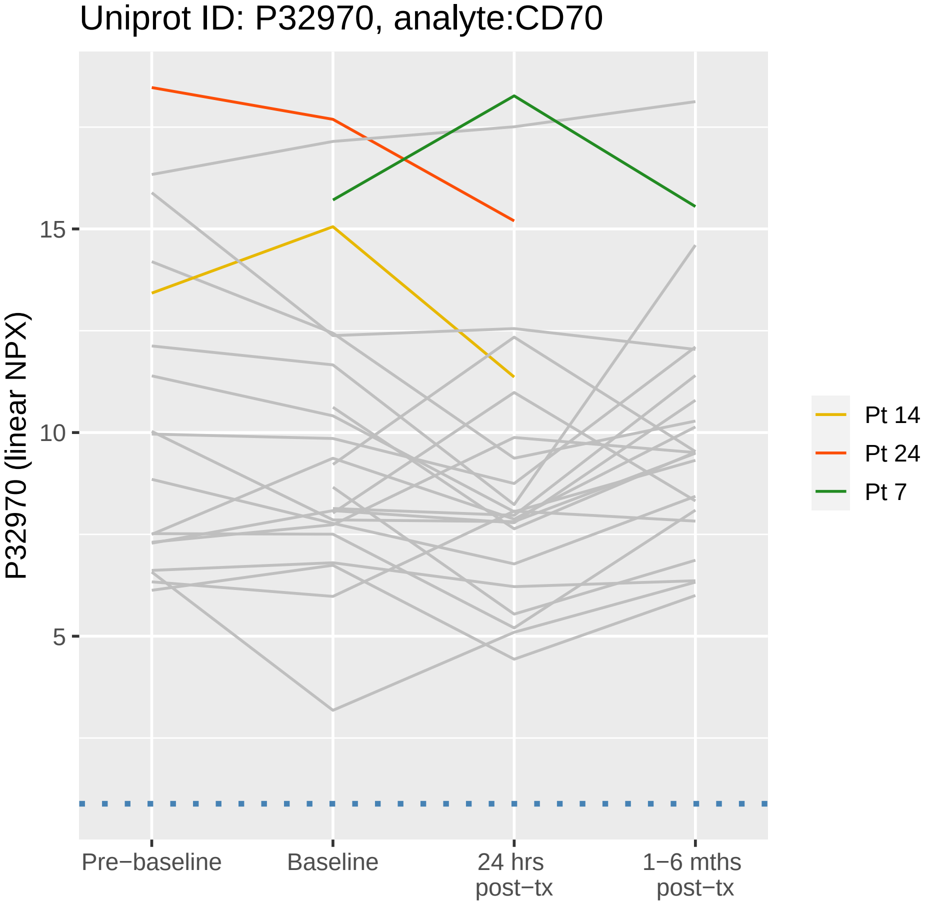
Uniprot ID: P32970, analyte:CD70
P32970 (linear NPX)
15
10
5
Pre−baseline
Baseline
24 hrs post−tx
1−6 mths post−tx
Pt 14
Pt 24
Pt 7

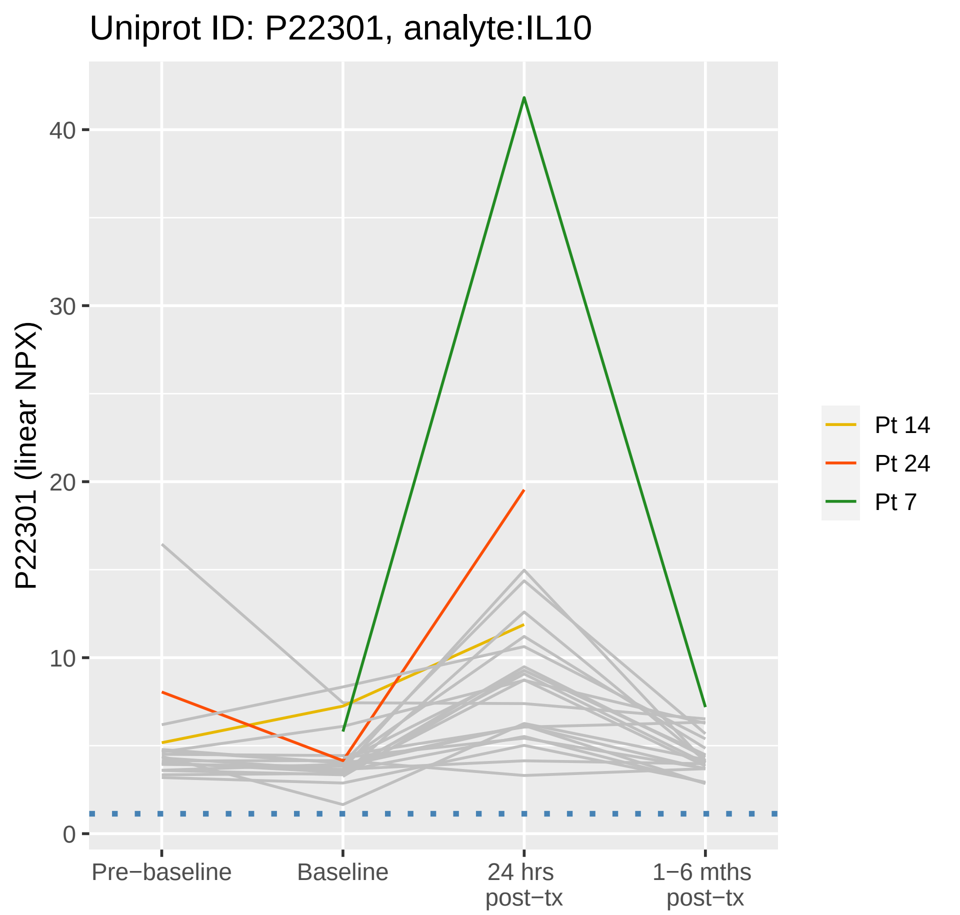
Uniprot ID: P22301, analyte:IL10
P22301 (linear NPX)
0
10
20
30
40
Pre−baseline
Baseline
24 hrs post−tx
1−6 mths post−tx
Pt 14
Pt 24
Pt 7

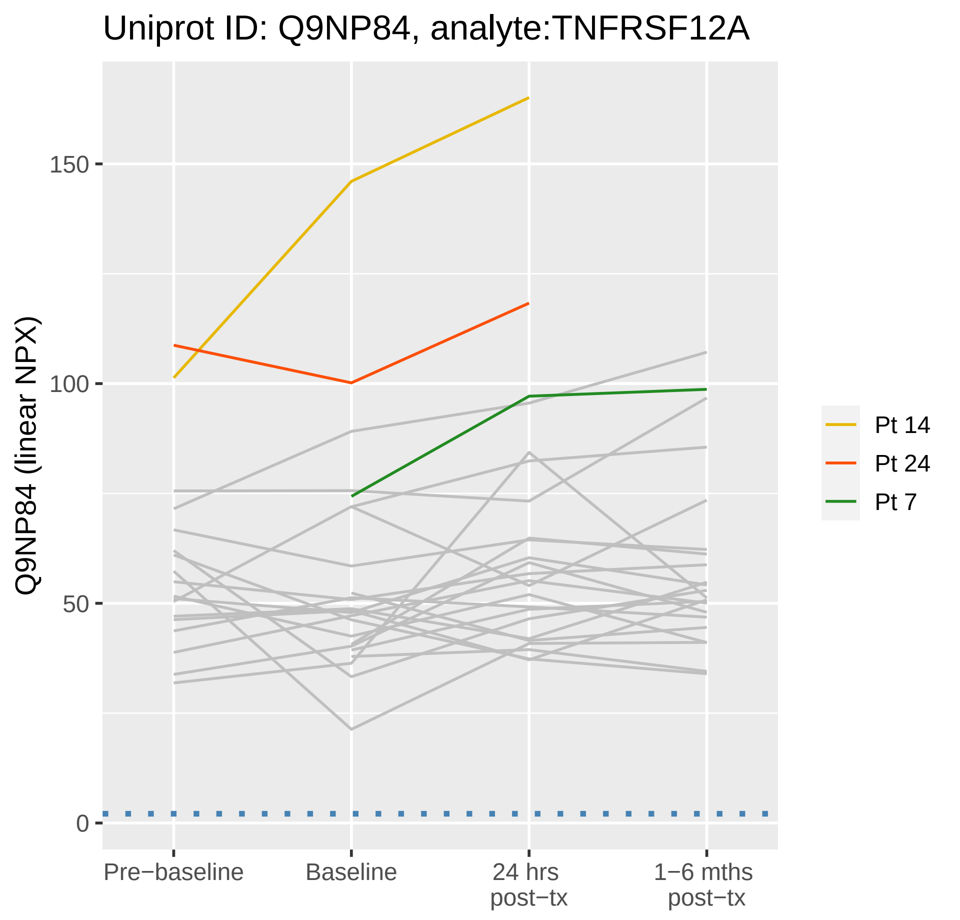
Uniprot ID: Q9NP84, analyte:TNFRSF12A
Q9NP84 (linear NPX)
150
100
50
0
Pre−baseline
Baseline
24 hrs post−tx
1−6 mths post−tx
Pt 14
Pt 24
Pt 7

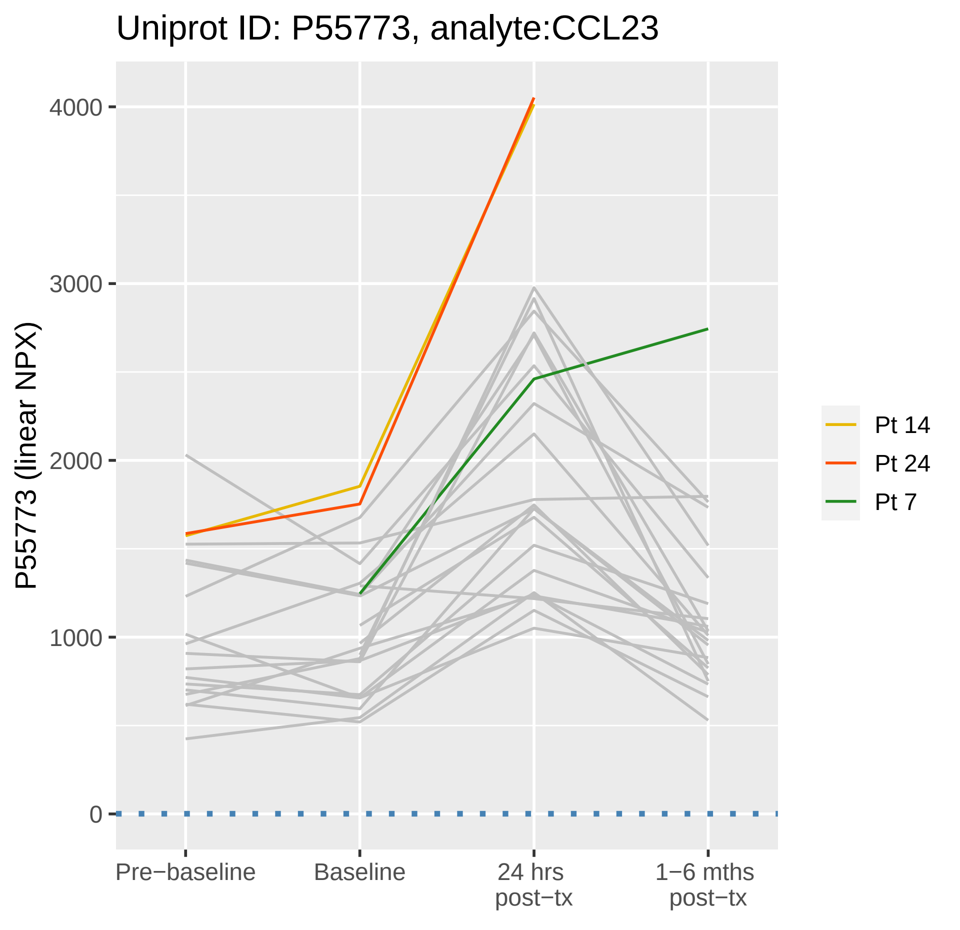

Uniprot ID: P55773, analyte:CCL23
P55773 (linear NPX)
4000
3000
2000
1000
0
Pre−baseline
Baseline
24 hrs
post−tx
1−6 mths
post−tx
Pt 14
Pt 24
Pt 7

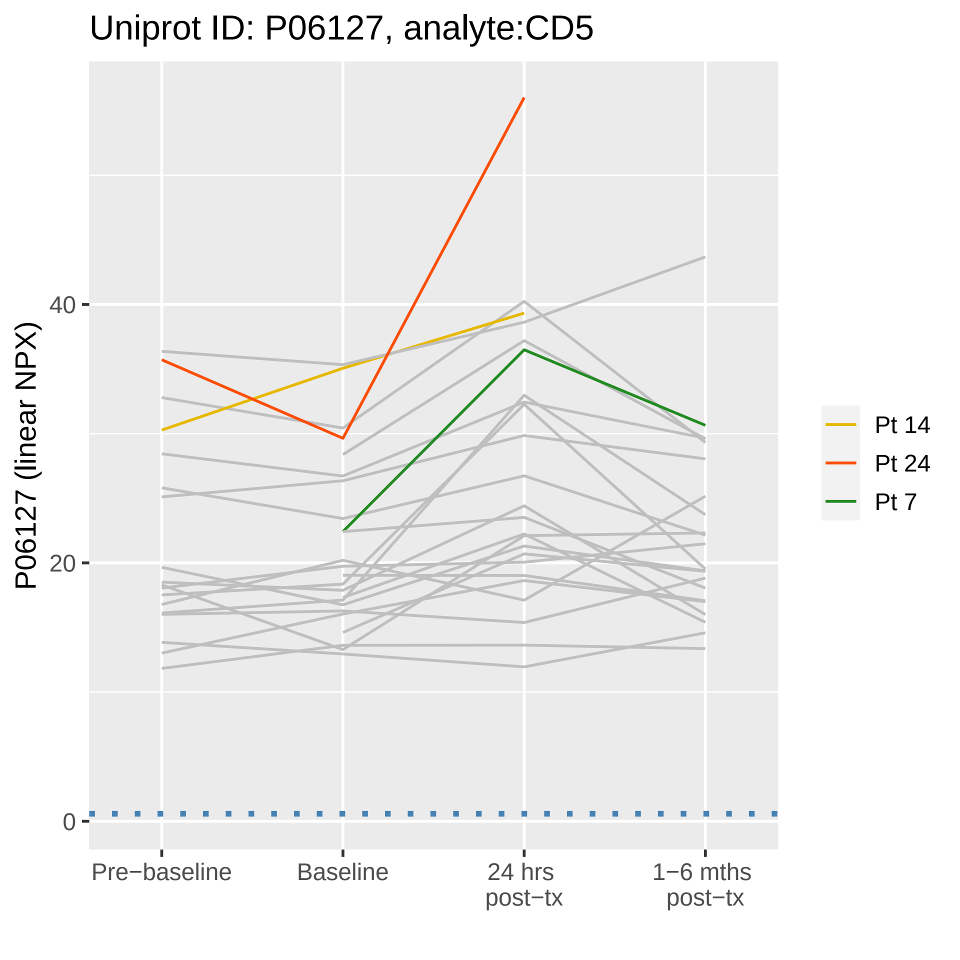

Uniprot ID: P06127, analyte:CD5
P06127 (linear NPX)
0
20
40
Pre−baseline
Baseline
24 hrs post−tx
1−6 mths post−tx
Pt 14
Pt 24
Pt 7

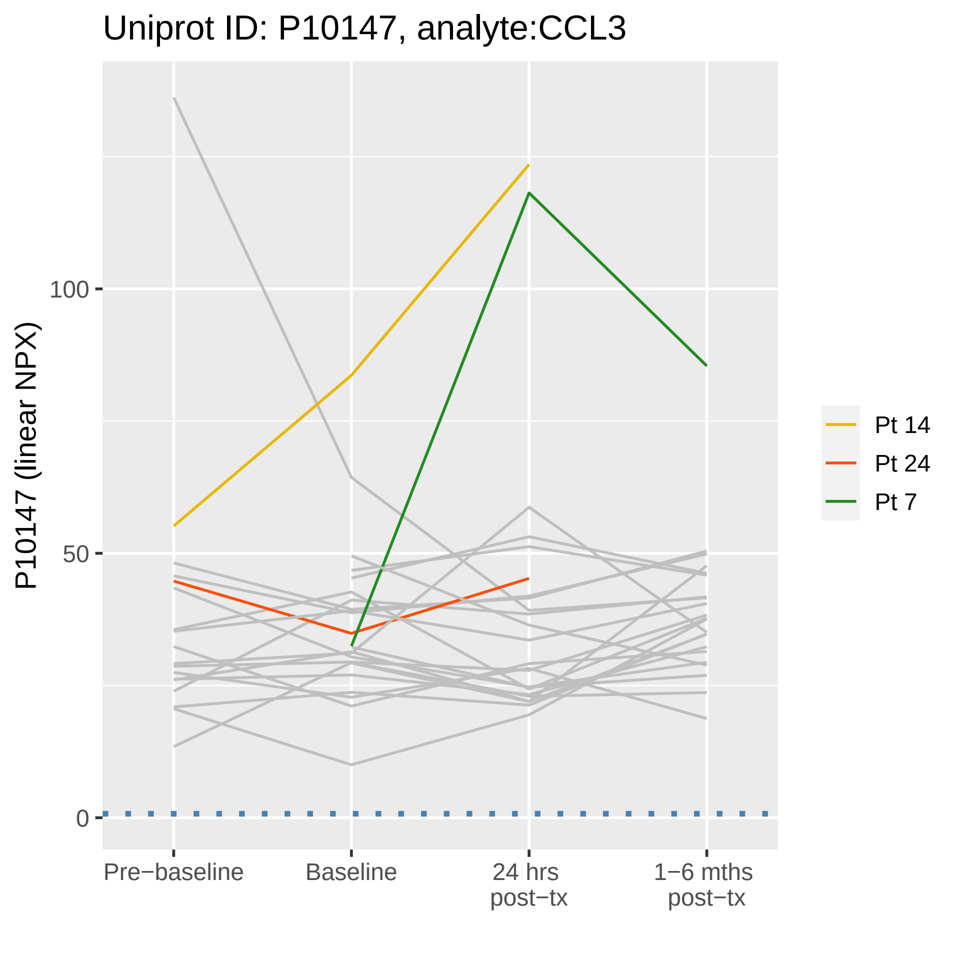

Uniprot ID: P10147, analyte:CCL3
P10147 (linear NPX)
100
50
0
Pre−baseline
Baseline
24 hrs post−tx
1−6 mths post−tx
Pt 14
Pt 24
Pt 7

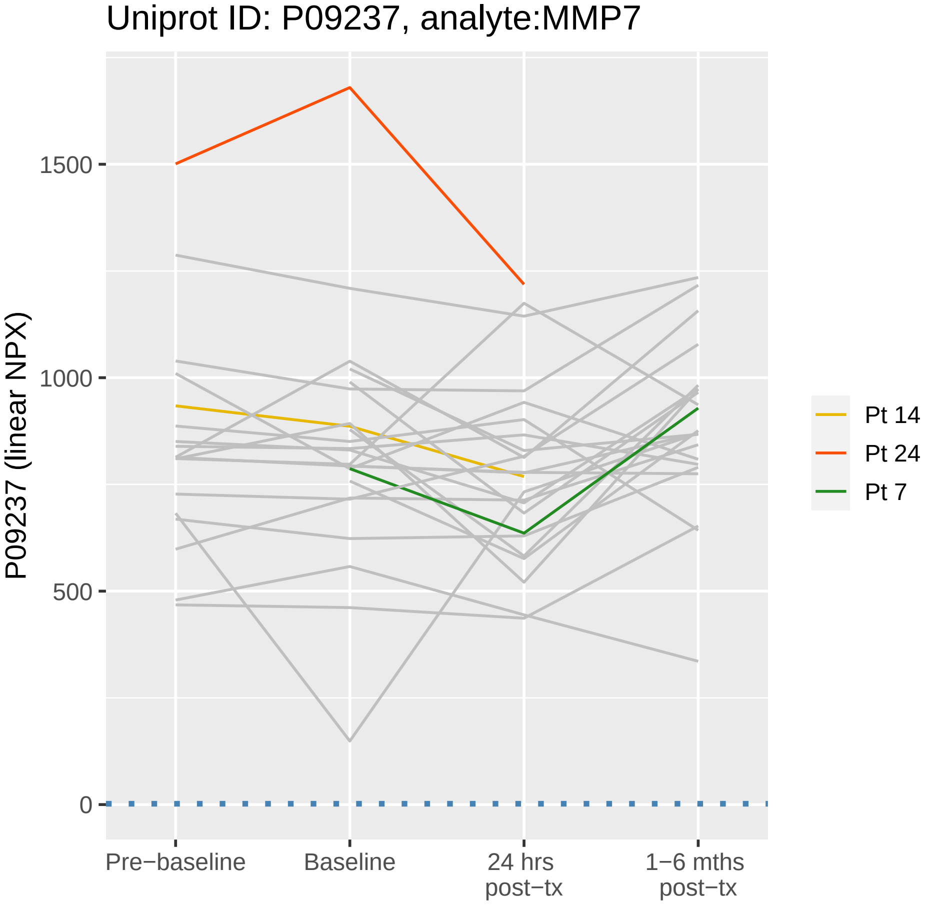

Uniprot ID: P09237, analyte:MMP7
P09237 (linear NPX)
1500
1000
500
0
Pre−baseline
Baseline
24 hrs post−tx
1−6 mths post−tx
Pt 14
Pt 24
Pt 7

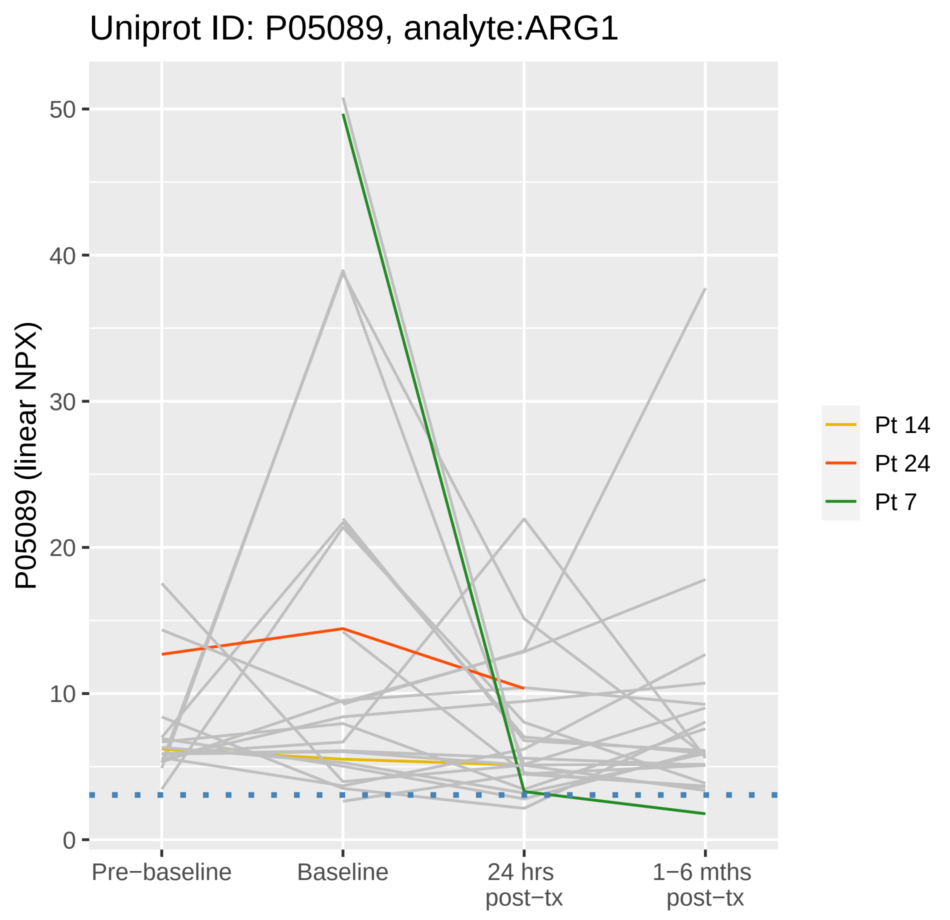
Uniprot ID: P05089, analyte:ARG1
P05089 (linear NPX)
0
10
20
30
40
50
Pre−baseline
Baseline
24 hrs post−tx
1−6 mths post−tx
Pt 14
Pt 24
Pt 7

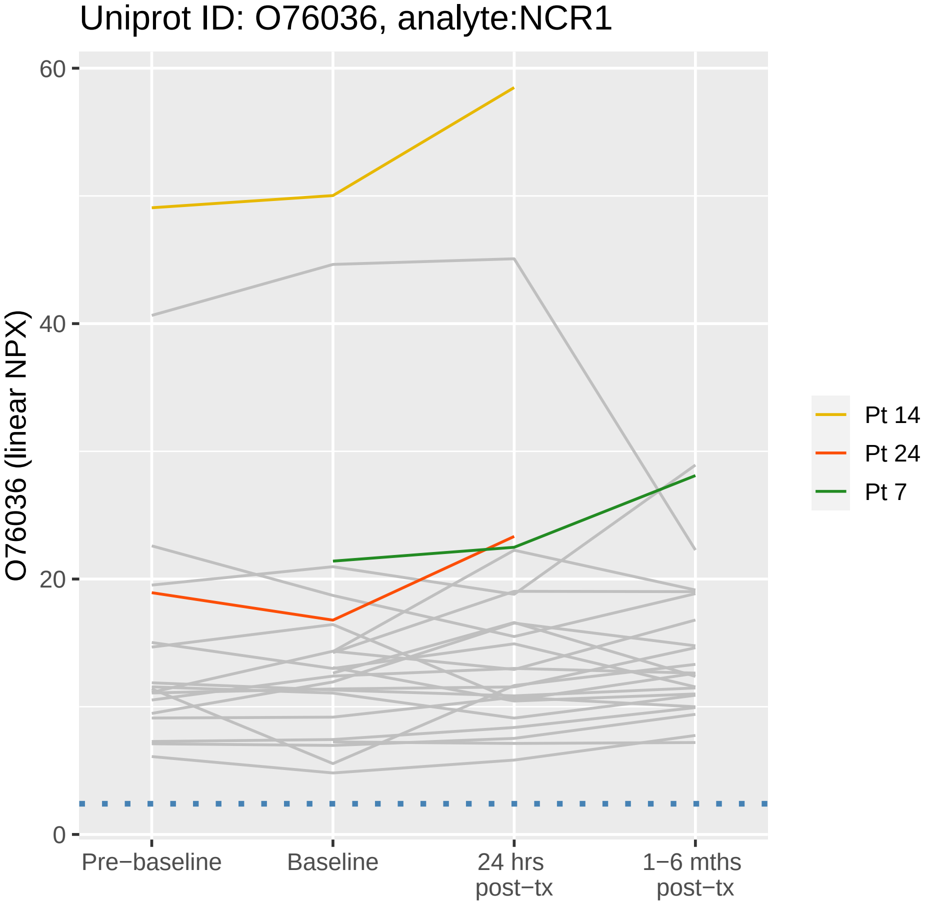
Uniprot ID: O76036, analyte:NCR1
O76036 (linear NPX)
60
40
20
0
Pre−baseline
Baseline
24 hrs post−tx
1−6 mths post−tx
Pt 14
Pt 24
Pt 7

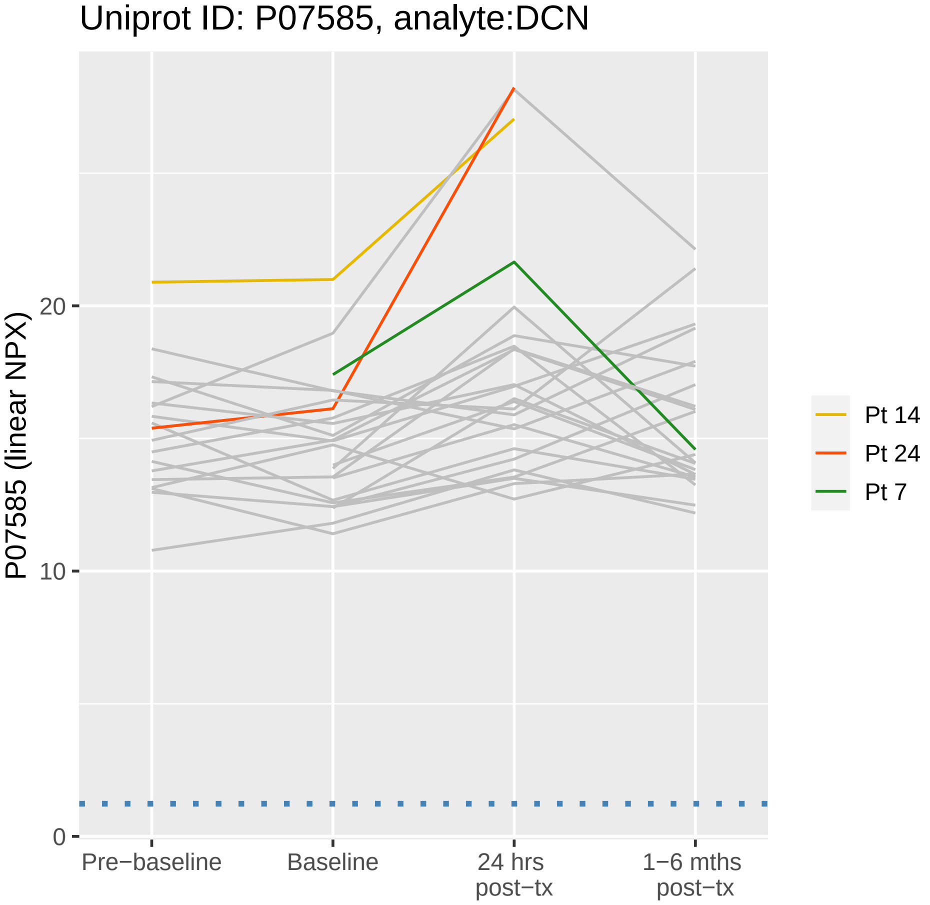

Uniprot ID: P07585, analyte:DCN
P07585 (linear NPX)
20
10
0
Pre−baseline
Baseline
24 hrs post−tx
1−6 mths post−tx
Pt 14
Pt 24
Pt 7

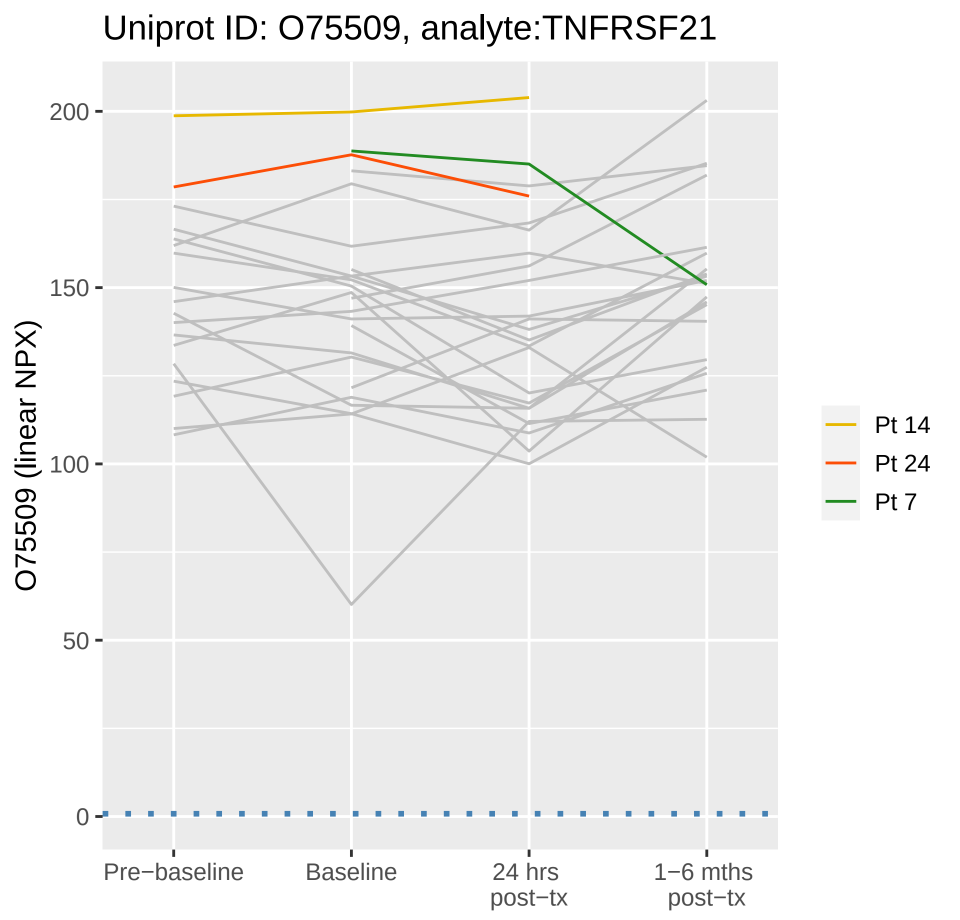

Uniprot ID: O75509, analyte:TNFRSF21
O75509 (linear NPX)
200
150
100
50
0
Pre−baseline
Baseline
24 hrs post−tx
1−6 mths post−tx
Pt 14
Pt 24
Pt 7

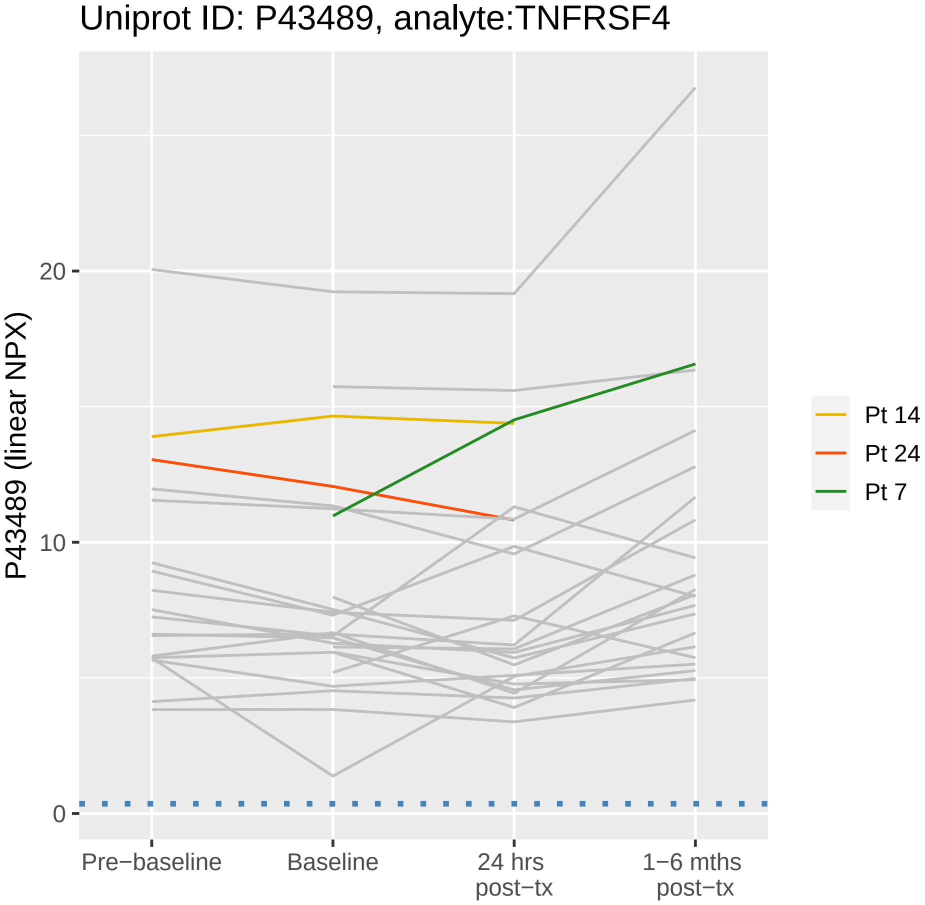

Uniprot ID: P43489, analyte:TNFRSF4
P43489 (linear NPX)
20
10
0
Pre−baseline
Baseline
24 hrs post−tx
1−6 mths post−tx
Pt 14
Pt 24
Pt 7

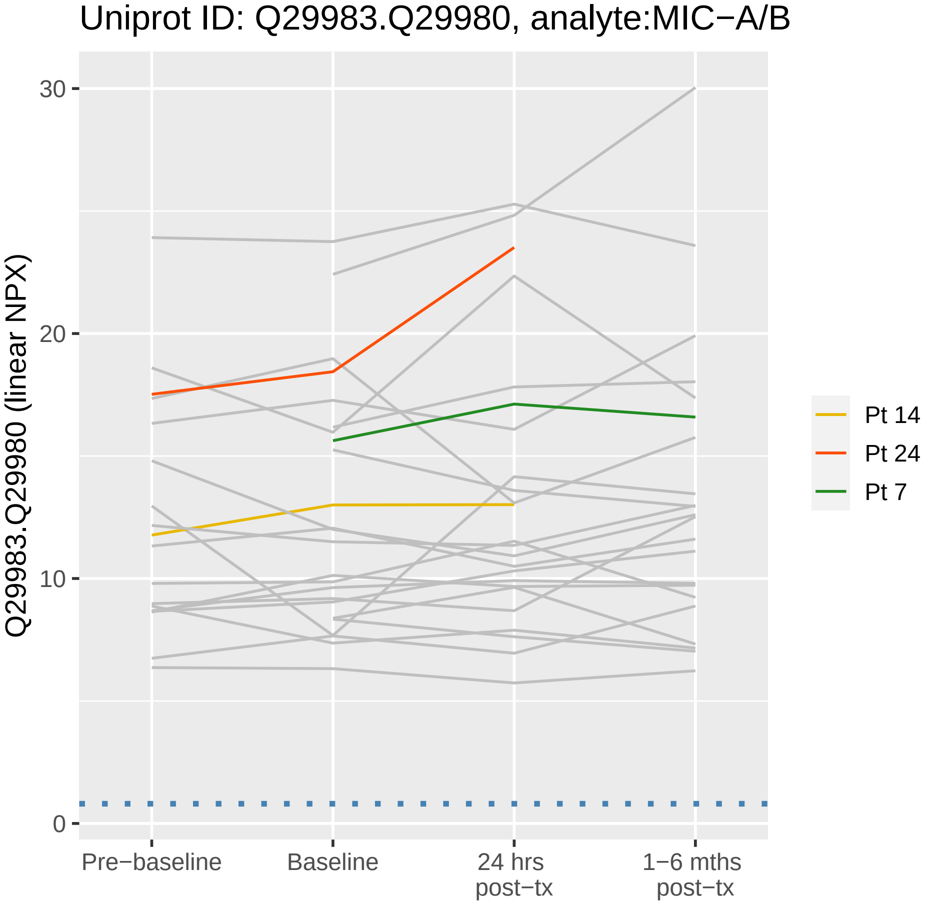

Uniprot ID: Q29983.Q29980, analyte:MIC−A/B
Q29983.Q29980 (linear NPX)
30
20
10
0
Pre−baseline
Baseline
24 hrs post−tx
1−6 mths post−tx
Pt 14
Pt 24
Pt 7

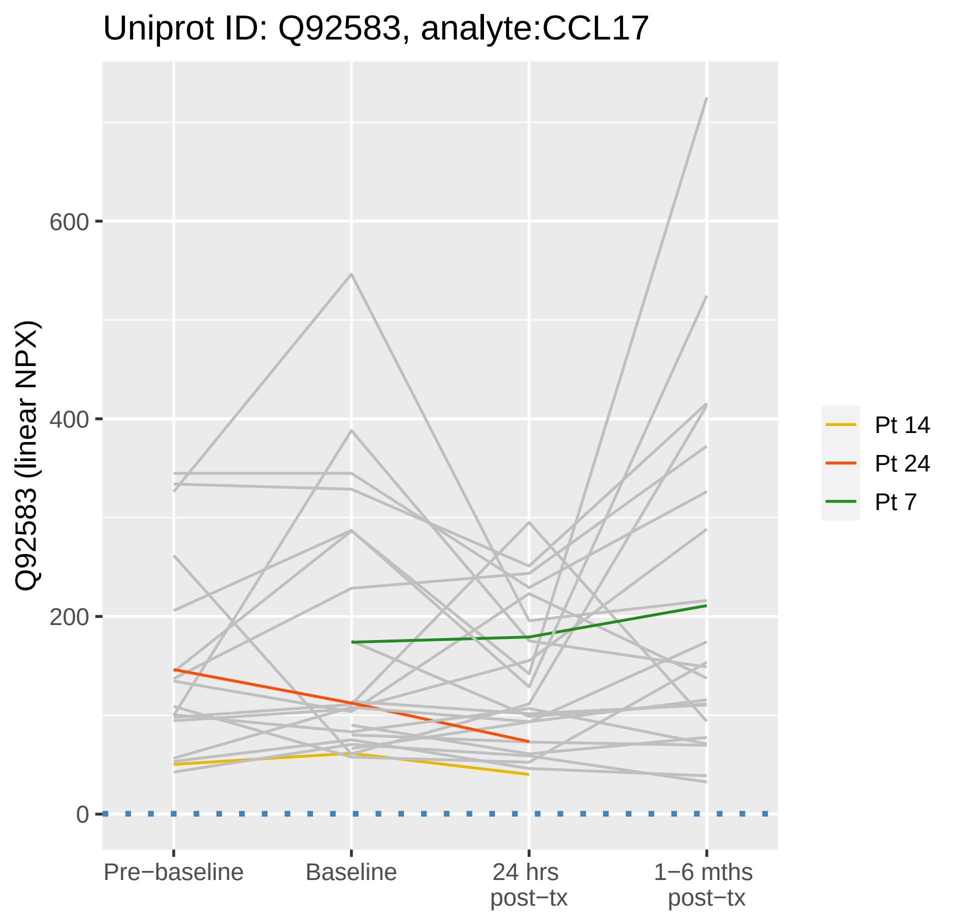

Uniprot ID: Q92583, analyte:CCL17
Q92583 (linear NPX)
600
400
200
0
Pre−baseline
Baseline
24 hrs post−tx
1−6 mths post−tx
Pt 14
Pt 24
Pt 7

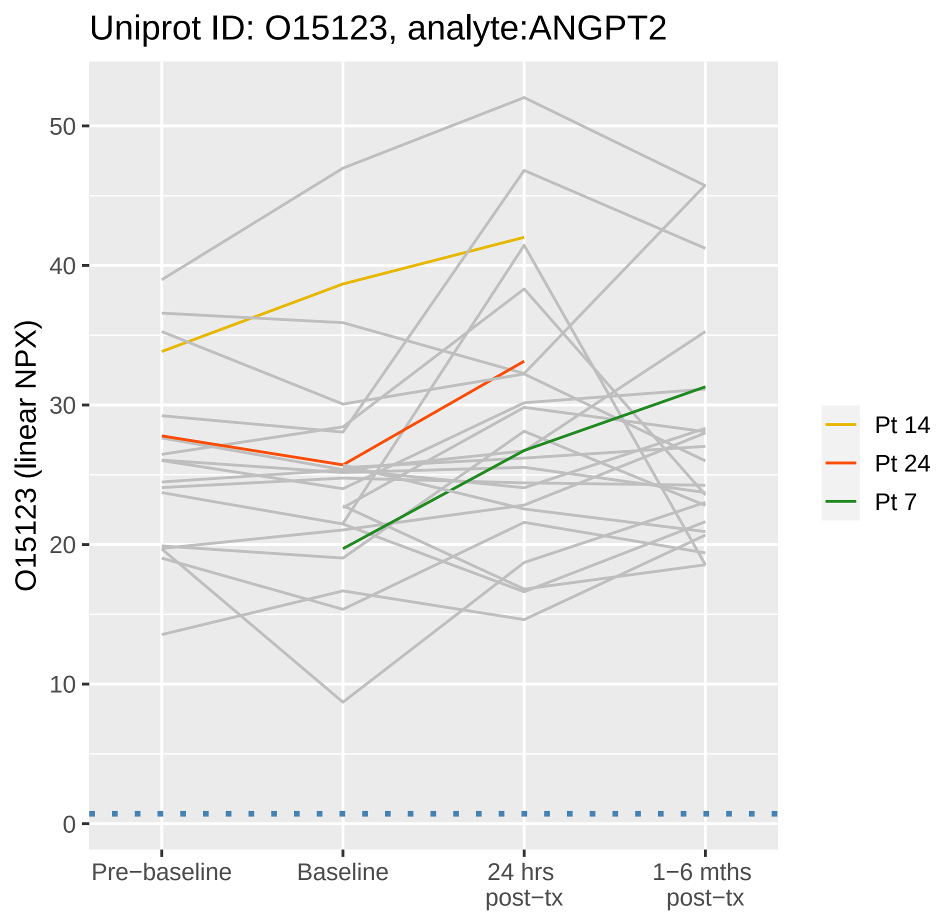
Uniprot ID: O15123, analyte:ANGPT2
O15123 (linear NPX)
50
40
30
20
10
0
Pre−baseline
Baseline
24 hrs post−tx
1−6 mths post−tx
Pt 14
Pt 24
Pt 7

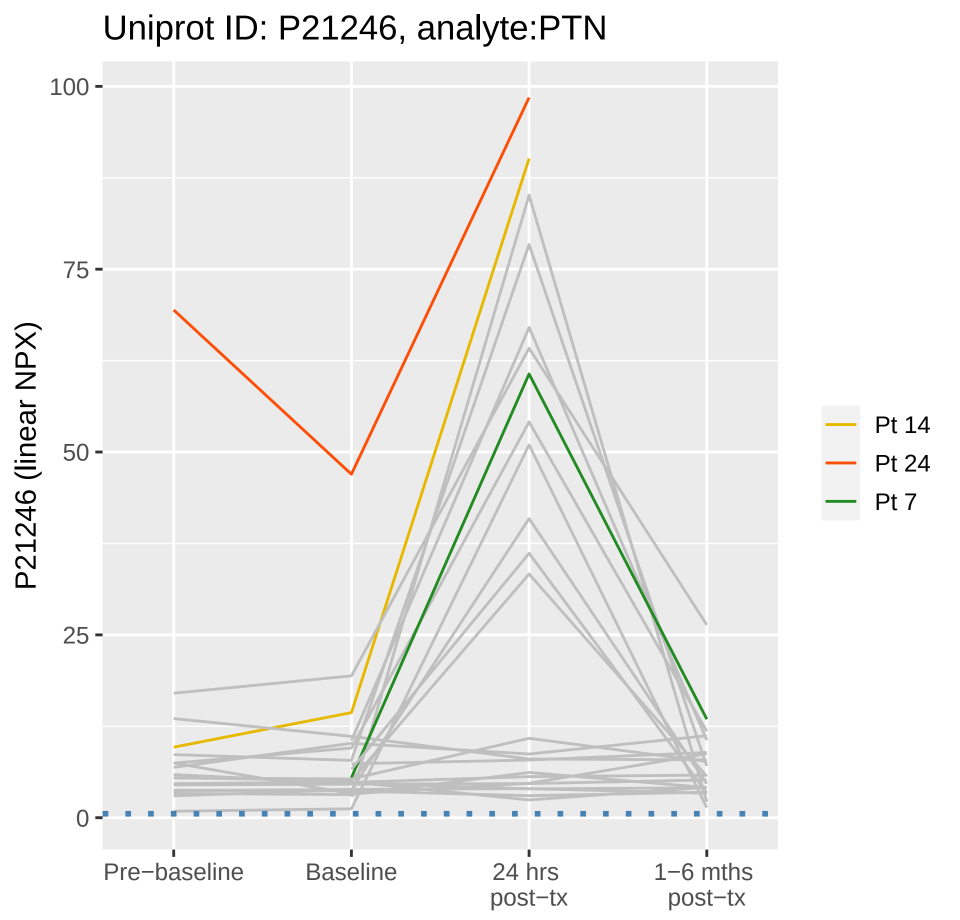
Uniprot ID: P21246, analyte:PTN
P21246 (linear NPX)
100
75
50
25
0
Pre−baseline
Baseline
24 hrs post−tx
1−6 mths post−tx
Pt 14
Pt 24
Pt 7

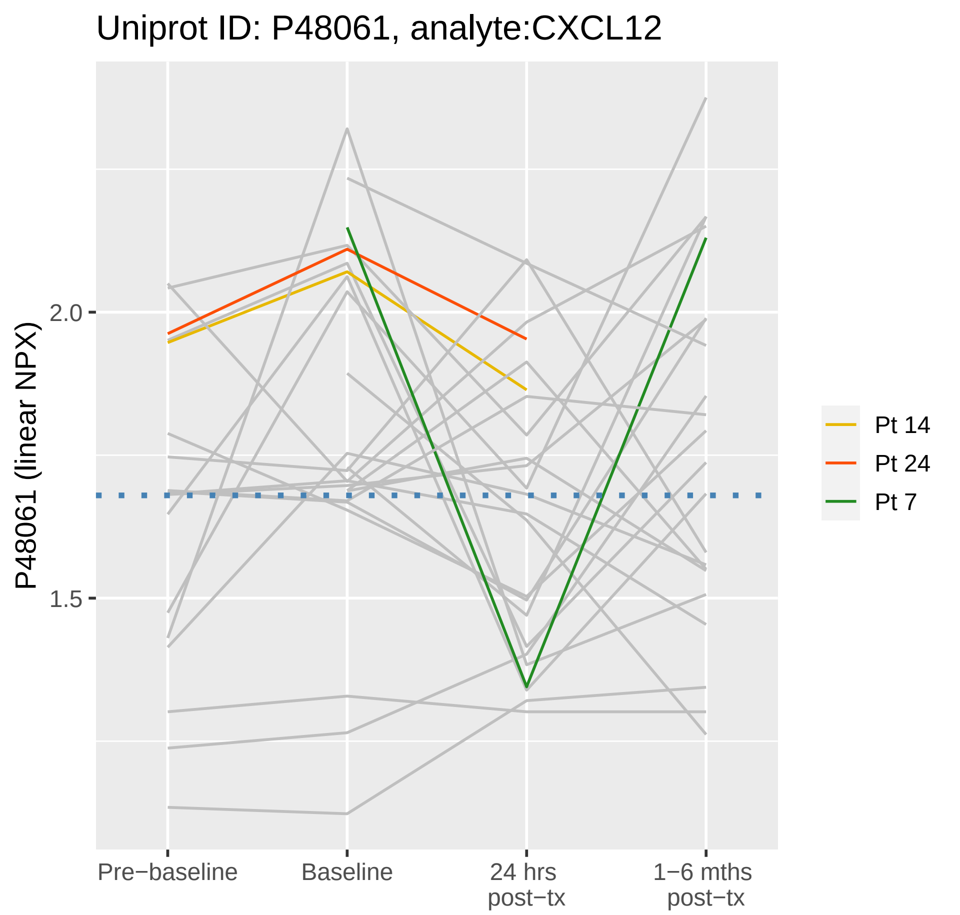

Uniprot ID: P48061, analyte:CXCL12
P48061 (linear NPX)
2.0
1.5
Pre−baseline
Baseline
24 hrs
post−tx
1−6 mths
post−tx
Pt 14
Pt 24
Pt 7

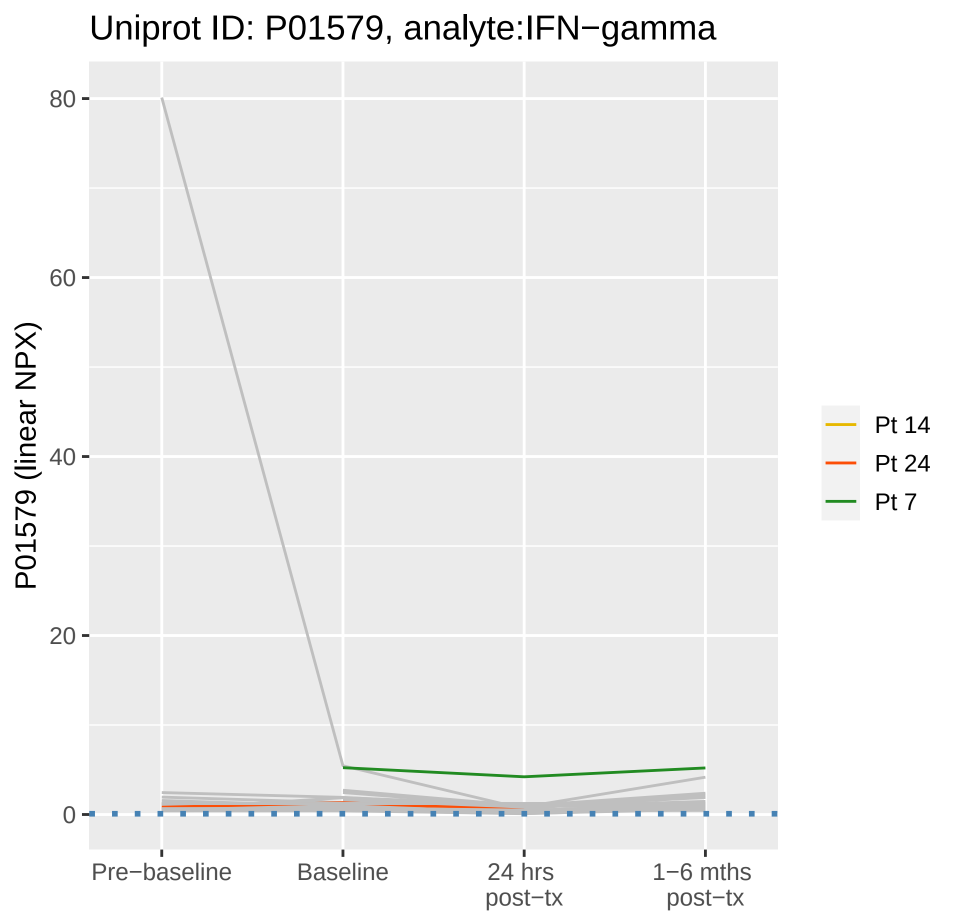

Uniprot ID: P01579, analyte:IFN−gamma
P01579 (linear NPX)
80
60
40
20
0
Pre−baseline
Baseline
24 hrs post−tx
1−6 mths post−tx
Pt 14
Pt 24
Pt 7

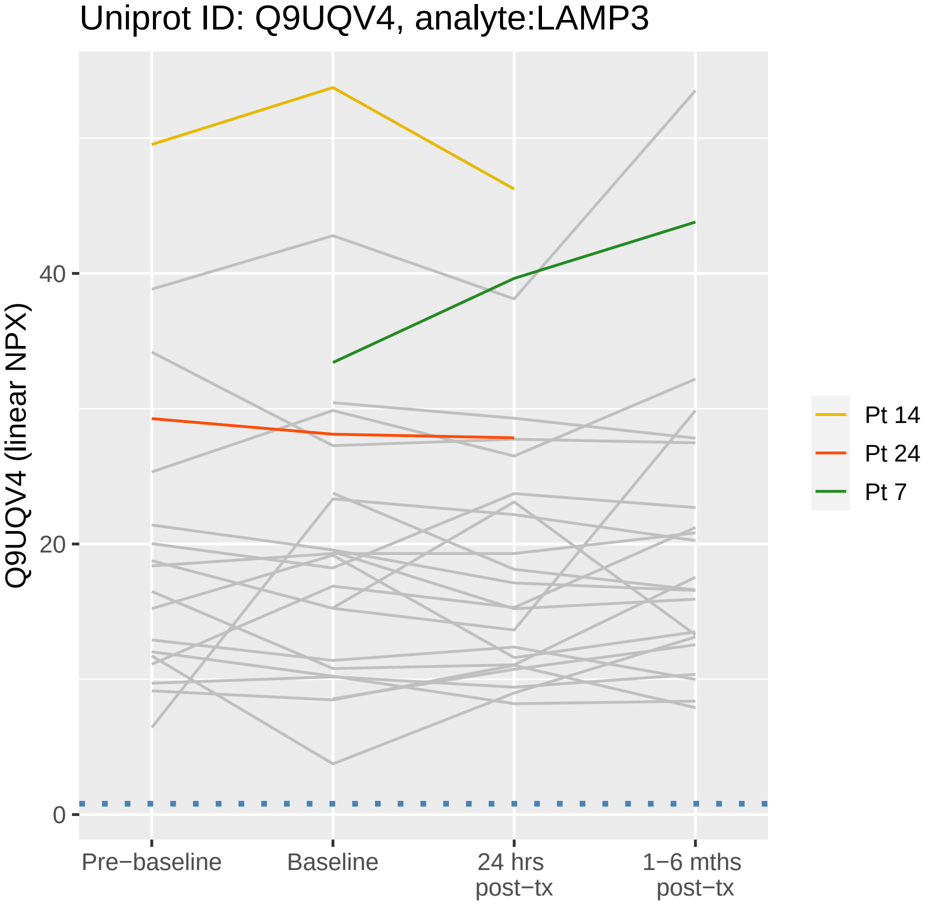

Uniprot ID: Q9UQV4, analyte:LAMP3
Q9UQV4 (linear NPX)
40
20
0
Pre−baseline
Baseline
24 hrs post−tx
1−6 mths post−tx
Pt 14
Pt 24
Pt 7

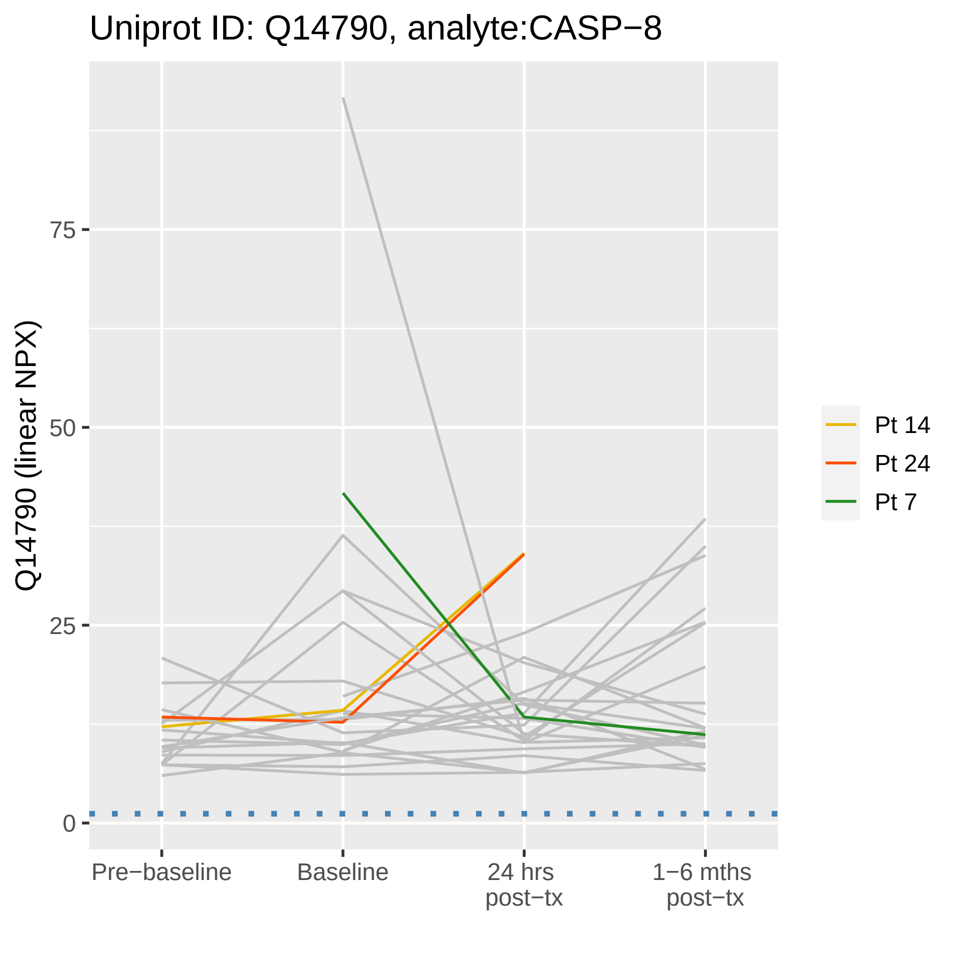
Uniprot ID: Q14790, analyte:CASP−8
Q14790 (linear NPX)
0
25
50
75
Pre−baseline
Baseline
24 hrs post−tx
1−6 mths post−tx
Pt 14
Pt 24
Pt 7

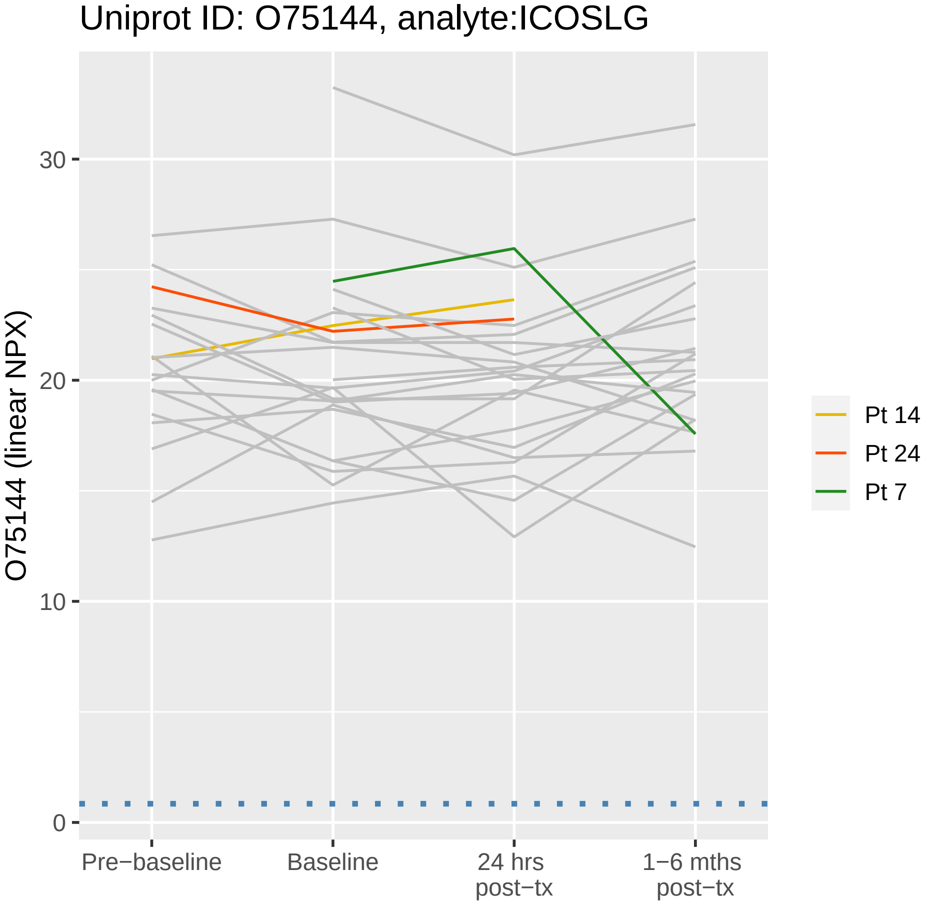

Uniprot ID: O75144, analyte:ICOSLG
O75144 (linear NPX)
30
20
10
0
Pre−baseline
Baseline
24 hrs post−tx
1−6 mths post−tx
Pt 14
Pt 24
Pt 7

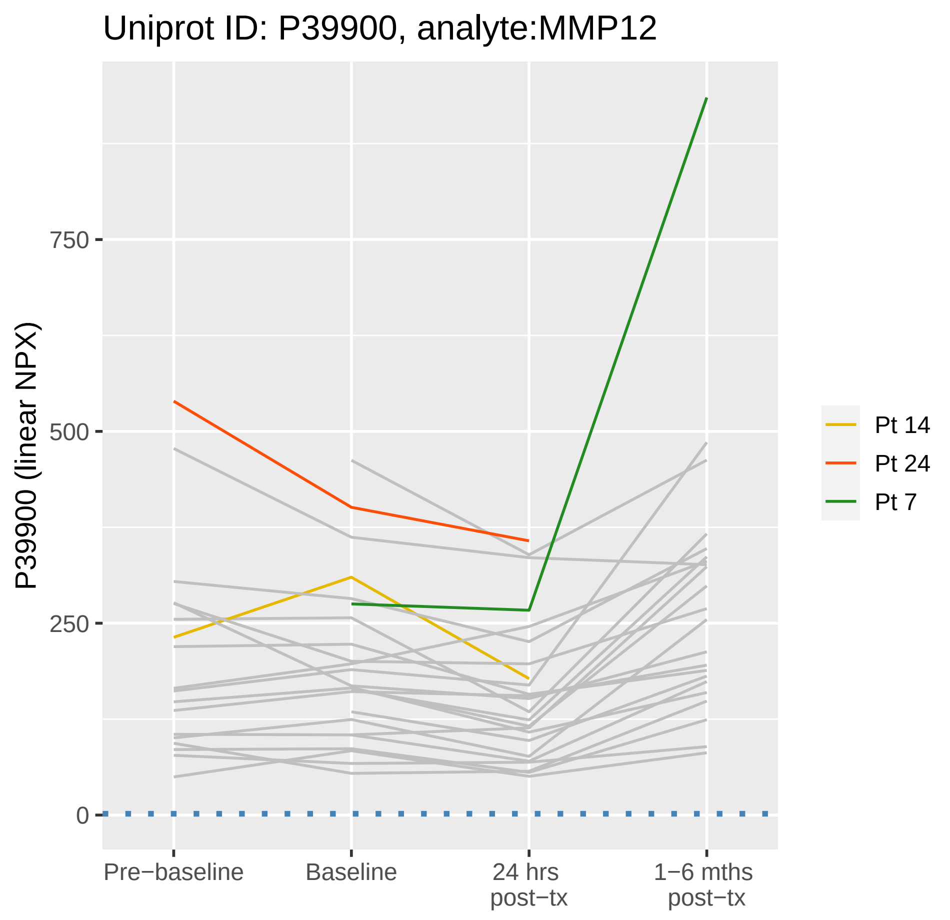

Uniprot ID: P39900, analyte:MMP12
P39900 (linear NPX)
750
500
250
0
Pre−baseline
Baseline
24 hrs post−tx
1−6 mths post−tx
Pt 14
Pt 24
Pt 7

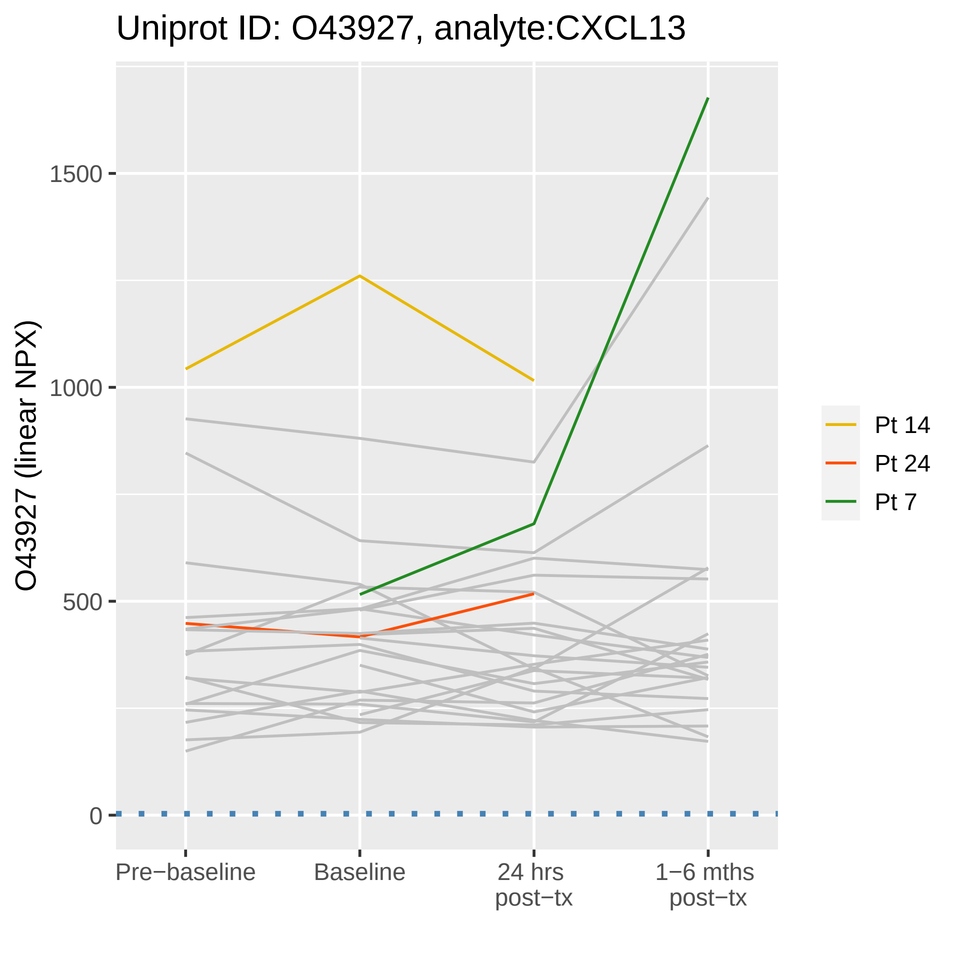

Uniprot ID: O43927, analyte:CXCL13
O43927 (linear NPX)
1500
1000
500
0
Pre−baseline
Baseline
24 hrs post−tx
1−6 mths post−tx
Pt 14
Pt 24
Pt 7

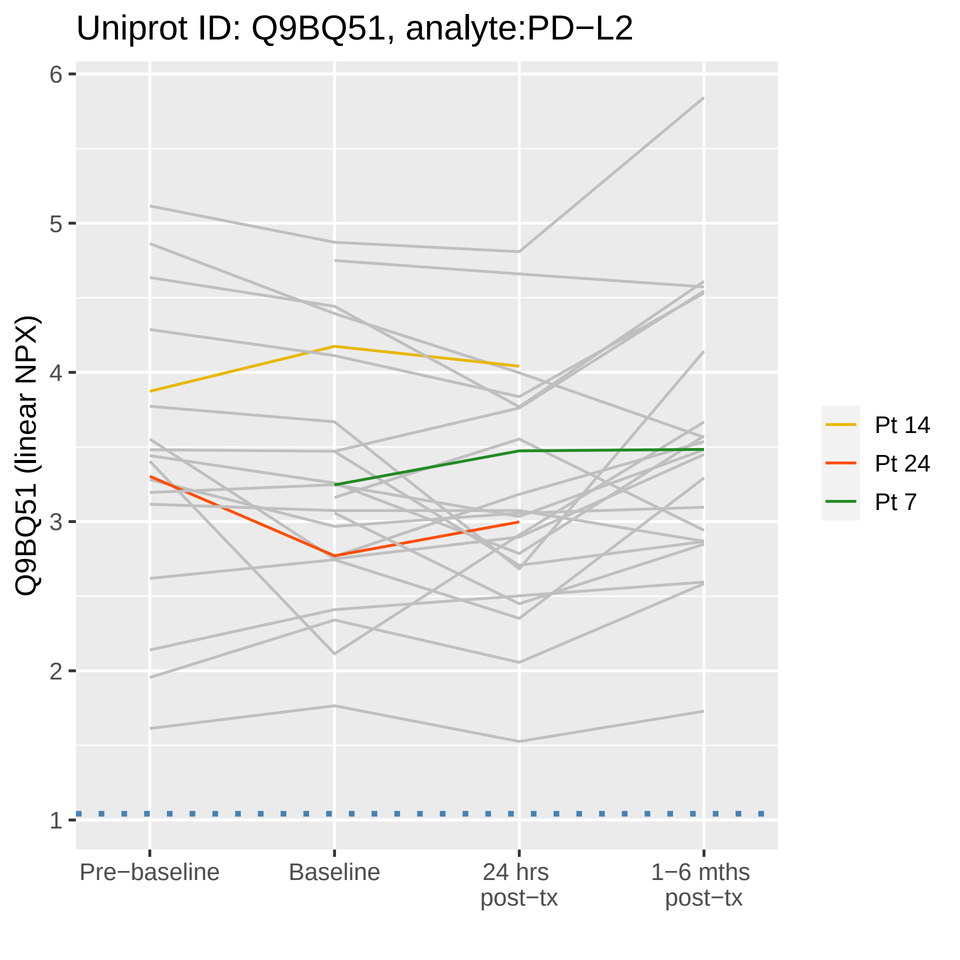

Uniprot ID: Q9BQ51, analyte:PD−L2
Q9BQ51 (linear NPX)
6
5
4
3
2
1
Pre−baseline
Baseline
24 hrs post−tx
1−6 mths post−tx
Pt 14
Pt 24
Pt 7

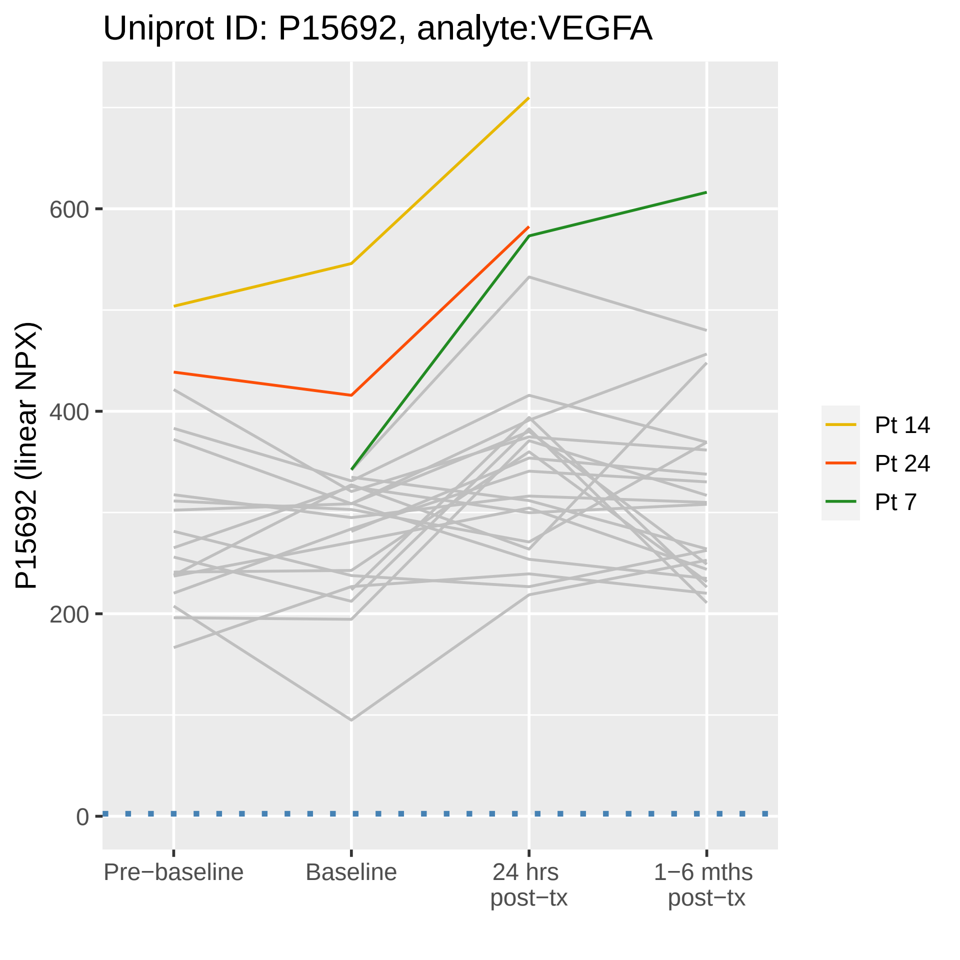

Uniprot ID: P15692, analyte:VEGFA
P15692 (linear NPX)
600
400
200
0
Pre−baseline
Baseline
24 hrs post−tx
1−6 mths post−tx
Pt 14
Pt 24
Pt 7

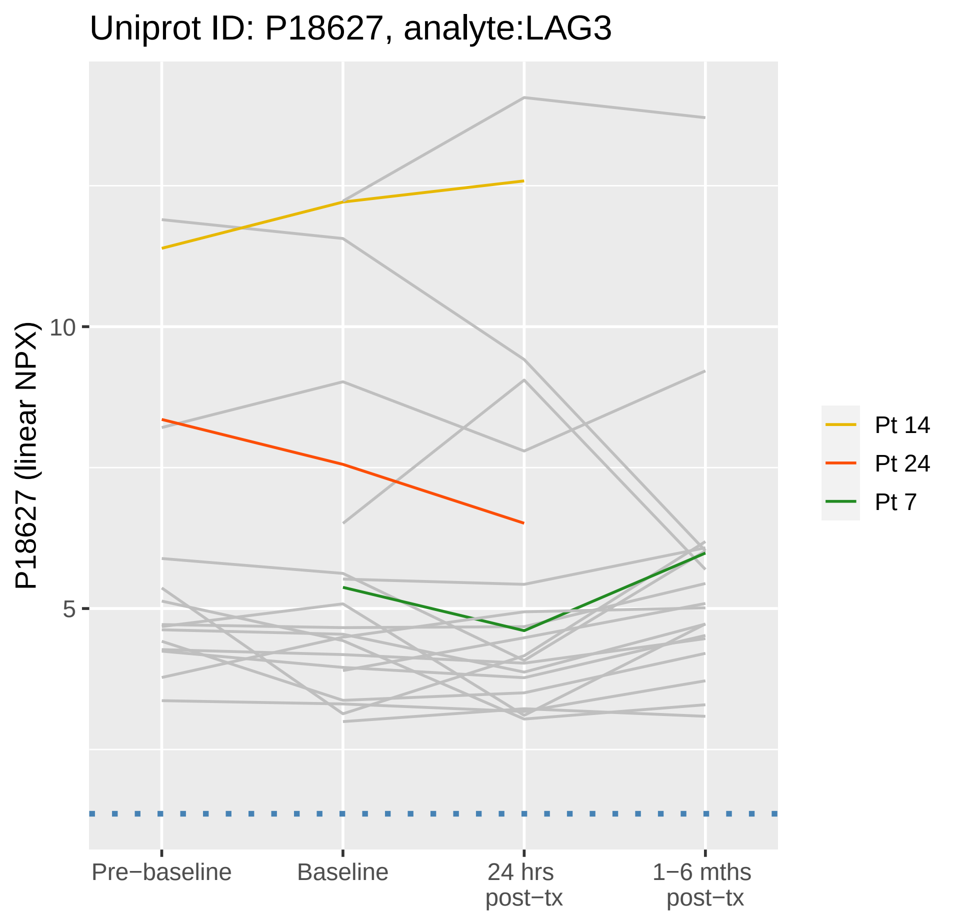
Uniprot ID: P18627, analyte:LAG3
P18627 (linear NPX)
10
5
Pre−baseline
Baseline
24 hrs
post−tx
1−6 mths
post−tx
Pt 14
Pt 24
Pt 7

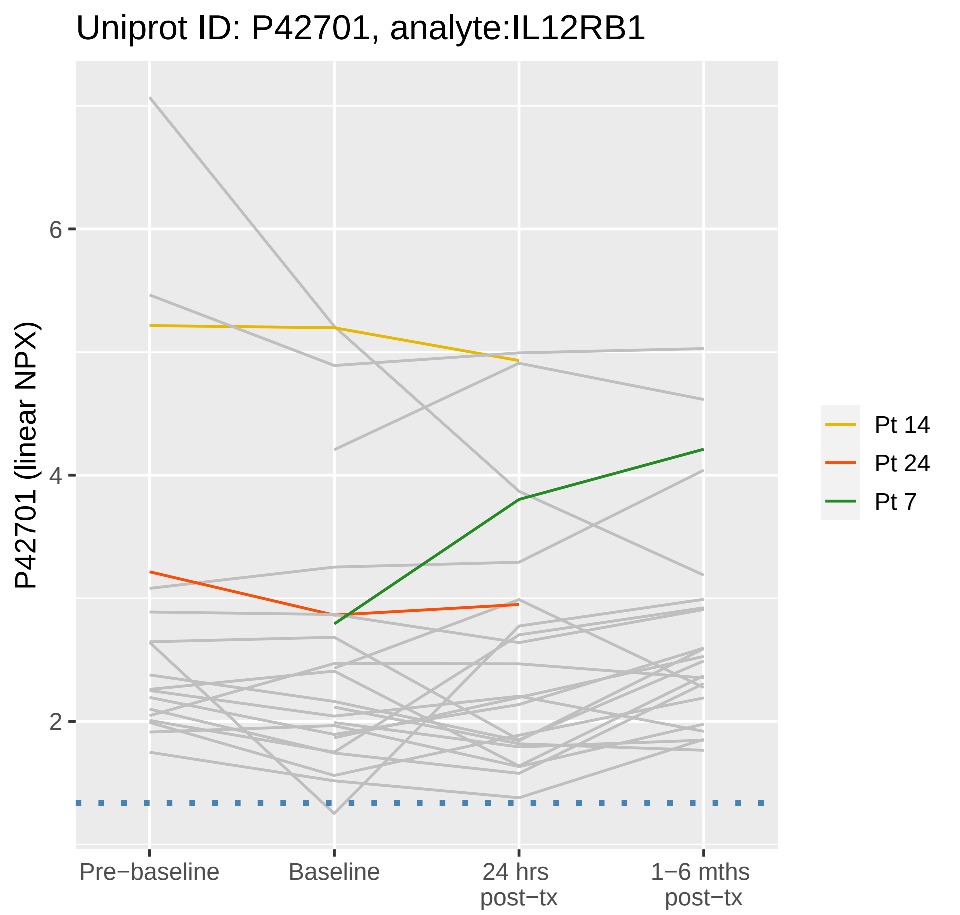
Uniprot ID: P42701, analyte:IL12RB1
P42701 (linear NPX)
6
4
2
Pre−baseline
Baseline
24 hrs
post−tx
1−6 mths
post−tx
Pt 14
Pt 24
Pt 7

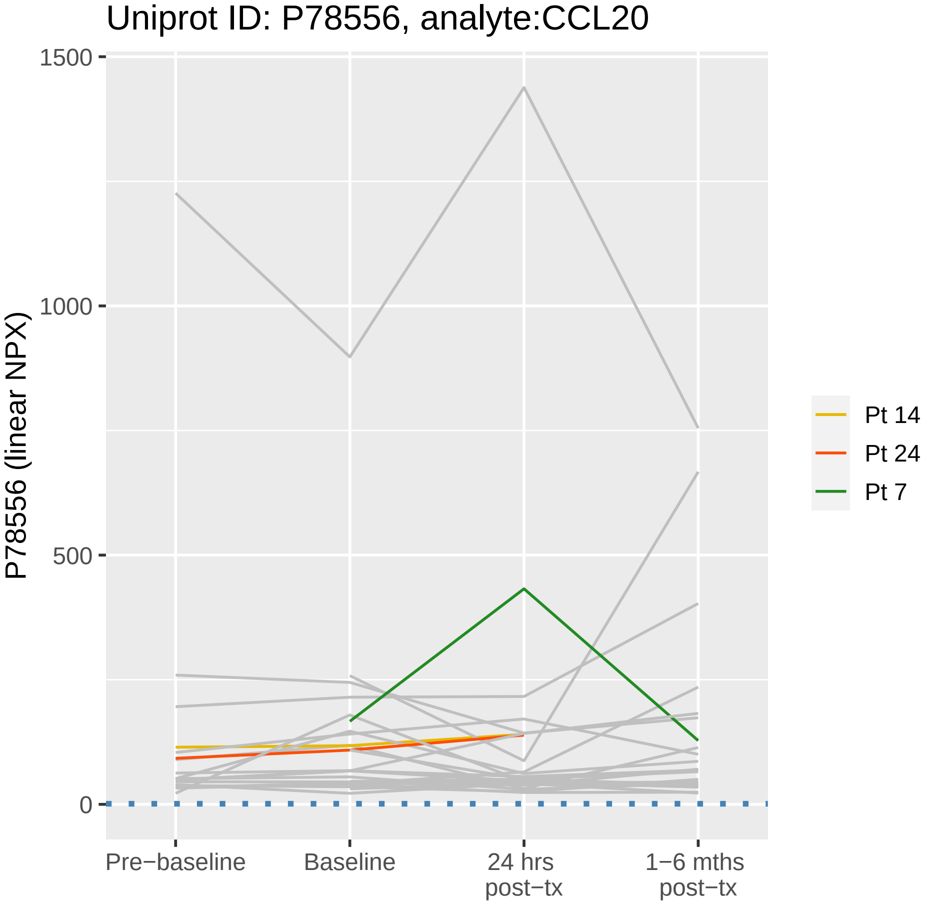

Uniprot ID: P78556, analyte:CCL20
P78556 (linear NPX)
1500
1000
500
0
Pre−baseline
Baseline
24 hrs
post−tx
1−6 mths
post−tx
Pt 14
Pt 24
Pt 7

Uniprot ID: P01375, analyte:TNF

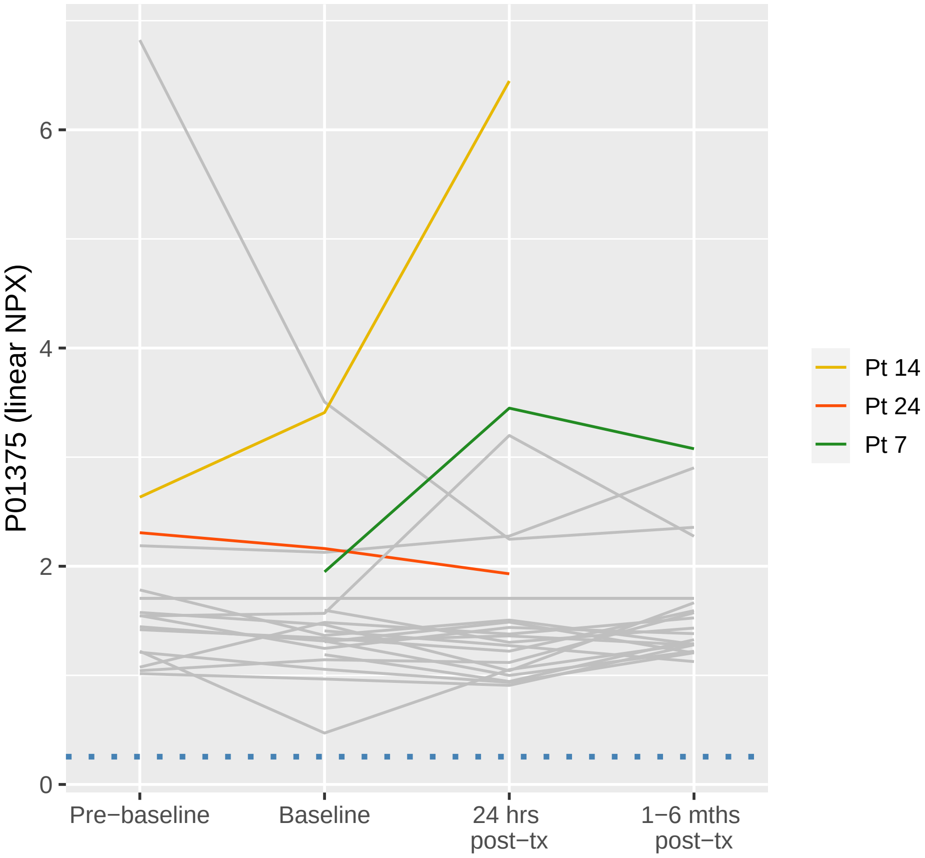

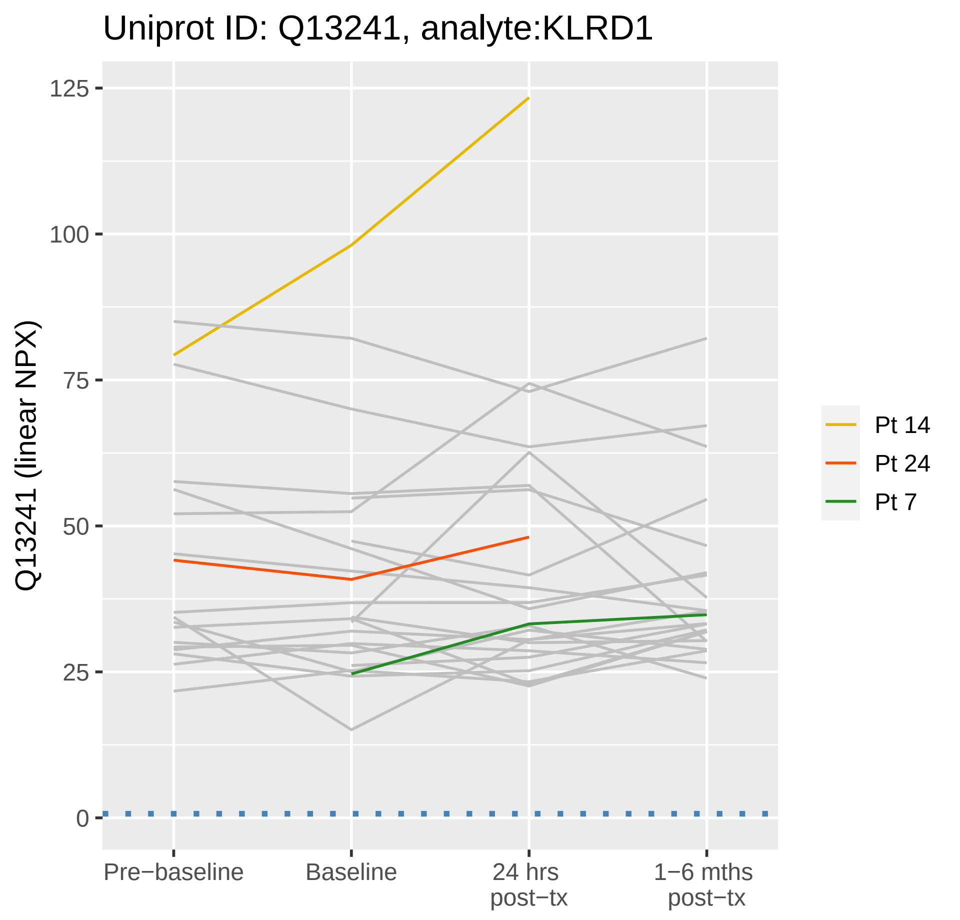
Uniprot ID: Q13241, analyte:KLRD1
Q13241 (linear NPX)
125
100
75
50
25
0
Pre−baseline
Baseline
24 hrs post−tx
1−6 mths post−tx
Pt 14
Pt 24
Pt 7

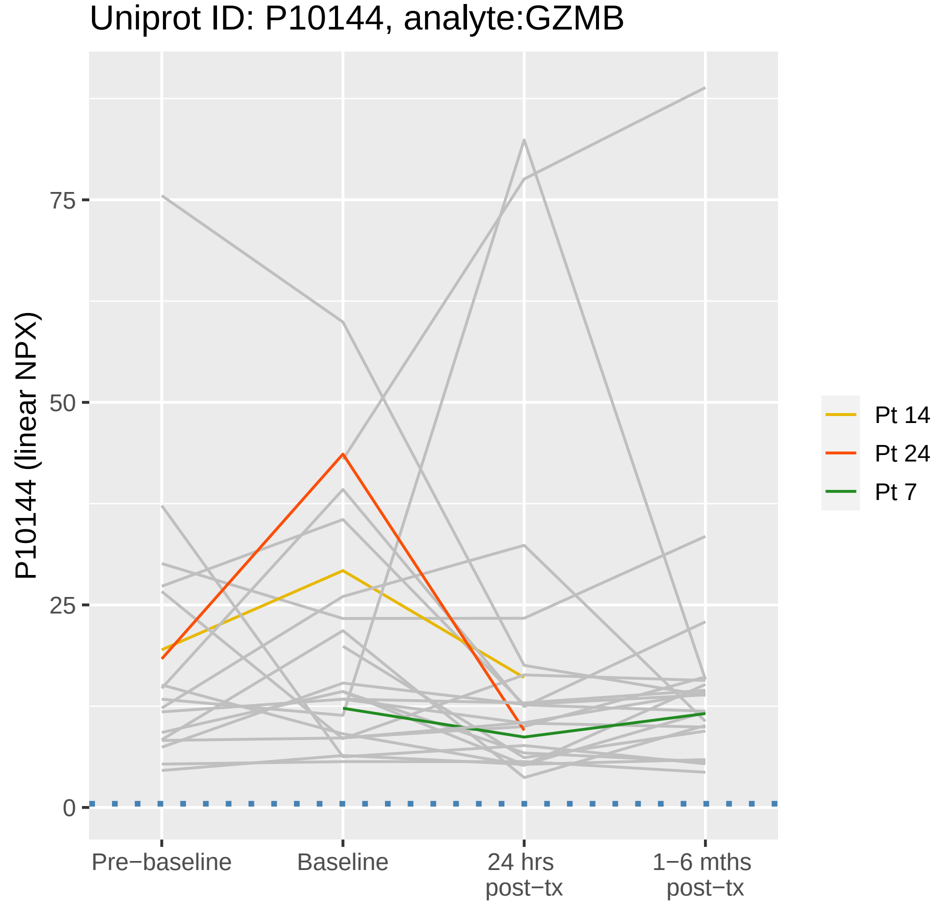
Uniprot ID: P10144, analyte:GZMB
P10144 (linear NPX)
75
50
25
0
Pre−baseline
Baseline
24 hrs
post−tx
1−6 mths
post−tx
Pt 14
Pt 24
Pt 7

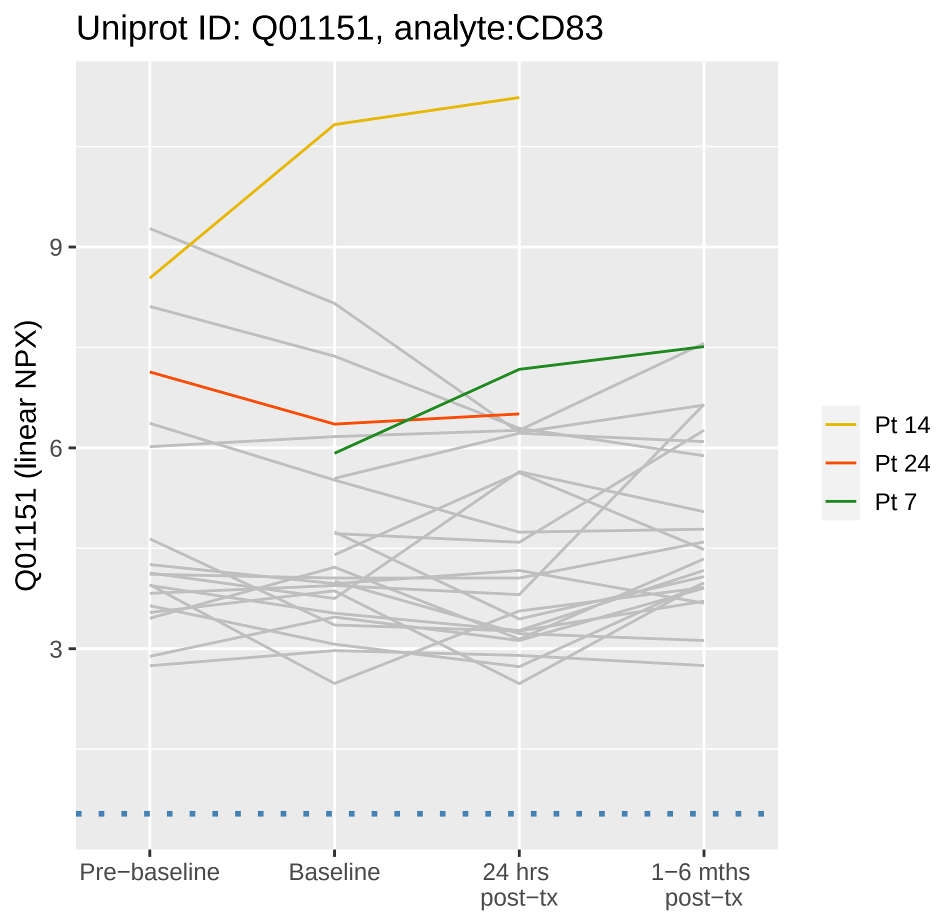
Uniprot ID: Q01151, analyte:CD83
Q01151 (linear NPX)
9
6
3
Pre−baseline
Baseline
24 hrs post−tx
1−6 mths post−tx
Pt 14
Pt 24
Pt 7

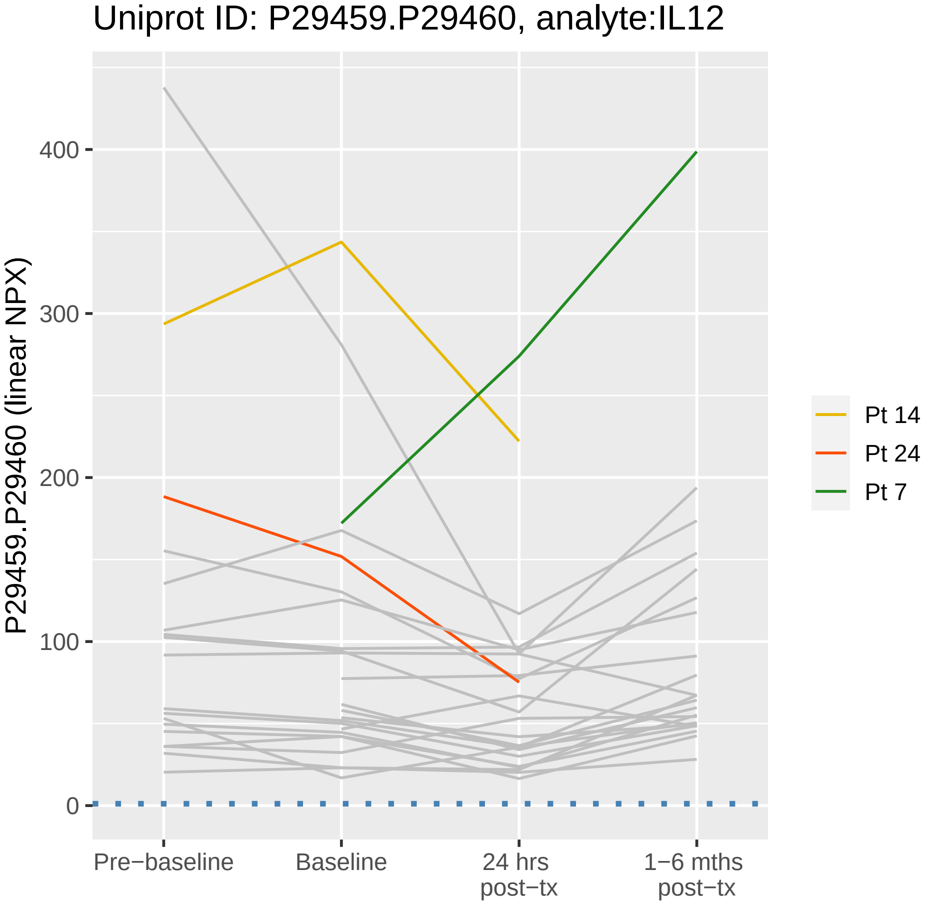

Uniprot ID: P29459.P29460, analyte:IL12
P29459.P29460 (linear NPX)
400
300
200
100
0
Pre−baseline
Baseline
24 hrs post−tx
1−6 mths post−tx
Pt 14
Pt 24
Pt 7

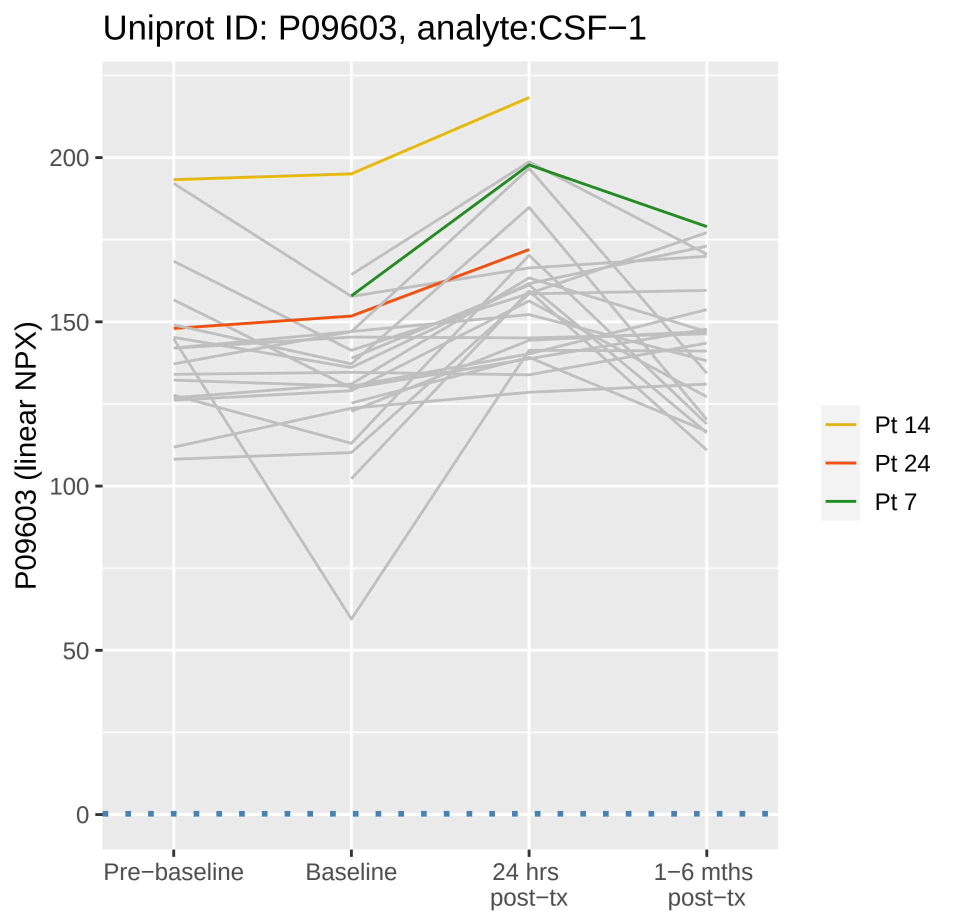

Uniprot ID: P09603, analyte:CSF−1
P09603 (linear NPX)
200
150
100
50
0
Pre−baseline
Baseline
24 hrs post−tx
1−6 mths post−tx
Pt 14
Pt 24
Pt 7
