# Supplementary material for: An Exploratory Analysis of Changes in Circulating Plasma Protein Profiles Following Image-Guided Ablation of Renal Tumours Provides Evidence for Effects on Multiple Biological Processes
Source: Cancers (Basel). 2021 Nov 30;13(23):6037. doi: 10.3390/cancers13236037 (PMC8656737; doi:10.3390/cancers13236037)
Supplement: Supplementary file 1 [file cancers-13-06037-s001.zip › Supplementary File/Figure S9.pdf]

Figure S9. Longitudinal profiles of all plasma proteins on the Oncology II panel, prior to and post-ablation. Results for each protein are shown as linear NPX values and are shown for patients receiving CRYO (n=25) with the three patients who developed acute complications post-CRYO highlighted. Dotted lines indicate the lower limit of quantitation.

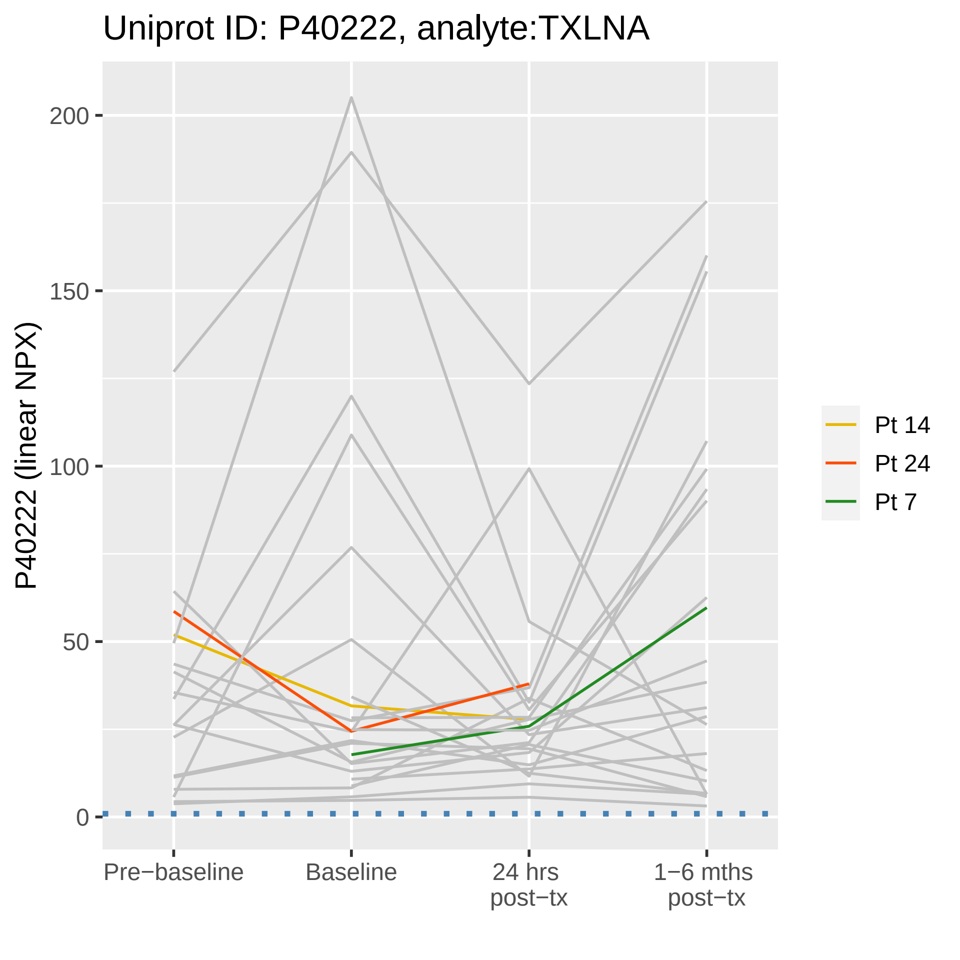

Uniprot ID: P40222, analyte:TXLNA
P40222 (linear NPX)
200
150
100
50
0
Pre−baseline
Baseline
24 hrs post−tx
1−6 mths post−tx
Pt 14
Pt 24
Pt 7

Uniprot ID: P15692, analyte:VEGFA

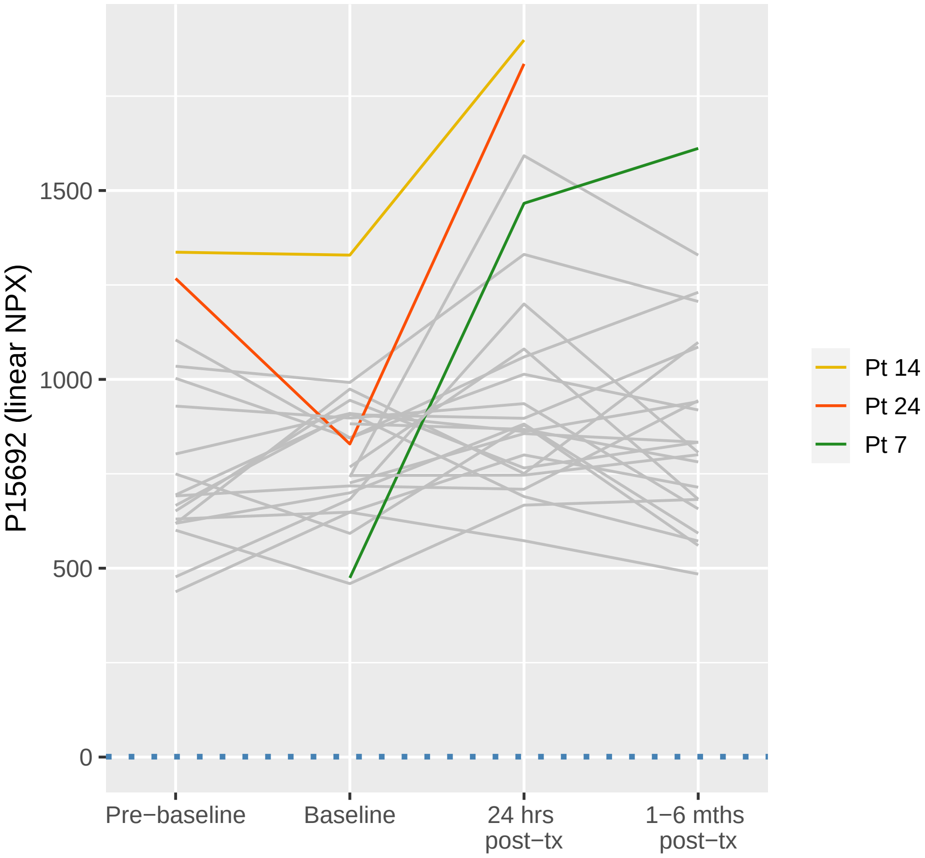

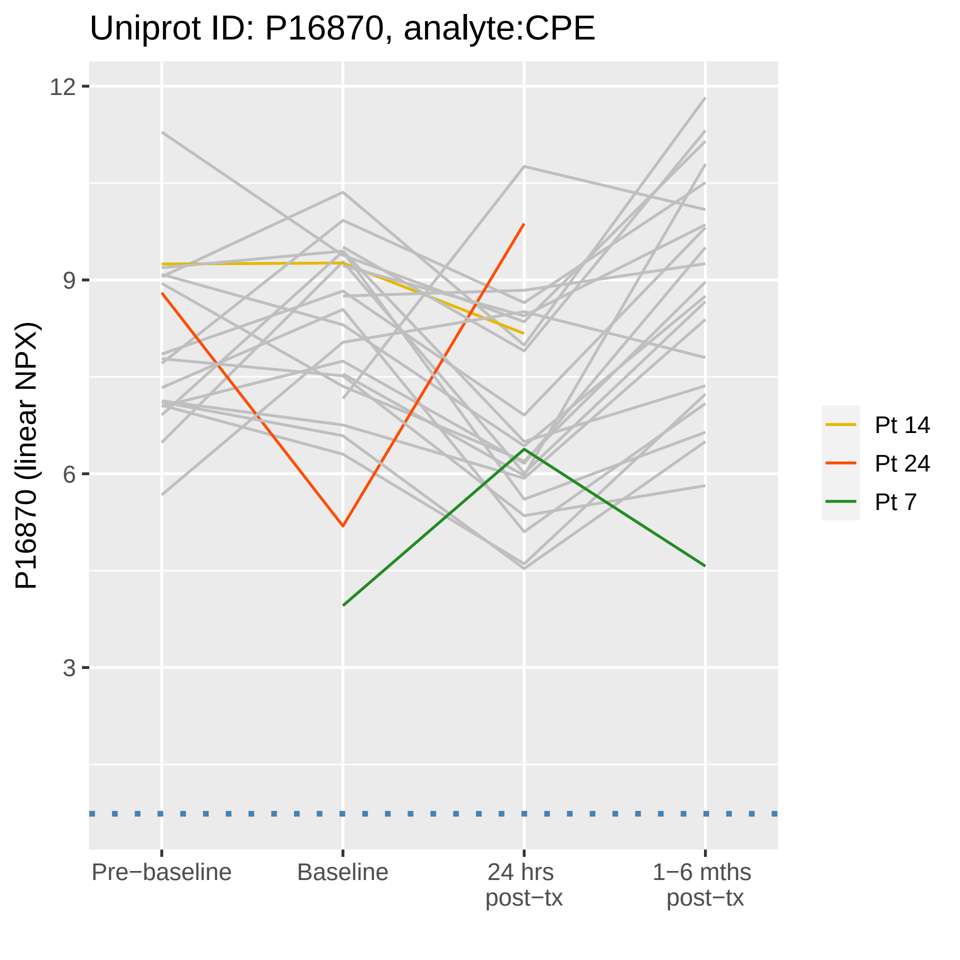

Uniprot ID: P16870, analyte:CPE
P16870 (linear NPX)
12
9
6
3
Pre−baseline
Baseline
24 hrs post−tx
1−6 mths post−tx
Pt 14
Pt 24
Pt 7

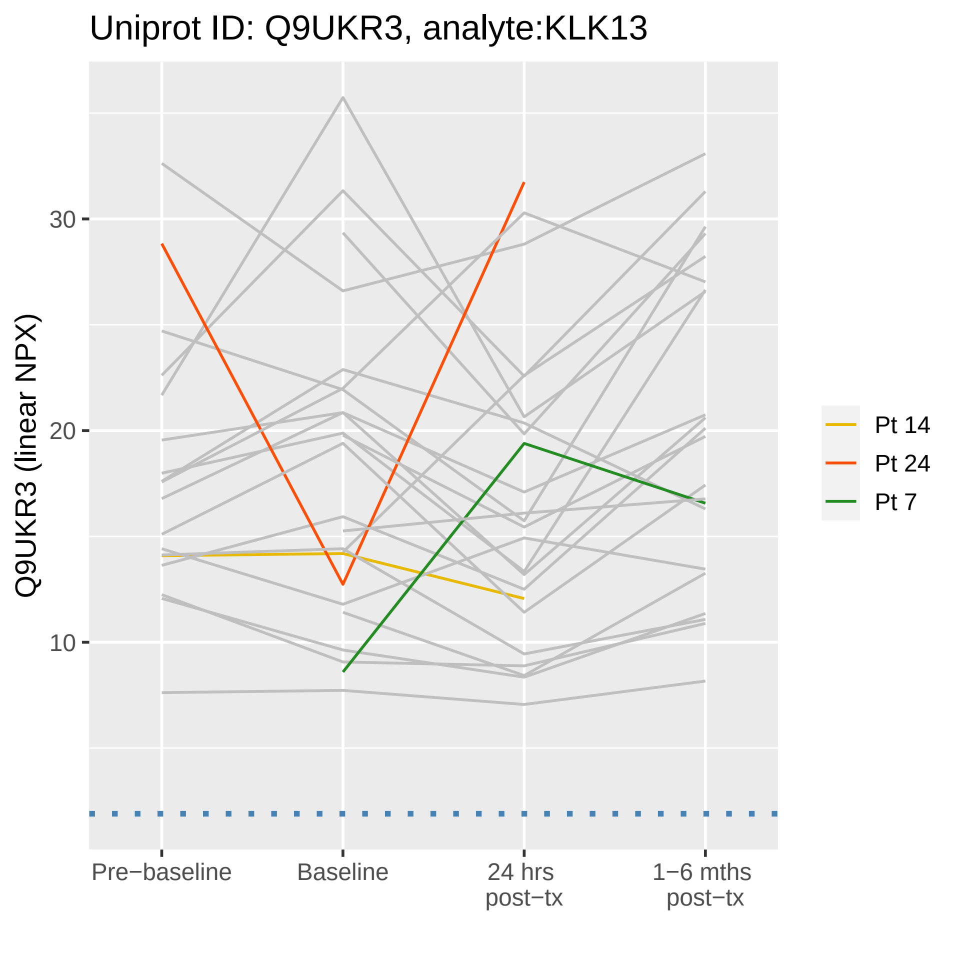

Uniprot ID: Q9UKR3, analyte:KLK13
Q9UKR3 (linear NPX)
30
20
10
Pre−baseline
Baseline
24 hrs
post−tx
1−6 mths
post−tx
Pt 14
Pt 24
Pt 7

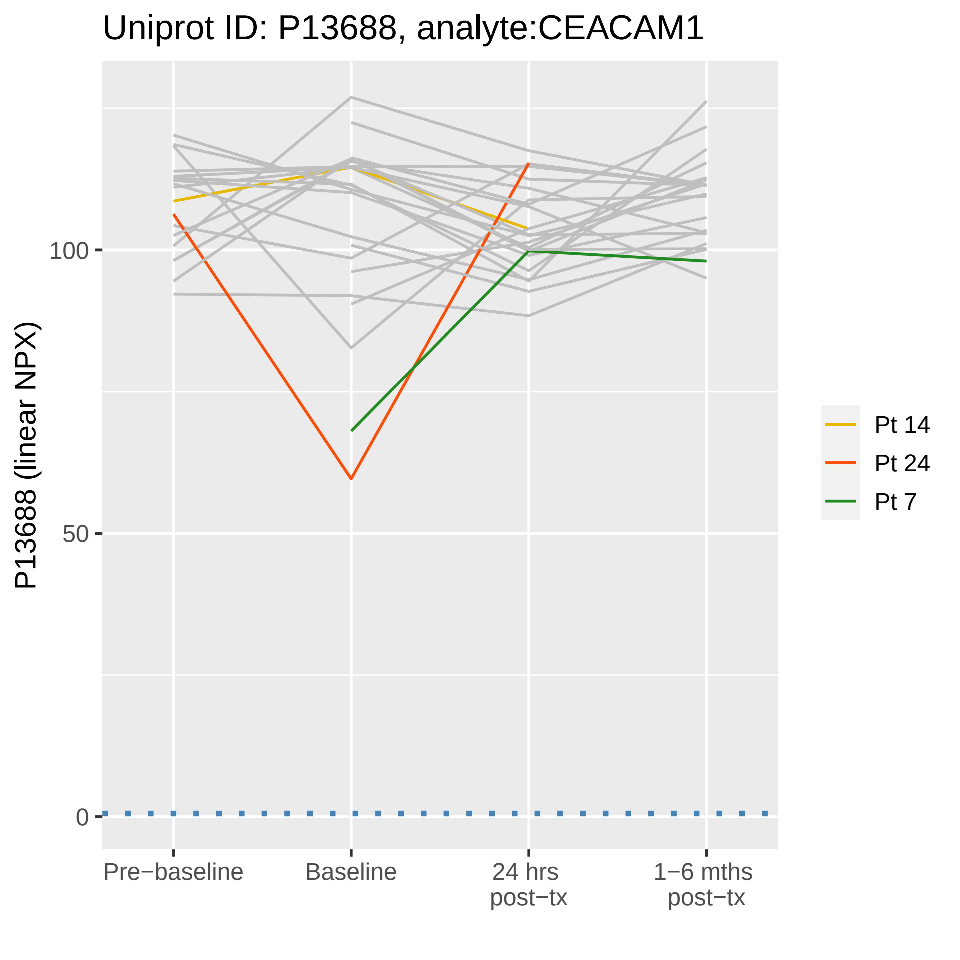
Uniprot ID: P13688, analyte:CEACAM1
P13688 (linear NPX)
100
50
0
Pre−baseline
Baseline
24 hrs post−tx
1−6 mths post−tx
Pt 14
Pt 24
Pt 7

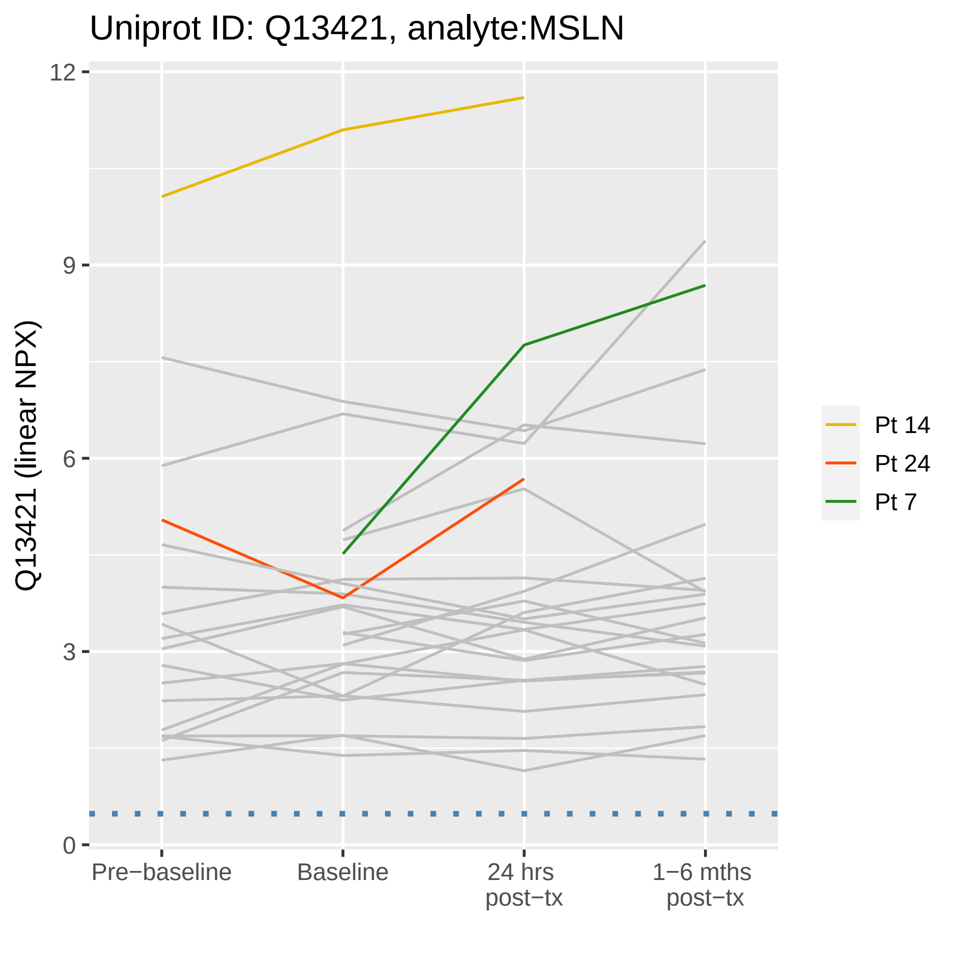

Uniprot ID: Q13421, analyte:MSLN
Q13421 (linear NPX)
12
9
6
3
0
Pre−baseline
Baseline
24 hrs post−tx
1−6 mths post−tx
Pt 14
Pt 24
Pt 7

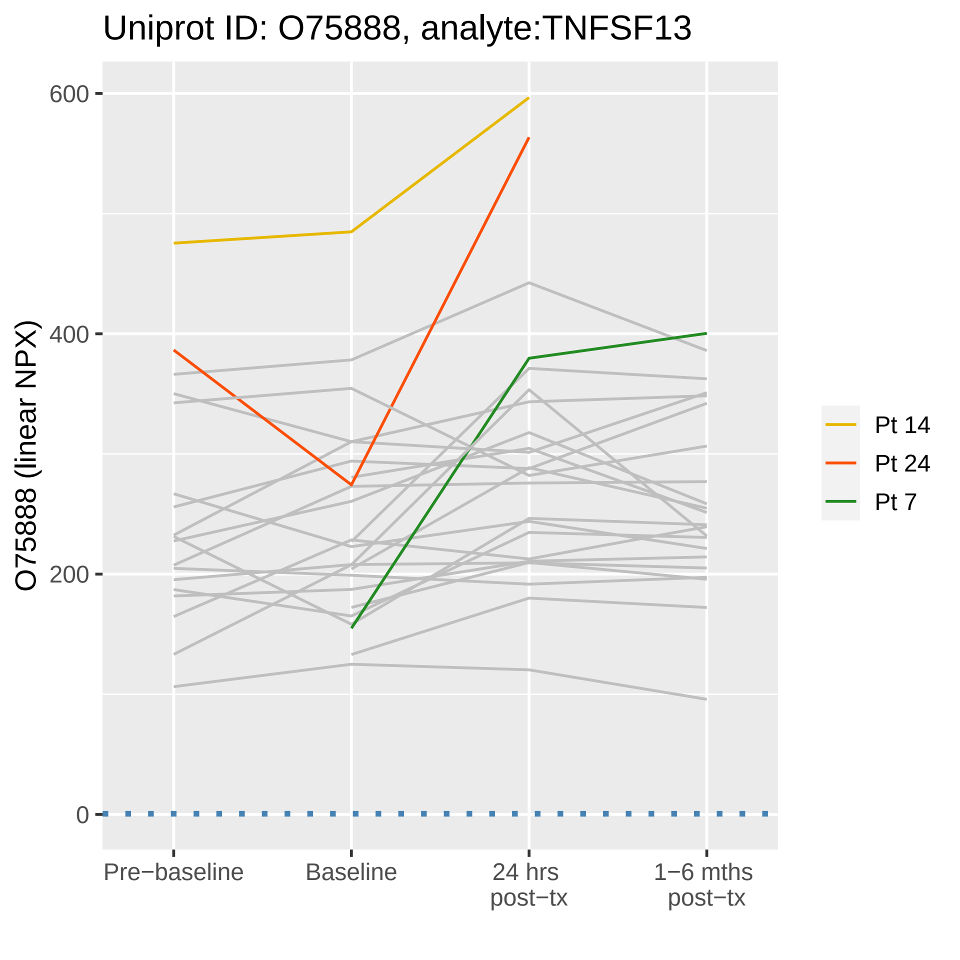

Uniprot ID: O75888, analyte:TNFSF13
O75888 (linear NPX)
600
400
200
0
Pre−baseline
Baseline
24 hrs post−tx
1−6 mths post−tx
Pt 14
Pt 24
Pt 7

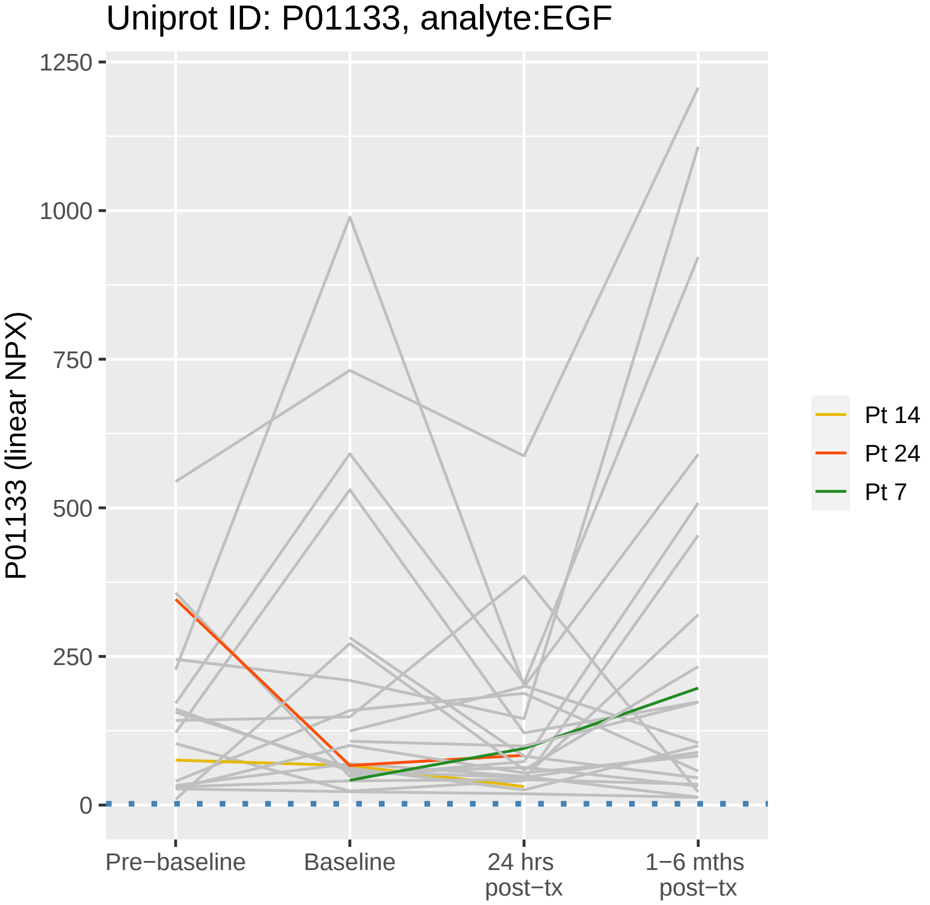

Uniprot ID: P01133, analyte:EGF
P01133 (linear NPX)
1250
1000
750
500
250
0
Pre−baseline
Baseline
24 hrs post−tx
1−6 mths post−tx
Pt 14
Pt 24
Pt 7

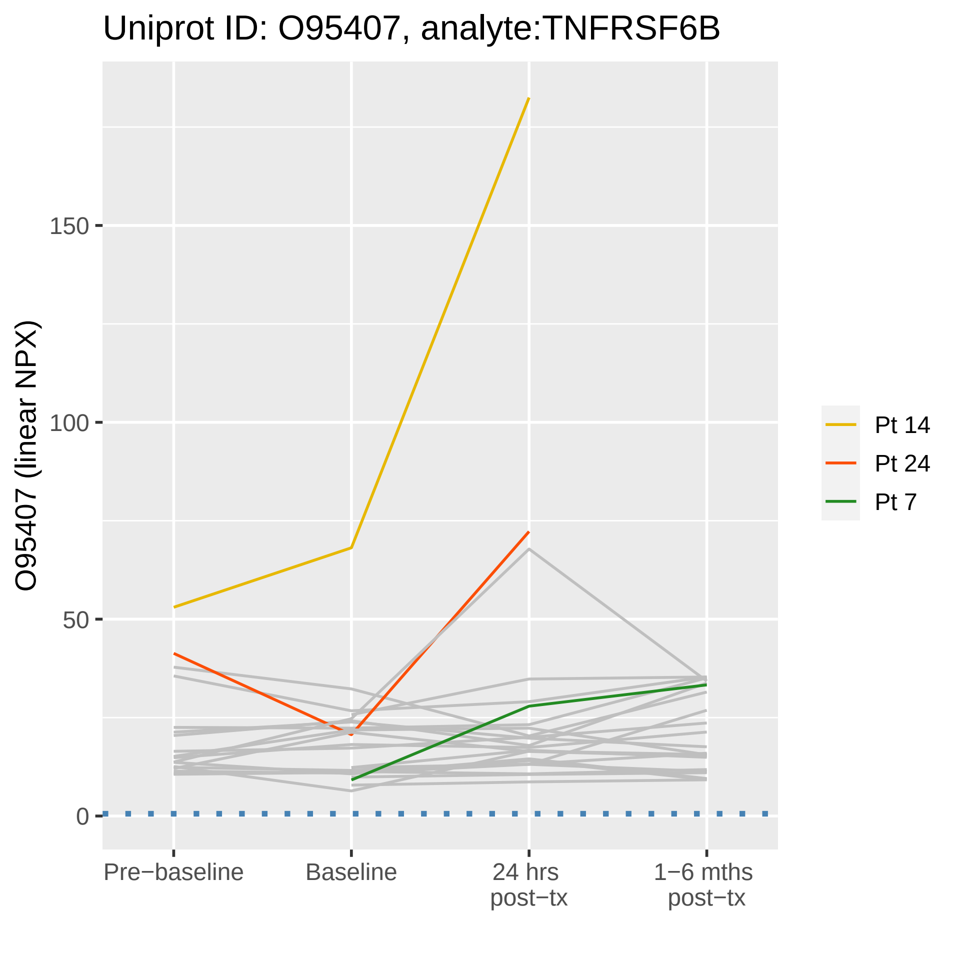

Uniprot ID: O95407, analyte:TNFRSF6B
O95407 (linear NPX)
150
100
50
0
Pre−baseline
Baseline
24 hrs post−tx
1−6 mths post−tx
Pt 14
Pt 24
Pt 7

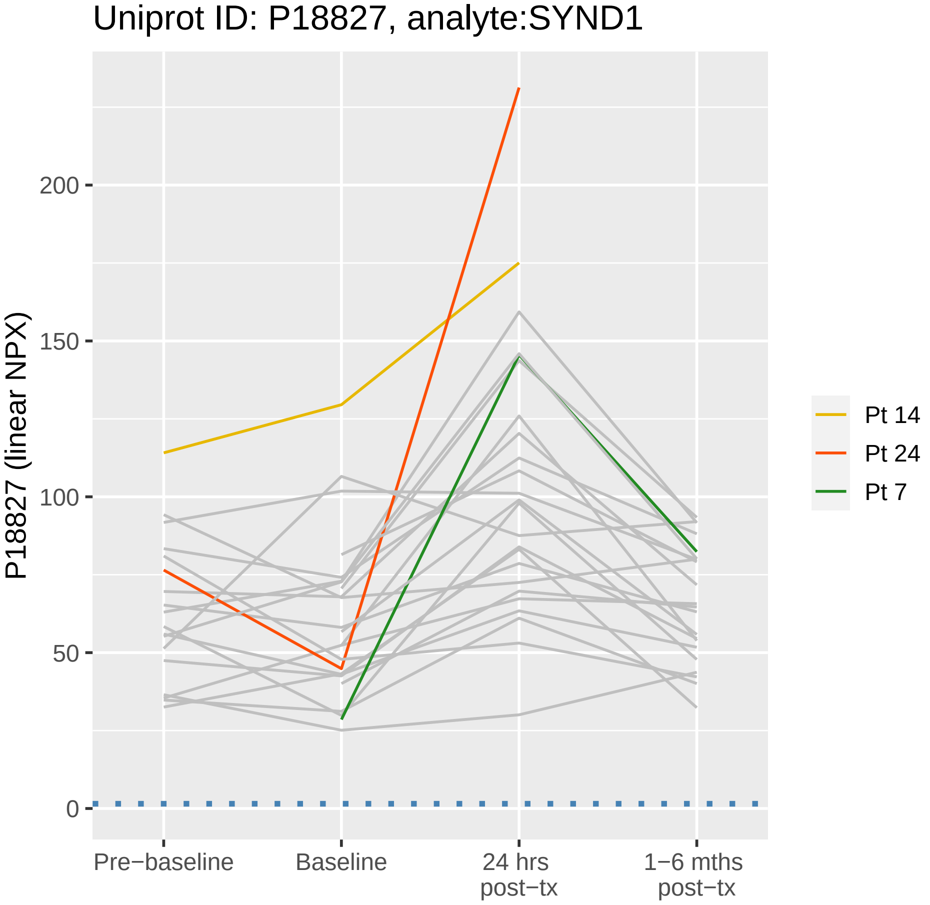

Uniprot ID: P18827, analyte:SYND1
P18827 (linear NPX)
200
150
100
50
0
Pre−baseline
Baseline
24 hrs post−tx
1−6 mths post−tx
Pt 14
Pt 24
Pt 7

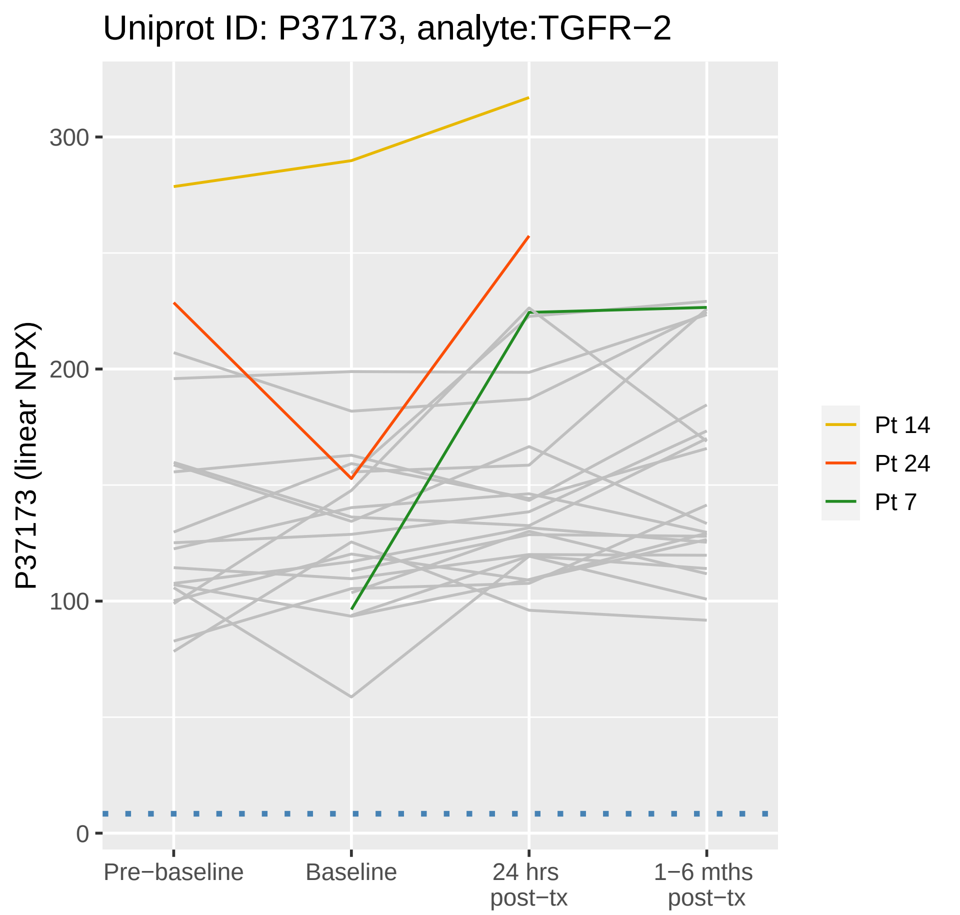

Uniprot ID: P37173, analyte:TGFR−2
P37173 (linear NPX)
300
200
100
0
Pre−baseline
Baseline
24 hrs post−tx
1−6 mths post−tx
Pt 14
Pt 24
Pt 7

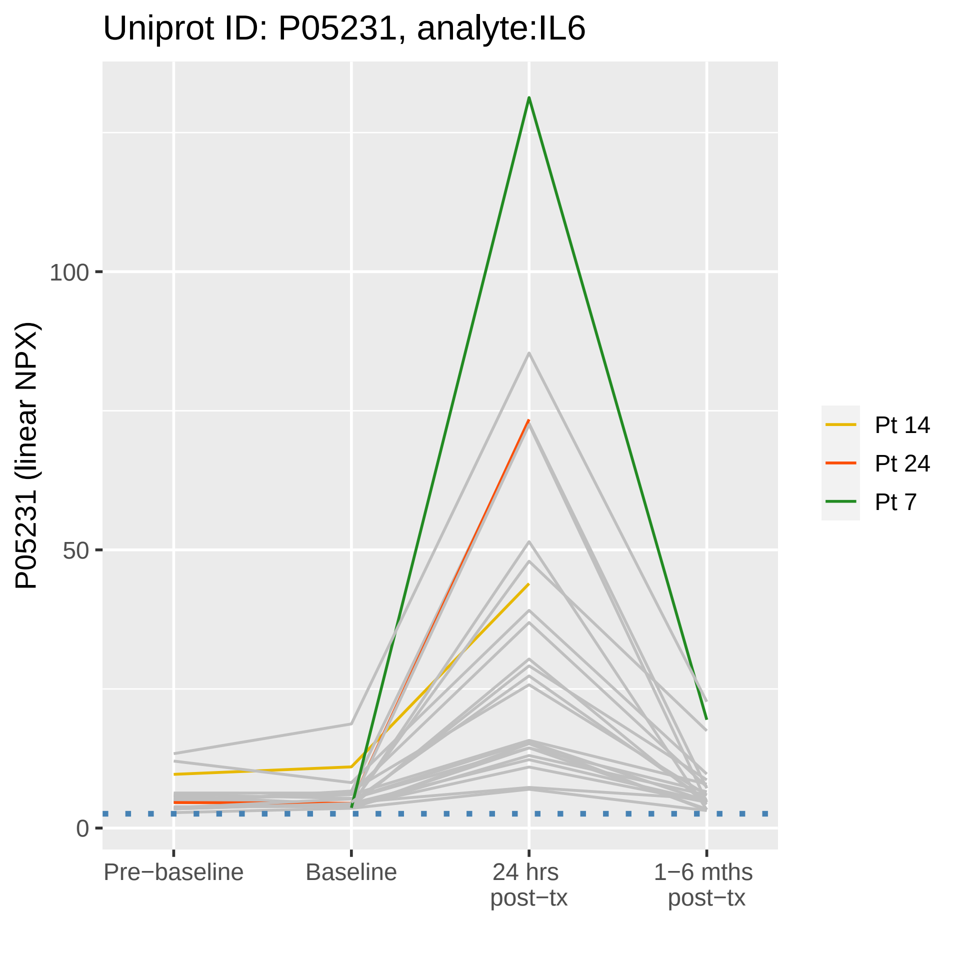

Uniprot ID: P05231, analyte:IL6
P05231 (linear NPX)
100
50
0
Pre−baseline
Baseline
24 hrs
post−tx
1−6 mths
post−tx
Pt 14
Pt 24
Pt 7

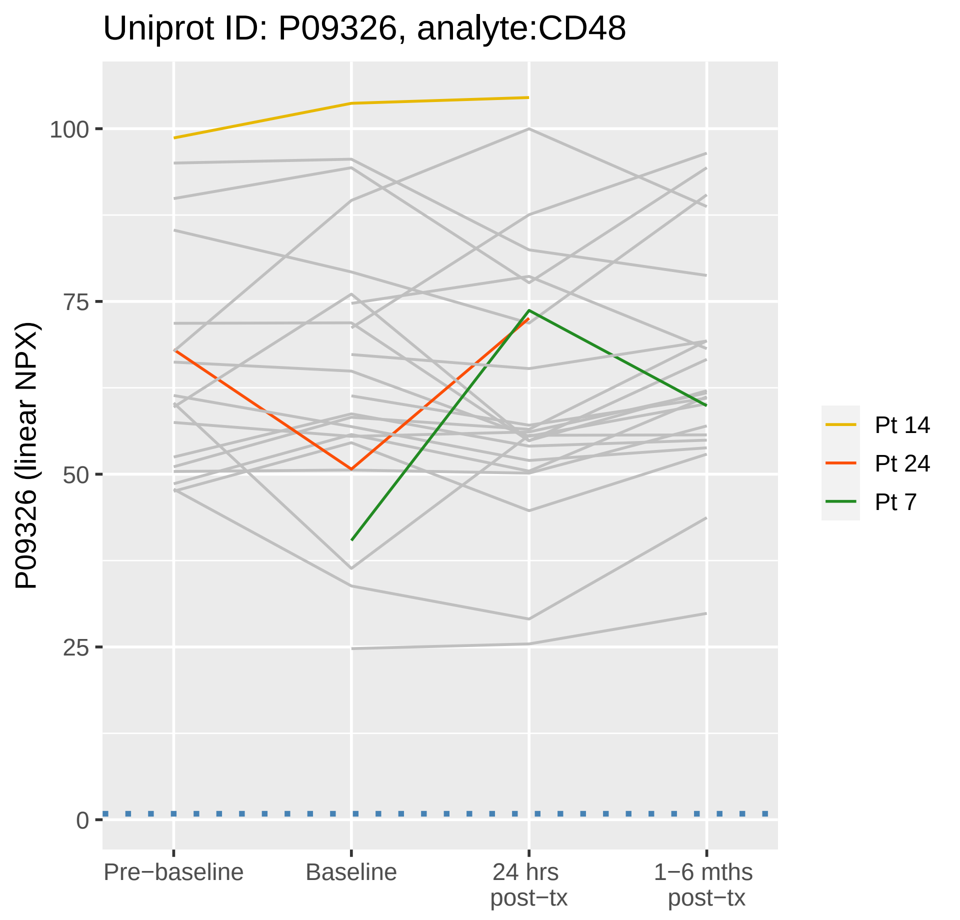

Uniprot ID: P09326, analyte:CD48
P09326 (linear NPX)
100
75
50
25
0
Pre−baseline
Baseline
24 hrs post−tx
1−6 mths post−tx
Pt 14
Pt 24
Pt 7

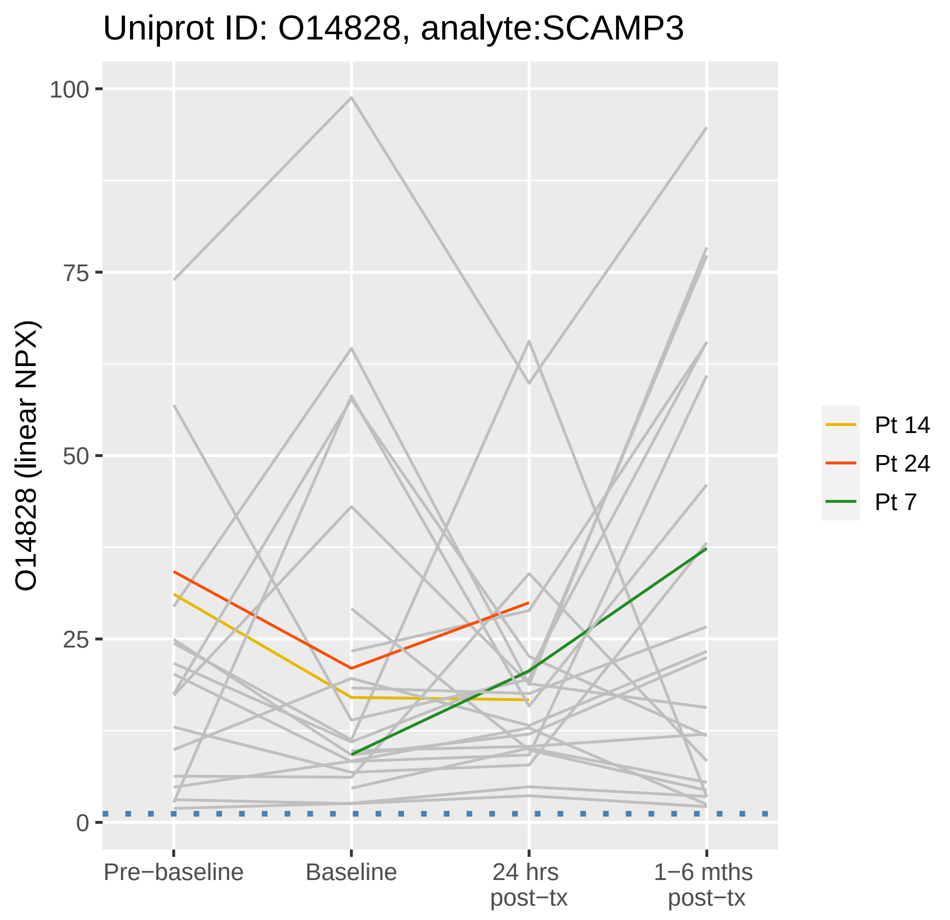

Uniprot ID: O14828, analyte:SCAMP3
O14828 (linear NPX)
100
75
50
25
0
Pre−baseline
Baseline
24 hrs post−tx
1−6 mths post−tx
Pt 14
Pt 24
Pt 7

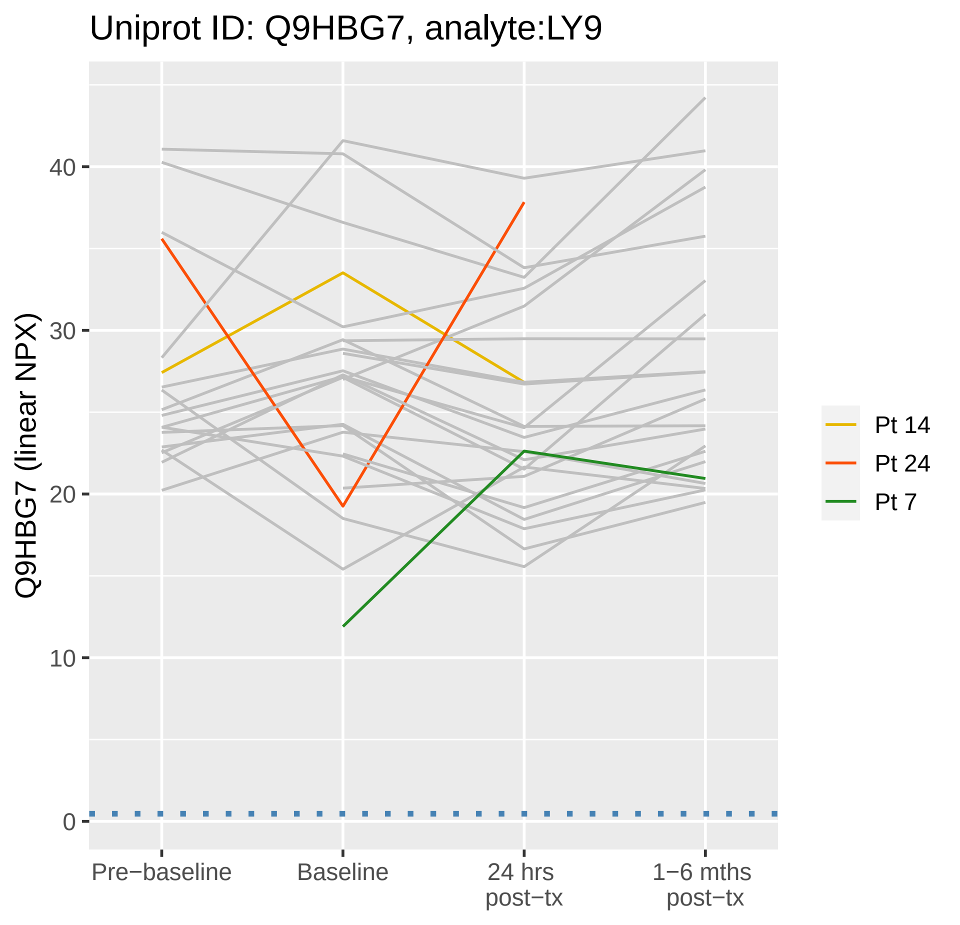

Uniprot ID: Q9HBG7, analyte:LY9
Q9HBG7 (linear NPX)
40
30
20
10
0
Pre−baseline
Baseline
24 hrs post−tx
1−6 mths post−tx
Pt 14
Pt 24
Pt 7

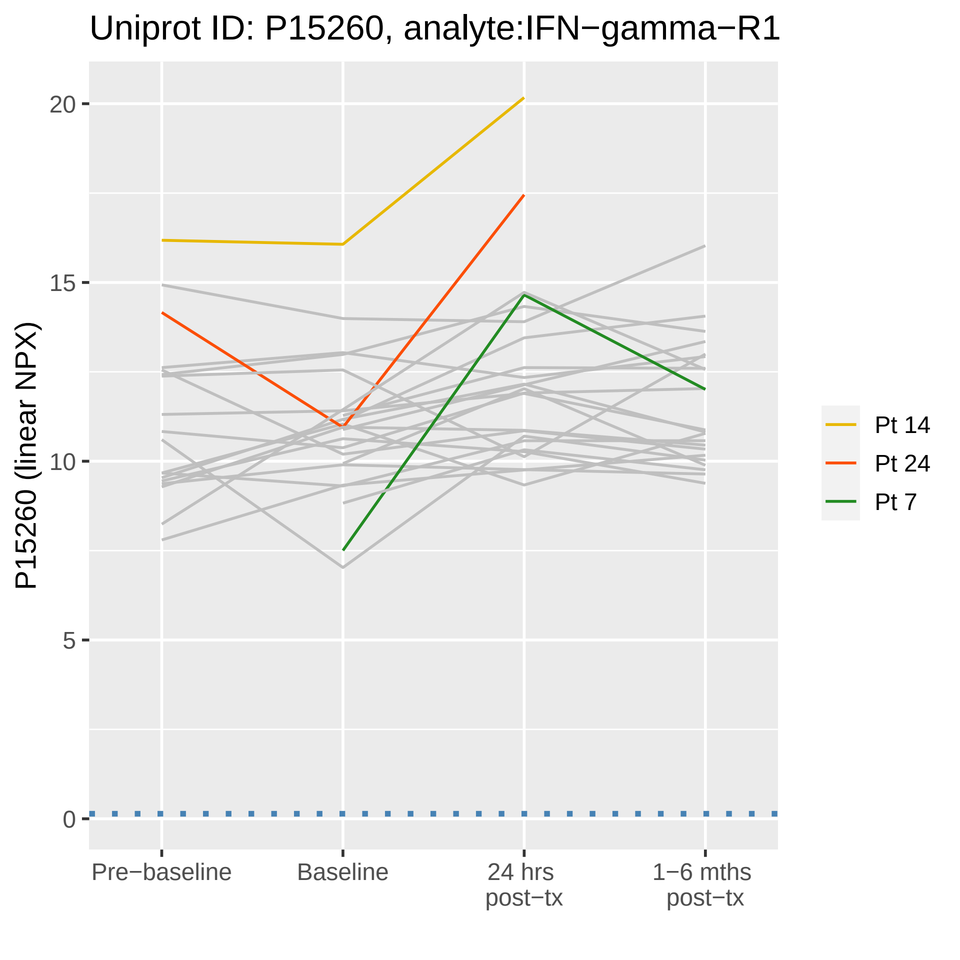

Uniprot ID: P15260, analyte:IFN−gamma−R1
P15260 (linear NPX)
20
15
10
5
0
Pre−baseline
Baseline
24 hrs post−tx
1−6 mths post−tx
Pt 14
Pt 24
Pt 7

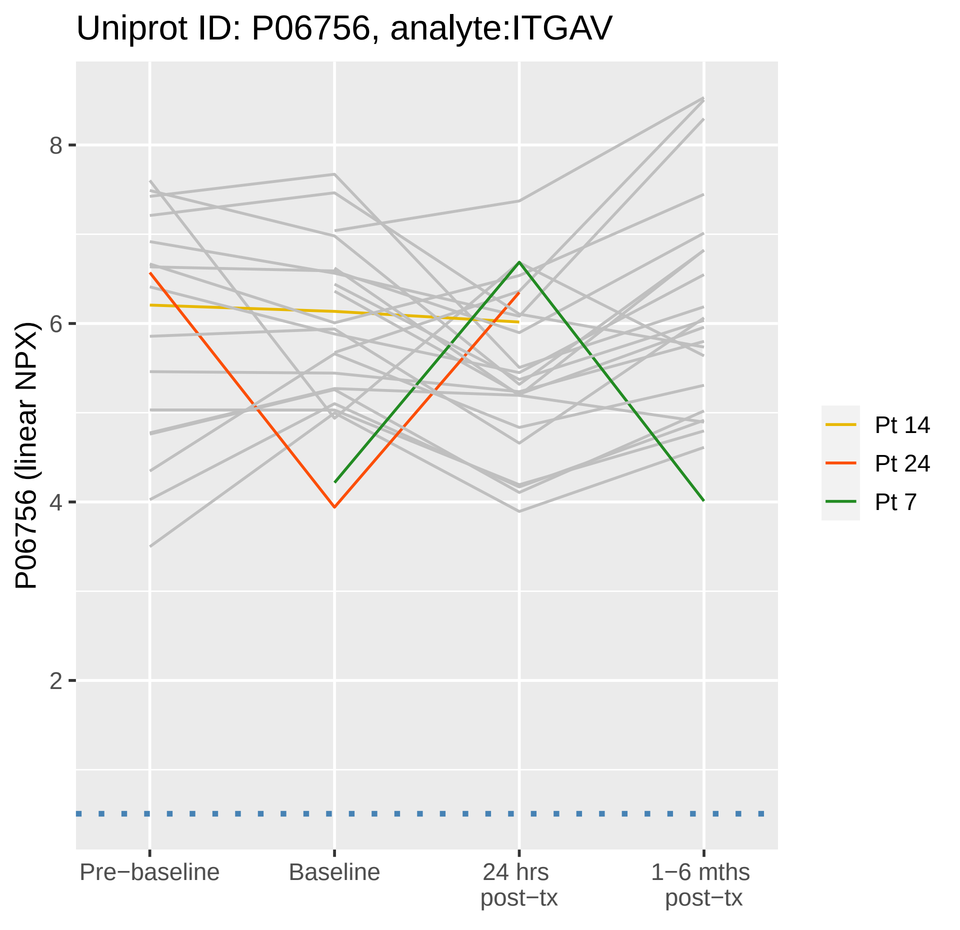
Uniprot ID: P06756, analyte:ITGAV
P06756 (linear NPX)
8
6
4
2
Pre−baseline
Baseline
24 hrs post−tx
1−6 mths post−tx
Pt 14
Pt 24
Pt 7

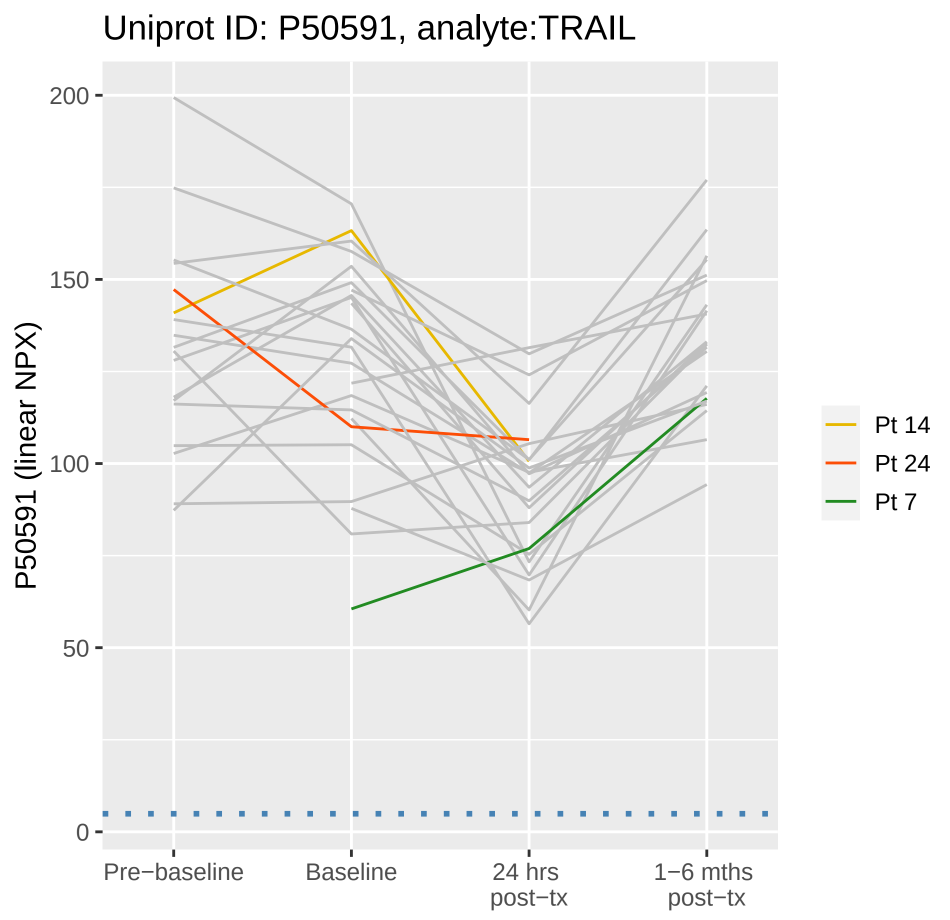

Uniprot ID: P50591, analyte:TRAIL
P50591 (linear NPX)
200
150
100
50
0
Pre−baseline
Baseline
24 hrs post−tx
1−6 mths post−tx
Pt 14
Pt 24
Pt 7

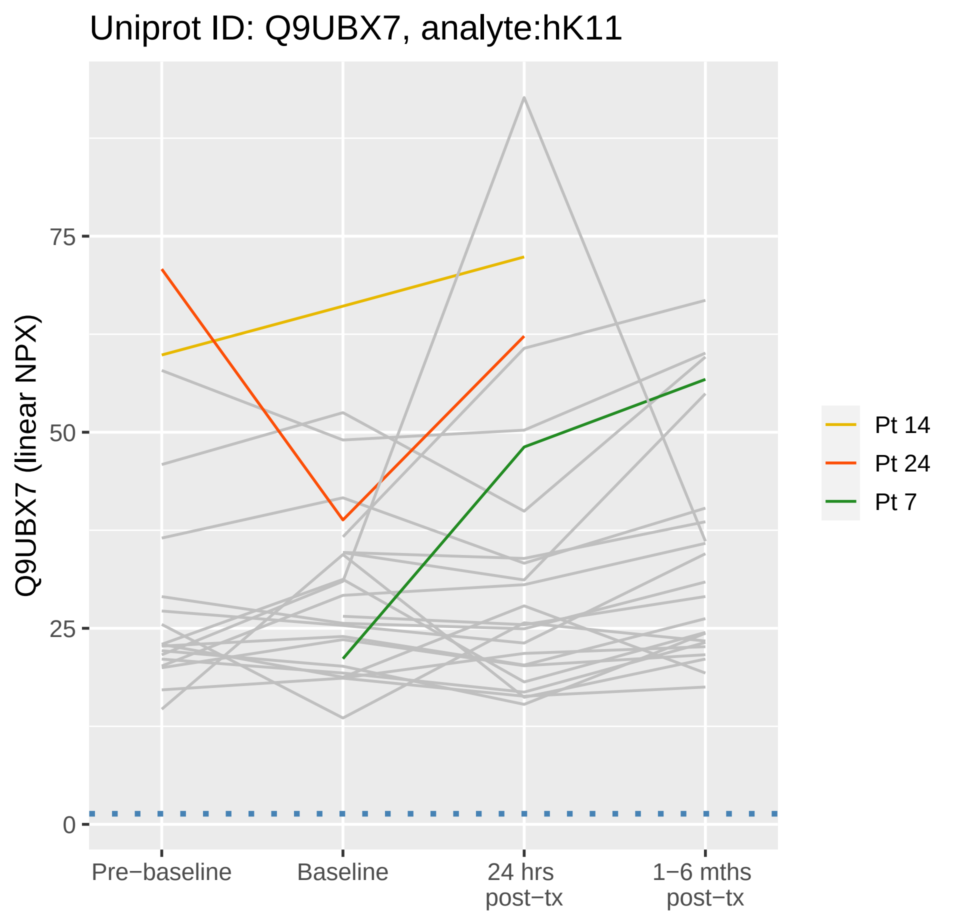

Uniprot ID: Q9UBX7, analyte:hK11
Q9UBX7 (linear NPX)
75
50
25
0
Pre−baseline
Baseline
24 hrs post−tx
1−6 mths post−tx
Pt 14
Pt 24
Pt 7

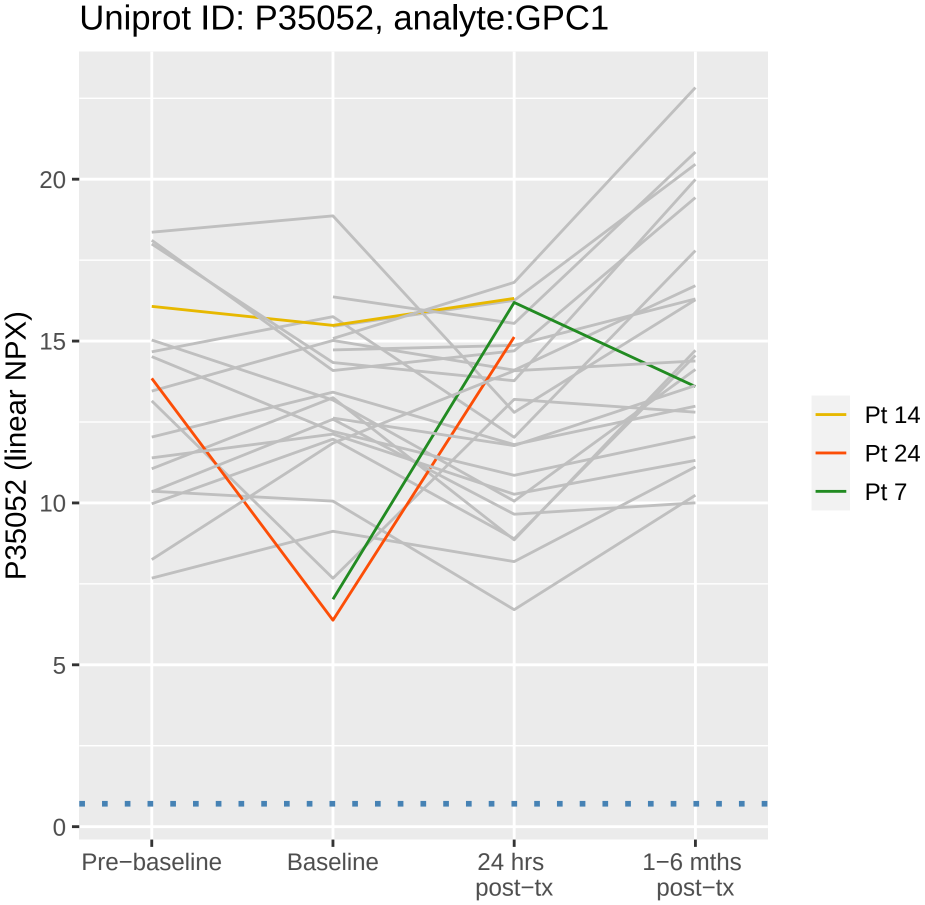
Uniprot ID: P35052, analyte:GPC1
P35052 (linear NPX)
0
5
10
15
20
Pre−baseline
Baseline
24 hrs post−tx
1−6 mths post−tx
Pt 14
Pt 24
Pt 7

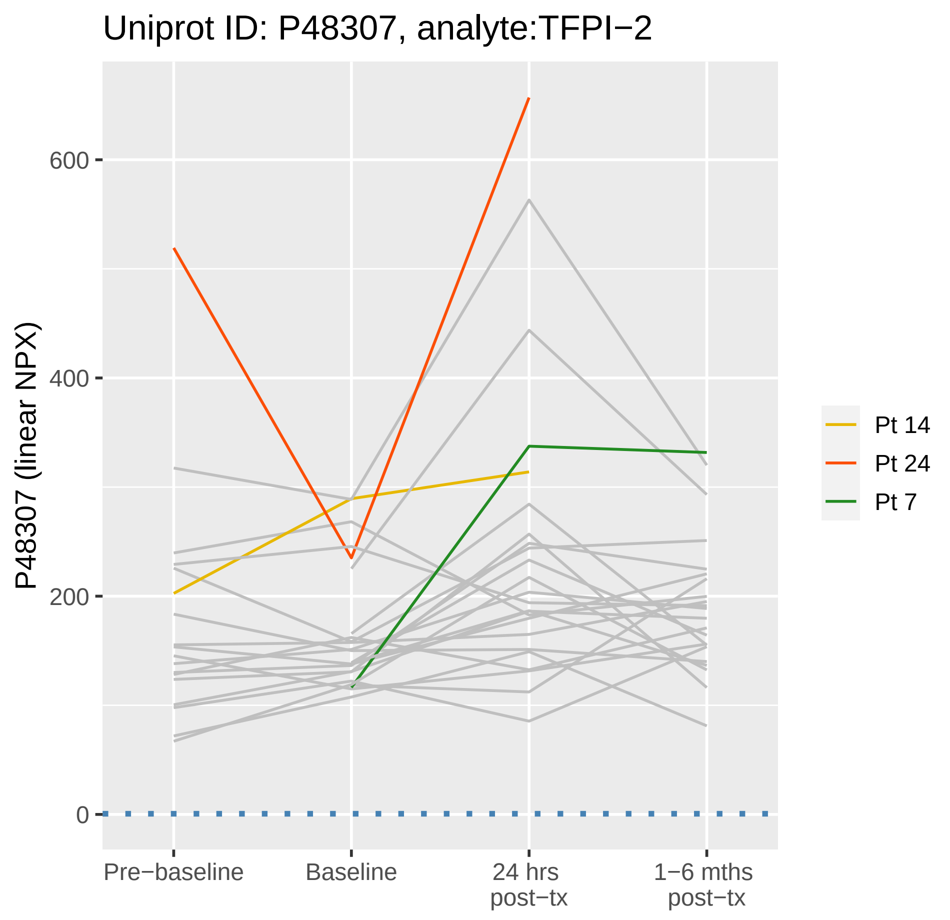

Uniprot ID: P48307, analyte:TFPI−2
P48307 (linear NPX)
600
400
200
0
Pre−baseline
Baseline
24 hrs
post−tx
1−6 mths
post−tx
Pt 14
Pt 24
Pt 7

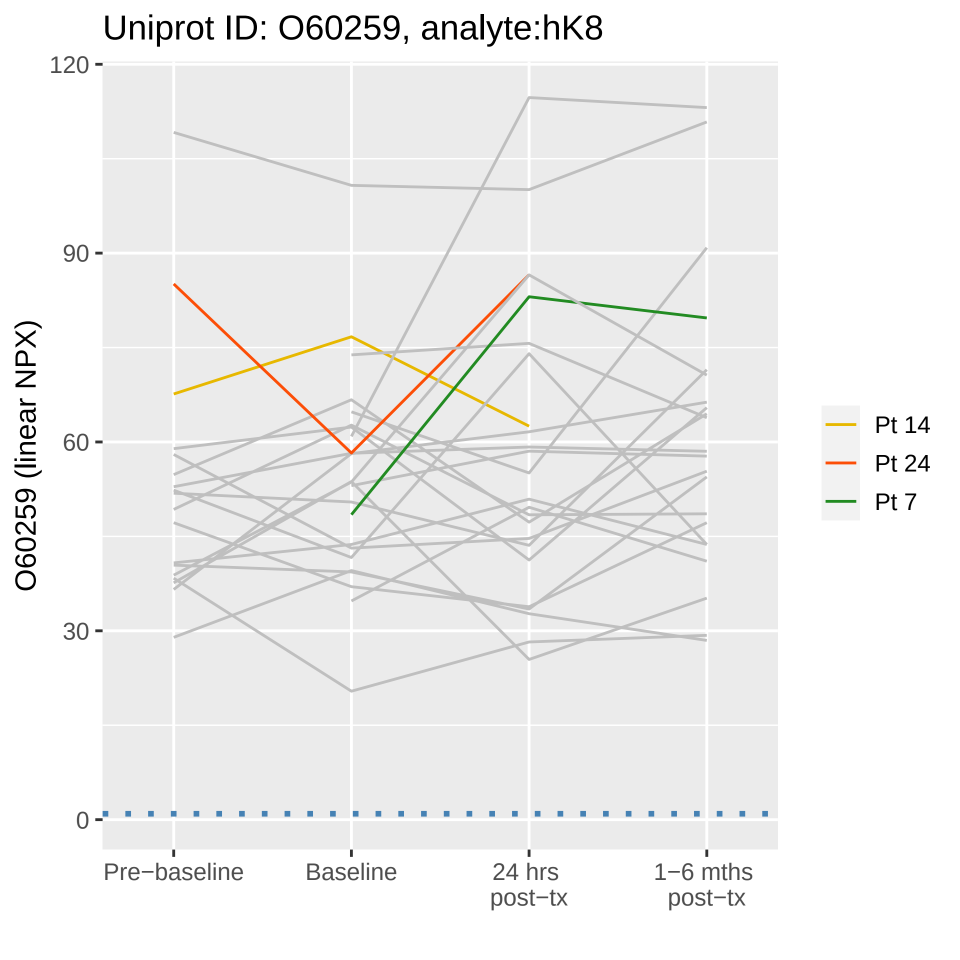

Uniprot ID: O60259, analyte:hK8
O60259 (linear NPX)
120
90
60
30
0
Pre−baseline
Baseline
24 hrs post−tx
1−6 mths post−tx
Pt 14
Pt 24
Pt 7

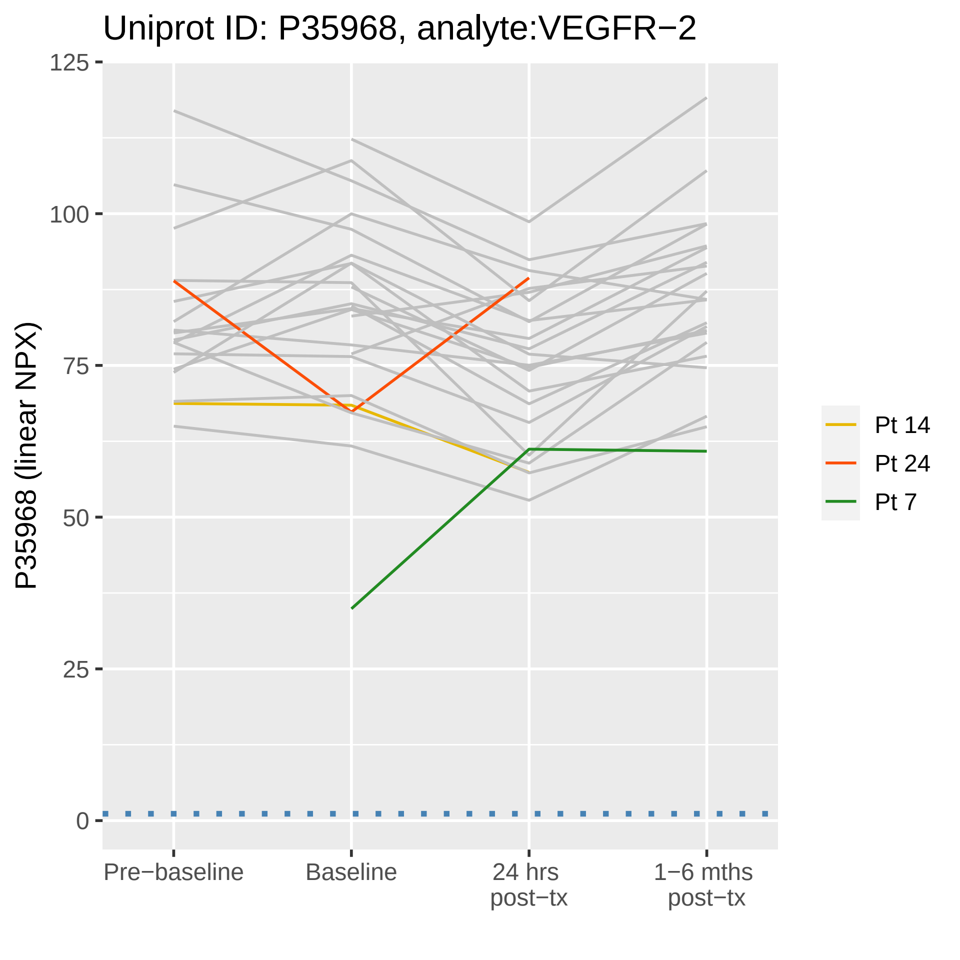
Uniprot ID: P35968, analyte:VEGFR−2
P35968 (linear NPX)
125
100
75
50
25
0
Pre−baseline
Baseline
24 hrs post−tx
1−6 mths post−tx
Pt 14
Pt 24
Pt 7

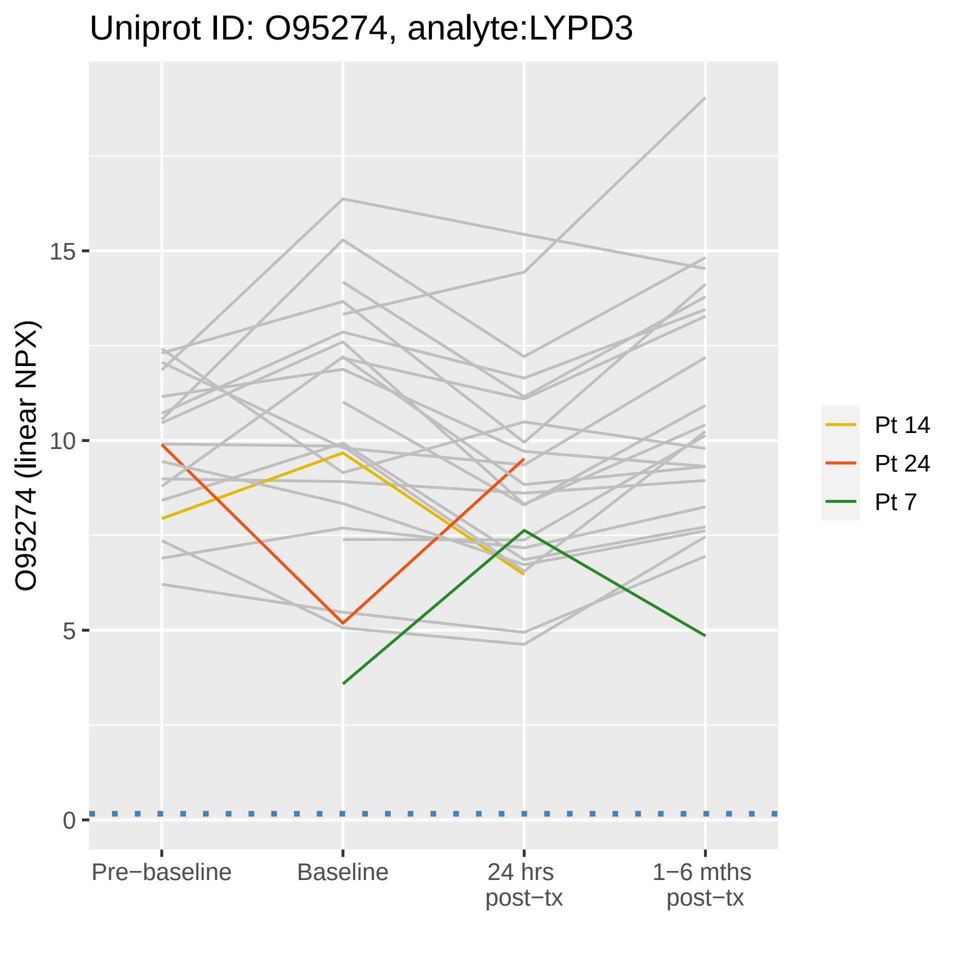

Uniprot ID: O95274, analyte:LYPD3
O95274 (linear NPX)
15
10
5
0
Pre−baseline
Baseline
24 hrs post−tx
1−6 mths post−tx
Pt 14
Pt 24
Pt 7

Uniprot ID: O00592, analyte:PODXL

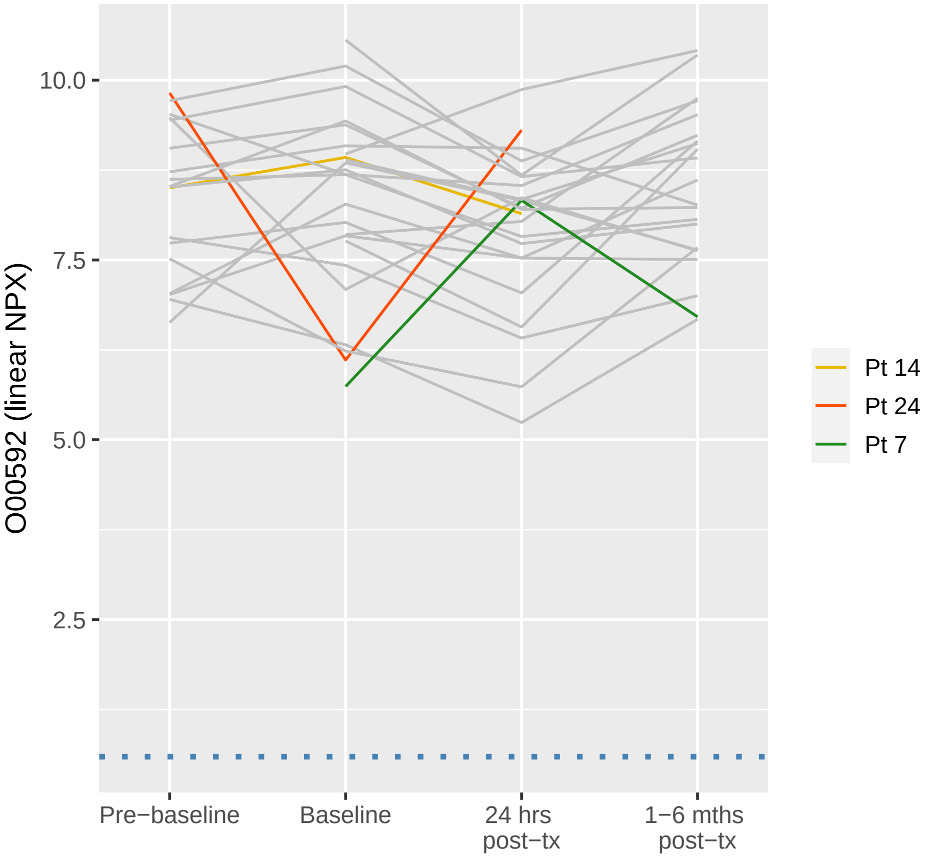

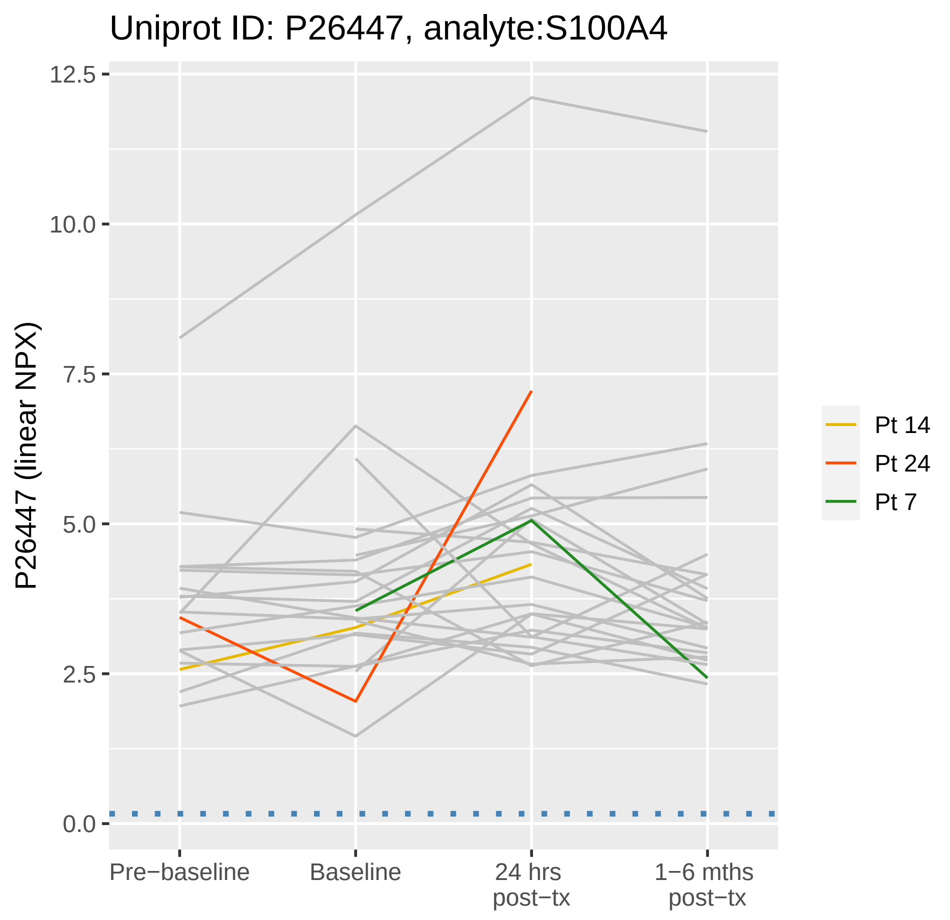

Uniprot ID: P26447, analyte:S100A4
P26447 (linear NPX)
12.5
10.0
7.5
5.0
2.5
0.0
Pre−baseline
Baseline
24 hrs post−tx
1−6 mths post−tx
Pt 14
Pt 24
Pt 7

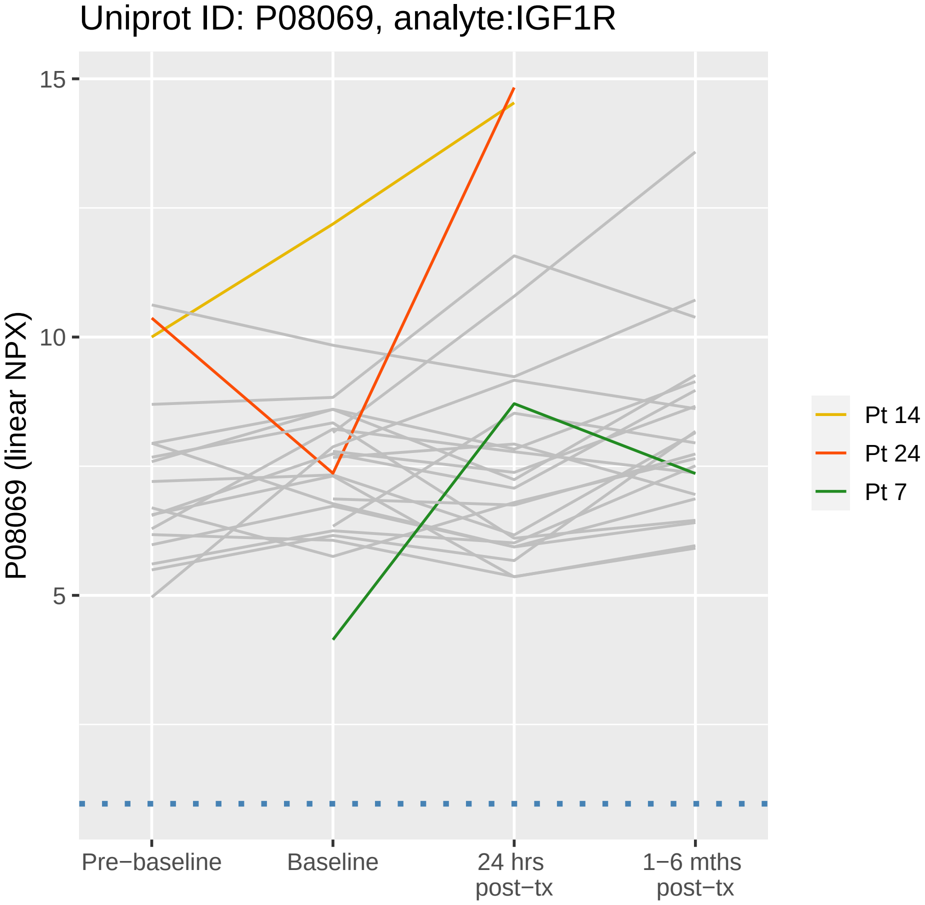

Uniprot ID: P08069, analyte:IGF1R
P08069 (linear NPX)
15
10
5
Pre−baseline
Baseline
24 hrs
post−tx
1−6 mths
post−tx
Pt 14
Pt 24
Pt 7

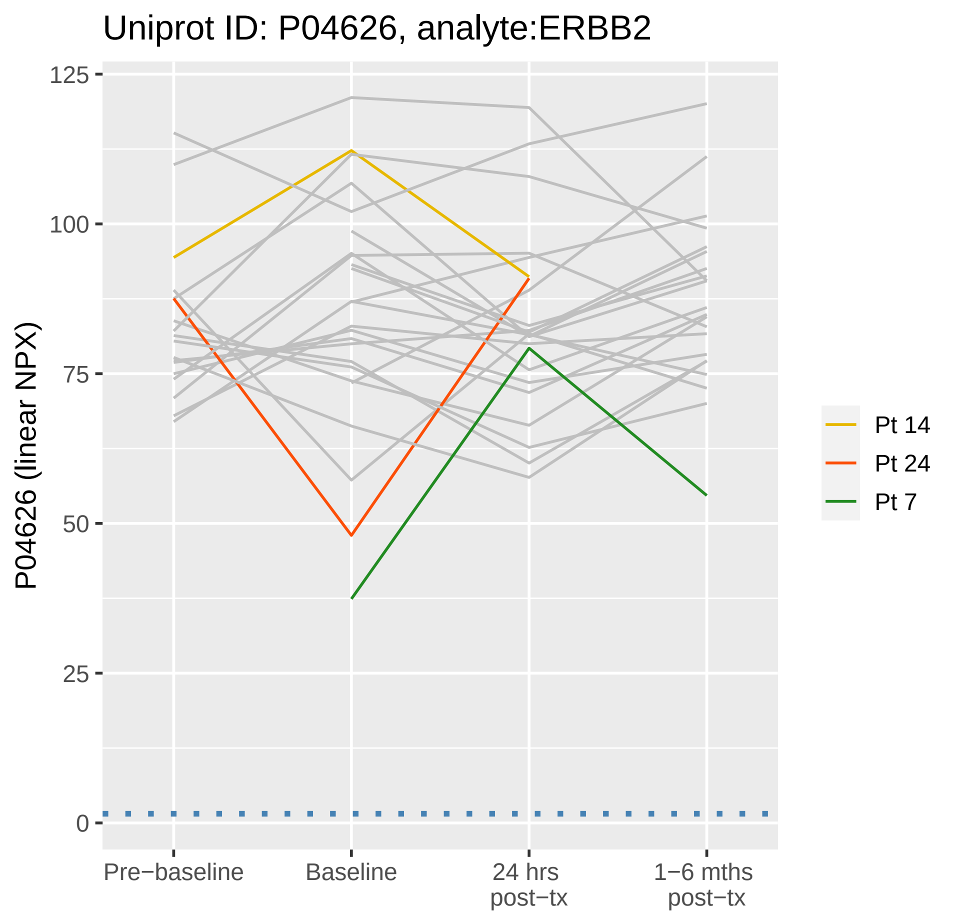

Uniprot ID: P04626, analyte:ERBB2
P04626 (linear NPX)
125
100
75
50
25
0
Pre−baseline
Baseline
24 hrs post−tx
1−6 mths post−tx
Pt 14
Pt 24
Pt 7

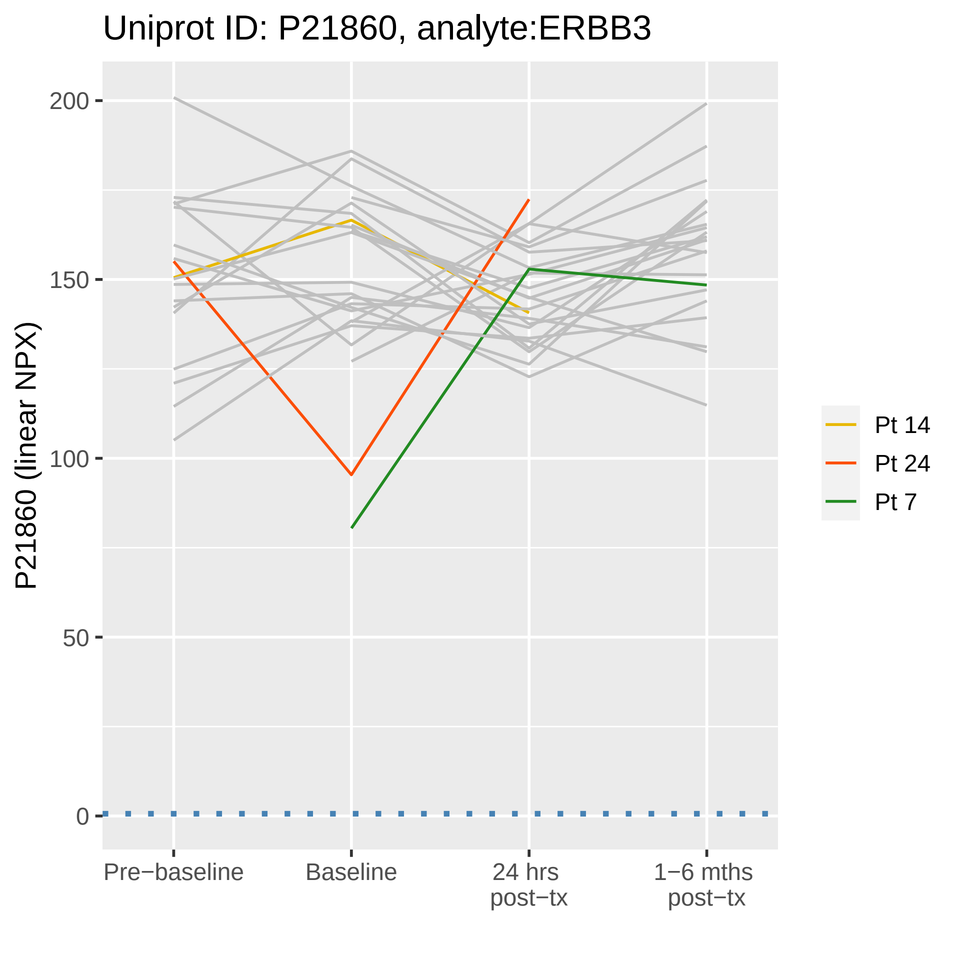

Uniprot ID: P21860, analyte:ERBB3
P21860 (linear NPX)
200
150
100
50
0
Pre−baseline
Baseline
24 hrs post−tx
1−6 mths post−tx
Pt 14
Pt 24
Pt 7

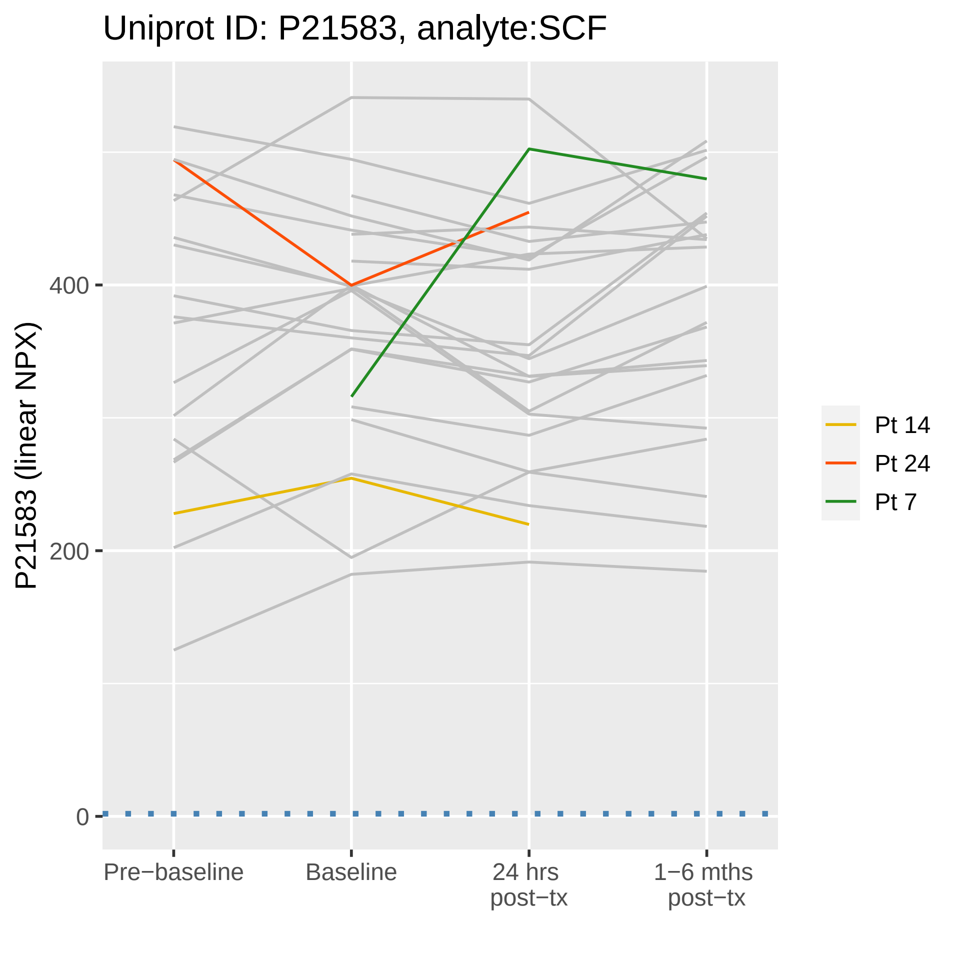

Uniprot ID: P21583, analyte:SCF
P21583 (linear NPX)
400
200
0
Pre−baseline
Baseline
24 hrs
post−tx
1−6 mths
post−tx
Pt 14
Pt 24
Pt 7

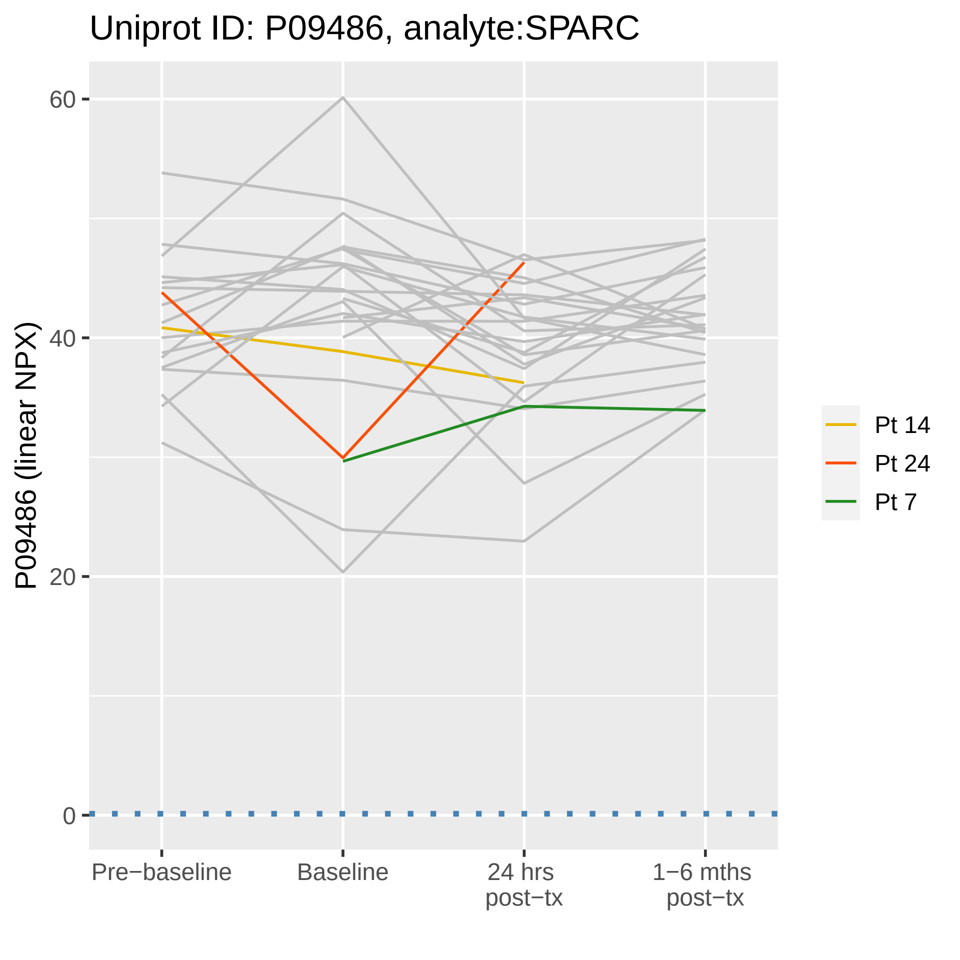

Uniprot ID: P09486, analyte:SPARC
P09486 (linear NPX)
60
40
20
0
Pre−baseline
Baseline
24 hrs post−tx
1−6 mths post−tx
Pt 14
Pt 24
Pt 7

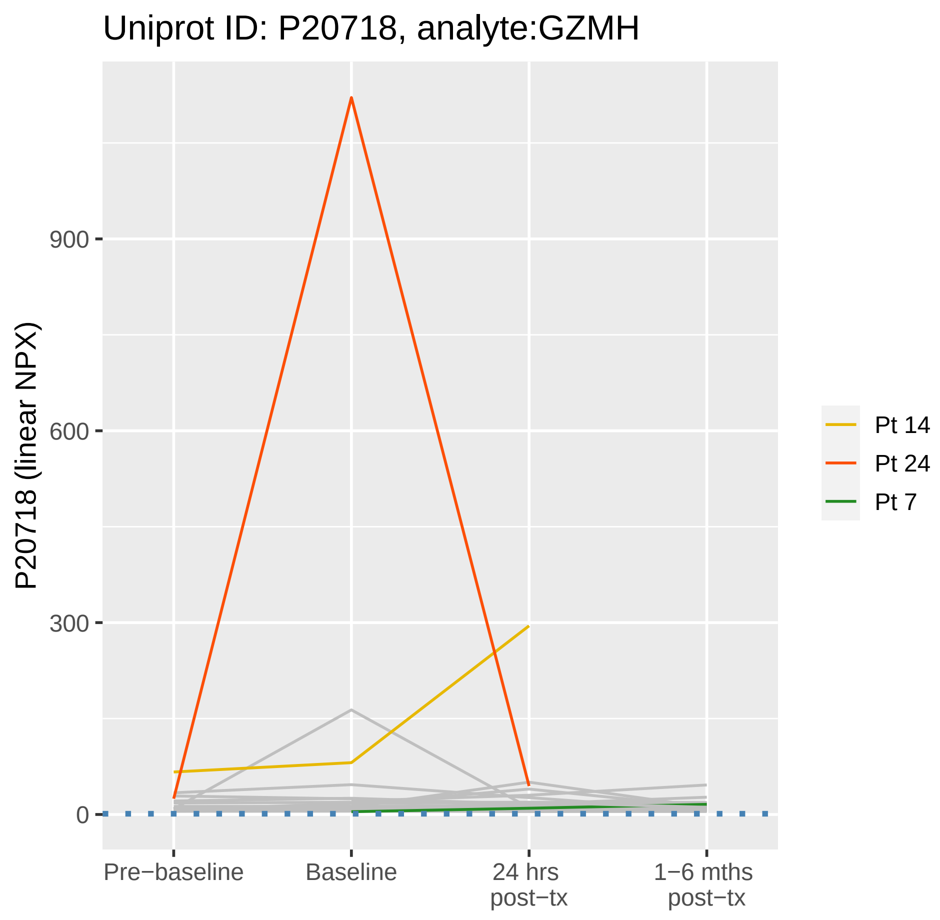
Uniprot ID: P20718, analyte:GZMH
P20718 (linear NPX)
900
600
300
0
Pre−baseline
Baseline
24 hrs
post−tx
1−6 mths
post−tx
Pt 14
Pt 24
Pt 7

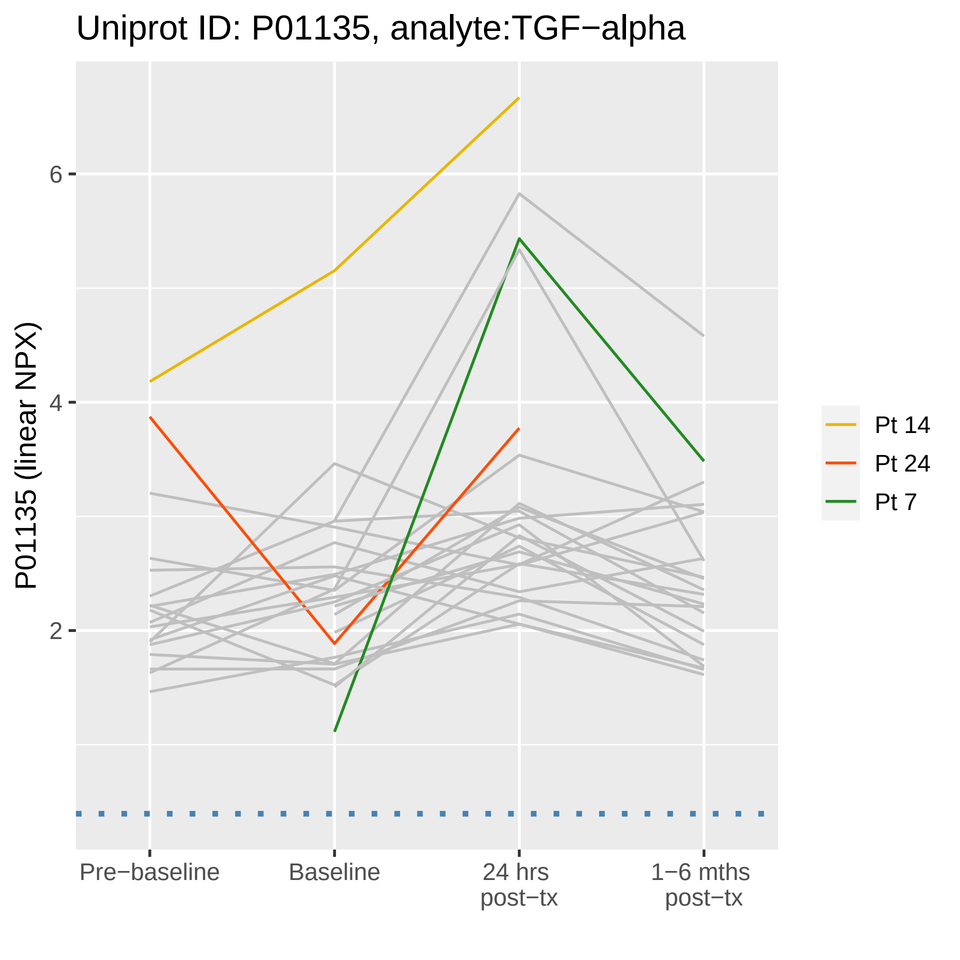
Uniprot ID: P01135, analyte:TGF−alpha
P01135 (linear NPX)
6
4
2
Pre−baseline
Baseline
24 hrs
post−tx
1−6 mths
post−tx
Pt 14
Pt 24
Pt 7

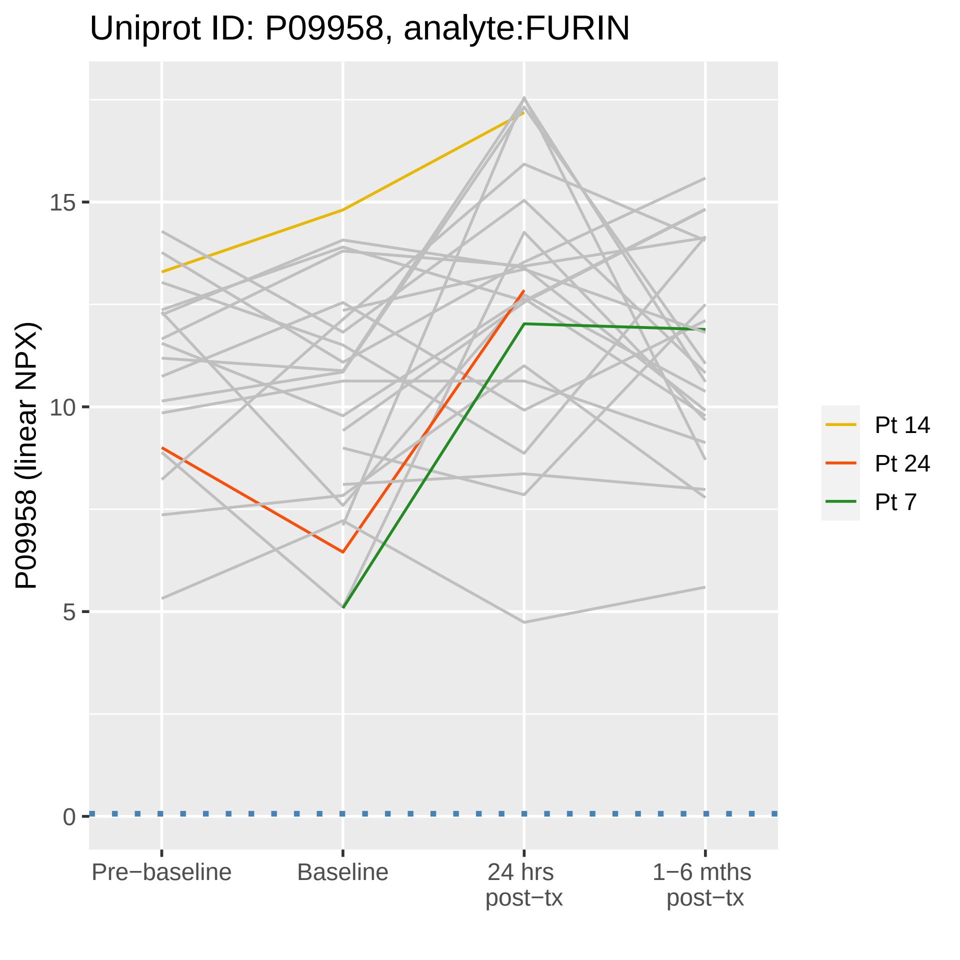
Uniprot ID: P09958, analyte:FURIN
P09958 (linear NPX)
15
10
5
0
Pre−baseline
Baseline
24 hrs post−tx
1−6 mths post−tx
Pt 14
Pt 24
Pt 7

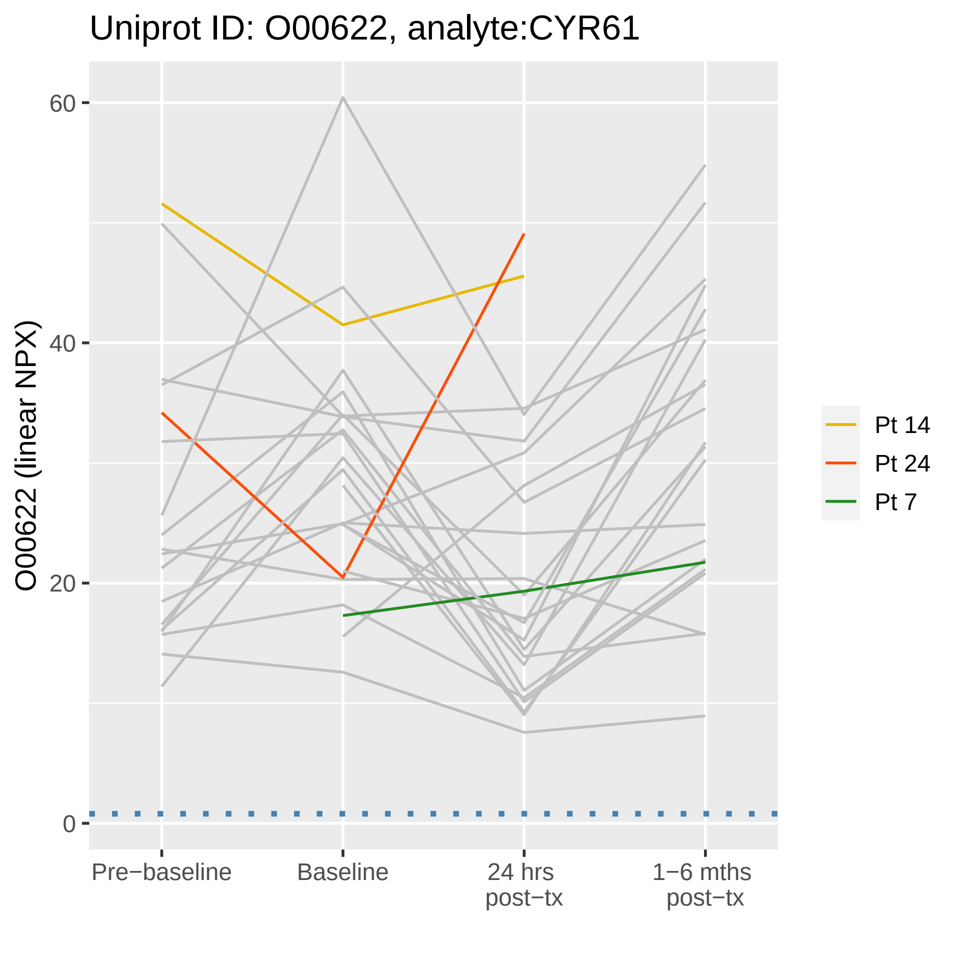

Uniprot ID: O00622, analyte:CYR61
O00622 (linear NPX)
60
40
20
0
Pre−baseline
Baseline
24 hrs
post−tx
1−6 mths
post−tx
Pt 14
Pt 24
Pt 7

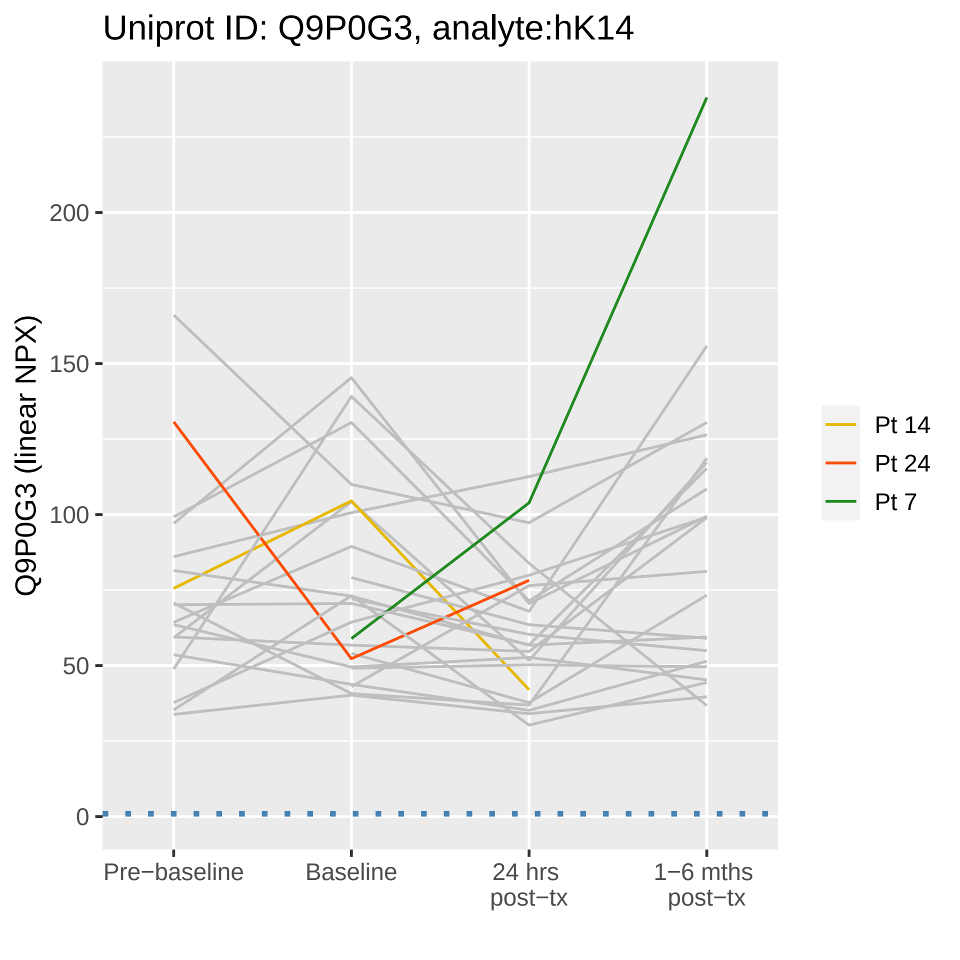

Uniprot ID: Q9P0G3, analyte:hK14
Q9P0G3 (linear NPX)
200
150
100
50
0
Pre−baseline
Baseline
24 hrs post−tx
1−6 mths post−tx
Pt 14
Pt 24
Pt 7

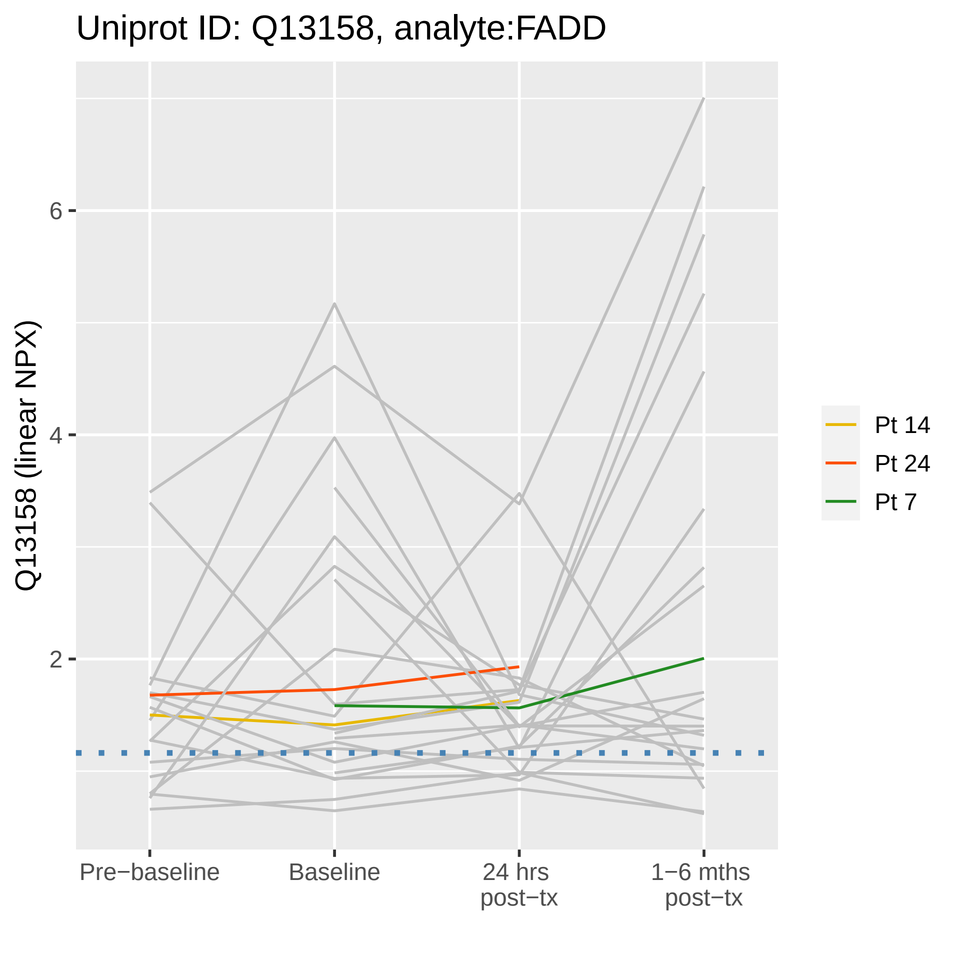
Uniprot ID: Q13158, analyte:FADD
Q13158 (linear NPX)
6
4
2
Pre−baseline
Baseline
24 hrs post−tx
1−6 mths post−tx
Pt 14
Pt 24
Pt 7

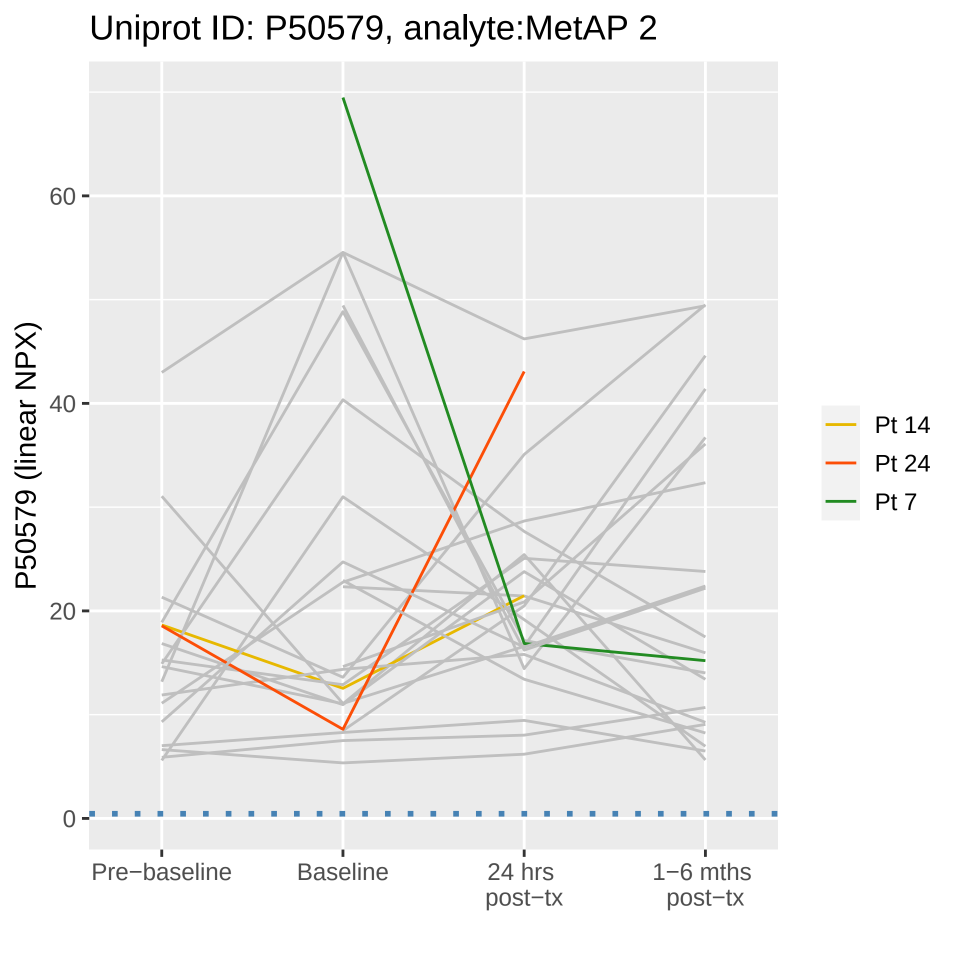
Uniprot ID: P50579, analyte:MetAP 2
P50579 (linear NPX)
60
40
20
0
Pre−baseline
Baseline
24 hrs post−tx
1−6 mths post−tx
Pt 14
Pt 24
Pt 7

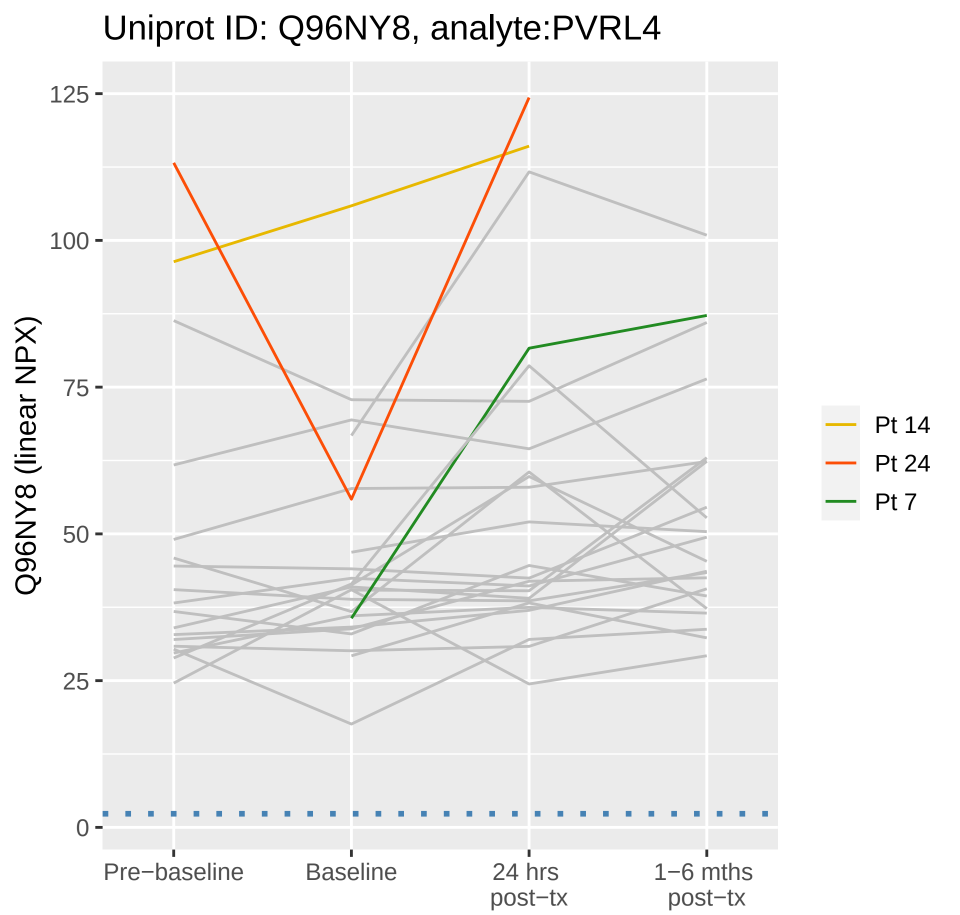

Uniprot ID: Q96NY8, analyte:PVRL4
Q96NY8 (linear NPX)
125
100
75
50
25
0
Pre−baseline
Baseline
24 hrs post−tx
1−6 mths post−tx
Pt 14
Pt 24
Pt 7

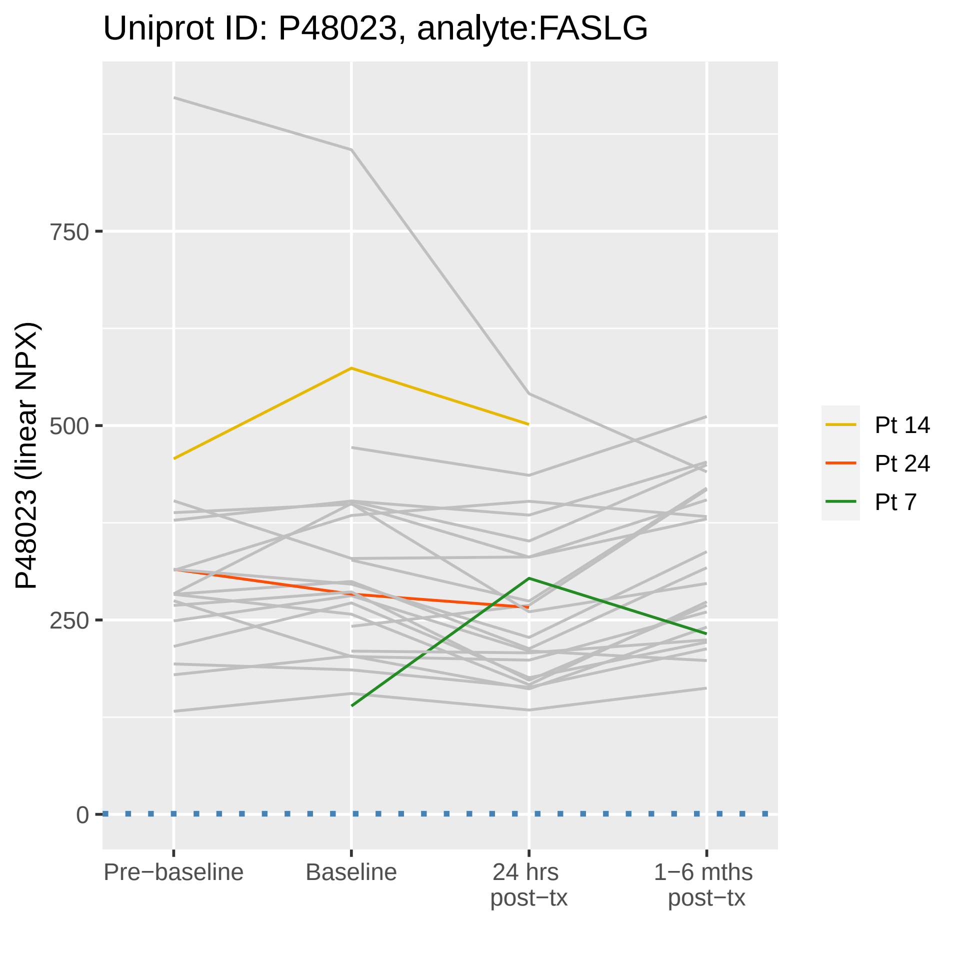

Uniprot ID: P48023, analyte:FASLG
P48023 (linear NPX)
750
500
250
0
Pre−baseline
Baseline
24 hrs post−tx
1−6 mths post−tx
Pt 14
Pt 24
Pt 7

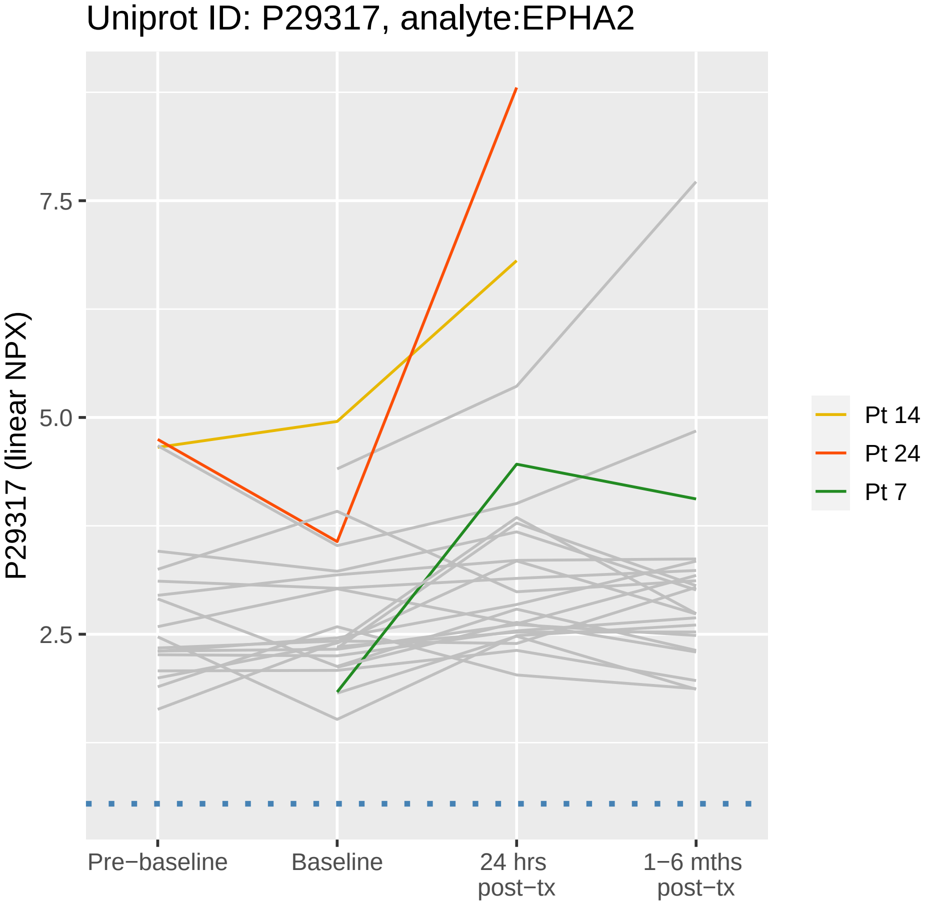

Uniprot ID: P29317, analyte:EPHA2
P29317 (linear NPX)
7.5
5.0
2.5
Pre−baseline
Baseline
24 hrs post−tx
1−6 mths post−tx
Pt 14
Pt 24
Pt 7

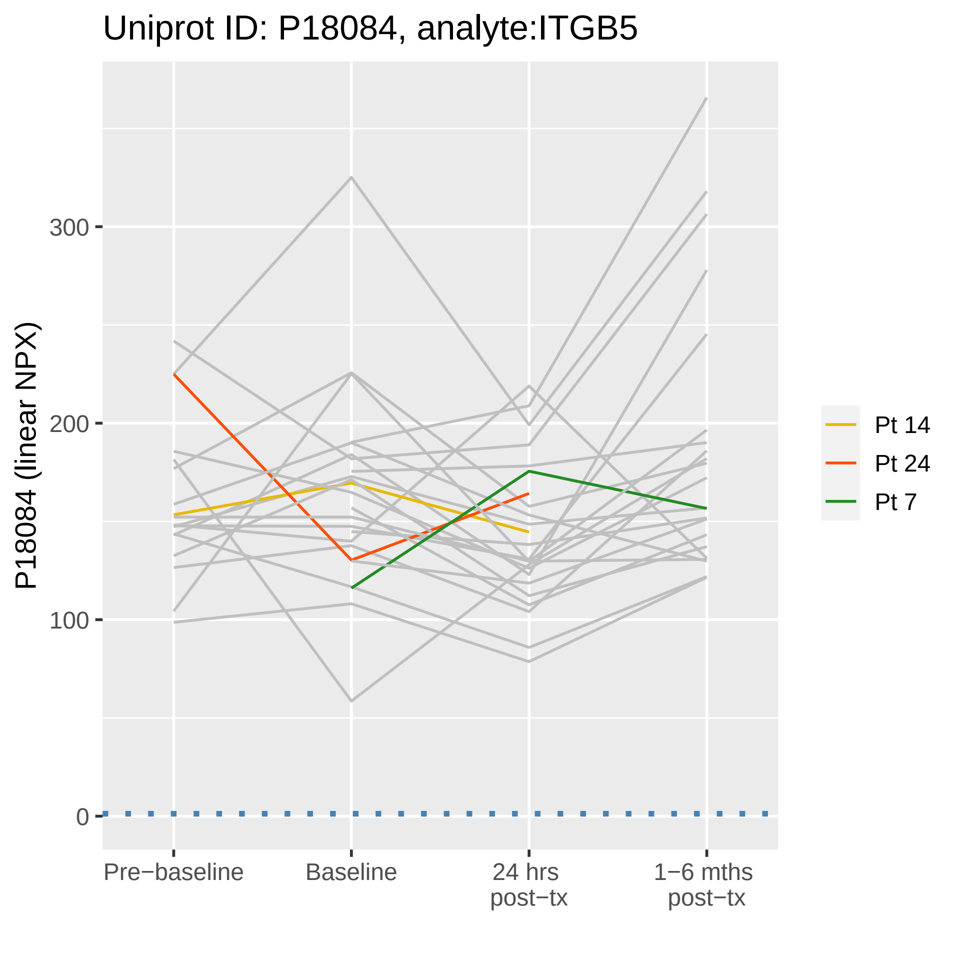
Uniprot ID: P18084, analyte:ITGB5
P18084 (linear NPX)
300
200
100
0
Pre−baseline
Baseline
24 hrs post−tx
1−6 mths post−tx
Pt 14
Pt 24
Pt 7

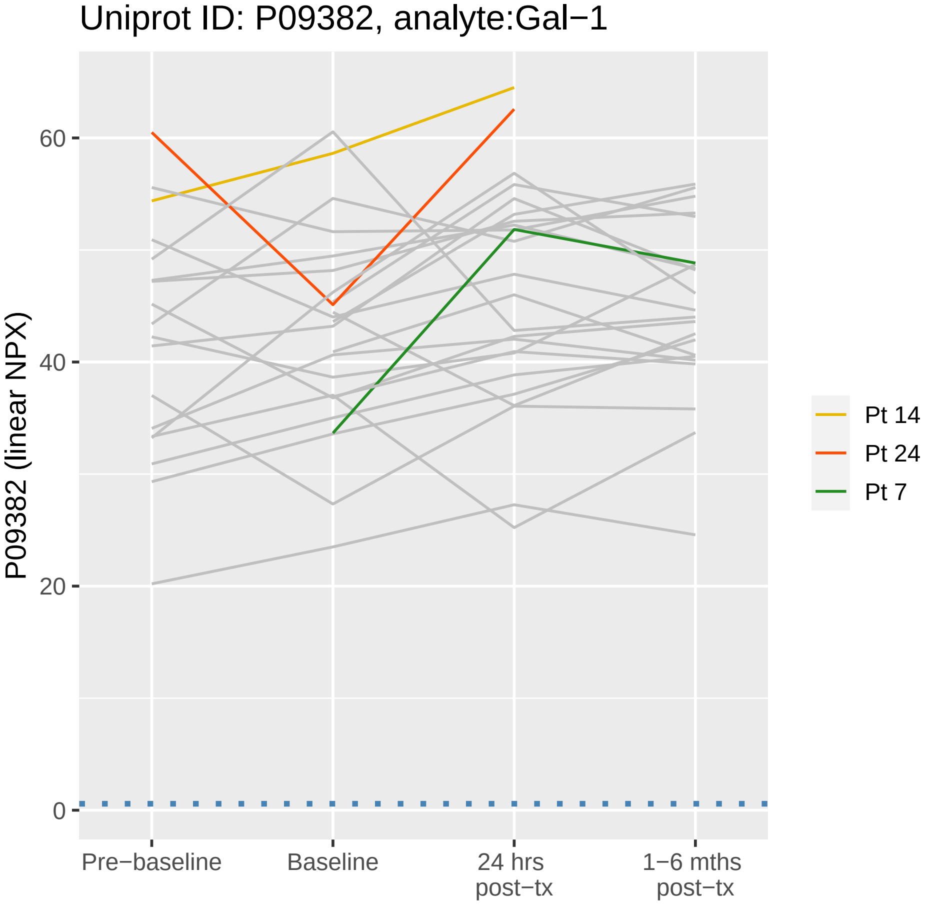
Uniprot ID: P09382, analyte:Gal−1
P09382 (linear NPX)
60
40
20
0
Pre−baseline
Baseline
24 hrs post−tx
1−6 mths post−tx
Pt 14
Pt 24
Pt 7

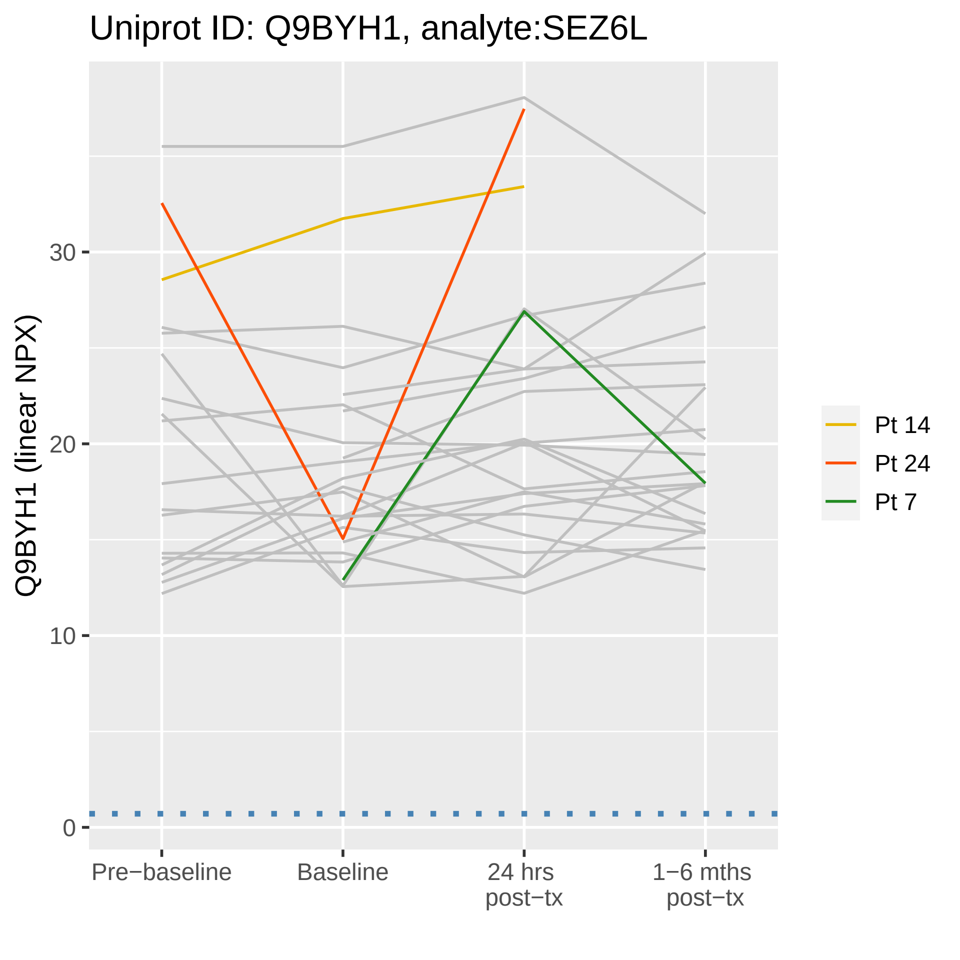

Uniprot ID: Q9BYH1, analyte:SEZ6L
Q9BYH1 (linear NPX)
30
20
10
0
Pre−baseline
Baseline
24 hrs post−tx
1−6 mths post−tx
Pt 14
Pt 24
Pt 7

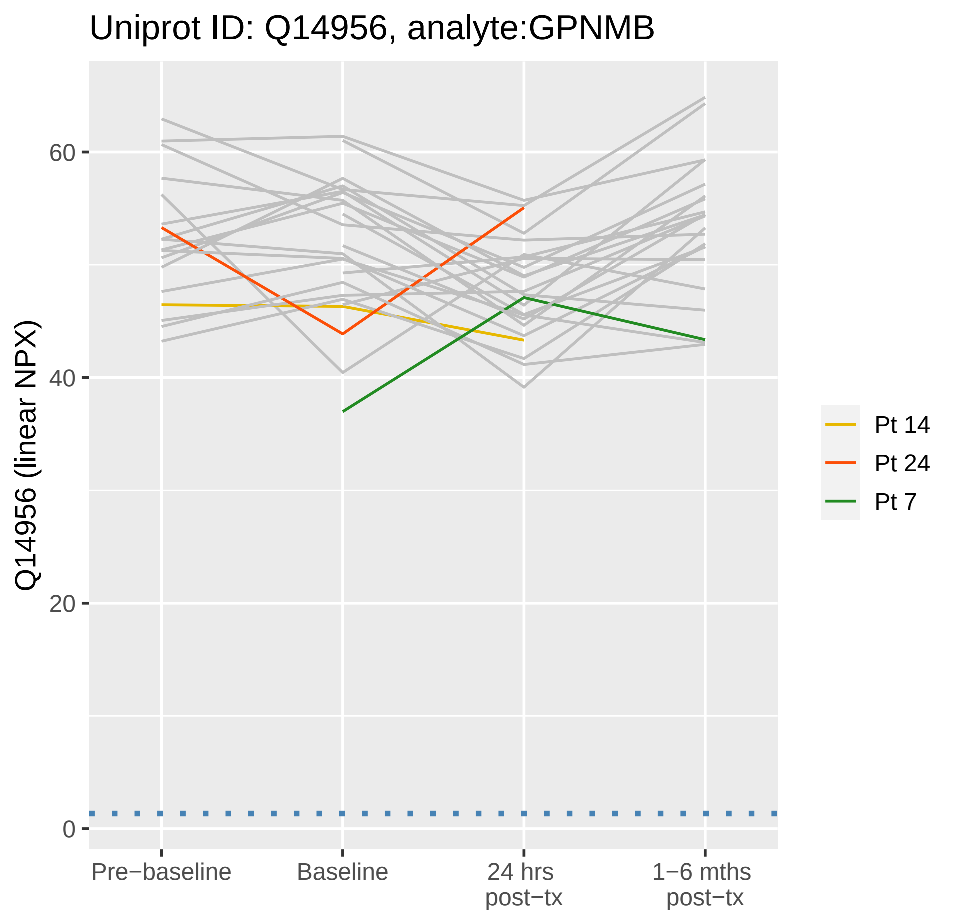
Uniprot ID: Q14956, analyte:GPNMB
Q14956 (linear NPX)
60
40
20
0
Pre−baseline
Baseline
24 hrs post−tx
1−6 mths post−tx
Pt 14
Pt 24
Pt 7

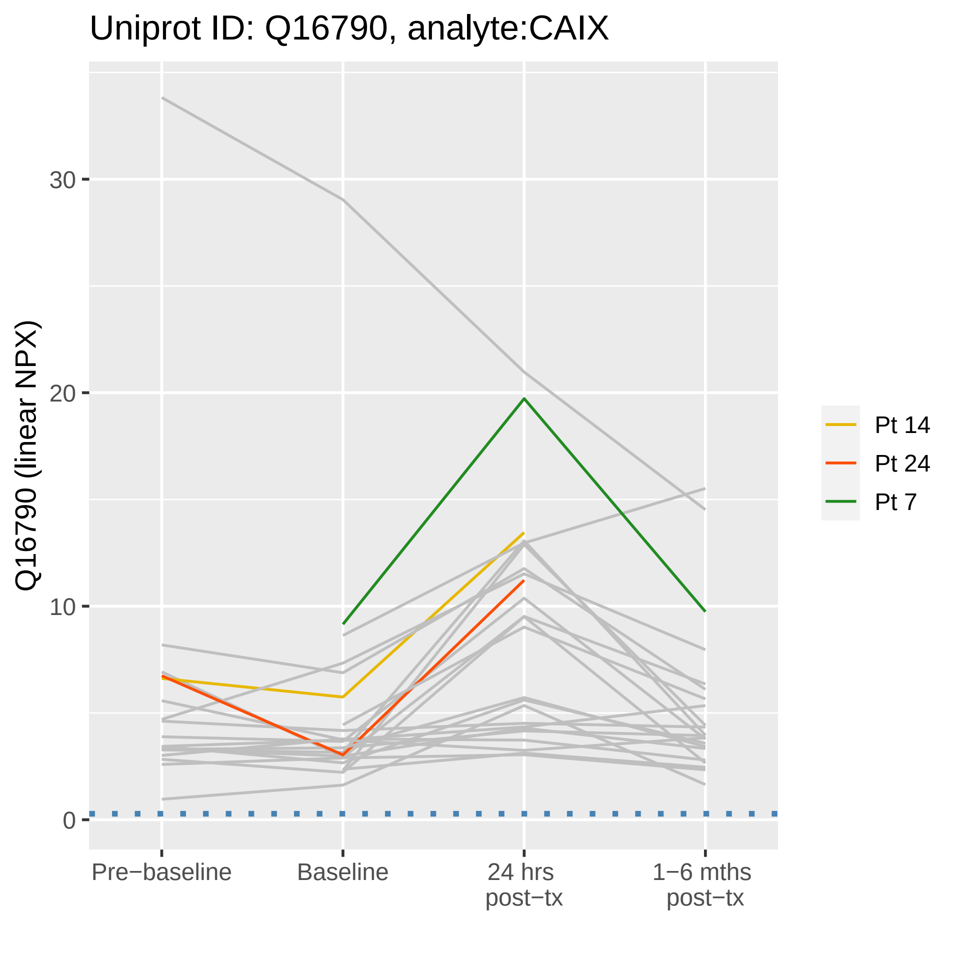

Uniprot ID: Q16790, analyte:CAIX
Q16790 (linear NPX)
30
20
10
0
Pre−baseline
Baseline
24 hrs
post−tx
1−6 mths
post−tx
Pt 14
Pt 24
Pt 7

Uniprot ID: Q16674, analyte:MIA

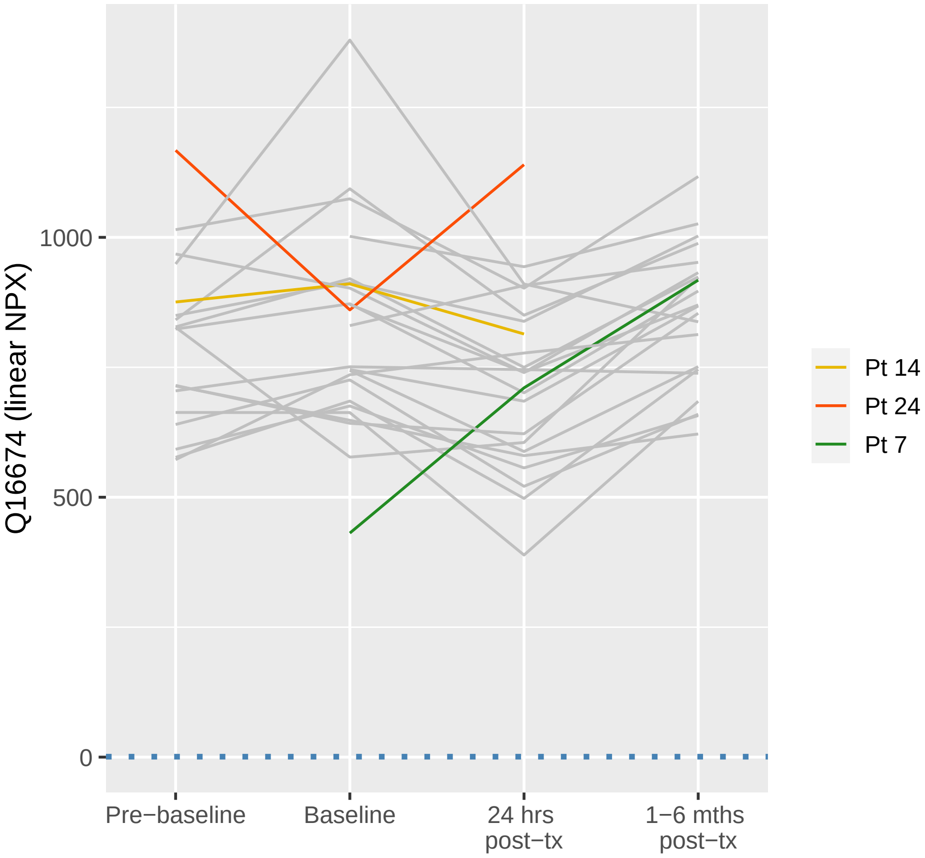

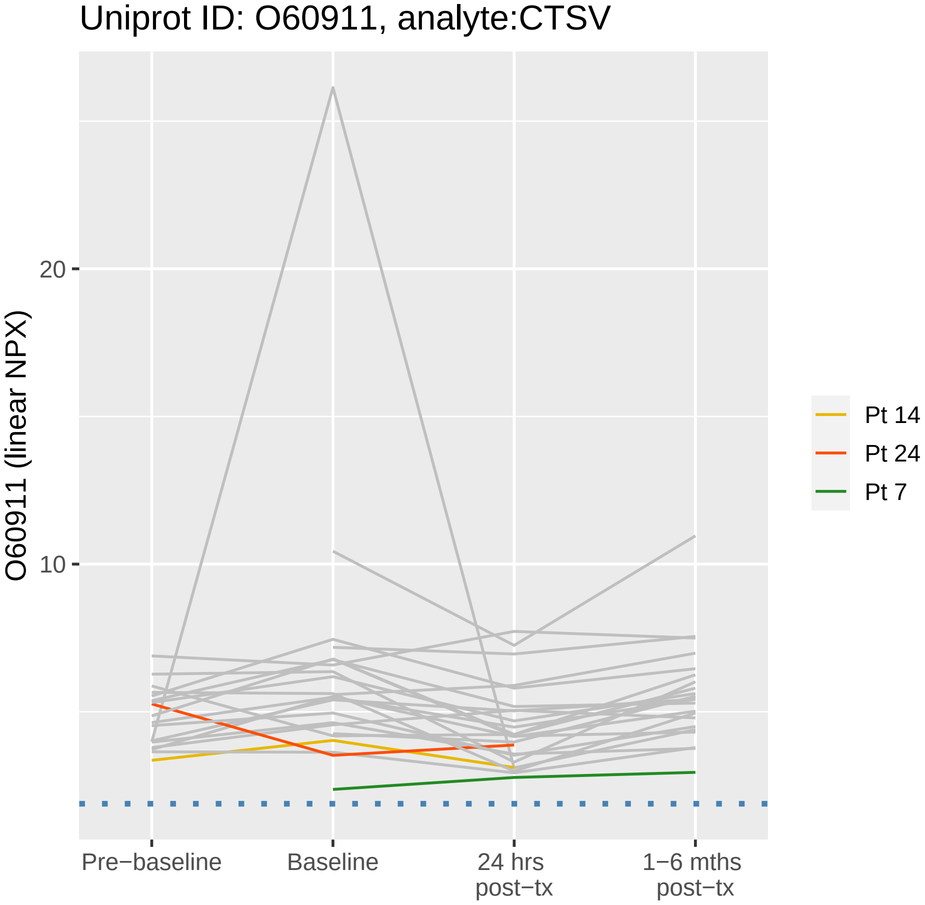
Uniprot ID: O60911, analyte:CTSV
O60911 (linear NPX)
20
10
Pre−baseline
Baseline
24 hrs post−tx
1−6 mths post−tx
Pt 14
Pt 24
Pt 7

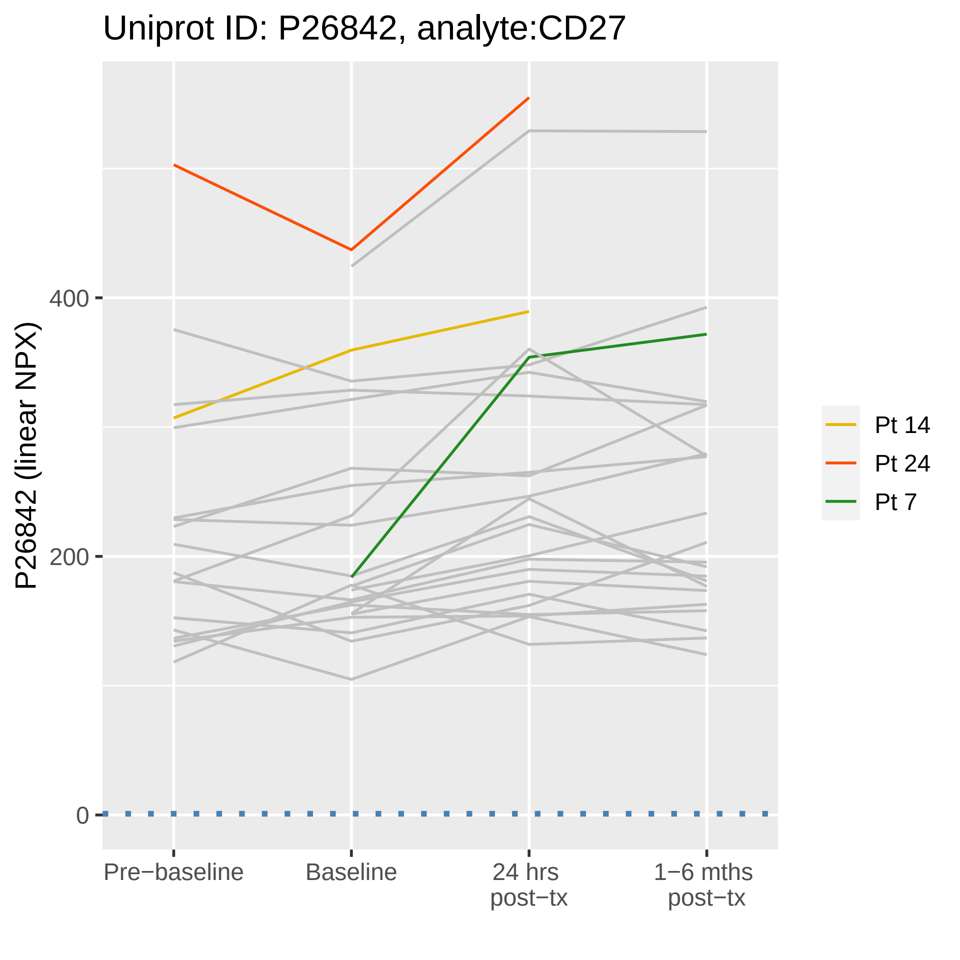

Uniprot ID: P26842, analyte:CD27
P26842 (linear NPX)
400
200
0
Pre−baseline
Baseline
24 hrs post−tx
1−6 mths post−tx
Pt 14
Pt 24
Pt 7

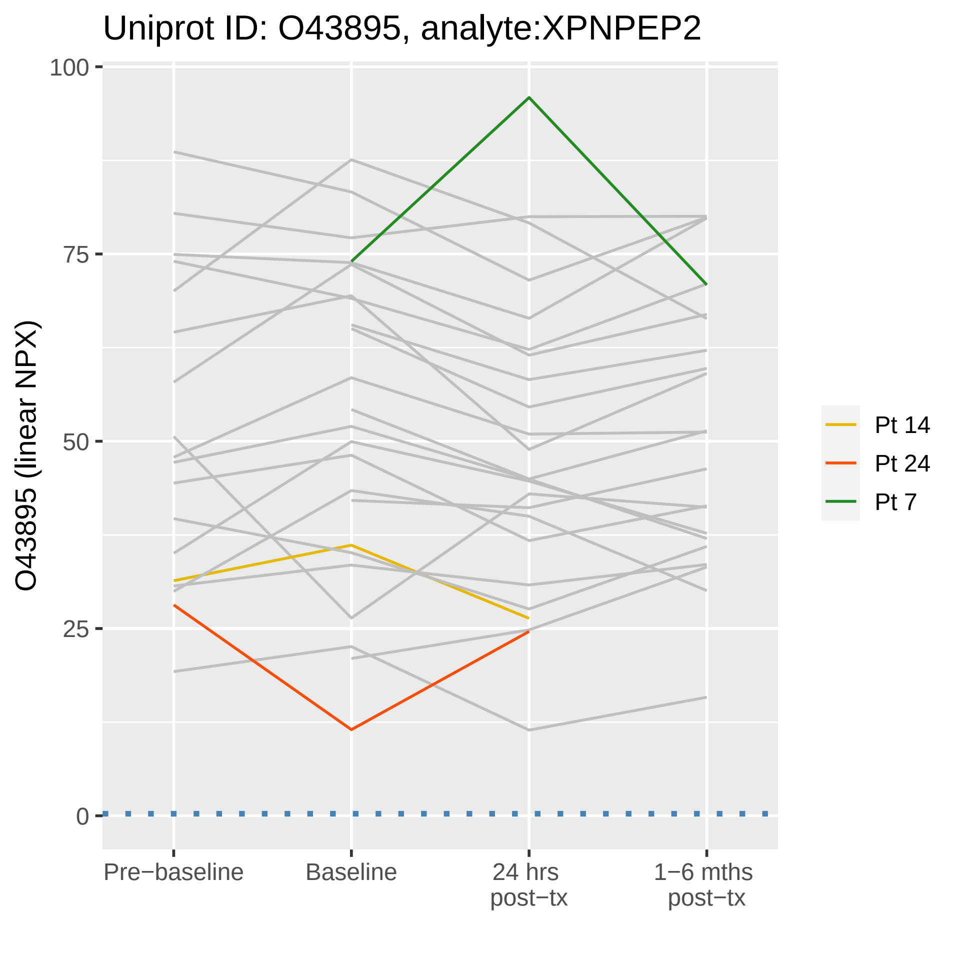

Uniprot ID: O43895, analyte:XPNPEP2
O43895 (linear NPX)
100
75
50
25
0
Pre−baseline
Baseline
24 hrs post−tx
1−6 mths post−tx
Pt 14
Pt 24
Pt 7

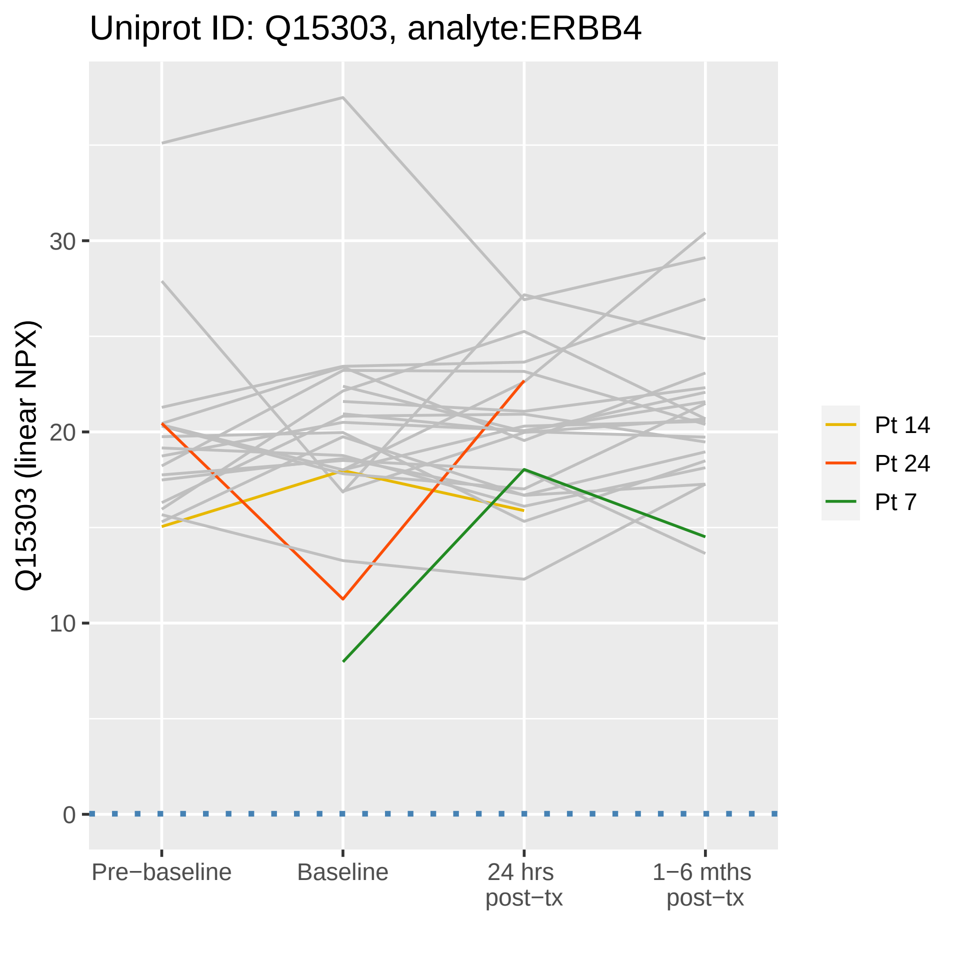

Uniprot ID: Q15303, analyte:ERBB4
Q15303 (linear NPX)
30
20
10
0
Pre−baseline
Baseline
24 hrs post−tx
1−6 mths post−tx
Pt 14
Pt 24
Pt 7

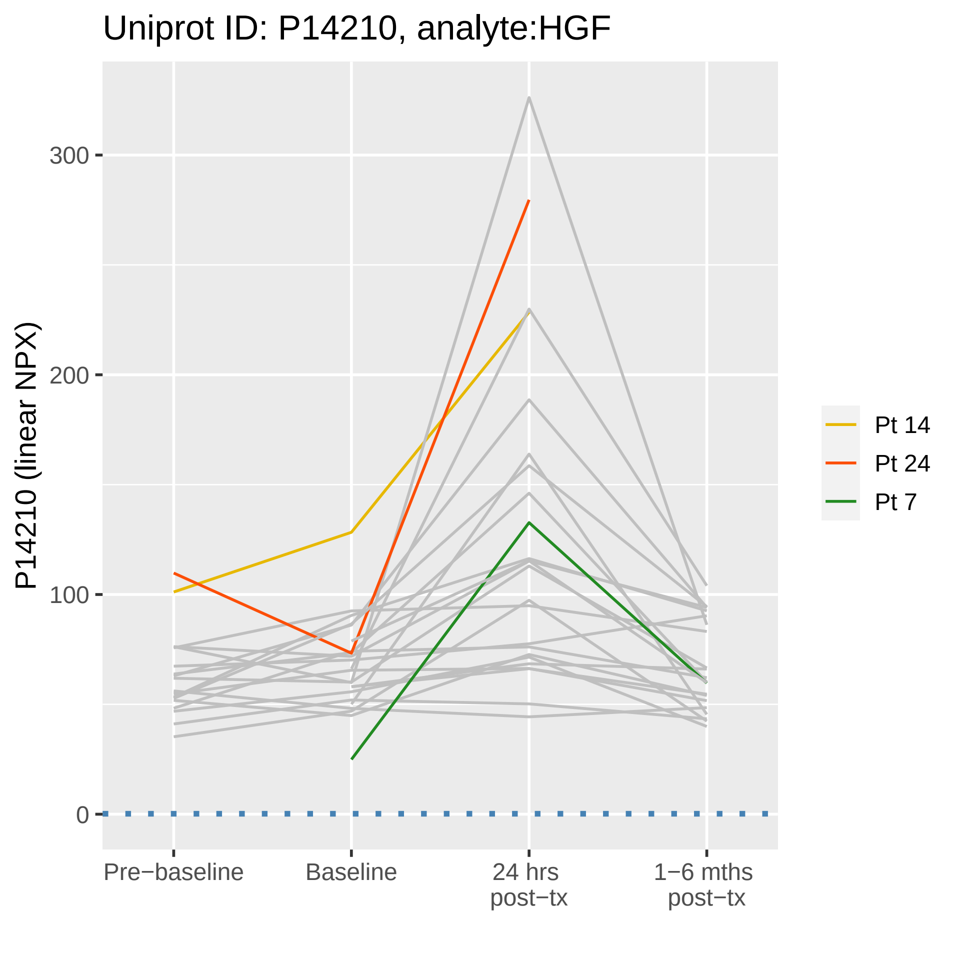

Uniprot ID: P14210, analyte:HGF
P14210 (linear NPX)
300
200
100
0
Pre−baseline
Baseline
24 hrs
post−tx
1−6 mths
post−tx
Pt 14
Pt 24
Pt 7

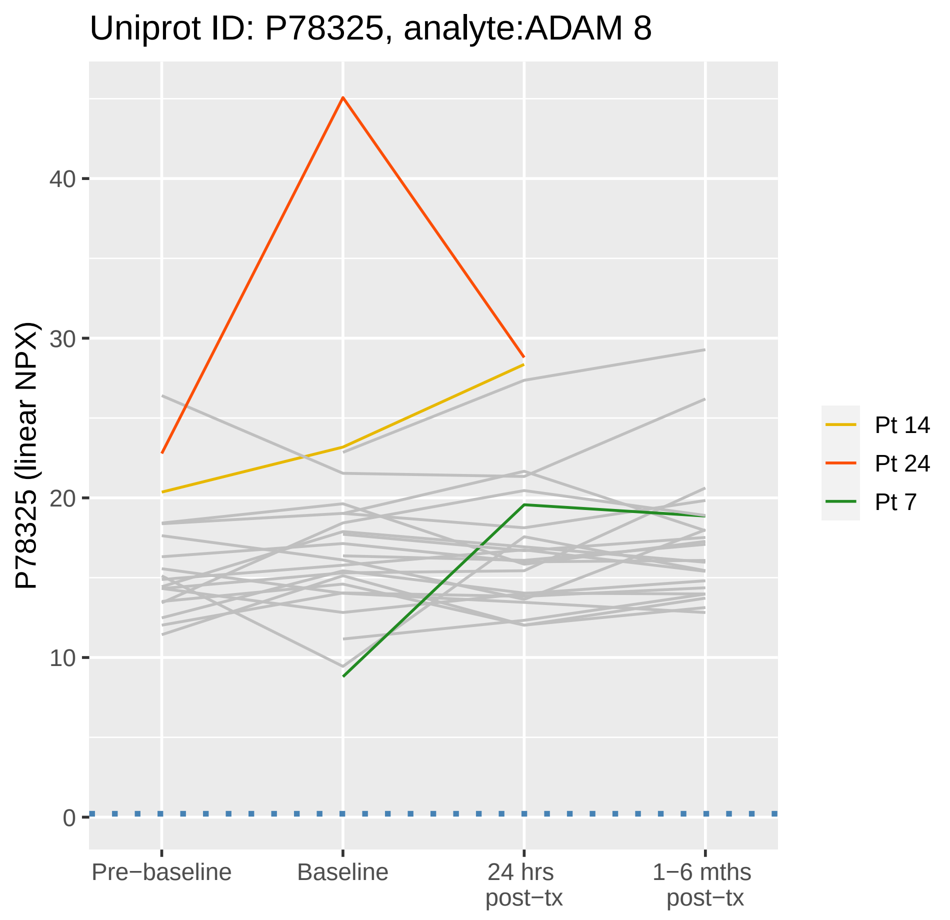
Uniprot ID: P78325, analyte:ADAM 8
P78325 (linear NPX)
40
30
20
10
0
Pre−baseline
Baseline
24 hrs post−tx
1−6 mths post−tx
Pt 14
Pt 24
Pt 7

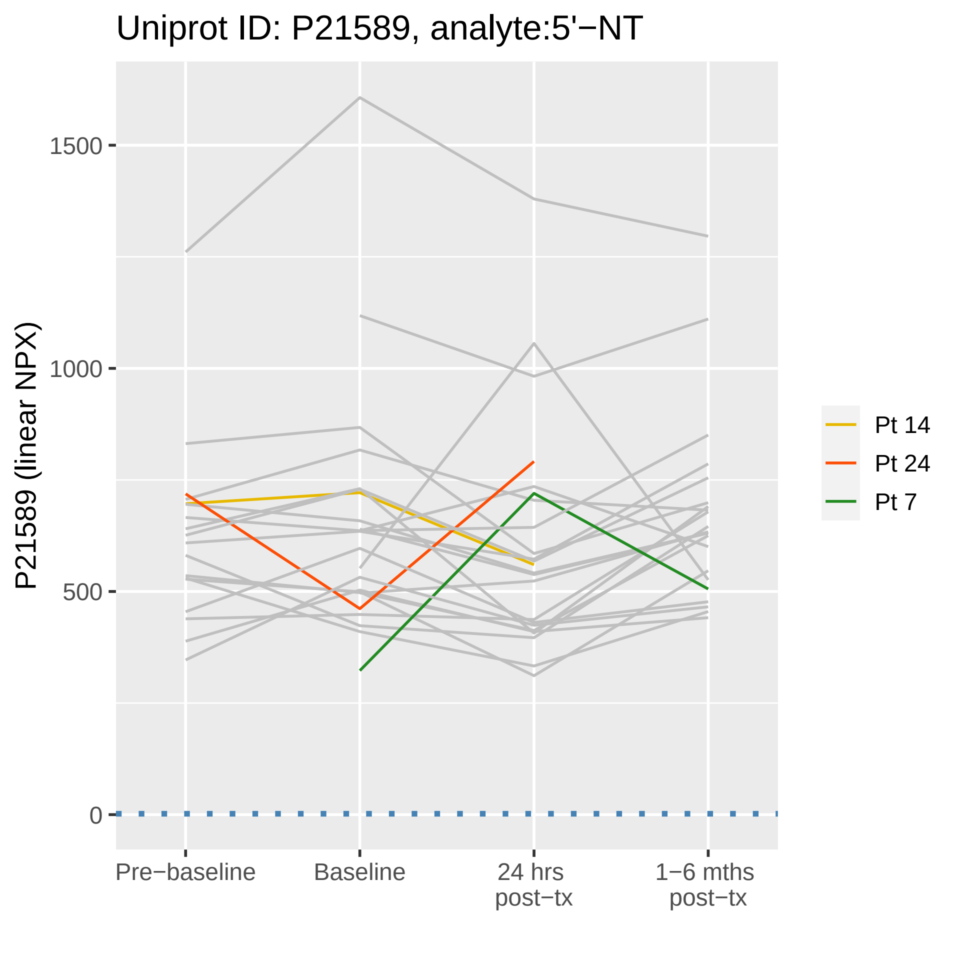

Uniprot ID: P21589, analyte:5'−NT
P21589 (linear NPX)
1500
1000
500
0
Pre−baseline
Baseline
24 hrs post−tx
1−6 mths post−tx
Pt 14
Pt 24
Pt 7

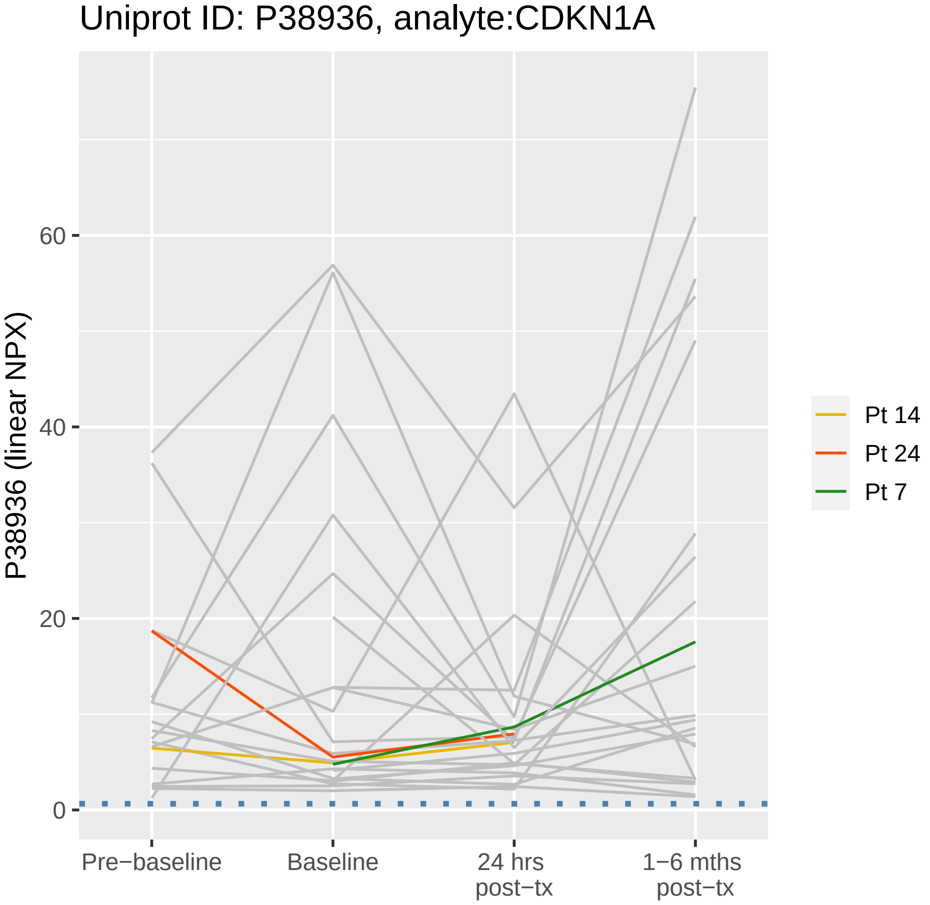

Uniprot ID: P38936, analyte:CDKN1A
P38936 (linear NPX)
60
40
20
0
Pre−baseline
Baseline
24 hrs post−tx
1−6 mths post−tx
Pt 14
Pt 24
Pt 7

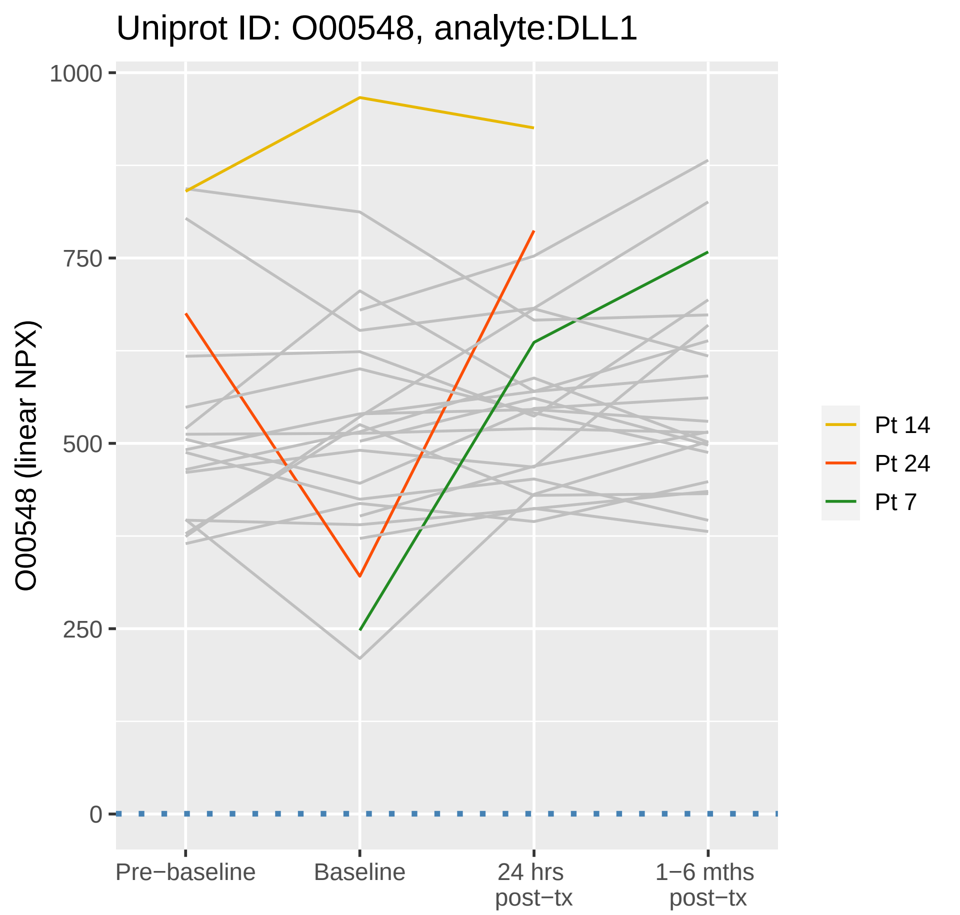
Uniprot ID: O00548, analyte:DLL1
1000
750
500
250
0
O00548 (linear NPX)
Pre−baseline
Baseline
24 hrs post−tx
1−6 mths post−tx
Pt 14
Pt 24
Pt 7

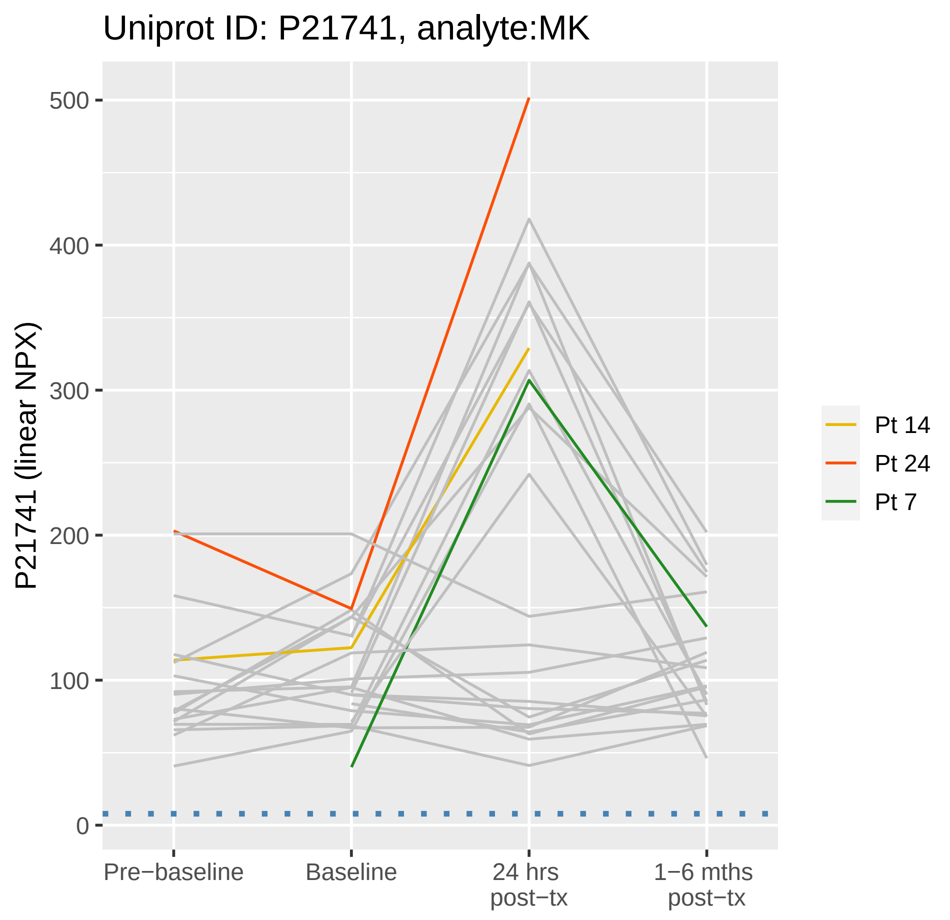
Uniprot ID: P21741, analyte:MK
P21741 (linear NPX)
500
400
300
200
100
0
Pre−baseline
Baseline
24 hrs post−tx
1−6 mths post−tx
Pt 14
Pt 24
Pt 7

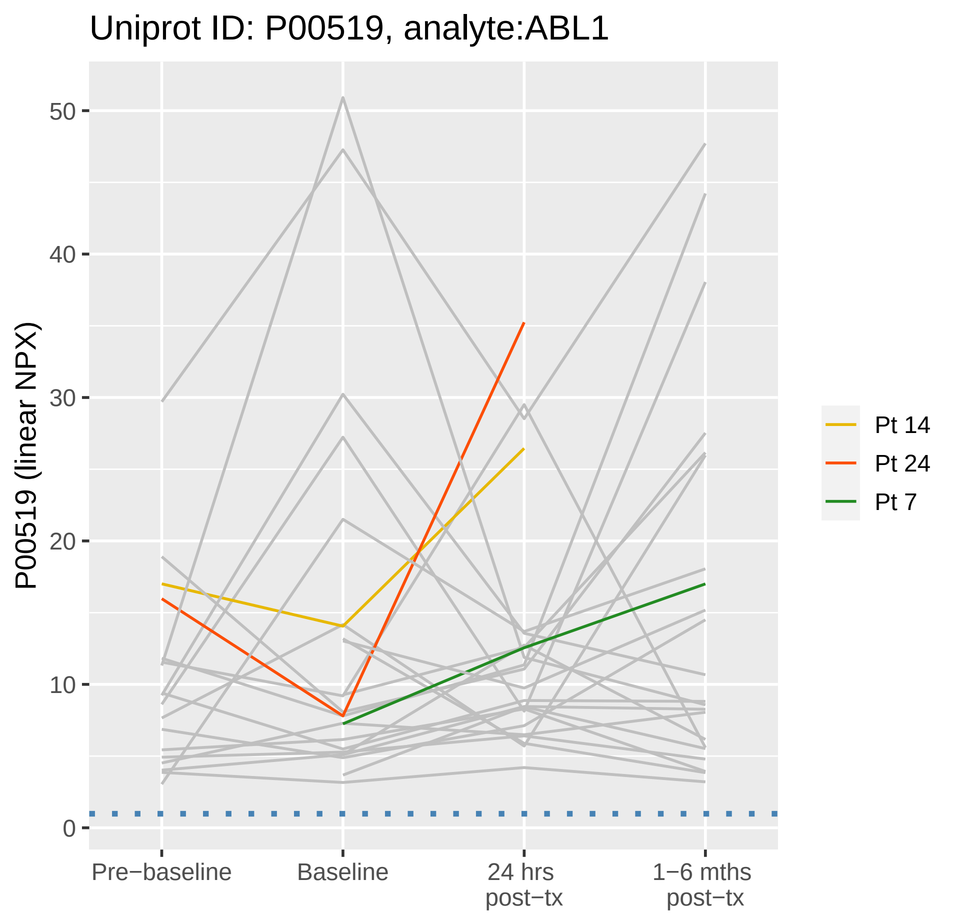
Uniprot ID: P00519, analyte:ABL1
P00519 (linear NPX)
0
10
20
30
40
50
Pre−baseline
Baseline
24 hrs post−tx
1−6 mths post−tx
Pt 14
Pt 24
Pt 7

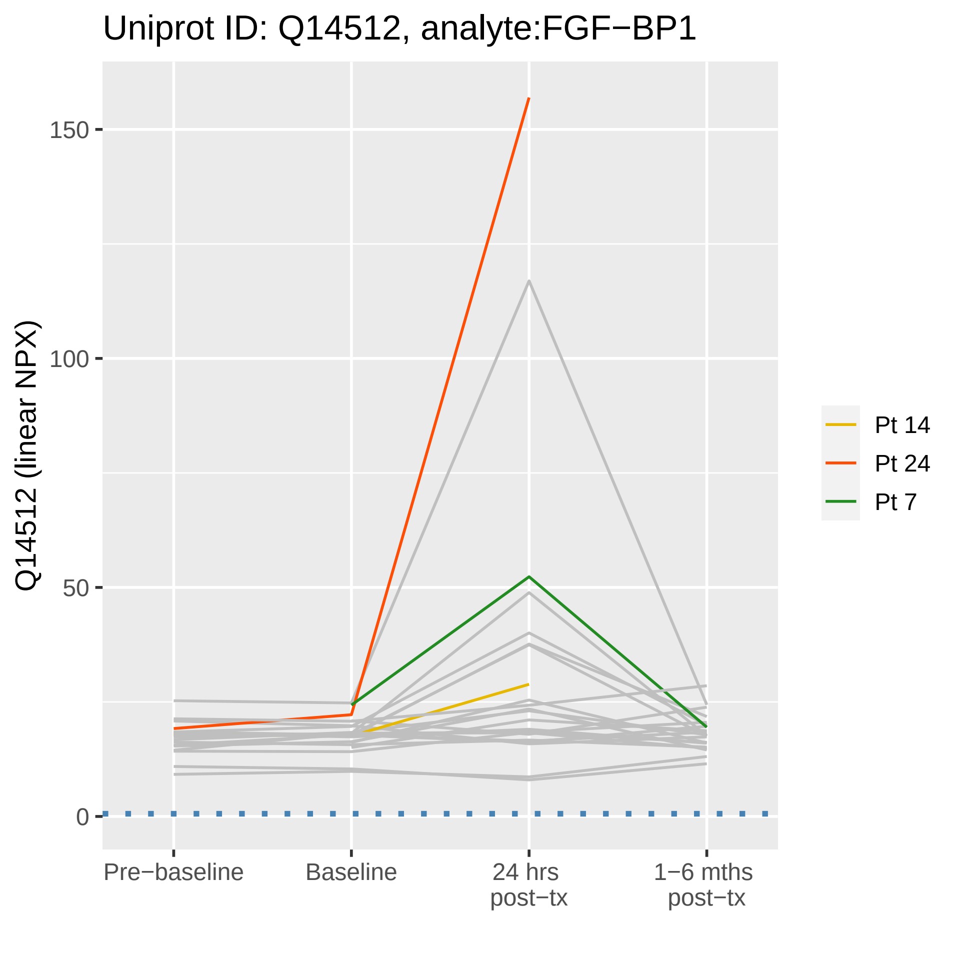
Uniprot ID: Q14512, analyte:FGF−BP1
Q14512 (linear NPX)
150
100
50
0
Pre−baseline
Baseline
24 hrs post−tx
1−6 mths post−tx
Pt 14
Pt 24
Pt 7

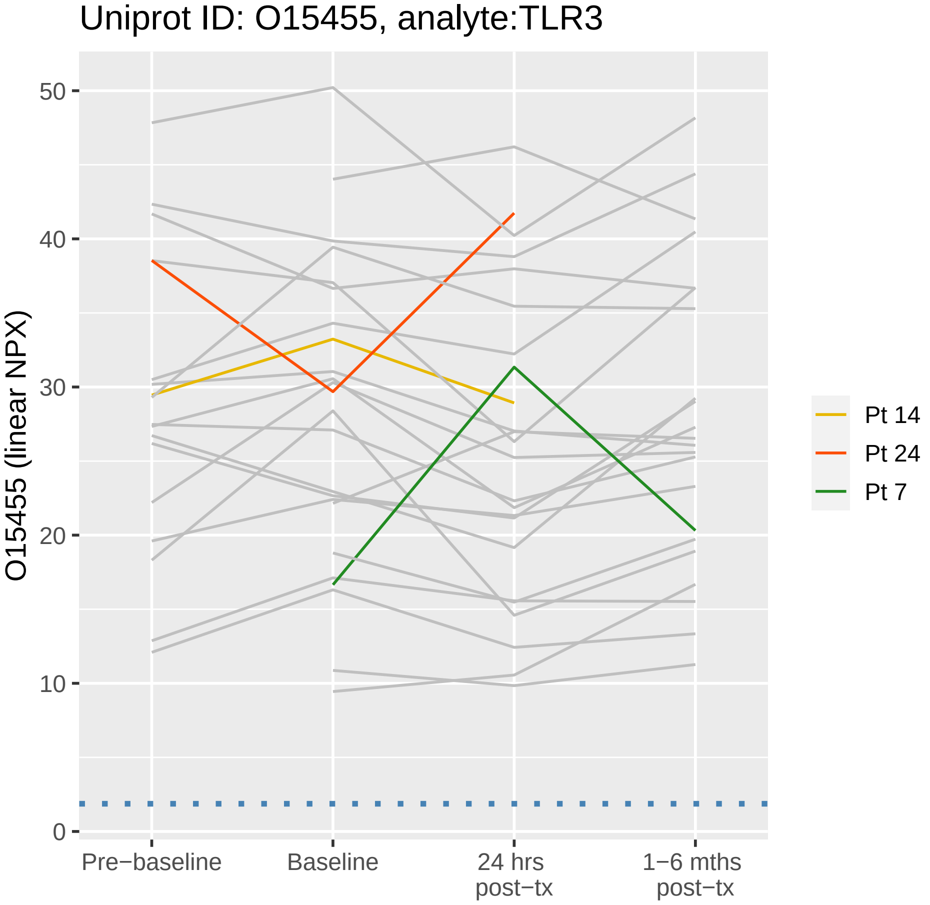

Uniprot ID: O15455, analyte:TLR3
O15455 (linear NPX)
50
40
30
20
10
0
Pre−baseline
Baseline
24 hrs post−tx
1−6 mths post−tx
Pt 14
Pt 24
Pt 7

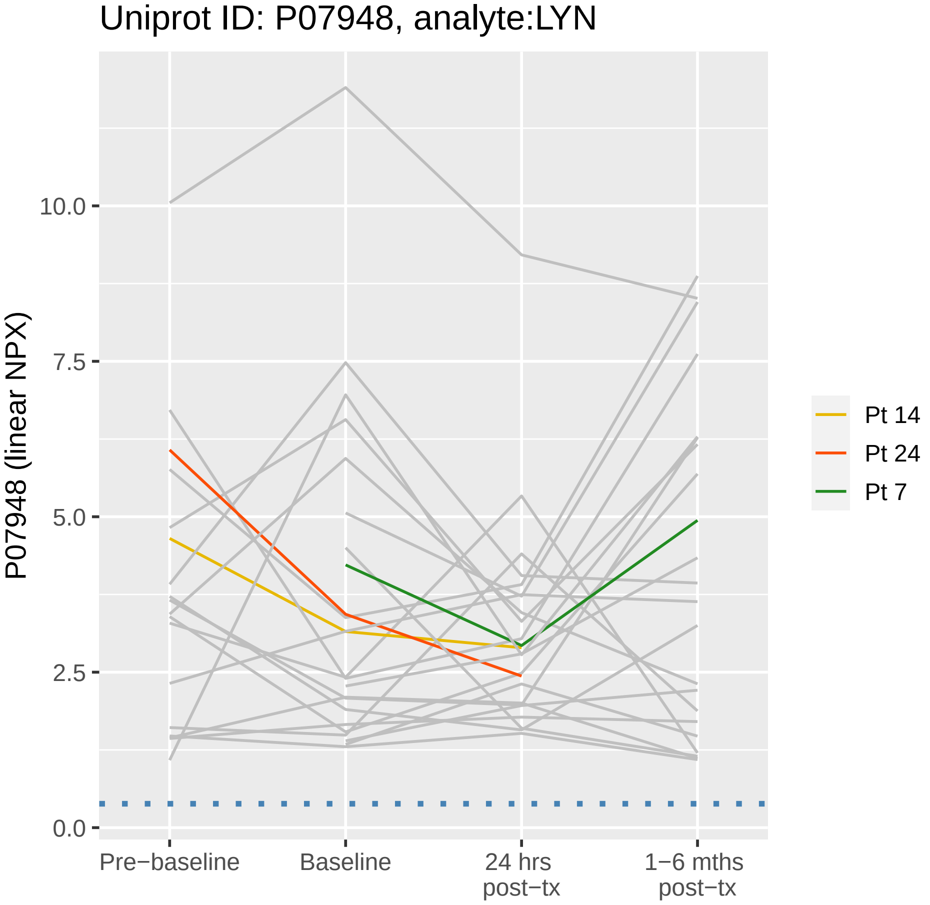
Uniprot ID: P07948, analyte:LYN
P07948 (linear NPX)
10.0
7.5
5.0
2.5
0.0
Pre−baseline
Baseline
24 hrs post−tx
1−6 mths post−tx
Pt 14
Pt 24
Pt 7

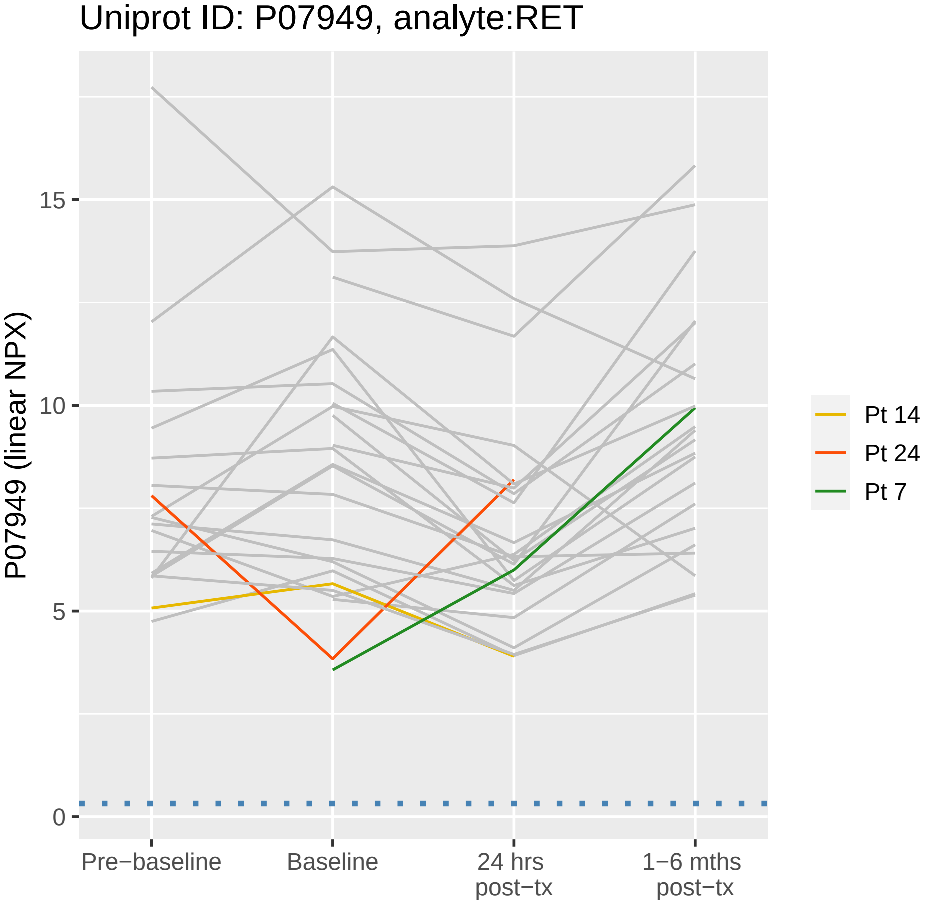

Uniprot ID: P07949, analyte:RET
P07949 (linear NPX)
15
10
5
0
Pre−baseline
Baseline
24 hrs post−tx
1−6 mths post−tx
Pt 14
Pt 24
Pt 7

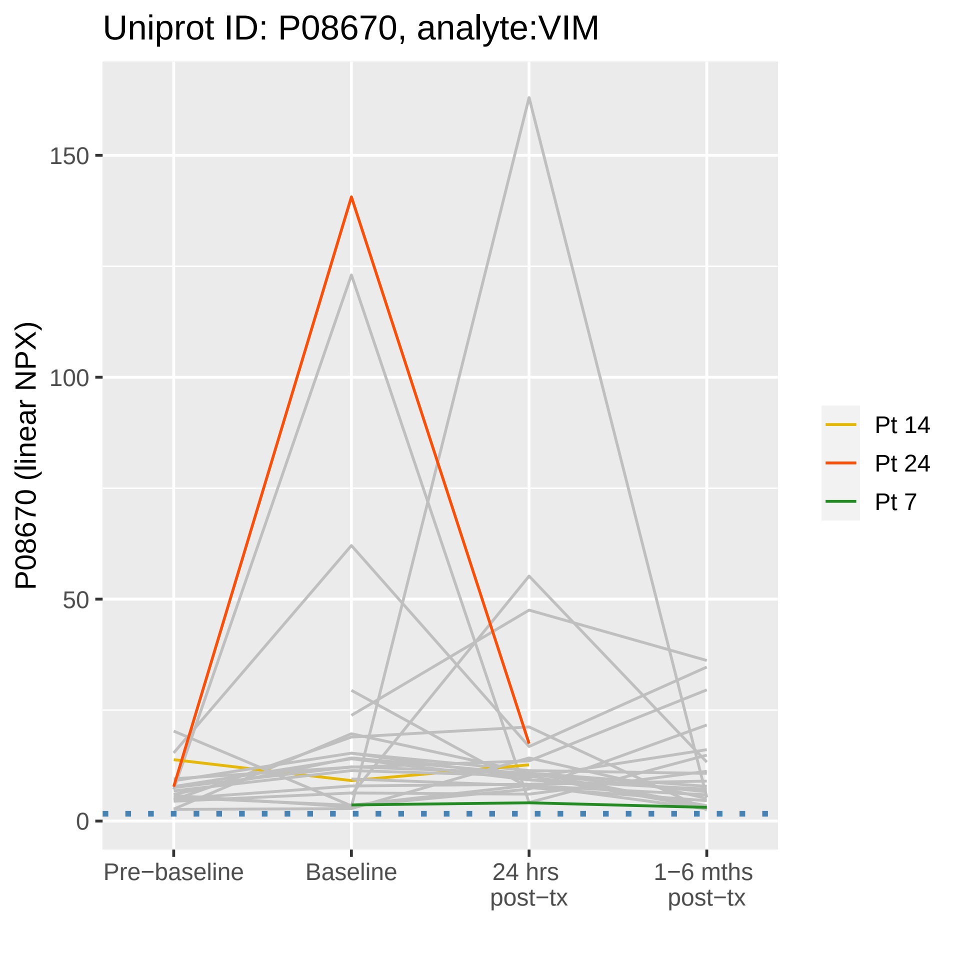

Uniprot ID: P08670, analyte:VIM
P08670 (linear NPX)
150
100
50
0
Pre−baseline
Baseline
24 hrs post−tx
1−6 mths post−tx
Pt 14
Pt 24
Pt 7

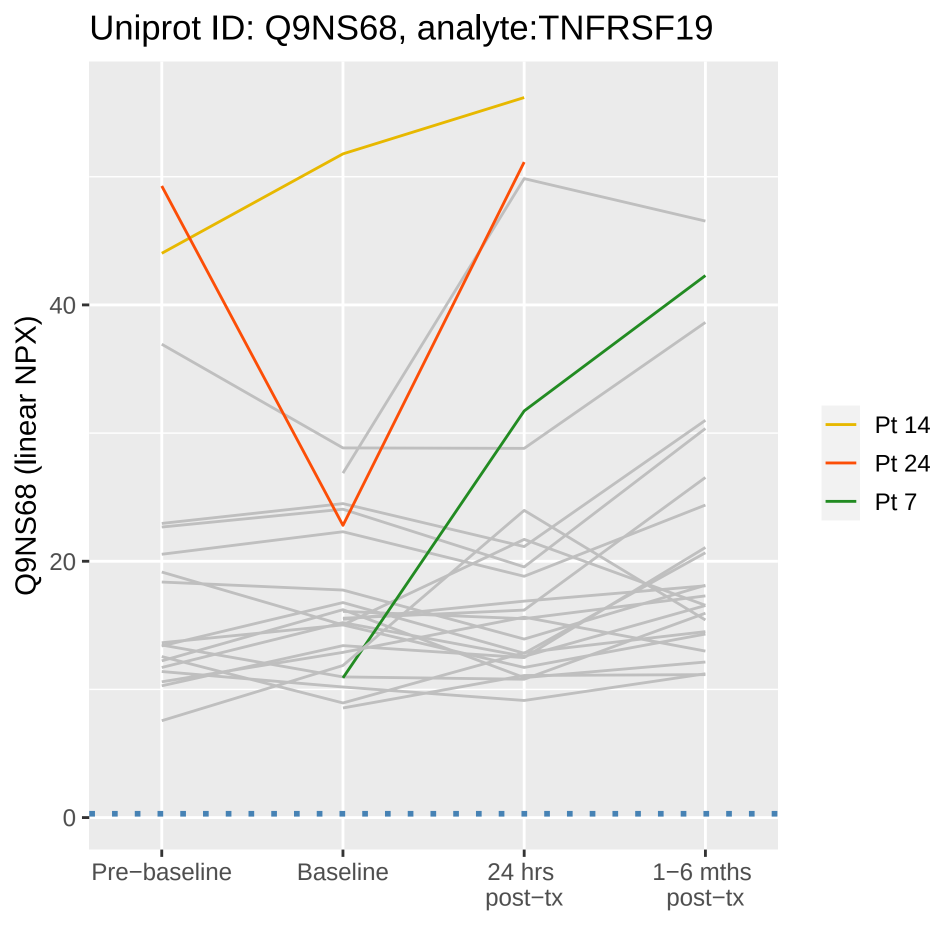

Uniprot ID: Q9NS68, analyte:TNFRSF19
Q9NS68 (linear NPX)
40
20
0
Pre−baseline
Baseline
24 hrs post−tx
1−6 mths post−tx
Pt 14
Pt 24
Pt 7

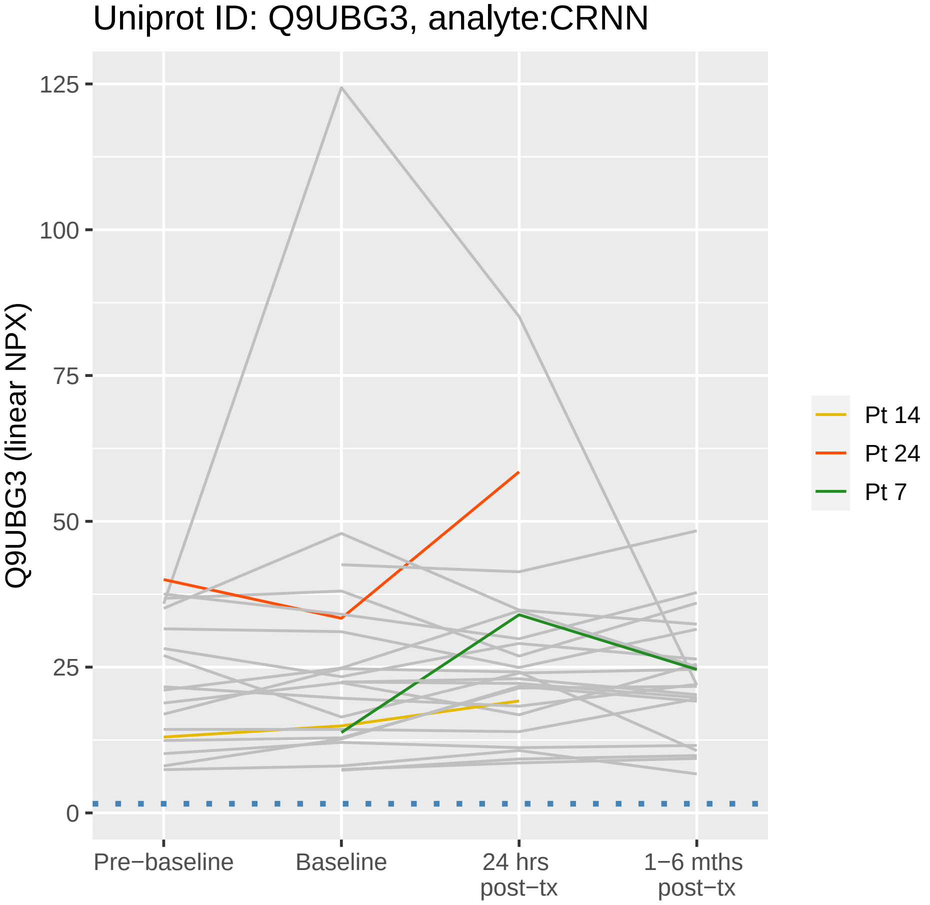

Uniprot ID: Q9UBG3, analyte:CRNN
Q9UBG3 (linear NPX)
125
100
75
50
25
0
Pre−baseline
Baseline
24 hrs post−tx
1−6 mths post−tx
Pt 14
Pt 24
Pt 7

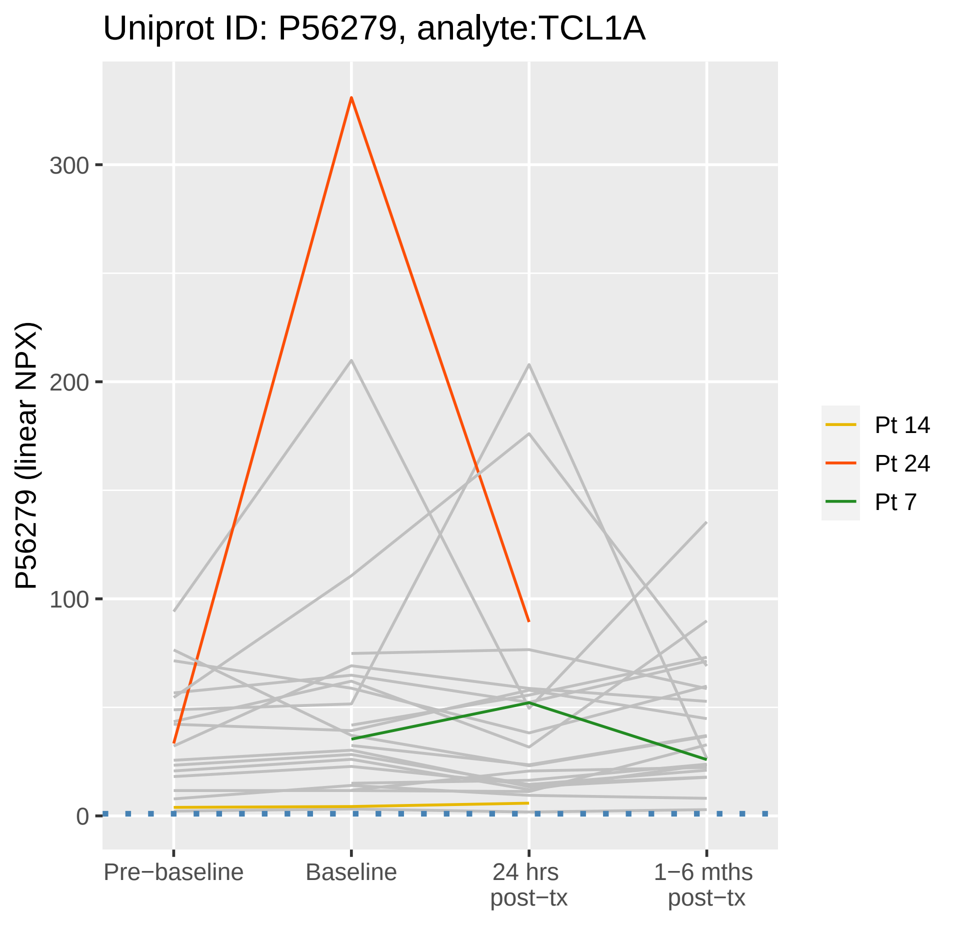

Uniprot ID: P56279, analyte:TCL1A
P56279 (linear NPX)
300
200
100
0
Pre−baseline
Baseline
24 hrs post−tx
1−6 mths post−tx
Pt 14
Pt 24
Pt 7

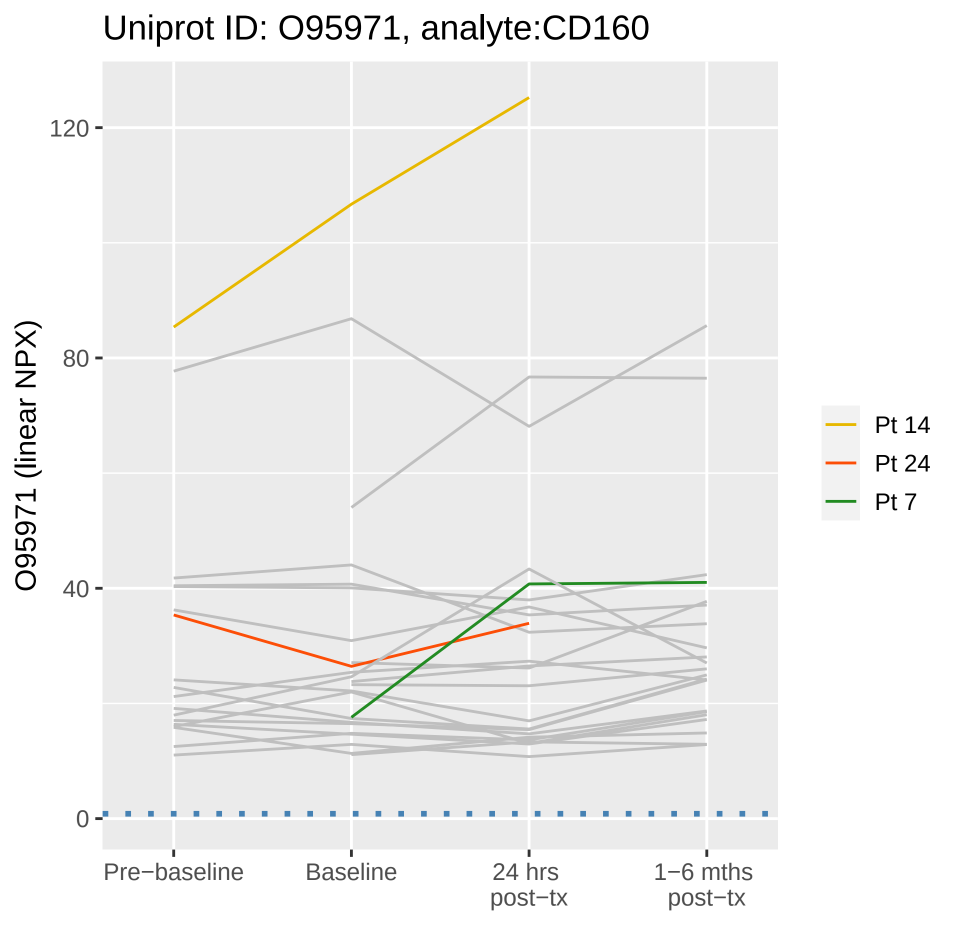

Uniprot ID: O95971, analyte:CD160
O95971 (linear NPX)
120
80
40
0
Pre−baseline
Baseline
24 hrs post−tx
1−6 mths post−tx
Pt 14
Pt 24
Pt 7

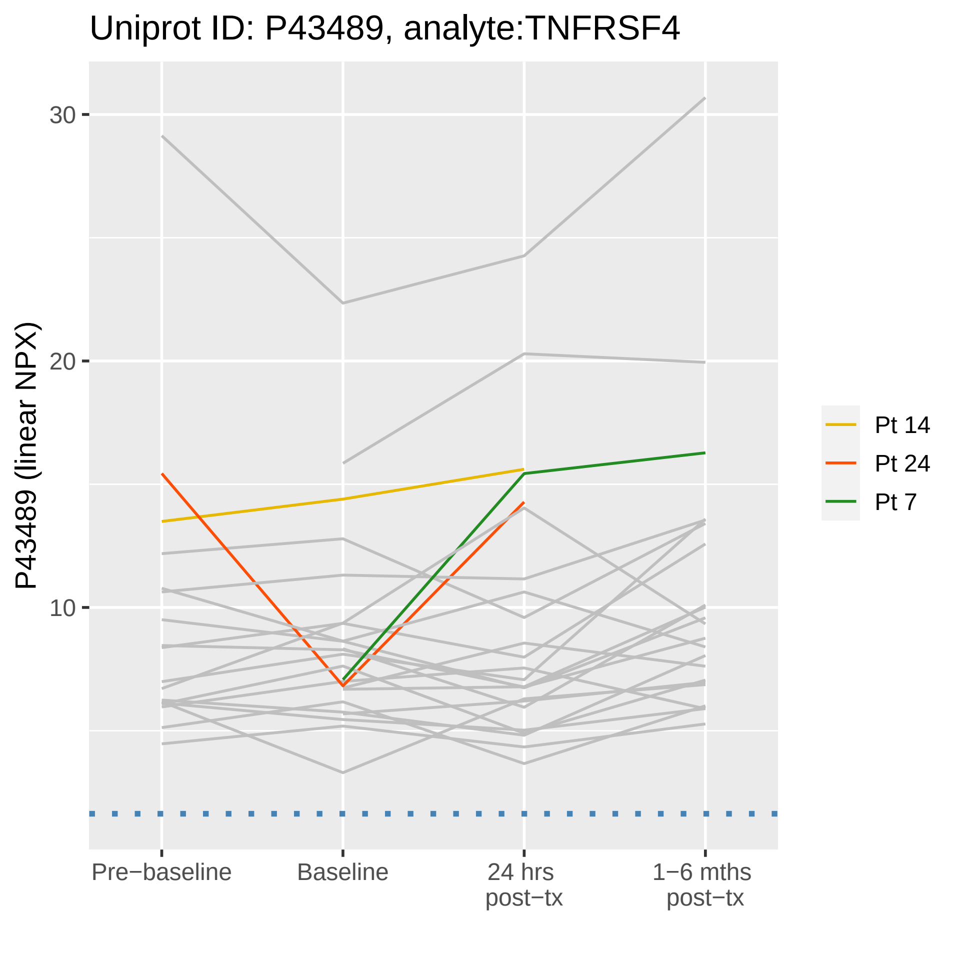

Uniprot ID: P43489, analyte:TNFRSF4
P43489 (linear NPX)
30
20
10
Pre−baseline
Baseline
24 hrs post−tx
1−6 mths post−tx
Pt 14
Pt 24
Pt 7

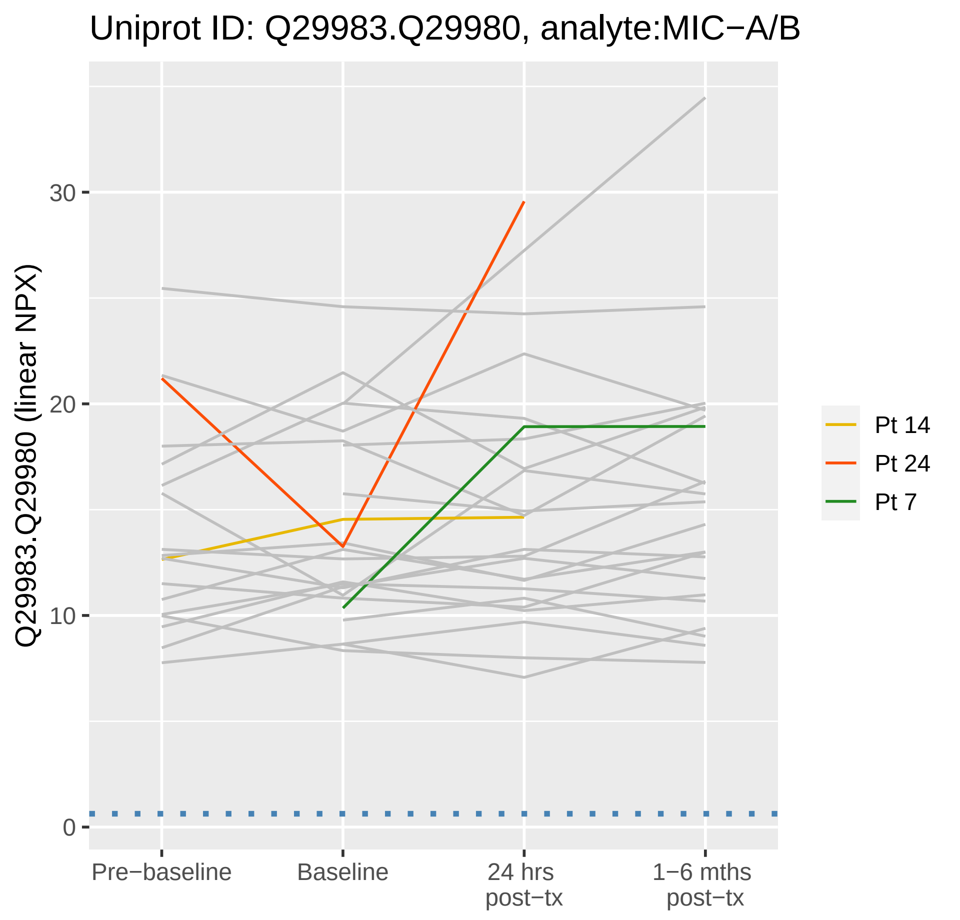

Uniprot ID: Q29983.Q29980, analyte:MIC−A/B
Q29983.Q29980 (linear NPX)
30
20
10
0
Pre−baseline
Baseline
24 hrs post−tx
1−6 mths post−tx
Pt 14
Pt 24
Pt 7

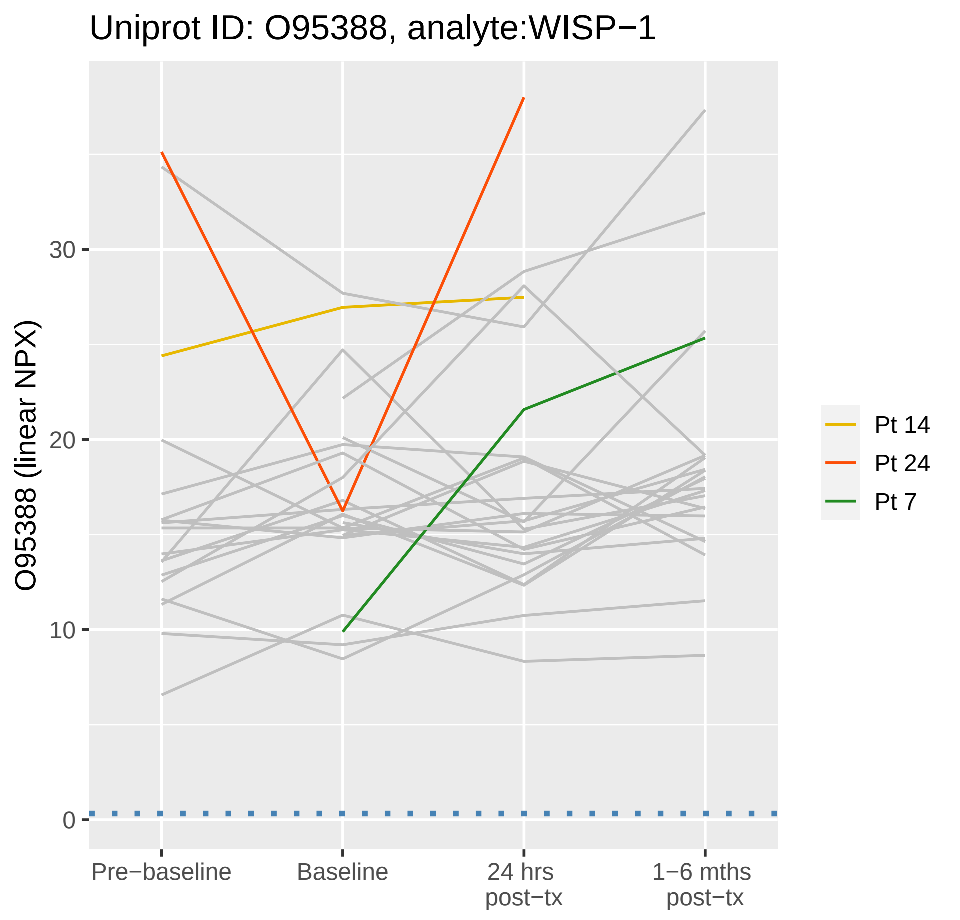

Uniprot ID: O95388, analyte:WISP−1
O95388 (linear NPX)
0
10
20
30
Pre−baseline
Baseline
24 hrs post−tx
1−6 mths post−tx
Pt 14
Pt 24
Pt 7

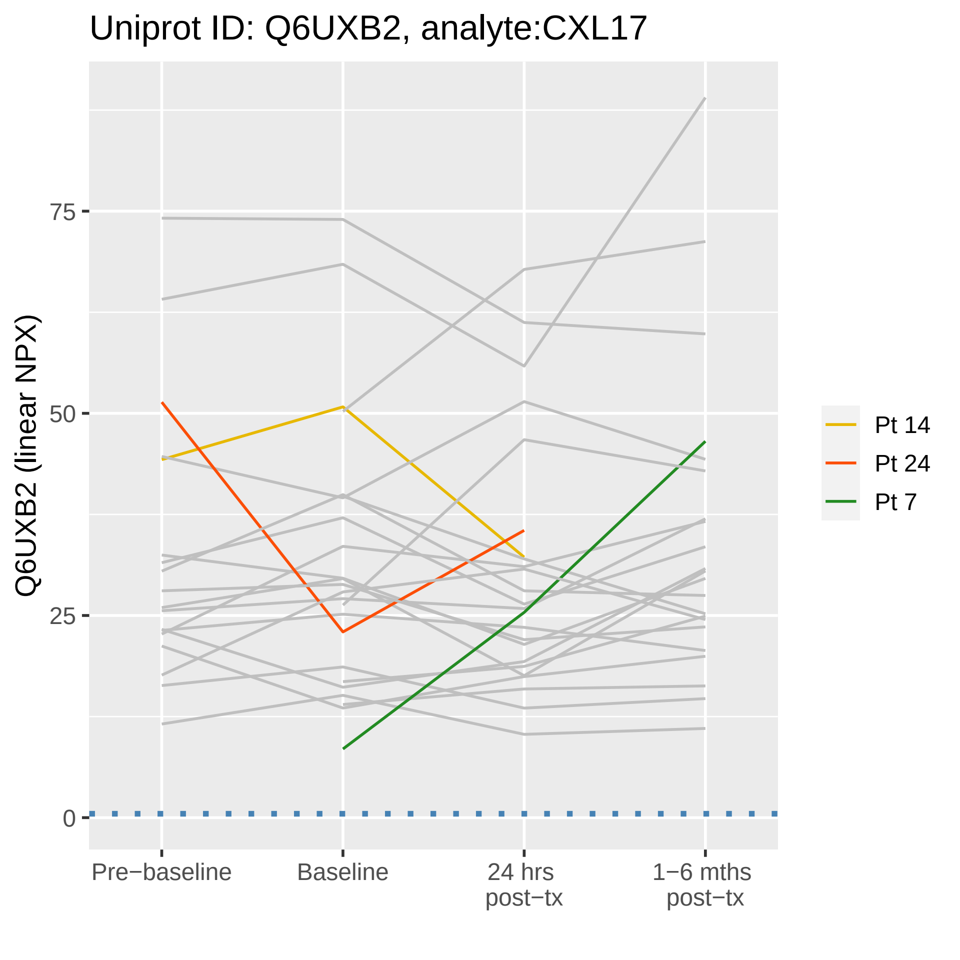
Uniprot ID: Q6UXB2, analyte:CXL17
Q6UXB2 (linear NPX)
75
50
25
0
Pre−baseline
Baseline
24 hrs post−tx
1−6 mths post−tx
Pt 14
Pt 24
Pt 7

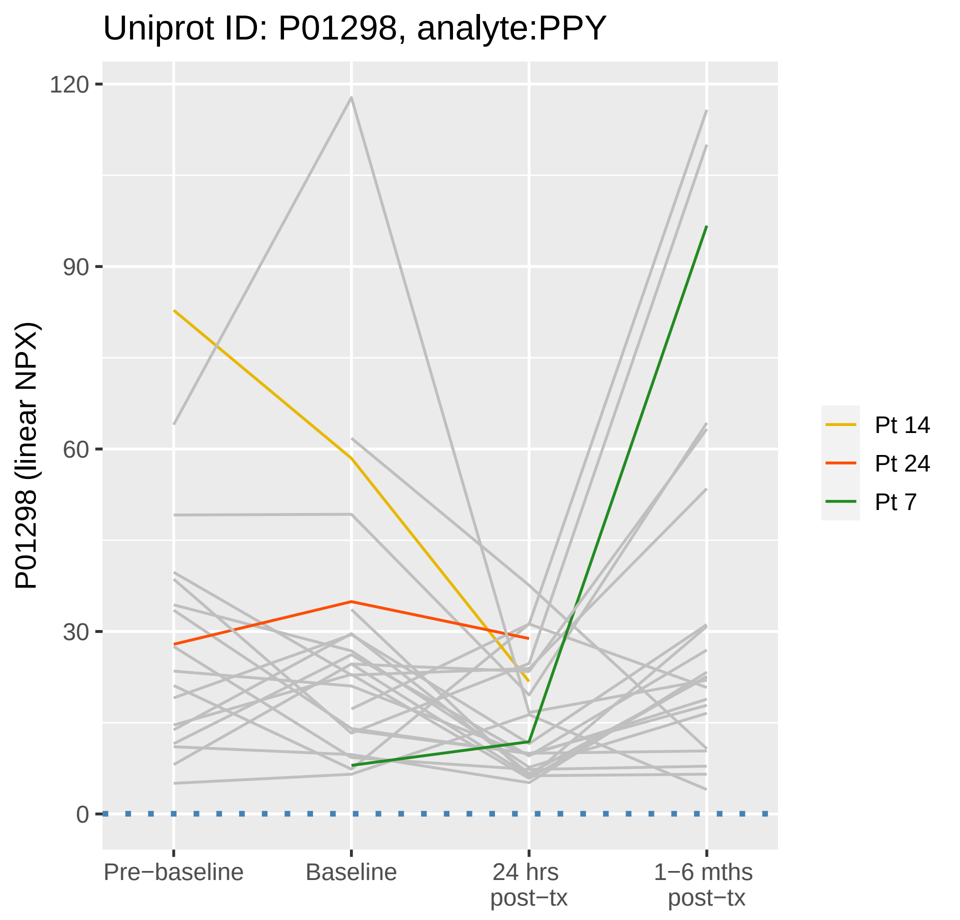

Uniprot ID: P01298, analyte:PPY
P01298 (linear NPX)
120
90
60
30
0
Pre−baseline
Baseline
24 hrs post−tx
1−6 mths post−tx
Pt 14
Pt 24
Pt 7

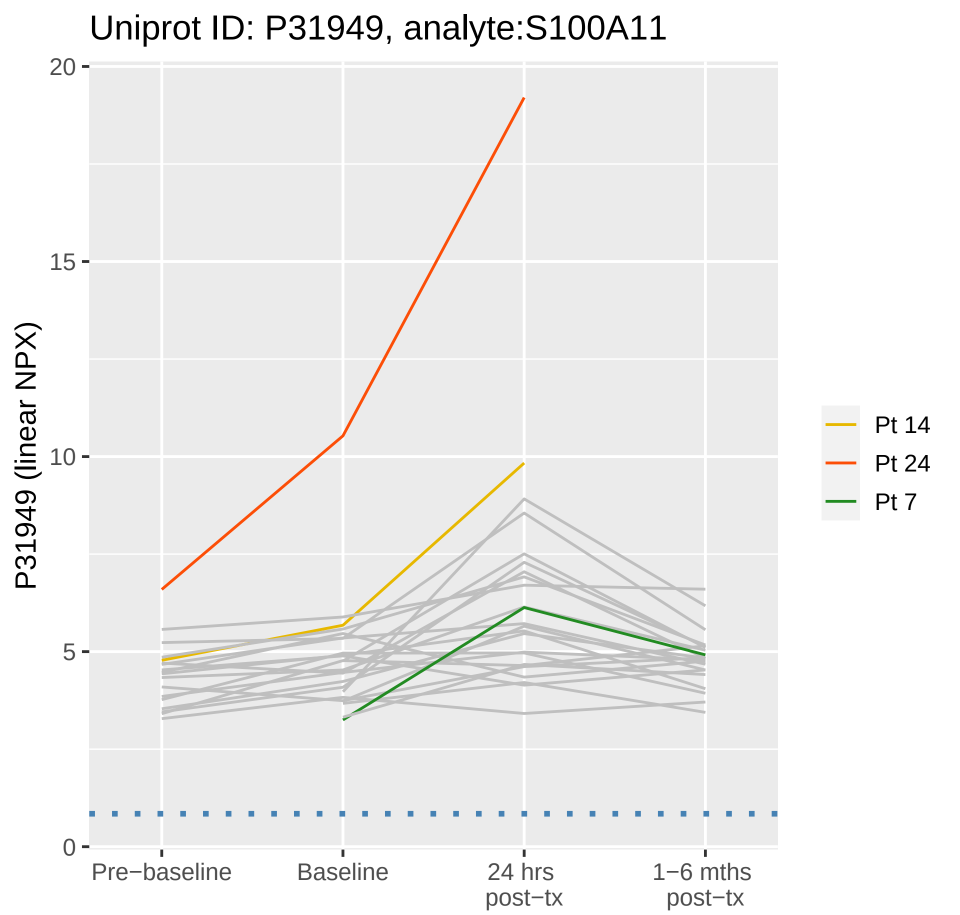
Uniprot ID: P31949, analyte:S100A11
P31949 (linear NPX)
20
15
10
5
0
Pre−baseline
Baseline
24 hrs post−tx
1−6 mths post−tx
Pt 14
Pt 24
Pt 7

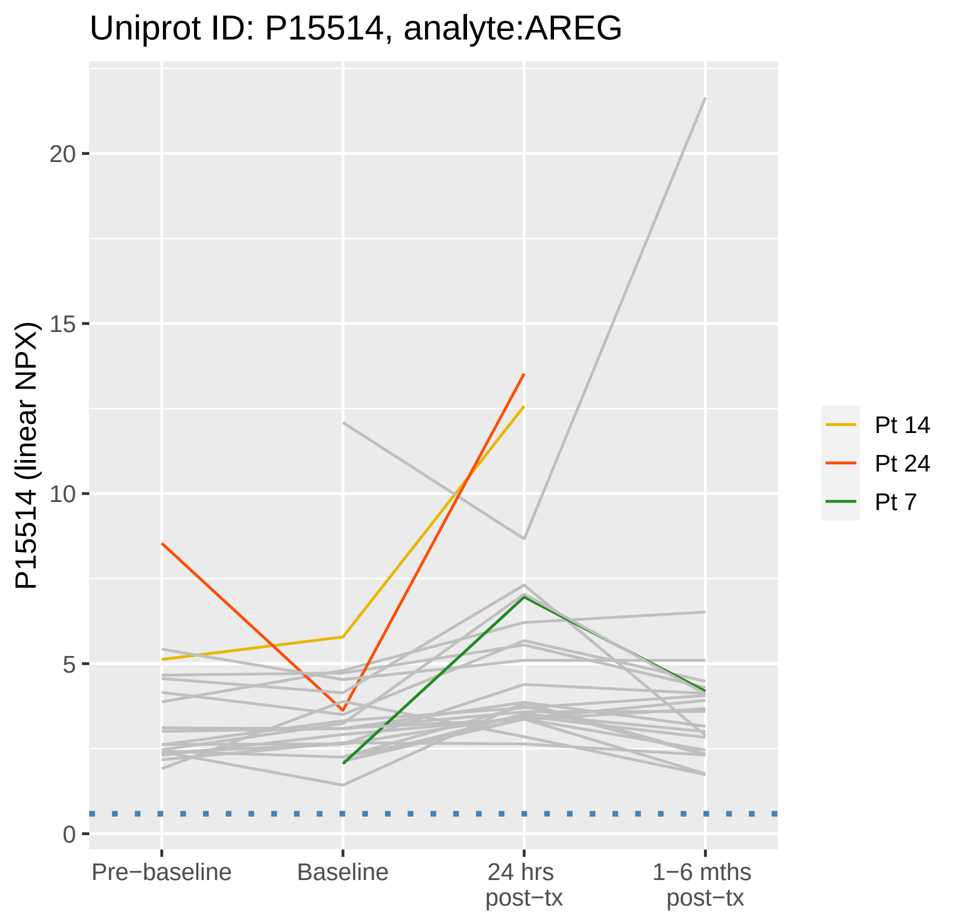
Uniprot ID: P15514, analyte:AREG
P15514 (linear NPX)
0
5
10
15
20
Pre−baseline
Baseline
24 hrs post−tx
1−6 mths post−tx
Pt 14
Pt 24
Pt 7

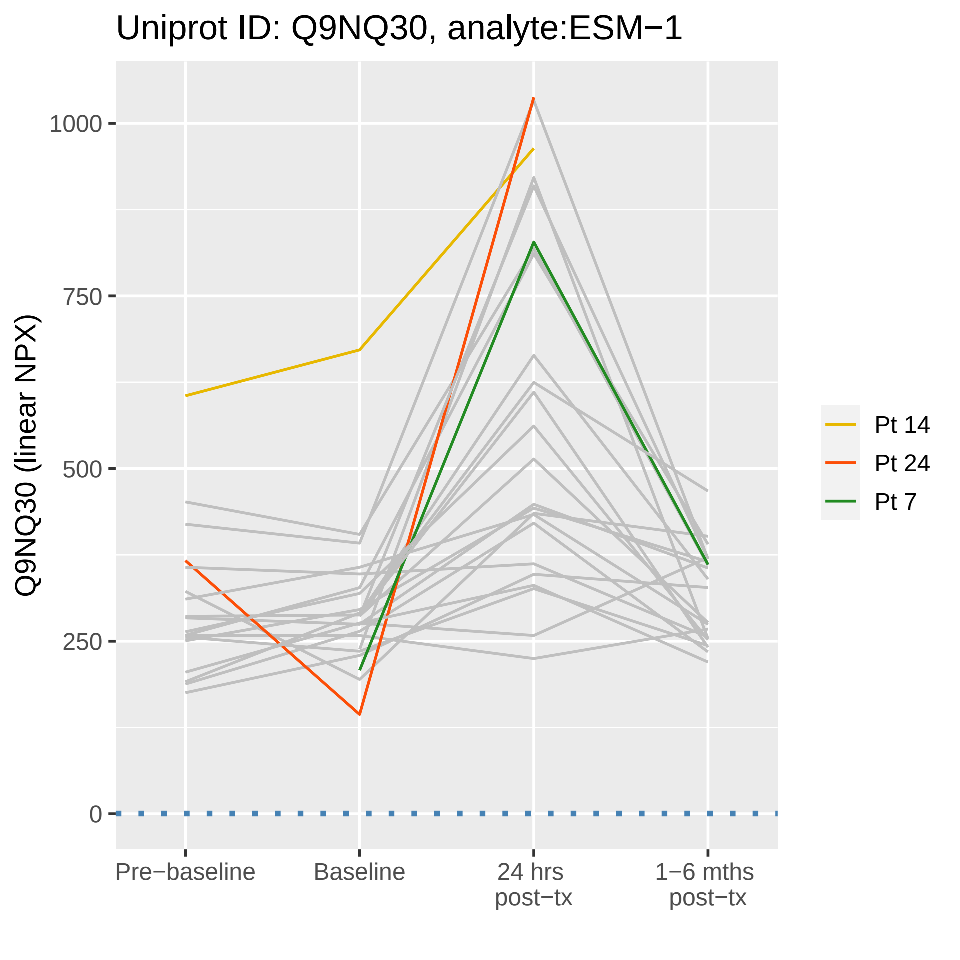
Uniprot ID: Q9NQ30, analyte:ESM−1
Q9NQ30 (linear NPX)
1000
750
500
250
0
Pre−baseline
Baseline
24 hrs post−tx
1−6 mths post−tx
Pt 14
Pt 24
Pt 7

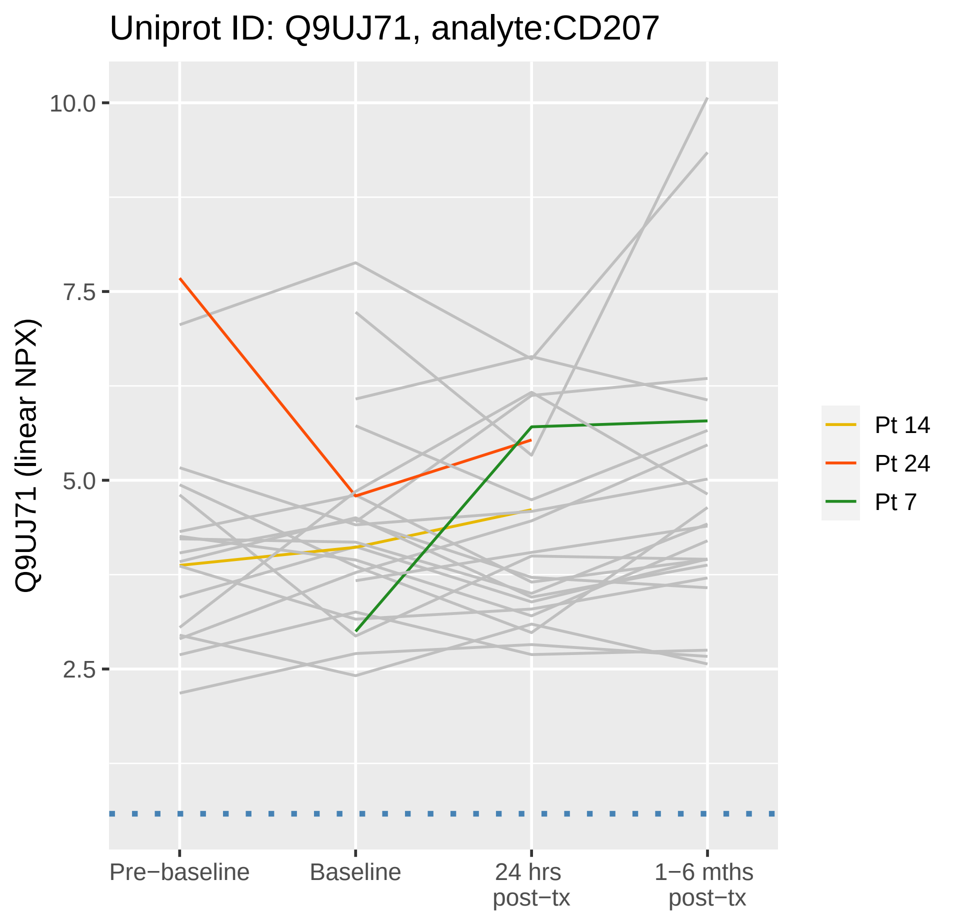
Uniprot ID: Q9UJ71, analyte:CD207
Q9UJ71 (linear NPX)
10.0
7.5
5.0
2.5
Pre−baseline
Baseline
24 hrs post−tx
1−6 mths post−tx
Pt 14
Pt 24
Pt 7

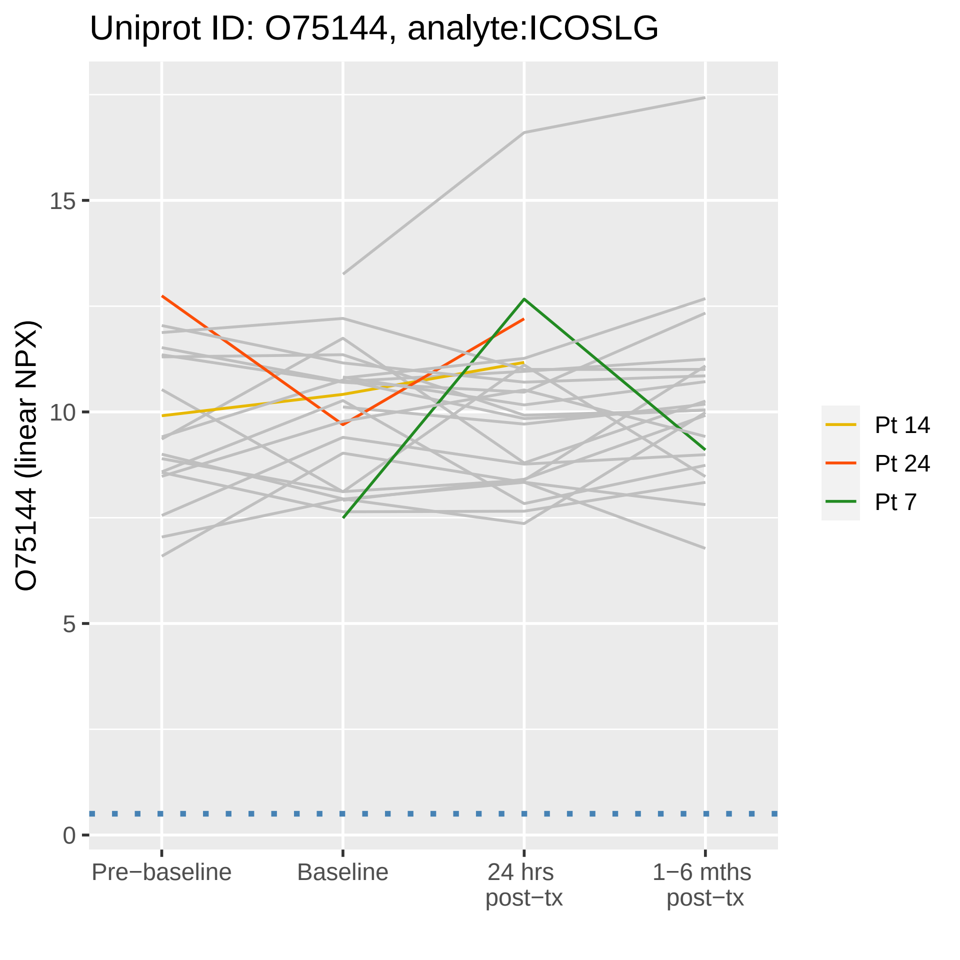

Uniprot ID: O75144, analyte:ICOSLG
O75144 (linear NPX)
15
10
5
0
Pre−baseline
Baseline
24 hrs post−tx
1−6 mths post−tx
Pt 14
Pt 24
Pt 7

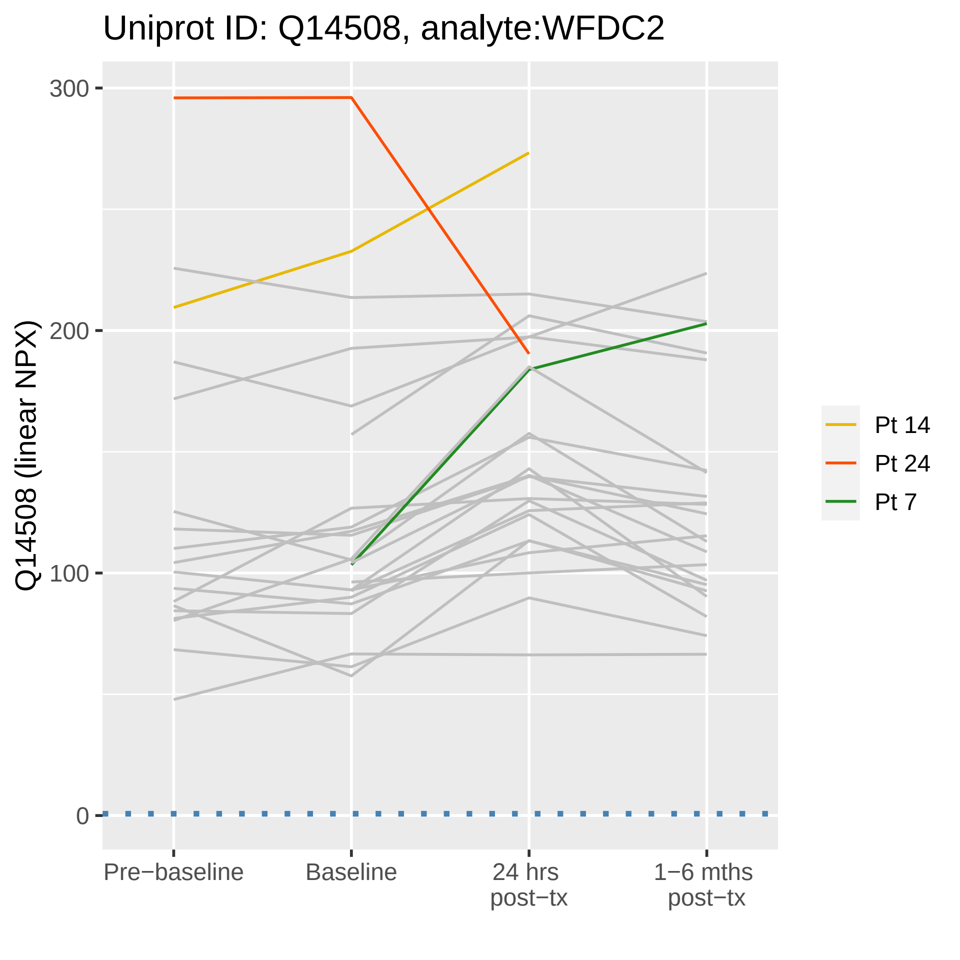

Uniprot ID: Q14508, analyte:WFDC2
Q14508 (linear NPX)
300
200
100
0
Pre−baseline
Baseline
24 hrs post−tx
1−6 mths post−tx
Pt 14
Pt 24
Pt 7

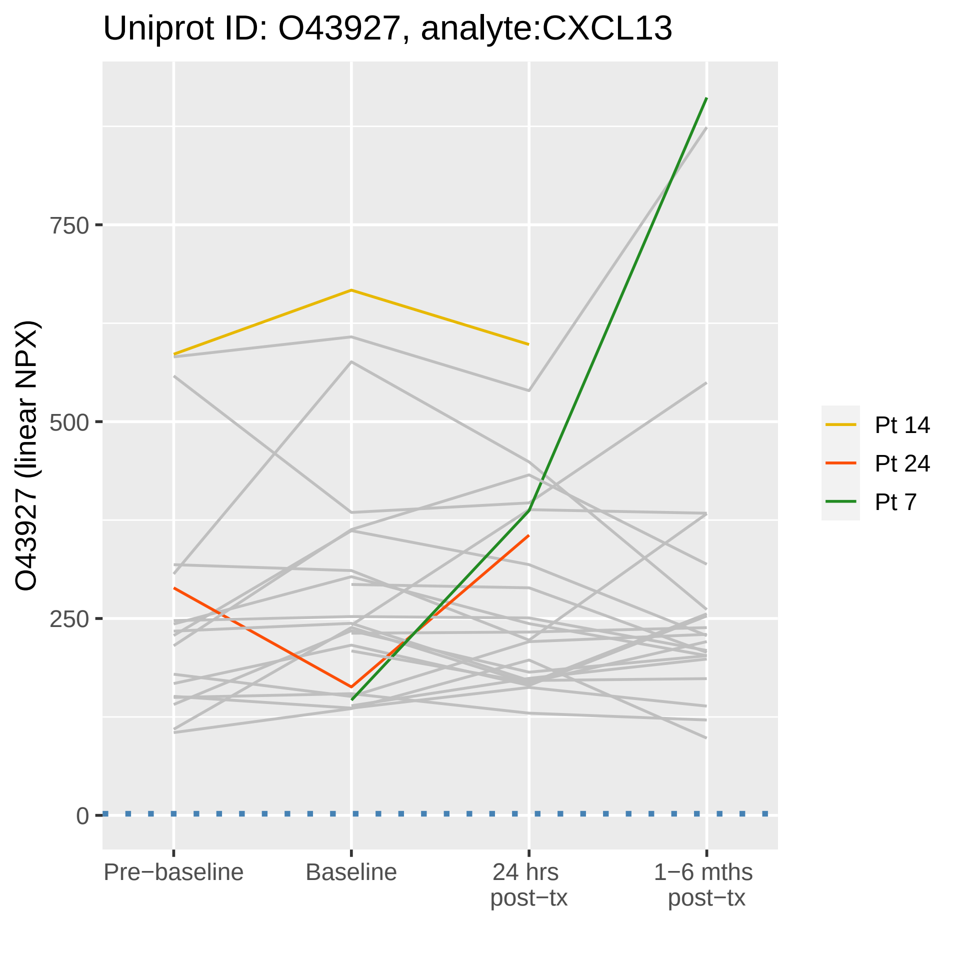

Uniprot ID: O43927, analyte:CXCL13
O43927 (linear NPX)
750
500
250
0
Pre−baseline
Baseline
24 hrs post−tx
1−6 mths post−tx
Pt 14
Pt 24
Pt 7

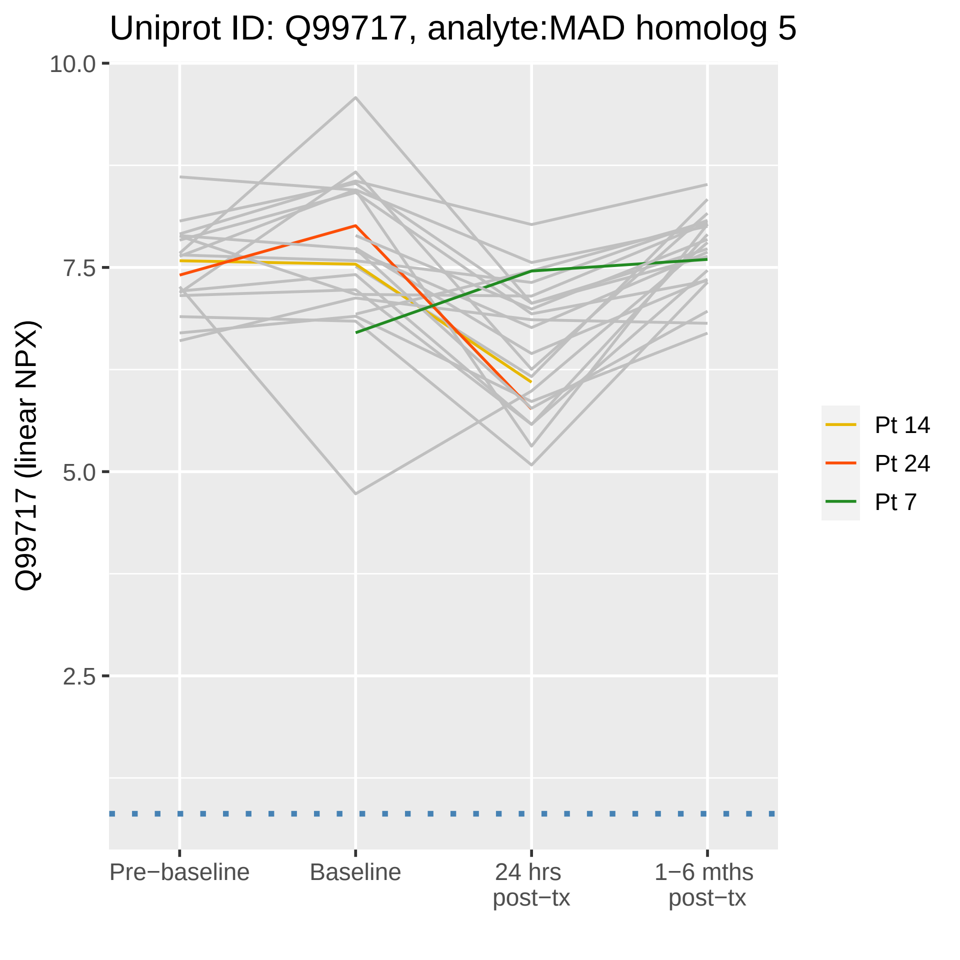

Uniprot ID: Q99717, analyte:MAD homolog 5
Q99717 (linear NPX)
10.0
7.5
5.0
2.5
Pre−baseline
Baseline
24 hrs
post−tx
1−6 mths
post−tx
Pt 14
Pt 24
Pt 7

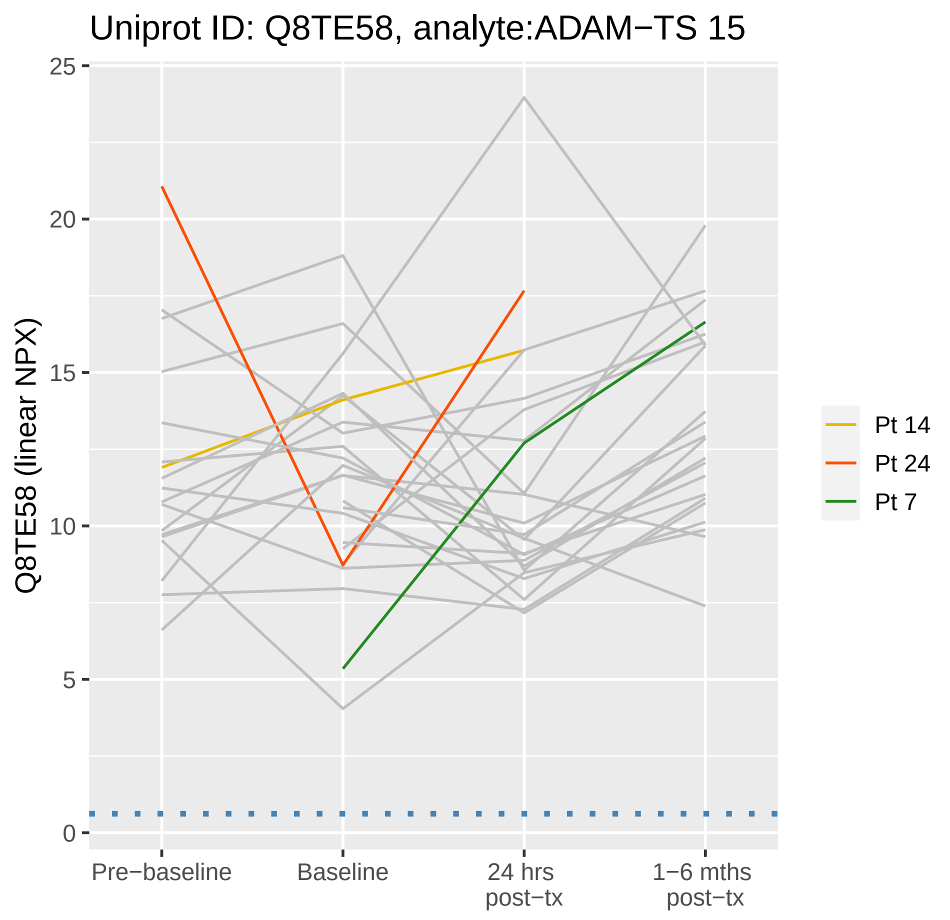

Uniprot ID: Q8TE58, analyte:ADAM−TS 15
Q8TE58 (linear NPX)
25
20
15
10
5
0
Pre−baseline
Baseline
24 hrs post−tx
1−6 mths post−tx
Pt 14
Pt 24
Pt 7

Uniprot ID: P32970, analyte:CD70

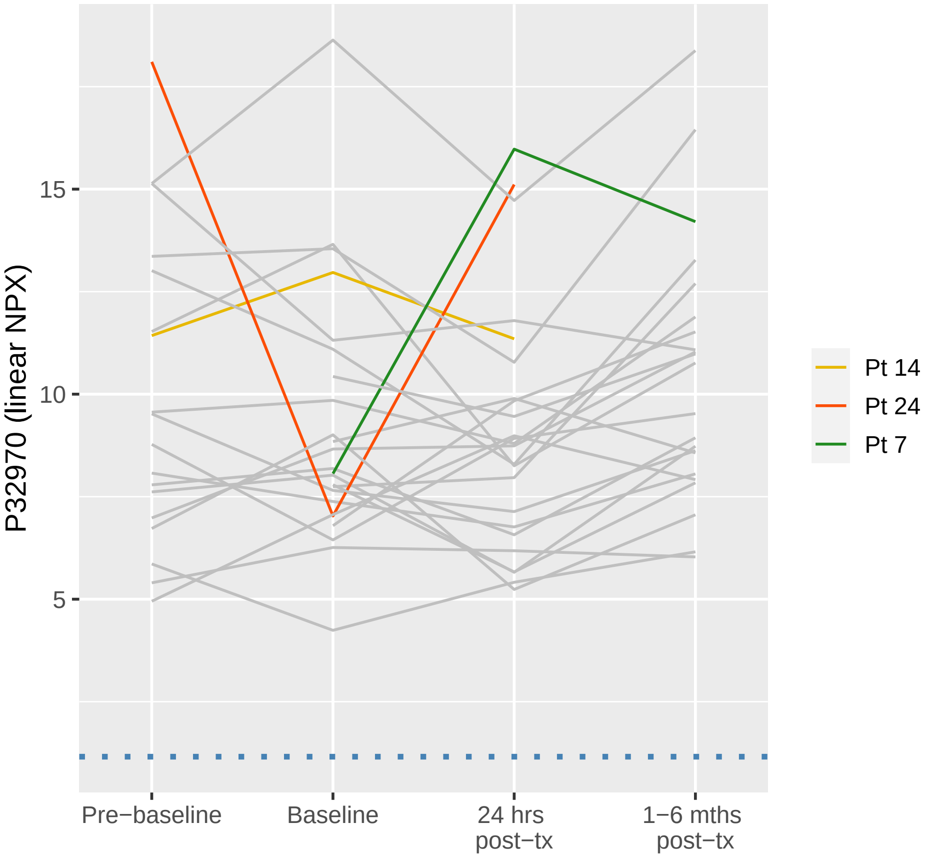

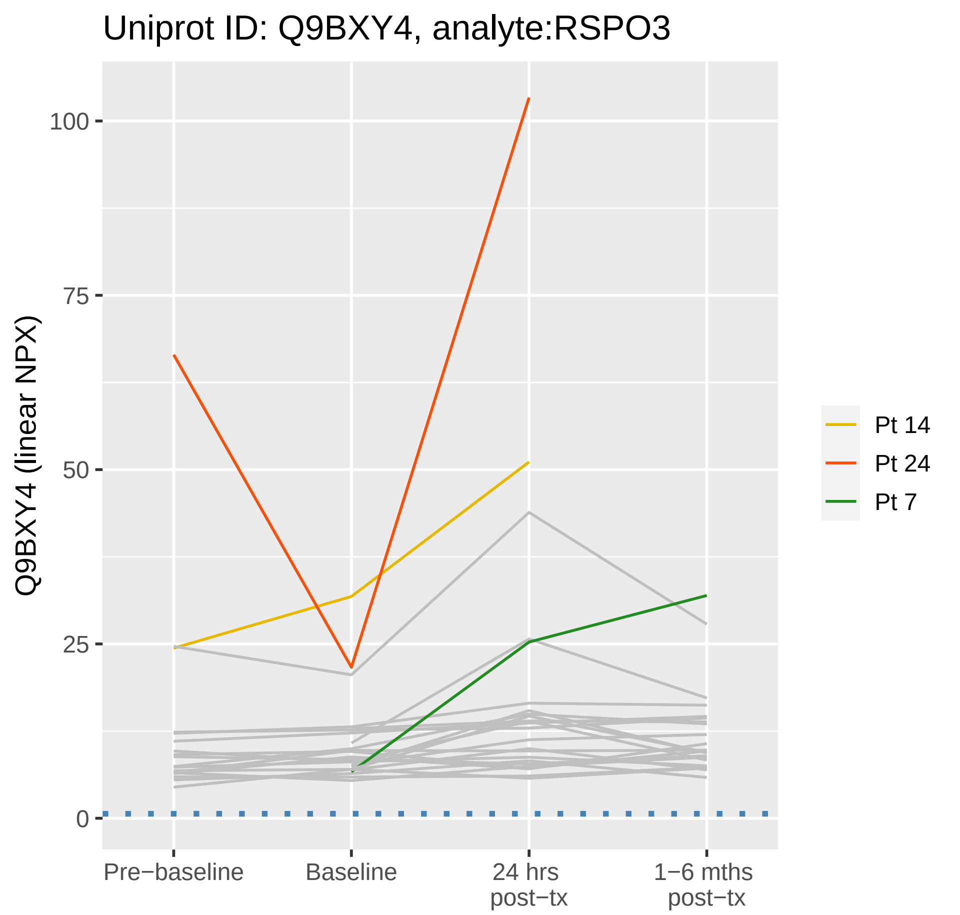
Uniprot ID: Q9BXY4, analyte:RSPO3
Q9BXY4 (linear NPX)
100
75
50
25
0
Pre−baseline
Baseline
24 hrs post−tx
1−6 mths post−tx
Pt 14
Pt 24
Pt 7

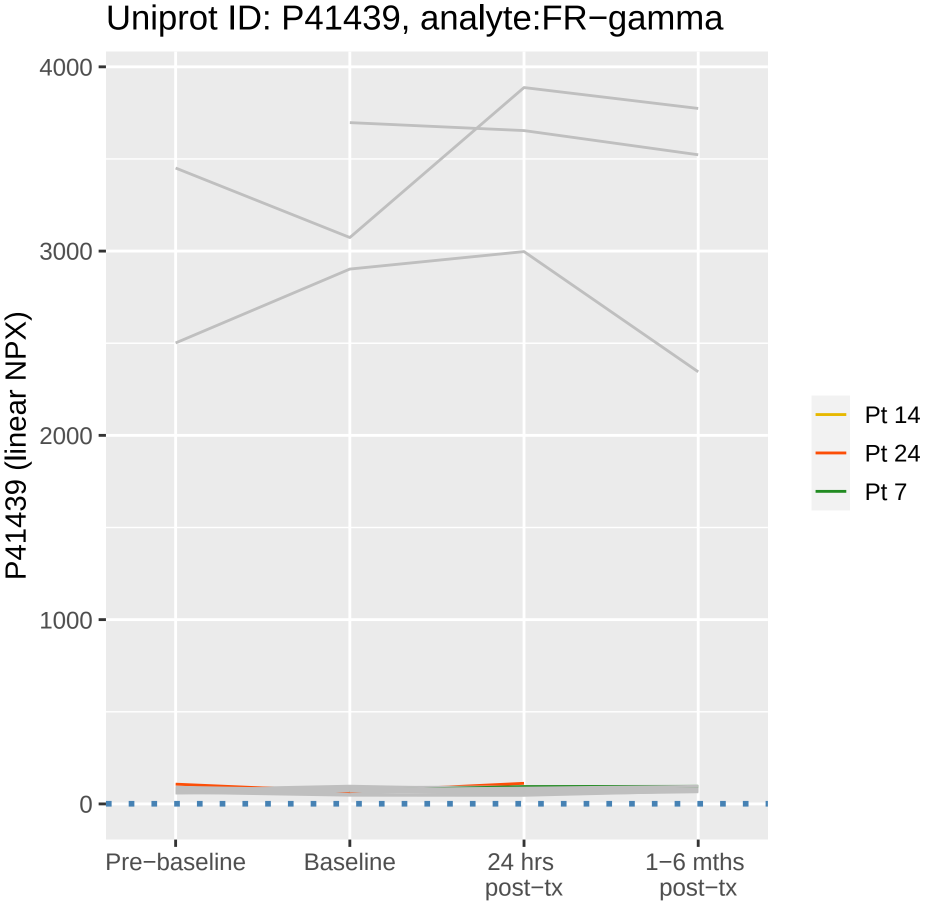
Uniprot ID: P41439, analyte:FR−gamma
P41439 (linear NPX)
4000
3000
2000
1000
0
Pre−baseline
Baseline
24 hrs post−tx
1−6 mths post−tx
Pt 14
Pt 24
Pt 7

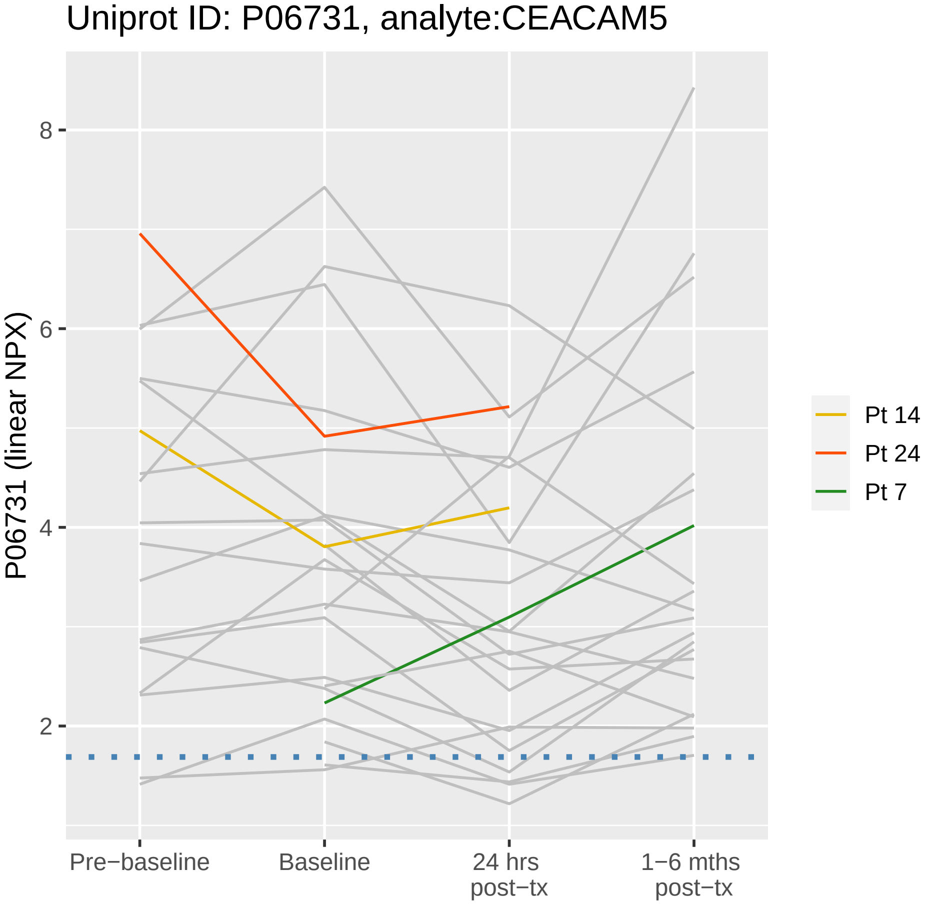

Uniprot ID: P06731, analyte:CEACAM5
P06731 (linear NPX)
8
6
4
2
Pre−baseline
Baseline
24 hrs post−tx
1−6 mths post−tx
Pt 14
Pt 24
Pt 7

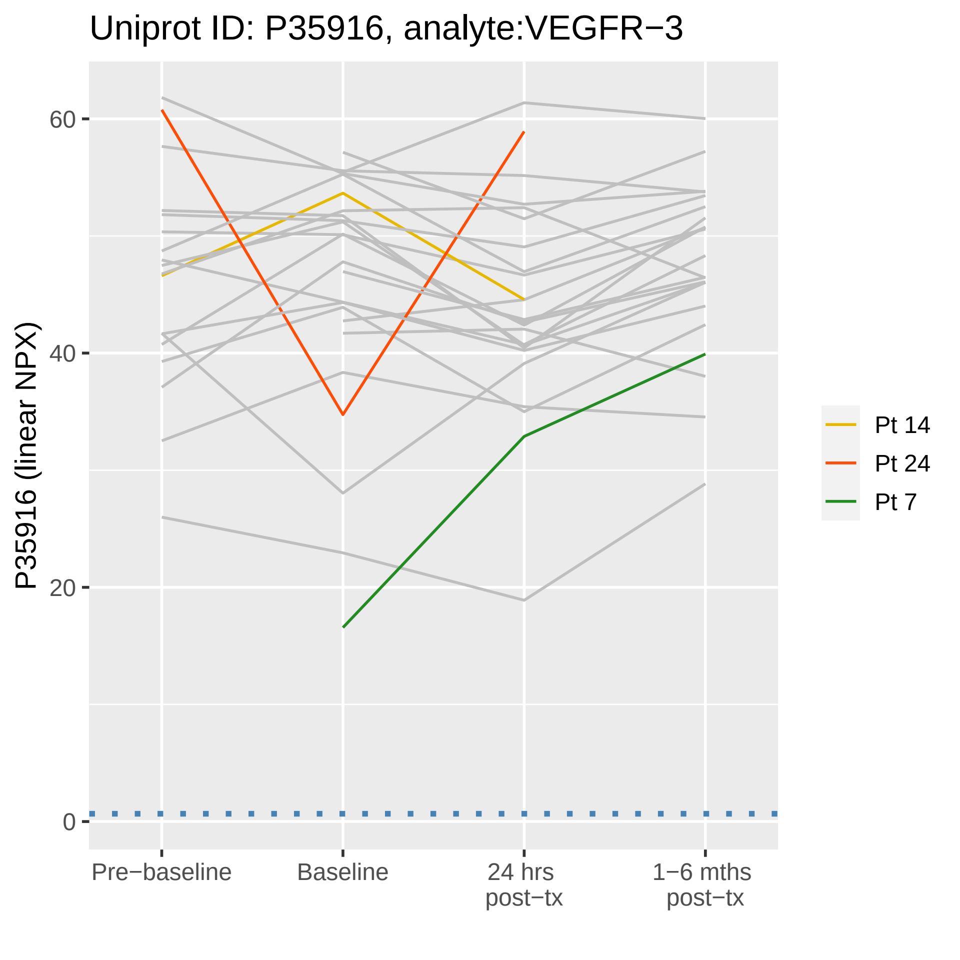

Uniprot ID: P35916, analyte:VEGFR−3
P35916 (linear NPX)
60
40
20
0
Pre−baseline
Baseline
24 hrs
post−tx
1−6 mths
post−tx
Pt 14
Pt 24
Pt 7

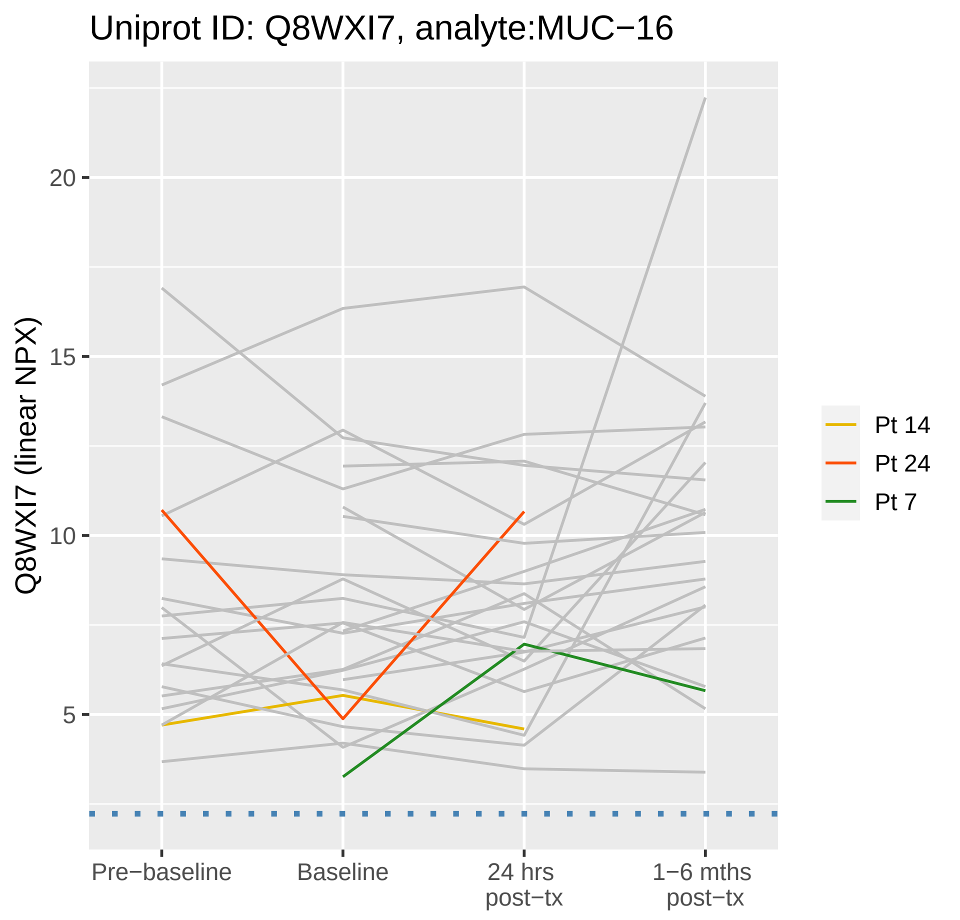
Uniprot ID: Q8WXI7, analyte:MUC−16
Q8WXI7 (linear NPX)
20
15
10
5
Pre−baseline
Baseline
24 hrs post−tx
1−6 mths post−tx
Pt 14
Pt 24
Pt 7

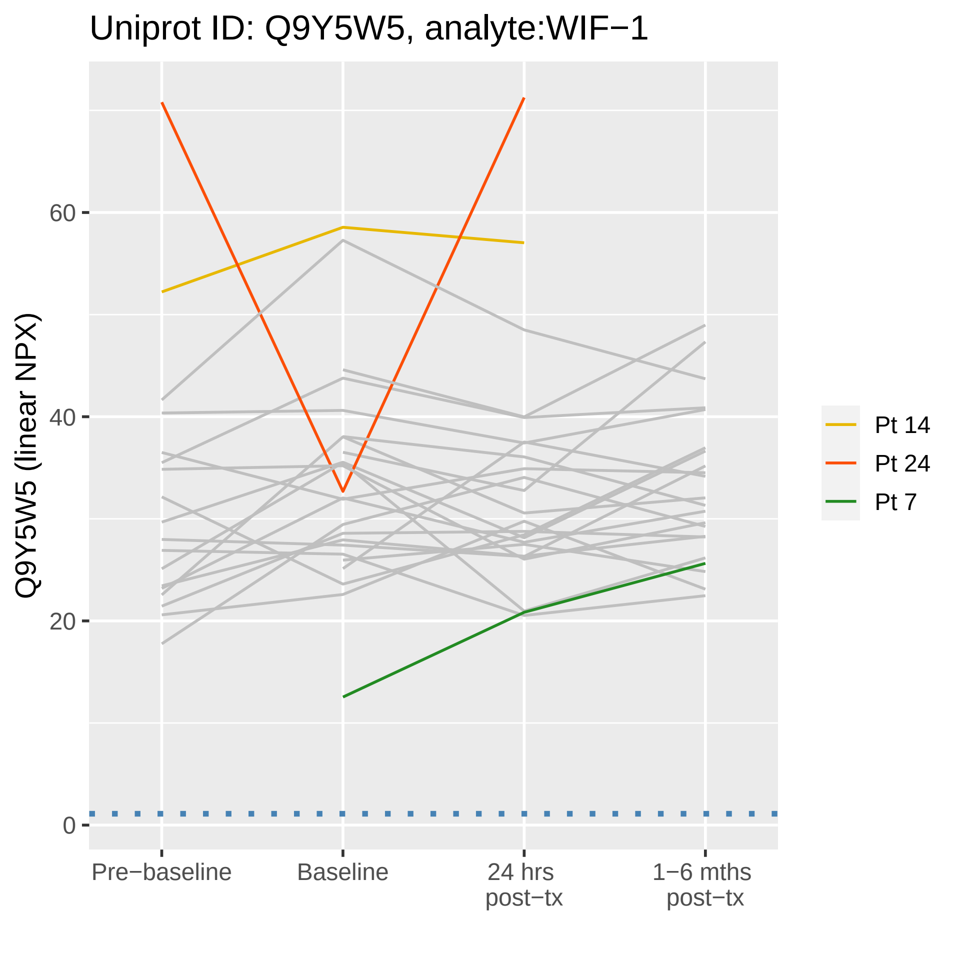
Uniprot ID: Q9Y5W5, analyte:WIF−1
Q9Y5W5 (linear NPX)
60
40
20
0
Pre−baseline
Baseline
24 hrs post−tx
1−6 mths post−tx
Pt 14
Pt 24
Pt 7

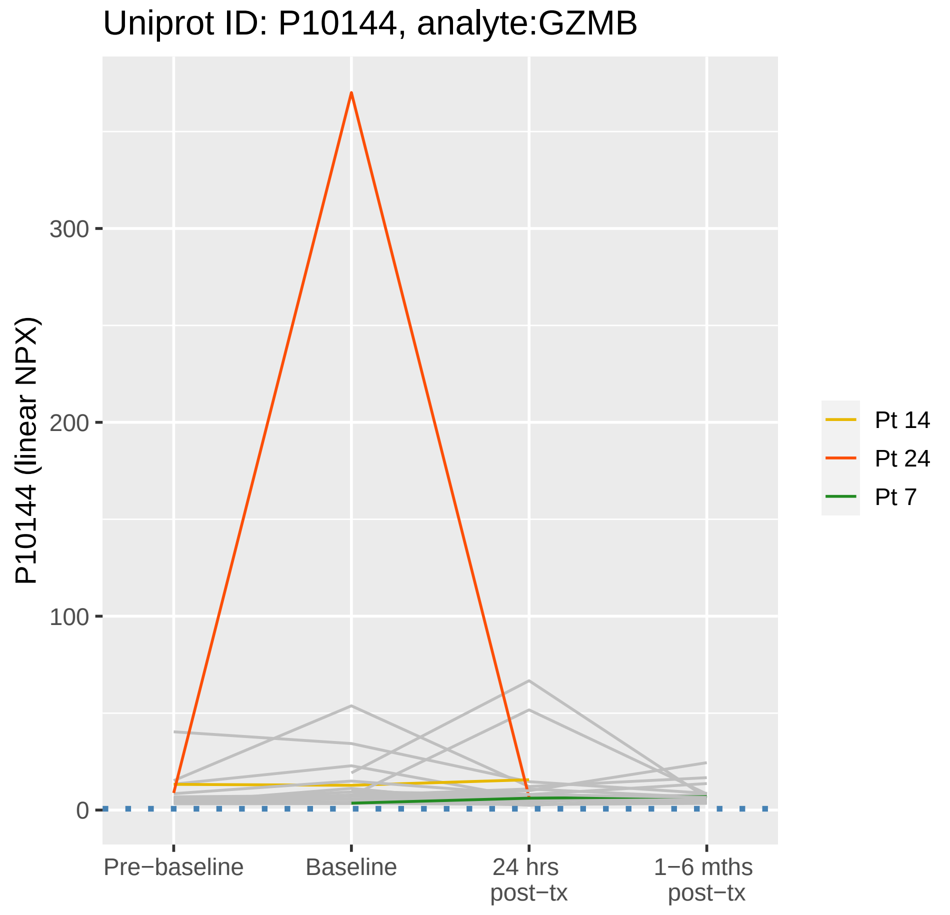

Uniprot ID: P10144, analyte:GZMB
P10144 (linear NPX)
300
200
100
0
Pre−baseline
Baseline
24 hrs
post−tx
1−6 mths
post−tx
Pt 14
Pt 24
Pt 7

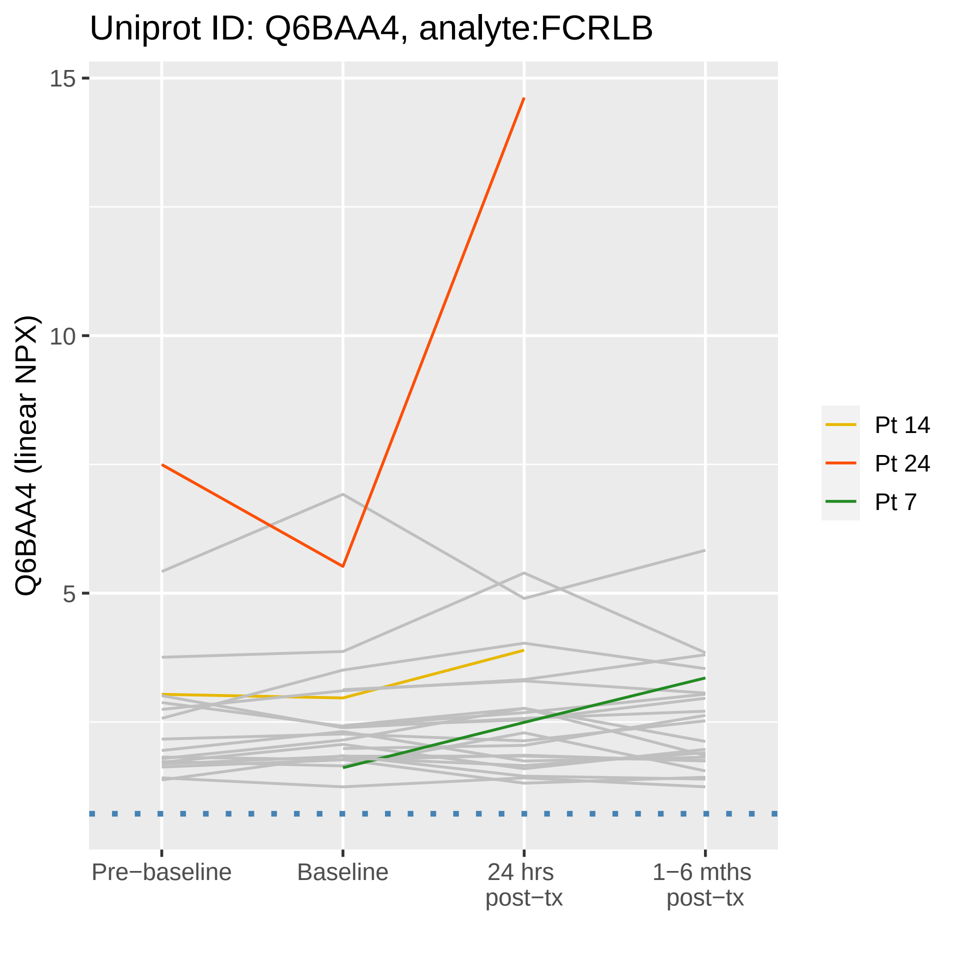

Uniprot ID: Q6BAA4, analyte:FCRLB
Q6BAA4 (linear NPX)
15
10
5
Pre−baseline
Baseline
24 hrs post−tx
1−6 mths post−tx
Pt 14
Pt 24
Pt 7

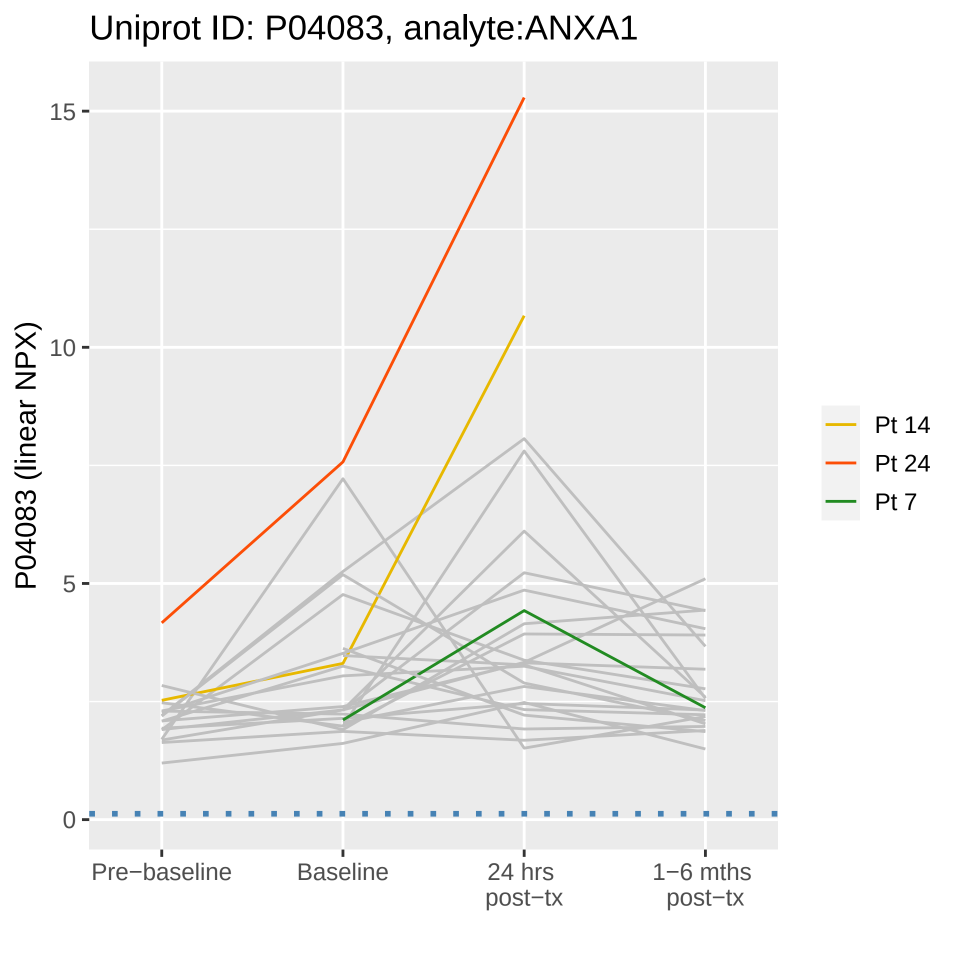

Uniprot ID: P04083, analyte:ANXA1
P04083 (linear NPX)
15
10
5
0
Pre−baseline
Baseline
24 hrs post−tx
1−6 mths post−tx
Pt 14
Pt 24
Pt 7

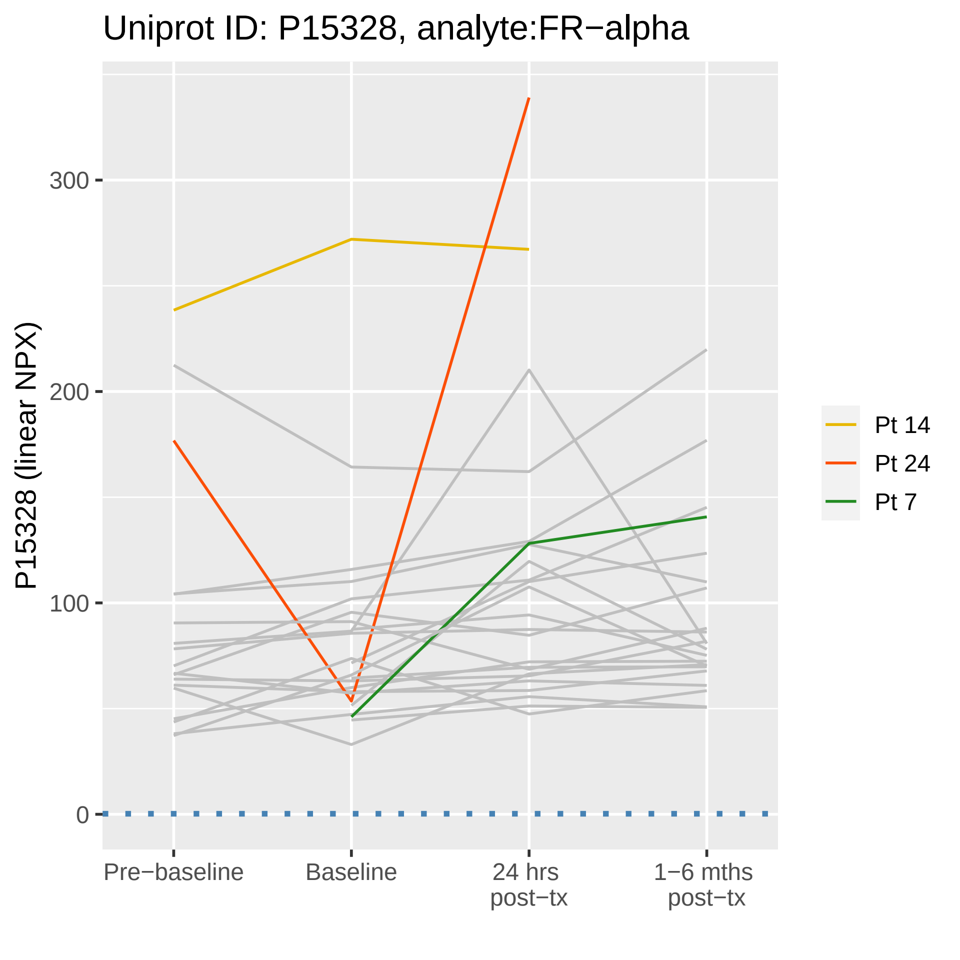

Uniprot ID: P15328, analyte:FR−alpha
P15328 (linear NPX)
300
200
100
0
Pre−baseline
Baseline
24 hrs post−tx
1−6 mths post−tx
Pt 14
Pt 24
Pt 7
