# Supplementary material for: An Exploratory Analysis of Changes in Circulating Plasma Protein Profiles Following Image-Guided Ablation of Renal Tumours Provides Evidence for Effects on Multiple Biological Processes
Source: Cancers (Basel). 2021 Nov 30;13(23):6037. doi: 10.3390/cancers13236037 (PMC8656737; doi:10.3390/cancers13236037)
Supplement: Supplementary file 1 [file cancers-13-06037-s001.zip › Supplementary File/Table S2.pdf]

Table S2. Proteins differing significantly (p<0.05 and q<0.01) between healthy controls (HC; n=11) and patients with RCC undergoing ablation at baseline prior to treatment (n=34).

Uniprot accession number	Protein	HC: median linear NPX (range)	RCC: median linear NPX (range)	p value	q value
P09038	FGF2	0.95 (0.66–1.36)	1.46 (0.96-56.91)	<0.001	<0.001
O00182	Gal-9	118.66 (85.34-178.93)	167.72 (127.14-308.18)	<0.001	<0.001
P09341	CXCL1	165.53 (60.35-319.99)	303.73 (107.63-1590.19)	<0.001	0.003
Q9NZQ7	PD-L1	14.43 (11.49-21.67)	21.55 (8.20-59.34)	<0.001	0.008
P00519	ABL1	3.44 (2.38-6.71)	7.90 (3.16-50.91)	<0.001	0.004
O95407	TNFRSF6B	8.46 (8.46-5.83)	13.95 (6.37-68.13)	<0.001	0.004
P50579	MetAP 2	6.75 (4.07-13.97)	13.99 (5.35-69.47)	<0.001	0.004
O14828	SCAMP3	5.58 (1.92-11.25)	11.91 (2.55-98.80)	<0.001	0.004
Q14508	WFDC2	74.59 (58.45-105.77)	103.25 (57.56-296.07)	<0.001	0.004
P43489	TNFRSF4	5.35 (3.99-6.82)	7.07 (3.30-22.35)	<0.001	0.004
P40222	TXLNA	7.70 (2.22-17.14)	23.05 (4.72-205.07)	<0.001	0.004
P38936	CDKN1A	1.74 (1.28-5.64)	5.77 (2.00-56.90)	<0.001	0.004
P26842	CD27	146.40 (97.54-195.54)	177.41 (104.86-437.14)	<0.001	0.007
P07948	LYN	1.30 (0.92-3.52)	2.66 (1.30-11.90)	0.001	0.008
P05231	IL6	2.99 (2.49-9.49)	4.56 (2.11-18.71)	<0.001	0.005
P15514	AREG	2.28 (1.56-2.69)	3.00 (1.43-12.09)	0.001	0.009
