# Supplementary material for: An Exploratory Analysis of Changes in Circulating Plasma Protein Profiles Following Image-Guided Ablation of Renal Tumours Provides Evidence for Effects on Multiple Biological Processes
Source: Cancers (Basel). 2021 Nov 30;13(23):6037. doi: 10.3390/cancers13236037 (PMC8656737; doi:10.3390/cancers13236037)
Supplement: Supplementary file 1 [file cancers-13-06037-s001.zip › Supplementary File/Table S3.pdf]

Table S3. Plasma proteins increasing significantly 24 hours post-CRYO (TP3) compared with the reference baseline assessment (TP2) prior to CRYO (n=24 as one TP3 sample failed analytical QC) in a matched pair analysis (Wilcoxon signed rank test) Results are presented both as linear NPX medians and ranges for TP2 and TP3 and as percentage change for TP3 compared with baseline. The number of patients showing an increase of any magnitude in each protein is presented together with the number that also exceeded the baseline protein-specific variability. *Due to changes in the Immuno-oncology panel, measurements for protein P40933 were only available in the 2020 dataset and so n=20 for that protein. **Four proteins were on both panels with similar results for both, with those from the Immuno-oncology panel presented.

Protein	Uniprot Accession Number	Baseline (TP2) linear NPX values	24 hrs post-CRYO (TP3) linear NPX values	TP3 protein result as % of TP2 baseline; median (range)	Number of patients with increase	Number of patients with increase >IQR of baseline variability	p value	q value (FDR)
Interleukin-6 (IL-6)	P05231**	14.79 (7.79-45.04)	93.42 (22.87-528.47)	518.7 (172.1-2917)	24 (100%)	24 (100%)	<0.001	<0.001
Interleukin-10 (IL-10)	P22301	3.94 (1.65-8.34)	8.74 (2.05-41.82)	199.6 (79.6-721)	22 (91.7%)	22 (91.7%)	<0.001	<0.001
C-C motif chemokine 23 (CCL23)	P55773	1066.48 (520.15-2248.50	1778.52 (1050.78-4052.33)	192.3 (94.4-338.6)	23 (95.8%)	22 (91.7%)	<0.001	<0.001
Heme oxygenase 1 (HO-1)	P09601	3136.65 (1496.88-5337.35)	4713.55 (2807.34-6211.16)	140.9 (99.6-407.1)	23 (95.8%)	21 (87.5%)	<0.001	<0.001
Endothelial cell-specific molecule 1 (ESM-1)	Q9NQ30	286.49 (143.93-671.86)	537.71 (224.82-1037.62)	177.7 (87.1-720.9)	22 (91.7%)	21 (87.5%)	<0.001	<0.001
Decorin (DCN)	P07585	14.91 (11.41-20.99)	16.39 (12.08-28.23)	116.8 (86.1-175.1)	20 (83.3%)	20 (83.3%)	<0.001	<0.001
Interleukin-15 (IL-15)	P40933*	22.48 (15.61-33.28)	32.28 (17.63-49.75)	154.2 (77.3-229.1)	16 (80%)	16 (80%)	<0.001	<0.001
Carbonic anhydrase-IX (CA-IX)	Q16790**	8.97 (3.48-43.07)	16.11 (6.26-51.53)	147.6 (76.9-473.2)	21 (87.5%)	19 (79.2%)	<0.001	<0.001
Macrophage colony stimulating factor 1 (CSF-1/M-CSF)	P09603	136.09 (59.52-195.06)	158.62 (53.10-218.28)	116.8 (99.4-237.5)	22 (91.7%)	18 (75%)	<0.001	<0.001
Tumour necrosis factor ligand superfamily member 14 (TNFSF14)	O43557	8.01 (3.53-20.91)	10.98 (3.44-31.28)	154.1 (49.1-360.4)	19 (79.2%)	18 (75%)	<0.001	0.002
Syndecan-1 (SYND1)	P18827	56.70 (25.11-129.54)	98.49 (30.06-231.30)	174.3 (82.2-515.6)	22 (91.7%)	18 (75%)	<0.001	<0.001
Transforming growth factor-alpha (TGFA)	P01135	2.25 (1.11-5.15)	2.82 (2.06-6.67)	129.7 (81.2-488)	19 (79.2%)	18 (75%)	<0.001	0.002

Hepatocyte growth factor (HGF)	P14210**	174.84 (94.59-407.48)	282.32 (124.50-841.86)	145.0 (95.9-545.7)	23 (95.8%)	18 (75%)	<0.001	<0.001
WAP-four disulfide core domain protein 2 (WFDC2)	Q14508	105.42 (57.56-296.07)	140.17 (66.26-273.31)	130.4 (64.3-197)	22 (91.7%)	18 (75%)	<0.001	<0.001
Amphiregulin (AREG)	P15514	3.09 (1.43-12.09)	3.86 (2.64-13.53)	140.5 (71.7-373.1)	21 (87.5%)	18 (75%)	<0.001	0.002
Protein S100-A11 (S100-A11)	P31949	4.76 (3.25-10.53)	5.69 (3.41-19.20)	126.5 (79.6-224.5)	20 (83.3%)	18 (75%)	<0.001	<0.001
Ephrin type-A receptor 2 (EPHA2)	P29317	2.42 (1.52-4.95)	2.92 (2.03-8.80)	114.1 (58.6-246.7)	20 (83.3%)	17 (70.8%)	<0.001	0.004
Placenta growth factor (PlGF)	P49763	292.36 (90.58-758.38)	339.32 (172.57-720.33)	126.0 (79.6-235.7)	20 (83.3%)	17 (70.8%)	<0.001	0.001
Fibroblast growth factor-binding protein 1 (FGFBP1)	Q14512	17.82 (9.85-24.79)	22.07 (7.98-156.96)	125.7 (76.1-706.6)	18 (75%)	17 (70.8%)	0.001	0.005
T cell surface glycoprotein CD5 (CD5)	P06127	19.04 (12.95-35.34)	23.52 (11.53-56.02)	118.8 (84.7-192.5)	20 (83.3%)	16 (66.7%)	<0.001	<0.001
Pleiotrophin (PTN)	P21246	5.07 (1.22-46.97)	10.86 (1.39-98.47)	207.2 (49.8-4195.3)	19 (79.2%)	16 (66.7%)	<0.001	0.002
Tissue factor pathway inhibitor 2 (TFPI-2)	P48307	139.03 (107.59-289.35)	198.83 (85.49-657)	134.7 (47.5-290.1)	19 (79.2%)	16 (66.7%)	<0.001	0.004
Galectin-1 (GAL-1)	P09382	58.74 (25.90-81.24)	68.59 (25.36-92.17)	111.8 (74.5-183.6)	18 (75%)	15 (62.5%)	0.002	0.003
R-spondin 3 (RSPO3)	Q9BXY4	8.79 (5.40-31.80)	13.30 (5.74-103.36)	133.3 (75.3-477.4)	19 (79.2%)	15 (62.5%)	<0.001	0.002
Monocyte chemotactic protein 3 (MCP-3)	P80098	2.72 (1.38-7.02)	3.94 (1.29-11.64)	152.6 (52.9-330.5)	19 (79.2%)	14 (58.3%)	<0.001	0.001
Interleukin-8 (IL-8)	P10145	48.18 (21.36-90.23)	74.33 (22.19-154.55)	154.1 (59.7-348.1)	18 (75%)	13 (54.2%)	<0.001	0.002
Vascular endothelial growth factor-A (VEGF-A)	P15692	302.93 (94.88-545.93)	353.83 (151.09-709.86)	124.3 (80.6-230.6)	18 (75%)	12 (50%)	<0.001	0.002
CD27 antigen (CD27)	P26842**	184.72 (79.24-447.18)	217.72 (123.38-469.03)	110.5 (85.5-170.1)	21 (87.5%)	12 (50%)	<0.001	<0.001
Tumour necrosis factor ligand superfamily member 13 (TNFSF13)	O75888	227.01 (125.01-484.84)	284.94 (120.35-596.60)	112.5 (71.8-245.1)	18 (75%)	12 (50%)	0.001	0.005
