# Supplementary material for: An Exploratory Analysis of Changes in Circulating Plasma Protein Profiles Following Image-Guided Ablation of Renal Tumours Provides Evidence for Effects on Multiple Biological Processes
Source: Cancers (Basel). 2021 Nov 30;13(23):6037. doi: 10.3390/cancers13236037 (PMC8656737; doi:10.3390/cancers13236037)
Supplement: Supplementary file 1 [file cancers-13-06037-s001.zip › Supplementary File/Table S4.pdf]

Table S4. Plasma proteins decreasing significantly 24 hours post-CRYO (TP3) compared with the reference baseline assessment (TP2) prior to CRYO (n=24 as one TP3 sample failed analytical QC) in a matched pair analysis (Wilcoxon signed rank test). Results are presented both as linear NPX medians and ranges for TP2 and TP3 and as percentage change for TP3 compared with baseline. The number of patients showing a decrease of any magnitude in each protein is presented together with the number that also exceeded the baseline protein-specific variability. *Two proteins were on both panels with similar results for both, with those from the Immuno-oncology panel presented.

Protein	Uniprot Accession Number	Baseline (TP2) linear NPX values	24 hrs post-ablation (TP3) linear NPX values	TP3 protein result as % of TP2 baseline; median (range)	Number of patients with decrease	Number of patients with decrease >IQR of baseline variability	p value	q value (FDR)
TNF-related apoptosis inducing ligand (TRAIL)	P50591*	183.25 (87.80-257.57)	128.00 (68.11-199.47)	72.3 (45.1-132.5)	23 (95.8%)	22 (91.7%)	<0.001	<0.001
C-X-C motif chemokine 10 (CXCL10)	P02778	256.15 (141.33-1314.23)	151.03 (46.94-688.18)	64.1 (42.3-186)	21 (87.5%)	20 (83.3%)	<0.001	<0.001
C-X-C motif chemokine 11 (CXCL11)	O14625	104.67 (31.34-2288.20)	61.93 (16.80-1398.57)	55.8 (2.8-155)	23 (95.8%)	20 (83.3%)	<0.001	<0.001
Interferon gamma (IFN-gamma)	P01579	1.22 (0.42-5.45)	0.53 (0.09-4.21)	55.0 (8.8-102.2)	20 (83.3%)	20 (83.3%)	<0.001	<0.001
Mothers against decapentaplegic homolog 5 (MAD homolog 5)	Q99717	7.58 (4.73-9.58)	6.60 (5.08-8.02)	83.0 (62.9-126.6)	21 (87.5%)	18 (75%)	<0.001	<0.001
Monocyte chemotactic protein 2 (MCP-2)	P80075	99.02 (29.75-223.06)	69.85 (43.56-130.56)	72.3 (45.1-146.4)	22 (91.7%)	17 (70.8%)	<0.001	<0.001
Monocyte chemotactic protein 4 (MCP-4)	Q99616	212.57(119.29-544.06)	133.25 (54.28-444.91)	59.2 (13.5-146.5)	23 (95.8%)	17 (70.8%)	<0.001	<0.001
T cell-specific surface glycoprotein CD28 (CD28)	P10747	1.94 (1.09-4.10)	1.63 (0.82-3.81)	88.6 (62.5-137.2)	17 (70.8%)	16 (66.7%)	0.005	0.009
Fas ligand (FASL)	P48023*	46.48 (23.22-127.89)	38.36 (19.02-95.40)	80.2 (66-116.8)	20 (83.3%)	16 (66.7%)	<0.001	0.001
Matrix metalloproteinase-12 (MMP-12)	P39900	168.38 (54.35-543.21)	151.83 (50.48-357.36)	77.9 (52.4-124.7)	20 (83.3%)	16 (66.7%)	<0.001	<0.001
Proto-oncogene tyrosine-protein kinase receptor Ret (RET)	P07949	8.56 (3.57-16.85)	6.28 (3.90-13.88)	79.2 (50.5-213.4)	20 (83.3%)	15 (62.5%)	0.002	0.007

Cathepsin L2 (CTSV)	O60911	5.52 (2.37-26.14)	4.20 (2.78-7.72)	77.5 (11.9-117.3)	18 (75%)	15 (62.5%)	<0.001	0.004
Vascular endothelial growth factor receptor 2 (VEGFR-2)	P35968	92.10 (55.41-137.53)	81.46 (48.04-106.89)	91.4 (72.7-127.9)	20 (83.3%)	14 (58.3%)	0.001	0.003
Angiopoietin-1 (ANGPT-1)	Q15389	243.88 (51.44-1016.60)	139.35 (45.80-621.91)	63.4 (23-199.7)	19 (79.2%)	14 (58.3%)	0.003	0.005
Platelet-derived growth factor subunit B (PDGF subunit B)	P01127	990.83 (198.17-1890.24)	765.56 (162.63-1639.06)	69.3 (32-208)	20 (83.3%)	12 (50%)	<0.001	0.002
Natural killer cell receptor 2B4 (CD244)	Q9BZW8	46.97 (21.93-118.96)	39.75 (17.55-76.83)	84.4 (64.6-181.3)	17 (70.8%)	11 (45.8%)	0.004	0.007
Fibroblast growth factor 2 (FGF-2)	P09038	1.50 (0.96-56.91)	1.29 (0.60-5.08)	81.4 (1.8-200.2)	18 (75%)	11 (45.8%)	0.001	0.003
C-X-C motif chemokine 5 (CXCL5)	P42830	845.36 (105.59-4482.71)	402.68 (103.13-4953.03)	61.0 (15.7-358.5)	20 (83.3%)	9 (37.5%)	<0.001	0.002
